# Investigations on the dose–response relationship of combined exposure to low doses of three anti-androgens in Wistar rats

**DOI:** 10.1007/s00204-017-2053-3

**Published:** 2017-09-06

**Authors:** Steffen Schneider, Karma C. Fussell, Stephanie Melching-Kollmuss, Roland Buesen, Sibylle Gröters, Volker Strauss, Xiaoqi Jiang, Bennard van Ravenzwaay

**Affiliations:** 1 Experimental Toxicology and Ecology, BASF SE, 67056 Ludwigshafen, Germany; 2 Chemical Food Safety, Nestlé, Lausanne, Switzerland; 3 Product Safety, Regulations, Toxicology and Ecology, BASF SE, Ludwigshafen, Germany

**Keywords:** Mixture, Low dose, Non-monotonic dose–response, Anti-androgenic, Endocrine disruptor, Experimental test guidelines, Additive

## Abstract

The current investigation examines whether combined exposure to three anti-androgens (flutamide, prochloraz, vinclozolin) result in interference with endocrine homeostasis when applied at very low dose levels, and whether the results of combined exposure are more pronounced than to the individual compounds. A pre–post-natal in vivo study design was chosen with more parameters than regulatory testing protocols require (additional endpoints addressing hormone levels, morphology and histopathological examinations). Dose levels were chosen to represent the lowest observed adverse effect level (LOAEL), the no observed adverse effect level (NOAEL), and the acceptable daily intake for each individual substance. Anti-androgenic changes were observable at the effect level (LOAEL) but not at lower exposures. Nipple/areola counts appeared to be a sensitive measure of effect, in addition to male sex organ weights at sexual maturation, and finally gross findings. The results indicate the absence of evidence for effects at low or very low dose levels. No (adverse) effects were seen at the NOAEL dose. A non-monotonic dose–response relationship was not evident. Combined exposure at LOAEL level resulted in enhanced responses for anogenital index, number of areolas/nipples, delayed preputial separation and reduced ventral prostate weight in comparison to the individual compounds.

**Electronic supplementary material:**

The online version of this article (doi:10.1007/s00204-017-2053-3) contains supplementary material, which is available to authorized users.

## Introduction

The regulation of substances is mostly based on single compound assessment. Moreover, human exposure is rarely to a single-substance, but rather to a variety of chemicals, cosmetic ingredients, drugs, biocides, pesticides and natural products. The need for mixture risk assessments has been discussed for several years and state-of-the-art reports on mixture toxicity—funded by DG Environment—were published (e.g., Kortenkamp et al. 2009). In the context of the regulation of pesticides in the EU (e.g., via the MRL Regulation (EC) No. 396/2005) due to the potential presence of pesticide residues in food “*…* account shall be taken of … known cumulative and synergistic effects, when methods to assess such effects are available.” Similar text is included in Regulation (EC) No. 1107/2009, concerning the placing of PPPs on the market in the EU. Current work is ongoing to identify active ingredients with common target organs to be included in Cumulative Assessment groups (e.g., EFSA 2013a; External Scientific Report 2016).

It is generally acknowledged that the combined toxicological effects of two or more compounds can take one of three forms: independent action, dose addition or interaction (Wilkinson et al. 2000; Feron and Groten 2002). Independent action, also known as response addition, occurs when the toxicological effects of the individual compounds in a mixture are a consequence of separate mechanisms/modes of action. Dose addition, also referred to as simple similar action, occurs when the individual compounds in a mixture share the same mechanism/mode of action of their toxicological effects, and they differ only in their potencies. The term interaction includes all forms of joint action that depart from either dose- or response-addition. Hence, the combined effects of two or more interacting chemicals is either greater (synergistic) or lesser (antagonistic) than that predicted based on dose addition or response addition.

The basic assumption for conducting combined/cumulative risk assessment is dose addition for compounds with similar mode/mechanism of action, often simplified by “having effects on the same target organ” (EFSA 2013a, b). As the evidence for synergism is very weak (Boobis et al. 2008, ECETOC Monograph “low dose interaction”), the dose additivity assumption is considered protective for human health assessments; moreover, there is experimental evidence that dose additivity is a conservative assumption (Schmidt et al. 2016).

Anti-androgens are compounds with similar downstream effects on male sexual development. Specific modes of action may vary, but all involve disruption of androgen signaling within the endocrine system, generally via inhibition of androgen hormone biosynthesis or by blocking receptor-mediated signaling (Gray et al. 2006; Hellwig et al. 2000) and can lead to non-reversible effects in male offspring when exposure occurs during certain windows of development (Schneider et al. 2011). This signaling is important for the development and maintenance of male sexual health (Fridmans et al. 2005). Flutamide has been shown to cause reduced AGDs, increased nipple retention, pubertal delays, decreased sex organ weights, hypospadias and reduced penile length in male rat offspring (summarized in Fussell et al. 2015) and prochloraz was found to increase transient nipple retentions and caused delayed entries into puberty (Christiansen et al. 2009; Laier et al. 2006).

Mixture effects have been reported, when assumed anti-androgenic phthalates and pesticides have been simultaneously tested in vivo at individual effect or NOAEL levels (Boobis et al. 2008; Christiansen et al. 2008; Gray et al. 2007; Hass et al. 2007; Howdeshell et al. 2008a, b). Dose additivity has been found also for compounds with different modes/mechanisms of action, but displaying similar downstream in vivo effects (Blystone et al. 2009; Rider et al. 2010). Similarly, in none of the publications, adequate studies on individual compounds and on the mixtures have been conducted in terms of animal numbers, the guideline used, and the determined endpoints, which significantly hamper the overall assessment on the type of observed mixture toxicity (dose additivity or synergy). Thus, it is quite likely that some of the reported NOAELs in the literature, and consequently the literature-derived “NOAEL doses” used in published combination experiments, are actually effect levels, rather than true NOAELs (Jacobsen et al. 2012).

Human exposure to chemicals, pesticides, biocides are usually below safe threshold levels (e.g., ADIs, DNELs, RfC), which are in turn far below the NOAELs identified in the animal studies. However, mixture studies using realistic human mixture exposures are scarce (ECETOC TR 115 2012).

Here side-by-side extended pre/post-natal developmental toxicity studies on individual compounds and a mixture study were conducted, using vinclozolin and flutamide as androgen receptor antagonists and prochloraz as an inhibitor of steroid hormone biosynthesis (EFSA 2011) with weak androgen receptor antagonistic properties. Dose levels of the individual substances were selected representing a LOAEL and a NOAEL for anti-androgenic effects, as well as the acceptable daily intake (ADI, as determined by regulatory agencies and usually 100× below the lowest NOAEL), which were then combined together into a LOAEL-MIX, a NOAEL-MIX, and an ADI-MIX (dosing each compound at its specific LOAEL/NOAEL/ADI). Thus, the experiments were designed to test whether any combined effects occur at individual reference values, and to compare the extent or grades of the expected effects with the determined effects in the mixtures. The results of the single compound testing have been published previously, i.e. flutamide (Fussell et al. 2015), prochloraz (Melching-Kollmuss et al. 2017) and vinclozolin (Flick et al. 2016). Here we report on the effects of combined exposure to all three anti-androgens.

## Materials and methods

The investigations reported herein exceeded the requirements of any specific regulatory guideline, but reference is made to OECD 414 and 416 (OECD 2001a, b) and 443 (Fegert et al. 2012; OECD 2011) as well as OPPTS 870.3700 (US Environmental Protection Agency 1998). The study was performed according to the OECD Principles of Good Laboratory Practice and the GLP Principles of the German “Chemikaliengesetz” (Chemicals Act) which (apart from few recognized differences) meet the United States Environmental Protection Agency Good Laboratory Practice Standards [40 CFR Part 160 (FIFRA) and Part 792 (TSCA)] (US Environmental Protection Agency 1972, 1976) . Hormone levels determination followed good scientific practice but was not according to formal GLP standards, but principally meeting EPA/FDA requirements. The study was performed in an AAALAC-approved laboratory. Permission for this study was obtained from the local regulatory agencies (permission number LRI-EMSG56-BASF/G11-3-013), and all study protocols were in compliance with the German Animal Welfare Act and the effective European Council Directive.

**Fig. 1:**
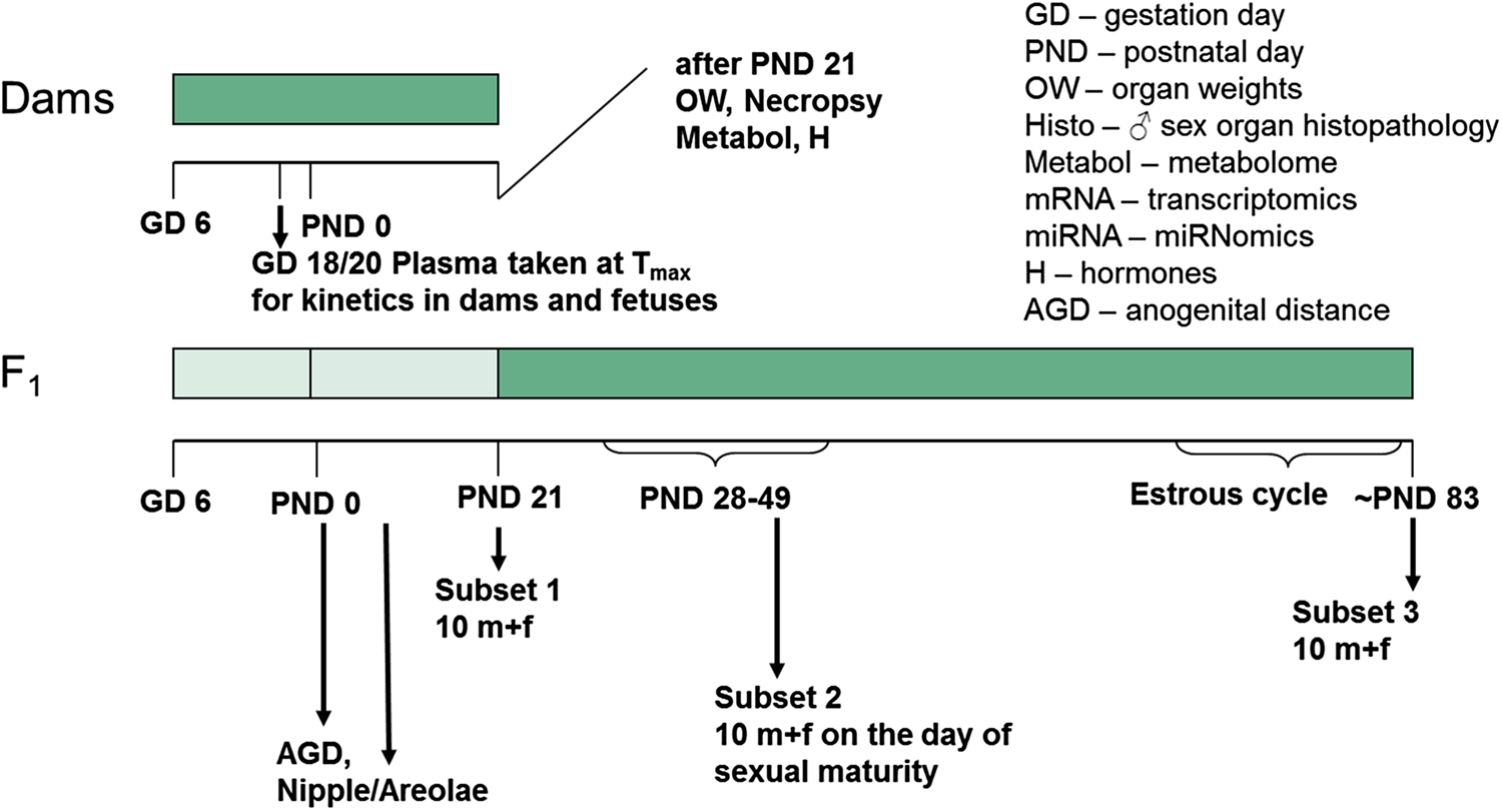
Experimental design of the study. It should be noted that samples were taken for future molecular analyses (metabolome, miRNome, and transcriptome), the results of which are not described in this publication. Also, the plasma kinetics of dams and fetuses taken from an additional number of five animals killed on GD 18 are not described in this publication

An overview of the experimental design is given in Fig. 1 as well as Table 1.

**Table 1:** Parameters investigated

Parameter	Dams (~PND 30)	Subset 1 (PND 21)	Subset 2 (Puberty)	Subset 3 (PND 83)
Full necropsy	+	+	+	+
Organ weights	+	+	+	+
Histopathology	+	+	+	+
Hormone levels	+	+	+	+
Estrous cycle				+
Spermatology				+

### Test substances

Vinclozolin (VIN), (RS)-3-(3,5-dichlorophenyl)-5-methyl-5-vinyloxazolidine-2,4-dione (CAS number 50471-44-8), and prochloraz (PRO), *N*-propyl-*N*-[2-(2, 4, 6-trichlorophenoxy)ethyl]-1H-imidazole-1-carboxamide (CAS number 67747-09-5) were synthesized and fully characterized at BASF SE (Ludwigshafen, Germany) as a white powder of purity >99% and a brownish solid of purity 98 ± 1%, respectively. Flutamide, 2-methyl-*N*-(4-nitro-3-[trifluoromethyl]phenyl)propanamide (FLT), CAS number 13311-84-7, was purchased from Sigma-Aldrich (St. Louis, MO, USA) as a yellow powder of purity >99%. These solids were thoroughly dissolved in the corn oil vehicle to prepare each dosing solution. The correctness and the homogeneity of prepared gavage formulations were determined by HPLC–MS analyses of several aliquots sampled from the bottom, middle and top of the preparation vessels. Furthermore, the stability of vinclozolin, flutamide, and prochloraz in corn oil vehicle was proven by testing a sample stored at room temperature at intervals over a period of 7 days. Fresh gavage solutions were prepared weekly.

The mixtures data reported in this investigation were captured in the final of a series of three separate experiments of the same study design addressing the theme of single and mixed exposures to low dose levels of anti-androgens. Since we have previously published the effects of the single-substance exposures to the three test substances in prior reports (Fussell et al. 2015; Melching-Kollmuss et al. 2017; Flick et al. 2016), these outcomes will not be discussed here in detail. Instead, the included single-substance exposure data are presented only for the purposes of comparison to the mixed exposures tested in this specific investigation and will be discussed only in this context. Since these data arise from three separate experiments, all three concurrent control groups (called α, β, and γ) have also been included. However, all statistical tests are based on the comparison between a treatment group and its concurrent control group only. The three equivalent concurrent control groups were also separately compared with each other to establish a control range for each parameter (Table 2, Supplementary Figures 1–3 and all Supplementary Tables).

**Table 2:** Summary of findings for VIN, FLT and PRO at (endocrine) LOAEL

Substance	LOAEL dose	Findings
VIN	20 mg/kg bw/d	Parental females No test substance-related adverse findings Pre-weaning Increased incidence of nipple/areolae in male pups on PND 12 (38% above control) Weaning Weight decrease of seminal vesicles Puberty Delay of preputial separation (about 1 day beyond the historical control range) Weight decrease of ventral prostate Juveno-adult transition in epididymides of two males Young adulthood Increased progesterone values in females around PND 83 in proestrus Weight decrease of cauda epididymis, epididymides, musc. bulb. lev. ani as well as total and ventral prostate Size reduction of seminal vesicles with decreased secretion in 1/10 male Size reduction of prostate with decreased secretion in 2/10 males
FLT	0.25 mg/kg bw/d	Parental females No test substance-related adverse findings Pre-weaning Increased incidence of nipple/areolae in male pups on PND 12 (28% above control) Weaning No test substance-related adverse findings Puberty Delay of preputial separation (about 1 day beyond the historical control range) Weight decrease of total/ventral prostate Young adulthood No test substance-related adverse findings
PRO	30 mg/kg bw/d	Parental females Decreased food consumption during GD 18–20 (about 11% below control) and during PND 0–21 (up to 23% below control) Decreased body weights during PND 0–21 (up to 8% below control) Increased duration of gestation (4% above control) Increased testosterone values in dams after weaning Decreased estradiol values in dams after weaning Pre-weaning Increased number of dams with stillborn pups (10 vs. 1 in control) Decreased number of live-born pups (30% below control) Increased number of stillborn pups (42 vs. 1 in control) Increased number of dead pups (8 vs. 0 in control) Increased number of cannibalized pups (7 vs. 0 in control) Increased incidence of nipple/areolae in male pups on PND 12 (37% above control) Decreased anogenital distance in female pups (10% below control) Decreased anogenital index in female pups (9% below control) Weaning No test substance-related adverse findings Puberty Juveno-adult transition in epididymides of three males Young adulthood Decreased food consumption in males during weeks 5–6 and weeks 7–8 (13% below control, respectively) Decreased mean body weights in males during weeks 5–8 (up to 12% below control, respectively) Decreased mean body weight change in males during several parts of the study phase (up to 31% below control, respectively) Increased testosterone values in females around PND 83 (in proestrus) Decreased estradiol values in females around PND 83 (in proestrus) Increased androstenedione and progesterone values in females around PND 83 (in proestrus) Decrease of terminal body weight in males Size reduction of seminal vesicles with decreased secretion in 1/10 male Size reduction of prostate with decreased secretion in 2/10 males

### Parental female animals

Permission for animal studies was obtained from local regulatory agencies, and all study protocols were in compliance with German and EU animal welfare requirements. The study was performed in an AAALAC-approved laboratory. Time-mated outbred Wistar rats (WI:Han) were obtained from Charles River Laboratories (Sulzfeld, Germany) on gestational day zero (GD 0), defined by the presence of sperm or a vaginal plug in the vaginal canal. Throughout the study, all animals were maintained under standard conditions: one animal (or litter) per Makrolon type MIII cage with LTE E001 bedding (ABEDD, Vienna, Austria) and maintained at 20–24 °C and 30–70% humidity with 15 air changes per hour and a 12-h light/dark cycle. All animals had free access to food (two batches of ground Kliba maintenance diet mouse/rat ‘GLP’ containing 102.5 and 122 ppm of total genistein equivalents [Provimi-Kliba, Kaiseraugst, Switzerland]), water and a wooden gnawing block as enrichment. The animals were allowed to acclimatize to the laboratory conditions until GD 6, when the dams were randomized into four dose groups of 25 animals per group.

Dams were treated with mixtures of vinclozolin, flutamide and prochloraz at three different dose levels representing the lowest observed adverse effect level (LOAEL), the no observed adverse effect level (NOAEL), and the acceptable daily intake (ADI) for each substance. The LOAEL-MIX contained 20, 0.25, and 30 mg/kg bw/d; the NOAEL-MIX 4, 0.025, and 5 mg/kg bw/d; and the ADI-MIX 0.005, 0.00025 and 0.01 mg/kg bw/d vinclozolin (see Flick et al. 2016), flutamide (see Fussell et al. 2015), and prochloraz (see Melching-Kollmuss et al. 2017), respectively. Each dam was administered its respective mixture in corn oil by gavage every morning from gestational day (GD) 6 until the day of killing [GD 20 or after weaning around post-natal day 30 (PND 30)], except during labor. In prior studies, these dose levels were also administered as single-substance exposures; a larger dose of flutamide (2.5 mg/kg bw/d) was also administered as a “clear effect level” positive control. These dose groups are also presented alongside the mixtures data for comparison purposes; however, as they have been previously reported, they are not discussed in detail as part of this investigation.

On GD 20, blood (1 mL with 10 μL of 10% EDTA as an anticoagulant) was collected from five dams in each dose group. Thereafter, these dams were killed by cervical dislocation under isoflurane anesthesia and necropsied. The pregnant uteri were dissected, opened, and the fetuses were removed. All implants and fetal weights were recorded before the fetuses were killed by snap freezing in liquid nitrogen and stored for future kinetic studies. The remaining dams were allowed to deliver and rear their pups until PND 21 (weaning).

During gestation and lactation, each dam was examined daily for clinical signs of morbidity and toxicity, as well as parturition and lactation behavior. The food consumption and body weight of each dam was evaluated on GD 0, 6, 13, 18, and 20. The food consumption was also determined weekly for each litter during lactation (PND 0–21) and later for every weaned F1 animal in subsets 2 and 3 (for explanation of subsets see 3.3). All females, which littered, were weighed on PND 0, 7, 14, and 21. Females, which did not litter, were killed and examined for gross abnormalities. The uteri were removed from these animals and stained with Salewski stain for implantation sites (Salewski 1964).

### Offspring

The gender, status (live- or stillborn) and any gross morphological abnormalities of each delivered pup were recorded as soon as possible after birth. Pup viability, mortality and any clinical signs of toxicity or morbidity were determined at least daily. The pups were weighed on PND 1, 4, 7, 14, 21, as well as on the day of sexual maturation (vaginal opening or preputial separation). Anogenital measurements were obtained on all living pups on PND 1 using a measuring ocular in a blind, randomized fashion. All living, male pups were examined for the presence or absence of nipple/areola anlagen on PND 12, and re-examined on PND 20. Males of the control and LOAEL-MIX groups were also checked on PND 38, and again on the day of preputial separation.

Before weaning on PND 21, twenty pups of each gender per dose group were selected randomly to be allowed to mature (ten per gender in each of subsets 2 and 3). After blood sampling on PND 21 (at least 1 mL with 10 μL of 10% EDTA per mL as an anticoagulant and a further 300 μL without anticoagulant from the dams for estradiol measurement), the dams and a further ten male and ten female pups (subset 1) were killed under isoflurane/carbon dioxide anesthesia and dissected. The relevant tissues (Supplementary Table 1) were harvested from these animals for pathological and molecular analyses. Any surplus pups were also similarly killed on PND 21, but only macroscopically examined for gross abnormalities.

From PND 21 until sexual maturity (subset 2) or young adulthood (subset 3), the maturing F1 pups were also orally treated with the mixtures by gavage. On the day of vaginal opening (females) or preputial separation (males), blood was sampled from each animal in subset 2 (about 500 µL with 5 µL of 10% EDTA as an anticoagulant) before it was killed and necropsied. The relevant tissues (Supplementary Table 1) were harvested from these animals for pathological and molecular (metabolome, miRNome and transcriptome) analyses. Similarly, blood was sampled from each F1 animal of subset 3 (again, approx. 1 mL with 10 μL of 10% EDTA as an anticoagulant and another 300 μL of female blood without anticoagulant) before killing as a young adult (PND 81–85). Again, the sexually dimorphic tissues were harvested.

### Hormone analysis

#### Plasma/serum samples

The blood samples collected with EDTA after weaning (both parental and filial samples), sexual maturity, and during young adulthood were centrifuged under refrigeration to separate out the plasma. This plasma was aliquoted (>200 μL per aliquot) and stored under nitrogen at −80°C for general hormone analysis. Serum was also similarly prepared and stored from the blood sampled without anticoagulant from all females, with the exception of the PND 21 pups, for the measurement of estradiol levels.

Steroid hormones (androstenedione, testosterone, progesterone and corticosterone) were measured in plasma by a proprietary online (solid phase extraction LC–MS/MS; Yamada et al. 2002; Zhang et al. 2011). Absolute quantification was performed by means of stable isotope-labeled standards.

Estradiol concentration in serum samples was determined using a commercially available ELISA kit from DRG Diagnostics (EIA-4399; Marburg, Germany) measured on a Sunrise MTP-reader (Tecan AG, Maennedorf, Switzerland) and evaluated by the Magellan Software of the instrument producer. Estradiol was not measured in subset 1 females or in males at any age, as estradiol concentrations in these animals are known to be below the technical lower limit of quantitation for this determination.

#### Testosterone in testes of male pups at gestation day 20 after ex vivo incubation

This investigation was solely conducted in the mixture experiment, because it is described as more sensitive method to measure endocrine effects (Borch et al. 2006). No equivalent data are available from the single-substance experiments, and can thus not be used for doing the combined toxicity assessment. Left and right fetal testes of all male fetuses of five dams per group were weighed and immediately incubated separately, each in 500 µL DMEM/F12 medium (without phenol red addition; with HEPES, 0.1 g/L gentamicin and 0.1% fetal bovine serum addition) for 5 h in a humidified incubator with 5%CO_2_ enriched atmosphere at 37 °C on a horizontal rotator (150 rpm). The supernatant was stored at −80 °C until measurement.

Testosterone was measured after 1:4 dilution with DMEM/F12 medium with a Testosterone ELISA (DRG, cat no. EIA-1559) on a Sunrise MTP-reader (Tecan AG, Maennedorf, Switzerland) and evaluated with the Magellan Software of the instrument producer. The lowest quantifiable testosterone value is 1.2 nmol/L.

### Tissue preparation and histopathological analysis

At all time points, the organs of dams and offspring were carefully trimmed of excess adhering fat and tissue and were weighed (fresh, unfixed). Adrenal glands, brain, cauda epididymis, epididymides, kidneys, liver, ovaries, spleen, testes and uterus were weighed without blotting to the nearest 0.001 g. Any other tissues (musc. levator ani together with musc. bulbocavernosus, Cowper’s gland, glans penis, pituitary gland, prostate [total, ventral only], seminal vesicles with coagulating glands, thyroid glands) were weighed without blotting to the nearest 0.1 mg. The anesthetized animals were weighed to the nearest 0.1 g. Immediately after weighing, the ventral prostate was cut longitudinally in two halves (males of subsets 1 and 2). One of these halves, as well as the right testis and the right seminal vesicle, was snap frozen in liquid nitrogen and stored at −80°C for future analyses. In male animals of subset 3, both the ventral prostate and the right testis were halved after weighing. Half of each tissue plus the right seminal vesicle were snap frozen for possible future transcriptome analyses. In subset 3 males, sperm analysis was performed with the residual half of the right testis and the complete right epididymis: cauda epididymis sperm motility according to the method in Slott et al. (1991), sperm morphology, spermatid head count in the testes, and sperm head count in the cauda epididymis (Feuston et al. 1989, slightly modified).

All weighed tissues were preserved (except of glans penis, Cowper’s gland, musc. levator ani together with musc. bulbocavernosus) and fixed in 10% neutral-buffered formalin or modified Davidson’s solution (ovaries, left epididymis, and left testis). In addition to the organs mentioned above, oviducts and male/female mammary glands were sampled. Light-microscopical assessment was performed in all male offspring of all three subsets (all gross lesions, adrenal glands, left coagulating gland, left epididymis, pituitary gland, left prostate, left testis, left seminal vesicle). Therefore, the fixed tissues were trimmed, paraplast-embedded, cut with a thickness of 2–3 µm, mounted on glass slides and stained with routine hematoxylin–eosin stain. All work was done according to published literature (“Revised guides for organ sampling and trimming in rats and mice” (Ruehl-Fehlert et al. 2003; Kittel et al. 2004; Morawietz et al. 2004; Creasy et al. 2012) and assessment was performed by a board certified veterinary pathologist (DECVP) followed by an internal peer review.

### Statistical analysis

#### Statistical analysis for the assessment of measured parameters

Means and standard deviations were calculated. In addition, the following statistical analyses were carried out.

#### Statistical analysis for the assessment of potential additivity of effects

For selected parameters (where applicable) the Loewe additivity model (Loewe 1953) was used for compound combination evaluations to examine whether more than additive effects of the test substances at LOAEL concentrations occurred. For a combination of *k* (*k* ≥ 2) compounds, the interaction index *τ* based on Loewe additivity model can be expressed as

$$\tau = \frac{{d_{1} }}{{D_{y,1} }} + \cdots + \frac{{d_{k} }}{{D_{y,k} }}\left\{ {\begin{array}{*{20}c} { < 1, } \\ { = 1,} \\ { > 1,} \\ \end{array} } \right. \,\,\,\,\begin{array}{l} {synergy} \\ {additivity} \\ {antagonism} \\ \end{array} ,$$

where *d*_1_,…*d*_*k*_ are doses of each compound in the mixture of *k* compounds resulting in effect *y* and *D*_*y*,1_,…*D*_*y*,*k*_, are the doses of compounds that result in the same effect *y* for each respective compound given alone. A confidence interval for the interaction index *τ* was constructed to account for variabilities in estimating dose-effect models. The combination dose is synergistic if the upper limit of the confidence interval is less than 1, antagonistic if the lower limit of the confidence interval is greater than 1, and additive if the confidence interval embraces the number 1. For the effect *y* observed in the mixture experiment, the corresponding doses of each single compound resulting in the same effect *y* can be estimated using inverse regression model. For AGI and day of preputial separation, a normalization procedure taking into account the concurrent study control data was performed. Specifically, the individual parameter value was divided by the mean for the corresponding control.

##### Dose–response modeling and calculation of interaction index based on Loewe additivity model

The dose–response relationship is fitted using a non-linear regression model. The selection of regression model depends on the quality of a response. Modeling the probability of a binary response as a function of dose can be use a logistic regression. Gamma regression is suitable for modeling non-positive data and Poisson regression can be used to relate count responses to predictor.

Gamma regression was used to fit the relationship between dose and the weight of ventral prostate. The corresponding dose–response curve fits for the individual chemicals and mixture were displayed in Supplementary Figure 31. The horizontal lines indicate the mean weight of ventral prostate observed in the mixture experiment. The vertical lines indicate the estimated doses of each individual chemical resulting in the same mean weight of ventral prostate using the inverse regression model. The dose values (*d*) used in mixture experiment and the estimated dose values (*D*) of individual chemicals were substituted into the formula *τ* = *d*_1_/*D*_1_ + *d*_2_/*D*_2_ + *d*_3_/*D*_3_ used for calculation of interaction index based on Loewe additivity model. The calculation results are shown in Supplementary Table 38.

## Results

All concentration control analyses showed that the achieved substance concentrations were within acceptable limits, prepared dosing formulations were stable and homogeneous. Contaminants in the feed or water or changes in the environmental conditions, which might have influenced the outcome of the studies, were not observed (data not shown).

### Gestation and littering

Despite a few idiopathic cases divided evenly among the dose groups, parental female fertility and mortality were unaffected by treatment; nearly all dams in this study were pregnant and survived until scheduled termination. A few clinical signs were observed in the dams after receiving either the LOAEL or NOAEL mixes or the LOAEL dose of prochloraz as a single-substance. These findings after treatment were minor and limited to piloerection, indicating stress, and salivation, likely a result of an unpleasant taste of the test substance or by local irritation of the upper digestive tract. Neither is considered to be a sign of systemic toxicity. Food consumption and body weight parameters were comparable to the concurrent control groups and within historical control ranges throughout the entire dosing period.

However, some alterations to reproductive performance were observed (Table 3). Statistically significantly, lengthened gestation was observed as a result of treatment with the LOAEL mixes. An increased duration of gestation was also seen for the NOAEL mixes but the value lies within the historical control range for the test facility (21.5–22.5 days) and is therefore not considered to be biologically relevant. Increased gestational length was also observed after treatment with the LOAEL of prochloraz, but not flutamide or vinclozolin, suggesting that this effect of the LOAEL mixture is specific to prochloraz.

**Table 3:** Summary of findings for FLT at positive control dose

Substance	Dose	Findings
FLT	2.5 mg/kg bw/d	Parental females No test substance-related adverse findings Pre-weaning Increased incidence of nipple/areolae in male pups on PND 12 (49% above control) and PND 20 (76% above control) Decreased anogenital distance in male pups (21% below control) and both sexes combined (20% below control) Decreased anogenital index in male pups (21% below control) and both sexes combined (18% below control) Increased body weight at criterion of preputial separation (33% above control) Weaning Weight decrease of cauda epididymis, epididymides, musc. bulb. lev. ani, total prostate and ventral prostate Puberty Delay of preputial separation (about 10 days beyond the historical control range) Increased testosterone and androstenedione values in males Weight decrease of total/ventral prostate Hypospadias in one male animal Juveno-adult transition in epididymides of all males Young adulthood Increased androstenedione levels in females around PND 83 in proestrus Weight decrease of bulbourethral gland, cauda epididymis, epididymides, glans penis, musc. bulb. lev. ani, total and ventral prostate as well as seminal vesicles Size reduction of testes, epididymides, prostate and seminal vesicles in one animal Size reduction of seminal vesicles with decreased secretion in 9/10 males Size reduction of prostate with decreased secretion in 5/10 males Unilateral minimal multifocal testicular tubular degeneration in 1/10 male Unilateral severe diffuse testicular tubular degeneration with Leydig cell hyperplasia in 1/10 male

Treatment with the LOAEL-MIX decreased the number of live-born and increased the number of stillborn pups across a number of the litters of these dose groups. A similar change was observed for the LOAEL dose of prochloraz (Melching-Kollmuss et al. 2017). However, in the current study, litter size and offspring weights remained unaffected. A statistically significant increase in pup death was observed in the first four post-natal days in animals exposed to the LOAEL of prochloraz, which, when combined with the statistically significant increase in cannibalization, contributed to reduced pup viability in this group. Therefore, it is not unlikely that prochloraz contributed to this observation. The sex ratio was unaffected in this study and in the single compound studies.

Pup mortality was increased in the NOAEL-MIX group, but as all dead pups and four of the six cannibalized pups were from the same litter, this effect is assessed as not related to the test substance administration. No other maternal findings or effects on reproductive performance were observed.

### Offspring development

#### Anogenital distance and index

**Fig. 2:**
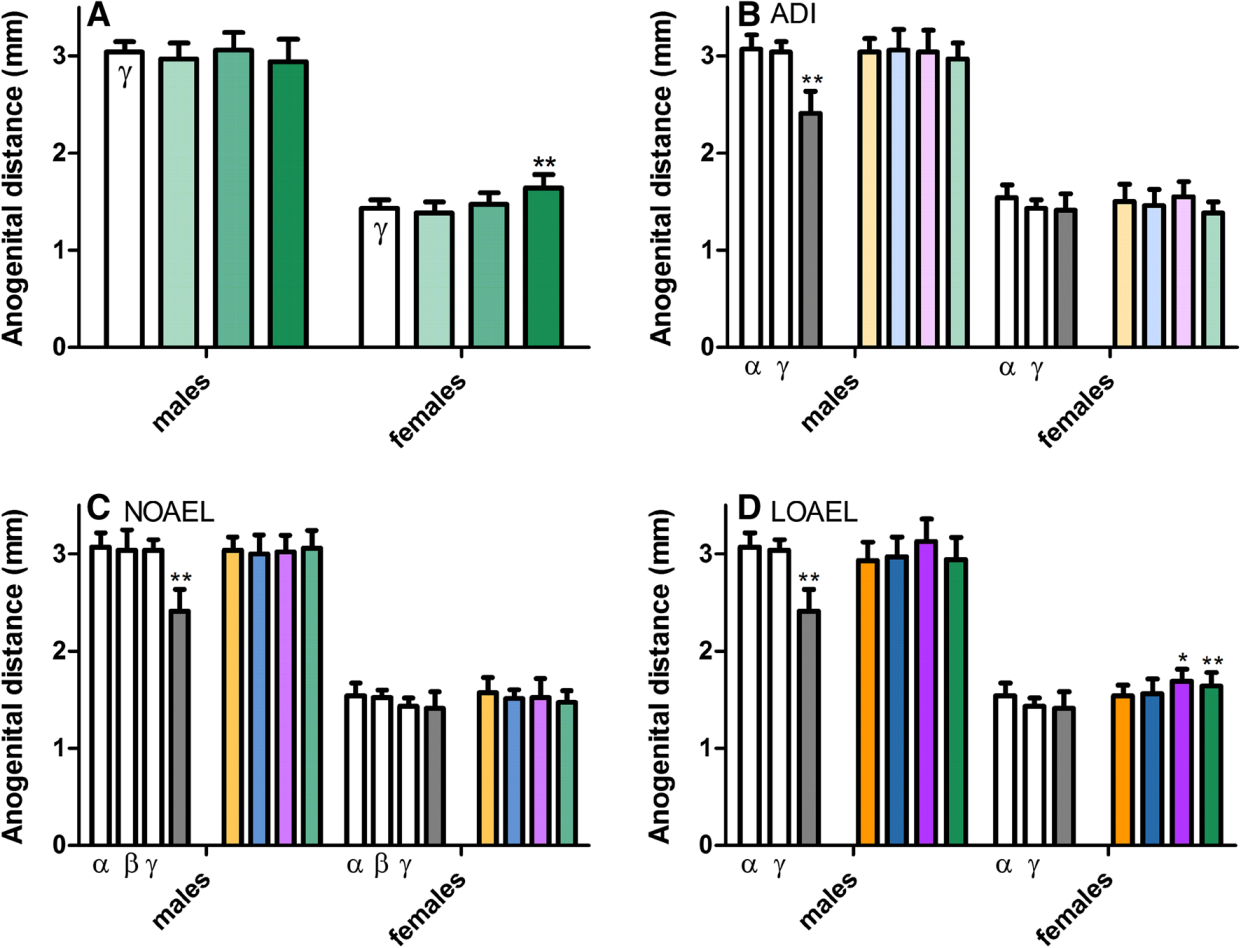
Effect of increasing dose of mixtures of vinclozolin, flutamide, and prochloraz on anogenital distance. On PND 1, the anogenital distance (AGD) of all live-born pups exposed in utero to the vehicle control *open rectangle*, ADI-MIX *filled very light green rectangle*, NOAEL-MIX *filled light green rectangle* and LOAEL-MIX *filled thick green rectangle* was measured (**a**). These mixture results were then compared to the effects of the single-substance exposures to vinclozolin *filled yellow rectangle*, flutamide *filled blue rectangle*, and prochloraz *filled pink rectangle* at each dose level: ADI (**b**), NOAEL (**c**) and LOAEL (**d**), as well as vehicle *open rectangle* and positive controls *filled grey rectangle*. (Data are shown as mean ± SD.) Prenatal exposure to LOAEL dose levels of these anti-androgens, but not ADI or NOAEL levels, significantly altered AGDs. These data were collected in the course of three different experiments with similar study design. For clarity, only the concurrent controls relating to the exposures at each dose level are depicted in each graph. A comparison of the three control datasets can be found in **a** of Supplementary Figure 1 (color figure online)

As pup development was also monitored post-partum, a number of test-substance-related clinical findings were observed in the developing F1 offspring. On the day after birth, a small, but statistically significant increase in the anogenital distance (AGD) of female pups, as well as a statistically insignificant decrease in that of the male pups, were both observed in offspring exposed to the LOAEL-MIX (Fig. 2 and Supplementary Table 3). This increase in female AGD mirrored that observed because of the single-substance exposure to the LOAEL of prochloraz, but not the other substances; thus it is assumed that this effect of the mixture is specific to prochloraz. Non-statistically significantly, decreased male AGD were observed at the LOAEL doses of vinclozolin and flutamide, but not with prochloraz. No effects on the AGD were observed at the NOAEL or ADI dose levels.

**Fig. 3:**
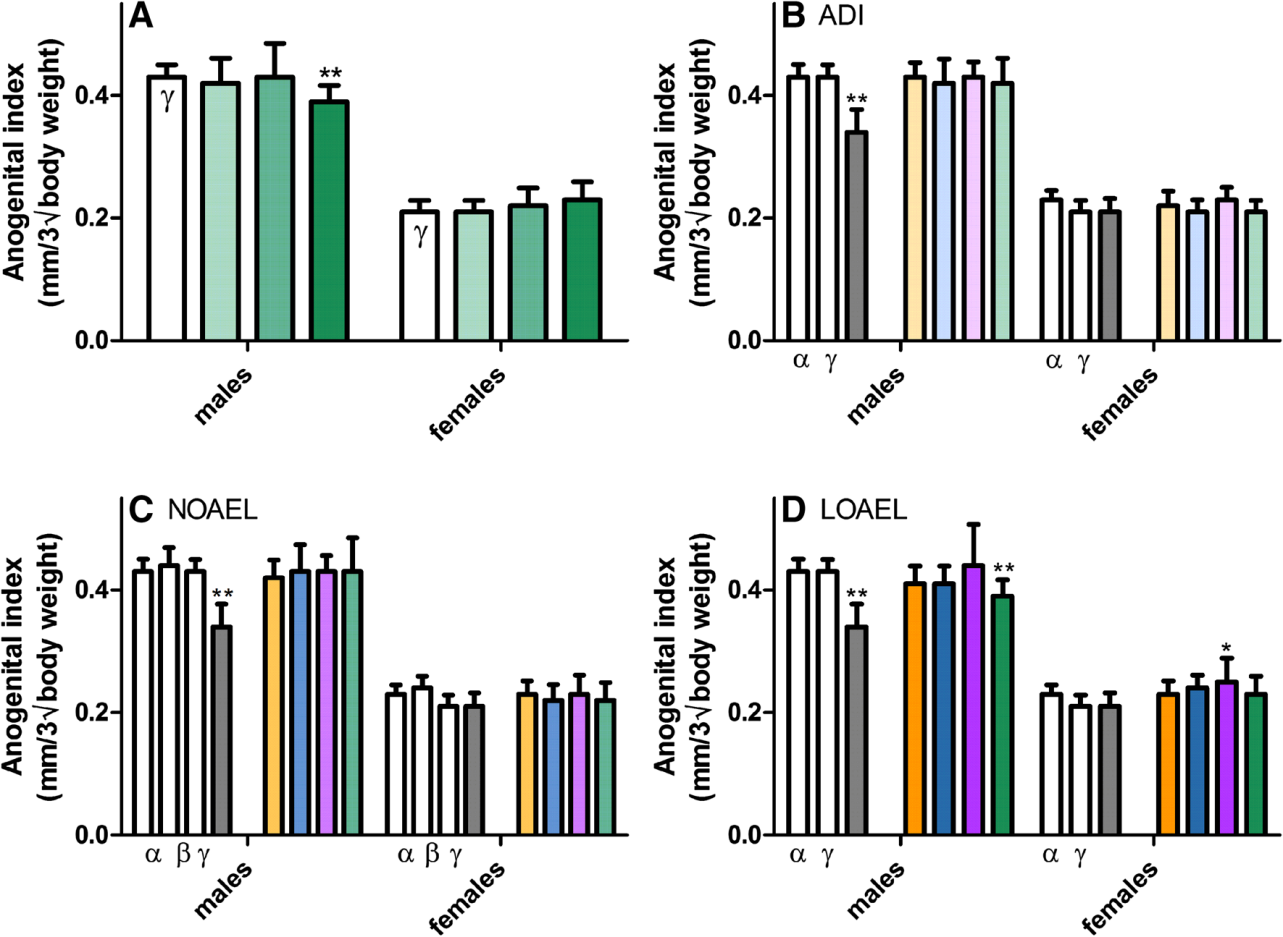
Effect of increasing dose of mixtures of vinclozolin, flutamide, and prochloraz on the anogenital index. The anogenital index (AGI), a parameter which accounts for any differences in animal size, was calculated from the AGDs of all live-born pups exposed in utero to the vehicle control *open rectangle*, ADI-MIX *filled very light green rectangle*, NOAEL-MIX *filled light green rectangle* and LOAEL-MIX *filled thick green rectangle* (**a**). These mixture results were then compared to the effects of the single-substance exposures to vinclozolin *filled yellow rectangle*, flutamide *filled blue rectangle*, and prochloraz *filled pink rectangle* at each dose level: ADI (**b**), NOAEL (**c**) and LOAEL (**d**), as well as vehicle *open rectangle* and positive controls *filled grey rectangle*. (Data are shown as mean ± SD.) Prenatal exposure to LOAEL dose levels of these anti-androgens, but not ADI or NOAEL levels, significantly altered calculated AGIs. These data were collected in the course of three different experiments with similar study design. For clarity, only the concurrent controls relating to the exposures at each dose level are depicted in each graph. A comparison of the three control datasets can be found in **b** of Supplementary Figure 1 (color figure online)

These results are largely in concordance with those of the calculated anogenital index (AGI), a parameter where the weight of the animal is also taken into account, except that the statistical significance was reversed. In this case, the increase in the AGI of female pups was statistically insignificant after LOAEL-MIX exposure, while the decrease in the AGI of male pups with the LOAEL mixture was significant (Fig. 3 and Supplementary Table 3). The AGI in male pups was decreased in the LOAEL doses of flutamide and vinclozolin, but not in the prochloraz group and did not reach statistical significance. The AGI in female pups was increased at the prochloraz LOAEL dose (see Melching-Kollmuss et al. 2017).

No effects on the male or female AGI were observed at the NOAEL-MIX or ADI-MIX dose levels. When the Loewe additivity model was applied, the effects on male AGI of the LOAEL mixture group were more than expected assuming additivity (see boxplot in Supplementary Figure 28). It should be noted, however, that this was solely contributable to the absence of an effect on AGI in the prochloraz-treated male animals. Model calculations for vinclozolin and flutamide demonstrated approximate additivity of effects. Using the dose–response relationship for flutamide (including the positive control dose of 2.5 mg/kg bw/d) it can be calculated that the effect of the LOAEL mixture group on AGI is equivalent to a flutamide dose of 1.08 mg/kg bw/d.

#### Areolas/nipples

**Fig. 4:**
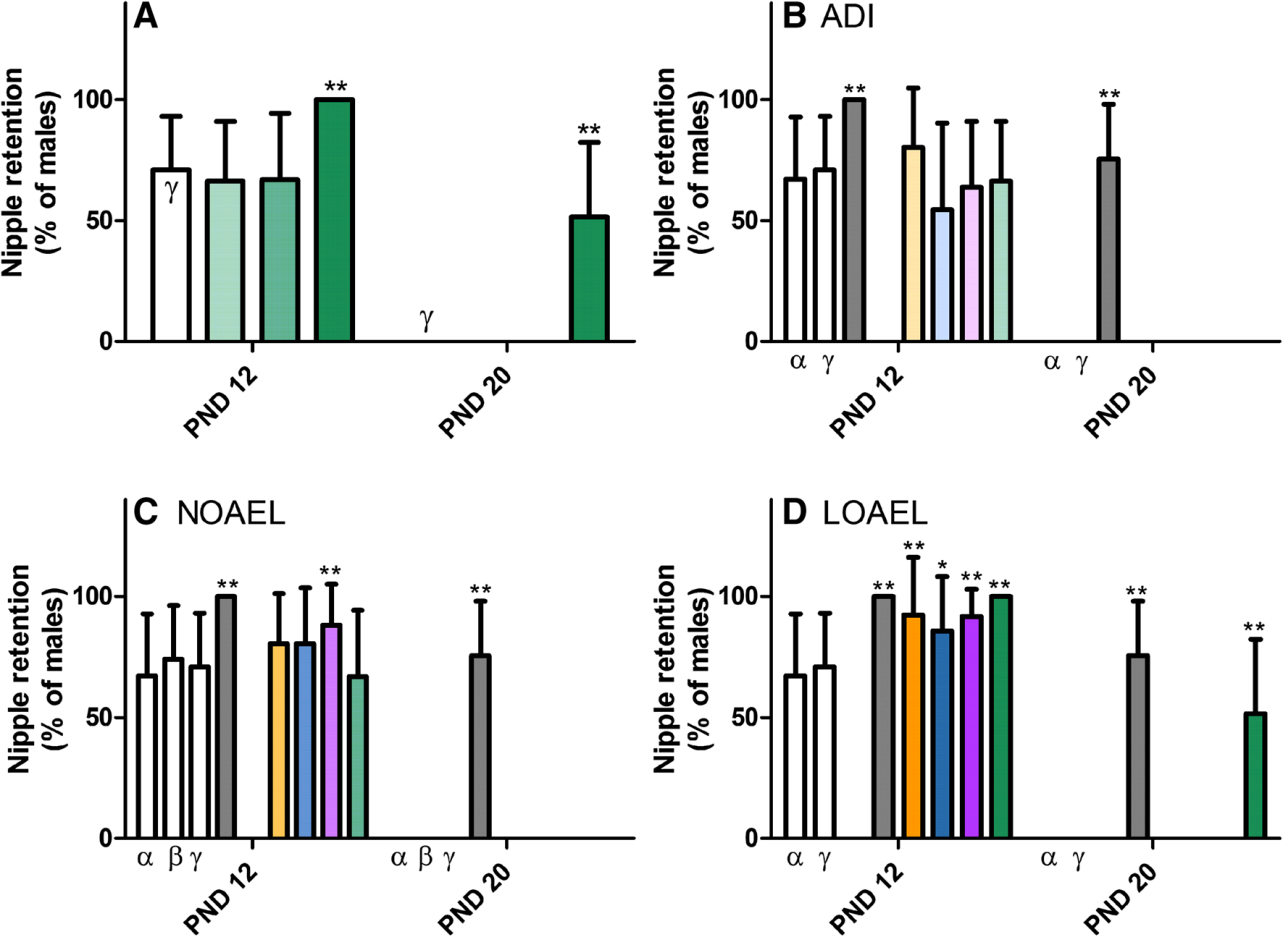
Effect of increasing single-substance and mixed anti-androgen exposures on the incidence of male pups with nipples or areolae. On PND 12, the number of male pups exposed to the vehicle control *open rectangle*, ADI-MIX *filled very light green rectangle*, NOAEL-MIX *filled light green rectangle* and LOAEL-MIX *filled thick green rectangle*, which had retained nipples or areolae was counted. These animals were then recounted on PND 20 (**a**). These mixture results were then compared to the effects of the single-substance exposures to vinclozolin *filled yellow rectangle*, flutamide *filled blue rectangle* and prochloraz *filled pink rectangle* at each dose level: ADI (**b**), NOAEL (**c**) and LOAEL (**d**) as well as vehicle *open rectangle* and positive controls *filled grey rectangle*. (Data are shown as litter mean ± SD.) Despite the relatively high background rate of areola retention in control animals, prenatal and/or lactational exposure to the LOAEL-MIX, but not the ADI or NOAEL mixes, statistically significantly increased the number of male pups with nipple/areola retention on PND 12. This effect was somewhat transient; by PND 20, only the positive control and LOAEL-MIX dose groups had males with nipples/areolae. Exposure to the LOAEL-MIX of these anti-androgens, but not the single-substance exposures, significantly increased areola retention in male pups past PND 20. These data were collected in the course of three different experiments with similar study design. For clarity, only the concurrent controls relating to the exposures at each dose level are depicted in each graph. A comparison of the three control datasets can be found in **c** of Supplementary Figure 1 (color figure online)

**Fig. 5:**
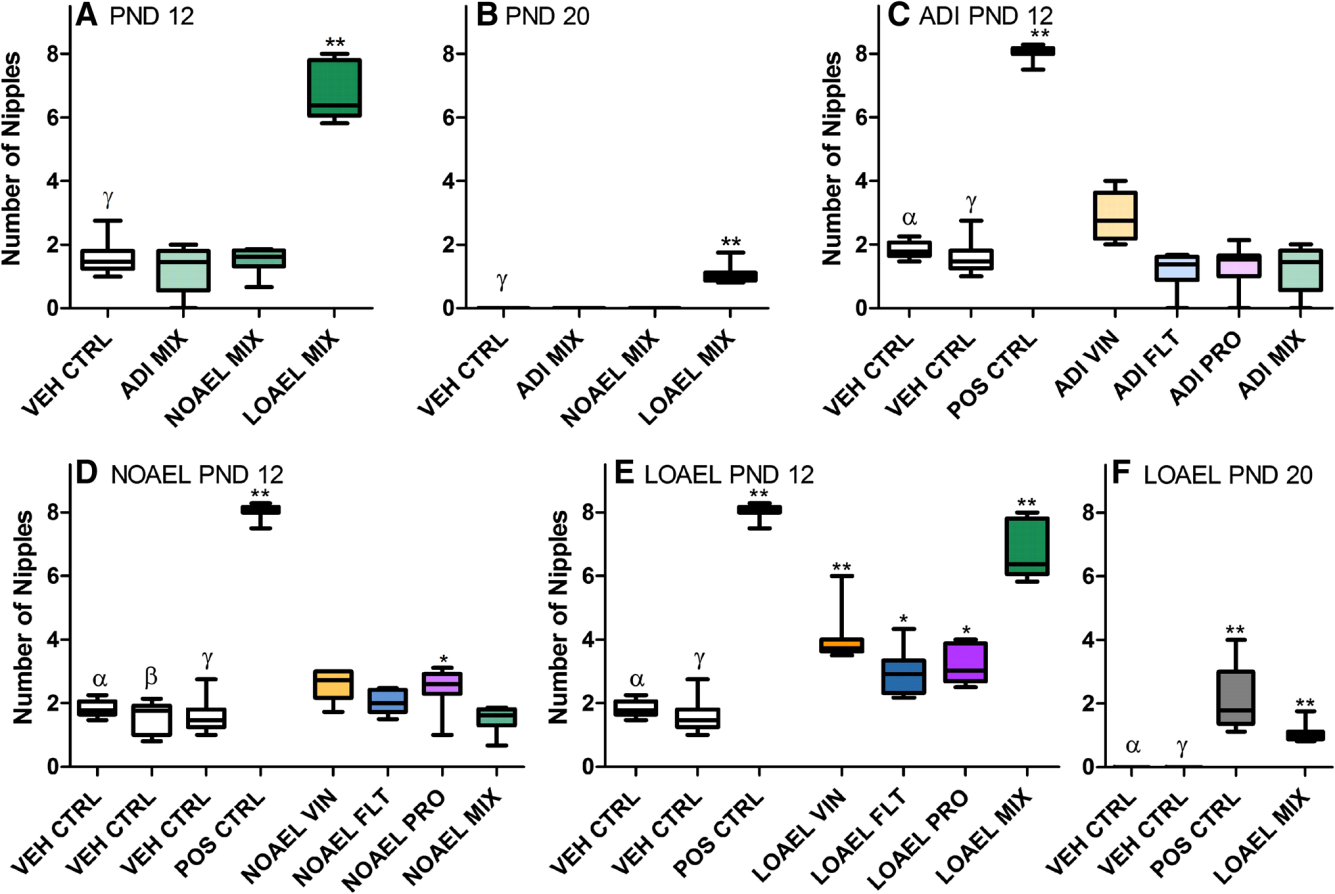
Effect of increasing dose of mixtures of vinclozolin, flutamide, and prochloraz on the number of nipples/areolae on the male pups. On PND 12, the number of remaining nipples or areolae on each male pup exposed to the vehicle control *open rectangle*, ADI-MIX *filled very light green rectangle*, NOAEL-MIX *filled light green rectangle* and LOAEL-MIX *filled thick green rectangle* were counted (**a**). These animals were then recounted on PND 20 (**b**). The mixture results on PND 12 were then compared to the effects of the single-substance exposures to vinclozolin *filled yellow rectangle*, flutamide *filled blue rectangle* and prochloraz *filled pink rectangle* at each dose level: ADI (**c**), NOAEL (**d**) and LOAEL (**e**), as well as vehicle *open rectangle* and positive controls *filled grey rectangle*. (Data are presented as *boxplots* indicating the median [*bar*], interquartile range [*box*] and range [*whiskers*].) Prenatal and/or lactational exposure to the LOAEL-MIX, but not the ADI or NOAEL mixes, statistically significantly increased the number of nipples or areolae in male pups on PND 12. By PND 20 (**f**), most of these had completely receded. Only the positive control and LOAEL-MIX males still had nipples/areolae, but the number remaining was significantly reduced. Nonetheless, no retained areolae were observed in the single-substance exposure groups on PND 20, thus this observation represents an important difference between the LOAEL-MIX and its anti-androgen components. These data were collected in the course of three different experiments with similar study design. For clarity, only the concurrent controls relating to the exposures at each dose level are depicted in each graph. A comparison of the three control datasets can be found in **d** of Supplementary Figure 1 (color figure online)

While male rats are born with mammary areolae like their female siblings, shortly after birth they undergo a post-natal increase in androgen levels, sometimes termed ‘mini puberty’, which has been shown to influence male external development, including triggering areola regression (Kratochwil 1971; Kratochwil and Schwartz 1976; Foster and McIntyre 2002). Peak regression occurs around PND 12–14; therefore, significant retention of the areolae beyond this age may be an external indication of disturbances in androgen signaling. All male pups exposed to the LOAEL-MIX had retained at least one areola or hairless spot along the milk line until PND 12; this was consistent with the areola retention observed after single-substance LOAEL exposures (Fig. 4 and Supplementary Table 3). The numbers of areolae or hairless spots were also substantially increased on PND 12 in the male pups of the single compound LOAEL dose groups (Fig. 5 and Supplementary Table 3), compared to the controls. In the single-substance exposure groups pups retained 3–4 of the possible 12 areolae (compared to approximately 2 in the control animals), whereas in the LOAEL-MIX males had on average 6–8 areolae remaining on PND 12. These data reveal that areola regression occurred in all LOAEL groups, but was less in the LOAEL-MIX group indicating a contribution of multiple compounds, rather than a single dominating compound. Due to the nature of the data, the Loewe model could not be used to assess the quantitative nature of the interaction (i.e. additive, less than additive or more than additive).

Increased nipple retention was not observed after exposure to the NOAEL or ADI mixes. The incidence of males with at least one nipple/areola remaining was slightly elevated in the NOAEL prochloraz group, but the number of nipple/areolae retained is similar to the other NOAEL exposures, as well as the range provided by the three concurrent control groups. Moreover, as no increase in nipple parameters was noted with the current NOAEL-MIX treatment this observation is not considered to be biologically relevant (could be a consequence of the lower variation in the concurrent control alpha, rather than a true anti-androgenic effect of prochloraz treatment at this dose).

Owing to the high background incidence of areola retention on PND 12, we habitually reexamine all male pups for residual areolae on PND 20, by which age the process of regression should be completed. Indeed, no males in the vehicle control, ADI-MIX, NOAEL-MIX, or any of the single-substance exposure groups still had areolae. However, around half the males of the LOAEL-MIX exposure group still had at least a single areola, but the number of areolae retained had reduced dramatically to an average of one per individual. Overall, these data indicate that the areolae did recede substantially by PND 20 and that much of the areola retention is a transient effect. For the individual compounds, areola/nipples were not observed at LOAEL LEVEL at PND 20.

To follow-up on these observations, the male offspring of this exposure group (as well as their corresponding concurrent controls) selected for Subsets 2 and 3 (all other offspring having been killed on PND 21) was re-examined for areolae on PND 38 and again on the day of sexual maturation. No change in the incidences of areola retention or the areola counts were observed at either time point in these animals. These data suggest that the areolae retained until PND 20 are permanent. Thus, the LOAEL mixture of vinclozolin, flutamide, and prochloraz was able to permanently alter male areola regression, while only transient effects observed after single-substance exposures. Otherwise, permanent nipples were only observed in the positive control group (i.e. flutamide dosed at 2.5 mg/kg bw/d). Therefore, the lack of disappearance of the areolae in the LOAEL-MIX treatment group indicates a combination effect of the substances when these are mixed. The nature of the data (no areolae in all single-substance exposure groups and two mixture groups), unfortunately, does not allow for the determination on the type of mixture toxicity.

#### Sexual maturation

**Fig. 6:**
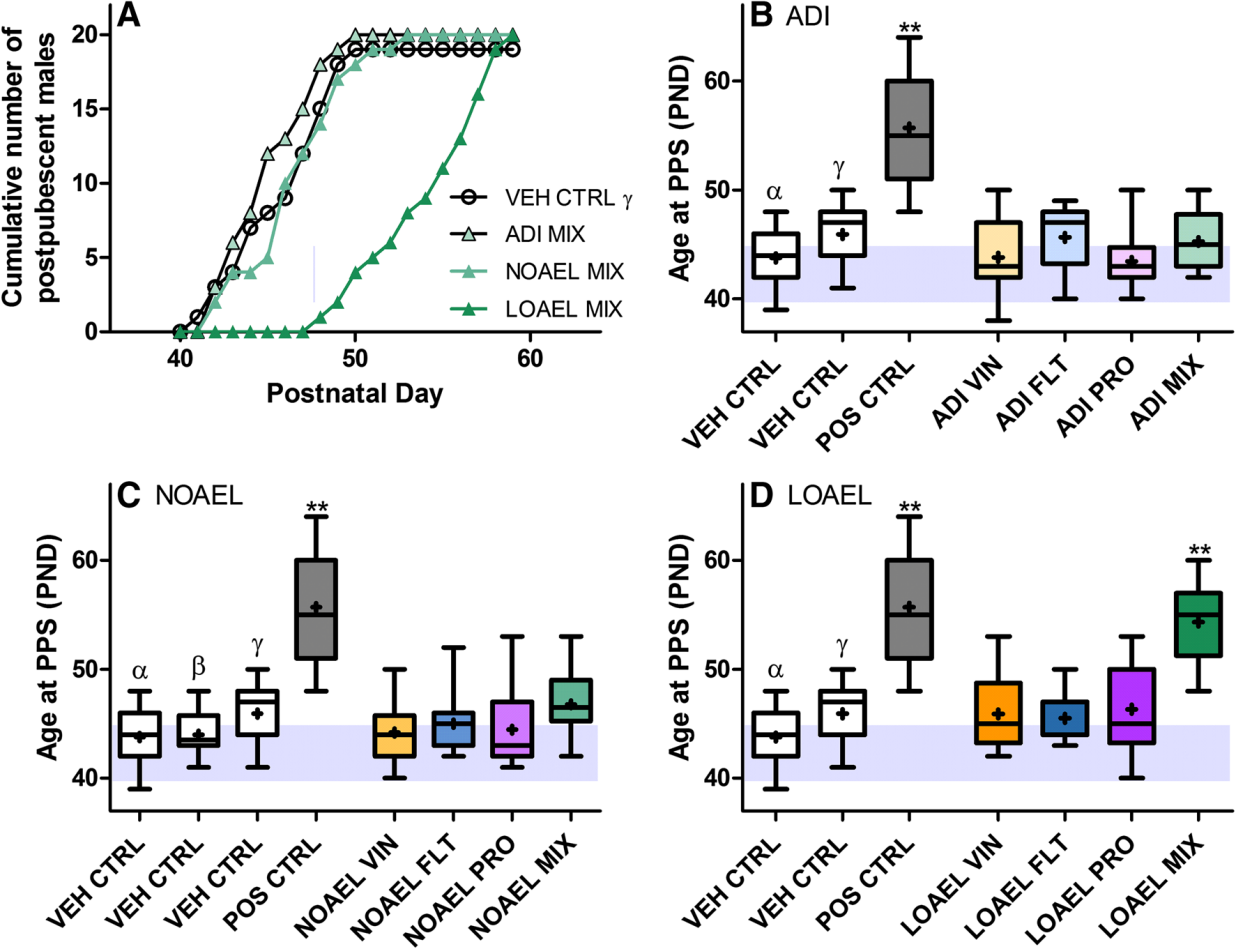
Sexual maturation of male rats exposed to mixtures of vinclozolin, flutamide, and prochloraz. Twenty male offspring per exposure group were examined daily for the onset of external puberty from PND 38–64. The exact day of preputial separation was recorded for every male exposed to vehicle (*open circle*), ADI-MIX (*filled light green triangle with black border*), NOAEL-MIX (*filled light green triangle*), or LOAEL-MIX (*filled thick green triangle*); the cumulative results are presented here as a Kaplan–Meier plot (**a**). These mixture results were then compared to the effects of the single-substance exposures to vinclozolin, flutamide and prochloraz at each dose level: ADI (**b**), NOAEL (**c**) and LOAEL (**d**), as well as vehicle and positive controls. (Data are presented as *boxplots* indicating the median [*bar*], interquartile range [*box*] and range [*whiskers*].) Exposure to the LOAEL-MIX, but not the ADI or NOAEL mixes, statistically significantly delayed preputial separation in the developing offspring. Although statistically insignificant, the means (*plus sign*) of the males from the LOAEL single-substance exposures were also delayed beyond the historical control range (*shaded area*) for this rat strain. While biologically relevant, these delays were not nearly as dramatic as the delay observed when these substances were mixed. These data were collected in the course of three different experiments with similar study design. For clarity, only the concurrent controls relating to the single-substance exposures at each dose level are depicted in each graph. A comparison of the three control datasets can be found in **e** of Supplementary Figure 1 (color figure online)

Each pup, which was selected to become a part of subset 2 or 3 was also evaluated for commencement of sexual maturity, as indicated by vaginal opening in the females and balano-preputial separation in the males. No effect on the age at female vaginal opening was observed in any dose group, nor were any delays in preputial separation noted as a consequence of NOAEL- or ADI-level treatments. However, a statistically significant delay in the onset of preputial separation was observed because of LOAEL-MIX treatment (Fig. 6 and Supplementary Table 3); the mean age at male sexual maturation was nearly 10 days beyond the historical control range and about 9 days beyond the statistically insignificant delays resulting from the single-substance LOAEL exposures.

Using the Loewe additivity model to assess the quantitative nature of the interaction, with data being normalized to the concurrent study controls, the obtained value of 0.772 (95% confidence interval being 0.569–0.976) indicates a slightly more than additive effect. The scatter plot in Supplementary Figure 29 exhibits that the effect of LOAEL-MIX treatment approaches the effect of positive control dose of flutamide. Using the dose–response relationship for flutamide (including the positive control dose of 2.5 mg/kg bw/d it can be calculated that the effect of the mixture group on the onset of preputial separation is equivalent to a flutamide dose of 1.72 mg/kg bw/d.

#### Role of hormone disruption in anti-androgen delayed sexual maturation

While it is true that treatment-related delays in preputial separation may be indicative of an endocrine mechanism of toxicity, impaired general growth has also been shown to alter the onset of puberty (Melching-Kollmuß et al. 2014). To separate any specific substance-dependent effects on preputial separation from the non-specific substance-dependent effects on general growth, we evaluated the body weights of individual animals on the day of sexual maturation. These individual results were then compared to the mean normal body weight development of the control animals from subset 3 (PND 83 ± 2), which were chosen because they were the only ones to be raised beyond puberty. To generate this growth curve, the ages and body weights from the PND 83 controls from the three similar studies were pooled (gray diamonds, shown as mean ± standard deviation) and analyzed by least mean squares regression. Thus, by this method we are able to assess whether changes observed in the day of preputial separation are secondary to alterations in body weight.

**Fig. 7:**
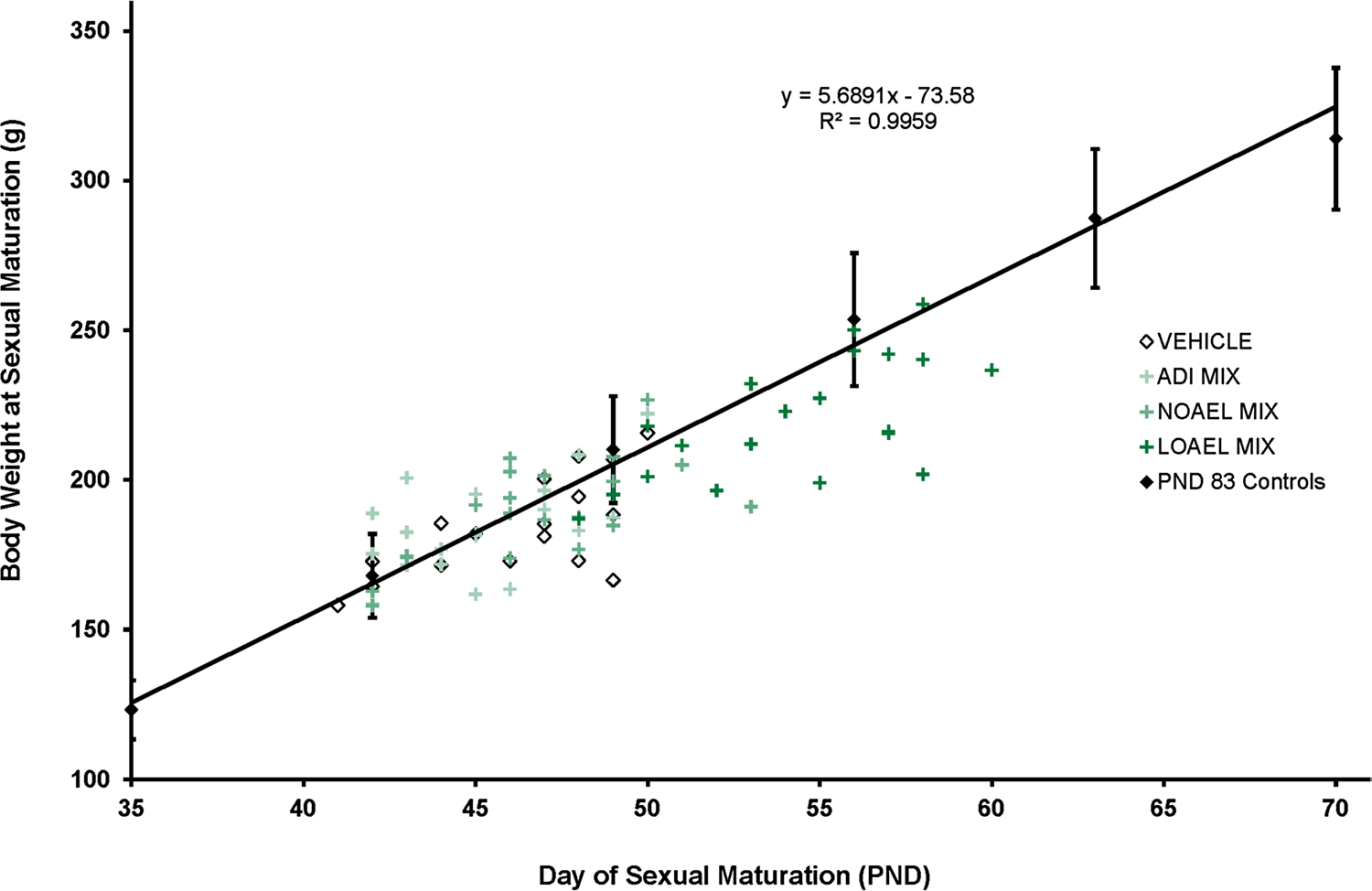
Comparison of age and body weight of individual male offspring at sexual maturation (Subsets 2 and 3). The body weights (*y* axis) of individual male offspring exposed to the vehicle control (*open diamond*), positive control (*filled black diamond*), the LOAELs of vinclozolin (*filled yellow square*), flutamide (*filled blue triangle*), or prochloraz (*filled pink diamond*), or the LOAEL-MIX (*filled green plus sign*) were plotted against their corresponding ages on the day of preputial separation. These scatterplots were then compared to the normal body weight development of Subset 3 control animals (*filled grey diamond* ± SD). For clarity, only the controls or treatment groups with delayed preputial separation are shown. Overall, this plot indicates that the males with delayed sexual maturation were heavier in proportion to their increased age, suggesting that these delays were not a secondary effect of systemic toxicity. However, a closer examination of the individuals exposed to the LOAEL dose of prochloraz reveals that these animals are slightly underweight for their ages. Thus, while much of the delay in preputial separation in this dose group is due to the anti-androgen effects of this substance, it is probable that there is also some contribution from a generally slowed development due to non-specific prochloraz toxicity (color figure online)

Overall, the evaluation performed, as shown in Fig. 7, indicates that the males with delayed sexual maturation were heavier in proportion to their increased age, suggesting that these delays were not a secondary effect of systemic toxicity. However, a closer examination of the individuals exposed to the LOAEL dose of prochloraz reveals that these animals are slightly underweight for their ages. Thus, while much of the delay in preputial separation in this dose group is due to the anti-androgen effects of this substance, it is probable that there is also some contribution from a generally slowed development due to non-specific prochloraz toxicity, as has been discussed previously (Melching-Kollmuss et al. 2017).

### Estrous cycle and sperm analyses

Estrous cycle data were generated for all young adult females during the 3 weeks prior to necropsy. These observations revealed regular cycles in the females of all test groups including the control. The mean estrous cycle duration was also similar across all test groups: 5.2 days after treatment with the vehicle, 6.3 days in the ADI-MIX, 6.0 days in the NOAEL-MIX and 6.8 days in the LOAEL-MIX groups. These data correlated very well with similar observations from the single-substance exposures. Similarly, sperm and spermatids were collected from young adult (PND 83 ± 2) male offspring at killing, and were analyzed as a metric for male fertility (Tables 4, 5 and 6). The results from these analyses indicate no change in sperm parameters with mixtures treatment at any dose level. For prochloraz tested as a single substance, there was a borderline, but statistically significant decrease in sperm motility at the LOAEL dose. Taken these results of the mixture study into account, it is unlikely that this observation was test substance related, as previously assessed (Melching-Kollmuss et al. 2017).

**Table 4:** Statistical analyses used in the assessment of measured parameters

Parameter	Statistical test
Food consumption, body weight and body weight change (parental animals and pups); estrous cycle length; duration of gestation; number of delivered pups per litter; developmental landmarks (days up to preputial separation or opening of the vagina); anogenital distance and index; implantation sites; post-implantation loss, weight of the fetuses, implantations, pre- and post-implantation losses, resorptions and live fetuses	DUNNETT’s test (two-sided)
Number of live and dead pups and different indices (e.g., mating index, fertility index and gestation index) and number of litters with necropsy findings in pups; developmental landmarks (preputial separation or opening of the vagina), sperm morphology, incidence of males with a specific amount of abnormal sperm (cutoff value: 0.9-quantile [90%] of control groups)	FISHER’s exact test
Proportion of pups with necropsy findings per litter, presence of areolas/nipples, sperm evaluation (with Bonferroni–Holms correction.	WILCOXON test (one-sided)
Weight of the anesthetized animals and absolute and relative organ weights (all organs excl. organs listed below); hormones	KRUSKAL–WALLIS and WILCOXON or Mann–Whitney *U* test (latter for hormones)
Weight parameters of ventral prostate (VP), seminal vesicles with coagulating gland (SVCG), Musc. levator ani together with Musc. bulbocavernosus (LABC), Cowpers gland (Bulbourethral gland) (COW), glans penis (GP)	DUNNETT’s test (one-sided)

**Table 5:** Effects of single-substance and mixed exposures to anti-androgens on reproductive performance

	Remaining dams pregnant	Duration of Gest. (d)	Litters with livebirths/stillbirths/all stillbirths	Live-born/stillborn pups	Pups died/cannibalized	Males PND 0/PND 21 (%)
Vehicle control^α^	20	22.1	20/1/0	208/1	0/0	50.0/50.0
Vehicle control^β^	19	21.7	19/0/0	183/0	0/0	53.0/53.0
Vehicle control^γ^	20	22.0	20/1/0	192/1	0/0	46.9/46.9
Positive control^α^	20	22.0	20/0/0	202/0	3/2	44.1/43.9
VIN ADI^α^	19	22.0	19/0/0	191/0	0/1	52.9/52.6
VIN NOAEL^α^	18	22.1	18/0/0	177/0	0/2	52.0/52.0
VIN LOAEL^α^	19	22.1	18/1/0	182/1	0/1	45.6/45.9
FLT ADI^γ^	19	22.1	19/2/0	172/5	1/1	48.3/48.8
FLT NOAEL^β^	19	21.7	19/0/0	171/0	1/1	49.7/50.6
FLT LOAEL^α^	20	22.2	20/1/0	205/5	2/7**	48.3/49.5
PRO ADI^α^	20	22.0	20/2/0	201/2	0/1	50.2/50.3
PRO NOAEL^α^	19	21.9	19/2/0	205/2	0/0	54.6/54.6
PRO LOAEL^α^	19	22.9**	17/10**/2	146**/42**	8**/7**	55.5/57.7
ADI-MIX^γ^	20	21.8	20/1/0	198/1	0/0	42.9/42.9
NOAEL-MIX^γ^	20	22.3*	20/2/0	200/2	7**/6*	51.0/51.9
LOAEL-MIX^γ^	20	22.9**	17/8**/2	153**/26**	0/3	53.6/54.0

Data are presented as mean ± SD (*N*)

* *p* ≤ 0.05

** *p* ≤ 0.01

^α^ indicates that statistical comparison was performed against concurrent control group α

^β^ indicates that statistical comparison was performed against concurrent control group β

^γ^ indicates that statistical comparison was performed against concurrent control group γ

**Table 6:** Analysis of sperm and spermatids of male offspring (PND 83 ± 2)

Treatment	Motile sperm	Abnormal sperm morphology	Spermatid count (×10^6^/g testes)	Spermatozoa count (×10^6^/g cauda epididymis)
Vehicle control^α^	88 ± 4% (10)	3 ± 3% (10)	190 ± 31.37 (10)	566 ± 114 (10)
Vehicle control^β^	89 ± 4% (10)	6 ± 0% (10)	132 ± 29 (10)	515 ± 103 (10)
Vehicle control^γ^	91 ± 6% (9)	2 ± 1% (9)	199 ± 23 (9)	643 ± 100 (9)
Positive control^α^	82* ± 6% (9)	6 ± 6% (9)	186 ± 76 (10)	493 ± 185 (10)
VIN ADI^α^	89 ± 5% (10)	3 ± 1% (10)	192 ± 17 (10)	622 ± 101 (10)
VIN NOAEL^α^	87 ± 6% (10)	4 ± 2% (10)	215 ± 31 ( (10)	638 ± 144 (10)
VIN LOAEL^α^	84 ± 6% (10)	3 ± 2% (10)	193 ± 23 (10)	544 ± 178 (10)
FLT ADI^γ^	92 ± 6% (10)	2 ± 1% (10)	233 ± 49 (10)	677 ± 125 (10)
FLT NOAEL^β^	90 ± 5% (10)	6 ± 0% (10)	118 ± 14 (10)	488 ± 84 (10)
FLT LOAEL^α^	86 ± 5% (10)	3 ± 2% (10)	188 ± 28 (10)	616 ± 138 (10)
PRO ADI^α^	85 ± 5% (10)	2 ± 1% (10)	191 ± 19 (10)	641 ± 156 (10)
PRO NOAEL^α^	86 ± 4% (10)	3 ± 2% (10)	191 ± 32 (10)	535 ± 127 (10)
PRO LOAEL^α^	83** ± 3% (10)	4 ± 3% (10)	198 ± 20 (10)	590 ± 129 (10)
ADI-MIX^γ^	88 ± 8% (10)	2 ± 2% (10)	209 ± 32 (10)	614 ± 110 (10)
NOAEL-MIX^γ^	92 ± 4% (10)	2 ± 1% (10)	213 ± 27 (10)	725 ± 188 (10)
LOAEL-MIX^γ^	92 ± 6% (10)	2 ± 1% (10)	198 ± 22 (10)	731 ± 262 (10)

Data are presented as mean ± SD (*N*)

* *p* ≤ 0.05

** *p* ≤ 0.01

^α^ Indicates that statistical comparison was performed against concurrent control group α

^β^ Indicates that statistical comparison was performed against concurrent control group β

^γ^ Indicates that statistical comparison was performed against concurrent control group γ

#### Hormone analysis

The steroid hormones were graphed as boxplots for easy comparison, although due to the volume of data, only selected hormone levels in young adult offspring (PND 83 ± 2) are shown here (Figs. 8, 9). The remaining data can be found in Supplementary Figures 7–27 and Supplementary Tables 24–37.

**Fig. 8:**
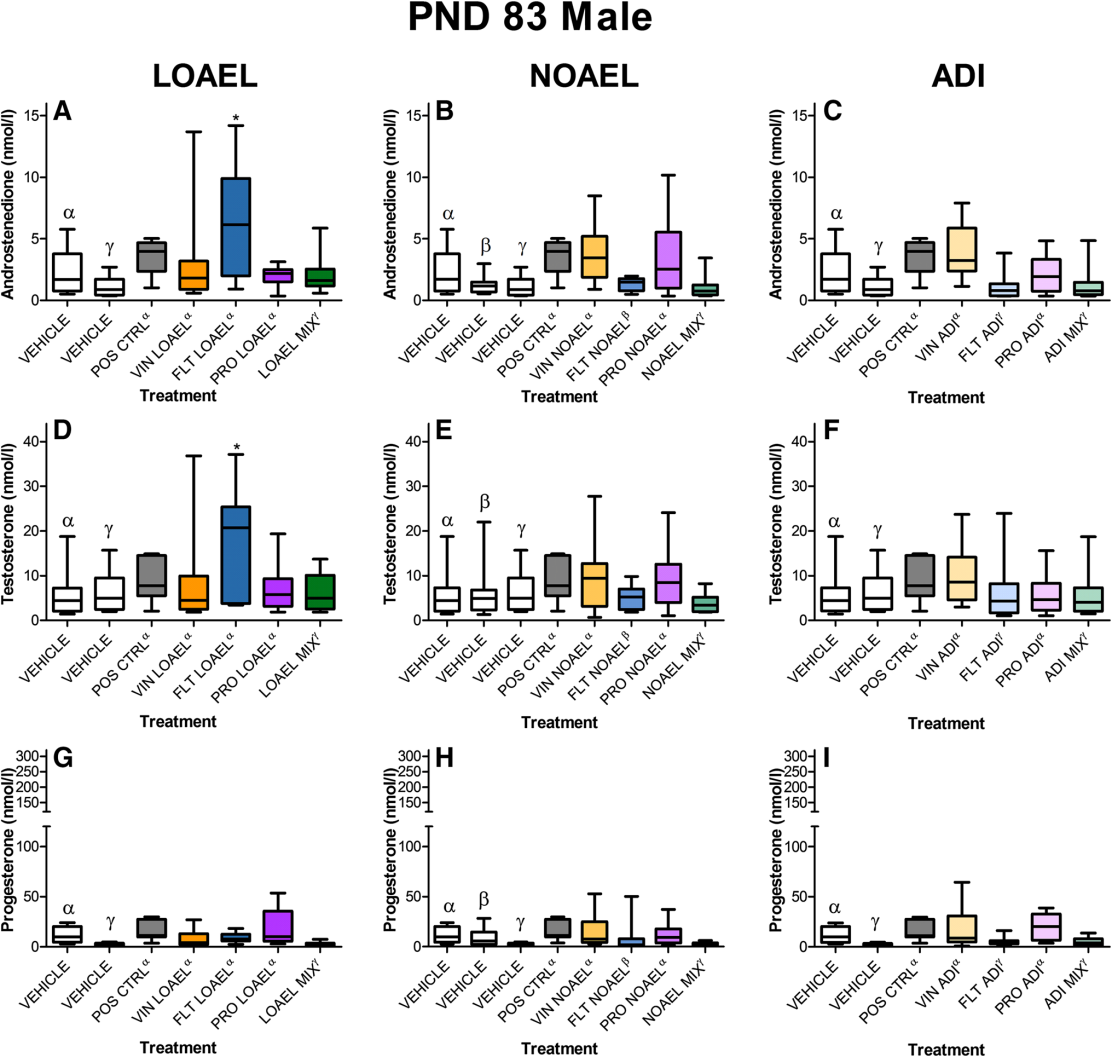
Concentrations of selected steroid hormones in young adult males exposed to anti-androgens. Circulating steroid hormone concentrations were measured in the serum taken from ten male offspring on PND 83 ± 2 using LC–MS/MS. The serum concentrations of the mixture-treated animals *filled thick green rectangle* were then compared to the effects of the single-substance exposures to vinclozolin *filled yellow rectangle*, flutamide *filled blue rectangle* and prochloraz *filled pink rectangle* at each dose level: LOAEL (*left panels*), NOAEL (*center panels*) and ADI (*right panels*) as well as vehicle *open rectangle* and positive controls *filled grey rectangle*. Data are graphed as *boxplots* representing the median (*bar*), interquartile range (*box*) and range (*whiskers*) for each treatment group. Each graph represents a different steroid hormone and dose level. ^*α,β,γ*^These data were collected in the course of three different experiments with similar study design; for the purposes of statistical testing, each treatment group was only compared to its corresponding concurrent control as indicated by the superscript. As not all dose levels were evaluated in all studies, only the concurrent controls relating to the exposures are depicted in each panel. Due to the data volume, only the most relevant sex steroids are shown here; a complete set may be found in the Supplementary Figures 7–27. A comparison of the hormone levels in control males (Supplementary Figure 2) is also available (color figure online)

**Fig. 9:**
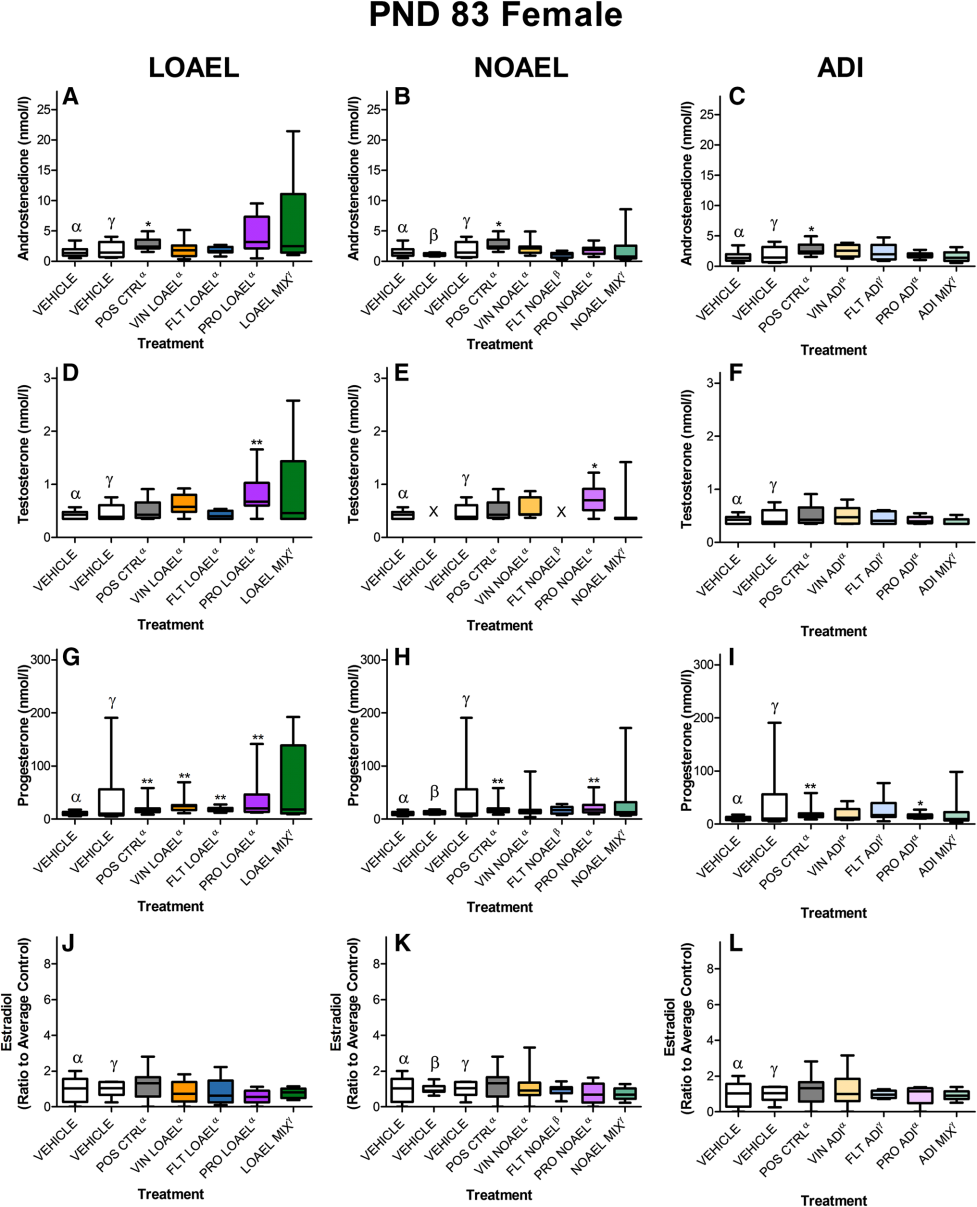
Concentrations of selected steroid hormones in young adult females exposed to anti-androgens. Circulating steroid hormone concentrations were measured in the serum taken from ten female offspring on PND 83 ± 2 using LC–MS/MS. The serum concentrations of the mixture-treated animals *filled thick green rectangle* were then compared to the effects of the single-substance exposures to vinclozolin *filled yellow rectangle*, flutamide *filled blue rectangle* and prochloraz *filled pink rectangle* at each dose level: LOAEL (*left panels*), NOAEL (*center panels*) and ADI (*right panels*) as well as vehicle *open rectangle* and positive controls *filled grey rectangle*. Data are graphed as *boxplots* representing the median (*bar*), interquartile range (*box*) and range (*whiskers*) for each treatment group. Each graph represents a different steroid hormone and dose level. ^*α,β,γ*^These data were collected in the course of three different experiments with similar study design; for the purposes of statistical testing, each treatment group was only compared to its corresponding concurrent control as indicated by the superscript. As not all dose levels were evaluated in all studies, only the concurrent controls relating to the exposures are depicted in each panel. Due to the data volume, only the most relevant sex steroids are shown here; a complete set may be found in Supplementary Figures 7–27. A comparison of the hormone levels in control females (Supplementary Figure 3) is also available (color figure online)

A global assessment of the serum hormone measurements from all dose groups and developmental time points revealed a few key observations about this dataset. For the most part, the effect of continued development was clear in the sex hormone levels of both male and female offspring. Passage through puberty increased male androstenedione and testosterone levels, as well as female progesterone and estradiol levels. This trend was observed largely regardless of the dose group. In other words, the chance in hormone level over time was more profound than any treatment-related effects.

When treatment and control groups were compared at the same developmental stage, relatively few changes in the serum steroid hormone concentrations were observed. Moreover, little consistency was noted when comparing these treatment-related changes with those from same dose group at other developmental stages; and when the mixed- and single-substance exposure data were compared.

With all this in mind, we noted only a few changes in the juvenile animals, which seemed to be due to treatment. For instance, decreased progesterone levels were observed in male, but not female; NOAEL-MIX and LOAEL-MIX offspring killed on PND 21. No findings were observed in the ADI-MIX group in either gender, nor were any effects at all observed at puberty.

The clearest picture of hormonal changes was observed in adult animals. Androstenedione levels were statistically insignificantly increased and estradiol levels mildly but statistically significantly decreased in the parental female animals treated with the LOAEL-MIX. Estradiol levels were also found to be decreased in the females of the NOAEL-MIX group. When compared to the prochloraz-only results, which is known to have aromatase inhibiting properties, statistically significantly increased testosterone levels and not significantly decreased estradiol levels were seen in the prochloraz LOAEL group, while significantly increased testosterone was also seen in the prochloraz NOAEL group (Melching-Kollmuss et al. 2017). However, without any concurrent effect on reproductive parameters or other apical end points, these hormone level changes are regarded as suggestive of a start of an effect of the compound on the organisms, but not as adverse in nature, because of no functional or anatomical alteration. The hormone changes were mirrored in the subset 3 offspring which lived to early adulthood (PND 83 ± 2), where serum androstenedione concentrations were statistically insignificantly elevated in both male and female offspring treated with the LOAEL-MIX, but not the NOAEL- or ADI-mixes (Table 7). All other findings were considered spurious, as explained in the Supplementary Information.

**Table 7:** Blood hormone changes in dams after weaning in proestrus and in F1 male and female pups at PND21, sexual maturity and around PND83 (in females in proestrus) in dosed rats compared to controls

	Males			Females			
	Progesterone	Androstenedione	Testosterone	Progesterone	Androstenedione	Testosterone	Estradiol
ADI							
Prochloraz							
F0 dams				=	=	=	=
F1 PND22 pups	=	=	=	=	=	nm	nm
F1 sexual maturity	=	=	=	=	=	=	=
F1 PND83	=	=	=	=	=	=	=
Flutamide							
F0 dams				=	=	=	=
F1 PND22 pups	=	=	=	=	=	nm	nm
F1 sexual maturity	=	=	=	=	=	=	=
F1 PND83	=	=	=	=	=	=	=
Vinclozoline							
F0 dams				=	=	=	=
F1 PND21 pups	=	=	=	=	=	nm	nm
F1 sexual maturity	=	=	=	=	=	=	=
F1 PND83	=	=	=	=	=	=	=
Mixture							
F0 dams				=	=	=	=
F1 PND21 pups	=	=	=	=	=	nm	nm
F1 sexual maturity	=	=	=	=	=	=	=
F1 PND83	=	=	=	=	=	=	=
NOAEL							
Prochloraz							
F0 dams				=	=	↑	=
F1 PND21 pups	=	=	=	=	=	nm	nm
F1 sexual maturity	=	=	=	=	=	=	=
F1 PND83	=	=	=	=	=	↑	=
Flutamide							
F0 dams				=	=	=	=
F1 PND21 pups	=	=	=	=	=	nm	nm
F1 sexual maturity	=	=	=	=	=	=	=
F1 PND83	=	=	=	=	=	↑	=
Vinclozoline							
F0 dams				=	=	=	=
F1 PND21 pups	=	=	=	=	=	nm	nm
F1 sexual maturity	=	=	=	=	=	=	=
F1 PND83	=	=	=	=	=	=	=
Mixture							
F0 dams				=	=	=	↓↓
F1 PND21 pups	↓↓	=	=	=	=	nm	nm
F1 sexual maturity	=	=	=	=	=	=	=
F1 PND83	=	=	=	=	=	=	=
LOAEL							
Prochloraz							
F0 dams				=	=	(↑)	(↓)
F1 PND21 pups	=	=	=	=	=	nm	nm
F1 sexual maturity	=	=	=	=	=	=	=
F1 PND83	=	=	=	=	(↑)	↑↑	(↓)
Flutamide							
F0 dams				=	=	=	=
F1 PND21 pups	=	=	=	=	=	nm	nm
F1 sexual maturity	=	↑	↑	=	=	=	=
F1 PND83	=	=	=	=	↑	↑↑	=
Vinclozoline							
F0 dams				=	=	=	=
F1 PND21 pups	=	=	=	=	=	nm	nm
F1 sexual maturity	=	=	=	=	=	=	=
F1 PND83	=	=	=	↑↑	=	=	=
Mixture							
F0 dams				=	(↑)	=	↓↓
F1 PND21 pups	↓↓	=	=	=	=	nm	nm
F1 sexual maturity	=	=	=	=	=	=	=
F1 PND83	=	(↑)	=	=	(↑)	=	=

Dose groups were established with one compound only, or with a mixture of the mentioned three compounds at levels of the ADI (prochloraz 0.01, flutamide 0.25, vinclozolin 0.005 mg/kg bw/d), NOAEL (prochloraz 5, flutamide 0.25, vinclozolin 4 mg/kg bw/d and LOAEL (prochloraz 30, flutamide 2.5, vinclozolin 20 mg/kg bw/d). Hormone levels were measured in about 20 dams per groups and in about 10 F1 pups per group and sex. Only dose-dependent hormone changes beyond the historical control ranges were regarded. = no hormone change, (↑) or (↓) relevant but not statistical significant change, ↑ or ↓ weak significant change (*p* < 0.05), ↑↑ or ↓↓ significant change (*p* < 0.01), nm: not measured. Kruskal–Wallis test followed by two-sided Mann–Whitney *U* or Wilcoxon test were applied. Details are mentioned in the supplementary tables

A clear picture was also obtained by the measurement of testosterone in testes of male pups at GD 20 after ex vivo incubation. The mean testosterone values were statistically significantly decreased in the animals treated with the LOAEL-MIX, but not the NOAEL- or ADI-mixes (Fig. 8), and the variability of the data was lower than seen with serum hormone concentrations.

### Pathology

#### Sex organ weights

**Fig. 10:**
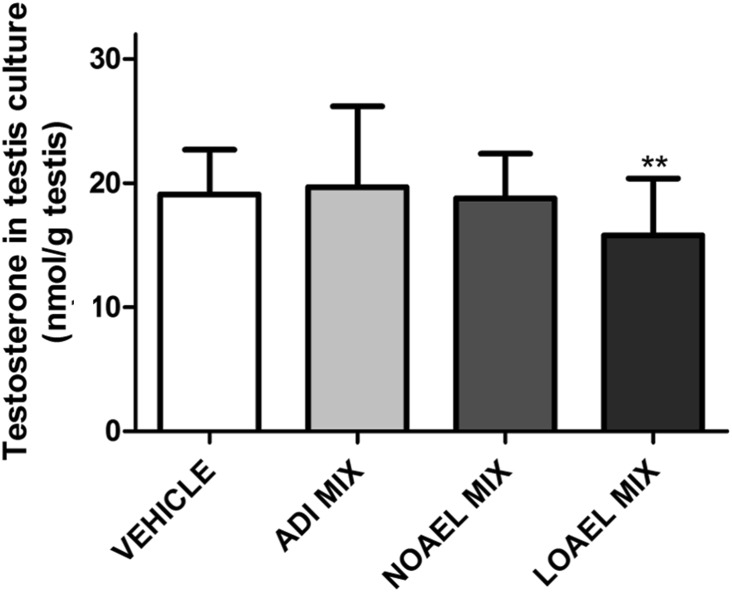
Concentrations of testosterone in both testes of male fetuses at GD 20 exposed to anti-androgens (color figure online)

**Fig. 11:**
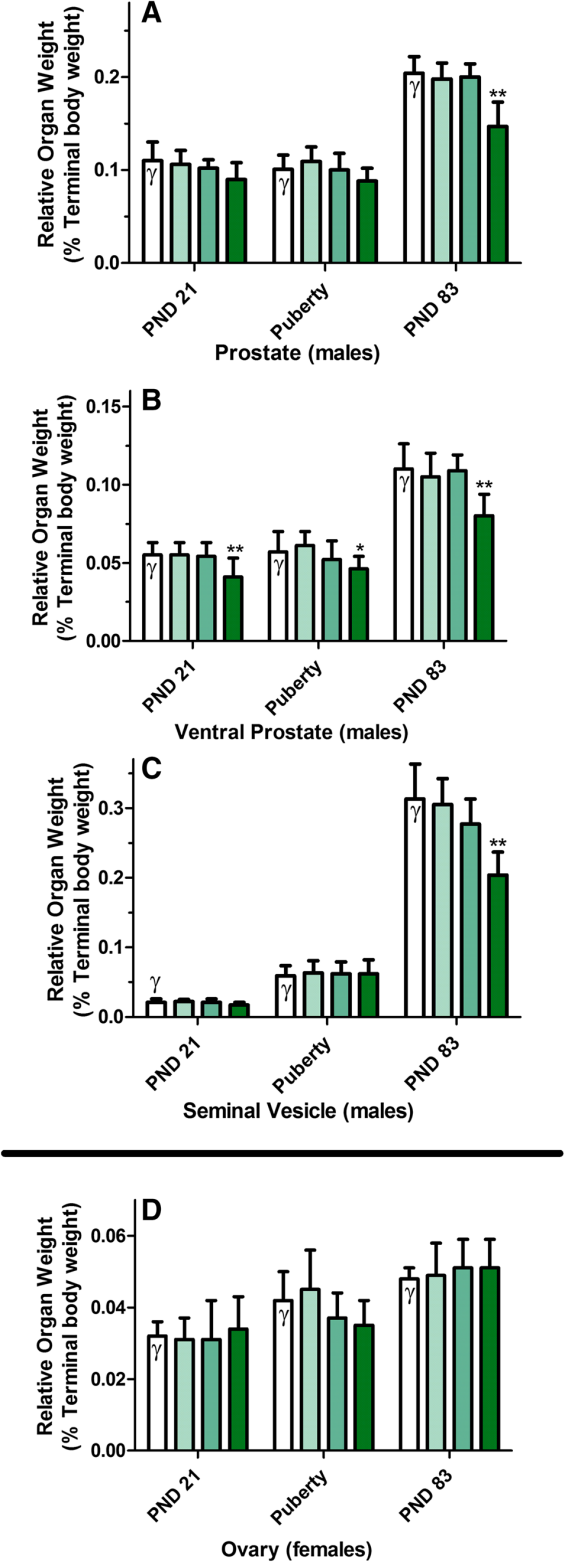
Weights of selected sexual organs of rats exposed to mixtures of vinclozolin, flutamide, and prochloraz. On PND 21 (Subset 1), the day of preputial separation or vaginal opening (Puberty, Subset 2) and PND 83 ± 2 (Subset 3), the sex organs from each of ten male and ten female rats were preserved, weighed and reported as relative organ weights. These data are graphed as vehicle control *open rectangle*, ADI-MIX *filled very light green rectangle*, NOAEL-MIX *filled light green rectangle*, and LOAEL-MIX *filled thick green rectangle* at each time-point. In general, increasing anti-androgen exposures reduced male, but not female, sex organ weights dose-dependently. Three of the most sensitive male sex organs are shown (**a**–**c**) in comparison to a female sex organ (**d**) (color figure online)

After killing, a number of organs, including the reproductive organs, were removed from both the male and female animals of all dose groups and weighed. These absolute organ weights were then used to calculate organ weights relative to animal body weight (Supplementary Figures 5–6 and Supplementary Tables 4–23). A number of organ weight alterations were noted; generally though only the ones observed in the male sexual reproduction organs were considered to be related to treatment. Most were significantly altered as a result of LOAEL-MIX exposure. A variety of these organ weights were consistently reduced either on PND 21 (relative ventral prostate) or in early adulthood (PND 83 ± 2), including bulbourethral gland, cauda epididymis (absolute only), glans penis (absolute only), bulb. and lev. ani muscles, total and ventral prostates and finally the seminal vesicles. The most sensitive of these organ weights are shown as relative weights in Figs. 10, 11 and (dose response) and 12 (comparison to the single-substance exposures); all remaining organ weights are located in Supplementary Figures 5–6 and Supplementary Tables 4–23.

**Fig. 12:**
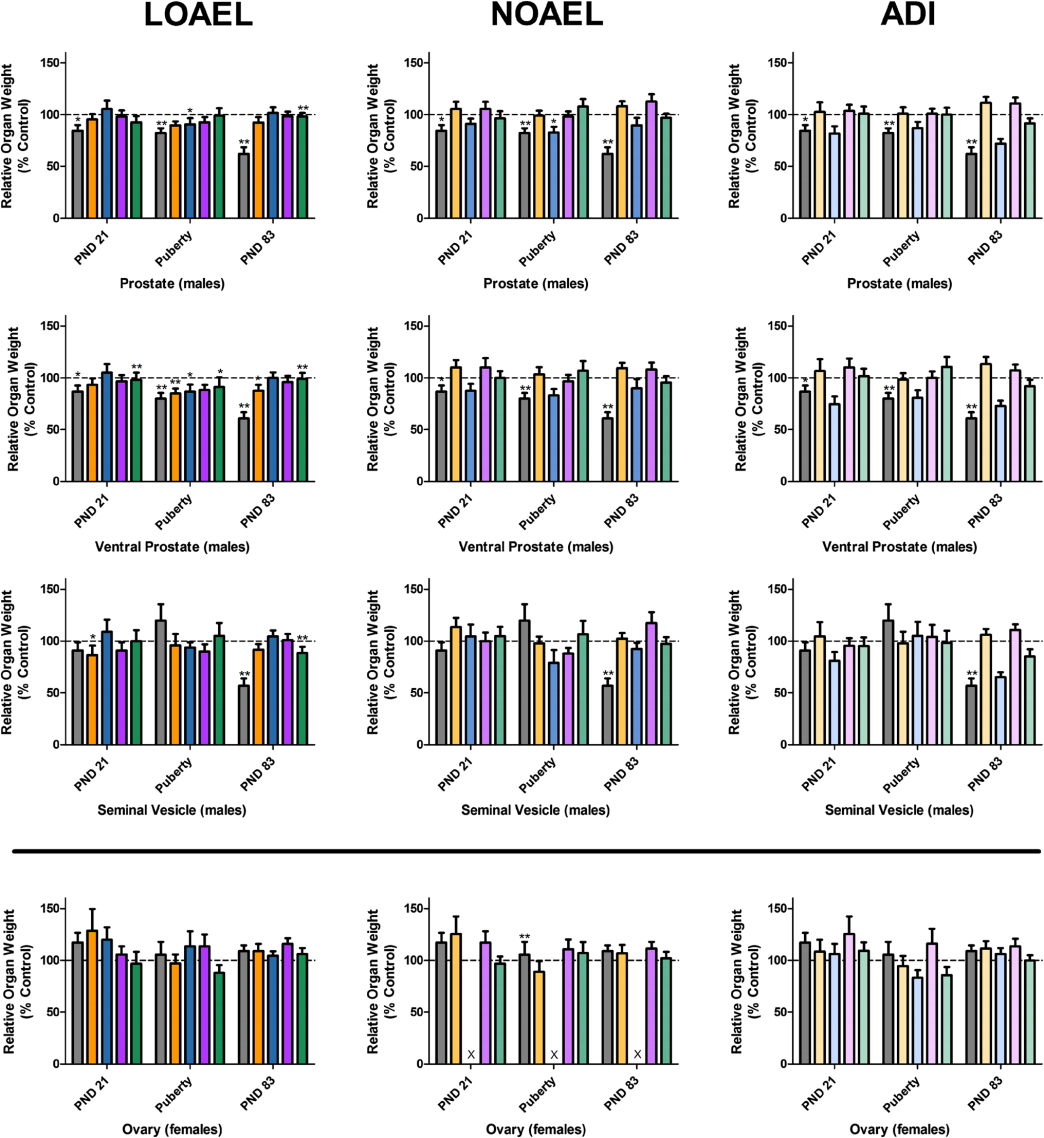
Comparison of the weights of selected sexual organs of male rats exposed to anti-androgens. On PND 21 (Subset 1), the day of preputial separation or vaginal opening (Puberty, Subset 2) and PND 83 ± 2 (Subset 3), the sex organs from each of ten male and ten female rats were preserved, weighed and reported as relative organ weights. The relative organ weights of the mixture-treated animals *filled thick green rectangle* were then compared to the effects of the single-substance exposures to vinclozolin *filled yellow rectangle*, flutamide *filled blue rectangle* and prochloraz *filled pink rectangle* at each dose level: LOAEL (*left panels*), NOAEL (*center panels*) and ADI (*right panels*) as well as vehicle *open rectangle* and positive controls *filled grey rectangle* (Data are shown as mean ± SD). Generally, male sex organ weights were reduced in animals exposed to the anti-androgens at the LOAEL level, but not the NOAEL or ADI. For brevity, only selected organ weights are shown here, the remaining male sex organs can be found as part of Supplementary Figures 5 and 6. These data were collected in the course of three different experiments with similar study design. For clarity, all data have been normalized to the concurrent controls relating to the exposure. A comparison of the three control datasets for all male sex organs, as well as the female ovary weights used for comparison, can be found in Supplementary Figure 4. *X* no control data was available for normalization (color figure online)

The same is true of the male organ weights at puberty, although these must be appraised somewhat cautiously, as they were measured at the same developmental stage (preputial separation) rather than at the same post-partum age, which complicates the issue of biological significance. Given the pubertal delay in the males treated with the LOAEL-MIX, it is understandable that these animals would also be heavier (in proportion to their increased age), resulting in the observed increased terminal body weight at killing when compared to the control group. Furthermore, the increased adrenal gland-, kidney-, liver- and thyroid gland weights in the LOAEL-MIX group are probably also effects of the increased animal size at killing, and therefore, also secondary to the delay in preputial separation. This is confirmed by the fact that the relative adrenal gland-, kidney-, liver- and thyroid gland weights remain unaltered. Thus, while the increased terminal body and absolute organ weights in this dose group are undoubtedly treatment-related effects of the LOAEL-MIX, they should be considered to be indirect effects and not indicative of organ toxicity.

In contrast, no alterations to male sex organ weights were observed in the ADI-MIX dose group. Moreover, it is unlikely that any change observed at the NOAEL-level is related to treatment. A small, but statistically significant decrease was observed in both the absolute weights of the cauda epididymis and the relative weights of bulbourethral glands in young adult male offspring (Subset 3) treated with the NOAEL-MIX. These very slight weight deviations are most likely unrelated to treatment because the absolute and relative weights of these organs are inconsistent with each other, because these organs are not thought to be the organs most sensitive to anti-androgens, and because no other organs are affected in this test group. Moreover, these parameters fell within the range formed by the means of the control animals of the other two preliminary single-substance exposure studies (0.020–0.024). Finally, the histology of epididymides was comparable between control animals and NOAEL-MIX animals, in contrast to differing morphology observed in the epididymides of those offspring treated with the LOAEL-MIX. Taken together, a treatment-related etiology for the decrease in male sex organ weights after NOAEL-MIX exposure seems unlikely.

Using the Loewe additivity model, the effects on ventral prostate weight of the LOAEL mixture group in three subsets were additive in nature, based on their 95% confidence intervals for the interaction index including 1 (see also boxplot in Supplementary Figure 30).

#### Gross and histopathology

All gross pathological findings occurred individually and were of the usual type for Wistar rats. The histopathological investigations of these lesions revealed no ontological cause; therefore, they were considered to be idiopathic without any relationship to treatment. The reproductive organs were also excised from the male offspring at each developmental stage, fixed, stained, and evaluated histopathologically. This assessment also included developmental staging of each tissue.

The reproductive organs of all male offspring on PND 21 (subset 1) were at an immature stage, which was common to all control and treatment groups. However, by the onset of puberty (subset 2), development of these tissues had advanced towards a juvenile stage. The seminal vesicles, coagulating glands as well as prostate (ventral and dorsolateral parts) of all control and treated animals were judged to be at a comparable juvenile stage with a moderate amount of secretion. The left testis was also at a juvenile stage with fully developed spermatogenic cycle. This corresponded to a juvenile stage, either without any sperms or only some sperms in the region of the head, in the epididymal tissues of control males and those exposed to the NOAEL and ADI mixes. In contrast, all animals treated with LOAEL-MIX were at the more advanced so-called juveno-adult transition stage characterized by many sperms in all compartments of the epididymis and a heightened epithelium.

This more advanced development was only a transitory consequence of treatment, as all male reproductive organs examined (left testis, left epididymis, seminal vesicles, coagulating glands, prostate) had developed to full maturity by early adulthood (subset 3, PND 83 ± 2). This mature stage was comparable between the animals of all treatment groups. Further examination of the prostate and seminal vesicles for signs of reduced secretion, revealed no morphological correlate in LOAEL-MIX treated males that might explain the reduced absolute and relative weights of these organs in this dose group. No other histopathological findings were observed in the male reproductive tissues. Furthermore, histopathologic examination of the adrenal and pituitary glands also revealed no findings at any time point.

## Discussion

There are only very few studies available to assess whether mixtures of endocrine active substances alter sexual development at dose levels well below single-substance activity levels, or as mixtures thereof. To address this concern, we performed a series of side-by side pre-/post-natal reproductive toxicity studies to measure the developmental toxicity of low single and mixture doses of three substances with an anti-androgenic mode of action: vinclozolin, flutamide and prochloraz. Dose levels were selected to mimic the LOAEL as well as the NOAEL for anti-androgenic effects, and the acceptable daily intake (ADI) for each compound, which were then combined together into three mixtures of the LOAELs, NOAELs, and ADIs. Due to the complexity of the study design and the number of treatment groups in this investigation, this project has been sub-divided into a number of publications for analysis; the focus of the present publication will be the mixtures data. Therefore, as the single-substance exposures have been previously discussed in detail, they will only be discussed here insofar as they assist in the interpretation of the mixtures data (Fussell et al. 2015; Melching-Kollmuss et al. 2017; Flick et al. 2016).

### Maternal and developmental toxicity

While no maternal toxicity was observed at any dose level, a number of endocrine-mediated adverse effects were observed at delivery and during development of the F1 generation after LOAEL-MIX treatment. Gestation was lengthened by almost a day, and increased numbers of stillborn pups (and litters with stillborn pups) were noted after parturition, resulting in a reduced live-birth index. A small, but statistically significantly lengthened gestation was observed as a result of NOAEL-MIX treatment, but this value lies within the historical control range of the test facility. Furthermore, no other effect on reproduction or delivery was observed at this NOAEL-MIX, nor was any similar effect on gestation observed after single-substance exposure. Thus, this effect is assessed as incidental and not a result of treatment. Absolutely no changes on reproduction and delivery were observed in the ADI-MIX dose group. Mortality was increased in the pups treated with the NOAEL-MIX, but all dead pups and 4 of the 6 cannibalized pups were from the same litter. This is a relatively common incidental finding in this rat strain and may occur for a number of reasons unrelated to treatment including difficult parturition and poor maternal post-partum care. No dose dependency was observed; moreover, the lower viability index in the NOAEL-MIX group was within the historical control range of the test facility. When this litter was excluded from analysis, pup mortality returned to control levels; further evidence that this is probably a spurious finding. These effects are all consistent with the previously observed single-substance effects of prochloraz at this dose level, but not of vinclozolin or flutamide, and therefore, are considered associated with aromatase inhibiting effects by prochloraz (Andersen et al. 2002).

No other effects on reproductive performance or mortality were observed as a result of exposure to either the NOAEL or ADI mixtures

### Effects in female offspring

In addition, a small, statistically significant increase in the anogenital distance of female pups and a similar, but statistically insignificant increase in the anogenital index of female pups were observed in this dose group on PND 1. These findings are in line with findings in the offspring, exposed to prochloraz-only at its LOAEL dose, and therefore, are assessed to have arisen from this component of the mixture (Melching-Kollmuss et al. 2017; Laier et al. 2006). Regardless, these were the only clinical effects observed in the female offspring; no changes in either onset of puberty or the female sex organ weights were noted. All other findings occurred in the males only.

### Effects in male offspring

#### Anogenital distance/index (AGD/AGI)

Clinical findings, typical for anti-androgenicity, were observed in the developing F1 male offspring after treatment with the LOAEL mixture. A small, but statistically significant decrease in the anogenital index as well as a statistically insignificant reduction in the anogenital distance of male pups, were both observed in the LOAEL-MIX treatment group on the day after birth. Reduced anogenital distances and indices were also observed after vinclozolin and flutamide single-substance exposures (Fussell et al. 2015; Flick et al. 2016); however, these results were less pronounced and not statistically significant, when tested in single compound studies. According to the statistical evaluations using a Loewe additivity model, the degree of the effect on AGI in the LOAEL mixture was higher than what would have been expected; however, it has to be mentioned that the combination effect on the body weight development of the three compounds in the LOAEL mixture has confounded this observation. No respective effects on the male AGD, which does not take into account the body weights of the pups, in the LOAEL mixture has been seen accordingly.

#### Retained nipples/areola

A full 100% of male pups treated with the LOAEL-MIX had retained at least one nipple, areola or hairless spot along the milk line at PND 12. The numbers of nipples, areolae or hairless spots were also substantially increased in this dose group. While the most of the areolae had substantially receded by PND 20, they did not disappear completely in all animals and persisted until puberty, suggesting that while much of the nipple retention is transient, some of the remaining areolae were permanent. This permanent retention is unique feature of the LOAEL mixture group as single-substance administration of the LOAEL dose levels of vinclozolin, flutamide, and prochloraz were unable to produce more than a transient delay in nipple regression. Only the 2.5 mg/kg bw/d flutamide positive control, chosen because it represents a clear anti-androgen effect level, was able to achieve similar permanence (Fussell et al. 2015). When taken together with the anogenital distances/indices recorded just after birth, these data suggest that the clinical effects of LOAEL-MIX exposure were consistent with those previously observed during the single-substance exposures to the individual LOAEL dose levels of vinclozolin, flutamide, and prochloraz, but no assessment can be made on whether this represents a dose-additive or a super-additive response. Importantly, no new, qualitatively different findings were observed in any dose group of the combined exposure study.

#### Preputial separation (entry into male puberty)

The mean age at male sexual maturation was nearly 10 days beyond the historical control range, much longer than the delay arising from the LOAEL single-substance exposures (flutamide: 1 day, vinclozolin: 1 day, prochloraz: 1 day) and close to the positive control group flutamide (nearly 2 weeks). In addition, the comparison of the expected with the observed effects on preputial separation using the Loewe additivity model revealed a slightly more than additive effect. Moreover, this delay in the age at preputial separation corresponded to an increase in body weight commensurate with the more advanced age of the animals. Thus, while the delays in preputial separation can be indicative of both specific (endocrine disruption) and non-specific (impaired general growth) forms of developmental toxicity (Melching-Kollmuß et al. 2014), the principle cause of the delay in sexual development of the LOAEL mixture was not slower body weight development but instead a specific mode of action, one which is consistent with the expected effects of an anti-androgen. The grade of the observed statistically significantly increased delay in preputial separation in the offspring exposed to the LOAEL-MIX compared to the expected effects gives some indication for a more than additive response in the mixtures. No effects were seen for entry into male puberty in the NOAEL mixture group. No effects on female puberty were observed.

#### Hormone measurements

Scattered and mild, but statistically significant, serum hormone levels were noted in male and female animals in the LOAEL mixture group. Increased androstenedione concentrations at killing were noted in both male and female PND 83 ± 2 (Subset 3) offspring as well as the parental female animals. Decreased estradiol levels were observed in the serum of the dams but not their young–adult offspring. Similar hormone changes were not observed in the developing offspring on PND 21 (subset 1) or at puberty (subset 2). These findings were analogous to, although less potent than, those of the single-substance exposure to the LOAEL of prochloraz, resulting in an increase in serum testosterone and reduction in estradiol levels in both the parental females and the PND 83 ± 2 female offspring (subset 3). As prochloraz is known to act via different modes of action which include overall obstruction of steroidogenesis (possibly via Cyp17), inhibition of androgen receptor and specific inhibition of aromatase activity; the increased androgen and decreased estrogen levels in females would be consistent with a prochloraz-specific activity of the mixture due to aromatase inhibition (Melching-Kollmuss et al. 2017).

The majority of the findings are generally compatible with antagonism of the androgen receptor and/or disruption of androgen signaling and subsequent activation of the hypothalamic–pituitary–gonad (HPG) negative-feedback loop (Stocco and McPhaul 2006). This explanation is also consistent with the decreased testosterone levels in fetal testis as a result of in utero LOAEL-MIX exposure, which has also been described by Blystone et al. (2007). Although this HPG feedback loop is only established post-natally, antagonism of the androgen receptors at this developmental stage would be expected to result in decreased, rather than increased testosterone production by Leydig cells.

Although no hormone effects were observed after single-substance treatment with the receptor antagonists at LOAEL dose levels, a similar increase in androgen concentrations was observed in male and female rat serum after administration of a higher flutamide dose (2.5 mg/kg bw/d, used as a positive control). These serum hormone findings have also been described in the literature at a variety of vinclozolin and flutamide dose levels above the LOAEL (Chandolia et al. 1991).

#### Reproduction organs

No treatment-related findings were observed in the organ weights of the female parental animals of any test group; however, both absolute and relative weight changes were observed in the sex organs of the male offspring exposed to the LOAEL-MIX. Decreased reproductive organ weights were observed across a number of organs and are interpreted as an indication of altered androgen signaling (see supplement). More specifically, it is noted that a dose level capable of inducing an anti-androgenic effect in one organ, generally also induces similar effects in the other male sex organs. Furthermore, as exposure continued in the offspring, the number of affected organs increased; by adulthood these included most of the accessory sex glands. Although the most logical explanation for these findings in adults would be a reduced secretion by the glands, histopathological evaluations revealed no apparent morphological correlate and no reduced secretion in the accessory sex glands.

In this study, some differences in anti-androgen sensitivity between the organs were observed. For instance, on PND 21 (subset 1) and at puberty (subset 2), the responses of the relative ventral and total prostate weights to LOAEL-MIX treatment differed, suggesting that at different time points the ventral prostate weight is more sensitive than the total prostate weight followed by the weight of seminal vesicles. Using the statistical Loewe model, dose additivity was most accurately reflecting the changes seen in ventral prostate weights of the LOAEL-MIX compared to the single compound LOAEL dose groups.

The histopathologically observed differences in the left epididymis of LOAEL-MIX treated subset 2 (at puberty) animals correlated very well with the higher age of these animals and the delayed day of preputial separation (day of necropsy) leading to a more advanced stage of developed testis and epididymis. This may indicate that the local androgen level in the testes is still sufficient for testicular maturation and spermatogenesis.

When all findings in the male offspring (reduced anogenital distance/index, areola retention, delayed puberty, increased serum androgen/decreased serum estrogen levels and reduced reproductive organ weights) are considered in a quantitative way, there is evidence for a combined toxicity effect in the LOAEL mixture group, relative to the individual compounds. The overall quantitative nature of the interaction, as analyzed using the Loewe model can be best described as additive (reproductive organ weights, anogenital distance) or slightly more than additive (preputial separation and less convincing anogenital index) (see Supplementary Figures 28–30). Additivity of parameters sensitive to anti-androgenicity would be in concordance with previously described expectations of the additive effects of a mixture of endocrine modulators, even in case of different modes of actions (Birkhoj et al. 2004; Kjaerstad et al. 2010; Rider et al. 2008a, b, 2009; Blystone et al. 2009).

The three AR antagonists (vinclozolin, flutamide and procymidone) used by Hass et al. (2007) and Metzdorff et al. (2007) also showed dose addition for anogenital distance and nipple retention. However, a combination of two phthalates, i.e. DEHP and DEHA, did not show a mixture effect (Jarfelt et al. 2005). When administered from GD 14–18 a binary phthalate and 5-component phthalate mixture showed dose addition for several anti-androgenic parameters (Howdeshell et al. 2007, 2008a, b). Essentially the same picture was drawn after administration of mixtures of phthalates (BBP, DBP, DEHP) and pesticides (vinclozolin, procymidone, linuron, and prochloraz). According conclusions drawn from these studies on mixtures of anti-androgenic compounds with differing mechanisms of action, the concept of independent action would lead to underestimation of effects. An overall reasonable agreement is seen with concept of dose addition (Rider et al. 2008a, b). An anti-androgenic mixture with different mechanism of action (DEHP, vinclozolin, prochloraz, finasteride) in a perinatal study design (dosage from GD 7-PND 16) showed for the majority of endpoints the same result, however, for a single endpoint a synergistic mode of action was postulated (Christiansen et al. 2009).

Mixture effects were not observed at the NOAEL-MIX. This means that dose levels were too low to cause effects as single-substances, and in the present study, were also not able to jointly cause a substantial effect as a mixture. In particular, no effects were noted after examination of the same developmental endpoints used to clinically assess the anti-androgenic activity of the LOAEL-MIX. No decrease in anogenital distance or index was noted in male pups exposed in utero to either the ADI-MIX or NOAEL-MIX when compared to those of concurrent controls. Nipple retention was not increased by exposure to either mixture, not even transiently. Nor were any delays in male (or female) sexual maturation noted as a result of treatment with these mixtures. Similar results were obtained for non-endocrine effects with no adverse effects seen in NOAEL mixtures of four compounds (Schmidt et al. 2016).

#### Relevance of historical control data and adversity of effects

Typically, these standardized experiments are designed to model the effects of a test substance in humans, necessitating the choice of an outbred animal model to more accurately imitate the amount of genetic variation in a human population. This is not without experimental trade-offs; increased genetic variation in the animal model results in a larger range of biological responses to the test substance (higher biological variation) and even recessive genetic phenotypes that can manifest as clinical signs similar to substance toxicity but having nothing to do with substance exposure.

The historical control ranges should accurately reflect the amount of phenotypic variation around the time of the study and describe the inter-study variation for each endpoint, which is often larger than the intra-study variation described by the concurrent controls. When used in concert with the concurrent controls from the study, historical control data can provide some justification for why a statistically significant observation may or may not be biologically relevant.

Thus, a lack of historical control data to reference can seriously hinder the interpretation of inconclusive data. This is often the case when novel endpoints, non-standardized methodologies and alternative time points are employed in a study design. In the course of the current investigation, we found this to be true for a few observations: a statistically significantly reduced serum estradiol concentration in the parental females and decreased absolute cauda epididymis and relative bulbourethral gland weights in PND 83 ± 2 (Subset 3) males exposed to the NOAEL-MIX. In all cases, these observations were borderline effects with uncertain interpretations.

Chiefly, the problem with the interpretation of such data is the inconsistencies inherent in such observations, particularly if outbred animal strains are being used, as it is general custom for toxicological investigations. For instance, a reduced serum estradiol concentration in dams is consistent with aromatase inhibition by prochloraz; however, no decrease in serum estradiol level, not even a statistically insignificant one, was observed after treatment with the single-substance. While this effect was observed in NOAEL-MIX dams, it was not observed in the adult female offspring of the NOAEL-MIX group; this pattern is totally dissimilar to the pattern of estrogen and androgen hormonal changes that were observed at the LOAEL level in both parental and filial adult females.

These inconsistencies are also relevant to the interpretation of the decreased absolute cauda epididymis and relative bulbourethral gland weights in PND 83 ± 2 (subset 3) males. Decreased male reproductive organ weights make sense in the context of anti-androgenic treatment; however, usually any effects in adults would be noted in both absolute and relative organ weights. Moreover, this anti-androgenic response generally is noted in a variety of sex organs and secondary glands, rather than just two. Due to the comparative scarcity of other findings in this treatment group, we assess that these borderline findings have no biological relevance.

A more likely explanation lies in the random nature of observed events. If we compare these results to the control groups from the other studies in a sort of faux historical control comparison, we find that these hormone and body weight findings lie well within the “historical control range” described by just these two control groups. Thus, these findings are probably not considered relevant to NOAEL mixture treatment. Importantly, these observations also highlight the magnitude of the inherent variation between studies, even under controlled laboratory situations. This level of variation seems to be quite high at some measured endpoints, especially the serum hormone concentrations, suggesting that some biological processes have a wider tolerance for ‘normal’ responses while others are tightly controlled. This variation is not necessarily reflected in the standard deviations observed within a single study, as all the animals enrolled in the study are descendants of the same outbred colony in the same animal room at the same breeder. Thus, each parameter needs to be evaluated individually, both in the context of the concurrent control and the historical dataset.

One logical reason for this variation is the diversity of developmental triggers. Some parameters are controlled by time (e.g., somite development, neural tube closure), some by general developmental factors (e.g., body weight), some by hormonal influences (e.g., male external genital development, nipple regression), but most are controlled by a combination of these factors (e.g., onset of puberty, sexual behavior). The more apical and terminal the phenotypic event, the more developmental factors are likely to be involved. Thus, each individual represents a unique, but perfectly normal, solution to the problem of gene–environment interaction, but one solution which reflects both the gene pool from which it originates and the environment.

It is reasonable then to assume that this spectrum of biological variation will result in a spectrum of responses to a test chemical for each endpoint. Furthermore, this variation of biological response will be higher at some endpoints than others. This is partly due to the number and type of factors that might be involved at that stage of development, but also because of the performance involved in making the measurement or observation. This kind of variation may be more or less relevant at certain endpoints, but becomes extremely pertinent for others. For instance, anogenital distance measurements vary substantially depending on the exact technique used. Therefore, it is important to standardize the observations as much as possible to minimize the effect of the technical variation on the outcome of the study and understand the limitations of the technique chosen, to inform the ultimate data assessment and prevent over-interpretation of the results. The combination of method standardization and the use of historical control ranges ultimately define the limits of what is ‘normal’, be it derived from the technical limitations of the study design or biological limitations of the animal model. This range must be taken into account when assessing the toxicological relevance of data.

The question of adversity also needs to be addressed. It is often unclear to what extent the observed findings adversely affect animal health (Lewis et al. 2002). As Foster and McIntyre (2002) reported, rare, permanent structural defects which compromise the quality of life (e.g., reproductive tract malformations) should be considered adverse findings. But they go on to note that the relationship between statistically significant changes in endpoints considered to be indicators of impaired androgen status and such malformations is uncertain. Moreover, these indicators of impaired endocrine status are functional, rather than toxicologically induced morphologic changes. Put another way, every individual organism interacts differently with its environment to solve the endocrine homeostasis problem; even when the environmental stimuli are exactly the same, one might produce more hormonal signal, while a second might upregulate the receptor to reassert homeostasis. Such regulations generally are considered normal, adaptive, and necessary as long as they are transient and within the normal homeostatic range (Goodman et al. 2010; Rhomberg and Goodman 2012). It seems logical that the same adaptive processes, which allow humans to reassert hormonal homeostasis in a changing natural environment, might also compensate for exposure to endocrine active substances at low dose levels individually or as mixtures. As long as these processes remain truly adaptive, then they do not necessarily pose an increased hazard to humans. Therefore, it is important to determine not only whether effects are observed at human-relevant exposures, but also whether any effects observed are adverse.

## Conclusion

The present experiments were designed to test for combined effects at NOAEL and human relevant low doses of substances at the same site of action, as well as the possibility of synergism between different modes of action at effect levels. Actual side-by-side testing of single compounds and the mixed combinations were performed in experiments with an appropriate experimental design including a suitable number of test subjects, appropriate dosing, apposite time-windows and duration of exposure, and all relevant endpoints (OECD 2002).

Three compounds having anti-androgenic properties were chosen to represent two different modes of action, vinclozolin and flutamide, are both androgen receptor antagonists, thereby disrupting androgen signaling while prochloraz primarily disrupts steroid hormone biosynthesis, but also inhibits the aromatase and is an androgen receptor antagonist. Dose levels were selected to mimic a LOAEL as well as a NOAEL for anti-androgenic effects, and the acceptable daily intake (ADI) for each individual compound, which were then combined together into three mixtures of the LOAELs, NOAELs, and ADIs. Mixture effects would be established through direct comparison of the single-substance and mixture exposure groups.

In general, anti-androgenic changes were observed at the LOAEL-MIX (20, 0.25, and 30 mg/kg bw/d vinclozolin, flutamide, and prochloraz, respectively), but not at lower exposures (NOAEL-MIX 4, 0.025, and 5 mg/kg bw/d; ADI-MIX 0.005, 0.00025 and 0.01 mg/kg bw/d vinclozolin, flutamide, and prochloraz, respectively). Neither the small changes in serum androgen hormone concentrations nor the borderline reduction in single male sex organ weights observed with NOAEL-MIX treatment were noteworthy enough to be considered adverse. Thus, the NOAEL-MIX is truly a NOAEL. Furthermore, since there were absolutely no findings in the ADI-MIX, this dose level is definitely below the NOEL.

Nipple/areola counts appeared to be the most sensitive measure of effect, closely followed by age at sexual maturation, then anogenital distance/anogenital index and male sex organ weights, esp. ventral prostate weight, and finally gross and histopathological findings. This order generally coincides with the order of sensitivities seen in the literature (Borgert et al. 2014). The quantification of circulating hormone levels showed little consistency when comparing possibly treatment-related changes with those from same dose group at other developmental stages or when mixed- and single-substance exposure data were compared. However, adult serum hormone levels were mildly affected by LOAEL mixture treatment while similar hormone changes were not observed in the developing offspring on PND 21 (subset 1) or at puberty (subset 2). In contrast, testosterone changes in testes of male fetuses at GD 20 seems to represent an appropriate biomarker for potential anti-androgenic effects in a sensitive tissue during a critical developmental window. Combined exposure at LOAEL level resulted in more than additive responses for decreased male anogenital index (but not for anogenital distance), and delayed preputial separation in comparison to the expected effects of the individual compounds.

While these endpoints for anti-androgenicity had varying sensitivities, when taken together these data reveal two important observations: The dose–response curve clearly indicates a monotonic process and no evidence for an interaction of the compounds at the individual NOAEL or lower doses. In summary, endocrine toxicity is sometimes said to represent a special case with regard to dose–response at low dose levels, but in our experiments, we found no evidence for non-monotonicity.

## Electronic supplementary material

Below is the link to the electronic supplementary material.

Supplementary material 1 (DOC 6422 kb)

Supplementary material 2 (DOCX 1000 kb)

Supplementary material 3 (DOCX 14 kb)

Supplementary material 4 (DOCX 13 kb)

Supplementary material 5 (PDF 14858 kb)

Supplementary material 6 (PDF 4710 kb)

Supplementary material 7 (PDF 9532 kb)

## References

[CR1] Andersen HR, Vinggaard AM, Rasmussen TH, Gjermandsen IM, Bonefeld-Jorgensen EC (2002). Effects of currently used pesticides in assays for estrogenicity, androgenicity, and aromatase activity in vitro. Toxicol Appl Pharmacol.

[CR2] Birkhoj M, Nellemann C, Jarfelt K, Jacobsen H, Andersen HR, Dalgaard M, Vinggaard AM (2004). The combined anti-androgenic effects of five commonly used pesticides. Toxicol Appl Pharmacol.

[CR3] Blystone CR, Lambright CS, Howdeshell KL, Furr J, Sternberg RM, Butterworth BC, Durhan EJ, Makynen EA, Ankley GT, Wilson VS, LeBlanc GA, Grau LE (2007). Sensitivity of fetal rat testicular steroidogenesis to maternal prochloraz exposure and the underlying mechanism of inhibition. Toxicol Sci.

[CR4] Blystone CR, Lambright CS, Cardon MC, Furr J, Rider CV, Hartig PC, Wilson VS, Gray LE (2009). Cumulative and antagonistic effects of a mixture of the antiandrogens vinclozolin and iprodione in the pubertal male rat. Toxicol Sci.

[CR5] Boobis AR, Ossendorp BC, Banasiak U, Hamey PY, Sebestyen I, Moretto A (2008). Cumulative risk assessment of pesticide residues in food. Toxicol Lett.

[CR6] Borch J, Axelstad M, Vinggaard AM, Dalgaard M (2006). Diisobutyl phthalate has comparable anti-androgenic effects to di-*n*-butyl phthalate in fetal rat testis. Toxicol Lett.

[CR7] Borgert CJ, Stuchal LD, Mihaich EM, Becker RA, Bentley KS, Brausch JM, Coady K, Geter DR, Gordon E, Guiney PD, Hess F, Holmes CM, LeBaron MJ, Levine S, Marty S, Mukhi S, Neal BH, Ortego LS, Saltmiras DA, Snajdr S, Staveley J, Tobia A (2014). Relevance weighting of tier 1 endocrine screening endpoints by rank order. Birth Defects Res.

[CR8] Chandolia RK, Weinbauer GF, Behre HM, Nieschlag E (1991). Evaluation of a peripherally selective antiandrogen (Casodex) as a tool for studying the relationship between testosterone and spermatogenesis in the rat. J Steroid Biochem Mol Biol.

[CR9] Christiansen S, Scholze M, Axelstad M, Boberg J, Kortenkamp A, Hass U (2008). Combined exposure to anti-androgens causes markedly increased frequencies of hypospadias in the rat. Int J Androl.

[CR10] Christiansen S, Scholze M, Dalgaard M, Vinggaard AM, Axelstad M, Kortenkamp A, Hass U (2009). Synergistic disruption of external male sex organ development by a mixture of four antiandrogens. Environ Health Perspect.

[CR11] Creasy D, Bube A, De Rijk E, Kandori H, Kuwahara M, Masson M, Nolte T, Reams R, Regan K, Rehm S, Rogerson P, Whitney K (2012). Proliferative and nonproliferative lesions of the rat and mouse male reproductive system. Toxicol Path.

[CR12] ECETOC technical report 115 (2012) Effects of chemical co-exposures at doses relevant for human safety assessments

[CR13] EFSA (2011). Conclusion on the peer review of the pesticide risk assessment of the active substance prochloraz. EFSA J.

[CR14] EFSA (2013). Scientific Opinion on the identification of pesticides to be included in cumulative assessment groups on the basis of their toxicological profile. EFSA J.

[CR15] EFSA (2013). Scientific Opinion on the relevance of dissimilar mode of action and its appropriate application for cumulative risk assessment of pesticides residues in food. EFSA J.

[CR16] External Scientific Report (2016) Toxicological data collection and analysis to support grouping of pesticide active substances for cumulative risk assessment of effects on the nervous system, liver, adrenal, eye, reproduction and development and thyroid system. Published: 17 Feb 2016

[CR17] Fegert I, Billington R, Botham P, Carney E, FitzGerald RE, Hanley T, Lewis R, Marty MS, Schneider S, Sheets LP, Stahl B, van Ravenzwaay B (2012). Feasibility of the extended one-generation reproductive toxicity study (OECD 443). Reprod Toxicol.

[CR18] Feron VJ, Groten JP (2002). Toxicological evaluation of chemical mixtures. Food Chem Toxicol.

[CR19] Feuston MH, Bodnar KR, Kerstetter SL, Grink CP, Belcak MJ, Singer J (1989). Reproductive toxicity of 2-methoxyethanol applied dermally to occluded and nonoccluded sites in male rats. Toxico. Appl Pharmacol.

[CR20] Flick B, Schneider S, Melching-Kollmuss S, Fussell KC, Groeters S, Buesen R, Strauss V, van Ravenzwaay B (2016). Investigations of putative reproductive toxicity of low-dose exposures to vinclozolin in Wistar rats. Arch Toxicol.

[CR21] Foster PMD, McIntyre BS (2002). Endocrine active agents: implications of adverse and non-adverse changes. Toxicol Pathol.

[CR22] Fridmans A, Chertin B, Koulikov D, Lindenberg T, Gelber H, Leiter C, Farkas A, Spitz IM (2005). Reversibility of androgen deprivation therapy in patients with prostate cancer. J Urol.

[CR23] Fussell KC, Schneider S, Buesen R, Groeters S, Strauss V, Melching-Kollmuss S, van Ravenzwaay B (2015). Investigations of putative reproductive toxicity of low-dose exposures to flutamide in Wistar rats. Arch Toxicol.

[CR24] Goodman JE, Kerper LE, Boyce CP, Prueitt RL, Rhomberg LR (2010). Weight-of-evidence analysis of human exposures to dioxins and dioxin-like compounds and associations with thyroid hormone levels during early development. Regul Toxicol Pharmacol.

[CR25] Gray LE, Wilson VS, Stocker T, Lambright C, Furr J, Noriega N, Howdeshell K, Ankley GT, Guillette L (2006). Adverse effects of environmental antiandrogens and androgens on reproductive development in mammals. Int J Androl.

[CR26] Gray LE, Furr J, Howdeshell K, Hotchkiss A, Wilson V, Rider C (2007). Cumulative effects of in utero administration of mixtures of “antiandrogens” on male rat reproductive development. Toxicology.

[CR27] Hass U, Scholze M, Christiansen S, Dalgaard M, Vinggaard AM, Axelstad M, Metzdorff SB, Kortenkamp A (2007). Combined exposure to anti-androgens exacerbates disruption of sexual differentiation in the rat. Environ Health Perspect.

[CR28] Hellwig J, van Ravenzwaay B, Mayer M, Gembardt C (2000). Pre- and post-natal oral toxicity of vinclozolin in Wistar and Long-Evans rats. Regul Toxicol Pharmacol.

[CR29] Howdeshell KL, Furr J, Lambright CR, Rider CV, Wilson VS, Gray LE (2007). Cumulative effects of dibutyl phthalate and diethylhexyl phthalate on male rat reproductive tract development: altered fetal steroid hormones and genes. Toxicol Sci.

[CR30] Howdeshell KL, Rider CV, Wilson VS, Gray LE (2008). Mechanisms of action of phthalate esters, individually and in combination, to induce abnormal reproductive development in male laboratory rats. Environ Res.

[CR31] Howdeshell KL, Wilson VS, Furr J, Lambright CR, Rider CV, Blystone CR, Hotchkiss AK, Gray LE (2008). A mixture of five phthalate esters inhibits fetal testicular testosterone production in the Sprague-Dawley rat in a cumulative, dose-additive manner. Toxicol Sci.

[CR32] Jacobsen PR, Axelstad M, Boberg J, Isling LK, Christiansen S, Mandrup KR, Berthelsen LO, Vinggaard AM, Hass U (2012). Persistent developmental toxicity in rat offspring after low dose exposure to a mixture of endocrine disrupting pesticides. Reprod Toxicol.

[CR33] Jarfelt K, Dalgaard M, Hass U, Boberg J, Jacobsen H, Ladefoged O (2005). Antiandrogenic effects in male rats perinatally exposed to a mixture of di(2-ethylhexyl) phthalate and di(2-ethylhexyl) adipate. Reprod Toxicol.

[CR34] Kittel B, Ruehl-Fehlert C, Morawietz G, Klapwijk J, Elwell MR, Lenz B, O’Sullivan MG, Roth DR, Wadsworth PF (2004). Revised guides for organ sampling and trimming in rats and mice—part 2. Exp Toxicol Pathol.

[CR35] Kjaerstad MB, Taxvig C, Andersen HR, Nellemann C (2010). Mixture effects of endocrine disrupting compounds in vitro. Int J Androl.

[CR36] Kortenkamp A, Backhaus T, Faust M (2009) State of the art report on mixture toxicity final report. 070307/2007/485103/ETU/D.1

[CR37] Kratochwil K (1971). In vitro analysis of the hormonal basis for the sexual dimorphism in the embryonic development of the mouse mammary gland. J Embry Exp Morph.

[CR38] Kratochwil K, Schwartz P (1976). Tissue interaction in androgen response of embryonic mammary rudiment of mouse: identification of target tissue for testosterone. Proc Natl Acad Sci USA.

[CR39] Laier P, Metzdorff SB, Borch J, Hagen ML, Hass U, Christiansen S, Axelstad M, Kledal T, Dalgaard M, McKinnell C, Brokken LJS, Vinggaard AM (2006). Mechanisms of action underlying the antiandrogenic effects of the fungicide prochloraz. Toxicol Appl Pharmacol.

[CR40] Lewis RW, Billington R, Debryune E, Gamer A, Lang B, Carpanini F (2002). Recongnition of adverse and nonadverse effects in toxicity studies. Toxicol Pathol.

[CR41] Loewe S (1953). The problem of synergism and antagonism of combined drugs. Arzneimittelforschung.

[CR42] Melching-Kollmuß S, Fussell K, Buesen R, Dammann M, Schneider S, Tennekes H, van Ravenzwaay B (2014). Anti-androgenicity can only be evaluated using a weight of evidence approach. Regul Toxicol Pharmacol.

[CR43] Melching-Kollmuss S, Fussell KC, Schneider S, Buesen R, Groeters S, Strauss V, van Ravenzwaay B (2017). Comparing effect levels of regulatory studies with endpoints derived in targeted anti-androgenic studies—example prochloraz. Arch Toxicol.

[CR44] Metzdorff SB, Dalgaard M, Christiansen S, Axelstad M, Hass U, Kiersgaard M, Scholze M, Kortenkamp A, Vinggaard AM (2007). Dysgenesis and histological changes of genitals and perturbations of gene expression in male rats after in utero exposure to antiandrogen mixtures. Toxicol Sci.

[CR45] Morawietz G, Ruehl-Fehlert C, Kittel B, Bube A, Keane K, Halm S, Heuser A, Hellmann J (2004). Revised guides for organ sampling and trimming in rats and mice—part 3. Exp Toxicol Pathol.

[CR46] OECD (2001a) OECD guideline for the testing of chemicals 414: prenatal developmental toxicity study

[CR47] OECD (2001b) OECD guideline for the testing of chemicals 416: two-generation reproduction toxicity study

[CR48] OECD (2002) Series on testing and assessment no. 21, appraisal of test methods for sex hormone disrupting chemicals, ENV/JM/MONO(2002)

[CR49] OECD (2011) OECD guideline for the testing of chemicals 443: extended one-generation reproductive toxicity study

[CR50] REGULATION (EC) No 1107/2009 OF THE EUROPEAN PARLIAMENT AND OF THE COUNCIL of 21 October 2009 concerning the placing of plant protection products on the market and repealing Council Directives 79/117/EEC and 91/414/EEC

[CR51] REGULATION (EC) NO 396/2005 OF THE EUROPEAN PARLIAMENT AND OF THE COUNCIL of 23 February 2005 on maximum residue levels of pesticides in or on food and feed of plant and animal origin and amending Council Directive 91/414/EEC

[CR52] Rhomberg LR, Goodman JE (2012). Low-dose effects and nonmonotonic dose–responses of endocrine disrupting chemicals: has the case been made?. Regul Toxicol Pharmacol.

[CR53] Rider CV, Furr J, Wilson VS, Gray LE (2008). A mixture of seven antiandrogens induces reproductive malformations in rats. Int J Androl.

[CR54] Rider CV, Furr J, Wilson VS, Gray LE (2008). A mixture of seven antiandrogens induces reproductive malformations in rats. Int J Androl.

[CR55] Rider CV, Wilson VS, Howdeshell KL, Hotchkiss AK, Furr JR, Lambright CR, Gray LE (2009). Cumulative effects of in utero administration of mixtures of “antiandrogens” on male rat reproductive development. Toxicol Pathol.

[CR56] Rider CV, Furr JR, Wilson VS, Gray LE (2010). Cumulative effects of in utero administration of mixtures of reproductive toxicants that disrupt common target tissues via diverse mechanisms of toxicity. Int J Androl.

[CR57] Ruehl-Fehlert C, Kittel B, Morawietz G, Deslex P, Keenan C, Mahrt CR, Nolte T, Robinson M, Stuart BP, Deschl U (2003). Revised guides for organ sampling and trimming in rats and mice—part 1. Exp Toxicol Pathol.

[CR58] Salewski E (1964). Färbemethode zum makroskopischen Nachweis von Implantationsstellen am Uterus der Ratte. Naunyn Schmiedeberg’s Arch Exp Pathol Pharmakol.

[CR59] Schmidt F, Marx-Stoelting P, Haider W, Heise T, Kneuer C, Ladwig M, Banneke S, Rieke S, Niemann L (2016). Combination effects of azole fungicides in male rats in a broad dose range. Toxicology.

[CR60] Schneider S, Kaufmann W, Strauss V, van Ravenzwaay B (2011). Vinclozolin: a feasibility and sensitivity study of the ILSI-HESI extended one generation reproduction protocol. Regul Toxicol Pharmacol.

[CR61] Slott VL, Suarez JD, Perault SD (1991). Rat sperm motility analysis, methodologic considerations. Reprod Toxicol.

[CR62] Stocco DM, McPhaul MJ, Neill JD (2006). Physiology of testicular steroidogenesis. Knobil and Neill’s Physiology of Reproduction.

[CR63] US Environmental Protection Agency (1972) US Federal Insecticide, Fungicide, and Rodenticide Act (FIFRA): Good Laboratory Practice Standards. 40 Code of Federal Regulations Part 160

[CR64] US Environmental Protection Agency (1976) US Toxic Substances Control Act (TSCA): Good Laboratory Practice Standards. 40 Code of Federal Regulations Part 792

[CR65] US Environmental Protection Agency (1998) US EPA health effects test guideline: OPPTS 870.3700 prenatal developmental toxicity study. EPA-HQ-OPPT-2009-0156-0017

[CR66] Wilkinson CF, Christoph GR, Julien E, Kelley JM, Kronenberg J, McCarty J, Reiss R (2000). Assessing the risk of exposures to multiple chemicals with a common mechanism of toxicity: how to cumulate?. Regul Toxicol Pharmacol.

[CR67] Yamada H, Yamahara A, Yasuda S, Abe M, Oguri K, Fukushima S, Ikeda-Wada S (2002). Dansyl chloride derivatization of methamphetamine: a method with advantages for screening and analysis of methamphetamine in urine. J Anal Toxicol.

[CR68] Zhang F, Rick DL, Kan LH, Perala AW, Geter DR, LeBaron MJ, Bartels MJ (2011). Simultaneous quantitation of testosterone and estradiol in human cell line (H295R) by liquid chromatography/positive atmospheric pressure photoionization tandem mass spectrometry. Rapid Commun Mass Spectrom.

