# Supplementary material for: Investigations on the dose–response relationship of combined exposure to low doses of three anti-androgens in Wistar rats
Source: Arch Toxicol. 2017 Sep 6;91(12):3961–89. doi: 10.1007/s00204-017-2053-3 (PMC5719133; doi:10.1007/s00204-017-2053-3)
Supplement: Supplementary file 1 — Supplementary material 1 (DOC 6422 kb) [file 204_2017_2053_MOESM1_ESM.doc]

# Supplementary Data


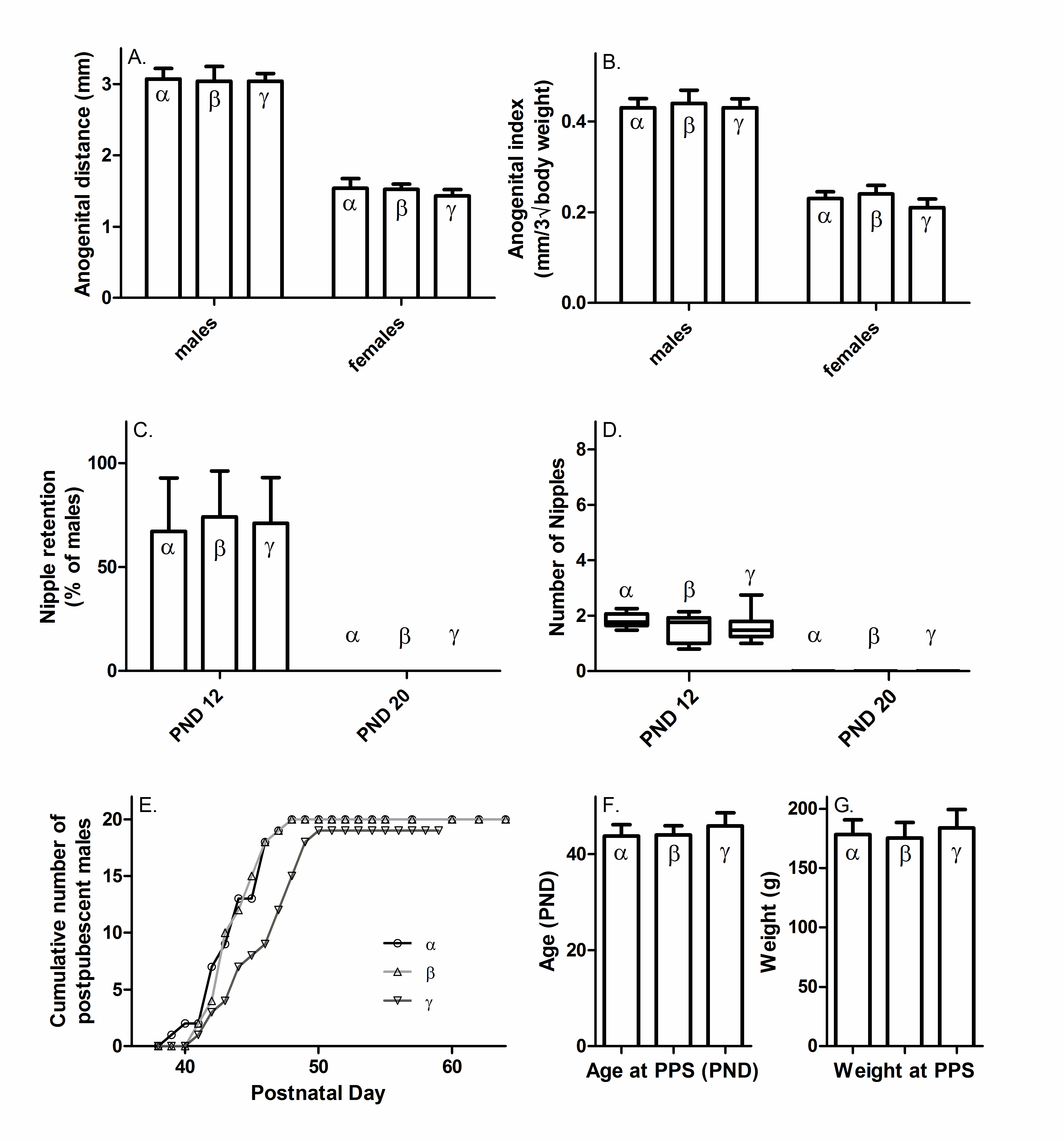


Supplementary Figure 1: Comparison of the controls of three different experiments across a number of developmental endpoints. For technical reasons, these investigations were performed over two years as three separate experiments of the same study design. To confirm that the results of these experiments were analogous, the three concurrent control groups were compared for their equivalency across all developmental parameters measured. All statistical evaluations are based on comparisons between a treatment group and its concurrent control group.


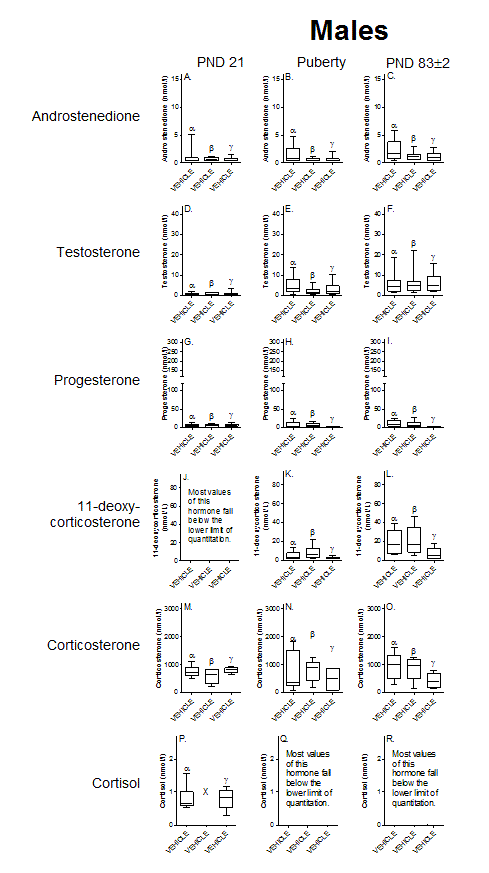


Supplementary Figure 2: Comparison of the selected hormone levels of the male controls of three different experiments. For technical reasons, these investigations were performed over two years as three separate experiments of similar study design. To confirm that the results of these experiments were analogous, the three concurrent control groups were compared for their equivalency across all hormone levels measured, including those shown here. No statistically significant or biologically relevant difference was detected between these control groups; however, all statistical evaluations are based on comparisons between a treatment group and its concurrent control group only.


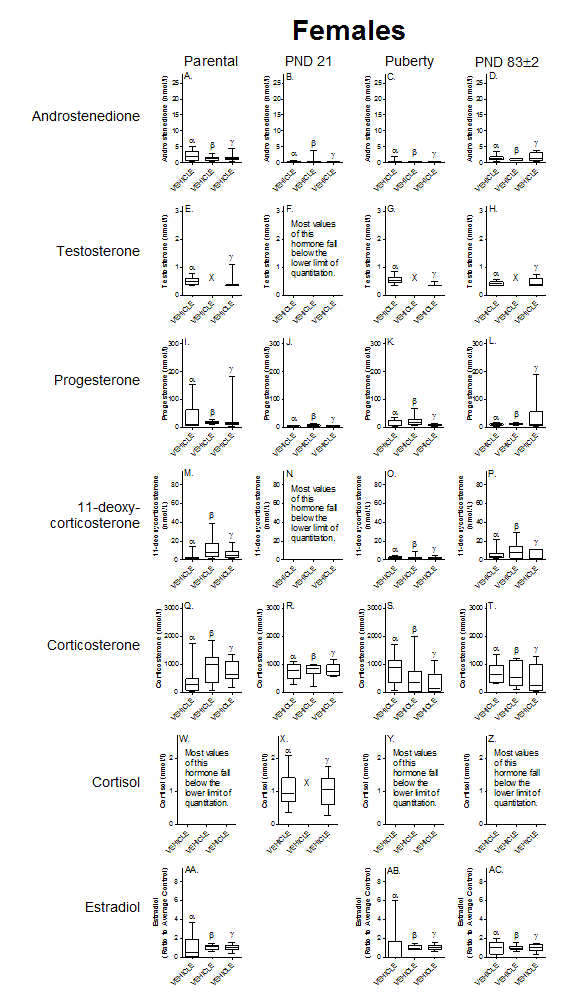


Supplementary Figure 3: Comparison of the selected hormone levels of the male controls of three different experiments. For technical reasons, these investigations were performed over two years as three separate experiments of similar study design. To confirm that the results of these experiments were analogous, the three concurrent control groups were compared for their equivalency across all hormone levels measured, including those shown here. No statistically significant or biologically relevant difference was detected between these control groups; however, all statistical evaluations are based on comparisons between a treatment group and its concurrent control group only.


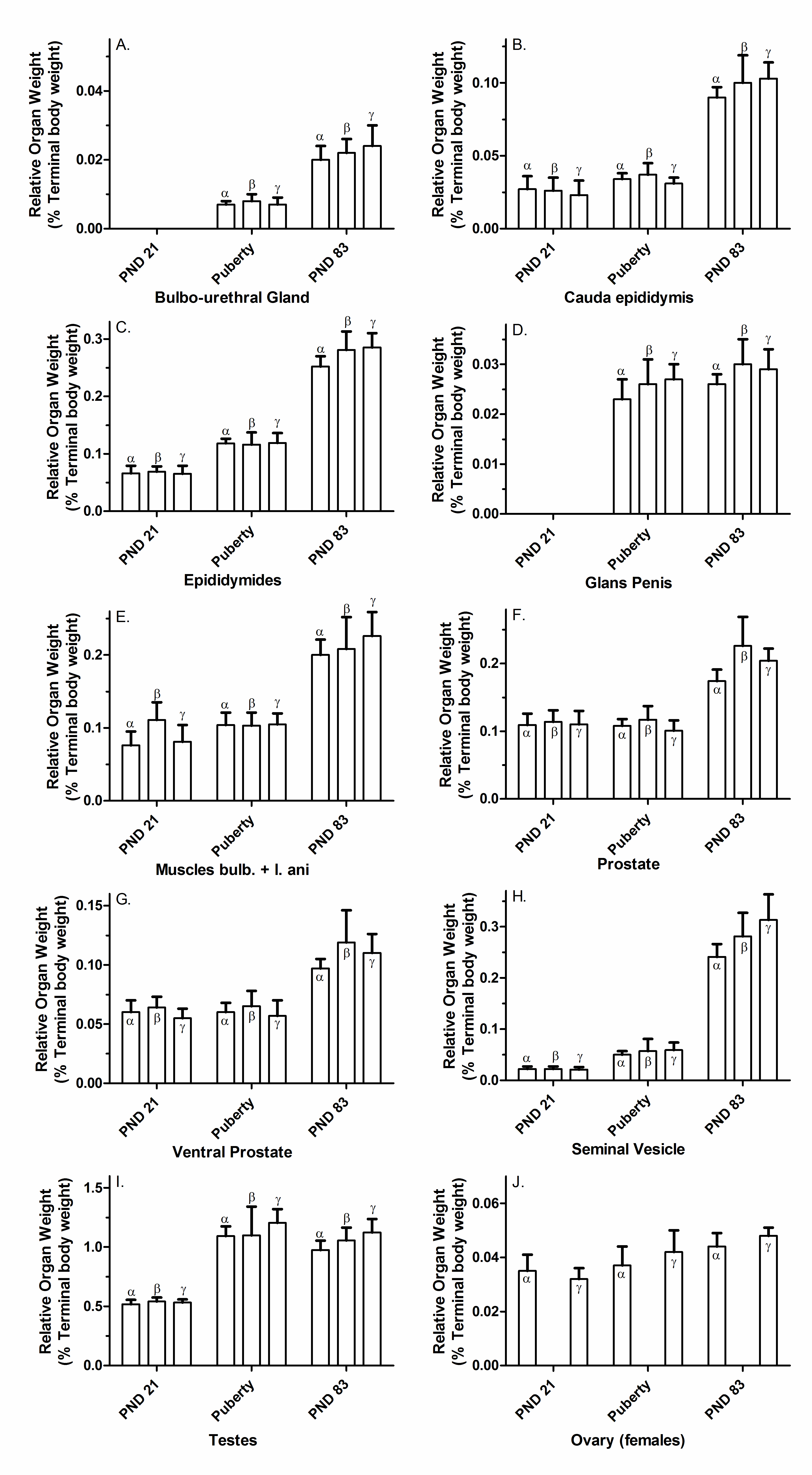


Supplementary Figure 4: Sex organ weight comparison of the controls of three different experiments. For technical reasons, these investigations were performed over two years as three separate experiments of similar study design. To confirm that the results of these experiments were analogous, the three concurrent control groups were compared for their equivalency across all sex organ weights measured. No statistically significant or biologically relevant difference was detected between these control groups; regardless, all statistical evaluations of the treatment data are based on the comparison between a treatment group and its concurrent control.


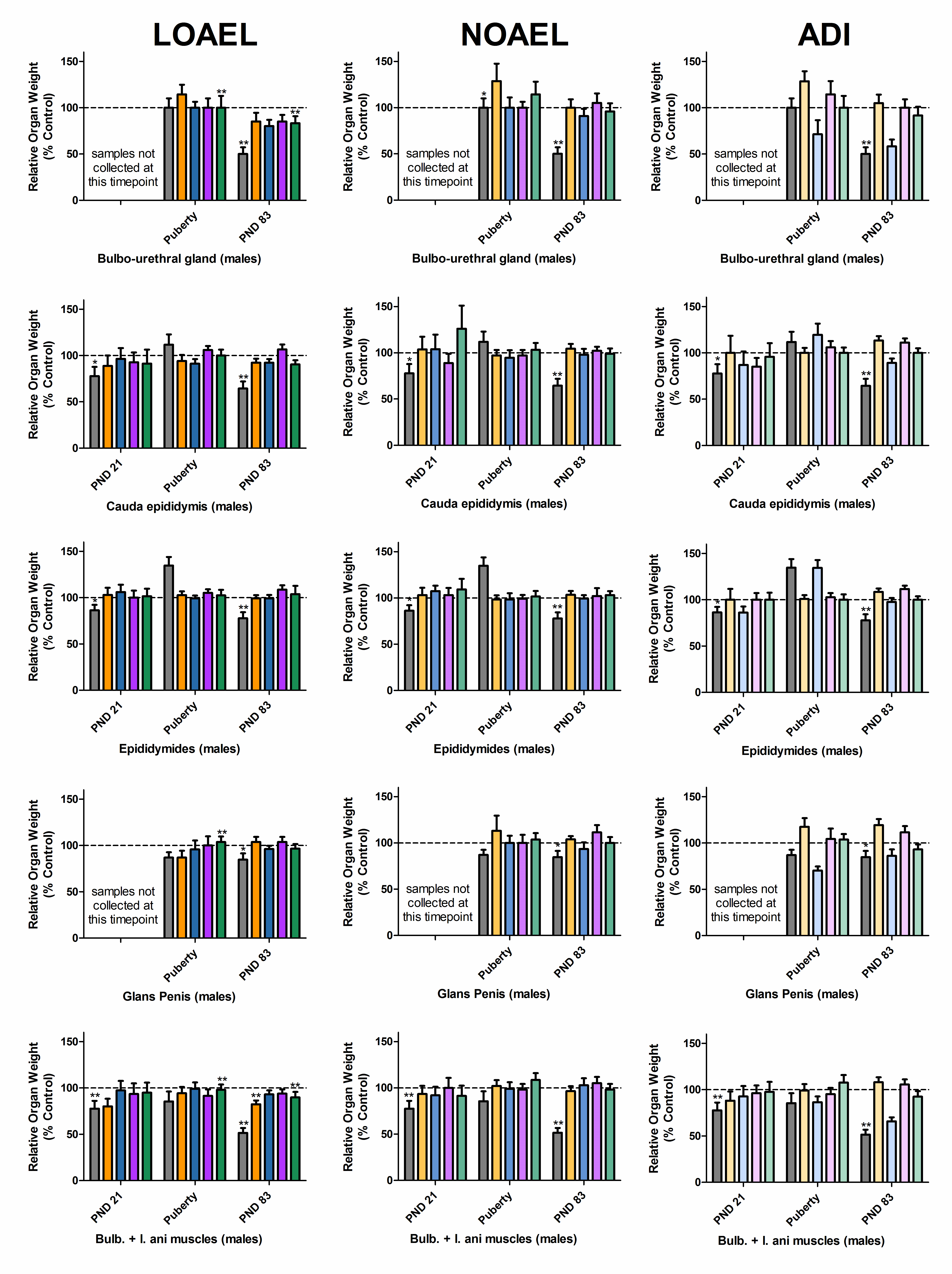


Supplementary Figure 5: Comparison of the weights of half of the sexual organs of male rats exposed to anti-androgens. On PND 21 (Subset 1), the day of preputial separation or vaginal opening (Puberty, Subset 2) and PND 83±2 (Subset 3), the sex organs from each of 10 male and 10 female rats were asservated, weighed and reported as relative organ weights. The relative organ weights of the mixture-treated animals were then compared to the effects of the single-substance exposures to vinclozolin , flutamide and prochloraz at each dose level: LOAEL *(left panels),* NOAEL *(center panels)* and ADI *(right panels)* as well as vehicle and positive controls . (Data are shown as means ± SD and all statistical evaluations are based on comparisons between a treatment group and its concurrent control group.) Generally, male sex organ weights were reduced in animals exposed to the anti-androgens at the LOAEL level, but not the NOAEL or ADI.


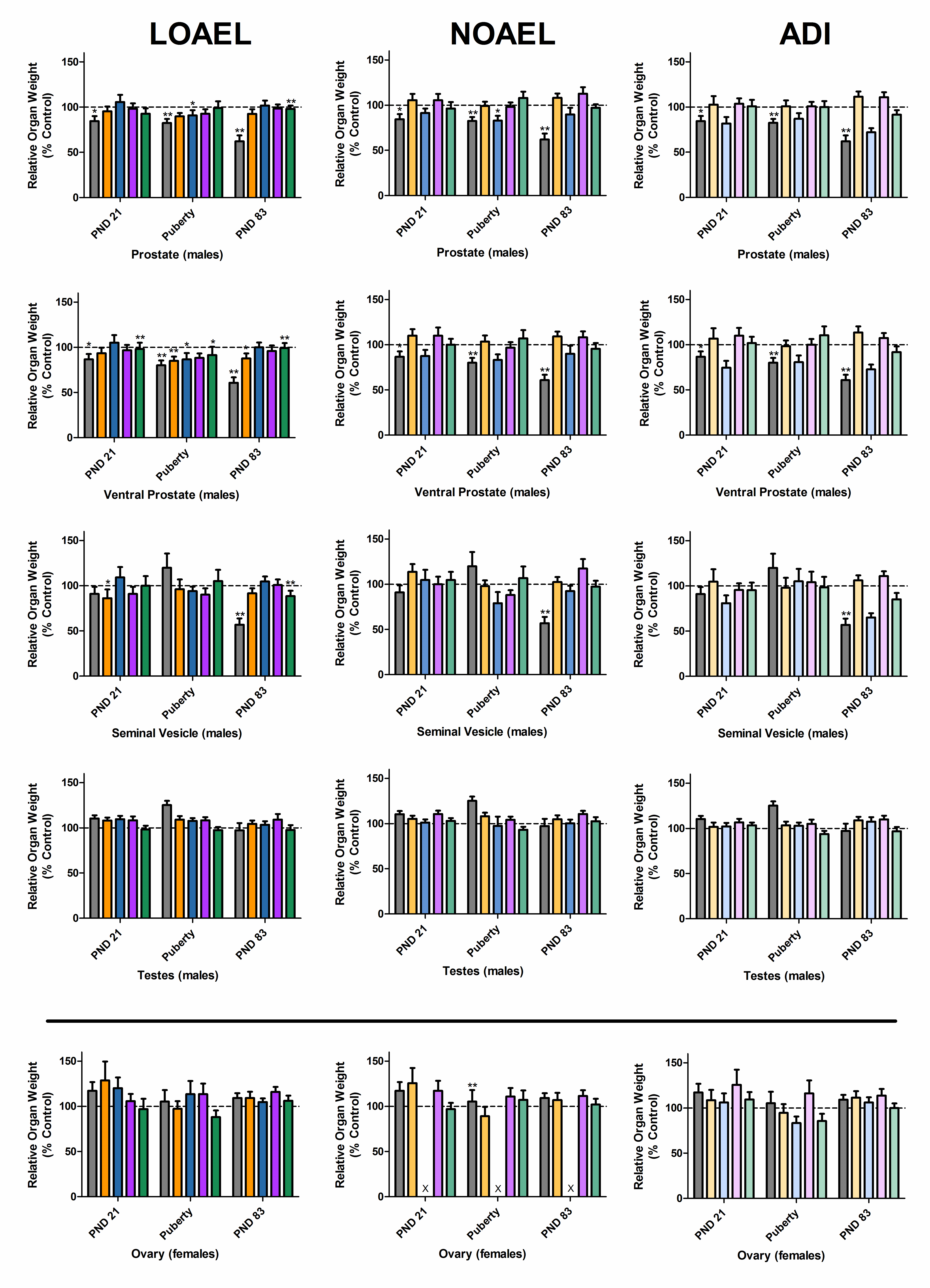


Supplementary Figure 6: Comparison of the weights of the other half of the sexual organs of male rats exposed to anti-androgens. On PND 21 (Subset 1), the day of preputial separation or vaginal opening (Puberty, Subset 2) and PND 83±2 (Subset 3), the sex organs from each of 10 male and 10 female rats were asservated, weighed and reported as relative organ weights. The relative organ weights of the mixture-treated animals were then compared to the effects of the single-substance exposures to vinclozolin , flutamide and prochloraz at each dose level: LOAEL *(left panels),* NOAEL *(center panels)* and ADI *(right panels)* as well as vehicle and positive controls . (Data are shown as means ± SD and all statistical evaluations are based on comparisons between a treatment group and its concurrent control group.) Generally, male sex organ weights were reduced in animals exposed to the anti-androgens at the LOAEL level, but not the NOAEL or ADI. No effect was observed in the female sex organs, as represented here by the ovaries. *Note: ^X^No control data available for normalization.*


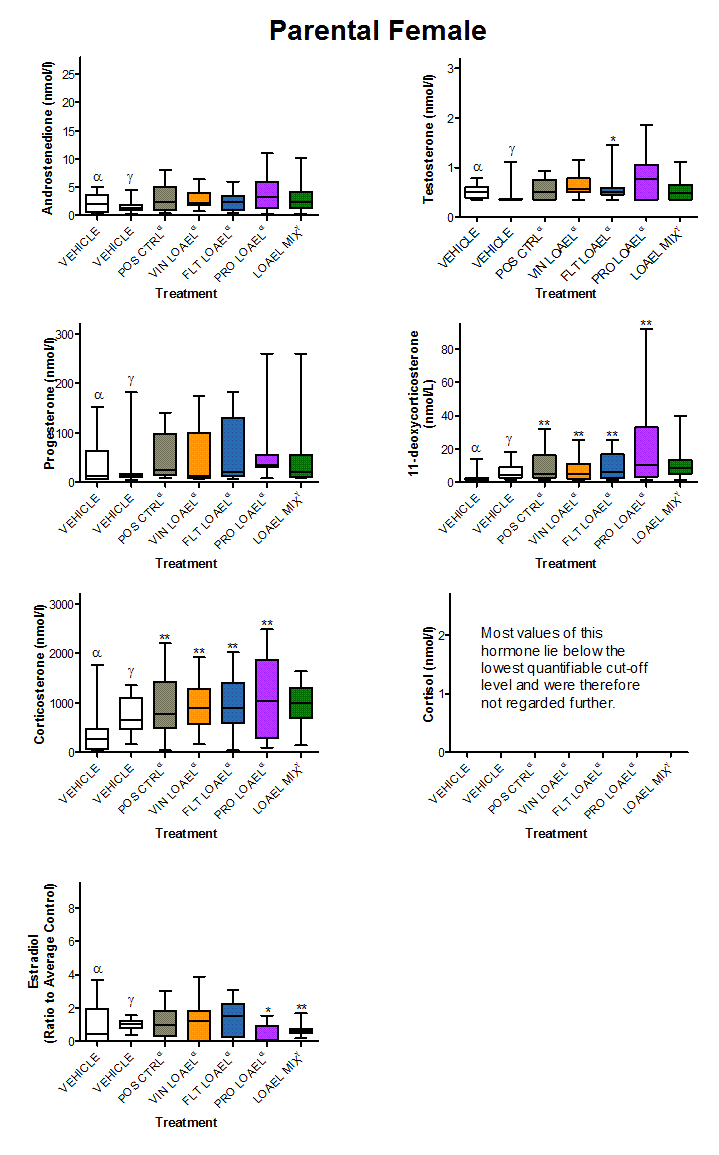


Supplementary Figure 7: Comparison of serum hormone levels in parental females after single-substance and mixed exposures to anti-androgens at LOAEL levels. For technical reasons, these investigations were performed over two years as three separate experiments of similar study design. Therefore, all statistical evaluations of the treatment data are based on the comparison between a treatment group and its concurrent control.


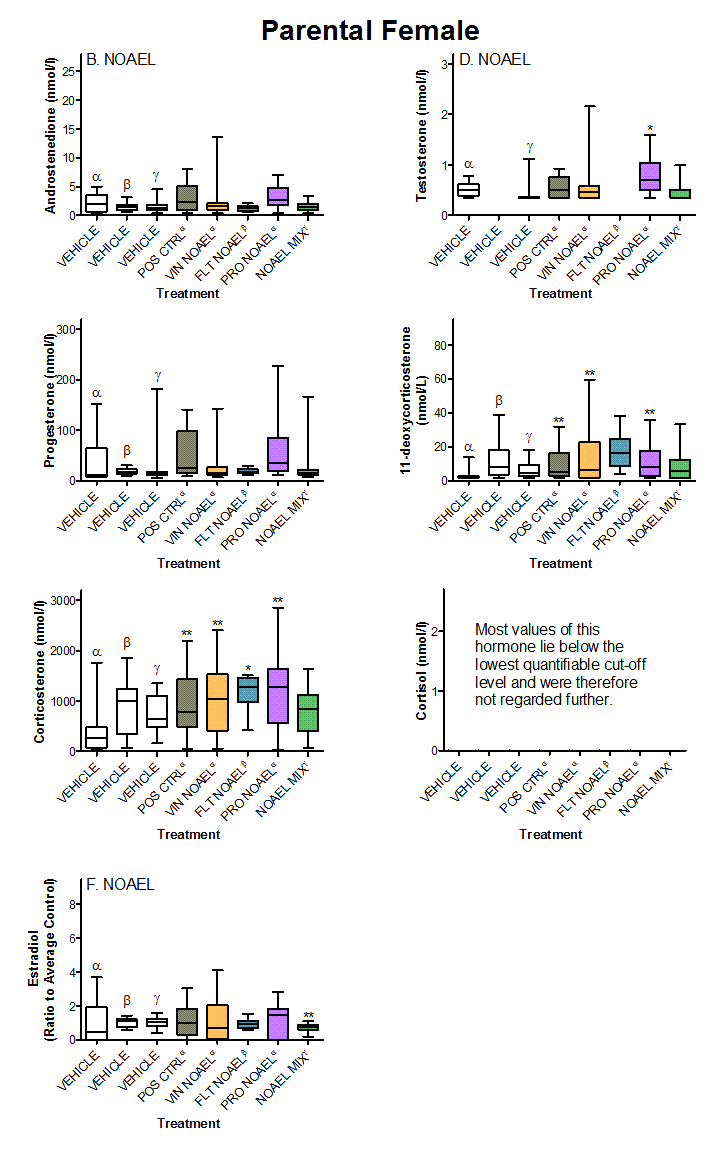


Supplementary Figure 8: Comparison of serum hormone levels in parental females after single-substance and mixed exposures to anti-androgens at NOAEL levels. For technical reasons, these investigations were performed over two years as three separate experiments of similar study design. Therefore, all statistical evaluations of the treatment data are based on the comparison between a treatment group and its concurrent control.


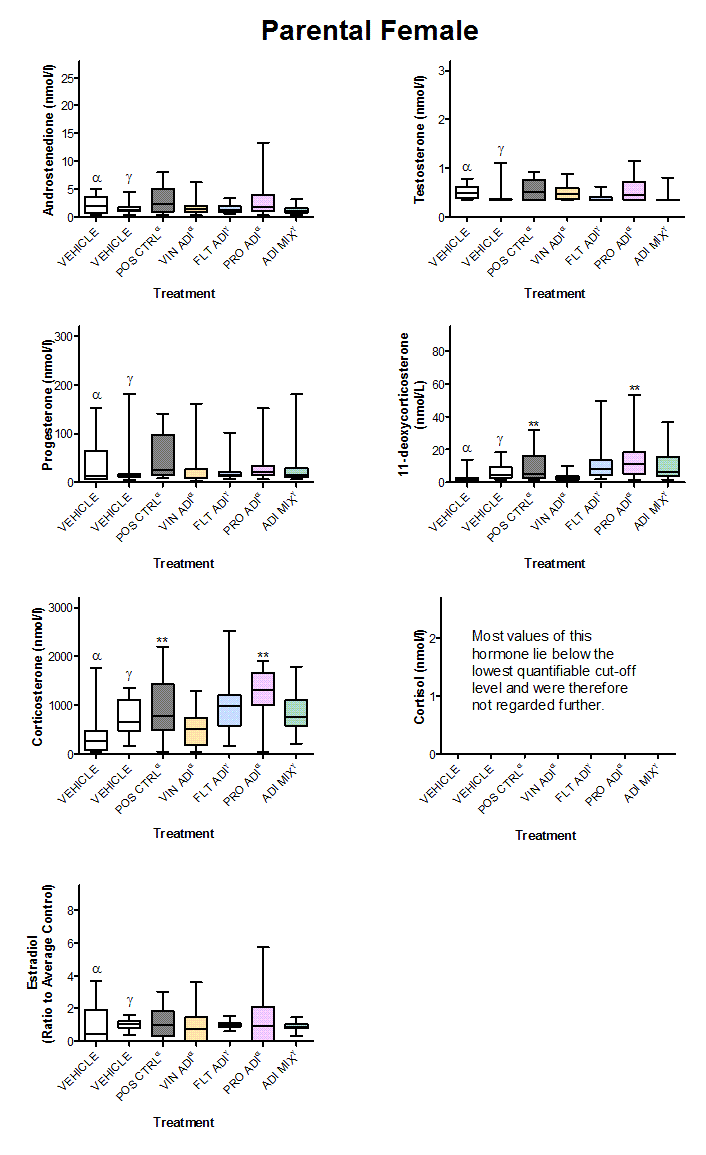


Supplementary Figure 9: Comparison of serum hormone levels in parental females after single-substance and mixed exposures to anti-androgens at ADI levels. For technical reasons, these investigations were performed over two years as three separate experiments of similar study design. Therefore, all statistical evaluations of the treatment data are based on the comparison between a treatment group and its concurrent control.


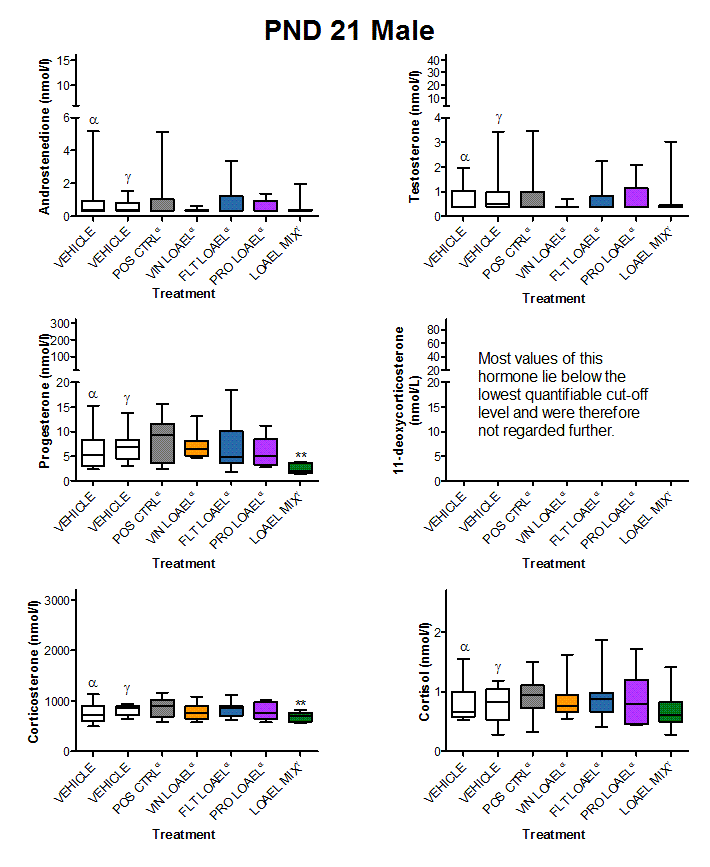


Supplementary Figure 10: Comparison of serum hormone levels in PND 21 male offspring after single-substance and mixed exposures to anti-androgens at LOAEL levels. For technical reasons, these investigations were performed over two years as three separate experiments of similar study design. Therefore, all statistical evaluations of the treatment data are based on the comparison between a treatment group and its concurrent control.


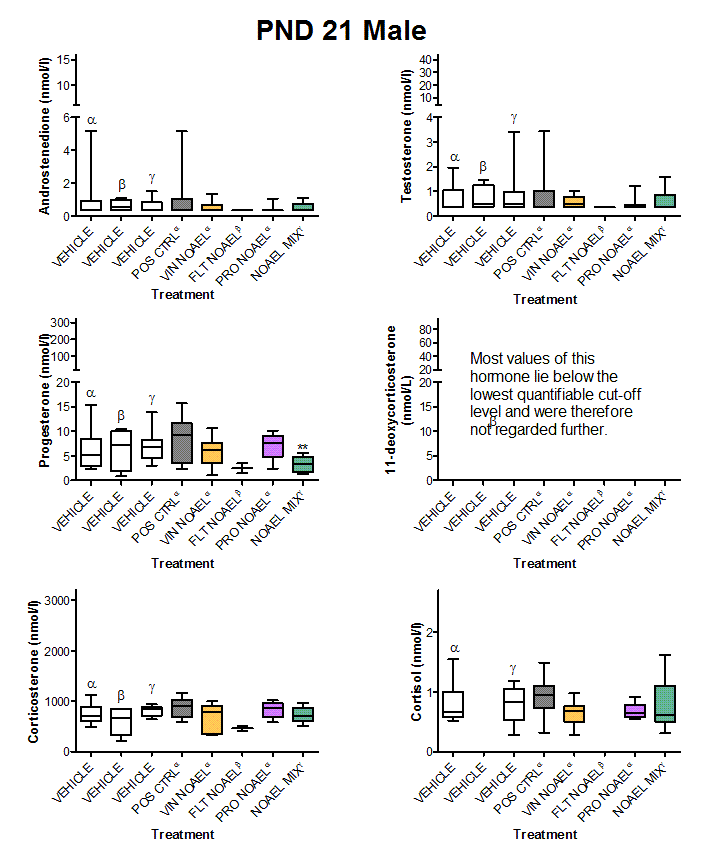


Supplementary Figure 11: Comparison of serum hormone levels in PND 21 male offspring after single-substance and mixed exposures to anti-androgens at NOAEL levels. For technical reasons, these investigations were performed over two years as three separate experiments of similar study design. Therefore, all statistical evaluations of the treatment data are based on the comparison between a treatment group and its concurrent control.


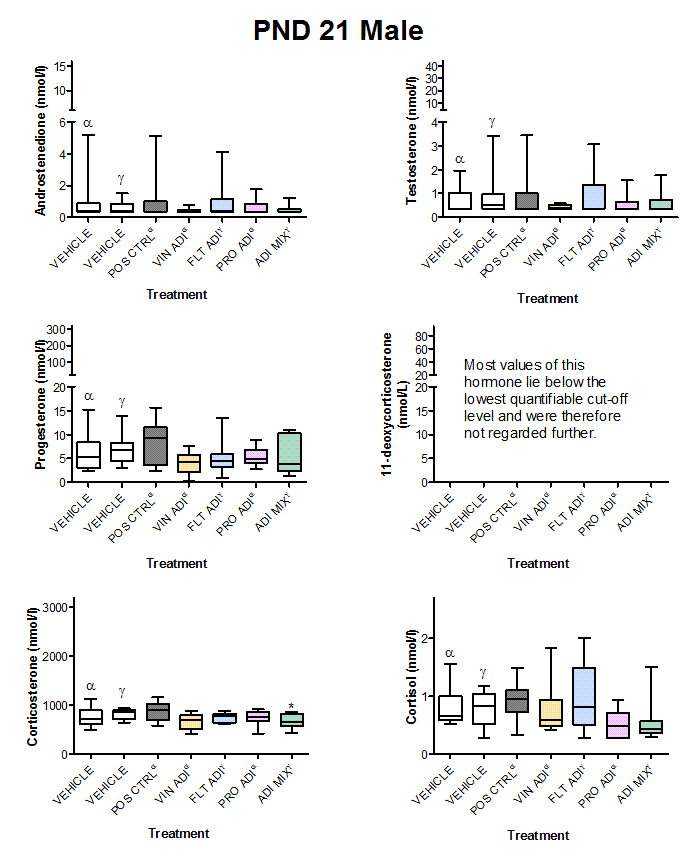


Supplementary Figure 12: Comparison of serum hormone levels in PND 21 male offspring after single-substance and mixed exposures to anti-androgens at ADI levels. For technical reasons, these investigations were performed over two years as three separate experiments of similar study design. Therefore, all statistical evaluations of the treatment data are based on the comparison between a treatment group and its concurrent control.


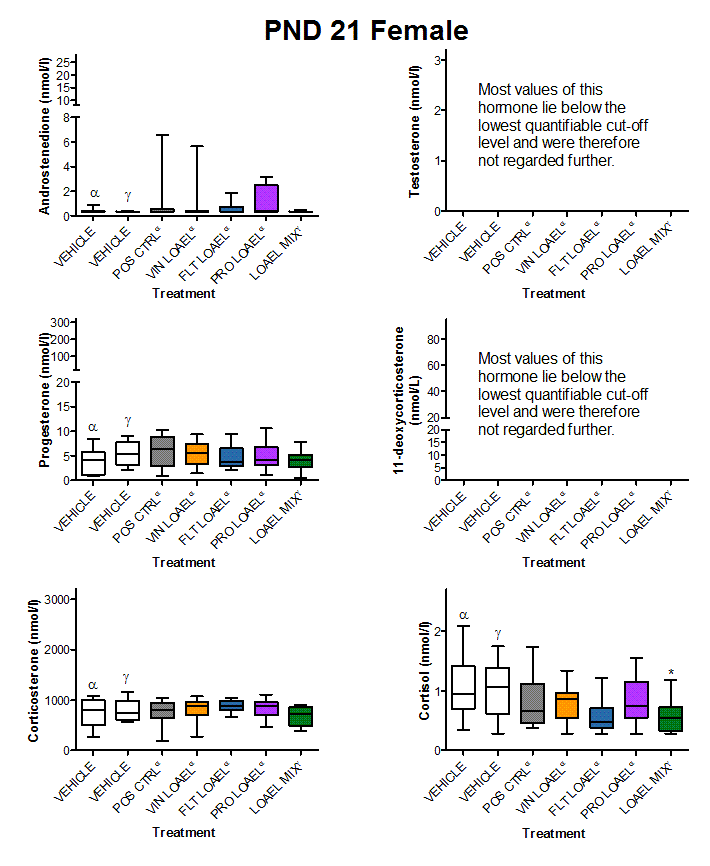


Supplementary Figure 13: Comparison of serum hormone levels in PND 21 female offspring after single-substance and mixed exposures to anti-androgens at LOAEL levels. For technical reasons, these investigations were performed over two years as three separate experiments of similar study design. Therefore, all statistical evaluations of the treatment data are based on the comparison between a treatment group and its concurrent control.


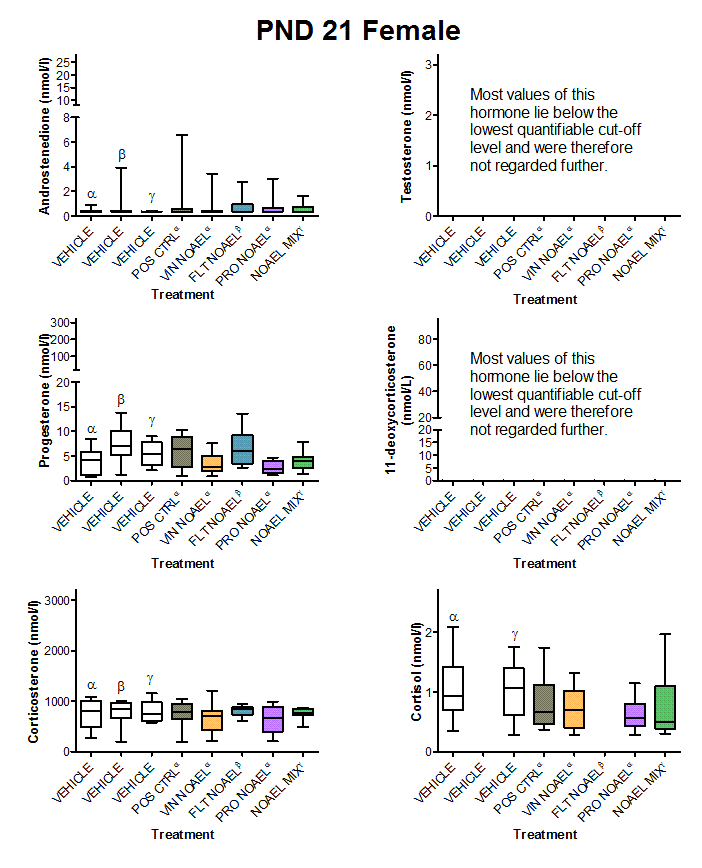


Supplementary Figure 14: Comparison of serum hormone levels in PND 21 female offspring after single-substance and mixed exposures to anti-androgens at NOAEL levels. For technical reasons, these investigations were performed over two years as three separate experiments of similar study design. Therefore, all statistical evaluations of the treatment data are based on the comparison between a treatment group and its concurrent control.


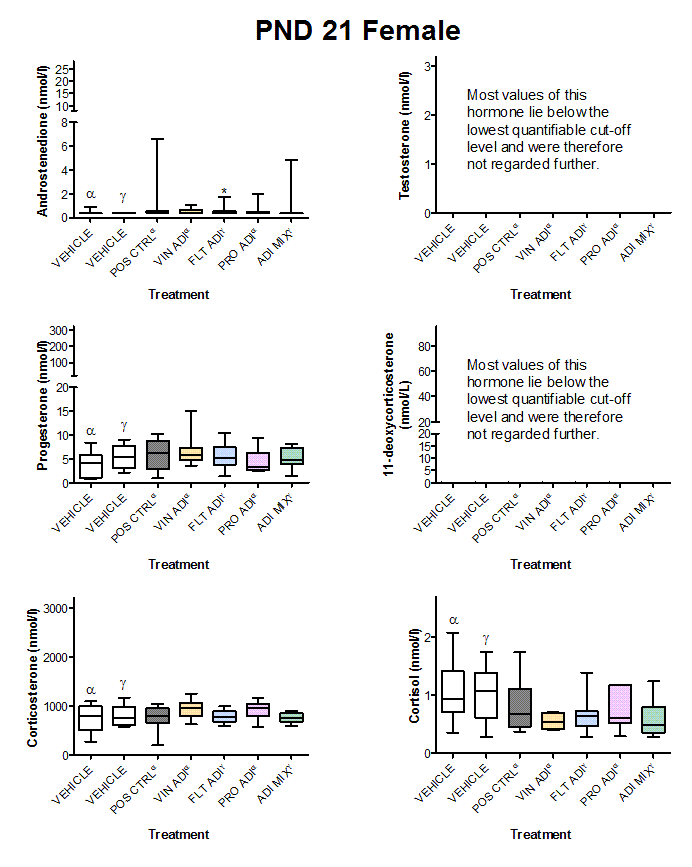


Supplementary Figure 15: Comparison of serum hormone levels in PND 21 female offspring after single-substance and mixed exposures to anti-androgens at ADI levels. For technical reasons, these investigations were performed over two years as three separate experiments of similar study design. Therefore, all statistical evaluations of the treatment data are based on the comparison between a treatment group and its concurrent control.


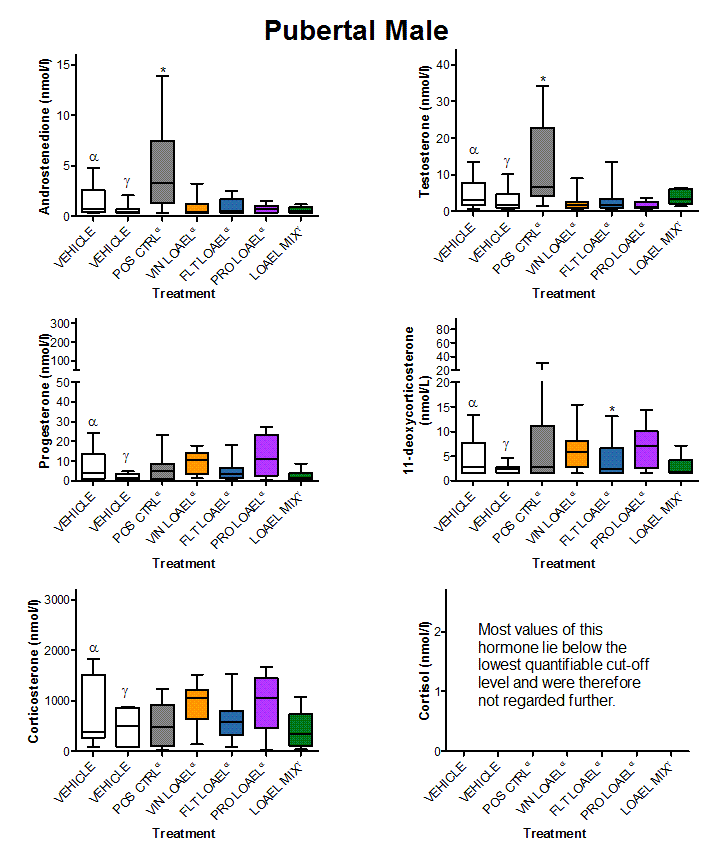


Supplementary Figure 16: Comparison of serum hormone levels in male offspring on the day of sexual maturation after single-substance and mixed exposures to anti-androgens at LOAEL levels. For technical reasons, these investigations were performed over two years as three separate experiments of similar study design. Therefore, all statistical evaluations of the treatment data are based on the comparison between a treatment group and its concurrent control.


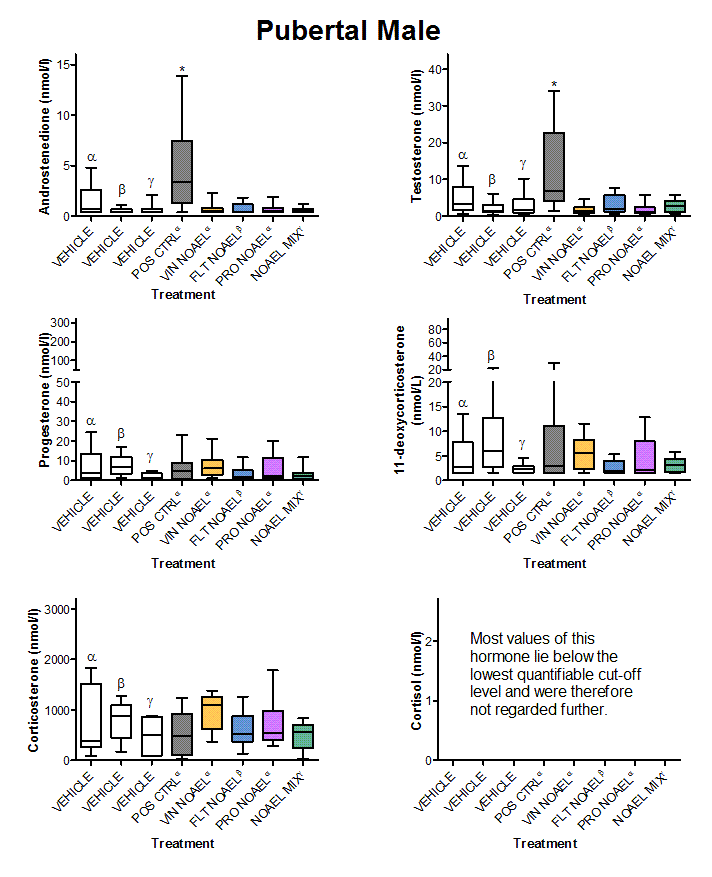


Supplementary Figure 17: Comparison of serum hormone levels in male offspring on the day of sexual maturation after single-substance and mixed exposures to anti-androgens at NOAEL levels. For technical reasons, these investigations were performed over two years as three separate experiments of similar study design. Therefore, all statistical evaluations of the treatment data are based on the comparison between a treatment group and its concurrent control.


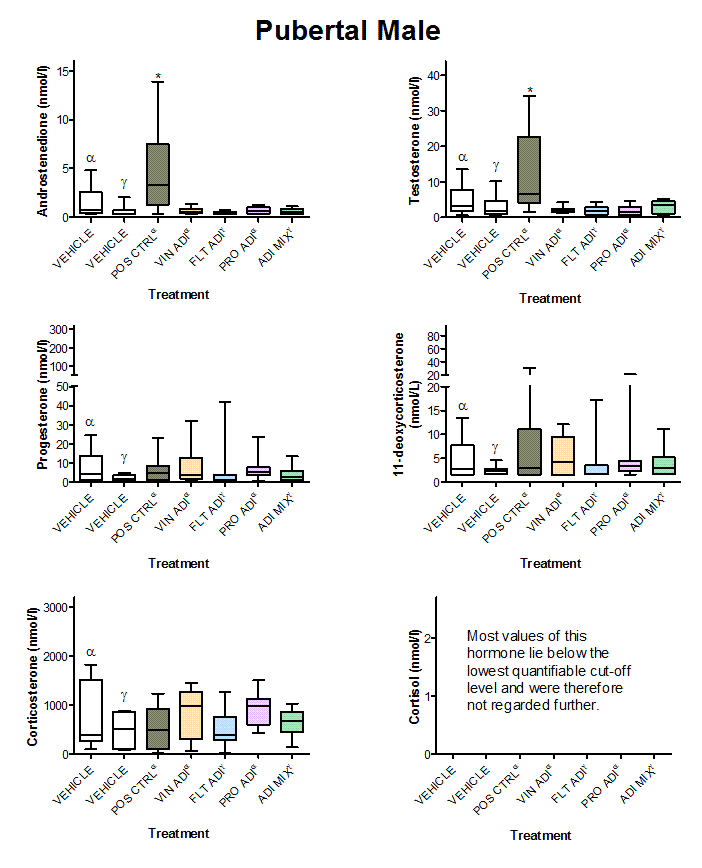


Supplementary Figure 18: Comparison of serum hormone levels in male offspring on the day of sexual maturation after single-substance and mixed exposures to anti-androgens at ADI levels. For technical reasons, these investigations were performed over two years as three separate experiments of similar study design. Therefore, all statistical evaluations of the treatment data are based on the comparison between a treatment group and its concurrent control.


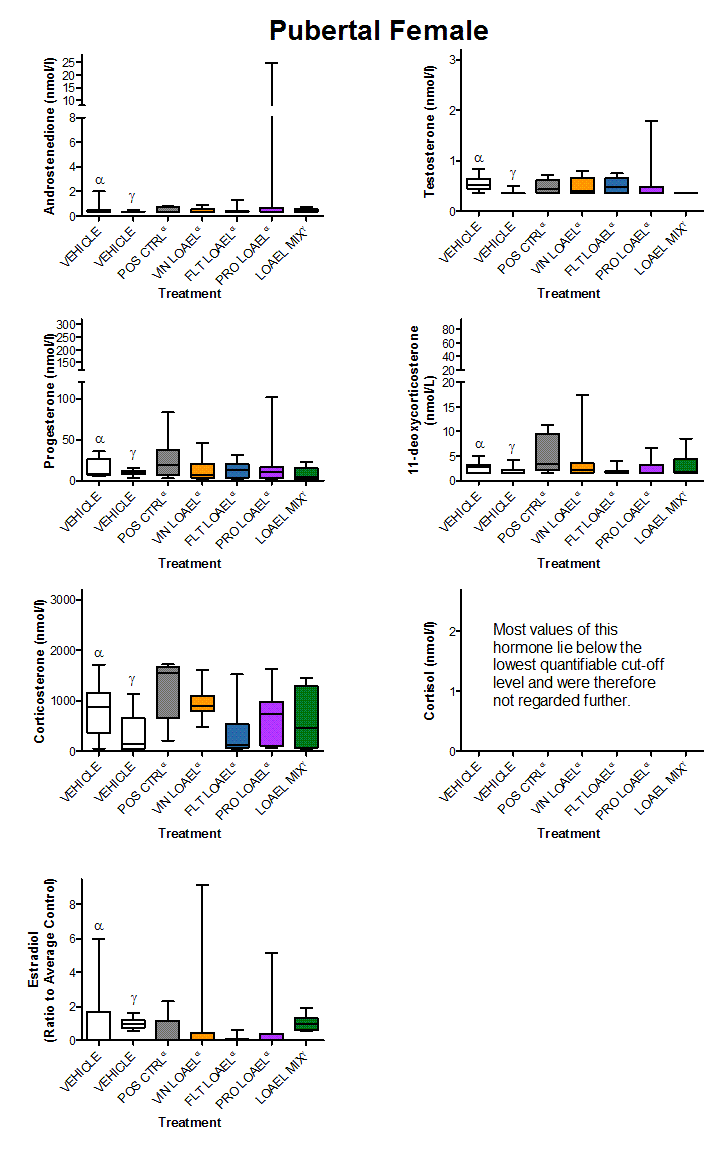


Supplementary Figure 19: Comparison of serum hormone levels in female offspring on the day of sexual maturation after single-substance and mixed exposures to anti-androgens at LOAEL levels. For technical reasons, these investigations were performed over two years as three separate experiments of similar study design. Therefore, all statistical evaluations of the treatment data are based on the comparison between a treatment group and its concurrent control.


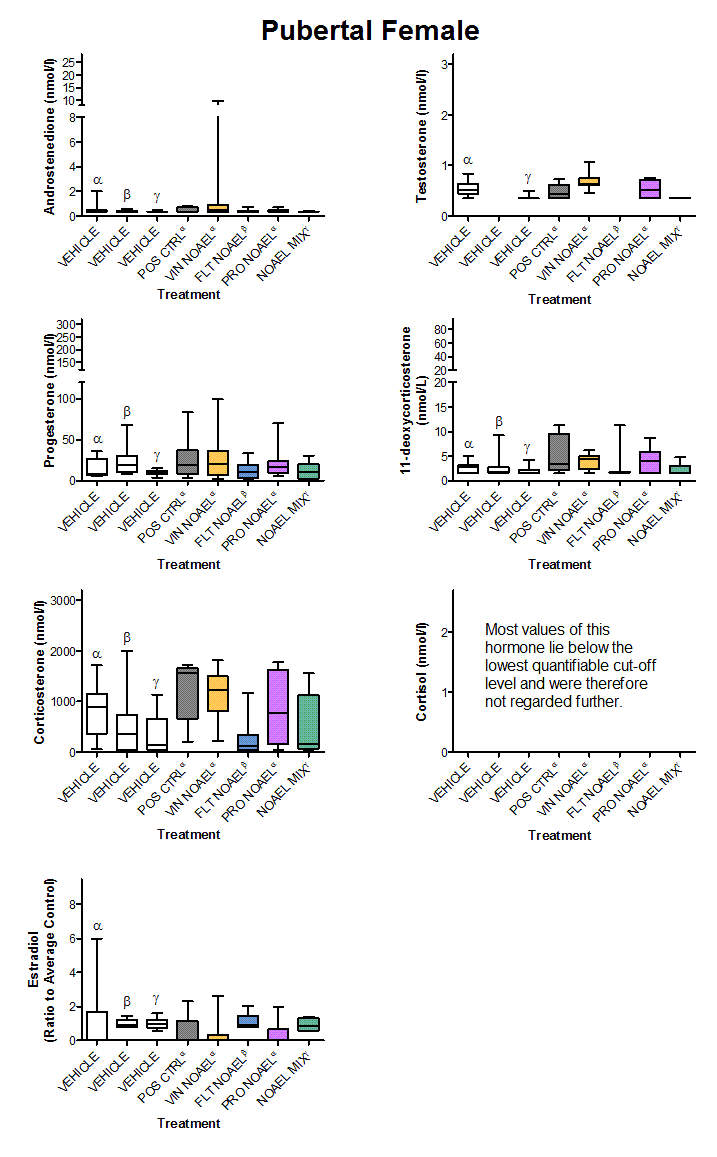


Supplementary Figure 20: Comparison of serum hormone levels in female offspring on the day of sexual maturation after single-substance and mixed exposures to anti-androgens at NOAEL levels. For technical reasons, these investigations were performed over two years as three separate experiments of similar study design. Therefore, all statistical evaluations of the treatment data are based on the comparison between a treatment group and its concurrent control.


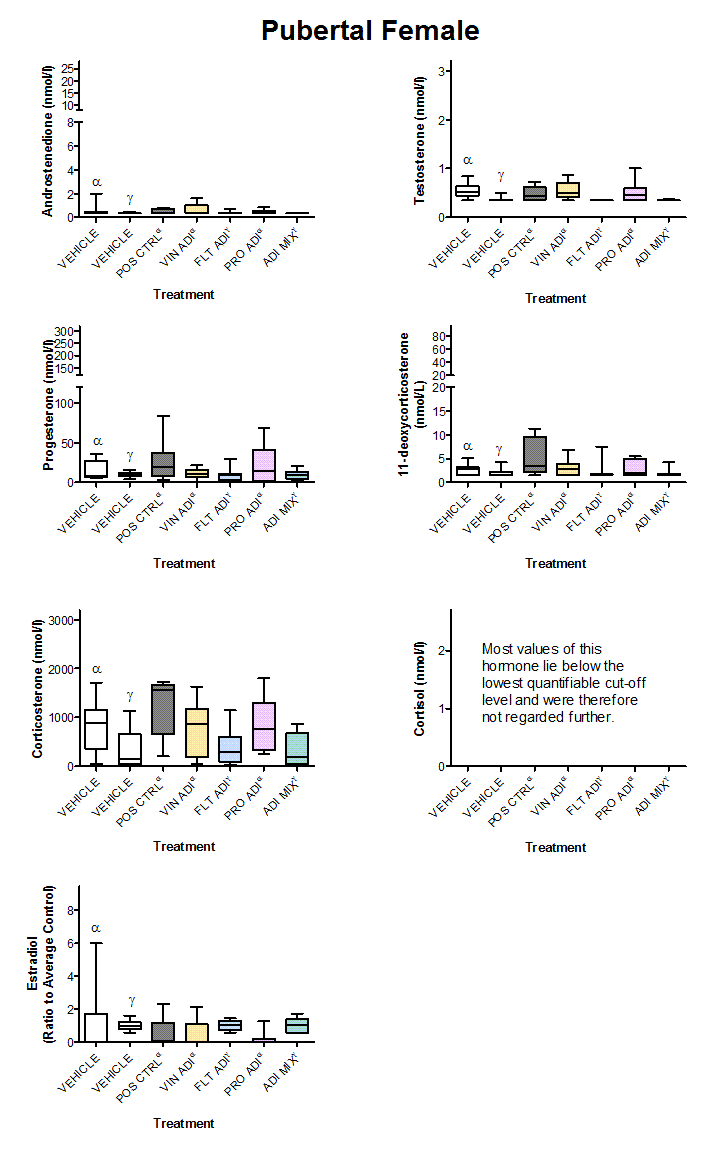


Supplementary Figure 21: Comparison of serum hormone levels in female offspring on the day of sexual maturation after single-substance and mixed exposures to anti-androgens at ADI levels. For technical reasons, these investigations were performed over two years as three separate experiments of similar study design. Therefore, all statistical evaluations of the treatment data are based on the comparison between a treatment group and its concurrent control.


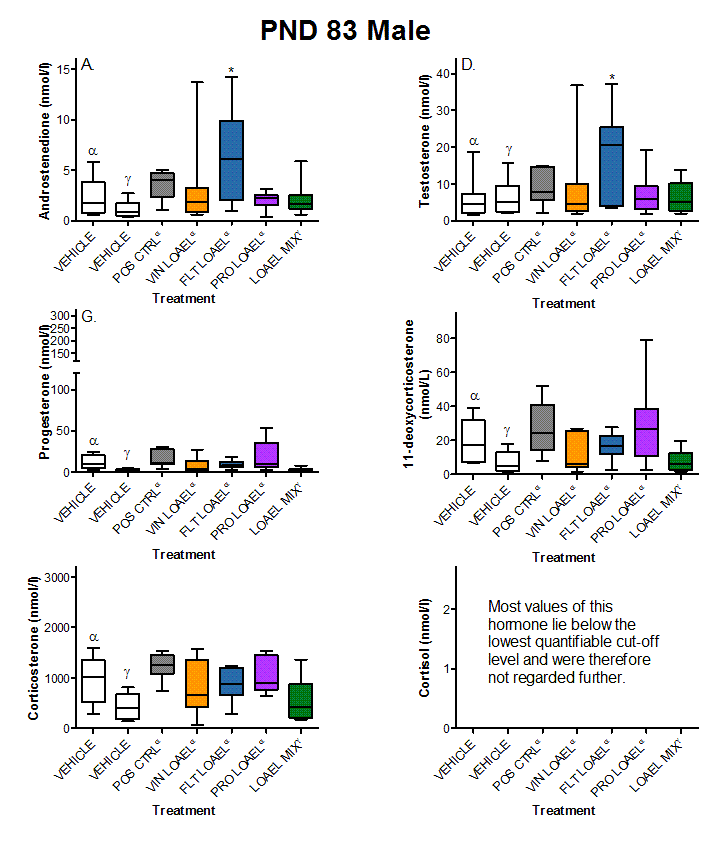


Supplementary Figure 22: Comparison of serum hormone levels in PND 83±2 male offspring after single-substance and mixed exposures to anti-androgens at LOAEL levels. For technical reasons, these investigations were performed over two years as three separate experiments of similar study design. Therefore, all statistical evaluations of the treatment data are based on the comparison between a treatment group and its concurrent control.


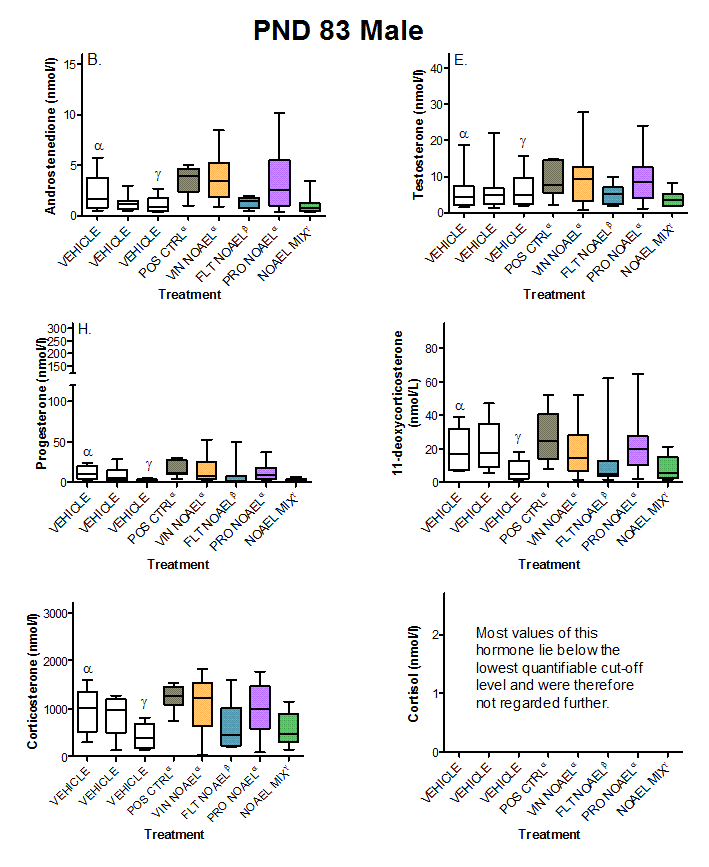


Supplementary Figure 23: Comparison of serum hormone levels in PND 83±2 male offspring after single-substance and mixed exposures to anti-androgens at NOAEL levels. For technical reasons, these investigations were performed over two years as three separate experiments of similar study design. Therefore, all statistical evaluations of the treatment data are based on the comparison between a treatment group and its concurrent control.


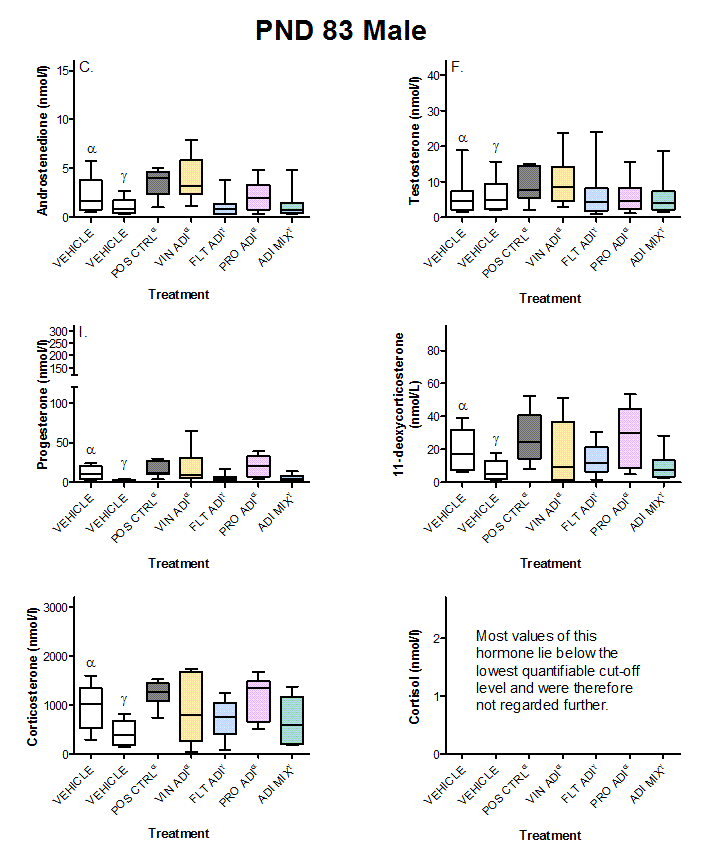


Supplementary Figure 24: Comparison of serum hormone levels in PND 83±2 male offspring after single-substance and mixed exposures to anti-androgens at ADI levels. For technical reasons, these investigations were performed over two years as three separate experiments of similar study design. Therefore, all statistical evaluations of the treatment data are based on the comparison between a treatment group and its concurrent control.


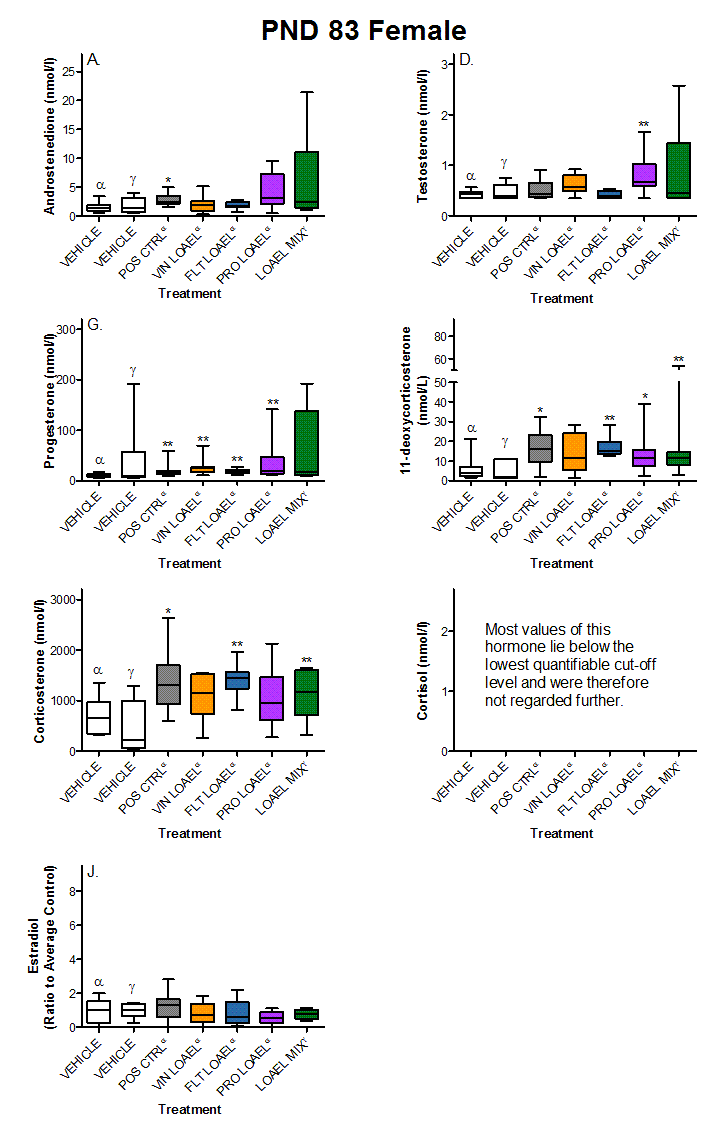


Supplementary Figure 25: Comparison of serum hormone levels in PND 83±2 female offspring after single-substance and mixed exposures to anti-androgens at LOAEL levels. For technical reasons, these investigations were performed over two years as three separate experiments of similar study design. Therefore, all statistical evaluations of the treatment data are based on the comparison between a treatment group and its concurrent control.


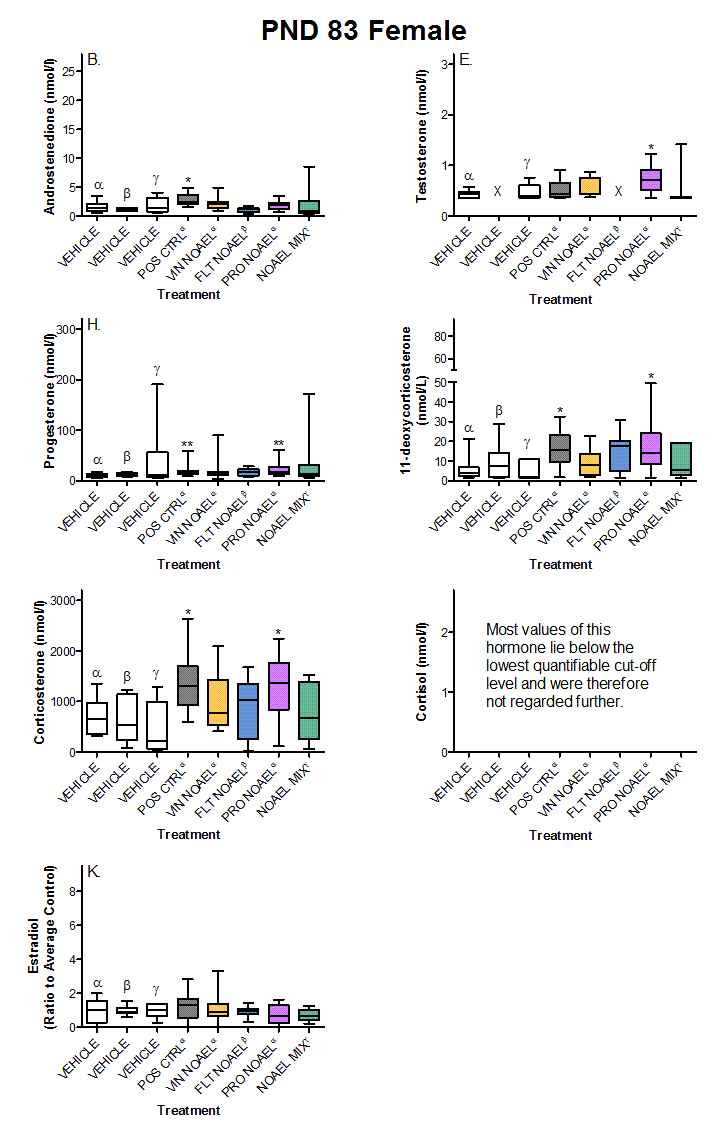


Supplementary Figure 26: Comparison of serum hormone levels in PND 83±2 female offspring after single-substance and mixed exposures to anti-androgens at NOAEL levels. For technical reasons, these investigations were performed over two years as three separate experiments of similar study design. Therefore, all statistical evaluations of the treatment data are based on the comparison between a treatment group and its concurrent control.


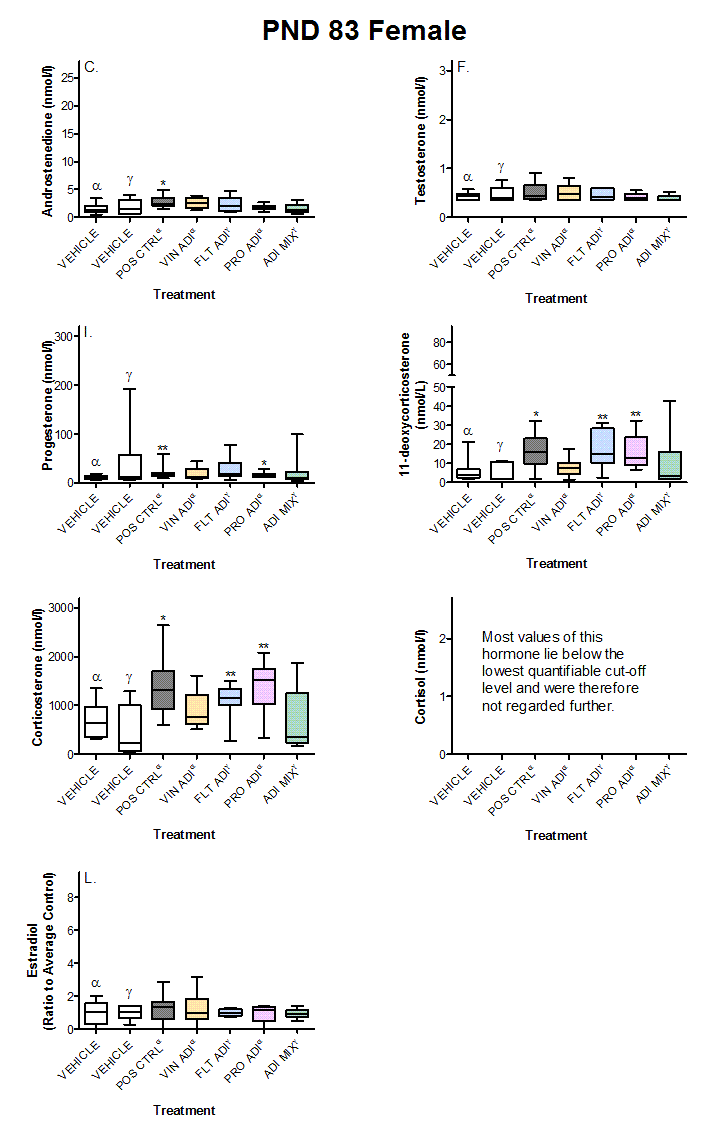


Supplementary Figure 27: Comparison of serum hormone levels in PND 83±2 female offspring after single-substance and mixed exposures to anti-androgens at ADI levels. For technical reasons, these investigations were performed over two years as three separate experiments of similar study design. Therefore, all statistical evaluations of the treatment data are based on the comparison between a treatment group and its concurrent control.


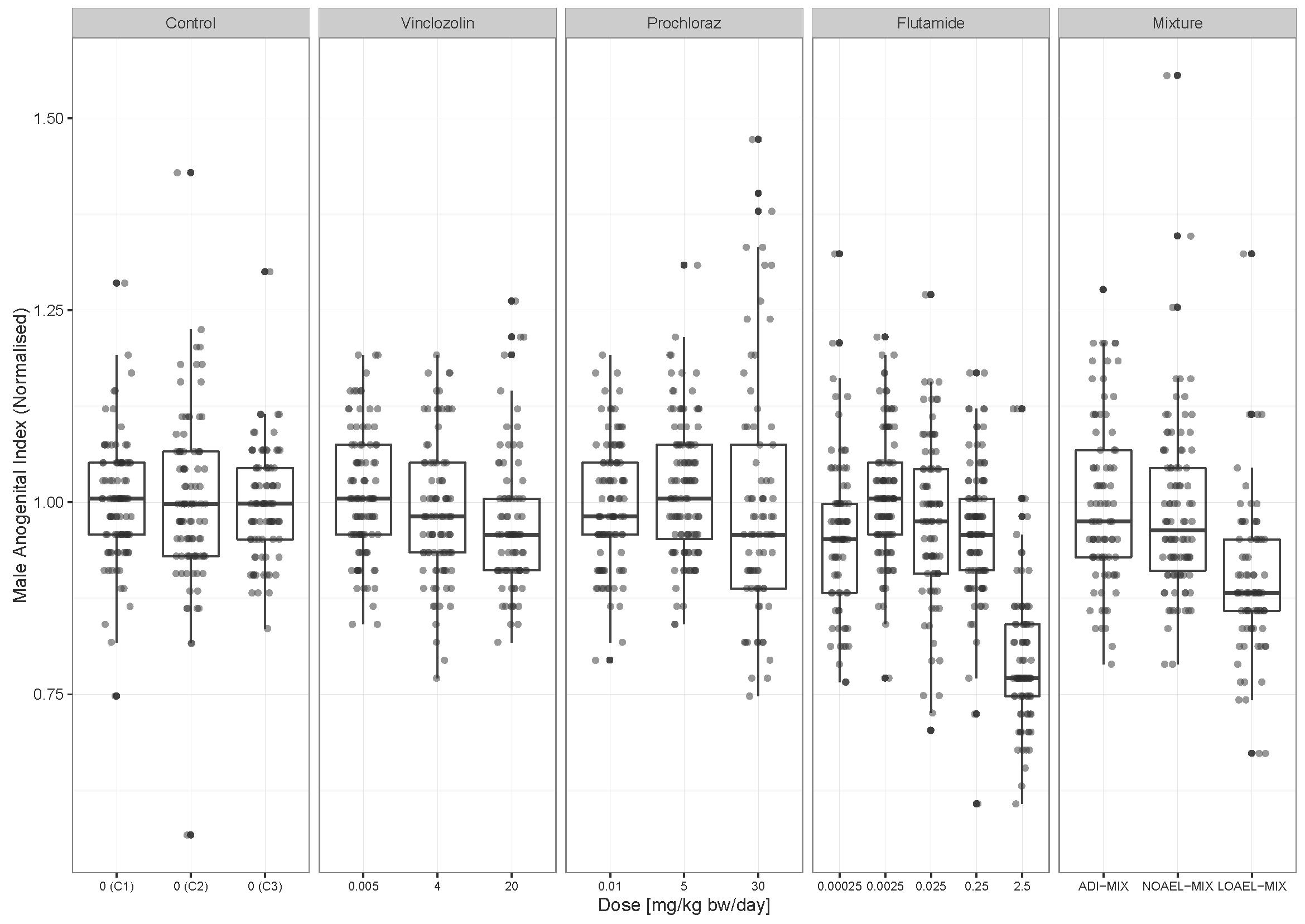


Supplementary Figure 28: Boxplot of normalised male anogential index for individual male offspring in control and each treatment group.


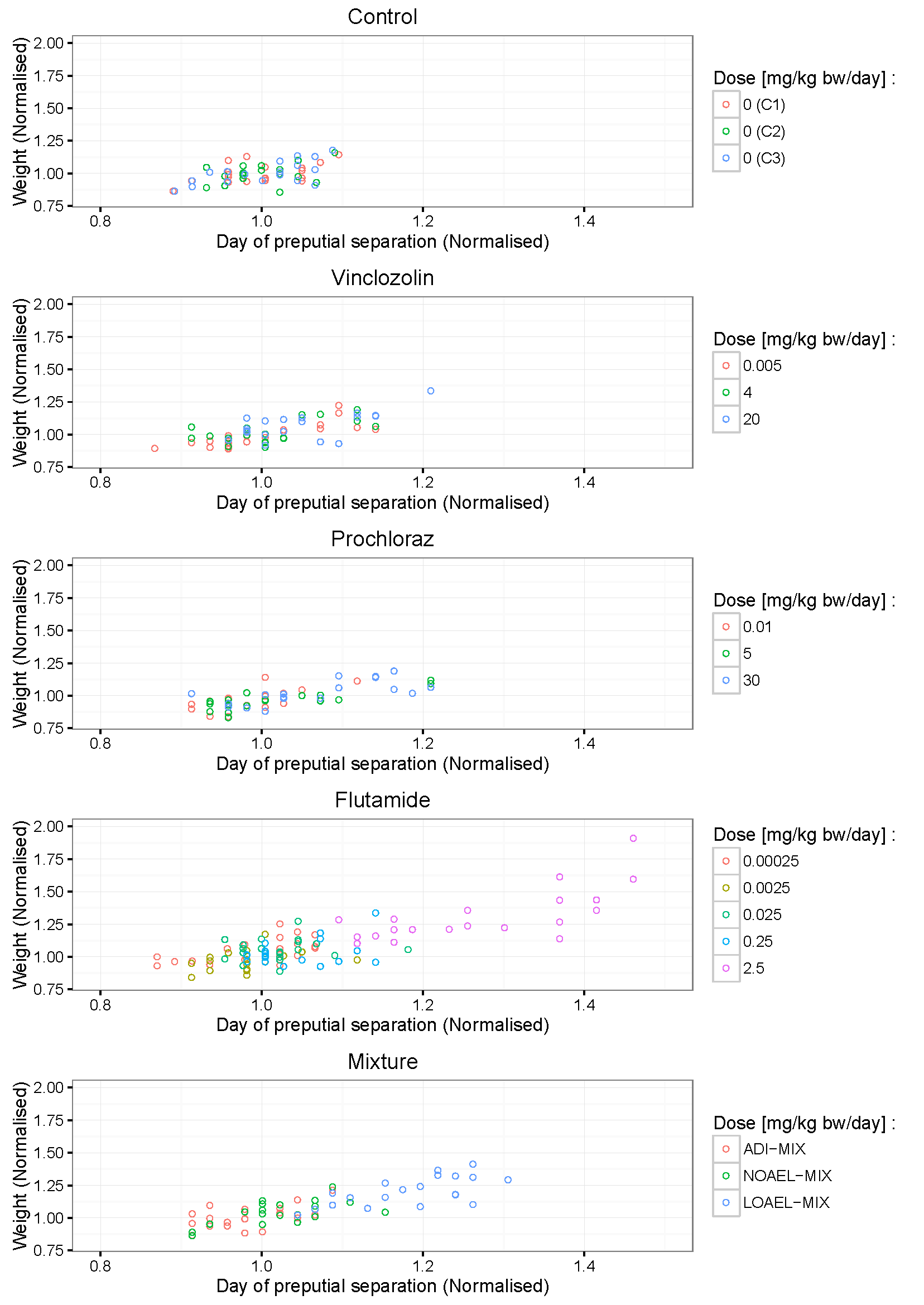


Supplementary Figure 29: Scatter plot of body weight for individual male offspring versus day of preputial separation.


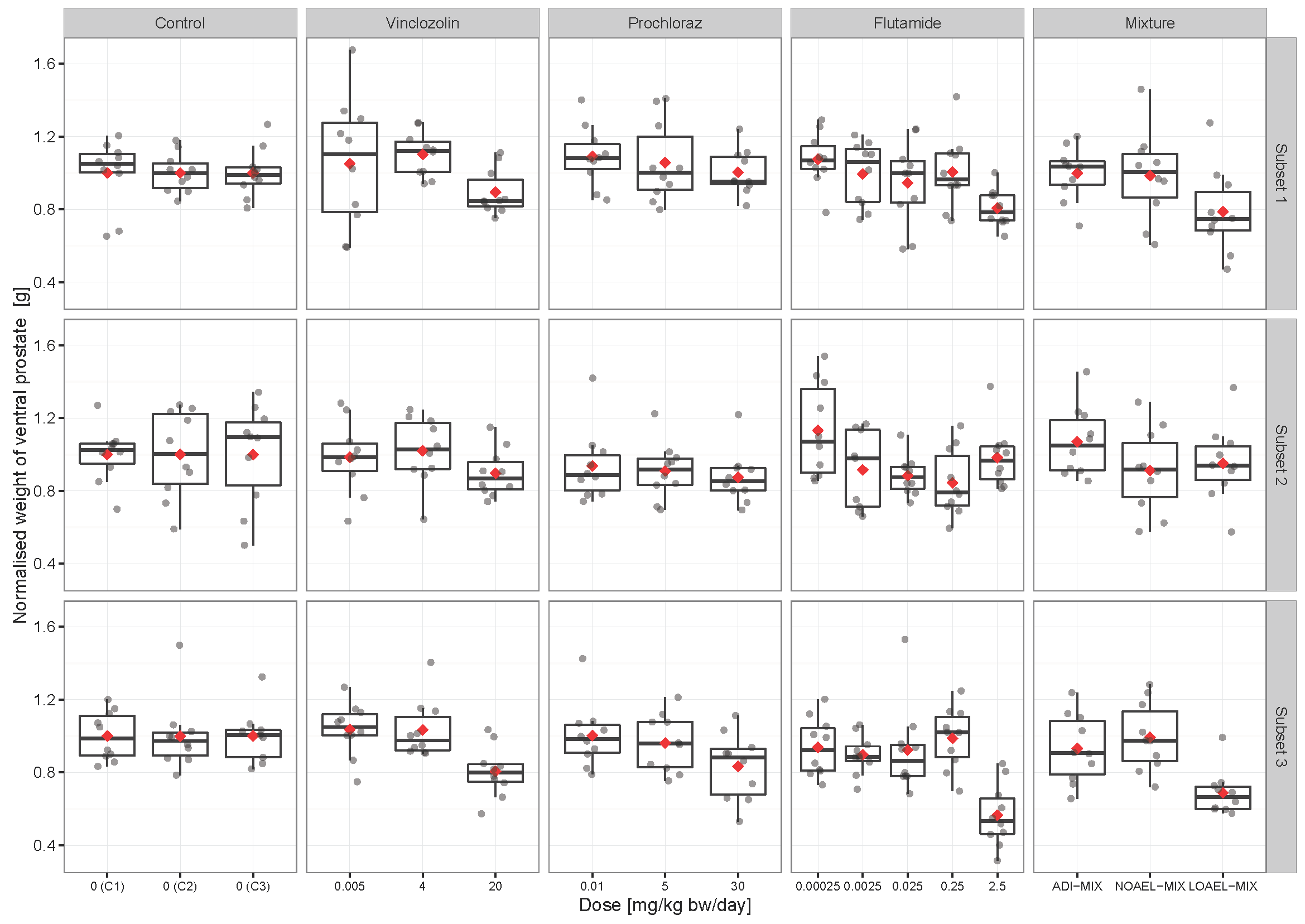


Supplementary Figure 30: Boxplot of normalised weight of ventral prostate for individual male offspring in control and each treatment group. Red squares indicate the mean of each group.


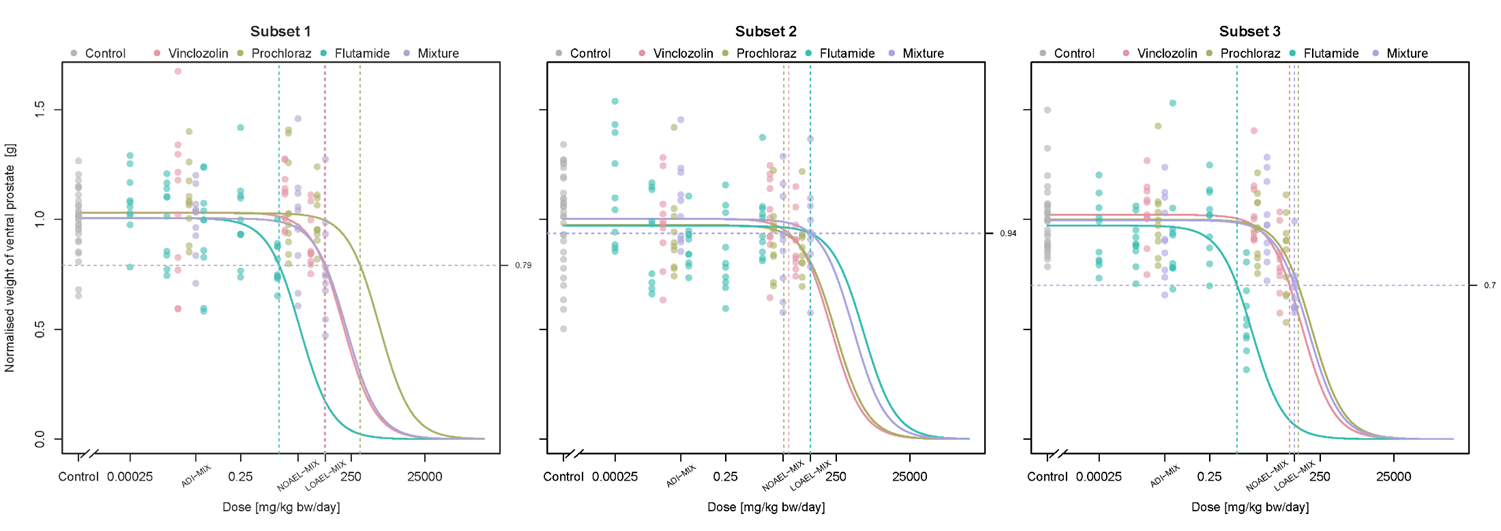


Supplementary Figure 31: Dose-response curve fits of the weight of ventral prostate for the individual chemicals and mixture experiment.


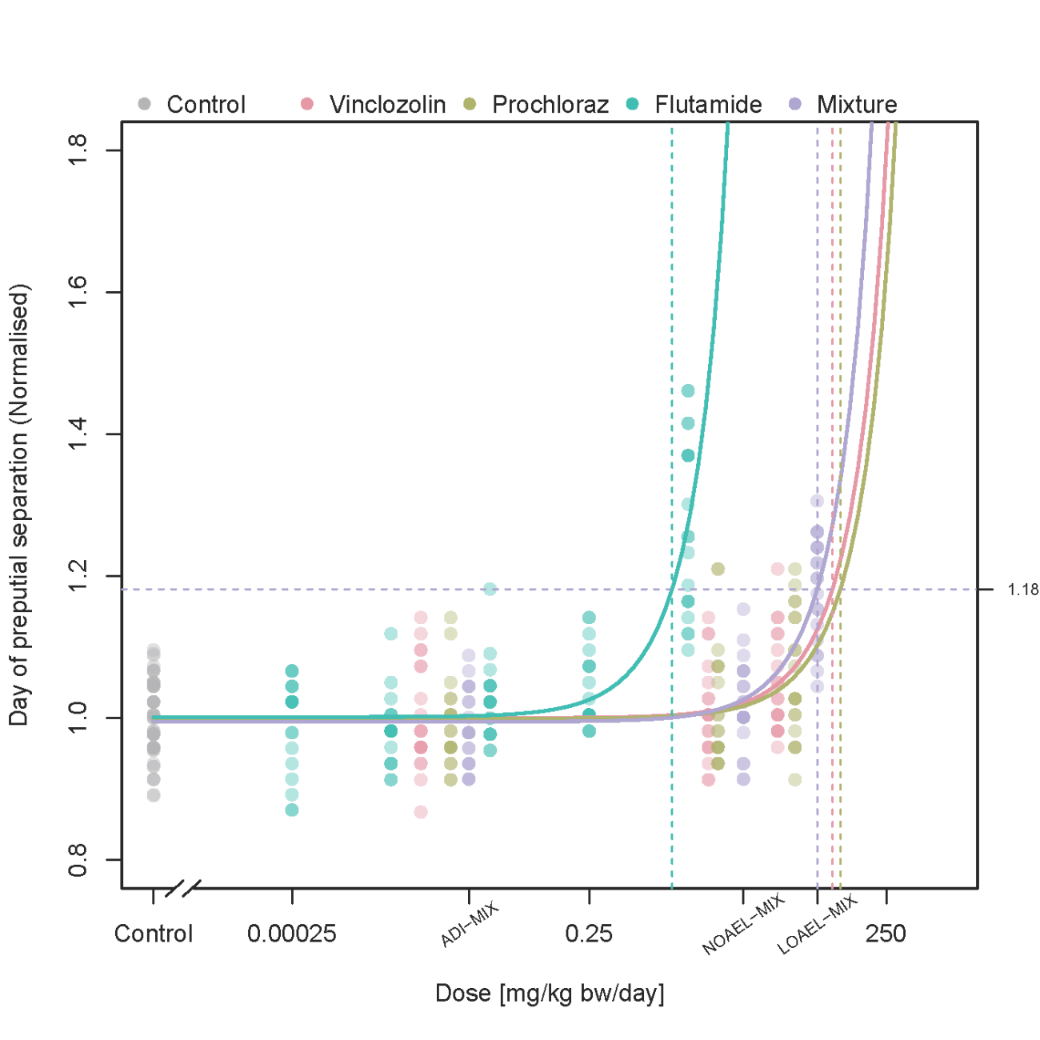


Supplementary Figure 32: Dose-response curve fits of day of preputial separation for the individual chemicals and mixture experiment.

Supplementary Table 1: Investigated tissues

|  | **Parental Females** | **F1 Offspring** | | |
| --- | --- | --- | --- | --- |
|  |  | **Subset 1 (PND 21)** | **Subset 2 (Puberty)** | **Subset 3 (PND 83)** |
| All gross lesions | Fixed only | H&E Histopathology | H&E Histopathology | H&E Histopathology |
| Adrenal glands | Fixed only | H&E Histopathology | H&E Histopathology | H&E Histopathology |
| Brain | Fixed only | Fixed only | Fixed only | Fixed only |
| Cervix uteri | Fixed only | Fixed only | Fixed only | Fixed only |
| Left coagulating gland |  | H&E Histopathology | H&E Histopathology | H&E Histopathology |
| Right coagulating gland |  | Fixed only | Fixed only | Fixed only |
| Left epididymis |  | H&E Histopathology | H&E Histopathology | H&E Histopathology |
| Right epididymis |  | Fixed only | Fixed only | Fixed only |
| Mammary gland incl. skin (both male and female) |  | Fixed only | Fixed only | Fixed only |
| Kidneys | Fixed only | Fixed only | Fixed only | Fixed only |
| Liver | Fixed only | Fixed only | Fixed only | Fixed only |
| Ovaries | Fixed only | Fixed only | Fixed only | Fixed only |
| Ovaducts | Fixed only | Fixed only | Fixed only | Fixed only |
| Pituitary gland | Fixed only | H&E Histopathology | H&E Histopathology | H&E Histopathology |
| Dorsolateral prostate |  | H&E Histopathology | H&E Histopathology | H&E Histopathology |
| Ventral prostate |  | 1/2: H&E Histopathology,  1/2: Flash frozen | 1/2: H&E Histopathology,  1/2: Flash frozen | 1/2: H&E Histopathology,  1/2: Flash frozen |
| Left seminal vesicle |  | H&E Histopathology | H&E Histopathology | H&E Histopathology |
| Right seminal vesicle |  | Flash frozen | Flash frozen | Flash frozen |
| Spleen | Fixed only | Fixed only | Fixed only | Fixed only |
| Left testis |  | H&E Histopathology | H&E Histopathology | H&E Histopathology |
| Right testis |  | Flash frozen | Flash frozen | 1/2: Spermatology,  1/2: Flash frozen |
| Thyroid glands with parathyroid glands | Fixed only | Fixed only | Fixed only | Fixed only |
| Uterus | Fixed only | Fixed only | Fixed only | Fixed only |
| Vagina | Fixed only | Fixed only | Fixed only | Fixed only |

Supplementary Table 2: Statistical tests used to compare measured parameters

| **Parameter** | **Statistical test** |
| --- | --- |
| Food consumption, body weight and body weight change (parental animals and pups); estrous cycle length; duration of gestation; number of delivered pups per litter; developmental landmarks (days up to preputial separation or opening of the vagina); anogenital distance and index; implantation sites; postimplantation loss, weight of the fetuses, implantations, pre‑ and postimplantation losses, resorptions and live fetuses | DUNNETT test (two-sided) |
| Number of live and dead pups and different indices (e.g. mating index, fertility index and gestation index) and number of litters with necropsy findings in pups; developmental landmarks (preputial separation or opening of the vagina), sperm morphology, incidence of males with a specific amount of abnormal sperm (cutoff value: 0.9-quantile [90%] of control groups) | FISHER's exact test |
| Proportion of pups with necropsy findings per litter, presence of areolas/nipples, sperm evaluation (except for morphology) | WILCOXON test (one-sided) |
| Hormone measurements, weight of the anesthetized animals and absolute and relative organ weights (all organs excl. organs listed below) | KRUSKAL-WALLIS and WILCOXON test |
| Sex organ weight parameters including: bulbourethral gland (Cowpers gland), cauda epididymis, epididymides, musc. levator ani together with musc. bulbocavernosus, glans penis, prostate, ventral prostate, seminal vesicles with coagulating gland , testes and ovaries | DUNNETT’s test (one-sided) |

Supplementary Table 3: Effects of single-substance and mixed exposures to anti-androgens on pup development

| **Treatment** | **Anogenital Distance (mm)** | | **Anogenital index (mm/3√body weight)** | | **Male pup nipple retention** | | **Number of nipples retained** | | **Preputial Separation** | |
| --- | --- | --- | --- | --- | --- | --- | --- | --- | --- | --- |
|  | **male** | **female** | **male** | **female** | **PND 12** | **PND 20** | **PND 12** | **PND 20** | **Age** | **Weight** |
| **Vehicle Control^α^** | 3.07 ± 0.149 (20) 100% | 1.54 ± 0.132 (20) 100% | 0.43 ± 0.021 (20) 100% | 0.23 ± 0.015 (20) 100% | 67.2 ± 25.65% | 0.0 ± 0.00% | 1.8 ± 1.4 (20) | 0.0 ± 0.0 (20) | 43.8 ± 2.38 (20) | 178.2 ± 12.50 (15) |
| **Vehicle Control^β^** | 3.04 ± 0.209 (19) 100% | 1.52 ± 0.132 (20) 100% | 0.44 ± 0.029 (19) 100% | 0.24 ± 0.019 (19) 100% | 74.2 ± 22.07% | 0.0 ± 0.00% | 1.7 ± 0.8 (19) | 0.0 ± 0.0 (19) | 44.0 ± 1.95 (20) | 175.3 ± 12.75 (18) |
| **Vehicle Control^γ^** | 3.04 ± 0.108 (20) 100% | 1.43 ± 0.087 (20) 100% | 0.43 ± 0.020 (20) 100% | 0.21 ± 0.019 (20) 100% | 71.0± 22.09% | 0.0 ± 0.00% | 1.6 ± 0.8 (20) | 0.0 ± 0.0 (19) | 45.9 ± 2.76 (19) | 183.9 ± 15.45 (16) |
| **Positive Control^α^** | **2.41**** ± 0.224 (20) 78% | 1.41 ± 0.173 (20) 92% | **0.34**** ± 0.037 (20) 79% | 0.23 ± 0.015 (20) 100% | **100.0**** ± 0.00% | **75.6**** ± 22.40% | **7.9**** ± 0.9 (18) | **1.4**** ± 0.9 (18) | **55.7**** ± 5.42 (20) | **236.4****± 34.65 (15) |
| **VIN ADI^α^** | 3.04 ± 0.139 (19) 99% | 1.50 ± 0.178 (19) 97% | 0.43 ± 0.024 (19) 100% | 0.22 ± 0.024 (19) 96% | 80.3 ± 24.51% | 0.0 ± 0.00% | 2.3 ± 1.3 (19) | 0.0 ± 0.0 (19) | 43.8 ± 3.28 (20) | 178.3 ± 13.55 (15) |
| **VIN NOAEL^α^** | 3.04 ± 0.135 (18) 99% | 1.57 ± 0.155 (18) 102% | 0.42 ± 0.029 (18) 98% | 0.23 ± 0.022 (18) 100% | 80.6 ± 20.62% | 0.0 ± 0.00% | 2.4 ± 1.2 (18) | 0.0 ± 0.0 (19) | 44.2 ± 2.86 (20) | 182.2 ± 14.18 (16) |
| **VIN LOAEL^α^** | 2.93 ± 0.192 (18) 95% | 1.54 ± 0.110 (18) 100% | 0.41 ± 0.029 (18) 100% | 0.23 ± 0.022 (18) 100% | **92.5**** ± 23.77% | 0.0 ± 0.00% | **3.8**** ± 0.8 (16) | 0.0 ± 0.0 (19) | 45.9 ± 3.02 (20) | 191.2 ± 19.29 (17) |
| **FLT ADI^γ^** | 3.06 ± 0.214 (18) 101% | 1.46 ± 0.165 (18) 102% | 0.42 ± 0.040 (18) 98% | 0.21 ± 0.020 (18) 100% | 54.4 ± 36.00% | 0.0 ± 0.00% | 1.4 ± 1.1 (18) | 0.0 ± 0.0 (18) | 45.7 ± 3.00 (20) | 189.8 ± 14.23 (16) |
| **FLT NOAEL^β^** | 3.00 ± 0.194 (19) 99% | 1.51 ± 0.090 (19) 99% | 0.43 ± 0.044 (19) 98% | 0.22 ± 0.026 (19) 92% | 80.6 ± 22.89% | 0.0 ± 0.00% | 2.1 ± 0.8 (19) | 0.0 ± 0.0 (19) | 45.0 ± 2.32 (20) | 185.1 ± 14.82 (16) |
| **FLT LOAEL^α^** | 2.97 ± 0.204 (19) 97% | 1.56 ± 0.155 (20) 102% | 0.41 ± 0.029 (19) 95% | 0.23 ± 0.015 (20) 100% | **85.7*** ± 22.67% | 0.0 ± 0.00% | **2.9*** ± 1.5 (18) | 0.0 ± 0.0 (19) | 45.5 ± 2.33 (20) | 185.5 ± 18.18 (18) |
| **PRO ADI^α^** | 3.04 ± 0.225 (20) 99% | 1.55 ± 0.154 (20) 101% | 0.43 ± 0.025 (20) 100% | 0.23 ± 0.020 (20) 100% | 63.9 ± 27.10% | 0.0 ± 0.00% | 1.6 ± 1.1 (20) | 0.0 ± 0.0 (19) | 43.5 ± 2.65 (20) | 175.1 ± 15.99 (16) |
| **PRO NOAEL^α^** | 3.02 ± 0.173 (19) 98% | 1.52 ± 0.197 (19) 100% | 0.43 ± 0.026 (19) 100% | 0.23 ± 0.031 (19) 100% | **88.3**** ± 16.77% | 0.0 ± 0.00% | **2.5*** ± 1.1 (19) | 0.0 ± 0.0 (19) | 44.5 ± 3.72 (20) | 170.6 ± 10.00 (15) |
| **PRO LOAEL^α^** | 3.13 ± 0.228 (15) 102% | **1.69*** ± 0.124 (16) 110% | 0.44 ± 0.067 (15) 100% | **0.25*** ± 0.039 (16) 109% | **91.8**** ± 11.12% | 0.0 ± 0.00% | **2.8*** ± 1.3 (14) | 0.0 ± 0.0 (19) | 46.3 ± 3.83 (20) | 180.6 ± 14.51 (15) |
| **ADI MIX^γ^** | 2.97 ± 0.164 (18) 98% | 1.38 ± 0.118 (20) 97% | 0.42 ± 0.041 (18) 98% | 0.21 ± 0.019 (20) 100% | 66.3 ± 24.59% | 0.0 ± 0.00% | 1.4 ± 0.8 (18) | 0.0 ± 0.0 (18) | 45.3 ± 2.47 (20) | 185.2 ± 13.04 (15) |
| **NOAEL MIX^γ^** | 3.06 ± 0.181 (20) 101% | 1.47 ± 0.121 (20) 103% | 0.43 ± 0.055 (20) 100% | 0.22 ± 0.029 (20) 105% | 66.9 ± 27.38% | 0.0 ± 0.00% | 1.6 ± 0.8 (20) | 0.0 ± 0.0 (20) | 46.8 ± 2.95 (20) | 189.6 ± 17.22 (17) |
| **LOAEL MIX^γ^** | 2.94 ± 0.232 (17) 97% | **1.64**** ± 0.140 (17) 115% | **0.39**** ± 0.027 (17) 91% | 0.23 ± 0.029 (17) 110% | **100.0**** ± 0.00% | **51.5**** ± 30.98% | **6.4**** ± 1.1 (17) | **1.0**** ± 0.7 (17) | **54.3**** ± 3.45 (20) | **220.3**** ± 19.74 (17) |

Data are presented as mean ± SD (N) % mean; *: p ≤ 0.05, **: p ≤ 0.01; ^α^ indicates that statistical comparison was performed against concurrent control group α, ^β^ indicates that statistical comparison was performed against concurrent control group β, ^γ^ indicates that statistical comparison was performed against concurrent control group γ.

Supplementary Table 4: Absolute organ weights of parental females after single-substance and mixed exposures to anti-androgens

| **Absolute Weights** | **Terminal Body Weight** | **Adrenal Glands** | **Brain** | **Kidneys** | **Liver** | **Ovaries** | **Pituitary Gland** | **Spleen** | **Thyroid Gland** | **Uterus** |
| --- | --- | --- | --- | --- | --- | --- | --- | --- | --- | --- |
| **Positive Control^α^** | 98% | 94% | 102% | 100% | 102% | 100% | 100% | 106% | 101% | 82% |
| **VIN ADI^α^** | 98% | 99% | 102% | 100% | 96% | 103% | 100% | 103% | 101% | 79% |
| **VIN NOAEL^α^** | 98% | 109% | 100% | 100% | 98% | 104% | 101% | 104% | 99% | 75% |
| **VIN LOAEL^α^** | 98% | 107% | 102% | 104% | 101% | 107% | 102% | 106% | 100% | 74% |
| **FLT ADI^γ^** | 103% | 107% | 102% | 104%* | 99% | 98% | 97% | 105% | 104% | 96% |
| **FLT NOAEL^β^** | 102% | 97% | 100% | 103% | 99% | 98% | 89% | 105% | 96% | 105% |
| **FLT LOAEL^α^** | 97% | 102% | 102% | 99% | 98% | 108% | 104% | 109% | 106% | 73% |
| **PRO ADI^α^** | 98% | 101% | 100% | 99% | 100% | 107% | 100% | 102% | 104% | 74% |
| **PRO NOAEL^α^** | 99% | 99% | 100% | 100% | 101% | 106% | 97% | 103% | 91% | 84% |
| **PRO LOAEL^α^** | 98% | 96% | 101% | 100% | 106% | 108% | 94% | 99% | 96% | 84% |
| **ADI MIX^γ^** | 102% | 102% | 102% | 102% | 99% | 106% | 114% | 107% | 104% | 98% |
| **NOAEL MIX^γ^** | 100% | 106% | 101% | 101% | 99% | 108% | 108% | 103% | 104% | 89% |
| **LOAEL MIX^γ^** | 102% | 117%** | 101% | 108%** | 122%** | 114% | 101% | 107% | 111% | 96% |

Data are absolute organ weights presented as a percentage of the corresponding concurrent control group, *: p ≤ 0.05, **: p ≤ 0.01

Supplementary Table 5: Relative organ weights of parental females after single-substance and mixed exposures to anti-androgens

| **Relative Weights** | **Adrenal Glands** | **Brain** | **Kidneys** | **Liver** | **Ovaries** | **Pituitary Gland** | **Spleen** | **Thyroid Gland** | **Uterus** |
| --- | --- | --- | --- | --- | --- | --- | --- | --- | --- |
| **Positive Control^α^** | 96% | 104%** | 102% | 105% | 103% | 102% | 109% | 103% | 84% |
| **VIN ADI^α^** | 100% | 104%** | 101% | 97% | 105% | 102% | 105% | 102% | 80% |
| **VIN NOAEL^α^** | 111%* | 103% | 102% | 100% | 107% | 103% | 106% | 101% | 77% |
| **VIN LOAEL^α^** | 109% | 104%* | 106% | 103% | 109% | 104% | 108% | 102% | 76% |
| **FLT ADI^γ^** | 105% | 99% | 102% | 96%* | 96% | 94% | 103% | 101% | 92% |
| **FLT NOAEL^β^** | 96% | 98% | 101% | 97% | 96% | 88% | 103% | 94% | 104% |
| **FLT LOAEL^α^** | 105% | 105%** | 102% | 100% | 112%* | 107% | 112%** | 108% | 75% |
| **PRO ADI^α^** | 102% | 101% | 101% | 102% | 109% | 101% | 104% | 104% | 75% |
| **PRO NOAEL^α^** | 100% | 101% | 101% | 101% | 107% | 98% | 103% | 92% | 84% |
| **PRO LOAEL^α^** | 97% | 103% | 101% | 108%** | 110% | 95% | 101% | 97% | 85% |
| **ADI MIX^γ^** | 99% | 100% | 99% | 96% | 104% | 111% | 105% | 102% | 95% |
| **NOAEL MIX^γ^** | 106% | 101% | 101% | 98% | 107% | 108% | 103% | 104% | 89% |
| **LOAEL MIX^γ^** | 115%** | 100% | 106%** | 119%** | 113% | 100% | 106% | 109% | 94% |

Data are organ weights relative to terminal body weights presented as a percentage of the corresponding concurrent control group, *: p ≤ 0.05, **: p ≤ 0.01

Supplementary Table 6: Absolute organ weights of Subset 1 (weaning) male offspring after single-substance and mixed exposures to anti-androgens

| **Absolute Weights** | **Terminal Body Weight** | **Adrenal Glands** | **Brain** | **Kidneys** | **Liver** | **Pituitary Gland** | **Spleen** | **Thyroid Gland** |
| --- | --- | --- | --- | --- | --- | --- | --- | --- |
| **Positive Control^α^** | 95% | 85% | 100% | 102% | 91%** | 100% | 87% | 109% |
| **VIN ADI^α^** | 99% | 87% | 102% | 110% | 99% | 97% | 98% | 109% |
| **VIN NOAEL^α^** | 101% | 104% | 102% | 109% | 99% | 106% | 97% | 111% |
| **VIN LOAEL^α^** | 97% | 84% | 100% | 101% | 96% | 99% | 80% | 114% |
| **FLT ADI^γ^** | 105% | 101% | 103% | 106% | 107% | 113% | 104% | 143% |
| **FLT NOAEL^β^** | 106% | 89% | 103% | 104% | 109% | 103% | 114% | 107% |
| **FLT LOAEL^α^** | 96% | 92% | 101% | 105% | 93%* | 97% | 88% | 103% |
| **PRO ADI^α^** | 100% | 91% | 103% | 113% | 98% | 102% | 99% | 129% |
| **PRO NOAEL^α^** | 98% | 88% | 100% | 105% | 94% | 102% | 84% | 113% |
| **PRO LOAEL^α^** | 105% | 105% | 101% | 112% | 104% | 96% | 98% | 101% |
| **ADI MIX^γ^** | 127% | 96% | 101% | 92% | 102% | 95% | 101% | 110% |
| **NOAEL MIX^γ^** | 99% | 109% | 102% | 100% | 98% | 68%* | 94% | 127% |
| **LOAEL MIX^γ^** | 106% | 105% | 103% | 106% | 108% | 149%* | 103% | 154% |

Data are presented as percentages of control values, *: p ≤ 0.05, **: p ≤ 0.01

Supplementary Table 7: Absolute sex organ weights of Subset 1 (weaning) male offspring after single-substance and mixed exposures to anti-androgens

| **Absolute Weights** | **Cauda Epididymis** | **Epididymides** | **Musc. Bulb. % L. ani.** | **Prostate** | **Prostate Ventr. Fresh** | **Seminal Vesicle** | **Testes** |
| --- | --- | --- | --- | --- | --- | --- | --- |
| **Positive Control^α^** | 72%* | 82%* | 73%** | 79%** | 81%** | 83% | 104% |
| **VIN ADI^α^** | 102% | 99% | 87% | 102% | 105% | 99% | 101% |
| **VIN NOAEL^α^** | 104% | 104% | 94% | 106% | 110% | 110% | 106% |
| **VIN LOAEL^α^** | 85% | 99% | 77% | 92% | 89% | 81%* | 104% |
| **FLT ADI^γ^** | 104% | 104% | 104% | 106% | 107% | 103% | 108% |
| **FLT NOAEL^β^** | 113% | 113% | 96% | 97% | 95% | 112% | 107% |
| **FLT LOAEL^α^** | 91% | 101% | 93% | 102% | 101% | 105% | 105% |
| **PRO ADI^α^** | 87% | 100% | 96% | 104% | 109% | 93% | 107% |
| **PRO NOAEL^α^** | 85% | 100% | 98% | 102% | 106% | 97% | 108% |
| **PRO LOAEL^α^** | 95% | 103% | 96% | 102% | 100% | 91% | 113% |
| **ADI MIX^γ^** | 127% | 109% | 92% | 97% | 100% | 106% | 103% |
| **NOAEL MIX^γ^** | 94% | 101% | 95% | 93% | 98% | 99% | 98% |
| **LOAEL MIX^γ^** | 92% | 92% | 100% | 87% | 79% | 88% | 108% |

Data are presented as percentages of control values, *: p ≤ 0.05, **: p ≤ 0.01

Supplementary Table 8: Relative organ weights of Subset 1 (weaning) male offspring after single-substance and mixed exposures to anti-androgens

| **Relative Weights** | **Adrenal Glands** | **Brain** | **Kidneys** | **Liver** | **Pituitary Gland** | **Spleen** | **Thyroid Gland** |
| --- | --- | --- | --- | --- | --- | --- | --- |
| **Positive Control^α^** | 90% | 106% | 108% | 96% | 107% | 92% | 116% |
| **VIN ADI^α^** | 88% | 103% | 110% | 99% | 98% | 98% | 110% |
| **VIN NOAEL^α^** | 104% | 102% | 108% | 97% | 105% | 96% | 112% |
| **VIN LOAEL^α^** | 86% | 104% | 105% | 99% | 103% | 83% | 119% |
| **FLT ADI^γ^** | 97% | 99% | 101% | 103% | 107% | 100% | 137% |
| **FLT NOAEL^β^** | 83% | 99% | 99% | 103% | 98% | 106% | 102% |
| **FLT LOAEL^α^** | 96% | 105% | 110% | 96% | 101% | 92% | 107% |
| **PRO ADI^α^** | 90% | 103% | 113% | 98% | 102% | 98% | 130% |
| **PRO NOAEL^α^** | 90% | 103% | 108% | 96% | 105% | 85% | 117% |
| **PRO LOAEL^α^** | 100% | 96% | 107% | 100% | 92% | 94% | 97% |
| **ADI MIX^γ^** | 96% | 102% | 93% | 102% | 94% | 100% | 109% |
| **NOAEL MIX^γ^** | 110% | 103% | 101% | 99% | 68% | 94% | 126% |
| **LOAEL MIX^γ^** | 100% | 97% | 100% | 102% | 138% | 97% | 146% |

Data are presented as percentages relative to control values, *: p ≤ 0.05, **: p ≤ 0.01

Supplementary Table 9: Relative sex organ weights of Subset 1 (weaning) male offspring after single-substance and mixed exposures to anti-androgens

| **Relative Weights** | **Cauda Epididymis** | **Epididymides** | **Musc. Bulb. % L. ani.** | **Prostate** | **Prostate Ventr. Fresh** | **Seminal Vesicle** | **Testes** |
| --- | --- | --- | --- | --- | --- | --- | --- |
| **Positive Control^α^** | 77%* | 87%* | 78%** | 84%* | 86%* | 88% | 110% |
| **VIN ADI^α^** | 102% | 100% | 88% | 103% | 105% | 100% | 102% |
| **VIN NOAEL^α^** | 103% | 103% | 93% | 106% | 110% | 110% | 105% |
| **VIN LOAEL^α^** | 88% | 103% | 80% | 96% | 93% | 85%* | 108% |
| **FLT ADI^γ^** | 98% | 99% | 99% | 102% | 103% | 97% | 104% |
| **FLT NOAEL^β^** | 107% | 107% | 92% | 92% | 89% | 106% | 101% |
| **FLT LOAEL^α^** | 96% | 105% | 97% | 106% | 105% | 109% | 110% |
| **PRO ADI^α^** | 87% | 100% | 96% | 104% | 110% | 93% | 107% |
| **PRO NOAEL^α^** | 88% | 103% | 100% | 106% | 109% | 100% | 111% |
| **PRO LOAEL^α^** | 91% | 99% | 93% | 98% | 96% | 88% | 108% |
| **ADI MIX^γ^** | 126% | 108% | 91% | 96% | 100% | 106% | 103% |
| **NOAEL MIX^γ^** | 92% | 101% | 95% | 93% | 98% | 101% | 98% |
| **LOAEL MIX^γ^** | 85% | 86% | 93% | 82% | 74%** | 83% | 102% |

Data are presented as percentages relative to control values, *: p ≤ 0.05, **: p ≤ 0.01

Supplementary Table 10: Absolute organ weights of Subset 1 (weaning) female offspring after single-substance and mixed exposures to anti-androgens

| **Absolute Weights** | **Terminal Body Weight** | **Adrenal Glands** | **Brain** | **Kidneys** | **Liver** | **Ovaries** | **Pituitary Gland** | **Spleen** | **Thyroid Gland** | **Uterus** |
| --- | --- | --- | --- | --- | --- | --- | --- | --- | --- | --- |
| **Positive Control^α^** | 103% | 99% | 99% | 109% | 107% | 122% | 112% | 121% | 103% | 121% |
| **VIN ADI^α^** | 96% | 91% | 99% | 98% | 97% | 104% | 99% | 102% | 105% | 106% |
| **VIN NOAEL^α^** | 100% | 108% | 100% | 106% | 101% | 127% | 107% | 114% | 100% | 118% |
| **VIN LOAEL^α^** | 100% | 97% | 98% | 103% | 104% | 127% | 106% | 111% | 114% | 111% |
| **FLT ADI^γ^** | 104% | 98% | 103%* | 108% | 107% | 112% | 128% | 110% | 120% | 108% |
| **FLT NOAEL^β^** | 101% | 77% | 101% | 98% | 102% | 75% | 97% | 94% | 101% | 92% |
| **FLT LOAEL^α^** | 99% | 94% | 98% | 102% | 101% | 120% | 103% | 108% | 106% | 115% |
| **PRO ADI^α^** | 97% | 100% | 99% | 103% | 99% | 124% | 106% | 108% | 100% | 111% |
| **PRO NOAEL^α^** | 96% | 115% | 98% | 101% | 96% | 114% | 99% | 99% | 98% | 112% |
| **PRO LOAEL^α^** | 101% | 97% | 9% | 105% | 107% | 108% | 98% | 129% | 89% | 116% |
| **ADI MIX^γ^** | 103% | 84% | 99% | 105% | 104% | 97% | 116% | 98% | 87% | 110% |
| **NOAEL MIX^γ^** | 100% | 83% | 99% | 103% | 103% | 96% | 98% | 97% | 104% | 111% |
| **LOAEL MIX^γ^** | 108% | 108% | 102% | 109% | 112% | 114% | 117% | 117% | 144%* | 121%** |

Data are presented as percentages of control values, *: p ≤ 0.05, **: p ≤ 0.01

Supplementary Table 11: Relative organ weights of Subset 1 (weaning) female offspring after single-substance and mixed exposures to anti-androgens

| **Relative Weights** | **Adrenal Glands** | **Brain** | **Kidneys** | **Liver** | **Ovaries** | **Pituitary Gland** | **Spleen** | **Thyroid Gland** | **Uterus** |
| --- | --- | --- | --- | --- | --- | --- | --- | --- | --- |
| **Positive Control^α^** | 96% | 96% | 106% | 104% | 119% | 109% | 117% | 101% | 116% |
| **VIN ADI^α^** | 94% | 104% | 102% | 102% | 108% | 104% | 107% | 109% | 110% |
| **VIN NOAEL^α^** | 110% | 100% | 106% | 101% | 128% | 107% | 114% | 100% | 118%** |
| **VIN LOAEL^α^** | 100% | 99% | 103% | 104% | 130% | 106% | 111% | 116% | 113% |
| **FLT ADI^γ^** | 94% | 100% | 104% | 103% | 107% | 122% | 104% | 116% | 104% |
| **FLT NOAEL^β^** | 77% | 100% | 97% | 101% | 75% | 96% | 93% | 100% | 92% |
| **FLT LOAEL^α^** | 95% | 100% | 103% | 103% | 122% | 104% | 109% | 109% | 117% |
| **PRO ADI^α^** | 103% | 102% | 105% | 101% | 127% | 110% | 111% | 104% | 114% |
| **PRO NOAEL^α^** | 120% | 103% | 105% | 99% | 118% | 102% | 103% | 103% | 117% |
| **PRO LOAEL^α^** | 97% | 97% | 104% | 106%** | 107% | 98% | 130% | 89% | 115% |
| **ADI MIX^γ^** | 83%* | 98% | 102% | 101% | 96% | 110% | 95% | 85% | 108% |
| **NOAEL MIX^γ^** | 82% | 99% | 102% | 103% | 96% | 97% | 97% | 102% | 111% |
| **LOAEL MIX^γ^** | 100% | 95% | 101% | 104% | 106% | 107% | 108% | 133% | 113% |

Data are presented as percentages relative to control group 0, *: p ≤ 0.05, **: p ≤ 0.01

Supplementary Table 12: Absolute organ weights of Subset 2 (puberty) male offspring after single-substance and mixed exposures to anti-androgens

| **Absolute Weights** | **Terminal Body Weight** | **Adrenal Glands** | **Brain** | **Kidneys** | **Liver** | **Pituitary Gland** | **Spleen** | **Thyroid Gland** |
| --- | --- | --- | --- | --- | --- | --- | --- | --- |
| **Positive Control^α^** | 124%** | 117% | 103% | 112%** | 116%** | 121%** | 119% | 107% |
| **VIN ADI^α^** | 100% | 84% | 100% | 96% | 97% | 100% | 110% | 95% |
| **VIN NOAEL^α^** | 99% | 89% | 100% | 95% | 97% | 98% | 104% | 95% |
| **VIN LOAEL^α^** | 105% | 98% | 101% | 105% | 104% | 111%* | 112% | 97% |
| **FLT ADI^γ^** | 102% | 104% | 102% | 106% | 105% | 101% | 102% | 116%* |
| **FLT NOAEL^β^** | 107%* | 97% | 102% | 107% | 115% | 108% | 112% | 109% |
| **FLT LOAEL^α^** | 96% | 86% | 100% | 93% | 95% | 103% | 110% | 109% |
| **PRO ADI^α^** | 93% | 89% | 96% | 92% | 90% | 94% | 102% | 104% |
| **PRO NOAEL^α^** | 94% | 86% | 98% | 93% | 91% | 103% | 105% | 88% |
| **PRO LOAEL^α^** | 98% | 92% | 99% | 94% | 101% | 97% | 104% | 94% |
| **ADI MIX^γ^** | 101% | 98% | 101% | 100% | 102% | 102% | 100% | 109% |
| **NOAEL MIX^γ^** | 101% | 100% | 99% | 99% | 102% | 99% | 99% | 103% |
| **LOAEL MIX^γ^** | 118%** | 120%* | 101% | 113%* | 118%* | 102% | 108% | 129%** |

Data are presented as percentages relative to control group 0, *: p ≤ 0.05, **: p ≤ 0.01

Supplementary Table 13: Absolute sex organ weights of Subset 2 (puberty) male offspring after single-substance and mixed exposures to anti-androgens

| **Absolute Weights** | **Bulbo-Urethral Gland** | **Cauda Epididymis** | **Epididymides** | **Glans Penis** | **Musc. Bulb. % L. ani.** | **Prostate** | **Prostate Ventr. Fresh** | **Seminal Vesicle** | **Testes** |
| --- | --- | --- | --- | --- | --- | --- | --- | --- | --- |
| **Positive Control^α^** | 114% | 142% | 170% | 107% | 104% | 100% | 98% | 152% | 154% |
| **VIN ADI^α^** | 122% | 100% | 102% | 114% | 101% | 102% | 98% | 100% | 104% |
| **VIN NOAEL^α^** | 129% | 95% | 97% | 110% | 102% | 98% | 102% | 97% | 107% |
| **VIN LOAEL^α^** | 118% | 99% | 109% | 91% | 100% | 95% | 90% | 100% | 115% |
| **FLT ADI^γ^** | 101% | 102% | 103% | 106% | 111% | 119% | 113% | 101% | 96% |
| **FLT NOAEL^β^** | 100% | 100% | 104% | 104% | 106% | 88% | 88% | 83% | 105% |
| **FLT LOAEL^α^** | 90% | 86%* | 96% | 91% | 96% | 88% | 84% | 89% | 104% |
| **PRO ADI^α^** | 106% | 96% | 95% | 94% | 89% | 94% | 94% | 96% | 97% |
| **PRO NOAEL^α^** | 99% | 92% | 94% | 95% | 93% | 93% | 91% | 83% | 99% |
| **PRO LOAEL^α^** | 104% | 104% | 104% | 97% | 91% | 91% | 87% | 88% | 106% |
| **ADI MIX^γ^** | 111% | 103% | 103% | 106% | 109% | 109% | 107% | 107% | 94% |
| **NOAEL MIX^γ^** | 101% | 100% | 103% | 107% | 99% | 99% | 91% | 106% | 98% |
| **LOAEL MIX^γ^** | 82% | 139% | 159% | 85%* | 102% | 103% | 95% | 125% | 122% |

Data are presented as percentages relative to control group 0, *: p ≤ 0.05, **: p ≤ 0.01

Supplementary Table 14: Relative organ weights of Subset 2 (puberty) male offspring after single-substance and mixed exposures to anti-androgens

| **Relative Weights** | **Adrenal Glands** | **Brain** | **Kidneys** | **Liver** | **Pituitary Gland** | **Spleen** | **Thyroid Gland** |
| --- | --- | --- | --- | --- | --- | --- | --- |
| **Positive Control^α^** | 96% | 84%** | 92% | 95% | 99% | 99% | 88% |
| **VIN ADI^α^** | 84% | 100% | 95% | 97% | 100% | 112% | 95% |
| **VIN NOAEL^α^** | 90% | 101% | 96% | 98% | 99% | 106% | 96% |
| **VIN LOAEL^α^** | 93% | 96% | 100% | 99% | 106% | 106% | 93% |
| **FLT ADI^γ^** | 103% | 99% | 103% | 103% | 99% | 100% | 112% |
| **FLT NOAEL^β^** | 91% | 95%* | 99% | 107% | 100% | 104% | 102% |
| **FLT LOAEL^α^** | 89% | 104% | 96% | 99% | 106% | 114% | 113%* |
| **PRO ADI^α^** | 97% | 104% | 99% | 97% | 101% | 110% | 113% |
| **PRO NOAEL^α^** | 92% | 103% | 99% | 97% | 109% | 111% | 94% |
| **PRO LOAEL^α^** | 94% | 101% | 97% | 104% | 100% | 107% | 97% |
| **ADI MIX^γ^** | 98% | 99% | 99% | 102% | 101% | 99% | 108% |
| **NOAEL MIX^γ^** | 100% | 98% | 98% | 101% | 98% | 98% | 101% |
| **LOAEL MIX^γ^** | 102% | 86%** | 96% | 100% | 86%* | 92% | 109% |

Data are presented as percentages relative to control group 0, *: p ≤ 0.05, **: p ≤ 0.01

Supplementary Table 15: Relative sex organ weights of Subset 2 (puberty) male offspring after single-substance and mixed exposures to anti-androgens

| **Relative Weights** | **Bulbo-Urethral Gland** | **Cauda Epididymis** | **Epididymides** | **Glans Penis** | **Musc. Bulb. % L. ani.** | **Prostate** | **Prostate Ventr. Fresh** | **Seminal Vesicle** | **Testes** |
| --- | --- | --- | --- | --- | --- | --- | --- | --- | --- |
| **Positive Control^α^** | 94% | 112% | 135% | 87% | 86% | 82%** | 80%** | 120% | 125% |
| **VIN ADI^α^** | 121% | 100% | 101% | 114% | 99% | 101% | 98% | 98% | 103% |
| **VIN NOAEL^α^** | 128% | 96% | 98% | 111% | 102% | 99% | 103% | 98% | 108% |
| **VIN LOAEL^α^** | 113% | 94% | 103% | 86% | 94% | 90% | 85%** | 94% | 109% |
| **FLT ADI^γ^** | 98% | 100% | 100% | 103% | 108% | 116% | 111% | 98% | 94% |
| **FLT NOAEL^β^** | 95% | 94% | 98% | 98% | 99% | 83%** | 83%* | 79% | 98% |
| **FLT LOAEL^α^** | 94% | 89% | 100% | 95% | 99% | 91%* | 88%* | 93% | 108% |
| **PRO ADI^α^** | 116% | 104% | 103% | 103% | 96% | 101% | 101% | 103% | 105% |
| **PRO NOAEL^α^** | 105% | 98% | 99% | 100% | 99% | 98% | 96% | 88% | 104% |
| **PRO LOAEL^α^** | 106% | 106% | 105% | 99% | 91% | 93% | 90% | 89% | 108% |
| **ADI MIX^γ^** | 110% | 103% | 102% | 105% | 108% | 109% | 107% | 107% | 93% |
| **NOAEL MIX^γ^** | 100% | 100% | 103% | 105% | 98% | 99% | 92% | 106% | 98% |
| **LOAEL MIX^γ^** | 68%** | 118% | 135% | 73%** | 86%** | 87% | 81%* | 105% | 103% |

Data are presented as percentages relative to control group 0, *: p ≤ 0.05, **: p ≤ 0.01

Supplementary Table 16: Absolute organ weights of Subset 2 (puberty) female offspring after single-substance and mixed exposures to anti-androgens

| **Absolute Weights** | **Terminal Body Weight** | **Adrenal Glands** | **Brain** | **Kidneys** | **Liver** | **Ovaries** | **Pituitary Gland** | **Spleen** | **Thyroid Gland** | **Uterus** |
| --- | --- | --- | --- | --- | --- | --- | --- | --- | --- | --- |
| **Positive Control^α^** | 88% | 90% | 100% | 91% | 89% | 94% | 96% | 102% | 85% | 98% |
| **VIN ADI^α^** | 91% | 111% | 101% | 96% | 92% | 87% | 99% | 95% | 93% | 109% |
| **VIN NOAEL^α^** | 99% | 104% | 101% | 104% | 101% | 89% | 110% | 101% | 92% | 142% |
| **VIN LOAEL^α^** | 90% | 102% | 99% | 94% | 91% | 91% | 97% | 95% | 98% | 106% |
| **FLT ADI^γ^** | 103% | 91% | 103% | 99% | 98% | 86% | 96% | 102% | 106% | 98% |
| **FLT NOAEL^β^** | 106% | 106% | 101% | 106% | 108% | 75%** | 98% | 97% | 116% | 114%* |
| **FLT LOAEL^α^** | 94% | 105% | 100% | 99% | 95% | 108% | 103% | 91% | 106% | 109% |
| **PRO ADI^α^** | 91% | 102% | 99% | 100% | 93% | 108% | 101% | 99% | 110% | 94% |
| **PRO NOAEL^α^** | 91% | 98% | 99% | 97% | 95% | 103% | 105% | 96% | 96% | 130% |
| **PRO LOAEL^α^** | 93% | 111% | 99% | 96% | 92% | 108% | 91% | 86% | 91% | 110% |
| **ADI MIX^γ^** | 97% | 90% | 102% | 103% | 99% | 101% | 105% | 103% | 90% | 108% |
| **NOAEL MIX^γ^** | 97% | 95% | 103% | 99% | 96% | 86% | 86% | 101% | 114% | 106% |
| **LOAEL MIX^γ^** | 99% | 98% | 103% | 99% | 104% | 81% | 72%** | 106% | 122% | 109% |

Data are presented as percentages relative to control group 0, *: p ≤ 0.05, **: p ≤ 0.01

Supplementary Table 17: Relative organ weights of Subset 2 (puberty) female offspring after single-substance and mixed exposures to anti-androgens

| **Relative Weights** | **Adrenal Glands** | **Brain** | **Kidneys** | **Liver** | **Ovaries** | **Pituitary Gland** | **Spleen** | **Thyroid Gland** | **Uterus** |
| --- | --- | --- | --- | --- | --- | --- | --- | --- | --- |
| **Positive Control^α^** | 104% | 114% | 103% | 102% | 107% | 109% | 117% | 97% | 114% |
| **VIN ADI^α^** | 123% | 111% | 105% | 101% | 97% | 108% | 105% | 102% | 117% |
| **VIN NOAEL^α^** | 105% | 103% | 105% | 102% | 91% | 111% | 103% | 93% | 141% |
| **VIN LOAEL^α^** | 113% | 112% | 104% | 101% | 100% | 109% | 108% | 107% | 116% |
| **FLT ADI^γ^** | 89% | 100% | 97% | 95% | 85% | 94% | 99% | 102% | 95% |
| **FLT NOAEL^β^** | 100% | 96% | 100% | 102% | 72%** | 93% | 93% | 110% | 109% |
| **FLT LOAEL^α^** | 112% | 106% | 105% | 101% | 115% | 109% | 99% | 113% | 116% |
| **PRO ADI^α^** | 113% | 109% | 110%* | 102% | 117% | 109% | 110% | 124% | 102% |
| **PRO NOAEL^α^** | 108% | 108% | 106%* | 104% | 113% | 115% | 105% | 105% | 141% |
| **PRO LOAEL^α^** | 119% | 106% | 102% | 99% | 115% | 97% | 92% | 97% | 116% |
| **ADI MIX^γ^** | 93% | 105% | 106% | 103% | 106% | 108% | 106% | 94% | 108% |
| **NOAEL MIX^γ^** | 99% | 106% | 102% | 99% | 88% | 88% | 103% | 118% | 109% |
| **LOAEL MIX^γ^** | 100% | 104% | 100% | 105% | 82% | 73%** | 106% | 122%* | 108% |

Data are presented as percentages relative to control group 0, *: p ≤ 0.05, **: p ≤ 0.01

Supplementary Table 18: Absolute organ weights of Subset 3 (PND 83±2) male offspring after single-substance and mixed exposures to anti-androgens

| **Absolute Weights** | **Terminal Body Weight** | **Adrenal Glands** | **Brain** | **Kidneys** | **Liver** | **Pituitary Gland** | **Spleen** | **Thyroid Gland** |
| --- | --- | --- | --- | --- | --- | --- | --- | --- |
| **Positive Control^α^** | 94% | 97% | 100% | 85%** | 88% | 109% | 96% | 99% |
| **VIN ADI^α^** | 92% | 103% | 101% | 94% | 89% | 98% | 92% | 89% |
| **VIN NOAEL^α^** | 96% | 99% | 98% | 93% | 94% | 96% | 93% | 90% |
| **VIN LOAEL^α^** | 93% | 108% | 98% | 92% | 90% | 100% | 95% | 100% |
| **FLT ADI^γ^** | 102% | 104% | 102% | 102% | 97% | 96% | 102% | 104% |
| **FLT NOAEL^β^** | 101% | 98% | 99% | 102% | 104% | 97% | 98% | 95% |
| **FLT LOAEL^α^** | 99% | 108% | 101% | 97% | 95% | 108% | 98% | 101% |
| **PRO ADI^α^** | 94% | 92% | 100% | 90% | 90% | 104% | 100% | 94% |
| **PRO NOAEL^α^** | 89%** | 95% | 97%** | 86%** | 84% | 96% | 86% | 109% |
| **PRO LOAEL^α^** | 87%** | 90% | 96%* | 87%** | 88% | 86%** | 84%* | 104% |
| **ADI MIX^γ^** | 97% | 106% | 102% | 101% | 94% | 97% | 98% | 100% |
| **NOAEL MIX^γ^** | 99% | 95% | 100% | 105% | 98% | 94% | 101% | 99% |
| **LOAEL MIX^γ^** | 95% | 106% | 100% | 102% | 103% | 90% | 103% | 110% |

Data are presented as percentages relative to control group 0, *: p ≤ 0.05, **: p ≤ 0.01

Supplementary Table 19: Absolute sex organ weights of Subset 3 (PND 83±2) male offspring after single-substance and mixed exposures to anti-androgens

| **Absolute Weights** | **Bulbo-Urethral Gland** | **Cauda Epididymis** | **Epididymides** | **Glans Penis** | **Musc. Bulb. % L. ani.** | **Prostate** | **Prostate Ventr. Fresh** | **Seminal Vesicle** | **Testes** |
| --- | --- | --- | --- | --- | --- | --- | --- | --- | --- |
| **Positive Control^α^** | 43%** | 61%** | 74%** | 81%* | 49%** | 57%** | 57%** | 54%** | 93% |
| **VIN ADI^α^** | 95% | 104% | 100% | 113% | 99% | 102% | 104% | 97% | 101% |
| **VIN NOAEL^α^** | 97% | 99% | 99% | 100% | 92% | 103% | 103% | 97% | 100% |
| **VIN LOAEL^α^** | 79% | 86%** | 92%* | 97% | 77%** | 86%* | 81%** | 85% | 97% |
| **FLT ADI^γ^** | 96% | 102% | 102% | 95% | 95% | 94% | 94% | 87% | 99% |
| **FLT NOAEL^β^** | 93% | 99% | 100% | 98% | 104% | 91% | 92% | 94% | 101% |
| **FLT LOAEL^α^** | 78% | 91% | 98% | 95% | 92% | 101% | 99% | 103% | 102% |
| **PRO ADI^α^** | 94% | 105% | 105% | 106% | 100% | 105% | 100% | 104% | 104% |
| **PRO NOAEL^α^** | 93% | 91%* | 92% | 101% | 93% | 10% | 96% | 104% | 99% |
| **PRO LOAEL^α^** | 75% | 92%* | 94% | 92% | 82%** | 85% | 83% | 87% | 94% |
| **ADI MIX^γ^** | 93% | 96% | 100% | 100% | 96% | 95% | 93% | 94% | 100% |
| **NOAEL MIX^γ^** | 83% | 89%* | 102% | 97% | 90% | 98% | 99% | 87% | 97% |
| **LOAEL MIX^γ^** | 56%** | 85%** | 93% | 82%** | 63%** | 69%** | 69%** | 62%** | 102% |

Data are presented as percentages relative to control group 0, *: p ≤ 0.05, **: p ≤ 0.01

Supplementary Table 20: Relative organ weights of Subset 3 (PND 83±2) male offspring after single-substance and mixed exposures to anti-androgens

| **Relative Weights** | **Adrenal Glands** | **Brain** | **Kidneys** | **Liver** | **Pituitary Gland** | **Spleen** | **Thyroid Gland** |
| --- | --- | --- | --- | --- | --- | --- | --- |
| **Positive Control^α^** | 102% | 107% | 91% | 94% | 118% | 104% | 105% |
| **VIN ADI^α^** | 111% | 109% | 102% | 96% | 106% | 101% | 96% |
| **VIN NOAEL^α^** | 103% | 102% | 98% | 99% | 101% | 98% | 94% |
| **VIN LOAEL^α^** | 115% | 105% | 99% | 97% | 107% | 103% | 107% |
| **FLT ADI^γ^** | 102% | 100% | 100% | 95% | 94% | 99% | 101% |
| **FLT NOAEL^β^** | 96% | 98% | 101% | 104% | 97% | 98% | 95% |
| **FLT LOAEL^α^** | 108% | 102% | 98% | 97% | 109% | 100% | 102% |
| **PRO ADI^α^** | 97% | 106% | 96% | 96% | 110%* | 107% | 98% |
| **PRO NOAEL^α^** | 107% | 109%* | 97% | 94% | 107% | 97% | 121% |
| **PRO LOAEL^α^** | 104% | 110%* | 100% | 102% | 99% | 97% | 119%* |
| **ADI MIX^γ^** | 110% | 106% | 104% | 97% | 100% | 101% | 107% |
| **NOAEL MIX^γ^** | 95% | 101% | 107% | 99% | 95% | 101% | 99% |
| **LOAEL MIX^γ^** | 110% | 105% | 107% | 108% | 94% | 107% | 115% |

Data are presented as percentages relative to control group 0, *: p ≤ 0.05, **: p ≤ 0.01

Supplementary Table 21: Relative sex organ weights of Subset 3 (PND 83±2) male offspring after single-substance and mixed exposures to anti-androgens

| **Relative Weights** | **Bulbo-Urethral Gland** | **Cauda Epididymis** | **Epididymides** | **Glans Penis** | **Musc. Bulb. % L. ani.** | **Prostate** | **Prostate Ventr. Fresh** | **Seminal Vesicle** | **Testes** |
| --- | --- | --- | --- | --- | --- | --- | --- | --- | --- |
| **Positive Control^α^** | 48%** | 64%** | 78%** | 87%* | 52%** | 62%** | 61%** | 57%** | 97% |
| **VIN ADI^α^** | 104% | 113% | 108% | 122% | 108% | 112% | 113% | 106% | 109% |
| **VIN NOAEL^α^** | 102% | 104% | 104% | 105% | 96% | 108% | 108% | 102% | 105% |
| **VIN LOAEL^α^** | 85% | 92% | 99% | 104% | 82%** | 93% | 87%* | 92% | 104% |
| **FLT ADI^γ^** | 94% | 99% | 100% | 93% | 92% | 92%* | 92% | 85%* | 97% |
| **FLT NOAEL^β^** | 93% | 97% | 99% | 96% | 103% | 90% | 90% | 93% | 101% |
| **FLT LOAEL^α^** | 79% | 93% | 99% | 96% | 93% | 102% | 100% | 104% | 103% |
| **PRO ADI^α^** | 100% | 111% | 112% | 113% | 105% | 111% | 106% | 111% | 110% |
| **PRO NOAEL^α^** | 104% | 102% | 102% | 114% | 105% | 113% | 108% | 117% | 111% |
| **PRO LOAEL^α^** | 86% | 107% | 109% | 105% | 94% | 98% | 96% | 101% | 109% |
| **ADI MIX^γ^** | 97% | 99% | 103% | 102% | 98% | 97% | 95% | 97% | 103% |
| **NOAEL MIX^γ^** | 84%* | 90% | 104% | 98% | 90% | 98% | 99% | 88% | 98% |
| **LOAEL MIX^γ^** | 59%** | 90% | 98% | 86% | 66%** | 72%** | 72%** | 65%** | 107% |

Data are presented as percentages relative to control group 0, *: p ≤ 0.05, **: p ≤ 0.01

Supplementary Table 22: Absolute organ weights of Subset 3 (PND 83±2) female offspring after single-substance and mixed exposures to anti-androgens

| **Absolute Weights** | **Terminal Body Weight** | **Adrenal Glands** | **Brain** | **Kidneys** | **Liver** | **Ovaries** | **Pituitary Gland** | **Spleen** | **Thyroid Gland** | **Uterus** |
| --- | --- | --- | --- | --- | --- | --- | --- | --- | --- | --- |
| **Positive Control^α^** | 97% | 92% | 100% | 99% | 97% | 107% | 113% | 109% | 98% | 88% |
| **VIN ADI^α^** | 100% | 103% | 101% | 103% | 101% | 111% | 105% | 115% | 111% | 110% |
| **VIN NOAEL^α^** | 99% | 102% | 101% | 101% | 102% | 106% | 107% | 108% | 102% | 83% |
| **VIN LOAEL^α^** | 97% | 98% | 101% | 100% | 97% | 106% | 104% | 106% | 99% | 99% |
| **FLT ADI^γ^** | 104% | 99% | 103% | 104% | 103% | 103% | 108% | 102% | 105% | 125% |
| **FLT NOAEL^β^** | 103% | 103% | 99% | 110% | 105% | 110% | 99% | 104% | 107% | 108% |
| **FLT LOAEL^α^** | 97% | 93% | 100% | 101% | 95% | 102% | 114% | 104% | 102% | 96% |
| **PRO ADI^α^** | 98% | 98% | 102% | 102% | 101% | 111% | 115%* | 102% | 104% | 91% |
| **PRO NOAEL^α^** | 96% | 93% | 99% | 99% | 97% | 107% | 114% | 103% | 95% | 79% |
| **PRO LOAEL^α^** | 97% | 93% | 99% | 104% | 105% | 113% | 96% | 102% | 102% | 99% |
| **ADI MIX^γ^** | 102% | 94% | 103% | 99% | 106% | 103% | 103% | 100% | 98% | 120% |
| **NOAEL MIX^γ^** | 106% | 104% | 101% | 103% | 114% | 113% | 106% | 114% | 111% | 146% |
| **LOAEL MIX^γ^** | 103% | 104% | 97% | 103% | 114% | 109% | 76%** | 105% | 115% | 124% |

Data are presented as percentages relative to control group 0, *: p ≤ 0.05, **: p ≤ 0.01

Supplementary Table 23: Relative organ weights of Subset 3 (PND 83±2) female offspring after single-substance and mixed exposures to anti-androgens

| **Relative Weights** | **Adrenal Glands** | **Brain** | **Kidneys** | **Liver** | **Ovaries** | **Pituitary Gland** | **Spleen** | **Thyroid Gland** | **Uterus** |
| --- | --- | --- | --- | --- | --- | --- | --- | --- | --- |
| **Positive Control^α^** | 95% | 103% | 101% | 99% | 110% | 115% | 111% | 100% | 91% |
| **VIN ADI^α^** | 103% | 101% | 102% | 101% | 111% | 104% | 115% | 110% | 109% |
| **VIN NOAEL^α^** | 104% | 102% | 104% | 101% | 107% | 109% | 109% | 104% | 86% |
| **VIN LOAEL^α^** | 101% | 104% | 103% | 100% | 110% | 106% | 109% | 102% | 101% |
| **FLT ADI^γ^** | 96% | 99% | 100% | 100% | 100% | 104% | 100% | 102% | 122% |
| **FLT NOAEL^β^** | 99% | 95% | 107% | 102% | 106% | 96% | 102% | 104% | 105% |
| **FLT LOAEL^α^** | 97% | 104% | 104% | 99% | 105% | 117% | 107% | 105% | 101% |
| **PRO ADI^α^** | 101% | 104% | 104% | 103% | 113% | 117% | 104% | 106% | 94% |
| **PRO NOAEL^α^** | 98% | 103% | 104% | 101% | 112% | 118% | 108% | 99% | 82% |
| **PRO LOAEL^α^** | 96% | 102% | 106% | 108% | 117% | 99% | 104% | 105% | 101% |
| **ADI MIX^γ^** | 92% | 101% | 97% | 104% | 102% | 102% | 99% | 97% | 119% |
| **NOAEL MIX^γ^** | 99% | 96% | 97% | 108% | 107% | 100% | 107% | 105% | 137% |
| **LOAEL MIX^γ^** | 104% | 94% | 100% | 111% | 106% | 74%** | 102% | 113% | 122% |

Data are presented as percentages relative to control group 0, *: p ≤ 0.05, **: p ≤ 0.01

Supplementary Table 24: Serum hormone levels in parental females after single-substance and mixed exposures to anti-androgens

| **Treatment** | **Androstenedione (nmol/L)** | | | | **Testosterone (nmol/L)** | | | | **Progesterone (nmol/L)** | | | | **Estradiol (pmol/L)** | | | |
| --- | --- | --- | --- | --- | --- | --- | --- | --- | --- | --- | --- | --- | --- | --- | --- | --- |
|  | **Mean** | **SD** | **N** | **Median** | **Mean** | **SD** | **N** | **Median** | **Mean** | **SD** | **N** | **Median** | **Mean** | **SD** | **N** | **Median** |
| **Vehicle Control^α^** | 2.15 | 1.44 | 20 | 1.96 | 0.52 | 0.14 | 20 | 0.50 | 40.60 | 50.16 | 19 | 11.83 | 10.45 | 11.17 | 20 | 4.83 |
| **Vehicle Control^β^** | 1.50 | 0.74 | 19 | 1.42 | 18.30 | 6.53 | 19 | 16.76 | 11.75 | 10.74 | 19 | 8.27 | 25.93 | 6.85 | 19 | 29.20 |
| **Vehicle Control^γ^** | 1.60 | 0.99 | 17 | 1.28 | 0.42 | 0.19 | 17 | 0.35 | 24.65 | 41.11 | 17 | 13.04 | 26.76 | 7.22 | 19 | 27.88 |
| **Positive Control^α^** | 3.10 | 2.33 | 18 | 2.31 | 0.55 | 0.20 | 18 | 0.51 | 54.85 | 47.53 | 18 | 25.65 | 11.94 | 9.80 | 20 | 10.12 |
| **VIN ADI^α^** | 1.95 | 1.54 | 19 | 1.34 | 0.49 | 0.15 | 19 | 0.47 | 27.26 | 39.56 | 19 | 9.54 | 9.51 | 9.63 | 19 | 8.01 |
| **VIN NOAEL^α^** | 2.35 | 3.15 | 16 | 1.60 | 0.57 | 0.44 | 17 | 0.47 | 31.95 | 40.79 | 17 | 14.88 | 11.43 | 12.12 | 18 | 7.41 |
| **VIN LOAEL^α^** | 2.82 | 1.59 | 17 | 2.28 | 0.65 | 0.21 | 18 | 0.57 | 49.04 | 59.13 | 17 | 13.64 | 12.11 | 12.07 | 18 | 12.83 |
| **FLT ADI^γ^** | 1.48 | 0.75 | 18 | 1.27 | 0.39 | 0.20 | 18 | 0.35 | 22.00 | 22.48 | 18 | 14.76 | 26.79 | 5.89 | 19 | 26.45 |
| **FLT NOAEL^β^** | 1.23 | 0.47 | 16 | 1.34 | 18.39 | 4.84 | 16 | 17.54 | 17.21 | 10.39 | 16 | 16.49 | 24.53 | 6.16 | 18 | 24.46 |
| **FLT LOAEL^α^** | 2.45 | 1.48 | 20 | 2.42 | 0.56 | 0.24 | 20 | 0.50 | 56.79 | 63.94 | 20 | 20.24 | 14.76 | 11.32 | 20 | 15.72 |
| **PRO ADI^α^** | 2.94 | 3.14 | 19 | 1.85 | 0.56 | 0.26 | 19 | 0.46 | 33.21 | 35.65 | 19 | 22.13 | 14.59 | 16.53 | 19 | 9.62 |
| **PRO NOAEL^α^** | 2.99 | 2.02 | 18 | 2.58 | **0.79*** | 0.35 | 19 | 0.71 | 58.25 | 58.54 | 19 | 35.62 | 12.01 | 10.78 | 19 | 15.11 |
| **PRO LOAEL^α^** | 3.77 | 3.07 | 16 | 3.26 | 0.80 | 0.42 | 18 | 0.77 | 66.84 | 74.17 | 18 | 35.62 | 4.29 | 6.15 | 18 | 0.71 |
| **ADI MIX^γ^** | 1.26 | 0.78 | 20 | 1.00 | 0.39 | 0.11 | 20 | 0.35 | 32.27 | 48.28 | 20 | 15.12 | 23.32 | 7.06 | 20 | 23.49 |
| **NOAEL MIX^γ^** | 1.55 | 0.88 | 19 | 1.48 | 0.44 | 0.17 | 19 | 0.35 | 27.66 | 39.67 | 19 | 15.84 | **19.87**** | 6.09 | 19 | 20.48 |
| **LOAEL MIX^γ^** | 2.93 | 2.51 | 17 | 2.45 | 0.55 | 0.25 | 17 | 0.48 | 55.03 | 79.39 | 17 | 20.07 | **16.66**** | 8.45 | 18 | 17.18 |

Supplementary Table 25: Serum hormone levels in parental females after single-substance and mixed exposures to anti-androgens

| **Treatment** | **11-Deoxycorticosterone (nmol/L)** | | | | **Corticosterone (nmol/L)** | | | | **Cortisol (nmol/L)** | | | |
| --- | --- | --- | --- | --- | --- | --- | --- | --- | --- | --- | --- | --- |
|  | **Mean** | **SD** | **N** | **Median** | **Mean** | **SD** | **N** | **Median** | **Mean** | **SD** | **N** | **Median** |
| **Vehicle Control^α^** | 3.37 | 3.88 | 20 | 1.52 | 435.8 | 485.6 | 20 | 268.8 |  |  |  |  |
| **Vehicle Control^β^** |  |  |  |  | 852.0 | 516.5 | 19 | 1011.5 |  |  |  |  |
| **Vehicle Control^γ^** | 6.34 | 4.55 | 17 | 4.67 | 731.6 | 0.5 | 17 | 650.3 |  |  |  |  |
| **Positive Control^α^** | **9.2**** | 8.92 | 18 | 5.26 | **961.0**** | 670.2 | 18 | 783.2 |  |  |  |  |
| **VIN ADI^α^** | 3.39 | 2.34 | 19 | 2.49 | 517.0 | 364.8 | 19 | 514.4 |  |  |  |  |
| **VIN NOAEL^α^** | **13.34**** | 15.92 | 17 | 6.48 | **1021.3**** | 704.8 | 17 | 1052.0 |  |  |  |  |
| **VIN LOAEL^α^** | **7.02**** | 8.94 | 18 | 4.68 | **924.7**** | 495.3 | 18 | 897.4 |  |  |  |  |
| **FLT ADI^γ^** | 10.84 | 11.02 | 18 | 8.03 | 969.9 | 565.4 | 18 | 984.0 |  |  |  |  |
| **FLT NOAEL^β^** |  |  |  |  | **1188.3*** | 334.0 | 16 | 1281.7 |  |  |  |  |
| **FLT LOAEL^α^** | **9.28**** | 7.89 | 20 | 6.26 | **972.1**** | 579.6 | 20 | 895.9 |  |  |  |  |
| **PRO ADI^α^** | **14.68**** | 13.21 | 19 | 11.33 | **1185.7**** | 608.0 | 19 | 1658.9 |  |  |  |  |
| **PRO NOAEL^α^** | **10.52**** | 8.92 | 19 | 8.30 | **1164.4**** | 757.0 | 19 | 1283.2 |  |  |  |  |
| **PRO LOAEL^α^** | **22.4**** | 25.95 | 18 | 10.30 | **1119.6**** | 807.2 | 18 | 1040.4 |  |  |  |  |
| **ADI MIX^γ^** | 10.96 | 10.37 | 20 | 6.42 | 863.3 | 438.6 | 20 | 764.4 |  |  |  |  |
| **NOAEL MIX^γ^** | 7.76 | 7.65 | 19 | 5.69 | 843.0 | 448.6 | 19 | 849.7 |  |  |  |  |
| **LOAEL MIX^γ^** | 10.75 | 8.86 | 17 | 8.78 | 965.2 | 396.2 | 17 | 997.1 |  |  |  |  |

Supplementary Table 26: Serum hormone levels in PND 21 males after single-substance and mixed exposures to anti-androgens

| **Treatment** | **Androstenedione (nmol/L)** | | | | **Testosterone (nmol/L)** | | | | **Progesterone (nmol/L)** | | | |
| --- | --- | --- | --- | --- | --- | --- | --- | --- | --- | --- | --- | --- |
|  | **Mean** | **SD** | **N** | **Median** | **Mean** | **SD** | **N** | **Median** | **Mean** | **SD** | **N** | **Median** |
| **Vehicle Control^α^** | 1.03 | 1.57 | 9 | 0.37 | 0.86 | 0.94 | 10 | 0.50 | 6.49 | 4.04 | 9 | 5.25 |
| **Vehicle Control^β^** | 0.62 | 0.35 | 4 | 0.52 | 0.70 | 0.53 | 4 | 0.48 | 6.21 | 4.12 | 5 | 7.22 |
| **Vehicle Control^γ^** | 0.62 | 0.48 | 10 | 0.38 | 0.72 | 0.64 | 10 | 0.35 | 6.96 | 3.18 | 10 | 6.81 |
| **Positive Control^α^** | 1.10 | 1.66 | 8 | 0.35 | 0.83 | 0.98 | 10 | 0.35 | 8.26 | 4.63 | 10 | 9.24 |
| **VIN ADI^α^** | 0.43 | 0.15 | 7 | 0.35 | 0.42 | 0.10 | 9 | 0.38 | 4.01 | 2.25 | 9 | 4.13 |
| **VIN NOAEL^α^** | 0.53 | 0.33 | 10 | 0.35 | 0.56 | 0.24 | 10 | 0.47 | 5.75 | 2.81 | 10 | 6.13 |
| **VIN LOAEL^α^** | 0.39 | 0.11 | 7 | 0.35 | 0.40 | 0.10 | 10 | 0.37 | 7.19 | 2.63 | 10 | 6.39 |
| **FLT ADI^γ^** | 0.94 | 1.21 | 10 | 0.40 | 0.90 | 0.98 | 10 | 0.35 | 5.04 | 3.36 | 10 | 4.40 |
| **FLT NOAEL^β^** | 0.35 |  | 1 | 0.35 | 0.35 |  | 1 | 0.35 | 2.75 | 1.44 | 2 | 2.75 |
| **FLT LOAEL^α^** | 0.88 | 1.07 | 8 | 0.35 | 0.71 | 0.66 | 10 | 0.39 | 7.23 | 5.23 | 10 | 4.83 |
| **PRO ADI^α^** | 0.60 | 0.52 | 8 | 0.35 | 0.57 | 0.41 | 10 | 0.35 | 5.25 | 1.86 | 10 | 4.88 |
| **PRO NOAEL^α^** | 0.42 | 0.22 | 10 | 0.35 | 0.48 | 0.27 | 10 | 0.39 | 6.83 | 2.68 | 10 | 7.65 |
| **PRO LOAEL^α^** | 0.64 | 0.38 | 9 | 0.35 | 0.74 | 0.59 | 10 | 0.36 | 5.86 | 2.90 | 10 | 5.15 |
| **ADI MIX^γ^** | 0.48 | 0.29 | 9 | 0.35 | 0.59 | 0.47 | 9 | 0.35 | 5.57 | 3.96 | 9 | 3.78 |
| **NOAEL MIX^γ^** | 0.53 | 0.26 | 9 | 0.35 | 0.64 | 0.42 | 9 | 0.35 | **3.4**** | 1.55 | 9 | 3.37 |
| **LOAEL MIX^γ^** | 0.54 | 0.54 | 9 | 0.35 | 0.37 | 0.89 | 9 | 0.35 | **2.44**** | 1.03 | 9 | 2.04 |

Supplementary Table 27: Serum hormone levels in PND 21 males after single-substance and mixed exposures to anti-androgens

| **Treatment** | **11-Deoxycorticosterone (nmol/L)** | | | | **Corticosterone (nmol/L)** | | | | **Cortisol (nmol/L)** | | | |
| --- | --- | --- | --- | --- | --- | --- | --- | --- | --- | --- | --- | --- |
|  | **Mean** | **SD** | **N** | **Median** | **Mean** | **SD** | **N** | **Median** | **Mean** | **SD** | **N** | **Median** |
| **Vehicle Control^α^** |  |  |  |  | 756.2 | 197.0 | 9 | 713.8 | 0.82 | 0.35 | 10 | 0.66 |
| **Vehicle Control^β^** |  |  |  |  | 604.8 | 273.5 | 5 | 670.5 |  |  |  |  |
| **Vehicle Control^γ^** |  |  |  |  | 811.2 | 106.8 | 10 | 851.1 | 0.79 | 0.30 | 10 | 0.83 |
| **Positive Control^α^** |  |  |  |  | 866.7 | 193.7 | 10 | 901.7 | 0.92 | 0.33 | 10 | 0.95 |
| **VIN ADI^α^** |  |  |  |  | 660.5 | 162.8 | 9 | 690.7 | 0.77 | 0.44 | 9 | 0.59 |
| **VIN NOAEL^α^** |  |  |  |  | 691.3 | 264.1 | 10 | 791.9 | 0.66 | 0.19 | 10 | 0.69 |
| **VIN LOAEL^α^** |  |  |  |  | 797.9 | 172.7 | 10 | 765.9 | 0.84 | 0.30 | 10 | 0.76 |
| **FLT ADI^γ^** |  |  |  |  | 743.6 | 102.3 | 10 | 784.6 | 0.98 | 0.61 | 10 | 0.81 |
| **FLT NOAEL^β^** |  |  |  |  | 461.0 | 67.4 | 2 | 461.0 |  |  |  |  |
| **FLT LOAEL^α^** |  |  |  |  | 837.2 | 144.0 | 10 | 869.9 | 0.90 | 0.39 | 10 | 0.88 |
| **PRO ADI^α^** |  |  |  |  | 748.8 | 149.0 | 10 | 764.5 | 0.52 | 0.25 | 10 | 0.48 |
| **PRO NOAEL^α^** |  |  |  |  | 827.4 | 150.7 | 10 | 859.8 | 0.67 | 0.13 | 10 | 0.65 |
| **PRO LOAEL^α^** |  |  |  |  | 792.5 | 167.3 | 10 | 763.0 | 0.85 | 0.43 | 10 | 0.79 |
| **ADI MIX^γ^** |  |  |  |  | 673.7* | 146.1 | 9 | 647.4 | **0.56** | 0.39 | 8 | 0.43 |
| **NOAEL MIX^γ^** |  |  |  |  | 739.5 | 153.5 | 9 | 710.9 | 0.80 | 0.42 | 9 | 0.62 |
| **LOAEL MIX^γ^** |  |  |  |  | 589.1** | 93.0 | 9 | 702.3 | **0.70** | 0.32 | 9 | 0.61 |

Supplementary Table 28: Serum hormone levels in PND 21 females after single-substance and mixed exposures to anti-androgens

| **Treatment** | **Androstenedione (nmol/L)** | | | | **Testosterone (nmol/L)** | | | | **Progesterone (nmol/L)** | | | |
| --- | --- | --- | --- | --- | --- | --- | --- | --- | --- | --- | --- | --- |
|  | **Mean** | **SD** | **N** | **Median** | **Mean** | **SD** | **N** | **Median** | **Mean** | **SD** | **N** | **Median** |
| **Vehicle Control^α^** | 0.42 | 0.17 | 10 | 0.35 |  |  |  |  | 3.90 | 2.61 | 10 | 4.18 |
| **Vehicle Control^β^** | 0.72 | 1.13 | 10 | 0.35 |  |  |  |  | 7.24 | 3.71 | 10 | 6.92 |
| **Vehicle Control^γ^** | 0.35 | 0.02 | 10 | 0.35 |  |  |  |  | 5.52 | 2.50 | 9 | 5.37 |
| **Positive Control^α^** | 1.02 | 1.96 | 10 | 0.35 |  |  |  |  | 6.02 | 3.29 | 10 | 6.31 |
| **VIN ADI^α^** | 0.50 | 0.29 | 9 | 0.35 |  |  |  |  | 6.66 | 3.24 | 10 | 5.87 |
| **VIN NOAEL^α^** | 0.67 | 0.97 | 10 | 0.35 |  |  |  |  | 3.26 | 2.00 | 10 | 2.71 |
| **VIN LOAEL^α^** | 0.90 | 1.67 | 10 | 0.35 |  |  |  |  | 5.35 | 2.57 | 10 | 5.61 |
| **FLT ADI^γ^** | **0.54*** | 0.43 | 10 | 0.38 |  |  |  |  | 5.58 | 2.74 | 10 | 5.28 |
| **FLT NOAEL^β^** | 0.81 | 0.92 | 10 | 0.35 |  |  |  |  | 6.55 | 3.50 | 10 | 5.99 |
| **FLT LOAEL^α^** | 0.57 | 0.47 | 10 | 0.35 |  |  |  |  | 4.62 | 2.53 | 9 | 3.78 |
| **PRO ADI^α^** | 0.56 | 0.55 | 9 | 0.35 |  |  |  |  | 4.48 | 2.41 | 9 | 3.28 |
| **PRO NOAEL^α^** | 0.71 | 0.84 | 10 | 0.35 |  |  |  |  | 2.71 | 1.30 | 9 | 2.37 |
| **PRO LOAEL^α^** | 1.10 | 1.26 | 8 | 0.43 |  |  |  |  | 4.94 | 3.10 | 10 | 4.23 |
| **ADI MIX^γ^** | 0.82 | 1.42 | 10 | 0.50 |  |  |  |  | 5.20 | 2.15 | 9 | 4.83 |
| **NOAEL MIX^γ^** | 0.57 | 0.44 | 10 | 0.35 |  |  |  |  | 4.00 | 1.79 | 10 | 4.02 |
| **LOAEL MIX^γ^** | 0.36 | 0.04 | 10 | 0.35 |  |  |  |  | 4.02 | 1.99 | 10 | 4.09 |

Supplementary Table 29: Serum hormone levels in PND 21 females after single-substance and mixed exposures to anti-androgens

| **Treatment** | **11-Deoxycorticosterone (nmol/L)** | | | | **Corticosterone (nmol/L)** | | | | **Cortisol (nmol/L)** | | | |
| --- | --- | --- | --- | --- | --- | --- | --- | --- | --- | --- | --- | --- |
|  | **Mean** | **SD** | **N** | **Median** | **Mean** | **SD** | **N** | **Median** | **Mean** | **SD** | **N** | **Median** |
| **Vehicle Control^α^** |  |  |  |  | 751.0 | 289.3 | 9 | 797.6 | 1.05 | 0.52 | 9 | 0.94 |
| **Vehicle Control^β^** |  |  |  |  | 770.6 | 241.4 | 10 | 842.4 |  |  |  |  |
| **Vehicle Control^γ^** |  |  |  |  | 802.6 | 211.4 | 10 | 751.4 | 1.02 | 0.51 | 10 | 1.07 |
| **Positive Control^α^** |  |  |  |  | 747.6 | 289.4 | 10 | 796.2 | 0.80 | 0.47 | 9 | 0.67 |
| **VIN ADI^α^** |  |  |  |  | 937.8 | 205.0 | 10 | 953.7 | 0.56 | 0.12 | 7 | 0.54 |
| **VIN NOAEL^α^** |  |  |  |  | 662.2 | 284.2 | 10 | 703.8 | 0.73 | 0.36 | 8 | 0.69 |
| **VIN LOAEL^α^** |  |  |  |  | 813.7 | 236.6 | 10 | 880.0 | 0.79 | 0.34 | 7 | 0.86 |
| **FLT ADI^γ^** |  |  |  |  | 781.7 | 134.7 | 10 | 761.5 | 0.65 | 0.31 | 9 | 0.64 |
| **FLT NOAEL^β^** |  |  |  |  | 815.8 | 111.8 | 10 | 851.1 |  |  |  |  |
| **FLT LOAEL^α^** |  |  |  |  | 882.4 | 114.9 | 9 | 884.3 | 0.57 | 0.29 | 9 | 0.48 |
| **PRO ADI^α^** |  |  |  |  | 906.8 | 182.4 | 9 | 947.9 | 0.74 | 0.35 | 7 | 0.60 |
| **PRO NOAEL^α^** |  |  |  |  | 635.7 | 267.3 | 10 | 672.0 | 0.62 | 0.29 | 6 | 0.56 |
| **PRO LOAEL^α^** |  |  |  |  | 836.1 | 189.3 | 10 | 882.9 | 0.83 | 0.44 | 10 | 0.74 |
| **ADI MIX^γ^** |  |  |  |  | 756.6 | 106.3 | 10 | 758.6 | 0.59 | 0.33 | 9 | 0.48 |
| **NOAEL MIX^γ^** |  |  |  |  | 762.1 | 114.1 | 10 | 765.9 | 0.77 | 0.56 | 10 | 0.50 |
| **LOAEL MIX^γ^** |  |  |  |  | 689.6 | 189.2 | 10 | 716.7 | 0.58* | 0.31 | 10 | 0.55 |

Supplementary Table 30: Serum hormone levels in males at puberty after single-substance and mixed exposures to anti-androgens

| **Treatment** | **Androstenedione (nmol/L)** | | | | **Testosterone (nmol/L)** | | | | **Progesterone (nmol/L)** | | | |
| --- | --- | --- | --- | --- | --- | --- | --- | --- | --- | --- | --- | --- |
|  | **Mean** | **SD** | **N** | **Median** | **Mean** | **SD** | **N** | **Median** | **Mean** | **SD** | **N** | **Median** |
| **Vehicle Control^α^** | 1.60 | 1.71 | 10 | 0.72 | 4.72 | 4.13 | 10 | 3.19 | 7.24 | 8.00 | 10 | 3.98 |
| **Vehicle Control^β^** | 0.54 | 0.28 | 10 | 0.39 | 2.03 | 1.71 | 10 | 1.34 | 7.96 | 5.62 | 10 | 6.93 |
| **Vehicle Control^γ^** | 0.65 | 0.54 | 10 | 0.40 | 3.06 | 3.06 | 10 | 1.74 | 1.95 | 1.60 | 10 | 1.56 |
| **Positive Control^α^** | **4.77*** | 4.40 | 10 | 3.33 | **12.66*** | 11.96 | 10 | 6.68 | 6.43 | 7.41 | 8 | 4.67 |
| **VIN ADI^α^** | 0.69 | 0.38 | 10 | 0.55 | 2.05 | 0.91 | 10 | 1.83 | 8.18 | 9.45 | 10 | 3.66 |
| **VIN NOAEL^α^** | 0.75 | 0.61 | 9 | 0.51 | 1.68 | 1.21 | 10 | 1.36 | 7.50 | 6.06 | 10 | 6.03 |
| **VIN LOAEL^α^** | 0.86 | 0.91 | 10 | 0.42 | 2.32 | 2.47 | 10 | 1.70 | 9.18 | 5.70 | 9 | 5.79 |
| **FLT ADI^γ^** | 0.46 | 0.16 | 10 | 0.35 | 1.94 | 1.32 | 10 | 1.78 | 5.51 | 12.77 | 10 | 1.02 |
| **FLT NOAEL^β^** | 0.75 | 0.55 | 9 | 0.40 | 3.23 | 2.60 | 9 | 2.00 | 3.66 | 3.60 | 9 | 1.60 |
| **FLT LOAEL^α^** | 0.89 | 0.79 | 10 | 0.48 | 3.08 | 3.85 | 10 | 1.84 | 4.75 | 5.25 | 10 | 3.43 |
| **PRO ADI^α^** | 0.69 | 0.36 | 8 | 0.62 | 1.92 | 1.40 | 9 | 1.58 | 6.84 | 6.72 | 9 | 5.02 |
| **PRO NOAEL^α^** | 0.68 | 0.56 | 7 | 0.46 | 1.78 | 1.71 | 8 | 1.07 | 5.96 | 7.48 | 9 | 2.24 |
| **PRO LOAEL^α^** | 0.76 | 0.40 | 9 | 0.70 | 1.70 | 1.08 | 10 | 1.30 | 12.54 | 10.55 | 10 | 10.81 |
| **ADI MIX^γ^** | 0.60 | 0.29 | 10 | 0.54 | 2.96 | 1.81 | 10 | 3.52 | 3.89 | 4.22 | 10 | 2.52 |
| **NOAEL MIX^γ^** | 0.57 | 0.27 | 10 | 0.54 | 3.59 | 3.05 | 10 | 2.82 | 3.03 | 3.41 | 10 | 2.23 |
| **LOAEL MIX^γ^** | 0.62 | 0.31 | 10 | 0.52 | 3.94 | 2.30 | 10 | 3.41 | 2.34 | 2.58 | 10 | 1.62 |

Supplementary Table 31: Serum hormone levels in males at puberty after single-substance and mixed exposures to anti-androgens

| **Treatment** | **11-Deoxycorticosterone (nmol/L)** | | | | **Corticosterone (nmol/L)** | | | | **Cortisol (nmol/L)** | | | |
| --- | --- | --- | --- | --- | --- | --- | --- | --- | --- | --- | --- | --- |
|  | **Mean** | **SD** | **N** | **Median** | **Mean** | **SD** | **N** | **Median** | **Mean** | **SD** | **N** | **Median** |
| **Vehicle Control^α^** | 4.91 | 4.25 | 10 | 2.76 | 765.9 | 660.2 | 10 | 377.2 |  |  |  |  |
| **Vehicle Control^β^** | 8.08 | 6.78 | 10 | 6.04 | 814.1 | 363.8 | 10 | 893.0 |  |  |  |  |
| **Vehicle Control^γ^** | 2.43 | 1.01 | 10 | 2.30 | 466.8 | 335.5 | 10 | 504.3 |  |  |  |  |
| **Positive Control^α^** | 7.37 | 9.06 | 10 | 2.90 | 533.0 | 454.3 | 10 | 481.6 |  |  |  |  |
| **VIN ADI^α^** | 5.44 | 4.03 | 10 | 4.26 | 804.6 | 518.6 | 10 | 976.9 |  |  |  |  |
| **VIN NOAEL^α^** | 5.66 | 3.38 | 10 | 5.62 | 965.3 | 348.5 | 10 | 1104.0 |  |  |  |  |
| **VIN LOAEL^α^** | 6.13 | 4.13 | 10 | 5.79 | 946.0 | 419.9 | 10 | 1065.0 |  |  |  |  |
| **FLT ADI^γ^** | 3.73 | 4.86 | 10 | 1.72 | 503.4 | 353.5 | 10 | 391.6 |  |  |  |  |
| **FLT NOAEL^β^** | **2.71*** | 1.39 | 9 | 1.99 | 613.2 | 356.1 | 9 | 523.1 |  |  |  |  |
| **FLT LOAEL^α^** | 4.18 | 3.76 | 10 | 2.34 | 614.5 | 414.8 | 10 | 583.8 |  |  |  |  |
| **PRO ADI^α^** | 5.02 | 5.94 | 9 | 3.39 | 899.1 | 342.2 | 9 | 976.8 |  |  |  |  |
| **PRO NOAEL^α^** | 4.38 | 4.72 | 9 | 2.10 | 711.6 | 474.9 | 9 | 543.3 |  |  |  |  |
| **PRO LOAEL^α^** | 6.64 | 4.27 | 10 | 6.97 | 938.3 | 562.5 | 10 | 1063.5 |  |  |  |  |
| **ADI MIX^γ^** | 3.81 | 2.95 | 10 | 3.02 | 635.9 | 274.2 | 10 | 667.6 |  |  |  |  |
| **NOAEL MIX^γ^** | 3.21 | 1.51 | 10 | 3.06 | 476.0 | 273.3 | 10 | 554.9 |  |  |  |  |
| **LOAEL MIX^γ^** | 2.92 | 1.97 | 10 | 1.75 | 428.8 | 35.1 | 10 | 343.9 |  |  |  |  |

Supplementary Table 32: Serum hormone levels in females at puberty after single-substance and mixed exposures to anti-androgens

| **Treatment** | **Androstenedione (nmol/L)** | | | | **Testosterone (nmol/L)** | | | | **Progesterone (nmol/L)** | | | | **Estradiol (pmol/L)** | | | |
| --- | --- | --- | --- | --- | --- | --- | --- | --- | --- | --- | --- | --- | --- | --- | --- | --- |
|  | **Mean** | **SD** | **N** | **Median** | **Mean** | **SD** | **N** | **Median** | **Mean** | **SD** | **N** | **Median** | **Mean** | **SD** | **N** | **Median** |
| **Vehicle Control^α^** | 0.56 | 0.52 | 10 | 0.35 | 0.55 | 0.15 | 10 | 0.52 | 14.89 | 11.29 | 10 | 8.48 | 4.08 | 7.90 | 10 | 0.00 |
| **Vehicle Control^β^** | 0.38 | 0.08 | 10 | 0.35 |  |  |  |  | 23.46 | 17.35 | 10 | 19.73 | 6.34 | 1.66 | 10 | 5.74 |
| **Vehicle Control^γ^** | 0.36 | 0.04 | 10 | 0.35 | 0.36 | 0.05 | 9 | 0.35 | 9.93 | 3.12 | 10 | 10.08 | 8.80 | 2.72 | 10 | 8.54 |
| **Positive Control^α^** | 0.49 | 0.19 | 9 | 0.36 | 0.48 | 0.14 | 9 | 0.43 | 25.93 | 25.45 | 9 | 19.78 | 2.29 | 3.40 | 9 | 0.21 |
| **VIN ADI^α^** | 0.66 | 0.50 | 10 | 0.35 | 0.54 | 0.18 | 10 | 0.49 | 10.55 | 6.16 | 10 | 10.00 | 1.80 | 3.11 | 10 | 0.00 |
| **VIN NOAEL^α^** | 1.47 | 2.91 | 10 | 0.50 | 0.68 | 0.16 | 10 | 0.64 | 26.51 | 28.57 | 10 | 20.32 | 1.62 | 3.64 | 10 | 0.00 |
| **VIN LOAEL^α^** | 0.45 | 0.18 | 10 | 0.35 | 0.48 | 0.17 | 10 | 0.39 | 13.83 | 14.19 | 10 | 6.47 | 4.22 | 11.68 | 10 | 0.00 |
| **FLT ADI^γ^** | 0.38 | 0.11 | 10 | 0.35 | 0.35 | 0.00 | 8 | 0.35 | 9.00 | 8.02 | 10 | 8.75 | 9.02 | 2.88 | 10 | 9.19 |
| **FLT NOAEL^β^** | 0.40 | 0.13 | 10 | 0.35 |  |  |  |  | 12.41 | 10.22 | 10 | 10.78 | 7.12 | 2.95 | 10 | 5.81 |
| **FLT LOAEL^α^** | 0.46 | 0.30 | 10 | 0.35 | 0.50 | 0.15 | 10 | 0.47 | 13.27 | 10.53 | 10 | 12.49 | 0.42 | 0.91 | 10 | 0.00 |
| **PRO ADI^α^** | 0.44 | 0.17 | 10 | 0.35 | 0.51 | 0.21 | 10 | 0.46 | 22.36 | 23.30 | 10 | 14.47 | 0.82 | 1.81 | 10 | 0.00 |
| **PRO NOAEL^α^** | 0.42 | 0.11 | 10 | 0.36 | 0.53 | 0.17 | 10 | 0.51 | 21.28 | 18.29 | 10 | 16.71 | 1.65 | 2.72 | 10 | 0.00 |
| **PRO LOAEL^α^** | 3.14 | 8.10 | 9 | 0.35 | 0.55 | 0.47 | 9 | 0.36 | 18.69 | 31.60 | 9 | 10.46 | 2.54 | 6.57 | 10 | 0.00 |
| **ADI MIX^γ^** | 0.35 | 0.01 | 10 | 0.35 | 0.35 | 0.01 | 10 | 0.35 | 9.85 | 6.57 | 10 | 9.08 | 9.14 | 3.81 | 10 | 9.22 |
| **NOAEL MIX^γ^** | 0.36 | 0.03 | 10 | 0.35 | 0.35 | 0.00 | 10 | 0.35 | 11.74 | 10.48 | 10 | 10.08 | 8.30 | 3.00 | 10 | 7.45 |
| **LOAEL MIX^γ^** | 0.44 | 0.13 | 10 | 0.39 | 0.35 | 0.00 | 10 | 0.35 | 33.49 | 80.29 | 10 | 6.34 | **8.90** | 4.06 | 10 | 8.38 |

Supplementary Table 33: Serum hormone levels in females at puberty after single-substance and mixed exposures to anti-androgens

| **Treatment** | **11-Deoxycorticosterone (nmol/L)** | | | | **Corticosterone (nmol/L)** | | | | **Cortisol (nmol/L)** | | | |
| --- | --- | --- | --- | --- | --- | --- | --- | --- | --- | --- | --- | --- |
|  | **Mean** | **SD** | **N** | **Median** | **Mean** | **SD** | **N** | **Median** | **Mean** | **SD** | **N** | **Median** |
| **Vehicle Control^α^** | 2.64 | 1.20 | 10 | 2.77 | 844.4 | 527.8 | 10 | 880.0 |  |  |  |  |
| **Vehicle Control^β^** | 2.75 | 2.47 | 9 | 1.78 | 494.1 | 605.7 | 10 | 356.0 |  |  |  |  |
| **Vehicle Control^γ^** | 2.06 | 0.98 | 10 | 1.59 | 343.7 | 417.6 | 10 | 142.9 |  |  |  |  |
| **Positive Control^α^** | 5.77 | 3.91 | 9 | 3.45 | 1217.1 | 575.9 | 9 | 1554.8 |  |  |  |  |
| **VIN ADI^α^** | 2.99 | 1.65 | 10 | 2.89 | 784.7 | 569.7 | 10 | 877.2 |  |  |  |  |
| **VIN NOAEL^α^** | 3.99 | 1.57 | 10 | 4.49 | 1132.9 | 491.0 | 10 | 1225.4 |  |  |  |  |
| **VIN LOAEL^α^** | 3.89 | 4.84 | 10 | 2.26 | 942.4 | 327.0 | 10 | 897.4 |  |  |  |  |
| **FLT ADI^γ^** | 2.24 | 1.87 | 10 | 1.59 | 381.0 | 377.8 | 10 | 285.2 |  |  |  |  |
| **FLT NOAEL^β^** | 2.72 | 3.21 | 9 | 1.59 | 259.7 | 353.5 | 10 | 107.7 |  |  |  |  |
| **FLT LOAEL^α^** | 1.96 | 0.94 | 10 | 1.52 | 351.5 | 490.2 | 10 | 129.7 |  |  |  |  |
| **PRO ADI^α^** | 2.91 | 1.68 | 10 | 2.01 | 853.8 | 554.4 | 10 | 767.3 |  |  |  |  |
| **PRO NOAEL^α^** | 4.13 | 2.68 | 10 | 3.97 | 898.0 | 683.9 | 10 | 776.0 |  |  |  |  |
| **PRO LOAEL^α^** | 2.48 | 1.81 | 9 | 1.52 | 678.0 | 521.7 | 9 | 739.8 |  |  |  |  |
| **ADI MIX^γ^** | 1.91 | 0.83 | 10 | 1.59 | 324.0 | 324.6 | 10 | 191.8 |  |  |  |  |
| **NOAEL MIX^γ^** | 2.30 | 1.28 | 10 | 1.59 | 463.2 | 572.1 | 10 | 160.0 |  |  |  |  |
| **LOAEL MIX^γ^** | 3.02 | 2.40 | 10 | 1.79 | 636.8 | 568.2 | 10 | 450.8 |  |  |  |  |

Supplementary Table 34: Serum hormone levels in PND 83 males after single-substance and mixed exposures to anti-androgens

| **Treatment** | **Androstenedione (nmol/L)** | | | | **Testosterone (nmol/L)** | | | | **Progesterone (nmol/L)** | | | |
| --- | --- | --- | --- | --- | --- | --- | --- | --- | --- | --- | --- | --- |
|  | **Mean** | **SD** | **N** | **Median** | **Mean** | **SD** | **N** | **Median** | **Mean** | **SD** | **N** | **Median** |
| **Vehicle Control^α^** | 2.27 | 1.81 | 10 | 1.72 | 5.79 | 5.27 | 10 | 4.45 | 11.26 | 7.93 | 10 | 9.99 |
| **Vehicle Control^β^** | 1.29 | 0.73 | 10 | 1.18 | 6.15 | 5.94 | 10 | 4.94 | 9.27 | 8.84 | 9 | 5.79 |
| **Vehicle Control^γ^** | 1.15 | 0.84 | 8 | 0.88 | 6.48 | 4.76 | 8 | 5.00 | 2.06 | 1.70 | 8 | 1.63 |
| **Positive Control^α^** | 3.65 | 1.33 | 10 | 3.99 | 9.09 | 4.51 | 10 | 7.78 | 16.04 | 9.45 | 10 | 11.23 |
| **VIN ADI^α^** | 3.93 | 2.36 | 9 | 3.23 | 10.12 | 6.59 | 9 | 8.61 | 18.89 | 22.52 | 7 | 8.71 |
| **VIN NOAEL^α^** | 3.76 | 2.34 | 9 | 3.45 | 9.89 | 7.78 | 10 | 9.46 | 15.48 | 16.42 | 9 | 7.63 |
| **VIN LOAEL^α^** | 3.05 | 3.89 | 10 | 1.82 | 8.66 | 10.74 | 10 | 4.50 | 8.35 | 8.47 | 10 | 4.05 |
| **FLT ADI^γ^** | 1.09 | 1.07 | 10 | 0.81 | 6.40 | 6.84 | 10 | 4.35 | 5.19 | 4.70 | 10 | 3.75 |
| **FLT NOAEL^β^** | 1.32 | 0.53 | 8 | 1.49 | 5.15 | 2.72 | 8 | 5.26 | 8.37 | 15.95 | 9 | 1.92 |
| **FLT LOAEL^α^** | **6.55*** | 4.45 | 10 | 6.14 | **17.4*** | 13.02 | 10 | 20.68 | 9.07 | 5.08 | 10 | 7.75 |
| **PRO ADI^α^** | 2.13 | 1.55 | 10 | 1.94 | 5.81 | 4.58 | 10 | 4.67 | 20.42 | 13.76 | 10 | 20.45 |
| **PRO NOAEL^α^** | 3.64 | 3.40 | 10 | 2.54 | 9.32 | 7.06 | 10 | 8.49 | 12.79 | 11.74 | 10 | 9.50 |
| **PRO LOAEL^α^** | 2.01 | 0.85 | 10 | 2.20 | 7.08 | 5.41 | 10 | 5.83 | 18.49 | 18.57 | 10 | 10.11 |
| **ADI MIX^γ^** | 1.29 | 1.38 | 10 | 0.78 | 5.69 | 5.16 | 10 | 4.08 | 4.93 | 4.37 | 10 | 3.79 |
| **NOAEL MIX^γ^** | 1.06 | 0.95 | 10 | 0.77 | 3.92 | 2.07 | 10 | 3.44 | 2.28 | 1.95 | 10 | 1.79 |
| **LOAEL MIX^γ^** | 2.05 | 1.52 | 10 | 1.63 | 6.37 | 4.24 | 10 | 4.98 | 2.76 | 2.39 | 10 | 2.07 |

Supplementary Table 35: Serum hormone levels in PND 83 males after single-substance and mixed exposures to anti-androgens

| **Treatment** | **11-Deoxycorticosterone (nmol/L)** | | | | **Corticosterone (nmol/L)** | | | | **Cortisol (nmol/L)** | | | |
| --- | --- | --- | --- | --- | --- | --- | --- | --- | --- | --- | --- | --- |
|  | **Mean** | **SD** | **N** | **Median** | **Mean** | **SD** | **N** | **Median** | **Mean** | **SD** | **N** | **Median** |
| **Vehicle Control^α^** | 19.23 | 11.88 | 10 | 17.09 | 959.2 | 446.6 | 10 | 1020.2 |  |  |  |  |
| **Vehicle Control^β^** | 21.14 | 15.75 | 9 | 17.24 | 838.9 | 390.2 | 10 | 965.3 |  |  |  |  |
| **Vehicle Control^γ^** | 7.39 | 6.13 | 8 | 5.04 | 434.5 | 250.3 | 8 | 395.9 |  |  |  |  |
| **Positive Control^α^** | 27.23 | 15.31 | 10 | 24.56 | 1227.4 | 235.3 | 10 | 1263.0 |  |  |  |  |
| **VIN ADI^α^** | 17.69 | 18.89 | 9 | 9.42 | 948.6 | 720.0 | 9 | 791.9 |  |  |  |  |
| **VIN NOAEL^α^** | 19.12 | 15.79 | 10 | 14.34 | 1087.5 | 573.6 | 10 | 1218.2 |  |  |  |  |
| **VIN LOAEL^α^** | 11.85 | 10.20 | 10 | 6.14 | 822.0 | 502.9 | 10 | 660.4 |  |  |  |  |
| **FLT ADI^γ^** | 19.93 | 9.36 | 10 | 11.75 | 716.0 | 375.8 | 10 | 752.8 |  |  |  |  |
| **FLT NOAEL^β^** | 13.28 | 20.13 | 8 | 4.88 | 649.6 | 495.7 | 9 | 445.1 |  |  |  |  |
| **FLT LOAEL^α^** | 16.20 | 7.74 | 10 | 16.79 | 872.2 | 305.6 | 10 | 877.2 |  |  |  |  |
| **PRO ADI^α^** | 28.11 | 17.26 | 10 | 30.03 | 1145.3 | 435.8 | 10 | 1341.0 |  |  |  |  |
| **PRO NOAEL^α^** | 22.62 | 17.53 | 10 | 20.09 | 981.8 | 539.1 | 10 | 982.6 |  |  |  |  |
| **PRO LOAEL^α^** | 29.47 | 23.94 | 10 | 26.85 | 1052.6 | 344.3 | 10 | 893.1 |  |  |  |  |
| **ADI MIX^γ^** | 9.63 | 8.09 | 10 | 7.55 | 674.1 | 457.7 | 10 | 593.9 |  |  |  |  |
| **NOAEL MIX^γ^** | 8.57 | 7.05 | 10 | 5.44 | 564.1 | 339.7 | 10 | 465.3 |  |  |  |  |
| **LOAEL MIX^γ^** | 8.06 | 6.29 | 10 | 5.88 | 568.4 | 414.6 | 10 | 426.3 |  |  |  |  |

Supplementary Table 36: Serum hormone levels in PND 83 females after single-substance and mixed exposures to anti-androgens

| **Treatment** | **Androstenedione (nmol/L)** | | | | **Testosterone (nmol/L)** | | | | **Progesterone (nmol/L)** | | | | **Estradiol (pmol/L)** | | | |
| --- | --- | --- | --- | --- | --- | --- | --- | --- | --- | --- | --- | --- | --- | --- | --- | --- |
|  | **Mean** | **SD** | **N** | **Median** | **Mean** | **SD** | **N** | **Median** | **Mean** | **SD** | **N** | **Median** | **Mean** | **SD** | **N** | **Median** |
| **Vehicle Control^α^** | 1.54 | 0.89 | 10 | 1.36 | 0.43 | 0.08 | 10 | 0.43 | 10.82 | 4.11 | 10 | 10.29 | 16.62 | 11.60 | 10 | 17.17 |
| **Vehicle Control^β^** | 1.16 | 0.24 | 10 | 1.28 |  |  |  |  | 12.00 | 3.31 | 10 | 11.11 | 23.06 | 6.10 | 10 | 20.91 |
| **Vehicle Control^γ^** | 1.87 | 1.28 | 9 | 1.43 | 0.47 | 0.16 | 9 | 0.39 | 40.27 | 60.15 | 9 | 10.53 | 23.55 | 9.78 | 8 | 24.61 |
| **Positive Control^α^** | **2.81*** | 1.08 | 9 | 2.44 | 0.51 | 0.21 | 9 | 0.43 | **20.51**** | 14.75 | 9 | 17.33 | 20.54 | 13.67 | 10 | 21.86 |
| **VIN ADI^α^** | 2.50 | 1.01 | 7 | 2.56 | 0.51 | 0.17 | 8 | 0.48 | 18.18 | 12.76 | 8 | 12.08 | 20.18 | 15.60 | 9 | 16.48 |
| **VIN NOAEL^α^** | 2.21 | 1.09 | 10 | 2.14 | 0.55 | 0.19 | 9 | 0.44 | 21.00 | 24.63 | 10 | 15.17 | 18.55 | 15.13 | 10 | 15.19 |
| **VIN LOAEL^α^** | 2.01 | 1.43 | 10 | 1.83 | 0.62 | 0.19 | 10 | 0.58 | **26.66**** | 15.99 | 10 | 24.74 | 13.36 | 10.48 | 10 | 11.98 |
| **FLT ADI^γ^** | 2.42 | 1.36 | 10 | 2.00 | 0.45 | 0.12 | 10 | 0.41 | 28.05 | 26.12 | 10 | 16.89 |  |  |  |  |
| **FLT NOAEL^β^** | 1.07 | 0.46 | 8 | 1.13 |  |  |  |  | 16.94 | 7.00 | 8 | 17.14 | 21.51 | 7.58 | 8 | 22.82 |
| **FLT LOAEL^α^** | 1.80 | 0.61 | 10 | 1.67 | 0.42 | 0.08 | 10 | 0.40 | **18.22**** | 4.42 | 10 | 17.35 | 14.08 | 12.52 | 10 | 10.45 |
| **PRO ADI^α^** | 1.83 | 0.50 | 10 | 1.75 | 0.42 | 0.07 | 10 | 0.39 | **15.27*** | 5.13 | 10 | 14.25 | 15.24 | 8.70 | 10 | 19.05 |
| **PRO NOAEL^α^** | 1.83 | 0.79 | 10 | 1.87 | **0.71*** | 0.27 | 10 | 0.71 | **22.53**** | 14.79 | 10 | 17.75 | 11.86 | 9.29 | 10 | 11.30 |
| **PRO LOAEL^α^** | 4.23 | 3.01 | 10 | 3.18 | **0.82**** | 0.39 | 10 | 0.67 | **37.93**** | 40.10 | 10 | 20.07 | 9.29 | 6.14 | 10 | 9.19 |
| **ADI MIX^γ^** | 1.61 | 0.89 | 10 | 1.39 | 0.39 | 0.07 | 10 | 0.35 | 20.25 | 28.40 | 10 | 9.83 | 21.90 | 6.05 | 10 | 21.38 |
| **NOAEL MIX^γ^** | 1.86 | 2.54 | 10 | 0.81 | 0.46 | 0.34 | 10 | 0.35 | 33.66 | 51.53 | 10 | 13.28 | 17.00 | 8.38 | 10 | 15.88 |
| **LOAEL MIX^γ^** | 6.29 | 7.58 | 10 | 2.52 | 0.88 | 0.79 | 10 | 0.46 | 58.93 | 70.64 | 10 | 18.29 | 18.12 | 6.72 | 10 | 19.05 |

Supplementary Table 37: Serum hormone levels in PND 83 females after single-substance and mixed exposures to anti-androgens

| **Treatment** | **11-Deoxycorticosterone (nmol/L)** | | | | **Corticosterone (nmol/L)** | | | | **Cortisol (nmol/L)** | | | |
| --- | --- | --- | --- | --- | --- | --- | --- | --- | --- | --- | --- | --- |
|  | **Mean** | **SD** | **N** | **Median** | **Mean** | **SD** | **N** | **Median** | **Mean** | **SD** | **N** | **Median** |
| **Vehicle Control^α^** | 5.98 | 5.89 | 10 | 3.81 | 697.1 | 356.6 | 10 | 645.9 |  |  |  |  |
| **Vehicle Control^β^** | 9.36 | 8.71 | 10 | 7.68 | 662.0 | 451.2 | 10 | 543.3 |  |  |  |  |
| **Vehicle Control^γ^** | 4.90 | 4.62 | 9 | 1.73 | 460.1 | 491.3 | 9 | 225.7 |  |  |  |  |
| **Positive Control^α^** | **16.41*** | 9.20 | 9 | 15.85 | **1399.7*** | 607.9 | 9 | 1312.1 |  |  |  |  |
| **VIN ADI^α^** | 7.87 | 4.93 | 8 | 7.65 | 901.0 | 378.1 | 8 | 767.3 |  |  |  |  |
| **VIN NOAEL^α^** | 9.31 | 6.66 | 10 | 8.24 | 954.3 | 543.0 | 10 | 776.0 |  |  |  |  |
| **VIN LOAEL^α^** | 13.34 | 9.64 | 10 | 11.47 | 1083.6 | 476.6 | 10 | 1156.0 |  |  |  |  |
| **FLT ADI^γ^** | **17.53**** | 10.18 | 10 | 14.74 | **1109.5**** | 343.4 | 10 | 1158.9 |  |  |  |  |
| **FLT NOAEL^β^** | 15.43 | 9.81 | 8 | 17.47 | 908.8 | 596.2 | 8 | 1021.6 |  |  |  |  |
| **FLT LOAEL^α^** | **17.20**** | 5.02 | 10 | 14.99 | **1411.5**** | 308.6 | 10 | 1437.8 |  |  |  |  |
| **PRO ADI^α^** | **16.00**** | 8.53 | 10 | 12.97 | **1389.5**** | 505.9 | 10 | 1512.9 |  |  |  |  |
| **PRO NOAEL^α^** | **17.80*** | 14.24 | 10 | 14.37 | **1295.1*** | 634.7 | 10 | 1369.9 |  |  |  |  |
| **PRO LOAEL^α^** | **13.60*** | 10.58 | 10 | 11.62 | 1057.8 | 581.4 | 10 | 962.4 |  |  |  |  |
| **ADI MIX^γ^** | 10.22 | 13.79 | 10 | 3.66 | 701.2 | 609.7 | 10 | 354.0 |  |  |  |  |
| **NOAEL MIX^γ^** | 9.32 | 7.43 | 10 | 5.57 | 750.6 | 540.8 | 10 | 682.0 |  |  |  |  |
| **LOAEL MIX^γ^** | 14.83 | 14.16 | 10 | 11.69 | **1083.2**** | 488.0 | 10 | 1170.5 |  |  |  |  |

Supplementary Table 38: The interaction index with 95%confifidence interval for the weight of ventral prostate. The dose values (d) of individual chemicals resulting in the same effect in the mixture experiment were estimated using inverse regression model.

|  |  | **Dose in Mixture (d)** | **Dose in Single-Substance (D)** | **d/D** | **τ with 95% confidence interval** |
| --- | --- | --- | --- | --- | --- |
|  |  | [mg/kg bw/day] | [mg/kg bw/day] |  |  |
| Subset 1 | Vinclozolin | 20 | 46.50 | 0.430 | 0.589 [0.108, 1.069] |
|  | Flutamide | 0.25 | 2.78 | 0.090 |  |
|  | Prochloraz | 30 | 437.80 | 0.069 |  |
| Subset 2 | Vinclozolin | 20 | 12.97 | 1.542 | 4.665 [0.541, 8.790] |
|  | Flutamide | 0.25 | 49.71 | 0.005 |  |
|  | Prochloraz | 30 | 9.62 | 3.118 |  |
| Subset 3 | Vinclozolin | 20 | 36.87 | 0.542 | 1.191 [0.852, 1.531] |
|  | Flutamide | 0.25 | 1.39 | 0.179 |  |
|  | Prochloraz | 30 | 63.87 | 0.470 |  |

Supplementary Table 39: The interaction index with 95%confifidence interval for day of preputial separation. The dose values (d) of individual chemicals resulting in the same effect in the mixture experiment were estimated using inverse regression model.

|  | **Dose in Mixture (d)**  [mg/kg bw/day] | **Dose in Single-Substance (D)** [mg/kg bw/day] | **d/D** | **τ with 95% confidence interval** |
| --- | --- | --- | --- | --- |
| Vinclozolin | 20 | 71.58 | 0.279 | 0.772 [0.569, 0.976] |
| Flutamide | 0.25 | 1.72 | 0.145 |
| Prochloraz | 30 | 86.24 | 0.348 |
