# Supplementary material for: Investigations on the dose–response relationship of combined exposure to low doses of three anti-androgens in Wistar rats
Source: Arch Toxicol. 2017 Sep 6;91(12):3961–89. doi: 10.1007/s00204-017-2053-3 (PMC5719133; doi:10.1007/s00204-017-2053-3)
Supplement: Supplementary file 2 — Supplementary material 2 (DOCX 1000 kb) [file 204_2017_2053_MOESM2_ESM.docx]

Supplementary Figure 31: Dose-response curve fits of the weight of ventral prostate for the individual chemicals and mixture experiment.


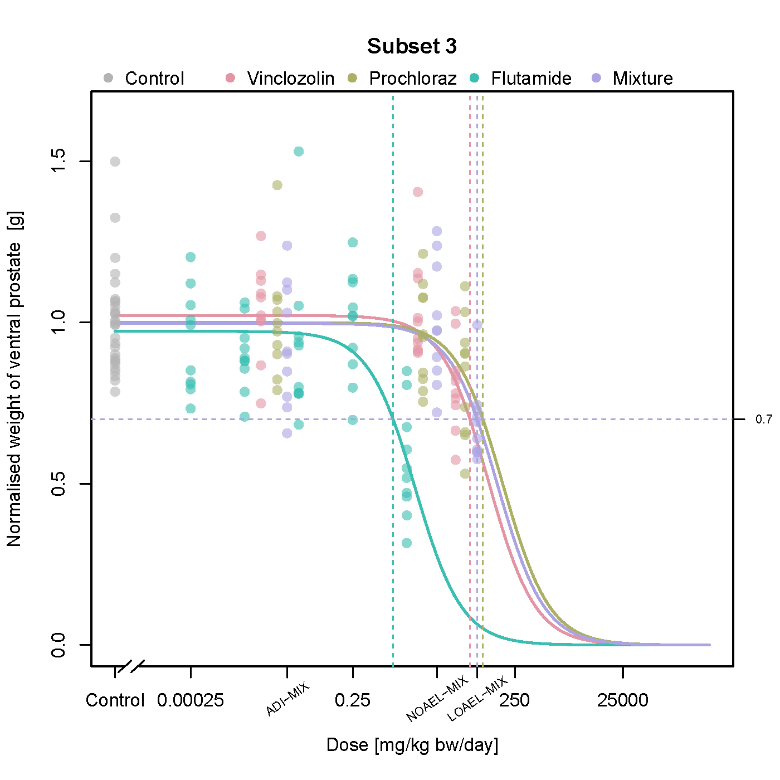

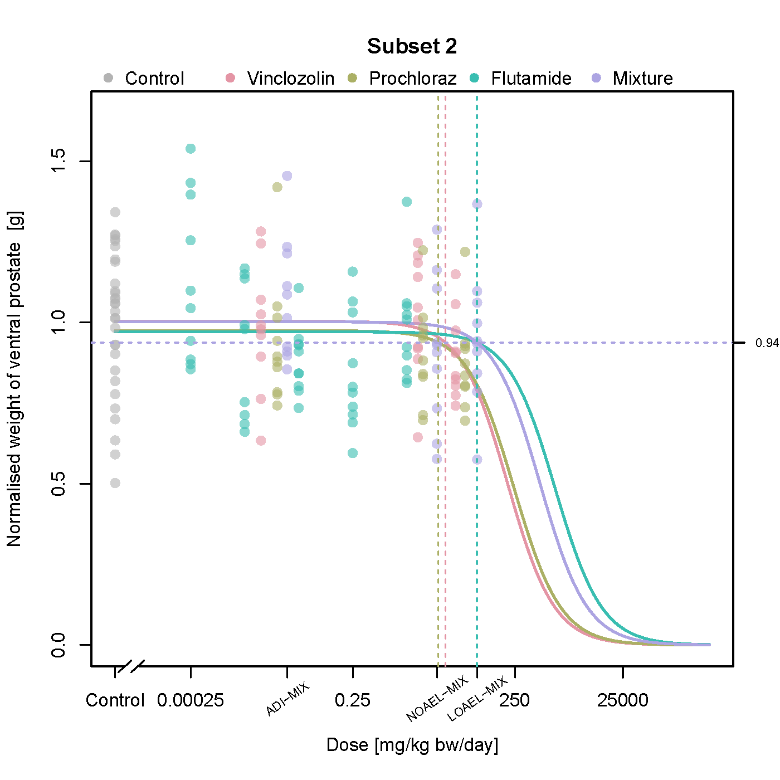


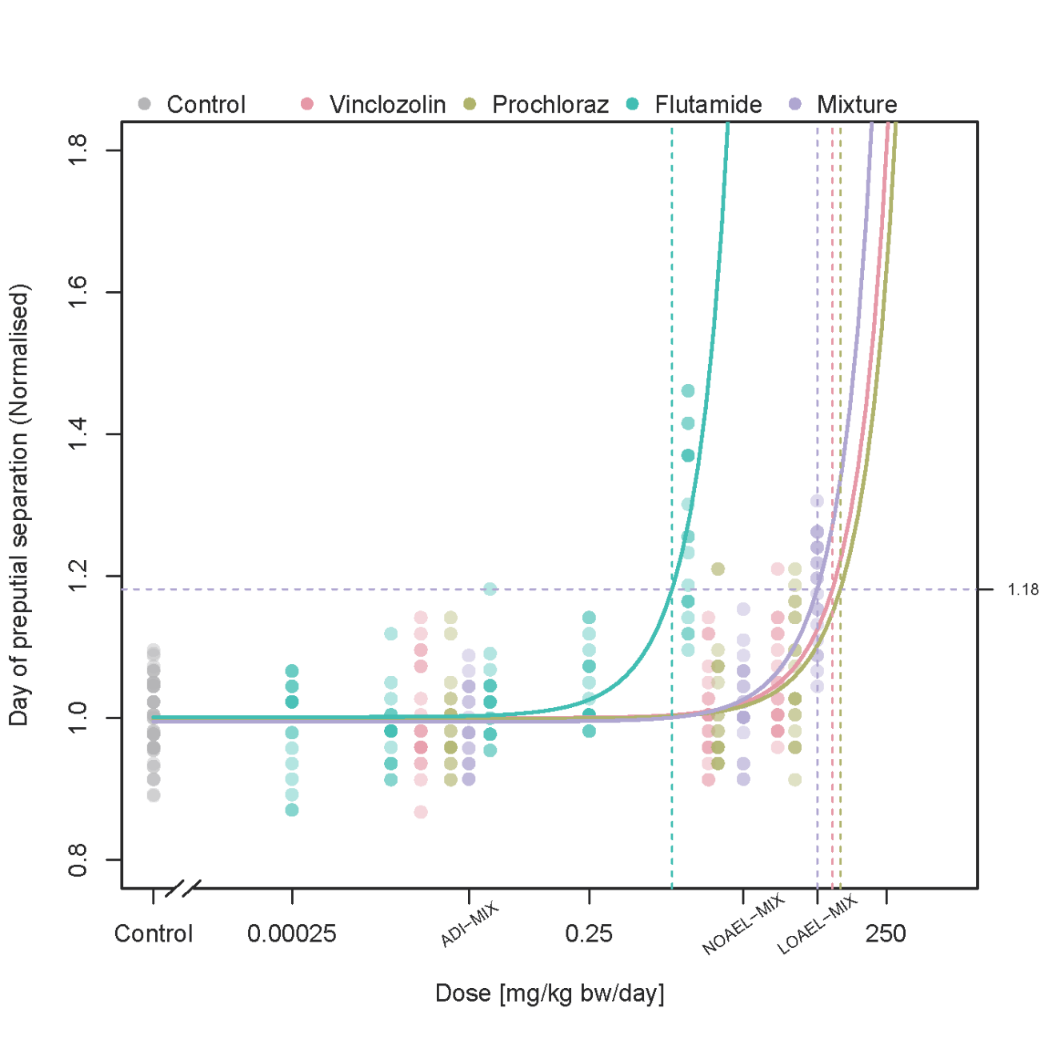


Supplementary Figure 31: Dose-response curve fits of day of preputial separation for the individual chemicals and mixture experiment.
