# Supplementary material for: Investigations on the dose–response relationship of combined exposure to low doses of three anti-androgens in Wistar rats
Source: Arch Toxicol. 2017 Sep 6;91(12):3961–89. doi: 10.1007/s00204-017-2053-3 (PMC5719133; doi:10.1007/s00204-017-2053-3)
Supplement: Supplementary file 3 — Supplementary material 3 (DOCX 14 kb) [file 204_2017_2053_MOESM3_ESM.docx]

Supplementary Table 38: The interaction index with 95%confifidence interval for the weight of ventral prostate. The dose values (d) of individual chemicals resulting in the same effect in the mixture experiment were estimated using inverse regression model.

|  |  | **Dose in Mixture (d)** | **Dose in Single-Substance (D)** | **d/D** | **τ with 95% confidence interval** |
| --- | --- | --- | --- | --- | --- |
|  |  | [mg/kg bw/day] | [mg/kg bw/day] |  |  |
| Subset 1 | Vinclozolin | 20 | 46.50 | 0.430 | 0.589 [0.108, 1.069] |
|  | Flutamide | 0.25 | 2.78 | 0.090 |  |
|  | Prochloraz | 30 | 437.80 | 0.069 |  |
| Subset 2 | Vinclozolin | 20 | 12.97 | 1.542 | 4.665 [0.541, 8.790] |
|  | Flutamide | 0.25 | 49.71 | 0.005 |  |
|  | Prochloraz | 30 | 9.62 | 3.118 |  |
| Subset 3 | Vinclozolin | 20 | 36.87 | 0.542 | 1.191 [0.852, 1.531] |
|  | Flutamide | 0.25 | 1.39 | 0.179 |  |
|  | Prochloraz | 30 | 63.87 | 0.470 |  |
