# Supplementary material for: Investigations on the dose–response relationship of combined exposure to low doses of three anti-androgens in Wistar rats
Source: Arch Toxicol. 2017 Sep 6;91(12):3961–89. doi: 10.1007/s00204-017-2053-3 (PMC5719133; doi:10.1007/s00204-017-2053-3)
Supplement: Supplementary file 4 — Supplementary material 4 (DOCX 13 kb) [file 204_2017_2053_MOESM4_ESM.docx]

Supplementary Table 39: The interaction index with 95%confifidence interval for day of preputial separation. The dose values (d) of individual chemicals resulting in the same effect in the mixture experiment were estimated using inverse regression model.

|  | **Dose in Mixture (d)**  [mg/kg bw/day] | **Dose in Single-Substance (D)** [mg/kg bw/day] | **d/D** | **τ with 95% confidence interval** |
| --- | --- | --- | --- | --- |
| Vinclozolin | 20 | 71.58 | 0.279 | 0.772 [0.569, 0.976] |
| Flutamide | 0.25 | 1.72 | 0.145 |  |
| Prochloraz | 30 | 86.24 | 0.348 |  |
