# Supplementary material for: Investigations on the dose–response relationship of combined exposure to low doses of three anti-androgens in Wistar rats
Source: Arch Toxicol. 2017 Sep 6;91(12):3961–89. doi: 10.1007/s00204-017-2053-3 (PMC5719133; doi:10.1007/s00204-017-2053-3)
Supplement: Supplementary file 5 — Supplementary material 5 (PDF 14858 kb) [file 204_2017_2053_MOESM5_ESM.pdf]

PR.NO.60R0375/88R002: REPRODUCTIVE TOX. STUDY TO DETECT EFFECTS
OF MIXED ANTI-ANDROGENIC SUBSTANCES IN RATS; ORAL ADM. (GAVAGE)
INDIVIDUAL MATERNAL CLINICAL OBSERVATIONS DURING GESTATION

TEST GROUP 0 (0 MG/KG BW/D)

FEMALE#	OBSERVATIONS / DAY OF GESTATION	0	1	2	3	4	5	6	7	8	9	10	11	12	13	14	15	16	17	18	19	20	21	22	23	24	25
1	NOTHING ABNORMAL DETECTED	P	P	P	P	P	P	P	P	P	P	P	P	P	P	P	P	P	P	P	P	P	P				
2	NOTHING ABNORMAL DETECTED	P	P	P	P	P	P	P	P	P	P	P	P	P	P	P	P	P	P	P	P	P	P				
3	NOTHING ABNORMAL DETECTED	P	P	P	P	P	P	P	P	P	P	P	P	P	P	P	P	P	P	P	P	P	P				
4	NOTHING ABNORMAL DETECTED	P	P	P	P	P	P	P	P	P	P	P	P	P	P	P	P	P	P	P	P	P	P				
5	NOTHING ABNORMAL DETECTED	P	P	P	P	P	P	P	P	P	P	P	P	P	P	P	P	P	P	P	P	P	P				
6	NOTHING ABNORMAL DETECTED	P	P	P	P	P	P	P	P	P	P	P	P	P	P	P	P	P	P	P	P	P	P				
7	NOTHING ABNORMAL DETECTED	P	P	P	P	P	P	P	P	P	P	P	P	P	P	P	P	P	P	P	P	P	P				
8	NOTHING ABNORMAL DETECTED	P	P	P	P	P	P	P	P	P	P	P	P	P	P	P	P	P	P	P	P	P	P				
9	NOTHING ABNORMAL DETECTED	P	P	P	P	P	P	P	P	P	P	P	P	P	P	P	P	P	P	P	P	P	P				
10	NOTHING ABNORMAL DETECTED	P	P	P	P	P	P	P	P	P	P	P	P	P	P	P	P	P	P	P	P	P	P				
11	NOTHING ABNORMAL DETECTED	P	P	P	P	P	P	P	P	P	P	P	P	P	P	P	P	P	P	P	P	P	P				
12	NOTHING ABNORMAL DETECTED	P	P	P	P	P	P	P	P	P	P	P	P	P	P	P	P	P	P	P	P	P					
	SCHEDULED SACRIFICE																					P					
13	NOTHING ABNORMAL DETECTED	P	P	P	P	P	P	P	P	P	P	P	P	P	P	P	P	P	P	P	P	P					
	SCHEDULED SACRIFICE																					P					
14	NOTHING ABNORMAL DETECTED	P	P	P	P	P	P	P	P	P	P	P	P	P	P	P	P	P	P	P	P	P					
	SCHEDULED SACRIFICE																					P					
15	NOTHING ABNORMAL DETECTED	P	P	P	P	P	P	P	P	P	P	P	P	P	P	P	P	P	P	P	P	P					
	SCHEDULED SACRIFICE																					P					
16	NOTHING ABNORMAL DETECTED	P	P	P	P	P	P	P	P	P	P	P	P	P	P	P	P	P	P	P	P	P					
	SCHEDULED SACRIFICE																					P					
17	NOTHING ABNORMAL DETECTED	P	P	P	P	P	P	P	P	P	P	P	P	P	P	P	P	P	P	P	P	P	P				
18	NOTHING ABNORMAL DETECTED	P	P	P	P	P	P	P	P	P	P	P	P	P	P	P	P	P	P	P	P	P	P				

CODE: 1-SLIGHT 2-MODERATE 3-MARKED P-PRESENT

PR.NO.60R0375/88R002: REPRODUCTIVE TOX. STUDY TO DETECT EFFECTS
OF MIXED ANTI-ANDROGENIC SUBSTANCES IN RATS; ORAL ADM. (GAVAGE)
INDIVIDUAL MATERNAL CLINICAL OBSERVATIONS DURING GESTATION

TEST GROUP 0 (0 MG/KG BW/D)

FEMALE#	OBSERVATIONS	DAY OF GESTATION: 0	1	2	3	4	5	6	7	8	9	10	11	12	13	14	15	16	17	18	19	20	21	22	23	24	25
19	NOTHING ABNORMAL DETECTED	P	P	P	P	P	P	P	P	P	P	P	P	P	P	P	P	P	P	P	P	P	P				
20	NOTHING ABNORMAL DETECTED	P	P	P	P	P	P	P	P	P	P	P	P	P	P	P	P	P	P	P	P	P	P				
21	NOTHING ABNORMAL DETECTED	P	P	P	P	P	P	P	P	P	P	P	P	P	P	P	P	P	P	P	P	P	P				
22	NOTHING ABNORMAL DETECTED	P	P	P	P	P	P	P	P	P	P	P	P	P	P	P	P	P	P	P	P	P					
23	NOTHING ABNORMAL DETECTED	P	P	P	P	P	P	P	P	P	P	P	P	P	P	P	P	P	P	P	P	P	P				
24	NOTHING ABNORMAL DETECTED	P	P	P	P	P	P	P	P	P	P	P	P	P	P	P	P	P	P	P	P	P	P				
25	NOTHING ABNORMAL DETECTED	P	P	P	P	P	P	P	P	P	P	P	P	P	P	P	P	P	P	P	P	P	P				

CODE: 1-SLIGHT 2-MODERATE 3-MARKED P-PRESENT

PR.NO.60R0375/88R002: REPRODUCTIVE TOX. STUDY TO DETECT EFFECTS
OF MIXED ANTI-ANDROGENIC SUBSTANCES IN RATS; ORAL ADM. (GAVAGE)
INDIVIDUAL MATERNAL CLINICAL OBSERVATIONS DURING GESTATION

TEST GROUP 1 (ADI-MIX)

FEMALE#	OBSERVATIONS	DAY OF GESTATION 0	1	2	3	4	5	6	7	8	9	10	11	12	13	14	15	16	17	18	19	20	21	22	23	24	25
26	NOTHING ABNORMAL DETECTED	P	P	P	P	P	P	P	P	P	P	P	P	P	P	P	P	P	P	P	P	P	P				
27	NOTHING ABNORMAL DETECTED	P	P	P	P	P	P	P	P	P	P	P	P	P	P	P	P	P	P	P	P	P	P				
28	NOTHING ABNORMAL DETECTED	P	P	P	P	P	P	P	P	P	P	P	P	P	P	P	P	P	P	P	P	P	P				
29	NOTHING ABNORMAL DETECTED	P	P	P	P	P	P	P	P	P	P	P	P	P	P	P	P	P	P	P	P	P	P				
30	NOTHING ABNORMAL DETECTED	P	P	P	P	P	P	P	P	P	P	P	P	P	P	P	P	P	P	P	P	P					
31	NOTHING ABNORMAL DETECTED	P	P	P	P	P	P	P	P	P	P	P	P	P	P	P	P	P	P	P	P	P	P				
32	NOTHING ABNORMAL DETECTED	P	P	P	P	P	P	P	P	P	P	P	P	P	P	P	P	P	P	P	P	P	P				
33	NOTHING ABNORMAL DETECTED	P	P	P	P	P	P	P	P	P	P	P	P	P	P	P	P	P	P	P	P	P	P				
34	NOTHING ABNORMAL DETECTED	P	P	P	P	P	P	P	P	P	P	P	P	P	P	P	P	P	P	P	P	P	P				
35	NOTHING ABNORMAL DETECTED	P	P	P	P	P	P	P	P	P	P	P	P	P	P	P	P	P	P	P	P	P	P				
36	NOTHING ABNORMAL DETECTED	P	P	P	P	P	P	P	P	P	P	P	P	P	P	P	P	P	P	P	P	P	P				
37	NOTHING ABNORMAL DETECTED	P	P	P	P	P	P	P	P	P	P	P	P	P	P	P	P	P	P	P	P	P					
38	NOTHING ABNORMAL DETECTED	P	P	P	P	P	P	P	P	P	P	P	P	P	P	P	P	P	P	P	P	P					
	SCHEDULED SACRIFICE																					P					
39	NOTHING ABNORMAL DETECTED	P	P	P	P	P	P	P	P	P	P	P	P	P	P	P	P	P	P	P	P	P					
	SCHEDULED SACRIFICE																					P					
40	NOTHING ABNORMAL DETECTED	P	P	P	P	P	P	P	P	P	P	P	P	P	P	P	P	P	P	P	P	P					
	SCHEDULED SACRIFICE																					P					
41	NOTHING ABNORMAL DETECTED	P	P	P	P	P	P	P	P	P	P	P	P	P	P	P	P	P	P	P	P	P					
	SCHEDULED SACRIFICE																					P					
42	NOTHING ABNORMAL DETECTED	P	P	P	P	P	P	P	P	P	P	P	P	P	P	P	P	P	P	P	P	P					
	SCHEDULED SACRIFICE																					P					
43	NOTHING ABNORMAL DETECTED	P	P	P	P	P	P	P	P	P	P	P	P	P	P	P	P	P	P	P	P	P	P				

CODE: 1-SLIGHT 2-MODERATE 3-MARKED P-PRESENT

PR.NO.60R0375/88R002: REPRODUCTIVE TOX. STUDY TO DETECT EFFECTS
OF MIXED ANTI-ANDROGENIC SUBSTANCES IN RATS; ORAL ADM. (GAVAGE)
INDIVIDUAL MATERNAL CLINICAL OBSERVATIONS DURING GESTATION

TEST GROUP 1 (ADI-MIX)

FEMALE#	OBSERVATIONS	DAY OF GESTATION: 0	1	2	3	4	5	6	7	8	9	10	11	12	13	14	15	16	17	18	19	20	21	22	23	24	25
44	NOTHING ABNORMAL DETECTED	P	P	P	P	P	P	P	P	P	P	P	P	P	P	P	P	P	P	P	P	P	P				
45	NOTHING ABNORMAL DETECTED	P	P	P	P	P	P	P	P	P	P	P	P	P	P	P	P	P	P	P	P	P					
46	NOTHING ABNORMAL DETECTED	P	P	P	P	P	P	P	P	P	P	P	P	P	P	P	P	P	P	P	P	P					
47	NOTHING ABNORMAL DETECTED	P	P	P	P	P	P	P	P	P	P	P	P	P	P	P	P	P	P	P	P	P	P				
48	NOTHING ABNORMAL DETECTED	P	P	P	P	P	P	P	P	P	P	P	P	P	P	P	P	P	P	P	P	P					
49	NOTHING ABNORMAL DETECTED	P	P	P	P	P	P	P	P	P	P	P	P	P	P	P	P	P	P	P	P	P	P				
50	NOTHING ABNORMAL DETECTED	P	P	P	P	P	P	P	P	P	P	P	P	P	P	P	P	P	P	P	P	P	P				

CODE: 1-SLIGHT 2-MODERATE 3-MARKED P-PRESENT

22-AUG-13

88R002 TABLE : IIA- 005

PR.NO.60R0375/88R002: REPRODUCTIVE TOX. STUDY TO DETECT EFFECTS
OF MIXED ANTI-ANDROGENIC SUBSTANCES IN RATS; ORAL ADM. (GAVAGE)
INDIVIDUAL MATERNAL CLINICAL OBSERVATIONS DURING GESTATION

TEST GROUP 2 (NOAEL-MIX)

FEMALE#	OBSERVATIONS	DAY OF GESTATION	0	1	2	3	4	5	6	7	8	9	10	11	12	13	14	15	16	17	18	19	20	21	22	23	24	25
51	NOTHING ABNORMAL DETECTED		P	P	P	P	P	P	P	P	P	P	P	P	P	P	P	P	P	P	P	P	P	P				
52	NOTHING ABNORMAL DETECTED		P	P	P	P	P	P	P	P	P	P	P	P	P	P	P	P	P	P	P	P	P	P				
53	NOTHING ABNORMAL DETECTED		P	P	P	P	P	P	P	P	P	P	P	P	P	P	P	P	P	P	P	P	P	P				
54	NOTHING ABNORMAL DETECTED		P	P	P	P	P	P	P	P	P	P	P	P	P	P	P	P	P	P	P	P	P	P				
55	NOTHING ABNORMAL DETECTED		P	P	P	P	P	P	P	P	P	P	P	P	P	P	P	P	P	P	P	P	P	P				
56	NOTHING ABNORMAL DETECTED		P	P	P	P	P	P	P	P	P	P	P	P	P	P	P	P	P	P	P	P	P	P	P			
57	NOTHING ABNORMAL DETECTED		P	P	P	P	P	P	P	P	P	P	P	P	P	P	P	P	P	P	P	P	P	P				
58	NOTHING ABNORMAL DETECTED		P	P	P	P	P	P	P	P	P	P	P	P	P	P	P	P	P	P	P	P	P	P	P			
59	NOTHING ABNORMAL DETECTED		P	P	P	P	P	P	P	P	P	P	P	P	P	P	P	P	P		P	P	P	P				
	SALIVATION AFTER TREATMENT																			P								
60	NOTHING ABNORMAL DETECTED		P	P	P	P	P	P	P	P	P	P	P	P	P	P	P	P			P	P						
	SALIVATION AFTER TREATMENT																		P	P			P	P				
61	NOTHING ABNORMAL DETECTED		P	P	P	P	P	P	P	P	P	P	P	P	P	P	P	P	P	P	P	P	P	P				
62	NOTHING ABNORMAL DETECTED		P	P	P	P	P	P	P	P	P	P	P	P	P	P	P	P	P	P	P	P	P	P	P			
63	NOTHING ABNORMAL DETECTED		P	P	P	P	P	P	P	P	P	P	P	P	P	P	P	P	P	P	P	P	P					
	SCHEDULED SACRIFICE																						P					
64	NOTHING ABNORMAL DETECTED		P	P	P	P	P	P	P	P	P	P	P	P	P	P	P	P	P	P	P	P	P					
	SCHEDULED SACRIFICE																						P					
65	NOTHING ABNORMAL DETECTED		P	P	P	P	P	P	P	P	P	P	P	P	P	P	P	P	P	P	P	P	P					
	SCHEDULED SACRIFICE																						P					
66	NOTHING ABNORMAL DETECTED		P	P	P	P	P	P	P	P	P	P	P	P	P	P	P	P	P	P	P	P	P					
	SCHEDULED SACRIFICE																						P					
67	NOTHING ABNORMAL DETECTED		P	P	P	P	P	P	P	P	P	P	P	P	P	P	P	P	P	P	P	P	P					
	SCHEDULED SACRIFICE																						P					

CODE: 1-SLIGHT 2-MODERATE 3-MARKED P-PRESENT

PR.NO.60R0375/88R002: REPRODUCTIVE TOX. STUDY TO DETECT EFFECTS
OF MIXED ANTI-ANDROGENIC SUBSTANCES IN RATS; ORAL ADM. (GAVAGE)
INDIVIDUAL MATERNAL CLINICAL OBSERVATIONS DURING GESTATION

TEST GROUP 2 (NOAEL-MIX)

FEMALE#	OBSERVATIONS	DAY OF GESTATION 0	1	2	3	4	5	6	7	8	9	10	11	12	13	14	15	16	17	18	19	20	21	22	23	24	25
68	NOTHING ABNORMAL DETECTED	P	P	P	P	P	P	P	P	P	P	P	P	P	P	P	P	P	P	P	P	P	P				
69	NOTHING ABNORMAL DETECTED	P	P	P	P	P	P	P	P	P	P	P	P	P	P	P	P	P	P	P	P	P	P				
70	NOTHING ABNORMAL DETECTED	P	P	P	P	P	P	P	P	P	P	P	P	P	P	P	P	P	P	P	P	P	P	P			
71	NOTHING ABNORMAL DETECTED	P	P	P	P	P	P	P	P	P	P	P	P	P	P	P	P	P	P	P	P	P	P				
72	NOTHING ABNORMAL DETECTED	P	P	P	P	P	P	P	P	P	P	P	P	P	P	P	P	P	P	P	P	P	P				
73	NOTHING ABNORMAL DETECTED	P	P	P	P	P	P	P	P	P	P	P	P	P	P	P	P	P	P	P	P	P	P	P			
74	NOTHING ABNORMAL DETECTED	P	P	P	P	P	P	P	P	P	P	P	P	P	P	P	P	P	P	P	P	P	P	P			
75	NOTHING ABNORMAL DETECTED	P	P	P	P	P	P	P	P	P	P	P	P	P	P	P	P	P	P	P	P	P	P				

CODE: 1-SLIGHT 2-MODERATE 3-MARKED P-PRESENT

PR.NO.60R0375/88R002: REPRODUCTIVE TOX. STUDY TO DETECT EFFECTS
OF MIXED ANTI-ANDROGENIC SUBSTANCES IN RATS; ORAL ADM. (GAVAGE)
INDIVIDUAL MATERNAL CLINICAL OBSERVATIONS DURING GESTATION

TEST GROUP 3 (LOAEL-MIX)

FEMALE#	OBSERVATIONS	DAY OF GESTATION: 0	1	2	3	4	5	6	7	8	9	10	11	12	13	14	15	16	17	18	19	20	21	22	23	24	25
76	NOTHING ABNORMAL DETECTED	P	P	P	P	P	P	P	P	P	P	P	P	P			P	P		P		P	P				
	ABDOMINAL POSITION																								P		
	HYPOTHERMIA																								P		
	SALIVATION AFTER TREATMENT														P	P			P		P			P			
	PILOERECTION																								P		
	UNABLE TO DELIVER																								P		
	SACRIFICED MORIBUND																								P		
77	NOTHING ABNORMAL DETECTED	P	P	P	P	P	P	P	P	P	P	P	P	P	P	P		P	P								
	SALIVATION AFTER TREATMENT																P			P	P	P	P				
78	NOTHING ABNORMAL DETECTED	P	P	P	P	P	P	P	P	P	P	P	P	P		P			P	P		P	P	P			
	SALIVATION AFTER TREATMENT														P		P	P			P						
79	NOTHING ABNORMAL DETECTED	P	P	P	P	P	P	P	P	P	P	P	P	P		P				P							
	SALIVATION AFTER TREATMENT														P		P	P	P		P	P	P	P	P		
80	NOTHING ABNORMAL DETECTED	P	P	P	P	P	P	P	P	P	P	P	P	P	P	P	P	P									
	SALIVATION AFTER TREATMENT																		P	P	P	P	P	P			
81	NOTHING ABNORMAL DETECTED	P	P	P	P	P	P	P	P	P	P	P	P	P		P							P				
	SALIVATION AFTER TREATMENT														P		P	P	P	P	P	P		P			
82	NOTHING ABNORMAL DETECTED	P	P	P	P	P	P	P	P	P	P	P	P	P			P										
	SALIVATION AFTER TREATMENT														P	P		P	P	P	P	P	P	P			
83	NOTHING ABNORMAL DETECTED	P	P	P	P	P	P	P	P	P	P	P	P	P	P	P	P	P			P	P					
	SALIVATION AFTER TREATMENT																		P	P			P	P			
84	NOTHING ABNORMAL DETECTED	P	P	P	P	P	P	P	P	P	P	P	P	P	P					P	P	P					
	SALIVATION AFTER TREATMENT															P	P	P	P				P	P			
85	NOTHING ABNORMAL DETECTED	P	P	P	P	P	P	P	P	P	P	P	P	P	P	P	P	P	P		P	P	P	P			
	SALIVATION AFTER TREATMENT																			P							
86	NOTHING ABNORMAL DETECTED	P	P	P	P	P	P	P	P	P	P	P	P		P		P				P						
	SALIVATION AFTER TREATMENT													P		P		P	P	P		P	P	P			
87	NOTHING ABNORMAL DETECTED	P	P	P	P	P	P	P	P	P	P	P	P		P	P				P		P					
	SALIVATION AFTER TREATMENT													P			P	P	P		P		P	P			

CODE: 1-SLIGHT 2-MODERATE 3-MARKED P-PRESENT

22-AUG-13

88R002 TABLE : IIA- 008

PR.NO.60R0375/88R002: REPRODUCTIVE TOX. STUDY TO DETECT EFFECTS
OF MIXED ANTI-ANDROGENIC SUBSTANCES IN RATS; ORAL ADM. (GAVAGE)
INDIVIDUAL MATERNAL CLINICAL OBSERVATIONS DURING GESTATION

TEST GROUP 3 (LOAEL-MIX)

FEMALE#	OBSERVATIONS	DAY OF GESTATION: 0	1	2	3	4	5	6	7	8	9	10	11	12	13	14	15	16	17	18	19	20	21	22	23	24	25
88	NOTHING ABNORMAL DETECTED	P	P	P	P	P	P	P	P	P	P	P	P	P	P		P	P	P		P						
	SALIVATION AFTER TREATMENT															P				P		P					
	SCHEDULED SACRIFICE																					P					
89	NOTHING ABNORMAL DETECTED	P	P	P	P	P	P	P	P	P	P	P	P	P	P	P	P	P	P		P	P					
	SALIVATION AFTER TREATMENT																			P							
	SCHEDULED SACRIFICE																					P					
90	NOTHING ABNORMAL DETECTED	P	P	P	P	P	P	P	P	P	P	P	P	P		P						P					
	SALIVATION AFTER TREATMENT														P		P	P	P	P	P						
	SCHEDULED SACRIFICE																					P					
91	NOTHING ABNORMAL DETECTED	P	P	P	P	P	P	P	P	P	P	P	P	P													
	SALIVATION AFTER TREATMENT														P	P	P	P	P	P	P	P					
	SCHEDULED SACRIFICE																					P					
92	NOTHING ABNORMAL DETECTED	P	P	P	P	P	P	P	P	P	P	P		P						P	P						
	SALIVATION AFTER TREATMENT												P		P	P	P	P	P			P					
	SCHEDULED SACRIFICE																					P					
93	NOTHING ABNORMAL DETECTED	P	P	P	P	P	P	P	P	P	P	P	P	P						P							
	SALIVATION AFTER TREATMENT														P	P	P	P	P		P	P	P	P			
94	NOTHING ABNORMAL DETECTED	P	P	P	P	P	P	P	P	P	P	P	P	P	P			P		P	P	P					
	SALIVATION AFTER TREATMENT															P	P		P				P	P			
95	NOTHING ABNORMAL DETECTED	P	P	P	P	P	P	P	P	P	P	P	P							P							
	SALIVATION AFTER TREATMENT													P	P	P	P	P	P		P	P	P				
96	NOTHING ABNORMAL DETECTED	P	P	P	P	P	P	P	P	P	P	P	P	P						P	P	P					
	SALIVATION AFTER TREATMENT														P	P	P	P	P				P	P			
97	NOTHING ABNORMAL DETECTED	P	P	P	P	P	P	P	P	P	P	P		P	P	P	P			P	P	P		P			
	SALIVATION AFTER TREATMENT												P					P	P				P				
98	NOTHING ABNORMAL DETECTED	P	P	P	P	P	P	P	P	P	P	P							P			P					
	SALIVATION AFTER TREATMENT												P	P	P	P	P	P		P	P		P	P			
99	NOTHING ABNORMAL DETECTED	P	P	P	P	P	P	P	P	P	P	P	P		P		P			P	P		P	P			
	SALIVATION AFTER TREATMENT													P		P		P	P			P					

CODE: 1-SLIGHT 2-MODERATE 3-MARKED P-PRESENT

22-AUG-13

88R002 | TABLE : IIA- | 009

PR.NO.60R0375/88R002: REPRODUCTIVE TOX. STUDY TO DETECT EFFECTS
OF MIXED ANTI-ANDROGENIC SUBSTANCES IN RATS; ORAL ADM. (GAVAGE)
INDIVIDUAL MATERNAL CLINICAL OBSERVATIONS DURING GESTATION

TEST GROUP 3 (LOAEL-MIX)

FEMALE#	OBSERVATIONS	DAY OF GESTATION	0	1	2	3	4	5	6	7	8	9	10	11	12	13	14	15	16	17	18	19	20	21	22	23	24	25
100	NOTHING ABNORMAL DETECTED		P	P	P	P	P	P	P	P	P	P	P		P													
	SALIVATION AFTER TREATMENT													P		P	P	P	P	P	P	P	P	P	P			

CODE: 1-SLIGHT 2-MODERATE 3-MARKED P-PRESENT

PR.NO.60R0375/88R002: REPRODUCTIVE TOX. STUDY TO DETECT EFFECTS
OF MIXED ANTI-ANDROGENIC SUBSTANCES IN RATS; ORAL ADM. (GAVAGE)
INDIVIDUAL MATERNAL CLINICAL OBSERVATIONS DURING GESTATION

TEST GROUP 4(0.00025 MG/KG BW/D)

FEMALE#	OBSERVATIONS DAY OF GESTATION	0	1	2	3	4	5	6	7	8	9	10	11	12	13	14	15	16	17	18	19	20	21	22	23	24	25
101	NOTHING ABNORMAL DETECTED	P	P	P	P	P	P	P	P	P	P	P	P	P	P	P	P	P	P	P	P	P	P	P			
102	NOTHING ABNORMAL DETECTED	P	P	P	P	P	P	P	P	P	P	P	P	P	P	P	P	P	P	P	P	P	P				
103	NOTHING ABNORMAL DETECTED	P	P	P	P	P	P	P	P	P	P	P	P	P	P	P	P	P	P	P	P	P					
104	NOTHING ABNORMAL DETECTED	P	P	P	P	P	P	P	P	P	P	P	P	P	P	P	P	P	P	P	P	P	P				
105	NOTHING ABNORMAL DETECTED	P	P	P	P	P	P	P	P	P	P	P	P	P	P	P	P	P	P	P	P	P	P				
106	NOTHING ABNORMAL DETECTED	P	P	P	P	P	P	P	P	P	P	P	P	P	P	P	P	P	P	P	P	P	P				
107	NOTHING ABNORMAL DETECTED	P	P	P	P	P	P	P	P	P	P	P	P	P	P	P	P	P	P	P	P	P	P				
108	NOTHING ABNORMAL DETECTED	P	P	P	P	P	P	P	P	P	P	P	P	P	P	P	P	P	P	P	P	P	P				
109	NOTHING ABNORMAL DETECTED	P	P	P	P	P	P	P	P	P	P	P	P	P	P	P	P	P	P	P	P	P	P				
110	NOTHING ABNORMAL DETECTED	P	P	P	P	P	P	P	P	P	P	P	P	P	P	P	P	P	P	P	P	P	P				
111	NOTHING ABNORMAL DETECTED	P	P	P	P	P	P	P	P	P	P	P	P	P	P	P	P	P	P	P	P	P	P				
112	NOTHING ABNORMAL DETECTED	P	P	P	P	P	P	P	P	P	P	P	P	P	P	P	P	P	P	P	P	P					
	SCHEDULED SACRIFICE																					P					
113	NOTHING ABNORMAL DETECTED	P	P	P	P	P	P	P	P	P	P	P	P	P	P	P	P	P	P	P	P	P					
	SCHEDULED SACRIFICE																					P					
114	NOTHING ABNORMAL DETECTED	P	P	P	P	P	P	P	P	P	P	P	P	P	P	P	P	P	P	P	P	P					
	SCHEDULED SACRIFICE																					P					
115	NOTHING ABNORMAL DETECTED	P	P	P	P	P	P	P	P	P	P	P	P	P	P	P	P	P	P	P	P	P					
	SCHEDULED SACRIFICE																					P					
116	NOTHING ABNORMAL DETECTED	P	P	P	P	P	P	P	P	P	P	P	P	P	P	P	P	P	P	P	P	P					
	SCHEDULED SACRIFICE																					P					
117	NOTHING ABNORMAL DETECTED	P	P	P	P	P	P	P	P	P	P	P	P	P	P	P	P	P	P	P	P	P	P				
118	NOTHING ABNORMAL DETECTED	P	P	P	P	P	P	P	P	P	P	P	P	P	P	P	P	P	P	P	P	P	P				

CODE: 1-SLIGHT 2-MODERATE 3-MARKED P-PRESENT

PR.NO.60R0375/88R002: REPRODUCTIVE TOX. STUDY TO DETECT EFFECTS OF MIXED ANTI-ANDROGENIC SUBSTANCES IN RATS; ORAL ADM. (GAVAGE)

INDIVIDUAL MATERNAL CLINICAL OBSERVATIONS DURING GESTATION

TEST GROUP 4(0.00025 MG/KG BW/D)

FEMALE#	OBSERVATIONS	DAY OF GESTATION: 0	1	2	3	4	5	6	7	8	9	10	11	12	13	14	15	16	17	18	19	20	21	22	23	24	25
119	NOTHING ABNORMAL DETECTED	P	P	P	P	P	P	P	P	P	P	P	P	P	P	P	P	P	P	P	P	P	P	P			
120	NOTHING ABNORMAL DETECTED	P	P	P	P	P	P	P	P	P	P	P	P	P	P	P	P	P	P	P	P	P	P	P			
121	NOTHING ABNORMAL DETECTED	P	P	P	P	P	P	P	P	P	P	P	P	P	P	P	P	P	P	P	P	P					
122	NOTHING ABNORMAL DETECTED	P	P	P	P	P	P	P	P	P	P	P	P	P	P	P	P	P	P	P	P	P	P	P	P	P	
	NO PUPS DELIVERED (F1)																										P
	SCHEDULED SACRIFICE																										P
123	NOTHING ABNORMAL DETECTED	P	P	P	P	P	P	P	P	P	P	P	P	P	P	P	P	P	P	P	P	P	P				
124	NOTHING ABNORMAL DETECTED	P	P	P	P	P	P	P	P	P	P	P	P	P	P	P	P	P	P	P	P	P	P				
125	NOTHING ABNORMAL DETECTED	P	P	P	P	P	P	P	P	P	P	P	P	P	P	P	P	P	P	P	P	P	P				

CODE: 1-SLIGHT 2-MODERATE 3-MARKED P-PRESENT

PR.NO.60R0375/88R002: REPRODUCTIVE TOX. STUDY TO DETECT EFFECTS
OF MIXED ANTI-ANDROGENIC SUBSTANCES IN RATS; ORAL ADM. (GAVAGE)
INDIVIDUAL MATERNAL/PUP CLINICAL OBSERVATIONS DURING LACTATION

TEST GROUP 0 (0 MG/KG BW/D)

FEMALE#	OBSERVATIONS	DAY OF LACTATION 0	1	2	3	4	5	6	7	8	9	10	11	12	13	14	15	16	17	18	19	20	21	22	23	24	25
1	NOTHING ABNORMAL DETECTED	P	P	P	P	P	P	P	P	P	P	P	P	P	P	P	P	P	P	P	P	P	P	P	P	P	P
2	NOTHING ABNORMAL DETECTED	P	P	P	P	P	P	P	P	P	P	P	P	P	P	P	P	P	P	P	P	P	P	P	P	P	P
3	NOTHING ABNORMAL DETECTED	P	P	P	P	P	P	P	P	P	P	P	P	P	P	P	P	P	P	P	P	P	P	P	P	P	P
4	NOTHING ABNORMAL DETECTED	P	P	P	P	P	P	P	P	P	P	P	P	P	P	P	P	P	P	P	P	P	P	P	P	P	P
5	NOTHING ABNORMAL DETECTED	P	P	P	P	P	P	P	P	P	P	P	P	P	P	P	P	P	P	P	P	P	P	P	P	P	P
6	NOTHING ABNORMAL DETECTED	P	P	P	P	P	P	P	P	P	P	P	P	P	P	P	P	P	P	P	P	P	P	P	P	P	P
7	NOTHING ABNORMAL DETECTED	P	P	P	P	P	P	P	P	P	P	P	P	P	P	P	P	P	P	P	P	P	P	P	P	P	P
8	NOTHING ABNORMAL DETECTED	P	P	P	P	P	P	P	P	P	P	P	P	P	P	P	P	P	P	P	P	P	P	P	P	P	P
9	NOTHING ABNORMAL DETECTED	P	P	P	P	P	P	P	P	P	P	P	P	P	P	P	P	P	P	P	P	P	P	P	P	P	P
10	NOTHING ABNORMAL DETECTED	P	P	P	P	P	P	P	P	P	P	P	P	P	P	P	P	P	P	P	P	P	P	P	P	P	P
11	NOTHING ABNORMAL DETECTED	P	P	P	P	P	P	P	P	P	P	P	P	P	P	P	P	P	P	P	P	P	P	P	P	P	P
17	NOTHING ABNORMAL DETECTED	P	P	P	P	P	P	P	P	P	P	P	P	P	P	P	P	P	P	P	P	P	P	P	P	P	P
18	NOTHING ABNORMAL DETECTED	P	P	P	P	P	P	P	P	P	P	P	P	P	P	P	P	P	P	P	P	P	P	P	P	P	P
19	NOTHING ABNORMAL DETECTED	P	P	P	P	P	P	P	P	P	P	P	P	P	P	P	P	P	P	P	P	P	P	P	P	P	P
20	NOTHING ABNORMAL DETECTED	P	P	P	P	P	P	P	P	P	P	P	P	P	P	P	P	P	P	P	P	P	P	P	P	P	P
21	NOTHING ABNORMAL DETECTED	P	P	P	P	P	P	P	P	P	P	P	P	P	P	P	P	P	P	P	P	P	P	P	P	P	P
22	NOTHING ABNORMAL DETECTED	P	P	P	P	P	P	P	P	P	P	P	P	P	P	P	P	P	P	P	P	P	P	P	P	P	P
23	NOTHING ABNORMAL DETECTED	P	P	P	P	P	P	P	P	P	P	P	P	P	P	P	P	P	P	P	P	P	P	P	P	P	P
24	NOTHING ABNORMAL DETECTED	P	P	P	P	P	P	P	P	P	P	P	P	P	P	P	P	P	P	P	P	P	P	P	P	P	P
25	NOTHING ABNORMAL DETECTED	P	P	P	P	P	P	P	P	P	P	P	P	P	P	P	P	P	P	P	P	P	P	P	P	P	P

CODE: 1-SLIGHT 2-MODERATE 3-MARKED P-PRESENT

PR.NO.60R0375/88R002: REPRODUCTIVE TOX. STUDY TO DETECT EFFECTS
OF MIXED ANTI-ANDROGENIC SUBSTANCES IN RATS; ORAL ADM. (GAVAGE)
INDIVIDUAL MATERNAL/PUP CLINICAL OBSERVATIONS DURING LACTATION

TEST GROUP 1 (ADI-MIX)

FEMALE#	OBSERVATIONS	DAY OF LACTATION	0	1	2	3	4	5	6	7	8	9	10	11	12	13	14	15	16	17	18	19	20	21	22	23	24	25
26	NOTHING ABNORMAL DETECTED		P	P	P	P	P	P	P	P	P	P	P	P	P	P	P	P	P	P	P	P	P	P	P	P	P	P
27	NOTHING ABNORMAL DETECTED		P	P	P	P	P	P	P	P	P	P	P	P	P	P	P	P	P	P	P	P	P	P	P	P	P	P
28	NOTHING ABNORMAL DETECTED		P	P	P	P	P	P	P	P	P	P	P	P	P	P	P	P	P	P	P	P	P	P	P	P	P	P
29	NOTHING ABNORMAL DETECTED		P	P	P	P	P	P	P	P	P	P	P	P	P	P	P	P	P	P	P	P	P	P	P	P	P	P
30	NOTHING ABNORMAL DETECTED		P	P	P	P	P	P	P	P	P	P	P	P	P	P	P	P	P	P	P	P	P	P	P	P	P	P
31	NOTHING ABNORMAL DETECTED		P	P	P	P	P	P	P	P	P	P	P	P	P	P	P	P	P	P	P	P	P	P	P	P	P	P
32	NOTHING ABNORMAL DETECTED		P	P	P	P	P	P	P	P	P	P	P	P	P	P	P	P	P	P	P	P	P	P	P	P	P	P
33	NOTHING ABNORMAL DETECTED		P	P	P	P	P	P	P	P	P	P	P	P	P	P	P	P	P	P	P	P	P	P	P	P	P	P
34	NOTHING ABNORMAL DETECTED		P	P	P	P	P	P	P	P	P	P	P	P	P	P	P	P	P	P	P	P	P	P	P	P	P	P
35	NOTHING ABNORMAL DETECTED		P	P	P	P	P	P	P	P	P	P	P	P	P	P	P	P	P	P	P	P	P	P	P	P	P	P
36	NOTHING ABNORMAL DETECTED		P	P	P	P	P	P	P	P	P	P	P	P	P	P	P	P	P	P	P	P	P	P	P	P	P	P
37	NOTHING ABNORMAL DETECTED		P	P	P	P	P	P	P	P	P	P	P	P	P	P	P	P	P	P	P	P	P	P	P	P	P	P
43	NOTHING ABNORMAL DETECTED		P	P	P	P	P	P	P	P	P	P	P	P	P	P	P	P	P	P	P	P	P	P	P	P	P	P
44	NOTHING ABNORMAL DETECTED		P	P	P	P	P	P	P	P	P	P	P	P	P	P	P	P	P	P	P	P	P	P	P	P	P	P
45	NOTHING ABNORMAL DETECTED		P	P	P	P	P	P	P	P	P	P	P	P	P	P	P	P	P	P	P	P	P	P	P	P	P	P
46	NOTHING ABNORMAL DETECTED		P	P	P	P	P	P	P	P	P	P	P	P	P	P	P	P	P	P	P	P	P	P	P	P	P	P
47	NOTHING ABNORMAL DETECTED		P	P	P	P	P	P	P	P	P	P	P	P	P	P	P	P	P	P	P	P	P	P	P	P	P	P
48	NOTHING ABNORMAL DETECTED		P	P	P	P	P	P	P	P	P	P	P	P	P	P	P	P	P	P	P	P	P	P	P	P	P	P
49	NOTHING ABNORMAL DETECTED		P	P	P	P	P	P	P	P	P	P	P	P	P	P	P	P	P	P	P	P	P	P	P	P	P	P
50	NOTHING ABNORMAL DETECTED		P	P	P	P	P	P	P	P	P	P	P	P	P	P	P	P	P	P	P	P	P	P	P	P	P	P

CODE: 1-SLIGHT 2-MODERATE 3-MARKED P-PRESENT

PR.NO.60R0375/88R002: REPRODUCTIVE TOX. STUDY TO DETECT EFFECTS
OF MIXED ANTI-ANDROGENIC SUBSTANCES IN RATS; ORAL ADM. (GAVAGE)
INDIVIDUAL MATERNAL/PUP CLINICAL OBSERVATIONS DURING LACTATION

TEST GROUP 2 (NOAEL-MIX)

FEMALE#	OBSERVATIONS	0	1	2	3	4	5	6	7	8	9	10	11	12	13	14	15	16	17	18	19	20	21	22	23	24	25
51	NOTHING ABNORMAL DETECTED	P	P	P	P	P	P	P	P	P	P	P	P	P	P	P	P	P		P	P	P	P	P	P	P	P
	SALIVATION AFTER TREATMENT																		P								
52	NOTHING ABNORMAL DETECTED	P	P	P	P	P	P	P	P	P	P	P	P	P	P	P	P	P	P	P	P	P	P	P	P	P	P
53	NOTHING ABNORMAL DETECTED	P	P	P	P	P	P	P	P	P	P	P	P	P	P	P	P	P	P	P	P	P	P	P	P	P	P
54	NOTHING ABNORMAL DETECTED	P	P	P	P	P	P	P	P	P	P	P	P	P	P	P	P	P	P	P	P	P	P	P	P	P	P
55	NOTHING ABNORMAL DETECTED	P	P	P	P	P	P	P	P	P	P	P	P	P	P	P	P	P	P	P	P	P	P	P	P	P	P
56	NOTHING ABNORMAL DETECTED	P					P	P	P	P	P	P	P	P	P	P	P	P	P	P	P	P	P	P	P	P	P
	PUP(S) NOT PROPERLY NURSED (NO/LESS MILK IN STOMACH)		P	P	P	P																					
57	NOTHING ABNORMAL DETECTED	P	P	P	P	P	P	P	P	P	P	P	P	P	P	P	P	P	P	P	P	P	P	P	P	P	P
58	NOTHING ABNORMAL DETECTED	P	P	P	P	P	P	P	P	P	P	P	P	P	P	P	P	P	P	P	P	P	P	P	P	P	P
59	NOTHING ABNORMAL DETECTED	P	P	P	P	P	P	P	P	P	P	P	P	P	P	P	P	P	P	P	P	P	P	P	P	P	
	SALIVATION AFTER TREATMENT																										P
60	NOTHING ABNORMAL DETECTED	P	P	P	P	P	P	P	P	P	P	P			P	P	P	P	P		P			P			
	SALIVATION AFTER TREATMENT												P	P						P		P	P		P	P	P
61	NOTHING ABNORMAL DETECTED	P	P	P	P	P	P	P	P	P	P	P	P	P	P	P	P	P	P	P	P	P	P	P	P	P	P
62	NOTHING ABNORMAL DETECTED	P	P	P	P	P	P	P	P	P	P	P	P	P	P	P	P	P	P	P	P	P	P	P	P	P	P
68	NOTHING ABNORMAL DETECTED	P	P	P	P	P	P	P	P	P	P	P	P	P	P	P	P	P	P	P	P	P	P	P	P	P	P
69	NOTHING ABNORMAL DETECTED	P	P	P	P	P	P	P	P	P	P	P	P	P	P	P	P	P	P	P	P	P	P	P	P	P	P
70	NOTHING ABNORMAL DETECTED	P	P	P	P	P	P	P	P	P	P	P	P	P	P	P	P	P	P	P	P	P	P	P	P	P	P
71	NOTHING ABNORMAL DETECTED	P	P	P	P	P	P	P	P	P	P	P	P	P	P	P	P	P	P	P	P	P	P	P	P	P	P
72	NOTHING ABNORMAL DETECTED	P	P	P	P	P	P	P	P	P	P	P	P	P	P	P	P	P	P	P	P	P	P	P	P	P	P
73	NOTHING ABNORMAL DETECTED	P	P	P	P	P	P	P	P	P	P	P	P	P	P	P	P	P	P	P	P	P	P	P	P	P	P
74	NOTHING ABNORMAL DETECTED	P	P	P	P	P	P	P	P	P	P	P	P	P	P	P	P	P	P	P	P	P	P	P	P	P	P

CODE: 1-SLIGHT 2-MODERATE 3-MARKED P-PRESENT

PR.NO.60R0375/88R002: REPRODUCTIVE TOX. STUDY TO DETECT EFFECTS
OF MIXED ANTI-ANDROGENIC SUBSTANCES IN RATS; ORAL ADM. (GAVAGE)
INDIVIDUAL MATERNAL/PUP CLINICAL OBSERVATIONS DURING LACTATION

TEST GROUP 2 (NOAEL-MIX)

FEMALE#	OBSERVATIONS	DAY OF LACTATION	0	1	2	3	4	5	6	7	8	9	10	11	12	13	14	15	16	17	18	19	20	21	22	23	24	25
75	NOTHING ABNORMAL DETECTED		P	P	P	P	P	P	P	P	P	P	P	P	P	P	P	P	P	P	P	P	P	P	P	P	P	P

CODE: 1-SLIGHT 2-MODERATE 3-MARKED P-PRESENT

PR.NO.60R0375/88R002: REPRODUCTIVE TOX. STUDY TO DETECT EFFECTS
OF MIXED ANTI-ANDROGENIC SUBSTANCES IN RATS; ORAL ADM. (GAVAGE)
INDIVIDUAL MATERNAL/PUP CLINICAL OBSERVATIONS DURING LACTATION

TEST GROUP 3 (LOAEL-MIX)

FEMALE#	OBSERVATIONS	DAY OF LACTATION 0	1	2	3	4	5	6	7	8	9	10	11	12	13	14	15	16	17	18	19	20	21	22	23	24	25
77	SALIVATION AFTER TREATMENT	P	P	P	P	P	P	P	P	P	P	P	P	P	P	P	P	P	P	P	P	P	P	P	P	P	P
78	SALIVATION AFTER TREATMENT	P	P	P	P	P	P	P	P	P	P	P	P	P	P	P	P	P	P	P	P	P	P	P	P	P	P
79	SALIVATION AFTER TREATMENT	P	P	P	P	P	P	P	P	P	P	P	P	P	P	P	P	P	P	P	P	P	P	P	P	P	P
	ALL PUPS STILLBORN	P																									
80	SALIVATION AFTER TREATMENT	P	P	P	P	P	P	P	P	P	P	P	P	P	P	P	P	P	P	P	P	P	P	P	P	P	P
81	SALIVATION AFTER TREATMENT		P	P	P	P	P	P	P	P	P	P	P	P	P	P	P	P	P	P	P	P	P	P	P	P	P
	ALL PUPS STILLBORN	P																									
82	SALIVATION AFTER TREATMENT	P	P	P	P	P	P	P	P	P	P	P	P	P	P	P	P	P	P	P	P	P	P	P	P	P	P
83	SALIVATION AFTER TREATMENT	P	P	P	P	P	P	P	P	P	P	P	P	P	P	P	P	P	P	P	P	P	P	P	P	P	P
84	SALIVATION AFTER TREATMENT	P	P	P	P	P	P	P	P	P	P	P	P	P	P	P	P	P	P	P	P	P	P	P	P	P	P
85	SALIVATION AFTER TREATMENT	P	P	P	P	P	P	P	P	P	P	P	P	P	P	P	P	P	P	P	P	P	P	P	P	P	P
86	SALIVATION AFTER TREATMENT	P	P	P	P	P	P	P	P	P	P	P	P	P	P	P	P	P	P	P	P	P	P	P	P	P	P
87	SALIVATION AFTER TREATMENT	P	P	P	P	P	P	P	P	P	P	P	P	P	P	P	P	P	P	P	P	P	P	P	P	P	P
93	SALIVATION AFTER TREATMENT	P	P	P	P	P	P	P	P	P	P	P	P	P	P	P	P	P	P	P	P	P	P	P	P	P	P
94	SALIVATION AFTER TREATMENT	P	P	P	P	P	P	P	P	P	P	P	P	P	P	P	P	P	P	P	P	P	P	P	P	P	P
95	SALIVATION AFTER TREATMENT	P	P	P	P	P	P	P	P	P	P	P	P	P	P	P	P	P	P	P	P	P	P	P	P	P	P
96	SALIVATION AFTER TREATMENT	P	P	P	P	P	P	P	P	P	P	P	P	P	P	P	P	P	P	P	P	P	P	P	P	P	P
97	SALIVATION AFTER TREATMENT	P	P	P	P	P	P	P	P	P	P	P	P	P	P	P	P	P	P	P	P	P	P	P	P	P	P
98	SALIVATION AFTER TREATMENT	P	P	P	P	P	P	P	P	P	P	P	P	P	P	P	P	P	P	P	P	P	P	P	P	P	P
99	SALIVATION AFTER TREATMENT	P	P	P	P	P	P	P	P	P	P	P	P	P	P	P	P	P	P	P	P	P	P	P	P	P	P
100	SALIVATION AFTER TREATMENT	P	P	P	P	P	P	P	P	P	P	P	P	P	P	P	P	P	P	P	P	P	P	P	P	P	P

CODE: 1-SLIGHT 2-MODERATE 3-MARKED P-PRESENT

22-AUG-13

88R002 TABLE : IIA- 017

PR.NO.60R0375/88R002: REPRODUCTIVE TOX. STUDY TO DETECT EFFECTS
OF MIXED ANTI-ANDROGENIC SUBSTANCES IN RATS; ORAL ADM. (GAVAGE)
INDIVIDUAL MATERNAL/PUP CLINICAL OBSERVATIONS DURING LACTATION

TEST GROUP 4(0.00025 MG/KG BW/D)

FEMALE#	OBSERVATIONS	DAY OF LACTATION	0	1	2	3	4	5	6	7	8	9	10	11	12	13	14	15	16	17	18	19	20	21	22	23	24	25
101	NOTHING ABNORMAL DETECTED		P	P	P	P	P	P	P	P	P	P	P	P	P	P	P	P	P	P	P	P	P	P	P	P	P	P
102	NOTHING ABNORMAL DETECTED		P	P	P	P	P	P	P	P	P	P	P	P	P	P	P	P	P	P	P	P	P	P	P	P	P	P
103	NOTHING ABNORMAL DETECTED		P	P	P	P	P	P	P	P	P	P	P	P	P	P	P	P	P	P	P	P	P	P	P	P	P	P
104	NOTHING ABNORMAL DETECTED		P	P	P	P	P	P	P	P	P	P	P	P	P	P	P	P	P	P	P	P	P	P	P	P	P	P
105	NOTHING ABNORMAL DETECTED		P	P	P	P	P	P	P	P	P	P	P	P	P	P	P	P	P	P	P	P	P	P	P	P	P	P
106	NOTHING ABNORMAL DETECTED		P	P	P	P	P	P	P	P	P	P	P	P	P	P	P	P	P	P	P	P	P	P	P	P	P	P
107	NOTHING ABNORMAL DETECTED		P	P	P	P	P	P	P	P	P	P	P	P	P	P	P	P	P	P	P	P	P	P	P	P	P	P
108	NOTHING ABNORMAL DETECTED		P	P	P	P	P	P	P	P	P	P	P	P	P	P	P	P	P	P	P	P	P	P	P	P	P	P
109	NOTHING ABNORMAL DETECTED		P	P	P	P	P	P	P	P	P	P	P	P	P	P	P	P	P	P	P	P	P	P	P	P	P	P
110	NOTHING ABNORMAL DETECTED		P		P	P	P	P	P	P	P	P	P	P	P	P	P	P	P	P	P	P	P	P	P	P	P	P
	NO MORE PUPS ALIVE			P																								
111	NOTHING ABNORMAL DETECTED		P	P	P	P	P	P	P	P	P	P	P	P	P	P	P	P	P	P	P	P	P	P	P	P	P	P
117	NOTHING ABNORMAL DETECTED		P	P	P	P	P	P	P	P	P	P	P	P	P	P	P	P	P	P	P	P	P	P	P	P	P	P
118	NOTHING ABNORMAL DETECTED		P	P	P	P	P	P	P	P	P	P	P	P	P	P	P	P	P	P	P	P	P	P	P	P	P	P
119	NOTHING ABNORMAL DETECTED		P	P	P	P	P	P	P	P	P	P	P	P	P	P	P	P	P	P	P	P	P	P	P	P	P	P
120	NOTHING ABNORMAL DETECTED		P	P	P	P	P	P	P	P	P	P	P	P	P	P	P	P	P	P	P	P	P	P	P	P	P	P
121	NOTHING ABNORMAL DETECTED		P	P	P	P	P	P	P	P	P	P	P	P	P	P	P	P	P	P	P	P	P	P	P	P	P	P
123	NOTHING ABNORMAL DETECTED		P	P	P	P	P	P	P	P	P	P	P	P	P	P	P	P	P	P	P	P	P	P	P	P	P	P
124	NOTHING ABNORMAL DETECTED		P	P	P	P	P	P	P	P	P	P	P	P	P	P	P	P	P	P	P	P	P	P	P	P	P	P
125	NOTHING ABNORMAL DETECTED		P	P	P	P	P	P	P	P	P	P	P	P	P	P	P	P	P	P	P	P	P	P	P	P	P	P

CODE: 1-SLIGHT 2-MODERATE 3-MARKED P-PRESENT

22-AUG-13

88R002 TABLE : IIA- 018

PR.NO.60R0375/88R002: REPRODUCTIVE TOX. STUDY TO DETECT EFFECTS
OF MIXED ANTI-ANDROGENIC SUBSTANCES IN RATS; ORAL ADM. (GAVAGE)
INDIVIDUAL MATERNAL/PUP CLINICAL OBSERVATIONS DURING LACTATION

TEST GROUP 0 (0 MG/KG BW/D)

FEMALE#	OBSERVATIONS	DAY OF LACTATION	26	27	28	29	30	31	32	33	34	35	36	37	38	39	40	41	42	43	44	45	46	47	48	49	50	51	52
1	NOTHING ABNORMAL DETECTED		P	P	P	P	P	P	P	P	P	P	P	P	P	P	P	P	P	P	P	P	P	P	P				
	SCHEDULED SACRIFICE																								P				
2	NOTHING ABNORMAL DETECTED		P	P	P	P	P	P	P	P	P	P	P	P	P	P	P	P	P	P	P	P	P	P	P				
	SCHEDULED SACRIFICE																								P				
3	NOTHING ABNORMAL DETECTED		P	P	P	P	P	P	P	P	P	P	P	P	P	P	P	P	P	P	P	P	P	P	P				
	SCHEDULED SACRIFICE																								P				
4	NOTHING ABNORMAL DETECTED		P	P	P	P	P	P	P	P	P	P	P	P	P	P	P	P	P	P	P	P	P	P	P				
	SCHEDULED SACRIFICE																								P				
5	NOTHING ABNORMAL DETECTED		P	P	P	P	P	P	P	P	P	P	P	P	P	P	P	P	P	P	P	P	P	P	P				
	SCHEDULED SACRIFICE																								P				
6	NOTHING ABNORMAL DETECTED		P	P	P	P	P	P	P	P	P	P	P	P	P	P	P	P	P	P	P	P	P	P	P				
	SCHEDULED SACRIFICE																								P				
7	NOTHING ABNORMAL DETECTED		P	P	P	P	P	P	P	P	P	P	P	P	P	P	P	P	P	P	P	P	P	P	P				
	SCHEDULED SACRIFICE																								P				
8	NOTHING ABNORMAL DETECTED		P	P	P	P	P	P	P	P	P	P	P	P	P	P	P	P	P	P	P	P	P	P					
	SCHEDULED SACRIFICE																							P					
9	NOTHING ABNORMAL DETECTED		P	P	P	P	P	P	P	P	P	P	P	P	P	P	P	P	P	P	P	P	P	P					
	SCHEDULED SACRIFICE																							P					
10	NOTHING ABNORMAL DETECTED		P	P	P	P	P	P	P	P	P	P	P	P	P	P	P	P	P	P	P	P	P	P					
	SCHEDULED SACRIFICE																							P					
11	NOTHING ABNORMAL DETECTED		P	P	P	P	P	P	P	P	P	P	P	P	P	P	P	P	P	P	P	P	P	P	P	P	P	P	
	SCHEDULED SACRIFICE																											P	
17	NOTHING ABNORMAL DETECTED		P	P	P	P	P	P	P	P	P	P	P	P	P	P	P	P	P	P	P	P	P	P	P	P	P		
	SCHEDULED SACRIFICE																										P		
18	NOTHING ABNORMAL DETECTED		P	P	P	P	P	P	P	P	P	P	P	P	P	P	P	P	P	P	P	P	P	P	P	P	P		
	SCHEDULED SACRIFICE																										P		
19	NOTHING ABNORMAL DETECTED		P	P	P	P	P	P	P	P	P	P	P	P	P	P	P	P	P	P	P	P	P	P	P	P	P		
	SCHEDULED SACRIFICE																										P		

CODE: 1-SLIGHT 2-MODERATE 3-MARKED P-PRESENT

PR.NO.60R0375/88R002: REPRODUCTIVE TOX. STUDY TO DETECT EFFECTS
OF MIXED ANTI-ANDROGENIC SUBSTANCES IN RATS; ORAL ADM. (GAVAGE)
INDIVIDUAL MATERNAL/PUP CLINICAL OBSERVATIONS DURING LACTATION

TEST GROUP 0 (0 MG/KG BW/D)

FEMALE#	OBSERVATIONS	DAY OF LACTATION	26	27	28	29	30	31	32	33	34	35	36	37	38	39	40	41	42	43	44	45	46	47	48	49	50	51	52
20	NOTHING ABNORMAL DETECTED		P	P	P	P	P	P	P	P	P	P	P	P	P	P	P	P	P	P	P	P	P	P	P	P	P		
	SCHEDULED SACRIFICE																										P		
21	NOTHING ABNORMAL DETECTED		P	P	P	P	P	P	P	P	P	P	P	P	P	P	P	P	P	P	P	P	P	P	P	P	P		
	SCHEDULED SACRIFICE																										P		
22	NOTHING ABNORMAL DETECTED		P	P	P	P	P	P	P	P	P	P	P	P	P	P	P	P	P	P	P	P	P	P	P	P	P	P	
	SCHEDULED SACRIFICE																											P	
23	NOTHING ABNORMAL DETECTED		P	P	P	P	P	P	P	P	P	P	P	P	P	P	P	P	P	P	P	P	P	P	P	P			
	SCHEDULED SACRIFICE																									P			
24	NOTHING ABNORMAL DETECTED		P	P	P	P	P	P	P	P	P	P	P	P	P	P	P	P	P	P	P	P	P	P	P	P			
	SCHEDULED SACRIFICE																									P			
25	NOTHING ABNORMAL DETECTED		P	P	P	P	P	P	P	P	P	P	P	P	P	P	P	P	P	P	P	P	P	P	P	P			
	SCHEDULED SACRIFICE																									P			

CODE: 1-SLIGHT 2-MODERATE 3-MARKED P-PRESENT

PR.NO.60R0375/88R002: REPRODUCTIVE TOX. STUDY TO DETECT EFFECTS
OF MIXED ANTI-ANDROGENIC SUBSTANCES IN RATS; ORAL ADM. (GAVAGE)
INDIVIDUAL MATERNAL/PUP CLINICAL OBSERVATIONS DURING LACTATION

TEST GROUP 1 (ADI-MIX)

FEMALE#	OBSERVATIONS	DAY OF LACTATION	26	27	28	29	30	31	32	33	34	35	36	37	38	39	40	41	42	43	44	45	46	47	48	49	50	51	52
26	NOTHING ABNORMAL DETECTED		P	P	P	P	P	P	P	P	P	P	P	P	P	P	P	P	P	P	P	P	P	P	P				
	SCHEDULED SACRIFICE																								P				
27	NOTHING ABNORMAL DETECTED		P	P	P	P	P	P	P	P	P	P	P	P	P	P	P	P	P	P	P	P	P	P	P				
	SCHEDULED SACRIFICE																								P				
28	NOTHING ABNORMAL DETECTED		P	P	P	P	P	P																					
	FOUND DEAD								P																				
29	NOTHING ABNORMAL DETECTED		P	P	P	P	P	P	P	P	P	P	P	P	P	P	P	P	P	P	P	P	P	P	P				
	SCHEDULED SACRIFICE																								P				
30	NOTHING ABNORMAL DETECTED		P	P	P	P	P	P	P	P	P	P	P	P	P	P	P	P	P	P	P	P	P	P	P	P			
	SCHEDULED SACRIFICE																									P			
31	NOTHING ABNORMAL DETECTED		P	P	P	P	P	P	P	P	P	P	P	P	P	P	P	P	P	P	P	P	P	P	P				
	SCHEDULED SACRIFICE																								P				
32	NOTHING ABNORMAL DETECTED		P	P	P	P	P	P	P	P	P	P	P	P	P	P	P	P	P	P	P	P	P	P	P				
	SCHEDULED SACRIFICE																								P				
33	NOTHING ABNORMAL DETECTED		P	P	P	P	P	P	P	P	P	P	P	P	P	P	P	P	P	P	P	P	P	P					
	SCHEDULED SACRIFICE																							P					
34	NOTHING ABNORMAL DETECTED		P	P	P	P	P	P	P	P	P	P	P	P	P	P	P	P	P	P	P	P	P	P					
	SCHEDULED SACRIFICE																							P					
35	NOTHING ABNORMAL DETECTED		P	P	P	P	P	P	P	P	P	P	P	P	P	P	P	P	P	P	P	P	P	P					
	SCHEDULED SACRIFICE																							P					
36	NOTHING ABNORMAL DETECTED		P	P	P	P	P	P	P	P	P	P	P	P	P	P	P	P	P	P	P	P	P	P	P	P	P	P	
	SCHEDULED SACRIFICE																											P	
37	NOTHING ABNORMAL DETECTED		P	P	P	P	P	P	P	P	P	P	P	P	P	P	P	P	P	P	P	P	P	P	P	P	P	P	P
	SCHEDULED SACRIFICE																												P
43	NOTHING ABNORMAL DETECTED		P	P	P	P	P	P	P	P	P	P	P	P	P	P	P	P	P	P	P	P	P	P	P	P	P		
	SCHEDULED SACRIFICE																										P		
44	NOTHING ABNORMAL DETECTED		P	P	P	P	P	P	P	P	P	P	P	P	P	P	P	P	P	P	P	P	P	P	P	P	P		
	SCHEDULED SACRIFICE																										P		

CODE: 1-SLIGHT 2-MODERATE 3-MARKED P-PRESENT

PR.NO.60R0375/88R002: REPRODUCTIVE TOX. STUDY TO DETECT EFFECTS
OF MIXED ANTI-ANDROGENIC SUBSTANCES IN RATS; ORAL ADM. (GAVAGE)
INDIVIDUAL MATERNAL/PUP CLINICAL OBSERVATIONS DURING LACTATION

TEST GROUP 1 (ADI-MIX)

FEMALE#	OBSERVATIONS	DAY OF LACTATION	26	27	28	29	30	31	32	33	34	35	36	37	38	39	40	41	42	43	44	45	46	47	48	49	50	51	52
45	NOTHING ABNORMAL DETECTED		P	P	P	P	P	P	P	P	P	P	P	P	P	P	P	P	P	P	P	P	P	P	P	P	P	P	
	SCHEDULED SACRIFICE																											P	
46	NOTHING ABNORMAL DETECTED		P	P	P	P	P	P	P	P	P	P	P	P	P	P	P	P	P	P	P	P	P	P	P	P	P	P	
	SCHEDULED SACRIFICE																											P	
47	NOTHING ABNORMAL DETECTED		P	P	P	P	P	P	P	P	P	P	P	P	P	P	P	P	P	P	P	P	P	P	P	P	P		
	SCHEDULED SACRIFICE																										P		
48	NOTHING ABNORMAL DETECTED		P	P	P	P	P	P	P	P	P	P	P	P	P	P	P	P	P	P	P	P	P	P	P	P	P		
	SCHEDULED SACRIFICE																										P		
49	NOTHING ABNORMAL DETECTED		P	P	P	P	P	P	P	P	P	P	P	P	P	P	P	P	P	P	P	P	P	P	P	P			
	SCHEDULED SACRIFICE																									P			
50	NOTHING ABNORMAL DETECTED		P	P	P	P	P	P	P	P	P	P	P	P	P	P	P	P	P	P	P	P	P	P	P	P			
	SCHEDULED SACRIFICE																									P			

CODE: 1-SLIGHT 2-MODERATE 3-MARKED P-PRESENT

22-AUG-13

88R002 TABLE : IIA- 022

PR.NO.60R0375/88R002: REPRODUCTIVE TOX. STUDY TO DETECT EFFECTS
OF MIXED ANTI-ANDROGENIC SUBSTANCES IN RATS; ORAL ADM. (GAVAGE)
INDIVIDUAL MATERNAL/PUP CLINICAL OBSERVATIONS DURING LACTATION

TEST GROUP 2 (NOAEL-MIX)

FEMALE#	OBSERVATIONS	DAY OF LACTATION	26	27	28	29	30	31	32	33	34	35	36	37	38	39	40	41	42	43	44	45	46	47	48	49	50	51	52
51	NOTHING ABNORMAL DETECTED		P	P	P	P	P	P	P	P	P	P	P	P	P	P	P	P	P	P	P	P	P	P	P				
	SCHEDULED SACRIFICE																								P				
52	NOTHING ABNORMAL DETECTED		P	P	P	P	P	P	P	P	P	P	P	P	P	P	P	P	P	P	P	P	P	P	P				
	SCHEDULED SACRIFICE																								P				
53	NOTHING ABNORMAL DETECTED		P	P	P	P	P	P	P	P	P	P	P	P	P	P	P	P	P	P	P	P	P	P	P				
	SCHEDULED SACRIFICE																								P				
54	NOTHING ABNORMAL DETECTED		P	P	P	P	P	P	P	P	P	P	P	P	P	P	P	P	P	P	P	P	P	P	P				
	SCHEDULED SACRIFICE																								P				
55	NOTHING ABNORMAL DETECTED		P	P	P	P	P	P	P	P	P	P	P	P	P	P	P	P	P	P	P	P	P	P	P				
	SCHEDULED SACRIFICE																								P				
56	NOTHING ABNORMAL DETECTED		P	P	P	P	P	P	P	P	P	P	P	P	P	P	P	P	P	P	P	P	P	P					
	SCHEDULED SACRIFICE																							P					
57	NOTHING ABNORMAL DETECTED		P	P	P	P	P	P	P	P	P	P	P	P	P	P	P	P	P	P	P	P	P	P	P				
	SCHEDULED SACRIFICE																								P				
58	NOTHING ABNORMAL DETECTED		P	P	P	P	P	P	P	P	P	P	P	P	P	P	P	P	P	P	P	P	P						
	SCHEDULED SACRIFICE																						P						
59	NOTHING ABNORMAL DETECTED					P				P	P	P	P	P	P	P	P	P	P	P	P	P	P	P					
	SALIVATION AFTER TREATMENT		P	P	P		P	P	P																				
	SCHEDULED SACRIFICE																							P					
60	NOTHING ABNORMAL DETECTED		P		P	P				P	P		P		P		P	P	P	P	P	P		P					
	SALIVATION AFTER TREATMENT			P			P	P	P			P		P		P							P						
	SCHEDULED SACRIFICE																							P					
61	NOTHING ABNORMAL DETECTED		P	P	P	P	P	P	P	P	P	P	P	P	P	P	P	P	P	P	P	P	P	P	P	P	P	P	
	SCHEDULED SACRIFICE																											P	
62	NOTHING ABNORMAL DETECTED		P	P	P	P	P	P	P	P	P	P	P	P	P	P	P	P	P	P	P	P	P	P	P	P	P		
	SCHEDULED SACRIFICE																										P		
68	NOTHING ABNORMAL DETECTED		P	P	P	P	P	P	P	P	P	P	P	P	P	P	P	P	P	P	P	P	P	P	P	P	P		
	SCHEDULED SACRIFICE																										P		

CODE: 1-SLIGHT 2-MODERATE 3-MARKED P-PRESENT

22-AUG-13

88R002 TABLE : IIA- 023

PR.NO.60R0375/88R002: REPRODUCTIVE TOX. STUDY TO DETECT EFFECTS
OF MIXED ANTI-ANDROGENIC SUBSTANCES IN RATS; ORAL ADM. (GAVAGE)
INDIVIDUAL MATERNAL/PUP CLINICAL OBSERVATIONS DURING LACTATION

TEST GROUP 2 (NOAEL-MIX)

FEMALE#	OBSERVATIONS	DAY OF LACTATION	26	27	28	29	30	31	32	33	34	35	36	37	38	39	40	41	42	43	44	45	46	47	48	49	50	51	52
69	NOTHING ABNORMAL DETECTED		P	P	P	P	P	P	P	P	P	P	P	P	P	P	P	P	P	P	P	P	P	P	P	P	P		
	SCHEDULED SACRIFICE																										P		
70	NOTHING ABNORMAL DETECTED		P	P	P	P	P	P	P	P	P	P	P	P	P	P	P	P	P	P	P	P	P	P	P	P			
	SCHEDULED SACRIFICE																									P			
71	NOTHING ABNORMAL DETECTED		P	P	P	P	P	P	P	P	P	P	P	P	P	P	P	P	P	P	P	P	P	P	P	P	P		
	SCHEDULED SACRIFICE																										P		
72	NOTHING ABNORMAL DETECTED		P	P	P	P	P	P	P	P	P	P	P	P	P	P	P	P	P	P	P	P	P	P	P	P	P		
	SCHEDULED SACRIFICE																										P		
73	NOTHING ABNORMAL DETECTED		P	P	P	P	P	P	P	P	P	P	P	P	P	P	P	P	P	P	P	P	P	P	P				
	SCHEDULED SACRIFICE																								P				
74	NOTHING ABNORMAL DETECTED		P	P	P	P	P	P	P	P	P	P	P	P	P	P	P	P	P	P	P	P	P	P	P				
	SCHEDULED SACRIFICE																								P				
75	NOTHING ABNORMAL DETECTED		P	P	P	P	P	P	P	P	P	P	P	P	P	P	P	P	P	P	P	P	P	P	P	P			
	SCHEDULED SACRIFICE																									P			

CODE: 1-SLIGHT 2-MODERATE 3-MARKED P-PRESENT

PR.NO.60R0375/88R002: REPRODUCTIVE TOX. STUDY TO DETECT EFFECTS
OF MIXED ANTI-ANDROGENIC SUBSTANCES IN RATS; ORAL ADM. (GAVAGE)
INDIVIDUAL MATERNAL/PUP CLINICAL OBSERVATIONS DURING LACTATION

TEST GROUP 3 (LOAEL-MIX)

FEMALE#	OBSERVATIONS DAY OF LACTATION	26	27	28	29	30	31	32	33	34	35	36	37	38	39	40	41	42	43	44	45	46	47	48	49	50	51	52
77	NOTHING ABNORMAL DETECTED																							P				
	SALIVATION AFTER TREATMENT	P	P	P	P	P	P	P	P	P	P	P	P	P	P	P	P	P	P	P	P	P	P					
	SCHEDULED SACRIFICE																							P				
78	NOTHING ABNORMAL DETECTED																						P					
	SALIVATION AFTER TREATMENT	P	P	P	P	P	P	P	P	P	P	P	P	P	P	P	P	P	P	P	P	P						
	SCHEDULED SACRIFICE																						P					
79	NOTHING ABNORMAL DETECTED																					P						
	SALIVATION AFTER TREATMENT	P	P	P	P	P	P	P	P	P	P	P	P	P	P	P	P	P	P	P	P							
	SCHEDULED SACRIFICE																					P						
80	NOTHING ABNORMAL DETECTED																						P					
	SALIVATION AFTER TREATMENT	P	P	P	P	P	P	P	P	P	P	P	P	P	P	P	P	P	P	P	P	P						
	SCHEDULED SACRIFICE																						P					
81	NOTHING ABNORMAL DETECTED																						P					
	SALIVATION AFTER TREATMENT	P	P	P	P	P	P	P	P	P	P	P	P	P	P	P	P	P	P	P	P	P						
	SCHEDULED SACRIFICE																						P					
82	NOTHING ABNORMAL DETECTED																						P					
	SALIVATION AFTER TREATMENT	P	P	P	P	P	P	P	P	P	P	P	P	P	P	P	P	P	P	P	P	P						
	SCHEDULED SACRIFICE																						P					
83	NOTHING ABNORMAL DETECTED																					P						
	SALIVATION AFTER TREATMENT	P	P	P	P	P	P	P	P	P	P	P	P	P	P	P	P	P	P	P	P							
	SCHEDULED SACRIFICE																					P						
84	NOTHING ABNORMAL DETECTED																					P						
	SALIVATION AFTER TREATMENT	P	P	P	P	P	P	P	P	P	P	P	P	P	P	P	P	P	P	P	P							
	SCHEDULED SACRIFICE																					P						
85	NOTHING ABNORMAL DETECTED																					P						
	SALIVATION AFTER TREATMENT	P	P	P	P	P	P	P	P	P	P	P	P	P	P	P	P	P	P	P	P							
	SCHEDULED SACRIFICE																					P						
86	NOTHING ABNORMAL DETECTED																					P						
	SALIVATION AFTER TREATMENT	P	P	P	P	P	P	P	P	P	P	P	P	P	P	P	P	P	P	P	P							
	SCHEDULED SACRIFICE																					P						
87	NOTHING ABNORMAL DETECTED																									P		
	SALIVATION AFTER TREATMENT	P	P	P	P	P	P	P	P	P	P	P	P	P	P	P	P	P	P	P	P	P	P	P	P			
	SCHEDULED SACRIFICE																									P		

CODE: 1-SLIGHT 2-MODERATE 3-MARKED P-PRESENT

PR.NO.60R0375/88R002: REPRODUCTIVE TOX. STUDY TO DETECT EFFECTS
OF MIXED ANTI-ANDROGENIC SUBSTANCES IN RATS; ORAL ADM. (GAVAGE)
INDIVIDUAL MATERNAL/PUP CLINICAL OBSERVATIONS DURING LACTATION

TEST GROUP 3 (LOAEL-MIX)

FEMALE#	OBSERVATIONS \ DAY OF LACTATION	26	27	28	29	30	31	32	33	34	35	36	37	38	39	40	41	42	43	44	45	46	47	48	49	50	51	52
93	NOTHING ABNORMAL DETECTED																								P			
	SALIVATION AFTER TREATMENT	P	P	P	P	P	P	P	P	P	P	P	P	P	P	P	P	P	P	P	P	P	P	P				
	SCHEDULED SACRIFICE																								P			
94	NOTHING ABNORMAL DETECTED																								P			
	SALIVATION AFTER TREATMENT	P	P	P	P	P	P	P	P	P	P	P	P	P	P	P	P	P	P	P	P	P	P	P				
	SCHEDULED SACRIFICE																								P			
95	NOTHING ABNORMAL DETECTED																									P		
	SALIVATION AFTER TREATMENT	P	P	P	P	P	P	P	P	P	P	P	P	P	P	P	P	P	P	P	P	P	P	P	P			
	SCHEDULED SACRIFICE																									P		
96	NOTHING ABNORMAL DETECTED																								P			
	SALIVATION AFTER TREATMENT	P	P	P	P	P	P	P	P	P	P	P	P	P	P	P	P	P	P	P	P	P	P	P				
	SCHEDULED SACRIFICE																								P			
97	NOTHING ABNORMAL DETECTED																								P			
	SALIVATION AFTER TREATMENT	P	P	P	P	P	P	P	P	P	P	P	P	P	P	P	P	P	P	P	P	P	P	P				
	SCHEDULED SACRIFICE																								P			
98	NOTHING ABNORMAL DETECTED																							P				
	SALIVATION AFTER TREATMENT	P	P	P	P	P	P	P	P	P	P	P	P	P	P	P	P	P	P	P	P	P	P					
	SCHEDULED SACRIFICE																							P				
99	NOTHING ABNORMAL DETECTED																							P				
	SALIVATION AFTER TREATMENT	P	P	P	P	P	P	P	P	P	P	P	P	P	P	P	P	P	P	P	P	P	P					
	SCHEDULED SACRIFICE																							P				
100	NOTHING ABNORMAL DETECTED																							P				
	SALIVATION AFTER TREATMENT	P	P	P	P	P	P	P	P	P	P	P	P	P	P	P	P	P	P	P	P	P	P					
	SCHEDULED SACRIFICE																							P				

CODE: 1-SLIGHT 2-MODERATE 3-MARKED P-PRESENT

22-AUG-13

88R002 TABLE : IIA- 026

PR.NO.60R0375/88R002: REPRODUCTIVE TOX. STUDY TO DETECT EFFECTS
OF MIXED ANTI-ANDROGENIC SUBSTANCES IN RATS; ORAL ADM. (GAVAGE)
INDIVIDUAL MATERNAL/PUP CLINICAL OBSERVATIONS DURING LACTATION

TEST GROUP 4(0.00025 MG/KG BW/D)

FEMALE#	OBSERVATIONS	DAY OF LACTATION	26	27	28	29	30	31	32	33	34	35	36	37	38	39	40	41	42	43	44	45	46	47	48	49	50	51	52
101	NOTHING ABNORMAL DETECTED		P	P	P	P	P	P	P	P	P	P	P	P	P	P	P	P	P	P	P	P	P	P					
	SCHEDULED SACRIFICE																							P					
102	NOTHING ABNORMAL DETECTED		P	P	P	P	P	P	P	P	P	P	P	P	P	P	P	P	P	P	P	P	P	P	P				
	SCHEDULED SACRIFICE																								P				
103	NOTHING ABNORMAL DETECTED		P	P	P	P	P	P	P	P	P	P	P	P	P	P	P	P	P	P	P	P	P	P	P	P			
	SCHEDULED SACRIFICE																									P			
104	NOTHING ABNORMAL DETECTED		P	P	P	P	P	P	P	P	P	P	P	P	P	P	P	P	P	P	P	P	P	P	P				
	SCHEDULED SACRIFICE																								P				
105	NOTHING ABNORMAL DETECTED		P	P	P	P	P	P	P	P	P	P	P	P	P	P	P	P	P	P	P	P	P	P	P				
	SCHEDULED SACRIFICE																								P				
106	NOTHING ABNORMAL DETECTED		P	P	P	P	P	P	P	P	P	P	P	P	P	P	P	P	P	P	P	P	P	P	P				
	SCHEDULED SACRIFICE																								P				
107	NOTHING ABNORMAL DETECTED		P	P	P	P	P	P	P	P	P	P	P	P	P	P	P	P	P	P	P	P	P	P	P				
	SCHEDULED SACRIFICE																								P				
108	NOTHING ABNORMAL DETECTED		P	P	P	P	P	P	P	P	P	P	P	P	P	P	P	P	P	P	P	P	P	P					
	SCHEDULED SACRIFICE																							P					
109	NOTHING ABNORMAL DETECTED		P	P	P	P	P	P	P	P	P	P	P	P	P	P	P	P	P	P	P	P	P	P					
	SCHEDULED SACRIFICE																							P					
110	NOTHING ABNORMAL DETECTED		P	P	P	P	P	P	P	P	P	P	P	P	P	P	P	P	P	P	P	P	P	P					
	SCHEDULED SACRIFICE																							P					
111	NOTHING ABNORMAL DETECTED		P	P	P	P	P	P	P	P	P	P	P	P	P	P	P	P	P	P	P	P	P	P	P	P	P	P	
	SCHEDULED SACRIFICE																											P	
117	NOTHING ABNORMAL DETECTED		P	P	P	P	P	P	P	P	P	P	P	P	P	P	P	P	P	P	P	P	P	P	P	P	P		
	SCHEDULED SACRIFICE																										P		
118	NOTHING ABNORMAL DETECTED		P	P	P	P	P	P	P	P	P	P	P	P	P	P	P	P	P	P	P	P	P	P	P	P	P		
	SCHEDULED SACRIFICE																										P		
119	NOTHING ABNORMAL DETECTED		P	P	P	P	P	P	P	P	P	P	P	P	P	P	P	P	P	P	P	P	P	P	P	P			
	SCHEDULED SACRIFICE																									P			

CODE: 1-SLIGHT 2-MODERATE 3-MARKED P-PRESENT

PR.NO.60R0375/88R002: REPRODUCTIVE TOX. STUDY TO DETECT EFFECTS
OF MIXED ANTI-ANDROGENIC SUBSTANCES IN RATS; ORAL ADM. (GAVAGE)
INDIVIDUAL MATERNAL/PUP CLINICAL OBSERVATIONS DURING LACTATION

TEST GROUP 4(0.00025 MG/KG BW/D)

FEMALE#	OBSERVATIONS	DAY OF LACTATION	26	27	28	29	30	31	32	33	34	35	36	37	38	39	40	41	42	43	44	45	46	47	48	49	50	51	52
120	NOTHING ABNORMAL DETECTED		P	P	P	P	P	P	P	P	P	P	P	P	P	P	P	P	P	P	P	P	P	P	P	P			
	SCHEDULED SACRIFICE																									P			
121	NOTHING ABNORMAL DETECTED		P	P	P	P	P	P	P	P	P	P	P	P	P	P	P	P	P	P	P	P	P	P	P	P	P	P	
	SCHEDULED SACRIFICE																											P	
123	NOTHING ABNORMAL DETECTED		P	P	P	P	P	P	P	P	P	P	P	P	P	P	P	P	P	P	P	P	P	P	P	P			
	SCHEDULED SACRIFICE																									P			
124	NOTHING ABNORMAL DETECTED		P	P	P	P	P	P	P	P	P	P	P	P	P	P	P	P	P	P	P	P	P	P	P	P			
	SCHEDULED SACRIFICE																									P			
125	NOTHING ABNORMAL DETECTED		P	P	P	P	P	P	P	P	P	P	P	P	P	P	P	P	P	P	P	P	P	P	P	P			
	SCHEDULED SACRIFICE																									P			

CODE: 1-SLIGHT 2-MODERATE 3-MARKED P-PRESENT

PR.NO.60R0375/88R002: REPRODUCTIVE TOX. STUDY TO DETECT EFFECTS
OF MIXED ANTI-ANDROGENIC SUBSTANCES IN RATS; ORAL ADM. (GAVAGE)
INDIVIDUAL MATERNAL FOOD CONSUMPTION DURING GESTATION -- GRAMS/ANIMAL/DAY

TEST GROUP 0 (0 MG/KG BW/D)

	DAY OF GESTATION		
FEMALE#	0 - 6	6 - 13	13 - 20
1	16.2	17.8	20.1
2	16.3	15.9	17.6
3	16.4	16.5	17.0
4	17.2	17.2	18.5
5	15.7	15.5	19.1
6	14.0	14.5	16.2
7	16.8	17.2	19.4
8	14.5	15.2	17.1
9	14.1	14.9	17.4
10	15.1	18.0	19.2
11	14.2	15.6	19.1
12	16.1	15.4	17.4
13	15.8	16.3	17.5
14	18.2	18.9	19.1
15	19.3	21.8	26.9
16	16.8	18.4	20.4
17	16.9	17.7	20.2
18	16.5	17.8	20.6
19	14.4	14.9	16.6
20	13.9	13.7	16.7
21	17.6	19.3	21.1
22	18.9	20.9	22.6
23	18.0	20.1	21.6
24	15.6	17.9	19.3
25	14.2	15.7	18.3
MEAN	16.1	17.1	19.2
S.D.	1.57	2.06	2.32
N	25	25	25

PR.NO.60R0375/88R002: REPRODUCTIVE TOX. STUDY TO DETECT EFFECTS
OF MIXED ANTI-ANDROGENIC SUBSTANCES IN RATS; ORAL ADM. (GAVAGE)
INDIVIDUAL MATERNAL FOOD CONSUMPTION DURING GESTATION -- GRAMS/ANIMAL/DAY

TEST GROUP 1 (ADI-MIX)

	DAY OF GESTATION		
FEMALE#	0 - 6	6 - 13	13 - 20
26	15.1	15.7	18.3
27	19.4	20.7	22.8
28	18.3	18.4	21.4
29	16.2	17.9	21.2
30	17.3	17.7	20.6
31	15.7	16.8	19.1
32	16.1	17.7	20.3
33	15.2	17.7	20.8
34	14.4	13.3	15.6
35	18.9	20.6	20.6
36	16.1	17.3	19.3
37	12.3	14.6	16.4
38	14.6	16.2	18.1
39	15.2	16.9	19.6
40	15.3	16.0	19.2
41	17.2	16.9	19.4
42	16.9	18.6	21.4
43	14.6	16.5	18.2
44	15.7	17.2	19.0
45	16.0	15.6	17.5
46	15.5	16.8	20.0
47	16.8	17.9	18.6
48	16.1	16.6	19.8
49	16.0	17.7	19.8
50	16.0	17.3	19.1
MEAN	16.0	17.1	19.4
S.D.	1.49	1.58	1.60
N	25	25	25

PR.NO.60R0375/88R002: REPRODUCTIVE TOX. STUDY TO DETECT EFFECTS
OF MIXED ANTI-ANDROGENIC SUBSTANCES IN RATS; ORAL ADM. (GAVAGE)
INDIVIDUAL MATERNAL FOOD CONSUMPTION DURING GESTATION -- GRAMS/ANIMAL/DAY

TEST GROUP 2 (NOAEL-MIX)

	DAY OF GESTATION		
FEMALE#	0 - 6	6 - 13	13 - 20
51	15.6	16.1	17.0
52	18.6	19.8	22.3
53	16.6	16.4	17.7
54	15.2	19.0	19.3
55	16.1	16.6	20.6
56	17.3	17.0	15.7
57	18.7	19.6	21.0
58	14.0	14.7	17.9
59	16.7	18.3	19.7
60	17.8	20.0	20.6
61	17.0	18.3	20.7
62	14.0	14.6	15.4
63	16.8	17.8	19.6
64	18.4	20.0	19.5
65	14.7	16.4	17.2
66	16.5	17.6	19.2
67	16.6	18.4	16.4
68	15.8	16.6	19.8
69	15.8	15.7	18.5
70	16.1	17.5	18.8
71	13.6	14.6	17.3
72	16.3	18.7	21.3
73	13.4	13.2	16.4
74	12.1	13.2	14.7
75	14.9	16.2	17.4
MEAN	15.9	17.1	18.6
S.D.	1.67	2.00	2.00
N	25	25	25

PR.NO.60R0375/88R002: REPRODUCTIVE TOX. STUDY TO DETECT EFFECTS
OF MIXED ANTI-ANDROGENIC SUBSTANCES IN RATS; ORAL ADM. (GAVAGE)
INDIVIDUAL MATERNAL FOOD CONSUMPTION DURING GESTATION -- GRAMS/ANIMAL/DAY

TEST GROUP 3 (LOAEL-MIX)

FEMALE#		DAY OF GESTATION 0 - 6	6 - 13	13 - 20
76	UDS	18.0	18.5	18.2
77		17.6	17.8	19.0
78		18.6	18.6	19.6
79		16.7	17.5	19.9
80		14.8	15.4	17.6
81		16.6	16.6	17.3
82		16.3	17.1	18.8
83		16.9	18.5	19.6
84		14.2	15.3	15.9
85		15.4	16.5	17.7
86		16.3	19.5	20.1
87		17.4	17.8	19.7
88		15.2	15.2	15.6
89		18.1	16.6	18.1
90		18.0	17.6	18.3
91		18.4	18.3	20.3
92		16.7	15.7	16.7
93		17.1	16.7	18.5
94		17.8	18.3	21.1
95		17.1	17.8	19.6
96		17.2	16.6	20.3
97		16.4	17.5	19.1
98		13.9	15.4	17.3
99		15.6	14.8	16.6
100		17.2	17.0	18.6
MEAN		16.7	17.1	18.5
S.D.		1.27	1.25	1.44
N		25	25	25

UDS=UNABLE TO DELIVER, SAC'D

PR.NO.60R0375/88R002: REPRODUCTIVE TOX. STUDY TO DETECT EFFECTS
OF MIXED ANTI-ANDROGENIC SUBSTANCES IN RATS; ORAL ADM. (GAVAGE)
INDIVIDUAL MATERNAL FOOD CONSUMPTION DURING GESTATION -- GRAMS/ANIMAL/DAY

TEST GROUP 4(0.00025 MG/KG BW/D)

		DAY OF GESTATION		
FEMALE#		0 - 6	6 - 13	13 - 20
101		16.5	16.6	18.3
102		15.3	16.4	16.9
103		16.1	18.9	23.1
104		20.2	20.6	21.9
105		16.4	17.0	18.7
106		17.3	18.4	20.2
107		17.3	18.2	19.7
108		14.6	13.7	17.1
109		17.9	18.5	20.0
110		14.8	15.0	15.7
111		16.4	17.1	18.4
112		17.9	18.5	22.1
113		17.0	19.0	20.3
114		16.5	18.6	21.5
115		18.0	18.4	19.9
116		17.5	20.1	22.8
117		13.6	15.5	16.8
118		18.7	19.3	19.6
119		15.0	17.6	22.0
120		16.7	17.9	19.3
121		18.3	20.1	24.2
122x	NP	15.2	13.6	14.0
123		15.8	17.3	19.6
124		17.3	19.3	19.8
125		16.8	18.9	20.9
MEAN		16.7	17.9	20.0
S.D.		1.46	1.66	2.14
N		24	24	24

NP=NOT PREGNANT x=EXCLUDED FROM MEAN

PR.NO.60R0375/88R002: REPRODUCTIVE TOX. STUDY TO DETECT EFFECTS
OF MIXED ANTI-ANDROGENIC SUBSTANCES IN RATS; ORAL ADM. (GAVAGE)
INDIVIDUAL MATERNAL FOOD CONSUMPTION DURING LACTATION -- GRAMS/ANIMAL/DAY

TEST GROUP 0 (0 MG/KG BW/D)

	DAY OF LACTATION		
FEMALE#	0 - 7	7 - 14	14 - 21
1	28.7	47.2	58.3
2	30.0	50.2	60.5
3	28.4	47.9	59.0
4	31.7	52.0	64.1
5	34.5	54.5	66.9
6	30.2	43.3	58.9
7	33.2	54.3	62.8
8	28.8	45.8	58.7
9	23.1	36.6	49.2
10	28.5	46.9	56.2
11	31.6	50.2	58.7
17	26.8	41.6	52.2
18	37.7	58.1	68.1
19	26.2	44.0	52.9
20	30.2	48.2	62.6
21	38.4	57.9	68.5
22	27.2	44.9	53.8
23	30.6	50.3	59.4
24	32.5	51.4	65.9
25	33.4	53.5	60.9
MEAN	30.6	48.9	59.9
S.D.	3.73	5.44	5.35
N	20	20	20

PR.NO.60R0375/88R002: REPRODUCTIVE TOX. STUDY TO DETECT EFFECTS
OF MIXED ANTI-ANDROGENIC SUBSTANCES IN RATS; ORAL ADM. (GAVAGE)
INDIVIDUAL MATERNAL FOOD CONSUMPTION DURING LACTATION -- GRAMS/ANIMAL/DAY

TEST GROUP 1 (ADI-MIX)

	DAY OF LACTATION		
FEMALE#	0 - 7	7 - 14	14 - 21
26	30.4	50.1	59.9
27	32.2	51.2	63.8
28	29.3	48.9	56.0
29	35.9	55.6	73.6
30	34.2	58.8	67.0
31	34.4	55.2	63.9
32	30.0	50.0	60.1
33	35.4	57.5	67.1
34	28.2	46.0	57.4
35	30.8	51.0	63.0
36	35.1	51.7	65.8
37	22.3	40.6	45.7
43	33.9	50.8	64.2
44	31.3	51.2	61.8
45	27.3	48.4	60.3
46	31.6	53.5	64.2
47	27.9	42.4	52.3
48	27.7	45.6	56.7
49	37.3	57.3	70.2
50	27.8	43.8	55.9
MEAN	31.2	50.5	61.4
S.D.	3.74	5.05	6.38
N	20	20	20

PR.NO.60R0375/88R002: REPRODUCTIVE TOX. STUDY TO DETECT EFFECTS
OF MIXED ANTI-ANDROGENIC SUBSTANCES IN RATS; ORAL ADM. (GAVAGE)
INDIVIDUAL MATERNAL FOOD CONSUMPTION DURING LACTATION -- GRAMS/ANIMAL/DAY

TEST GROUP 2 (NOAEL-MIX)

	DAY OF LACTATION		
FEMALE#	0 - 7	7 - 14	14 - 21
51	26.5	51.9	65.4
52	33.3	55.3	63.8
53	25.2	43.7	58.7
54	32.2	52.5	62.2
55	31.6	51.3	64.8
56	13.0	19.9	22.9
57	30.1	52.6	63.9
58	29.1	43.5	56.8
59	30.6	54.1	62.8
60	34.2	50.5	61.1
61	31.4	46.9	63.4
62	25.9	42.6	56.1
68	34.6	52.0	63.6
69	28.9	48.0	60.3
70	23.8	38.5	51.2
71	29.6	46.9	59.6
72	33.4	54.4	67.8
73	29.3	45.2	58.8
74	29.2	47.6	61.7
75	30.7	51.2	54.8
MEAN	29.1	47.4	59.0
S.D.	4.80	7.89	9.39
N	20	20	20

PR.NO.60R0375/88R002: REPRODUCTIVE TOX. STUDY TO DETECT EFFECTS
OF MIXED ANTI-ANDROGENIC SUBSTANCES IN RATS; ORAL ADM. (GAVAGE)
INDIVIDUAL MATERNAL FOOD CONSUMPTION DURING LACTATION -- GRAMS/ANIMAL/DAY

TEST GROUP 3 (LOAEL-MIX)

FEMALE#		DAY OF LACTATION 0 - 7	7 - 14	14 - 21
76	UDS			
77		24.2	43.3	59.2
78		31.9	55.0	66.8
79		10.7	13.0	14.8
80		21.3	31.5	38.5
81		12.2	11.9	12.5
82		32.8	49.7	68.5
83		33.1	49.6	64.4
84		25.0	39.8	51.2
85		28.1	44.5	58.8
86		29.6	56.5	70.3
87		39.0	58.0	70.9
93		28.3	46.1	55.7
94		35.4	56.1	65.3
95		30.6	52.5	65.7
96		30.7	47.5	62.5
97		33.8	53.3	64.1
98		26.3	50.2	67.7
99		31.4	50.5	67.7
100		35.4	54.8	67.2
MEAN		28.4	45.5	57.5
S.D.		7.35	13.30	17.26
N		19	19	19

UDS=UNABLE TO DELIVER, SAC'D

PR.NO.60R0375/88R002: REPRODUCTIVE TOX. STUDY TO DETECT EFFECTS
OF MIXED ANTI-ANDROGENIC SUBSTANCES IN RATS; ORAL ADM. (GAVAGE)
INDIVIDUAL MATERNAL FOOD CONSUMPTION DURING LACTATION -- GRAMS/ANIMAL/DAY

TEST GROUP 4(0.00025 MG/KG BW/D)

FEMALE#		DAY OF LACTATION 0 - 7	7 - 14	14 - 21
101		35.2	53.7	67.3
102		29.9	42.8	60.7
103		27.2	44.8	57.6
104		36.4	56.6	65.4
105		26.9	44.8	56.9
106		32.4	53.8	61.5
107		33.0	52.0	65.4
108		30.0	47.4	61.3
109		28.4	49.0	59.2
110		10.2	11.1	13.0
111		35.4	51.9	64.0
117		28.6	45.5	58.4
118		35.7	54.6	66.7
119		34.9	56.4	65.9
120		24.8	42.1	49.7
121		33.9	56.4	66.1
122x	NP			
123		29.1	51.5	64.6
124		33.2	56.8	68.6
125		31.4	51.2	67.5
MEAN		30.3	48.6	60.0
S.D.		5.94	10.27	12.32
N		19	19	19

NP=NOT PREGNANT x=EXCLUDED FROM MEAN

PR.NO.60R0375/88R002: REPRODUCTIVE TOX. STUDY TO DETECT EFFECTS
OF MIXED ANTI-ANDROGENIC SUBSTANCES IN RATS; ORAL ADM. (GAVAGE)
INDIVIDUAL MATERNAL BODY WEIGHTS DURING GESTATION -- GRAMS

TEST GROUP 0 (0 MG/KG BW/D)

	DAY OF GESTATION			
FEMALE#	0	6	13	20
1	155.6	185.8	218.9	273.8
2	170.7	199.1	226.2	279.6
3	180.3	207.1	231.6	288.6
4	168.7	200.6	227.7	296.0
5	170.8	198.5	229.7	299.4
6	158.8	177.8	201.1	258.0
7	184.4	215.2	247.7	309.7
8	161.7	187.2	215.1	269.2
9	156.7	182.0	207.9	258.6
10	165.4	192.9	225.5	281.9
11	172.8	190.7	216.8	289.4
12	182.8	205.7	232.5	294.9
13	161.7	187.5	212.2	251.7
14	184.1	215.6	248.9	308.4
15	189.8	233.9	281.2	359.2
16	157.4	192.9	222.9	277.8
17	160.6	191.5	224.4	278.0
18	164.5	195.4	232.5	298.1
19	156.2	183.7	209.5	259.3
20	155.8	181.0	206.1	262.0
21	177.2	209.3	246.0	321.5
22	161.4	191.8	228.8	292.5
23	182.0	222.6	261.5	327.9
24	167.9	197.2	234.2	295.1
25	173.6	198.3	230.0	291.3
MEAN	168.8	197.7	228.8	288.9
S.D.	10.55	13.70	18.04	24.60
N	25	25	25	25

PR.NO.60R0375/88R002: REPRODUCTIVE TOX. STUDY TO DETECT EFFECTS
OF MIXED ANTI-ANDROGENIC SUBSTANCES IN RATS; ORAL ADM. (GAVAGE)
INDIVIDUAL MATERNAL BODY WEIGHTS DURING GESTATION -- GRAMS

TEST GROUP 1 (ADI-MIX)

	DAY OF GESTATION			
FEMALE#	0	6	13	20
26	165.2	190.1	222.2	282.8
27	184.6	228.1	267.0	338.3
28	181.3	210.9	244.0	302.9
29	194.0	218.6	252.8	327.5
30	172.4	206.6	239.6	310.8
31	175.6	208.1	242.0	308.0
32	182.2	213.0	246.3	302.3
33	150.7	185.0	225.3	297.1
34	168.2	187.8	205.6	264.9
35	184.3	222.4	262.8	325.2
36	186.6	217.8	248.7	313.6
37	157.1	175.4	202.6	260.7
38	174.9	202.4	230.6	289.0
39	153.6	180.4	212.3	268.1
40	166.8	190.4	222.4	298.0
41	187.1	219.9	246.4	318.1
42	165.5	195.7	237.5	305.3
43	182.3	202.1	229.2	291.5
44	153.5	182.5	219.0	280.5
45	156.4	182.8	211.4	272.4
46	184.6	212.0	240.9	315.3
47	171.9	197.8	231.4	275.7
48	165.0	190.8	222.3	280.1
49	152.4	189.4	228.6	302.6
50	148.8	178.2	206.6	259.8
MEAN	170.6	199.5	231.9	295.6
S.D.	13.49	15.47	17.37	21.79
N	25	25	25	25

PR.NO.60R0375/88R002: REPRODUCTIVE TOX. STUDY TO DETECT EFFECTS
OF MIXED ANTI-ANDROGENIC SUBSTANCES IN RATS; ORAL ADM. (GAVAGE)
INDIVIDUAL MATERNAL BODY WEIGHTS DURING GESTATION -- GRAMS

TEST GROUP 2 (NOAEL-MIX)

	DAY OF GESTATION			
FEMALE#	0	6	13	20
51	161.3	186.1	213.5	273.2
52	196.1	227.4	262.3	328.7
53	173.7	199.4	226.1	282.5
54	176.9	199.7	233.9	303.9
55	169.1	196.3	228.9	298.0
56	172.6	207.6	227.0	295.9
57	179.6	219.9	254.6	325.2
58	161.3	182.2	206.1	263.9
59	190.7	217.7	251.1	309.3
60	162.1	198.7	237.5	307.8
61	163.8	200.5	231.0	295.9
62	176.8	200.2	223.1	279.0
63	189.9	213.8	242.1	300.9
64	168.7	203.0	231.0	285.7
65	162.9	187.1	217.9	280.3
66	180.3	208.6	242.3	313.3
67	176.5	206.8	228.7	248.0
68	165.6	193.9	226.4	293.4
69	153.8	179.6	206.1	265.0
70	168.1	195.2	227.9	276.7
71	146.6	171.1	201.9	263.4
72	173.1	211.5	250.3	323.3
73	155.2	173.9	203.9	257.0
74	157.1	179.3	205.4	261.5
75	152.5	181.3	212.6	267.3
MEAN	169.4	197.6	227.7	288.0
S.D.	12.40	14.85	16.80	22.71
N	25	25	25	25

PR.NO.60R0375/88R002: REPRODUCTIVE TOX. STUDY TO DETECT EFFECTS
OF MIXED ANTI-ANDROGENIC SUBSTANCES IN RATS; ORAL ADM. (GAVAGE)
INDIVIDUAL MATERNAL BODY WEIGHTS DURING GESTATION -- GRAMS

TEST GROUP 3 (LOAEL-MIX)

		DAY OF GESTATION			
FEMALE#		0	6	13	20
76	UDS	183.6	217.6	246.2	327.1
77		180.3	212.3	242.2	298.6
78		185.5	218.0	248.9	320.5
79		184.4	211.4	240.0	304.8
80		148.2	174.5	203.4	261.8
81		174.1	203.0	231.3	295.9
82		178.9	205.6	232.2	296.5
83		166.5	194.7	224.8	283.0
84		156.5	182.4	203.6	250.1
85		182.7	211.3	235.0	300.5
86		181.1	210.1	244.5	315.1
87		180.3	217.0	247.1	329.2
88		181.2	207.8	230.3	283.9
89		165.5	194.5	226.0	301.3
90		165.7	198.3	226.2	284.5
91		175.4	203.8	238.5	316.1
92		153.1	188.3	217.6	274.4
93		148.8	183.7	213.8	262.0
94		172.3	202.5	239.6	305.1
95		169.6	202.2	238.0	304.7
96		154.7	185.8	220.5	290.6
97		161.2	188.6	223.8	284.2
98		164.5	189.0	220.3	287.2
99		152.6	184.6	212.2	272.7
100		154.0	195.9	227.7	287.2
MEAN		168.8	199.3	229.3	293.5
S.D.		12.41	12.38	12.98	20.19
N		25	25	25	25

UDS=UNABLE TO DELIVER, SAC'D

PR.NO.60R0375/88R002: REPRODUCTIVE TOX. STUDY TO DETECT EFFECTS
OF MIXED ANTI-ANDROGENIC SUBSTANCES IN RATS; ORAL ADM. (GAVAGE)
INDIVIDUAL MATERNAL BODY WEIGHTS DURING GESTATION -- GRAMS

TEST GROUP 4(0.00025 MG/KG BW/D)

FEMALE#		DAY OF GESTATION 0	6	13	20
101		181.4	209.5	239.4	302.3
102		175.4	204.7	232.7	291.6
103		176.4	206.1	235.1	301.2
104		178.1	213.1	246.5	310.6
105		184.9	217.2	243.5	294.2
106		182.2	211.1	242.3	305.5
107		175.4	209.1	243.3	306.0
108		157.5	177.7	181.7	251.7
109		176.2	206.8	236.8	295.2
110		163.9	187.0	209.4	229.6
111		187.6	212.8	251.4	316.9
112		183.2	216.7	246.1	321.4
113		172.6	203.8	237.3	295.0
114		159.8	189.2	232.1	296.7
115		175.4	206.9	235.0	295.2
116		166.3	200.8	244.7	323.9
117		154.8	179.1	208.6	261.5
118		165.2	202.3	238.9	305.9
119		162.0	191.0	232.1	296.5
120		158.4	184.5	225.3	279.3
121		176.8	214.8	250.9	333.8
122x	NP	157.4	181.3	192.0	203.6
123		159.6	189.9	227.0	282.7
124		161.5	199.1	239.3	303.8
125		148.5	178.5	214.1	280.2
MEAN		170.1	200.5	233.1	295.0
S.D.		10.79	12.68	15.94	23.02
N		24	24	24	24

NP=NOT PREGNANT x=EXCLUDED FROM MEAN

PR.NO.60R0375/88R002: REPRODUCTIVE TOX. STUDY TO DETECT EFFECTS
OF MIXED ANTI-ANDROGENIC SUBSTANCES IN RATS; ORAL ADM. (GAVAGE)
INDIVIDUAL MATERNAL BODY WEIGHT CHANGE DURING GESTATION -- GRAMS

TEST GROUP 0 (0 MG/KG BW/D)

	DAY OF GESTATION		
FEMALE#	0 - 6	6 - 13	13 - 20
1	30.2	33.1	54.9
2	28.4	27.1	53.4
3	26.8	24.5	57.0
4	31.9	27.1	68.3
5	27.7	31.2	69.7
6	19.0	23.3	56.9
7	30.8	32.5	62.0
8	25.5	27.9	54.1
9	25.3	25.9	50.7
10	27.5	32.6	56.4
11	17.9	26.1	72.6
12	22.9	26.8	62.4
13	25.8	24.7	39.5
14	31.5	33.3	59.5
15	44.1	47.3	78.0
16	35.5	30.0	54.9
17	30.9	32.9	53.6
18	30.9	37.1	65.6
19	27.5	25.8	49.8
20	25.2	25.1	55.9
21	32.1	36.7	75.5
22	30.4	37.0	63.7
23	40.6	38.9	66.4
24	29.3	37.0	60.9
25	24.7	31.7	61.3
MEAN	28.9	31.0	60.1
S.D.	5.71	5.77	8.70
N	25	25	25

PR.NO.60R0375/88R002: REPRODUCTIVE TOX. STUDY TO DETECT EFFECTS
OF MIXED ANTI-ANDROGENIC SUBSTANCES IN RATS; ORAL ADM. (GAVAGE)
INDIVIDUAL MATERNAL BODY WEIGHT CHANGE DURING GESTATION -- GRAMS

TEST GROUP 1 (ADI-MIX)

FEMALE#	DAY OF GESTATION 0 - 6	6 - 13	13 - 20
26	24.9	32.1	60.6
27	43.5	38.9	71.3
28	29.6	33.1	58.9
29	24.6	34.2	74.7
30	34.2	33.0	71.2
31	32.5	33.9	66.0
32	30.8	33.3	56.0
33	34.3	40.3	71.8
34	19.6	17.8	59.3
35	38.1	40.4	62.4
36	31.2	30.9	64.9
37	18.3	27.2	58.1
38	27.5	28.2	58.4
39	26.8	31.9	55.8
40	23.6	32.0	75.6
41	32.8	26.5	71.7
42	30.2	41.8	67.8
43	19.8	27.1	62.3
44	29.0	36.5	61.5
45	26.4	28.6	61.0
46	27.4	28.9	74.4
47	25.9	33.6	44.3
48	25.8	31.5	57.8
49	37.0	39.2	74.0
50	29.4	28.4	53.2
MEAN	28.9	32.4	63.7
S.D.	5.91	5.38	7.96
N	25	25	25

PR.NO.60R0375/88R002: REPRODUCTIVE TOX. STUDY TO DETECT EFFECTS
OF MIXED ANTI-ANDROGENIC SUBSTANCES IN RATS; ORAL ADM. (GAVAGE)
INDIVIDUAL MATERNAL BODY WEIGHT CHANGE DURING GESTATION -- GRAMS

TEST GROUP 2 (NOAEL-MIX)

	DAY OF GESTATION		
FEMALE#	0 - 6	6 - 13	13 - 20
51	24.8	27.4	59.7
52	31.3	34.9	66.4
53	25.7	26.7	56.4
54	22.8	34.2	70.0
55	27.2	32.6	69.1
56	35.0	19.4	68.9
57	40.3	34.7	70.6
58	20.9	23.9	57.8
59	27.0	33.4	58.2
60	36.6	38.8	70.3
61	36.7	30.5	64.9
62	23.4	22.9	55.9
63	23.9	28.3	58.8
64	34.3	28.0	54.7
65	24.2	30.8	62.4
66	28.3	33.7	71.0
67	30.3	21.9	19.3
68	28.3	32.5	67.0
69	25.8	26.5	58.9
70	27.1	32.7	48.8
71	24.5	30.8	61.5
72	38.4	38.8	73.0
73	18.7	30.0	53.1
74	22.2	26.1	56.1
75	28.8	31.3	54.7
MEAN	28.3	30.0	60.3
S.D.	5.77	4.94	10.86
N	25	25	25

PR.NO.60R0375/88R002: REPRODUCTIVE TOX. STUDY TO DETECT EFFECTS
OF MIXED ANTI-ANDROGENIC SUBSTANCES IN RATS; ORAL ADM. (GAVAGE)
INDIVIDUAL MATERNAL BODY WEIGHT CHANGE DURING GESTATION -- GRAMS

TEST GROUP 3 (LOAEL-MIX)

FEMALE#		DAY OF GESTATION 0 - 6	6 - 13	13 - 20
76	UDS	34.0	28.6	80.9
77		32.0	29.9	56.4
78		32.5	30.9	71.6
79		27.0	28.6	64.8
80		26.3	28.9	58.4
81		28.9	28.3	64.6
82		26.7	26.6	64.3
83		28.2	30.1	58.2
84		25.9	21.2	46.5
85		28.6	23.7	65.5
86		29.0	34.4	70.6
87		36.7	30.1	82.1
88		26.6	22.5	53.6
89		29.0	31.5	75.3
90		32.6	27.9	58.3
91		28.4	34.7	77.6
92		35.2	29.3	56.8
93		34.9	30.1	48.2
94		30.2	37.1	65.5
95		32.6	35.8	66.7
96		31.1	34.7	70.1
97		27.4	35.2	60.4
98		24.5	31.3	66.9
99		32.0	27.6	60.5
100		41.9	31.8	59.5
MEAN		30.5	30.0	64.1
S.D.		4.01	4.01	9.13
N		25	25	25

UDS=UNABLE TO DELIVER, SAC'D

PR.NO.60R0375/88R002: REPRODUCTIVE TOX. STUDY TO DETECT EFFECTS
OF MIXED ANTI-ANDROGENIC SUBSTANCES IN RATS; ORAL ADM. (GAVAGE)
INDIVIDUAL MATERNAL BODY WEIGHT CHANGE DURING GESTATION -- GRAMS

TEST GROUP 4(0.00025 MG/KG BW/D)

FEMALE#		DAY OF GESTATION 0 - 6	6 - 13	13 - 20
101		28.1	29.9	62.9
102		29.3	28.0	58.9
103		29.7	29.0	66.1
104		35.0	33.4	64.1
105		32.3	26.3	50.7
106		28.9	31.2	63.2
107		33.7	34.2	62.7
108		20.2	4.0	70.0
109		30.6	30.0	58.4
110		23.1	22.4	20.2
111		25.2	38.6	65.5
112		33.5	29.4	75.3
113		31.2	33.5	57.7
114		29.4	42.9	64.6
115		31.5	28.1	60.2
116		34.5	43.9	79.2
117		24.3	29.5	52.9
118		37.1	36.6	67.0
119		29.0	41.1	64.4
120		26.1	40.8	54.0
121		38.0	36.1	82.9
122x	NP	23.9	10.7	11.6
123		30.3	37.1	55.7
124		37.6	40.2	64.5
125		30.0	35.6	66.1
MEAN		30.4	32.6	62.0
S.D.		4.52	8.29	11.75
N		24	24	24

NP=NOT PREGNANT x=EXCLUDED FROM MEAN

PR.NO.60R0375/88R002: REPRODUCTIVE TOX. STUDY TO DETECT EFFECTS
OF MIXED ANTI-ANDROGENIC SUBSTANCES IN RATS; ORAL ADM. (GAVAGE)
INDIVIDUAL MATERNAL BODY WEIGHTS DURING LACTATION -- GRAMS

TEST GROUP 0 (0 MG/KG BW/D)

	DAY OF LACTATION								
FEMALE#	0	7	14	21	28	35	42	49	56
1	216.5	243.8	255.3	244.7	213.4	227.2	233.7		
2	225.6	243.0	263.6	254.9	213.9	223.5	227.8		
3	234.1	244.3	257.4	241.0	227.8	229.3	240.0		
4	228.8	247.4	246.8	256.6	224.8	231.7	232.9		
5	239.0	263.5	275.4	268.4	230.6	237.4	240.0		
6	200.8	222.4	233.9	229.3	196.5	201.2	209.4		
7	258.1	280.3	281.9	281.8	243.4	249.1	252.0		
8	203.2	237.7	256.2	251.1	219.2	220.5	225.0		
9	216.9	220.4	238.4	235.3	195.7	209.2	208.8		
10	214.7	242.8	240.1	235.5	212.0	223.8	228.9		
11	214.4	235.6	242.9	221.8	209.6	224.9	225.2	229.5	
17	218.5	240.9	253.4	248.0	220.4	224.6	238.0	237.8	
18	233.7	262.3	269.3	260.1	217.4	232.7	240.3	244.2	
19	201.9	220.5	229.3	235.1	204.9	208.3	216.3	221.8	
20	199.5	230.9	239.3	251.7	204.0	205.8	207.7	211.3	
21	235.0	264.8	265.7	261.4	233.9	252.4	254.2	249.8	
22	237.2	236.8	246.1	244.7	215.4	230.5	236.1	236.1	
23	269.4	291.9	273.8	282.4	243.8	253.7	260.3	264.0	
24	228.6	259.9	281.5	273.8	233.5	260.2	260.1	254.7	
25	221.6	248.9	266.7	256.0	225.5	239.2	243.1	242.2	
MEAN	224.9	246.9	255.9	251.7	219.3	229.3	234.0	239.1	
S.D.	18.29	18.92	16.00	16.64	13.79	16.27	15.88	15.59	
N	20	20	20	20	20	20	20	10	

PR.NO.60R0375/88R002: REPRODUCTIVE TOX. STUDY TO DETECT EFFECTS
OF MIXED ANTI-ANDROGENIC SUBSTANCES IN RATS; ORAL ADM. (GAVAGE)
INDIVIDUAL MATERNAL BODY WEIGHTS DURING LACTATION -- GRAMS

TEST GROUP 1 (ADI-MIX)

	DAY OF LACTATION								
FEMALE#	0	7	14	21	28	35	42	49	56
26	231.9	251.9	257.8	257.2	216.2	228.8	230.5		
27	270.6	285.2	296.9	263.9	255.4	263.3	265.1		
28	244.7	260.8	275.7	264.7	239.0				
29	253.6	279.0	290.1	276.7	243.8	252.1	261.2		
30	243.7	266.6	292.6	262.6	231.5	236.7	240.8	242.0	
31	232.6	264.0	267.0	256.2	233.6	242.3	248.5		
32	248.4	261.3	278.0	267.3	246.8	258.0	267.6		
33	235.5	255.6	275.4	261.1	230.9	236.3	245.5		
34	202.3	234.1	250.8	244.7	213.5	216.1	221.3		
35	246.0	266.9	290.1	260.4	257.9	260.5	266.7		
36	249.7	274.0	272.7	266.6	244.9	250.6	246.8	242.5	
37	209.8	220.9	235.1	224.9	209.6	210.8	213.9	213.1	
43	225.5	249.7	250.4	244.7	217.9	221.9	225.6	227.5	
44	217.6	243.6	258.8	251.3	222.7	239.3	236.2	236.7	
45	215.0	238.8	264.4	262.1	210.1	220.5	223.6	222.9	
46	248.5	267.3	280.5	266.0	234.3	238.2	243.3	243.3	
47	227.3	242.0	254.3	268.1	234.9	251.5	243.6	243.2	
48	229.7	251.0	261.8	248.2	223.9	229.5	235.6	231.8	
49	234.4	260.8	273.2	264.7	225.5	236.9	247.3	251.2	
50	200.0	218.6	247.7	245.6	214.8	225.1	222.7	226.1	
MEAN	233.3	254.6	268.7	257.9	230.4	237.8	241.4	234.6	
S.D.	18.14	17.77	16.79	11.70	14.54	15.31	16.12	11.30	
N	20	20	20	20	20	19	19	11	

PR.NO.60R0375/88R002: REPRODUCTIVE TOX. STUDY TO DETECT EFFECTS
OF MIXED ANTI-ANDROGENIC SUBSTANCES IN RATS; ORAL ADM. (GAVAGE)
INDIVIDUAL MATERNAL BODY WEIGHTS DURING LACTATION -- GRAMS

TEST GROUP 2 (NOAEL-MIX)

FEMALE#	DAY OF LACTATION 0	7	14	21	28	35	42	49	56
51	209.7	232.1	259.3	245.8	211.5	216.1	219.8		
52	261.2	277.4	282.7	272.9	239.2	250.7	256.9		
53	223.3	235.4	249.0	236.0	215.0	218.9	221.6		
54	232.3	254.1	271.0	269.2	230.6	234.2	241.0		
55	224.4	241.6	260.8	261.1	228.7	230.8	241.1		
56	222.6	227.1	246.1	250.9	234.2	239.1	241.5		
57	254.9	267.0	279.7	262.1	237.5	236.9	253.6		
58	203.4	236.4	238.9	244.3	210.7	215.8	220.5		
59	270.2	278.7	298.6	276.5	243.1	257.2	260.8		
60	230.2	264.9	266.5	250.6	236.9	244.1	261.9		
61	227.1	252.8	269.5	272.8	228.5	241.5	248.1	245.2	
62	215.7	232.8	257.0	257.1	232.3	233.7	228.0	236.6	
68	219.8	250.0	256.0	260.8	216.7	231.2	236.9	235.6	
69	205.2	232.7	241.1	240.3	206.9	218.8	220.5	220.9	
70	229.0	232.2	251.3	246.1	223.5	239.5	244.3	240.6	
71	199.2	227.1	232.5	240.5	204.8	205.8	213.0	213.7	
72	244.4	264.1	284.0	274.4	233.8	251.3	257.0	254.0	
73	194.1	223.2	228.6	228.5	200.6	212.9	210.6		
74	187.0	222.1	238.3	238.1	200.7	216.1	215.7		
75	216.4	236.9	262.7	239.0	225.9	233.8	233.6	236.1	
MEAN	223.5	244.4	258.7	253.4	223.1	231.4	236.3	235.3	
S.D.	21.80	17.93	18.68	14.68	13.58	14.32	16.82	12.83	
N	20	20	20	20	20	20	20	8	

PR.NO.60R0375/88R002: REPRODUCTIVE TOX. STUDY TO DETECT EFFECTS
OF MIXED ANTI-ANDROGENIC SUBSTANCES IN RATS; ORAL ADM. (GAVAGE)
INDIVIDUAL MATERNAL BODY WEIGHTS DURING LACTATION -- GRAMS

TEST GROUP 3 (LOAEL-MIX)

	DAY OF LACTATION								
FEMALE#	0	7	14	21	28	35	42	49	56
76 UDS									
77	248.7	251.8	261.1	281.0	237.6	244.5	256.1		
78	243.6	252.4	278.7	256.5	242.8	243.3	245.3		
79	245.4	223.9	234.6	240.4	248.6	250.1	247.1		
80	206.3	206.4	223.9	229.3	217.3	227.4	228.9		
81	218.7	226.7	224.0	230.5	234.4	242.0	244.8		
82	221.9	259.9	267.7	259.2	227.9	232.7	243.6		
83	219.8	236.9	252.6	261.7	230.1	234.1	239.4		
84	198.7	213.9	227.8	226.8	195.8	202.3	202.9		
85	224.2	247.6	256.3	265.6	223.9	241.0	240.8		
86	223.7	236.5	268.9	260.7	237.6	242.0	238.7		
87	238.5	274.0	283.9	275.7	238.3	245.0	249.3	253.0	
93	218.4	238.9	254.6	242.8	220.0	227.9	235.2	238.1	
94	235.9	238.6	270.0	249.0	236.8	247.5	249.3	250.7	
95	239.0	258.7	280.6	280.6	249.5	251.1	251.8	256.6	
96	211.4	236.5	246.7	242.8	219.6	235.5	255.6	242.1	
97	215.4	236.4	245.3	245.7	214.9	235.8	244.3	231.1	
98	202.9	221.5	253.5	252.5	215.9	221.3	224.6		
99	197.1	229.6	243.3	259.5	217.3	218.9	216.2		
100	229.7	255.8	266.0	269.7	231.3	244.2	247.4		
MEAN	223.1	239.3	254.7	254.2	228.4	236.1	240.1	245.3	
S.D.	15.78	17.06	18.53	16.48	13.46	12.31	13.60	9.80	
N	19	19	19	19	19	19	19	6	

UDS=UNABLE TO DELIVER, SAC'D

PR.NO.60R0375/88R002: REPRODUCTIVE TOX. STUDY TO DETECT EFFECTS
OF MIXED ANTI-ANDROGENIC SUBSTANCES IN RATS; ORAL ADM. (GAVAGE)
INDIVIDUAL MATERNAL BODY WEIGHTS DURING LACTATION -- GRAMS

TEST GROUP 4(0.00025 MG/KG BW/D)

	DAY OF LACTATION								
FEMALE#	0	7	14	21	28	35	42	49	56
101	239.4	266.3	274.4	265.1	254.3	263.5	270.3		
102	227.2	253.3	260.9	259.2	228.3	235.5	231.7		
103	245.5	246.2	256.8	257.3	228.0	238.7	241.0	244.4	
104	249.2	267.9	277.2	263.1	236.8	246.9	252.4		
105	241.6	251.7	265.4	261.8	241.0	249.4	249.8		
106	231.3	259.4	273.5	272.0	233.0	242.8	245.3		
107	244.6	259.1	281.2	269.3	239.5	241.2	250.5		
108	199.9	219.7	233.9	222.9	199.5	196.2	199.8		
109	236.2	240.0	265.5	247.8	237.8	247.0	242.1		
110	212.5	208.7	214.5	207.8	217.3	222.5	222.3		
111	244.4	274.0	251.3	268.3	239.8	256.5	263.0	265.3	
117	200.5	229.0	236.8	223.3	195.4	203.4	207.0	205.9	
118	234.9	256.5	270.1	267.0	222.0	230.7	238.1	236.2	
119	233.0	252.1	284.4	278.5	240.8	250.4	258.1	249.1	
120	218.7	236.2	253.6	234.7	231.0	247.6	238.8	247.3	
121	264.9	270.4	279.0	280.6	245.5	255.1	262.9	262.0	
122x NP									
123	237.9	246.9	263.4	248.3	227.0	232.2	232.3	228.0	
124	237.6	261.5	275.3	273.9	239.2	253.3	264.5	264.4	
125	223.8	240.0	245.8	252.9	218.4	219.5	225.9	225.5	
MEAN	232.8	249.4	261.2	255.5	230.2	238.5	241.9	242.8	
S.D.	16.28	17.34	18.39	20.26	14.83	17.80	19.11	19.23	
N	19	19	19	19	19	19	19	10	

NP=NOT PREGNANT x=EXCLUDED FROM MEAN

PR.NO.60R0375/88R002: REPRODUCTIVE TOX. STUDY TO DETECT EFFECTS
OF MIXED ANTI-ANDROGENIC SUBSTANCES IN RATS; ORAL ADM. (GAVAGE)
INDIVIDUAL MATERNAL BODY WEIGHT CHANGE DURING LACTATION -- GRAMS

TEST GROUP 0 (0 MG/KG BW/D)

	DAY OF LACTATION							
FEMALE#	0 - 7	7 - 14	14 - 21	21 - 28	28 - 35	35 - 42	42 - 49	49 - 56
1	27.3	11.5	-10.6	-31.3	13.8	6.5		
2	17.4	20.6	-8.7	-41.0	9.6	4.3		
3	10.2	13.1	-16.4	-13.2	1.5	10.7		
4	18.6	-0.6	9.8	-31.8	6.9	1.2		
5	24.5	11.9	-7.0	-37.8	6.8	2.6		
6	21.6	11.5	-4.6	-32.8	4.7	8.2		
7	22.2	1.6	-0.1	-38.4	5.7	2.9		
8	34.5	18.5	-5.1	-31.9	1.3	4.5		
9	3.5	18.0	-3.1	-39.6	13.5	-0.4		
10	28.1	-2.7	-4.6	-23.5	11.8	5.1		
11	21.2	7.3	-21.1	-12.2	15.3	0.3	4.3	
17	22.4	12.5	-5.4	-27.6	4.2	13.4	-0.2	
18	28.6	7.0	-9.2	-42.7	15.3	7.6	3.9	
19	18.6	8.8	5.8	-30.2	3.4	8.0	5.5	
20	31.4	8.4	12.4	-47.7	1.8	1.9	3.6	
21	29.8	0.9	-4.3	-27.5	18.5	1.8	-4.4	
22	-0.4	9.3	-1.4	-29.3	15.1	5.6	0.0	
23	22.5	-18.1	8.6	-38.6	9.9	6.6	3.7	
24	31.3	21.6	-7.7	-40.3	26.7	-0.1	-5.4	
25	27.3	17.8	-10.7	-30.5	13.7	3.9	-0.9	
MEAN	22.0	8.9	-4.2	-32.4	10.0	4.7	1.0	
S.D.	9.07	9.41	8.42	9.05	6.63	3.67	3.80	
N	20	20	20	20	20	20	10	

PR.NO.60R0375/88R002: REPRODUCTIVE TOX. STUDY TO DETECT EFFECTS
OF MIXED ANTI-ANDROGENIC SUBSTANCES IN RATS; ORAL ADM. (GAVAGE)
INDIVIDUAL MATERNAL BODY WEIGHT CHANGE DURING LACTATION -- GRAMS

TEST GROUP 1 (ADI-MIX)

	DAY OF LACTATION							
FEMALE#	0 - 7	7 - 14	14 - 21	21 - 28	28 - 35	35 - 42	42 - 49	49 - 56
26	20.0	5.9	-0.6	-41.0	12.6	1.7		
27	14.6	11.7	-33.0	-8.5	7.9	1.8		
28	16.1	14.9	-11.0	-25.7				
29	25.4	11.1	-13.4	-32.9	8.3	9.1		
30	22.9	26.0	-30.0	-31.1	5.2	4.1	1.2	
31	31.4	3.0	-10.8	-22.6	8.7	6.2		
32	12.9	16.7	-10.7	-20.5	11.2	9.6		
33	20.1	19.8	-14.3	-30.2	5.4	9.2		
34	31.8	16.7	-6.1	-31.2	2.6	5.2		
35	20.9	23.2	-29.7	-2.5	2.6	6.2		
36	24.3	-1.3	-6.1	-21.7	5.7	-3.8	-4.3	
37	11.1	14.2	-10.2	-15.3	1.2	3.1	-0.8	
43	24.2	0.7	-5.7	-26.8	4.0	3.7	1.9	
44	26.0	15.2	-7.5	-28.6	16.6	-3.1	0.5	
45	23.8	25.6	-2.3	-52.0	10.4	3.1	-0.7	
46	18.8	13.2	-14.5	-31.7	3.9	5.1	0.0	
47	14.7	12.3	13.8	-33.2	16.6	-7.9	-0.4	
48	21.3	10.8	-13.6	-24.3	5.6	6.1	-3.8	
49	26.4	12.4	-8.5	-39.2	11.4	10.4	3.9	
50	18.6	29.1	-2.1	-30.8	10.3	-2.4	3.4	
MEAN	21.3	14.1	-10.8	-27.5	7.9	3.5	0.1	
S.D.	5.67	8.17	10.77	11.00	4.51	4.97	2.57	
N	20	20	20	20	19	19	11	

PR.NO.60R0375/88R002: REPRODUCTIVE TOX. STUDY TO DETECT EFFECTS
OF MIXED ANTI-ANDROGENIC SUBSTANCES IN RATS; ORAL ADM. (GAVAGE)
INDIVIDUAL MATERNAL BODY WEIGHT CHANGE DURING LACTATION -- GRAMS

TEST GROUP 2 (NOAEL-MIX)

	DAY OF LACTATION							
FEMALE#	0 - 7	7 - 14	14 - 21	21 - 28	28 - 35	35 - 42	42 - 49	49 - 56
51	22.4	27.2	-13.5	-34.3	4.6	3.7		
52	16.2	5.3	-9.8	-33.7	11.5	6.2		
53	12.1	13.6	-13.0	-21.0	3.9	2.7		
54	21.8	16.9	-1.8	-38.6	3.6	6.8		
55	17.2	19.2	0.3	-32.4	2.1	10.3		
56	4.5	19.0	4.8	-16.7	4.9	2.4		
57	12.1	12.7	-17.6	-24.6	-0.6	16.7		
58	33.0	2.5	5.4	-33.6	5.1	4.7		
59	8.5	19.9	-22.1	-33.4	14.1	3.6		
60	34.7	1.6	-15.9	-13.7	7.2	17.8		
61	25.7	16.7	3.3	-44.3	13.0	6.6	-2.9	
62	17.1	24.2	0.1	-24.8	1.4	-5.7	8.6	
68	30.2	6.0	4.8	-44.1	14.5	5.7	-1.3	
69	27.5	8.4	-0.8	-33.4	11.9	1.7	0.4	
70	3.2	19.1	-5.2	-22.6	16.0	4.8	-3.7	
71	27.9	5.4	8.0	-35.7	1.0	7.2	0.7	
72	19.7	19.9	-9.6	-40.6	17.5	5.7	-3.0	
73	29.1	5.4	-0.1	-27.9	12.3	-2.3		
74	35.1	16.2	-0.2	-37.4	15.4	-0.4		
75	20.5	25.8	-23.7	-13.1	7.9	-0.2	2.5	
MEAN	20.9	14.3	-5.3	-30.3	8.4	4.9	0.2	
S.D.	9.61	7.92	9.64	9.39	5.73	5.58	4.03	
N	20	20	20	20	20	20	8	

PR.NO.60R0375/88R002: REPRODUCTIVE TOX. STUDY TO DETECT EFFECTS
OF MIXED ANTI-ANDROGENIC SUBSTANCES IN RATS; ORAL ADM. (GAVAGE)
INDIVIDUAL MATERNAL BODY WEIGHT CHANGE DURING LACTATION -- GRAMS

TEST GROUP 3 (LOAEL-MIX)

	DAY OF LACTATION							
FEMALE#	0 - 7	7 - 14	14 - 21	21 - 28	28 - 35	35 - 42	42 - 49	49 - 56
76	UDS							
77	3.1	9.3	19.9	-43.4	6.9	11.6		
78	8.8	26.3	-22.2	-13.7	0.5	2.0		
79	-21.5	10.7	5.8	8.2	1.5	-3.0		
80	0.1	17.5	5.4	-12.0	10.1	1.5		
81	8.0	-2.7	6.5	3.9	7.6	2.8		
82	38.0	7.8	-8.5	-31.3	4.8	10.9		
83	17.1	15.7	9.1	-31.6	4.0	5.3		
84	15.2	13.9	-1.0	-31.0	6.5	0.6		
85	23.4	8.7	9.3	-41.7	17.1	-0.2		
86	12.8	32.4	-8.2	-23.1	4.4	-3.3		
87	35.5	9.9	-8.2	-37.4	6.7	4.3	3.7	
93	20.5	15.7	-11.8	-22.8	7.9	7.3	2.9	
94	2.7	31.4	-21.0	-12.2	10.7	1.8	1.4	
95	19.7	21.9	0.0	-31.1	1.6	0.7	4.8	
96	25.1	10.2	-3.9	-23.2	15.9	20.1	-13.5	
97	21.0	8.9	0.4	-30.8	20.9	8.5	-13.2	
98	18.6	32.0	-1.0	-36.6	5.4	3.3		
99	32.5	13.7	16.2	-42.2	1.6	-2.7		
100	26.1	10.2	3.7	-38.4	12.9	3.2		
MEAN	16.1	15.4	-0.5	-25.8	7.7	3.9	-2.3	
S.D.	14.09	9.45	11.15	14.85	5.67	5.78	8.62	
N	19	19	19	19	19	19	6	

UDS=UNABLE TO DELIVER, SAC'D

PR.NO.60R0375/88R002: REPRODUCTIVE TOX. STUDY TO DETECT EFFECTS
OF MIXED ANTI-ANDROGENIC SUBSTANCES IN RATS; ORAL ADM. (GAVAGE)
INDIVIDUAL MATERNAL BODY WEIGHT CHANGE DURING LACTATION -- GRAMS

TEST GROUP 4(0.00025 MG/KG BW/D)

	DAY OF LACTATION							
FEMALE#	0 - 7	7 - 14	14 - 21	21 - 28	28 - 35	35 - 42	42 - 49	49 - 56
101	26.9	8.1	-9.3	-10.8	9.2	6.8		
102	26.1	7.6	-1.7	-30.9	7.2	-3.8		
103	0.7	10.6	0.5	-29.3	10.7	2.3	3.4	
104	18.7	9.3	-14.1	-26.3	10.1	5.5		
105	10.1	13.7	-3.6	-20.8	8.4	0.4		
106	28.1	14.1	-1.5	-39.0	9.8	2.5		
107	14.5	22.1	-11.9	-29.8	1.7	9.3		
108	19.8	14.2	-11.0	-23.4	-3.3	3.6		
109	3.8	25.5	-17.7	-10.0	9.2	-4.9		
110	-3.8	5.8	-6.7	9.5	5.2	-0.2		
111	29.6	-22.7	17.0	-28.5	16.7	6.5	2.3	
117	28.5	7.8	-13.5	-27.9	8.0	3.6	-1.1	
118	21.6	13.6	-3.1	-45.0	8.7	7.4	-1.9	
119	19.1	32.3	-5.9	-37.7	9.6	7.7	-9.0	
120	17.5	17.4	-18.9	-3.7	16.6	-8.8	8.5	
121	5.5	8.6	1.6	-35.1	9.6	7.8	-0.9	
122x NP								
123	9.0	16.5	-15.1	-21.3	5.2	0.1	-4.3	
124	23.9	13.8	-1.4	-34.7	14.1	11.2	-0.1	
125	16.2	5.8	7.1	-34.5	1.1	6.4	-0.4	
MEAN	16.6	11.8	-5.7	-25.2	8.3	3.3	-0.4	
S.D.	10.00	10.84	8.98	13.43	4.94	5.21	4.63	
N	19	19	19	19	19	19	10	

NP=NOT PREGNANT x=EXCLUDED FROM MEAN

PR.NO.60R0375/88R002: REPRODUCTIVE TOX. STUDY TO DETECT EFFECTS
OF MIXED ANTI-ANDROGENIC SUBSTANCES IN RATS; ORAL ADM. (GAVAGE)
INDIVIDUAL MATERNAL NECROPSY OBSERVATIONS

TEST GROUP 0 (0 MG/KG BW/D)

ANIMAL#	OBSERVATION	POSITION/GRADE
12	NOTHING ABNORMAL DETECTED	
13	NOTHING ABNORMAL DETECTED	
14	NOTHING ABNORMAL DETECTED	
15	NOTHING ABNORMAL DETECTED	
16	NOTHING ABNORMAL DETECTED	

POSITION/GRADE CODE: R-RIGHT, L-LEFT, B-BILATE , 1-SLIGHT, 2-MODERATE, 3-MARKED, P-PRESENT

PR.NO.60R0375/88R002: REPRODUCTIVE TOX. STUDY TO DETECT EFFECTS
OF MIXED ANTI-ANDROGENIC SUBSTANCES IN RATS; ORAL ADM. (GAVAGE)
INDIVIDUAL MATERNAL NECROPSY OBSERVATIONS

TEST GROUP 1 (ADI-MIX)

ANIMAL#	OBSERVATION	POSITION/GRADE
38	NOTHING ABNORMAL DETECTED	
39	NOTHING ABNORMAL DETECTED	
40	NOTHING ABNORMAL DETECTED	
41	NOTHING ABNORMAL DETECTED	
42	NOTHING ABNORMAL DETECTED	

POSITION/GRADE CODE: R-RIGHT, L-LEFT, B-BILATE , 1-SLIGHT, 2-MODERATE, 3-MARKED, P-PRESENT

PR.NO.60R0375/88R002: REPRODUCTIVE TOX. STUDY TO DETECT EFFECTS
OF MIXED ANTI-ANDROGENIC SUBSTANCES IN RATS; ORAL ADM. (GAVAGE)
INDIVIDUAL MATERNAL NECROPSY OBSERVATIONS

TEST GROUP 2 (NOAEL-MIX)

ANIMAL#	OBSERVATION	POSITION/GRADE
63	NOTHING ABNORMAL DETECTED	
64	NOTHING ABNORMAL DETECTED	
65	NOTHING ABNORMAL DETECTED	
66	NOTHING ABNORMAL DETECTED	
67	NOTHING ABNORMAL DETECTED	

POSITION/GRADE CODE: R-RIGHT, L-LEFT, B-BILATE , 1-SLIGHT, 2-MODERATE, 3-MARKED, P-PRESENT

PR.NO.60R0375/88R002: REPRODUCTIVE TOX. STUDY TO DETECT EFFECTS
OF MIXED ANTI-ANDROGENIC SUBSTANCES IN RATS; ORAL ADM. (GAVAGE)
INDIVIDUAL MATERNAL NECROPSY OBSERVATIONS

TEST GROUP 3 (LOAEL-MIX)

ANIMAL#	OBSERVATION	POSITION/GRADE
88	NOTHING ABNORMAL DETECTED	
89	NOTHING ABNORMAL DETECTED	
90	NOTHING ABNORMAL DETECTED	
91	NOTHING ABNORMAL DETECTED	
92	NOTHING ABNORMAL DETECTED	

POSITION/GRADE CODE: R-RIGHT, L-LEFT, B-BILATE , 1-SLIGHT, 2-MODERATE, 3-MARKED, P-PRESENT

PR.NO.60R0375/88R002: REPRODUCTIVE TOX. STUDY TO DETECT EFFECTS
OF MIXED ANTI-ANDROGENIC SUBSTANCES IN RATS; ORAL ADM. (GAVAGE)
INDIVIDUAL MATERNAL NECROPSY OBSERVATIONS

TEST GROUP 4(0.00025 MG/KG BW/D)

ANIMAL#		OBSERVATION	POSITION/GRADE
112		NOTHING ABNORMAL DETECTED	
113		NOTHING ABNORMAL DETECTED	
114		NOTHING ABNORMAL DETECTED	
115		NOTHING ABNORMAL DETECTED	
116		NOTHING ABNORMAL DETECTED	
122	NOT PREGNANT	NOTHING ABNORMAL DETECTED	

POSITION/GRADE CODE: R-RIGHT, L-LEFT, B-BILATE , 1-SLIGHT, 2-MODERATE, 3-MARKED, P-PRESENT

PR.NO.60R0375/88R002: REPRODUCTIVE TOX. STUDY TO DETECT EFFECTS
OF MIXED ANTI-ANDROGENIC SUBSTANCES IN RATS; ORAL ADM. (GAVAGE)
INDIVIDUAL REPRODUCTION DATA

TEST GROUP 0 (0 MG/KG BW/D)

	CORPORA	IMPLANT	RESORPTIONS			FETUSES		SEX		AVERAGE FETAL BODY WEIGHT			%IMPLANTATION LOSS	
FEMALE#	LUTEA	SITES	EARLY	LATE	TOTAL	DEAD	LIVE	MALE	FEMALE	MALES	FEMALES	LITTER	PRE	POST
12	13	13	1	0	1	0	12	6	6	3.6	3.4	3.5	0.0	7.7
13	8	6	0	1	1	0	5	3	2	3.7	3.5	3.6	25.0	16.7
14	11	11	0	0	0	0	11	6	5	3.7	3.5	3.6	0.0	0.0
15	11	10	0	0	0	0	10	5	5	3.9	3.8	3.9	9.1	0.0
16	10	9	1	0	1	0	8	4	4	3.6	3.4	3.5	10.0	11.1
MEAN	10.6	9.8	0.4	0.2	0.6	0.0	9.2	4.8	4.4	3.7	3.5	3.6	8.8	7.1
S.D.	1.8	2.6	0.5	0.4	0.5	0.0	2.8	1.3	1.5	0.1	0.2	0.1	10.2	7.2
N	5	5	5	5	5	5	5	5	5	5	5	5	5	5

PR.NO.60R0375/88R002: REPRODUCTIVE TOX. STUDY TO DETECT EFFECTS
OF MIXED ANTI-ANDROGENIC SUBSTANCES IN RATS; ORAL ADM. (GAVAGE)
INDIVIDUAL REPRODUCTION DATA

TEST GROUP 1 (ADI-MIX)

	CORPORA	IMPLANT	RESORPTIONS			FETUSES		SEX		AVERAGE FETAL BODY WEIGHT			%IMPLANTATION LOSS	
FEMALE#	LUTEA	SITES	EARLY	LATE	TOTAL	DEAD	LIVE	MALE	FEMALE	MALES	FEMALES	LITTER	PRE	POST
38	11	9	0	0	0	0	9	4	5	3.6	3.4	3.5	18.2	0.0
39	10	10	1	0	1	0	9	7	2	3.5	3.4	3.5	0.0	10.0
40	15	12	0	0	0	0	12	8	4	3.6	3.1	3.4	20.0	0.0
41	13	13	0	0	0	0	13	7	6	3.3	3.2	3.2	0.0	0.0
42	14	13	1	0	1	0	12	7	5	3.3	3.2	3.2	7.1	7.7
MEAN	12.6	11.4	0.4	0.0	0.4	0.0	11.0	6.6	4.4	3.4	3.3	3.4	9.1	3.5
S.D.	2.1	1.8	0.5	0.0	0.5	0.0	1.9	1.5	1.5	0.2	0.1	0.1	9.6	4.9
N	5	5	5	5	5	5	5	5	5	5	5	5	5	5

PR.NO.60R0375/88R002: REPRODUCTIVE TOX. STUDY TO DETECT EFFECTS
OF MIXED ANTI-ANDROGENIC SUBSTANCES IN RATS; ORAL ADM. (GAVAGE)
INDIVIDUAL REPRODUCTION DATA

TEST GROUP 2 (NOAEL-MIX)

	CORPORA	IMPLANT	RESORPTIONS			FETUSES		SEX		AVERAGE FETAL BODY WEIGHT			%IMPLANTATION LOSS	
FEMALE#	LUTEA	SITES	EARLY	LATE	TOTAL	DEAD	LIVE	MALE	FEMALE	MALES	FEMALES	LITTER	PRE	POST
63	9	9	0	0	0	0	9	4	5	3.6	3.4	3.5	0.0	0.0
64	13	13	2	1	3	0	10	4	6	3.8	3.6	3.7	0.0	23.1
65	12	11	0	0	0	0	11	4	7	3.8	3.6	3.7	8.3	0.0
66	14	14	2	0	2	0	12	4	8	3.6	3.4	3.5	0.0	14.3
67	12	1	0	0	0	0	1	0	1	-	3.6	3.6	91.7	0.0
MEAN	12.0	9.6	0.8	0.2	1.0	0.0	8.6	3.2	5.4	3.7	3.5	3.6	20.0	7.5
S.D.	1.9	5.2	1.1	0.4	1.4	0.0	4.4	1.8	2.7	0.1	0.1	0.1	40.2	10.7
N	5	5	5	5	5	5	5	5	5	4	5	5	5	5

PR.NO.60R0375/88R002: REPRODUCTIVE TOX. STUDY TO DETECT EFFECTS
OF MIXED ANTI-ANDROGENIC SUBSTANCES IN RATS; ORAL ADM. (GAVAGE)
INDIVIDUAL REPRODUCTION DATA

TEST GROUP 3 (LOAEL-MIX)

	CORPORA	IMPLANT	RESORPTIONS			FETUSES		SEX		AVERAGE FETAL BODY WEIGHT			%IMPLANTATION LOSS	
FEMALE#	LUTEA	SITES	EARLY	LATE	TOTAL	DEAD	LIVE	MALE	FEMALE	MALES	FEMALES	LITTER	PRE	POST
88	12	9	0	0	0	0	9	4	5	3.8	3.7	3.8	25.0	0.0
89	13	13	0	0	0	0	13	7	6	3.7	3.6	3.7	0.0	0.0
90	11	11	0	0	0	0	11	4	7	3.7	3.3	3.4	0.0	0.0
91	12	12	1	0	1	0	11	6	5	3.8	3.8	3.8	0.0	8.3
92	10	9	0	0	0	0	9	5	4	3.9	3.7	3.8	10.0	0.0
MEAN	11.6	10.8	0.2	0.0	0.2	0.0	10.6	5.2	5.4	3.8	3.6	3.7	7.0	1.7
S.D.	1.1	1.8	0.4	0.0	0.4	0.0	1.7	1.3	1.1	0.1	0.2	0.2	11.0	3.7
N	5	5	5	5	5	5	5	5	5	5	5	5	5	5

PR.NO.60R0375/88R002: REPRODUCTIVE TOX. STUDY TO DETECT EFFECTS
OF MIXED ANTI-ANDROGENIC SUBSTANCES IN RATS; ORAL ADM. (GAVAGE)
INDIVIDUAL REPRODUCTION DATA

TEST GROUP 4(0.00025 MG/KG BW/D)

	CORPORA	IMPLANT	RESORPTIONS			FETUSES		SEX		AVERAGE FETAL BODY WEIGHT			%IMPLANTATION LOSS	
FEMALE#	LUTEA	SITES	EARLY	LATE	TOTAL	DEAD	LIVE	MALE	FEMALE	MALES	FEMALES	LITTER	PRE	POST
112	14	13	1	0	1	0	12	5	7	3.6	3.4	3.5	7.1	7.7
113	10	10	0	0	0	0	10	4	6	3.6	3.4	3.4	0.0	0.0
114	10	10	0	0	0	0	10	6	4	3.8	3.4	3.6	0.0	0.0
115	13	13	2	0	2	0	11	6	5	3.7	3.4	3.5	0.0	15.4
116	13	13	0	0	0	0	13	4	9	3.6	3.4	3.5	0.0	0.0
MEAN	12.0	11.8	0.6	0.0	0.6	0.0	11.2	5.0	6.2	3.6	3.4	3.5	1.4	4.6
S.D.	1.9	1.6	0.9	0.0	0.9	0.0	1.3	1.0	1.9	0.1	0.0	0.1	3.2	6.9
N	5	5	5	5	5	5	5	5	5	5	5	5	5	5

PR.NO.60R0375/88R002: REPRODUCTIVE TOX. STUDY TO DETECT EFFECTS
OF MIXED ANTI-ANDROGENIC SUBSTANCES IN RATS; ORAL ADM. (GAVAGE)
INDIVIDUAL FETAL STATUS AND UTERINE LOCATION

TEST GROUP 0 (0 MG/KG BW/D)

FEMALE#	IMPLANT # 1	2	3	4	5	6	7	8	9	10	11	12	13	14	15	16	17	18	19	20	21	22	23
12	FA	MA	FA	FA	FA	FA	FA	MA	/MA	MA	MA	MA	E										
13	L	FA	FA	MA	/MA	MA																	
14	MA	MA	MA	FA	FA	MA	/FA	FA	MA	MA	FA												
15	MA	MA	MA	FA	/FA	FA	MA	FA	MA	FA													
16	MA	MA	MA	FA	FA	/MA	E	FA	FA														

M MALE F FEMALE U UNCERTAIN / DENOTES POSITION OF CERVIX
A ALIVE E EARLY RESORPTION L LATE RESORPTION D DEAD FETUS B ABORTED FETUS P PREMATURE DELIVERY

PR.NO.60R0375/88R002: REPRODUCTIVE TOX. STUDY TO DETECT EFFECTS
OF MIXED ANTI-ANDROGENIC SUBSTANCES IN RATS; ORAL ADM. (GAVAGE)
INDIVIDUAL FETAL STATUS AND UTERINE LOCATION

TEST GROUP 1 (ADI-MIX)

FEMALE#	IMPLANT # 1	2	3	4	5	6	7	8	9	10	11	12	13	14	15	16	17	18	19	20	21	22	23
38	MA	FA	FA	FA	FA	/FA	MA	MA	MA														
39	MA	MA	FA	E	FA	/MA	MA	MA	MA	MA													
40	MA	FA	MA	MA	MA	MA	/FA	MA	FA	FA	MA	MA											
41	MA	FA	MA	FA	MA	FA	MA	MA	FA	MA	/FA	FA	MA										
42	MA	E	MA	MA	FA	FA	MA	MA	/MA	MA	FA	FA	FA										

M MALE F FEMALE U UNCERTAIN / DENOTES POSITION OF CERVIX
A ALIVE E EARLY RESORPTION L LATE RESORPTION D DEAD FETUS B ABORTED FETUS P PREMATURE DELIVERY

PR.NO.60R0375/88R002: REPRODUCTIVE TOX. STUDY TO DETECT EFFECTS
OF MIXED ANTI-ANDROGENIC SUBSTANCES IN RATS; ORAL ADM. (GAVAGE)
INDIVIDUAL FETAL STATUS AND UTERINE LOCATION

TEST GROUP 2 (NOAEL-MIX)

FEMALE#	IMPLANT # 1	2	3	4	5	6	7	8	9	10	11	12	13	14	15	16	17	18	19	20	21	22	23
63	FA	/MA	FA	MA	MA	FA	MA	FA	FA														
64	FA	E	E	MA	FA	L	FA	/FA	MA	FA	FA	MA	MA										
65	FA	MA	FA	FA	FA	MA	FA	/MA	FA	FA	MA												
66	FA	FA	FA	E	FA	FA	MA	MA	/FA	MA	FA	E	FA	MA									
67	/FA																						

M MALE F FEMALE U UNCERTAIN / DENOTES POSITION OF CERVIX
A ALIVE E EARLY RESORPTION L LATE RESORPTION D DEAD FETUS B ABORTED FETUS P PREMATURE DELIVERY

PR.NO.60R0375/88R002: REPRODUCTIVE TOX. STUDY TO DETECT EFFECTS
OF MIXED ANTI-ANDROGENIC SUBSTANCES IN RATS; ORAL ADM. (GAVAGE)
INDIVIDUAL FETAL STATUS AND UTERINE LOCATION

TEST GROUP 3 (LOAEL-MIX)

FEMALE#	IMPLANT # 1	2	3	4	5	6	7	8	9	10	11	12	13	14	15	16	17	18	19	20	21	22	23
88	MA	MA	MA	FA	FA	FA	/FA	MA	FA														
89	FA	MA	MA	MA	MA	FA	/FA	FA	MA	MA	FA	MA	FA										
90	FA	MA	FA	MA	FA	/FA	FA	MA	FA	MA	FA												
91	FA	FA	E	MA	FA	FA	/FA	MA	MA	MA	MA	MA											
92	FA	FA	MA	FA	/FA	MA	MA	MA	MA														

M MALE F FEMALE U UNCERTAIN / DENOTES POSITION OF CERVIX
A ALIVE E EARLY RESORPTION L LATE RESORPTION D DEAD FETUS B ABORTED FETUS P PREMATURE DELIVERY

PR.NO.60R0375/88R002: REPRODUCTIVE TOX. STUDY TO DETECT EFFECTS
OF MIXED ANTI-ANDROGENIC SUBSTANCES IN RATS; ORAL ADM. (GAVAGE)
INDIVIDUAL FETAL STATUS AND UTERINE LOCATION

TEST GROUP 4(0.00025 MG/KG BW/D)

FEMALE#	IMPLANT # 1	2	3	4	5	6	7	8	9	10	11	12	13	14	15	16	17	18	19	20	21	22	23
112	MA	FA	FA	FA	/FA	FA	FA	MA	E	MA	MA	FA	MA										
113	FA	FA	MA	FA	FA	MA	/FA	FA	MA	MA													
114	FA	FA	MA	FA	FA	MA	MA	/MA	MA	MA													
115	MA	MA	FA	E	FA	FA	/MA	MA	FA	MA	E	MA	FA										
116	FA	FA	FA	FA	MA	MA	FA	MA	FA	/FA	MA	FA	FA										

M MALE F FEMALE U UNCERTAIN / DENOTES POSITION OF CERVIX
A ALIVE E EARLY RESORPTION L LATE RESORPTION D DEAD FETUS B ABORTED FETUS P PREMATURE DELIVERY

PR.NO.60R0375/88R002: REPRODUCTIVE TOX. STUDY TO DETECT EFFECTS
OF MIXED ANTI-ANDROGENIC SUBSTANCES IN RATS; ORAL ADM. (GAVAGE)
INDIVIDUAL FETAL BODY WEIGHTS -- GRAMS

TEST GROUP 0 (0 MG/KG BW/D)

FEMALE#	MEAN	FETUS# 1	2	3	4	5	6	7	8	9	10	11	12	13	14	15	16	17	18	19
12	3.5	3.1	3.2	3.5	3.7	3.5	3.4	3.4	3.8/	3.4	3.7	3.7	3.4	E						
13	3.6	L	3.5	3.5	3.9/	3.7	3.5													
14	3.6	3.8	3.7	3.7	3.6	3.5	3.8/	3.9	3.6	3.7	3.5	2.7								
15	3.9	3.9	4.0	4.0	3.9/	3.9	3.9	3.7	3.8	3.8	3.5									
16	3.5	3.7	3.9	3.5	3.2	3.3/	3.5	E	3.2	3.8										
MEAN	3.6																			
S.D.	0.1																			
N	5																			

E-EARLY RESORPTION L-LATE RESORPTION D-DEAD FETUS /-DENOTES POSITION OF CERVIX

PR.NO.60R0375/88R002: REPRODUCTIVE TOX. STUDY TO DETECT EFFECTS
OF MIXED ANTI-ANDROGENIC SUBSTANCES IN RATS; ORAL ADM. (GAVAGE)
INDIVIDUAL FETAL BODY WEIGHTS -- GRAMS

TEST GROUP 1 (ADI-MIX)

FEMALE#	MEAN	FETUS# 1	2	3	4	5	6	7	8	9	10	11	12	13	14	15	16	17	18	19
38	3.5	3.6	3.6	3.1	3.3	3.7/	3.4	3.7	3.6	3.5										
39	3.5	3.3	3.4	3.4	E	3.4/	3.5	3.5	3.7	3.6	3.6									
40	3.4	3.3	3.4	3.8	3.8	3.6	3.6/	2.9	3.8	3.2	2.8	3.7	3.3							
41	3.2	3.1	3.2	3.2	3.2	3.3	3.1	3.4	3.4	3.3	3.3/	3.3	3.4	3.2						
42	3.2	2.2	E	3.5	3.1	2.7	3.3	3.2	3.6/	3.6	3.7	3.1	3.2	3.9						
MEAN	3.4																			
S.D.	0.1																			
N	5																			

E-EARLY RESORPTION L-LATE RESORPTION D-DEAD FETUS /-DENOTES POSITION OF CERVIX

PR.NO.60R0375/88R002: REPRODUCTIVE TOX. STUDY TO DETECT EFFECTS
OF MIXED ANTI-ANDROGENIC SUBSTANCES IN RATS; ORAL ADM. (GAVAGE)
INDIVIDUAL FETAL BODY WEIGHTS -- GRAMS

TEST GROUP 2 (NOAEL-MIX)

FEMALE#	MEAN	FETUS# 1	2	3	4	5	6	7	8	9	10	11	12	13	14	15	16	17	18	19
63	3.5	3.6/	3.5	3.7	3.5	3.5	3.4	3.7	2.9	3.2										
64	3.7	3.6	E	E	3.7	3.3	L	3.6/	3.9	4.0	3.5	3.6	3.9	3.4						
65	3.7	4.0	3.8	3.5	3.8	3.7	3.6	3.5/	4.0	3.5	3.5	3.6								
66	3.5	3.7	3.5	2.9	E	3.6	3.7	3.8	3.8/	3.4	3.4	3.3	E	3.3	3.3					
67	3.6 /	3.6																		
MEAN	3.6																			
S.D.	0.1																			
N	5																			

E-EARLY RESORPTION L-LATE RESORPTION D-DEAD FETUS /-DENOTES POSITION OF CERVIX

PR.NO.60R0375/88R002: REPRODUCTIVE TOX. STUDY TO DETECT EFFECTS
OF MIXED ANTI-ANDROGENIC SUBSTANCES IN RATS; ORAL ADM. (GAVAGE)
INDIVIDUAL FETAL BODY WEIGHTS -- GRAMS

TEST GROUP 3 (LOAEL-MIX)

FEMALE#	MEAN	FETUS# 1	2	3	4	5	6	7	8	9	10	11	12	13	14	15	16	17	18	19
88	3.8	2.9	4.2	4.1	3.9	3.7	3.8/	3.7	4.1	3.6										
89	3.7	3.6	3.9	3.7	4.3	3.9	3.7/	3.6	3.4	3.5	3.5	3.8	3.3	3.5						
90	3.4	3.5	3.6	2.9	3.8	3.3/	3.7	3.4	3.8	3.3	3.4	2.7								
91	3.8	3.5	3.7	E	3.8	4.1	4.0/	3.9	3.9	3.3	3.8	4.1	3.8							
92	3.8	3.7	3.7	3.8	3.8/	3.5	3.8	4.1	4.0	3.8										
MEAN	3.7																			
S.D.	0.2																			
N	5																			

E-EARLY RESORPTION L-LATE RESORPTION D-DEAD FETUS /-DENOTES POSITION OF CERVIX

PR.NO.60R0375/88R002: REPRODUCTIVE TOX. STUDY TO DETECT EFFECTS
OF MIXED ANTI-ANDROGENIC SUBSTANCES IN RATS; ORAL ADM. (GAVAGE)
INDIVIDUAL FETAL BODY WEIGHTS -- GRAMS

TEST GROUP 4(0.00025 MG/KG BW/D)

FEMALE#	MEAN	FETUS# 1	2	3	4	5	6	7	8	9	10	11	12	13	14	15	16	17	18	19
112	3.5	3.8	3.7	3.3	3.5/	3.1	3.8	3.2	3.6	E	3.6	3.5	3.3	3.7						
113	3.4	2.9	3.2	3.6	3.6	3.5	3.9/	3.5	3.4	3.6	3.2									
114	3.6	3.5	3.4	3.4	3.4	3.4	3.9	4.0/	3.7	3.9	3.7									
115	3.5	3.5	3.6	3.4	E	3.4	3.3/	3.9	3.3	3.5	3.9	E	3.8	3.4						
116	3.5	3.4	3.3	3.5	2.8	3.5	3.5	3.5	3.4	3.5/	3.4	3.9	3.7	3.4						
MEAN	3.5																			
S.D.	0.1																			
N	5																			

E-EARLY RESORPTION L-LATE RESORPTION D-DEAD FETUS /-DENOTES POSITION OF CERVIX

PR.NO.60R0375/88R002: REPRODUCTIVE TOX. STUDY TO DETECT EFFECTS
OF MIXED ANTI-ANDROGENIC SUBSTANCES IN RATS; ORAL ADM. (GAVAGE)
INDIVIDUAL FETAL EXTERNAL OBSERVATIONS

TEST GROUP 0 (0 MG/KG BW/D)

FEMALE#	FETUS#	OBSERVATION	POSITION/GRADE
12	1	NOTHING ABNORMAL DETECTED	
	2	NOTHING ABNORMAL DETECTED	
	3	NOTHING ABNORMAL DETECTED	
	4	NOTHING ABNORMAL DETECTED	
	5	NOTHING ABNORMAL DETECTED	
	6	NOTHING ABNORMAL DETECTED	
	7	NOTHING ABNORMAL DETECTED	
	8	NOTHING ABNORMAL DETECTED	
	9	NOTHING ABNORMAL DETECTED	
	10	NOTHING ABNORMAL DETECTED	
	11	NOTHING ABNORMAL DETECTED	
	12	NOTHING ABNORMAL DETECTED	
13	2	NOTHING ABNORMAL DETECTED	
	3	NOTHING ABNORMAL DETECTED	
	4	NOTHING ABNORMAL DETECTED	
	5	NOTHING ABNORMAL DETECTED	
	6	NOTHING ABNORMAL DETECTED	
14	1	NOTHING ABNORMAL DETECTED	
	2	NOTHING ABNORMAL DETECTED	
	3	NOTHING ABNORMAL DETECTED	
	4	NOTHING ABNORMAL DETECTED	
	5	NOTHING ABNORMAL DETECTED	
	6	NOTHING ABNORMAL DETECTED	
	7	NOTHING ABNORMAL DETECTED	
	8	NOTHING ABNORMAL DETECTED	
	9	NOTHING ABNORMAL DETECTED	
	10	NOTHING ABNORMAL DETECTED	
	11	NOTHING ABNORMAL DETECTED	
15	1	NOTHING ABNORMAL DETECTED	
	2	NOTHING ABNORMAL DETECTED	
	3	NOTHING ABNORMAL DETECTED	
	4	NOTHING ABNORMAL DETECTED	
	5	NOTHING ABNORMAL DETECTED	
	6	NOTHING ABNORMAL DETECTED	
	7	NOTHING ABNORMAL DETECTED	
	8	NOTHING ABNORMAL DETECTED	

POSITION/GRADE CODE: R-RIGHT, L-LEFT, B-BILATE , 1-SLIGHT, 2-MODERATE, 3-MARKED, P-PRESENT
FINDING CODE: M-MALFORMATION V-VARIATION R-RETARDATIO U-UNCLASSIFIED OBSERVATION

PR.NO.60R0375/88R002: REPRODUCTIVE TOX. STUDY TO DETECT EFFECTS
OF MIXED ANTI-ANDROGENIC SUBSTANCES IN RATS; ORAL ADM. (GAVAGE)
INDIVIDUAL FETAL EXTERNAL OBSERVATIONS

TEST GROUP 0 (0 MG/KG BW/D)

FEMALE#	FETUS#	OBSERVATION	POSITION/GRADE
15 (CONTINUED)			
	9	NOTHING ABNORMAL DETECTED	
	10	NOTHING ABNORMAL DETECTED	
16	1	NOTHING ABNORMAL DETECTED	
	2	NOTHING ABNORMAL DETECTED	
	3	NOTHING ABNORMAL DETECTED	
	4	NOTHING ABNORMAL DETECTED	
	5	NOTHING ABNORMAL DETECTED	
	6	NOTHING ABNORMAL DETECTED	
	8	NOTHING ABNORMAL DETECTED	
	9	NOTHING ABNORMAL DETECTED	

POSITION/GRADE CODE: R-RIGHT, L-LEFT, B-BILATE , 1-SLIGHT, 2-MODERATE, 3-MARKED, P-PRESENT
FINDING CODE: M-MALFORMATION V-VARIATION R-RETARDATIO U-UNCLASSIFIED OBSERVATION

PR.NO.60R0375/88R002: REPRODUCTIVE TOX. STUDY TO DETECT EFFECTS
OF MIXED ANTI-ANDROGENIC SUBSTANCES IN RATS; ORAL ADM. (GAVAGE)
INDIVIDUAL FETAL EXTERNAL OBSERVATIONS

TEST GROUP 1 (ADI-MIX)

FEMALE#	FETUS#	OBSERVATION	POSITION/GRADE
38	1	NOTHING ABNORMAL DETECTED	
	2	NOTHING ABNORMAL DETECTED	
	3	NOTHING ABNORMAL DETECTED	
	4	NOTHING ABNORMAL DETECTED	
	5	NOTHING ABNORMAL DETECTED	
	6	NOTHING ABNORMAL DETECTED	
	7	NOTHING ABNORMAL DETECTED	
	8	NOTHING ABNORMAL DETECTED	
	9	NOTHING ABNORMAL DETECTED	
39	1	NOTHING ABNORMAL DETECTED	
	2	NOTHING ABNORMAL DETECTED	
	3	NOTHING ABNORMAL DETECTED	
	5	NOTHING ABNORMAL DETECTED	
	6	NOTHING ABNORMAL DETECTED	
	7	NOTHING ABNORMAL DETECTED	
	8	NOTHING ABNORMAL DETECTED	
	9	NOTHING ABNORMAL DETECTED	
	10	NOTHING ABNORMAL DETECTED	
40	1	NOTHING ABNORMAL DETECTED	
	2	NOTHING ABNORMAL DETECTED	
	3	NOTHING ABNORMAL DETECTED	
	4	NOTHING ABNORMAL DETECTED	
	5	NOTHING ABNORMAL DETECTED	
	6	NOTHING ABNORMAL DETECTED	
	7	NOTHING ABNORMAL DETECTED	
	8	NOTHING ABNORMAL DETECTED	
	9	NOTHING ABNORMAL DETECTED	
	10	NOTHING ABNORMAL DETECTED	
	11	NOTHING ABNORMAL DETECTED	
	12	NOTHING ABNORMAL DETECTED	
41	1	NOTHING ABNORMAL DETECTED	
	2	NOTHING ABNORMAL DETECTED	
	3	NOTHING ABNORMAL DETECTED	
	4	NOTHING ABNORMAL DETECTED	
	5	NOTHING ABNORMAL DETECTED	
	6	NOTHING ABNORMAL DETECTED	

POSITION/GRADE CODE: R-RIGHT, L-LEFT, B-BILATE , 1-SLIGHT, 2-MODERATE, 3-MARKED, P-PRESENT
FINDING CODE: M-MALFORMATION V-VARIATION R-RETARDATIO U-UNCLASSIFIED OBSERVATION

PR.NO.60R0375/88R002: REPRODUCTIVE TOX. STUDY TO DETECT EFFECTS
OF MIXED ANTI-ANDROGENIC SUBSTANCES IN RATS; ORAL ADM. (GAVAGE)
INDIVIDUAL FETAL EXTERNAL OBSERVATIONS

TEST GROUP 1 (ADI-MIX)

FEMALE#	FETUS#	OBSERVATION	POSITION/GRADE
41 (CONTINUED)			
	7	NOTHING ABNORMAL DETECTED	
	8	NOTHING ABNORMAL DETECTED	
	9	NOTHING ABNORMAL DETECTED	
	10	NOTHING ABNORMAL DETECTED	
	11	NOTHING ABNORMAL DETECTED	
	12	NOTHING ABNORMAL DETECTED	
	13	NOTHING ABNORMAL DETECTED	
42	1	NOTHING ABNORMAL DETECTED	
	3	NOTHING ABNORMAL DETECTED	
	4	NOTHING ABNORMAL DETECTED	
	5	NOTHING ABNORMAL DETECTED	
	6	NOTHING ABNORMAL DETECTED	
	7	NOTHING ABNORMAL DETECTED	
	8	NOTHING ABNORMAL DETECTED	
	9	NOTHING ABNORMAL DETECTED	
	10	NOTHING ABNORMAL DETECTED	
	11	NOTHING ABNORMAL DETECTED	
	12	NOTHING ABNORMAL DETECTED	
	13	NOTHING ABNORMAL DETECTED	

POSITION/GRADE CODE: R-RIGHT, L-LEFT, B-BILATE , 1-SLIGHT, 2-MODERATE, 3-MARKED, P-PRESENT
FINDING CODE: M-MALFORMATION V-VARIATION R-RETARDATIO U-UNCLASSIFIED OBSERVATION

PR.NO.60R0375/88R002: REPRODUCTIVE TOX. STUDY TO DETECT EFFECTS
OF MIXED ANTI-ANDROGENIC SUBSTANCES IN RATS; ORAL ADM. (GAVAGE)
INDIVIDUAL FETAL EXTERNAL OBSERVATIONS

TEST GROUP 2 (NOAEL-MIX)

FEMALE#	FETUS#	OBSERVATION	POSITION/GRADE
63	1	NOTHING ABNORMAL DETECTED	
	2	NOTHING ABNORMAL DETECTED	
	3	NOTHING ABNORMAL DETECTED	
	4	NOTHING ABNORMAL DETECTED	
	5	NOTHING ABNORMAL DETECTED	
	6	NOTHING ABNORMAL DETECTED	
	7	NOTHING ABNORMAL DETECTED	
	8	NOTHING ABNORMAL DETECTED	
	9	NOTHING ABNORMAL DETECTED	
64	1	NOTHING ABNORMAL DETECTED	
	4	NOTHING ABNORMAL DETECTED	
	5	NOTHING ABNORMAL DETECTED	
	7	NOTHING ABNORMAL DETECTED	
	8	NOTHING ABNORMAL DETECTED	
	9	NOTHING ABNORMAL DETECTED	
	10	NOTHING ABNORMAL DETECTED	
	11	NOTHING ABNORMAL DETECTED	
	12	NOTHING ABNORMAL DETECTED	
	13	NOTHING ABNORMAL DETECTED	
65	1	NOTHING ABNORMAL DETECTED	
	2	NOTHING ABNORMAL DETECTED	
	3	NOTHING ABNORMAL DETECTED	
	4	NOTHING ABNORMAL DETECTED	
	5	NOTHING ABNORMAL DETECTED	
	6	NOTHING ABNORMAL DETECTED	
	7	NOTHING ABNORMAL DETECTED	
	8	NOTHING ABNORMAL DETECTED	
	9	NOTHING ABNORMAL DETECTED	
	10	NOTHING ABNORMAL DETECTED	
	11	NOTHING ABNORMAL DETECTED	
66	1	NOTHING ABNORMAL DETECTED	
	2	NOTHING ABNORMAL DETECTED	
	3	NOTHING ABNORMAL DETECTED	
	5	NOTHING ABNORMAL DETECTED	
	6	NOTHING ABNORMAL DETECTED	
	7	NOTHING ABNORMAL DETECTED	

POSITION/GRADE CODE: R-RIGHT, L-LEFT, B-BILATE , 1-SLIGHT, 2-MODERATE, 3-MARKED, P-PRESENT
FINDING CODE: M-MALFORMATION V-VARIATION R-RETARDATIO U-UNCLASSIFIED OBSERVATION

PR.NO.60R0375/88R002: REPRODUCTIVE TOX. STUDY TO DETECT EFFECTS
OF MIXED ANTI-ANDROGENIC SUBSTANCES IN RATS; ORAL ADM. (GAVAGE)
INDIVIDUAL FETAL EXTERNAL OBSERVATIONS

TEST GROUP 2 (NOAEL-MIX)

FEMALE#	FETUS#	OBSERVATION	POSITION/GRADE
66 (CONTINUED)			
	8	NOTHING ABNORMAL DETECTED	
	9	NOTHING ABNORMAL DETECTED	
	10	NOTHING ABNORMAL DETECTED	
	11	NOTHING ABNORMAL DETECTED	
	13	NOTHING ABNORMAL DETECTED	
	14	NOTHING ABNORMAL DETECTED	
67	1	V LIMB HYPEREXTENSION HINDLIMBS	B
		M HEAD MISSHAPEN	
		- SHORT SNOUT	
		- PINNA MALPOSITIONED	

POSITION/GRADE CODE: R-RIGHT, L-LEFT, B-BILATE , 1-SLIGHT, 2-MODERATE, 3-MARKED, P-PRESENT
FINDING CODE: M-MALFORMATION V-VARIATION R-RETARDATIO U-UNCLASSIFIED OBSERVATION

PR.NO.60R0375/88R002: REPRODUCTIVE TOX. STUDY TO DETECT EFFECTS
OF MIXED ANTI-ANDROGENIC SUBSTANCES IN RATS; ORAL ADM. (GAVAGE)
INDIVIDUAL FETAL EXTERNAL OBSERVATIONS

TEST GROUP 3 (LOAEL-MIX)

FEMALE#	FETUS#	OBSERVATION	POSITION/GRADE
88	1	NOTHING ABNORMAL DETECTED	
	2	NOTHING ABNORMAL DETECTED	
	3	NOTHING ABNORMAL DETECTED	
	4	NOTHING ABNORMAL DETECTED	
	5	NOTHING ABNORMAL DETECTED	
	6	NOTHING ABNORMAL DETECTED	
	7	NOTHING ABNORMAL DETECTED	
	8	NOTHING ABNORMAL DETECTED	
	9	NOTHING ABNORMAL DETECTED	
89	1	NOTHING ABNORMAL DETECTED	
	2	NOTHING ABNORMAL DETECTED	
	3	NOTHING ABNORMAL DETECTED	
	4	NOTHING ABNORMAL DETECTED	
	5	NOTHING ABNORMAL DETECTED	
	6	NOTHING ABNORMAL DETECTED	
	7	NOTHING ABNORMAL DETECTED	
	8	NOTHING ABNORMAL DETECTED	
	9	NOTHING ABNORMAL DETECTED	
	10	NOTHING ABNORMAL DETECTED	
	11	NOTHING ABNORMAL DETECTED	
	12	NOTHING ABNORMAL DETECTED	
	13	NOTHING ABNORMAL DETECTED	
90	1	NOTHING ABNORMAL DETECTED	
	2	NOTHING ABNORMAL DETECTED	
	3	NOTHING ABNORMAL DETECTED	
	4	NOTHING ABNORMAL DETECTED	
	5	NOTHING ABNORMAL DETECTED	
	6	NOTHING ABNORMAL DETECTED	
	7	NOTHING ABNORMAL DETECTED	
	8	NOTHING ABNORMAL DETECTED	
	9	NOTHING ABNORMAL DETECTED	
	10	NOTHING ABNORMAL DETECTED	
	11	NOTHING ABNORMAL DETECTED	
91	1	NOTHING ABNORMAL DETECTED	
	2	NOTHING ABNORMAL DETECTED	
	4	NOTHING ABNORMAL DETECTED	

POSITION/GRADE CODE: R-RIGHT, L-LEFT, B-BILATE , 1-SLIGHT, 2-MODERATE, 3-MARKED, P-PRESENT
FINDING CODE: M-MALFORMATION V-VARIATION R-RETARDATIO U-UNCLASSIFIED OBSERVATION

PR.NO.60R0375/88R002: REPRODUCTIVE TOX. STUDY TO DETECT EFFECTS
OF MIXED ANTI-ANDROGENIC SUBSTANCES IN RATS; ORAL ADM. (GAVAGE)
INDIVIDUAL FETAL EXTERNAL OBSERVATIONS

TEST GROUP 3 (LOAEL-MIX)

FEMALE#	FETUS#	OBSERVATION	POSITION/GRADE
91 (CONTINUED)			
	5	NOTHING ABNORMAL DETECTED	
	6	NOTHING ABNORMAL DETECTED	
	7	NOTHING ABNORMAL DETECTED	
	8	NOTHING ABNORMAL DETECTED	
	9	NOTHING ABNORMAL DETECTED	
	10	NOTHING ABNORMAL DETECTED	
	11	NOTHING ABNORMAL DETECTED	
	12	NOTHING ABNORMAL DETECTED	
92	1	NOTHING ABNORMAL DETECTED	
	2	NOTHING ABNORMAL DETECTED	
	3	NOTHING ABNORMAL DETECTED	
	4	NOTHING ABNORMAL DETECTED	
	5	NOTHING ABNORMAL DETECTED	
	6	NOTHING ABNORMAL DETECTED	
	7	NOTHING ABNORMAL DETECTED	
	8	NOTHING ABNORMAL DETECTED	
	9	NOTHING ABNORMAL DETECTED	

POSITION/GRADE CODE: R-RIGHT, L-LEFT, B-BILATE , 1-SLIGHT, 2-MODERATE, 3-MARKED, P-PRESENT
FINDING CODE: M-MALFORMATION V-VARIATION R-RETARDATIO U-UNCLASSIFIED OBSERVATION

PR.NO.60R0375/88R002: REPRODUCTIVE TOX. STUDY TO DETECT EFFECTS
OF MIXED ANTI-ANDROGENIC SUBSTANCES IN RATS; ORAL ADM. (GAVAGE)
INDIVIDUAL FETAL EXTERNAL OBSERVATIONS

TEST GROUP 4(0.00025 MG/KG BW/D)

FEMALE#	FETUS#	OBSERVATION	POSITION/GRADE
112	1	NOTHING ABNORMAL DETECTED	
	2	NOTHING ABNORMAL DETECTED	
	3	NOTHING ABNORMAL DETECTED	
	4	NOTHING ABNORMAL DETECTED	
	5	NOTHING ABNORMAL DETECTED	
	6	NOTHING ABNORMAL DETECTED	
	7	NOTHING ABNORMAL DETECTED	
	8	NOTHING ABNORMAL DETECTED	
	10	NOTHING ABNORMAL DETECTED	
	11	NOTHING ABNORMAL DETECTED	
	12	NOTHING ABNORMAL DETECTED	
	13	NOTHING ABNORMAL DETECTED	
113	1	NOTHING ABNORMAL DETECTED	
	2	NOTHING ABNORMAL DETECTED	
	3	NOTHING ABNORMAL DETECTED	
	4	NOTHING ABNORMAL DETECTED	
	5	NOTHING ABNORMAL DETECTED	
	6	NOTHING ABNORMAL DETECTED	
	7	NOTHING ABNORMAL DETECTED	
	8	NOTHING ABNORMAL DETECTED	
	9	NOTHING ABNORMAL DETECTED	
	10	NOTHING ABNORMAL DETECTED	
114	1	NOTHING ABNORMAL DETECTED	
	2	NOTHING ABNORMAL DETECTED	
	3	NOTHING ABNORMAL DETECTED	
	4	NOTHING ABNORMAL DETECTED	
	5	NOTHING ABNORMAL DETECTED	
	6	NOTHING ABNORMAL DETECTED	
	7	NOTHING ABNORMAL DETECTED	
	8	NOTHING ABNORMAL DETECTED	
	9	NOTHING ABNORMAL DETECTED	
	10	NOTHING ABNORMAL DETECTED	
115	1	NOTHING ABNORMAL DETECTED	
	2	NOTHING ABNORMAL DETECTED	
	3	NOTHING ABNORMAL DETECTED	
	5	NOTHING ABNORMAL DETECTED	

POSITION/GRADE CODE: R-RIGHT, L-LEFT, B-BILATE , 1-SLIGHT, 2-MODERATE, 3-MARKED, P-PRESENT
FINDING CODE: M-MALFORMATION V-VARIATION R-RETARDATIO U-UNCLASSIFIED OBSERVATION

PR.NO.60R0375/88R002: REPRODUCTIVE TOX. STUDY TO DETECT EFFECTS
OF MIXED ANTI-ANDROGENIC SUBSTANCES IN RATS; ORAL ADM. (GAVAGE)
INDIVIDUAL FETAL EXTERNAL OBSERVATIONS

TEST GROUP 4(0.00025 MG/KG BW/D)

FEMALE#	FETUS#	OBSERVATION	POSITION/GRADE
115 (CONTINUED)			
	6	NOTHING ABNORMAL DETECTED	
	7	NOTHING ABNORMAL DETECTED	
	8	NOTHING ABNORMAL DETECTED	
	9	NOTHING ABNORMAL DETECTED	
	10	NOTHING ABNORMAL DETECTED	
	12	NOTHING ABNORMAL DETECTED	
	13	NOTHING ABNORMAL DETECTED	
116	1	NOTHING ABNORMAL DETECTED	
	2	NOTHING ABNORMAL DETECTED	
	3	NOTHING ABNORMAL DETECTED	
	4	NOTHING ABNORMAL DETECTED	
	5	NOTHING ABNORMAL DETECTED	
	6	NOTHING ABNORMAL DETECTED	
	7	NOTHING ABNORMAL DETECTED	
	8	NOTHING ABNORMAL DETECTED	
	9	NOTHING ABNORMAL DETECTED	
	10	NOTHING ABNORMAL DETECTED	
	11	NOTHING ABNORMAL DETECTED	
	12	NOTHING ABNORMAL DETECTED	
	13	NOTHING ABNORMAL DETECTED	

POSITION/GRADE CODE: R-RIGHT, L-LEFT, B-BILATE , 1-SLIGHT, 2-MODERATE, 3-MARKED, P-PRESENT
FINDING CODE: M-MALFORMATION V-VARIATION R-RETARDATIO U-UNCLASSIFIED OBSERVATION

PR.NO.60R0375/88R002: REPRODUCTIVE TOX. STUDY TO DETECT EFFECTS
OF MIXED ANTI-ANDROGENIC SUBSTANCES IN RATS; ORAL ADM. (GAVAGE)
INDIVIDUAL DELIVERY AND LITTER DATA

TEST GROUP 0 (0 MG/KG BW/D)

FEMALE#	LITTER DELIVERED LIVE N	DEAD N	TOTAL N	NUMBER OF LIVE PUPS DAYS 1 M	1 F	4 M	4 F	7 M	7 F	14 M	14 F	21 M	21 F	DURATION OF GESTATION (DAYS) N
1	8	0	8	6	2	6	2	6	2	6	2	6	2	22
2	10	0	10	5	5	5	5	5	5	5	5	5	5	22
3	9	0	9	5	4	5	4	5	4	5	4	5	4	22
4	11	0	11	4	7	4	7	4	7	4	7	4	7	22
5	11	0	11	7	4	7	4	7	4	7	4	7	4	22
6	9	0	9	4	5	4	5	4	5	4	5	4	5	22
7	8	0	8	5	3	5	3	5	3	5	3	5	3	22
8	9	0	9	4	5	4	5	4	5	4	5	4	5	22
9	7	0	7	4	3	4	3	4	3	4	3	4	3	22
10	10	0	10	5	5	5	5	5	5	5	5	5	5	22
11	12	0	12	5	7	5	7	5	7	5	7	5	7	22
17	8	0	8	2	6	2	6	2	6	2	6	2	6	22
18	10	0	10	5	5	5	5	5	5	5	5	5	5	22
19	8	0	8	2	6	2	6	2	6	2	6	2	6	22
20	10	0	10	2	8	2	8	2	8	2	8	2	8	22
21	12	0	12	7	5	7	5	7	5	7	5	7	5	22
22	9	0	9	4	5	4	5	4	5	4	5	4	5	21
23	9	1	10	3	6	3	6	3	6	3	6	3	6	22
24	11	0	11	6	5	6	5	6	5	6	5	6	5	22
25	11	0	11	5	6	5	6	5	6	5	6	5	6	22

M=MALE, F=FEMALE

PR.NO.60R0375/88R002: REPRODUCTIVE TOX. STUDY TO DETECT EFFECTS
OF MIXED ANTI-ANDROGENIC SUBSTANCES IN RATS; ORAL ADM. (GAVAGE)
INDIVIDUAL DELIVERY AND LITTER DATA

TEST GROUP 1 (ADI-MIX)

	LITTER DELIVERED			NUMBER OF LIVE PUPS DAYS										DURATION OF GESTATION (DAYS)
	LIVE	DEAD	TOTAL	1		4		7		14		21		
FEMALE#	N	N	N	M	F	M	F	M	F	M	F	M	F	N
26	10	0	10	5	5	5	5	5	5	5	5	5	5	22
27	10	0	10	4	6	4	6	4	6	4	6	4	6	22
28	8	0	8	4	4	4	4	4	4	4	4	4	4	22
29	11	0	11	9	2	9	2	9	2	9	2	9	2	22
30	12	0	12	7	5	7	5	7	5	7	5	7	5	21
31	12	0	12	2	10	2	10	2	10	2	10	2	10	22
32	8	0	8	2	6	2	6	2	6	2	6	2	6	22
33	10	0	10	4	6	4	6	4	6	4	6	4	6	22
34	10	0	10	5	5	5	5	5	5	5	5	5	5	22
35	8	0	8	3	5	3	5	3	5	3	5	3	5	22
36	10	0	10	4	6	4	6	4	6	4	6	4	6	22
37	10	0	10	5	5	5	5	5	5	5	5	5	5	21
43	12	0	12	6	6	6	6	6	6	6	6	6	6	22
44	9	1	10	0	9	0	9	0	9	0	9	0	9	22
45	9	0	9	5	4	5	4	5	4	5	4	5	4	21
46	13	0	13	7	6	7	6	7	6	7	6	7	6	21
47	7	0	7	4	3	4	3	4	3	4	3	4	3	22
48	9	0	9	0	9	0	9	0	9	0	9	0	9	21
49	12	0	12	4	8	4	8	4	8	4	8	4	8	22
50	8	0	8	5	3	5	3	5	3	5	3	5	3	22

M=MALE, F=FEMALE

PR.NO.60R0375/88R002: REPRODUCTIVE TOX. STUDY TO DETECT EFFECTS
OF MIXED ANTI-ANDROGENIC SUBSTANCES IN RATS; ORAL ADM. (GAVAGE)
INDIVIDUAL DELIVERY AND LITTER DATA

TEST GROUP 2 (NOAEL-MIX)

	LITTER DELIVERED			NUMBER OF LIVE PUPS DAYS										DURATION OF GESTATION (DAYS)
	LIVE	DEAD	TOTAL	1		4		7		14		21		
FEMALE#	N	N	N	M	F	M	F	M	F	M	F	M	F	N
51	10	0	10	3	7	3	7	3	7	3	7	3	7	22
52	11	0	11	5	6	5	6	5	6	5	6	5	6	22
53	8	1	9	5	3	5	3	5	3	5	3	5	3	22
54	10	0	10	6	4	6	4	6	4	6	4	6	4	22
55	12	0	12	7	5	7	5	7	5	7	5	7	5	22
56	13	1	14	2	3	1	1	1	1	1	1	1	1	23
57	12	0	12	6	5	6	5	6	5	6	5	6	5	22
58	8	0	8	5	3	5	3	5	3	5	3	5	3	23
59	9	0	9	4	5	4	5	4	5	4	5	4	5	22
60	13	0	13	4	9	4	9	4	9	4	9	4	9	22
61	9	0	9	5	4	5	4	5	4	5	4	5	4	22
62	8	0	8	6	2	6	2	6	2	6	2	6	2	23
68	10	0	10	4	6	4	6	4	6	4	6	4	6	22
69	9	0	9	8	1	8	1	8	1	8	1	8	1	22
70	7	0	7	3	3	3	3	3	3	3	3	3	3	23
71	9	0	9	3	6	3	6	3	6	3	6	3	6	22
72	12	0	12	8	4	8	4	8	4	8	4	8	4	22
73	10	0	10	4	6	4	6	4	6	4	6	4	6	23
74	11	0	11	4	7	4	7	4	7	4	7	4	7	23
75	9	0	9	6	3	6	3	6	3	6	3	6	3	22

M=MALE, F=FEMALE

PR.NO.60R0375/88R002: REPRODUCTIVE TOX. STUDY TO DETECT EFFECTS
OF MIXED ANTI-ANDROGENIC SUBSTANCES IN RATS; ORAL ADM. (GAVAGE)
INDIVIDUAL DELIVERY AND LITTER DATA

TEST GROUP 3 (LOAEL-MIX)

		LITTER DELIVERED			NUMBER OF LIVE PUPS DAYS										DURATION OF GESTATION (DAYS)
		LIVE	DEAD	TOTAL	1		4		7		14		21		
FEMALE#		N	N	N	M	F	M	F	M	F	M	F	M	F	N
76	UDS														
77		8	0	8	4	4	4	4	4	4	4	4	4	4	22
78		9	1	10	7	2	7	2	7	2	7	2	7	2	23
79		0	5	5	0	0	0	0	0	0	0	0	0	0	24
80		5	4	9	3	2	3	2	3	2	3	1	3	1	23
81		0	11	11	0	0	0	0	0	0	0	0	0	0	23
82		11	0	11	6	5	6	5	6	5	6	5	6	5	23
83		9	1	10	4	5	4	5	4	5	4	5	4	5	23
84		7	0	7	6	1	6	1	6	1	6	1	6	1	23
85		8	2	10	1	5	1	5	1	5	1	5	1	5	23
86		11	0	11	5	6	5	6	5	6	5	6	5	6	23
87		12	0	12	5	7	5	7	5	7	5	7	5	7	23
93		7	0	7	5	2	5	2	5	2	5	2	5	2	23
94		10	0	10	6	4	6	4	6	4	6	4	6	4	23
95		10	0	10	4	6	4	6	4	6	4	6	4	6	22
96		9	0	9	5	4	5	4	5	4	5	4	5	4	23
97		9	0	9	7	2	7	2	7	2	7	2	7	2	23
98		10	1	11	4	6	4	6	4	6	4	6	4	6	23
99		9	1	10	5	4	5	4	5	4	5	4	5	4	23
100		9	0	9	4	5	4	5	4	5	4	5	4	5	23

M=MALE, F=FEMALE

UDS=UNABLE TO DELIVER, SAC'D

PR.NO.60R0375/88R002: REPRODUCTIVE TOX. STUDY TO DETECT EFFECTS
OF MIXED ANTI-ANDROGENIC SUBSTANCES IN RATS; ORAL ADM. (GAVAGE)
INDIVIDUAL DELIVERY AND LITTER DATA

TEST GROUP 4(0.00025 MG/KG BW/D)

FEMALE#		LITTER DELIVERED LIVE N	DEAD N	TOTAL N	NUMBER OF LIVE PUPS DAYS 1 M	1 F	4 M	4 F	7 M	7 F	14 M	14 F	21 M	21 F	DURATION OF GESTATION (DAYS) N
101		9	0	9	6	3	6	3	6	3	6	3	6	3	23
102		10	0	10	7	3	7	3	7	3	7	3	7	3	22
103		10	0	10	4	6	4	6	4	6	4	6	4	6	21
104		11	0	11	8	3	8	3	8	3	8	3	8	3	22
105		7	0	7	5	2	5	2	5	2	5	2	5	2	22
106		9	0	9	6	3	6	3	6	3	6	3	6	3	22
107		10	0	10	4	6	4	6	4	6	4	6	4	6	22
108		9	0	9	3	6	3	6	3	6	3	6	3	6	22
109		9	0	9	4	5	4	5	4	5	4	5	4	5	22
110		1	0	1	0	0	0	0	0	0	0	0	0	0	22
111		10	0	10	6	4	6	4	6	4	6	4	6	4	22
117		9	0	9	3	6	3	6	3	6	3	6	3	6	22
118		12	0	12	6	6	6	6	6	6	6	6	6	6	22
119		8	1	9	3	5	3	5	3	5	3	5	3	5	23
120		6	4	10	3	2	3	2	3	2	3	2	3	2	23
121		12	0	12	1	11	1	11	1	11	1	11	1	11	21
122	NP														
123		8	0	8	4	4	4	4	4	4	4	4	4	4	22
124		11	0	11	3	8	3	8	3	8	3	8	3	8	22
125		11	0	11	7	4	7	4	7	4	7	4	7	4	22

M=MALE, F=FEMALE

NP=NOT PREGNANT

PR.NO.60R0375/88R002: REPRODUCTIVE TOX. STUDY TO DETECT EFFECTS
OF MIXED ANTI-ANDROGENIC SUBSTANCES IN RATS; ORAL ADM. (GAVAGE)
INDIVIDUAL DELIVERY AND POSTIMPLANTATION LOSS DATA

TEST GROUP 0 (0 MG/KG BW/D)

FEMALE#	IMPLANTATION SITES	LITTER DELIVERED LIVE	DEAD	UNC.	TOTAL	POSTIMPLANTATION LOSS TOTAL	%
1	8	8	0	0	8	0	0.0
2	10	10	0	0	10	0	0.0
3	9	9	0	0	9	0	0.0
4	12	11	0	0	11	1	8.3
5	11	11	0	0	11	0	0.0
6	9	9	0	0	9	0	0.0
7	8	8	0	0	8	0	0.0
8	9	9	0	0	9	0	0.0
9	8	7	0	0	7	1	12.5
10	10	10	0	0	10	0	0.0
11	12	12	0	0	12	0	0.0
17	9	8	0	0	8	1	11.1
18	10	10	0	0	10	0	0.0
19	9	8	0	0	8	1	11.1
20	10	10	0	0	10	0	0.0
21	13	12	0	0	12	1	7.7
22	9	9	0	0	9	0	0.0
23	10	9	1	0	10	0	0.0
24	12	11	0	0	11	1	8.3
25	11	11	0	0	11	0	0.0

$ UNC. = UNCERTAIN

PR.NO.60R0375/88R002: REPRODUCTIVE TOX. STUDY TO DETECT EFFECTS
OF MIXED ANTI-ANDROGENIC SUBSTANCES IN RATS; ORAL ADM. (GAVAGE)
INDIVIDUAL DELIVERY AND POSTIMPLANTATION LOSS DATA

TEST GROUP 1 (ADI-MIX)

	IMPLANTATION	LITTER DELIVERED				POSTIMPLANTATION LOSS	
FEMALE#	SITES	LIVE	DEAD	UNC.	TOTAL	TOTAL	%
26	10	10	0	0	10	0	0.0
27	10	10	0	0	10	0	0.0
28	12	8	0	0	8	4	33.3
29	11	11	0	0	11	0	0.0
30	12	12	0	0	12	0	0.0
31	12	12	0	0	12	0	0.0
32	9	8	0	0	8	1	11.1
33	10	10	0	0	10	0	0.0
34	10	10	0	0	10	0	0.0
35	10	8	0	0	8	2	20.0
36	10	10	0	0	10	0	0.0
37	10	10	0	0	10	0	0.0
43	12	12	0	0	12	0	0.0
44	10	9	1	0	10	0	0.0
45	9	9	0	0	9	0	0.0
46	13	13	0	0	13	0	0.0
47	10	7	0	0	7	3	30.0
48	9	9	0	0	9	0	0.0
49	12	12	0	0	12	0	0.0
50	10	8	0	0	8	2	20.0

$ UNC. = UNCERTAIN

PR.NO.60R0375/88R002: REPRODUCTIVE TOX. STUDY TO DETECT EFFECTS
OF MIXED ANTI-ANDROGENIC SUBSTANCES IN RATS; ORAL ADM. (GAVAGE)
INDIVIDUAL DELIVERY AND POSTIMPLANTATION LOSS DATA

TEST GROUP 2 (NOAEL-MIX)

FEMALE#	IMPLANTATION SITES	LITTER DELIVERED LIVE	DEAD	UNC.	TOTAL	POSTIMPLANTATION LOSS TOTAL	%
51	10	10	0	0	10	0	0.0
52	11	11	0	0	11	0	0.0
53	9	8	1	0	9	0	0.0
54	10	10	0	0	10	0	0.0
55	12	12	0	0	12	0	0.0
56	14	13	1	0	14	0	0.0
57	12	12	0	0	12	0	0.0
58	10	8	0	0	8	2	20.0
59	10	9	0	0	9	1	10.0
60	14	13	0	0	13	1	7.1
61	10	9	0	0	9	1	10.0
62	10	8	0	0	8	2	20.0
68	10	10	0	0	10	0	0.0
69	9	9	0	0	9	0	0.0
70	9	7	0	0	7	2	22.2
71	9	9	0	0	9	0	0.0
72	12	12	0	0	12	0	0.0
73	10	10	0	0	10	0	0.0
74	11	11	0	0	11	0	0.0
75	9	9	0	0	9	0	0.0

$ UNC. = UNCERTAIN

PR.NO.60R0375/88R002: REPRODUCTIVE TOX. STUDY TO DETECT EFFECTS
OF MIXED ANTI-ANDROGENIC SUBSTANCES IN RATS; ORAL ADM. (GAVAGE)
INDIVIDUAL DELIVERY AND POSTIMPLANTATION LOSS DATA

TEST GROUP 3 (LOAEL-MIX)

FEMALE#	IMPLANTATION SITES	LITTER DELIVERED LIVE	DEAD	UNC.	TOTAL	POSTIMPLANTATION LOSS TOTAL	%
76 UDS							
77	9	8	0	0	8	1	11.1
78	12	9	1	0	10	2	16.7
79	5	0	5	0	5	0	0.0
80	9	5	4	0	9	0	0.0
81	12	0	11	0	11	1	8.3
82	11	11	0	0	11	0	0.0
83	11	9	1	0	10	1	9.1
84	9	7	0	0	7	2	22.2
85	10	8	2	0	10	0	0.0
86	11	11	0	0	11	0	0.0
87	12	12	0	0	12	0	0.0
93	9	7	0	0	7	2	22.2
94	10	10	0	0	10	0	0.0
95	10	10	0	0	10	0	0.0
96	11	9	0	0	9	2	18.2
97	11	9	0	0	9	2	18.2
98	11	10	1	0	11	0	0.0
99	10	9	1	0	10	0	0.0
100	10	9	0	0	9	1	10.0

$ UNC. = UNCERTAIN

UDS=UNABLE TO DELIVER, SAC'D

PR.NO.60R0375/88R002: REPRODUCTIVE TOX. STUDY TO DETECT EFFECTS
OF MIXED ANTI-ANDROGENIC SUBSTANCES IN RATS; ORAL ADM. (GAVAGE)
INDIVIDUAL DELIVERY AND POSTIMPLANTATION LOSS DATA

TEST GROUP 4(0.00025 MG/KG BW/D)

FEMALE#	IMPLANTATION SITES	LITTER DELIVERED LIVE	DEAD	UNC.	TOTAL	POSTIMPLANTATION LOSS TOTAL	%
101	9	9	0	0	9	0	0.0
102	11	10	0	0	10	1	9.1
103	10	10	0	0	10	0	0.0
104	11	11	0	0	11	0	0.0
105	7	7	0	0	7	0	0.0
106	9	9	0	0	9	0	0.0
107	10	10	0	0	10	0	0.0
108	10	9	0	0	9	1	10.0
109	10	9	0	0	9	1	10.0
110	1	1	0	0	1	0	0.0
111	10	10	0	0	10	0	0.0
117	9	9	0	0	9	0	0.0
118	12	12	0	0	12	0	0.0
119	10	8	1	0	9	1	10.0
120	11	6	4	0	10	1	9.1
121	12	12	0	0	12	0	0.0
122 NP							
123	8	8	0	0	8	0	0.0
124	12	11	0	0	11	1	8.3
125	12	11	0	0	11	1	8.3

$ UNC. = UNCERTAIN

NP=NOT PREGNANT

PR.NO.60R0375/88R002: REPRODUCTIVE TOX. STUDY TO DETECT EFFECTS
OF MIXED ANTI-ANDROGENIC SUBSTANCES IN RATS; ORAL ADM. (GAVAGE)
INDIVIDUAL PUP SEX AND STATUS POST PARTUM

TEST GROUP 0 (0 MG/KG BW/D)

FEMALE#	PUP # 1	2	3	4	5	6	7	8	9	10	11	12	13	14	15	16	17	18	19	20	21	22	23
1	MK21	MP	MK21	MP	MK21	MK21	FP	FK21															
2	MK21	MK21	MK21	MK21	MP	FK21	FK21	FP	FK21	FP													
3	MP	MK21	MK21	MK21	MK21	FP	FP	FK21	FK21														
4	MK21	MP	MK21	MP	FK21	FK21	FK21	FK21	FP	FK21	FK21												
5	MK21	MK21	MP	MK21	MK21	MK21	MK21	FP	FK21	FP	FK21												
6	MK21	MP	MK21	MP	FP	FK21	FK21	FK21	FK21														
7	MP	MK21	MK21	MK21	MK21	FP	FP	FK21															
8	MK21	MK21	MK21	MP	FK21	FK21	FP	FK21	FP														
9	MP	MK21	MK21	MP	FP	FK21	FK21																
10	MK21	MP	MK21	MK21	MP	FK21	FK21	FK21	FK21	FP													
11	MK21	MK21	MP	MK21	MK21	FP	FK21	FK21	FK21	FK21	FK21	FP											
17	MP	MK21	FK21	FP	FK21	FK21	FK21	FP															
18	MK21	MP	MP	MK21	MK21	FK21	FK21	FP	FK21	FK21													
19	MP	MK21	FK21	FP	FK21	FK21	FP	FK21															
20	MK21	MP	FP	FK21	FK21	FK21	FK21	FK21	FK21	FP													
21	MK21	MK21	MK21	MK21	MP	MK21	MP	FK21	FK21	FK21	FP	FK21											
22	MP	MK21	MK21	MP	FK21	FP	FK21	FK21	FK21														
23	MK21	MP	MK21	MS	FP	FK21	FK21	FP	FK21	FK21													
24	MK21	MK21	MP	MK21	MK21	MP	FP	FK21	FK21	FK21	FK21												
25	MP	MK21	MK21	MK21	MP	FK21	FK21	FK21	FP	FK21	FK21												

SEX CODES: M-MALE F-FEMALE U-UNCERTAIN
PUP STATUS CODES: A-ALIVE S-STILLBORN K-SCHEDULED SACRIFICE P-SELECTED AS PARENT/RAISED
NUMBER FOLLOWING PUP STATUS = DAY POST PARTUM OF DEATH

PR.NO.60R0375/88R002: REPRODUCTIVE TOX. STUDY TO DETECT EFFECTS
OF MIXED ANTI-ANDROGENIC SUBSTANCES IN RATS; ORAL ADM. (GAVAGE)
INDIVIDUAL PUP SEX AND STATUS POST PARTUM

TEST GROUP 1 (ADI-MIX)

FEMALE#	PUP # 1	2	3	4	5	6	7	8	9	10	11	12	13	14	15	16	17	18	19	20	21	22	23
26	MK21	MP	MP	MK21	MK21	FK21	FP	FK21	FK21	FK21													
27	MP	MK21	MK21	MK21	FK21	FP	FK21	FK21	FP	FK21													
28	MP	MP	MK21	MK21	FP	FK21	FK21	FK21															
29	MK21	MK21	MK21	MK21	MK21	MP	MK21	MP	MK21	FP	FK21												
30	MK21	MK21	MK21	MP	MK21	MK21	MK21	FK21	FP	FK21	FK21	FP											
31	MP	MK21	FP	FK21	FK21	FK21	FK21	FK21	FK21	FP	FK21	FK21											
32	MK21	MP	FK21	FP	FK21	FK21	FK21	FP															
33	MP	MP	MK21	MK21	FK21	FP	FK21	FK21	FK21	FK21													
34	MK21	MK21	MP	MK21	MP	FK21	FK21	FK21	FP	FK21													
35	MK21	MK21	MP	FP	FK21	FK21	FK21	FP															
36	MP	MK21	MK21	MP	FK21	FP	FK21	FK21	FK21	FK21													
37	MK21	MK21	MK21	MK21	MP	FK21	FP	FK21	FP	FK21													
43	MK21	MK21	MK21	MP	MK21	MP	FK21	FK21	FK21	FP	FK21	FK21											
44	MS	FP	FK21	FK21	FP	FK21	FK21	FP	FK21	FK21													
45	MK21	MK21	MP	MP	MK21	FP	FK21	FK21	FK21														
46	MP	MK21	MK21	MK21	MK21	MK21	MP	FK21	FK21	FK21	FK21	FK21	FP										
47	MK21	MP	MP	MK21	FP	FK21	FK21																
48	FP	FK21	FK21	FK21	FK21	FP	FK21	FP	FK21														
49	MK21	MP	MK21	MP	FK21	FK21	FK21	FK21	FK21	FP	FK21	FK21											
50	MP	MP	MK21	MK21	MK21	FK21	FK21	FP															

SEX CODES: M-MALE F-FEMALE U-UNCERTAIN
PUP STATUS CODES: A-ALIVE S-STILLBORN K-SCHEDULED SACRIFICE P-SELECTED AS PARENT/RAISED
NUMBER FOLLOWING PUP STATUS = DAY POST PARTUM OF DEATH

PR.NO.60R0375/88R002: REPRODUCTIVE TOX. STUDY TO DETECT EFFECTS
OF MIXED ANTI-ANDROGENIC SUBSTANCES IN RATS; ORAL ADM. (GAVAGE)
INDIVIDUAL PUP SEX AND STATUS POST PARTUM

TEST GROUP 2 (NOAEL-MIX)

FEMALE#	PUP # 1	2	3	4	5	6	7	8	9	10	11	12	13	14	15	16	17	18	19	20	21	22	23
51	MK21	MP	MK21	FP	FK21	FK21	FP	FK21	FK21	FK21													
52	MK21	MK21	MP	MK21	MK21	FP	FK21	FK21	FK21	FP	FK21												
53	MP	MP	MK21	MK21	MK21	MS	FK21	FP	FK21														
54	MK21	MK21	MP	MK21	MP	MK21	FP	FK21	FK21	FK21													
55	MK21	MK21	MK21	MP	MK21	MK21	MK21	FP	FK21	FP	FK21	FK21											
56	MZ 3	MP	MD 1	MD 1	MD 1	FZ 3	FK21	FD 2	FZ 1	FD 1	FD 1	FD 1	FZ 1	FS									
57	MP	MK21	MK21	MK21	MP	MK21	MZ 1	FK21	FK21	FK21	FK21	FP											
58	MK21	MK21	MP	MP	MK21	FP	FK21	FK21															
59	MK21	MK21	MP	MK21	FP	FK21	FP	FK21	FK21														
60	MP	MK21	MK21	MK21	FK21	FK21	FK21	FP	FK21	FK21	FP	FK21	FK21										
61	MK21	MP	MK21	MK21	MP	FK21	FK21	FK21	FP														
62	MK21	MK21	MP	MP	MK21	MK21	FP	FK21															
68	MP	MK21	MK21	MK21	FK21	FP	FK21	FK21	FK21	FP													
69	MK21	MK21	MK21	MK21	MP	MK21	MK21	MP	FP														
70	MP	MK21	MK21	FP	FK21	FP	FZ 1																
71	MK21	MK21	MP	FK21	FK21	FP	FK21	FK21	FP														
72	MP	MK21	MK21	MK21	MP	MK21	MK21	MK21	FK21	FK21	FP	FK21											
73	MK21	MP	MK21	MK21	FK21	FP	FP	FK21	FP	FK21													
74	MP	MK21	MK21	MP	FK21	FP	FK21	FP	FK21	FK21	FK21												
75	MK21	MK21	MP	MK21	MK21	MP	FP	FK21	FK21														

SEX CODES: M-MALE F-FEMALE U-UNCERTAIN
PUP STATUS CODES: A-ALIVE S-STILLBORN D-DIED Z-CANNIBALIZED K-SCHEDULED SACRIFICE P-SELECTED AS PARENT/RAISED
NUMBER FOLLOWING PUP STATUS = DAY POST PARTUM OF DEATH

PR.NO.60R0375/88R002: REPRODUCTIVE TOX. STUDY TO DETECT EFFECTS
OF MIXED ANTI-ANDROGENIC SUBSTANCES IN RATS; ORAL ADM. (GAVAGE)
INDIVIDUAL PUP SEX AND STATUS POST PARTUM

TEST GROUP 3 (LOAEL-MIX)

FEMALE#	PUP # 1	2	3	4	5	6	7	8	9	10	11	12	13	14	15	16	17	18	19	20	21	22	23
76																			UNABLE TO DELIVER, SAC'D				
77	MK21	MK21	MP	MK21	FP	FK21	FK21	FP															
78	MK21	MP	MK21	MK21	MK21	MK21	MP	FK21	FP	FS													
79	MS	MS	MS	FS	FS																		
80	MP	MK21	MP	MS	MS	FZ 9	FP	FS	FS														
81	MS	MS	MS	MS	MS	MS	FS	FS	FS	FS	FS												
82	MK21	MP	MK21	MK21	MK21	MP	FK21	FP	FK21	FK21	FK21												
83	MK21	MK21	MK21	MP	MS	FK21	FK21	FK21	FP	FP													
84	MP	MK21	MP	MK21	MK21	MK21	FP																
85	MP	MZ 1	MS	MS	FP	FK21	FK21	FK21	FP	FZ 1													
86	MK21	MP	MK21	MP	MK21	FP	FK21	FK21	FK21	FK21	FK21												
87	MP	MK21	MK21	MK21	MP	FK21	FP	FK21	FK21	FP	FK21	FK21											
93	MK21	MK21	MP	MK21	MP	FP	FK21																
94	MK21	MK21	MK21	MP	MK21	MK21	FP	FP	FP	FK21													
95	MP	MK21	MK21	MK21	FK21	FK21	FP	FK21	FK21	FP													
96	MK21	MK21	MP	MK21	MP	FP	FK21	FP	FP														
97	MK21	MK21	MK21	MP	MK21	MK21	MP	FK21	FP														
98	MK21	MP	MP	MK21	FP	FP	FK21	FK21	FK21	FP	FS												
99	MP	MP	MP	MK21	MK21	MS	FP	FP	FK21	FK21													
100	MP	MK21	MK21	MP	FP	FK21	FK21	FK21	FP														

SEX CODES: M-MALE F-FEMALE U-UNCERTAIN
PUP STATUS CODES: A-ALIVE S-STILLBORN Z-CANNIBALIZED K-SCHEDULED SACRIFICE P-SELECTED AS PARENT/RAISED
NUMBER FOLLOWING PUP STATUS = DAY POST PARTUM OF DEATH

PR.NO.60R0375/88R002: REPRODUCTIVE TOX. STUDY TO DETECT EFFECTS
OF MIXED ANTI-ANDROGENIC SUBSTANCES IN RATS; ORAL ADM. (GAVAGE)
INDIVIDUAL PUP SEX AND STATUS POST PARTUM

TEST GROUP 4(0.00025 MG/KG BW/D)

FEMALE#	PUP # 1	2	3	4	5	6	7	8	9	10	11	12	13	14	15	16	17	18	19	20	21	22	23
101	MK21	MP	MK21	MK21	MK21	MP	FK21	FP	FK21														
102	MK21	MK21	MP	MK21	MP	MK21	MK21	FK21	FP	FK21													
103	MP	MP	MK21	MP	FK21	FK21	FP	FK21	FK21	FP													
104	MP	MK21	MK21	MK21	MK21	MK21	MK21	MP	FK21	FK21	FP												
105	MK21	MK21	MP	MP	MK21	FP	FK21																
106	MK21	MP	MK21	MK21	MK21	MK21	FP	FP	FK21														
107	MP	MK21	MK21	MK21	FP	FP	FK21	FK21	FK21	FK21													
108	MK21	MK21	MP	FK21	FK21	FK21	FP	FP	FK21														
109	MK21	MK21	MP	MK21	FP	FK21	FK21	FK21	FP														
110	FD 1																						
111	MK21	MK21	MP	MK21	MK21	MP	FK21	FK21	FP	FK21													
117	MK21	MP	MK21	FK21	FK21	FK21	FP	FP	FK21														
118	MP	MK21	MK21	MK21	MP	MK21	FK21	FK21	FK21	FK21	FP	FK21											
119	MP	MP	MK21	MS	FP	FK21	FP	FK21	FK21														
120	MK21	MK21	MP	MS	FP	FP	FZ 1	FS	FS	FS													
121	MK91	FK91	FK21	FK21	FK21	FK21	FK21	FK21	FP	FK21	FK21	FK21											
123	MK89	MK21	MK21	MP	FK21	FK89	FK21	FK21															
124	MK21	MK89	MK89	FK21	FK89	FK21	FK21	FK21	FK89	FK21	FP												
125	MP	MK21	MK21	MK21	MK21	MK21	MK89	FP	FK21	FK89	FK21												

SEX CODES: M-MALE F-FEMALE U-UNCERTAIN
PUP STATUS CODES: A-ALIVE S-STILLBORN D-DIED Z-CANNIBALIZED K-SCHEDULED SACRIFICE P-SELECTED AS PARENT/RAISED
NUMBER FOLLOWING PUP STATUS = DAY POST PARTUM OF DEATH

PR.NO.60R0375/88R002: REPRODUCTIVE TOX. STUDY TO DETECT EFFECTS
OF MIXED ANTI-ANDROGENIC SUBSTANCES IN RATS; ORAL ADM. (GAVAGE)
NUMBER OF PUPS WITH MEASURE PRESENT / NUMBER TESTED

TEST GROUP 0 (0 MG/KG BW/D) AREOLAS/NIPPLES

	POSTPARTUM DAY
FEMALE#	12
1	4/ 6
2	4/ 5
3	3/ 5
4	1/ 4
5	6/ 7
6	2/ 4
7	2/ 5
8	3/ 4
9	4/ 4
10	4/ 5
11	2/ 5
17	2/ 2
18	4/ 5
19	1/ 2
20	2/ 2
21	6/ 7
22	3/ 4
23	3/ 3
24	4/ 6
25	3/ 5
TOTAL	63/ 90
CUMULATIVE	63/ 90<br>70%

PR.NO.60R0375/88R002: REPRODUCTIVE TOX. STUDY TO DETECT EFFECTS
OF MIXED ANTI-ANDROGENIC SUBSTANCES IN RATS; ORAL ADM. (GAVAGE)
NUMBER OF PUPS WITH MEASURE PRESENT / NUMBER TESTED

TEST GROUP 1 (ADI-MIX) AREOLAS/NIPPLES

FEMALE#	POSTPARTUM DAY 12
26	2/ 5
27	4/ 4
28	4/ 4
29	5/ 9
30	5/ 7
31	1/ 2
32	1/ 2
33	3/ 4
34	3/ 5
35	1/ 3
36	2/ 4
37	3/ 5
43	1/ 6
45	4/ 5
46	5/ 7
47	4/ 4
49	4/ 4
50	4/ 5
TOTAL	56/ 85
CUMULATIVE	56/ 85<br>66%

PR.NO.60R0375/88R002: REPRODUCTIVE TOX. STUDY TO DETECT EFFECTS
OF MIXED ANTI-ANDROGENIC SUBSTANCES IN RATS; ORAL ADM. (GAVAGE)
NUMBER OF PUPS WITH MEASURE PRESENT / NUMBER TESTED

TEST GROUP 2 (NOAEL-MIX) AREOLAS/NIPPLES

FEMALE#	POSTPARTUM DAY 12
51	3/ 3
52	3/ 5
53	4/ 5
54	4/ 6
55	5/ 7
56	0/ 1
57	4/ 6
58	1/ 5
59	4/ 4
60	1/ 4
61	2/ 5
62	4/ 6
68	4/ 4
69	6/ 8
70	2/ 3
71	2/ 3
72	8/ 8
73	3/ 4
74	3/ 4
75	5/ 6
TOTAL	68/ 97
CUMULATIVE	68/ 97<br>70%

PR.NO.60R0375/88R002: REPRODUCTIVE TOX. STUDY TO DETECT EFFECTS
OF MIXED ANTI-ANDROGENIC SUBSTANCES IN RATS; ORAL ADM. (GAVAGE)
NUMBER OF PUPS WITH MEASURE PRESENT / NUMBER TESTED

TEST GROUP 3 (LOAEL-MIX) AREOLAS/NIPPLES

FEMALE#	POSTPARTUM DAY 12
77	4/ 4
78	7/ 7
80	3/ 3
82	6/ 6
83	4/ 4
84	6/ 6
85	1/ 1
86	5/ 5
87	5/ 5
93	5/ 5
94	6/ 6
95	4/ 4
96	5/ 5
97	7/ 7
98	4/ 4
99	5/ 5
100	4/ 4
TOTAL	81/ 81
CUMULATIVE	81/ 81<br>100%

PR.NO.60R0375/88R002: REPRODUCTIVE TOX. STUDY TO DETECT EFFECTS
OF MIXED ANTI-ANDROGENIC SUBSTANCES IN RATS; ORAL ADM. (GAVAGE)
NUMBER OF PUPS WITH MEASURE PRESENT / NUMBER TESTED

TEST GROUP 4(0.00025 MG/KG BW/D) AREOLAS/NIPPLES

FEMALE#	POSTPARTUM DAY 12
101	0/ 6
102	3/ 7
103	4/ 4
104	3/ 8
105	4/ 5
106	1/ 6
107	4/ 4
108	2/ 3
109	2/ 4
111	4/ 6
117	1/ 3
118	4/ 6
119	0/ 3
120	1/ 3
121	0/ 1
123	4/ 4
124	3/ 3
125	6/ 7
TOTAL	46/ 83
CUMULATIVE	46/ 83<br>55%

PR.NO.60R0375/88R002: REPRODUCTIVE TOX. STUDY TO DETECT EFFECTS
OF MIXED ANTI-ANDROGENIC SUBSTANCES IN RATS; ORAL ADM. (GAVAGE)
NUMBER OF PUPS WITH MEASURE PRESENT / NUMBER TESTED

TEST GROUP 0 (0 MG/KG BW/D) AREOLAS/NIPPLES - SECOND OBS.

FEMALE#	POSTPARTUM DAY 20
1	0/ 6
2	0/ 5
3	0/ 5
4	0/ 4
5	0/ 7
6	0/ 4
7	0/ 5
8	0/ 4
9	0/ 4
10	0/ 5
11	0/ 5
17	0/ 2
18	0/ 5
19	0/ 2
20	0/ 2
21	0/ 7
22	0/ 4
23	0/ 3
24	0/ 6
25	0/ 5
TOTAL	0/ 90

PR.NO.60R0375/88R002: REPRODUCTIVE TOX. STUDY TO DETECT EFFECTS
OF MIXED ANTI-ANDROGENIC SUBSTANCES IN RATS; ORAL ADM. (GAVAGE)
NUMBER OF PUPS WITH MEASURE PRESENT / NUMBER TESTED

TEST GROUP 1 (ADI-MIX) AREOLAS/NIPPLES - SECOND OBS.

FEMALE#	POSTPARTUM DAY 20
26	0/ 5
27	0/ 4
28	0/ 4
29	0/ 9
30	0/ 7
31	0/ 2
32	0/ 2
33	0/ 4
34	0/ 5
35	0/ 3
36	0/ 4
37	0/ 5
43	0/ 6
45	0/ 5
46	0/ 7
47	0/ 4
49	0/ 4
50	0/ 5
TOTAL	0/ 85

PR.NO.60R0375/88R002: REPRODUCTIVE TOX. STUDY TO DETECT EFFECTS
OF MIXED ANTI-ANDROGENIC SUBSTANCES IN RATS; ORAL ADM. (GAVAGE)
NUMBER OF PUPS WITH MEASURE PRESENT / NUMBER TESTED

TEST GROUP 2 (NOAEL-MIX) AREOLAS/NIPPLES - SECOND OBS.

FEMALE#	POSTPARTUM DAY 20
51	0/ 3
52	0/ 5
53	0/ 5
54	0/ 6
55	0/ 7
56	0/ 1
57	0/ 6
58	0/ 5
59	0/ 4
60	0/ 4
61	0/ 5
62	0/ 6
68	0/ 4
69	0/ 8
70	0/ 3
71	0/ 3
72	0/ 8
73	0/ 4
74	0/ 4
75	0/ 6
TOTAL	0/ 97

PR.NO.60R0375/88R002: REPRODUCTIVE TOX. STUDY TO DETECT EFFECTS
OF MIXED ANTI-ANDROGENIC SUBSTANCES IN RATS; ORAL ADM. (GAVAGE)
NUMBER OF PUPS WITH MEASURE PRESENT / NUMBER TESTED

TEST GROUP 3 (LOAEL-MIX) AREOLAS/NIPPLES - SECOND OBS.

FEMALE#	POSTPARTUM DAY 20
77	0/ 4
78	5/ 7
80	1/ 3
82	4/ 6
83	3/ 4
84	4/ 6
85	1/ 1
86	2/ 5
87	3/ 5
93	3/ 5
94	2/ 6
95	3/ 4
96	1/ 5
97	1/ 7
98	0/ 4
99	3/ 5
100	4/ 4
TOTAL	40/ 81

PR.NO.60R0375/88R002: REPRODUCTIVE TOX. STUDY TO DETECT EFFECTS
OF MIXED ANTI-ANDROGENIC SUBSTANCES IN RATS; ORAL ADM. (GAVAGE)
NUMBER OF PUPS WITH MEASURE PRESENT / NUMBER TESTED

TEST GROUP 4(0.00025 MG/KG BW/D) AREOLAS/NIPPLES - SECOND OBS.

FEMALE#	POSTPARTUM DAY 20
101	0/ 6
102	0/ 7
103	0/ 4
104	0/ 8
105	0/ 5
106	0/ 6
107	0/ 4
108	0/ 3
109	0/ 4
111	0/ 6
117	0/ 3
118	0/ 6
119	0/ 3
120	0/ 3
121	0/ 1
123	0/ 4
124	0/ 3
125	0/ 7
TOTAL	0/ 83

PR.NO.60R0375/88R002: REPRODUCTIVE TOX. STUDY TO DETECT EFFECTS
OF MIXED ANTI-ANDROGENIC SUBSTANCES IN RATS; ORAL ADM. (GAVAGE)
INDIVIDUAL PUP DATA -- AG DIST DAY 1 IN MM

TEST GROUP 0 (0 MG/KG BW/D)

FEMALE#	MEAN	PUP# 1	2	3	4	5	6	7	8	9	10	11	12	13	14	15	16	17	18	19	20	21	22	23
1	2.55	2.80	3.00	3.00	3.10	3.10	2.90	1.20	1.30															
2	2.20	3.10	2.90	3.00	3.10	3.10	1.20	1.30	1.50	1.40	1.40													
3	2.37	3.10	3.20	3.00	3.00	2.90	1.50	1.50	1.50	1.60														
4	1.95	2.90	3.10	3.20	2.80	1.50	1.40	1.20	1.40	1.30	1.30	1.40												
5	2.45	3.10	3.10	2.90	3.00	3.10	3.00	2.90	1.50	1.50	1.40	1.40												
6	2.26	3.10	3.20	3.20	3.00	1.50	1.50	1.60	1.50	1.70														
7	2.45	3.00	3.00	3.20	3.30	3.20	1.30	1.20	1.40															
8	2.14	3.00	3.00	3.00	3.00	1.50	1.50	1.30	1.50	1.50														
9	2.31	3.00	3.00	2.90	3.00	1.50	1.40	1.40																
10	2.19	3.00	2.90	3.00	2.90	3.00	1.30	1.40	1.50	1.40	1.50													
11	2.13	3.00	3.00	3.00	3.00	3.00	1.50	1.50	1.50	1.50	1.50	1.50	1.50											
17	1.86	3.20	3.10	1.40	1.50	1.40	1.50	1.40	1.40															
18	2.36	3.20	3.40	3.50	3.40	3.00	1.20	1.30	1.40	1.60	1.60													
19	1.76	2.90	3.00	1.40	1.40	1.30	1.40	1.30	1.40															
20	1.84	3.20	3.10	1.50	1.40	1.60	1.50	1.50	1.60	1.50	1.50													
21	2.43	3.10	3.00	3.00	3.10	3.00	3.10	3.00	1.60	1.50	1.60	1.60	1.50											
22	2.13	3.00	2.90	3.00	2.90	1.60	1.50	1.50	1.40	1.40														
23	1.91	3.00	3.00	3.00	S	1.20	1.50	1.20	1.40	1.40	1.50													
24	2.41	3.00	3.60	3.00	3.20	3.00	3.40	1.50	1.50	1.30	1.50	1.50												
25	1.99	2.80	2.90	3.00	2.70	2.70	1.20	1.40	1.20	1.30	1.40	1.30												

MEAN 2.18
S.D. 0.234
N 20

PUP STATUS CODES: S-STILLBORN

PR.NO.60R0375/88R002: REPRODUCTIVE TOX. STUDY TO DETECT EFFECTS
OF MIXED ANTI-ANDROGENIC SUBSTANCES IN RATS; ORAL ADM. (GAVAGE)
INDIVIDUAL PUP DATA -- AG DIST DAY 1 IN MM

TEST GROUP 1 (ADI-MIX)

FEMALE#	MEAN	PUP# 1	2	3	4	5	6	7	8	9	10	11	12	13	14	15	16	17	18	19	20	21	22	23
26	2.27	3.00	3.10	3.10	2.90	3.00	1.50	1.40	1.50	1.60	1.60													
27	2.13	3.20	3.10	3.10	3.20	1.40	1.50	1.40	1.40	1.50	1.50													
28	2.10	2.90	3.00	3.10	2.80	1.30	1.20	1.20	1.30															
29	2.72	3.00	2.90	3.00	3.00	3.10	3.00	3.00	2.90	3.10	1.50	1.40												
30	2.40	3.10	3.10	3.10	3.10	3.00	3.00	3.10	1.40	1.50	1.50	1.40	1.50											
31	1.71	3.00	3.00	1.40	1.40	1.40	1.50	1.50	1.40	1.50	1.40	1.50	1.50											
32	1.83	3.00	3.00	1.40	1.50	1.60	1.40	1.40	1.30															
33	2.11	3.30	3.00	3.10	3.00	1.50	1.40	1.50	1.50	1.40	1.40													
34	2.07	3.00	2.50	2.80	2.80	3.00	1.20	1.30	1.30	1.40	1.40													
35	2.05	3.00	3.00	3.10	1.40	1.40	1.50	1.50	1.50															
36	2.03	2.80	2.90	3.00	2.90	1.40	1.40	1.50	1.50	1.50	1.40													
37	1.80	2.50	2.50	2.30	2.50	2.60	1.20	1.10	1.00	1.20	1.10													
43	2.23	3.40	3.00	3.20	2.80	3.00	2.60	1.40	1.50	1.40	1.50	1.50	1.50											
44	1.46	S	1.50	1.50	1.40	1.50	1.50	1.30	1.50	1.50	1.40													
45	2.14	2.90	2.90	3.00	2.80	2.80	1.30	1.20	1.20	1.20														
46	2.13	3.00	3.00	2.60	3.00	2.80	2.90	3.00	1.20	1.20	1.30	1.20	1.20	1.30										
47	2.37	3.10	3.00	3.00	3.10	1.60	1.40	1.40																
48	1.20	1.20	1.30	1.20	1.30	1.30	1.20	1.10	1.10	1.10														
49	1.83	3.00	2.80	2.80	2.80	1.30	1.30	1.30	1.30	1.50	1.40	1.20	1.30											
50	2.60	3.50	3.30	3.00	3.50	3.00	1.50	1.50	1.50															

MEAN	2.06
S.D.	0.358
N	20

PUP STATUS CODES: S-STILLBORN

PR.NO.60R0375/88R002: REPRODUCTIVE TOX. STUDY TO DETECT EFFECTS
OF MIXED ANTI-ANDROGENIC SUBSTANCES IN RATS; ORAL ADM. (GAVAGE)
INDIVIDUAL PUP DATA -- AG DIST DAY 1 IN MM

TEST GROUP 2 (NOAEL-MIX)

FEMALE#	MEAN	PUP# 1	2	3	4	5	6	7	8	9	10	11	12	13	14	15	16	17	18	19	20	21	22	23
51	1.91	3.00	3.20	3.00	1.40	1.30	1.40	1.50	1.50	1.50	1.30													
52	2.18	3.00	3.10	3.00	3.10	3.10	1.40	1.50	1.40	1.50	1.50	1.40												
53	2.42	3.00	2.90	2.90	3.00	3.30	S	1.50	1.30	1.50														
54	2.47	3.30	3.20	3.00	3.10	3.00	3.10	1.60	1.50	1.50	1.40													
55	2.20	3.00	3.00	2.60	2.60	3.00	2.50	3.00	1.30	1.30	1.50	1.30	1.30											
56	2.08	3.20	3.10	D	D	D	1.60	1.60	1.50	1.50	D	D	D	Z	S									
57	2.25	3.10	2.90	2.80	2.80	3.00	2.90	Z	1.50	1.40	1.50	1.40	1.40											
58	2.74	3.20	3.40	3.30	3.40	3.40	1.80	1.70	1.70															
59	2.08	2.90	2.80	2.90	2.90	1.40	1.40	1.50	1.40	1.50														
60	1.90	2.80	2.80	2.90	3.00	1.50	1.40	1.50	1.40	1.50	1.50	1.50	1.40	1.50										
61	2.11	2.60	2.80	2.80	2.90	2.70	1.30	1.30	1.30	1.30														
62	2.63	3.00	2.90	3.00	3.00	3.10	3.00	1.60	1.40															
68	2.10	2.90	3.00	2.90	2.80	1.60	1.50	1.50	1.60	1.60	1.60													
69	2.91	3.50	3.00	3.30	3.00	3.00	3.30	2.70	3.20	1.20														
70	2.57	3.50	3.30	3.50	1.50	1.80	1.80	Z																
71	2.02	3.00	3.00	2.90	1.50	1.60	1.60	1.50	1.60	1.50														
72	2.57	3.20	3.00	2.90	3.00	3.50	3.00	3.10	3.20	1.40	1.50	1.50	1.50											
73	2.18	3.20	3.30	3.30	3.00	1.70	1.50	1.50	1.50	1.50	1.30													
74	2.08	3.30	3.20	3.30	3.30	1.40	1.30	1.50	1.50	1.40	1.30	1.40												
75	2.66	3.50	3.00	3.50	3.20	2.90	3.50	1.50	1.50	1.30														

MEAN	2.30
S.D.	0.293
N	20

PUP STATUS CODES: S-STILLBORN D -DIED Z -CANNIBALIZED

PR.NO.60R0375/88R002: REPRODUCTIVE TOX. STUDY TO DETECT EFFECTS
OF MIXED ANTI-ANDROGENIC SUBSTANCES IN RATS; ORAL ADM. (GAVAGE)
INDIVIDUAL PUP DATA -- AG DIST DAY 1 IN MM

TEST GROUP 3 (LOAEL-MIX)

FEMALE#	MEAN	PUP# 1	2	3	4	5	6	7	8	9	10	11	12	13	14	15	16	17	18	19
76	UDS																			
77	2.20	2.90	3.00	2.80	2.90	1.50	1.50	1.40	1.60											
78	2.88	3.00	3.50	3.00	3.00	3.50	3.00	3.20	1.70	2.00	S									
80	2.22	2.60	2.70	2.70	S	S	1.50	1.60	S	S										
82	2.28	2.90	2.90	2.80	2.90	3.00	2.80	1.60	1.60	1.60	1.50	1.50								
83	2.43	3.40	3.50	3.40	3.00	S	1.70	1.90	1.50	1.70	1.80									
84	2.49	2.70	2.60	2.60	2.70	2.60	2.60	1.60												
85	1.80	3.00	Z	S	S	1.50	1.60	1.50	1.60	1.60	Z									
86	2.21	3.10	2.90	2.90	3.00	2.70	1.60	1.60	1.70	1.60	1.60	1.60								
87	2.33	3.30	2.80	3.00	3.00	3.00	1.80	2.00	1.70	1.80	2.00	1.80	1.80							
93	2.37	2.80	2.80	2.50	2.80	2.60	1.50	1.60												
94	2.60	3.40	3.50	3.00	3.00	3.20	3.00	1.50	1.70	2.00	1.70									
95	1.82	2.80	2.50	2.50	2.20	1.20	1.30	1.50	1.30	1.40	1.50									
96	2.62	3.60	3.00	3.50	3.20	3.20	2.00	1.70	1.70	1.70										
97	2.77	3.20	3.20	3.00	3.00	3.00	3.10	2.80	1.80	1.80										
98	2.03	2.80	3.00	2.70	2.70	1.50	1.50	1.50	1.50	1.60	1.50	S								
99	2.48	2.90	3.00	3.00	3.00	3.20	S	1.70	2.00	1.70	1.80									
100	2.16	2.80	3.00	3.00	2.80	1.50	1.70	1.60	1.50	1.50										

MEAN	2.33
S.D.	0.297
N	17

PUP STATUS CODES: S-STILLBORN Z -CANNIBALIZED

UDS=UNABLE TO DELIVER, SAC'D

PR.NO.60R0375/88R002: REPRODUCTIVE TOX. STUDY TO DETECT EFFECTS
OF MIXED ANTI-ANDROGENIC SUBSTANCES IN RATS; ORAL ADM. (GAVAGE)
INDIVIDUAL PUP DATA -- AG DIST DAY 1 IN MM

TEST GROUP 4(0.00025 MG/KG BW/D)

FEMALE#	MEAN	PUP# 1	2	3	4	5	6	7	8	9	10	11	12	13	14	15	16	17	18	19	20	21	22	23
101	2.67	3.10	3.20	3.10	3.20	3.10	3.30	1.70	1.60	1.70														
102	2.55	3.00	3.00	3.30	3.00	3.00	3.00	3.20	1.30	1.40	1.30													
103	2.12	2.90	2.80	3.00	3.10	1.60	1.60	1.50	1.60	1.60	1.50													
104	2.55	3.00	3.10	3.00	3.00	3.00	3.00	3.00	3.00	1.30	1.50	1.20												
105	2.50	3.00	2.90	2.90	2.80	3.00	1.40	1.50																
106	2.68	3.10	3.10	3.10	3.30	3.20	3.30	1.70	1.60	1.70														
107	2.08	2.90	3.10	3.10	2.90	1.40	1.50	1.40	1.50	1.50	1.50													
108	2.02	3.10	3.00	3.00	1.60	1.50	1.50	1.50	1.60	1.40														
109	2.11	3.00	2.80	3.00	2.80	1.50	1.50	1.40	1.50	1.50														
111	2.18	3.00	3.00	3.00	2.50	2.60	2.80	1.30	1.20	1.10	1.30													
117	1.91	3.00	3.00	3.20	1.30	1.30	1.30	1.30	1.50	1.30														
118	2.19	2.90	2.90	3.00	2.90	3.00	2.80	1.50	1.40	1.40	1.50	1.50	1.50											
119	2.60	3.80	3.80	3.80	S	2.00	2.00	2.00	1.60	1.80														
120	2.56	3.30	3.40	3.00	S	1.50	1.60	Z	S	S	S													
121	1.38	2.90	1.30	1.20	1.20	1.20	1.20	1.20	1.30	1.30	1.30	1.20	1.20											
123	2.19	3.00	3.00	3.00	3.20	1.40	1.50	1.20	1.20															
124	1.85	3.20	3.00	3.00	1.40	1.50	1.20	1.30	1.30	1.40	1.50	1.50												
125	2.41	3.10	3.10	3.00	3.00	2.90	3.00	2.80	1.50	1.30	1.50	1.30												

MEAN	2.25
S.D.	0.345
N	18

PUP STATUS CODES: S-STILLBORN Z -CANNIBALIZED

PR.NO.60R0375/88R002: REPRODUCTIVE TOX. STUDY TO DETECT EFFECTS
OF MIXED ANTI-ANDROGENIC SUBSTANCES IN RATS; ORAL ADM. (GAVAGE)
INDIVIDUAL PUP DATA -- AG DIST DAY 1 to BODY WEIGHT RATIO (AG Index)

TEST GROUP 0 (0 MG/KG BW/D)

FEMALE#	MEAN	PUP# 1	2	3	4	5	6	7	8	9	10	11	12	13	14	15	16	17	18	19	20	21	22	23
1	0.34	0.41	0.38	0.38	0.39	0.39	0.40	0.16	0.18															
2	0.31	0.41	0.43	0.39	0.40	0.46	0.17	0.19	0.20	0.20	0.20													
3	0.33	0.44	0.48	0.42	0.39	0.39	0.20	0.21	0.20	0.27														
4	0.29	0.41	0.44	0.45	0.47	0.22	0.20	0.19	0.21	0.19	0.20	0.21												
5	0.36	0.44	0.48	0.46	0.43	0.42	0.41	0.47	0.23	0.20	0.23	0.22												
6	0.31	0.42	0.44	0.45	0.41	0.21	0.20	0.24	0.21	0.24														
7	0.34	0.40	0.45	0.43	0.43	0.46	0.18	0.16	0.20															
8	0.30	0.40	0.39	0.45	0.45	0.21	0.22	0.18	0.21	0.22														
9	0.33	0.42	0.43	0.41	0.43	0.23	0.21	0.22																
10	0.32	0.42	0.48	0.41	0.42	0.43	0.20	0.21	0.21	0.22	0.23													
11	0.33	0.46	0.44	0.56	0.43	0.45	0.22	0.23	0.22	0.25	0.23	0.21	0.24											
17	0.27	0.43	0.44	0.20	0.23	0.24	0.21	0.22	0.22															
18	0.32	0.44	0.46	0.46	0.45	0.42	0.18	0.18	0.19	0.20	0.21													
19	0.26	0.39	0.43	0.22	0.22	0.21	0.22	0.20	0.22															
20	0.26	0.44	0.42	0.23	0.22	0.24	0.23	0.20	0.22	0.22	0.22													
21	0.35	0.42	0.42	0.42	0.44	0.42	0.44	0.43	0.28	0.23	0.23	0.24	0.24											
22	0.34	0.46	0.47	0.46	0.45	0.25	0.24	0.25	0.22	0.24														
23	0.25	0.39	0.36	0.43	S	0.16	0.18	0.15	0.20	0.17	0.21													
24	0.35	0.48	0.48	0.46	0.46	0.39	0.46	0.23	0.23	0.20	0.23	0.23												
25	0.29	0.38	0.42	0.42	0.40	0.42	0.20	0.20	0.18	0.19	0.21	0.22												

MEAN	0.31
S.D.	0.032
N	20

PUP STATUS CODES: S-STILLBORN

PR.NO.60R0375/88R002: REPRODUCTIVE TOX. STUDY TO DETECT EFFECTS
OF MIXED ANTI-ANDROGENIC SUBSTANCES IN RATS; ORAL ADM. (GAVAGE)
INDIVIDUAL PUP DATA -- AG DIST DAY 1 to BODY WEIGHT RATIO (AG Index)

TEST GROUP 1 (ADI-MIX)

FEMALE#	MEAN	PUP# 1	2	3	4	5	6	7	8	9	10	11	12	13	14	15	16	17	18	19	20	21	22	23
26	0.34	0.43	0.43	0.46	0.42	0.46	0.23	0.24	0.22	0.25	0.23													
27	0.29	0.42	0.41	0.40	0.41	0.19	0.22	0.19	0.19	0.22	0.21													
28	0.27	0.35	0.37	0.40	0.36	0.17	0.17	0.16	0.18															
29	0.36	0.41	0.37	0.40	0.38	0.39	0.39	0.42	0.41	0.40	0.20	0.18												
30	0.40	0.50	0.48	0.48	0.52	0.51	0.49	0.52	0.24	0.25	0.28	0.25	0.27											
31	0.25	0.45	0.44	0.19	0.21	0.21	0.22	0.22	0.20	0.22	0.21	0.24	0.23											
32	0.24	0.39	0.36	0.18	0.19	0.21	0.19	0.20	0.19															
33	0.29	0.45	0.43	0.41	0.40	0.22	0.21	0.22	0.21	0.20	0.19													
34	0.31	0.43	0.37	0.41	0.47	0.42	0.17	0.22	0.21	0.21	0.23													
35	0.27	0.38	0.39	0.40	0.18	0.18	0.19	0.20	0.20															
36	0.28	0.34	0.37	0.39	0.40	0.19	0.20	0.22	0.23	0.22	0.21													
37	0.33	0.49	0.46	0.43	0.44	0.44	0.22	0.22	0.21	0.22	0.21													
43	0.35	0.48	0.45	0.52	0.48	0.44	0.45	0.21	0.24	0.22	0.23	0.23	0.23											
44	0.20	S	0.21	0.20	0.18	0.21	0.21	0.18	0.20	0.20	0.19													
45	0.32	0.42	0.41	0.45	0.41	0.41	0.19	0.18	0.20	0.19														
46	0.38	0.52	0.51	0.47	0.51	0.52	0.50	0.55	0.21	0.23	0.24	0.22	0.26	0.24										
47	0.33	0.44	0.41	0.45	0.41	0.21	0.21	0.20																
48	0.20	0.20	0.22	0.19	0.22	0.20	0.20	0.18	0.19	0.19														
49	0.26	0.40	0.36	0.40	0.38	0.19	0.21	0.18	0.21	0.22	0.20	0.19	0.19											
50	0.35	0.43	0.48	0.39	0.44	0.41	0.21	0.20	0.21															

MEAN	0.30
S.D.	0.056
N	20

PUP STATUS CODES: S-STILLBORN

PR.NO.60R0375/88R002: REPRODUCTIVE TOX. STUDY TO DETECT EFFECTS
OF MIXED ANTI-ANDROGENIC SUBSTANCES IN RATS; ORAL ADM. (GAVAGE)
INDIVIDUAL PUP DATA -- AG DIST DAY 1 to BODY WEIGHT RATIO (AG Index)

TEST GROUP 2 (NOAEL-MIX)

FEMALE#	MEAN	PUP# 1	2	3	4	5	6	7	8	9	10	11	12	13	14	15	16	17	18	19	20	21	22	23
51	0.31	0.45	0.50	0.47	0.25	0.20	0.24	0.26	0.25	0.23	0.21													
52	0.31	0.40	0.44	0.41	0.43	0.42	0.22	0.21	0.20	0.22	0.21	0.20												
53	0.32	0.36	0.37	0.39	0.38	0.43	S	0.21	0.17	0.21														
54	0.33	0.42	0.42	0.40	0.40	0.44	0.39	0.21	0.20	0.22	0.23													
55	0.35	0.46	0.47	0.40	0.40	0.45	0.41	0.47	0.21	0.22	0.24	0.21	0.22											
56	0.42	0.67	0.58	D	D	D	0.26	0.35	0.34	0.32	D	D	D	Z	S									
57	0.32	0.44	0.41	0.41	0.38	0.40	0.40	Z	0.22	0.23	0.22	0.21	0.23											
58	0.33	0.40	0.40	0.38	0.38	0.39	0.22	0.21	0.24															
59	0.28	0.40	0.37	0.40	0.39	0.18	0.19	0.20	0.19	0.21														
60	0.30	0.43	0.42	0.44	0.49	0.24	0.22	0.26	0.22	0.23	0.24	0.22	0.22	0.25										
61	0.28	0.34	0.37	0.37	0.38	0.37	0.18	0.19	0.19	0.18														
62	0.35	0.39	0.38	0.37	0.34	0.48	0.41	0.20	0.20															
68	0.28	0.39	0.41	0.39	0.37	0.22	0.21	0.20	0.23	0.22	0.20													
69	0.42	0.48	0.41	0.47	0.41	0.40	0.46	0.54	0.47	0.17														
70	0.32	0.40	0.39	0.43	0.20	0.23	0.24	Z																
71	0.28	0.42	0.39	0.42	0.21	0.23	0.23	0.21	0.21	0.22														
72	0.36	0.44	0.44	0.41	0.43	0.46	0.41	0.41	0.46	0.20	0.22	0.22	0.20											
73	0.31	0.45	0.50	0.45	0.42	0.24	0.21	0.22	0.23	0.21	0.21													
74	0.30	0.45	0.48	0.45	0.46	0.21	0.19	0.23	0.21	0.21	0.22	0.21												
75	0.39	0.48	0.43	0.46	0.43	0.50	0.49	0.22	0.23	0.29														

MEAN	0.33
S.D.	0.043
N	20

PUP STATUS CODES: S-STILLBORN D -DIED Z -CANNIBALIZED

PR.NO.60R0375/88R002: REPRODUCTIVE TOX. STUDY TO DETECT EFFECTS
OF MIXED ANTI-ANDROGENIC SUBSTANCES IN RATS; ORAL ADM. (GAVAGE)
INDIVIDUAL PUP DATA -- AG DIST DAY 1 to BODY WEIGHT RATIO (AG Index)

TEST GROUP 3 (LOAEL-MIX)

FEMALE#	MEAN	PUP# 1	2	3	4	5	6	7	8	9	10	11	12	13	14	15	16	17	18	19
76	UDS																			
77	0.31	0.38	0.41	0.42	0.48	0.21	0.19	0.20	0.21											
78	0.39	0.38	0.48	0.37	0.43	0.39	0.42	0.41	0.22	0.40	S									
80	0.32	0.40	0.38	0.36	S	S	0.22	0.26	S	S										
82	0.31	0.38	0.38	0.40	0.44	0.38	0.36	0.23	0.21	0.24	0.22	0.22								
83	0.30	0.42	0.43	0.41	0.38	S	0.21	0.23	0.20	0.21	0.23									
84	0.32	0.35	0.32	0.29	0.33	0.37	0.37	0.18												
85	0.24	0.37	Z	S	S	0.25	0.23	0.19	0.23	0.20	Z									
86	0.29	0.41	0.37	0.36	0.38	0.35	0.21	0.21	0.22	0.22	0.21	0.22								
87	0.30	0.39	0.37	0.38	0.38	0.38	0.26	0.27	0.21	0.23	0.25	0.22	0.25							
93	0.34	0.34	0.39	0.37	0.42	0.42	0.23	0.23												
94	0.32	0.40	0.41	0.35	0.39	0.39	0.38	0.18	0.22	0.25	0.22									
95	0.24	0.36	0.32	0.35	0.29	0.15	0.17	0.21	0.18	0.21	0.21									
96	0.32	0.42	0.33	0.38	0.37	0.41	0.26	0.21	0.22	0.25										
97	0.35	0.37	0.43	0.36	0.36	0.37	0.37	0.38	0.24	0.24										
98	0.32	0.42	0.45	0.39	0.48	0.24	0.24	0.24	0.23	0.23	0.26	S								
99	0.33	0.35	0.35	0.37	0.57	0.41	S	0.21	0.26	0.23	0.25									
100	0.28	0.33	0.38	0.39	0.37	0.20	0.22	0.21	0.21	0.19										

MEAN	0.31
S.D.	0.035
N	17

PUP STATUS CODES: S-STILLBORN Z -CANNIBALIZED

UDS=UNABLE TO DELIVER, SAC'D

PR.NO.60R0375/88R002: REPRODUCTIVE TOX. STUDY TO DETECT EFFECTS
OF MIXED ANTI-ANDROGENIC SUBSTANCES IN RATS; ORAL ADM. (GAVAGE)
INDIVIDUAL PUP DATA -- AG DIST DAY 1 to BODY WEIGHT RATIO (AG Index)

TEST GROUP 4(0.00025 MG/KG BW/D)

FEMALE#	MEAN	PUP# 1	2	3	4	5	6	7	8	9	10	11	12	13	14	15	16	17	18	19	20	21	22	23
101	0.30	0.35	0.33	0.34	0.37	0.35	0.37	0.19	0.19	0.20														
102	0.37	0.42	0.43	0.52	0.45	0.46	0.41	0.45	0.19	0.20	0.18													
103	0.33	0.48	0.40	0.49	0.46	0.25	0.25	0.23	0.25	0.25	0.23													
104	0.36	0.41	0.46	0.41	0.45	0.41	0.41	0.43	0.43	0.18	0.22	0.19												
105	0.31	0.36	0.36	0.35	0.35	0.38	0.18	0.21																
106	0.33	0.38	0.36	0.37	0.41	0.42	0.38	0.22	0.19	0.23														
107	0.30	0.39	0.42	0.42	0.41	0.20	0.23	0.23	0.22	0.21	0.23													
108	0.29	0.42	0.45	0.43	0.23	0.22	0.21	0.23	0.23	0.22														
109	0.30	0.42	0.38	0.42	0.39	0.21	0.22	0.20	0.22	0.21														
111	0.28	0.38	0.36	0.38	0.33	0.33	0.35	0.18	0.17	0.14	0.17													
117	0.26	0.41	0.42	0.41	0.19	0.19	0.19	0.18	0.21	0.19														
118	0.32	0.43	0.40	0.41	0.42	0.43	0.42	0.22	0.22	0.23	0.23	0.22	0.24											
119	0.28	0.40	0.40	0.42	S	0.20	0.23	0.22	0.18	0.19														
120	0.41	0.50	0.49	0.57	S	0.25	0.24	Z	S	S	S													
121	0.22	0.44	0.22	0.21	0.19	0.20	0.19	0.20	0.22	0.21	0.22	0.20	0.19											
123	0.31	0.43	0.45	0.40	0.46	0.19	0.22	0.18	0.16															
124	0.26	0.40	0.45	0.43	0.20	0.21	0.17	0.19	0.19	0.20	0.22	0.21												
125	0.34	0.41	0.41	0.43	0.42	0.44	0.39	0.36	0.22	0.18	0.23	0.20												

MEAN	0.31
S.D.	0.044
N	18

PUP STATUS CODES: S-STILLBORN Z -CANNIBALIZED

PR.NO.60R0375/88R002: REPRODUCTIVE TOX. STUDY TO DETECT EFFECTS
OF MIXED ANTI-ANDROGENIC SUBSTANCES IN RATS; ORAL ADM. (GAVAGE)
INDIVIDUAL PUP BODY WEIGHTS -- GRAMS

TEST GROUP 0 (0 MG/KG BW/D) LACTATION DAY 1

FEMALE#	MEAN	PUP# 1	2	3	4	5	6	7	8	9	10	11	12	13	14	15	16	17	18	19
1	7.5	6.8	7.8	7.8	7.9	7.9	7.2	7.3	7.2											
2	7.2	7.6	6.8	7.6	7.7	6.7	6.9	6.7	7.5	7.1	6.9									
3	7.1	7.0	6.7	7.2	7.7	7.4	7.4	7.3	7.4	5.9										
4	6.7	7.0	7.1	7.1	6.0	6.9	7.0	6.3	6.7	6.9	6.4	6.6								
5	6.8	7.0	6.5	6.3	7.0	7.4	7.4	6.2	6.4	7.4	6.2	6.5								
6	7.2	7.4	7.3	7.1	7.3	7.2	7.5	6.8	7.2	7.0										
7	7.2	7.5	6.7	7.5	7.7	7.0	7.1	7.3	7.0											
8	7.1	7.5	7.6	6.7	6.7	7.2	6.8	7.2	7.0	6.8										
9	6.9	7.1	7.0	7.1	7.0	6.6	6.8	6.5												
10	6.8	7.1	6.1	7.4	6.9	7.0	6.6	6.7	7.1	6.4	6.6									
11	6.5	6.5	6.8	5.4	7.0	6.6	6.7	6.6	6.8	6.0	6.6	7.1	6.3							
17	6.7	7.4	7.0	7.1	6.4	5.8	7.0	6.4	6.5											
18	7.4	7.3	7.4	7.6	7.6	7.1	6.6	7.3	7.2	8.0	7.6									
19	6.6	7.4	6.9	6.4	6.3	6.3	6.3	6.4	6.4											
20	6.9	7.3	7.3	6.4	6.5	6.7	6.4	7.5	7.2	6.8	6.8									
21	6.8	7.4	7.2	7.1	7.1	7.1	7.0	6.9	5.8	6.5	6.9	6.7	6.2							
22	6.3	6.5	6.2	6.5	6.4	6.3	6.3	5.9	6.5	5.9										
23	7.6	7.6	8.4	7.0	S	7.5	8.2	7.8	7.0	8.0	7.2									
24	6.8	6.3	7.5	6.5	7.0	7.7	7.4	6.6	6.4	6.4	6.6	6.5								
25	6.7	7.3	6.9	7.2	6.8	6.4	6.0	7.1	6.5	6.8	6.8	6.0								
MEAN	6.9																			
S.D.	0.3																			
N	20																			

PUP STATUS CODES: S-STILLBORN

PR.NO.60R0375/88R002: REPRODUCTIVE TOX. STUDY TO DETECT EFFECTS
OF MIXED ANTI-ANDROGENIC SUBSTANCES IN RATS; ORAL ADM. (GAVAGE)
INDIVIDUAL PUP BODY WEIGHTS -- GRAMS

TEST GROUP 1 (ADI-MIX) LACTATION DAY 1

FEMALE#	MEAN	PUP# 1	2	3	4	5	6	7	8	9	10	11	12	13	14	15	16	17	18	19
26	6.7	7.0	7.2	6.8	6.9	6.5	6.5	5.8	6.7	6.5	7.0									
27	7.3	7.7	7.6	7.7	7.8	7.2	6.8	7.2	7.3	6.8	7.1									
28	7.6	8.2	8.1	7.7	7.7	7.5	6.9	7.5	7.1											
29	7.5	7.3	7.8	7.5	7.8	8.0	7.6	7.1	7.1	7.7	7.4	7.6								
30	6.0	6.2	6.4	6.4	6.0	5.9	6.1	6.0	5.9	6.0	5.4	5.7	5.6							
31	6.7	6.6	6.8	7.2	6.7	6.7	6.8	6.8	6.9	6.8	6.6	6.3	6.5							
32	7.5	7.7	8.3	7.6	7.7	7.5	7.4	6.9	7.0											
33	7.1	7.4	7.0	7.5	7.5	6.8	6.6	6.8	7.1	7.1	7.5									
34	6.6	7.0	6.7	6.8	6.0	7.2	6.9	6.0	6.2	6.6	6.2									
35	7.7	7.9	7.7	7.8	7.7	7.6	7.8	7.5	7.4											
36	7.2	8.3	7.8	7.6	7.3	7.3	7.0	6.7	6.4	6.9	6.7									
37	5.3	5.1	5.4	5.3	5.7	5.9	5.5	5.1	4.8	5.4	5.3									
43	6.4	7.1	6.6	6.1	5.8	6.8	5.8	6.6	6.3	6.3	6.4	6.4	6.4							
44	7.4	S	7.3	7.5	7.7	7.2	7.0	7.1	7.6	7.6	7.3									
45	6.7	6.9	7.1	6.6	6.8	6.8	6.7	6.5	6.1	6.4										
46	5.5	5.8	5.9	5.5	5.9	5.4	5.8	5.5	5.7	5.3	5.4	5.4	4.6	5.5						
47	7.1	7.0	7.4	6.7	7.6	7.7	6.6	6.9												
48	6.0	5.9	5.8	6.4	5.8	6.5	6.1	6.0	5.7	5.7										
49	6.9	7.5	7.7	7.0	7.4	6.8	6.1	7.1	6.2	6.9	6.9	6.2	6.7							
50	7.5	8.2	6.9	7.6	7.9	7.3	7.2	7.5	7.0											

MEAN	6.8
S.D.	0.7
N	20

PUP STATUS CODES: S-STILLBORN

PR.NO.60R0375/88R002: REPRODUCTIVE TOX. STUDY TO DETECT EFFECTS
OF MIXED ANTI-ANDROGENIC SUBSTANCES IN RATS; ORAL ADM. (GAVAGE)
INDIVIDUAL PUP BODY WEIGHTS -- GRAMS

TEST GROUP 2 (NOAEL-MIX) LACTATION DAY 1

FEMALE#	MEAN	PUP# 1	2	3	4	5	6	7	8	9	10	11	12	13	14	15	16	17	18	19
51	6.2	6.6	6.4	6.4	5.7	6.5	5.9	5.8	6.1	6.6	6.1									
52	7.1	7.5	7.1	7.4	7.2	7.3	6.5	7.1	7.0	6.9	7.1	6.9								
53	7.6	8.4	7.8	7.5	7.8	7.6	S	7.3	7.6	7.1										
54	7.4	7.9	7.6	7.5	7.8	6.8	8.0	7.7	7.6	6.7	6.2									
55	6.3	6.5	6.4	6.5	6.5	6.7	6.1	6.4	6.1	5.9	6.3	6.1	5.8							
56	5.0	4.8	5.3	D	D	D	6.1	4.6	4.4	4.7	D	D	D	Z	S					
57	6.9	7.1	7.0	6.9	7.3	7.5	7.3	Z	6.7	6.0	6.8	6.8	6.1							
58	8.3	8.0	8.5	8.7	8.9	8.7	8.0	8.1	7.1											
59	7.4	7.3	7.5	7.3	7.5	7.6	7.5	7.4	7.3	7.3										
60	6.4	6.5	6.7	6.6	6.1	6.2	6.4	5.8	6.4	6.5	6.3	6.7	6.4	6.0						
61	7.3	7.7	7.6	7.5	7.6	7.3	7.4	6.9	6.9	7.2										
62	7.6	7.7	7.6	8.1	8.8	6.5	7.4	8.0	6.9											
68	7.4	7.4	7.4	7.4	7.6	7.3	7.0	7.5	7.1	7.3	8.0									
69	6.9	7.3	7.4	7.0	7.3	7.5	7.2	5.0	6.8	7.0										
70	8.0	8.8	8.5	8.1	7.5	7.7	7.5	Z												
71	7.1	7.2	7.6	6.9	7.0	7.1	7.0	7.2	7.5	6.8										
72	7.1	7.3	6.8	7.1	6.9	7.6	7.3	7.5	7.0	7.0	6.8	6.7	7.4							
73	6.9	7.1	6.6	7.4	7.1	7.0	7.3	6.7	6.5	7.3	6.1									
74	6.8	7.3	6.6	7.3	7.2	6.6	6.7	6.6	7.1	6.6	6.0	6.8								
75	6.7	7.3	6.9	7.6	7.4	5.8	7.2	6.7	6.6	4.5										
MEAN	7.0																			
S.D.	0.7																			
N	20																			

PUP STATUS CODES: S-STILLBORN D -DIED Z -CANNIBALIZED

PR.NO.60R0375/88R002: REPRODUCTIVE TOX. STUDY TO DETECT EFFECTS
OF MIXED ANTI-ANDROGENIC SUBSTANCES IN RATS; ORAL ADM. (GAVAGE)
INDIVIDUAL PUP BODY WEIGHTS -- GRAMS

TEST GROUP 3 (LOAEL-MIX) LACTATION DAY 1

FEMALE#	MEAN	PUP# 1	2	3	4	5	6	7	8	9	10	11	12	13	14	15	16	17	18	19
77	7.2	7.7	7.4	6.7	6.0	7.3	7.9	6.9	7.8											
78	7.5	8.0	7.3	8.2	7.0	8.9	7.1	7.9	7.7	5.0	S									
80	6.9	6.5	7.2	7.6	S	S	6.8	6.2	S	S										
82	7.2	7.7	7.7	7.0	6.6	7.8	7.7	7.0	7.6	6.8	6.8	6.9								
83	8.0	8.1	8.2	8.2	7.8	S	8.1	8.4	7.5	8.1	7.9									
84	8.0	7.8	8.1	8.9	8.3	7.0	7.0	8.8												
85	7.4	8.1	Z	S	S	6.1	7.1	8.0	7.1	7.9	Z									
86	7.6	7.5	7.9	8.0	7.8	7.7	7.5	7.5	7.7	7.3	7.8	7.2								
87	7.8	8.4	7.6	7.9	8.0	7.8	7.0	7.5	8.0	7.8	8.0	8.0	7.3							
93	6.9	8.2	7.1	6.8	6.7	6.2	6.4	6.9												
94	8.1	8.4	8.6	8.5	7.7	8.3	7.8	8.2	7.9	8.1	7.9									
95	7.5	7.7	7.9	7.1	7.6	8.0	7.8	7.3	7.3	6.8	7.3									
96	8.2	8.6	9.0	9.2	8.6	7.8	7.7	8.1	7.9	6.7										
97	8.0	8.7	7.5	8.4	8.3	8.1	8.3	7.3	7.6	7.6										
98	6.4	6.6	6.7	7.0	5.6	6.3	6.3	6.2	6.6	6.9	5.8	S								
99	7.6	8.2	8.5	8.1	5.3	7.8	S	8.0	7.8	7.3	7.3									
100	7.7	8.5	7.9	7.6	7.6	7.6	7.9	7.5	7.2	7.7										

MEAN	7.5
S.D.	0.5
N	17

PUP STATUS CODES: S-STILLBORN Z -CANNIBALIZED

PR.NO.60R0375/88R002: REPRODUCTIVE TOX. STUDY TO DETECT EFFECTS
OF MIXED ANTI-ANDROGENIC SUBSTANCES IN RATS; ORAL ADM. (GAVAGE)
INDIVIDUAL PUP BODY WEIGHTS -- GRAMS

TEST GROUP 4(0.00025 MG/KG BW/D) LACTATION DAY 1

FEMALE#	MEAN	PUP# 1	2	3	4	5	6	7	8	9	10	11	12	13	14	15	16	17	18	19
101	8.9	8.9	9.6	9.0	8.6	8.9	9.0	8.8	8.5	8.4										
102	6.9	7.1	7.0	6.4	6.7	6.5	7.4	7.1	6.9	6.9	7.4									
103	6.4	6.0	7.0	6.1	6.8	6.3	6.4	6.4	6.5	6.3	6.6									
104	7.0	7.4	6.7	7.3	6.6	7.4	7.3	7.0	7.0	7.1	6.9	6.4								
105	7.9	8.4	8.1	8.3	7.9	8.0	7.8	7.1												
106	8.1	8.2	8.5	8.3	8.0	7.6	8.7	7.9	8.3	7.4										
107	6.9	7.5	7.3	7.4	7.0	7.0	6.5	6.1	6.9	7.0	6.6									
108	6.8	7.4	6.6	7.0	6.9	6.7	7.1	6.4	7.0	6.3										
109	7.1	7.1	7.4	7.1	7.2	7.0	6.9	7.0	6.7	7.1										
111	7.7	7.8	8.3	7.9	7.5	7.8	8.1	7.3	7.2	7.8	7.5									
117	7.1	7.4	7.2	7.9	6.7	7.0	6.9	7.2	7.1	6.9										
118	6.7	6.7	7.2	7.3	6.9	7.0	6.6	6.8	6.3	6.0	6.5	6.8	6.3							
119	9.3	9.6	9.6	9.1	S	9.8	8.8	9.2	9.0	9.5										
120	6.3	6.6	6.9	5.3	S	6.1	6.6	Z	S	S	S									
121	6.1	6.6	6.0	5.8	6.4	6.0	6.2	6.1	6.0	6.1	5.9	6.0	6.2							
123	7.0	7.0	6.6	7.5	7.0	7.4	6.9	6.5	7.4											
124	7.0	8.1	6.7	7.0	7.1	7.2	6.9	6.7	7.0	7.1	6.7	7.0								
125	7.1	7.5	7.6	6.9	7.1	6.6	7.6	7.8	6.8	7.1	6.4	6.6								
MEAN	7.3																			
S.D.	0.8																			
N	18																			

PUP STATUS CODES: S-STILLBORN Z -CANNIBALIZED

PR.NO.60R0375/88R002: REPRODUCTIVE TOX. STUDY TO DETECT EFFECTS
OF MIXED ANTI-ANDROGENIC SUBSTANCES IN RATS; ORAL ADM. (GAVAGE)
INDIVIDUAL PUP BODY WEIGHTS -- GRAMS

TEST GROUP 0 (0 MG/KG BW/D) LACTATION DAY 4

FEMALE#	MEAN	PUP# 1	2	3	4	5	6	7	8	9	10	11	12	13	14	15	16	17	18	19
1	11.8	10.8	12.4	12.0	12.4	12.1	11.4	11.5	11.6											
2	11.2	11.8	10.9	11.7	12.0	10.5	11.0	10.4	11.7	11.1	11.1									
3	11.3	10.7	10.7	11.7	12.0	11.7	11.9	11.4	11.9	9.5										
4	11.2	11.3	11.4	11.5	10.4	11.5	11.3	11.1	11.3	11.6	11.1	11.1								
5	10.6	10.7	10.2	10.3	10.7	11.5	11.4	9.8	10.3	11.7	9.9	9.9								
6	11.3	11.4	11.5	10.9	11.4	11.6	11.6	10.9	11.0	11.2										
7	12.3	12.6	10.7	13.3	12.5	12.6	12.3	12.6	12.0											
8	11.2	11.7	12.0	10.0	10.9	11.7	11.0	11.9	11.4	10.1										
9	11.0	11.3	11.2	11.0	11.3	10.5	11.0	10.5												
10	10.3	11.0	9.6	11.2	10.5	10.7	9.8	10.0	10.7	9.8	10.1									
11	10.4	10.4	10.9	8.6	10.9	10.4	10.9	10.8	10.4	9.7	10.6	10.9	10.0							
17	10.3	11.1	10.5	11.1	9.6	8.6	10.8	10.3	10.2											
18	11.6	11.4	11.4	12.0	12.2	11.5	10.8	11.4	11.4	12.5	11.8									
19	9.9	10.6	10.5	9.4	10.0	10.0	9.6	9.5	9.5											
20	10.6	11.2	10.8	9.9	10.1	10.4	10.1	11.2	11.1	10.6	10.6									
21	11.3	12.3	11.4	11.6	11.8	11.3	11.3	11.5	9.4	10.8	11.7	11.6	10.4							
22	10.2	10.8	10.2	10.5	10.4	10.1	10.3	9.5	10.7	9.5										
23	11.6	11.6	12.3	11.0	S	11.1	12.3	12.0	11.0	12.0	11.5									
24	10.1	9.4	10.8	9.6	10.4	11.1	10.6	10.1	9.5	9.7	10.2	9.9								
25	9.9	10.5	9.7	10.7	10.3	9.8	8.9	10.4	9.6	10.1	10.2	8.9								
MEAN	10.9																			
S.D.	0.7																			
N	20																			

PUP STATUS CODES: S-STILLBORN

PR.NO.60R0375/88R002: REPRODUCTIVE TOX. STUDY TO DETECT EFFECTS
OF MIXED ANTI-ANDROGENIC SUBSTANCES IN RATS; ORAL ADM. (GAVAGE)
INDIVIDUAL PUP BODY WEIGHTS -- GRAMS

TEST GROUP 1 (ADI-MIX) LACTATION DAY 4

FEMALE#	MEAN	PUP# 1	2	3	4	5	6	7	8	9	10	11	12	13	14	15	16	17	18	19
26	10.5	10.8	11.1	10.6	10.9	10.4	10.0	9.6	10.9	10.2	10.7									
27	11.3	11.3	12.5	11.6	12.0	11.5	10.9	11.2	11.2	10.5	10.3									
28	12.1	12.9	13.0	12.2	12.0	12.1	10.9	12.2	11.6											
29	11.9	11.8	12.3	11.9	12.2	12.2	12.1	11.5	11.4	11.9	11.7	11.8								
30	9.3	9.5	9.7	10.1	9.3	9.4	9.6	9.1	9.2	9.5	8.6	9.4	8.6							
31	10.6	10.5	11.1	11.5	10.3	10.8	10.5	11.0	11.1	11.1	10.4	10.0	9.2							
32	12.5	12.5	13.3	12.8	12.9	12.5	12.4	11.7	11.9											
33	11.4	11.5	10.8	11.7	11.5	11.2	10.9	11.2	11.8	11.3	11.8									
34	9.5	9.7	9.3	9.6	8.9	10.2	9.8	9.0	9.3	9.6	9.3									
35	12.9	13.2	12.8	13.3	13.0	12.8	13.1	12.7	12.7											
36	11.8	13.3	12.1	12.4	11.7	11.8	11.5	11.4	10.7	11.5	11.3									
37	7.6	7.1	7.8	7.7	7.7	8.3	8.0	7.5	7.0	7.8	7.5									
43	9.8	10.8	10.0	9.7	8.7	10.3	9.1	9.8	9.4	9.7	9.7	10.1	9.8							
44	11.9	S	11.9	11.9	12.4	11.7	11.2	11.6	12.6	12.1	12.1									
45	10.1	10.4	10.2	10.0	10.2	10.3	10.7	9.8	9.5	9.9										
46	8.9	8.9	9.6	8.7	9.2	9.0	9.6	8.7	9.1	8.5	9.2	9.0	7.7	8.7						
47	11.5	11.1	11.8	10.6	12.8	12.5	10.6	11.4												
48	9.5	9.6	9.1	9.9	9.4	10.3	9.5	9.4	9.1	9.1										
49	10.7	11.3	11.5	11.1	11.2	10.8	9.9	11.0	10.1	10.5	10.5	9.7	10.7							
50	11.1	12.1	10.3	11.2	11.7	10.9	10.7	11.1	10.5											

MEAN	10.7
S.D.	1.4
N	20

PUP STATUS CODES: S-STILLBORN

PR.NO.60R0375/88R002: REPRODUCTIVE TOX. STUDY TO DETECT EFFECTS
OF MIXED ANTI-ANDROGENIC SUBSTANCES IN RATS; ORAL ADM. (GAVAGE)
INDIVIDUAL PUP BODY WEIGHTS -- GRAMS

TEST GROUP 2 (NOAEL-MIX) LACTATION DAY 4

FEMALE#	MEAN	PUP# 1	2	3	4	5	6	7	8	9	10	11	12	13	14	15	16	17	18	19
51	8.9	9.3	9.2	9.1	8.3	9.3	8.7	8.5	8.9	9.1	8.7									
52	11.1	11.8	11.3	11.3	11.1	11.3	10.3	11.2	10.9	10.7	11.2	10.8								
53	11.9	12.9	12.1	11.8	12.4	11.8	S	11.6	11.6	11.4										
54	12.0	12.6	12.5	12.4	12.9	11.3	12.7	12.1	12.0	11.1	10.5									
55	9.6	9.7	9.4	9.8	10.1	10.1	9.3	9.7	9.7	9.3	9.8	9.2	8.9							
56	6.6	Z	7.6	D	D	D	Z	5.5	D	Z	D	D	D	Z	S					
57	10.0	10.5	10.3	9.9	10.4	10.7	10.1	Z	9.6	8.8	9.9	10.3	9.2							
58	12.7	12.3	13.2	13.3	13.3	12.9	12.5	12.3	11.7											
59	11.9	11.5	12.1	11.7	11.9	12.1	12.1	11.9	12.2	11.5										
60	9.4	9.8	9.8	9.8	9.4	9.3	9.2	8.9	9.4	9.5	9.3	9.8	9.3	8.9						
61	11.5	11.8	11.6	12.0	11.7	11.6	11.9	10.9	10.8	11.3										
62	11.4	11.4	11.1	12.3	12.5	10.1	11.1	11.8	10.6											
68	11.9	11.9	11.9	11.8	12.4	12.0	11.4	12.0	11.5	11.9	12.4									
69	10.6	11.2	11.7	10.9	11.0	11.3	10.8	7.7	10.4	10.8										
70	12.3	13.4	12.6	12.5	11.5	12.1	11.7	Z												
71	11.8	11.9	12.3	11.4	11.6	12.2	11.8	11.7	12.3	11.3										
72	11.2	11.6	10.6	11.3	11.0	11.9	11.5	11.9	11.2	11.1	10.7	10.4	11.7							
73	10.5	10.6	10.3	10.6	10.8	10.5	11.0	9.9	10.1	10.9	9.9									
74	10.0	10.5	9.9	10.5	10.4	9.8	9.5	9.8	10.5	9.3	9.5	10.0								
75	10.8	11.8	11.4	12.4	12.1	9.2	11.7	10.9	10.6	7.5										
MEAN	10.8																			
S.D.	1.4																			
N	20																			

PUP STATUS CODES: S-STILLBORN D -DIED Z -CANNIBALIZED

PR.NO.60R0375/88R002: REPRODUCTIVE TOX. STUDY TO DETECT EFFECTS
OF MIXED ANTI-ANDROGENIC SUBSTANCES IN RATS; ORAL ADM. (GAVAGE)
INDIVIDUAL PUP BODY WEIGHTS -- GRAMS

TEST GROUP 3 (LOAEL-MIX) LACTATION DAY 4

FEMALE#	MEAN	PUP# 1	2	3	4	5	6	7	8	9	10	11	12	13	14	15	16	17	18	19
77	11.3	11.9	11.6	10.6	9.3	11.8	12.1	10.9	11.9											
78	11.5	12.0	11.7	12.7	11.0	13.0	11.0	12.1	11.5	8.7	S									
80	11.3	11.0	11.4	12.5	S	S	11.2	10.6	S	S										
82	10.3	10.6	10.8	10.0	9.2	11.2	10.7	10.4	10.9	9.6	9.8	9.9								
83	12.6	12.8	13.0	12.9	12.5	S	12.6	12.6	11.5	12.6	12.7									
84	12.0	11.9	11.8	13.4	12.7	10.4	10.8	13.3												
85	12.9	13.9	Z	S	S	10.9	12.7	13.4	12.6	13.6	Z									
86	11.2	11.1	11.8	11.4	11.5	11.3	10.9	11.0	11.2	10.7	11.2	10.7								
87	11.3	11.7	11.9	11.7	11.3	11.3	10.5	11.4	11.5	11.2	11.0	11.5	10.7							
93	11.3	12.6	11.3	11.3	11.1	10.2	10.8	11.8												
94	12.6	13.3	13.3	13.0	12.1	12.6	12.0	12.9	12.1	12.4	12.4									
95	11.1	11.3	11.1	10.9	11.1	11.3	11.5	11.3	11.2	10.4	10.7									
96	12.3	12.8	13.1	13.2	12.4	11.9	11.9	12.3	11.9	10.8										
97	12.4	12.5	11.8	12.7	13.1	12.6	12.7	11.9	12.0	12.3										
98	9.2	9.1	9.3	9.8	8.3	9.0	9.3	9.0	9.3	9.8	9.1	S								
99	11.1	11.8	11.7	11.8	8.6	11.2	S	11.6	11.6	10.9	10.9									
100	12.0	12.9	12.3	11.9	11.6	11.8	12.2	11.5	11.5	11.9										
MEAN	11.5																			
S.D.	0.9																			
N	17																			

PUP STATUS CODES: S-STILLBORN Z -CANNIBALIZED

PR.NO.60R0375/88R002: REPRODUCTIVE TOX. STUDY TO DETECT EFFECTS
OF MIXED ANTI-ANDROGENIC SUBSTANCES IN RATS; ORAL ADM. (GAVAGE)
INDIVIDUAL PUP BODY WEIGHTS -- GRAMS

TEST GROUP 4(0.00025 MG/KG BW/D) LACTATION DAY 4

FEMALE#	MEAN	PUP# 1	2	3	4	5	6	7	8	9	10	11	12	13	14	15	16	17	18	19
101	13.8	13.8	14.6	14.0	12.9	13.6	14.3	13.8	13.4	13.6										
102	9.9	10.1	10.0	9.4	9.3	9.2	10.8	10.1	10.1	10.1	10.3									
103	9.8	9.0	10.1	9.3	10.2	9.8	9.8	9.8	9.8	9.8	10.1									
104	11.2	11.6	11.0	11.4	10.5	11.5	11.8	10.9	11.0	11.4	11.2	10.4								
105	12.5	13.2	12.5	13.2	12.5	12.6	12.1	11.7												
106	13.2	13.5	13.8	13.5	13.2	12.4	14.3	13.2	13.2	11.8										
107	10.6	11.1	11.2	11.0	10.6	10.6	10.2	9.1	10.6	10.8	10.4									
108	10.5	11.4	10.1	10.8	10.6	10.5	10.9	9.8	10.8	9.9										
109	11.1	11.6	11.2	11.4	11.9	10.5	10.7	11.8	9.3	11.5										
111	12.0	12.0	12.9	12.3	11.8	12.1	12.5	11.7	11.2	12.0	11.5									
117	11.3	11.4	11.4	12.4	11.1	10.8	11.2	11.4	11.2	11.1										
118	10.7	10.5	11.2	11.2	10.9	10.9	10.5	10.9	10.2	9.7	10.6	11.1	10.3							
119	14.3	14.5	14.9	14.0	S	15.0	13.4	14.0	14.1	14.3										
120	12.0	12.4	13.2	10.1	S	11.6	12.5	Z	S	S	S									
121	9.5	10.0	9.5	9.2	9.8	9.5	9.8	9.7	9.2	9.1	9.3	9.3	9.7							
123	11.2	11.0	11.0	11.4	11.0	11.5	11.3	10.5	11.7											
124	10.3	11.4	10.3	10.4	10.0	9.8	9.4	10.2	10.4	10.5	10.3	10.4								
125	10.1	10.7	11.0	9.7	10.2	9.6	10.5	10.8	9.7	10.1	9.4	9.3								
MEAN	11.3																			
S.D.	1.4																			
N	18																			

PUP STATUS CODES: S-STILLBORN Z -CANNIBALIZED

PR.NO.60R0375/88R002: REPRODUCTIVE TOX. STUDY TO DETECT EFFECTS
OF MIXED ANTI-ANDROGENIC SUBSTANCES IN RATS; ORAL ADM. (GAVAGE)
INDIVIDUAL PUP BODY WEIGHTS -- GRAMS

TEST GROUP 0 (0 MG/KG BW/D) LACTATION DAY 7

FEMALE#	MEAN	PUP# 1	2	3	4	5	6	7	8	9	10	11	12	13	14	15	16	17	18	19
1	17.2	15.9	18.3	17.6	18.0	17.6	16.6	16.5	17.2											
2	15.1	15.5	16.2	17.2	16.0	16.1	14.7	10.9	13.1	16.9	14.8									
3	16.5	15.8	16.2	16.7	17.5	16.9	17.2	17.2	17.0	14.3										
4	16.1	16.5	16.3	16.4	15.0	16.4	16.6	15.7	15.8	16.7	15.8	16.1								
5	15.5	15.8	14.8	15.0	15.8	16.8	16.6	14.7	14.9	16.4	14.3	15.1								
6	16.3	16.2	16.7	16.0	16.4	16.9	16.7	15.8	15.7	16.4										
7	18.7	19.1	17.0	19.6	19.3	19.1	18.3	18.9	18.3											
8	16.6	16.9	17.5	15.2	16.3	17.0	16.4	17.8	16.8	15.2										
9	16.3	16.6	16.8	16.4	16.5	15.9	16.3	15.5												
10	15.4	16.3	14.1	16.6	15.7	16.0	15.2	15.1	15.6	14.7	15.2									
11	15.1	15.1	15.7	12.9	16.0	15.0	15.9	15.7	15.5	14.2	15.1	15.5	14.1							
17	15.4	16.3	15.2	16.5	14.7	13.8	15.8	15.4	15.6											
18	17.5	17.3	17.3	17.8	18.1	17.2	16.3	17.1	17.2	18.4	17.8									
19	14.7	15.3	15.5	14.3	15.0	14.7	14.2	14.1	14.6											
20	15.5	16.5	16.0	14.8	14.8	15.3	14.6	16.2	16.1	15.3	15.5									
21	17.0	18.3	17.6	17.7	17.5	17.5	16.7	17.5	14.2	17.5	17.2	17.2	15.6							
22	15.6	16.2	15.5	16.3	16.1	15.1	15.7	14.8	15.9	14.7										
23	16.9	16.9	17.5	16.2	S	16.8	16.7	17.8	17.2	16.2	17.1									
24	14.6	14.2	15.2	14.0	14.9	15.8	14.9	14.1	14.0	14.3	14.7	14.3								
25	14.4	15.1	13.8	15.3	14.9	14.6	13.2	14.6	14.4	14.7	14.7	13.1								

MEAN	16.0
S.D.	1.1
N	20

PUP STATUS CODES: S-STILLBORN

PR.NO.60R0375/88R002: REPRODUCTIVE TOX. STUDY TO DETECT EFFECTS
OF MIXED ANTI-ANDROGENIC SUBSTANCES IN RATS; ORAL ADM. (GAVAGE)
INDIVIDUAL PUP BODY WEIGHTS -- GRAMS

TEST GROUP 1 (ADI-MIX) LACTATION DAY 7

FEMALE#	MEAN	PUP# 1	2	3	4	5	6	7	8	9	10	11	12	13	14	15	16	17	18	19
26	15.9	15.9	16.1	16.1	16.5	16.0	14.8	14.7	16.3	15.5	16.7									
27	16.6	17.5	17.6	16.7	16.6	16.7	16.4	16.5	16.0	15.7	16.0									
28	17.9	19.3	18.7	17.8	17.6	17.7	16.5	17.7	17.6											
29	17.2	17.2	17.7	16.7	18.2	17.3	17.6	16.7	16.4	17.1	16.9	17.0								
30	14.2	14.5	15.0	15.0	14.3	14.3	14.7	14.1	13.3	13.8	13.3	14.4	13.5							
31	15.0	15.0	15.8	14.7	15.1	15.6	15.0	15.7	16.1	15.8	14.2	13.7	12.8							
32	18.2	18.7	19.5	18.4	18.4	18.2	18.3	16.8	17.4											
33	17.2	17.5	16.4	17.7	17.3	16.4	16.4	17.4	18.4	16.8	17.5									
34	13.6	13.3	13.8	13.6	13.0	14.5	13.8	13.2	13.6	13.8	13.2									
35	19.2	19.8	19.4	19.1	19.4	19.1	19.2	18.9	18.5											
36	17.7	19.6	18.5	18.3	17.8	17.8	17.4	16.9	16.7	17.2	16.7									
37	11.2	10.6	11.1	11.0	11.5	11.9	11.9	10.9	9.5	11.8	11.5									
43	14.4	15.3	14.5	14.5	13.0	15.1	13.6	14.7	14.3	14.4	14.3	14.9	14.5							
44	17.6	S	17.2	17.1	18.4	17.3	16.8	17.5	18.8	17.4	17.8									
45	14.9	15.2	14.9	14.4	15.3	14.6	15.5	14.7	14.5	14.6										
46	13.8	14.1	14.8	13.2	14.1	13.9	14.8	13.9	14.1	13.1	13.9	13.5	11.9	14.3						
47	17.3	16.9	18.2	16.4	18.8	18.4	15.9	16.5												
48	14.7	14.8	14.3	15.1	14.2	16.1	14.8	14.8	14.2	14.4										
49	15.6	16.4	16.6	16.0	16.1	15.8	15.3	15.9	15.2	15.3	15.3	14.1	15.6							
50	15.4	16.6	14.6	15.8	16.0	15.3	14.7	15.3	14.8											

MEAN	15.9
S.D.	2.0
N	20

PUP STATUS CODES: S-STILLBORN

PR.NO.60R0375/88R002: REPRODUCTIVE TOX. STUDY TO DETECT EFFECTS
OF MIXED ANTI-ANDROGENIC SUBSTANCES IN RATS; ORAL ADM. (GAVAGE)
INDIVIDUAL PUP BODY WEIGHTS -- GRAMS

TEST GROUP 2 (NOAEL-MIX) LACTATION DAY 7

FEMALE#	MEAN	PUP# 1	2	3	4	5	6	7	8	9	10	11	12	13	14	15	16	17	18	19
51	13.7	14.3	13.7	13.5	13.3	14.2	13.5	13.1	13.9	14.1	13.4									
52	16.4	17.1	16.3	16.4	16.5	16.4	15.3	16.8	16.4	16.0	16.6	16.4								
53	16.8	18.2	17.1	17.0	17.6	16.6	S	16.3	16.3	15.6										
54	16.9	17.5	17.6	17.0	18.0	16.2	17.5	17.3	16.7	15.8	15.4									
55	13.7	14.1	13.5	14.0	14.1	14.7	13.5	14.0	13.7	13.3	13.5	13.1	12.9							
56	8.6	Z	10.9	D	D	D	Z	6.3	D	Z	D	D	D	Z	S					
57	14.4	15.7	14.5	14.2	14.9	15.5	13.8	Z	13.8	13.1	14.3	14.8	13.3							
58	18.0	17.6	18.4	18.8	18.9	18.8	17.7	17.9	16.2											
59	17.9	17.1	18.4	17.6	18.2	17.8	18.3	17.7	18.6	17.2										
60	13.4	14.0	13.7	12.9	13.5	12.6	13.1	12.4	13.6	14.1	13.6	14.7	13.4	12.8						
61	17.4	17.9	17.2	17.9	17.7	17.6	17.4	16.4	16.9	17.4										
62	16.3	16.8	16.1	17.6	17.4	14.7	15.9	17.5	14.3											
68	18.0	18.2	18.2	18.0	18.7	18.0	17.5	18.4	17.0	18.0	18.3									
69	15.9	16.1	17.0	15.8	16.5	16.8	16.5	12.2	15.9	16.0										
70	18.2	18.7	18.5	18.7	17.2	18.4	17.5	Z												
71	17.5	17.5	17.5	17.4	17.7	18.2	17.1	17.2	18.1	16.4										
72	16.0	16.2	15.5	15.8	15.8	16.3	16.2	17.2	16.9	15.9	15.1	15.0	16.3							
73	14.7	15.1	14.5	14.7	15.2	14.9	15.5	14.0	14.1	15.0	13.9									
74	14.0	14.6	14.0	14.5	14.7	13.6	13.6	14.0	14.6	13.4	13.3	14.2								
75	15.4	16.6	16.6	17.0	16.2	13.5	17.0	15.5	15.2	10.8										

MEAN	15.7
S.D.	2.3
N	20

PUP STATUS CODES: S-STILLBORN D -DIED Z -CANNIBALIZED

PR.NO.60R0375/88R002: REPRODUCTIVE TOX. STUDY TO DETECT EFFECTS
OF MIXED ANTI-ANDROGENIC SUBSTANCES IN RATS; ORAL ADM. (GAVAGE)
INDIVIDUAL PUP BODY WEIGHTS -- GRAMS

TEST GROUP 3 (LOAEL-MIX) LACTATION DAY 7

FEMALE#	MEAN	PUP# 1	2	3	4	5	6	7	8	9	10	11	12	13	14	15	16	17	18	19
77	15.8	16.7	15.9	15.2	13.4	16.5	17.0	15.3	16.5											
78	16.7	17.1	17.1	17.8	16.5	18.0	16.4	17.2	16.6	13.5	S									
80	16.5	17.3	18.0	19.4	S	S	10.5	17.4	S	S										
82	14.5	14.4	15.3	14.2	13.1	15.6	15.0	15.1	15.3	13.7	13.9	14.3								
83	17.4	17.6	18.3	17.7	17.4	S	17.8	17.1	16.5	16.9	17.2									
84	17.7	17.5	17.5	19.4	18.3	15.8	16.1	19.0												
85	20.1	22.0	Z	S	S	17.6	19.7	20.9	19.7	20.5	Z									
86	15.8	15.9	16.7	16.3	16.3	15.7	15.4	15.5	15.8	15.5	15.4	15.3								
87	16.0	16.5	16.3	15.8	16.5	16.6	15.6	15.9	15.7	15.1	16.0	15.7	15.9							
93	17.8	19.4	17.8	17.7	17.2	16.6	17.4	18.5												
94	17.7	18.3	18.8	18.6	17.4	17.6	16.8	18.1	16.8	17.5	17.4									
95	15.8	16.1	15.7	15.6	15.7	16.1	16.3	16.2	16.1	15.0	15.2									
96	17.4	18.2	18.2	18.4	17.7	16.9	17.3	17.5	17.2	15.6										
97	17.7	18.0	17.2	17.3	18.4	17.8	17.9	17.5	17.4	18.0										
98	12.8	12.5	13.1	13.8	11.5	12.6	12.7	12.9	12.7	13.1	12.7	S								
99	16.1	17.4	16.5	16.7	13.2	16.1	S	16.5	16.4	15.7	16.1									
100	17.4	18.4	18.1	17.3	16.9	17.1	17.6	16.9	17.0	17.3										
MEAN	16.7																			
S.D.	1.6																			
N	17																			

PUP STATUS CODES: S-STILLBORN Z -CANNIBALIZED

PR.NO.60R0375/88R002: REPRODUCTIVE TOX. STUDY TO DETECT EFFECTS
OF MIXED ANTI-ANDROGENIC SUBSTANCES IN RATS; ORAL ADM. (GAVAGE)
INDIVIDUAL PUP BODY WEIGHTS -- GRAMS

TEST GROUP 4(0.00025 MG/KG BW/D) LACTATION DAY 7

FEMALE#	MEAN	PUP# 1	2	3	4	5	6	7	8	9	10	11	12	13	14	15	16	17	18	19
101	19.2	19.2	20.6	19.5	17.9	19.2	19.8	18.8	18.9	19.1										
102	14.7	14.8	14.4	14.1	13.9	14.1	15.7	14.8	14.9	15.2	14.8									
103	14.3	13.7	14.6	13.2	15.1	14.4	14.0	14.5	14.3	14.3	14.4									
104	16.1	16.5	15.8	16.7	15.2	16.1	16.9	15.5	15.8	16.6	16.1	15.4								
105	18.0	19.0	17.6	18.8	17.6	18.1	17.7	17.2												
106	19.5	19.5	20.1	19.8	19.5	18.4	21.1	19.7	19.4	17.7										
107	15.4	16.5	16.8	16.1	15.4	15.4	15.2	13.1	15.3	15.4	15.0									
108	15.5	16.3	15.4	16.1	15.6	15.4	15.8	14.6	15.3	15.2										
109	15.8	16.6	16.0	16.5	16.9	14.8	15.4	16.6	13.4	16.4										
111	17.6	17.7	18.9	18.0	17.3	17.7	18.0	17.0	16.0	17.8	17.4									
117	16.8	16.2	17.1	17.9	17.0	16.4	16.9	16.7	16.5	16.6										
118	15.8	15.9	16.3	16.6	16.7	15.8	15.7	16.2	15.1	14.6	15.6	16.3	15.2							
119	20.9	21.3	21.8	20.3	S	22.1	20.1	20.5	20.3	20.8										
120	19.3	20.0	20.8	16.9	S	19.0	19.9	Z	S	S	S									
121	14.1	14.1	14.3	13.8	14.3	14.2	14.4	14.3	13.6	13.5	13.8	13.6	14.8							
123	16.9	16.6	16.6	17.3	16.6	17.4	16.7	16.0	17.7											
124	15.2	15.4	15.4	15.7	14.8	14.9	14.3	15.4	14.7	15.8	15.3	15.2								
125	14.8	15.5	15.8	14.0	14.5	14.4	15.5	15.6	14.6	14.5	14.1	14.0								
MEAN	16.7																			
S.D.	2.0																			
N	18																			

PUP STATUS CODES: S-STILLBORN Z -CANNIBALIZED

PR.NO.60R0375/88R002: REPRODUCTIVE TOX. STUDY TO DETECT EFFECTS
OF MIXED ANTI-ANDROGENIC SUBSTANCES IN RATS; ORAL ADM. (GAVAGE)
INDIVIDUAL PUP BODY WEIGHTS -- GRAMS

TEST GROUP 0 (0 MG/KG BW/D) LACTATION DAY 14

FEMALE#	MEAN	PUP# 1	2	3	4	5	6	7	8	9	10	11	12	13	14	15	16	17	18	19
1	32.4	30.1	33.9	32.9	33.2	33.6	31.7	31.6	32.4											
2	28.9	29.5	29.4	31.2	30.1	30.3	28.5	24.1	26.9	30.4	28.5									
3	29.7	29.0	29.3	28.8	30.7	30.4	32.0	30.8	29.5	26.4										
4	28.4	28.9	28.9	28.7	27.0	28.3	28.4	28.9	28.4	28.9	28.6	27.8								
5	28.2	29.4	27.4	27.2	29.3	30.4	29.8	27.8	27.1	30.2	25.5	26.6								
6	28.0	28.2	28.0	27.4	28.3	28.9	29.0	27.4	26.9	27.5										
7	36.5	37.1	34.5	38.0	37.4	37.3	35.6	36.5	35.8											
8	30.4	30.5	31.5	29.0	29.8	30.7	30.0	32.1	31.3	29.0										
9	29.2	29.4	29.6	30.5	28.9	28.6	28.9	28.3												
10	28.5	30.4	27.0	29.7	28.6	30.0	27.8	28.6	27.1	27.0	28.6									
11	26.6	27.1	27.4	24.5	27.0	25.9	27.9	27.2	27.9	25.1	26.7	27.0	25.5							
17	27.9	28.6	27.6	29.4	27.2	26.4	28.3	27.5	27.9											
18	32.1	31.5	31.9	32.5	33.1	32.7	30.8	31.9	32.0	33.2	31.8									
19	28.8	29.4	30.2	28.0	29.1	28.9	28.6	27.4	28.8											
20	28.0	28.9	27.9	27.6	26.5	28.3	27.4	28.8	29.0	27.9	27.5									
21	29.9	33.3	30.0	30.0	30.1	30.3	30.2	29.6	26.1	30.0	30.5	30.9	28.1							
22	28.5	29.0	29.2	29.9	29.6	27.9	28.2	26.9	28.7	27.3										
23	32.6	33.4	33.0	32.1	S	32.0	32.6	33.4	31.7	33.0	32.5									
24	26.6	26.1	27.9	26.4	27.1	28.8	26.9	25.9	25.2	26.0	26.7	25.1								
25	27.2	26.7	26.4	28.8	28.3	28.1	25.2	26.7	27.1	27.6	28.0	25.8								

MEAN	29.4
S.D.	2.4
N	20

PUP STATUS CODES: S-STILLBORN

PR.NO.60R0375/88R002: REPRODUCTIVE TOX. STUDY TO DETECT EFFECTS
OF MIXED ANTI-ANDROGENIC SUBSTANCES IN RATS; ORAL ADM. (GAVAGE)
INDIVIDUAL PUP BODY WEIGHTS -- GRAMS

TEST GROUP 1 (ADI-MIX) LACTATION DAY 14

FEMALE#	MEAN	PUP# 1	2	3	4	5	6	7	8	9	10	11	12	13	14	15	16	17	18	19
26	28.6	28.5	29.4	29.6	29.8	28.9	26.9	27.3	28.9	28.3	28.9									
27	31.1	31.3	32.5	31.7	31.0	32.2	31.6	30.1	30.6	30.0	30.0									
28	33.6	34.9	34.2	33.8	33.7	33.9	31.7	33.6	32.6											
29	30.7	30.7	31.8	30.0	31.9	31.4	31.6	30.4	29.7	30.3	30.3	30.0								
30	27.6	27.9	28.3	28.6	28.4	28.2	27.6	27.3	26.5	27.2	26.8	27.6	26.7							
31	27.6	26.8	28.7	29.0	27.5	28.7	26.4	30.0	28.6	28.2	25.5	27.2	24.8							
32	34.2	34.9	36.3	34.7	34.3	34.0	34.8	31.3	33.3											
33	32.1	31.4	31.0	33.0	32.1	31.8	30.8	32.8	32.9	32.4	32.6									
34	24.7	23.9	25.2	24.8	24.8	25.7	24.2	24.5	25.2	24.7	24.4									
35	35.3	36.9	36.0	35.1	35.7	35.1	35.1	35.1	33.3											
36	31.2	33.7	31.1	32.6	32.0	31.5	31.1	30.2	29.7	30.8	29.1									
37	21.1	20.0	21.8	19.9	21.9	23.6	20.5	19.3	20.9	22.8	20.2									
43	25.4	26.3	25.9	26.1	24.0	26.6	25.0	25.4	25.1	25.0	25.5	25.9	24.5							
44	31.4	S	31.3	31.1	32.4	31.1	30.2	30.9	32.7	31.1	31.4									
45	29.5	30.4	29.5	28.8	30.5	28.9	29.6	29.7	29.6	28.4										
46	25.6	26.2	27.2	25.2	27.3	24.9	27.5	25.9	26.8	24.8	25.1	24.9	21.8	25.0						
47	32.0	31.6	33.2	31.5	32.8	33.6	29.8	31.7												
48	28.9	29.1	28.1	28.7	28.0	31.0	28.8	28.7	28.5	28.8										
49	28.2	29.2	29.3	27.9	29.0	28.2	27.7	28.4	27.4	28.0	28.5	26.1	28.2							
50	28.2	28.1	27.5	29.1	28.2	28.1	27.0	29.0	28.6											

MEAN	29.3
S.D.	3.5
N	20

PUP STATUS CODES: S-STILLBORN

PR.NO.60R0375/88R002: REPRODUCTIVE TOX. STUDY TO DETECT EFFECTS
OF MIXED ANTI-ANDROGENIC SUBSTANCES IN RATS; ORAL ADM. (GAVAGE)
INDIVIDUAL PUP BODY WEIGHTS -- GRAMS

TEST GROUP 2 (NOAEL-MIX) LACTATION DAY 14

FEMALE#	MEAN	PUP# 1	2	3	4	5	6	7	8	9	10	11	12	13	14	15	16	17	18	19
51	27.3	28.2	27.6	27.1	27.1	27.9	27.0	26.6	27.8	27.3	26.8									
52	30.2	30.8	30.6	30.0	31.0	29.7	28.9	30.8	29.8	29.7	30.4	30.3								
53	30.2	32.2	30.7	30.9	30.5	29.3	S	29.7	29.4	29.0										
54	30.4	30.9	31.4	30.0	32.4	29.2	31.8	30.6	30.1	29.4	28.3									
55	25.4	26.6	24.7	25.7	25.9	26.9	24.8	25.6	26.1	25.1	25.6	23.9	23.8							
56	16.7	Z	20.5	D	D	D	Z	12.9	D	Z	D	D	D	Z	S					
57	28.0	29.4	28.7	27.7	28.1	29.3	27.5	Z	27.0	26.1	28.3	28.5	26.9							
58	30.6	30.3	30.8	31.8	32.4	30.9	29.5	30.1	28.6											
59	33.7	33.3	35.1	33.6	34.0	33.2	34.8	32.7	34.9	31.7										
60	23.4	24.6	23.9	22.6	23.2	22.0	22.6	22.1	22.9	24.5	25.0	24.5	23.6	22.3						
61	31.3	31.9	30.8	32.0	31.2	32.0	31.6	30.0	31.5	30.3										
62	29.7	29.0	30.1	31.0	31.4	29.0	30.1	29.5	27.6											
68	32.5	33.3	32.6	32.5	33.6	31.7	31.8	33.4	31.4	32.1	32.9									
69	29.4	29.8	30.3	29.7	30.3	30.6	29.6	24.3	30.0	30.4										
70	33.2	34.6	34.1	33.8	31.0	34.3	31.3	Z												
71	31.3	31.1	31.6	32.0	32.0	32.1	30.4	30.5	32.6	29.7										
72	28.0	28.2	26.6	27.3	28.4	29.5	29.3	29.5	27.8	27.6	26.6	26.7	28.1							
73	26.8	27.1	27.0	26.9	27.5	26.7	27.8	26.1	25.6	27.5	25.8									
74	25.1	25.2	25.7	26.3	25.4	24.7	24.4	25.6	26.0	23.6	23.6	25.5								
75	30.1	32.9	32.0	32.0	31.7	28.3	31.2	29.6	29.8	23.7										

MEAN	28.7
S.D.	3.9
N	20

PUP STATUS CODES: S-STILLBORN D -DIED Z -CANNIBALIZED

22-AUG-13

88R002 TABLE : IIA- 141

PR.NO.60R0375/88R002: REPRODUCTIVE TOX. STUDY TO DETECT EFFECTS
OF MIXED ANTI-ANDROGENIC SUBSTANCES IN RATS; ORAL ADM. (GAVAGE)
INDIVIDUAL PUP BODY WEIGHTS -- GRAMS

TEST GROUP 3 (LOAEL-MIX) LACTATION DAY 14

FEMALE#	MEAN	PUP# 1	2	3	4	5	6	7	8	9	10	11	12	13	14	15	16	17	18	19
77	30.6	30.8	31.1	30.3	28.1	30.8	31.0	30.1	32.2											
78	31.8	31.4	32.3	33.1	31.5	33.1	31.5	33.5	31.3	28.1	S									
80	35.1	35.1	34.5	36.2	S	S	Z	34.4	S	S										
82	26.2	26.8	27.2	25.2	25.1	26.0	26.6	26.6	27.2	25.6	26.2	25.3								
83	29.3	28.5	30.8	29.9	29.9	S	29.7	28.8	28.6	28.5	29.0									
84	31.3	31.6	30.6	32.9	32.3	29.6	30.0	32.3												
85	38.2	39.2	Z	S	S	36.6	38.6	38.7	38.4	37.7	Z									
86	29.2	29.6	30.6	30.3	29.5	29.2	28.0	28.4	29.5	29.1	28.5	28.6								
87	28.3	30.0	27.6	28.2	28.9	27.6	28.3	27.6	28.6	27.1	29.4	27.9	28.9							
93	34.1	36.7	34.3	33.3	33.7	32.3	34.0	34.7												
94	31.3	31.4	32.5	32.9	31.9	30.6	29.6	32.0	29.8	31.1	30.7									
95	28.5	29.2	27.8	28.8	28.3	28.7	29.7	29.5	27.7	27.5	27.9									
96	30.7	32.4	31.5	32.0	30.2	29.5	31.0	30.7	30.6	28.8										
97	32.3	32.8	32.1	32.0	33.4	32.5	32.6	31.8	31.0	32.6										
98	26.1	25.5	27.6	27.8	24.5	25.8	26.0	26.2	25.4	26.2	26.1	S								
99	29.9	31.5	31.1	30.3	26.4	29.7	S	31.1	29.0	30.2	30.2									
100	32.3	33.8	33.3	32.4	31.2	31.5	32.8	32.1	31.7	31.8										

MEAN	30.9
S.D.	3.1
N	17

PUP STATUS CODES: S-STILLBORN Z -CANNIBALIZED

PR.NO.60R0375/88R002: REPRODUCTIVE TOX. STUDY TO DETECT EFFECTS
OF MIXED ANTI-ANDROGENIC SUBSTANCES IN RATS; ORAL ADM. (GAVAGE)
INDIVIDUAL PUP BODY WEIGHTS -- GRAMS

TEST GROUP 4(0.00025 MG/KG BW/D) LACTATION DAY 14

FEMALE#	MEAN	PUP# 1	2	3	4	5	6	7	8	9	10	11	12	13	14	15	16	17	18	19
101	35.0	35.5	36.6	36.2	32.6	35.4	35.1	34.7	34.1	34.6										
102	24.9	25.0	24.7	24.4	23.8	24.4	26.2	25.3	24.7	25.0	25.6									
103	26.0	25.9	26.2	25.3	26.9	26.2	25.5	25.9	26.3	25.8	25.9									
104	29.7	30.6	30.1	31.1	28.6	29.6	30.5	28.1	30.2	28.6	29.6	30.2								
105	33.4	34.5	33.2	34.8	32.7	33.7	33.0	32.2												
106	35.6	35.5	36.3	35.9	35.9	34.0	37.2	35.9	36.3	33.6										
107	28.6	30.5	30.8	29.4	29.2	27.8	28.4	25.0	27.5	28.0	29.1									
108	28.9	29.8	28.3	29.9	28.6	28.8	29.8	27.8	28.2	29.3										
109	30.6	31.2	31.1	31.2	31.5	29.7	30.7	31.3	28.1	31.0										
111	30.6	30.7	32.5	31.0	30.4	31.3	31.2	29.4	28.6	30.4	30.1									
117	30.4	30.1	30.9	31.6	30.5	29.8	30.4	29.8	30.0	30.2										
118	28.0	28.8	29.0	29.0	28.9	27.7	28.0	28.2	27.2	26.3	28.0	28.1	26.8							
119	37.5	38.7	39.0	36.5	S	38.5	36.8	35.9	37.1	37.1										
120	38.4	39.4	40.4	35.0	S	39.3	37.9	Z	S	S	S									
121	26.9	26.6	26.7	26.7	26.9	27.2	27.6	27.6	25.8	24.9	27.3	26.8	29.2							
123	33.4	33.2	33.2	33.5	33.4	33.7	33.0	32.7	34.7											
124	27.7	27.6	28.2	27.7	27.2	27.7	26.5	29.1	27.3	28.7	27.3	27.3								
125	26.4	27.2	26.6	25.9	26.2	25.9	28.1	27.6	26.3	26.0	25.7	25.1								

MEAN	30.7
S.D.	4.0
N	18

PUP STATUS CODES: S-STILLBORN Z -CANNIBALIZED

PR.NO.60R0375/88R002: REPRODUCTIVE TOX. STUDY TO DETECT EFFECTS
OF MIXED ANTI-ANDROGENIC SUBSTANCES IN RATS; ORAL ADM. (GAVAGE)
INDIVIDUAL PUP BODY WEIGHTS -- GRAMS

TEST GROUP 0 (0 MG/KG BW/D) LACTATION DAY 21

FEMALE#	MEAN	PUP# 1	2	3	4	5	6	7	8	9	10	11	12	13	14	15	16	17	18	19
1	50.6	48.9	49.5	52.6	52.6	52.9	51.4	51.4	45.6											
2	45.5	47.5	46.2	49.4	47.0	45.7	45.7	38.2	41.9	48.3	44.9									
3	46.2	43.0	43.2	44.6	49.9	48.2	49.0	48.8	47.3	42.1										
4	44.2	44.0	45.2	46.8	40.0	44.7	45.8	44.1	43.8	44.8	43.8	43.0								
5	45.5	47.4	43.1	44.1	45.6	49.5	48.6	43.4	44.1	48.7	41.9	44.0								
6	44.2	43.9	44.7	43.2	44.4	46.0	45.9	43.6	41.6	44.3										
7	55.2	57.3	53.9	58.1	56.4	56.5	52.9	54.2	52.1											
8	46.0	45.5	48.4	43.4	46.6	45.9	45.1	47.8	46.4	44.6										
9	45.7	46.2	46.2	48.1	47.6	43.4	45.4	43.0												
10	41.8	43.4	37.9	44.3	42.9	43.7	41.6	41.2	40.1	40.4	42.2									
11	39.8	40.3	40.1	36.4	39.7	36.9	43.2	41.6	40.6	38.4	39.8	40.7	39.9							
17	44.3	44.4	43.3	45.9	43.8	42.8	47.1	44.0	43.0											
18	50.3	49.4	49.5	51.0	50.6	51.2	48.5	50.9	52.1	50.6	49.6									
19	45.7	47.9	47.5	45.4	46.3	45.8	44.0	43.2	45.5											
20	44.5	46.0	43.9	43.5	43.8	44.3	43.5	46.4	44.5	45.8	43.0									
21	45.7	51.2	45.4	47.2	47.0	44.4	45.6	44.2	40.9	45.8	46.2	46.8	44.0							
22	43.6	46.6	42.8	44.1	45.6	42.6	42.9	41.4	43.6	43.0										
23	48.5	49.1	49.9	49.5	S	46.9	48.5	46.2	46.4	50.8	49.5									
24	42.2	41.4	43.6	41.7	44.0	44.7	43.3	42.7	37.7	41.9	42.6	40.2								
25	41.0	39.2	39.5	41.4	42.1	43.2	39.7	41.6	40.5	41.3	43.1	38.9								

MEAN	45.5
S.D.	3.6
N	20

PUP STATUS CODES: S-STILLBORN

PR.NO.60R0375/88R002: REPRODUCTIVE TOX. STUDY TO DETECT EFFECTS
OF MIXED ANTI-ANDROGENIC SUBSTANCES IN RATS; ORAL ADM. (GAVAGE)
INDIVIDUAL PUP BODY WEIGHTS -- GRAMS

TEST GROUP 1 (ADI-MIX) LACTATION DAY 21

FEMALE#	MEAN	PUP# 1	2	3	4	5	6	7	8	9	10	11	12	13	14	15	16	17	18	19
26	43.9	41.2	45.6	44.3	45.8	44.3	41.2	42.6	46.0	43.8	44.6									
27	49.3	48.8	51.9	48.7	46.8	51.8	51.7	48.8	47.2	49.1	48.6									
28	50.8	52.9	51.9	50.9	50.3	50.4	49.7	51.0	49.2											
29	50.2	49.5	51.7	50.8	51.7	50.0	51.2	49.6	49.5	50.7	49.9	47.2								
30	41.8	41.6	43.2	43.4	42.0	42.4	41.0	40.6	41.5	42.0	39.7	41.8	41.9							
31	41.9	41.5	42.1	45.0	41.6	43.4	40.7	45.4	42.8	40.7	39.7	40.9	39.1							
32	53.0	55.7	56.6	53.7	52.6	52.8	53.4	46.8	52.3											
33	49.7	49.7	49.2	51.1	50.8	49.3	47.7	48.3	51.3	49.8	50.3									
34	39.3	37.5	40.4	39.7	39.0	42.1	35.4	39.7	40.2	39.7	39.6									
35	54.2	57.2	55.2	54.6	54.7	54.1	52.9	53.0	52.3											
36	48.9	54.0	47.6	51.2	49.1	49.6	49.1	47.5	47.0	47.7	46.0									
37	31.6	30.5	31.8	31.6	33.6	34.9	30.9	29.6	29.3	33.9	29.6									
43	41.2	41.1	41.6	40.5	38.3	42.8	39.8	41.5	41.6	40.6	43.5	44.0	38.6							
44	48.2	S	49.4	48.9	49.4	47.5	43.1	47.8	50.9	49.1	47.3									
45	47.4	48.8	46.6	46.0	48.1	46.5	48.0	48.9	47.1	46.6										
46	38.0	39.1	40.3	37.4	39.4	37.4	39.3	38.6	40.7	37.0	37.4	38.8	31.4	37.0						
47	49.2	50.6	48.6	48.1	50.6	50.0	46.8	49.4												
48	44.7	44.6	45.4	46.2	41.6	47.4	45.9	44.4	43.1	44.1										
49	43.5	45.8	45.5	42.5	43.4	44.1	43.7	44.3	41.4	43.2	42.5	42.0	43.7							
50	43.4	42.5	42.1	46.3	44.6	42.7	40.9	45.0	42.8											

MEAN	45.5
S.D.	5.6
N	20

PUP STATUS CODES: S-STILLBORN

PR.NO.60R0375/88R002: REPRODUCTIVE TOX. STUDY TO DETECT EFFECTS
OF MIXED ANTI-ANDROGENIC SUBSTANCES IN RATS; ORAL ADM. (GAVAGE)
INDIVIDUAL PUP BODY WEIGHTS -- GRAMS

TEST GROUP 2 (NOAEL-MIX) LACTATION DAY 21

FEMALE#	MEAN	PUP# 1	2	3	4	5	6	7	8	9	10	11	12	13	14	15	16	17	18	19
51	43.9	44.7	44.5	42.6	44.9	45.2	44.6	42.1	44.0	44.4	41.6									
52	46.4	48.5	44.3	46.8	46.0	45.8	44.6	48.1	46.2	45.1	46.8	47.7								
53	48.4	49.9	50.1	51.4	48.5	47.2	S	46.2	46.7	47.2										
54	48.8	50.0	50.1	47.7	51.7	47.2	50.5	50.0	47.9	46.4	46.5									
55	39.9	41.1	38.8	39.1	40.8	43.0	38.5	39.4	40.5	38.9	41.6	39.6	37.8							
56	28.4	Z	35.0	D	D	D	Z	21.8	D	Z	D	D	D	Z	S					
57	44.3	46.8	45.0	44.9	45.9	45.6	43.8	Z	43.1	42.2	42.6	44.7	43.2							
58	47.8	47.5	44.8	51.2	50.8	48.8	46.6	47.3	45.2											
59	51.8	51.0	52.1	50.9	51.9	52.9	54.7	50.2	51.9	50.8										
60	35.6	38.5	37.1	32.9	35.8	35.7	35.0	35.1	35.6	36.0	37.7	35.7	34.4	33.8						
61	48.4	49.6	48.4	50.3	48.6	48.0	47.3	46.3	49.3	48.1										
62	48.4	45.9	49.4	49.7	51.2	46.0	50.3	48.2	46.3											
68	47.5	47.4	46.1	47.8	49.3	45.4	45.9	49.8	47.2	48.0	48.5									
69	45.5	45.5	45.2	46.4	47.6	49.5	45.7	37.8	45.8	45.6										
70	52.8	55.8	53.6	53.7	48.4	53.6	51.6	Z												
71	48.4	49.9	48.3	49.6	48.4	49.4	46.4	46.7	51.4	45.5										
72	43.4	44.2	40.4	41.6	43.2	45.2	45.1	45.2	43.3	43.2	42.2	41.9	45.4							
73	42.2	41.6	42.6	42.7	42.5	40.1	45.9	41.9	40.3	44.2	40.6									
74	40.7	41.2	41.4	41.3	40.4	41.4	40.5	40.8	41.7	38.8	40.0	40.5								
75	43.5	48.4	45.6	46.8	44.7	40.4	44.1	42.2	43.8	35.3										

MEAN	44.8
S.D.	5.6
N	20

PUP STATUS CODES: S-STILLBORN D -DIED Z -CANNIBALIZED

PR.NO.60R0375/88R002: REPRODUCTIVE TOX. STUDY TO DETECT EFFECTS
OF MIXED ANTI-ANDROGENIC SUBSTANCES IN RATS; ORAL ADM. (GAVAGE)
INDIVIDUAL PUP BODY WEIGHTS -- GRAMS

TEST GROUP 3 (LOAEL-MIX) LACTATION DAY 21

FEMALE#	MEAN	PUP# 1	2	3	4	5	6	7	8	9	10	11	12	13	14	15	16	17	18	19
77	47.5	48.9	49.1	47.2	44.0	46.9	47.7	46.5	49.7											
78	52.0	52.8	49.8	55.4	51.3	55.4	52.6	53.7	50.8	46.0	S									
80	54.2	52.0	54.8	55.9	S	S	Z	54.1	S	S										
82	43.5	44.7	47.1	42.2	42.5	45.2	43.0	42.8	43.1	43.1	41.5	42.9								
83	46.1	44.9	46.5	47.1	48.1	S	45.9	47.7	44.7	43.9	46.0									
84	48.3	47.6	47.1	50.4	50.0	44.5	45.9	52.3												
85	59.3	61.6	Z	S	S	56.0	60.3	60.8	59.6	57.6	Z									
86	46.3	45.9	48.3	50.4	48.5	42.6	45.3	43.1	49.6	46.5	44.9	44.4								
87	43.4	47.5	42.2	40.7	45.1	42.6	44.2	42.9	42.1	41.1	43.6	43.1	45.3							
93	51.6	54.7	50.7	52.1	51.6	49.0	50.9	52.3												
94	49.5	51.0	51.5	50.8	48.3	48.8	45.6	51.0	48.5	50.5	48.8									
95	45.2	47.7	43.7	44.5	43.7	45.2	48.3	45.8	45.4	41.8	45.6									
96	48.4	51.3	48.1	50.1	48.0	48.0	47.4	48.5	47.7	46.7										
97	48.6	51.3	49.7	48.8	49.4	49.2	48.0	47.9	45.8	47.4										
98	44.0	43.7	43.6	47.2	41.2	43.5	43.8	43.1	43.3	45.4	45.7	S								
99	48.3	51.5	48.9	48.3	41.4	49.0	S	48.0	50.1	47.9	49.7									
100	50.1	49.8	52.8	52.6	46.5	48.1	52.3	49.2	48.7	51.0										
MEAN	48.6																			
S.D.	4.1																			
N	17																			

PUP STATUS CODES: S-STILLBORN Z -CANNIBALIZED

PR.NO.60R0375/88R002: REPRODUCTIVE TOX. STUDY TO DETECT EFFECTS
OF MIXED ANTI-ANDROGENIC SUBSTANCES IN RATS; ORAL ADM. (GAVAGE)
INDIVIDUAL PUP BODY WEIGHTS -- GRAMS

TEST GROUP 4(0.00025 MG/KG BW/D) LACTATION DAY 21

FEMALE#	MEAN	PUP# 1	2	3	4	5	6	7	8	9	10	11	12	13	14	15	16	17	18	19
101	54.7	55.6	55.5	57.7	51.5	55.7	56.7	52.9	53.2	53.9										
102	40.3	40.8	41.7	39.6	38.2	39.8	40.7	39.9	39.3	41.7	41.8									
103	40.9	38.8	42.1	39.0	42.9	41.1	41.6	39.8	41.7	39.5	42.9									
104	45.6	46.0	46.1	47.4	43.2	44.8	45.0	43.7	47.9	45.9	44.6	46.9								
105	52.2	53.7	53.1	54.1	51.3	52.7	52.4	48.3												
106	52.7	52.0	54.7	53.6	52.1	50.6	54.0	53.8	53.7	50.2										
107	45.9	48.0	49.1	45.4	44.7	46.7	47.7	41.1	46.0	44.7	45.3									
108	46.4	48.6	47.8	48.3	45.3	46.4	47.2	43.6	45.9	44.3										
109	47.4	50.5	46.2	49.0	49.9	45.1	46.9	48.0	43.3	48.0										
111	46.2	48.4	48.5	46.5	46.0	46.1	45.5	45.8	42.9	46.2	46.3									
117	48.2	47.1	50.4	49.9	48.1	47.7	48.0	47.0	47.1	48.6										
118	44.6	45.5	46.0	46.5	44.8	44.9	44.0	44.4	43.1	43.1	45.3	44.4	43.0							
119	57.3	58.0	62.1	58.8	S	61.2	54.9	54.9	52.4	56.3										
120	57.8	58.3	60.9	54.0	S	58.8	56.8	Z	S	S	S									
121	41.5	42.4	41.3	41.5	42.3	41.9	43.0	39.1	40.2	39.4	41.7	41.0	43.9							
123	52.5	53.0	51.9	54.0	51.7	54.2	52.8	49.4	52.8											
124	44.5	43.8	47.3	44.1	44.5	43.5	43.7	45.9	44.0	45.4	43.4	44.3								
125	41.5	42.3	42.7	39.6	42.1	38.4	43.2	44.8	41.0	40.5	39.5	42.1								
MEAN	47.8																			
S.D.	5.5																			
N	18																			

PUP STATUS CODES: S-STILLBORN Z -CANNIBALIZED

PR.NO.60R0375/88R002: REPRODUCTIVE TOX. STUDY TO DETECT EFFECTS
OF MIXED ANTI-ANDROGENIC SUBSTANCES IN RATS; ORAL ADM. (GAVAGE)
INDIVIDUAL PUP NECROPSY OBSERVATIONS

TEST GROUP 0 (0 MG/KG BW/D)

FEMALE#	PUP#	STATUS	DAY	OBSERVATION	POSITION/GRADE
1	1	K	21	NOTHING ABNORMAL DETECTED	
	3	K	21	NOTHING ABNORMAL DETECTED	
	5	K	21	NOTHING ABNORMAL DETECTED	
	6	K	21	NOTHING ABNORMAL DETECTED	
	8	K	21	NOTHING ABNORMAL DETECTED	
2	1	K	21	NOTHING ABNORMAL DETECTED	
	2	K	21	NOTHING ABNORMAL DETECTED	
	3	K	21	NOTHING ABNORMAL DETECTED	
	4	K	21	NOTHING ABNORMAL DETECTED	
	6	K	21	NOTHING ABNORMAL DETECTED	
	7	K	21	NOTHING ABNORMAL DETECTED	
	9	K	21	NOTHING ABNORMAL DETECTED	
3	2	K	21	NOTHING ABNORMAL DETECTED	
	3	K	21	NOTHING ABNORMAL DETECTED	
	4	K	21	NOTHING ABNORMAL DETECTED	
	5	K	21	NOTHING ABNORMAL DETECTED	
	8	K	21	NOTHING ABNORMAL DETECTED	
	9	K	21	NOTHING ABNORMAL DETECTED	
4	1	K	21	NOTHING ABNORMAL DETECTED	
	3	K	21	NOTHING ABNORMAL DETECTED	
	5	K	21	NOTHING ABNORMAL DETECTED	
	6	K	21	NOTHING ABNORMAL DETECTED	
	7	K	21	NOTHING ABNORMAL DETECTED	
	8	K	21	NOTHING ABNORMAL DETECTED	
	10	K	21	NOTHING ABNORMAL DETECTED	
	11	K	21	NOTHING ABNORMAL DETECTED	
5	1	K	21	NOTHING ABNORMAL DETECTED	
	2	K	21	NOTHING ABNORMAL DETECTED	
	4	K	21	NOTHING ABNORMAL DETECTED	
	5	K	21	NOTHING ABNORMAL DETECTED	
	6	K	21	NOTHING ABNORMAL DETECTED	
	7	K	21	NOTHING ABNORMAL DETECTED	
	9	K	21	NOTHING ABNORMAL DETECTED	
	11	K	21	NOTHING ABNORMAL DETECTED	

POSITION/GRADE CODE: R-RIGHT, L-LEFT, B-BILATE , 1-SLIGHT, 2-MODERATE, 3-MARKED, P-PRESENT
PUP STATUS CODES: K-SCHEDULED SACRIFICE
NUMBER FOLLOWING PUP STATUS = DAY POST PARTUM OF DEATH

PR.NO.60R0375/88R002: REPRODUCTIVE TOX. STUDY TO DETECT EFFECTS
OF MIXED ANTI-ANDROGENIC SUBSTANCES IN RATS; ORAL ADM. (GAVAGE)
INDIVIDUAL PUP NECROPSY OBSERVATIONS

TEST GROUP 0 (0 MG/KG BW/D)

FEMALE#	PUP#	STATUS	DAY	OBSERVATION	POSITION/GRADE
6	1	K	21	NOTHING ABNORMAL DETECTED	
	3	K	21	NOTHING ABNORMAL DETECTED	
	6	K	21	NOTHING ABNORMAL DETECTED	
	7	K	21	NOTHING ABNORMAL DETECTED	
	8	K	21	NOTHING ABNORMAL DETECTED	
	9	K	21	NOTHING ABNORMAL DETECTED	
7	2	K	21	NOTHING ABNORMAL DETECTED	
	3	K	21	NOTHING ABNORMAL DETECTED	
	4	K	21	NOTHING ABNORMAL DETECTED	
	5	K	21	NOTHING ABNORMAL DETECTED	
	8	K	21	NOTHING ABNORMAL DETECTED	
8	1	K	21	NOTHING ABNORMAL DETECTED	
	2	K	21	NOTHING ABNORMAL DETECTED	
	3	K	21	NOTHING ABNORMAL DETECTED	
	5	K	21	NOTHING ABNORMAL DETECTED	
	6	K	21	NOTHING ABNORMAL DETECTED	
	8	K	21	NOTHING ABNORMAL DETECTED	
9	2	K	21	NOTHING ABNORMAL DETECTED	
	3	K	21	NOTHING ABNORMAL DETECTED	
	6	K	21	NOTHING ABNORMAL DETECTED	
	7	K	21	NOTHING ABNORMAL DETECTED	
10	1	K	21	NOTHING ABNORMAL DETECTED	
	3	K	21	NOTHING ABNORMAL DETECTED	
	4	K	21	NOTHING ABNORMAL DETECTED	
	6	K	21	NOTHING ABNORMAL DETECTED	
	7	K	21	NOTHING ABNORMAL DETECTED	
	8	K	21	NOTHING ABNORMAL DETECTED	
	9	K	21	NOTHING ABNORMAL DETECTED	
11	1	K	21	NOTHING ABNORMAL DETECTED	
	2	K	21	NOTHING ABNORMAL DETECTED	
	4	K	21	NOTHING ABNORMAL DETECTED	
	5	K	21	NOTHING ABNORMAL DETECTED	
	7	K	21	NOTHING ABNORMAL DETECTED	
	8	K	21	NOTHING ABNORMAL DETECTED	

POSITION/GRADE CODE: R-RIGHT, L-LEFT, B-BILATE , 1-SLIGHT, 2-MODERATE, 3-MARKED, P-PRESENT
PUP STATUS CODES: K-SCHEDULED SACRIFICE
NUMBER FOLLOWING PUP STATUS = DAY POST PARTUM OF DEATH

PR.NO.60R0375/88R002: REPRODUCTIVE TOX. STUDY TO DETECT EFFECTS
OF MIXED ANTI-ANDROGENIC SUBSTANCES IN RATS; ORAL ADM. (GAVAGE)
INDIVIDUAL PUP NECROPSY OBSERVATIONS

TEST GROUP 0 (0 MG/KG BW/D)

FEMALE#	PUP#	STATUS	DAY	OBSERVATION	POSITION/GRADE
11 (CONTINUED)					
	9	K	21	NOTHING ABNORMAL DETECTED	
	10	K	21	NOTHING ABNORMAL DETECTED	
	11	K	21	NOTHING ABNORMAL DETECTED	
17	2	K	21	NOTHING ABNORMAL DETECTED	
	3	K	21	NOTHING ABNORMAL DETECTED	
	5	K	21	NOTHING ABNORMAL DETECTED	
	6	K	21	NOTHING ABNORMAL DETECTED	
	7	K	21	NOTHING ABNORMAL DETECTED	
18	1	K	21	NOTHING ABNORMAL DETECTED	
	4	K	21	NOTHING ABNORMAL DETECTED	
	5	K	21	NOTHING ABNORMAL DETECTED	
	6	K	21	NOTHING ABNORMAL DETECTED	
	7	K	21	NOTHING ABNORMAL DETECTED	
	9	K	21	NOTHING ABNORMAL DETECTED	
	10	K	21	NOTHING ABNORMAL DETECTED	
19	2	K	21	NOTHING ABNORMAL DETECTED	
	3	K	21	HYDRONEPHROSIS	L
				HYDROURETER	L
				DILATED RENAL PELVIS	R
	5	K	21	NOTHING ABNORMAL DETECTED	
	6	K	21	NOTHING ABNORMAL DETECTED	
	8	K	21	NOTHING ABNORMAL DETECTED	
20	1	K	21	INCISORS SLOPED UPPER AND LOWER	
	4	K	21	NOTHING ABNORMAL DETECTED	
	5	K	21	NOTHING ABNORMAL DETECTED	
	6	K	21	NOTHING ABNORMAL DETECTED	
	7	K	21	NOTHING ABNORMAL DETECTED	
	8	K	21	NOTHING ABNORMAL DETECTED	
	9	K	21	NOTHING ABNORMAL DETECTED	
21	1	K	21	NOTHING ABNORMAL DETECTED	
	2	K	21	NOTHING ABNORMAL DETECTED	
	3	K	21	NOTHING ABNORMAL DETECTED	
	4	K	21	NOTHING ABNORMAL DETECTED	
	6	K	21	NOTHING ABNORMAL DETECTED	

POSITION/GRADE CODE: R-RIGHT, L-LEFT, B-BILATE , 1-SLIGHT, 2-MODERATE, 3-MARKED, P-PRESENT
PUP STATUS CODES: K-SCHEDULED SACRIFICE
NUMBER FOLLOWING PUP STATUS = DAY POST PARTUM OF DEATH

PR.NO.60R0375/88R002: REPRODUCTIVE TOX. STUDY TO DETECT EFFECTS OF MIXED ANTI-ANDROGENIC SUBSTANCES IN RATS; ORAL ADM. (GAVAGE)

INDIVIDUAL PUP NECROPSY OBSERVATIONS

TEST GROUP 0 (0 MG/KG BW/D)

FEMALE#	PUP#	STATUS	DAY	OBSERVATION	POSITION/GRADE
21 (CONTINUED)					
	8	K	21	NOTHING ABNORMAL DETECTED	
	9	K	21	NOTHING ABNORMAL DETECTED	
	10	K	21	NOTHING ABNORMAL DETECTED	
	12	K	21	NOTHING ABNORMAL DETECTED	
22	2	K	21	NOTHING ABNORMAL DETECTED	
	3	K	21	NOTHING ABNORMAL DETECTED	
	5	K	21	NOTHING ABNORMAL DETECTED	
	7	K	21	NOTHING ABNORMAL DETECTED	
	8	K	21	DILATED RENAL PELVIS	R
	9	K	21	NOTHING ABNORMAL DETECTED	
23	1	K	21	NOTHING ABNORMAL DETECTED	
	3	K	21	NOTHING ABNORMAL DETECTED	
	4	S		NOTHING ABNORMAL DETECTED	
	6	K	21	NOTHING ABNORMAL DETECTED	
	7	K	21	NOTHING ABNORMAL DETECTED	
	9	K	21	NOTHING ABNORMAL DETECTED	
	10	K	21	NOTHING ABNORMAL DETECTED	
24	1	K	21	NOTHING ABNORMAL DETECTED	
	2	K	21	NOTHING ABNORMAL DETECTED	
	4	K	21	NOTHING ABNORMAL DETECTED	
	5	K	21	NOTHING ABNORMAL DETECTED	
	8	K	21	NOTHING ABNORMAL DETECTED	
	9	K	21	NOTHING ABNORMAL DETECTED	
	10	K	21	NOTHING ABNORMAL DETECTED	
	11	K	21	NOTHING ABNORMAL DETECTED	
25	2	K	21	NOTHING ABNORMAL DETECTED	
	3	K	21	NOTHING ABNORMAL DETECTED	
	4	K	21	NOTHING ABNORMAL DETECTED	
	6	K	21	NOTHING ABNORMAL DETECTED	
	7	K	21	NOTHING ABNORMAL DETECTED	
	8	K	21	NOTHING ABNORMAL DETECTED	
	10	K	21	NOTHING ABNORMAL DETECTED	
	11	K	21	NOTHING ABNORMAL DETECTED	

POSITION/GRADE CODE: R-RIGHT, L-LEFT, B-BILATE , 1-SLIGHT, 2-MODERATE, 3-MARKED, P-PRESENT
PUP STATUS CODES: S-STILLBORN K-SCHEDULED SACRIFICE
NUMBER FOLLOWING PUP STATUS = DAY POST PARTUM OF DEATH

PR.NO.60R0375/88R002: REPRODUCTIVE TOX. STUDY TO DETECT EFFECTS
OF MIXED ANTI-ANDROGENIC SUBSTANCES IN RATS; ORAL ADM. (GAVAGE)
INDIVIDUAL PUP NECROPSY OBSERVATIONS

TEST GROUP 1 (ADI-MIX)

FEMALE#	PUP#	STATUS	DAY	OBSERVATION	POSITION/GRADE
26	1	K	21	NOTHING ABNORMAL DETECTED	
	4	K	21	NOTHING ABNORMAL DETECTED	
	5	K	21	NOTHING ABNORMAL DETECTED	
	6	K	21	NOTHING ABNORMAL DETECTED	
	8	K	21	DILATED RENAL PELVIS	R
	9	K	21	NOTHING ABNORMAL DETECTED	
	10	K	21	NOTHING ABNORMAL DETECTED	
27	2	K	21	NOTHING ABNORMAL DETECTED	
	3	K	21	NOTHING ABNORMAL DETECTED	
	4	K	21	NOTHING ABNORMAL DETECTED	
	5	K	21	NOTHING ABNORMAL DETECTED	
	7	K	21	NOTHING ABNORMAL DETECTED	
	8	K	21	NOTHING ABNORMAL DETECTED	
	10	K	21	NOTHING ABNORMAL DETECTED	
28	3	K	21	NOTHING ABNORMAL DETECTED	
	4	K	21	NOTHING ABNORMAL DETECTED	
	6	K	21	NOTHING ABNORMAL DETECTED	
	7	K	21	NOTHING ABNORMAL DETECTED	
	8	K	21	NOTHING ABNORMAL DETECTED	
29	1	K	21	NOTHING ABNORMAL DETECTED	
	2	K	21	NOTHING ABNORMAL DETECTED	
	3	K	21	NOTHING ABNORMAL DETECTED	
	4	K	21	NOTHING ABNORMAL DETECTED	
	5	K	21	NOTHING ABNORMAL DETECTED	
	7	K	21	NOTHING ABNORMAL DETECTED	
	9	K	21	NOTHING ABNORMAL DETECTED	
	11	K	21	NOTHING ABNORMAL DETECTED	
30	1	K	21	NOTHING ABNORMAL DETECTED	
	2	K	21	NOTHING ABNORMAL DETECTED	
	3	K	21	NOTHING ABNORMAL DETECTED	
	5	K	21	NOTHING ABNORMAL DETECTED	
	6	K	21	NOTHING ABNORMAL DETECTED	
	7	K	21	NOTHING ABNORMAL DETECTED	
	8	K	21	NOTHING ABNORMAL DETECTED	
	10	K	21	NOTHING ABNORMAL DETECTED	

POSITION/GRADE CODE: R-RIGHT, L-LEFT, B-BILATE , 1-SLIGHT, 2-MODERATE, 3-MARKED, P-PRESENT
PUP STATUS CODES: K-SCHEDULED SACRIFICE
NUMBER FOLLOWING PUP STATUS = DAY POST PARTUM OF DEATH

PR.NO.60R0375/88R002: REPRODUCTIVE TOX. STUDY TO DETECT EFFECTS
OF MIXED ANTI-ANDROGENIC SUBSTANCES IN RATS; ORAL ADM. (GAVAGE)
INDIVIDUAL PUP NECROPSY OBSERVATIONS

TEST GROUP 1 (ADI-MIX)

FEMALE#	PUP#	STATUS	DAY	OBSERVATION	POSITION/GRADE
30 (CONTINUED)					
	11	K	21	NOTHING ABNORMAL DETECTED	
31	2	K	21	NOTHING ABNORMAL DETECTED	
	4	K	21	NOTHING ABNORMAL DETECTED	
	5	K	21	NOTHING ABNORMAL DETECTED	
	6	K	21	NOTHING ABNORMAL DETECTED	
	7	K	21	NOTHING ABNORMAL DETECTED	
	8	K	21	NOTHING ABNORMAL DETECTED	
	9	K	21	NOTHING ABNORMAL DETECTED	
	11	K	21	NOTHING ABNORMAL DETECTED	
	12	K	21	NOTHING ABNORMAL DETECTED	
32	1	K	21	NOTHING ABNORMAL DETECTED	
	3	K	21	NOTHING ABNORMAL DETECTED	
	5	K	21	NOTHING ABNORMAL DETECTED	
	6	K	21	NOTHING ABNORMAL DETECTED	
	7	K	21	NOTHING ABNORMAL DETECTED	
33	3	K	21	NOTHING ABNORMAL DETECTED	
	4	K	21	NOTHING ABNORMAL DETECTED	
	5	K	21	NOTHING ABNORMAL DETECTED	
	7	K	21	NOTHING ABNORMAL DETECTED	
	8	K	21	NOTHING ABNORMAL DETECTED	
	9	K	21	NOTHING ABNORMAL DETECTED	
	10	K	21	NOTHING ABNORMAL DETECTED	
34	1	K	21	NOTHING ABNORMAL DETECTED	
	2	K	21	NOTHING ABNORMAL DETECTED	
	4	K	21	NOTHING ABNORMAL DETECTED	
	6	K	21	NOTHING ABNORMAL DETECTED	
	7	K	21	NOTHING ABNORMAL DETECTED	
	8	K	21	NOTHING ABNORMAL DETECTED	
	10	K	21	NOTHING ABNORMAL DETECTED	
35	1	K	21	NOTHING ABNORMAL DETECTED	
	2	K	21	NOTHING ABNORMAL DETECTED	
	5	K	21	NOTHING ABNORMAL DETECTED	
	6	K	21	NOTHING ABNORMAL DETECTED	
	7	K	21	NOTHING ABNORMAL DETECTED	

POSITION/GRADE CODE: R-RIGHT, L-LEFT, B-BILATE , 1-SLIGHT, 2-MODERATE, 3-MARKED, P-PRESENT
PUP STATUS CODES: K-SCHEDULED SACRIFICE
NUMBER FOLLOWING PUP STATUS = DAY POST PARTUM OF DEATH

PR.NO.60R0375/88R002: REPRODUCTIVE TOX. STUDY TO DETECT EFFECTS
OF MIXED ANTI-ANDROGENIC SUBSTANCES IN RATS; ORAL ADM. (GAVAGE)
INDIVIDUAL PUP NECROPSY OBSERVATIONS

TEST GROUP 1 (ADI-MIX)

FEMALE#	PUP#	STATUS	DAY	OBSERVATION	POSITION/GRADE
36	2	K	21	NOTHING ABNORMAL DETECTED	
	3	K	21	NOTHING ABNORMAL DETECTED	
	5	K	21	NOTHING ABNORMAL DETECTED	
	7	K	21	NOTHING ABNORMAL DETECTED	
	8	K	21	NOTHING ABNORMAL DETECTED	
	9	K	21	NOTHING ABNORMAL DETECTED	
	10	K	21	NOTHING ABNORMAL DETECTED	
37	1	K	21	NOTHING ABNORMAL DETECTED	
	2	K	21	NOTHING ABNORMAL DETECTED	
	3	K	21	NOTHING ABNORMAL DETECTED	
	4	K	21	NOTHING ABNORMAL DETECTED	
	6	K	21	NOTHING ABNORMAL DETECTED	
	8	K	21	NOTHING ABNORMAL DETECTED	
	10	K	21	NOTHING ABNORMAL DETECTED	
43	1	K	21	NOTHING ABNORMAL DETECTED	
	2	K	21	NOTHING ABNORMAL DETECTED	
	3	K	21	NOTHING ABNORMAL DETECTED	
	5	K	21	INCISORS SLOPED UPPER	
	7	K	21	NOTHING ABNORMAL DETECTED	
	8	K	21	NOTHING ABNORMAL DETECTED	
	9	K	21	NOTHING ABNORMAL DETECTED	
	11	K	21	NOTHING ABNORMAL DETECTED	
	12	K	21	NOTHING ABNORMAL DETECTED	
44	1	S		NOTHING ABNORMAL DETECTED	
	3	K	21	NOTHING ABNORMAL DETECTED	
	4	K	21	NOTHING ABNORMAL DETECTED	
	6	K	21	NOTHING ABNORMAL DETECTED	
	7	K	21	NOTHING ABNORMAL DETECTED	
	9	K	21	NOTHING ABNORMAL DETECTED	
	10	K	21	NOTHING ABNORMAL DETECTED	
45	1	K	21	NOTHING ABNORMAL DETECTED	
	2	K	21	NOTHING ABNORMAL DETECTED	
	5	K	21	NOTHING ABNORMAL DETECTED	
	7	K	21	NOTHING ABNORMAL DETECTED	

POSITION/GRADE CODE: R-RIGHT, L-LEFT, B-BILATE , 1-SLIGHT, 2-MODERATE, 3-MARKED, P-PRESENT
PUP STATUS CODES: S-STILLBORN K-SCHEDULED SACRIFICE
NUMBER FOLLOWING PUP STATUS = DAY POST PARTUM OF DEATH

PR.NO.60R0375/88R002: REPRODUCTIVE TOX. STUDY TO DETECT EFFECTS
OF MIXED ANTI-ANDROGENIC SUBSTANCES IN RATS; ORAL ADM. (GAVAGE)
INDIVIDUAL PUP NECROPSY OBSERVATIONS

TEST GROUP 1 (ADI-MIX)

FEMALE#	PUP#	STATUS	DAY	OBSERVATION	POSITION/GRADE
45 (CONTINUED)					
	8	K	21	NOTHING ABNORMAL DETECTED	
	9	K	21	NOTHING ABNORMAL DETECTED	
46	2	K	21	NOTHING ABNORMAL DETECTED	
	3	K	21	NOTHING ABNORMAL DETECTED	
	4	K	21	NOTHING ABNORMAL DETECTED	
	5	K	21	NOTHING ABNORMAL DETECTED	
	6	K	21	NOTHING ABNORMAL DETECTED	
	8	K	21	NOTHING ABNORMAL DETECTED	
	9	K	21	NOTHING ABNORMAL DETECTED	
	10	K	21	NOTHING ABNORMAL DETECTED	
	11	K	21	NOTHING ABNORMAL DETECTED	
	12	K	21	NOTHING ABNORMAL DETECTED	
47	1	K	21	NOTHING ABNORMAL DETECTED	
	4	K	21	NOTHING ABNORMAL DETECTED	
	6	K	21	NOTHING ABNORMAL DETECTED	
	7	K	21	NOTHING ABNORMAL DETECTED	
48	2	K	21	NOTHING ABNORMAL DETECTED	
	3	K	21	NOTHING ABNORMAL DETECTED	
	4	K	21	NOTHING ABNORMAL DETECTED	
	5	K	21	NOTHING ABNORMAL DETECTED	
	7	K	21	NOTHING ABNORMAL DETECTED	
	9	K	21	NOTHING ABNORMAL DETECTED	
49	1	K	21	NOTHING ABNORMAL DETECTED	
	3	K	21	NOTHING ABNORMAL DETECTED	
	5	K	21	NOTHING ABNORMAL DETECTED	
	6	K	21	NOTHING ABNORMAL DETECTED	
	7	K	21	NOTHING ABNORMAL DETECTED	
	8	K	21	NOTHING ABNORMAL DETECTED	
	9	K	21	NOTHING ABNORMAL DETECTED	
	11	K	21	NOTHING ABNORMAL DETECTED	
	12	K	21	NOTHING ABNORMAL DETECTED	
50	3	K	21	NOTHING ABNORMAL DETECTED	
	4	K	21	NOTHING ABNORMAL DETECTED	
	5	K	21	NOTHING ABNORMAL DETECTED	
	6	K	21	NOTHING ABNORMAL DETECTED	

POSITION/GRADE CODE: R-RIGHT, L-LEFT, B-BILATE , 1-SLIGHT, 2-MODERATE, 3-MARKED, P-PRESENT
PUP STATUS CODES: K-SCHEDULED SACRIFICE
NUMBER FOLLOWING PUP STATUS = DAY POST PARTUM OF DEATH

PR.NO.60R0375/88R002: REPRODUCTIVE TOX. STUDY TO DETECT EFFECTS OF MIXED ANTI-ANDROGENIC SUBSTANCES IN RATS; ORAL ADM. (GAVAGE)

INDIVIDUAL PUP NECROPSY OBSERVATIONS

TEST GROUP 1 (ADI-MIX)

FEMALE#	PUP#	STATUS	DAY	OBSERVATION	POSITION/GRADE
50 (CONTINUED)					
	7	K	21	NOTHING ABNORMAL DETECTED	

POSITION/GRADE CODE: R-RIGHT, L-LEFT, B-BILATE , 1-SLIGHT, 2-MODERATE, 3-MARKED, P-PRESENT
PUP STATUS CODES: K-SCHEDULED SACRIFICE
NUMBER FOLLOWING PUP STATUS = DAY POST PARTUM OF DEATH

PR.NO.60R0375/88R002: REPRODUCTIVE TOX. STUDY TO DETECT EFFECTS
OF MIXED ANTI-ANDROGENIC SUBSTANCES IN RATS; ORAL ADM. (GAVAGE)
INDIVIDUAL PUP NECROPSY OBSERVATIONS

TEST GROUP 2 (NOAEL-MIX)

FEMALE#	PUP#	STATUS	DAY	OBSERVATION	POSITION/GRADE
51	1	K	21	NOTHING ABNORMAL DETECTED	
	3	K	21	NOTHING ABNORMAL DETECTED	
	5	K	21	NOTHING ABNORMAL DETECTED	
	6	K	21	NOTHING ABNORMAL DETECTED	
	8	K	21	NOTHING ABNORMAL DETECTED	
	9	K	21	NOTHING ABNORMAL DETECTED	
	10	K	21	NOTHING ABNORMAL DETECTED	
52	1	K	21	NOTHING ABNORMAL DETECTED	
	2	K	21	NOTHING ABNORMAL DETECTED	
	4	K	21	NOTHING ABNORMAL DETECTED	
	5	K	21	NOTHING ABNORMAL DETECTED	
	7	K	21	NOTHING ABNORMAL DETECTED	
	8	K	21	NOTHING ABNORMAL DETECTED	
	9	K	21	NOTHING ABNORMAL DETECTED	
	11	K	21	NOTHING ABNORMAL DETECTED	
53	3	K	21	NOTHING ABNORMAL DETECTED	
	4	K	21	NOTHING ABNORMAL DETECTED	
	5	K	21	NOTHING ABNORMAL DETECTED	
	6	S		NOTHING ABNORMAL DETECTED	
	7	K	21	NOTHING ABNORMAL DETECTED	
	9	K	21	NOTHING ABNORMAL DETECTED	
54	1	K	21	NOTHING ABNORMAL DETECTED	
	2	K	21	NOTHING ABNORMAL DETECTED	
	4	K	21	NOTHING ABNORMAL DETECTED	
	6	K	21	NOTHING ABNORMAL DETECTED	
	8	K	21	NOTHING ABNORMAL DETECTED	
	9	K	21	NOTHING ABNORMAL DETECTED	
	10	K	21	NOTHING ABNORMAL DETECTED	
55	1	K	21	NOTHING ABNORMAL DETECTED	
	2	K	21	NOTHING ABNORMAL DETECTED	
	3	K	21	NOTHING ABNORMAL DETECTED	
	5	K	21	NOTHING ABNORMAL DETECTED	
	6	K	21	NOTHING ABNORMAL DETECTED	
	7	K	21	NOTHING ABNORMAL DETECTED	
	9	K	21	NOTHING ABNORMAL DETECTED	

POSITION/GRADE CODE: R-RIGHT, L-LEFT, B-BILATE , 1-SLIGHT, 2-MODERATE, 3-MARKED, P-PRESENT
PUP STATUS CODES: S-STILLBORN K-SCHEDULED SACRIFICE
NUMBER FOLLOWING PUP STATUS = DAY POST PARTUM OF DEATH

PR.NO.60R0375/88R002: REPRODUCTIVE TOX. STUDY TO DETECT EFFECTS
OF MIXED ANTI-ANDROGENIC SUBSTANCES IN RATS; ORAL ADM. (GAVAGE)
INDIVIDUAL PUP NECROPSY OBSERVATIONS

TEST GROUP 2 (NOAEL-MIX)

FEMALE#	PUP#	STATUS	DAY	OBSERVATION	POSITION/GRADE
55 (CONTINUED)					
	11	K	21	NOTHING ABNORMAL DETECTED	
	12	K	21	NOTHING ABNORMAL DETECTED	
56	3	D	1	POST MORTEM AUTOLYSIS	
	4	D	1	NOTHING ABNORMAL DETECTED	
	5	D	1	POST MORTEM AUTOLYSIS	
	7	K	21	NOTHING ABNORMAL DETECTED	
	8	D	2	EMPTY STOMACH	
	10	D	1	NOTHING ABNORMAL DETECTED	
	11	D	1	POST MORTEM AUTOLYSIS	
	12	D	1	NOTHING ABNORMAL DETECTED	
	14	S		NOTHING ABNORMAL DETECTED	
57	2	K	21	NOTHING ABNORMAL DETECTED	
	3	K	21	NOTHING ABNORMAL DETECTED	
	4	K	21	NOTHING ABNORMAL DETECTED	
	6	K	21	NOTHING ABNORMAL DETECTED	
	8	K	21	NOTHING ABNORMAL DETECTED	
	9	K	21	NOTHING ABNORMAL DETECTED	
	10	K	21	NOTHING ABNORMAL DETECTED	
	11	K	21	NOTHING ABNORMAL DETECTED	
58	1	K	21	NOTHING ABNORMAL DETECTED	
	2	K	21	NOTHING ABNORMAL DETECTED	
	5	K	21	NOTHING ABNORMAL DETECTED	
	7	K	21	NOTHING ABNORMAL DETECTED	
	8	K	21	NOTHING ABNORMAL DETECTED	
59	1	K	21	NOTHING ABNORMAL DETECTED	
	2	K	21	NOTHING ABNORMAL DETECTED	
	4	K	21	NOTHING ABNORMAL DETECTED	
	6	K	21	NOTHING ABNORMAL DETECTED	
	8	K	21	NOTHING ABNORMAL DETECTED	
	9	K	21	NOTHING ABNORMAL DETECTED	
60	2	K	21	NOTHING ABNORMAL DETECTED	
	3	K	21	NOTHING ABNORMAL DETECTED	
	4	K	21	NOTHING ABNORMAL DETECTED	
	5	K	21	NOTHING ABNORMAL DETECTED	
	6	K	21	NOTHING ABNORMAL DETECTED	

POSITION/GRADE CODE: R-RIGHT, L-LEFT, B-BILATE , 1-SLIGHT, 2-MODERATE, 3-MARKED, P-PRESENT
PUP STATUS CODES: S-STILLBORN D-DIED K-SCHEDULED SACRIFICE
NUMBER FOLLOWING PUP STATUS = DAY POST PARTUM OF DEATH

PR.NO.60R0375/88R002: REPRODUCTIVE TOX. STUDY TO DETECT EFFECTS
OF MIXED ANTI-ANDROGENIC SUBSTANCES IN RATS; ORAL ADM. (GAVAGE)
INDIVIDUAL PUP NECROPSY OBSERVATIONS

TEST GROUP 2 (NOAEL-MIX)

FEMALE#	PUP#	STATUS	DAY	OBSERVATION	POSITION/GRADE
60 (CONTINUED)					
	7	K	21	NOTHING ABNORMAL DETECTED	
	9	K	21	NOTHING ABNORMAL DETECTED	
	10	K	21	NOTHING ABNORMAL DETECTED	
	12	K	21	NOTHING ABNORMAL DETECTED	
	13	K	21	NOTHING ABNORMAL DETECTED	
61	1	K	21	NOTHING ABNORMAL DETECTED	
	3	K	21	NOTHING ABNORMAL DETECTED	
	4	K	21	NOTHING ABNORMAL DETECTED	
	6	K	21	NOTHING ABNORMAL DETECTED	
	7	K	21	NOTHING ABNORMAL DETECTED	
	8	K	21	NOTHING ABNORMAL DETECTED	
62	1	K	21	NOTHING ABNORMAL DETECTED	
	2	K	21	NOTHING ABNORMAL DETECTED	
	5	K	21	NOTHING ABNORMAL DETECTED	
	6	K	21	NOTHING ABNORMAL DETECTED	
	8	K	21	NOTHING ABNORMAL DETECTED	
68	2	K	21	NOTHING ABNORMAL DETECTED	
	3	K	21	NOTHING ABNORMAL DETECTED	
	4	K	21	NOTHING ABNORMAL DETECTED	
	5	K	21	NOTHING ABNORMAL DETECTED	
	7	K	21	NOTHING ABNORMAL DETECTED	
	8	K	21	NOTHING ABNORMAL DETECTED	
	9	K	21	NOTHING ABNORMAL DETECTED	
69	1	K	21	NOTHING ABNORMAL DETECTED	
	2	K	21	NOTHING ABNORMAL DETECTED	
	3	K	21	NOTHING ABNORMAL DETECTED	
	4	K	21	NOTHING ABNORMAL DETECTED	
	6	K	21	NOTHING ABNORMAL DETECTED	
	7	K	21	NOTHING ABNORMAL DETECTED	
70	2	K	21	NOTHING ABNORMAL DETECTED	
	3	K	21	NOTHING ABNORMAL DETECTED	
	5	K	21	NOTHING ABNORMAL DETECTED	
71	1	K	21	NOTHING ABNORMAL DETECTED	
	2	K	21	NOTHING ABNORMAL DETECTED	

POSITION/GRADE CODE: R-RIGHT, L-LEFT, B-BILATE , 1-SLIGHT, 2-MODERATE, 3-MARKED, P-PRESENT
PUP STATUS CODES: K-SCHEDULED SACRIFICE
NUMBER FOLLOWING PUP STATUS = DAY POST PARTUM OF DEATH

PR.NO.60R0375/88R002: REPRODUCTIVE TOX. STUDY TO DETECT EFFECTS
OF MIXED ANTI-ANDROGENIC SUBSTANCES IN RATS; ORAL ADM. (GAVAGE)
INDIVIDUAL PUP NECROPSY OBSERVATIONS

TEST GROUP 2 (NOAEL-MIX)

FEMALE#	PUP#	STATUS	DAY	OBSERVATION	POSITION/GRADE
71 (CONTINUED)					
	4	K	21	NOTHING ABNORMAL DETECTED	
	5	K	21	NOTHING ABNORMAL DETECTED	
	7	K	21	NOTHING ABNORMAL DETECTED	
	8	K	21	NOTHING ABNORMAL DETECTED	
72	2	K	21	NOTHING ABNORMAL DETECTED	
	3	K	21	NOTHING ABNORMAL DETECTED	
	4	K	21	NOTHING ABNORMAL DETECTED	
	6	K	21	NOTHING ABNORMAL DETECTED	
	7	K	21	NOTHING ABNORMAL DETECTED	
	8	K	21	NOTHING ABNORMAL DETECTED	
	9	K	21	NOTHING ABNORMAL DETECTED	
	10	K	21	NOTHING ABNORMAL DETECTED	
	12	K	21	NOTHING ABNORMAL DETECTED	
73	1	K	21	NOTHING ABNORMAL DETECTED	
	3	K	21	NOTHING ABNORMAL DETECTED	
	4	K	21	NOTHING ABNORMAL DETECTED	
	5	K	21	NOTHING ABNORMAL DETECTED	
	8	K	21	NOTHING ABNORMAL DETECTED	
	10	K	21	NOTHING ABNORMAL DETECTED	
74	2	K	21	NOTHING ABNORMAL DETECTED	
	3	K	21	NOTHING ABNORMAL DETECTED	
	5	K	21	NOTHING ABNORMAL DETECTED	
	7	K	21	NOTHING ABNORMAL DETECTED	
	9	K	21	NOTHING ABNORMAL DETECTED	
	10	K	21	NOTHING ABNORMAL DETECTED	
	11	K	21	NOTHING ABNORMAL DETECTED	
75	1	K	21	NOTHING ABNORMAL DETECTED	
	2	K	21	NOTHING ABNORMAL DETECTED	
	4	K	21	NOTHING ABNORMAL DETECTED	
	5	K	21	DIAPHRAGMATIC HERNIA	
	8	K	21	NOTHING ABNORMAL DETECTED	
	9	K	21	NOTHING ABNORMAL DETECTED	

POSITION/GRADE CODE: R-RIGHT, L-LEFT, B-BILATE , 1-SLIGHT, 2-MODERATE, 3-MARKED, P-PRESENT
PUP STATUS CODES: K-SCHEDULED SACRIFICE
NUMBER FOLLOWING PUP STATUS = DAY POST PARTUM OF DEATH

PR.NO.60R0375/88R002: REPRODUCTIVE TOX. STUDY TO DETECT EFFECTS
OF MIXED ANTI-ANDROGENIC SUBSTANCES IN RATS; ORAL ADM. (GAVAGE)
INDIVIDUAL PUP NECROPSY OBSERVATIONS

TEST GROUP 3 (LOAEL-MIX)

FEMALE#	PUP#	STATUS	DAY	OBSERVATION	POSITION/GRADE
77	1	K	21	NOTHING ABNORMAL DETECTED	
	2	K	21	NOTHING ABNORMAL DETECTED	
	4	K	21	NOTHING ABNORMAL DETECTED	
	6	K	21	NOTHING ABNORMAL DETECTED	
	7	K	21	NOTHING ABNORMAL DETECTED	
78	1	K	21	INCISORS SLOPED	
				UPPER	
	3	K	21	NOTHING ABNORMAL DETECTED	
	4	K	21	NOTHING ABNORMAL DETECTED	
	5	K	21	NOTHING ABNORMAL DETECTED	
	6	K	21	NOTHING ABNORMAL DETECTED	
	8	K	21	NOTHING ABNORMAL DETECTED	
	10	S		NOTHING ABNORMAL DETECTED	
79	1	S		NOTHING ABNORMAL DETECTED	
	2	S		NOTHING ABNORMAL DETECTED	
	3	S		POST MORTEM AUTOLYSIS	
	4	S		POST MORTEM AUTOLYSIS	
	5	S		POST MORTEM AUTOLYSIS	
80	2	K	21	NOTHING ABNORMAL DETECTED	
	4	S		POST MORTEM AUTOLYSIS	
	5	S		POST MORTEM AUTOLYSIS	
	8	S		NOTHING ABNORMAL DETECTED	
	9	S		POST MORTEM AUTOLYSIS	
81	1	S		NOTHING ABNORMAL DETECTED	
	2	S		NOTHING ABNORMAL DETECTED	
	3	S		POST MORTEM AUTOLYSIS	
	4	S		NOTHING ABNORMAL DETECTED	
	5	S		NOTHING ABNORMAL DETECTED	
	6	S		NOTHING ABNORMAL DETECTED	
	7	S		NOTHING ABNORMAL DETECTED	
	8	S		POST MORTEM AUTOLYSIS	
	9	S		POST MORTEM AUTOLYSIS	
	10	S		NOTHING ABNORMAL DETECTED	
	11	S		NOTHING ABNORMAL DETECTED	

POSITION/GRADE CODE: R-RIGHT, L-LEFT, B-BILATE , 1-SLIGHT, 2-MODERATE, 3-MARKED, P-PRESENT
PUP STATUS CODES: S-STILLBORN K-SCHEDULED SACRIFICE
NUMBER FOLLOWING PUP STATUS = DAY POST PARTUM OF DEATH

PR.NO.60R0375/88R002: REPRODUCTIVE TOX. STUDY TO DETECT EFFECTS
OF MIXED ANTI-ANDROGENIC SUBSTANCES IN RATS; ORAL ADM. (GAVAGE)
INDIVIDUAL PUP NECROPSY OBSERVATIONS

TEST GROUP 3 (LOAEL-MIX)

FEMALE#	PUP#	STATUS	DAY	OBSERVATION	POSITION/GRADE
82	1	K	21	NOTHING ABNORMAL DETECTED	
	3	K	21	NOTHING ABNORMAL DETECTED	
	4	K	21	NOTHING ABNORMAL DETECTED	
	5	K	21	NOTHING ABNORMAL DETECTED	
	7	K	21	NOTHING ABNORMAL DETECTED	
	9	K	21	NOTHING ABNORMAL DETECTED	
	10	K	21	NOTHING ABNORMAL DETECTED	
	11	K	21	NOTHING ABNORMAL DETECTED	
83	1	K	21	NOTHING ABNORMAL DETECTED	
	2	K	21	NOTHING ABNORMAL DETECTED	
	3	K	21	NOTHING ABNORMAL DETECTED	
	5	S		POST MORTEM AUTOLYSIS	
	6	K	21	NOTHING ABNORMAL DETECTED	
	7	K	21	NOTHING ABNORMAL DETECTED	
	8	K	21	NOTHING ABNORMAL DETECTED	
84	2	K	21	NOTHING ABNORMAL DETECTED	
	4	K	21	NOTHING ABNORMAL DETECTED	
	5	K	21	NOTHING ABNORMAL DETECTED	
	6	K	21	NOTHING ABNORMAL DETECTED	
85	3	S		NOTHING ABNORMAL DETECTED	
	4	S		NOTHING ABNORMAL DETECTED	
	6	K	21	NOTHING ABNORMAL DETECTED	
	7	K	21	NOTHING ABNORMAL DETECTED	
	8	K	21	NOTHING ABNORMAL DETECTED	
86	1	K	21	NOTHING ABNORMAL DETECTED	
	3	K	21	NOTHING ABNORMAL DETECTED	
	5	K	21	NOTHING ABNORMAL DETECTED	
	7	K	21	NOTHING ABNORMAL DETECTED	
	8	K	21	NOTHING ABNORMAL DETECTED	
	9	K	21	NOTHING ABNORMAL DETECTED	
	10	K	21	NOTHING ABNORMAL DETECTED	
	11	K	21	NOTHING ABNORMAL DETECTED	
87	2	K	21	NOTHING ABNORMAL DETECTED	
	3	K	21	NOTHING ABNORMAL DETECTED	

POSITION/GRADE CODE: R-RIGHT, L-LEFT, B-BILATE , 1-SLIGHT, 2-MODERATE, 3-MARKED, P-PRESENT
PUP STATUS CODES: S-STILLBORN K-SCHEDULED SACRIFICE
NUMBER FOLLOWING PUP STATUS = DAY POST PARTUM OF DEATH

PR.NO.60R0375/88R002: REPRODUCTIVE TOX. STUDY TO DETECT EFFECTS
OF MIXED ANTI-ANDROGENIC SUBSTANCES IN RATS; ORAL ADM. (GAVAGE)
INDIVIDUAL PUP NECROPSY OBSERVATIONS

TEST GROUP 3 (LOAEL-MIX)

FEMALE#	PUP#	STATUS	DAY	OBSERVATION	POSITION/GRADE
87 (CONTINUED)					
	4	K	21	NOTHING ABNORMAL DETECTED	
	6	K	21	NOTHING ABNORMAL DETECTED	
	8	K	21	NOTHING ABNORMAL DETECTED	
	9	K	21	NOTHING ABNORMAL DETECTED	
	11	K	21	NOTHING ABNORMAL DETECTED	
	12	K	21	NOTHING ABNORMAL DETECTED	
93	1	K	21	NOTHING ABNORMAL DETECTED	
	2	K	21	NOTHING ABNORMAL DETECTED	
	4	K	21	NOTHING ABNORMAL DETECTED	
	7	K	21	DILATED RENAL PELVIS	B
94	1	K	21	NOTHING ABNORMAL DETECTED	
	2	K	21	NOTHING ABNORMAL DETECTED	
	3	K	21	NOTHING ABNORMAL DETECTED	
	5	K	21	NOTHING ABNORMAL DETECTED	
	6	K	21	NOTHING ABNORMAL DETECTED	
	10	K	21	NOTHING ABNORMAL DETECTED	
95	2	K	21	NOTHING ABNORMAL DETECTED	
	3	K	21	NOTHING ABNORMAL DETECTED	
	4	K	21	NOTHING ABNORMAL DETECTED	
	5	K	21	NOTHING ABNORMAL DETECTED	
	6	K	21	NOTHING ABNORMAL DETECTED	
	8	K	21	NOTHING ABNORMAL DETECTED	
	9	K	21	INFARCT OF LIVER	
				LOBUS SINISTER ACCESSORIUS	
96	1	K	21	NOTHING ABNORMAL DETECTED	
	2	K	21	NOTHING ABNORMAL DETECTED	
	4	K	21	NOTHING ABNORMAL DETECTED	
	7	K	21	NOTHING ABNORMAL DETECTED	
97	1	K	21	NOTHING ABNORMAL DETECTED	
	2	K	21	NOTHING ABNORMAL DETECTED	
	3	K	21	NOTHING ABNORMAL DETECTED	
	5	K	21	NOTHING ABNORMAL DETECTED	
	6	K	21	NOTHING ABNORMAL DETECTED	
	8	K	21	NOTHING ABNORMAL DETECTED	

POSITION/GRADE CODE: R-RIGHT, L-LEFT, B-BILATE , 1-SLIGHT, 2-MODERATE, 3-MARKED, P-PRESENT
PUP STATUS CODES: K-SCHEDULED SACRIFICE
NUMBER FOLLOWING PUP STATUS = DAY POST PARTUM OF DEATH

PR.NO.60R0375/88R002: REPRODUCTIVE TOX. STUDY TO DETECT EFFECTS
OF MIXED ANTI-ANDROGENIC SUBSTANCES IN RATS; ORAL ADM. (GAVAGE)
INDIVIDUAL PUP NECROPSY OBSERVATIONS

TEST GROUP 3 (LOAEL-MIX)

FEMALE#	PUP#	STATUS	DAY	OBSERVATION	POSITION/GRADE
98	1	K	21	NOTHING ABNORMAL DETECTED	
	4	K	21	NOTHING ABNORMAL DETECTED	
	7	K	21	NOTHING ABNORMAL DETECTED	
	8	K	21	NOTHING ABNORMAL DETECTED	
	9	K	21	NOTHING ABNORMAL DETECTED	
	11	S		NOTHING ABNORMAL DETECTED	
99	4	K	21	NOTHING ABNORMAL DETECTED	
	5	K	21	NOTHING ABNORMAL DETECTED	
	6	S		NOTHING ABNORMAL DETECTED	
	9	K	21	NOTHING ABNORMAL DETECTED	
	10	K	21	NOTHING ABNORMAL DETECTED	
100	2	K	21	NOTHING ABNORMAL DETECTED	
	3	K	21	NOTHING ABNORMAL DETECTED	
	6	K	21	NOTHING ABNORMAL DETECTED	
	7	K	21	NOTHING ABNORMAL DETECTED	
	8	K	21	NOTHING ABNORMAL DETECTED	

POSITION/GRADE CODE: R-RIGHT, L-LEFT, B-BILATE , 1-SLIGHT, 2-MODERATE, 3-MARKED, P-PRESENT
PUP STATUS CODES: S-STILLBORN K-SCHEDULED SACRIFICE
NUMBER FOLLOWING PUP STATUS = DAY POST PARTUM OF DEATH

PR.NO.60R0375/88R002: REPRODUCTIVE TOX. STUDY TO DETECT EFFECTS OF MIXED ANTI-ANDROGENIC SUBSTANCES IN RATS; ORAL ADM. (GAVAGE)

INDIVIDUAL PUP NECROPSY OBSERVATIONS

TEST GROUP 4(0.00025 MG/KG BW/D)

FEMALE#	PUP#	STATUS	DAY	OBSERVATION	POSITION/GRADE
101	1	K	21	NOTHING ABNORMAL DETECTED	
	3	K	21	NOTHING ABNORMAL DETECTED	
	4	K	21	NOTHING ABNORMAL DETECTED	
	5	K	21	NOTHING ABNORMAL DETECTED	
	7	K	21	NOTHING ABNORMAL DETECTED	
	9	K	21	NOTHING ABNORMAL DETECTED	
102	1	K	21	NOTHING ABNORMAL DETECTED	
	2	K	21	NOTHING ABNORMAL DETECTED	
	4	K	21	NOTHING ABNORMAL DETECTED	
	6	K	21	NOTHING ABNORMAL DETECTED	
	7	K	21	NOTHING ABNORMAL DETECTED	
	8	K	21	NOTHING ABNORMAL DETECTED	
	10	K	21	NOTHING ABNORMAL DETECTED	
103	3	K	21	NOTHING ABNORMAL DETECTED	
	5	K	21	NOTHING ABNORMAL DETECTED	
	6	K	21	NOTHING ABNORMAL DETECTED	
	8	K	21	NOTHING ABNORMAL DETECTED	
	9	K	21	NOTHING ABNORMAL DETECTED	
104	2	K	21	NOTHING ABNORMAL DETECTED	
	3	K	21	NOTHING ABNORMAL DETECTED	
	4	K	21	NOTHING ABNORMAL DETECTED	
	5	K	21	NOTHING ABNORMAL DETECTED	
	6	K	21	NOTHING ABNORMAL DETECTED	
	7	K	21	NOTHING ABNORMAL DETECTED	
	9	K	21	NOTHING ABNORMAL DETECTED	
	10	K	21	NOTHING ABNORMAL DETECTED	
105	1	K	21	NOTHING ABNORMAL DETECTED	
	2	K	21	NOTHING ABNORMAL DETECTED	
	5	K	21	NOTHING ABNORMAL DETECTED	
	7	K	21	NOTHING ABNORMAL DETECTED	
106	1	K	21	NOTHING ABNORMAL DETECTED	
	3	K	21	NOTHING ABNORMAL DETECTED	
	4	K	21	NOTHING ABNORMAL DETECTED	
	5	K	21	NOTHING ABNORMAL DETECTED	

POSITION/GRADE CODE: R-RIGHT, L-LEFT, B-BILATE , 1-SLIGHT, 2-MODERATE, 3-MARKED, P-PRESENT
PUP STATUS CODES: K-SCHEDULED SACRIFICE
NUMBER FOLLOWING PUP STATUS = DAY POST PARTUM OF DEATH

PR.NO.60R0375/88R002: REPRODUCTIVE TOX. STUDY TO DETECT EFFECTS
OF MIXED ANTI-ANDROGENIC SUBSTANCES IN RATS; ORAL ADM. (GAVAGE)
INDIVIDUAL PUP NECROPSY OBSERVATIONS

TEST GROUP 4(0.00025 MG/KG BW/D)

FEMALE#	PUP#	STATUS	DAY	OBSERVATION	POSITION/GRADE
106 (CONTINUED)					
	6	K	21	NOTHING ABNORMAL DETECTED	
	9	K	21	NOTHING ABNORMAL DETECTED	
107	2	K	21	NOTHING ABNORMAL DETECTED	
	3	K	21	NOTHING ABNORMAL DETECTED	
	4	K	21	NOTHING ABNORMAL DETECTED	
	7	K	21	NOTHING ABNORMAL DETECTED	
	8	K	21	NOTHING ABNORMAL DETECTED	
	9	K	21	NOTHING ABNORMAL DETECTED	
	10	K	21	NOTHING ABNORMAL DETECTED	
108	1	K	21	NOTHING ABNORMAL DETECTED	
	2	K	21	NOTHING ABNORMAL DETECTED	
	4	K	21	NOTHING ABNORMAL DETECTED	
	5	K	21	NOTHING ABNORMAL DETECTED	
	6	K	21	NOTHING ABNORMAL DETECTED	
	9	K	21	NOTHING ABNORMAL DETECTED	
109	1	K	21	NOTHING ABNORMAL DETECTED	
	2	K	21	NOTHING ABNORMAL DETECTED	
	4	K	21	NOTHING ABNORMAL DETECTED	
	6	K	21	NOTHING ABNORMAL DETECTED	
	7	K	21	NOTHING ABNORMAL DETECTED	
	8	K	21	NOTHING ABNORMAL DETECTED	
110	1	D	1	NOTHING ABNORMAL DETECTED	
111	1	K	21	NOTHING ABNORMAL DETECTED	
	2	K	21	NOTHING ABNORMAL DETECTED	
	4	K	21	NOTHING ABNORMAL DETECTED	
	5	K	21	NOTHING ABNORMAL DETECTED	
	7	K	21	NOTHING ABNORMAL DETECTED	
	8	K	21	NOTHING ABNORMAL DETECTED	
	10	K	21	NOTHING ABNORMAL DETECTED	
117	1	K	21	NOTHING ABNORMAL DETECTED	
	3	K	21	NOTHING ABNORMAL DETECTED	
	4	K	21	NOTHING ABNORMAL DETECTED	
	5	K	21	NOTHING ABNORMAL DETECTED	
	6	K	21	NOTHING ABNORMAL DETECTED	

POSITION/GRADE CODE: R-RIGHT, L-LEFT, B-BILATE , 1-SLIGHT, 2-MODERATE, 3-MARKED, P-PRESENT
PUP STATUS CODES: D-DIED K-SCHEDULED SACRIFICE
NUMBER FOLLOWING PUP STATUS = DAY POST PARTUM OF DEATH

PR.NO.60R0375/88R002: REPRODUCTIVE TOX. STUDY TO DETECT EFFECTS
OF MIXED ANTI-ANDROGENIC SUBSTANCES IN RATS; ORAL ADM. (GAVAGE)
INDIVIDUAL PUP NECROPSY OBSERVATIONS

TEST GROUP 4(0.00025 MG/KG BW/D)

FEMALE#	PUP#	STATUS	DAY	OBSERVATION	POSITION/GRADE
117 (CONTINUED)					
	9	K	21	NOTHING ABNORMAL DETECTED	
118	2	K	21	NOTHING ABNORMAL DETECTED	
	3	K	21	NOTHING ABNORMAL DETECTED	
	4	K	21	NOTHING ABNORMAL DETECTED	
	6	K	21	NOTHING ABNORMAL DETECTED	
	7	K	21	NOTHING ABNORMAL DETECTED	
	8	K	21	NOTHING ABNORMAL DETECTED	
	9	K	21	NOTHING ABNORMAL DETECTED	
	10	K	21	NOTHING ABNORMAL DETECTED	
	12	K	21	NOTHING ABNORMAL DETECTED	
119	3	K	21	NOTHING ABNORMAL DETECTED	
	4	S		NOTHING ABNORMAL DETECTED	
	6	K	21	NOTHING ABNORMAL DETECTED	
	8	K	21	NOTHING ABNORMAL DETECTED	
	9	K	21	NOTHING ABNORMAL DETECTED	
120	1	K	21	NOTHING ABNORMAL DETECTED	
	2	K	21	NOTHING ABNORMAL DETECTED	
	4	S		NOTHING ABNORMAL DETECTED	
	8	S		POST MORTEM AUTOLYSIS	
	9	S		NOTHING ABNORMAL DETECTED	
	10	S		PARTLY CANNIBALIZED	
121	3	K	21	NOTHING ABNORMAL DETECTED	
	4	K	21	NOTHING ABNORMAL DETECTED	
	5	K	21	NOTHING ABNORMAL DETECTED	
	6	K	21	NOTHING ABNORMAL DETECTED	
	7	K	21	NOTHING ABNORMAL DETECTED	
	8	K	21	NOTHING ABNORMAL DETECTED	
	10	K	21	NOTHING ABNORMAL DETECTED	
	11	K	21	NOTHING ABNORMAL DETECTED	
	12	K	21	NOTHING ABNORMAL DETECTED	
123	2	K	21	NOTHING ABNORMAL DETECTED	
	3	K	21	NOTHING ABNORMAL DETECTED	
	5	K	21	NOTHING ABNORMAL DETECTED	
	7	K	21	NOTHING ABNORMAL DETECTED	
	8	K	21	NOTHING ABNORMAL DETECTED	

POSITION/GRADE CODE: R-RIGHT, L-LEFT, B-BILATE , 1-SLIGHT, 2-MODERATE, 3-MARKED, P-PRESENT
PUP STATUS CODES: S-STILLBORN K-SCHEDULED SACRIFICE
NUMBER FOLLOWING PUP STATUS = DAY POST PARTUM OF DEATH

PR.NO.60R0375/88R002: REPRODUCTIVE TOX. STUDY TO DETECT EFFECTS OF MIXED ANTI-ANDROGENIC SUBSTANCES IN RATS; ORAL ADM. (GAVAGE)

INDIVIDUAL PUP NECROPSY OBSERVATIONS

TEST GROUP 4(0.00025 MG/KG BW/D)

FEMALE#	PUP#	STATUS	DAY	OBSERVATION	POSITION/GRADE
124	1	K	21	NOTHING ABNORMAL DETECTED	
	4	K	21	NOTHING ABNORMAL DETECTED	
	6	K	21	NOTHING ABNORMAL DETECTED	
	7	K	21	DILATED RENAL PELVIS	B
	8	K	21	NOTHING ABNORMAL DETECTED	
	10	K	21	NOTHING ABNORMAL DETECTED	
125	2	K	21	NOTHING ABNORMAL DETECTED	
	3	K	21	NOTHING ABNORMAL DETECTED	
	4	K	21	NOTHING ABNORMAL DETECTED	
	5	K	21	NOTHING ABNORMAL DETECTED	
	6	K	21	NOTHING ABNORMAL DETECTED	
	9	K	21	NOTHING ABNORMAL DETECTED	
	11	K	21	NOTHING ABNORMAL DETECTED	

POSITION/GRADE CODE: R-RIGHT, L-LEFT, B-BILATE , 1-SLIGHT, 2-MODERATE, 3-MARKED, P-PRESENT
PUP STATUS CODES: K-SCHEDULED SACRIFICE
NUMBER FOLLOWING PUP STATUS = DAY POST PARTUM OF DEATH

PR.NO.60R0375/88R002: REPRODUCTIVE TOX. STUDY TO DETECT EFFECTS OF MIXED ANTI-ANDROGENIC SUBSTANCES IN RATS; ORAL ADM. (GAVAGE)

INDIVIDUAL MALE CLINICAL OBSERVATIONS

MALES TEST GROUP 0 (0 MG/KG BW/D)

ANIMAL#	OBSERVATIONS	WEEK OF STUDY 0	1	2	3	4	5
501	NOTHING ABNORMAL DETECTED	P	P	P	P	P	
	SCHEDULED SACRIFICE					P	
502	NOTHING ABNORMAL DETECTED	P	P	P	P		
	SCHEDULED SACRIFICE				P		
503	NOTHING ABNORMAL DETECTED	P	P	P	P		
	SCHEDULED SACRIFICE				P		
504	NOTHING ABNORMAL DETECTED	P	P	P	P	P	
	SCHEDULED SACRIFICE					P	
505	NOTHING ABNORMAL DETECTED	P	P	P	P		
	SCHEDULED SACRIFICE				P		
506	NOTHING ABNORMAL DETECTED	P	P	P	P		
	SCHEDULED SACRIFICE				P		
507	NOTHING ABNORMAL DETECTED	P	P	P	P		
	SCHEDULED SACRIFICE				P		
508	NOTHING ABNORMAL DETECTED	P	P	P	P	P	
	SCHEDULED SACRIFICE					P	
509	NOTHING ABNORMAL DETECTED	P	P	P	P		
	SCHEDULED SACRIFICE				P		
510	NOTHING ABNORMAL DETECTED	P	P	P	P		
	SCHEDULED SACRIFICE				P		

CODE: 1-SLIGHT 2-MODERATE 3-MARKED P-PRESENT

PR.NO.60R0375/88R002: REPRODUCTIVE TOX. STUDY TO DETECT EFFECTS
OF MIXED ANTI-ANDROGENIC SUBSTANCES IN RATS; ORAL ADM. (GAVAGE)
INDIVIDUAL MALE CLINICAL OBSERVATIONS

MALES TEST GROUP 1 (ADI-MIX)

ANIMAL#	OBSERVATIONS	WEEK OF STUDY 0	1	2	3	4	5
511	NOTHING ABNORMAL DETECTED	P	P	P	P		
	SCHEDULED SACRIFICE				P		
512	NOTHING ABNORMAL DETECTED	P	P	P	P		
	SCHEDULED SACRIFICE				P		
513	NOTHING ABNORMAL DETECTED	P	P	P	P		
	SCHEDULED SACRIFICE				P		
514	NOTHING ABNORMAL DETECTED	P	P	P	P		
	SCHEDULED SACRIFICE				P		
515	NOTHING ABNORMAL DETECTED	P	P	P	P		
	SCHEDULED SACRIFICE				P		
516	NOTHING ABNORMAL DETECTED	P	P	P	P		
	SCHEDULED SACRIFICE				P		
517	NOTHING ABNORMAL DETECTED	P	P	P	P		
	SCHEDULED SACRIFICE				P		
518	NOTHING ABNORMAL DETECTED	P	P	P	P	P	
	SCHEDULED SACRIFICE					P	
519	NOTHING ABNORMAL DETECTED	P	P	P	P		
	SCHEDULED SACRIFICE				P		
520	NOTHING ABNORMAL DETECTED	P	P	P	P		
	SCHEDULED SACRIFICE				P		

CODE: 1-SLIGHT 2-MODERATE 3-MARKED P-PRESENT

PR.NO.60R0375/88R002: REPRODUCTIVE TOX. STUDY TO DETECT EFFECTS
OF MIXED ANTI-ANDROGENIC SUBSTANCES IN RATS; ORAL ADM. (GAVAGE)
INDIVIDUAL MALE CLINICAL OBSERVATIONS

MALES TEST GROUP 2 (NOAEL-MIX)

ANIMAL#	OBSERVATIONS	0	1	2	3	4	5
521	NOTHING ABNORMAL DETECTED	P	P	P	P		
	SCHEDULED SACRIFICE				P		
522	NOTHING ABNORMAL DETECTED	P	P	P	P		
	SCHEDULED SACRIFICE				P		
523	NOTHING ABNORMAL DETECTED	P	P	P	P		
	SCHEDULED SACRIFICE				P		
524	NOTHING ABNORMAL DETECTED	P	P	P	P		
	SCHEDULED SACRIFICE				P		
525	NOTHING ABNORMAL DETECTED	P	P	P	P		
	SCHEDULED SACRIFICE				P		
526	NOTHING ABNORMAL DETECTED	P	P	P	P	P	
	SCHEDULED SACRIFICE					P	
527	NOTHING ABNORMAL DETECTED	P	P	P	P		
	SCHEDULED SACRIFICE				P		
528	NOTHING ABNORMAL DETECTED	P	P	P	P		
	SCHEDULED SACRIFICE				P		
529	NOTHING ABNORMAL DETECTED	P	P	P	P	P	
	SCHEDULED SACRIFICE					P	
530	NOTHING ABNORMAL DETECTED	P	P	P	P		
	SCHEDULED SACRIFICE				P		

(Columns 0–5: WEEK OF STUDY)

CODE: 1-SLIGHT 2-MODERATE 3-MARKED P-PRESENT

22-AUG-13

88R002S2 TABLE : IIA- 172

PR.NO.60R0375/88R002: REPRODUCTIVE TOX. STUDY TO DETECT EFFECTS
OF MIXED ANTI-ANDROGENIC SUBSTANCES IN RATS; ORAL ADM. (GAVAGE)
INDIVIDUAL MALE CLINICAL OBSERVATIONS

MALES TEST GROUP 3 (LOAEL-MIX)

ANIMAL#	OBSERVATIONS	WEEK OF STUDY 0	1	2	3	4	5
531	NOTHING ABNORMAL DETECTED	P	P				P
	SALIVATION AFTER TREATMENT		P	P	P	P	P
	SCHEDULED SACRIFICE						P
532	NOTHING ABNORMAL DETECTED	P	P	P		P	
	SALIVATION AFTER TREATMENT		P	P	P	P	
	SCHEDULED SACRIFICE					P	
533	NOTHING ABNORMAL DETECTED	P	P		P		
	SALIVATION AFTER TREATMENT		P	P	P		
	SCHEDULED SACRIFICE				P		
534	NOTHING ABNORMAL DETECTED	P	P			P	
	SALIVATION AFTER TREATMENT		P	P	P	P	
	SCHEDULED SACRIFICE					P	
535	NOTHING ABNORMAL DETECTED	P	P	P		P	
	SALIVATION AFTER TREATMENT			P	P	P	
	SCHEDULED SACRIFICE					P	
536	NOTHING ABNORMAL DETECTED	P	P	P			P
	SALIVATION AFTER TREATMENT			P	P	P	
	SCHEDULED SACRIFICE						P
537	NOTHING ABNORMAL DETECTED	P	P			P	
	SALIVATION AFTER TREATMENT		P	P	P	P	
	SCHEDULED SACRIFICE					P	
538	NOTHING ABNORMAL DETECTED	P	P				P
	SALIVATION AFTER TREATMENT		P	P	P	P	P
	SCHEDULED SACRIFICE						P
539	NOTHING ABNORMAL DETECTED	P	P				P
	SALIVATION AFTER TREATMENT		P	P	P	P	P
	SCHEDULED SACRIFICE						P
540	NOTHING ABNORMAL DETECTED	P	P				P
	SALIVATION AFTER TREATMENT		P	P	P	P	P
	SCHEDULED SACRIFICE						P

CODE: 1-SLIGHT 2-MODERATE 3-MARKED P-PRESENT

PR.NO.60R0375/88R002: REPRODUCTIVE TOX. STUDY TO DETECT EFFECTS
OF MIXED ANTI-ANDROGENIC SUBSTANCES IN RATS; ORAL ADM. (GAVAGE)
INDIVIDUAL MALE CLINICAL OBSERVATIONS

MALES TEST GROUP 4(0.00025 MG/KG BW/D)

ANIMAL#	OBSERVATIONS	WEEK OF STUDY 0	1	2	3	4	5
541	NOTHING ABNORMAL DETECTED	P	P	P	P		
	SCHEDULED SACRIFICE				P		
542	NOTHING ABNORMAL DETECTED	P	P	P	P		
	SCHEDULED SACRIFICE				P		
543	NOTHING ABNORMAL DETECTED	P	P	P	P		
	SCHEDULED SACRIFICE				P		
544	NOTHING ABNORMAL DETECTED	P	P	P	P		
	SCHEDULED SACRIFICE				P		
545	NOTHING ABNORMAL DETECTED	P	P	P			
	SCHEDULED SACRIFICE			P			
546	NOTHING ABNORMAL DETECTED	P	P	P	P		
	SCHEDULED SACRIFICE				P		
547	NOTHING ABNORMAL DETECTED	P	P	P			
	SCHEDULED SACRIFICE			P			
548	NOTHING ABNORMAL DETECTED	P	P	P	P		
	SCHEDULED SACRIFICE				P		
549	NOTHING ABNORMAL DETECTED	P	P	P	P		
	SCHEDULED SACRIFICE				P		
550	NOTHING ABNORMAL DETECTED	P	P	P	P	P	
	SCHEDULED SACRIFICE					P	

CODE: 1-SLIGHT 2-MODERATE 3-MARKED P-PRESENT

PR.NO.60R0375/88R002: REPRODUCTIVE TOX. STUDY TO DETECT EFFECTS OF MIXED ANTI-ANDROGENIC SUBSTANCES IN RATS; ORAL ADM. (GAVAGE)

INDIVIDUAL FEMALE CLINICAL OBSERVATIONS

FEMALES TEST GROUP 0 (0 MG/KG BW/D)

ANIMAL#	OBSERVATIONS	WEEK OF STUDY 0	1
601	NOTHING ABNORMAL DETECTED	P	P
	SCHEDULED SACRIFICE		P
602	NOTHING ABNORMAL DETECTED	P	P
	SCHEDULED SACRIFICE		P
603	NOTHING ABNORMAL DETECTED	P	P
	SCHEDULED SACRIFICE		P
604	NOTHING ABNORMAL DETECTED	P	P
	SCHEDULED SACRIFICE		P
605	NOTHING ABNORMAL DETECTED	P	P
	SCHEDULED SACRIFICE		P
606	NOTHING ABNORMAL DETECTED	P	P
	SCHEDULED SACRIFICE		P
607	NOTHING ABNORMAL DETECTED	P	P
	SCHEDULED SACRIFICE		P
608	NOTHING ABNORMAL DETECTED	P	P
	SCHEDULED SACRIFICE		P
609	NOTHING ABNORMAL DETECTED	P	P
	SCHEDULED SACRIFICE		P
610	NOTHING ABNORMAL DETECTED	P	P
	SCHEDULED SACRIFICE		P

CODE: 1-SLIGHT 2-MODERATE 3-MARKED P-PRESENT

PR.NO.60R0375/88R002: REPRODUCTIVE TOX. STUDY TO DETECT EFFECTS
OF MIXED ANTI-ANDROGENIC SUBSTANCES IN RATS; ORAL ADM. (GAVAGE)
INDIVIDUAL FEMALE CLINICAL OBSERVATIONS

FEMALES TEST GROUP 1 (ADI-MIX)

ANIMAL#	OBSERVATIONS	WEEK OF STUDY 0	1
611	NOTHING ABNORMAL DETECTED	P	P
	SCHEDULED SACRIFICE		P
612	NOTHING ABNORMAL DETECTED	P	P
	SCHEDULED SACRIFICE		P
613	NOTHING ABNORMAL DETECTED	P	P
	SCHEDULED SACRIFICE		P
614	NOTHING ABNORMAL DETECTED	P	P
	SCHEDULED SACRIFICE		P
615	NOTHING ABNORMAL DETECTED	P	P
	SCHEDULED SACRIFICE		P
616	NOTHING ABNORMAL DETECTED	P	P
	SCHEDULED SACRIFICE		P
617	NOTHING ABNORMAL DETECTED	P	P
	SCHEDULED SACRIFICE		P
618	NOTHING ABNORMAL DETECTED	P	P
	SCHEDULED SACRIFICE		P
619	NOTHING ABNORMAL DETECTED	P	P
	SCHEDULED SACRIFICE		P
620	NOTHING ABNORMAL DETECTED	P	P
	SCHEDULED SACRIFICE		P

CODE: 1-SLIGHT 2-MODERATE 3-MARKED P-PRESENT

PR.NO.60R0375/88R002: REPRODUCTIVE TOX. STUDY TO DETECT EFFECTS
OF MIXED ANTI-ANDROGENIC SUBSTANCES IN RATS; ORAL ADM. (GAVAGE)
INDIVIDUAL FEMALE CLINICAL OBSERVATIONS

FEMALES TEST GROUP 2 (NOAEL-MIX)

ANIMAL#	OBSERVATIONS	WEEK OF STUDY 0	1
621	NOTHING ABNORMAL DETECTED	P	P
	SCHEDULED SACRIFICE		P
622	NOTHING ABNORMAL DETECTED	P	P
	SCHEDULED SACRIFICE		P
623	NOTHING ABNORMAL DETECTED	P	P
	SCHEDULED SACRIFICE		P
624	NOTHING ABNORMAL DETECTED	P	P
	SCHEDULED SACRIFICE		P
625	NOTHING ABNORMAL DETECTED	P	P
	SCHEDULED SACRIFICE		P
626	NOTHING ABNORMAL DETECTED	P	P
	SCHEDULED SACRIFICE		P
627	NOTHING ABNORMAL DETECTED	P	P
	SCHEDULED SACRIFICE		P
628	NOTHING ABNORMAL DETECTED	P	P
	SCHEDULED SACRIFICE		P
629	NOTHING ABNORMAL DETECTED	P	P
	SCHEDULED SACRIFICE		P
630	NOTHING ABNORMAL DETECTED	P	P
	SCHEDULED SACRIFICE		P

CODE: 1-SLIGHT 2-MODERATE 3-MARKED P-PRESENT

PR.NO.60R0375/88R002: REPRODUCTIVE TOX. STUDY TO DETECT EFFECTS OF MIXED ANTI-ANDROGENIC SUBSTANCES IN RATS; ORAL ADM. (GAVAGE)

INDIVIDUAL FEMALE CLINICAL OBSERVATIONS

FEMALES TEST GROUP 3 (LOAEL-MIX)

ANIMAL#	OBSERVATIONS	WEEK OF STUDY 0	1
631	NOTHING ABNORMAL DETECTED	P	P
	SCHEDULED SACRIFICE		P
632	NOTHING ABNORMAL DETECTED	P	P
	SCHEDULED SACRIFICE		P
633	NOTHING ABNORMAL DETECTED	P	P
	SCHEDULED SACRIFICE		P
634	NOTHING ABNORMAL DETECTED	P	P
	SCHEDULED SACRIFICE		P
635	NOTHING ABNORMAL DETECTED	P	P
	SCHEDULED SACRIFICE		P
636	NOTHING ABNORMAL DETECTED	P	P
	SCHEDULED SACRIFICE		P
637	NOTHING ABNORMAL DETECTED	P	P
	SCHEDULED SACRIFICE		P
638	NOTHING ABNORMAL DETECTED	P	P
	SCHEDULED SACRIFICE		P
639	NOTHING ABNORMAL DETECTED	P	P
	SCHEDULED SACRIFICE		P
640	NOTHING ABNORMAL DETECTED	P	P
	SCHEDULED SACRIFICE		P

CODE: 1-SLIGHT 2-MODERATE 3-MARKED P-PRESENT

PR.NO.60R0375/88R002: REPRODUCTIVE TOX. STUDY TO DETECT EFFECTS OF MIXED ANTI-ANDROGENIC SUBSTANCES IN RATS; ORAL ADM. (GAVAGE)

INDIVIDUAL FEMALE CLINICAL OBSERVATIONS

FEMALES TEST GROUP 4(0.00025 MG/KG BW/D)

ANIMAL#	OBSERVATIONS	WEEK OF STUDY 0	1
641	NOTHING ABNORMAL DETECTED	P	P
	SCHEDULED SACRIFICE		P
642	NOTHING ABNORMAL DETECTED	P	P
	SCHEDULED SACRIFICE		P
643	NOTHING ABNORMAL DETECTED	P	P
	SCHEDULED SACRIFICE		P
644	NOTHING ABNORMAL DETECTED	P	P
	SCHEDULED SACRIFICE		P
645	NOTHING ABNORMAL DETECTED	P	P
	SCHEDULED SACRIFICE		P
646	NOTHING ABNORMAL DETECTED	P	P
	SCHEDULED SACRIFICE		P
647	NOTHING ABNORMAL DETECTED	P	P
	SCHEDULED SACRIFICE		P
648	NOTHING ABNORMAL DETECTED	P	P
	SCHEDULED SACRIFICE		P
649	NOTHING ABNORMAL DETECTED	P	P
	SCHEDULED SACRIFICE		P
650	NOTHING ABNORMAL DETECTED	P	P
	SCHEDULED SACRIFICE		P

CODE: 1-SLIGHT 2-MODERATE 3-MARKED P-PRESENT

PR.NO.60R0375/88R002: REPRODUCTIVE TOX. STUDY TO DETECT EFFECTS
OF MIXED ANTI-ANDROGENIC SUBSTANCES IN RATS; ORAL ADM. (GAVAGE)
INDIVIDUAL MALE CLINICAL OBSERVATIONS

MALES TEST GROUP 0 (0 MG/KG BW/D)

ANIMAL#	OBSERVATIONS	WEEK OF STUDY 0	1	2	3	4	5	6	7	8	9
701	NOTHING ABNORMAL DETECTED	P	P	P	P	P	P	P	P	P	
	SCHEDULED SACRIFICE									P	
702	NOTHING ABNORMAL DETECTED	P	P	P	P	P	P	P	P	P	
	SCHEDULED SACRIFICE									P	
703	NOTHING ABNORMAL DETECTED	P	P								
	OBLIQUE HEAD POSITION		P								
	HEAD: SWELLING		P								
	LACRIMATION		P								
	SACRIFICED MORIBUND		P								
704	NOTHING ABNORMAL DETECTED	P	P	P	P	P	P	P	P	P	
	SCHEDULED SACRIFICE									P	
705	NOTHING ABNORMAL DETECTED	P	P	P	P	P	P	P	P	P	
	SCHEDULED SACRIFICE									P	
706	NOTHING ABNORMAL DETECTED	P	P	P	P	P	P	P	P	P	
	SCHEDULED SACRIFICE									P	
707	NOTHING ABNORMAL DETECTED	P	P	P	P	P	P	P	P	P	P
	SCHEDULED SACRIFICE										P
708	NOTHING ABNORMAL DETECTED	P	P	P	P	P	P	P	P	P	P
	SCHEDULED SACRIFICE										P
709	NOTHING ABNORMAL DETECTED	P	P	P	P	P	P	P	P	P	
	SCHEDULED SACRIFICE									P	
710	NOTHING ABNORMAL DETECTED	P	P	P	P	P	P	P	P	P	
	SCHEDULED SACRIFICE									P	

CODE: 1-SLIGHT 2-MODERATE 3-MARKED P-PRESENT

PR.NO.60R0375/88R002: REPRODUCTIVE TOX. STUDY TO DETECT EFFECTS
OF MIXED ANTI-ANDROGENIC SUBSTANCES IN RATS; ORAL ADM. (GAVAGE)
INDIVIDUAL MALE CLINICAL OBSERVATIONS

MALES TEST GROUP 1 (ADI-MIX)

ANIMAL#	OBSERVATIONS	0	1	2	3	4	5	6	7	8	9
711	NOTHING ABNORMAL DETECTED	P	P	P	P	P	P	P	P	P	P
	SCHEDULED SACRIFICE										P
712	NOTHING ABNORMAL DETECTED	P	P	P	P	P	P	P	P	P	
	SCHEDULED SACRIFICE									P	
713	NOTHING ABNORMAL DETECTED	P	P	P	P	P	P	P	P	P	
	SCHEDULED SACRIFICE									P	
714	NOTHING ABNORMAL DETECTED	P	P	P	P	P	P	P	P	P	
	SCHEDULED SACRIFICE									P	
715	NOTHING ABNORMAL DETECTED	P	P	P	P	P	P	P	P	P	
	SCHEDULED SACRIFICE									P	
716	NOTHING ABNORMAL DETECTED	P	P	P	P	P	P	P	P	P	
	SCHEDULED SACRIFICE									P	
717	NOTHING ABNORMAL DETECTED	P	P	P	P	P	P	P	P	P	
	SCHEDULED SACRIFICE									P	
718	NOTHING ABNORMAL DETECTED	P	P	P	P	P	P	P	P	P	P
	SCHEDULED SACRIFICE										P
719	NOTHING ABNORMAL DETECTED	P	P	P	P	P	P	P	P	P	
	SCHEDULED SACRIFICE									P	
720	NOTHING ABNORMAL DETECTED	P	P	P	P	P	P	P	P	P	
	SCHEDULED SACRIFICE									P	

(Columns 0–9: WEEK OF STUDY)

CODE: 1-SLIGHT 2-MODERATE 3-MARKED P-PRESENT

PR.NO.60R0375/88R002: REPRODUCTIVE TOX. STUDY TO DETECT EFFECTS
OF MIXED ANTI-ANDROGENIC SUBSTANCES IN RATS; ORAL ADM. (GAVAGE)
INDIVIDUAL MALE CLINICAL OBSERVATIONS

MALES TEST GROUP 2 (NOAEL-MIX)

ANIMAL#	OBSERVATIONS	WEEK OF STUDY: 0	1	2	3	4	5	6	7	8	9
721	NOTHING ABNORMAL DETECTED	P	P	P	P	P	P	P	P	P	
	SCHEDULED SACRIFICE									P	
722	NOTHING ABNORMAL DETECTED	P	P	P	P	P	P	P	P	P	
	SCHEDULED SACRIFICE									P	
723	NOTHING ABNORMAL DETECTED	P	P	P	P	P	P	P	P	P	
	SCHEDULED SACRIFICE									P	
724	NOTHING ABNORMAL DETECTED	P	P	P	P	P	P	P	P	P	
	SCHEDULED SACRIFICE									P	
725	NOTHING ABNORMAL DETECTED	P	P	P	P	P	P	P	P	P	P
	SCHEDULED SACRIFICE										P
726	NOTHING ABNORMAL DETECTED	P	P	P	P	P	P	P	P	P	P
	SCHEDULED SACRIFICE										P
727	NOTHING ABNORMAL DETECTED	P	P	P	P	P	P	P	P	P	P
	SCHEDULED SACRIFICE										P
728	NOTHING ABNORMAL DETECTED	P	P	P	P	P	P	P	P	P	P
	SCHEDULED SACRIFICE										P
729	NOTHING ABNORMAL DETECTED	P	P	P	P	P	P	P	P	P	
	SCHEDULED SACRIFICE									P	
730	NOTHING ABNORMAL DETECTED	P	P	P	P	P	P	P	P	P	
	SCHEDULED SACRIFICE									P	

CODE: 1-SLIGHT 2-MODERATE 3-MARKED P-PRESENT

PR.NO.60R0375/88R002: REPRODUCTIVE TOX. STUDY TO DETECT EFFECTS
OF MIXED ANTI-ANDROGENIC SUBSTANCES IN RATS; ORAL ADM. (GAVAGE)
INDIVIDUAL MALE CLINICAL OBSERVATIONS

MALES TEST GROUP 3 (LOAEL-MIX)

ANIMAL#	OBSERVATIONS	WEEK OF STUDY 0	1	2	3	4	5	6	7	8	9
731	NOTHING ABNORMAL DETECTED	P	P							P	
	SALIVATION AFTER TREATMENT		P	P	P	P	P	P	P	P	
	SCHEDULED SACRIFICE									P	
732	NOTHING ABNORMAL DETECTED	P	P						P	P	
	SALIVATION AFTER TREATMENT		P	P	P	P	P	P	P	P	
	SCHEDULED SACRIFICE									P	
733	NOTHING ABNORMAL DETECTED	P	P						P		P
	SALIVATION AFTER TREATMENT		P	P	P	P	P	P	P	P	
	SCHEDULED SACRIFICE										P
734	NOTHING ABNORMAL DETECTED	P	P						P		P
	SALIVATION AFTER TREATMENT		P	P	P	P	P	P	P	P	
	SCHEDULED SACRIFICE										P
735	NOTHING ABNORMAL DETECTED	P	P						P	P	P
	SALIVATION AFTER TREATMENT		P	P	P	P	P	P	P	P	
	SCHEDULED SACRIFICE										P
736	NOTHING ABNORMAL DETECTED	P	P							P	
	SALIVATION AFTER TREATMENT		P	P	P	P	P	P	P	P	
	SCHEDULED SACRIFICE									P	
737	NOTHING ABNORMAL DETECTED	P	P						P	P	
	SALIVATION AFTER TREATMENT		P	P	P	P	P	P	P	P	
	SCHEDULED SACRIFICE									P	
738	NOTHING ABNORMAL DETECTED	P	P						P	P	
	SALIVATION AFTER TREATMENT		P	P	P	P	P	P	P	P	
	SCHEDULED SACRIFICE									P	
739	NOTHING ABNORMAL DETECTED	P	P						P	P	
	SALIVATION AFTER TREATMENT		P	P	P	P	P	P	P	P	
	SCHEDULED SACRIFICE									P	
740	NOTHING ABNORMAL DETECTED	P	P					P	P	P	
	SALIVATION AFTER TREATMENT		P	P	P	P	P	P	P	P	
	SCHEDULED SACRIFICE									P	

CODE: 1-SLIGHT 2-MODERATE 3-MARKED P-PRESENT

PR.NO.60R0375/88R002: REPRODUCTIVE TOX. STUDY TO DETECT EFFECTS
OF MIXED ANTI-ANDROGENIC SUBSTANCES IN RATS; ORAL ADM. (GAVAGE)
INDIVIDUAL MALE CLINICAL OBSERVATIONS

MALES TEST GROUP 4(0.00025 MG/KG BW/D)

ANIMAL#	OBSERVATIONS	WEEK OF STUDY 0	1	2	3	4	5	6	7	8	9
741	NOTHING ABNORMAL DETECTED	P	P	P	P	P	P	P	P	P	P
	SCHEDULED SACRIFICE										P
742	NOTHING ABNORMAL DETECTED	P	P	P	P	P	P	P	P	P	
	SCHEDULED SACRIFICE									P	
743	NOTHING ABNORMAL DETECTED	P	P	P	P	P	P	P	P	P	
	SCHEDULED SACRIFICE									P	
744	NOTHING ABNORMAL DETECTED	P	P	P	P	P	P	P	P	P	
	SCHEDULED SACRIFICE									P	
745	NOTHING ABNORMAL DETECTED	P	P	P	P	P	P	P	P	P	P
	SCHEDULED SACRIFICE										P
746	NOTHING ABNORMAL DETECTED	P	P	P	P	P	P	P	P	P	P
	SCHEDULED SACRIFICE										P
747	NOTHING ABNORMAL DETECTED	P	P	P	P	P	P	P	P	P	
	SCHEDULED SACRIFICE									P	
748	NOTHING ABNORMAL DETECTED	P	P	P	P	P	P	P	P	P	
	SCHEDULED SACRIFICE									P	
749	NOTHING ABNORMAL DETECTED	P	P	P	P	P	P	P	P	P	
	SCHEDULED SACRIFICE									P	
750	NOTHING ABNORMAL DETECTED	P	P	P	P	P	P	P	P	P	
	SCHEDULED SACRIFICE									P	

CODE: 1-SLIGHT 2-MODERATE 3-MARKED P-PRESENT

PR.NO.60R0375/88R002: REPRODUCTIVE TOX. STUDY TO DETECT EFFECTS
OF MIXED ANTI-ANDROGENIC SUBSTANCES IN RATS; ORAL ADM. (GAVAGE)
INDIVIDUAL FEMALE CLINICAL OBSERVATIONS

FEMALES TEST GROUP 0 (0 MG/KG BW/D)

ANIMAL#	OBSERVATIONS (WEEK OF STUDY)	0	1	2	3	4	5	6	7	8	9
801	NOTHING ABNORMAL DETECTED	P	P	P	P	P	P	P	P	P	
	SCHEDULED SACRIFICE									P	
802	NOTHING ABNORMAL DETECTED	P	P	P	P	P	P	P	P	P	
	SCHEDULED SACRIFICE									P	
803	NOTHING ABNORMAL DETECTED	P	P	P	P	P	P	P	P	P	
	SCHEDULED SACRIFICE									P	
804	NOTHING ABNORMAL DETECTED	P	P	P	P	P	P	P	P	P	
	SCHEDULED SACRIFICE									P	
805	NOTHING ABNORMAL DETECTED	P	P								
	INDRAWN FLANKS		P								
	LABORED RESPIRATION		P								
	PILOERECTION		P								
	SACRIFICED MORIBUND		P								
806	NOTHING ABNORMAL DETECTED	P	P	P	P	P	P	P	P	P	
	SCHEDULED SACRIFICE									P	
807	NOTHING ABNORMAL DETECTED	P	P	P	P	P	P	P	P	P	P
	SCHEDULED SACRIFICE										P
808	NOTHING ABNORMAL DETECTED	P	P	P	P	P	P	P	P	P	P
	SCHEDULED SACRIFICE										P
809	NOTHING ABNORMAL DETECTED	P	P	P	P	P	P	P	P	P	P
	SCHEDULED SACRIFICE										P
810	NOTHING ABNORMAL DETECTED	P	P	P	P	P	P	P	P	P	
	SCHEDULED SACRIFICE									P	

CODE: 1-SLIGHT 2-MODERATE 3-MARKED P-PRESENT

PR.NO.60R0375/88R002: REPRODUCTIVE TOX. STUDY TO DETECT EFFECTS
OF MIXED ANTI-ANDROGENIC SUBSTANCES IN RATS; ORAL ADM. (GAVAGE)
INDIVIDUAL FEMALE CLINICAL OBSERVATIONS

FEMALES TEST GROUP 1 (ADI-MIX)

ANIMAL#	OBSERVATIONS	0	1	2	3	4	5	6	7	8	9
811	NOTHING ABNORMAL DETECTED	P	P	P	P	P	P	P	P	P	
	SCHEDULED SACRIFICE									P	
812	NOTHING ABNORMAL DETECTED	P	P	P	P	P	P	P	P	P	
	SCHEDULED SACRIFICE									P	
813	NOTHING ABNORMAL DETECTED	P	P	P	P	P	P	P	P	P	
	SCHEDULED SACRIFICE									P	
814	NOTHING ABNORMAL DETECTED	P	P	P	P	P	P	P	P	P	
	VAGINAL HEMORRHAGE									P	
	SCHEDULED SACRIFICE									P	
815	NOTHING ABNORMAL DETECTED	P	P	P	P	P	P	P	P	P	P
	SCHEDULED SACRIFICE										P
816	NOTHING ABNORMAL DETECTED	P	P	P	P	P	P	P	P	P	
	SCHEDULED SACRIFICE									P	
817	NOTHING ABNORMAL DETECTED	P	P	P	P	P	P	P	P	P	
	SCHEDULED SACRIFICE									P	
818	NOTHING ABNORMAL DETECTED	P	P	P	P	P	P	P	P	P	
	SCHEDULED SACRIFICE									P	
819	NOTHING ABNORMAL DETECTED	P	P	P	P	P	P	P	P	P	P
	SCHEDULED SACRIFICE										P
820	NOTHING ABNORMAL DETECTED	P	P	P	P	P	P	P	P	P	P
	SCHEDULED SACRIFICE										P

(Columns 0–9: WEEK OF STUDY)

CODE: 1-SLIGHT 2-MODERATE 3-MARKED P-PRESENT

PR.NO.60R0375/88R002: REPRODUCTIVE TOX. STUDY TO DETECT EFFECTS
OF MIXED ANTI-ANDROGENIC SUBSTANCES IN RATS; ORAL ADM. (GAVAGE)
INDIVIDUAL FEMALE CLINICAL OBSERVATIONS

FEMALES TEST GROUP 2 (NOAEL-MIX)

ANIMAL#	OBSERVATIONS	WEEK OF STUDY 0	1	2	3	4	5	6	7	8	9
821	NOTHING ABNORMAL DETECTED	P	P	P	P	P	P	P	P	P	
	SCHEDULED SACRIFICE									P	
822	NOTHING ABNORMAL DETECTED	P	P	P	P	P	P	P	P	P	
	SCHEDULED SACRIFICE									P	
823	NOTHING ABNORMAL DETECTED	P	P	P	P	P	P	P	P	P	
	SCHEDULED SACRIFICE									P	
824	NOTHING ABNORMAL DETECTED	P	P	P	P	P	P	P	P	P	
	SCHEDULED SACRIFICE									P	
825	NOTHING ABNORMAL DETECTED	P	P	P	P	P	P	P	P	P	
	SCHEDULED SACRIFICE									P	
826	NOTHING ABNORMAL DETECTED	P	P	P	P	P	P	P	P	P	P
	SCHEDULED SACRIFICE										P
827	NOTHING ABNORMAL DETECTED	P	P	P	P	P	P	P	P	P	P
	SCHEDULED SACRIFICE										P
828	NOTHING ABNORMAL DETECTED	P	P	P	P	P	P	P	P	P	
	SCHEDULED SACRIFICE									P	
829	NOTHING ABNORMAL DETECTED	P	P	P	P	P	P	P	P	P	
	SCHEDULED SACRIFICE									P	
830	NOTHING ABNORMAL DETECTED	P	P	P	P	P	P	P	P	P	
	SCHEDULED SACRIFICE									P	

CODE: 1-SLIGHT 2-MODERATE 3-MARKED P-PRESENT

PR.NO.60R0375/88R002: REPRODUCTIVE TOX. STUDY TO DETECT EFFECTS
OF MIXED ANTI-ANDROGENIC SUBSTANCES IN RATS; ORAL ADM. (GAVAGE)
INDIVIDUAL FEMALE CLINICAL OBSERVATIONS

FEMALES TEST GROUP 3 (LOAEL-MIX)

ANIMAL#	OBSERVATIONS	0	1	2	3	4	5	6	7	8	9
		WEEK OF STUDY									
831	NOTHING ABNORMAL DETECTED	P	P		P				P	P	
	SALIVATION AFTER TREATMENT		P	P	P	P	P	P	P	P	
	SCHEDULED SACRIFICE									P	
832	NOTHING ABNORMAL DETECTED	P	P		P				P	P	
	SALIVATION AFTER TREATMENT		P	P	P	P	P	P	P	P	
	SCHEDULED SACRIFICE									P	
833	NOTHING ABNORMAL DETECTED	P	P								P
	SALIVATION AFTER TREATMENT		P	P	P	P	P	P	P	P	
	SCHEDULED SACRIFICE										P
834	NOTHING ABNORMAL DETECTED	P	P						P		P
	SALIVATION AFTER TREATMENT		P	P	P	P	P	P	P	P	
	SCHEDULED SACRIFICE										P
835	NOTHING ABNORMAL DETECTED	P	P						P		P
	SALIVATION AFTER TREATMENT		P	P	P	P	P	P	P	P	
	SCHEDULED SACRIFICE										P
836	NOTHING ABNORMAL DETECTED	P	P							P	
	SALIVATION AFTER TREATMENT		P	P	P	P	P	P	P	P	
	SCHEDULED SACRIFICE									P	
837	NOTHING ABNORMAL DETECTED	P	P					P	P	P	
	SALIVATION AFTER TREATMENT		P	P	P	P	P	P	P	P	
	SCHEDULED SACRIFICE									P	
838	NOTHING ABNORMAL DETECTED	P	P							P	
	SALIVATION AFTER TREATMENT		P	P	P	P	P	P	P	P	
	SCHEDULED SACRIFICE									P	
839	NOTHING ABNORMAL DETECTED	P	P							P	
	SALIVATION AFTER TREATMENT		P	P	P	P	P	P	P	P	
	SCHEDULED SACRIFICE									P	
840	NOTHING ABNORMAL DETECTED	P	P					P	P	P	
	SALIVATION AFTER TREATMENT		P	P	P	P	P	P	P	P	
	SCHEDULED SACRIFICE									P	

CODE: 1-SLIGHT 2-MODERATE 3-MARKED P-PRESENT

PR.NO.60R0375/88R002: REPRODUCTIVE TOX. STUDY TO DETECT EFFECTS
OF MIXED ANTI-ANDROGENIC SUBSTANCES IN RATS; ORAL ADM. (GAVAGE)
INDIVIDUAL FEMALE CLINICAL OBSERVATIONS

FEMALES TEST GROUP 4(0.00025 MG/KG BW/D)

ANIMAL#	OBSERVATIONS	WEEK OF STUDY 0	1	2	3	4	5	6	7	8	9
841	NOTHING ABNORMAL DETECTED	P	P	P	P	P	P	P	P	P	
	SCHEDULED SACRIFICE									P	
842	NOTHING ABNORMAL DETECTED	P	P	P	P	P	P	P	P	P	
	SCHEDULED SACRIFICE									P	
843	NOTHING ABNORMAL DETECTED	P	P	P	P	P	P	P	P	P	
	SCHEDULED SACRIFICE									P	
844	NOTHING ABNORMAL DETECTED	P	P	P	P	P	P	P	P	P	
	SCHEDULED SACRIFICE									P	
845	NOTHING ABNORMAL DETECTED	P	P	P	P	P	P	P	P	P	
	SCHEDULED SACRIFICE									P	
846	NOTHING ABNORMAL DETECTED	P	P	P	P	P	P	P	P	P	
	SCHEDULED SACRIFICE									P	
847	NOTHING ABNORMAL DETECTED	P	P	P	P	P	P	P	P	P	P
	SCHEDULED SACRIFICE										P
848	NOTHING ABNORMAL DETECTED	P	P	P	P	P	P	P	P	P	
	SCHEDULED SACRIFICE									P	
849	NOTHING ABNORMAL DETECTED	P	P	P	P	P	P	P	P	P	
	SCHEDULED SACRIFICE									P	
850	NOTHING ABNORMAL DETECTED	P	P	P	P	P	P	P	P	P	
	SCHEDULED SACRIFICE									P	

CODE: 1-SLIGHT 2-MODERATE 3-MARKED P-PRESENT

PR.NO.60R0375/88R002: REPRODUCTIVE TOX. STUDY TO DETECT EFFECTS
OF MIXED ANTI-ANDROGENIC SUBSTANCES IN RATS; ORAL ADM. (GAVAGE)
INDIVIDUAL FOOD CONSUMPTION -- GRAMS/ANIMAL/DAY

MALES TEST GROUP 0 (0 MG/KG BW/D)

	WEEK OF STUDY				
ANIMAL#	0- 1	1- 2	2- 3	3- 4	4- 5
501	8.5	13.9	18.4	20.0	
502	8.6	14.2	18.2		
503	8.6	14.1	18.2		
504	7.7	11.8	14.3	14.5	
505	7.8	12.8	17.4		
506	8.2	15.3	20.2		
507	8.5	12.7	16.7		
508	9.0	14.4	18.3	17.7	
509	9.2	13.9	17.9		
510	10.2	14.4	17.8		
MEAN	8.6	13.7	17.8	17.4	
S.D.	0.73	1.03	1.50	2.75	
N	10	10	10	3	

PR.NO.60R0375/88R002: REPRODUCTIVE TOX. STUDY TO DETECT EFFECTS
OF MIXED ANTI-ANDROGENIC SUBSTANCES IN RATS; ORAL ADM. (GAVAGE)
INDIVIDUAL FOOD CONSUMPTION -- GRAMS/ANIMAL/DAY

MALES TEST GROUP 1 (ADI-MIX)

	WEEK OF STUDY				
ANIMAL#	0- 1	1- 2	2- 3	3- 4	4- 5
511	7.9	12.6	16.2		
512	9.3	15.1	19.4		
513	10.0	16.1	20.7		
514	8.7	14.8	19.4		
515	9.1	14.9	17.8		
516	8.6	14.6	19.9		
517	7.9	13.1	17.7		
518	8.9	13.1	17.4	17.7	
519	9.0	13.3	19.3		
520	9.0	14.8	19.5		
MEAN	8.8	14.3	18.7	17.7	
S.D.	0.63	1.13	1.37	0.00	
N	10	10	10	1	

PR.NO.60R0375/88R002: REPRODUCTIVE TOX. STUDY TO DETECT EFFECTS
OF MIXED ANTI-ANDROGENIC SUBSTANCES IN RATS; ORAL ADM. (GAVAGE)
INDIVIDUAL FOOD CONSUMPTION -- GRAMS/ANIMAL/DAY

MALES TEST GROUP 2 (NOAEL-MIX)

	WEEK OF STUDY				
ANIMAL#	0- 1	1- 2	2- 3	3- 4	4- 5
521	8.6	14.2	18.0		
522	8.3	13.5	17.6		
523	9.5	15.2	19.6		
524	8.9	14.9	18.5		
525	9.2	15.1	20.9		
526	8.8	14.0	18.6	19.2	
527	9.1	13.7	18.5		
528	9.5	14.3	17.5		
529	9.0	13.4	17.0	18.5	
530	8.9	12.5	16.1		
MEAN	9.0	14.1	18.2	18.8	
S.D.	0.36	0.84	1.37	0.56	
N	10	10	10	2	

PR.NO.60R0375/88R002: REPRODUCTIVE TOX. STUDY TO DETECT EFFECTS
OF MIXED ANTI-ANDROGENIC SUBSTANCES IN RATS; ORAL ADM. (GAVAGE)
INDIVIDUAL FOOD CONSUMPTION -- GRAMS/ANIMAL/DAY

MALES TEST GROUP 3 (LOAEL-MIX)

	WEEK OF STUDY				
ANIMAL#	0- 1	1- 2	2- 3	3- 4	4- 5
531	7.6	12.5	17.1	19.6	19.7
532	8.9	14.0	18.2	19.5	
533	9.1	13.3	16.9		
534	9.2	13.7	16.1	18.7	
535	7.8	12.5	15.8	17.0	
536	9.3	14.6	19.7	20.8	22.3
537	8.0	12.7	15.6	18.3	
538	8.1	12.8	16.1	16.9	17.7
539	8.3	12.6	15.4	17.0	17.8
540	9.7	13.9	16.5	18.3	19.8
MEAN	8.6	13.3	16.8	18.5	19.5
S.D.	0.72	0.75	1.34	1.37	1.90
N	10	10	10	9	5

PR.NO.60R0375/88R002: REPRODUCTIVE TOX. STUDY TO DETECT EFFECTS
OF MIXED ANTI-ANDROGENIC SUBSTANCES IN RATS; ORAL ADM. (GAVAGE)
INDIVIDUAL FOOD CONSUMPTION -- GRAMS/ANIMAL/DAY

MALES TEST GROUP 4(0.00025 MG/KG BW/D)

	WEEK OF STUDY				
ANIMAL#	0- 1	1- 2	2- 3	3- 4	4- 5
541	8.0	13.1	18.0		
542	8.1	13.0	17.2		
543	9.1	14.9	18.8		
544	8.9	15.2	NM		
545	10.1	16.2			
546	8.4	14.6	18.8		
547	9.8	15.9			
548	9.7	15.9	19.5		
549	10.5	15.9	20.9		
550	11.0	16.3	19.9	19.4	
MEAN	9.4	15.1	19.0	19.4	
S.D.	1.02	1.23	1.23	0.00	
N	10	10	7	1	

NM=NOT MEASURED/NO VALUE DETERMINABLE

PR.NO.60R0375/88R002: REPRODUCTIVE TOX. STUDY TO DETECT EFFECTS
OF MIXED ANTI-ANDROGENIC SUBSTANCES IN RATS; ORAL ADM. (GAVAGE)
INDIVIDUAL FOOD CONSUMPTION -- GRAMS/ANIMAL/DAY

FEMALES TEST GROUP 0 (0 MG/KG BW/D)

ANIMAL#	WEEK OF STUDY 0- 1
601	9.3
602	8.1
603	8.6
604	7.3
605	7.3
606	8.9
607	9.8
608	8.5
609	7.9
610	9.1
MEAN	8.5
S.D.	0.84
N	10

PR.NO.60R0375/88R002: REPRODUCTIVE TOX. STUDY TO DETECT EFFECTS
OF MIXED ANTI-ANDROGENIC SUBSTANCES IN RATS; ORAL ADM. (GAVAGE)
INDIVIDUAL FOOD CONSUMPTION -- GRAMS/ANIMAL/DAY

FEMALES TEST GROUP 1 (ADI-MIX)

ANIMAL#	WEEK OF STUDY 0- 1
611	8.5
612	9.6
613	7.2
614	8.7
615	8.1
616	8.3
617	8.0
618	8.9
619	8.7
620	9.0
MEAN	8.5
S.D.	0.65
N	10

PR.NO.60R0375/88R002: REPRODUCTIVE TOX. STUDY TO DETECT EFFECTS
OF MIXED ANTI-ANDROGENIC SUBSTANCES IN RATS; ORAL ADM. (GAVAGE)
INDIVIDUAL FOOD CONSUMPTION -- GRAMS/ANIMAL/DAY

FEMALES TEST GROUP 2 (NOAEL-MIX)

ANIMAL#	WEEK OF STUDY 0- 1
621	9.3
622	8.8
623	8.7
624	7.9
625	9.0
626	9.0
627	7.8
628	8.5
629	9.0
630	8.6
MEAN	8.6
S.D.	0.50
N	10

PR.NO.60R0375/88R002: REPRODUCTIVE TOX. STUDY TO DETECT EFFECTS
OF MIXED ANTI-ANDROGENIC SUBSTANCES IN RATS; ORAL ADM. (GAVAGE)
INDIVIDUAL FOOD CONSUMPTION -- GRAMS/ANIMAL/DAY

FEMALES TEST GROUP 3 (LOAEL-MIX)

ANIMAL#	WEEK OF STUDY 0- 1
631	7.8
632	7.7
633	8.5
634	7.7
635	7.4
636	9.5
637	8.4
638	9.0
639	8.8
640	8.5
MEAN	8.3
S.D.	0.66
N	10

PR.NO.60R0375/88R002: REPRODUCTIVE TOX. STUDY TO DETECT EFFECTS
OF MIXED ANTI-ANDROGENIC SUBSTANCES IN RATS; ORAL ADM. (GAVAGE)
INDIVIDUAL FOOD CONSUMPTION -- GRAMS/ANIMAL/DAY

FEMALES TEST GROUP 4(0.00025 MG/KG BW/D)

ANIMAL#	WEEK OF STUDY 0- 1
641	8.9
642	8.7
643	9.9
644	7.2
645	8.5
646	7.7
647	8.3
648	8.8
649	9.5
650	7.5
MEAN	8.5
S.D.	0.87
N	10

PR.NO.60R0375/88R002: REPRODUCTIVE TOX. STUDY TO DETECT EFFECTS
OF MIXED ANTI-ANDROGENIC SUBSTANCES IN RATS; ORAL ADM. (GAVAGE)
INDIVIDUAL FOOD CONSUMPTION -- GRAMS/ANIMAL/DAY

MALES TEST GROUP 0 (0 MG/KG BW/D)

	WEEK OF STUDY								
ANIMAL#	0- 1	1- 2	2- 3	3- 4	4- 5	5- 6	6- 7	7- 8	8- 9
701	9.9	14.7	18.3	19.0	20.5	21.1	20.5	20.1	
702	8.8	14.6	19.9	20.6	21.5	20.9	21.0	20.0	
703	8.2								
704	8.4	12.9	16.1	17.6	18.7	18.5	17.8	18.0	
705	9.5	15.5	20.3	19.9	21.9	20.9	19.7	18.7	
706	9.2	14.2	19.6	21.1	23.2	21.9	22.4	22.4	
707	9.5	14.5	18.3	19.7	20.8	20.5	18.7	19.0	19.6
708	8.7	13.0	16.1	17.1	17.7	18.5	18.0	17.7	17.2
709	7.5	13.2	18.5	19.4	20.2	22.0	21.7	22.7	
710	8.4	13.4	17.7	19.5	20.0	20.5	20.5	20.0	
MEAN	8.8	14.0	18.3	19.3	20.5	20.5	20.0	19.9	18.4
S.D.	0.74	0.92	1.51	1.28	1.66	1.26	1.61	1.75	1.65
N	10	9	9	9	9	9	9	9	2

PR.NO.60R0375/88R002: REPRODUCTIVE TOX. STUDY TO DETECT EFFECTS
OF MIXED ANTI-ANDROGENIC SUBSTANCES IN RATS; ORAL ADM. (GAVAGE)
INDIVIDUAL FOOD CONSUMPTION -- GRAMS/ANIMAL/DAY

MALES TEST GROUP 1 (ADI-MIX)

	WEEK OF STUDY								
ANIMAL#	0- 1	1- 2	2- 3	3- 4	4- 5	5- 6	6- 7	7- 8	8- 9
711	8.3	13.6	18.5	18.4	19.3	17.4	16.0	16.2	16.4
712	9.0	13.5	17.1	17.7	17.3	18.7	17.6	17.1	
713	9.2	14.4	18.3	18.7	19.7	19.9	19.7	19.8	
714	10.0	15.3	20.0	21.2	21.2	22.4	21.9	22.4	
715	8.8	14.0	18.4	19.3	19.8	20.2	18.5	16.9	
716	9.0	14.4	18.4	19.3	19.8	20.3	20.1	19.0	
717	7.3	13.1	17.4	18.2	19.3	18.7	19.1	18.5	
718	9.1	13.1	16.7	16.7	18.0	18.5	17.1	17.6	17.8
719	9.5	15.1	20.5	21.2	21.4	20.9	20.9	20.3	
720	8.6	13.8	18.8	20.1	20.2	21.3	22.0	20.2	
MEAN	8.9	14.0	18.4	19.1	19.6	19.9	19.3	18.8	17.1
S.D.	0.72	0.77	1.17	1.44	1.26	1.51	2.02	1.92	1.04
N	10	10	10	10	10	10	10	10	2

PR.NO.60R0375/88R002: REPRODUCTIVE TOX. STUDY TO DETECT EFFECTS
OF MIXED ANTI-ANDROGENIC SUBSTANCES IN RATS; ORAL ADM. (GAVAGE)
INDIVIDUAL FOOD CONSUMPTION -- GRAMS/ANIMAL/DAY

MALES TEST GROUP 2 (NOAEL-MIX)

	WEEK OF STUDY								
ANIMAL#	0- 1	1- 2	2- 3	3- 4	4- 5	5- 6	6- 7	7- 8	8- 9
721	8.4	13.0	16.7	17.1	17.9	17.3	18.4	18.1	
722	9.0	14.4	17.8	18.4	20.1	20.3	19.3	19.1	
723	9.1	13.6	18.3	20.1	20.7	21.2	20.7	21.5	
724	8.7	13.1	16.8	18.2	20.1	19.9	18.1	18.5	
725	8.7	13.1	17.4	18.2	20.3	20.0	18.1	18.3	19.3
726	8.8	13.9	15.2	17.3	18.9	19.4	17.3	17.0	16.8
727	9.9	15.4	19.5	19.9	22.0	22.8	22.2	22.8	22.8
728	9.1	14.3	19.0	20.1	21.4	23.1	22.1	22.0	22.3
729	8.8	14.0	17.5	18.4	19.4	19.8	19.5	18.5	
730	9.1	13.8	16.7	16.6	16.6	17.5	17.1	17.0	
MEAN	9.0	13.9	17.5	18.4	19.7	20.1	19.3	19.3	20.3
S.D.	0.40	0.74	1.24	1.25	1.62	1.91	1.86	2.07	2.79
N	10	10	10	10	10	10	10	10	4

PR.NO.60R0375/88R002: REPRODUCTIVE TOX. STUDY TO DETECT EFFECTS
OF MIXED ANTI-ANDROGENIC SUBSTANCES IN RATS; ORAL ADM. (GAVAGE)
INDIVIDUAL FOOD CONSUMPTION -- GRAMS/ANIMAL/DAY

MALES TEST GROUP 3 (LOAEL-MIX)

ANIMAL#	WEEK OF STUDY 0- 1	1- 2	2- 3	3- 4	4- 5	5- 6	6- 7	7- 8	8- 9
731	9.1	13.6	17.6	18.5	19.1	19.3	19.2	19.0	
732	8.8	14.2	18.1	19.6	20.5	20.7	19.9	19.1	
733	9.0	13.6	18.5	19.2	19.8	20.8	19.6	17.9	18.5
734	9.2	13.4	16.7	18.1	20.3	19.5	19.8	19.4	19.5
735	8.3	13.8	18.4	19.4	21.5	23.2	22.8	23.4	23.8
736	8.3	11.7	15.3	16.0	16.4	17.8	16.2	16.0	
737	8.6	7.5	15.0	16.2	17.0	18.1	18.0	17.2	
738	9.2	15.1	18.1	20.9	21.8	22.6	20.7	20.7	
739	9.3	13.7	16.5	17.8	19.3	19.9	20.8	20.4	
740	8.8	13.3	16.4	19.0	19.3	20.9	22.4	22.7	
MEAN	8.9	13.0	17.1	18.5	19.5	20.3	19.9	19.6	20.6
S.D.	0.35	2.10	1.29	1.49	1.74	1.75	1.95	2.32	2.82
N	10	10	10	10	10	10	10	10	3

PR.NO.60R0375/88R002: REPRODUCTIVE TOX. STUDY TO DETECT EFFECTS
OF MIXED ANTI-ANDROGENIC SUBSTANCES IN RATS; ORAL ADM. (GAVAGE)
INDIVIDUAL FOOD CONSUMPTION -- GRAMS/ANIMAL/DAY

MALES TEST GROUP 4(0.00025 MG/KG BW/D)

	WEEK OF STUDY								
ANIMAL#	0- 1	1- 2	2- 3	3- 4	4- 5	5- 6	6- 7	7- 8	8- 9
741	9.8	14.7	20.1	21.3	23.5	23.2	21.1	20.4	19.7
742	9.5	14.3	18.9	19.3	19.7	19.6	18.9	18.6	
743	9.1	14.6	18.5	18.4	18.3	18.9	17.9	19.5	
744	9.7	14.9	20.5	21.3	22.3	21.7	21.8	21.6	
745	9.4	14.0	18.4	19.9	22.2	21.3	22.6	22.3	22.7
746	8.7	14.8	20.2	20.4	21.4	22.3	22.1	21.7	21.5
747	11.4	17.7	21.9	22.8	22.1	21.3	18.2	17.7	
748	8.6	14.3	17.5	18.6	18.1	18.8	18.6	18.7	
749	9.4	14.2	18.2	20.5	20.7	22.2	21.3	21.4	
750	9.5	14.7	18.8	19.6	19.6	21.6	20.1	20.1	
MEAN	9.5	14.8	19.3	20.2	20.8	21.1	20.3	20.2	21.3
S.D.	0.77	1.05	1.33	1.34	1.83	1.51	1.74	1.57	1.53
N	10	10	10	10	10	10	10	10	3

PR.NO.60R0375/88R002: REPRODUCTIVE TOX. STUDY TO DETECT EFFECTS
OF MIXED ANTI-ANDROGENIC SUBSTANCES IN RATS; ORAL ADM. (GAVAGE)
INDIVIDUAL FOOD CONSUMPTION -- GRAMS/ANIMAL/DAY

FEMALES TEST GROUP 0 (0 MG/KG BW/D)

ANIMAL#	WEEK OF STUDY 0- 1	1- 2	2- 3	3- 4	4- 5	5- 6	6- 7	7- 8	8- 9
801	8.5	12.6	14.0	13.6	13.8	14.0	13.2	14.4	
802	9.0	13.0	15.1	14.4	14.5	14.2	13.9	14.7	
803	8.0	11.7	13.0	12.3	11.9	12.0	11.9	11.6	
804	8.0	12.3	13.7	14.4	14.4	15.6	14.9	16.9	
805	6.8								
806	8.4	12.7	14.6	14.2	14.3	14.4	14.8	15.0	
807	7.4	8.0	11.6	13.9	14.1	13.5	15.0	13.2	13.9
808	8.8	13.1	14.2	13.2	12.5	12.1	12.7	15.8	11.4
809	8.8	12.7	14.5	13.8	13.8	14.3	15.1	15.8	15.1
810	8.6	12.5	12.7	13.7	13.3	14.1	15.8	14.3	
MEAN	8.2	12.1	13.7	13.7	13.6	13.8	14.1	14.6	13.5
S.D.	0.71	1.57	1.10	0.65	0.90	1.15	1.29	1.56	1.88
N	10	9	9	9	9	9	9	9	3

PR.NO.60R0375/88R002: REPRODUCTIVE TOX. STUDY TO DETECT EFFECTS
OF MIXED ANTI-ANDROGENIC SUBSTANCES IN RATS; ORAL ADM. (GAVAGE)
INDIVIDUAL FOOD CONSUMPTION -- GRAMS/ANIMAL/DAY

FEMALES TEST GROUP 1 (ADI-MIX)

	WEEK OF STUDY								
ANIMAL#	0- 1	1- 2	2- 3	3- 4	4- 5	5- 6	6- 7	7- 8	8- 9
811	9.2	14.5	16.7	15.5	14.4	14.3	13.5	13.7	
812	9.1	13.1	14.7	13.7	13.7	13.9	13.2	13.6	
813	8.0	12.3	14.1	14.2	15.0	15.4	14.5	14.4	
814	7.0	8.7	12.2	13.7	13.0	13.6	12.8	13.7	
815	8.3	12.0	13.7	13.5	13.3	13.6	14.3	15.8	13.1
816	7.8	12.4	14.6	14.0	13.9	14.1	14.1	14.3	
817	7.4	11.7	13.7	13.8	13.8	14.2	16.9	12.2	
818	8.8	12.5	13.7	14.3	14.4	14.6	17.1	15.1	
819	8.2	11.7	13.5	13.0	12.7	12.7	13.0	14.6	11.3
820	7.9	12.0	15.1	14.4	15.6	14.7	17.8	14.9	15.2
MEAN	8.2	12.1	14.2	14.0	14.0	14.1	14.7	14.2	13.2
S.D.	0.70	1.44	1.19	0.68	0.91	0.74	1.85	1.01	1.97
N	10	10	10	10	10	10	10	10	3

PR.NO.60R0375/88R002: REPRODUCTIVE TOX. STUDY TO DETECT EFFECTS
OF MIXED ANTI-ANDROGENIC SUBSTANCES IN RATS; ORAL ADM. (GAVAGE)
INDIVIDUAL FOOD CONSUMPTION -- GRAMS/ANIMAL/DAY

FEMALES TEST GROUP 2 (NOAEL-MIX)

	WEEK OF STUDY								
ANIMAL#	0- 1	1- 2	2- 3	3- 4	4- 5	5- 6	6- 7	7- 8	8- 9
821	8.4	12.3	14.8	14.9	14.6	14.3	13.6	13.7	
822	8.3	11.4	13.6	13.1	12.9	13.9	13.4	13.4	
823	8.4	12.9	14.4	14.9	15.1	15.1	17.2	17.6	
824	8.0	10.9	13.2	12.9	13.2	13.4	15.8	13.0	
825	7.7	8.8	13.8	15.7	14.7	14.7	15.4	18.4	
826	8.6	12.5	13.9	13.9	14.8	14.9	15.8	16.0	14.5
827	8.0	11.0	11.9	11.4	12.1	12.7	14.5	13.2	12.3
828	9.0	12.8	14.0	15.0	14.7	15.2	17.1	15.2	
829	8.8	12.6	13.8	15.1	15.3	15.5	15.9	15.5	
830	9.5	14.2	16.4	15.9	15.9	16.0	16.4	16.7	
MEAN	8.5	11.9	14.0	14.3	14.3	14.6	15.5	15.3	13.4
S.D.	0.53	1.49	1.15	1.42	1.19	0.99	1.32	1.93	1.52
N	10	10	10	10	10	10	10	10	2

PR.NO.60R0375/88R002: REPRODUCTIVE TOX. STUDY TO DETECT EFFECTS
OF MIXED ANTI-ANDROGENIC SUBSTANCES IN RATS; ORAL ADM. (GAVAGE)
INDIVIDUAL FOOD CONSUMPTION -- GRAMS/ANIMAL/DAY

FEMALES TEST GROUP 3 (LOAEL-MIX)

	WEEK OF STUDY								
ANIMAL#	0- 1	1- 2	2- 3	3- 4	4- 5	5- 6	6- 7	7- 8	8- 9
831	7.9	11.5	13.7	13.9	13.6	14.0	17.5	15.2	
832	8.7	12.8	14.9	15.0	14.1	13.9	16.1	13.6	
833	8.4	12.1	13.4	13.2	14.3	14.2	15.6	14.4	14.1
834	9.0	12.2	13.9	14.0	14.6	15.0	15.4	16.3	15.7
835	9.1	13.3	15.6	16.1	18.2	17.0	19.7	18.1	16.0
836	7.9	11.4	13.2	14.8	14.1	14.5	16.0	12.6	
837	8.9	12.3	14.0	14.3	14.1	14.7	15.5	15.6	
838	8.7	12.1	13.1	12.9	13.7	13.2	15.7	16.0	
839	8.5	12.4	12.8	13.8	13.4	13.5	13.4	13.1	
840	10.0	13.5	13.9	14.3	14.2	15.1	16.3	16.1	
MEAN	8.7	12.4	13.9	14.2	14.4	14.5	16.1	15.1	15.3
S.D.	0.60	0.67	0.85	0.92	1.35	1.07	1.60	1.67	0.99
N	10	10	10	10	10	10	10	10	3

PR.NO.60R0375/88R002: REPRODUCTIVE TOX. STUDY TO DETECT EFFECTS
OF MIXED ANTI-ANDROGENIC SUBSTANCES IN RATS; ORAL ADM. (GAVAGE)
INDIVIDUAL FOOD CONSUMPTION -- GRAMS/ANIMAL/DAY

FEMALES TEST GROUP 4(0.00025 MG/KG BW/D)

	WEEK OF STUDY								
ANIMAL#	0- 1	1- 2	2- 3	3- 4	4- 5	5- 6	6- 7	7- 8	8- 9
841	8.3	11.4	12.4	11.6	12.1	11.9	14.0	10.9	
842	9.0	12.6	15.3	14.8	15.2	15.1	14.4	16.5	
843	8.4	12.9	13.9	13.6	13.8	13.3	13.4	14.5	
844	9.3	13.0	14.5	13.1	12.9	13.5	13.2	13.1	
845	9.6	13.9	15.8	15.0	15.5	15.2	16.4	16.3	
846	7.4	11.3	13.3	13.6	13.5	13.1	15.2	17.4	
847	7.4	11.2	14.0	13.2	13.6	13.0	13.9	15.0	13.1
848	10.2	14.6	16.5	16.6	15.2	16.2	16.3	15.5	
849	9.0	12.7	13.9	14.1	13.6	13.4	13.4	13.3	
850	9.7	14.4	15.8	14.8	15.6	16.3	15.6	16.5	
MEAN	8.8	12.8	14.5	14.0	14.1	14.1	14.6	14.9	13.1
S.D.	0.96	1.26	1.27	1.34	1.19	1.51	1.22	1.98	0.00
N	10	10	10	10	10	10	10	10	1

PR.NO.60R0375/88R002: REPRODUCTIVE TOX. STUDY TO DETECT EFFECTS
OF MIXED ANTI-ANDROGENIC SUBSTANCES IN RATS; ORAL ADM. (GAVAGE)
INDIVIDUAL BODY WEIGHTS -- GRAMS

MALES TEST GROUP 0 (0 MG/KG BW/D)

	WEEK OF STUDY					
ANIMAL#	0	1	2	3	4	5
501	42.0	73.0	118.6	165.1	209.3	
502	39.5	73.1	117.6	162.0		
503	44.6	76.0	123.5	164.7		
504	45.2	72.4	109.6	136.6	166.4	
505	37.6	67.3	110.9	152.4		
506	44.7	77.1	125.8	172.8		
507	43.0	71.3	109.8	145.7		
508	46.8	83.8	133.1	172.6	206.6	
509	43.5	78.8	122.0	157.9		
510	47.8	85.1	129.7	167.4		
MEAN	43.5	75.8	120.1	159.7	194.1	
S.D.	3.13	5.58	8.29	11.75	24.03	
N	10	10	10	10	3	

PR.NO.60R0375/88R002: REPRODUCTIVE TOX. STUDY TO DETECT EFFECTS
OF MIXED ANTI-ANDROGENIC SUBSTANCES IN RATS; ORAL ADM. (GAVAGE)
INDIVIDUAL BODY WEIGHTS -- GRAMS

MALES TEST GROUP 1 (ADI-MIX)

ANIMAL#	WEEK OF STUDY 0	1	2	3	4	5
511	43.7	72.4	115.7	157.7		
512	50.1	85.6	133.6	176.0		
513	50.4	87.5	137.2	188.3		
514	48.6	78.3	128.7	174.9		
515	42.5	75.6	123.0	162.5		
516	53.2	84.0	129.8	177.3		
517	37.7	68.2	112.1	151.1		
518	37.6	70.9	107.8	147.3	186.2	
519	47.5	80.3	122.0	171.5		
520	43.1	74.9	124.0	170.6		
MEAN	45.4	77.8	123.4	167.7	186.2	
S.D.	5.37	6.52	9.41	12.82	0.00	
N	10	10	10	10	1	

PR.NO.60R0375/88R002: REPRODUCTIVE TOX. STUDY TO DETECT EFFECTS
OF MIXED ANTI-ANDROGENIC SUBSTANCES IN RATS; ORAL ADM. (GAVAGE)
INDIVIDUAL BODY WEIGHTS -- GRAMS

MALES TEST GROUP 2 (NOAEL-MIX)

	WEEK OF STUDY					
ANIMAL#	0	1	2	3	4	5
521	43.7	74.7	119.7	162.6		
522	39.7	70.8	112.7	158.8		
523	47.1	78.9	125.1	173.6		
524	50.4	81.5	130.3	171.5		
525	47.2	83.3	130.3	181.4		
526	48.4	77.6	122.8	167.3	207.0	
527	44.6	77.0	125.8	167.6		
528	54.5	86.8	132.7	173.5		
529	46.9	77.3	120.3	159.2	196.6	
530	40.0	73.7	111.8	151.7		
MEAN	46.3	78.2	123.2	166.7	201.8	
S.D.	4.51	4.73	7.17	8.79	7.35	
N	10	10	10	10	2	

PR.NO.60R0375/88R002: REPRODUCTIVE TOX. STUDY TO DETECT EFFECTS
OF MIXED ANTI-ANDROGENIC SUBSTANCES IN RATS; ORAL ADM. (GAVAGE)
INDIVIDUAL BODY WEIGHTS -- GRAMS

MALES TEST GROUP 3 (LOAEL-MIX)

	WEEK OF STUDY					
ANIMAL#	0	1	2	3	4	5
531	46.7	71.3	113.9	156.5	190.6	222.9
532	50.4	82.9	124.1	166.5	201.3	
533	47.0	77.7	121.0	157.0		
534	46.8	76.6	115.8	146.0	183.6	
535	45.9	69.4	110.5	142.5	179.0	
536	46.3	78.7	123.4	168.5	210.5	251.7
537	51.7	79.6	120.7	158.7	199.7	
538	46.9	72.1	111.2	146.4	177.9	211.0
539	46.2	72.6	114.0	146.5	178.0	209.8
540	50.6	81.1	123.0	164.3	199.1	237.9
MEAN	47.9	76.2	117.8	155.3	191.1	226.7
S.D.	2.16	4.58	5.24	9.45	12.08	18.01
N	10	10	10	10	9	5

PR.NO.60R0375/88R002: REPRODUCTIVE TOX. STUDY TO DETECT EFFECTS
OF MIXED ANTI-ANDROGENIC SUBSTANCES IN RATS; ORAL ADM. (GAVAGE)
INDIVIDUAL BODY WEIGHTS -- GRAMS

MALES TEST GROUP 4(0.00025 MG/KG BW/D)

	WEEK OF STUDY					
ANIMAL#	0	1	2	3	4	5
541	37.9	66.2	109.8	156.5		
542	39.5	71.1	111.5	148.7		
543	47.3	79.4	123.4	168.7		
544	53.2	86.4	131.9	179.4		
545	53.8	94.6	147.1			
546	45.9	79.1	131.3	170.6		
547	44.2	81.7	135.3			
548	57.2	93.1	140.2	183.4		
549	53.0	90.0	140.4	187.6		
550	46.7	86.3	135.0	179.5	216.3	
MEAN	47.9	82.8	130.6	171.8	216.3	
S.D.	6.37	9.20	12.26	13.52	0.00	
N	10	10	10	8	1	

PR.NO.60R0375/88R002: REPRODUCTIVE TOX. STUDY TO DETECT EFFECTS
OF MIXED ANTI-ANDROGENIC SUBSTANCES IN RATS; ORAL ADM. (GAVAGE)
INDIVIDUAL BODY WEIGHTS -- GRAMS

FEMALES TEST GROUP 0 (0 MG/KG BW/D)

ANIMAL#	WEEK OF STUDY 0	1
601	50.6	80.6
602	44.0	70.5
603	43.6	72.3
604	51.5	76.4
605	42.1	69.2
606	38.7	68.8
607	49.3	83.6
608	42.4	71.9
609	41.4	68.0
610	40.4	73.3
MEAN	44.4	73.5
S.D.	4.48	5.21
N	10	10

PR.NO.60R0375/88R002: REPRODUCTIVE TOX. STUDY TO DETECT EFFECTS
OF MIXED ANTI-ANDROGENIC SUBSTANCES IN RATS; ORAL ADM. (GAVAGE)
INDIVIDUAL BODY WEIGHTS -- GRAMS

FEMALES TEST GROUP 1 (ADI-MIX)

ANIMAL#	WEEK OF STUDY 0	1
611	40.3	70.5
612	47.8	81.6
613	38.7	62.8
614	50.8	82.2
615	33.5	59.0
616	47.6	77.0
617	47.1	75.4
618	49.1	78.9
619	43.5	75.6
620	41.5	72.4
MEAN	44.0	73.5
S.D.	5.45	7.64
N	10	10

PR.NO.60R0375/88R002: REPRODUCTIVE TOX. STUDY TO DETECT EFFECTS
OF MIXED ANTI-ANDROGENIC SUBSTANCES IN RATS; ORAL ADM. (GAVAGE)
INDIVIDUAL BODY WEIGHTS -- GRAMS

FEMALES TEST GROUP 2 (NOAEL-MIX)

	WEEK OF STUDY	
ANIMAL#	0	1
621	45.5	78.4
622	46.3	75.7
623	48.8	78.4
624	34.9	64.0
625	45.7	77.0
626	47.0	75.4
627	44.2	71.9
628	45.2	74.7
629	43.6	74.4
630	40.9	70.2
MEAN	44.2	74.0
S.D.	3.89	4.37
N	10	10

PR.NO.60R0375/88R002: REPRODUCTIVE TOX. STUDY TO DETECT EFFECTS
OF MIXED ANTI-ANDROGENIC SUBSTANCES IN RATS; ORAL ADM. (GAVAGE)
INDIVIDUAL BODY WEIGHTS -- GRAMS

FEMALES TEST GROUP 3 (LOAEL-MIX)

ANIMAL#	WEEK OF STUDY 0	1
631	44.8	68.4
632	41.7	66.6
633	50.3	78.4
634	55.3	78.9
635	42.2	67.3
636	49.1	79.7
637	46.6	71.9
638	45.8	77.1
639	44.1	70.4
640	47.2	75.1
MEAN	46.7	73.4
S.D.	4.07	5.07
N	10	10

PR.NO.60R0375/88R002: REPRODUCTIVE TOX. STUDY TO DETECT EFFECTS
OF MIXED ANTI-ANDROGENIC SUBSTANCES IN RATS; ORAL ADM. (GAVAGE)
INDIVIDUAL BODY WEIGHTS -- GRAMS

FEMALES TEST GROUP 4(0.00025 MG/KG BW/D)

	WEEK OF STUDY	
ANIMAL#	0	1
641	39.4	70.6
642	52.2	83.7
643	47.1	81.8
644	42.2	67.9
645	48.2	73.2
646	40.0	69.4
647	46.2	70.6
648	56.0	83.1
649	43.2	76.7
650	38.1	64.5
MEAN	45.3	74.2
S.D.	5.81	6.81
N	10	10

PR.NO.60R0375/88R002: REPRODUCTIVE TOX. STUDY TO DETECT EFFECTS
OF MIXED ANTI-ANDROGENIC SUBSTANCES IN RATS; ORAL ADM. (GAVAGE)
INDIVIDUAL BODY WEIGHTS -- GRAMS

MALES TEST GROUP 0 (0 MG/KG BW/D)

	WEEK OF STUDY									
ANIMAL#	0	1	2	3	4	5	6	7	8	9
701	50.8	85.2	133.3	177.9	213.7	251.8	280.6	309.7	328.8	
702	44.1	75.9	118.8	158.9	202.0	240.2	259.6	287.7	304.6	
703	44.2	74.2								
704	45.7	76.1	115.8	152.0	186.7	219.6	243.9	256.3	275.6	
705	42.6	81.0	126.9	170.7	212.2	251.6	278.5	298.8	310.7	
706	45.1	80.8	125.0	175.7	223.3	266.1	300.3	332.5	359.6	
707	49.4	82.4	126.7	163.9	204.5	241.3	264.8	283.9	302.8	316.1
708	41.0	73.4	115.7	151.7	186.9	215.6	238.6	259.1	279.1	283.9
709	41.4	67.9	110.7	154.4	193.7	231.9	261.7	288.3	319.2	
710	43.1	71.5	115.5	157.9	197.6	235.9	260.7	284.6	301.9	
MEAN	44.7	76.8	120.9	162.6	202.3	239.3	265.4	289.0	309.1	300.0
S.D.	3.21	5.40	7.34	10.04	12.50	15.99	18.94	23.53	25.44	22.77
N	10	10	9	9	9	9	9	9	9	2

PR.NO.60R0375/88R002: REPRODUCTIVE TOX. STUDY TO DETECT EFFECTS
OF MIXED ANTI-ANDROGENIC SUBSTANCES IN RATS; ORAL ADM. (GAVAGE)
INDIVIDUAL BODY WEIGHTS -- GRAMS

MALES TEST GROUP 1 (ADI-MIX)

ANIMAL#	WEEK OF STUDY 0	1	2	3	4	5	6	7	8	9
711	41.6	72.5	119.5	160.5	192.7	221.4	237.9	250.7	259.5	272.5
712	44.7	77.4	119.6	160.8	194.2	223.6	253.4	274.2	291.9	
713	52.2	82.4	122.8	167.6	204.9	237.9	260.8	284.9	308.2	
714	54.6	92.3	140.9	195.3	239.2	279.7	318.7	352.6	374.1	
715	47.7	81.7	122.2	162.5	198.9	231.8	256.6	272.2	289.3	
716	45.0	78.5	122.6	166.2	199.5	239.0	269.8	290.7	312.0	
717	38.2	66.3	109.8	151.9	191.3	225.6	251.5	273.6	295.8	
718	39.4	69.1	107.3	145.8	176.8	207.8	232.2	250.2	268.0	285.7
719	44.4	77.4	123.8	171.7	216.5	253.1	277.3	298.0	316.7	
720	41.6	74.0	121.6	163.7	202.4	236.6	264.6	281.9	299.3	
MEAN	44.9	77.2	121.0	164.6	201.6	235.7	262.3	282.9	301.5	279.1
S.D.	5.30	7.41	9.00	13.15	16.71	19.73	24.04	28.93	31.31	9.33
N	10	10	10	10	10	10	10	10	10	2

PR.NO.60R0375/88R002: REPRODUCTIVE TOX. STUDY TO DETECT EFFECTS
OF MIXED ANTI-ANDROGENIC SUBSTANCES IN RATS; ORAL ADM. (GAVAGE)
INDIVIDUAL BODY WEIGHTS -- GRAMS

MALES TEST GROUP 2 (NOAEL-MIX)

ANIMAL#	WEEK OF STUDY 0	1	2	3	4	5	6	7	8	9
721	49.2	77.0	113.5	153.6	186.4	218.3	226.5	267.1	288.8	
722	47.2	78.9	121.5	161.5	194.2	228.8	255.2	276.5	294.0	
723	44.5	76.3	120.7	168.6	216.9	249.6	282.3	308.6	334.6	
724	47.1	78.8	121.3	165.2	202.5	238.2	269.6	288.0	302.2	
725	50.4	80.1	122.7	167.9	202.1	237.9	265.8	288.7	310.8	333.1
726	46.6	80.0	121.7	159.4	183.9	221.2	247.8	262.6	277.6	280.0
727	49.1	82.9	131.1	177.1	214.9	264.4	294.8	324.6	351.1	367.4
728	48.6	84.6	130.3	176.9	215.0	252.0	284.3	316.5	337.4	361.8
729	41.9	72.4	117.0	158.0	195.4	235.8	265.4	288.8	304.8	
730	39.2	72.3	115.8	152.3	174.7	204.2	226.4	248.2	262.2	
MEAN	46.4	78.3	121.6	164.1	198.6	235.0	261.8	287.0	306.4	335.6
S.D.	3.54	4.00	5.66	8.72	14.39	17.77	23.20	24.41	27.97	39.98
N	10	10	10	10	10	10	10	10	10	4

PR.NO.60R0375/88R002: REPRODUCTIVE TOX. STUDY TO DETECT EFFECTS
OF MIXED ANTI-ANDROGENIC SUBSTANCES IN RATS; ORAL ADM. (GAVAGE)
INDIVIDUAL BODY WEIGHTS -- GRAMS

MALES TEST GROUP 3 (LOAEL-MIX)

ANIMAL#	WEEK OF STUDY 0	1	2	3	4	5	6	7	8	9
731	52.6	83.0	124.9	157.7	195.5	228.6	258.9	276.9	294.7	
732	46.1	77.8	119.3	159.5	202.5	233.8	263.5	283.6	298.4	
733	49.4	80.7	126.4	171.6	213.1	243.3	266.8	287.8	300.8	306.4
734	60.5	90.3	130.5	166.9	204.3	241.9	265.4	289.1	306.0	320.7
735	41.7	70.3	116.2	157.4	198.3	241.0	277.5	309.3	339.8	364.0
736	47.2	71.9	109.4	146.3	175.9	209.6	227.1	242.6	252.9	
737	49.9	76.8	91.3	131.1	163.9	194.8	221.6	241.9	258.2	
738	47.3	77.3	126.7	166.0	205.3	244.6	281.5	302.4	323.7	
739	47.5	80.0	123.2	164.0	201.6	234.3	264.4	292.2	312.7	
740	46.2	76.5	116.6	158.2	198.0	231.2	257.3	288.1	307.3	
MEAN	48.8	78.5	118.4	157.9	195.8	230.3	258.4	281.4	299.5	330.4
S.D.	4.99	5.64	11.40	11.68	14.78	16.14	19.48	22.54	26.65	29.99
N	10	10	10	10	10	10	10	10	10	3

PR.NO.60R0375/88R002: REPRODUCTIVE TOX. STUDY TO DETECT EFFECTS
OF MIXED ANTI-ANDROGENIC SUBSTANCES IN RATS; ORAL ADM. (GAVAGE)
INDIVIDUAL BODY WEIGHTS -- GRAMS

MALES TEST GROUP 4(0.00025 MG/KG BW/D)

ANIMAL#	WEEK OF STUDY 0	1	2	3	4	5	6	7	8	9
741	42.6	76.9	125.2	177.2	222.0	272.1	297.6	313.8	327.4	347.1
742	47.3	79.1	125.3	167.8	207.8	248.5	270.9	293.8	312.2	
743	47.8	80.2	123.1	164.0	194.0	224.0	248.2	268.1	278.9	
744	42.2	78.4	127.9	178.0	223.2	265.1	297.9	323.1	352.2	
745	48.9	78.6	118.8	161.4	201.0	242.8	270.0	300.2	320.3	341.9
746	44.8	78.6	132.1	183.7	226.1	264.9	301.4	332.9	356.2	370.0
747	60.5	101.5	153.0	196.3	242.4	278.9	290.8	302.1	317.8	
748	53.0	81.4	122.4	165.4	193.7	227.1	246.1	260.5	279.2	
749	43.3	74.8	116.0	157.3	196.8	234.8	262.6	289.8	322.2	
750	44.2	77.7	120.3	159.9	192.6	230.4	254.3	272.5	287.3	
MEAN	47.5	80.7	126.4	171.1	210.0	248.9	274.0	295.7	315.4	353.0
S.D.	5.67	7.52	10.41	12.39	17.36	20.08	21.47	23.81	27.25	14.95
N	10	10	10	10	10	10	10	10	10	3

PR.NO.60R0375/88R002: REPRODUCTIVE TOX. STUDY TO DETECT EFFECTS
OF MIXED ANTI-ANDROGENIC SUBSTANCES IN RATS; ORAL ADM. (GAVAGE)
INDIVIDUAL BODY WEIGHTS -- GRAMS

FEMALES TEST GROUP 0 (0 MG/KG BW/D)

	WEEK OF STUDY									
ANIMAL#	0	1	2	3	4	5	6	7	8	9
801	40.3	70.8	106.8	129.6	139.9	155.6	169.0	176.1	186.4	
802	46.9	77.6	109.7	135.9	152.5	169.1	183.4	192.7	197.0	
803	41.3	68.9	98.4	120.2	133.1	136.8	158.8	164.1	167.5	
804	53.2	79.7	117.1	144.1	160.7	176.4	190.6	204.4	219.2	
805	44.5	67.2								
806	42.8	73.5	108.3	129.3	146.9	164.7	176.1	184.5	196.2	
807	41.8	66.8	79.3	108.2	130.9	146.5	162.8	178.3	186.4	193.3
808	45.7	75.3	108.7	133.7	149.0	160.5	168.3	184.8	203.7	197.0
809	45.2	78.0	110.0	134.5	150.5	160.7	180.2	200.3	208.1	213.4
810	46.0	73.9	103.7	117.6	137.3	152.1	165.8	188.5	191.1	
MEAN	44.8	73.2	104.7	128.1	144.5	158.0	172.8	186.0	195.1	201.2
S.D.	3.70	4.60	10.76	10.96	9.85	11.95	10.46	12.42	14.82	10.70
N	10	10	9	9	9	9	9	9	9	3

PR.NO.60R0375/88R002: REPRODUCTIVE TOX. STUDY TO DETECT EFFECTS
OF MIXED ANTI-ANDROGENIC SUBSTANCES IN RATS; ORAL ADM. (GAVAGE)
INDIVIDUAL BODY WEIGHTS -- GRAMS

FEMALES TEST GROUP 1 (ADI-MIX)

ANIMAL#	WEEK OF STUDY 0	1	2	3	4	5	6	7	8	9
811	50.1	84.5	126.2	159.0	174.8	181.9	202.4	212.3	216.5	
812	48.3	79.0	115.4	135.7	157.3	172.3	176.9	191.6	201.3	
813	43.5	72.1	105.1	133.1	151.3	161.8	182.8	194.1	199.8	
814	29.4	53.9	73.2	105.9	125.1	141.3	155.3	157.8	173.8	
815	46.8	73.9	106.4	127.3	143.3	156.1	166.3	176.6	199.1	193.3
816	39.5	68.5	104.1	129.2	146.3	159.6	170.9	186.3	186.2	
817	50.4	77.1	110.9	134.3	153.6	164.2	182.5	210.2	200.7	
818	45.8	71.4	112.7	122.4	142.8	160.0	177.2	197.2	204.2	
819	49.2	76.6	105.6	129.4	147.1	158.6	170.5	185.7	200.5	194.0
820	42.0	68.2	109.3	130.3	143.0	162.4	172.5	197.4	189.5	206.1
MEAN	44.5	72.5	106.9	130.7	148.5	161.8	175.7	190.9	197.2	197.8
S.D.	6.41	8.22	13.54	13.10	12.68	10.50	12.37	15.89	11.55	7.20
N	10	10	10	10	10	10	10	10	10	3

PR.NO.60R0375/88R002: REPRODUCTIVE TOX. STUDY TO DETECT EFFECTS
OF MIXED ANTI-ANDROGENIC SUBSTANCES IN RATS; ORAL ADM. (GAVAGE)
INDIVIDUAL BODY WEIGHTS -- GRAMS

FEMALES TEST GROUP 2 (NOAEL-MIX)

	WEEK OF STUDY									
ANIMAL#	0	1	2	3	4	5	6	7	8	9
821	44.1	71.4	105.0	134.4	154.1	169.3	177.6	188.8	198.5	
822	44.0	75.3	107.1	133.3	145.3	152.9	177.5	184.7	191.2	
823	41.2	73.2	113.3	138.1	157.9	177.9	183.3	218.9	228.5	
824	49.2	75.9	102.6	124.7	141.8	153.1	170.8	192.6	191.7	
825	35.6	61.5	77.8	118.6	144.6	157.4	176.6	193.9	218.2	
826	47.6	77.7	116.1	138.1	157.7	171.4	190.2	212.3	217.2	228.4
827	41.0	65.4	97.6	116.2	130.6	146.9	151.3	177.8	179.9	187.4
828	49.5	78.2	113.7	134.0	151.1	166.0	182.5	203.4	208.9	
829	42.6	73.4	104.5	127.0	147.2	164.0	176.0	187.9	197.7	
830	44.7	81.4	121.8	149.1	170.7	189.4	199.5	211.3	223.5	
MEAN	44.0	73.3	105.9	131.4	150.1	164.8	178.5	197.2	205.5	207.9
S.D.	4.22	6.01	12.23	9.90	10.91	12.89	12.60	13.57	16.07	28.99
N	10	10	10	10	10	10	10	10	10	2

PR.NO.60R0375/88R002: REPRODUCTIVE TOX. STUDY TO DETECT EFFECTS
OF MIXED ANTI-ANDROGENIC SUBSTANCES IN RATS; ORAL ADM. (GAVAGE)
INDIVIDUAL BODY WEIGHTS -- GRAMS

FEMALES TEST GROUP 3 (LOAEL-MIX)

ANIMAL#	WEEK OF STUDY 0	1	2	3	4	5	6	7	8	9
831	46.2	71.7	102.5	123.5	142.6	151.5	163.7	185.5	186.6	
832	52.3	81.8	112.8	136.2	151.7	163.1	181.5	201.9	198.9	
833	43.2	70.7	98.3	120.4	136.8	157.1	172.3	195.8	201.9	208.9
834	43.8	71.7	108.1	127.1	144.3	159.2	182.7	193.8	202.6	211.9
835	43.4	73.9	109.8	136.6	148.9	178.8	197.0	221.9	224.6	235.1
836	49.8	75.7	106.0	133.9	154.3	169.2	183.1	199.0	196.3	
837	47.0	74.7	109.0	133.7	151.3	161.6	177.3	199.0	204.9	
838	45.9	71.7	103.6	126.4	141.7	152.8	170.2	192.9	197.2	
839	42.9	71.1	102.6	122.3	142.4	158.7	172.0	178.1	189.3	
840	48.0	82.9	115.8	140.5	157.1	162.0	188.2	211.8	209.8	
MEAN	46.3	74.6	106.8	130.1	147.1	161.4	178.8	198.0	201.2	218.6
S.D.	3.12	4.41	5.29	6.96	6.49	7.95	9.74	12.38	10.74	14.34
N	10	10	10	10	10	10	10	10	10	3

PR.NO.60R0375/88R002: REPRODUCTIVE TOX. STUDY TO DETECT EFFECTS
OF MIXED ANTI-ANDROGENIC SUBSTANCES IN RATS; ORAL ADM. (GAVAGE)
INDIVIDUAL BODY WEIGHTS -- GRAMS

FEMALES TEST GROUP 4(0.00025 MG/KG BW/D)

	WEEK OF STUDY									
ANIMAL#	0	1	2	3	4	5	6	7	8	9
841	40.7	69.0	96.1	115.6	126.1	139.8	155.3	173.7	171.8	
842	46.2	75.0	111.7	136.4	149.5	169.8	184.6	194.8	208.4	
843	51.8	80.3	114.3	132.9	141.9	157.3	169.4	172.4	189.1	
844	53.3	84.3	121.5	138.5	158.4	170.5	184.7	197.3	204.7	
845	51.5	87.2	121.9	145.0	161.2	179.5	186.3	203.5	214.2	
846	46.0	68.6	103.1	125.0	143.9	164.0	173.2	194.1	206.5	
847	45.4	70.8	103.7	131.3	148.6	167.7	174.2	189.2	209.9	208.9
848	59.7	93.8	134.9	149.1	179.2	189.3	201.6	219.5	229.8	
849	52.5	81.5	113.0	132.2	150.1	163.9	173.8	179.4	190.0	
850	44.1	81.7	121.3	145.1	161.3	169.5	194.2	195.8	224.9	
MEAN	49.1	79.2	114.2	135.1	152.0	167.1	179.7	192.0	204.9	208.9
S.D.	5.60	8.30	11.35	10.10	14.19	13.05	13.30	14.26	17.36	0.00
N	10	10	10	10	10	10	10	10	10	1

PR.NO.60R0375/88R002: REPRODUCTIVE TOX. STUDY TO DETECT EFFECTS
OF MIXED ANTI-ANDROGENIC SUBSTANCES IN RATS; ORAL ADM. (GAVAGE)
INDIVIDUAL BODY WEIGHT CHANGE -- GRAMS

MALES TEST GROUP 0 (0 MG/KG BW/D)

	WEEK OF STUDY				
ANIMAL#	0- 1	1- 2	2- 3	3- 4	4- 5
501	31.0	45.6	46.5	44.2	
502	33.6	44.5	44.4		
503	31.4	47.5	41.2		
504	27.2	37.2	27.0	29.8	
505	29.7	43.6	41.5		
506	32.4	48.7	47.0		
507	28.3	38.5	35.9		
508	37.0	49.3	39.5	34.0	
509	35.3	43.2	35.9		
510	37.3	44.6	37.7		
MEAN	32.3	44.3	39.7	36.0	
S.D.	3.48	3.97	5.97	7.41	
N	10	10	10	3	

PR.NO.60R0375/88R002: REPRODUCTIVE TOX. STUDY TO DETECT EFFECTS
OF MIXED ANTI-ANDROGENIC SUBSTANCES IN RATS; ORAL ADM. (GAVAGE)
INDIVIDUAL BODY WEIGHT CHANGE -- GRAMS

MALES TEST GROUP 1 (ADI-MIX)

ANIMAL#	WEEK OF STUDY 0- 1	1- 2	2- 3	3- 4	4- 5
511	28.7	43.3	42.0		
512	35.5	48.0	42.4		
513	37.1	49.7	51.1		
514	29.7	50.4	46.2		
515	33.1	47.4	39.5		
516	30.8	45.8	47.5		
517	30.5	43.9	39.0		
518	33.3	36.9	39.5	38.9	
519	32.8	41.7	49.5		
520	31.8	49.1	46.6		
MEAN	32.3	45.6	44.3	38.9	
S.D.	2.60	4.22	4.42	0.00	
N	10	10	10	1	

PR.NO.60R0375/88R002: REPRODUCTIVE TOX. STUDY TO DETECT EFFECTS
OF MIXED ANTI-ANDROGENIC SUBSTANCES IN RATS; ORAL ADM. (GAVAGE)
INDIVIDUAL BODY WEIGHT CHANGE -- GRAMS

MALES TEST GROUP 2 (NOAEL-MIX)

	WEEK OF STUDY				
ANIMAL#	0- 1	1- 2	2- 3	3- 4	4- 5
521	31.0	45.0	42.9		
522	31.1	41.9	46.1		
523	31.8	46.2	48.5		
524	31.1	48.8	41.2		
525	36.1	47.0	51.1		
526	29.2	45.2	44.5	39.7	
527	32.4	48.8	41.8		
528	32.3	45.9	40.8		
529	30.4	43.0	38.9	37.4	
530	33.7	38.1	39.9		
MEAN	31.9	45.0	43.6	38.6	
S.D.	1.91	3.27	3.95	1.63	
N	10	10	10	2	

PR.NO.60R0375/88R002: REPRODUCTIVE TOX. STUDY TO DETECT EFFECTS
OF MIXED ANTI-ANDROGENIC SUBSTANCES IN RATS; ORAL ADM. (GAVAGE)
INDIVIDUAL BODY WEIGHT CHANGE -- GRAMS

MALES TEST GROUP 3 (LOAEL-MIX)

ANIMAL#	WEEK OF STUDY 0- 1	1- 2	2- 3	3- 4	4- 5
531	24.6	42.6	42.6	34.1	32.3
532	32.5	41.2	42.4	34.8	
533	30.7	43.3	36.0		
534	29.8	39.2	30.2	37.6	
535	23.5	41.1	32.0	36.5	
536	32.4	44.7	45.1	42.0	41.2
537	27.9	41.1	38.0	41.0	
538	25.2	39.1	35.2	31.5	33.1
539	26.4	41.4	32.5	31.5	31.8
540	30.5	41.9	41.3	34.8	38.8
MEAN	28.4	41.6	37.5	36.0	35.4
S.D.	3.29	1.71	5.15	3.72	4.27
N	10	10	10	9	5

PR.NO.60R0375/88R002: REPRODUCTIVE TOX. STUDY TO DETECT EFFECTS
OF MIXED ANTI-ANDROGENIC SUBSTANCES IN RATS; ORAL ADM. (GAVAGE)
INDIVIDUAL BODY WEIGHT CHANGE -- GRAMS

MALES TEST GROUP 4(0.00025 MG/KG BW/D)

ANIMAL#	WEEK OF STUDY 0- 1	1- 2	2- 3	3- 4	4- 5
541	28.3	43.6	46.7		
542	31.6	40.4	37.2		
543	32.1	44.0	45.3		
544	33.2	45.5	47.5		
545	40.8	52.5			
546	33.2	52.2	39.3		
547	37.5	53.6			
548	35.9	47.1	43.2		
549	37.0	50.4	47.2		
550	39.6	48.7	44.5	36.8	
MEAN	34.9	47.8	43.9	36.8	
S.D.	3.90	4.41	3.79	0.00	
N	10	10	8	1	

PR.NO.60R0375/88R002: REPRODUCTIVE TOX. STUDY TO DETECT EFFECTS
OF MIXED ANTI-ANDROGENIC SUBSTANCES IN RATS; ORAL ADM. (GAVAGE)
INDIVIDUAL BODY WEIGHT CHANGE -- GRAMS

FEMALES TEST GROUP 0 (0 MG/KG BW/D)

ANIMAL#	WEEK OF STUDY 0- 1
601	30.0
602	26.5
603	28.7
604	24.9
605	27.1
606	30.1
607	34.3
608	29.5
609	26.6
610	32.9
MEAN	29.1
S.D.	2.95
N	10

PR.NO.60R0375/88R002: REPRODUCTIVE TOX. STUDY TO DETECT EFFECTS
OF MIXED ANTI-ANDROGENIC SUBSTANCES IN RATS; ORAL ADM. (GAVAGE)
INDIVIDUAL BODY WEIGHT CHANGE -- GRAMS

FEMALES TEST GROUP 1 (ADI-MIX)

ANIMAL#	WEEK OF STUDY 0- 1
611	30.2
612	33.8
613	24.1
614	31.4
615	25.5
616	29.4
617	28.3
618	29.8
619	32.1
620	30.9
MEAN	29.5
S.D.	2.94
N	10

PR.NO.60R0375/88R002: REPRODUCTIVE TOX. STUDY TO DETECT EFFECTS
OF MIXED ANTI-ANDROGENIC SUBSTANCES IN RATS; ORAL ADM. (GAVAGE)
INDIVIDUAL BODY WEIGHT CHANGE -- GRAMS

FEMALES TEST GROUP 2 (NOAEL-MIX)

ANIMAL#	WEEK OF STUDY 0- 1
621	32.9
622	29.4
623	29.6
624	29.1
625	31.3
626	28.4
627	27.7
628	29.5
629	30.8
630	29.3
MEAN	29.8
S.D.	1.50
N	10

PR.NO.60R0375/88R002: REPRODUCTIVE TOX. STUDY TO DETECT EFFECTS
OF MIXED ANTI-ANDROGENIC SUBSTANCES IN RATS; ORAL ADM. (GAVAGE)
INDIVIDUAL BODY WEIGHT CHANGE -- GRAMS

FEMALES TEST GROUP 3 (LOAEL-MIX)

ANIMAL#	WEEK OF STUDY 0- 1
631	23.6
632	24.9
633	28.1
634	23.6
635	25.1
636	30.6
637	25.3
638	31.3
639	26.3
640	27.9
MEAN	26.7
S.D.	2.73
N	10

PR.NO.60R0375/88R002: REPRODUCTIVE TOX. STUDY TO DETECT EFFECTS
OF MIXED ANTI-ANDROGENIC SUBSTANCES IN RATS; ORAL ADM. (GAVAGE)
INDIVIDUAL BODY WEIGHT CHANGE -- GRAMS

FEMALES TEST GROUP 4(0.00025 MG/KG BW/D)

ANIMAL#	WEEK OF STUDY 0- 1
641	31.2
642	31.5
643	34.7
644	25.7
645	25.0
646	29.4
647	24.4
648	27.1
649	33.5
650	26.4
MEAN	28.9
S.D.	3.69
N	10

PR.NO.60R0375/88R002: REPRODUCTIVE TOX. STUDY TO DETECT EFFECTS
OF MIXED ANTI-ANDROGENIC SUBSTANCES IN RATS; ORAL ADM. (GAVAGE)
INDIVIDUAL BODY WEIGHT CHANGE -- GRAMS

MALES TEST GROUP 0 (0 MG/KG BW/D)

ANIMAL#	WEEK OF STUDY 0- 1	1- 2	2- 3	3- 4	4- 5	5- 6	6- 7	7- 8	8- 9
701	34.4	48.1	44.6	35.8	38.1	28.8	29.1	19.1	
702	31.8	42.9	40.1	43.1	38.2	19.4	28.1	16.9	
703	30.0								
704	30.4	39.7	36.2	34.7	32.9	24.3	12.4	19.3	
705	38.4	45.9	43.8	41.5	39.4	26.9	20.3	11.9	
706	35.7	44.2	50.7	47.6	42.8	34.2	32.2	27.1	
707	33.0	44.3	37.2	40.6	36.8	23.5	19.1	18.9	13.3
708	32.4	42.3	36.0	35.2	28.7	23.0	20.5	20.0	4.8
709	26.5	42.8	43.7	39.3	38.2	29.8	26.6	30.9	
710	28.4	44.0	42.4	39.7	38.3	24.8	23.9	17.3	
MEAN	32.1	43.8	41.6	39.7	37.0	26.1	23.6	20.2	9.1
S.D.	3.52	2.35	4.79	4.16	4.05	4.38	6.12	5.63	6.01
N	10	9	9	9	9	9	9	9	2

PR.NO.60R0375/88R002: REPRODUCTIVE TOX. STUDY TO DETECT EFFECTS
OF MIXED ANTI-ANDROGENIC SUBSTANCES IN RATS; ORAL ADM. (GAVAGE)
INDIVIDUAL BODY WEIGHT CHANGE -- GRAMS

MALES TEST GROUP 1 (ADI-MIX)

	WEEK OF STUDY								
ANIMAL#	0- 1	1- 2	2- 3	3- 4	4- 5	5- 6	6- 7	7- 8	8- 9
711	30.9	47.0	41.0	32.2	28.7	16.5	12.8	8.8	13.0
712	32.7	42.2	41.2	33.4	29.4	29.8	20.8	17.7	
713	30.2	40.4	44.8	37.3	33.0	22.9	24.1	23.3	
714	37.7	48.6	54.4	43.9	40.5	39.0	33.9	21.5	
715	34.0	40.5	40.3	36.4	32.9	24.8	15.6	17.1	
716	33.5	44.1	43.6	33.3	39.5	30.8	20.9	21.3	
717	28.1	43.5	42.1	39.4	34.3	25.9	22.1	22.2	
718	29.7	38.2	38.5	31.0	31.0	24.4	18.0	17.8	17.7
719	33.0	46.4	47.9	44.8	36.6	24.2	20.7	18.7	
720	32.4	47.6	42.1	38.7	34.2	28.0	17.3	17.4	
MEAN	32.2	43.8	43.6	37.0	34.0	26.6	20.6	18.6	15.4
S.D.	2.68	3.51	4.60	4.75	3.94	5.90	5.73	4.11	3.32
N	10	10	10	10	10	10	10	10	2

PR.NO.60R0375/88R002: REPRODUCTIVE TOX. STUDY TO DETECT EFFECTS
OF MIXED ANTI-ANDROGENIC SUBSTANCES IN RATS; ORAL ADM. (GAVAGE)
INDIVIDUAL BODY WEIGHT CHANGE -- GRAMS

MALES TEST GROUP 2 (NOAEL-MIX)

	WEEK OF STUDY								
ANIMAL#	0- 1	1- 2	2- 3	3- 4	4- 5	5- 6	6- 7	7- 8	8- 9
721	27.8	36.5	40.1	32.8	31.9	8.2	40.6	21.7	
722	31.7	42.6	40.0	32.7	34.6	26.4	21.3	17.5	
723	31.8	44.4	47.9	48.3	32.7	32.7	26.3	26.0	
724	31.7	42.5	43.9	37.3	35.7	31.4	18.4	14.2	
725	29.7	42.6	45.2	34.2	35.8	27.9	22.9	22.1	22.3
726	33.4	41.7	37.7	24.5	37.3	26.6	14.8	15.0	2.4
727	33.8	48.2	46.0	37.8	49.5	30.4	29.8	26.5	16.3
728	36.0	45.7	46.6	38.1	37.0	32.3	32.2	20.9	24.4
729	30.5	44.6	41.0	37.4	40.4	29.6	23.4	16.0	
730	33.1	43.5	36.5	22.4	29.5	22.2	21.8	14.0	
MEAN	32.0	43.2	42.5	34.5	36.4	26.8	25.2	19.4	16.3
S.D.	2.30	3.04	3.95	7.33	5.52	7.27	7.44	4.71	9.91
N	10	10	10	10	10	10	10	10	4

PR.NO.60R0375/88R002: REPRODUCTIVE TOX. STUDY TO DETECT EFFECTS
OF MIXED ANTI-ANDROGENIC SUBSTANCES IN RATS; ORAL ADM. (GAVAGE)
INDIVIDUAL BODY WEIGHT CHANGE -- GRAMS

MALES TEST GROUP 3 (LOAEL-MIX)

	WEEK OF STUDY								
ANIMAL#	0- 1	1- 2	2- 3	3- 4	4- 5	5- 6	6- 7	7- 8	8- 9
731	30.4	41.9	32.8	37.8	33.1	30.3	18.0	17.8	
732	31.7	41.5	40.2	43.0	31.3	29.7	20.1	14.8	
733	31.3	45.7	45.2	41.5	30.2	23.5	21.0	13.0	5.6
734	29.8	40.2	36.4	37.4	37.6	23.5	23.7	16.9	14.7
735	28.6	45.9	41.2	40.9	42.7	36.5	31.8	30.5	24.2
736	24.7	37.5	36.9	29.6	33.7	17.5	15.5	10.3	
737	26.9	14.5	39.8	32.8	30.9	26.8	20.3	16.3	
738	30.0	49.4	39.3	39.3	39.3	36.9	20.9	21.3	
739	32.5	43.2	40.8	37.6	32.7	30.1	27.8	20.5	
740	30.3	40.1	41.6	39.8	33.2	26.1	30.8	19.2	
MEAN	29.6	40.0	39.4	38.0	34.5	28.1	23.0	18.1	14.8
S.D.	2.34	9.59	3.39	4.07	4.07	5.96	5.45	5.51	9.30
N	10	10	10	10	10	10	10	10	3

PR.NO.60R0375/88R002: REPRODUCTIVE TOX. STUDY TO DETECT EFFECTS
OF MIXED ANTI-ANDROGENIC SUBSTANCES IN RATS; ORAL ADM. (GAVAGE)
INDIVIDUAL BODY WEIGHT CHANGE -- GRAMS

MALES TEST GROUP 4(0.00025 MG/KG BW/D)

ANIMAL#	WEEK OF STUDY 0- 1	1- 2	2- 3	3- 4	4- 5	5- 6	6- 7	7- 8	8- 9
741	34.3	48.3	52.0	44.8	50.1	25.5	16.2	13.6	19.7
742	31.8	46.2	42.5	40.0	40.7	22.4	22.9	18.4	
743	32.4	42.9	40.9	30.0	30.0	24.2	19.9	10.8	
744	36.2	49.5	50.1	45.2	41.9	32.8	25.2	29.1	
745	29.7	40.2	42.6	39.6	41.8	27.2	30.2	20.1	21.6
746	33.8	53.5	51.6	42.4	38.8	36.5	31.5	23.3	13.8
747	41.0	51.5	43.3	46.1	36.5	11.9	11.3	15.7	
748	28.4	41.0	43.0	28.3	33.4	19.0	14.4	18.7	
749	31.5	41.2	41.3	39.5	38.0	27.8	27.2	32.4	
750	33.5	42.6	39.6	32.7	37.8	23.9	18.2	14.8	
MEAN	33.3	45.7	44.7	38.9	38.9	25.1	21.7	19.7	18.4
S.D.	3.53	4.78	4.67	6.42	5.41	6.85	6.83	6.85	4.07
N	10	10	10	10	10	10	10	10	3

PR.NO.60R0375/88R002: REPRODUCTIVE TOX. STUDY TO DETECT EFFECTS
OF MIXED ANTI-ANDROGENIC SUBSTANCES IN RATS; ORAL ADM. (GAVAGE)
INDIVIDUAL BODY WEIGHT CHANGE -- GRAMS

FEMALES TEST GROUP 0 (0 MG/KG BW/D)

	WEEK OF STUDY								
ANIMAL#	0- 1	1- 2	2- 3	3- 4	4- 5	5- 6	6- 7	7- 8	8- 9
801	30.5	36.0	22.8	10.3	15.7	13.4	7.1	10.3	
802	30.7	32.1	26.2	16.6	16.6	14.3	9.3	4.3	
803	27.6	29.5	21.8	12.9	3.7	22.0	5.3	3.4	
804	26.5	37.4	27.0	16.6	15.7	14.2	13.8	14.8	
805	22.7								
806	30.7	34.8	21.0	17.6	17.8	11.4	8.4	11.7	
807	25.0	12.5	28.9	22.7	15.6	16.3	15.5	8.1	6.9
808	29.6	33.4	25.0	15.3	11.5	7.8	16.5	18.9	-6.7
809	32.8	32.0	24.5	16.0	10.2	19.5	20.1	7.8	5.3
810	27.9	29.8	13.9	19.7	14.8	13.7	22.7	2.6	
MEAN	28.4	30.8	23.5	16.4	13.5	14.7	13.2	9.1	1.8
S.D.	3.06	7.37	4.38	3.59	4.40	4.19	6.04	5.45	7.43
N	10	9	9	9	9	9	9	9	3

PR.NO.60R0375/88R002: REPRODUCTIVE TOX. STUDY TO DETECT EFFECTS
OF MIXED ANTI-ANDROGENIC SUBSTANCES IN RATS; ORAL ADM. (GAVAGE)
INDIVIDUAL BODY WEIGHT CHANGE -- GRAMS

FEMALES TEST GROUP 1 (ADI-MIX)

	WEEK OF STUDY								
ANIMAL#	0- 1	1- 2	2- 3	3- 4	4- 5	5- 6	6- 7	7- 8	8- 9
811	34.4	41.7	32.8	15.8	7.1	20.5	9.9	4.2	
812	30.7	36.4	20.3	21.6	15.0	4.6	14.7	9.7	
813	28.6	33.0	28.0	18.2	10.5	21.0	11.3	5.7	
814	24.5	19.3	32.7	19.2	16.2	14.0	2.5	16.0	
815	27.1	32.5	20.9	16.0	12.8	10.2	10.3	22.5	-5.8
816	29.0	35.6	25.1	17.1	13.3	11.3	15.4	-0.1	
817	26.7	33.8	23.4	19.3	10.6	18.3	27.7	-9.5	
818	25.6	41.3	9.7	20.4	17.2	17.2	20.0	7.0	
819	27.4	29.0	23.8	17.7	11.5	11.9	15.2	14.8	-6.5
820	26.2	41.1	21.0	12.7	19.4	10.1	24.9	-7.9	16.6
MEAN	28.0	34.4	23.8	17.8	13.4	13.9	15.2	6.2	1.4
S.D.	2.86	6.78	6.72	2.57	3.66	5.27	7.47	10.21	13.14
N	10	10	10	10	10	10	10	10	3

PR.NO.60R0375/88R002: REPRODUCTIVE TOX. STUDY TO DETECT EFFECTS
OF MIXED ANTI-ANDROGENIC SUBSTANCES IN RATS; ORAL ADM. (GAVAGE)
INDIVIDUAL BODY WEIGHT CHANGE -- GRAMS

FEMALES TEST GROUP 2 (NOAEL-MIX)

ANIMAL#	WEEK OF STUDY 0- 1	1- 2	2- 3	3- 4	4- 5	5- 6	6- 7	7- 8	8- 9
821	27.3	33.6	29.4	19.7	15.2	8.3	11.2	9.7	
822	31.3	31.8	26.2	12.0	7.6	24.6	7.2	6.5	
823	32.0	40.1	24.8	19.8	20.0	5.4	35.6	9.6	
824	26.7	26.7	22.1	17.1	11.3	17.7	21.8	-0.9	
825	25.9	16.3	40.8	26.0	12.8	19.2	17.3	24.3	
826	30.1	38.4	22.0	19.6	13.7	18.8	22.1	4.9	11.2
827	24.4	32.2	18.6	14.4	16.3	4.4	26.5	2.1	7.5
828	28.7	35.5	20.3	17.1	14.9	16.5	20.9	5.5	
829	30.8	31.1	22.5	20.2	16.8	12.0	11.9	9.8	
830	36.7	40.4	27.3	21.6	18.7	10.1	11.8	12.2	
MEAN	29.4	32.6	25.4	18.8	14.7	13.7	18.6	8.4	9.3
S.D.	3.58	7.18	6.33	3.88	3.62	6.66	8.54	6.85	2.62
N	10	10	10	10	10	10	10	10	2

PR.NO.60R0375/88R002: REPRODUCTIVE TOX. STUDY TO DETECT EFFECTS
OF MIXED ANTI-ANDROGENIC SUBSTANCES IN RATS; ORAL ADM. (GAVAGE)
INDIVIDUAL BODY WEIGHT CHANGE -- GRAMS

FEMALES TEST GROUP 3 (LOAEL-MIX)

ANIMAL#	WEEK OF STUDY 0- 1	1- 2	2- 3	3- 4	4- 5	5- 6	6- 7	7- 8	8- 9
831	25.5	30.8	21.0	19.1	8.9	12.2	21.8	1.1	
832	29.5	31.0	23.4	15.5	11.4	18.4	20.4	-3.0	
833	27.5	27.6	22.1	16.4	20.3	15.2	23.5	6.1	7.0
834	27.9	36.4	19.0	17.2	14.9	23.5	11.1	8.8	9.3
835	30.5	35.9	26.8	12.3	29.9	18.2	24.9	2.7	10.5
836	25.9	30.3	27.9	20.4	14.9	13.9	15.9	-2.7	
837	27.7	34.3	24.7	17.6	10.3	15.7	21.7	5.9	
838	25.8	31.9	22.8	15.3	11.1	17.4	22.7	4.3	
839	28.2	31.5	19.7	20.1	16.3	13.3	6.1	11.2	
840	34.9	32.9	24.7	16.6	4.9	26.2	23.6	-2.0	
MEAN	28.3	32.3	23.2	17.1	14.3	17.4	19.2	3.2	8.9
S.D.	2.81	2.69	2.90	2.44	6.96	4.47	6.19	4.91	1.78
N	10	10	10	10	10	10	10	10	3

PR.NO.60R0375/88R002: REPRODUCTIVE TOX. STUDY TO DETECT EFFECTS
OF MIXED ANTI-ANDROGENIC SUBSTANCES IN RATS; ORAL ADM. (GAVAGE)
INDIVIDUAL BODY WEIGHT CHANGE -- GRAMS

FEMALES TEST GROUP 4(0.00025 MG/KG BW/D)

	WEEK OF STUDY								
ANIMAL#	0- 1	1- 2	2- 3	3- 4	4- 5	5- 6	6- 7	7- 8	8- 9
841	28.3	27.1	19.5	10.5	13.7	15.5	18.4	-1.9	
842	28.8	36.7	24.7	13.1	20.3	14.8	10.2	13.6	
843	28.5	34.0	18.6	9.0	15.4	12.1	3.0	16.7	
844	31.0	37.2	17.0	19.9	12.1	14.2	12.6	7.4	
845	35.7	34.7	23.1	16.2	18.3	6.8	17.2	10.7	
846	22.6	34.5	21.9	18.9	20.1	9.2	20.9	12.4	
847	25.4	32.9	27.6	17.3	19.1	6.5	15.0	20.7	-1.0
848	34.1	41.1	14.2	30.1	10.1	12.3	17.9	10.3	
849	29.0	31.5	19.2	17.9	13.8	9.9	5.6	10.6	
850	37.6	39.6	23.8	16.2	8.2	24.7	1.6	29.1	
MEAN	30.1	34.9	21.0	16.9	15.1	12.6	12.2	13.0	-1.0
S.D.	4.61	4.03	4.00	5.84	4.26	5.29	6.87	8.20	0.00
N	10	10	10	10	10	10	10	10	1

PR.NO.60R0375/88R002: REPRODUCTIVE TOX. STUDY TO DETECT EFFECTS
OF MIXED ANTI-ANDROGENIC SUBSTANCES IN RATS; ORAL ADM. (GAVAGE)
NUMBER OF PUPS WITH MEASURE PRESENT / NUMBER TESTED

TEST GROUP 0 (0 MG/KG BW/D) VAGINAL OPENING

	POSTPARTUM DAY												
FEMALE#	27	28	29	30	31	32	33	34	35	36	37	38	39
1	0/ 1	0/ 1	0/ 1	0/ 1	0/ 1	1/ 1							
2	0/ 2	0/ 2	0/ 2	0/ 2	0/ 2	1/ 2	1/ 1						
3	0/ 1	0/ 1	1/ 1										
5	0/ 2	1/ 2	0/ 1	0/ 1	0/ 1	0/ 1	1/ 1						
7	0/ 2	0/ 2	0/ 2	1/ 2	0/ 1	0/ 1	1/ 1						
8	0/ 1	0/ 1	0/ 1	0/ 1	0/ 1	0/ 1	0/ 1	1/ 1					
9	0/ 1	0/ 1	0/ 1	0/ 1	1/ 1								
11	0/ 2	1/ 2	0/ 1	0/ 1	0/ 1	1/ 1							
17	0/ 1	0/ 1	0/ 1	0/ 1	0/ 1	0/ 1	1/ 1						
18	0/ 1	0/ 1	1/ 1										
19	0/ 1	0/ 1	0/ 1	1/ 1									
20	0/ 1	0/ 1	0/ 1	0/ 1	1/ 1								
21	0/ 1	0/ 1	0/ 1	1/ 1									
23	0/ 1	0/ 1	0/ 1	1/ 1									
24	0/ 1	0/ 1	0/ 1	1/ 1									
25	0/ 1	0/ 1	0/ 1	1/ 1									
TOTAL	0/ 20	2/ 20	2/ 18	6/ 16	2/ 10	3/ 8	4/ 5	1/ 1					
CUMULATIVE	0/ 20<br>0%	2/ 20<br>10%	4/ 20<br>20%	10/ 20<br>50%	12/ 20<br>60%	15/ 20<br>75%	19/ 20<br>95%	20/ 20<br>100%					

PR.NO.60R0375/88R002: REPRODUCTIVE TOX. STUDY TO DETECT EFFECTS
OF MIXED ANTI-ANDROGENIC SUBSTANCES IN RATS; ORAL ADM. (GAVAGE)
NUMBER OF PUPS WITH MEASURE PRESENT / NUMBER TESTED

TEST GROUP 1 (ADI-MIX) VAGINAL OPENING

	POSTPARTUM DAY												
FEMALE#	27	28	29	30	31	32	33	34	35	36	37	38	39
27	0/ 2	0/ 2	1/ 2	0/ 1	1/ 1								
29	0/ 1	0/ 1	0/ 1	0/ 1	1/ 1								
30	0/ 1	0/ 1	0/ 1	0/ 1	1/ 1								
31	0/ 2	0/ 2	0/ 2	0/ 2	0/ 2	1/ 2	1/ 1						
32	0/ 1	0/ 1	0/ 1	1/ 1									
33	0/ 1	0/ 1	0/ 1	0/ 1	1/ 1								
34	0/ 1	0/ 1	0/ 1	0/ 1	1/ 1								
35	0/ 1	0/ 1	1/ 1										
36	0/ 1	0/ 1	0/ 1	0/ 1	1/ 1								
37	0/ 2	0/ 2	0/ 2	0/ 2	1/ 2	0/ 1	1/ 1						
44	0/ 2	2/ 2											
45	0/ 1	0/ 1	0/ 1	1/ 1									
47	0/ 1	0/ 1	0/ 1	1/ 1									
48	0/ 2	0/ 2	0/ 2	1/ 2	0/ 1	0/ 1	1/ 1						
50	0/ 1	0/ 1	1/ 1										
TOTAL	0/ 20	2/ 20	3/ 18	4/ 15	7/ 11	1/ 4	3/ 3						
CUMULATIVE	0/ 20<br>0%	2/ 20<br>10%	5/ 20<br>25%	9/ 20<br>45%	16/ 20<br>80%	17/ 20<br>85%	20/ 20<br>100%						

PR.NO.60R0375/88R002: REPRODUCTIVE TOX. STUDY TO DETECT EFFECTS
OF MIXED ANTI-ANDROGENIC SUBSTANCES IN RATS; ORAL ADM. (GAVAGE)
NUMBER OF PUPS WITH MEASURE PRESENT / NUMBER TESTED

TEST GROUP 2 (NOAEL-MIX) VAGINAL OPENING

	POSTPARTUM DAY												
FEMALE#	27	28	29	30	31	32	33	34	35	36	37	38	39
51	0/ 1	0/ 1	0/ 1	0/ 1	1/ 1								
52	0/ 2	0/ 2	1/ 2	1/ 1									
53	0/ 1	0/ 1	0/ 1	1/ 1									
54	0/ 1	1/ 1											
55	0/ 1	0/ 1	1/ 1										
58	0/ 1	0/ 1	1/ 1										
59	0/ 1	0/ 1	1/ 1										
60	0/ 2	0/ 2	0/ 2	0/ 2	1/ 2	0/ 1	0/ 1	1/ 1					
62	0/ 1	0/ 1	0/ 1	1/ 1									
68	0/ 1	0/ 1	0/ 1	0/ 1	1/ 1								
69	0/ 1	0/ 1	0/ 1	0/ 1	1/ 1								
70	0/ 1	0/ 1	0/ 1	1/ 1									
71	0/ 1	0/ 1	0/ 1	0/ 1	0/ 1	1/ 1							
72	0/ 1	0/ 1	1/ 1										
73	0/ 2	1/ 2	1/ 1										
74	0/ 1	0/ 1	0/ 1	1/ 1									
75	0/ 1	0/ 1	0/ 1	0/ 1	1/ 1								
TOTAL	0/ 20	2/ 20	6/ 18	5/ 12	5/ 7	1/ 2	0/ 1	1/ 1					
CUMULATIVE	0/ 20<br>0%	2/ 20<br>10%	8/ 20<br>40%	13/ 20<br>65%	18/ 20<br>90%	19/ 20<br>95%	19/ 20<br>95%	20/ 20<br>100%					

PR.NO.60R0375/88R002: REPRODUCTIVE TOX. STUDY TO DETECT EFFECTS
OF MIXED ANTI-ANDROGENIC SUBSTANCES IN RATS; ORAL ADM. (GAVAGE)
NUMBER OF PUPS WITH MEASURE PRESENT / NUMBER TESTED

TEST GROUP 3 (LOAEL-MIX) VAGINAL OPENING

	POSTPARTUM DAY												
FEMALE#	27	28	29	30	31	32	33	34	35	36	37	38	39
77	0/ 1	0/ 1	0/ 1	0/ 1	1/ 1								
78	0/ 1	0/ 1	1/ 1										
80	0/ 1	0/ 1	0/ 1	1/ 1									
82	0/ 1	0/ 1	0/ 1	0/ 1	0/ 1	0/ 1	1/ 1						
83	0/ 1	0/ 1	0/ 1	1/ 1									
84	0/ 1	0/ 1	1/ 1										
85	0/ 1	0/ 1	0/ 1	0/ 1	1/ 1								
86	0/ 1	1/ 1											
87	0/ 1	0/ 1	1/ 1										
93	0/ 1	0/ 1	0/ 1	1/ 1									
94	0/ 2	0/ 2	0/ 2	1/ 2	1/ 1								
95	0/ 1	0/ 1	0/ 1	1/ 1									
96	0/ 2	0/ 2	0/ 2	2/ 2									
97	0/ 1	0/ 1	0/ 1	0/ 1	1/ 1								
98	1/ 2	0/ 1	0/ 1	0/ 1	1/ 1								
99	0/ 1	0/ 1	1/ 1										
100	0/ 1	0/ 1	0/ 1	0/ 1	1/ 1								
TOTAL	1/ 20	1/ 19	4/ 18	7/ 14	6/ 7	0/ 1	1/ 1						
CUMULATIVE	1/ 20<br>5%	2/ 20<br>10%	6/ 20<br>30%	13/ 20<br>65%	19/ 20<br>95%	19/ 20<br>95%	20/ 20<br>100%						

PR.NO.60R0375/88R002: REPRODUCTIVE TOX. STUDY TO DETECT EFFECTS
OF MIXED ANTI-ANDROGENIC SUBSTANCES IN RATS; ORAL ADM. (GAVAGE)
NUMBER OF PUPS WITH MEASURE PRESENT / NUMBER TESTED

TEST GROUP 4(0.00025 MG/KG BW/D) VAGINAL OPENING

	POSTPARTUM DAY												
FEMALE#	27	28	29	30	31	32	33	34	35	36	37	38	39
101	0/ 1	0/ 1	0/ 1	1/ 1									
102	0/ 1	0/ 1	0/ 1	1/ 1									
103	0/ 1	0/ 1	0/ 1	1/ 1									
104	0/ 1	0/ 1	0/ 1	0/ 1	0/ 1	0/ 1	0/ 1	0/ 1	1/ 1				
105	0/ 1	0/ 1	0/ 1	1/ 1									
106	0/ 2	0/ 2	0/ 2	1/ 2	1/ 1								
107	0/ 1	0/ 1	1/ 1										
108	0/ 1	0/ 1	0/ 1	0/ 1	0/ 1	0/ 1	1/ 1						
109	0/ 2	0/ 2	0/ 2	2/ 2									
111	0/ 1	0/ 1	0/ 1	0/ 1	0/ 1	1/ 1							
117	0/ 1	1/ 1											
119	0/ 1	0/ 1	1/ 1										
120	0/ 1	0/ 1	0/ 1	0/ 1	1/ 1								
121	0/ 1	0/ 1	0/ 1	0/ 1	1/ 1								
123	0/ 1	0/ 1	0/ 1	0/ 1	1/ 1								
124	0/ 2	0/ 2	1/ 2	0/ 1	1/ 1								
125	0/ 1	0/ 1	0/ 1	0/ 1	0/ 1	1/ 1							
TOTAL	0/ 20	1/ 20	3/ 19	7/ 16	5/ 9	2/ 4	1/ 2	0/ 1	1/ 1				
CUMULATIVE	0/ 20<br>0%	1/ 20<br>5%	4/ 20<br>20%	11/ 20<br>55%	16/ 20<br>80%	18/ 20<br>90%	19/ 20<br>95%	19/ 20<br>95%	20/ 20<br>100%				

PR.NO.60R0375/88R002: REPRODUCTIVE TOX. STUDY TO DETECT EFFECTS
OF MIXED ANTI-ANDROGENIC SUBSTANCES IN RATS; ORAL ADM. (GAVAGE)
INDIVIDUAL PUP WEIGHTS (G) AT DAY OF VAGINAL OPENING

TEST GROUP 0 0 MG/KG BW/D			TEST GROUP 1 ADI-MIX			TEST GROUP 2 NOAEL-MIX			TEST GROUP 3 LOAEL-MIX			TEST GROUP 4 0.00025 MG/KG BW/D		
DAM-PUP	day	weight	DAM-PUP	day	weight	DAM-PUP	day	weight	DAM-PUP	day	weight	DAM-PUP	day	weight
1-07	32	100.1	27-06	29	90.3	51-04	31	85.6	77-05	31	81.3	101-08	30	99.0
2-08	33	96.4	27-09	31	96.8	52-06	29	79.0	78-09	29	74.4	102-09	30	77.5
2-10	32	90.5	29-10	31	95.8	52-10	30	89.9	80-07	30	90.0	103-07	30	82.4
3-07	29	83.1	30-09	31	85.4	53-08	30	85.4	82-08	33	88.5	104-11	35	112.0
5-08	28	72.3	31-03	32	89.8	54-07	28	78.4	83-09	30	77.9	105-06	30	91.2
5-10	33	92.9	31-10	33	86.6	55-10	29	77.9	84-07	29	83.8	106-07	30	95.3
7-06	30	82.5	32-04	30	94.4	58-06	29	81.4	85-09	31	92.0	106-08	31	96.6
7-07	33	107.7	33-06	31	86.9	59-07	29	80.2	86-06	28	72.8	107-06	29	90.6
8-09	34	61.2	34-09	31	82.7	60-08	31	82.1	87-10	29	71.2	108-07	33	92.2
9-05	31	83.3	35-08	29	80.1	60-11	34	74.7	93-06	30	84.4	109-05	30	79.3
11-06	28	73.8	36-06	31	91.2	62-07	30	87.9	94-07	30	90.5	109-09	30	85.3
11-12	32	92.3	37-07	33	74.5	68-10	31	90.5	94-08	31	90.0	111-09	32	89.0
17-08	33	73.6	37-09	31	71.9	69-09	31	83.1	95-10	30	84.0	117-07	28	71.9
18-08	29	90.0	44-05	28	74.2	70-06	30	88.4	96-06	30	83.1	119-05	29	102.3
19-04	30	85.8	44-08	28	76.8	71-06	32	95.4	96-09	30	82.7	120-06	31	95.1
20-03	31	88.8	45-06	30	85.7	72-11	29	73.0	97-09	31	87.2	121-02	31	85.0
21-11	30	87.1	47-05	30	85.7	73-06	28	82.4	98-06	27	67.1	123-06	31	95.1
23-05	30	82.7	48-01	30	89.0	73-09	29	80.7	98-10	31	84.1	124-05	31	92.7
24-07	30	77.5	48-08	33	90.5	74-08	30	63.3	99-08	29	89.0	124-09	29	85.5
25-09	30	80.0	50-08	29	75.7	75-07	31	88.6	100-05	31	88.0	125-10	32	82.6

PR.NO.60R0375/88R002: REPRODUCTIVE TOX. STUDY TO DETECT EFFECTS
OF MIXED ANTI-ANDROGENIC SUBSTANCES IN RATS; ORAL ADM. (GAVAGE)
NUMBER OF PUPS WITH MEASURE PRESENT / NUMBER TESTED

TEST GROUP 0 (0 MG/KG BW/D) PREPUTIAL SEPARATION

	POSTPARTUM DAY												
FEMALE#	38	39	40	41	42	43	44	45	46	47	48	49	50
1	0/ 1	0/ 1	0/ 1	0/ 1	0/ 1	0/ 1	1/ 1						
3	0/ 1	0/ 1	0/ 1	0/ 1	0/ 1	0/ 1	0/ 1	0/ 1	0/ 1	0/ 1	0/ 1	0/ 1	1/ 1
4	0/ 2	0/ 2	0/ 2	1/ 2	0/ 1	0/ 1	1/ 1						
6	0/ 1	0/ 1	0/ 1	0/ 1	1/ 1								
8	0/ 1	0/ 1	0/ 1	0/ 1	0/ 1	0/ 1	0/ 1	0/ 1	0/ 1	0/ 1	0/ 1	1/ 1	
9	0/ 1	0/ 1	0/ 1	0/ 1	0/ 1	0/ 1	0/ 1	0/ 1	1/ 1				
10	0/ 2	0/ 2	0/ 2	0/ 2	0/ 2	0/ 2	0/ 2	0/ 2	0/ 2	1/ 2	1/ 1		
17	0/ 1	0/ 1	0/ 1	0/ 1	0/ 1	0/ 1	0/ 1	0/ 1	0/ 1	0/ 1	1/ 1		
18	0/ 1	0/ 1	0/ 1	0/ 1	0/ 1	0/ 1	0/ 1	1/ 1					
19	0/ 1	0/ 1	0/ 1	0/ 1	0/ 1	0/ 1	0/ 1	0/ 1	0/ 1	0/ 1	0/ 1	1/ 1	
20	0/ 1	0/ 1	0/ 1	0/ 1	0/ 1	0/ 1	0/ 1	0/ 1	0/ 1	0/ 1	0/ 1	1/ 1	
21	0/ 1	0/ 1	0/ 1	0/ 1	0/ 1	0/ 1	0/ 1	0/ 1	0/ 1	0/ 1	1/ 1		
22	0/ 2	0/ 2	0/ 2	0/ 2	1/ 2	1/ 1							
23	0/ 1	0/ 1	0/ 1	0/ 1	0/ 1	0/ 1	0/ 1	0/ 1	0/ 1	1/ 1			
24	0/ 1	0/ 1	0/ 1	0/ 1	0/ 1	0/ 1	0/ 1	0/ 1	0/ 1	1/ 1			
25	0/ 1	0/ 1	0/ 1	0/ 1	0/ 1	0/ 1	1/ 1						
TOTAL	0/ 19	0/ 19	0/ 19	1/ 19	2/ 18	1/ 16	3/ 15	1/ 12	1/ 11	3/ 10	3/ 7	3/ 4	1/ 1
CUMULATIVE	0/ 19<br>0%	0/ 19<br>0%	0/ 19<br>0%	1/ 19<br>5%	3/ 19<br>16%	4/ 19<br>21%	7/ 19<br>37%	8/ 19<br>42%	9/ 19<br>47%	12/ 19<br>63%	15/ 19<br>79%	18/ 19<br>95%	19/ 19<br>100%

PR.NO.60R0375/88R002: REPRODUCTIVE TOX. STUDY TO DETECT EFFECTS
OF MIXED ANTI-ANDROGENIC SUBSTANCES IN RATS; ORAL ADM. (GAVAGE)
NUMBER OF PUPS WITH MEASURE PRESENT / NUMBER TESTED

TEST GROUP 1 (ADI-MIX) PREPUTIAL SEPARATION

	POSTPARTUM DAY												
FEMALE#	38	39	40	41	42	43	44	45	46	47	48	49	50
26	0/ 2	0/ 2	0/ 2	0/ 2	1/ 2	0/ 1	0/ 1	0/ 1	0/ 1	0/ 1	1/ 1		
28	0/ 2	0/ 2	0/ 2	0/ 2	1/ 2	0/ 1	1/ 1						
29	0/ 1	0/ 1	0/ 1	0/ 1	1/ 1								
30	0/ 1	0/ 1	0/ 1	0/ 1	0/ 1	0/ 1	1/ 1						
32	0/ 1	0/ 1	0/ 1	0/ 1	0/ 1	1/ 1							
33	0/ 1	0/ 1	0/ 1	0/ 1	0/ 1	0/ 1	0/ 1	0/ 1	0/ 1	0/ 1	1/ 1		
34	0/ 1	0/ 1	0/ 1	0/ 1	0/ 1	0/ 1	0/ 1	1/ 1					
35	0/ 1	0/ 1	0/ 1	0/ 1	0/ 1	1/ 1							
36	0/ 1	0/ 1	0/ 1	0/ 1	0/ 1	0/ 1	0/ 1	1/ 1					
43	0/ 2	0/ 2	0/ 2	0/ 2	0/ 2	0/ 2	0/ 2	1/ 2	0/ 1	0/ 1	0/ 1	1/ 1	
45	0/ 1	0/ 1	0/ 1	0/ 1	0/ 1	1/ 1							
46	0/ 2	0/ 2	0/ 2	0/ 2	0/ 2	0/ 2	0/ 2	0/ 2	1/ 2	0/ 1	1/ 1		
47	0/ 1	0/ 1	0/ 1	0/ 1	0/ 1	0/ 1	0/ 1	1/ 1					
49	0/ 2	0/ 2	0/ 2	0/ 2	0/ 2	0/ 2	0/ 2	0/ 2	0/ 2	1/ 2	0/ 1	0/ 1	1/ 1
50	0/ 1	0/ 1	0/ 1	0/ 1	0/ 1	0/ 1	0/ 1	0/ 1	0/ 1	1/ 1			
TOTAL	0/ 20	0/ 20	0/ 20	0/ 20	3/ 20	3/ 17	2/ 14	4/ 12	1/ 8	2/ 7	3/ 5	1/ 2	1/ 1
CUMULATIVE	0/ 20<br>0%	0/ 20<br>0%	0/ 20<br>0%	0/ 20<br>0%	3/ 20<br>15%	6/ 20<br>30%	8/ 20<br>40%	12/ 20<br>60%	13/ 20<br>65%	15/ 20<br>75%	18/ 20<br>90%	19/ 20<br>95%	20/ 20<br>100%

PR.NO.60R0375/88R002: REPRODUCTIVE TOX. STUDY TO DETECT EFFECTS
OF MIXED ANTI-ANDROGENIC SUBSTANCES IN RATS; ORAL ADM. (GAVAGE)
NUMBER OF PUPS WITH MEASURE PRESENT / NUMBER TESTED

TEST GROUP 2 (NOAEL-MIX) PREPUTIAL SEPARATION

	POSTPARTUM DAY												
FEMALE#	38	39	40	41	42	43	44	45	46	47	48	49	50
51	0/ 1	0/ 1	0/ 1	0/ 1	1/ 1								
53	0/ 1	0/ 1	0/ 1	0/ 1	0/ 1	0/ 1	0/ 1	0/ 1	1/ 1				
54	0/ 1	0/ 1	0/ 1	0/ 1	0/ 1	0/ 1	0/ 1	0/ 1	0/ 1	0/ 1	1/ 1		
55	0/ 1	0/ 1	0/ 1	0/ 1	1/ 1								
57	0/ 2	0/ 2	0/ 2	0/ 2	0/ 2	1/ 2	0/ 1	0/ 1	1/ 1				
58	0/ 1	0/ 1	0/ 1	0/ 1	0/ 1	0/ 1	0/ 1	0/ 1	1/ 1				
59	0/ 1	0/ 1	0/ 1	0/ 1	0/ 1	1/ 1							
61	0/ 2	0/ 2	0/ 2	0/ 2	0/ 2	0/ 2	0/ 2	0/ 2	1/ 2	0/ 1	0/ 1	1/ 1	
62	0/ 1	0/ 1	0/ 1	0/ 1	0/ 1	0/ 1	0/ 1	0/ 1	0/ 1	0/ 1	0/ 1	1/ 1	
68	0/ 1	0/ 1	0/ 1	0/ 1	0/ 1	0/ 1	0/ 1	0/ 1	0/ 1	0/ 1	0/ 1	1/ 1	
69	0/ 1	0/ 1	0/ 1	0/ 1	0/ 1	0/ 1	0/ 1	0/ 1	1/ 1				
70	0/ 1	0/ 1	0/ 1	0/ 1	0/ 1	0/ 1	0/ 1	0/ 1	0/ 1	1/ 1			
71	0/ 1	0/ 1	0/ 1	0/ 1	0/ 1	0/ 1	0/ 1	0/ 1	0/ 1	0/ 1	0/ 1	0/ 1	1/ 1
72	0/ 1	0/ 1	0/ 1	0/ 1	0/ 1	0/ 1	0/ 1	1/ 1					
73	0/ 1	0/ 1	0/ 1	0/ 1	0/ 1	0/ 1	0/ 1	0/ 1	0/ 1	1/ 1			
74	0/ 2	0/ 2	0/ 2	0/ 2	0/ 2	0/ 2	0/ 2	0/ 2	0/ 2	0/ 2	1/ 2	0/ 1	0/ 1
75	0/ 1	0/ 1	0/ 1	0/ 1	0/ 1	0/ 1	0/ 1	0/ 1	0/ 1	0/ 1	0/ 1	0/ 1	0/ 1
TOTAL	0/ 20	0/ 20	0/ 20	0/ 20	2/ 20	2/ 18	0/ 16	1/ 16	5/ 15	2/ 10	2/ 8	3/ 6	1/ 3
CUMULATIVE	0/ 20	0/ 20	0/ 20	0/ 20	2/ 20	4/ 20	4/ 20	5/ 20	10/ 20	12/ 20	14/ 20	17/ 20	18/ 20
	0%	0%	0%	0%	10%	20%	20%	25%	50%	60%	70%	85%	90%

PR.NO.60R0375/88R002: REPRODUCTIVE TOX. STUDY TO DETECT EFFECTS
OF MIXED ANTI-ANDROGENIC SUBSTANCES IN RATS; ORAL ADM. (GAVAGE)
NUMBER OF PUPS WITH MEASURE PRESENT / NUMBER TESTED

TEST GROUP 3 (LOAEL-MIX) PREPUTIAL SEPARATION

	POSTPARTUM DAY												
FEMALE#	38	39	40	41	42	43	44	45	46	47	48	49	50
77	0/ 1	0/ 1	0/ 1	0/ 1	0/ 1	0/ 1	0/ 1	0/ 1	0/ 1	0/ 1	0/ 1	0/ 1	0/ 1
78	0/ 1	0/ 1	0/ 1	0/ 1	0/ 1	0/ 1	0/ 1	0/ 1	0/ 1	0/ 1	0/ 1	1/ 1	
80	0/ 1	0/ 1	0/ 1	0/ 1	0/ 1	0/ 1	0/ 1	0/ 1	0/ 1	0/ 1	0/ 1	0/ 1	0/ 1
82	0/ 1	0/ 1	0/ 1	0/ 1	0/ 1	0/ 1	0/ 1	0/ 1	0/ 1	0/ 1	0/ 1	0/ 1	1/ 1
83	0/ 1	0/ 1	0/ 1	0/ 1	0/ 1	0/ 1	0/ 1	0/ 1	0/ 1	0/ 1	1/ 1		
84	0/ 1	0/ 1	0/ 1	0/ 1	0/ 1	0/ 1	0/ 1	0/ 1	0/ 1	0/ 1	0/ 1	0/ 1	1/ 1
85	0/ 1	0/ 1	0/ 1	0/ 1	0/ 1	0/ 1	0/ 1	0/ 1	0/ 1	0/ 1	0/ 1	0/ 1	0/ 1
86	0/ 1	0/ 1	0/ 1	0/ 1	0/ 1	0/ 1	0/ 1	0/ 1	0/ 1	0/ 1	0/ 1	0/ 1	0/ 1
87	0/ 2	0/ 2	0/ 2	0/ 2	0/ 2	0/ 2	0/ 2	0/ 2	0/ 2	0/ 2	0/ 2	0/ 2	0/ 2
93	0/ 1	0/ 1	0/ 1	0/ 1	0/ 1	0/ 1	0/ 1	0/ 1	0/ 1	0/ 1	0/ 1	0/ 1	0/ 1
94	0/ 1	0/ 1	0/ 1	0/ 1	0/ 1	0/ 1	0/ 1	0/ 1	0/ 1	0/ 1	0/ 1	0/ 1	0/ 1
95	0/ 1	0/ 1	0/ 1	0/ 1	0/ 1	0/ 1	0/ 1	0/ 1	0/ 1	0/ 1	0/ 1	0/ 1	0/ 1
96	0/ 2	0/ 2	0/ 2	0/ 2	0/ 2	0/ 2	0/ 2	0/ 2	0/ 2	0/ 2	0/ 2	0/ 2	0/ 2
97	0/ 1	0/ 1	0/ 1	0/ 1	0/ 1	0/ 1	0/ 1	0/ 1	0/ 1	0/ 1	0/ 1	0/ 1	0/ 1
98	0/ 1	0/ 1	0/ 1	0/ 1	0/ 1	0/ 1	0/ 1	0/ 1	0/ 1	0/ 1	0/ 1	0/ 1	0/ 1
99	0/ 2	0/ 2	0/ 2	0/ 2	0/ 2	0/ 2	0/ 2	0/ 2	0/ 2	0/ 2	0/ 2	0/ 2	0/ 2
100	0/ 1	0/ 1	0/ 1	0/ 1	0/ 1	0/ 1	0/ 1	0/ 1	0/ 1	0/ 1	0/ 1	0/ 1	0/ 1
TOTAL	0/ 20	0/ 20	0/ 20	0/ 20	0/ 20	0/ 20	0/ 20	0/ 20	0/ 20	0/ 20	1/ 20	1/ 19	2/ 18
CUMULATIVE	0/ 20 0%	0/ 20 0%	0/ 20 0%	0/ 20 0%	0/ 20 0%	0/ 20 0%	0/ 20 0%	0/ 20 0%	0/ 20 0%	0/ 20 0%	1/ 20 5%	2/ 20 10%	4/ 20 20%

PR.NO.60R0375/88R002: REPRODUCTIVE TOX. STUDY TO DETECT EFFECTS
OF MIXED ANTI-ANDROGENIC SUBSTANCES IN RATS; ORAL ADM. (GAVAGE)
NUMBER OF PUPS WITH MEASURE PRESENT / NUMBER TESTED

TEST GROUP 4(0.00025 MG/KG BW/D) PREPUTIAL SEPARATION

FEMALE#	POSTPARTUM DAY 38	39	40	41	42	43	44	45	46	47	48	49	50
101	0/ 1	0/ 1	1/ 1										
102	0/ 1	0/ 1	0/ 1	0/ 1	0/ 1	0/ 1	0/ 1	0/ 1	0/ 1	1/ 1			
103	0/ 2	0/ 2	0/ 2	0/ 2	0/ 2	0/ 2	0/ 2	0/ 2	0/ 2	2/ 2			
104	0/ 1	0/ 1	0/ 1	0/ 1	0/ 1	0/ 1	0/ 1	0/ 1	0/ 1	1/ 1			
105	0/ 1	0/ 1	0/ 1	0/ 1	1/ 1								
107	0/ 1	0/ 1	0/ 1	0/ 1	0/ 1	1/ 1							
108	0/ 1	0/ 1	0/ 1	0/ 1	0/ 1	0/ 1	0/ 1	0/ 1	0/ 1	0/ 1	0/ 1	1/ 1	
111	0/ 1	0/ 1	0/ 1	0/ 1	0/ 1	0/ 1	0/ 1	0/ 1	0/ 1	0/ 1	1/ 1		
117	0/ 1	0/ 1	0/ 1	0/ 1	0/ 1	0/ 1	0/ 1	1/ 1					
118	0/ 2	0/ 2	1/ 2	1/ 1									
119	0/ 2	0/ 2	0/ 2	0/ 2	0/ 2	0/ 2	0/ 2	0/ 2	0/ 2	1/ 2	1/ 1		
120	0/ 1	0/ 1	0/ 1	0/ 1	0/ 1	0/ 1	0/ 1	0/ 1	0/ 1	1/ 1			
121	0/ 1	0/ 1	0/ 1	0/ 1	0/ 1	0/ 1	0/ 1	1/ 1					
123	0/ 1	0/ 1	0/ 1	0/ 1	0/ 1	0/ 1	1/ 1						
124	0/ 2	0/ 2	0/ 2	0/ 2	0/ 2	0/ 2	0/ 2	0/ 2	0/ 2	0/ 2	0/ 2	2/ 2	
125	0/ 1	0/ 1	0/ 1	0/ 1	0/ 1	0/ 1	0/ 1	0/ 1	0/ 1	0/ 1	1/ 1		
TOTAL	0/ 20	0/ 20	2/ 20	1/ 18	1/ 17	1/ 16	1/ 15	2/ 14	0/ 12	6/ 12	3/ 6	3/ 3	
CUMULATIVE	0/ 20<br>0%	0/ 20<br>0%	2/ 20<br>10%	3/ 20<br>15%	4/ 20<br>20%	5/ 20<br>25%	6/ 20<br>30%	8/ 20<br>40%	8/ 20<br>40%	14/ 20<br>70%	17/ 20<br>85%	20/ 20<br>100%	

PR.NO.60R0375/88R002: REPRODUCTIVE TOX. STUDY TO DETECT EFFECTS
OF MIXED ANTI-ANDROGENIC SUBSTANCES IN RATS; ORAL ADM. (GAVAGE)
NUMBER OF PUPS WITH MEASURE PRESENT / NUMBER TESTED

TEST GROUP 2 (NOAEL-MIX) PREPUTIAL SEPARATION

	POSTPARTUM DAY												
FEMALE#	51	52	53	54	55	56	57	58	59	60	61	62	63
74	0/ 1	0/ 1	1/ 1										
75	1/ 1												
TOTAL	1/ 2	0/ 1	1/ 1										
CUMULATIVE	19/ 20<br>95%	19/ 20<br>95%	20/ 20<br>100%										

PR.NO.60R0375/88R002: REPRODUCTIVE TOX. STUDY TO DETECT EFFECTS
OF MIXED ANTI-ANDROGENIC SUBSTANCES IN RATS; ORAL ADM. (GAVAGE)
NUMBER OF PUPS WITH MEASURE PRESENT / NUMBER TESTED

TEST GROUP 3 (LOAEL-MIX) PREPUTIAL SEPARATION

	POSTPARTUM DAY												
FEMALE#	51	52	53	54	55	56	57	58	59	60	61	62	63
77	0/ 1	0/ 1	0/ 1	0/ 1	0/ 1	0/ 1	0/ 1	0/ 1	0/ 1	1/ 1			
80	1/ 1												
85	0/ 1	0/ 1	1/ 1										
86	0/ 1	0/ 1	1/ 1										
87	0/ 2	1/ 2	0/ 1	0/ 1	0/ 1	1/ 1							
93	0/ 1	0/ 1	0/ 1	0/ 1	1/ 1								
94	0/ 1	0/ 1	0/ 1	0/ 1	1/ 1								
95	0/ 1	0/ 1	0/ 1	0/ 1	0/ 1	1/ 1							
96	0/ 2	0/ 2	0/ 2	0/ 2	0/ 2	0/ 2	1/ 2	1/ 1					
97	0/ 1	0/ 1	0/ 1	0/ 1	0/ 1	0/ 1	0/ 1	1/ 1					
98	0/ 1	0/ 1	0/ 1	0/ 1	0/ 1	0/ 1	1/ 1						
99	0/ 2	0/ 2	0/ 2	1/ 2	0/ 1	0/ 1	1/ 1						
100	0/ 1	0/ 1	0/ 1	0/ 1	0/ 1	0/ 1	0/ 1	1/ 1					
TOTAL	1/ 16	1/ 15	2/ 14	1/ 12	2/ 11	2/ 9	3/ 7	3/ 4	0/ 1	1/ 1			
CUMULATIVE	5/ 20<br>25%	6/ 20<br>30%	8/ 20<br>40%	9/ 20<br>45%	11/ 20<br>55%	13/ 20<br>65%	16/ 20<br>80%	19/ 20<br>95%	19/ 20<br>95%	20/ 20<br>100%			

PR.NO.60R0375/88R002: REPRODUCTIVE TOX. STUDY TO DETECT EFFECTS
OF MIXED ANTI-ANDROGENIC SUBSTANCES IN RATS; ORAL ADM. (GAVAGE)
INDIVIDUAL PUP WEIGHTS (G) AT DAY OF PREPUTIAL SEPARATION

TEST GROUP 0 0 MG/KG BW/D			TEST GROUP 1 ADI-MIX			TEST GROUP 2 NOAEL-MIX			TEST GROUP 3 LOAEL-MIX			TEST GROUP 4 0.00025 MG/KG BW/D		
DAM-PUP	day	weight	DAM-PUP	day	weight	DAM-PUP	day	weight	DAM-PUP	day	weight	DAM-PUP	day	weight
1-04	44	185.5	26-02	42	158.5	51-02	42	162.9	77-03	60	236.7	101-02	40	183.0
3-01	50	215.7	26-03	48	187.6	53-02	46	173.8	78-07	49	195.1	102-05	47	171.2
4-02	41	158.1	28-01	44	176.9	54-03	48	187.5	80-01	51	211.5	103-01	47	178.9
4-04	44	171.5	28-02	42	175.3	55-04	42	158.0	82-02	50	201.1	103-04	47	210.3
6-02	42	164.4	29-06	42	188.8	57-01	46	194.0	83-04	48	187.0	104-08	47	194.4
8-04	49	166.5	30-04	44	171.8	57-05	43	174.1	84-03	50	218.0	105-03	42	177.1
9-01	46	172.9	32-02	43	200.7	58-03	46	188.8	85-01	53	232.1	107-01	43	172.0
10-02	47	185.3	33-01	48	208.4	59-03	43	174.5	86-02	53	212.0	108-03	49	195.4
10-05	48	207.9	34-05	45	181.9	61-02	46	207.3	87-01	52	196.5	111-03	48	201.4
17-01	48	173.0	35-03	43	182.5	61-05	49	199.5	87-05	56	243.1	117-02	45	179.0
18-03	45	182.0	36-04	45	181.5	62-03	49	207.7	93-03	55	227.3	118-01	40	170.4
19-01	49	206.8	43-04	49	187.3	68-01	49	184.8	94-04	55	199.0	118-05	41	176.3
20-02	49	188.4	43-06	45	161.8	69-05	46	202.8	95-01	56	250.1	119-01	48	218.0
21-05	48	194.4	45-03	43	171.5	70-01	47	201.4	96-03	58	201.9	119-02	47	229.4
22-01	43	184.7	46-01	46	163.5	71-03	50	226.8	96-05	57	216.1	120-03	47	186.4
22-04	42	172.8	46-07	48	183.1	72-05	45	191.6	97-07	58	258.7	121-01	45	200.2
23-02	47	200.4	47-02	45	195.2	73-02	47	186.7	98-03	57	215.5	123-01	44	194.4
24-03	47	181.1	49-02	50	222.1	74-01	48	176.8	99-01	57	242.0	124-02	49	214.0
25-05	44	171.5	49-04	47	196.5	74-04	53	191.1	99-02	54	222.9	124-03	49	197.6
			50-01	47	190.1	75-03	51	205.0	100-04	58	240.2	125-07	48	184.9

PR.NO.60R0375/88R002: REPRODUCTIVE TOX. STUDY TO DETECT EFFECTS
OF MIXED ANTI-ANDROGENIC SUBSTANCES IN RATS; ORAL ADM. (GAVAGE)
ESTROUS CYCLE STAGES

TEST GROUP 0 (0 MG/KG BW/D)

	DAY OF EVALUATION																												NUMBER OF DAYS IN STAGE					
FEMALE#	0	1	2	3	4	5	6	7	8	9	10	11	12	13	14	15	16	17	18	19	20	21	22	23	24	25	26	27	28	D	P	E	M	NC	MDE
801		D	P	/E	M	D	P	/E	M	D	P	/E	M	D	P	/E	M	M	P	/E	M	M	P	/E	M					4	6	6	8	5	4.0
802		D	/E	M	D	D	/E	M	D	P	/E	M	D	D	/E	M	M	P	/E	D	D	P	/E	M	D					9	3	6	6	5	4.0
803		/E	M	M	D	/E	M	M	D	/E	M	M	D	P	/E	M	D	D	/E	M	M	P	/E	M	D					6	2	6	10	5	4.2
804		M	D	P	/E	M	D	D	/E	M	D	D	D	D	D	D	D	D	D	D	/E	M	D	P	/E					14	2	4	4	3	6.7
806			D	/E	E	M	D	P	/E	M	D	P	/E	M	D	P	/E	M	D	P	/E	M	D	P	/E					6	5	7	5	5	4.2
807				M	D	D	D	D	D	D	D	D	D	/P	D	D	P	/E	M	D	D	/E	M	D	P	/E	M	D		15	3	3	4	3	4.0
808				D	P	/E	M	D	P	/E	M	D	D	D	D	D	D	D	D	D	D	D	D	D	P	/E	M	D		16	3	3	3	2	10.0
809				/E	M	D	P	/E	M	D	/P	M	D	D	D	D	D	D	D	/E	M	D	P	/E	M	D	P	/E		11	4	5	5	5	4.8
810					D	P	/E	M	D	D	/P	D	D	D	D	D	D	D	P	/E	M	M	P	/E	M	D	P	/E		11	5	4	4	4	5.3

STAGE P = PROESTRUS, STAGE E = ESTRUS, STAGE M = METESTRUS, STAGE D = DIESTRUS
NC=NUMBER OF CYCLES MDE=MEAN# OF DAYS OF CYCLE /=START OF CYCLE

PR.NO.60R0375/88R002: REPRODUCTIVE TOX. STUDY TO DETECT EFFECTS
OF MIXED ANTI-ANDROGENIC SUBSTANCES IN RATS; ORAL ADM. (GAVAGE)
ESTROUS CYCLE STAGES

TEST GROUP 1 (ADI-MIX)

	DAY OF EVALUATION																												NUMBER OF DAYS IN STAGE					
FEMALE#	0	1	2	3	4	5	6	7	8	9	10	11	12	13	14	15	16	17	18	19	20	21	22	23	24	25	26	27	28	D	P	E	M	NC	MDE
811		/E	M	D	P	/E	M	M	P	/E	M	D	P	/E	M	D	P	/E	M	D	P	/E	M	D	D					6	5	6	7	5	4.0
812		M	D	P	/E	M	D	P	/E	M	M	P	/E	M	D	P	/E	M	D	P	/E	M	D	P	/E					5	6	6	7	5	4.0
813		/E	M	D	D	/E	E	M	D	P	/E	M	D	P	/E	M	D	P	/E	M	D	D	/E	M	D					8	3	7	6	5	4.2
814		D	P	/E	M	D	P	/E	M	D	P	/E	M	D	P	/E	M	D	P	/E	M	D	P	/E	M					6	6	6	6	5	4.0
815			M	D	P	/E	M	D	P	/E	M	/E	M	M	D	D	D	D	D	D	D	D	/E	M	D	P	/E	M		11	3	5	7	4	5.3
816			D	/E	M	D	D	/E	M	D	D	D	D	D	P	/E	M	D	P	/E	M	D	P	/E	M					10	3	5	5	4	5.0
817			/E	M	D	/P	M	D	D	D	D	D	D	D	D	/E	M	D	P	/E	M	D	P	/E	M					11	3	4	5	4	5.3
818				D	P	/E	M	D	D	D	D	D	D	D	D	D	D	D	D	M	D	D	P	/E	M					15	2	2	3	1	18.0
819				D	P	/E	M	D	P	/E	M	D	D	D	D	D	D	D	D	D	D	D	/E	M	D	P	/E	M		14	3	4	4	3	7.0
820				D	/E	M	D	D	D	D	D	D	D	D	D	D	/E	M	D	P	/E	M	D	P	/E	M	D	P	/	14	3	4	4	4	6.0

STAGE P = PROESTRUS, STAGE E = ESTRUS, STAGE M = METESTRUS, STAGE D = DIESTRUS
NC=NUMBER OF CYCLES MDE=MEAN# OF DAYS OF CYCLE /=START OF CYCLE

16-APR-14

88R002S3 TABLE : IIA- 265

PR.NO.60R0375/88R002: REPRODUCTIVE TOX. STUDY TO DETECT EFFECTS
OF MIXED ANTI-ANDROGENIC SUBSTANCES IN RATS; ORAL ADM. (GAVAGE)
ESTROUS CYCLE STAGES

TEST GROUP 2 (NOAEL-MIX)

FEMALE#	DAY OF EVALUATION 0	1	2	3	4	5	6	7	8	9	10	11	12	13	14	15	16	17	18	19	20	21	22	23	24	25	26	27	28	NUMBER OF DAYS IN STAGE D	P	E	M	NC	MDE
821		M	D	P	/E	M	D	P	/E	M	D	P	/E	M	D	P	/E	M	D	P	/E	M	D	P	/E					6	6	6	6	5	4.0
822		/E	M	D	P	/E	M	D	P	/E	M	D	P	/E	M	D	P	/E	M	D	P	/E	M	D	D					7	5	6	6	5	4.0
823		M	D	P	/E	M	D	D	/P	M	D	D	D	D	D	D	D	/E	M	D	P	/E	M	D	P	/				12	4	3	5	4	5.3
824			/E	M	D	D	D	D	D	D	D	D	D	D	D	/E	D	D	P	/E	M	D	P	/E	M					14	2	4	3	3	7.0
825			D	/E	M	D	D	/E	E	M	M	D	D	D	D	D	D	D	D	D	D	P	/E	M	D					14	1	4	4	2	9.5
826				/E	M	D	D	/E	M	D	D	D	D	D	D	D	D	D	D	D	M	M	D	P	/E	M	D	P	/	15	2	3	5	3	8.3
827				M	D	P	/E	M	D	D	D	D	D	D	D	D	D	D	/P	M	M	P	/E	M	D	P	/E	M		12	4	3	6	3	6.7
828					D	P	/E	M	D	D	D	D	D	D	D	D	D	/P	M	D	D	P	/E	M	D	P	/E	M		13	4	3	4	3	6.7
829					M	D	/E	M	D	D	/E	M	M	D	/E	M	D	P	/E	M	D	P	/E	M	D	P	/E	M		7	3	6	8	5	4.0
830						D	P	/E	M	M	D	P	/E	M	D	P	/E	M	D	P	/E	M	D	P	/E	M	D	P	/	6	6	5	6	5	4.2

STAGE P = PROESTRUS, STAGE E = ESTRUS, STAGE M = METESTRUS, STAGE D = DIESTRUS
NC=NUMBER OF CYCLES MDE=MEAN# OF DAYS OF CYCLE /=START OF CYCLE

16-APR-14

88R002S3 TABLE : IIA- 266

PR.NO.60R0375/88R002: REPRODUCTIVE TOX. STUDY TO DETECT EFFECTS
OF MIXED ANTI-ANDROGENIC SUBSTANCES IN RATS; ORAL ADM. (GAVAGE)
ESTROUS CYCLE STAGES

TEST GROUP 3 (LOAEL-MIX)

	DAY OF EVALUATION																												NUMBER OF DAYS IN STAGE					
FEMALE#	0	1	2	3	4	5	6	7	8	9	10	11	12	13	14	15	16	17	18	19	20	21	22	23	24	25	26	27	28	D	P	E	M	NC	MDE
831		D	P	/E	M	D	D	D	D	D	D	D	D	D	D	P	/E	M	D	P	/E	M	D	P	/E					13	4	4	3	3	7.0
832			P	/E	M	D	D	D	D	D	D	D	D	D	D	/E	M	D	P	/E	M	D	P	/E	M					12	3	4	4	3	6.7
833				D	D	/E	M	D	D	D	D	D	D	D	D	D	D	/E	D	D	P	/E	D	D	P	/E	M	D		17	2	4	2	3	6.7
834				/E	M	D	D	P	/E	M	M	D	D	D	D	D	D	D	D	/P	M	D	D	P	/E	M	D	P	/	13	4	3	5	4	6.3
835				D	/E	M	D	D	D	D	D	D	D	D	D	D	P	/E	M	D	P	/E	M	D	P	/E	M	M		13	3	4	5	3	7.0
836					/E	M	D	D	D	D	D	D	D	D	D	D	/E	M	D	D	P	/E	M	D	D	P	/E	M		14	2	4	4	3	7.3
837					D	/E	E	E	E	M	M	D	D	D	D	D	D	D	D	D	/E	M	D	P	/E	M	D	D		13	1	6	4	2	9.5
838					D	/E	E	M	P	/E	M	D	D	D	D	D	D	D	D	P	/E	M	D	P	/E	M	M	P	/	10	4	5	5	4	5.8
839						/E	E	M	D	P	/E	M	D	D	P	/E	M	D	D	P	/E	M	D	D	P	/E	E	E		7	4	8	4	4	5.0
840						P	/E	M	D	/E	E	M	D	D	D	D	D	D	D	D	D	D	/E	M	D	P	/E	M		12	2	5	4	3	6.7

STAGE P = PROESTRUS, STAGE E = ESTRUS, STAGE M = METESTRUS, STAGE D = DIESTRUS
NC=NUMBER OF CYCLES MDE=MEAN# OF DAYS OF CYCLE /=START OF CYCLE

PR.NO.60R0375/88R002: REPRODUCTIVE TOX. STUDY TO DETECT EFFECTS
OF MIXED ANTI-ANDROGENIC SUBSTANCES IN RATS; ORAL ADM. (GAVAGE)
ESTROUS CYCLE STAGES

TEST GROUP 4(0.00025 MG/KG BW/D)

	DAY OF EVALUATION																												NUMBER OF DAYS IN STAGE					
FEMALE#	0	1	2	3	4	5	6	7	8	9	10	11	12	13	14	15	16	17	18	19	20	21	22	23	24	25	26	27	28	D	P	E	M	NC	MDE
841		/E	M	D	D	D	D	D	D	D	D	D	D	/E	M	D	P	/E	M	D	P	/E	M	D	P	/				13	3	4	4	4	6.0
842		D	/E	M	D	D	P	/E	M	D	/P	M	D	D	D	D	D	D	/E	M	D	P	/E	M	D					12	3	4	5	4	5.0
843		P	/E	M	D	P	/E	M	D	D	/E	E	M	D	D	P	/E	M	D	P	/E	M	D	P	/E					7	5	7	5	5	4.4
844		M	D	P	/E	M	D	P	/E	M	D	P	/E	M	D	P	/E	M	D	P	/E	M	D	P	/E					6	6	6	6	5	4.0
845			D	D	P	/E	M	D	/E	E	E	M	D	D	/E	M	D	P	/E	M	D	P	/E	M	D					8	3	7	5	4	4.3
846			D	/E	M	D	P	/E	M	D	D	D	D	D	D	D	D	D	D	D	M	M	D	P	/E					14	2	3	4	2	10.5
847			M	D	P	/E	M	D	D	/E	M	D	D	D	D	D	D	D	D	D	D	/E	M	D	P	/E	M	D		15	2	4	5	3	6.7
848					M	D	P	/E	M	D	P	/E	M	M	P	/E	M	D	P	/E	M	D	P	/E	M	D	P	/E		5	6	6	7	5	4.0
849					D	P	/E	M	D	P	/E	M	D	P	/E	D	D	P	/E	M	D	P	/E	M	D	P	/E	M		7	6	6	5	5	4.0
850					/E	M	D	P	/E	M	D	P	/E	M	D	P	/E	M	D	P	/E	M	D	P	/E	M	D	P	/	6	6	6	6	6	4.0

STAGE P = PROESTRUS, STAGE E = ESTRUS, STAGE M = METESTRUS, STAGE D = DIESTRUS
NC=NUMBER OF CYCLES MDE=MEAN# OF DAYS OF CYCLE /=START OF CYCLE

Study **60R0375/88R002** Study Type **R Reproduction**
Species **Rat** Article

Sex: **Female** - Phase: **In-life**

Dose Group	Animal Number	day 31
	001	32.45
	002	34.25
	003	29.66
	004	21.40
	005	32.32
	006	42.04
	007	32.58
	008	27.88
	009	30.06
G 0 / F	010	28.80
	011	21.88
	017	28.14
	018	26.48
	019	25.42
	020	26.28
	021	10.23
	022	15.36
	023	24.09
	025	19.07

Study **60R0375/88R002** Study Type **R Reproduction**
Species **Rat** Article

Sex: **Female** - Phase: **In-life**

Dose Group	Animal Number	day 31
G 1 / F	026	26.35
	027	21.74
	028	25.35
	029	18.48
	030	20.73
	031	21.20
	032	24.09
	033	23.22
	034	12.76
	035	38.89
	036	28.07
	037	32.85
	043	30.26
	044	11.43
	045	28.47
	046	23.29
	047	8.99
	048	25.00
	049	23.70
	050	21.57

Study **60R0375/88R002** Study Type **R Reproduction**
Species **Rat** Article

Sex: **Female** - Phase: **In-life**

Dose Group	Animal Number	day 31
	051	17.02
	052	26.08
	053	21.81
	054	14.06
	056	23.22
	057	18.48
	058	21.61
	059	29.33
	060	28.60
G 2 / F	061	18.20
	062	19.30
	068	27.08
	069	20.80
	070	24.02
	071	16.12
	072	14.27
	073	5.00
	074	12.04
	075	20.48

Study **60R0375/88R002** Study Type **R Reproduction**
Species **Rat** Article

Sex: **Female** - Phase: **In-life**

Dose Group	Animal Number	day 31
G 3 / F	078	13.55
	079	12.83
	080	5.00
	081	44.75
	082	18.27
	083	12.76
	084	19.09
	085	9.16
	086	20.19
	087	18.55
	093	13.19
	094	17.65
	095	19.91
	096	19.37
	097	5.00
	098	16.19
	099	16.83
	100	17.54

Study **60R0375/88R002** Study Type **R Reproduction**
Species **Rat** Article

Sex: **Female** - Phase: **In-life**

Dose Group	Animal Number	day 31
	101	34.45
	102	21.20
	103	23.62
	104	35.52
	105	25.55
	106	28.34
	107	29.13
	108	20.25
	109	26.55
G 4 / F	110	24.29
	111	31.19
	117	41.48
	118	24.55
	119	26.88
	120	26.02
	121	26.81
	123	26.45
	124	20.16
	125	16.58

Study **60R0375/88R002** Study Type **R Reproduction**
Species **Rat** Article

Sex: **Female** - Phase: **In-life**

Dose Group	Animal Number	day 29
G 0 / F	601	6.89
	602	14.09
	603	10.23
	604	5.00
	605	8.99
	606	10.04
	607	11.46
	608	6.76
	609	6.49
	610	8.08

Study	**60R0375/88R002**	Study Type	**R Reproduction**
Species	**Rat**	Article	

Sex: **Female** - Phase: **In-life**

Dose Group	Animal Number	day 29
G 1 / F	611	5.00
	612	8.54
	613	11.91
	614	5.02
	615	13.00
	616	5.83
	617	11.97
	618	9.91
	619	15.24
	620	5.00

Study **60R0375/88R002** Study Type **R Reproduction**
Species **Rat** Article

Sex: **Female** - Phase: **In-life**

Dose Group	Animal Number	day 29
G 2 / F	621	5.00
	622	10.62
	623	5.29
	624	12.04
	625	6.49
	626	5.00
	627	7.02
	628	7.88
	629	12.36
	630	11.33

Study	**60R0375/88R002**	Study Type	**R Reproduction**
Species	**Rat**	Article	

Sex: **Female** - Phase: **In-life**

Dose Group	Animal Number	day 29
G 3 / F	631	10.04
	632	5.00
	633	17.09
	634	8.28
	635	5.36
	636	5.00
	637	13.26
	638	5.36
	639	8.47
	640	11.13

Study **60R0375/88R002** Study Type **R Reproduction**
Species **Rat** Article

Sex: **Female** - Phase: **In-life**

Dose Group	Animal Number	day 29
G 4 / F	641	7.82
	642	5.89
	643	10.55
	644	5.00
	645	7.29
	646	12.81
	647	10.68
	648	6.36
	649	12.74
	650	11.01

Study **60R0375/88R002** Study Type **R Reproduction**
Species **Rat** Article

Sex: **Female** - Phase: **In-life**

Dose Group	Animal Number	day 70
G 0 / F	801	32.87
	802	22.67
	803	21.49
	804	13.89
	806	37.71OL
	807	33.16
	808	26.54
	809	5.86
	810	31.92

OL = Outlier

Study	**60R0375/88R002**	Study Type	**R Reproduction**
Species	**Rat**	Article	

Sex: **Female** - Phase: **In-life**

Dose Group	Animal Number	day 70
G 1 / F	811	20.93
	812	23.79
	813	11.92
	814	16.19
	815	21.83
	816	17.17
	817	20.58
	818	26.90
	819	26.90
	820	32.80

E2 [pmol/L] in female pups on about PND 83 in proestrus

ToxData© System 3.0

12-Feb-2014 11:53

Study	**60R0375/88R002**	Study Type	**R Reproduction**
Species	**Rat**	Article	

Sex: **Female** - Phase: **In-life**

Dose Group	Animal Number	day 70
G 2 / F	821	13.61
	822	18.21
	823	22.53
	824	18.14
	825	29.97
	826	5.56
	827	7.62
	828	11.77
	829	29.54
	830	13.04

Study	**60R0375/88R002**	Study Type	**R Reproduction**
Species	**Rat**	Article	

Sex: **Female** - Phase: **In-life**

Dose Group	Animal Number	day 70
G 3 / F	831	23.58
	832	25.76
	833	11.00
	834	11.99
	835	12.20
	836	27.11
	837	16.96
	838	22.53
	839	8.92
	840	21.14

Study **60R0375/88R002** Study Type **R Reproduction**
Species **Rat** Article

Sex: **Female** - Phase: **In-life**

Dose Group	Animal Number	day 70
G 4 / F	841	28.18
	842	27.82
	843	27.25
	844	21.14
	845	23.09
	846	16.89
	847	22.32
	848	29.68
	849	18.14
	850	18.28

Study	**60R0375/88R002**	Study Type	**R Reproduction**
Species	**Rat**	Article	

Sex: **Male** - Phase: **In-life**

Dose Group	Animal Number	MOTILE_C [%] day 84	TS/gT [---] day 84	TS/gC [---] day 84	ABNORMAL_C [%] day 84
G 0 / M	701	88	182	681	98
	702	85	164	600	98
	704	85	236	610	98
	705	83	201	598	99
	706	91	223	608	96
	707	97	216	608	98
	708	98	194	560	98
	709	95	204	623	96
	710	98	175	896	98

Study **60R0375/88R002** Study Type **R Reproduction**
Species **Rat** Article

Sex: **Male** - Phase: **In-life**

Dose Group	Animal Number	MOTILE_C [%] day 84	TS/gT [---] day 84	TS/gC [---] day 84	ABNORMAL_C [%] day 84
G 1 / M	711	82	211	619	98
	712	90	188	650	100
	713	86	179	456	100
	714	69	188	602	98
	715	90	211	611	96
	716	92	219	547	98
	717	88	284	818	99
	718	95	236	719	98
	719	96	181	659	96
	720	90	199	463	95

Study	**60R0375/88R002**	Study Type	**R Reproduction**
Species	**Rat**	Article	

Sex: **Male** - Phase: **In-life**

Dose Group	Animal Number	MOTILE_C [%] day 84	TS/gT [---] day 84	TS/gC [---] day 84	ABNORMAL_C [%] day 84
G 2 / M	721	88	214	1,000	98
	722	87	217	883	98
	723	92	214	486	98
	724	88	221	563	98
	725	97	200	529	98
	726	97	170	741	99
	727	98	264	785	98
	728	91	224	518	98
	729	90	173	918	99
	730	88	229	831	96

Study **60R0375/88R002** Study Type **R Reproduction**
Species **Rat** Article

Sex: **Male** - Phase: **In-life**

Dose Group	Animal Number	MOTILE_C [%] day 84	TS/gT [---] day 84	TS/gC [---] day 84	ABNORMAL_C [%] day 84
G 3 / M	731	78	213	1,304	100
	732	84	205	750	98
	733	97	177	483	96
	734	99	194	880	97
	735	93	164	607	98
	736	94	205	536	98
	737	92	220	476	97
	738	96	181	519	98
	739	93	236	895	96
	740	94	190	859	98

Study **60R0375/88R002** Study Type **R Reproduction**
Species **Rat** Article

Sex: **Male** - Phase: **In-life**

Dose Group	Animal Number	MOTILE_C [%] day 84	TS/gT [---] day 84	TS/gC [---] day 84	ABNORMAL_C [%] day 84
G 4 / M	741	85	204	523	98
	742	84	279	653	100
	743	80	239	494	99
	744	100	298	835	100
	745	93	311	710	99
	746	94	164	871	96
	747	97	203	683	96
	748	94	224	628	99
	749	95	230	779	98
	750	96	181	593	97

Project 60R0375/88R002
Dams after weaning in proestrus

IIB 21

ANIMAL_ID	DAY	Group	Androstenedione nmol/l	Testosterone nmol/l	Progesterone nmol/l	11-desoxycorticosterone nmol/l	Corticosterone nmol/l
1	41	0	1.45	0.37	13.26	2.81	459.5
2	41	0	1.39	0.36	18.86	6.39	826.5
3	41	0	1.84	0.35	6.93	1.62	191.6
4	41	0	1.16	0.35	11.35	10.62	1153.1
5	41	0	2.88	0.58	34.03	2.39	257.2
6	41	0	4.49	1.11	31.16	10.97	1231.1
7	41	0	1.76	0.35	13.55	4.77	520.2
8	41	0	2.81	0.53	13.04	7.41	933.5
9	41	0	1.73	0.35	5.02	1.59	165.3
10	41	0	0.92	0.35	11.45	4.01	650.3
11	41	0	1.00	0.35	12.37	4.26	554.9
17	41	0	1.18	0.37	11.73	7.92	1063.5
18	41	0	0.99	0.35	12.37	12.97	1156.0
20	41	0	0.89	0.35	11.54	4.48	586.7
21	41	0	0.35	0.35	181.58	2.72	500.0
23	41	0	1.02	0.35	15.01	4.67	832.3
25	41	0	1.28	0.35	15.71	18.16	1355.4

Project 60R0375/88R002
Dams after weaning in proestrus

ANIMAL_ID	DAY	Group	Androstenedione nmol/l	Testosterone nmol/l	Progesterone nmol/l	11-desoxycorticosterone nmol/l	Corticosterone nmol/l
26	41	1	1.35	0.35	15.11	9.73	604.0
27	41	1	0.88	0.35	8.65	5.02	580.9
28	41	1	0.93	0.35	11.00	5.06	734.1
29	41	1	0.91	0.35	12.50	7.98	864.1
30	41	1	0.46	0.35	27.98	25.41	1121.3
31	41	1	1.28	0.35	13.67	5.41	794.8
32	41	1	0.69	0.35	16.15	15.61	997.1
33	41	1	1.07	0.35	26.43	14.53	1367.0
34	41	1	0.59	0.35	6.30	2.03	526.0
35	41	1	3.19	0.81	29.61	3.75	656.0
36	41	1	2.62	0.45	11.99	7.44	572.2
37	41	1	1.67	0.53	9.89	3.43	502.9
43	41	1	1.38	0.35	29.99	31.61	1791.8
44	41	1	0.35	0.35	162.18	3.08	615.6
45	41	1	1.91	0.35	8.68	4.01	1034.6
46	41	1	0.89	0.35	15.14	12.75	1575.1
47	41	1	0.35	0.35	180.31	1.59	227.7
48	41	1	2.47	0.44	29.54	22.13	921.9
49	41	1	1.34	0.35	7.92	1.98	215.3
50	41	1	0.82	0.35	22.45	36.57	1563.5
51	41	2	1.00	0.35	20.92	10.40	1641.5
52	41	2	0.91	0.35	11.83	4.64	1063.5
53	41	2	1.99	0.51	18.48	13.83	1130.0
54	41	2	0.57	0.39	15.84	4.29	916.1
56	41	2	1.76	0.48	9.09	6.11	849.7
57	41	2	0.90	0.54	15.96	33.07	1416.1
58	41	2	3.27	0.99	23.12	12.78	1419.0
59	41	2	1.76	0.35	21.75	5.41	745.6
60	41	2	1.20	0.35	16.41	15.87	1104.0
61	41	2	2.69	0.61	11.70	1.59	404.6
62	41	2	1.16	0.35	18.44	1.59	72.3
68	41	2	2.42	0.35	11.07	12.12	1401.7
69	41	2	1.48	0.35	10.65	7.31	751.4
70	41	2	3.32	0.71	15.30	5.69	580.9
71	41	2	1.57	0.35	12.15	5.69	797.6
72	41	2	1.65	0.35	14.12	1.59	245.4
73	41	2	0.35	0.35	166.31	1.59	263.6
74	41	2	0.35	0.35	105.26	1.59	352.6
75	41	2	1.10	0.35	7.12	2.25	861.2
78	41	3	5.55	0.62	23.25	3.66	794.8
79	41	3	2.50	0.49	40.07	40.07	1632.9
80	41	3	0.38	0.35	260.12	11.89	1219.6
81	41	3	5.90	1.04	20.07	9.79	1288.9
82	41	3	2.45	0.48	12.56	18.22	1343.9
83	41	3	3.31	0.65	24.49	8.75	1014.4
84	41	3	1.61	0.35	11.13	8.78	997.1
85	41	3	0.35	0.35	239.45	6.93	531.8
86	41	3	2.76	0.88	16.73	6.20	1092.4
93	41	3	1.21	0.35	12.24	7.09	742.7
94	41	3	2.38	0.42	9.57	3.13	760.1
95	41	3	4.66	0.66	130.70	14.50	763.0
96	41	3	10.11	1.11	70.91	10.21	641.6
97	41	3	0.35	0.35	17.04	1.59	140.2
98	41	3	1.30	0.35	10.40	12.24	1549.0
99	41	3	3.48	0.63	28.17	15.87	1306.3
100	41	3	1.50	0.35	8.55	3.82	589.6

Project 60R0375/88R002
Dams after weaning in proestrus

ANIMAL_ID	DAY	Group	Androstenedione nmol/l	Testosterone nmol/l	Progesterone nmol/l	11-desoxycorticosterone nmol/l	Corticosterone nmol/l
101	41	4	0.82	0.35	12.72	4.74	612.7
102	41	4	0.58	0.35	13.64	1.86	167.0
103	41	4	1.12	0.35	21.43	6.26	742.7
104	41	4	2.80	0.56	15.33	8.68	1005.7
105	41	4	1.27	0.35	7.54	4.80	635.8
106	41	4	1.35	0.35	26.39	7.38	838.1
107	41	4	1.91	0.39	101.76	15.26	1317.8
108	41	4	0.84	0.35	53.74	49.61	2528.8
110	41	4	1.68	0.41	9.00	2.25	445.1
111	41	4	2.25	0.44	17.36	1.89	378.6
117	41	4	3.35	0.62	20.45	11.45	1176.2
118	41	4	0.83	0.35	11.32	8.68	1005.7
119	41	4	2.05	0.40	13.67	12.75	988.4
120	41	4	0.92	0.35	7.28	6.55	979.7
121	41	4	1.18	0.35	22.55	20.92	1728.2
123	41	4	1.65	0.35	12.24	4.20	346.8
124	41	4	1.26	0.35	14.79	15.39	1052.0
125	41	4	0.87	0.35	14.72	12.50	1508.6

Project 60R0375/88R002 IIB 24
Males PND21

ANIMAL_ID	DAY	Group	Androstenedione	Testosterone	Progesterone	Corticosterone	Cortisol
Unit			nmol/L	nmol/L	nmol/l	nmol/l	nmol/l
301	21	0	0.35	0.35	6.71	924.8	0.28
302	21	0	0.45	0.48	7.06	936.4	0.56
303	21	0	1.51	1.87	4.61	884.3	1.03
304	21	0	0.35	0.35	7.63	829.4	0.95
305	21	0	0.35	0.35	10.30	881.5	1.18
306	21	0	0.41	0.36	13.86	737.0	0.41
307	21	0	0.35	0.35	2.97	679.2	0.75
308	21	0	0.59	0.76	6.90	728.3	1.08
309	21	0	1.51	1.96	5.31	872.8	0.80
310	21	0	0.35	0.35	4.23	638.7	0.85

Project 60R0375/88R002
Males PND21

IIB 25

ANIMAL_ID Unit	DAY	Group	Androstenedione nmol/L	Testosterone nmol/L	Progesterone nmol/l	Corticosterone nmol/l	Cortisol nmol/l
311	21	1	0.35	0.35	11.03	858.3	0.38
312	21	1	0.55	0.82	1.95	621.4	0.44
313	21	1	0.35	0.35	1.41	436.4	0.42
314	21	1	0.35	0.35	3.78	647.4	0.35
315	21	1	0.45	0.66	2.64	523.1	0.52
316	21	1	0.35	0.35	6.30	794.8	1.50
317	21	1	1.23	1.76	10.91	864.1	0.30
318	21	1	0.35	0.35	9.54	696.5	
319	21	1	0.35	0.39	2.61	621.4	0.59
321	21	2	0.35	0.35	3.02	823.7	1.62
322	21	2	0.35	0.35	3.37	806.3	0.31
323	21	2	0.35	0.35	4.42	913.2	0.62
324	21	2	0.58	0.75	4.36	705.2	0.49
325	21	2	0.35	0.35	1.35	641.6	0.60
326	21	2	1.07	1.58	5.06	971.0	0.83
327	21	2	0.69	0.90	1.69	500.0	0.52
328	21	2	0.73	0.82	5.57	710.9	0.88
329	21	2	0.35	0.35	1.78	583.8	1.32
331	21	3	0.35	0.35	2.04	601.1	0.78
332	21	3	0.35	0.35	1.59	572.2	0.61
333	21	3	0.35	0.35	2.47	725.4	0.59
334	21	3	0.35	0.35	1.42	650.3	0.28
335	21	3	0.37	0.52	3.40	702.3	1.41
336	21	3	0.41	0.36	3.85	731.2	0.77
337	21	3	0.35	0.35	1.85	809.2	0.41
339	21	3	0.35	0.35	3.91	823.7	0.87
340	21	3	1.97	3.03	1.40	586.7	0.60

Project 60R0375/88R002 IIB 26
Males PND21

ANIMAL_ID	DAY	Group	Androstenedione	Testosterone	Progesterone	Corticosterone	Cortisol
Unit			nmol/L	nmol/L	nmol/l	nmol/l	nmol/l
341	21	4	0.89	1.07	5.57	615.6	0.48
342	21	4	0.35	0.35	3.28	765.9	0.77
343	21	4	0.37	0.35	4.20	644.5	0.86
344	21	4	0.35	0.35	4.61	803.4	0.28
345	21	4	0.35	0.35	6.14	817.9	1.08
346	21	4	0.35	0.35	0.84	624.2	0.52
347	21	4	0.43	0.45	3.16	635.8	0.53
348	21	4	0.42	0.35	3.21	841.0	2.00
349	21	4	4.11	3.07	13.45	881.5	1.95
350	21	4	1.85	2.28	5.91	806.3	1.34

Project 60R0375/88R002 IIB 27
Females PND21

ANIMAL_ID	DAY	Group	Androstenedione nmol/l	Progesterone nmol/l	Corticosterone nmol/l	Cortisol nmol/l
401	21	0	0.35	2.18	612.7	1.12
402	21	0	0.35	7.22	982.6	1.75
403	21	0	0.35	9.03	1161.8	0.75
404	21	0	0.35	2.10	572.2	0.36
405	21	0	0.35	4.77	699.4	0.28
406	21	0	0.35	6.71	803.4	1.02
407	21	0	0.42	8.30	973.9	1.23
408	21	0	0.35	5.37	997.1	1.28
409	21	0	0.35		595.3	0.69
410	21	0	0.35	4.01	627.1	1.73

Project 60R0375/88R002 IIB 28
Females PND21

ANIMAL_ID	DAY	Group	Androstenedione nmol/l	Progesterone nmol/l	Corticosterone nmol/l	Cortisol nmol/l
411	21	1	4.86		893.0	1.01
412	21	1	0.39	4.83	710.9	
413	21	1	0.35	4.83	765.9	0.48
414	21	1	0.51	7.50	751.4	0.43
415	21	1	0.35	4.55	838.1	0.59
416	21	1	0.35	7.22	867.0	1.24
417	21	1	0.35	3.37	846.8	0.46
418	21	1	0.35	1.37	589.6	0.55
419	21	1	0.35	4.96	606.9	0.28
420	21	1	0.35	8.17	696.5	0.28
421	21	2	1.66	7.79	739.8	0.41
422	21	2	0.35	2.64	789.0	0.57
423	21	2	0.35	4.42	867.0	1.48
424	21	2	0.61	5.44	849.7	0.78
425	21	2	0.35	4.64	742.7	0.37
426	21	2	0.35	3.01	835.2	1.96
427	21	2	0.35	4.39	716.7	0.39
428	21	2	0.35	3.66	869.9	0.42
429	21	2	1.02	1.41	485.5	0.30
430	21	2	0.35	2.64	725.4	0.97
431	21	3	0.35	7.85	904.6	1.18
432	21	3	0.35	5.18	812.1	0.50
433	21	3	0.48	2.72	430.6	0.36
434	21	3	0.35	4.26	734.1	0.28
435	21	3	0.35	5.44	699.4	0.61
436	21	3	0.35	3.91	895.9	1.04
437	21	3	0.35	3.43	664.7	0.59
438	21	3	0.35	2.24	514.4	0.28
439	21	3	0.35	4.58	852.6	0.63
440	21	3	0.35	0.57	387.3	0.35

Project 60R0375/88R002
Females PND21

ANIMAL_ID	DAY	Group	Androstenedione nmol/l	Progesterone nmol/l	Corticosterone nmol/l	Cortisol nmol/l
441	21	4	0.41	5.66	682.0	0.38
442	21	4	0.46	3.69	696.5	1.39
443	21	4	0.35	4.93	826.5	0.65
444	21	4	0.35	3.82	820.8	0.71
445	21	4	0.35	6.74	988.4	0.64
446	21	4	0.52	10.40	973.9	0.28
447	21	4	0.35	1.34	702.3	0.56
448	21	4	0.58	4.10	589.6	
449	21	4	0.35	5.63	670.5	0.54
450	21	4	1.75	9.54	867.0	0.73

Project 60R0375/88R002 IIB 30

Males at sexual maturity

ANIMAL_ID	DAY	Group	Androstenedione	Testosterone	Progesterone	11-desoxycorticosterone	Corticosterone
Unit			nmol/L	nmol/L	nmol/l	nmol/l	nmol/l
501	35	0	0.62	3.33	0.32	1.59	100.0
502	35	0	0.35	1.01	1.54	1.59	200.0
503	35	0	2.06	10.13	3.88	4.58	566.4
504	35	0	0.96	6.04	3.53	2.29	887.2
505	35	0	0.46	2.17	1.57	2.32	864.1
506	35	0	0.35	0.65	1.19	2.47	540.4
507	35	0	0.35	1.31	4.80	3.75	872.8
508	35	0	0.35	0.54	2.02	2.52	468.2
509	35	0	0.35	1.18	0.32	1.59	86.7
510	35	0	0.65	4.27	0.35	1.59	82.1

Project 60R0375/88R002
Males at sexual maturity

ANIMAL_ID<br>Unit	DAY	Group	Androstenedione<br>nmol/L	Testosterone<br>nmol/L	Progesterone<br>nmol/l	11-desoxycorticosterone<br>nmol/l	Corticosterone<br>nmol/l
511	35	1	1.16	4.93	0.90	1.78	381.5
512	35	1	1.01	5.31	8.65	5.47	867.0
513	35	1	0.35	1.07	4.96	5.09	789.0
514	35	1	0.49	3.96	0.75	1.59	137.0
515	35	1	0.65	3.19	2.73	2.42	566.4
516	35	1	0.35	0.72	13.64	11.10	1028.8
517	35	1	0.59	4.41	2.30	3.63	768.7
518	35	1	0.35	1.49	0.95	1.59	491.3
519	35	1	0.77	3.85	3.21	3.82	872.8
520	35	1	0.35	0.64	0.77	1.59	456.6
521	35	2	0.61	2.95	2.03	3.94	575.1
522	35	2	0.35	0.60	11.67	5.69	699.4
523	35	2	0.35		3.34	5.31	575.1
524	35	2	0.56	2.64	5.22	3.72	719.6
525	35	2	0.35	0.91	0.32	1.59	54.6
526	35	2	0.52	2.70	2.44	2.40	534.7
527	35	2	1.21	5.79	0.67	1.72	424.8
528	35	2	0.78	3.85	1.14	2.38	312.1
529	35	2	0.65	4.20	3.18	3.78	838.1
530	35	2	0.35	1.25	0.32	1.59	26.7
531	35	3	0.76	5.62	3.94	5.57	494.2
532	35	3	0.35	2.79	1.73	3.05	624.2
533	35	3	0.35	1.39	0.32	1.59	43.6
534	35	3	0.94	6.52	8.55	7.09	1075.1
535	35	3	0.35	1.65	0.32	1.59	128.6
536	35	3	0.93	6.21	1.52	1.59	193.6
537	35	3	0.35	2.11	0.63	1.59	113.6
538	35	3	1.19	6.25	0.32	1.59	117.9
539	35	3	0.54	3.34	3.94	3.66	754.3
540	35	3	0.50	3.47	2.09	1.91	742.7

Project 60R0375/88R002
Males at sexual maturity

IIB 32

ANIMAL_ID Unit	DAY	Group	Androstenedione nmol/L	Testosterone nmol/L	Progesterone nmol/l	11-desoxycorticosterone nmol/l	Corticosterone nmol/l
541	35	4	0.35	0.35	0.37	1.59	329.5
542	35	4	0.35	1.15	0.33	1.59	269.6
543	35	4	0.77	4.27	0.92	1.59	300.6
544	35	4	0.35	0.68	3.56	1.84	609.8
545	35	4	0.56	2.00	41.66	17.20	1265.8
546	35	4	0.35	1.56	0.77	1.59	286.1
547	35	4	0.64	2.30	3.88	4.99	751.4
548	35	4	0.57	3.78	0.32	1.59	18.8
549	35	4	0.35	0.74	2.15	2.29	748.5
550	35	4	0.35	2.53	1.12	3.03	453.7

Project 60R0375/88R002 IIB 33

Females at sexual maturity

ANIMAL_ID	DAY	Group	Androstenedione nmol/l	Testosterone nmol/l	Progesterone nmol/l	11-desoxycorticosterone nmol/l	Corticosterone nmol/l
601	35	0	0.35	0.35	11.64	1.59	528.9
602	35	0	0.49	0.35	9.41	4.20	1132.9
603	35	0	0.35	0.35	10.75	3.59	1020.2
604	35	0	0.35		8.11	1.59	20.5
605	35	0	0.35	0.35	15.49	1.69	312.1
606	35	0	0.35	0.35	12.02	1.59	69.6
607	35	0	0.35	0.35	11.19	1.59	13.9
608	35	0	0.35	0.50	7.92	1.59	126.6
609	35	0	0.35	0.35	3.69	1.59	53.5
610	35	0	0.35	0.35	9.06	1.59	159.2

Project 60R0375/88R002
Females at sexual maturity

ANIMAL_ID	DAY	Group	Androstenedione nmol/l	Testosterone nmol/l	Progesterone nmol/l	11-desoxycorticosterone nmol/l	Corticosterone nmol/l
611	35	1	0.35	0.37	8.87	1.59	27.3
612	35	1	0.35	0.35	11.13	1.59	18.5
613	35	1	0.35	0.35	5.41	4.20	731.2
614	35	1	0.35	0.35	1.20	1.59	43.1
615	35	1	0.35	0.35	21.15	1.59	656.0
616	35	1	0.35	0.35	9.19	2.19	858.3
617	35	1	0.35	0.35	19.65	1.59	243.3
618	35	1	0.35	0.35	11.16	1.59	63.0
619	35	1	0.36	0.35	1.81	1.59	459.5
620	35	1	0.35	0.35	8.97	1.59	140.2
621	35	2	0.35	0.35	10.27	1.59	53.8
622	35	2	0.35	0.35	1.07	1.59	46.2
623	35	2	0.35	0.35	4.13	1.59	220.2
624	35	2	0.41	0.35	0.45	1.59	121.4
625	35	2	0.35	0.35	26.17	1.59	184.7
626	35	2	0.35	0.35	17.90	1.59	135.3
627	35	2	0.35	0.35	9.89	1.59	58.4
628	35	2	0.35	0.35	14.47	4.80	1127.1
629	35	2	0.42	0.35	30.40	4.52	1557.7
630	35	2	0.35	0.35	2.66	2.55	1127.1
631	35	3	0.35	0.35	4.77	2.05	910.4
632	35	3	0.35	0.35	7.92	1.59	28.7
633	35	3	0.43	0.35	4.32	1.98	514.4
634	35	3	0.44	0.35	0.51	1.59	306.3
635	35	3	0.56	0.35		3.82	1268.7
636	35	3	0.35	0.35	0.43	1.59	72.3
637	35	3	0.72	0.35	2.85	1.59	387.3
638	35	3	0.35	0.35	12.21	1.59	50.9
639	35	3	0.55	0.35	22.61	8.55	1456.6
640	35	3	0.35	0.35	18.32	5.88	1372.8

Project 60R0375/88R002
Females at sexual maturity

IIB 35

ANIMAL_ID	DAY	Group	Androstenedione nmol/l	Testosterone nmol/l	Progesterone nmol/l	11-desoxycorticosterone nmol/l	Corticosterone nmol/l
641	35	4	0.35	0.35	0.91	1.59	235.2
642	35	4	0.35		10.72	1.59	32.1
643	35	4	0.70	0.35	29.10	7.54	1144.4
644	35	4	0.35	0.35	8.11	1.59	133.5
645	35	4	0.35		2.72	1.59	335.2
646	35	4	0.35	0.35	2.68	1.59	502.9
647	35	4	0.35	0.35	10.72	1.59	63.9
648	35	4	0.35	0.35	11.10	1.59	99.7
649	35	4	0.35	0.35	9.38	1.59	335.2
650	35	4	0.35	0.35	4.61	2.17	927.7

Project 60R0375/88R002
Males PND 83

ANIMAL_ID	DAY	Group	Androstenedione	Testosterone	Progesterone	11-desoxycorticosterone	Corticosterone
Unit			nmol/L	nmol/L	nmol/l	nmol/l	nmol/l
701	83	0	0.39	2.01	3.56	18.03	812.1
702	83	0	1.71	9.68	2.62	6.52	393.0
704	83	0	1.73	9.06	3.31	10.81	592.5
705	83	0	2.70	15.68	0.64	3.31	289.0
706	83	0	1.17	6.32	0.56	1.62	141.0
708	83	0	0.37	2.15	0.62	3.56	398.8
709	83	0	0.55	3.29	0.43	1.59	141.3
710	83	0	0.60	3.68	4.74	13.64	708.1

Project 60R0375/88R002
Males PND 83

IIB 37

ANIMAL_ID<br>Unit	DAY	Group	Androstenedione<br>nmol/L	Testosterone<br>nmol/L	Progesterone<br>nmol/l	11-desoxycorticosterone<br>nmol/l	Corticosterone<br>nmol/l
711	83	1	1.21	6.87	2.33	4.80	375.7
712	83	1	0.52	1.60	13.71	28.37	1176.2
713	83	1	2.21	8.47	1.21	3.69	410.4
714	83	1	1.23	6.59	5.25	10.30	777.4
715	83	1	0.46	2.24	9.00	11.83	1378.5
716	83	1	0.62	2.80	7.12	13.13	1156.0
717	83	1	0.93	4.48	0.68	2.55	176.3
718	83	1	0.44	3.68	1.35	3.12	208.4
719	83	1	4.86	18.70	1.08	3.37	198.0
720	83	1	0.35	1.49	7.57	15.14	884.3
721	83	2	0.58	1.87	0.32	1.59	232.9
722	83	2	1.05	4.13	6.23	17.55	852.6
723	83	2	0.37	2.01	0.42	1.75	145.7
724	83	2	3.45	8.19	1.59	11.38	693.6
725	83	2	0.84	4.96	1.98	5.91	326.6
726	83	2	1.90	5.97	0.93	2.53	323.7
727	83	2	0.75	3.82	4.83	21.34	1144.4
728	83	2	0.36	1.93	1.07	4.96	485.5
729	83	2	0.79	2.30	2.08	4.74	445.1
730	83	2	0.48	3.06	3.31	13.96	991.3
731	83	3	1.51	4.72	7.47	19.59	1080.9
732	83	3	1.31	7.53	2.13	11.48	815.0
733	83	3	3.07	13.74	2.00	4.36	282.6
734	83	3	0.75	1.85	2.76	10.56	531.8
735	83	3	1.68	5.24	1.12	4.04	320.8
736	83	3	2.39	9.37	6.65	16.50	1358.3
737	83	3	1.57	4.20	2.61	7.41	725.4
738	83	3	5.87	12.35	1.61	2.99	193.9
739	83	3	0.59	2.00	0.87	2.04	201.1
740	83	3	1.80	2.74	0.39	1.59	174.0

Project 60R0375/88R002
Males PND 83

ANIMAL_ID Unit	DAY	Group	Androstenedione nmol/L	Testosterone nmol/L	Progesterone nmol/l	11-desoxycorticosterone nmol/l	Corticosterone nmol/l
741	83	4	0.37	1.68	16.25	27.25	1106.9
742	83	4	1.79	10.48	3.88	19.14	800.5
743	83	4	0.36	1.73	3.05	6.36	936.4
744	83	4	0.97	5.03	4.58	15.65	705.2
745	83	4	0.35	1.03	3.21	9.03	300.6
746	83	4	1.20	7.46	3.63	12.82	528.9
747	83	4	0.40	2.54	0.32	1.59	73.4
748	83	4	3.83	23.91	10.59	30.56	1239.8
749	83	4	0.88	6.49	2.14	6.23	445.1
750	83	4	0.73	3.68	4.23	10.68	1023.1

Project 60R0375/88R002
Females around PND 83 in proestrus

ANIMAL_ID	DAY	Group	Androstenedione nmol/l	Testosterone nmol/l	Progesterone nmol/l	11-desoxycorticosterone nmol/l	Corticosterone nmol/l
801	83	0	1.43	0.46	10.53	1.73	192.2
802	83	0	1.35	0.35	7.60	1.59	35.3
803	83	0	0.83	0.35	4.80	2.88	274.8
804	83	0	0.57	0.35	5.79	1.59	72.3
806	83	0	1.67	0.39	8.71	10.56	1286.1
807	83	0	3.17	0.68	54.38	1.62	54.6
808	83	0	4.04	0.54	21.27	11.26	930.6
809	83	0	0.56	0.35	190.80	1.59	225.7
810	83	0	3.21	0.76	58.51	11.26	1069.3

Project 60R0375/88R002 IIB 40

Females around PND 83 in proestrus

ANIMAL_ID	DAY	Group	Androstenedione nmol/l	Testosterone nmol/l	Progesterone nmol/l	11-desoxycorticosterone nmol/l	Corticosterone nmol/l
811	83	1	1.42	0.35	6.42	2.08	178.6
812	83	1	0.94	0.35	7.12	3.82	375.7
813	83	1	1.61	0.35	8.52	1.59	247.1
814	83	1	0.55	0.35	7.92	5.22	832.3
815	83	1	3.17	0.51	98.58	2.29	303.5
816	83	1	0.75	0.35	25.89	42.61	1875.6
817	83	1	2.04	0.41	11.13	12.59	1167.6
818	83	1	1.36	0.35	21.47	26.71	1485.5
819	83	1	1.26	0.35	3.34	1.83	213.9
820	83	1	3.03	0.52	12.12	3.50	332.4
821	83	2	0.65	0.35	6.42	3.47	272.8
822	83	2	1.10	0.35	8.97	2.37	224.6
823	83	2	0.97	0.35	9.35	6.65	427.7
824	83	2	0.46	0.35	8.17	4.42	765.9
825	83	2	8.56	1.42	65.51	12.94	630.0
826	83	2	0.47	0.35	9.64	4.48	734.1
827	83	2	0.35	0.35	20.57	1.59	64.2
828	83	2	2.32	0.35	171.72	19.08	1502.8
829	83	2	3.25	0.44	19.33	19.21	1358.3
830	83	2	0.47	0.35	16.92	19.02	1525.9
831	83	3	2.35	0.43	11.67	9.32	858.3
832	83	3	21.42	2.58	192.71	10.78	1601.1
833	83	3	8.56	1.27	135.79	16.03	1210.9
834	83	3	1.77	0.35	147.55	13.67	1653.1
835	83	3	2.69	0.48	34.03	53.42	1615.5
836	83	3	1.02	0.35	15.45	12.56	832.3
837	83	3	3.66	0.66	10.97	10.81	1251.4
838	83	3	18.60	1.93	21.12	14.06	1130.0
839	83	3	1.40	0.44	9.51	2.97	326.6
840	83	3	1.44	0.35	10.56	4.71	352.6

Project 60R0375/88R002
Females around PND 83 in proestrus

ANIMAL_ID	DAY	Group	Androstenedione nmol/l	Testosterone nmol/l	Progesterone nmol/l	11-desoxycorticosterone nmol/l	Corticosterone nmol/l
841	83	4	3.27	0.50	75.37	12.12	1497.0
842	83	4	3.33	0.59	18.48	27.51	1052.0
843	83	4	4.76	0.61	77.27	30.78	1300.5
844	83	4	1.62	0.35	15.30	16.38	1011.5
845	83	4	0.90	0.35	14.41	13.10	1101.1
846	83	4	1.17	0.35	21.31	31.20	1225.4
847	83	4	1.06	0.35	5.15	2.53	274.8
848	83	4	2.26	0.47	11.58	6.65	1216.7
849	83	4	4.07	0.59	27.60	23.75	1453.7
850	83	4	1.74	0.35	14.02	11.26	962.4

Project No.: 60R0375/88R002

IIB 42

Group	Fetus No.	Testis	Final result testosterone (nmol/g)	Mean testosterone left/right testis
0	12/1	li	20.02	20.58
0	12/1	re	21.13	
0	12/2	li	14.89	14.63
0	12/2	re	14.36	
0	12/3	li	17.35	14.28
0	12/3	re	11.20	
0	12/4	li	21.13	21.95
0	12/4	re	22.78	
0	12/5	li	18.63	18.55
0	12/5	re	18.48	
0	12/6	li	25.09	23.32
0	12/6	re	21.55	
0	13/1	li	21.40	21.21
0	13/1	re	21.01	
0	13/2	li	20.28	20.96
0	13/2	re	21.64	
0	13/3	li	19.40	19.54
0	13/3	re	19.68	
0	14/1	li	15.20	17.53
0	14/1	re	19.86	
0	14/2	li	20.53	18.26
0	14/2	re	16.00	
0	14/3	li	20.83	19.34
0	14/3	re	17.85	
0	14/4	li	19.85	19.26
0	14/4	re	18.66	
0	14/5	li	12.40	11.31
0	14/5	re	10.23	
0	14/6	li	11.57	10.14
0	14/6	re	8.72	
0	15/1	li	21.29	21.18
0	15/1	re	21.06	
0	15/2	li	20.90	21.66
0	15/2	re	22.43	
0	15/3	li	21.59	23.30
0	15/3	re	25.01	
0	15/4	li	21.87	19.19
0	15/4	re	16.51	
0	15/5	li	26.01	25.10
0	15/5	re	24.20	
0	16/1	li	18.44	18.22
0	16/1	re	18.01	
0	16/2	li	19.88	19.01
0	16/2	re	18.13	
0	16/3	li	18.41	20.52
0	16/3	re	22.63	
0	16/4	li	24.24	20.54
0	16/4	re	16.83	

Project No.: 60R0375/88R002 IIB 43

Group	Fetus No.	Testis	Final result testosterone (nmol/g)	Mean testosterone left/right testis (nmol/g)
1	38/1	li	22.22	22.39
1	38/1	re	22.56	
1	38/2	li	13.54	17.06
1	38/2	re	20.59	
1	38/3	li	25.11	24.56
1	38/3	re	24.02	
1	38/4	li	20.01	20.88
1	38/4	re	21.75	
1	39/1	li	30.22	28.58
1	39/1	re	26.94	
1	39/2	li	41.55	36.15
1	39/2	re	30.75	
1	39/3	li	25.44	22.42
1	39/3	re	19.39	
1	39/4	li	16.15	15.08
1	39/4	re	14.01	
1	39/5	li	27.65	23.91
1	39/5	re	20.16	
1	39/6	li	31.29	26.88
1	39/6	re	22.48	
1	39/7	li	24.35	21.75
1	39/7	re	19.16	
1	40/1	li	22.15	20.00
1	40/1	re	17.84	
1	40/2	li	11.55	10.72
1	40/2	re	9.88	
1	40/3	li	10.66	10.95
1	40/3	re	11.25	
1	40/4	li	21.33	17.38
1	40/4	re	13.43	
1	40/5	li	3.96	5.48
1	40/5	re	7.00	
1	40/6	li	12.43	10.52
1	40/6	re	8.62	
1	40/7	li	15.62	13.68
1	40/7	re	11.73	
1	40/8	li	22.50	20.25
1	40/8	re	18.00	
1	41/1	li	22.46	18.78
1	41/1	re	15.10	
1	41/2	li	19.68	22.26
1	41/2	re	24.85	
1	41/4	li	7.04	11.44
1	41/4	re	15.83	
1	41/5	li	24.75	22.78
1	41/5	re	20.82	
1	41/6	li	20.62	24.65
1	41/6	re	28.68	
1	41/7	li	19.82	16.16
1	41/7	re	12.50	
1	42/1	li	20.38	22.86
1	42/1	re	25.35	
1	42/2	li	17.87	10.20
1	42/2	re	2.53	
1	42/3	li	19.70	23.43
1	42/3	re	27.15	
1	42/4	li	21.86	21.80
1	42/4	re	21.74	
1	42/5	li	36.35	29.61
1	42/5	re	22.87	
1	42/6	li	21.35	18.54
1	42/6	re	15.73	
1	42/7	li	19.55	18.05
1	42/7	re	16.54	

Project No.: 60R0375/88R002 IIB 44

Group	Fetus No.	Testis	Final result testosterone (nmol/g)	Mean testosterone left/right testis
2	63/1	li	25.16	25.00
2	63/1	re	24.85	
2	63/2	li	21.59	19.47
2	63/2	re	17.35	
2	63/3	li	16.81	21.06
2	63/3	re	25.30	
2	63/4	li	18.14	20.33
2	63/4	re	22.51	
2	64/1	li	17.59	14.15
2	64/1	re	10.72	
2	64/2	li	18.02	17.63
2	64/2	re	17.25	
2	64/3	li	11.66	13.96
2	64/3	re	16.26	
2	64/4	li	25.02	21.34
2	64/4	re	17.65	
2	65/1	li	18.43	17.71
2	65/1	re	17.00	
2	65/2	li	21.83	19.58
2	65/2	re	17.33	
2	65/3	li	24.16	20.16
2	65/3	re	16.15	
2	65/4	li	26.66	25.93
2	65/4	re	25.20	
2	66/1	li	18.81	15.74
2	66/1	re	12.68	
2	66/2	li	17.24	16.04
2	66/2	re	14.85	
2	66/3	li	14.94	19.14
2	66/3	re	23.35	
2	66/4	li	12.41	13.88
2	66/4	re	15.34	

Group	Fetus No.	Testis	Final result testosterone (nmol/g)	Mean testosterone left/right testis (nmol/g)
3	88/1	li	15.16	14.23
3	88/1	re	13.30	
3	88/2	li	17.68	19.10
3	88/2	re	20.51	
3	88/3	li	25.25	21.96
3	88/3	re	18.68	
3	88/4	li	14.51	14.08
3	88/4	re	13.64	
3	89/1	li	25.88	21.95
3	89/1	re	18.01	
3	89/2	li	14.87	15.44
3	89/2	re	16.01	
3	89/3	li	11.37	15.18
3	89/3	re	19.00	
3	89/4	li	12.23	12.68
3	89/4	re	13.14	
3	89/5	li	15.07	14.82
3	89/5	re	14.57	
3	89/6	li	12.77	14.38
3	89/6	re	16.00	
3	89/7	li	23.99	17.44
3	89/7	re	10.89	
3	90/1	li	1.67	1.01
3	90/1	re	0.35	
3	90/2	li	11.17	10.33
3	90/2	re	9.50	
3	90/3	li	11.15	8.82
3	90/3	re	6.50	
3	90/4	li	22.00	18.30
3	90/4	re	14.61	
3	91/1	li	23.55	24.40
3	91/1	re	25.24	
3	91/2	li	17.44	16.26
3	91/2	re	15.08	
3	91/3	li	12.28	13.89
3	91/3	re	15.51	
3	91/4	li	19.15	18.45
3	91/4	re	17.74	
3	91/5	li	15.26	18.03
3	91/5	re	20.81	
3	91/6	li	20.41	17.65
3	91/6	re	14.88	
3	92/1	li	22.01	19.95
3	92/1	re	17.88	
3	92/2	li	17.06	15.76
3	92/2	re	14.45	
3	92/3	li	13.90	13.83
3	92/3	re	13.77	
3	92/4	li	18.02	16.74
3	92/4	re	15.46	
3	92/5	li	15.39	17.04
3	92/5	re	18.69	

Project No.: 60R0375/88R002

Group	Fetus No.	Testis	Final result testosterone (nmol/g)	Mean testosterone left/right testis
4	112/1	re	30.63	
4	112/2	li	15.89	16.60
4	112/2	re	17.31	
4	112/3	li	18.51	19.43
4	112/3	re	20.34	
4	112/4	li	24.16	23.82
4	112/4	re	23.48	
4	112/5	li	21.71	21.08
4	112/5	re	20.44	
4	113/1	li	9.55	10.45
4	113/1	re	11.35	
4	113/2	li	12.92	12.12
4	113/2	re	11.31	
4	113/3	li	11.38	11.30
4	113/3	re	11.21	
4	113/4	li	19.69	19.69
4	113/4	re	19.69	
4	114/1	li	31.23	27.08
4	114/1	re	22.94	
4	114/2	li	17.00	17.26
4	114/2	re	17.53	
4	114/3	li	18.11	14.74
4	114/3	re	11.37	
4	114/4	li	20.93	17.84
4	114/4	re	14.75	
4	114/5	li	13.00	11.62
4	114/5	re	10.25	
4	114/6	li	13.64	12.13
4	114/6	re	10.62	
4	115/1	li	20.58	21.23
4	115/1	re	21.87	
4	115/2	li	23.88	20.34
4	115/2	re	16.81	
4	115/3	li	23.74	25.08
4	115/3	re	26.41	
4	115/4	li	27.10	27.98
4	115/4	re	28.85	
4	115/5	li	16.54	16.94
4	115/5	re	17.34	
4	115/6	li	32.87	31.15
4	115/6	re	29.44	
4	116/1	li	14.14	13.08
4	116/1	re	12.02	
4	116/2	li	14.27	13.14
4	116/2	re	12.02	
4	116/3	li	22.86	23.61
4	116/3	re	24.36	
4	116/4	li	20.02	20.64
4	116/4	re	21.27	

Reproductive Toxicity Study to detect potential effects to anti-androgenic substances in Wistar Rats (Gavage) 28.Mar.2014 SIGR

ABSOLUTE WEIGHTS - INDIVIDUAL VALUES
PARENTAL FEMALES

Sacrifice F1
Sex F
Group 0

	Term. body weight g	Adrenal glands mg	Brain g	Kidneys g	Liver g	Ovaries mg
M	231.23	70.75	1.862	1.616	7.46	115.35
SD	15.499	8.583	0.07	0.125	0.547	18.933
n	20	20	20	20	20	20
1	226.5	71.0	1.84	1.63	6.79	116.0
2	221.5	83.0	1.87	1.58	7.16	120.0
3	238.3	83.0	1.82	1.67	7.35	113.0
4	233.0	67.0	1.9	1.58	7.46	106.0
5	235.4	75.0	1.85	1.56	7.46	162.0
6	204.1	65.0	1.84	1.51	6.65	114.0
7	243.8	84.0	1.99	1.73	7.55	139.0
8	219.7	75.0	1.84	1.55	7.21	112.0
9	207.1	59.0	1.83	1.5	6.84	121.0
10	224.7	56.0	1.9	1.65	7.33	138.0
11	228.9	58.0	1.8	1.56	7.64	101.0
17	236.7	74.0	1.86	1.59	8.11	95.0
18	243.6	70.0	1.8	1.64	8.22	105.0
19	216.9	68.0	1.7	1.45	6.89	96.0
20	206.8	71.0	1.86	1.39	6.84	76.0
21	251.5	71.0	1.92	1.92	7.78	114.0
22	235.9	58.0	1.85	1.65	8.09	120.0
23	260.4	80.0	2.04	1.86	8.75	99.0
24	251.1	69.0	1.89	1.59	7.75	134.0
25	238.7	78.0	1.84	1.7	7.32	126.0

Reproductive Toxicity Study to detect potential effects to anti-androgenic substances in Wistar Rats (Gavage) 28.Mar.2014 SIGR

ABSOLUTE WEIGHTS - INDIVIDUAL VALUES
PARENTAL FEMALES

Sacrifice F1
Sex F
Group 0

	Pituitary gland mg	Spleen g	Thyroid glands mg	Uterus mg
M	11.85	0.448	18.85	709.0
SD	1.348	0.074	3.066	276.194
n	20	20	20	20
1	15.0	0.37	22.0	710.0
2	12.0	0.36	14.0	720.0
3	12.0	0.45	19.0	570.0
4	12.0	0.49	14.0	750.0
5	12.0	0.34	16.0	610.0
6	11.0	0.32	19.0	420.0
7	13.0	0.49	20.0	1650.0
8	12.0	0.46	22.0	660.0
9	12.0	0.41	14.0	950.0
10	11.0	0.46	17.0	750.0
11	10.0	0.5	21.0	780.0
17	12.0	0.55	26.0	530.0
18	12.0	0.39	18.0	560.0
19	11.0	0.39	18.0	800.0
20	10.0	0.43	18.0	940.0
21	11.0	0.48	22.0	400.0
22	11.0	0.4	18.0	890.0
23	14.0	0.54	21.0	480.0
24	14.0	0.54	20.0	470.0
25	10.0	0.58	18.0	540.0

Reproductive Toxicity Study to detect potential effects to anti-androgenic substances in Wistar Rats (Gavage) 28.Mar.2014 SIGR

ABSOLUTE WEIGHTS - INDIVIDUAL VALUES
PARENTAL FEMALES

Sacrifice F1
Sex F
Group 1

	Term. body weight g	Adrenal glands mg	Brain g	Kidneys g	Liver g	Ovaries mg
M	236.437	72.158	1.908	1.641	7.352	122.211
SD	16.024	11.102	0.065	0.142	0.892	15.292
n	19	19	19	19	19	19
26	221.1	75.0	1.86	1.55	6.02	123.0
27	267.0	75.0	1.93	1.96	8.74	116.0
29	261.3	97.0	1.87	1.7	9.19	141.0
30	238.4	68.0	1.98	1.64	7.36	134.0
31	244.4	53.0	1.92	1.69	7.46	119.0
32	259.7	86.0	1.92	1.79	7.84	147.0
33	234.7	63.0	2.02	1.65	7.18	141.0
34	214.5	57.0	1.87	1.54	6.41	110.0
35	252.3	79.0	2.0	1.71	7.4	117.0
36	243.7	81.0	1.85	1.64	7.56	110.0
37	215.3	51.0	1.76	1.31	6.73	112.0
43	220.2	66.0	1.94	1.54	6.87	124.0
44	226.6	74.0	1.9	1.62	7.18	153.0
45	220.2	71.0	1.9	1.53	6.68	119.0
46	234.0	76.0	1.94	1.53	6.34	117.0
47	240.2	74.0	1.92	1.63	6.34	128.0
48	234.4	73.0	1.85	1.69	8.14	92.0
49	244.5	80.0	1.83	1.88	8.86	112.0
50	219.8	72.0	1.99	1.58	7.38	107.0

Reproductive Toxicity Study to detect potential effects to anti-androgenic substances in Wistar Rats (Gavage)

28.Mar.2014 SIGR

ABSOLUTE WEIGHTS - INDIVIDUAL VALUES
PARENTAL FEMALES

Sacrifice F1
Sex F
Group 1

	Pituitary gland mg	Spleen g	Thyroid glands mg	Uterus mg
M	13.474	0.481	19.632	693.158
SD	2.458	0.068	4.072	242.145
n	19	19	19	19
26	11.0	0.42	18.0	580.0
27	12.0	0.54	18.0	900.0
29	10.0	0.66	19.0	670.0
30	11.0	0.6	12.0	610.0
31	14.0	0.48	21.0	570.0
32	18.0	0.45	21.0	860.0
33	13.0	0.43	17.0	740.0
34	13.0	0.44	16.0	630.0
35	13.0	0.48	17.0	760.0
36	13.0	0.53	17.0	510.0
37	17.0	0.45	24.0	460.0
43	12.0	0.45	19.0	1210.0
44	12.0	0.49	20.0	570.0
45	13.0	0.49	15.0	1350.0
46	13.0	0.49	29.0	700.0
47	14.0	0.35	18.0	470.0
48	14.0	0.44	24.0	530.0
49	20.0	0.47	22.0	570.0
50	13.0	0.48	26.0	480.0

ABSOLUTE WEIGHTS - INDIVIDUAL VALUES
PARENTAL FEMALES

Sacrifice F1
Sex F
Group 2

	Term. body weight g	Adrenal glands mg	Brain g	Kidneys g	Liver g	Ovaries mg
M	231.87	74.9	1.88	1.633	7.358	124.05
SD	15.524	9.894	0.063	0.088	0.472	21.951
n	20	20	20	20	20	20
51	213.4	95.0	1.8	1.66	7.23	139.0
52	249.7	85.0	1.83	1.65	7.7	131.0
53	217.1	63.0	1.91	1.61	6.42	132.0
54	237.2	72.0	1.82	1.59	7.34	121.0
55	237.2	57.0	1.89	1.63	7.53	96.0
56	241.7	80.0	1.91	1.73	7.79	174.0
57	251.4	73.0	1.93	1.62	7.3	129.0
58	216.8	72.0	1.74	1.51	7.62	122.0
59	250.6	78.0	1.87	1.86	8.06	138.0
60	246.8	86.0	1.95	1.71	7.51	133.0
61	235.8	88.0	1.98	1.62	7.18	143.0
62	227.6	69.0	1.94	1.62	7.15	129.0
68	235.8	77.0	1.88	1.61	7.38	105.0
69	219.0	80.0	1.88	1.67	7.42	112.0
70	241.8	79.0	1.83	1.6	7.38	109.0
71	205.4	65.0	1.78	1.5	6.85	97.0
72	253.9	79.0	1.94	1.75	8.23	109.0
73	212.6	57.0	1.94	1.63	6.88	102.0
74	209.7	71.0	1.9	1.47	6.4	94.0
75	233.9	72.0	1.87	1.62	7.78	166.0

Reproductive Toxicity Study to detect potential effects
to anti-androgenic substances in Wistar Rats (Gavage)

ABSOLUTE WEIGHTS - INDIVIDUAL VALUES
PARENTAL FEMALES

Sacrifice F1
Sex F
Group 2

	Pituitary gland mg	Spleen g	Thyroid glands mg	Uterus mg
M	12.8	0.463	19.65	633.0
SD	1.881	0.083	3.856	192.685
n	20	20	20	20
51	11.0	0.41	24.0	680.0
52	14.0	0.49	23.0	710.0
53	10.0	0.4	17.0	940.0
54	13.0	0.44	16.0	470.0
55	11.0	0.48	21.0	510.0
56	14.0	0.41	18.0	450.0
57	13.0	0.4	15.0	520.0
58	11.0	0.53	16.0	570.0
59	13.0	0.49	21.0	1040.0
60	15.0	0.34	26.0	590.0
61	15.0	0.62	27.0	730.0
62	13.0	0.49	16.0	580.0
68	12.0	0.56	14.0	500.0
69	13.0	0.52	18.0	530.0
70	14.0	0.56	20.0	560.0
71	12.0	0.4	19.0	1120.0
72	12.0	0.6	17.0	510.0
73	11.0	0.39	19.0	430.0
74	11.0	0.35	20.0	570.0
75	18.0	0.37	26.0	650.0

Reproductive Toxicity Study to detect potential effects to anti-androgenic substances in Wistar Rats (Gavage) 28.Mar.2014 SIGR

ABSOLUTE WEIGHTS - INDIVIDUAL VALUES
PARENTAL FEMALES

Sacrifice F1
Sex F
Group 3

	Term. body weight g	Adrenal glands mg	Brain g	Kidneys g	Liver g	Ovaries mg
M	234.953	82.789	1.884	1.74	9.066	131.368
SD	13.556	13.138	0.054	0.117	0.884	23.726
n	19	19	19	19	19	19
77	247.8	79.0	1.91	1.57	8.69	104.0
78	244.3	113.0	1.9	1.71	10.4	157.0
79	234.3	80.0	1.92	1.62	7.77	110.0
80	224.4	69.0	1.87	1.63	8.12	120.0
81	241.0	81.0	1.95	1.75	8.25	119.0
82	238.3	102.0	1.9	1.81	9.08	139.0
83	237.6	80.0	1.84	1.68	9.15	112.0
84	201.6	62.0	1.81	1.5	7.58	161.0
85	233.9	69.0	1.96	1.75	8.78	116.0
86	240.1	85.0	1.8	1.95	9.62	150.0
87	250.9	100.0	1.83	1.86	10.26	186.0
93	232.7	71.0	1.81	1.86	10.1	116.0
94	245.1	95.0	1.96	1.82	9.61	115.0
95	251.5	73.0	1.87	1.76	9.9	111.0
96	237.4	87.0	1.96	1.68	9.48	121.0
97	228.8	88.0	1.9	1.86	9.46	155.0
98	213.4	69.0	1.9	1.71	7.83	116.0
99	213.7	90.0	1.82	1.66	8.39	166.0
100	247.3	80.0	1.88	1.88	9.78	122.0

Reproductive Toxicity Study to detect potential effects to anti-androgenic substances in Wistar Rats (Gavage)

ABSOLUTE WEIGHTS - INDIVIDUAL VALUES
PARENTAL FEMALES

Sacrifice F1
Sex F
Group 3

	Pituitary gland mg	Spleen g	Thyroid glands mg	Uterus mg
M	12.0	0.481	20.947	681.579
SD	1.528	0.052	4.196	173.47
n	19	19	19	19
77	9.0	0.41	17.0	560.0
78	12.0	0.52	15.0	530.0
79	11.0	0.44	20.0	880.0
80	11.0	0.47	19.0	510.0
81	12.0	0.45	23.0	530.0
82	13.0	0.41	15.0	690.0
83	13.0	0.48	17.0	900.0
84	12.0	0.44	16.0	500.0
85	10.0	0.56	19.0	490.0
86	12.0	0.48	28.0	1020.0
87	15.0	0.48	28.0	710.0
93	11.0	0.53	22.0	510.0
94	13.0	0.53	20.0	630.0
95	14.0	0.44	27.0	940.0
96	14.0	0.57	25.0	520.0
97	11.0	0.53	24.0	840.0
98	10.0	0.42	22.0	620.0
99	12.0	0.44	23.0	830.0
100	13.0	0.54	18.0	740.0

ABSOLUTE WEIGHTS - INDIVIDUAL VALUES
PARENTAL FEMALES

Sacrifice F1
Sex F
Group 4

	Term. body weight g	Adrenal glands mg	Brain g	Kidneys g	Liver g	Ovaries mg
M	237.437	76.053	1.891	1.685	7.389	112.842
SD	18.521	10.363	0.086	0.147	0.727	14.237
n	19	19	19	19	19	19
101	268.9	79.0	1.95	1.74	8.77	105.0
102	235.1	65.0	2.01	1.63	7.38	123.0
103	237.7	80.0	1.93	1.59	7.54	146.0
104	243.0	89.0	1.85	1.73	7.66	98.0
105	248.7	82.0	2.03	1.8	7.84	104.0
106	240.6	83.0	1.93	1.73	7.02	132.0
107	244.0	83.0	1.9	1.66	7.51	113.0
108	195.3	64.0	1.68	1.34	5.67	93.0
109	239.8	53.0	1.85	1.56	6.66	114.0
110	219.4	65.0	1.96	1.44	6.43	94.0
111	260.5	90.0	1.98	1.82	8.08	98.0
117	207.0	70.0	1.76	1.6	6.62	123.0
118	231.4	81.0	1.83	1.65	7.58	107.0
119	246.3	72.0	1.9	1.68	7.49	115.0
120	235.8	70.0	1.84	1.77	8.13	108.0
121	254.9	80.0	1.94	1.89	7.82	117.0
123	226.2	86.0	1.84	1.95	6.93	136.0
124	259.0	88.0	1.9	1.8	8.17	109.0
125	217.7	65.0	1.84	1.63	7.1	109.0

Reproductive Toxicity Study to detect potential effects to anti-androgenic substances in Wistar Rats (Gavage) 28.Mar.2014 SIGR

ABSOLUTE WEIGHTS - INDIVIDUAL VALUES
PARENTAL FEMALES

Sacrifice F1
Sex F
Group 4

	Pituitary gland mg	Spleen g	Thyroid glands mg	Uterus mg
M	11.474	0.472	19.526	677.368
SD	1.867	0.06	3.627	289.211
n	19	19	19	19
101	12.0	0.48	14.0	560.0
102	10.0	0.47	19.0	880.0
103	12.0	0.43	17.0	470.0
104	13.0	0.49	23.0	540.0
105	12.0	0.55	19.0	1230.0
106	13.0	0.43	16.0	780.0
107	12.0	0.41	15.0	490.0
108	9.0	0.44	18.0	420.0
109	11.0	0.36	17.0	460.0
110	7.0	0.51	16.0	550.0
111	12.0	0.56	19.0	1320.0
117	9.0	0.45	17.0	560.0
118	14.0	0.46	24.0	490.0
119	12.0	0.44	28.0	480.0
120	10.0	0.59	20.0	1010.0
121	11.0	0.54	22.0	1120.0
123	14.0	0.4	22.0	570.0
124	14.0	0.52	24.0	510.0
125	11.0	0.44	21.0	430.0

RELATIVE WEIGHTS - INDIVIDUAL VALUES
PARENTAL FEMALES

Sacrifice F1
Sex F
Group 0

	Term. body weight %	Adrenal glands %	Brain %	Kidneys %	Liver %	Ovaries %
M	100.0	0.031	0.808	0.699	3.227	0.05
SD		0.004	0.048	0.031	0.128	0.008
n	20	20	20	20	20	20
1	100.0	0.031	0.812	0.72	2.998	0.051
2	100.0	0.037	0.844	0.713	3.233	0.054
3	100.0	0.035	0.764	0.701	3.084	0.047
4	100.0	0.029	0.815	0.678	3.202	0.045
5	100.0	0.032	0.786	0.663	3.169	0.069
6	100.0	0.032	0.902	0.74	3.258	0.056
7	100.0	0.034	0.816	0.71	3.097	0.057
8	100.0	0.034	0.838	0.706	3.282	0.051
9	100.0	0.028	0.884	0.724	3.303	0.058
10	100.0	0.025	0.846	0.734	3.262	0.061
11	100.0	0.025	0.786	0.682	3.338	0.044
17	100.0	0.031	0.786	0.672	3.426	0.04
18	100.0	0.029	0.739	0.673	3.374	0.043
19	100.0	0.031	0.784	0.669	3.177	0.044
20	100.0	0.034	0.899	0.672	3.308	0.037
21	100.0	0.028	0.763	0.763	3.093	0.045
22	100.0	0.025	0.784	0.699	3.429	0.051
23	100.0	0.031	0.783	0.714	3.36	0.038
24	100.0	0.027	0.753	0.633	3.086	0.053
25	100.0	0.033	0.771	0.712	3.067	0.053

RELATIVE WEIGHTS - INDIVIDUAL VALUES
PARENTAL FEMALES

Sacrifice F1
Sex F
Group 0

	Pituitary gland %	Spleen %	Thyroid glands %	Uterus %
M	0.005	0.193	0.008	0.309
SD	0.001	0.027	0.001	0.121
n	20	20	20	20
1	0.007	0.163	0.01	0.313
2	0.005	0.163	0.006	0.325
3	0.005	0.189	0.008	0.239
4	0.005	0.21	0.006	0.322
5	0.005	0.144	0.007	0.259
6	0.005	0.157	0.009	0.206
7	0.005	0.201	0.008	0.677
8	0.005	0.209	0.01	0.3
9	0.006	0.198	0.007	0.459
10	0.005	0.205	0.008	0.334
11	0.004	0.218	0.009	0.341
17	0.005	0.232	0.011	0.224
18	0.005	0.16	0.007	0.23
19	0.005	0.18	0.008	0.369
20	0.005	0.208	0.009	0.455
21	0.004	0.191	0.009	0.159
22	0.005	0.17	0.008	0.377
23	0.005	0.207	0.008	0.184
24	0.006	0.215	0.008	0.187
25	0.004	0.243	0.008	0.226

RELATIVE WEIGHTS - INDIVIDUAL VALUES
PARENTAL FEMALES

Sacrifice F1
Sex F
Group 1

	Term. body weight %	Adrenal glands %	Brain %	Kidneys %	Liver %	Ovaries %
M	100.0	0.03	0.81	0.694	3.106	0.052
SD		0.004	0.055	0.035	0.264	0.007
n	19	19	19	19	19	19
26	100.0	0.034	0.841	0.701	2.723	0.056
27	100.0	0.028	0.723	0.734	3.273	0.043
29	100.0	0.037	0.716	0.651	3.517	0.054
30	100.0	0.029	0.831	0.688	3.087	0.056
31	100.0	0.022	0.786	0.691	3.052	0.049
32	100.0	0.033	0.739	0.689	3.019	0.057
33	100.0	0.027	0.861	0.703	3.059	0.06
34	100.0	0.027	0.872	0.718	2.988	0.051
35	100.0	0.031	0.793	0.678	2.933	0.046
36	100.0	0.033	0.759	0.673	3.102	0.045
37	100.0	0.024	0.817	0.608	3.126	0.052
43	100.0	0.03	0.881	0.699	3.12	0.056
44	100.0	0.033	0.838	0.715	3.169	0.068
45	100.0	0.032	0.863	0.695	3.034	0.054
46	100.0	0.032	0.829	0.654	2.709	0.05
47	100.0	0.031	0.799	0.679	2.639	0.053
48	100.0	0.031	0.789	0.721	3.473	0.039
49	100.0	0.033	0.748	0.769	3.624	0.046
50	100.0	0.033	0.905	0.719	3.358	0.049

Reproductive Toxicity Study to detect potential effects to anti-androgenic substances in Wistar Rats (Gavage)
28.Mar.2014 SIGR

RELATIVE WEIGHTS - INDIVIDUAL VALUES
PARENTAL FEMALES

Sacrifice F1
Sex F
Group 1

	Pituitary gland %	Spleen %	Thyroid glands %	Uterus %
M	0.006	0.204	0.008	0.295
SD	0.001	0.025	0.002	0.11
n	19	19	19	19
26	0.005	0.19	0.008	0.262
27	0.004	0.202	0.007	0.337
29	0.004	0.253	0.007	0.256
30	0.005	0.252	0.005	0.256
31	0.006	0.196	0.009	0.233
32	0.007	0.173	0.008	0.331
33	0.006	0.183	0.007	0.315
34	0.006	0.205	0.007	0.294
35	0.005	0.19	0.007	0.301
36	0.005	0.217	0.007	0.209
37	0.008	0.209	0.011	0.214
43	0.005	0.204	0.009	0.55
44	0.005	0.216	0.009	0.252
45	0.006	0.223	0.007	0.613
46	0.006	0.209	0.012	0.299
47	0.006	0.146	0.007	0.196
48	0.006	0.188	0.01	0.226
49	0.008	0.192	0.009	0.233
50	0.006	0.218	0.012	0.218

RELATIVE WEIGHTS - INDIVIDUAL VALUES
PARENTAL FEMALES

Sacrifice F1
Sex F
Group 2

	Term. body weight %	Adrenal glands %	Brain %	Kidneys %	Liver %	Ovaries %
M	100.0	0.032	0.814	0.706	3.177	0.054
SD		0.004	0.053	0.037	0.157	0.009
n	20	20	20	20	20	20
51	100.0	0.045	0.843	0.778	3.388	0.065
52	100.0	0.034	0.733	0.661	3.084	0.052
53	100.0	0.029	0.88	0.742	2.957	0.061
54	100.0	0.03	0.767	0.67	3.094	0.051
55	100.0	0.024	0.797	0.687	3.175	0.04
56	100.0	0.033	0.79	0.716	3.223	0.072
57	100.0	0.029	0.768	0.644	2.904	0.051
58	100.0	0.033	0.803	0.696	3.515	0.056
59	100.0	0.031	0.746	0.742	3.216	0.055
60	100.0	0.035	0.79	0.693	3.043	0.054
61	100.0	0.037	0.84	0.687	3.045	0.061
62	100.0	0.03	0.852	0.712	3.141	0.057
68	100.0	0.033	0.797	0.683	3.13	0.045
69	100.0	0.037	0.858	0.763	3.388	0.051
70	100.0	0.033	0.757	0.662	3.052	0.045
71	100.0	0.032	0.867	0.73	3.335	0.047
72	100.0	0.031	0.764	0.689	3.241	0.043
73	100.0	0.027	0.913	0.767	3.236	0.048
74	100.0	0.034	0.906	0.701	3.052	0.045
75	100.0	0.031	0.799	0.693	3.326	0.071

Reproductive Toxicity Study to detect potential effects to anti-androgenic substances in Wistar Rats (Gavage) 28.Mar.2014 SIGR

RELATIVE WEIGHTS - INDIVIDUAL VALUES
PARENTAL FEMALES

Sacrifice F1
Sex F
Group 2

	Pituitary gland %	Spleen %	Thyroid glands %	Uterus %
M	0.006	0.2	0.009	0.275
SD	0.001	0.034	0.002	0.092
n	20	20	20	20
51	0.005	0.192	0.011	0.319
52	0.006	0.196	0.009	0.284
53	0.005	0.184	0.008	0.433
54	0.005	0.185	0.007	0.198
55	0.005	0.202	0.009	0.215
56	0.006	0.17	0.007	0.186
57	0.005	0.159	0.006	0.207
58	0.005	0.244	0.007	0.263
59	0.005	0.196	0.008	0.415
60	0.006	0.138	0.011	0.239
61	0.006	0.263	0.011	0.31
62	0.006	0.215	0.007	0.255
68	0.005	0.237	0.006	0.212
69	0.006	0.237	0.008	0.242
70	0.006	0.232	0.008	0.232
71	0.006	0.195	0.009	0.545
72	0.005	0.236	0.007	0.201
73	0.005	0.183	0.009	0.202
74	0.005	0.167	0.01	0.272
75	0.008	0.158	0.011	0.278

Reproductive Toxicity Study to detect potential effects to anti-androgenic substances in Wistar Rats (Gavage)

RELATIVE WEIGHTS - INDIVIDUAL VALUES
PARENTAL FEMALES

Sacrifice F1
Sex F
Group 3

	Term. body weight %	Adrenal glands %	Brain %	Kidneys %	Liver %	Ovaries %
M	100.0	0.035	0.804	0.742	3.856	0.056
SD		0.005	0.047	0.047	0.27	0.012
n	19	19	19	19	19	19
77	100.0	0.032	0.771	0.634	3.507	0.042
78	100.0	0.046	0.778	0.7	4.257	0.064
79	100.0	0.034	0.819	0.691	3.316	0.047
80	100.0	0.031	0.833	0.726	3.619	0.053
81	100.0	0.034	0.809	0.726	3.423	0.049
82	100.0	0.043	0.797	0.76	3.81	0.058
83	100.0	0.034	0.774	0.707	3.851	0.047
84	100.0	0.031	0.898	0.744	3.76	0.08
85	100.0	0.029	0.838	0.748	3.754	0.05
86	100.0	0.035	0.75	0.812	4.007	0.062
87	100.0	0.04	0.729	0.741	4.089	0.074
93	100.0	0.031	0.778	0.799	4.34	0.05
94	100.0	0.039	0.8	0.743	3.921	0.047
95	100.0	0.029	0.744	0.7	3.936	0.044
96	100.0	0.037	0.826	0.708	3.993	0.051
97	100.0	0.038	0.83	0.813	4.135	0.068
98	100.0	0.032	0.89	0.801	3.669	0.054
99	100.0	0.042	0.852	0.777	3.926	0.078
100	100.0	0.032	0.76	0.76	3.955	0.049

Reproductive Toxicity Study to detect potential effects to anti-androgenic substances in Wistar Rats (Gavage)

RELATIVE WEIGHTS - INDIVIDUAL VALUES
PARENTAL FEMALES

Sacrifice F1
Sex F
Group 3

	Pituitary gland %	Spleen %	Thyroid glands %	Uterus %
M	0.005	0.205	0.009	0.29
SD	0.001	0.022	0.002	0.072
n	19	19	19	19
77	0.004	0.165	0.007	0.226
78	0.005	0.213	0.006	0.217
79	0.005	0.188	0.009	0.376
80	0.005	0.209	0.008	0.227
81	0.005	0.187	0.01	0.22
82	0.005	0.172	0.006	0.29
83	0.005	0.202	0.007	0.379
84	0.006	0.218	0.008	0.248
85	0.004	0.239	0.008	0.209
86	0.005	0.2	0.012	0.425
87	0.006	0.191	0.011	0.283
93	0.005	0.228	0.009	0.219
94	0.005	0.216	0.008	0.257
95	0.006	0.175	0.011	0.374
96	0.006	0.24	0.011	0.219
97	0.005	0.232	0.01	0.367
98	0.005	0.197	0.01	0.291
99	0.006	0.206	0.011	0.388
100	0.005	0.218	0.007	0.299

Reproductive Toxicity Study to detect potential effects to anti-androgenic substances in Wistar Rats (Gavage) 28.Mar.2014 SIGR

RELATIVE WEIGHTS - INDIVIDUAL VALUES
PARENTAL FEMALES

Sacrifice F1
Sex F
Group 4

	Term. body weight %	Adrenal glands %	Brain %	Kidneys %	Liver %	Ovaries %
M	100.0	0.032	0.799	0.711	3.11	0.048
SD		0.004	0.046	0.051	0.156	0.007
n	19	19	19	19	19	19
101	100.0	0.029	0.725	0.647	3.261	0.039
102	100.0	0.028	0.855	0.693	3.139	0.052
103	100.0	0.034	0.812	0.669	3.172	0.061
104	100.0	0.037	0.761	0.712	3.152	0.04
105	100.0	0.033	0.816	0.724	3.152	0.042
106	100.0	0.034	0.802	0.719	2.918	0.055
107	100.0	0.034	0.779	0.68	3.078	0.046
108	100.0	0.033	0.86	0.686	2.903	0.048
109	100.0	0.022	0.771	0.651	2.777	0.048
110	100.0	0.03	0.893	0.656	2.931	0.043
111	100.0	0.035	0.76	0.699	3.102	0.038
117	100.0	0.034	0.85	0.773	3.198	0.059
118	100.0	0.035	0.791	0.713	3.276	0.046
119	100.0	0.029	0.771	0.682	3.041	0.047
120	100.0	0.03	0.78	0.751	3.448	0.046
121	100.0	0.031	0.761	0.741	3.068	0.046
123	100.0	0.038	0.813	0.862	3.064	0.06
124	100.0	0.034	0.734	0.695	3.154	0.042
125	100.0	0.03	0.845	0.749	3.261	0.05

Reproductive Toxicity Study to detect potential effects to anti-androgenic substances in Wistar Rats (Gavage) 28.Mar.2014 SIGR

RELATIVE WEIGHTS - INDIVIDUAL VALUES
PARENTAL FEMALES

Sacrifice F1
Sex F
Group 4

	Pituitary gland %	Spleen %	Thyroid glands %	Uterus %
M	0.005	0.199	0.008	0.283
SD	0.001	0.025	0.002	0.11
n	19	19	19	19
101	0.004	0.179	0.005	0.208
102	0.004	0.2	0.008	0.374
103	0.005	0.181	0.007	0.198
104	0.005	0.202	0.009	0.222
105	0.005	0.221	0.008	0.495
106	0.005	0.179	0.007	0.324
107	0.005	0.168	0.006	0.201
108	0.005	0.225	0.009	0.215
109	0.005	0.15	0.007	0.192
110	0.003	0.232	0.007	0.251
111	0.005	0.215	0.007	0.507
117	0.004	0.217	0.008	0.271
118	0.006	0.199	0.01	0.212
119	0.005	0.179	0.011	0.195
120	0.004	0.25	0.008	0.428
121	0.004	0.212	0.009	0.439
123	0.006	0.177	0.01	0.252
124	0.005	0.201	0.009	0.197
125	0.005	0.202	0.01	0.198

Reproductive Toxicity Study to detect potential effects to anti-androgenic substances in Wistar Rats (Gavage)

28.Mar.2014 SIGR

ABSOLUTE WEIGHTS - INDIVIDUAL VALUES
SUBSET 1 (POSTNATAL DAY 21)

Sacrifice R1
Sex M
Group 0

	Term. body weight g	Adrenal glands mg	Brain g	Cauda epididymis mg	Epididy- mides mg	Kidneys g
M	44.77	12.5	1.449	10.0	29.0	0.462
SD	5.531	4.17	0.091	3.916	5.981	0.078
n	10	10	10	10	10	10
301	48.5	9.0	1.452	8.0	23.0	0.511
302	44.2	7.0	1.445	8.0	23.0	0.427
303	43.5	12.0	1.487	11.0	29.0	0.46
304	55.5	20.0	1.551	7.0	27.0	0.637
305	46.6	12.0	1.493	11.0	39.0	0.478
306	35.7	9.0	1.415	8.0	21.0	0.384
307	48.7	13.0	1.458	7.0	30.0	0.51
308	43.6	11.0	1.489	7.0	33.0	0.422
309	42.8	19.0	1.486	19.0	37.0	0.427
310	38.6	13.0	1.211	14.0	28.0	0.368

Reproductive Toxicity Study to detect potential effects to anti-androgenic substances in Wistar Rats (Gavage)

28.Mar.2014 SIGR

ABSOLUTE WEIGHTS - INDIVIDUAL VALUES
SUBSET 1 (POSTNATAL DAY 21)

Sacrifice R1
Sex M
Group 0

	Liver g	Muscles bulb + l.a mg	Pituitary gland mg	Prostate mg	Prostate vent.fresh mg	Seminal vesicle mg
M	1.584	35.45	1.2	48.51	24.4	9.27
SD	0.251	8.759	0.368	5.773	3.25	1.989
n	10	10	10	10	10	10
301	1.774	27.4	1.1	51.2	30.9	9.4
302	1.534	32.6	0.5	46.6	22.8	10.1
303	1.58	29.9	0.9	42.0	23.8	8.5
304	1.983	32.0	1.3	53.2	24.5	8.9
305	1.56	27.0	1.6	46.7	23.4	7.7
306	1.131	38.2	1.3	59.1	25.2	10.0
307	1.859	53.3	1.5	52.7	28.0	14.3
308	1.49	28.6	0.8	44.8	19.7	7.4
309	1.619	46.2	1.6	49.1	24.9	8.0
310	1.307	39.3	1.4	39.7	20.8	8.4

ABSOLUTE WEIGHTS - INDIVIDUAL VALUES
SUBSET 1 (POSTNATAL DAY 21)

Sacrifice R1
Sex M
Group 0

	Spleen g	Testes mg	Thyroid glands mg
M	0.173	237.8	4.85
SD	0.048	27.868	2.058
n	10	10	10
301	0.134	233.0	3.1
302	0.179	221.0	2.2
303	0.149	229.0	3.2
304	0.28	286.0	4.3
305	0.221	264.0	4.8
306	0.147	193.0	4.3
307	0.153	267.0	9.1
308	0.178	240.0	5.6
309	0.171	234.0	7.3
310	0.113	211.0	4.6

Reproductive Toxicity Study to detect potential effects to anti-androgenic substances in Wistar Rats (Gavage) 28.Mar.2014 SIGR

ABSOLUTE WEIGHTS - INDIVIDUAL VALUES
SUBSET 1 (POSTNATAL DAY 21)

Sacrifice R1
Sex M
Group 1

	Term. body weight g	Adrenal glands mg	Brain g	Cauda epididymis mg	Epididy- mides mg	Kidneys g
M	44.64	12.0	1.467	12.7	31.6	0.423
SD	5.495	4.967	0.05	5.293	8.235	0.124
n	10	10	10	10	10	10
311	47.8	10.0	1.509	12.0	33.0	0.109
312	48.6	14.0	1.496	14.0	29.0	0.484
313	40.5	9.0	1.449	15.0	30.0	0.445
314	34.5	8.0	1.412	7.0	22.0	0.332
315	48.5	13.0	1.518	11.0	31.0	0.508
316	39.1	6.0	1.37	9.0	18.0	0.418
317	52.0	12.0	1.452	7.0	34.0	0.533
318	47.2	10.0	1.464	10.0	31.0	0.47
319	47.2	24.0	1.53	18.0	42.0	0.489
320	41.0	14.0	1.469	24.0	46.0	0.445

ABSOLUTE WEIGHTS - INDIVIDUAL VALUES
SUBSET 1 (POSTNATAL DAY 21)

Sacrifice R1
Sex M
Group 1

	Liver g	Muscles bulb + l.a mg	Pituitary gland mg	Prostate mg	Prostate vent.fresh mg	Seminal vesicle mg
M	1.608	32.61	1.14	47.09	24.36	9.85
SD	0.269	7.758	0.479	8.504	3.59	1.531
n	10	10	10	10	10	10
311	1.852	30.7	1.0	42.3	26.1	10.7
312	1.882	29.6	1.1	45.4	20.4	9.0
313	1.448	23.5	0.6	39.2	25.2	8.6
314	1.064	26.2	0.4	37.9	17.3	8.3
315	1.711	31.9	1.4	47.9	23.5	9.3
316	1.396	27.0	1.1	48.0	25.3	9.8
317	1.946	37.7	1.3	68.1	28.4	12.7
318	1.675	34.2	1.5	43.2	22.6	7.9
319	1.641	34.2	0.9	47.3	29.3	11.5
320	1.463	51.1	2.1	51.6	25.5	10.7

ABSOLUTE WEIGHTS - INDIVIDUAL VALUES
SUBSET 1 (POSTNATAL DAY 21)

Sacrifice R1
Sex M
Group 1

	Spleen g	Testes mg	Thyroid glands mg
M	0.174	245.0	5.32
SD	0.047	38.404	1.831
n	10	10	10
311	0.172	249.0	3.8
312	0.209	253.0	4.2
313	0.13	247.0	5.6
314	0.096	171.0	3.5
315	0.173	243.0	4.9
316	0.128	209.0	6.9
317	0.251	320.0	9.7
318	0.221	253.0	5.0
319	0.182	270.0	4.2
320	0.174	235.0	5.4

Reproductive Toxicity Study to detect potential effects to anti-androgenic substances in Wistar Rats (Gavage) 28.Mar.2014 SIGR

ABSOLUTE WEIGHTS - INDIVIDUAL VALUES
SUBSET 1 (POSTNATAL DAY 21)

Sacrifice R1
Sex M
Group 2

	Term. body weight g	Adrenal glands mg	Brain g	Cauda epididymis mg	Epididy-mides mg	Kidneys g
M	44.33	13.6	1.484	9.4	29.4	0.461
SD	5.091	4.402	0.098	3.026	5.621	0.059
n	10	10	10	10	10	10
321	45.3	12.0	1.445	10.0	27.0	0.454
322	48.7	16.0	1.561	9.0	31.0	0.516
323	45.2	24.0	1.538	16.0	38.0	0.499
324	34.5	8.0	1.264	8.0	22.0	0.354
325	37.7	13.0	1.47	6.0	24.0	0.385
326	49.9	9.0	1.577	8.0	32.0	0.516
327	50.5	13.0	1.593	11.0	36.0	0.535
328	45.0	14.0	1.502	12.0	33.0	0.481
329	43.3	15.0	1.486	6.0	29.0	0.437
330	43.2	12.0	1.405	8.0	22.0	0.436

Reproductive Toxicity Study to detect potential effects to anti-androgenic substances in Wistar Rats (Gavage)
28.Mar.2014 SIGR

ABSOLUTE WEIGHTS - INDIVIDUAL VALUES
SUBSET 1 (POSTNATAL DAY 21)

Sacrifice R1
Sex M
Group 2

	Liver g	Muscles bulb + l.a mg	Pituitary gland mg	Prostate mg	Prostate vent.fresh mg	Seminal vesicle mg
M	1.555	33.76	0.82	45.24	24.03	9.17
SD	0.244	7.186	0.478	6.754	6.009	1.532
n	10	10	10	10	10	10
321	1.571	23.5	0.9	42.4	27.3	10.2
322	1.738	32.4	0.5	49.8	27.9	10.1
323	1.624	32.8	0.4	49.6	25.8	11.8
324	1.141	30.3	0.3	36.5	16.2	11.1
325	1.302	26.6	0.6	37.0	14.8	7.6
326	1.866	42.7	0.6	56.0	35.6	8.2
327	1.918	37.4	1.2	49.9	25.4	8.5
328	1.476	26.9	1.1	41.6	23.3	7.7
329	1.516	43.0	0.7	50.6	23.6	8.9
330	1.398	42.0	1.9	39.0	20.4	7.6

ABSOLUTE WEIGHTS - INDIVIDUAL VALUES
SUBSET 1 (POSTNATAL DAY 21)

Sacrifice R1
Sex M
Group 2

	Spleen g	Testes mg	Thyroid glands mg
M	0.162	234.1	6.15
SD	0.039	51.682	2.507
n	10	10	10
321	0.176	223.0	4.5
322	0.189	295.0	3.4
323	0.185	263.0	3.4
324	0.094	152.0	3.3
325	0.113	195.0	5.9
326	0.17	301.0	10.0
327	0.23	296.0	8.4
328	0.169	211.0	7.3
329	0.151	214.0	6.1
330	0.144	191.0	9.2

Reproductive Toxicity Study to detect potential effects to anti-androgenic substances in Wistar Rats (Gavage) 28.Mar.2014 SIGR

ABSOLUTE WEIGHTS - INDIVIDUAL VALUES
SUBSET 1 (POSTNATAL DAY 21)

Sacrifice R1
Sex M
Group 3

	Term. body weight g	Adrenal glands mg	Brain g	Cauda epididymis mg	Epididy-mides mg	Kidneys g
M	47.37	13.1	1.497	9.2	26.6	0.49
SD	3.609	3.213	0.059	2.53	2.633	0.05
n	10	10	10	10	10	10
331	48.8	11.0	1.458	8.0	25.0	0.482
332	54.9	9.0	1.49	7.0	26.0	0.573
333	42.0	11.0	1.443	6.0	23.0	0.392
334	46.9	10.0	1.546	6.0	28.0	0.471
335	47.2	12.0	1.548	9.0	31.0	0.451
336	48.3	12.0	1.385	9.0	24.0	0.518
337	48.2	17.0	1.505	13.0	29.0	0.522
338	42.2	17.0	1.481	12.0	27.0	0.459
339	46.9	14.0	1.518	10.0	24.0	0.511
340	48.3	18.0	1.591	12.0	29.0	0.517

ABSOLUTE WEIGHTS - INDIVIDUAL VALUES
SUBSET 1 (POSTNATAL DAY 21)

Sacrifice R1
Sex M
Group 3

	Liver g	Muscles bulb + l.a mg	Pituitary gland mg	Prostate mg	Prostate vent.fresh mg	Seminal vesicle mg
M	1.705	35.5	1.79	42.35	19.21	8.15
SD	0.136	9.647	0.939	8.452	5.633	1.756
n	10	10	10	10	10	10
331	1.778	26.2	4.2	59.9	24.1	10.7
332	1.973	30.2	1.8	38.0	17.3	5.7
333	1.574	23.4	1.3	37.0	19.1	9.6
334	1.623	47.6	1.7	36.0	22.8	6.6
335	1.655	30.4	1.4	37.8	16.5	7.6
336	1.735	50.9	1.7	35.3	18.3	8.4
337	1.713	41.7	1.9	55.0	31.1	10.6
338	1.497	25.9	1.0	42.3	18.1	7.8
339	1.665	41.2	0.8	38.5	13.3	6.1
340	1.834	37.5	2.1	43.7	11.5	8.4

ABSOLUTE WEIGHTS - INDIVIDUAL VALUES
SUBSET 1 (POSTNATAL DAY 21)

Sacrifice R1
Sex M
Group 3

	Spleen g	Testes mg	Thyroid glands mg
M	0.178	257.9	7.46
SD	0.038	34.594	2.191
n	10	10	10
331	0.208	270.0	5.5
332	0.236	321.0	5.0
333	0.139	225.0	8.7
334	0.161	250.0	6.0
335	0.161	234.0	10.2
336	0.182	202.0	8.7
337	0.224	295.0	3.8
338	0.158	258.0	8.2
339	0.117	248.0	9.6
340	0.191	276.0	8.9

ABSOLUTE WEIGHTS - INDIVIDUAL VALUES
SUBSET 1 (POSTNATAL DAY 21)

Sacrifice R1
Sex M
Group 4

	Term. body weight g	Adrenal glands mg	Brain g	Cauda epididymis mg	Epididy- mides mg	Kidneys g
M	46.83	12.6	1.492	10.4	30.3	0.489
SD	5.576	2.413	0.048	2.011	4.029	0.064
n	10	10	10	10	10	10
341	41.8	9.0	1.532	10.0	30.0	0.422
342	38.5	10.0	1.382	11.0	27.0	0.394
343	44.6	13.0	1.488	6.0	27.0	0.485
344	49.3	17.0	1.538	9.0	32.0	0.494
345	52.8	14.0	1.546	13.0	38.0	0.567
346	56.3	13.0	1.483	12.0	30.0	0.583
347	48.4	13.0	1.455	9.0	32.0	0.517
348	44.5	15.0	1.496	12.0	32.0	0.448
349	50.5	11.0	1.498	11.0	32.0	0.547
350	41.6	11.0	1.502	11.0	23.0	0.434

Reproductive Toxicity Study to detect potential effects to anti-androgenic substances in Wistar Rats (Gavage) 28.Mar.2014 SIGR

ABSOLUTE WEIGHTS - INDIVIDUAL VALUES
SUBSET 1 (POSTNATAL DAY 21)

Sacrifice R1
Sex M
Group 4

	Liver g	Muscles bulb + l.a mg	Pituitary gland mg	Prostate mg	Prostate vent.fresh mg	Seminal vesicle mg
M	1.698	36.95	1.35	51.64	26.21	9.54
SD	0.255	6.688	0.46	4.062	3.529	1.936
n	10	10	10	10	10	10
341	1.474	34.7	1.0	43.2	19.1	5.7
342	1.361	27.7	1.3	51.3	25.8	7.5
343	1.584	36.9	1.5	52.8	26.3	9.8
344	1.77	35.2	0.5	50.7	25.1	10.7
345	1.914	37.6	1.3	58.8	28.5	11.4
346	2.223	43.5	1.6	49.9	24.9	12.1
347	1.726	33.8	1.3	49.6	26.5	10.5
348	1.628	29.2	1.0	52.1	23.8	8.2
349	1.836	40.4	2.0	52.4	31.5	10.3
350	1.462	50.5	2.0	55.6	30.6	9.2

ABSOLUTE WEIGHTS - INDIVIDUAL VALUES
SUBSET 1 (POSTNATAL DAY 21)

Sacrifice R1
Sex M
Group 4

	Spleen g	Testes mg	Thyroid glands mg
M	0.18	258.0	6.92
SD	0.037	36.518	3.293
n	10	10	10
341	0.137	229.0	7.3
342	0.161	201.0	3.8
343	0.162	235.0	6.1
344	0.196	262.0	5.4
345	0.24	276.0	5.8
346	0.216	297.0	6.2
347	0.211	267.0	8.9
348	0.136	237.0	15.4
349	0.195	328.0	4.5
350	0.144	248.0	5.8

Reproductive Toxicity Study to detect potential effects to anti-androgenic substances in Wistar Rats (Gavage) 28.Mar.2014 SIGR

ABSOLUTE WEIGHTS - INDIVIDUAL VALUES
SUBSET 1 (POSTNATAL DAY 21)

Sacrifice R1
Sex F
Group 0

	Term. body weight g	Adrenal glands mg	Brain g	Kidneys g	Liver g	Ovaries mg
M	43.37	15.7	1.438	0.472	1.538	14.0
SD	2.4	3.093	0.035	0.038	0.124	1.491
n	10	10	10	10	10	10
401	47.8	18.0	1.463	0.561	1.761	16.0
402	42.9	19.0	1.395	0.451	1.545	12.0
403	43.9	12.0	1.477	0.458	1.575	14.0
404	46.6	21.0	1.5	0.51	1.609	15.0
405	40.4	12.0	1.422	0.436	1.315	16.0
406	41.4	15.0	1.405	0.454	1.402	14.0
407	41.9	14.0	1.447	0.434	1.478	12.0
408	42.1	18.0	1.406	0.463	1.518	13.0
409	41.8	14.0	1.415	0.478	1.544	15.0
410	44.9	14.0	1.453	0.473	1.635	13.0

Reproductive Toxicity Study to detect potential effects to anti-androgenic substances in Wistar Rats (Gavage) 28.Mar.2014 SIGR

ABSOLUTE WEIGHTS - INDIVIDUAL VALUES
SUBSET 1 (POSTNATAL DAY 21)

Sacrifice R1
Sex F
Group 0

	Pituitary gland mg	Spleen g	Thyroid glands mg	Uterus mg
M	1.44	0.175	6.1	36.5
SD	0.677	0.032	1.493	4.72
n	10	10	10	10
401	0.6	0.239	4.7	44.0
402	0.1	0.147	4.1	30.0
403	1.2	0.137	4.4	36.0
404	1.3	0.2	6.2	40.0
405	1.3	0.165	7.7	33.0
406	1.8	0.17	6.8	35.0
407	2.1	0.177	6.8	40.0
408	1.9	0.146	8.8	36.0
409	2.0	0.162	6.0	30.0
410	2.1	0.206	5.5	41.0

ABSOLUTE WEIGHTS - INDIVIDUAL VALUES
SUBSET 1 (POSTNATAL DAY 21)

Sacrifice R1
Sex F
Group 1

	Term. body weight g	Adrenal glands mg	Brain g	Kidneys g	Liver g	Ovaries mg
M	44.46	13.2	1.429	0.495	1.593	13.6
SD	5.213	2.7	0.069	0.076	0.222	1.955
n	10	10	10	10	10	10
411	41.1	13.0	1.448	0.429	1.458	12.0
412	40.5	10.0	1.347	0.467	1.272	16.0
413	48.8	14.0	1.501	0.51	1.727	11.0
414	49.4	10.0	1.444	0.543	1.634	15.0
415	52.9	17.0	1.479	0.629	1.915	16.0
416	35.8	13.0	1.301	0.367	1.287	15.0
417	41.9	14.0	1.453	0.467	1.661	15.0
418	47.8	18.0	1.508	0.573	1.851	12.0
419	45.1	12.0	1.442	0.526	1.69	12.0
420	41.3	11.0	1.365	0.442	1.436	12.0

ABSOLUTE WEIGHTS - INDIVIDUAL VALUES
SUBSET 1 (POSTNATAL DAY 21)

Sacrifice R1
Sex F
Group 1

	Pituitary gland mg	Spleen g	Thyroid glands mg	Uterus mg
M	1.67	0.171	5.32	40.0
SD	0.729	0.038	2.926	5.715
n	10	10	10	10
411	1.6	0.167	1.8	31.0
412	0.5	0.161	5.2	33.0
413	0.7	0.18	1.0	46.0
414	2.2	0.247	4.7	45.0
415	2.8	0.186	11.5	40.0
416	1.1	0.118	6.7	44.0
417	1.7	0.121	5.9	39.0
418	2.1	0.198	4.7	47.0
419	2.3	0.179	7.3	41.0
420	1.7	0.151	4.4	34.0

Reproductive Toxicity Study to detect potential effects to anti-androgenic substances in Wistar Rats (Gavage) 28.Mar.2014 SIGR

ABSOLUTE WEIGHTS - INDIVIDUAL VALUES
SUBSET 1 (POSTNATAL DAY 21)

Sacrifice R1
Sex F
Group 2

	Term. body weight g	Adrenal glands mg	Brain g	Kidneys g	Liver g	Ovaries mg
M	43.53	13.0	1.427	0.484	1.591	13.5
SD	3.891	3.055	0.041	0.048	0.19	4.673
n	10	10	10	10	10	10
421	40.4	11.0	1.431	0.424	1.313	10.0
422	38.8	12.0	1.39	0.432	1.388	10.0
423	41.9	10.0	1.364	0.492	1.428	5.0
424	50.8	18.0	1.468	0.586	1.908	17.0
425	46.7	16.0	1.44	0.473	1.711	12.0
426	45.0	10.0	1.504	0.52	1.54	13.0
427	44.2	16.0	1.417	0.469	1.723	20.0
428	47.1	14.0	1.451	0.491	1.629	12.0
429	40.9	9.0	1.398	0.512	1.774	18.0
430	39.5	14.0	1.406	0.443	1.491	18.0

ABSOLUTE WEIGHTS - INDIVIDUAL VALUES
SUBSET 1 (POSTNATAL DAY 21)

Sacrifice R1
Sex F
Group 2

	Pituitary gland mg	Spleen g	Thyroid glands mg	Uterus mg
M	1.41	0.171	6.37	40.4
SD	0.367	0.029	3.355	3.471
n	10	10	10	10
421	1.7	0.139	2.2	39.0
422	0.9	0.137	1.8	36.0
423	1.2	0.176	4.9	42.0
424	1.3	0.205	6.8	39.0
425	1.3	0.179	4.3	37.0
426	1.2	0.198	13.2	41.0
427	1.7	0.185	8.5	43.0
428	1.8	0.208	8.2	38.0
429	2.0	0.137	6.1	41.0
430	1.0	0.141	7.7	48.0

Reproductive Toxicity Study to detect potential effects to anti-androgenic substances in Wistar Rats (Gavage) 28.Mar.2014 SIGR

ABSOLUTE WEIGHTS - INDIVIDUAL VALUES
SUBSET 1 (POSTNATAL DAY 21)

Sacrifice R1
Sex F
Group 3

	Term. body weight g	Adrenal glands mg	Brain g	Kidneys g	Liver g	Ovaries mg
M	46.66	16.9	1.465	0.514	1.717	15.9
SD	3.84	3.665	0.054	0.063	0.154	4.04
n	10	10	10	10	10	10
431	48.4	15.0	1.375	0.546	1.641	9.0
432	44.8	24.0	1.43	0.501	1.564	20.0
433	54.5	20.0	1.511	0.666	1.944	15.0
434	41.6	12.0	1.401	0.444	1.588	15.0
435	44.7	16.0	1.449	0.472	1.728	16.0
436	48.0	17.0	1.45	0.473	1.886	11.0
437	46.5	15.0	1.509	0.509	1.735	15.0
438	41.8	15.0	1.472	0.473	1.538	18.0
439	46.5	14.0	1.522	0.509	1.616	17.0
440	49.8	21.0	1.533	0.543	1.93	23.0

ABSOLUTE WEIGHTS - INDIVIDUAL VALUES
SUBSET 1 (POSTNATAL DAY 21)

Sacrifice R1
Sex F
Group 3

	Pituitary gland mg	Spleen g	Thyroid glands mg	Uterus mg
M	1.68	0.204	8.8	44.0
SD	0.485	0.052	2.878	4.163
n	10	10	10	10
431	0.9	0.249	9.2	46.0
432	1.5	0.197	6.7	54.0
433	2.5	0.324	11.8	44.0
434	1.9	0.187	9.6	46.0
435	1.9	0.173	7.7	42.0
436	1.9	0.169	4.2	41.0
437	1.0	0.228	14.6	39.0
438	1.4	0.154	6.8	43.0
439	1.8	0.155	8.3	44.0
440	2.0	0.202	9.1	41.0

ABSOLUTE WEIGHTS - INDIVIDUAL VALUES
SUBSET 1 (POSTNATAL DAY 21)

Sacrifice R1
Sex F
Group 4

	Term. body weight g	Adrenal glands mg	Brain g	Kidneys g	Liver g	Ovaries mg
M	45.27	15.4	1.479	0.51	1.651	15.7
SD	6.063	3.836	0.039	0.065	0.224	3.889
n	10	10	10	10	10	10
441	42.5	13.0	1.475	0.457	1.573	7.0
442	44.2	14.0	1.522	0.452	1.692	18.0
443	44.0	14.0	1.421	0.503	1.658	16.0
444	38.1	13.0	1.448	0.457	1.352	16.0
445	45.4	17.0	1.54	0.489	1.53	17.0
446	43.1	11.0	1.473	0.476	1.501	16.0
447	53.7	17.0	1.526	0.593	1.846	19.0
448	58.1	25.0	1.473	0.647	2.153	21.0
449	43.3	15.0	1.444	0.541	1.71	12.0
450	40.3	15.0	1.463	0.483	1.492	15.0

ABSOLUTE WEIGHTS - INDIVIDUAL VALUES
SUBSET 1 (POSTNATAL DAY 21)

Sacrifice R1
Sex F
Group 4

	Pituitary gland mg	Spleen g	Thyroid glands mg	Uterus mg
M	1.85	0.192	7.3	39.5
SD	0.443	0.067	2.785	8.436
n	10	10	10	10
441	1.2	0.102	13.4	32.0
442	1.4	0.181	5.9	37.0
443	1.6	0.238	4.2	35.0
444	1.5	0.145	5.9	35.0
445	2.2	0.191	8.6	46.0
446	2.1	0.172	9.0	31.0
447	1.8	0.207	8.3	35.0
448	2.6	0.354	3.9	59.0
449	1.8	0.162	6.0	44.0
450	2.3	0.171	7.8	41.0

Reproductive Toxicity Study to detect potential effects to anti-androgenic substances in Wistar Rats (Gavage) 28.Mar.2014 SIGR

RELATIVE WEIGHTS - INDIVIDUAL VALUES
SUBSET 1 (POSTNATAL DAY 21)

Sacrifice R1
Sex M
Group 0

	Term. body weight %	Adrenal glands %	Brain %	Cauda epididymis %	Epididy-mides %	Kidneys %
M	100.0	0.028	3.266	0.023	0.065	1.029
SD		0.008	0.328	0.01	0.014	0.06
n	10	10	10	10	10	10
301	100.0	0.019	2.994	0.016	0.047	1.054
302	100.0	0.016	3.269	0.018	0.052	0.966
303	100.0	0.028	3.418	0.025	0.067	1.057
304	100.0	0.036	2.795	0.013	0.049	1.148
305	100.0	0.026	3.204	0.024	0.084	1.026
306	100.0	0.025	3.964	0.022	0.059	1.076
307	100.0	0.027	2.994	0.014	0.062	1.047
308	100.0	0.025	3.415	0.016	0.076	0.968
309	100.0	0.044	3.472	0.044	0.086	0.998
310	100.0	0.034	3.137	0.036	0.073	0.953

RELATIVE WEIGHTS - INDIVIDUAL VALUES
SUBSET 1 (POSTNATAL DAY 21)

Sacrifice R1
Sex M
Group 0

	Liver %	Muscles bulb + l.a %	Pituitary gland %	Prostate %	Prostate vent.fresh %	Seminal vesicle %
M	3.525	0.081	0.003	0.11	0.055	0.021
SD	0.204	0.023	0.001	0.02	0.008	0.005
n	10	10	10	10	10	10
301	3.658	0.056	0.002	0.106	0.064	0.019
302	3.471	0.074	0.001	0.105	0.052	0.023
303	3.632	0.069	0.002	0.097	0.055	0.02
304	3.573	0.058	0.002	0.096	0.044	0.016
305	3.348	0.058	0.003	0.1	0.05	0.017
306	3.168	0.107	0.004	0.166	0.071	0.028
307	3.817	0.109	0.003	0.108	0.057	0.029
308	3.417	0.066	0.002	0.103	0.045	0.017
309	3.783	0.108	0.004	0.115	0.058	0.019
310	3.386	0.102	0.004	0.103	0.054	0.022

RELATIVE WEIGHTS - INDIVIDUAL VALUES
SUBSET 1 (POSTNATAL DAY 21)

Sacrifice R1
Sex M
Group 0

	Spleen %	Testes %	Thyroid glands %
M	0.383	0.532	0.011
SD	0.075	0.026	0.005
n	10	10	10
301	0.276	0.48	0.006
302	0.405	0.5	0.005
303	0.343	0.526	0.007
304	0.505	0.515	0.008
305	0.474	0.567	0.01
306	0.412	0.541	0.012
307	0.314	0.548	0.019
308	0.408	0.55	0.013
309	0.4	0.547	0.017
310	0.293	0.547	0.012

RELATIVE WEIGHTS - INDIVIDUAL VALUES
SUBSET 1 (POSTNATAL DAY 21)

Sacrifice R1
Sex M
Group 1

	Term. body weight %	Adrenal glands %	Brain %	Cauda epididymis %	Epididy- mides %	Kidneys %
M	100.0	0.027	3.326	0.029	0.071	0.954
SD		0.01	0.368	0.013	0.018	0.259
n	10	10	10	10	10	10
311	100.0	0.021	3.157	0.025	0.069	0.228
312	100.0	0.029	3.078	0.029	0.06	0.996
313	100.0	0.022	3.578	0.037	0.074	1.099
314	100.0	0.023	4.093	0.02	0.064	0.962
315	100.0	0.027	3.13	0.023	0.064	1.047
316	100.0	0.015	3.504	0.023	0.046	1.069
317	100.0	0.023	2.792	0.013	0.065	1.025
318	100.0	0.021	3.102	0.021	0.066	0.996
319	100.0	0.051	3.242	0.038	0.089	1.036
320	100.0	0.034	3.583	0.059	0.112	1.085

Reproductive Toxicity Study to detect potential effects to anti-androgenic substances in Wistar Rats (Gavage) 28.Mar.2014 SIGR

RELATIVE WEIGHTS - INDIVIDUAL VALUES
SUBSET 1 (POSTNATAL DAY 21)

Sacrifice R1
Sex M
Group 1

	Liver %	Muscles bulb + l.a %	Pituitary gland %	Prostate %	Prostate vent.fresh %	Seminal vesicle %
M	3.584	0.074	0.003	0.106	0.055	0.022
SD	0.226	0.019	0.001	0.015	0.008	0.003
n	10	10	10	10	10	10
311	3.874	0.064	0.002	0.088	0.055	0.022
312	3.872	0.061	0.002	0.093	0.042	0.019
313	3.575	0.058	0.001	0.097	0.062	0.021
314	3.084	0.076	0.001	0.11	0.05	0.024
315	3.528	0.066	0.003	0.099	0.048	0.019
316	3.57	0.069	0.003	0.123	0.065	0.025
317	3.742	0.073	0.003	0.131	0.055	0.024
318	3.549	0.072	0.003	0.092	0.048	0.017
319	3.477	0.072	0.002	0.1	0.062	0.024
320	3.568	0.125	0.005	0.126	0.062	0.026

RELATIVE WEIGHTS - INDIVIDUAL VALUES
SUBSET 1 (POSTNATAL DAY 21)

Sacrifice R1
Sex M
Group 1

	Spleen %	Testes %	Thyroid glands %
M	0.383	0.548	0.012
SD	0.067	0.043	0.004
n	10	10	10
311	0.36	0.521	0.008
312	0.43	0.521	0.009
313	0.321	0.61	0.014
314	0.278	0.496	0.01
315	0.357	0.501	0.01
316	0.327	0.535	0.018
317	0.483	0.615	0.019
318	0.468	0.536	0.011
319	0.386	0.572	0.009
320	0.424	0.573	0.013

RELATIVE WEIGHTS - INDIVIDUAL VALUES
SUBSET 1 (POSTNATAL DAY 21)

Sacrifice R1
Sex M
Group 2

	Term. body weight %	Adrenal glands %	Brain %	Cauda epididymis %	Epididy- mides %	Kidneys %
M	100.0	0.031	3.37	0.021	0.066	1.039
SD		0.009	0.244	0.006	0.009	0.033
n	10	10	10	10	10	10
321	100.0	0.026	3.19	0.022	0.06	1.002
322	100.0	0.033	3.205	0.018	0.064	1.06
323	100.0	0.053	3.403	0.035	0.084	1.104
324	100.0	0.023	3.664	0.023	0.064	1.026
325	100.0	0.034	3.899	0.016	0.064	1.021
326	100.0	0.018	3.16	0.016	0.064	1.034
327	100.0	0.026	3.154	0.022	0.071	1.059
328	100.0	0.031	3.338	0.027	0.073	1.069
329	100.0	0.035	3.432	0.014	0.067	1.009
330	100.0	0.028	3.252	0.019	0.051	1.009

RELATIVE WEIGHTS - INDIVIDUAL VALUES
SUBSET 1 (POSTNATAL DAY 21)

Sacrifice R1
Sex M
Group 2

	Liver<br>%	Muscles bulb + l.a<br>%	Pituitary gland<br>%	Prostate<br>%	Prostate vent.fresh<br>%	Seminal vesicle<br>%
M	3.495	0.077	0.002	0.102	0.054	0.021
SD	0.188	0.016	0.001	0.009	0.009	0.005
n	10	10	10	10	10	10
321	3.468	0.052	0.002	0.094	0.06	0.023
322	3.569	0.067	0.001	0.102	0.057	0.021
323	3.593	0.073	0.001	0.11	0.057	0.026
324	3.307	0.088	0.001	0.106	0.047	0.032
325	3.454	0.071	0.002	0.098	0.039	0.02
326	3.739	0.086	0.001	0.112	0.071	0.016
327	3.798	0.074	0.002	0.099	0.05	0.017
328	3.28	0.06	0.002	0.092	0.052	0.017
329	3.501	0.099	0.002	0.117	0.055	0.021
330	3.236	0.097	0.004	0.09	0.047	0.018

RELATIVE WEIGHTS - INDIVIDUAL VALUES
SUBSET 1 (POSTNATAL DAY 21)

Sacrifice R1
Sex M
Group 2

	Spleen %	Testes %	Thyroid glands %
M	0.361	0.523	0.014
SD	0.054	0.066	0.005
n	10	10	10
321	0.389	0.492	0.01
322	0.388	0.606	0.007
323	0.409	0.582	0.008
324	0.272	0.441	0.01
325	0.3	0.517	0.016
326	0.341	0.603	0.02
327	0.455	0.586	0.017
328	0.376	0.469	0.016
329	0.349	0.494	0.014
330	0.333	0.442	0.021

Reproductive Toxicity Study to detect potential effects to anti-androgenic substances in Wistar Rats (Gavage)
28.Mar.2014 SIGR

RELATIVE WEIGHTS - INDIVIDUAL VALUES
SUBSET 1 (POSTNATAL DAY 21)

Sacrifice R1
Sex M
Group 3

	Term. body weight %	Adrenal glands %	Brain %	Cauda epididymis %	Epididy- mides %	Kidneys %
M	100.0	0.028	3.174	0.02	0.056	1.033
SD		0.008	0.251	0.006	0.006	0.058
n	10	10	10	10	10	10
331	100.0	0.023	2.988	0.016	0.051	0.988
332	100.0	0.016	2.714	0.013	0.047	1.044
333	100.0	0.026	3.436	0.014	0.055	0.933
334	100.0	0.021	3.296	0.013	0.06	1.004
335	100.0	0.025	3.28	0.019	0.066	0.956
336	100.0	0.025	2.867	0.019	0.05	1.072
337	100.0	0.035	3.122	0.027	0.06	1.083
338	100.0	0.04	3.509	0.028	0.064	1.088
339	100.0	0.03	3.237	0.021	0.051	1.09
340	100.0	0.037	3.294	0.025	0.06	1.07

RELATIVE WEIGHTS - INDIVIDUAL VALUES
SUBSET 1 (POSTNATAL DAY 21)

Sacrifice R1
Sex M
Group 3

	Liver %	Muscles bulb + l.a %	Pituitary gland %	Prostate %	Prostate vent.fresh %	Seminal vesicle %
M	3.599	0.075	0.004	0.09	0.041	0.017
SD	0.104	0.02	0.002	0.018	0.012	0.004
n	10	10	10	10	10	10
331	3.643	0.054	0.009	0.123	0.049	0.022
332	3.594	0.055	0.003	0.069	0.032	0.01
333	3.748	0.056	0.003	0.088	0.045	0.023
334	3.461	0.101	0.004	0.077	0.049	0.014
335	3.506	0.064	0.003	0.08	0.035	0.016
336	3.592	0.105	0.004	0.073	0.038	0.017
337	3.554	0.087	0.004	0.114	0.065	0.022
338	3.547	0.061	0.002	0.1	0.043	0.018
339	3.55	0.088	0.002	0.082	0.028	0.013
340	3.797	0.078	0.004	0.09	0.024	0.017

RELATIVE WEIGHTS - INDIVIDUAL VALUES
SUBSET 1 (POSTNATAL DAY 21)

Sacrifice R1
Sex M
Group 3

	Spleen %	Testes %	Thyroid glands %
M	0.373	0.544	0.016
SD	0.061	0.058	0.005
n	10	10	10
331	0.426	0.553	0.011
332	0.43	0.585	0.009
333	0.331	0.536	0.021
334	0.343	0.533	0.013
335	0.341	0.496	0.022
336	0.377	0.418	0.018
337	0.465	0.612	0.008
338	0.374	0.611	0.019
339	0.249	0.529	0.02
340	0.395	0.571	0.018

Reproductive Toxicity Study to detect potential effects to anti-androgenic substances in Wistar Rats (Gavage)

28.Mar.2014 SIGR

RELATIVE WEIGHTS - INDIVIDUAL VALUES
SUBSET 1 (POSTNATAL DAY 21)

Sacrifice R1
Sex M
Group 4

	Term. body weight %	Adrenal glands %	Brain %	Cauda epididymis %	Epididy-mides %	Kidneys %
M	100.0	0.027	3.222	0.022	0.065	1.043
SD		0.004	0.345	0.005	0.007	0.033
n	10	10	10	10	10	10
341	100.0	0.022	3.665	0.024	0.072	1.01
342	100.0	0.026	3.59	0.029	0.07	1.023
343	100.0	0.029	3.336	0.013	0.061	1.087
344	100.0	0.034	3.12	0.018	0.065	1.002
345	100.0	0.027	2.928	0.025	0.072	1.074
346	100.0	0.023	2.634	0.021	0.053	1.036
347	100.0	0.027	3.006	0.019	0.066	1.068
348	100.0	0.034	3.362	0.027	0.072	1.007
349	100.0	0.022	2.966	0.022	0.063	1.083
350	100.0	0.026	3.611	0.026	0.055	1.043

RELATIVE WEIGHTS - INDIVIDUAL VALUES
SUBSET 1 (POSTNATAL DAY 21)

Sacrifice R1
Sex M
Group 4

	Liver<br>%	Muscles<br>bulb + l.a<br>%	Pituitary<br>gland<br>%	Prostate<br>%	Prostate<br>vent.fresh<br>%	Seminal<br>vesicle<br>%
M	3.615	0.079	0.003	0.111	0.056	0.02
SD	0.127	0.016	0.001	0.014	0.009	0.003
n	10	10	10	10	10	10
341	3.526	0.083	0.002	0.103	0.046	0.014
342	3.535	0.072	0.003	0.133	0.067	0.019
343	3.552	0.083	0.003	0.118	0.059	0.022
344	3.59	0.071	0.001	0.103	0.051	0.022
345	3.625	0.071	0.002	0.111	0.054	0.022
346	3.948	0.077	0.003	0.089	0.044	0.021
347	3.566	0.07	0.003	0.102	0.055	0.022
348	3.658	0.066	0.002	0.117	0.053	0.018
349	3.636	0.08	0.004	0.104	0.062	0.02
350	3.514	0.121	0.005	0.134	0.074	0.022

RELATIVE WEIGHTS - INDIVIDUAL VALUES
SUBSET 1 (POSTNATAL DAY 21)

Sacrifice R1
Sex M
Group 4

	Spleen %	Testes %	Thyroid glands %
M	0.382	0.551	0.015
SD	0.047	0.041	0.008
n	10	10	10
341	0.328	0.548	0.017
342	0.418	0.522	0.01
343	0.363	0.527	0.014
344	0.398	0.531	0.011
345	0.455	0.523	0.011
346	0.384	0.528	0.011
347	0.436	0.552	0.018
348	0.306	0.533	0.035
349	0.386	0.65	0.009
350	0.346	0.596	0.014

Reproductive Toxicity Study to detect potential effects to anti-androgenic substances in Wistar Rats (Gavage) 28.Mar.2014 SIGR

RELATIVE WEIGHTS - INDIVIDUAL VALUES
SUBSET 1 (POSTNATAL DAY 21)

Sacrifice R1
Sex F
Group 0

	Term. body weight %	Adrenal glands %	Brain %	Kidneys %	Liver %	Ovaries %
M	100.0	0.036	3.322	1.087	3.544	0.032
SD		0.006	0.133	0.045	0.14	0.004
n	10	10	10	10	10	10
401	100.0	0.038	3.061	1.174	3.684	0.033
402	100.0	0.044	3.252	1.051	3.601	0.028
403	100.0	0.027	3.364	1.043	3.588	0.032
404	100.0	0.045	3.219	1.094	3.453	0.032
405	100.0	0.03	3.52	1.079	3.255	0.04
406	100.0	0.036	3.394	1.097	3.386	0.034
407	100.0	0.033	3.453	1.036	3.527	0.029
408	100.0	0.043	3.34	1.1	3.606	0.031
409	100.0	0.033	3.385	1.144	3.694	0.036
410	100.0	0.031	3.236	1.053	3.641	0.029

Reproductive Toxicity Study to detect potential effects to anti-androgenic substances in Wistar Rats (Gavage) 28.Mar.2014 SIGR

RELATIVE WEIGHTS - INDIVIDUAL VALUES
SUBSET 1 (POSTNATAL DAY 21)

Sacrifice R1
Sex F
Group 0

	Pituitary gland %	Spleen %	Thyroid glands %	Uterus %
M	0.003	0.402	0.014	0.084
SD	0.002	0.057	0.004	0.008
n	10	10	10	10
401	0.001	0.5	0.01	0.092
402	0.0	0.343	0.01	0.07
403	0.003	0.312	0.01	0.082
404	0.003	0.429	0.013	0.086
405	0.003	0.408	0.019	0.082
406	0.004	0.411	0.016	0.085
407	0.005	0.422	0.016	0.095
408	0.005	0.347	0.021	0.086
409	0.005	0.388	0.014	0.072
410	0.005	0.459	0.012	0.091

Reproductive Toxicity Study to detect potential effects to anti-androgenic substances in Wistar Rats (Gavage) 28.Mar.2014 SIGR

RELATIVE WEIGHTS - INDIVIDUAL VALUES
SUBSET 1 (POSTNATAL DAY 21)

Sacrifice R1
Sex F
Group 1

	Term. body weight %	Adrenal glands %	Brain %	Kidneys %	Liver %	Ovaries %
M	100.0	0.03	3.24	1.111	3.581	0.031
SD		0.005	0.265	0.064	0.246	0.006
n	10	10	10	10	10	10
411	100.0	0.032	3.523	1.044	3.547	0.029
412	100.0	0.025	3.326	1.153	3.141	0.04
413	100.0	0.029	3.076	1.045	3.539	0.023
414	100.0	0.02	2.923	1.099	3.308	0.03
415	100.0	0.032	2.796	1.189	3.62	0.03
416	100.0	0.036	3.634	1.025	3.595	0.042
417	100.0	0.033	3.468	1.115	3.964	0.036
418	100.0	0.038	3.155	1.199	3.872	0.025
419	100.0	0.027	3.197	1.166	3.747	0.027
420	100.0	0.027	3.305	1.07	3.477	0.029

RELATIVE WEIGHTS - INDIVIDUAL VALUES
SUBSET 1 (POSTNATAL DAY 21)

Sacrifice R1
Sex F
Group 1

	Pituitary gland %	Spleen %	Thyroid glands %	Uterus %
M	0.004	0.382	0.012	0.091
SD	0.001	0.057	0.006	0.014
n	10	10	10	10
411	0.004	0.406	0.004	0.075
412	0.001	0.398	0.013	0.081
413	0.001	0.369	0.002	0.094
414	0.004	0.5	0.01	0.091
415	0.005	0.352	0.022	0.076
416	0.003	0.33	0.019	0.123
417	0.004	0.289	0.014	0.093
418	0.004	0.414	0.01	0.098
419	0.005	0.397	0.016	0.091
420	0.004	0.366	0.011	0.082

Reproductive Toxicity Study to detect potential effects to anti-androgenic substances in Wistar Rats (Gavage) 28.Mar.2014 SIGR

RELATIVE WEIGHTS - INDIVIDUAL VALUES
SUBSET 1 (POSTNATAL DAY 21)

Sacrifice R1
Sex F
Group 2

	Term. body weight %	Adrenal glands %	Brain %	Kidneys %	Liver %	Ovaries %
M	100.0	0.03	3.296	1.114	3.655	0.031
SD		0.006	0.235	0.073	0.311	0.011
n	10	10	10	10	10	10
421	100.0	0.027	3.542	1.05	3.25	0.025
422	100.0	0.031	3.582	1.113	3.577	0.026
423	100.0	0.024	3.255	1.174	3.408	0.012
424	100.0	0.035	2.89	1.154	3.756	0.033
425	100.0	0.034	3.084	1.013	3.664	0.026
426	100.0	0.022	3.342	1.156	3.422	0.029
427	100.0	0.036	3.206	1.061	3.898	0.045
428	100.0	0.03	3.081	1.042	3.459	0.025
429	100.0	0.022	3.418	1.252	4.337	0.044
430	100.0	0.035	3.559	1.122	3.775	0.046

RELATIVE WEIGHTS - INDIVIDUAL VALUES
SUBSET 1 (POSTNATAL DAY 21)

Sacrifice R1
Sex F
Group 2

	Pituitary gland %	Spleen %	Thyroid glands %	Uterus %
M	0.003	0.39	0.014	0.094
SD	0.001	0.04	0.007	0.013
n	10	10	10	10
421	0.004	0.344	0.005	0.097
422	0.002	0.353	0.005	0.093
423	0.003	0.42	0.012	0.1
424	0.003	0.404	0.013	0.077
425	0.003	0.383	0.009	0.079
426	0.003	0.44	0.029	0.091
427	0.004	0.419	0.019	0.097
428	0.004	0.442	0.017	0.081
429	0.005	0.335	0.015	0.1
430	0.003	0.357	0.019	0.122

Reproductive Toxicity Study to detect potential effects to anti-androgenic substances in Wistar Rats (Gavage) 28.Mar.2014 SIGR

RELATIVE WEIGHTS - INDIVIDUAL VALUES
SUBSET 1 (POSTNATAL DAY 21)

Sacrifice R1
Sex F
Group 3

	Term. body weight %	Adrenal glands %	Brain %	Kidneys %	Liver %	Ovaries %
M	100.0	0.036	3.155	1.099	3.682	0.034
SD		0.007	0.231	0.061	0.192	0.009
n	10	10	10	10	10	10
431	100.0	0.031	2.841	1.128	3.39	0.019
432	100.0	0.054	3.192	1.118	3.491	0.045
433	100.0	0.037	2.772	1.222	3.567	0.028
434	100.0	0.029	3.368	1.067	3.817	0.036
435	100.0	0.036	3.242	1.056	3.866	0.036
436	100.0	0.035	3.021	0.985	3.929	0.023
437	100.0	0.032	3.245	1.095	3.731	0.032
438	100.0	0.036	3.522	1.132	3.679	0.043
439	100.0	0.03	3.273	1.095	3.475	0.037
440	100.0	0.042	3.078	1.09	3.876	0.046

RELATIVE WEIGHTS - INDIVIDUAL VALUES
SUBSET 1 (POSTNATAL DAY 21)

Sacrifice R1
Sex F
Group 3

	Pituitary gland %	Spleen %	Thyroid glands %	Uterus %
M	0.004	0.434	0.019	0.095
SD	0.001	0.082	0.006	0.013
n	10	10	10	10
431	0.002	0.514	0.019	0.095
432	0.003	0.44	0.015	0.121
433	0.005	0.594	0.022	0.081
434	0.005	0.45	0.023	0.111
435	0.004	0.387	0.017	0.094
436	0.004	0.352	0.009	0.085
437	0.002	0.49	0.031	0.084
438	0.003	0.368	0.016	0.103
439	0.004	0.333	0.018	0.095
440	0.004	0.406	0.018	0.082

Reproductive Toxicity Study to detect potential effects to anti-androgenic substances in Wistar Rats (Gavage)

28.Mar.2014 SIGR

RELATIVE WEIGHTS - INDIVIDUAL VALUES
SUBSET 1 (POSTNATAL DAY 21)

Sacrifice R1
Sex F
Group 4

	Term. body weight %	Adrenal glands %	Brain %	Kidneys %	Liver %	Ovaries %
M	100.0	0.034	3.31	1.129	3.649	0.035
SD		0.005	0.37	0.069	0.184	0.007
n	10	10	10	10	10	10
441	100.0	0.031	3.471	1.075	3.701	0.016
442	100.0	0.032	3.443	1.023	3.828	0.041
443	100.0	0.032	3.23	1.143	3.768	0.036
444	100.0	0.034	3.801	1.199	3.549	0.042
445	100.0	0.037	3.392	1.077	3.37	0.037
446	100.0	0.026	3.418	1.104	3.483	0.037
447	100.0	0.032	2.842	1.104	3.438	0.035
448	100.0	0.043	2.535	1.114	3.706	0.036
449	100.0	0.035	3.335	1.249	3.949	0.028
450	100.0	0.037	3.63	1.199	3.702	0.037

RELATIVE WEIGHTS - INDIVIDUAL VALUES
SUBSET 1 (POSTNATAL DAY 21)

Sacrifice R1
Sex F
Group 4

	Pituitary gland %	Spleen %	Thyroid glands %	Uterus %
M	0.004	0.418	0.017	0.087
SD	0.001	0.099	0.007	0.014
n	10	10	10	10
441	0.003	0.24	0.032	0.075
442	0.003	0.41	0.013	0.084
443	0.004	0.541	0.01	0.08
444	0.004	0.381	0.015	0.092
445	0.005	0.421	0.019	0.101
446	0.005	0.399	0.021	0.072
447	0.003	0.385	0.015	0.065
448	0.004	0.609	0.007	0.102
449	0.004	0.374	0.014	0.102
450	0.006	0.424	0.019	0.102

Reproductive Toxicity Study to detect potential effects to anti-androgenic substances in Wistar Rats (Gavage) 28.Mar.2014 SIGR

ABSOLUTE WEIGHTS - INDIVIDUAL VALUES
SUBSET 2 (SEXUAL MATURITY)

Sacrifice R2
Sex M
Group 0

	Term. body weight g	Adrenal glands mg	Brain g	Bulbo-urethral gland mg	Cauda epididymis mg	Epididymides mg
M	184.71	38.6	1.813	13.86	57.7	220.2
SD	18.572	7.168	0.067	3.294	11.461	44.296
n	10	10	10	10	10	10
501	217.9	50.0	1.794	17.4	75.0	254.0
502	170.1	36.0	1.811	11.2	45.0	181.0
503	163.6	33.0	1.716	12.8	45.0	158.0
504	167.1	37.0	1.758	7.3	50.0	217.0
505	182.1	29.0	1.744	14.0	50.0	174.0
506	173.6	33.0	1.85	13.8	62.0	198.0
507	172.7	37.0	1.782	17.1	56.0	234.0
508	205.8	51.0	1.91	13.3	78.0	307.0
509	195.2	38.0	1.857	13.1	56.0	238.0
510	199.0	42.0	1.911	18.6	60.0	241.0

ABSOLUTE WEIGHTS - INDIVIDUAL VALUES
SUBSET 2 (SEXUAL MATURITY)

Sacrifice R2
Sex M
Group 0

	Glans penis mg	Kidneys g	Liver g	Muscles bulb + l.a mg	Pituitary gland mg	Prostate mg
M	49.53	1.582	8.294	194.66	6.64	187.1
SD	7.358	0.155	1.294	33.864	0.916	38.297
n	10	10	10	10	10	10
501	55.2	1.735	10.756	246.9	7.1	221.8
502	40.0	1.503	8.013	148.5	6.2	129.7
503	41.5	1.467	7.035	221.0	6.5	147.4
504	49.7	1.408	6.66	164.7	4.5	125.5
505	40.4	1.528	7.99	179.1	7.2	195.4
506	45.6	1.527	7.698	157.8	7.2	195.1
507	54.6	1.407	7.435	192.9	6.0	208.4
508	52.4	1.866	10.055	214.7	7.6	225.4
509	54.4	1.657	8.386	184.4	7.4	218.9
510	61.5	1.726	8.914	236.6	6.7	203.4

Reproductive Toxicity Study to detect potential effects to anti-androgenic substances in Wistar Rats (Gavage) 28.Mar.2014 SIGR

ABSOLUTE WEIGHTS - INDIVIDUAL VALUES
SUBSET 2 (SEXUAL MATURITY)

Sacrifice R2
Sex M
Group 0

	Prostate vent.fresh mg	Seminal vesicle mg	Spleen g	Testes mg	Thyroid glands mg
M	106.59	109.97	0.503	2227.1	13.87
SD	29.408	36.366	0.084	321.053	2.62
n	10	10	10	10	10
501	134.1	172.6	0.6	2354.0	12.9
502	53.5	86.4	0.447	2024.0	13.8
503	82.9	112.3	0.393	1691.0	11.1
504	67.6	61.1	0.378	2133.0	15.0
505	104.8	97.6	0.53	2022.0	13.3
506	116.9	66.9	0.542	1956.0	11.8
507	116.3	100.6	0.583	2344.0	14.3
508	127.4	143.7	0.483	2766.0	14.3
509	143.0	106.3	0.457	2579.0	11.8
510	119.4	152.2	0.613	2402.0	20.4

ABSOLUTE WEIGHTS - INDIVIDUAL VALUES
SUBSET 2 (SEXUAL MATURITY)

Sacrifice R2
Sex M
Group 1

	Term. body weight g	Adrenal glands mg	Brain g	Bulbo-uret hral gland mg	Cauda epididymis mg	Epididy-mides mg
M	186.5	38.0	1.829	15.43	59.7	225.9
SD	9.893	5.142	0.084	4.451	10.884	32.378
n	10	10	10	10	10	10
511	185.9	31.0	1.787	20.2	84.0	269.0
512	173.7	37.0	1.843	10.7	51.0	197.0
513	186.3	43.0	1.748	10.2	49.0	189.0
514	209.6	45.0	2.035	24.9	67.0	272.0
515	179.3	34.0	1.861	14.8	58.0	224.0
516	180.4	36.0	1.826	16.1	61.0	229.0
517	181.1	33.0	1.765	11.8	59.0	191.0
518	187.0	39.0	1.757	14.2	45.0	231.0
519	187.2	36.0	1.864	16.2	60.0	197.0
520	194.5	46.0	1.799	15.2	63.0	260.0

ABSOLUTE WEIGHTS - INDIVIDUAL VALUES
SUBSET 2 (SEXUAL MATURITY)

Sacrifice R2
Sex M
Group 1

	Glans penis mg	Kidneys g	Liver g	Muscles bulb + l.a mg	Pituitary gland mg	Prostate mg
M	52.69	1.577	8.485	213.13	6.75	204.36
SD	10.139	0.114	0.582	41.405	0.45	32.898
n	10	10	10	10	10	10
511	64.4	1.688	8.204	210.6	6.6	228.3
512	47.4	1.466	7.892	157.4	6.2	175.3
513	38.1	1.562	8.577	177.0	6.4	184.1
514	64.3	1.774	9.634	310.8	7.5	257.1
515	62.9	1.565	8.009	230.7	6.7	225.4
516	64.0	1.551	7.908	197.4	7.0	239.8
517	44.0	1.466	8.208	236.7	6.4	158.5
518	44.1	1.479	8.343	210.7	6.3	174.2
519	49.7	1.487	9.136	197.8	7.1	186.5
520	48.0	1.728	8.937	202.2	7.3	214.4

Reproductive Toxicity Study to detect potential effects to anti-androgenic substances in Wistar Rats (Gavage) 28.Mar.2014 SIGR

ABSOLUTE WEIGHTS - INDIVIDUAL VALUES
SUBSET 2 (SEXUAL MATURITY)

Sacrifice R2
Sex M
Group 1

	Prostate vent.fresh mg	Seminal vesicle mg	Spleen g	Testes mg	Thyroid glands mg
M	114.06	117.75	0.503	2093.9	15.16
SD	20.204	37.173	0.048	154.209	2.447
n	10	10	10	10	10
511	118.6	147.1	0.43	2267.0	17.5
512	91.1	73.9	0.51	1929.0	16.0
513	97.0	82.9	0.593	1961.0	10.8
514	155.0	181.1	0.491	2268.0	18.5
515	131.5	163.7	0.548	2046.0	12.6
516	129.4	88.9	0.544	2059.0	15.7
517	98.6	85.9	0.481	2047.0	17.8
518	95.6	100.7	0.452	2200.0	13.9
519	108.0	123.7	0.488	1867.0	15.1
520	115.8	129.6	0.497	2295.0	13.7

Reproductive Toxicity Study to detect potential effects to anti-androgenic substances in Wistar Rats (Gavage) 28.Mar.2014 SIGR

ABSOLUTE WEIGHTS - INDIVIDUAL VALUES
SUBSET 2 (SEXUAL MATURITY)

Sacrifice R2
Sex M
Group 2

	Term. body weight g	Adrenal glands mg	Brain g	Bulbo-uret hral gland mg	Cauda epididymis mg	Epididy- mides mg
M	186.08	38.7	1.796	13.94	57.6	227.8
SD	18.631	6.684	0.102	5.03	9.594	35.273
n	10	10	10	10	10	10
521	160.3	30.0	1.666	10.4	47.0	189.0
522	155.8	35.0	1.681	10.2	46.0	185.0
523	193.1	30.0	1.86	15.5	52.0	233.0
524	175.1	34.0	1.798	9.8	63.0	193.0
525	206.1	45.0	1.735	9.9	47.0	206.0
526	205.3	48.0	1.937	11.6	54.0	284.0
527	190.4	40.0	1.771	11.8	63.0	224.0
528	199.2	38.0	1.96	25.4	69.0	277.0
529	202.0	48.0	1.84	17.4	72.0	238.0
530	173.5	39.0	1.711	17.4	63.0	249.0

ABSOLUTE WEIGHTS - INDIVIDUAL VALUES
SUBSET 2 (SEXUAL MATURITY)

Sacrifice R2
Sex M
Group 2

	Glans penis mg	Kidneys g	Liver g	Muscles bulb + l.a mg	Pituitary gland mg	Prostate mg
M	52.82	1.57	8.437	191.78	6.56	185.06
SD	10.388	0.161	0.967	30.67	0.809	36.113
n	10	10	10	10	10	10
521	52.1	1.296	7.161	173.5	5.3	186.7
522	28.6	1.404	7.08	163.9	6.3	141.1
523	61.5	1.642	9.0	210.4	7.0	226.9
524	44.6	1.575	7.687	162.4	6.9	165.6
525	55.4	1.672	9.465	186.6	6.5	125.8
526	51.9	1.855	9.952	220.4	8.0	225.0
527	55.6	1.525	8.826	178.0	6.7	158.9
528	64.0	1.645	8.725	233.8	7.2	222.8
529	62.8	1.654	8.684	235.2	5.4	205.0
530	51.7	1.429	7.794	153.6	6.3	192.8

ABSOLUTE WEIGHTS - INDIVIDUAL VALUES
SUBSET 2 (SEXUAL MATURITY)

Sacrifice R2
Sex M
Group 2

	Prostate vent.fresh mg	Seminal vesicle mg	Spleen g	Testes mg	Thyroid glands mg
M	97.2	117.08	0.498	2190.6	14.25
SD	24.405	38.851	0.111	283.883	2.718
n	10	10	10	10	10
521	99.8	84.7	0.436	1711.0	10.8
522	78.2	61.7	0.479	1846.0	11.2
523	117.8	147.5	0.421	2255.0	12.5
524	99.1	88.0	0.509	1924.0	12.8
525	61.5	78.7	0.54	2260.0	17.5
526	123.9	153.8	0.743	2589.0	12.9
527	66.5	104.0	0.499	2205.0	17.9
528	137.2	179.2	0.586	2487.0	14.1
529	91.3	143.1	0.423	2412.0	18.0
530	96.7	130.1	0.339	2217.0	14.8

ABSOLUTE WEIGHTS - INDIVIDUAL VALUES
SUBSET 2 (SEXUAL MATURITY)

Sacrifice R2
Sex M
Group 3

	Term. body weight g	Adrenal glands mg	Brain g	Bulbo-urethral gland mg	Cauda epididymis mg	Epididymides mg
M	218.17	46.5	1.835	11.36	80.0	351.2
SD	18.994	7.106	0.074	6.218	22.341	68.14
n	10	10	10	10	10	10
531	239.0	57.0	1.873	23.8	101.0	450.0
532	209.9	41.0	1.736	12.9	63.0	304.0
533	189.7	46.0	1.794	4.0	55.0	224.0
534	210.7	37.0	1.81	2.2	53.0	278.0
535	195.7	50.0	1.755	10.8	125.0	350.0
536	247.9	54.0	1.814	12.3	82.0	416.0
537	224.6	42.0	1.961	8.3	75.0	387.0
538	215.2	48.0	1.877	8.4	88.0	373.0
539	211.1	37.0	1.797	15.6	67.0	334.0
540	237.9	53.0	1.934	15.3	91.0	396.0

ABSOLUTE WEIGHTS - INDIVIDUAL VALUES
SUBSET 2 (SEXUAL MATURITY)

Sacrifice R2
Sex M
Group 3

	Glans penis mg	Kidneys g	Liver g	Muscles bulb + l.a mg	Pituitary gland mg	Prostate mg
M	42.16	1.788	9.775	199.0	6.77	192.23
SD	6.29	0.175	0.665	42.468	1.029	38.35
n	10	10	10	10	10	10
531	49.3	1.922	10.402	305.7	6.6	278.9
532	45.4	1.736	9.778	197.8	6.2	186.6
533	44.6	1.514	9.077	146.1	6.9	183.5
534	38.8	1.67	9.555	176.0	5.6	156.9
535	44.9	1.67	9.546	180.8	6.5	152.2
536	38.6	2.152	11.256	196.1	8.0	207.9
537	36.5	1.809	9.914	203.6	8.2	173.8
538	50.0	1.781	9.387	210.5	6.3	228.4
539	29.5	1.719	8.983	167.7	5.3	162.7
540	44.0	1.909	9.85	205.7	8.1	191.4

ABSOLUTE WEIGHTS - INDIVIDUAL VALUES
SUBSET 2 (SEXUAL MATURITY)

Sacrifice R2
Sex M
Group 3

	Prostate vent.fresh mg	Seminal vesicle mg	Spleen g	Testes mg	Thyroid glands mg
M	101.38	137.3	0.543	2708.6	17.88
SD	22.219	54.67	0.075	217.225	2.304
n	10	10	10	10	10
531	145.7	243.9	0.597	2822.0	20.9
532	99.6	113.0	0.551	2728.0	16.1
533	100.4	106.2	0.505	2300.0	15.5
534	89.8	91.2	0.567	2651.0	18.0
535	61.3	84.0	0.68	2516.0	17.7
536	116.9	179.1	0.517	3136.0	16.5
537	83.7	102.5	0.609	2833.0	20.5
538	106.3	156.1	0.495	2714.0	15.8
539	97.0	96.3	0.41	2641.0	16.2
540	113.1	200.7	0.496	2745.0	21.6

Reproductive Toxicity Study to detect potential effects to anti-androgenic substances in Wistar Rats (Gavage)

ABSOLUTE WEIGHTS - INDIVIDUAL VALUES
SUBSET 2 (SEXUAL MATURITY)

Sacrifice R2
Sex M
Group 4

	Term. body weight g	Adrenal glands mg	Brain g	Bulbo-uret hral gland mg	Cauda epididymis mg	Epididy- mides mg
M	189.1	40.3	1.842	13.97	58.9	226.1
SD	16.616	3.129	0.063	4.559	11.808	42.275
n	10	10	10	10	10	10
541	177.3	39.0	1.868	8.8	52.0	204.0
542	168.9	34.0	1.81	13.8	48.0	196.0
543	191.9	45.0	1.737	18.9	59.0	226.0
544	174.5	41.0	1.849	7.6	53.0	193.0
545	180.9	38.0	1.836	10.5	54.0	184.0
546	200.4	38.0	1.898	13.2	64.0	247.0
547	174.2	41.0	1.778	10.5	52.0	183.0
548	215.4	42.0	1.909	19.5	54.0	259.0
549	194.3	42.0	1.798	18.6	64.0	256.0
550	213.2	43.0	1.939	18.3	89.0	313.0

ABSOLUTE WEIGHTS - INDIVIDUAL VALUES
SUBSET 2 (SEXUAL MATURITY)

Sacrifice R2
Sex M
Group 4

	Glans penis mg	Kidneys g	Liver g	Muscles bulb + l.a mg	Pituitary gland mg	Prostate mg
M	52.43	1.674	8.686	215.3	6.71	222.12
SD	8.923	0.191	0.819	51.306	0.551	39.177
n	10	10	10	10	10	10
541	45.8	1.534	7.674	179.5	5.9	179.3
542	57.6	1.429	7.634	239.4	6.5	216.7
543	48.2	1.822	8.449	279.4	7.4	217.2
544	40.7	1.494	8.002	170.5	6.7	174.7
545	51.2	1.689	9.354	171.3	5.9	184.2
546	63.8	1.652	9.329	225.2	7.0	236.0
547	39.2	1.62	7.99	143.9	7.0	199.8
548	61.2	1.574	9.757	306.0	7.0	265.8
549	62.7	1.875	9.172	206.1	6.3	261.3
550	53.9	2.05	9.497	231.7	7.4	286.2

Reproductive Toxicity Study to detect potential effects
to anti-androgenic substances in Wistar Rats (Gavage)

ABSOLUTE WEIGHTS - INDIVIDUAL VALUES
SUBSET 2 (SEXUAL MATURITY)

Sacrifice R2
Sex M
Group 4

	Prostate vent.fresh mg	Seminal vesicle mg	Spleen g	Testes mg	Thyroid glands mg	
M	120.63	110.54	0.512	2144.5	16.111	
SD	27.322	36.083	0.077	282.858	2.177	
n	10	10	10	10	9	
541	92.8	105.3	0.407	2062.0	13.5	
542	117.1	114.4	0.386	1926.0	13.5	
543	100.5	130.8	0.597	2052.0	16.4	
544	91.1	65.9	0.504	1787.0	15.5	
545	94.3	65.3	0.533	1948.0	12.0	s
546	148.8	110.9	0.624	2390.0	16.5	
547	111.3	61.3	0.58	1856.0	15.8	
548	164.0	149.5	0.474	2590.0	20.9	
549	133.7	148.9	0.515	2423.0	16.0	
550	152.7	153.1	0.501	2411.0	16.9	

ABSOLUTE WEIGHTS - INDIVIDUAL VALUES
SUBSET 2 (SEXUAL MATURITY)

Sacrifice R2
Sex F
Group 0

	Term. body weight g	Adrenal glands mg	Brain g	Kidneys g	Liver g	Ovaries mg
M	84.5	28.4	1.532	0.913	3.93	35.9
SD	8.125	8.796	0.044	0.072	0.519	9.469
n	10	10	10	10	10	10
601	99.7	35.0	1.542	1.061	4.32	48.0
602	82.4	32.0	1.526	0.898	3.738	33.0
603	70.0	18.0	1.524	0.798	3.064	23.0
604	81.9	34.0	1.464	0.899	3.385	40.0
605	90.1	47.0	1.523	0.982	4.661	53.0
606	89.0	26.0	1.473	0.914	4.231	31.0
607	86.5	19.0	1.59	0.92	3.84	39.0
608	88.0	27.0	1.606	0.937	4.609	36.0
609	76.9	23.0	1.547	0.869	3.785	31.0
610	80.5	23.0	1.529	0.856	3.667	25.0

ABSOLUTE WEIGHTS - INDIVIDUAL VALUES
SUBSET 2 (SEXUAL MATURITY)

Sacrifice R2
Sex F
Group 0

	Pituitary gland mg	Spleen g	Thyroid glands mg	Uterus mg
M	5.49	0.252	11.28	299.5
SD	0.824	0.031	1.827	53.303
n	10	10	10	10
601	6.1	0.224	11.2	266.0
602	5.1	0.283	13.8	404.0
603	4.7	0.196	9.6	387.0
604	7.1	0.268	12.7	302.0
605	4.7	0.238	10.4	284.0
606	4.8	0.287	10.2	277.0
607	6.2	0.263	10.0	249.0
608	5.8	0.272	12.1	287.0
609	5.7	0.219	8.7	252.0
610	4.7	0.272	14.1	287.0

ABSOLUTE WEIGHTS - INDIVIDUAL VALUES
SUBSET 2 (SEXUAL MATURITY)

Sacrifice R2
Sex F
Group 1

	Term. body weight g	Adrenal glands mg	Brain g	Kidneys g	Liver g	Ovaries mg
M	82.15	25.7	1.564	0.94	3.907	36.1
SD	8.242	7.454	0.06	0.084	0.545	7.965
n	10	10	10	10	10	10
611	83.0	31.0	1.602	0.919	3.556	35.0
612	70.5	24.0	1.609	1.004	4.548	33.0
613	86.4	23.0	1.477	0.895	4.62	22.0
614	93.2	31.0	1.645	1.064	4.393	27.0
615	70.9	16.0	1.493	0.771	3.029	35.0
616	91.5	30.0	1.56	0.944	4.052	44.0
617	85.9	35.0	1.6	0.979	3.85	44.0
618	75.6	26.0	1.615	0.921	3.332	41.0
619	87.9	30.0	1.547	1.022	4.209	33.0
620	76.6	11.0	1.493	0.878	3.48	47.0

ABSOLUTE WEIGHTS - INDIVIDUAL VALUES
SUBSET 2 (SEXUAL MATURITY)

Sacrifice R2
Sex F
Group 1

	Pituitary gland mg	Spleen g	Thyroid glands mg	Uterus mg
M	5.76	0.26	10.19	322.7
SD	1.071	0.053	1.233	99.992
n	10	10	10	10
611	7.8	0.253	11.5	272.0
612	6.3	0.306	11.7	258.0
613	5.4	0.283	10.5	552.0
614	5.8	0.244	10.0	408.0
615	4.8	0.162	11.8	241.0
616	6.0	0.328	9.9	309.0
617	6.2	0.32	10.3	285.0
618	4.8	0.219	9.2	252.0
619	6.5	0.272	8.8	396.0
620	4.0	0.212	8.2	254.0

ABSOLUTE WEIGHTS - INDIVIDUAL VALUES
SUBSET 2 (SEXUAL MATURITY)

Sacrifice R2
Sex F
Group 2

	Term. body weight g	Adrenal glands mg	Brain g	Kidneys g	Liver g	Ovaries mg
M	82.12	26.9	1.578	0.902	3.778	30.7
SD	8.496	7.37	0.039	0.092	0.459	7.602
n	10	10	10	10	10	10
621	86.8	39.0	1.568	0.968	4.328	38.0
622	83.9	22.0	1.641	0.952	3.682	24.0
623	77.0	20.0	1.604	0.799	3.562	28.0
624	81.1	24.0	1.574	0.858	3.945	28.0
625	82.3	21.0	1.598	0.88	3.696	40.0
626	91.4	35.0	1.578	1.023	4.312	38.0
627	82.6	32.0	1.608	0.978	3.555	38.0
628	93.6	26.0	1.529	0.986	4.316	32.0
629	79.7	17.0	1.575	0.818	3.499	22.0
630	62.8	33.0	1.508	0.753	2.883	19.0

Reproductive Toxicity Study to detect potential effects
to anti-androgenic substances in Wistar Rats (Gavage)

ABSOLUTE WEIGHTS - INDIVIDUAL VALUES
SUBSET 2 (SEXUAL MATURITY)

Sacrifice R2
Sex F
Group 2

	Pituitary gland mg	Spleen g	Thyroid glands mg	Uterus mg
M	4.71	0.254	12.89	318.7
SD	0.829	0.057	3.552	96.701
n	10	10	10	10
621	5.4	0.324	11.4	303.0
622	4.4	0.291	10.6	445.0
623	5.0	0.275	9.5	217.0
624	4.3	0.189	9.4	504.0
625	5.5	0.2	11.8	247.0
626	5.2	0.309	10.6	330.0
627	5.7	0.257	16.6	301.0
628	4.8	0.273	20.0	310.0
629	3.2	0.278	12.7	188.0
630	3.6	0.145	16.3	342.0

Reproductive Toxicity Study to detect potential effects to anti-androgenic substances in Wistar Rats (Gavage)

28.Mar.2014 SIGR

ABSOLUTE WEIGHTS - INDIVIDUAL VALUES
SUBSET 2 (SEXUAL MATURITY)

Sacrifice R2
Sex F
Group 3

	Term. body weight g	Adrenal glands mg	Brain g	Kidneys g	Liver g	Ovaries mg
M	83.64	27.8	1.575	0.905	4.091	28.9
SD	7.062	3.967	0.049	0.091	0.387	5.567
n	10	10	10	10	10	10
631	72.5	31.0	1.494	0.834	3.683	26.0
632	89.3	27.0	1.605	0.985	4.284	33.0
633	82.8	28.0	1.609	0.938	3.818	33.0
634	91.8	28.0	1.624	1.023	4.559	27.0
635	71.2	27.0	1.566	0.739	3.388	28.0
636	90.6	27.0	1.605	0.946	4.271	23.0
637	82.0	33.0	1.482	0.843	4.304	18.0
638	86.9	33.0	1.593	1.01	4.516	35.0
639	82.8	20.0	1.577	0.844	3.826	35.0
640	86.5	24.0	1.596	0.888	4.257	31.0

Reproductive Toxicity Study to detect potential effects to anti-androgenic substances in Wistar Rats (Gavage) 28.Mar.2014 SIGR

ABSOLUTE WEIGHTS - INDIVIDUAL VALUES
SUBSET 2 (SEXUAL MATURITY)

Sacrifice R2
Sex F
Group 3

	Pituitary gland mg	Spleen g	Thyroid glands mg	Uterus mg
M	3.97	0.266	13.76	325.5
SD	0.556	0.032	3.247	121.802
n	10	10	10	10
631	3.8	0.249	10.1	127.0
632	4.8	0.227	13.0	286.0
633	4.3	0.274	10.3	601.0
634	3.9	0.313	16.8	392.0
635	4.1	0.219	9.4	342.0
636	3.1	0.284	15.8	242.0
637	3.1	0.312	14.6	328.0
638	4.6	0.255	15.7	294.0
639	4.1	0.251	19.3	277.0
640	3.9	0.28	12.6	366.0

Reproductive Toxicity Study to detect potential effects to anti-androgenic substances in Wistar Rats (Gavage) 28.Mar.2014 SIGR

ABSOLUTE WEIGHTS - INDIVIDUAL VALUES
SUBSET 2 (SEXUAL MATURITY)

Sacrifice R2
Sex F
Group 4

	Term. body weight g	Adrenal glands mg	Brain g	Kidneys g	Liver g	Ovaries mg
M	86.92	25.8	1.572	0.909	3.845	31.0
SD	8.916	4.872	0.056	0.092	0.492	7.439
n	10	10	10	10	10	10
641	79.2	19.0	1.571	0.813	3.255	21.0
642	99.5	28.0	1.631	0.962	4.361	40.0
643	85.4	24.0	1.591	0.802	3.663	31.0
644	93.1	29.0	1.537	0.912	3.916	34.0
645	90.4	20.0	1.497	0.86	3.906	22.0
646	83.0	32.0	1.525	0.929	4.097	32.0
647	70.4	25.0	1.615	0.808	2.991	36.0
648	95.8	33.0	1.641	1.092	4.554	39.0
649	92.4	27.0	1.49	0.981	4.194	35.0
650	80.0	21.0	1.62	0.927	3.517	20.0

Reproductive Toxicity Study to detect potential effects to anti-androgenic substances in Wistar Rats (Gavage)

28.Mar.2014 SIGR

ABSOLUTE WEIGHTS - INDIVIDUAL VALUES
SUBSET 2 (SEXUAL MATURITY)

Sacrifice R2
Sex F
Group 4

	Pituitary gland mg	Spleen g	Thyroid glands mg	Uterus mg
M	5.29	0.257	11.93	294.4
SD	0.684	0.035	2.016	101.44
n	10	10	10	10
641	4.7	0.219	9.7	85.0
642	5.5	0.262	10.5	281.0
643	5.9	0.3	11.2	282.0
644	6.3	0.284	14.1	295.0
645	4.3	0.234	12.3	356.0
646	5.4	0.293	11.7	491.0
647	5.1	0.207	8.8	261.0
648	5.9	0.293	14.0	251.0
649	5.5	0.263	15.1	293.0
650	4.3	0.218	11.9	349.0

RELATIVE WEIGHTS - INDIVIDUAL VALUES
SUBSET 2 (SEXUAL MATURITY)

Sacrifice			R2			
Sex			M			
Group			0	1	2	3
Terminal body weight	%	M	100.0	100.0	100.0	100.0
		% dev	100	100	100	100
		n	10	10	10	10
Adrenal glands	%	M	0.021	0.02	0.021	0.021
		% dev	100	98	100	102
		SD	0.002	0.002	0.003	0.003
		n	10	10	10	10
Brain	%	M	0.988	0.982	0.97	0.845
		% dev	100	99	98	86
		SD	0.078	0.045	0.069	0.06
		n	10	10	10	10
Bulbo-urethral gland	%	M	0.007	0.008	0.007	0.005
		% dev	100	110	100	68
		SD	0.002	0.002	0.002	0.003
		n	10	10	10	10
Cauda epididymis	%	M	0.031	0.032	0.031	0.037
		% dev	100	103	100	118
		SD	0.004	0.006	0.005	0.011
		n	10	10	10	10
Epididymides	%	M	0.119	0.121	0.122	0.16
		% dev	100	102	103	135
		SD	0.017	0.014	0.014	0.022
		n	10	10	10	10
Glans penis	%	M	0.027	0.028	0.028	0.019
		% dev	100	105	105	73
		SD	0.003	0.005	0.004	0.003
		n	10	10	10	10
Kidneys	%	M	0.858	0.845	0.844	0.819
		% dev	100	99	98	96
		SD	0.036	0.039	0.041	0.025
		n	10	10	10	10
Liver	%	M	4.472	4.548	4.531	4.492
		% dev	100	102	101	100
		SD	0.294	0.141	0.161	0.233
		n	10	10	10	10
Muscles bulb + l.ani	%	M	0.105	0.114	0.103	0.091
		% dev	100	108	98	86
		SD	0.015	0.018	0.011	0.015
		n	10	10	10	10

RELATIVE WEIGHTS - INDIVIDUAL VALUES
SUBSET 2 (SEXUAL MATURITY)

Sacrifice			R2			
Sex			M	F		
Group			4	0	1	2
Terminal body weight	%	M	100.0	100.0	100.0	100.0
		% dev	100	100	100	100
		n	10	10	10	10
Adrenal glands	%	M	0.021	0.033	0.031	0.033
		% dev	103	160	149	159
		SD	0.002	0.009	0.008	0.01
		n	10	10	10	10
Brain	%	M	0.979	1.828	1.921	1.941
		% dev	99	185	194	196
		SD	0.072	0.18	0.199	0.209
		n	10	10	10	10
Bulbo-urethral gland	%	M	0.007			
		% dev	98			
		SD	0.002			
		n	10			
Cauda epididymis	%	M	0.031			
		% dev	100			
		SD	0.004			
		n	10			
Epididymides	%	M	0.119			
		% dev	100			
		SD	0.013			
		n	10			
Glans penis	%	M	0.028			
		% dev	103			
		SD	0.004			
		n	10			
Kidneys	%	M	0.886	1.083	1.149	1.1
		% dev	103	126	134	128
		SD	0.075	0.034	0.112	0.06
		n	10	10	10	10
Liver	%	M	4.595	4.646	4.772	4.597
		% dev	103	104	107	103
		SD	0.233	0.367	0.669	0.217
		n	10	10	10	10
Muscles bulb + l.ani	%	M	0.113			
		% dev	108			
		SD	0.022			
		n	10			

RELATIVE WEIGHTS - INDIVIDUAL VALUES
SUBSET 2 (SEXUAL MATURITY)

Sacrifice			R2	
Sex			F	
Group			3	4
Terminal body weight	%	M	100.0	100.0
		% dev	100	100
		n	10	10
Adrenal glands	%	M	0.034	0.03
		% dev	161	143
		SD	0.006	0.005
		n	10	10
Brain	%	M	1.893	1.827
		% dev	192	185
		SD	0.14	0.221
		n	10	10
Kidneys	%	M	1.082	1.049
		% dev	126	122
		SD	0.056	0.087
		n	10	10
Liver	%	M	4.891	4.418
		% dev	109	99
		SD	0.229	0.256
		n	10	10

RELATIVE WEIGHTS - INDIVIDUAL VALUES
SUBSET 2 (SEXUAL MATURITY)

Sacrifice			R2			
Sex			M			
Group			0	1	2	3
Pituitary gland	%	M	0.004	0.004	0.004	0.003
		% dev	100	101	98	86
		SD	0.0	0.0	0.0	0.0
		n	10	10	10	10
Prostate	%	M	0.101	0.109	0.1	0.088
		% dev	100	109	99	87
		SD	0.015	0.016	0.018	0.014
		n	10	10	10	10
Prostate ventr.fresh	%	M	0.057	0.061	0.052	0.046
		% dev	100	107	92	81
		SD	0.013	0.009	0.012	0.008
		n	10	10	10	10
Seminal vesicle	%	M	0.059	0.063	0.062	0.062
		% dev	100	107	106	105
		SD	0.015	0.018	0.017	0.02
		n	10	10	10	10
Spleen	%	M	0.272	0.271	0.267	0.251
		% dev	100	99	98	92
		SD	0.039	0.032	0.05	0.044
		n	10	10	10	10
Testes	%	M	1.205	1.123	1.175	1.243
		% dev	100	93	98	103
		SD	0.116	0.066	0.073	0.046
		n	10	10	10	10
Thyroid glands	%	M	0.008	0.008	0.008	0.008
		% dev	100	108	101	109
		SD	0.001	0.001	0.001	0.001
		n	10	10	10	10

RELATIVE WEIGHTS - INDIVIDUAL VALUES
SUBSET 2 (SEXUAL MATURITY)

Sacrifice			R2			
Sex			M	F		
Group			4	0	1	2
Ovaries	%	M		0.042	0.045	0.037
		SD		0.008	0.011	0.007
		n		10	10	10
Pituitary gland	%	M	0.004	0.007	0.007	0.006
		% dev	99	181	195	159
		SD	0.0	0.001	0.001	0.001
		n	10	10	10	10
Prostate	%	M	0.117			
		% dev	116			
		SD	0.013			
		n	10			
Prostate ventr.fresh	%	M	0.063			
		% dev	111			
		SD	0.01			
		n	10			
Seminal vesicle	%	M	0.058			
		% dev	98			
		SD	0.016			
		n	10			
Spleen	%	M	0.272	0.3	0.316	0.307
		% dev	100	110	116	113
		SD	0.041	0.037	0.06	0.055
		n	10	10	10	10
Testes	%	M	1.131			
		% dev	94			
		SD	0.071			
		n	10			
Thyroid glands	%	M	0.008	0.013	0.013	0.016
		% dev	112	178	167	211
		SD	0.001	0.002	0.002	0.005
		n	9	10	10	10
Uterus	%	M		0.36	0.39	0.392
		SD		0.091	0.099	0.128
		n		10	10	10

RELATIVE WEIGHTS - INDIVIDUAL VALUES
SUBSET 2 (SEXUAL MATURITY)

Sacrifice			R2	
Sex			F	
Group			3	4
Ovaries	%	M	0.035	0.036
		SD	0.007	0.008
		n	10	10
Pituitary gland	%	M	0.005	0.006
		% dev	133	170
		SD	0.001	0.001
		n	10	10
Spleen	%	M	0.319	0.297
		% dev	117	109
		SD	0.034	0.033
		n	10	10
Thyroid glands	%	M	0.016	0.014
		% dev	217	182
		SD	0.003	0.002
		n	10	10
Uterus	%	M	0.389	0.341
		SD	0.147	0.125
		n	10	10

ABSOLUTE WEIGHTS - INDIVIDUAL VALUES
SUBSET 3 (POSTNATAL DAY 83 +/- 2 DAYS)

Sacrifice R3
Sex M
Group 0

	Term. body weight g	Adrenal glands mg	Brain g	Bulbo-uret hral gland mg	Cauda epididymis mg	Epididy-mides mg
M	315.945	53.111	1.945	74.867	324.667	897.111
SD	27.423	6.772	0.082	20.09	31.914	80.093
n	9	9	9	9	9	9
701	338.3	53.0	2.04	69.5	360.0	950.0
702	308.0	43.0	1.93	118.1	300.0	830.0
704	277.5	65.0	1.908	46.5	332.0	929.0
705	317.5	51.0	1.954	77.9	303.0	841.0
706	368.4	56.0	2.079	87.5	376.0	1068.0
707	311.8	50.0	1.946	67.0	279.0	826.0
708	284.9	52.0	1.933	62.0	333.0	856.0
709	329.2	47.0	1.925	64.9	298.0	844.0
710	307.9	61.0	1.788	80.4	341.0	930.0

Reproductive Toxicity Study to detect potential effects to anti-androgenic substances in Wistar Rats (Gavage) 28.Mar.2014 SIGR

ABSOLUTE WEIGHTS - INDIVIDUAL VALUES
SUBSET 3 (POSTNATAL DAY 83 +/- 2 DAYS)

Sacrifice R3
Sex M
Group 0

	Glans penis mg	Kidneys g	Liver g	Muscles bulb + l.a mg	Pituitary gland mg	Prostate mg
M	89.878	2.186	11.201	711.167	9.156	642.556
SD	10.305	0.208	1.671	83.305	0.794	61.158
n	9	9	9	9	9	9
701	93.7	2.33	11.21	690.8	9.9	692.4
702	83.9	2.17	11.82	722.2	9.1	691.5
704	74.6	2.069	10.512	614.1	8.8	597.8
705	82.9	2.116	10.87	629.4	9.1	567.3
706	84.8	2.654	13.542	796.3	10.8	757.1
707	98.7	2.196	10.766	709.3	9.3	616.9
708	110.2	1.911	8.627	882.3	8.7	648.1
709	89.6	2.084	13.793	669.6	8.1	584.9
710	90.5	2.14	9.667	686.5	8.6	627.0

Reproductive Toxicity Study to detect potential effects to anti-androgenic substances in Wistar Rats (Gavage)

ABSOLUTE WEIGHTS - INDIVIDUAL VALUES
SUBSET 3 (POSTNATAL DAY 83 +/- 2 DAYS)

Sacrifice R3
Sex M
Group 0

	Prostate vent.fresh mg	Seminal vesicle mg	Spleen g	Testes mg	Thyroid glands mg
M	347.578	989.233	0.532	3530.222	21.122
SD	52.268	176.964	0.062	253.004	3.597
n	9	9	9	9	9
701	294.9	878.9	0.5	3580.0	19.1
702	357.0	1208.5	0.48	3370.0	21.6
704	359.1	839.2	0.541	3772.0	27.2
705	284.9	946.8	0.47	3334.0	20.0
706	460.4	1291.1	0.48	3944.0	25.9
707	306.9	938.8	0.55	3247.0	21.2
708	349.6	898.0	0.507	3384.0	15.3
709	344.7	776.2	0.635	3335.0	19.1
710	370.7	1125.6	0.627	3806.0	20.7

ABSOLUTE WEIGHTS - INDIVIDUAL VALUES
SUBSET 3 (POSTNATAL DAY 83 +/- 2 DAYS)

Sacrifice R3
Sex M
Group 1

	Term. body weight g	Adrenal glands mg	Brain g	Bulbo-urethral gland mg	Cauda epididymis mg	Epididymides mg
M	306.83	56.5	1.989	69.79	311.0	899.3
SD	33.902	10.08	0.072	9.276	36.283	81.4
n	10	10	10	10	10	10
711	268.8	56.0	2.01	64.6	320.0	890.0
712	295.3	53.0	1.97	73.4	230.0	770.0
713	310.9	43.0	2.038	85.1	295.0	860.0
714	391.1	56.0	2.098	66.7	319.0	1021.0
715	283.8	63.0	1.904	64.9	307.0	914.0
716	317.7	59.0	2.06	68.1	351.0	954.0
717	296.2	39.0	1.923	63.4	306.0	851.0
718	282.4	64.0	1.928	56.4	287.0	796.0
719	322.3	58.0	1.904	85.4	346.0	987.0
720	299.8	74.0	2.056	69.9	349.0	950.0

Reproductive Toxicity Study to detect potential effects to anti-androgenic substances in Wistar Rats (Gavage) 28.Mar.2014 SIGR

ABSOLUTE WEIGHTS - INDIVIDUAL VALUES
SUBSET 3 (POSTNATAL DAY 83 +/- 2 DAYS)

Sacrifice R3
Sex M
Group 1

	Glans penis mg	Kidneys g	Liver g	Muscles bulb + l.a mg	Pituitary gland mg	Prostate mg
M	89.48	2.197	10.551	680.97	8.86	607.7
SD	12.615	0.233	1.53	100.956	0.914	88.399
n	10	10	10	10	10	10
711	79.2	1.98	9.06	623.4	9.1	594.7
712	91.1	2.04	9.22	630.6	8.7	525.8
713	84.2	2.163	10.817	666.0	8.4	622.6
714	98.2	2.719	14.365	806.0	10.4	762.4
715	81.4	2.178	9.426	510.6	8.2	519.5
716	89.4	2.107	11.315	753.1	8.0	689.4
717	94.3	2.202	10.581	690.6	9.4	520.2
718	76.0	1.902	9.833	562.9	7.5	535.6
719	81.7	2.293	10.638	762.8	10.1	715.9
720	119.3	2.39	10.257	803.7	8.8	590.9

Reproductive Toxicity Study to detect potential effects to anti-androgenic substances in Wistar Rats (Gavage) 28.Mar.2014 SIGR

ABSOLUTE WEIGHTS - INDIVIDUAL VALUES
SUBSET 3 (POSTNATAL DAY 83 +/- 2 DAYS)

Sacrifice R3
Sex M
Group 1

	Prostate vent.fresh mg	Seminal vesicle mg	Spleen g	Testes mg	Thyroid glands mg
M	323.82	931.65	0.523	3516.9	22.05
SD	65.265	122.39	0.068	328.563	4.331
n	10	10	10	10	10
711	316.3	902.1	0.51	3450.0	15.8
712	267.6	720.4	0.41	3040.0	23.5
713	357.9	839.5	0.566	3419.0	18.5
714	430.4	1069.9	0.57	4092.0	30.5
715	313.8	881.6	0.493	3841.0	20.3
716	390.5	1026.8	0.621	3789.0	18.9
717	228.2	1073.0	0.436	3358.0	24.4
718	256.0	797.3	0.478	3085.0	19.0
719	382.7	971.7	0.581	3490.0	24.2
720	294.8	1034.2	0.562	3605.0	25.4

ABSOLUTE WEIGHTS - INDIVIDUAL VALUES
SUBSET 3 (POSTNATAL DAY 83 +/- 2 DAYS)

Sacrifice R3
Sex M
Group 2

	Term. body weight g	Adrenal glands mg	Brain g	Bulbo-urethral gland mg	Cauda epididymis mg	Epididymides mg
M	313.16	50.5	1.942	62.45	288.6	917.3
SD	31.418	7.59	0.066	7.508	29.871	203.492
n	10	10	10	10	10	10
721	295.9	46.0	1.97	66.6	255.0	1470.0
722	299.8	47.0	1.96	55.8	260.0	790.0
723	337.2	42.0	2.02	65.0	308.0	913.0
724	315.9	56.0	2.035	72.9	277.0	855.0
725	324.2	55.0	1.958	65.7	279.0	896.0
726	276.8	51.0	1.885	61.4	338.0	941.0
727	360.2	68.0	1.913	67.3	303.0	887.0
728	353.4	49.0	1.873	66.6	328.0	878.0
729	304.8	44.0	1.976	56.2	285.0	795.0
730	263.4	47.0	1.828	47.0	253.0	748.0

ABSOLUTE WEIGHTS - INDIVIDUAL VALUES
SUBSET 3 (POSTNATAL DAY 83 +/- 2 DAYS)

Sacrifice R3
Sex M
Group 2

	Glans penis mg	Kidneys g	Liver g	Muscles bulb + l.a mg	Pituitary gland mg	Prostate mg
M	87.52	2.305	10.967	640.78	8.62	628.74
SD	10.282	0.276	1.295	136.849	0.973	99.961
n	10	10	10	10	10	10
721	77.6	2.66	11.14	558.0	8.8	550.8
722	91.5	2.34	10.02	452.2	8.1	531.5
723	81.3	2.262	11.57	832.4	9.0	728.0
724	83.5	2.169	11.517	575.8	9.0	645.6
725	94.4	2.336	10.779	776.2	8.6	661.2
726	80.9	2.337	10.105	587.1	7.1	561.4
727	108.7	2.682	12.243	792.4	10.5	800.9
728	97.9	2.477	13.039	767.8	9.2	729.2
729	77.2	2.004	10.892	561.2	8.6	578.0
730	82.2	1.784	8.36	504.7	7.3	500.8

ABSOLUTE WEIGHTS - INDIVIDUAL VALUES
SUBSET 3 (POSTNATAL DAY 83 +/- 2 DAYS)

Sacrifice R3
Sex M
Group 2

	Prostate vent.fresh mg	Seminal vesicle mg	Spleen g	Testes mg	Thyroid glands mg
M	345.33	858.62	0.538	3412.6	20.85
SD	65.235	65.788	0.093	319.826	3.553
n	10	10	10	10	10
721	295.7	891.3	0.46	3930.0	15.0
722	310.3	740.1	0.44	3310.0	17.2
723	430.2	903.3	0.586	3419.0	21.8
724	339.0	814.2	0.55	3433.0	20.6
725	355.1	866.4	0.473	3315.0	18.1
726	280.3	933.3	0.57	3743.0	20.5
727	445.9	945.6	0.634	3225.0	24.6
728	407.7	802.1	0.718	3770.0	27.3
729	338.6	805.6	0.517	2946.0	21.3
730	250.5	884.3	0.427	3035.0	22.1

Reproductive Toxicity Study to detect potential effects to anti-androgenic substances in Wistar Rats (Gavage) 28.Mar.2014 SIGR

ABSOLUTE WEIGHTS - INDIVIDUAL VALUES
SUBSET 3 (POSTNATAL DAY 83 +/- 2 DAYS)

Sacrifice R3
Sex M
Group 3

	Term. body weight g	Adrenal glands mg	Brain g	Bulbo-urethral gland mg	Cauda epididymis mg	Epididymides mg
M	301.65	56.4	1.939	42.09	277.2	833.5
SD	29.511	8.514	0.05	12.354	29.439	72.995
n	10	10	10	10	10	10
731	297.9	58.0	2.04	31.3	250.0	820.0
732	297.2	52.0	1.93	52.6	270.0	770.0
733	301.8	46.0	1.975	67.8	335.0	970.0
734	313.1	59.0	1.912	39.6	314.0	929.0
735	358.2	65.0	1.882	50.6	272.0	835.0
736	258.4	53.0	1.883	33.8	239.0	771.0
737	257.1	47.0	1.951	31.7	266.0	782.0
738	326.2	72.0	1.991	48.1	288.0	851.0
739	305.7	63.0	1.924	35.7	284.0	864.0
740	300.9	49.0	1.905	29.7	254.0	743.0

ABSOLUTE WEIGHTS - INDIVIDUAL VALUES
SUBSET 3 (POSTNATAL DAY 83 +/- 2 DAYS)

Sacrifice R3
Sex M
Group 3

	Glans penis mg	Kidneys g	Liver g	Muscles bulb + l.a mg	Pituitary gland mg	Prostate mg
M	73.81	2.228	11.516	449.97	8.22	443.89
SD	12.521	0.151	1.522	72.34	0.872	86.817
n	10	10	10	10	10	10
731	70.2	2.08	12.15	465.6	7.4	384.1
732	72.7	2.17	10.01	430.2	8.5	440.2
733	70.9	2.276	10.778	582.3	8.6	654.8
734	65.8	2.348	12.498	405.0	9.1	374.6
735	57.4	2.529	14.213	574.7	8.0	508.1
736	83.8	2.084	9.485	429.9	7.2	404.5
737	64.4	2.046	10.23	366.5	6.8	356.7
738	74.3	2.315	13.37	426.6	9.6	466.9
739	75.4	2.144	11.197	400.2	8.5	438.7
740	103.2	2.291	11.232	418.7	8.5	410.3

ABSOLUTE WEIGHTS - INDIVIDUAL VALUES
SUBSET 3 (POSTNATAL DAY 83 +/- 2 DAYS)

Sacrifice R3
Sex M
Group 3

	Prostate vent.fresh mg	Seminal vesicle mg	Spleen g	Testes mg	Thyroid glands mg
M	239.12	611.7	0.546	3616.6	23.22
SD	42.61	104.974	0.075	280.736	4.487
n	10	10	10	10	10
731	210.5	617.8	0.45	3470.0	17.1
732	222.4	531.1	0.45	3290.0	27.7
733	344.7	721.5	0.597	4029.0	16.5
734	208.2	553.6	0.582	3991.0	17.8
735	240.1	825.4	0.642	3465.0	27.9
736	200.2	613.6	0.529	3429.0	23.3
737	207.5	626.8	0.605	3475.0	24.6
738	245.9	621.2	0.633	3996.0	25.5
739	258.8	564.6	0.464	3625.0	24.2
740	252.9	441.4	0.508	3396.0	27.6

Reproductive Toxicity Study to detect potential effects to anti-androgenic substances in Wistar Rats (Gavage) 28.Mar.2014 SIGR

ABSOLUTE WEIGHTS - INDIVIDUAL VALUES
SUBSET 3 (POSTNATAL DAY 83 +/- 2 DAYS)

Sacrifice R3
Sex M
Group 4

	Term. body weight g	Adrenal glands mg	Brain g	Bulbo-urethral gland mg	Cauda epididymis mg	Epididymides mg
M	322.73	55.4	1.992	71.91	330.0	914.5
SD	29.78	9.582	0.065	13.308	36.056	52.505
n	10	10	10	10	10	10
741	342.0	59.0	2.06	99.5	410.0	1040.0
742	314.8	59.0	2.09	65.1	320.0	910.0
743	286.8	50.0	1.98	65.1	320.0	880.0
744	359.9	71.0	2.099	72.8	327.0	907.0
745	337.4	42.0	1.95	69.9	340.0	941.0
746	365.7	63.0	1.967	81.3	313.0	929.0
747	323.0	50.0	1.93	84.0	370.0	934.0
748	279.7	49.0	1.935	64.2	295.0	871.0
749	326.1	45.0	1.963	52.0	316.0	867.0
750	291.9	66.0	1.946	65.2	289.0	866.0

Reproductive Toxicity Study to detect potential effects to anti-androgenic substances in Wistar Rats (Gavage) 28.Mar.2014 SIGR

ABSOLUTE WEIGHTS - INDIVIDUAL VALUES
SUBSET 3 (POSTNATAL DAY 83 +/- 2 DAYS)

Sacrifice R3
Sex M
Group 4

	Glans penis mg	Kidneys g	Liver g	Muscles bulb + l.a mg	Pituitary gland mg	Prostate mg
M	85.09	2.223	10.851	674.29	8.77	600.93
SD	6.802	0.176	1.154	111.897	1.046	86.794
n	10	10	10	10	10	10
741	92.2	2.18	11.88	856.7	8.5	570.4
742	81.5	2.05	11.15	687.0	9.3	696.5
743	75.9	1.96	9.3	588.8	8.4	678.2
744	85.6	2.508	12.974	763.4	8.0	727.8
745	85.9	2.395	11.401	661.3	9.7	543.4
746	89.1	2.368	11.088	743.5	11.1	642.4
747	89.4	2.292	11.064	681.6	8.2	633.7
748	94.8	2.031	9.272	475.8	7.7	468.6
749	73.5	2.262	10.479	548.7	7.8	535.6
750	83.0	2.187	9.905	736.1	9.0	512.7

ABSOLUTE WEIGHTS - INDIVIDUAL VALUES
SUBSET 3 (POSTNATAL DAY 83 +/- 2 DAYS)

Sacrifice R3
Sex M
Group 4

	Prostate vent.fresh mg	Seminal vesicle mg	Spleen g	Testes mg	Thyroid glands mg
M	325.95	862.33	0.545	3484.2	21.9
SD	55.203	187.161	0.077	214.774	3.637
n	10	10	10	10	10
741	295.9	808.0	0.51	3630.0	21.6
742	417.9	1009.1	0.5	3300.0	16.5
743	344.7	811.2	0.53	3170.0	22.1
744	389.6	991.7	0.593	3215.0	24.0
745	283.5	877.3	0.572	3642.0	19.4
746	366.2	1090.1	0.741	3532.0	26.0
747	350.5	1034.6	0.494	3785.0	28.9
748	254.7	631.4	0.481	3506.0	18.8
749	280.9	493.4	0.521	3349.0	20.0
750	275.6	876.5	0.507	3713.0	21.7

Reproductive Toxicity Study to detect potential effects to anti-androgenic substances in Wistar Rats (Gavage) 28.Mar.2014 SIGR

ABSOLUTE WEIGHTS - INDIVIDUAL VALUES
SUBSET 3 (POSTNATAL DAY 83 +/- 2 DAYS)

Sacrifice R3
Sex F
Group 0

	Term. body weight g	Adrenal glands mg	Brain g	Kidneys g	Liver g	Ovaries mg
M	196.978	63.556	1.841	1.523	6.577	94.111
SD	14.122	12.126	0.056	0.146	0.829	8.667
n	9	9	9	9	9	9
801	187.1	51.0	1.801	1.371	7.341	91.0
802	201.2	58.0	1.871	1.493	7.209	93.0
803	175.6	51.0	1.833	1.294	6.131	84.0
804	221.3	83.0	1.963	1.75	7.745	103.0
806	203.4	54.0	1.84	1.575	7.407	95.0
807	190.9	59.0	1.768	1.401	5.643	80.0
808	193.2	66.0	1.816	1.644	5.789	95.0
809	212.7	81.0	1.812	1.61	5.883	108.0
810	187.4	69.0	1.867	1.572	6.046	98.0

ABSOLUTE WEIGHTS - INDIVIDUAL VALUES
SUBSET 3 (POSTNATAL DAY 83 +/- 2 DAYS)

Sacrifice R3
Sex F
Group 0

	Pituitary gland mg	Spleen g	Thyroid glands mg	Uterus mg
M	10.333	0.444	18.189	454.778
SD	2.009	0.071	3.747	98.673
n	9	9	9	9
801	9.6	0.369	13.6	369.0
802	8.3	0.451	15.0	384.0
803	8.9	0.332	15.6	331.0
804	10.8	0.526	23.6	617.0
806	14.7	0.41	20.0	548.0
807	8.9	0.397	23.3	422.0
808	9.0	0.495	14.5	381.0
809	11.7	0.49	19.6	525.0
810	11.1	0.528	18.5	516.0

Reproductive Toxicity Study to detect potential effects to anti-androgenic substances in Wistar Rats (Gavage) 28.Mar.2014 SIGR

ABSOLUTE WEIGHTS - INDIVIDUAL VALUES
SUBSET 3 (POSTNATAL DAY 83 +/- 2 DAYS)

Sacrifice R3
Sex F
Group 1

	Term. body weight g	Adrenal glands mg	Brain g	Kidneys g	Liver g	Ovaries mg
M	200.45	59.6	1.891	1.513	6.992	96.6
SD	14.077	6.753	0.089	0.211	0.732	12.747
n	10	10	10	10	10	10
811	225.3	70.0	1.902	2.003	7.517	82.0
812	209.7	58.0	1.858	1.38	7.169	91.0
813	206.2	64.0	1.877	1.521	7.483	110.0
814	174.5	51.0	1.831	1.268	6.192	120.0
815	193.0	49.0	1.892	1.411	5.627	103.0
816	193.7	63.0	1.876	1.353	6.879	88.0
817	209.9	64.0	1.952	1.575	7.979	100.0
818	204.7	65.0	2.061	1.698	7.658	94.0
819	187.6	58.0	1.944	1.43	6.404	78.0
820	199.9	54.0	1.717	1.492	7.016	100.0

Reproductive Toxicity Study to detect potential effects to anti-androgenic substances in Wistar Rats (Gavage)

ABSOLUTE WEIGHTS - INDIVIDUAL VALUES
SUBSET 3 (POSTNATAL DAY 83 +/- 2 DAYS)

Sacrifice R3
Sex F
Group 1

	Pituitary gland mg	Spleen g	Thyroid glands mg	Uterus mg
M	10.64	0.445	17.74	546.0
SD	0.765	0.048	2.95	216.411
n	10	10	10	10
811	11.2	0.434	17.2	883.0
812	11.1	0.477	14.6	562.0
813	10.8	0.458	16.3	397.0
814	10.8	0.415	24.5	703.0
815	9.3	0.371	18.9	356.0
816	11.0	0.434	17.0	389.0
817	11.2	0.519	15.9	446.0
818	11.5	0.512	20.4	425.0
819	9.5	0.389	17.7	372.0
820	10.0	0.437	14.9	927.0

Reproductive Toxicity Study to detect potential effects to anti-androgenic substances in Wistar Rats (Gavage) 28.Mar.2014 SIGR

ABSOLUTE WEIGHTS - INDIVIDUAL VALUES
SUBSET 3 (POSTNATAL DAY 83 +/- 2 DAYS)

Sacrifice R3
Sex F
Group 2

	Term. body weight g	Adrenal glands mg	Brain g	Kidneys g	Liver g	Ovaries mg
M	208.14	66.2	1.862	1.559	7.507	106.0
SD	13.504	9.964	0.074	0.148	0.826	16.892
n	10	10	10	10	10	10
821	200.3	78.0	1.807	1.448	7.088	103.0
822	197.6	64.0	1.855	1.433	7.515	81.0
823	228.3	86.0	1.927	1.751	9.019	113.0
824	200.7	69.0	1.879	1.644	8.066	132.0
825	209.1	61.0	1.9	1.46	7.761	86.0
826	222.8	63.0	1.943	1.729	7.785	114.0
827	184.4	53.0	1.798	1.294	5.774	90.0
828	213.3	69.0	1.918	1.631	7.183	128.0
829	203.4	64.0	1.702	1.546	7.211	105.0
830	221.5	55.0	1.886	1.656	7.671	108.0

Reproductive Toxicity Study to detect potential effects to anti-androgenic substances in Wistar Rats (Gavage) 28.Mar.2014 SIGR

ABSOLUTE WEIGHTS - INDIVIDUAL VALUES
SUBSET 3 (POSTNATAL DAY 83 +/- 2 DAYS)

Sacrifice R3
Sex F
Group 2

	Pituitary gland mg	Spleen g	Thyroid glands mg	Uterus mg	
M	10.91	0.507	20.11	662.445	
SD	1.623	0.12	1.557	266.359	
n	10	10	10	9	
821	12.1	0.444	21.3	560.0	
822	10.9	0.512	20.1	1107.0	
823	11.1	0.798	22.3	798.0	
824	13.0	0.534	18.7	472.0	
825	10.9	0.428	20.9	435.0	
826	13.1	0.619	20.4	943.0	
827	8.2	0.431	16.6	359.0	
828	10.0	0.446	20.4	442.0	
829	8.7	0.436	20.7	281.0	s
830	11.1	0.418	19.7	846.0	

Reproductive Toxicity Study to detect potential effects to anti-androgenic substances in Wistar Rats (Gavage)
28.Mar.2014 SIGR

ABSOLUTE WEIGHTS - INDIVIDUAL VALUES
SUBSET 3 (POSTNATAL DAY 83 +/- 2 DAYS)

Sacrifice R3
Sex F
Group 3

	Term. body weight g	Adrenal glands mg	Brain g	Kidneys g	Liver g	Ovaries mg
M	202.35	67.6	1.784	1.57	7.478	102.2
SD	11.604	7.677	0.058	0.083	0.619	17.035
n	10	10	10	10	10	10
831	185.5	66.0	1.781	1.491	7.775	117.0
832	198.7	74.0	1.854	1.568	8.082	96.0
833	205.4	66.0	1.717	1.499	7.927	97.0
834	206.0	57.0	1.726	1.724	8.062	105.0
835	226.8	76.0	1.744	1.572	7.951	109.0
836	199.1	61.0	1.829	1.539	7.167	91.0
837	207.0	73.0	1.849	1.638	7.789	81.0
838	195.8	70.0	1.737	1.502	6.709	94.0
839	189.5	56.0	1.745	1.491	6.433	91.0
840	209.7	77.0	1.861	1.672	6.881	141.0

ABSOLUTE WEIGHTS - INDIVIDUAL VALUES
SUBSET 3 (POSTNATAL DAY 83 +/- 2 DAYS)

Sacrifice R3
Sex F
Group 3

	Pituitary gland mg	Spleen g	Thyroid glands mg	Uterus mg
M	7.83	0.465	20.91	565.6
SD	1.26	0.059	2.433	236.459
n	10	10	10	10
831	8.5	0.459	20.3	536.0
832	6.7	0.445	25.8	315.0
833	6.4	0.452	21.4	437.0
834	8.3	0.557	22.8	919.0
835	10.5	0.458	18.0	481.0
836	6.9	0.498	20.0	374.0
837	8.1	0.529	17.7	781.0
838	7.9	0.369	20.5	969.0
839	6.5	0.381	19.7	378.0
840	8.5	0.498	22.9	466.0

Reproductive Toxicity Study to detect potential effects to anti-androgenic substances in Wistar Rats (Gavage) 28.Mar.2014 SIGR

ABSOLUTE WEIGHTS - INDIVIDUAL VALUES
SUBSET 3 (POSTNATAL DAY 83 +/- 2 DAYS)

Sacrifice R3
Sex F
Group 4

	Term. body weight g	Adrenal glands mg	Brain g	Kidneys g	Liver g	Ovaries mg
M	204.17	62.8	1.889	1.58	6.772	97.1
SD	16.63	5.865	0.07	0.133	0.67	13.932
n	10	10	10	10	10	10
841	175.0	54.0	1.832	1.271	6.296	89.0
842	208.2	57.0	1.941	1.651	7.345	83.0
843	186.2	64.0	1.978	1.532	7.186	109.0
844	210.3	60.0	1.876	1.616	6.765	105.0
845	221.8	70.0	1.946	1.58	7.976	112.0
846	195.9	63.0	1.886	1.53	6.743	107.0
847	207.7	58.0	1.938	1.509	6.204	72.0
848	224.8	73.0	1.921	1.686	6.545	112.0
849	191.1	66.0	1.759	1.704	5.589	95.0
850	220.7	63.0	1.809	1.721	7.072	87.0

Reproductive Toxicity Study to detect potential effects to anti-androgenic substances in Wistar Rats (Gavage) 28.Mar.2014 SIGR

ABSOLUTE WEIGHTS - INDIVIDUAL VALUES
SUBSET 3 (POSTNATAL DAY 83 +/- 2 DAYS)

Sacrifice R3
Sex F
Group 4

	Pituitary gland mg	Spleen g	Thyroid glands mg	Uterus mg
M	11.16	0.454	19.13	566.2
SD	1.747	0.083	2.079	201.377
n	10	10	10	10
841	8.5	0.524	19.5	865.0
842	10.3	0.487	17.9	415.0
843	12.3	0.462	17.9	562.0
844	10.6	0.362	18.0	557.0
845	10.3	0.436	22.7	399.0
846	11.4	0.393	19.5	455.0
847	10.0	0.643	18.3	488.0
848	14.9	0.418	21.6	562.0
849	10.8	0.373	15.5	379.0
850	12.5	0.445	20.4	980.0

Reproductive Toxicity Study to detect potential effects to anti-androgenic substances in Wistar Rats (Gavage) 28.Mar.2014 SIGR

RELATIVE WEIGHTS - INDIVIDUAL VALUES
SUBSET 3 (POSTNATAL DAY 83 +/- 2 DAYS)

Sacrifice R3
Sex M
Group 0

	Term. body weight %	Adrenal glands %	Brain %	Bulbo-urethral gland %	Cauda epididymis %	Epididy-mides %
M	100.0	0.017	0.618	0.024	0.103	0.285
SD		0.003	0.042	0.006	0.011	0.025
n	9	9	9	9	9	9
701	100.0	0.016	0.603	0.021	0.106	0.281
702	100.0	0.014	0.627	0.038	0.097	0.269
704	100.0	0.023	0.688	0.017	0.12	0.335
705	100.0	0.016	0.615	0.025	0.095	0.265
706	100.0	0.015	0.564	0.024	0.102	0.29
707	100.0	0.016	0.624	0.021	0.089	0.265
708	100.0	0.018	0.678	0.022	0.117	0.3
709	100.0	0.014	0.585	0.02	0.091	0.256
710	100.0	0.02	0.581	0.026	0.111	0.302

Reproductive Toxicity Study to detect potential effects to anti-androgenic substances in Wistar Rats (Gavage) 28.Mar.2014 SIGR

RELATIVE WEIGHTS - INDIVIDUAL VALUES
SUBSET 3 (POSTNATAL DAY 83 +/- 2 DAYS)

Sacrifice R3
Sex M
Group 0

	Glans penis %	Kidneys %	Liver %	Muscles bulb + l.a %	Pituitary gland %	Prostate %
M	0.029	0.692	3.539	0.226	0.003	0.204
SD	0.004	0.033	0.368	0.033	0.0	0.018
n	9	9	9	9	9	9
701	0.028	0.689	3.314	0.204	0.003	0.205
702	0.027	0.705	3.838	0.234	0.003	0.225
704	0.027	0.746	3.788	0.221	0.003	0.215
705	0.026	0.666	3.424	0.198	0.003	0.179
706	0.023	0.72	3.676	0.216	0.003	0.206
707	0.032	0.704	3.453	0.227	0.003	0.198
708	0.039	0.671	3.028	0.31	0.003	0.227
709	0.027	0.633	4.19	0.203	0.002	0.178
710	0.029	0.695	3.14	0.223	0.003	0.204

RELATIVE WEIGHTS - INDIVIDUAL VALUES
SUBSET 3 (POSTNATAL DAY 83 +/- 2 DAYS)

Sacrifice R3
Sex M
Group 0

	Prostate vent.fresh %	Seminal vesicle %	Spleen %	Testes %	Thyroid glands %
M	0.11	0.313	0.17	1.123	0.007
SD	0.016	0.05	0.025	0.114	0.001
n	9	9	9	9	9
701	0.087	0.26	0.148	1.058	0.006
702	0.116	0.392	0.156	1.094	0.007
704	0.129	0.302	0.195	1.359	0.01
705	0.09	0.298	0.148	1.05	0.006
706	0.125	0.35	0.13	1.071	0.007
707	0.098	0.301	0.176	1.041	0.007
708	0.123	0.315	0.178	1.188	0.005
709	0.105	0.236	0.193	1.013	0.006
710	0.12	0.366	0.204	1.236	0.007

Reproductive Toxicity Study to detect potential effects to anti-androgenic substances in Wistar Rats (Gavage) 28.Mar.2014 SIGR

RELATIVE WEIGHTS - INDIVIDUAL VALUES
SUBSET 3 (POSTNATAL DAY 83 +/- 2 DAYS)

Sacrifice R3
Sex M
Group 1

	Term. body weight %	Adrenal glands %	Brain %	Bulbo-uret hral gland %	Cauda epididymis %	Epididy-mides %
M	100.0	0.019	0.653	0.023	0.102	0.294
SD		0.004	0.057	0.003	0.014	0.025
n	10	10	10	10	10	10
711	100.0	0.021	0.748	0.024	0.119	0.331
712	100.0	0.018	0.667	0.025	0.078	0.261
713	100.0	0.014	0.656	0.027	0.095	0.277
714	100.0	0.014	0.536	0.017	0.082	0.261
715	100.0	0.022	0.671	0.023	0.108	0.322
716	100.0	0.019	0.648	0.021	0.11	0.3
717	100.0	0.013	0.649	0.021	0.103	0.287
718	100.0	0.023	0.683	0.02	0.102	0.282
719	100.0	0.018	0.591	0.026	0.107	0.306
720	100.0	0.025	0.686	0.023	0.116	0.317

Reproductive Toxicity Study to detect potential effects to anti-androgenic substances in Wistar Rats (Gavage) 28.Mar.2014 SIGR

RELATIVE WEIGHTS - INDIVIDUAL VALUES
SUBSET 3 (POSTNATAL DAY 83 +/- 2 DAYS)

Sacrifice R3
Sex M
Group 1

	Glans penis %	Kidneys %	Liver %	Muscles bulb + l.a %	Pituitary gland %	Prostate %
M	0.029	0.717	3.43	0.222	0.003	0.198
SD	0.004	0.043	0.159	0.025	0.0	0.017
n	10	10	10	10	10	10
711	0.029	0.737	3.371	0.232	0.003	0.221
712	0.031	0.691	3.122	0.214	0.003	0.178
713	0.027	0.696	3.479	0.214	0.003	0.2
714	0.025	0.695	3.673	0.206	0.003	0.195
715	0.029	0.767	3.321	0.18	0.003	0.183
716	0.028	0.663	3.562	0.237	0.003	0.217
717	0.032	0.743	3.572	0.233	0.003	0.176
718	0.027	0.674	3.482	0.199	0.003	0.19
719	0.025	0.711	3.301	0.237	0.003	0.222
720	0.04	0.797	3.421	0.268	0.003	0.197

RELATIVE WEIGHTS - INDIVIDUAL VALUES
SUBSET 3 (POSTNATAL DAY 83 +/- 2 DAYS)

Sacrifice R3
Sex M
Group 1

	Prostate vent.fresh %	Seminal vesicle %	Spleen %	Testes %	Thyroid glands %
M	0.105	0.305	0.171	1.152	0.007
SD	0.015	0.037	0.02	0.105	0.001
n	10	10	10	10	10
711	0.118	0.336	0.19	1.283	0.006
712	0.091	0.244	0.139	1.029	0.008
713	0.115	0.27	0.182	1.1	0.006
714	0.11	0.274	0.146	1.046	0.008
715	0.111	0.311	0.174	1.353	0.007
716	0.123	0.323	0.195	1.193	0.006
717	0.077	0.362	0.147	1.134	0.008
718	0.091	0.282	0.169	1.092	0.007
719	0.119	0.301	0.18	1.083	0.008
720	0.098	0.345	0.187	1.202	0.008

RELATIVE WEIGHTS - INDIVIDUAL VALUES
SUBSET 3 (POSTNATAL DAY 83 +/- 2 DAYS)

Sacrifice R3
Sex M
Group 2

	Term. body weight %	Adrenal glands %	Brain %	Bulbo-uret hral gland %	Cauda epididymis %	Epididy- mides %
M	100.0	0.016	0.625	0.02	0.093	0.296
SD		0.002	0.058	0.002	0.011	0.075
n	10	10	10	10	10	10
721	100.0	0.016	0.666	0.023	0.086	0.497
722	100.0	0.016	0.654	0.019	0.087	0.264
723	100.0	0.012	0.599	0.019	0.091	0.271
724	100.0	0.018	0.644	0.023	0.088	0.271
725	100.0	0.017	0.604	0.02	0.086	0.276
726	100.0	0.018	0.681	0.022	0.122	0.34
727	100.0	0.019	0.531	0.019	0.084	0.246
728	100.0	0.014	0.53	0.019	0.093	0.248
729	100.0	0.014	0.648	0.018	0.094	0.261
730	100.0	0.018	0.694	0.018	0.096	0.284

Reproductive Toxicity Study to detect potential effects to anti-androgenic substances in Wistar Rats (Gavage)
28.Mar.2014 SIGR

RELATIVE WEIGHTS - INDIVIDUAL VALUES
SUBSET 3 (POSTNATAL DAY 83 +/- 2 DAYS)

Sacrifice R3
Sex M
Group 2

	Glans penis %	Kidneys %	Liver %	Muscles bulb + l.a %	Pituitary gland %	Prostate %
M	0.028	0.738	3.5	0.203	0.003	0.2
SD	0.002	0.08	0.192	0.029	0.0	0.014
n	10	10	10	10	10	10
721	0.026	0.899	3.765	0.189	0.003	0.186
722	0.031	0.781	3.342	0.151	0.003	0.177
723	0.024	0.671	3.431	0.247	0.003	0.216
724	0.026	0.687	3.646	0.182	0.003	0.204
725	0.029	0.721	3.325	0.239	0.003	0.204
726	0.029	0.844	3.651	0.212	0.003	0.203
727	0.03	0.745	3.399	0.22	0.003	0.222
728	0.028	0.701	3.69	0.217	0.003	0.206
729	0.025	0.657	3.573	0.184	0.003	0.19
730	0.031	0.677	3.174	0.192	0.003	0.19

Reproductive Toxicity Study to detect potential effects to anti-androgenic substances in Wistar Rats (Gavage) 28.Mar.2014 SIGR

RELATIVE WEIGHTS - INDIVIDUAL VALUES
SUBSET 3 (POSTNATAL DAY 83 +/- 2 DAYS)

Sacrifice R3
Sex M
Group 2

	Prostate vent.fresh %	Seminal vesicle %	Spleen %	Testes %	Thyroid glands %
M	0.109	0.277	0.171	1.099	0.007
SD	0.01	0.036	0.021	0.146	0.001
n	10	10	10	10	10
721	0.1	0.301	0.155	1.328	0.005
722	0.104	0.247	0.147	1.104	0.006
723	0.128	0.268	0.174	1.014	0.006
724	0.107	0.258	0.174	1.087	0.007
725	0.11	0.267	0.146	1.023	0.006
726	0.101	0.337	0.206	1.352	0.007
727	0.124	0.263	0.176	0.895	0.007
728	0.115	0.227	0.203	1.067	0.008
729	0.111	0.264	0.17	0.967	0.007
730	0.095	0.336	0.162	1.152	0.008

RELATIVE WEIGHTS - INDIVIDUAL VALUES
SUBSET 3 (POSTNATAL DAY 83 +/- 2 DAYS)

Sacrifice R3
Sex M
Group 3

	Term. body weight %	Adrenal glands %	Brain %	Bulbo-urethral gland %	Cauda epididymis %	Epididy-mides %
M	100.0	0.019	0.649	0.014	0.092	0.278
SD		0.002	0.065	0.004	0.01	0.028
n	10	10	10	10	10	10
731	100.0	0.019	0.685	0.011	0.084	0.275
732	100.0	0.017	0.649	0.018	0.091	0.259
733	100.0	0.015	0.654	0.022	0.111	0.321
734	100.0	0.019	0.611	0.013	0.1	0.297
735	100.0	0.018	0.525	0.014	0.076	0.233
736	100.0	0.021	0.729	0.013	0.092	0.298
737	100.0	0.018	0.759	0.012	0.103	0.304
738	100.0	0.022	0.61	0.015	0.088	0.261
739	100.0	0.021	0.629	0.012	0.093	0.283
740	100.0	0.016	0.633	0.01	0.084	0.247

RELATIVE WEIGHTS - INDIVIDUAL VALUES
SUBSET 3 (POSTNATAL DAY 83 +/- 2 DAYS)

Sacrifice R3
Sex M
Group 3

	Glans penis %	Kidneys %	Liver %	Muscles bulb + l.a %	Pituitary gland %	Prostate %
M	0.025	0.741	3.812	0.149	0.003	0.147
SD	0.005	0.039	0.245	0.02	0.0	0.026
n	10	10	10	10	10	10
731	0.024	0.698	4.079	0.156	0.002	0.129
732	0.024	0.73	3.368	0.145	0.003	0.148
733	0.023	0.754	3.571	0.193	0.003	0.217
734	0.021	0.75	3.992	0.129	0.003	0.12
735	0.016	0.706	3.968	0.16	0.002	0.142
736	0.032	0.807	3.671	0.166	0.003	0.157
737	0.025	0.796	3.979	0.143	0.003	0.139
738	0.023	0.71	4.099	0.131	0.003	0.143
739	0.025	0.701	3.663	0.131	0.003	0.144
740	0.034	0.761	3.733	0.139	0.003	0.136

RELATIVE WEIGHTS - INDIVIDUAL VALUES
SUBSET 3 (POSTNATAL DAY 83 +/- 2 DAYS)

Sacrifice R3
Sex M
Group 3

	Prostate vent.fresh %	Seminal vesicle %	Spleen %	Testes %	Thyroid glands %
M	0.08	0.204	0.182	1.207	0.008
SD	0.014	0.033	0.027	0.121	0.002
n	10	10	10	10	10
731	0.071	0.207	0.151	1.165	0.006
732	0.075	0.179	0.151	1.107	0.009
733	0.114	0.239	0.198	1.335	0.005
734	0.066	0.177	0.186	1.275	0.006
735	0.067	0.23	0.179	0.967	0.008
736	0.077	0.237	0.205	1.327	0.009
737	0.081	0.244	0.235	1.352	0.01
738	0.075	0.19	0.194	1.225	0.008
739	0.085	0.185	0.152	1.186	0.008
740	0.084	0.147	0.169	1.129	0.009

RELATIVE WEIGHTS - INDIVIDUAL VALUES
SUBSET 3 (POSTNATAL DAY 83 +/- 2 DAYS)

Sacrifice R3
Sex M
Group 4

	Term. body weight %	Adrenal glands %	Brain %	Bulbo-urethral gland %	Cauda epididymis %	Epididymides %
M	100.0	0.017	0.621	0.022	0.103	0.285
SD		0.003	0.053	0.004	0.011	0.021
n	10	10	10	10	10	10
741	100.0	0.017	0.602	0.029	0.12	0.304
742	100.0	0.019	0.664	0.021	0.102	0.289
743	100.0	0.017	0.69	0.023	0.112	0.307
744	100.0	0.02	0.583	0.02	0.091	0.252
745	100.0	0.012	0.578	0.021	0.101	0.279
746	100.0	0.017	0.538	0.022	0.086	0.254
747	100.0	0.015	0.598	0.026	0.115	0.289
748	100.0	0.018	0.692	0.023	0.105	0.311
749	100.0	0.014	0.602	0.016	0.097	0.266
750	100.0	0.023	0.667	0.022	0.099	0.297

Reproductive Toxicity Study to detect potential effects to anti-androgenic substances in Wistar Rats (Gavage) 28.Mar.2014 SIGR

RELATIVE WEIGHTS - INDIVIDUAL VALUES
SUBSET 3 (POSTNATAL DAY 83 +/- 2 DAYS)

Sacrifice R3
Sex M
Group 4

	Glans penis %	Kidneys %	Liver %	Muscles bulb + l.a %	Pituitary gland %	Prostate %
M	0.027	0.69	3.362	0.209	0.003	0.187
SD	0.003	0.036	0.169	0.028	0.0	0.026
n	10	10	10	10	10	10
741	0.027	0.637	3.474	0.25	0.002	0.167
742	0.026	0.651	3.542	0.218	0.003	0.221
743	0.026	0.683	3.243	0.205	0.003	0.236
744	0.024	0.697	3.605	0.212	0.002	0.202
745	0.025	0.71	3.379	0.196	0.003	0.161
746	0.024	0.648	3.032	0.203	0.003	0.176
747	0.028	0.71	3.425	0.211	0.003	0.196
748	0.034	0.726	3.315	0.17	0.003	0.168
749	0.023	0.694	3.213	0.168	0.002	0.164
750	0.028	0.749	3.393	0.252	0.003	0.176

RELATIVE WEIGHTS - INDIVIDUAL VALUES
SUBSET 3 (POSTNATAL DAY 83 +/- 2 DAYS)

Sacrifice R3
Sex M
Group 4

	Prostate vent.fresh %	Seminal vesicle %	Spleen %	Testes %	Thyroid glands %
M	0.101	0.267	0.169	1.088	0.007
SD	0.016	0.052	0.016	0.119	0.001
n	10	10	10	10	10
741	0.087	0.236	0.149	1.061	0.006
742	0.133	0.321	0.159	1.048	0.005
743	0.12	0.283	0.185	1.105	0.008
744	0.108	0.276	0.165	0.893	0.007
745	0.084	0.26	0.17	1.079	0.006
746	0.1	0.298	0.203	0.966	0.007
747	0.109	0.32	0.153	1.172	0.009
748	0.091	0.226	0.172	1.253	0.007
749	0.086	0.151	0.16	1.027	0.006
750	0.094	0.3	0.174	1.272	0.007

RELATIVE WEIGHTS - INDIVIDUAL VALUES
SUBSET 3 (POSTNATAL DAY 83 +/- 2 DAYS)

Sacrifice R3
Sex F
Group 0

	Term. body weight %	Adrenal glands %	Brain %	Kidneys %	Liver %	Ovaries %
M	100.0	0.032	0.938	0.773	3.343	0.048
SD		0.005	0.058	0.045	0.379	0.003
n	9	9	9	9	9	9
801	100.0	0.027	0.963	0.733	3.924	0.049
802	100.0	0.029	0.93	0.742	3.583	0.046
803	100.0	0.029	1.044	0.737	3.491	0.048
804	100.0	0.038	0.887	0.791	3.5	0.047
806	100.0	0.027	0.905	0.774	3.642	0.047
807	100.0	0.031	0.926	0.734	2.956	0.042
808	100.0	0.034	0.94	0.851	2.996	0.049
809	100.0	0.038	0.852	0.757	2.766	0.051
810	100.0	0.037	0.996	0.839	3.226	0.052

RELATIVE WEIGHTS - INDIVIDUAL VALUES
SUBSET 3 (POSTNATAL DAY 83 +/- 2 DAYS)

Sacrifice R3
Sex F
Group 0

	Pituitary gland %	Spleen %	Thyroid glands %	Uterus %
M	0.005	0.225	0.009	0.229
SD	0.001	0.03	0.002	0.038
n	9	9	9	9
801	0.005	0.197	0.007	0.197
802	0.004	0.224	0.007	0.191
803	0.005	0.189	0.009	0.188
804	0.005	0.238	0.011	0.279
806	0.007	0.202	0.01	0.269
807	0.005	0.208	0.012	0.221
808	0.005	0.256	0.008	0.197
809	0.006	0.23	0.009	0.247
810	0.006	0.282	0.01	0.275

Reproductive Toxicity Study to detect potential effects to anti-androgenic substances in Wistar Rats (Gavage) 28.Mar.2014 SIGR

RELATIVE WEIGHTS - INDIVIDUAL VALUES
SUBSET 3 (POSTNATAL DAY 83 +/- 2 DAYS)

Sacrifice R3
Sex F
Group 1

	Term. body weight %	Adrenal glands %	Brain %	Kidneys %	Liver %	Ovaries %
M	100.0	0.03	0.947	0.753	3.487	0.049
SD		0.002	0.072	0.065	0.247	0.009
n	10	10	10	10	10	10
811	100.0	0.031	0.844	0.889	3.336	0.036
812	100.0	0.028	0.886	0.658	3.419	0.043
813	100.0	0.031	0.91	0.738	3.629	0.053
814	100.0	0.029	1.049	0.727	3.548	0.069
815	100.0	0.025	0.98	0.731	2.916	0.053
816	100.0	0.033	0.969	0.699	3.551	0.045
817	100.0	0.03	0.93	0.75	3.801	0.048
818	100.0	0.032	1.007	0.83	3.741	0.046
819	100.0	0.031	1.036	0.762	3.414	0.042
820	100.0	0.027	0.859	0.746	3.51	0.05

RELATIVE WEIGHTS - INDIVIDUAL VALUES
SUBSET 3 (POSTNATAL DAY 83 +/- 2 DAYS)

Sacrifice R3
Sex F
Group 1

	Pituitary gland %	Spleen %	Thyroid glands %	Uterus %
M	0.005	0.222	0.009	0.272
SD	0.0	0.02	0.002	0.106
n	10	10	10	10
811	0.005	0.193	0.008	0.392
812	0.005	0.227	0.007	0.268
813	0.005	0.222	0.008	0.193
814	0.006	0.238	0.014	0.403
815	0.005	0.192	0.01	0.184
816	0.006	0.224	0.009	0.201
817	0.005	0.247	0.008	0.212
818	0.006	0.25	0.01	0.208
819	0.005	0.207	0.009	0.198
820	0.005	0.219	0.007	0.464

Reproductive Toxicity Study to detect potential effects to anti-androgenic substances in Wistar Rats (Gavage)

28.Mar.2014 SIGR

RELATIVE WEIGHTS - INDIVIDUAL VALUES
SUBSET 3 (POSTNATAL DAY 83 +/- 2 DAYS)

Sacrifice R3
Sex F
Group 2

	Term. body weight %	Adrenal glands %	Brain %	Kidneys %	Liver %	Ovaries %
M	100.0	0.032	0.896	0.748	3.602	0.051
SD		0.004	0.046	0.037	0.271	0.008
n	10	10	10	10	10	10
821	100.0	0.039	0.902	0.723	3.539	0.051
822	100.0	0.032	0.939	0.725	3.803	0.041
823	100.0	0.038	0.844	0.767	3.951	0.049
824	100.0	0.034	0.936	0.819	4.019	0.066
825	100.0	0.029	0.909	0.698	3.712	0.041
826	100.0	0.028	0.872	0.776	3.494	0.051
827	100.0	0.029	0.975	0.702	3.131	0.049
828	100.0	0.032	0.899	0.765	3.368	0.06
829	100.0	0.031	0.837	0.76	3.545	0.052
830	100.0	0.025	0.851	0.748	3.463	0.049

Reproductive Toxicity Study to detect potential effects to anti-androgenic substances in Wistar Rats (Gavage) 28.Mar.2014 SIGR

RELATIVE WEIGHTS - INDIVIDUAL VALUES
SUBSET 3 (POSTNATAL DAY 83 +/- 2 DAYS)

Sacrifice R3
Sex F
Group 2

	Pituitary gland %	Spleen %	Thyroid glands %	Uterus %
M	0.005	0.242	0.01	0.316
SD	0.001	0.047	0.001	0.124
n	10	10	10	9
821	0.006	0.222	0.011	0.28
822	0.006	0.259	0.01	0.56
823	0.005	0.35	0.01	0.35
824	0.006	0.266	0.009	0.235
825	0.005	0.205	0.01	0.208
826	0.006	0.278	0.009	0.423
827	0.004	0.234	0.009	0.195
828	0.005	0.209	0.01	0.207
829	0.004	0.214	0.01	0.138s
830	0.005	0.189	0.009	0.382

Reproductive Toxicity Study to detect potential effects to anti-androgenic substances in Wistar Rats (Gavage) 28.Mar.2014 SIGR

RELATIVE WEIGHTS - INDIVIDUAL VALUES
SUBSET 3 (POSTNATAL DAY 83 +/- 2 DAYS)

Sacrifice R3
Sex F
Group 3

	Term. body weight %	Adrenal glands %	Brain %	Kidneys %	Liver %	Ovaries %
M	100.0	0.033	0.884	0.777	3.7	0.051
SD		0.003	0.056	0.04	0.306	0.008
n	10	10	10	10	10	10
831	100.0	0.036	0.96	0.804	4.191	0.063
832	100.0	0.037	0.933	0.789	4.067	0.048
833	100.0	0.032	0.836	0.73	3.859	0.047
834	100.0	0.028	0.838	0.837	3.914	0.051
835	100.0	0.034	0.769	0.693	3.506	0.048
836	100.0	0.031	0.919	0.773	3.6	0.046
837	100.0	0.035	0.893	0.791	3.763	0.039
838	100.0	0.036	0.887	0.767	3.426	0.048
839	100.0	0.03	0.921	0.787	3.395	0.048
840	100.0	0.037	0.887	0.797	3.281	0.067

Reproductive Toxicity Study to detect potential effects to anti-androgenic substances in Wistar Rats (Gavage) 28.Mar.2014 SIGR

RELATIVE WEIGHTS - INDIVIDUAL VALUES
SUBSET 3 (POSTNATAL DAY 83 +/- 2 DAYS)

Sacrifice R3
Sex F
Group 3

	Pituitary gland %	Spleen %	Thyroid glands %	Uterus %
M	0.004	0.23	0.01	0.28
SD	0.001	0.027	0.001	0.118
n	10	10	10	10
831	0.005	0.247	0.011	0.289
832	0.003	0.224	0.013	0.159
833	0.003	0.22	0.01	0.213
834	0.004	0.27	0.011	0.446
835	0.005	0.202	0.008	0.212
836	0.003	0.25	0.01	0.188
837	0.004	0.256	0.009	0.377
838	0.004	0.188	0.01	0.495
839	0.003	0.201	0.01	0.199
840	0.004	0.237	0.011	0.222

RELATIVE WEIGHTS - INDIVIDUAL VALUES
SUBSET 3 (POSTNATAL DAY 83 +/- 2 DAYS)

Sacrifice R3
Sex F
Group 4

	Term. body weight %	Adrenal glands %	Brain %	Kidneys %	Liver %	Ovaries %
M	100.0	0.031	0.93	0.775	3.327	0.048
SD		0.003	0.078	0.053	0.327	0.007
n	10	10	10	10	10	10
841	100.0	0.031	1.047	0.726	3.598	0.051
842	100.0	0.027	0.932	0.793	3.528	0.04
843	100.0	0.034	1.062	0.823	3.859	0.059
844	100.0	0.029	0.892	0.768	3.217	0.05
845	100.0	0.032	0.877	0.712	3.596	0.05
846	100.0	0.032	0.963	0.781	3.442	0.055
847	100.0	0.028	0.933	0.727	2.987	0.035
848	100.0	0.032	0.855	0.75	2.911	0.05
849	100.0	0.035	0.92	0.892	2.925	0.05
850	100.0	0.029	0.82	0.78	3.204	0.039

RELATIVE WEIGHTS - INDIVIDUAL VALUES
SUBSET 3 (POSTNATAL DAY 83 +/- 2 DAYS)

Sacrifice R3
Sex F
Group 4

	Pituitary gland %	Spleen %	Thyroid glands %	Uterus %
M	0.005	0.224	0.009	0.28
SD	0.001	0.048	0.001	0.106
n	10	10	10	10
841	0.005	0.299	0.011	0.494
842	0.005	0.234	0.009	0.199
843	0.007	0.248	0.01	0.302
844	0.005	0.172	0.009	0.265
845	0.005	0.197	0.01	0.18
846	0.006	0.201	0.01	0.232
847	0.005	0.31	0.009	0.235
848	0.007	0.186	0.01	0.25
849	0.006	0.195	0.008	0.198
850	0.006	0.202	0.009	0.444

SINGLE ANIMAL SHEET
(GROSS LESIONS AND MICROSCOPIC FINDINGS)

Sacrifice	F1
Sex	F
Group	0
Animal	1

General information
Sex : Female
Group : 0 (0 mg/kg)
Sacrifice : Final sacrifice group
Necropsy status : Planned sacrifice
Date of death : 06.May.2013
64 days after start of exposure
1 day after end of exposure

Macroscopic findings
Animal without particular findings.

Microscopic findings
No histologic examination performed.

Animal 2

General information
Sex : Female
Group : 0 (0 mg/kg)
Sacrifice : Final sacrifice group
Necropsy status : Planned sacrifice
Date of death : 06.May.2013
64 days after start of exposure
1 day after end of exposure

Macroscopic findings
Animal without particular findings.

Microscopic findings
No histologic examination performed.

Animal 3

General information
Sex : Female
Group : 0 (0 mg/kg)
Sacrifice : Final sacrifice group
Necropsy status : Planned sacrifice
Date of death : 06.May.2013
64 days after start of exposure
1 day after end of exposure

SINGLE ANIMAL SHEET
(GROSS LESIONS AND MICROSCOPIC FINDINGS)

Sacrifice F1
Sex F
Group 0
cont. Animal 3

Macroscopic findings
Animal without particular findings.

Microscopic findings
No histologic examination performed.

Animal 4

General information
Sex : Female
Group : 0 (0 mg/kg)
Sacrifice : Final sacrifice group
Necropsy status : Planned sacrifice
Date of death : 06.May.2013
64 days after start of exposure
1 day after end of exposure

Macroscopic findings
Animal without particular findings.

Microscopic findings
No histologic examination performed.

Animal 5

General information
Sex : Female
Group : 0 (0 mg/kg)
Sacrifice : Final sacrifice group
Necropsy status : Planned sacrifice
Date of death : 06.May.2013
64 days after start of exposure
1 day after end of exposure

Macroscopic findings
Animal without particular findings.

Microscopic findings
No histologic examination performed.

Animal 6

General information
Sex : Female
Group : 0 (0 mg/kg)
Sacrifice : Final sacrifice group
Necropsy status : Planned sacrifice
Date of death : 06.May.2013
64 days after start of exposure

SINGLE ANIMAL SHEET
(GROSS LESIONS AND MICROSCOPIC FINDINGS)

Sacrifice F1
Sex F
Group 0
cont. Animal 6

1 day after end of exposure

Macroscopic findings
Animal without particular findings.

Microscopic findings
No histologic examination performed.

Animal 7

General information
Sex : Female
Group : 0 (0 mg/kg)
Sacrifice : Final sacrifice group
Necropsy status : Planned sacrifice
Date of death : 06.May.2013
64 days after start of exposure
1 day after end of exposure

Macroscopic findings
Animal without particular findings.

Microscopic findings
No histologic examination performed.

Animal 8

General information
Sex : Female
Group : 0 (0 mg/kg)
Sacrifice : Final sacrifice group
Necropsy status : Planned sacrifice
Date of death : 06.May.2013
63 days after start of exposure
1 day after end of exposure

Macroscopic findings
Animal without particular findings.

Microscopic findings
No histologic examination performed.

SINGLE ANIMAL SHEET
(GROSS LESIONS AND MICROSCOPIC FINDINGS)

Sacrifice F1
Sex F
Group 0
Animal 9

General information
Sex : Female
Group : 0 (0 mg/kg)
Sacrifice : Final sacrifice group
Necropsy status : Planned sacrifice
Date of death : 06.May.2013
63 days after start of exposure
1 day after end of exposure

Macroscopic findings
Animal without particular findings.

Microscopic findings
No histologic examination performed.

Animal 10

General information
Sex : Female
Group : 0 (0 mg/kg)
Sacrifice : Final sacrifice group
Necropsy status : Planned sacrifice
Date of death : 06.May.2013
63 days after start of exposure
1 day after end of exposure

Macroscopic findings
Animal without particular findings.

Microscopic findings
No histologic examination performed.

Animal 11

General information
Sex : Female
Group : 0 (0 mg/kg)
Sacrifice : Final sacrifice group
Necropsy status : Planned sacrifice
Date of death : 10.May.2013
67 days after start of exposure
1 day after end of exposure

SINGLE ANIMAL SHEET
(GROSS LESIONS AND MICROSCOPIC FINDINGS)

Sacrifice F1
Sex F
Group 0
cont. Animal 11

Macroscopic findings
Animal without particular findings.

Microscopic findings
No histologic examination performed.

Animal 17

General information
Sex : Female
Group : 0 (0 mg/kg)
Sacrifice : Final sacrifice group
Necropsy status : Planned sacrifice
Date of death : 10.May.2013
66 days after start of exposure
1 day after end of exposure

Macroscopic findings
Animal without particular findings.

Microscopic findings
No histologic examination performed.

Animal 18

General information
Sex : Female
Group : 0 (0 mg/kg)
Sacrifice : Final sacrifice group
Necropsy status : Planned sacrifice
Date of death : 10.May.2013
66 days after start of exposure
1 day after end of exposure

Macroscopic findings
Animal without particular findings.

Microscopic findings
No histologic examination performed.

Animal 19

General information
Sex : Female
Group : 0 (0 mg/kg)
Sacrifice : Final sacrifice group
Necropsy status : Planned sacrifice
Date of death : 10.May.2013
66 days after start of exposure

SINGLE ANIMAL SHEET
(GROSS LESIONS AND MICROSCOPIC FINDINGS)

Sacrifice F1
Sex F
Group 0
cont. Animal 19

1 day after end of exposure

Macroscopic findings
Animal without particular findings.

Microscopic findings
No histologic examination performed.

Animal 20

General information
Sex : Female
Group : 0 (0 mg/kg)
Sacrifice : Final sacrifice group
Necropsy status : Planned sacrifice
Date of death : 10.May.2013
66 days after start of exposure
1 day after end of exposure

Macroscopic findings
Animal without particular findings.

Microscopic findings
No histologic examination performed.

Animal 21

General information
Sex : Female
Group : 0 (0 mg/kg)
Sacrifice : Final sacrifice group
Necropsy status : Planned sacrifice
Date of death : 10.May.2013
66 days after start of exposure
1 day after end of exposure

Macroscopic findings
Animal without particular findings.

Microscopic findings
No histologic examination performed.

SINGLE ANIMAL SHEET
(GROSS LESIONS AND MICROSCOPIC FINDINGS)

Sacrifice F1
Sex F
Group 0
Animal 22

General information
Sex : Female
Group : 0 (0 mg/kg)
Sacrifice : Final sacrifice group
Necropsy status : Planned sacrifice
Date of death : 10.May.2013
66 days after start of exposure
1 day after end of exposure

Macroscopic findings
Animal without particular findings.

Microscopic findings
No histologic examination performed.

Animal 23

General information
Sex : Female
Group : 0 (0 mg/kg)
Sacrifice : Final sacrifice group
Necropsy status : Planned sacrifice
Date of death : 10.May.2013
65 days after start of exposure
1 day after end of exposure

Macroscopic findings
Animal without particular findings.

Microscopic findings
No histologic examination performed.

Animal 24

General information
Sex : Female
Group : 0 (0 mg/kg)
Sacrifice : Final sacrifice group
Necropsy status : Planned sacrifice
Date of death : 10.May.2013
65 days after start of exposure
1 day after end of exposure

SINGLE ANIMAL SHEET
(GROSS LESIONS AND MICROSCOPIC FINDINGS)

Sacrifice	F1
Sex	F
Group	0
cont. Animal	24

Macroscopic findings
Animal without particular findings.

Microscopic findings
No histologic examination performed.

Animal 25

General information
Sex : Female
Group : 0 (0 mg/kg)
Sacrifice : Final sacrifice group
Necropsy status : Planned sacrifice
Date of death : 10.May.2013
65 days after start of exposure
1 day after end of exposure

Macroscopic findings
Animal without particular findings.

Microscopic findings
No histologic examination performed.

SINGLE ANIMAL SHEET
(GROSS LESIONS AND MICROSCOPIC FINDINGS)

Sacrifice F1
Sex F
Group 1
Animal 26

General information
Sex : Female
Group : 1 (0.005RON/0.00025FLT/0.01 590F mg/kg)
Sacrifice : Final sacrifice group
Necropsy status : Planned sacrifice
Date of death : 06.May.2013
64 days after start of exposure
1 day after end of exposure

Macroscopic findings
Animal without particular findings.

Microscopic findings
No histologic examination performed.

Animal 27

General information
Sex : Female
Group : 1 (0.005RON/0.00025FLT/0.01 590F mg/kg)
Sacrifice : Final sacrifice group
Necropsy status : Planned sacrifice
Date of death : 06.May.2013
64 days after start of exposure
1 day after end of exposure

Macroscopic findings
Ovaries
Cyst, right side, diameter 2.0 mm.
All other organs without macroscopic findings.

Microscopic findings
Ovaries
Histopathologic evaluation of gross lesion(s) will not be performed.
No histologic examination performed.

Animal 28

General information
Sex : Female
Group : 1 (0.005RON/0.00025FLT/0.01 590F mg/kg)
Sacrifice : Final sacrifice group
Necropsy status : Spontaneous death
Date of death : 20.Apr.2013
48 days after start of exposure
1 day after end of exposure

SINGLE ANIMAL SHEET
(GROSS LESIONS AND MICROSCOPIC FINDINGS)

Sacrifice F1
Sex F
Group 1
cont. Animal 28

Macroscopic findings
  Abdominal cavity
    Effusion, content 5.0 ml bloody fluid.
  Liver
    Torsion, papillary process unilateral, diameter 18.0 mm, dark red.
  All other organs without macroscopic findings.

Microscopic findings
  Abdominal cavity
    Histopathologic evaluation of gross lesion(s) will not be performed.
  Liver
    Histopathologic evaluation of gross lesion(s) will not be performed.
  No histologic examination performed.

Animal 29

General information
  Sex : Female
  Group : 1 (0.005RON/0.00025FLT/0.01 590F mg/kg)
  Sacrifice : Final sacrifice group
  Necropsy status : Planned sacrifice
  Date of death : 06.May.2013
    64 days after start of exposure
    1 day after end of exposure

Macroscopic findings
  Animal without particular findings.

Microscopic findings
  No histologic examination performed.

Animal 30

General information
  Sex : Female
  Group : 1 (0.005RON/0.00025FLT/0.01 590F mg/kg)
  Sacrifice : Final sacrifice group
  Necropsy status : Planned sacrifice
  Date of death : 06.May.2013
    64 days after start of exposure
    1 day after end of exposure

SINGLE ANIMAL SHEET
(GROSS LESIONS AND MICROSCOPIC FINDINGS)

Sacrifice F1
Sex F
Group 1
cont. Animal 30

Macroscopic findings
Animal without particular findings.

Microscopic findings
No histologic examination performed.

Animal 31

General information
Sex : Female
Group : 1 (0.005RON/0.00025FLT/0.01 590F mg/kg)
Sacrifice : Final sacrifice group
Necropsy status : Planned sacrifice
Date of death : 06.May.2013
33 days after start of exposure
1 day after end of exposure

Macroscopic findings
Animal without particular findings.

Microscopic findings
No histologic examination performed.

Animal 32

General information
Sex : Female
Group : 1 (0.005RON/0.00025FLT/0.01 590F mg/kg)
Sacrifice : Final sacrifice group
Necropsy status : Planned sacrifice
Date of death : 06.May.2013
33 days after start of exposure
1 day after end of exposure

Macroscopic findings
Animal without particular findings.

Microscopic findings
No histologic examination performed.

Animal 33

General information
Sex : Female
Group : 1 (0.005RON/0.00025FLT/0.01 590F mg/kg)
Sacrifice : Final sacrifice group
Necropsy status : Planned sacrifice
Date of death : 06.May.2013
63 days after start of exposure

SINGLE ANIMAL SHEET
(GROSS LESIONS AND MICROSCOPIC FINDINGS)

Sacrifice F1
Sex F
Group 1
cont. Animal 33

1 day after end of exposure

Macroscopic findings
Animal without particular findings.

Microscopic findings
No histologic examination performed.

Animal 34

General information
Sex : Female
Group : 1 (0.005RON/0.00025FLT/0.01 590F mg/kg)
Sacrifice : Final sacrifice group
Necropsy status : Planned sacrifice
Date of death : 06.May.2013
63 days after start of exposure
1 day after end of exposure

Macroscopic findings
Animal without particular findings.

Microscopic findings
No histologic examination performed.

Animal 35

General information
Sex : Female
Group : 1 (0.005RON/0.00025FLT/0.01 590F mg/kg)
Sacrifice : Final sacrifice group
Necropsy status : Planned sacrifice
Date of death : 06.May.2013
63 days after start of exposure
1 day after end of exposure

Macroscopic findings
Animal without particular findings.

Microscopic findings
No histologic examination performed.

SINGLE ANIMAL SHEET
(GROSS LESIONS AND MICROSCOPIC FINDINGS)

Sacrifice F1
Sex F
Group 1
Animal 36

General information
Sex : Female
Group : 1 (0.005RON/0.00025FLT/0.01 590F mg/kg)
Sacrifice : Final sacrifice group
Necropsy status : Planned sacrifice
Date of death : 10.May.2013
67 days after start of exposure
1 day after end of exposure

Macroscopic findings
Animal without particular findings.

Microscopic findings
No histologic examination performed.

Animal 37

General information
Sex : Female
Group : 1 (0.005RON/0.00025FLT/0.01 590F mg/kg)
Sacrifice : Final sacrifice group
Necropsy status : Planned sacrifice
Date of death : 10.May.2013
67 days after start of exposure
1 day after end of exposure

Macroscopic findings
Animal without particular findings.

Microscopic findings
No histologic examination performed.

Animal 43

General information
Sex : Female
Group : 1 (0.005RON/0.00025FLT/0.01 590F mg/kg)
Sacrifice : Final sacrifice group
Necropsy status : Planned sacrifice
Date of death : 10.May.2013
66 days after start of exposure
1 day after end of exposure

SINGLE ANIMAL SHEET
(GROSS LESIONS AND MICROSCOPIC FINDINGS)

Sacrifice F1
Sex F
Group 1
cont. Animal 43

Macroscopic findings
  Animal without particular findings.

Microscopic findings
  No histologic examination performed.

Animal 44

General information
  Sex : Female
  Group : 1 (0.005RON/0.00025FLT/0.01 590F mg/kg)
  Sacrifice : Final sacrifice group
  Necropsy status : Planned sacrifice
  Date of death : 10.May.2013
  66 days after start of exposure
  1 day after end of exposure

Macroscopic findings
  Animal without particular findings.

Microscopic findings
  No histologic examination performed.

Animal 45

General information
  Sex : Female
  Group : 1 (0.005RON/0.00025FLT/0.01 590F mg/kg)
  Sacrifice : Final sacrifice group
  Necropsy status : Planned sacrifice
  Date of death : 10.May.2013
  66 days after start of exposure
  1 day after end of exposure

Macroscopic findings
  Animal without particular findings.

Microscopic findings
  No histologic examination performed.

Animal 46

General information
  Sex : Female
  Group : 1 (0.005RON/0.00025FLT/0.01 590F mg/kg)
  Sacrifice : Final sacrifice group
  Necropsy status : Planned sacrifice
  Date of death : 10.May.2013
  66 days after start of exposure

SINGLE ANIMAL SHEET
(GROSS LESIONS AND MICROSCOPIC FINDINGS)

Sacrifice F1
Sex F
Group 1
cont. Animal 46

1 day after end of exposure

Macroscopic findings
Animal without particular findings.

Microscopic findings
No histologic examination performed.

Animal 47

General information
Sex : Female
Group : 1 (0.005RON/0.00025FLT/0.01 590F mg/kg)
Sacrifice : Final sacrifice group
Necropsy status : Planned sacrifice
Date of death : 10.May.2013
66 days after start of exposure
1 day after end of exposure

Macroscopic findings
Animal without particular findings.

Microscopic findings
No histologic examination performed.

Animal 48

General information
Sex : Female
Group : 1 (0.005RON/0.00025FLT/0.01 590F mg/kg)
Sacrifice : Final sacrifice group
Necropsy status : Planned sacrifice
Date of death : 10.May.2013
65 days after start of exposure
1 day after end of exposure

Macroscopic findings
Animal without particular findings.

Microscopic findings
No histologic examination performed.

SINGLE ANIMAL SHEET
(GROSS LESIONS AND MICROSCOPIC FINDINGS)

Sacrifice F1
Sex F
Group 1
Animal 49

General information
Sex : Female
Group : 1 (0.005RON/0.00025FLT/0.01 590F mg/kg)
Sacrifice : Final sacrifice group
Necropsy status : Planned sacrifice
Date of death : 10.May.2013
65 days after start of exposure
1 day after end of exposure

Macroscopic findings
Animal without particular findings.

Microscopic findings
No histologic examination performed.

Animal 50

General information
Sex : Female
Group : 1 (0.005RON/0.00025FLT/0.01 590F mg/kg)
Sacrifice : Final sacrifice group
Necropsy status : Planned sacrifice
Date of death : 10.May.2013
65 days after start of exposure
1 day after end of exposure

Macroscopic findings
Animal without particular findings.

Microscopic findings
No histologic examination performed.

SINGLE ANIMAL SHEET
(GROSS LESIONS AND MICROSCOPIC FINDINGS)

Sacrifice F1
Sex F
Group 2
Animal 51

General information
Sex : Female
Group : 2 (4 RON/ 0.025 FLT/ 5 590F mg/kg)
Sacrifice : Final sacrifice group
Necropsy status : Planned sacrifice
Date of death : 06.May.2013
64 days after start of exposure
1 day after end of exposure

Macroscopic findings
Animal without particular findings.

Microscopic findings
No histologic examination performed.

Animal 52

General information
Sex : Female
Group : 2 (4 RON/ 0.025 FLT/ 5 590F mg/kg)
Sacrifice : Final sacrifice group
Necropsy status : Planned sacrifice
Date of death : 06.May.2013
64 days after start of exposure
1 day after end of exposure

Macroscopic findings
Animal without particular findings.

Microscopic findings
No histologic examination performed.

Animal 53

General information
Sex : Female
Group : 2 (4 RON/ 0.025 FLT/ 5 590F mg/kg)
Sacrifice : Final sacrifice group
Necropsy status : Planned sacrifice
Date of death : 06.May.2013
64 days after start of exposure
1 day after end of exposure

SINGLE ANIMAL SHEET
(GROSS LESIONS AND MICROSCOPIC FINDINGS)

Sacrifice F1
Sex F
Group 2
cont. Animal 53

Macroscopic findings
Animal without particular findings.

Microscopic findings
No histologic examination performed.

Animal 54

General information
Sex : Female
Group : 2 (4 RON/ 0.025 FLT/ 5 590F mg/kg)
Sacrifice : Final sacrifice group
Necropsy status : Planned sacrifice
Date of death : 06.May.2013
64 days after start of exposure
1 day after end of exposure

Macroscopic findings
Animal without particular findings.

Microscopic findings
No histologic examination performed.

Animal 55

General information
Sex : Female
Group : 2 (4 RON/ 0.025 FLT/ 5 590F mg/kg)
Sacrifice : Final sacrifice group
Necropsy status : Planned sacrifice
Date of death : 06.May.2013
64 days after start of exposure
1 day after end of exposure

Macroscopic findings
Animal without particular findings.

Microscopic findings
No histologic examination performed.

Animal 56

General information
Sex : Female
Group : 2 (4 RON/ 0.025 FLT/ 5 590F mg/kg)
Sacrifice : Final sacrifice group
Necropsy status : Planned sacrifice
Date of death : 06.May.2013
64 days after start of exposure

SINGLE ANIMAL SHEET
(GROSS LESIONS AND MICROSCOPIC FINDINGS)

Sacrifice F1
Sex F
Group 2
cont. Animal 56

1 day after end of exposure

Macroscopic findings
Animal without particular findings.

Microscopic findings
No histologic examination performed.

Animal 57

General information
Sex : Female
Group : 2 (4 RON/ 0.025 FLT/ 5 590F mg/kg)
Sacrifice : Final sacrifice group
Necropsy status : Planned sacrifice
Date of death : 06.May.2013
64 days after start of exposure
1 day after end of exposure

Macroscopic findings
Animal without particular findings.

Microscopic findings
No histologic examination performed.

Animal 58

General information
Sex : Female
Group : 2 (4 RON/ 0.025 FLT/ 5 590F mg/kg)
Sacrifice : Final sacrifice group
Necropsy status : Planned sacrifice
Date of death : 06.May.2013
63 days after start of exposure
1 day after end of exposure

Macroscopic findings
Animal without particular findings.

Microscopic findings
No histologic examination performed.

Reproductive Toxicity Study to detect potential effects to anti-androgenic substances in Wistar Rats (Gavage) 28.Mar.2014 SIGR

SINGLE ANIMAL SHEET
(GROSS LESIONS AND MICROSCOPIC FINDINGS)

Sacrifice F1
Sex F
Group 2
Animal 59

General information
Sex : Female
Group : 2 (4 RON/ 0.025 FLT/ 5 590F mg/kg)
Sacrifice : Final sacrifice group
Necropsy status : Planned sacrifice
Date of death : 06.May.2013
63 days after start of exposure
1 day after end of exposure

Macroscopic findings
Animal without particular findings.

Microscopic findings
No histologic examination performed.

Animal 60

General information
Sex : Female
Group : 2 (4 RON/ 0.025 FLT/ 5 590F mg/kg)
Sacrifice : Final sacrifice group
Necropsy status : Planned sacrifice
Date of death : 06.May.2013
63 days after start of exposure
1 day after end of exposure

Macroscopic findings
Animal without particular findings.

Microscopic findings
No histologic examination performed.

Animal 61

General information
Sex : Female
Group : 2 (4 RON/ 0.025 FLT/ 5 590F mg/kg)
Sacrifice : Final sacrifice group
Necropsy status : Planned sacrifice
Date of death : 10.May.2013
67 days after start of exposure
1 day after end of exposure

SINGLE ANIMAL SHEET
(GROSS LESIONS AND MICROSCOPIC FINDINGS)

Sacrifice F1
Sex F
Group 2
cont. Animal 61

Macroscopic findings
Animal without particular findings.

Microscopic findings
No histologic examination performed.

Animal 62

General information
Sex : Female
Group : 2 (4 RON/ 0.025 FLT/ 5 590F mg/kg)
Sacrifice : Final sacrifice group
Necropsy status : Planned sacrifice
Date of death : 10.May.2013
67 days after start of exposure
1 day after end of exposure

Macroscopic findings
Animal without particular findings.

Microscopic findings
No histologic examination performed.

Animal 68

General information
Sex : Female
Group : 2 (4 RON/ 0.025 FLT/ 5 590F mg/kg)
Sacrifice : Final sacrifice group
Necropsy status : Planned sacrifice
Date of death : 10.May.2013
66 days after start of exposure
1 day after end of exposure

Macroscopic findings
Animal without particular findings.

Microscopic findings
No histologic examination performed.

Animal 69

General information
Sex : Female
Group : 2 (4 RON/ 0.025 FLT/ 5 590F mg/kg)
Sacrifice : Final sacrifice group
Necropsy status : Planned sacrifice
Date of death : 10.May.2013
66 days after start of exposure

SINGLE ANIMAL SHEET
(GROSS LESIONS AND MICROSCOPIC FINDINGS)

Sacrifice F1
Sex F
Group 2
cont. Animal 69

1 day after end of exposure

Macroscopic findings
Animal without particular findings.

Microscopic findings
No histologic examination performed.

Animal 70

General information
Sex : Female
Group : 2 (4 RON/ 0.025 FLT/ 5 590F mg/kg)
Sacrifice : Final sacrifice group
Necropsy status : Planned sacrifice
Date of death : 10.May.2013
66 days after start of exposure
1 day after end of exposure

Macroscopic findings
Animal without particular findings.

Microscopic findings
No histologic examination performed.

Animal 71

General information
Sex : Female
Group : 2 (4 RON/ 0.025 FLT/ 5 590F mg/kg)
Sacrifice : Final sacrifice group
Necropsy status : Planned sacrifice
Date of death : 10.May.2013
66 days after start of exposure
1 day after end of exposure

Macroscopic findings
Animal without particular findings.

Microscopic findings
No histologic examination performed.

SINGLE ANIMAL SHEET
(GROSS LESIONS AND MICROSCOPIC FINDINGS)

Sacrifice F1
Sex F
Group 2
Animal 72

General information
Sex : Female
Group : 2 (4 RON/ 0.025 FLT/ 5 590F mg/kg)
Sacrifice : Final sacrifice group
Necropsy status : Planned sacrifice
Date of death : 10.May.2013
66 days after start of exposure
1 day after end of exposure

Macroscopic findings
Animal without particular findings.

Microscopic findings
No histologic examination performed.

Animal 73

General information
Sex : Female
Group : 2 (4 RON/ 0.025 FLT/ 5 590F mg/kg)
Sacrifice : Final sacrifice group
Necropsy status : Planned sacrifice
Date of death : 10.May.2013
65 days after start of exposure
1 day after end of exposure

Macroscopic findings
Animal without particular findings.

Microscopic findings
No histologic examination performed.

Animal 74

General information
Sex : Female
Group : 2 (4 RON/ 0.025 FLT/ 5 590F mg/kg)
Sacrifice : Final sacrifice group
Necropsy status : Planned sacrifice
Date of death : 10.May.2013
65 days after start of exposure
1 day after end of exposure

SINGLE ANIMAL SHEET
(GROSS LESIONS AND MICROSCOPIC FINDINGS)

Sacrifice	F1
Sex	F
Group	2
cont. Animal	74

Macroscopic findings
Animal without particular findings.

Microscopic findings
No histologic examination performed.

Animal 75

General information
Sex : Female
Group : 2 (4 RON/ 0.025 FLT/ 5 590F mg/kg)
Sacrifice : Final sacrifice group
Necropsy status : Planned sacrifice
Date of death : 10.May.2013
65 days after start of exposure
1 day after end of exposure

Macroscopic findings
Animal without particular findings.

Microscopic findings
No histologic examination performed.

SINGLE ANIMAL SHEET
(GROSS LESIONS AND MICROSCOPIC FINDINGS)

Sacrifice F1
Sex F
Group 3
Animal 76

General information
  Sex : Female
  Group : 3 (20 RON/ 0.25 FLT/ 30 590F mg/kg)
  Sacrifice : Final sacrifice group
  Necropsy status : Sacrificed moribund
  Date of death : 20.Mar.2013
  17 days after start of exposure

Macroscopic findings
  Forestomach
    Erosion/ulcer, few (2-5) diameter 2.0 mm, black.
  Glandular stomach
    Erosion/ulcer, few (2-5) diameter 2.0 mm, black.
  All other organs without macroscopic findings.

Microscopic findings
  Forestomach
    Histopathologic evaluation of gross lesion(s) will not be performed.
  Glandular stomach
    Histopathologic evaluation of gross lesion(s) will not be performed.
  No histologic examination performed.

Animal 77

General information
  Sex : Female
  Group : 3 (20 RON/ 0.25 FLT/ 30 590F mg/kg)
  Sacrifice : Final sacrifice group
  Necropsy status : Planned sacrifice
  Date of death : 06.May.2013
  64 days after start of exposure
  1 day after end of exposure

Macroscopic findings
  Animal without particular findings.

Microscopic findings
  No histologic examination performed.

Animal 78

General information
  Sex : Female
  Group : 3 (20 RON/ 0.25 FLT/ 30 590F mg/kg)
  Sacrifice : Final sacrifice group
  Necropsy status : Planned sacrifice
  Date of death : 06.May.2013
  64 days after start of exposure
  1 day after end of exposure

SINGLE ANIMAL SHEET
(GROSS LESIONS AND MICROSCOPIC FINDINGS)

Sacrifice F1
Sex F
Group 3
cont. Animal 78

Macroscopic findings
  Animal without particular findings.

Microscopic findings
  No histologic examination performed.

Animal 79

General information
  Sex : Female
  Group : 3 (20 RON/ 0.25 FLT/ 30 590F mg/kg)
  Sacrifice : Final sacrifice group
  Necropsy status : Planned sacrifice
  Date of death : 06.May.2013
  64 days after start of exposure
  1 day after end of exposure

Macroscopic findings
  Animal without particular findings.

Microscopic findings
  No histologic examination performed.

Animal 80

General information
  Sex : Female
  Group : 3 (20 RON/ 0.25 FLT/ 30 590F mg/kg)
  Sacrifice : Final sacrifice group
  Necropsy status : Planned sacrifice
  Date of death : 06.May.2013
  64 days after start of exposure
  1 day after end of exposure

Macroscopic findings
  Animal without particular findings.

Microscopic findings
  No histologic examination performed.

Animal 81

General information
  Sex : Female
  Group : 3 (20 RON/ 0.25 FLT/ 30 590F mg/kg)
  Sacrifice : Final sacrifice group
  Necropsy status : Planned sacrifice
  Date of death : 06.May.2013
  64 days after start of exposure

SINGLE ANIMAL SHEET
(GROSS LESIONS AND MICROSCOPIC FINDINGS)

Sacrifice F1
Sex F
Group 3
cont. Animal 81

1 day after end of exposure

Macroscopic findings
Animal without particular findings.

Microscopic findings
No histologic examination performed.

Animal 82

General information
Sex : Female
Group : 3 (20 RON/ 0.25 FLT/ 30 590F mg/kg)
Sacrifice : Final sacrifice group
Necropsy status : Planned sacrifice
Date of death : 06.May.2013
64 days after start of exposure
1 day after end of exposure

Macroscopic findings
Animal without particular findings.

Microscopic findings
No histologic examination performed.

Animal 83

General information
Sex : Female
Group : 3 (20 RON/ 0.25 FLT/ 30 590F mg/kg)
Sacrifice : Final sacrifice group
Necropsy status : Planned sacrifice
Date of death : 06.May.2013
63 days after start of exposure
1 day after end of exposure

Macroscopic findings
Animal without particular findings.

Microscopic findings
No histologic examination performed.

SINGLE ANIMAL SHEET
(GROSS LESIONS AND MICROSCOPIC FINDINGS)

Sacrifice F1
Sex F
Group 3
Animal 84

General information
  Sex : Female
  Group : 3 (20 RON/ 0.25 FLT/ 30 590F mg/kg)
  Sacrifice : Final sacrifice group
  Necropsy status : Planned sacrifice
  Date of death : 06.May.2013
  63 days after start of exposure
  1 day after end of exposure

Macroscopic findings
  Animal without particular findings.

Microscopic findings
  No histologic examination performed.

Animal 85

General information
  Sex : Female
  Group : 3 (20 RON/ 0.25 FLT/ 30 590F mg/kg)
  Sacrifice : Final sacrifice group
  Necropsy status : Planned sacrifice
  Date of death : 06.May.2013
  63 days after start of exposure
  1 day after end of exposure

Macroscopic findings
  Animal without particular findings.

Microscopic findings
  No histologic examination performed.

Animal 86

General information
  Sex : Female
  Group : 3 (20 RON/ 0.25 FLT/ 30 590F mg/kg)
  Sacrifice : Final sacrifice group
  Necropsy status : Planned sacrifice
  Date of death : 06.May.2013
  63 days after start of exposure
  1 day after end of exposure

SINGLE ANIMAL SHEET
(GROSS LESIONS AND MICROSCOPIC FINDINGS)

Sacrifice F1
Sex F
Group 3
cont. Animal 86

Macroscopic findings
Animal without particular findings.

Microscopic findings
No histologic examination performed.

Animal 87

General information
Sex : Female
Group : 3 (20 RON/ 0.25 FLT/ 30 590F mg/kg)
Sacrifice : Final sacrifice group
Necropsy status : Planned sacrifice
Date of death : 10.May.2013
67 days after start of exposure
1 day after end of exposure

Macroscopic findings
Animal without particular findings.

Microscopic findings
No histologic examination performed.

Animal 93

General information
Sex : Female
Group : 3 (20 RON/ 0.25 FLT/ 30 590F mg/kg)
Sacrifice : Final sacrifice group
Necropsy status : Planned sacrifice
Date of death : 10.May.2013
66 days after start of exposure
1 day after end of exposure

Macroscopic findings
Animal without particular findings.

Microscopic findings
No histologic examination performed.

Animal 94

General information
Sex : Female
Group : 3 (20 RON/ 0.25 FLT/ 30 590F mg/kg)
Sacrifice : Final sacrifice group
Necropsy status : Planned sacrifice
Date of death : 10.May.2013
66 days after start of exposure

SINGLE ANIMAL SHEET
(GROSS LESIONS AND MICROSCOPIC FINDINGS)

Sacrifice F1
Sex F
Group 3
cont. Animal 94

1 day after end of exposure

Macroscopic findings
Animal without particular findings.

Microscopic findings
No histologic examination performed.

Animal 95

General information
Sex : Female
Group : 3 (20 RON/ 0.25 FLT/ 30 590F mg/kg)
Sacrifice : Final sacrifice group
Necropsy status : Planned sacrifice
Date of death : 10.May.2013
66 days after start of exposure
1 day after end of exposure

Macroscopic findings
Animal without particular findings.

Microscopic findings
No histologic examination performed.

Animal 96

General information
Sex : Female
Group : 3 (20 RON/ 0.25 FLT/ 30 590F mg/kg)
Sacrifice : Final sacrifice group
Necropsy status : Planned sacrifice
Date of death : 10.May.2013
66 days after start of exposure
1 day after end of exposure

Macroscopic findings
Animal without particular findings.

Microscopic findings
No histologic examination performed.

SINGLE ANIMAL SHEET
(GROSS LESIONS AND MICROSCOPIC FINDINGS)

Sacrifice F1
Sex F
Group 3
Animal 97

General information
Sex : Female
Group : 3 (20 RON/ 0.25 FLT/ 30 590F mg/kg)
Sacrifice : Final sacrifice group
Necropsy status : Planned sacrifice
Date of death : 10.May.2013
66 days after start of exposure
1 day after end of exposure

Macroscopic findings
Animal without particular findings.

Microscopic findings
No histologic examination performed.

Animal 98

General information
Sex : Female
Group : 3 (20 RON/ 0.25 FLT/ 30 590F mg/kg)
Sacrifice : Final sacrifice group
Necropsy status : Planned sacrifice
Date of death : 10.May.2013
65 days after start of exposure
1 day after end of exposure

Macroscopic findings
Animal without particular findings.

Microscopic findings
No histologic examination performed.

Animal 99

General information
Sex : Female
Group : 3 (20 RON/ 0.25 FLT/ 30 590F mg/kg)
Sacrifice : Final sacrifice group
Necropsy status : Planned sacrifice
Date of death : 10.May.2013
65 days after start of exposure
1 day after end of exposure

SINGLE ANIMAL SHEET
(GROSS LESIONS AND MICROSCOPIC FINDINGS)

Sacrifice F1
Sex F
Group 3
cont. Animal 99

Macroscopic findings
  Animal without particular findings.

Microscopic findings
  No histologic examination performed.

Animal 100

General information
  Sex : Female
  Group : 3 (20 RON/ 0.25 FLT/ 30 590F mg/kg)
  Sacrifice : Final sacrifice group
  Necropsy status : Planned sacrifice
  Date of death : 10.May.2013
  65 days after start of exposure
  1 day after end of exposure

Macroscopic findings
  Animal without particular findings.

Microscopic findings
  No histologic examination performed.

SINGLE ANIMAL SHEET
(GROSS LESIONS AND MICROSCOPIC FINDINGS)

Sacrifice F1
Sex F
Group 4
Animal 101

General information
Sex : Female
Group : 4 (Flutamide 0.00025 mg/kg)
Sacrifice : Final sacrifice group
Necropsy status : Planned sacrifice
Date of death : 06.May.2013
64 days after start of exposure
1 day after end of exposure

Macroscopic findings
Animal without particular findings.

Microscopic findings
No histologic examination performed.

Animal 102

General information
Sex : Female
Group : 4 (Flutamide 0.00025 mg/kg)
Sacrifice : Final sacrifice group
Necropsy status : Planned sacrifice
Date of death : 06.May.2013
64 days after start of exposure
1 day after end of exposure

Macroscopic findings
Animal without particular findings.

Microscopic findings
No histologic examination performed.

Animal 103

General information
Sex : Female
Group : 4 (Flutamide 0.00025 mg/kg)
Sacrifice : Final sacrifice group
Necropsy status : Planned sacrifice
Date of death : 06.May.2013
64 days after start of exposure
1 day after end of exposure

SINGLE ANIMAL SHEET
(GROSS LESIONS AND MICROSCOPIC FINDINGS)

Sacrifice F1
Sex F
Group 4
cont. Animal 103

Macroscopic findings
Animal without particular findings.

Microscopic findings
No histologic examination performed.

Animal 104

General information
Sex : Female
Group : 4 (Flutamide 0.00025 mg/kg)
Sacrifice : Final sacrifice group
Necropsy status : Planned sacrifice
Date of death : 06.May.2013
64 days after start of exposure
1 day after end of exposure

Macroscopic findings
Animal without particular findings.

Microscopic findings
No histologic examination performed.

Animal 105

General information
Sex : Female
Group : 4 (Flutamide 0.00025 mg/kg)
Sacrifice : Final sacrifice group
Necropsy status : Planned sacrifice
Date of death : 06.May.2013
64 days after start of exposure
1 day after end of exposure

Macroscopic findings
Animal without particular findings.

Microscopic findings
No histologic examination performed.

Animal 106

General information
Sex : Female
Group : 4 (Flutamide 0.00025 mg/kg)
Sacrifice : Final sacrifice group
Necropsy status : Planned sacrifice
Date of death : 06.May.2013
64 days after start of exposure

SINGLE ANIMAL SHEET
(GROSS LESIONS AND MICROSCOPIC FINDINGS)

Sacrifice F1
Sex F
Group 4
cont. Animal 106

1 day after end of exposure

Macroscopic findings
Animal without particular findings.

Microscopic findings
No histologic examination performed.

Animal 107

General information
Sex : Female
Group : 4 (Flutamide 0.00025 mg/kg)
Sacrifice : Final sacrifice group
Necropsy status : Planned sacrifice
Date of death : 06.May.2013
64 days after start of exposure
1 day after end of exposure

Macroscopic findings
Animal without particular findings.

Microscopic findings
No histologic examination performed.

Animal 108

General information
Sex : Female
Group : 4 (Flutamide 0.00025 mg/kg)
Sacrifice : Final sacrifice group
Necropsy status : Planned sacrifice
Date of death : 06.May.2013
63 days after start of exposure
1 day after end of exposure

Macroscopic findings
Animal without particular findings.

Microscopic findings
No histologic examination performed.

SINGLE ANIMAL SHEET
(GROSS LESIONS AND MICROSCOPIC FINDINGS)

Sacrifice F1
Sex F
Group 4
Animal 109

General information
Sex : Female
Group : 4 (Flutamide 0.00025 mg/kg)
Sacrifice : Final sacrifice group
Necropsy status : Planned sacrifice
Date of death : 06.May.2013
63 days after start of exposure
1 day after end of exposure

Macroscopic findings
Animal without particular findings.

Microscopic findings
No histologic examination performed.

Animal 110

General information
Sex : Female
Group : 4 (Flutamide 0.00025 mg/kg)
Sacrifice : Final sacrifice group
Necropsy status : Planned sacrifice
Date of death : 06.May.2013
63 days after start of exposure
1 day after end of exposure

Macroscopic findings
Animal without particular findings.

Microscopic findings
No histologic examination performed.

Animal 111

General information
Sex : Female
Group : 4 (Flutamide 0.00025 mg/kg)
Sacrifice : Final sacrifice group
Necropsy status : Planned sacrifice
Date of death : 10.May.2013
67 days after start of exposure
1 day after end of exposure

SINGLE ANIMAL SHEET
(GROSS LESIONS AND MICROSCOPIC FINDINGS)

Sacrifice F1
Sex F
Group 4
cont. Animal 111

Macroscopic findings
  Animal without particular findings.

Microscopic findings
  No histologic examination performed.

Animal 117

General information
  Sex : Female
  Group : 4 (Flutamide 0.00025 mg/kg)
  Sacrifice : Final sacrifice group
  Necropsy status : Planned sacrifice
  Date of death : 10.May.2013
  66 days after start of exposure
  1 day after end of exposure

Macroscopic findings
  Animal without particular findings.

Microscopic findings
  No histologic examination performed.

Animal 118

General information
  Sex : Female
  Group : 4 (Flutamide 0.00025 mg/kg)
  Sacrifice : Final sacrifice group
  Necropsy status : Planned sacrifice
  Date of death : 10.May.2013
  66 days after start of exposure
  1 day after end of exposure

Macroscopic findings
  Animal without particular findings.

Microscopic findings
  No histologic examination performed.

Animal 119

General information
  Sex : Female
  Group : 4 (Flutamide 0.00025 mg/kg)
  Sacrifice : Final sacrifice group
  Necropsy status : Planned sacrifice
  Date of death : 10.May.2013
  66 days after start of exposure

SINGLE ANIMAL SHEET
(GROSS LESIONS AND MICROSCOPIC FINDINGS)

Sacrifice F1
Sex F
Group 4
cont. Animal 119

1 day after end of exposure

Macroscopic findings
Animal without particular findings.

Microscopic findings
No histologic examination performed.

Animal 120

General information
Sex : Female
Group : 4 (Flutamide 0.00025 mg/kg)
Sacrifice : Final sacrifice group
Necropsy status : Planned sacrifice
Date of death : 10.May.2013
66 days after start of exposure
1 day after end of exposure

Macroscopic findings
Animal without particular findings.

Microscopic findings
No histologic examination performed.

Animal 121

General information
Sex : Female
Group : 4 (Flutamide 0.00025 mg/kg)
Sacrifice : Final sacrifice group
Necropsy status : Planned sacrifice
Date of death : 10.May.2013
66 days after start of exposure
1 day after end of exposure

Macroscopic findings
Animal without particular findings.

Microscopic findings
No histologic examination performed.

SINGLE ANIMAL SHEET
(GROSS LESIONS AND MICROSCOPIC FINDINGS)

Sacrifice	F1
Sex	F
Group	4
Animal	123

General information
Sex : Female
Group : 4 (Flutamide 0.00025 mg/kg)
Sacrifice : Final sacrifice group
Necropsy status : Planned sacrifice
Date of death : 10.May.2013
65 days after start of exposure
1 day after end of exposure

Macroscopic findings
Animal without particular findings.

Microscopic findings
No histologic examination performed.

Animal 124

General information
Sex : Female
Group : 4 (Flutamide 0.00025 mg/kg)
Sacrifice : Final sacrifice group
Necropsy status : Planned sacrifice
Date of death : 10.May.2013
65 days after start of exposure
1 day after end of exposure

Macroscopic findings
Animal without particular findings.

Microscopic findings
No histologic examination performed.

Animal 125

General information
Sex : Female
Group : 4 (Flutamide 0.00025 mg/kg)
Sacrifice : Final sacrifice group
Necropsy status : Planned sacrifice
Date of death : 10.May.2013
65 days after start of exposure
1 day after end of exposure

SINGLE ANIMAL SHEET
(GROSS LESIONS AND MICROSCOPIC FINDINGS)

Sacrifice	F1
Sex	F
Group	4
cont. Animal	125

Macroscopic findings
Animal without particular findings.

Microscopic findings
No histologic examination performed.

Reproductive Toxicity Study to detect potential effects to anti-androgenic substances in Wistar Rats (Gavage) 28.Mar.2014 SIGR

SINGLE ANIMAL SHEET
(GROSS LESIONS AND MICROSCOPIC FINDINGS)

Sacrifice	R1
Sex	M
Group	0
Animal	301

General information
- Sex : Male
- Group : 0 (0 mg/kg)
- Sacrifice : Subset 1 (PND 21)
- Necropsy status : Planned sacrifice
- Date of death : 09.Apr.2013

Macroscopic findings
  Animal without particular findings.

Microscopic findings
- Coagulating glands
  - Immature stage.
- Left epididymis
  - Immature stage.
- Left testicle
  - Immature stage.
- Pituitary gland
  - No or insufficent tissue present on slide.
- Prostata ventral fixed
  - Immature stage.
  - Secretion, grade 1.
- Prostate, dorso-lateral, fixed
  - Immature stage.
- Seminal vesicle
  - Immature stage.
- All other organs examined without microscopic findings.

Animal 302

General information
- Sex : Male
- Group : 0 (0 mg/kg)
- Sacrifice : Subset 1 (PND 21)
- Necropsy status : Planned sacrifice
- Date of death : 09.Apr.2013

Macroscopic findings
  Animal without particular findings.

Microscopic findings
- Coagulating glands
  - Immature stage.
- Left epididymis
  - Immature stage.

SINGLE ANIMAL SHEET
(GROSS LESIONS AND MICROSCOPIC FINDINGS)

Sacrifice R1
Sex M
Group 0
cont. Animal 302

Left testicle
Immature stage.
Prostata ventral fixed
Immature stage.
Secretion, grade 1.
Prostate, dorso-lateral, fixed
Immature stage.
Seminal vesicle
Immature stage.
All other organs examined without microscopic findings.

Animal 303

General information
Sex : Male
Group : 0 (0 mg/kg)
Sacrifice : Subset 1 (PND 21)
Necropsy status : Planned sacrifice
Date of death : 09.Apr.2013

Macroscopic findings
Animal without particular findings.

Microscopic findings
Coagulating glands
Immature stage.
Left epididymis
Immature stage.
Left testicle
Immature stage.
Pituitary gland
No or insuffiicent tissue present on slide.
Prostata ventral fixed
Immature stage.
Secretion, grade 1.
Prostate, dorso-lateral, fixed
Immature stage.
Seminal vesicle
Immature stage.
All other organs examined without microscopic findings.

SINGLE ANIMAL SHEET
(GROSS LESIONS AND MICROSCOPIC FINDINGS)

Sacrifice R1
Sex M
Group 0
Animal 304

General information
Sex : Male
Group : 0 (0 mg/kg)
Sacrifice : Subset 1 (PND 21)
Necropsy status : Planned sacrifice
Date of death : 09.Apr.2013

Macroscopic findings
Animal without particular findings.

Microscopic findings
Coagulating glands
Immature stage.
Left epididymis
Immature stage.
Left testicle
Immature stage.
Prostata ventral fixed
Immature stage.
Secretion, grade 1.
Prostate, dorso-lateral, fixed
Immature stage.
Seminal vesicle
Immature stage.
All other organs examined without microscopic findings.

Animal 305

General information
Sex : Male
Group : 0 (0 mg/kg)
Sacrifice : Subset 1 (PND 21)
Necropsy status : Planned sacrifice
Date of death : 10.Apr.2013

Macroscopic findings
Animal without particular findings.

Microscopic findings
Coagulating glands
Immature stage.
Left epididymis
Immature stage.
Left testicle
Immature stage.

SINGLE ANIMAL SHEET
(GROSS LESIONS AND MICROSCOPIC FINDINGS)

Sacrifice R1
Sex M
Group 0
cont. Animal 305

Prostata ventral fixed
Immature stage.
Secretion, grade 1.
Prostate, dorso-lateral, fixed
Immature stage.
Seminal vesicle
Immature stage.
All other organs examined without microscopic findings.

Animal 306

General information
Sex : Male
Group : 0 (0 mg/kg)
Sacrifice : Subset 1 (PND 21)
Necropsy status : Planned sacrifice
Date of death : 10.Apr.2013

Macroscopic findings
Animal without particular findings.

Microscopic findings
Coagulating glands
Immature stage.
Left epididymis
Immature stage.
Left testicle
Immature stage.
Prostata ventral fixed
Immature stage.
Secretion, grade 1.
Prostate, dorso-lateral, fixed
No or insufficent tissue present on slide.
Seminal vesicle
Immature stage.
All other organs examined without microscopic findings.

Animal 307

General information
Sex : Male
Group : 0 (0 mg/kg)
Sacrifice : Subset 1 (PND 21)
Necropsy status : Planned sacrifice
Date of death : 11.Apr.2013

SINGLE ANIMAL SHEET
(GROSS LESIONS AND MICROSCOPIC FINDINGS)

Sacrifice	R1
Sex	M
Group	0
cont. Animal	307

Macroscopic findings
  Animal without particular findings.

Microscopic findings
  Coagulating glands
    Immature stage.
  Left epididymis
    Immature stage.
  Left testicle
    Immature stage.
  Prostata ventral fixed
    Immature stage.
    Secretion, grade 1.
  Prostate, dorso-lateral, fixed
    Immature stage.
  Seminal vesicle
    Immature stage.
  All other organs examined without microscopic findings.

Animal 308

General information
  Sex : Male
  Group : 0 (0 mg/kg)
  Sacrifice : Subset 1 (PND 21)
  Necropsy status : Planned sacrifice
  Date of death : 11.Apr.2013

Macroscopic findings
  Animal without particular findings.

Microscopic findings
  Adrenal cortex
    No or insufficent tissue present on slide.
  Adrenal medulla
    No or insufficent tissue present on slide.
  Coagulating glands
    Immature stage.
  Left epididymis
    Immature stage.
  Left testicle
    Immature stage.
  Prostata ventral fixed
    Immature stage.
    Secretion, grade 1.

SINGLE ANIMAL SHEET
(GROSS LESIONS AND MICROSCOPIC FINDINGS)

Sacrifice R1
Sex M
Group 0
cont. Animal 308

Prostate, dorso-lateral, fixed
Immature stage.
Seminal vesicle
Immature stage.
All other organs examined without microscopic findings.

Animal 309

General information
Sex : Male
Group : 0 (0 mg/kg)
Sacrifice : Subset 1 (PND 21)
Necropsy status : Planned sacrifice
Date of death : 12.Apr.2013

Macroscopic findings
Animal without particular findings.

Microscopic findings
Coagulating glands
Immature stage.
Left epididymis
Immature stage.
Left testicle
Immature stage.
Prostata ventral fixed
Immature stage.
Secretion, grade 1.
Prostate, dorso-lateral, fixed
Immature stage.
Seminal vesicle
Immature stage.
All other organs examined without microscopic findings.

Animal 310

General information
Sex : Male
Group : 0 (0 mg/kg)
Sacrifice : Subset 1 (PND 21)
Necropsy status : Planned sacrifice
Date of death : 12.Apr.2013

SINGLE ANIMAL SHEET
(GROSS LESIONS AND MICROSCOPIC FINDINGS)

Sacrifice	R1
Sex	M
Group	0
cont. Animal	310

Macroscopic findings
Animal without particular findings.

Microscopic findings
- Coagulating glands
  - Immature stage.
- Left epididymis
  - Immature stage.
- Left testicle
  - Immature stage.
- Pituitary gland
  - No or insufficent tissue present on slide.
- Prostata ventral fixed
  - Immature stage.
  - Secretion, grade 1.
- Prostate, dorso-lateral, fixed
  - Immature stage.
- Seminal vesicle
  - Immature stage.
- All other organs examined without microscopic findings.

SINGLE ANIMAL SHEET
(GROSS LESIONS AND MICROSCOPIC FINDINGS)

Sacrifice R1
Sex M
Group 1
Animal 311

General information
Sex : Male
Group : 1 (0.005RON/0.00025FLT/0.01 590F mg/kg)
Sacrifice : Subset 1 (PND 21)
Necropsy status : Planned sacrifice
Date of death : 09.Apr.2013

Macroscopic findings
Animal without particular findings.

Microscopic findings
Coagulating glands
Immature stage.
Left epididymis
Immature stage.
Left testicle
Immature stage.
Prostata ventral fixed
Immature stage.
Secretion, grade 1.
Prostate, dorso-lateral, fixed
Immature stage.
Seminal vesicle
Immature stage.
All other organs examined without microscopic findings.

Animal 312

General information
Sex : Male
Group : 1 (0.005RON/0.00025FLT/0.01 590F mg/kg)
Sacrifice : Subset 1 (PND 21)
Necropsy status : Planned sacrifice
Date of death : 09.Apr.2013

Macroscopic findings
Animal without particular findings.

Microscopic findings
Coagulating glands
Immature stage.
Left epididymis
Immature stage.
Left testicle
Immature stage.

SINGLE ANIMAL SHEET
(GROSS LESIONS AND MICROSCOPIC FINDINGS)

Sacrifice R1
Sex M
Group 1
cont. Animal 312

Prostata ventral fixed
Immature stage.
Secretion, grade 1.
Prostate, dorso-lateral, fixed
Immature stage.
Seminal vesicle
Immature stage.
All other organs examined without microscopic findings.

Animal 313

General information
Sex : Male
Group : 1 (0.005RON/0.00025FLT/0.01 590F mg/kg)
Sacrifice : Subset 1 (PND 21)
Necropsy status : Planned sacrifice
Date of death : 09.Apr.2013

Macroscopic findings
Animal without particular findings.

Microscopic findings
Coagulating glands
Immature stage.
Left epididymis
Immature stage.
Left testicle
Immature stage.
Prostata ventral fixed
Immature stage.
Secretion, grade 1.
Prostate, dorso-lateral, fixed
Immature stage.
Seminal vesicle
Immature stage.
All other organs examined without microscopic findings.

Animal 314

General information
Sex : Male
Group : 1 (0.005RON/0.00025FLT/0.01 590F mg/kg)
Sacrifice : Subset 1 (PND 21)
Necropsy status : Planned sacrifice
Date of death : 09.Apr.2013

SINGLE ANIMAL SHEET
(GROSS LESIONS AND MICROSCOPIC FINDINGS)

Sacrifice R1
Sex M
Group 1
cont. Animal 314

Macroscopic findings
  Animal without particular findings.

Microscopic findings
  Coagulating glands
    Immature stage.
  Left epididymis
    Immature stage.
  Left testicle
    Immature stage.
  Pituitary gland
    No or insuffficent tissue present on slide.
  Prostata ventral fixed
    Immature stage.
    Secretion, grade 1.
  Prostate, dorso-lateral, fixed
    Immature stage.
  Seminal vesicle
    Immature stage.
  All other organs examined without microscopic findings.

Animal 315

General information
  Sex : Male
  Group : 1 (0.005RON/0.00025FLT/0.01 590F mg/kg)
  Sacrifice : Subset 1 (PND 21)
  Necropsy status : Planned sacrifice
  Date of death : 10.Apr.2013

Macroscopic findings
  Animal without particular findings.

Microscopic findings
  Coagulating glands
    Immature stage.
  Left epididymis
    Immature stage.
  Left testicle
    Immature stage.
  Prostata ventral fixed
    Immature stage.
    Secretion, grade 1.
  Prostate, dorso-lateral, fixed
    Immature stage.

SINGLE ANIMAL SHEET
(GROSS LESIONS AND MICROSCOPIC FINDINGS)

Sacrifice R1
Sex M
Group 1
cont. Animal 315

Seminal vesicle
Immature stage.
All other organs examined without microscopic findings.

Animal 316

General information
Sex : Male
Group : 1 (0.005RON/0.00025FLT/0.01 590F mg/kg)
Sacrifice : Subset 1 (PND 21)
Necropsy status : Planned sacrifice
Date of death : 10.Apr.2013

Macroscopic findings
Animal without particular findings.

Microscopic findings
Coagulating glands
Immature stage.
Left epididymis
Immature stage.
Left testicle
Immature stage.
Prostata ventral fixed
Immature stage.
Secretion, grade 1.
Prostate, dorso-lateral, fixed
Immature stage.
Seminal vesicle
Immature stage.
All other organs examined without microscopic findings.

Animal 317

General information
Sex : Male
Group : 1 (0.005RON/0.00025FLT/0.01 590F mg/kg)
Sacrifice : Subset 1 (PND 21)
Necropsy status : Planned sacrifice
Date of death : 10.Apr.2013

SINGLE ANIMAL SHEET
(GROSS LESIONS AND MICROSCOPIC FINDINGS)

Sacrifice R1
Sex M
Group 1
cont. Animal 317

Macroscopic findings
  Animal without particular findings.

Microscopic findings
  Coagulating glands
    Immature stage.
  Left epididymis
    Immature stage.
  Left testicle
    Immature stage.
  Prostata ventral fixed
    Immature stage.
    Secretion, grade 1.
  Prostate, dorso-lateral, fixed
    Immature stage.
  Seminal vesicle
    Immature stage.
  All other organs examined without microscopic findings.

Animal 318

General information
  Sex : Male
  Group : 1 (0.005RON/0.00025FLT/0.01 590F mg/kg)
  Sacrifice : Subset 1 (PND 21)
  Necropsy status : Planned sacrifice
  Date of death : 10.Apr.2013

Macroscopic findings
  Animal without particular findings.

Microscopic findings
  Coagulating glands
    Immature stage.
  Left epididymis
    Immature stage.
  Left testicle
    Immature stage.
  Prostata ventral fixed
    Immature stage.
    Secretion, grade 1.
  Prostate, dorso-lateral, fixed
    Immature stage.
  Seminal vesicle
    Immature stage.
  All other organs examined without microscopic findings.

SINGLE ANIMAL SHEET
(GROSS LESIONS AND MICROSCOPIC FINDINGS)

Sacrifice R1
Sex M
Group 1
Animal 319

General information
Sex : Male
Group : 1 (0.005RON/0.00025FLT/0.01 590F mg/kg)
Sacrifice : Subset 1 (PND 21)
Necropsy status : Planned sacrifice
Date of death : 11.Apr.2013

Macroscopic findings
Animal without particular findings.

Microscopic findings
Coagulating glands
Immature stage.
Left epididymis
Immature stage.
Left testicle
Immature stage.
Prostata ventral fixed
Immature stage.
Secretion, grade 1.
Prostate, dorso-lateral, fixed
Immature stage.
Seminal vesicle
Immature stage.
All other organs examined without microscopic findings.

Animal 320

General information
Sex : Male
Group : 1 (0.005RON/0.00025FLT/0.01 590F mg/kg)
Sacrifice : Subset 1 (PND 21)
Necropsy status : Planned sacrifice
Date of death : 12.Apr.2013

Macroscopic findings
Animal without particular findings.

Microscopic findings
Coagulating glands
Immature stage.
Left epididymis
Immature stage.
Left testicle
Immature stage.

SINGLE ANIMAL SHEET
(GROSS LESIONS AND MICROSCOPIC FINDINGS)

Sacrifice	R1
Sex	M
Group	1
cont. Animal	320

Prostata ventral fixed
  Immature stage.
  Secretion, grade 1.
Prostate, dorso-lateral, fixed
  Immature stage.
Seminal vesicle
  Immature stage.
All other organs examined without microscopic findings.

SINGLE ANIMAL SHEET
(GROSS LESIONS AND MICROSCOPIC FINDINGS)

Sacrifice R1
Sex M
Group 2
Animal 321

General information
- Sex : Male
- Group : 2 (4 RON/ 0.025 FLT/ 5 590F mg/kg)
- Sacrifice : Subset 1 (PND 21)
- Necropsy status : Planned sacrifice
- Date of death : 09.Apr.2013

Macroscopic findings
Animal without particular findings.

Microscopic findings
- Coagulating glands
  - Immature stage.
- Left epididymis
  - Immature stage.
- Left testicle
  - Immature stage.
- Prostata ventral fixed
  - Immature stage.
  - Secretion, grade 1.
- Prostate, dorso-lateral, fixed
  - Immature stage.
- Seminal vesicle
  - Immature stage.
- All other organs examined without microscopic findings.

Animal 322

General information
- Sex : Male
- Group : 2 (4 RON/ 0.025 FLT/ 5 590F mg/kg)
- Sacrifice : Subset 1 (PND 21)
- Necropsy status : Planned sacrifice
- Date of death : 09.Apr.2013

Macroscopic findings
Animal without particular findings.

Microscopic findings
- Coagulating glands
  - Immature stage.
- Left epididymis
  - Immature stage.
- Left testicle
  - Immature stage.

SINGLE ANIMAL SHEET
(GROSS LESIONS AND MICROSCOPIC FINDINGS)

Sacrifice R1
Sex M
Group 2
cont. Animal 322

Prostata ventral fixed
  Immature stage.
  Secretion, grade 1.
Prostate, dorso-lateral, fixed
  Immature stage.
Seminal vesicle
  Immature stage.
All other organs examined without microscopic findings.

Animal 323

General information
  Sex : Male
  Group : 2 (4 RON/ 0.025 FLT/ 5 590F mg/kg)
  Sacrifice : Subset 1 (PND 21)
  Necropsy status : Planned sacrifice
  Date of death : 09.Apr.2013

Macroscopic findings
  Animal without particular findings.

Microscopic findings
  Coagulating glands
    Immature stage.
  Left epididymis
    Immature stage.
  Left testicle
    Immature stage.
  Prostata ventral fixed
    Immature stage.
    Secretion, grade 1.
  Prostate, dorso-lateral, fixed
    No or insufficent tissue present on slide.
  Seminal vesicle
    Immature stage.
  All other organs examined without microscopic findings.

Animal 324

General information
  Sex : Male
  Group : 2 (4 RON/ 0.025 FLT/ 5 590F mg/kg)
  Sacrifice : Subset 1 (PND 21)
  Necropsy status : Planned sacrifice
  Date of death : 10.Apr.2013

SINGLE ANIMAL SHEET
(GROSS LESIONS AND MICROSCOPIC FINDINGS)

Sacrifice R1
Sex M
Group 2
cont. Animal 324

Macroscopic findings
  Animal without particular findings.

Microscopic findings
  Coagulating glands
    Immature stage.
  Left epididymis
    Immature stage.
  Left testicle
    Immature stage.
  Prostata ventral fixed
    Immature stage.
    Secretion, grade 1.
  Prostate, dorso-lateral, fixed
    No or insufficent tissue present on slide.
  Seminal vesicle
    Immature stage.
  All other organs examined without microscopic findings.

Animal 325

General information
  Sex : Male
  Group : 2 (4 RON/ 0.025 FLT/ 5 590F mg/kg)
  Sacrifice : Subset 1 (PND 21)
  Necropsy status : Planned sacrifice
  Date of death : 10.Apr.2013

Macroscopic findings
  Animal without particular findings.

Microscopic findings
  Coagulating glands
    Immature stage.
  Left epididymis
    Immature stage.
  Left testicle
    Immature stage.
  Pituitary gland
    No or insufficent tissue present on slide.
  Prostata ventral fixed
    Immature stage.
    Secretion, grade 1.
  Prostate, dorso-lateral, fixed
    Immature stage.

SINGLE ANIMAL SHEET
(GROSS LESIONS AND MICROSCOPIC FINDINGS)

Sacrifice R1
Sex M
Group 2
cont. Animal 325

Seminal vesicle
Immature stage.
All other organs examined without microscopic findings.

Animal 326

General information
Sex : Male
Group : 2 (4 RON/ 0.025 FLT/ 5 590F mg/kg)
Sacrifice : Subset 1 (PND 21)
Necropsy status : Planned sacrifice
Date of death : 11.Apr.2013

Macroscopic findings
Animal without particular findings.

Microscopic findings
Coagulating glands
Immature stage.
Left epididymis
Immature stage.
Left testicle
Immature stage.
Pituitary gland
No or insufficent tissue present on slide.
Prostata ventral fixed
Immature stage.
Secretion, grade 1.
Prostate, dorso-lateral, fixed
Immature stage.
Seminal vesicle
Immature stage.
All other organs examined without microscopic findings.

Animal 327

General information
Sex : Male
Group : 2 (4 RON/ 0.025 FLT/ 5 590F mg/kg)
Sacrifice : Subset 1 (PND 21)
Necropsy status : Planned sacrifice
Date of death : 11.Apr.2013

SINGLE ANIMAL SHEET
(GROSS LESIONS AND MICROSCOPIC FINDINGS)

Sacrifice R1
Sex M
Group 2
cont. Animal 327

Macroscopic findings
  Animal without particular findings.

Microscopic findings
  Coagulating glands
    Immature stage.
  Left epididymis
    Immature stage.
  Left testicle
    Immature stage.
  Prostata ventral fixed
    Immature stage.
    Secretion, grade 1.
  Prostate, dorso-lateral, fixed
    Immature stage.
  Seminal vesicle
    Immature stage.
  All other organs examined without microscopic findings.

Animal 328

General information
  Sex : Male
  Group : 2 (4 RON/ 0.025 FLT/ 5 590F mg/kg)
  Sacrifice : Subset 1 (PND 21)
  Necropsy status : Planned sacrifice
  Date of death : 11.Apr.2013

Macroscopic findings
  Animal without particular findings.

Microscopic findings
  Coagulating glands
    Immature stage.
  Left epididymis
    Immature stage.
  Left testicle
    Immature stage.
  Prostata ventral fixed
    Immature stage.
    Secretion, grade 1.
  Prostate, dorso-lateral, fixed
    Immature stage.
  Seminal vesicle
    Immature stage.
  All other organs examined without microscopic findings.

SINGLE ANIMAL SHEET
(GROSS LESIONS AND MICROSCOPIC FINDINGS)

Sacrifice R1
Sex M
Group 2
Animal 329

General information
Sex : Male
Group : 2 (4 RON/ 0.025 FLT/ 5 590F mg/kg)
Sacrifice : Subset 1 (PND 21)
Necropsy status : Planned sacrifice
Date of death : 11.Apr.2013

Macroscopic findings
Animal without particular findings.

Microscopic findings
Coagulating glands
Immature stage.
Left epididymis
Immature stage.
Left testicle
Immature stage.
Prostata ventral fixed
Immature stage.
Secretion, grade 1.
Prostate, dorso-lateral, fixed
Immature stage.
Seminal vesicle
Immature stage.
All other organs examined without microscopic findings.

Animal 330

General information
Sex : Male
Group : 2 (4 RON/ 0.025 FLT/ 5 590F mg/kg)
Sacrifice : Subset 1 (PND 21)
Necropsy status : Planned sacrifice
Date of death : 12.Apr.2013

Macroscopic findings
Animal without particular findings.

Microscopic findings
Coagulating glands
Immature stage.
Left epididymis
Immature stage.
Left testicle
Immature stage.

SINGLE ANIMAL SHEET
(GROSS LESIONS AND MICROSCOPIC FINDINGS)

Sacrifice	R1
Sex	M
Group	2
cont. Animal	330

Prostata ventral fixed
  Immature stage.
  Secretion, grade 1.
Prostate, dorso-lateral, fixed
  Immature stage.
Seminal vesicle
  Immature stage.
All other organs examined without microscopic findings.

SINGLE ANIMAL SHEET
(GROSS LESIONS AND MICROSCOPIC FINDINGS)

Sacrifice R1
Sex M
Group 3
Animal 331

General information
Sex : Male
Group : 3 (20 RON/ 0.25 FLT/ 30 590F mg/kg)
Sacrifice : Subset 1 (PND 21)
Necropsy status : Planned sacrifice
Date of death : 10.Apr.2013

Macroscopic findings
Animal without particular findings.

Microscopic findings
Coagulating glands
Immature stage.
Left epididymis
Immature stage.
Left testicle
Immature stage.
Prostata ventral fixed
Immature stage.
Secretion, grade 1.
Prostate, dorso-lateral, fixed
No or insufficent tissue present on slide.
Seminal vesicle
Immature stage.
All other organs examined without microscopic findings.

Animal 332

General information
Sex : Male
Group : 3 (20 RON/ 0.25 FLT/ 30 590F mg/kg)
Sacrifice : Subset 1 (PND 21)
Necropsy status : Planned sacrifice
Date of death : 10.Apr.2013

Macroscopic findings
Animal without particular findings.

Microscopic findings
Coagulating glands
Immature stage.
Left epididymis
Immature stage.
Left testicle
Immature stage.

SINGLE ANIMAL SHEET
(GROSS LESIONS AND MICROSCOPIC FINDINGS)

Sacrifice R1
Sex M
Group 3
cont. Animal 332

Prostata ventral fixed
  Immature stage.
  Secretion, grade 1.
Prostate, dorso-lateral, fixed
  Immature stage.
Seminal vesicle
  Immature stage.
All other organs examined without microscopic findings.

Animal 333

General information
- Sex : Male
- Group : 3 (20 RON/ 0.25 FLT/ 30 590F mg/kg)
- Sacrifice : Subset 1 (PND 21)
- Necropsy status : Planned sacrifice
- Date of death : 10.Apr.2013

Macroscopic findings
  Animal without particular findings.

Microscopic findings
Coagulating glands
  Immature stage.
Left epididymis
  Immature stage.
Left testicle
  Immature stage.
Prostata ventral fixed
  Immature stage.
  Secretion, grade 1.
Prostate, dorso-lateral, fixed
  Immature stage.
Seminal vesicle
  Immature stage.
All other organs examined without microscopic findings.

Animal 334

General information
- Sex : Male
- Group : 3 (20 RON/ 0.25 FLT/ 30 590F mg/kg)
- Sacrifice : Subset 1 (PND 21)
- Necropsy status : Planned sacrifice
- Date of death : 11.Apr.2013

SINGLE ANIMAL SHEET
(GROSS LESIONS AND MICROSCOPIC FINDINGS)

Sacrifice R1
Sex M
Group 3
cont. Animal 334

Macroscopic findings
  Animal without particular findings.

Microscopic findings
  Coagulating glands
    Immature stage.
  Left epididymis
    Immature stage.
  Left testicle
    Immature stage.
  Prostata ventral fixed
    Immature stage.
    Secretion, grade 1.
  Prostate, dorso-lateral, fixed
    Immature stage.
  Seminal vesicle
    Immature stage.
  All other organs examined without microscopic findings.

Animal 335

General information
  Sex : Male
  Group : 3 (20 RON/ 0.25 FLT/ 30 590F mg/kg)
  Sacrifice : Subset 1 (PND 21)
  Necropsy status : Planned sacrifice
  Date of death : 11.Apr.2013

Macroscopic findings
  Animal without particular findings.

Microscopic findings
  Coagulating glands
    Immature stage.
  Left epididymis
    Immature stage.
  Left testicle
    Immature stage.
  Prostata ventral fixed
    Immature stage.
    Secretion, grade 1.
  Prostate, dorso-lateral, fixed
    Immature stage.
  Seminal vesicle
    Immature stage.
  All other organs examined without microscopic findings.

SINGLE ANIMAL SHEET
(GROSS LESIONS AND MICROSCOPIC FINDINGS)

Sacrifice R1
Sex M
Group 3
Animal 336

General information
  Sex : Male
  Group : 3 (20 RON/ 0.25 FLT/ 30 590F mg/kg)
  Sacrifice : Subset 1 (PND 21)
  Necropsy status : Planned sacrifice
  Date of death : 12.Apr.2013

Macroscopic findings
  Animal without particular findings.

Microscopic findings
  Coagulating glands
    Immature stage.
  Left epididymis
    Immature stage.
  Left testicle
    Immature stage.
  Prostata ventral fixed
    Immature stage.
    Secretion, grade 1.
  Prostate, dorso-lateral, fixed
    Immature stage.
  Seminal vesicle
    Immature stage.
  All other organs examined without microscopic findings.

Animal 337

General information
  Sex : Male
  Group : 3 (20 RON/ 0.25 FLT/ 30 590F mg/kg)
  Sacrifice : Subset 1 (PND 21)
  Necropsy status : Planned sacrifice
  Date of death : 12.Apr.2013

Macroscopic findings
  Animal without particular findings.

Microscopic findings
  Coagulating glands
    Immature stage.
  Left epididymis
    Immature stage.
  Left testicle
    Immature stage.

SINGLE ANIMAL SHEET
(GROSS LESIONS AND MICROSCOPIC FINDINGS)

Sacrifice R1
Sex M
Group 3
cont. Animal 337

Prostata ventral fixed
  Immature stage.
  Secretion, grade 1.
Prostate, dorso-lateral, fixed
  Immature stage.
Seminal vesicle
  Immature stage.
All other organs examined without microscopic findings.

Animal 338

General information
- Sex : Male
- Group : 3 (20 RON/ 0.25 FLT/ 30 590F mg/kg)
- Sacrifice : Subset 1 (PND 21)
- Necropsy status : Planned sacrifice
- Date of death : 13.Apr.2013

Macroscopic findings
  Animal without particular findings.

Microscopic findings
Coagulating glands
  Immature stage.
Left epididymis
  Immature stage.
Left testicle
  Immature stage.
Prostata ventral fixed
  Immature stage.
  Secretion, grade 1.
Prostate, dorso-lateral, fixed
  Immature stage.
Seminal vesicle
  Immature stage.
All other organs examined without microscopic findings.

Animal 339

General information
- Sex : Male
- Group : 3 (20 RON/ 0.25 FLT/ 30 590F mg/kg)
- Sacrifice : Subset 1 (PND 21)
- Necropsy status : Planned sacrifice
- Date of death : 13.Apr.2013

SINGLE ANIMAL SHEET
(GROSS LESIONS AND MICROSCOPIC FINDINGS)

Sacrifice R1
Sex M
Group 3
cont. Animal 339

Macroscopic findings
  Animal without particular findings.

Microscopic findings
  Coagulating glands
    Immature stage.
  Left epididymis
    Immature stage.
  Left testicle
    Immature stage.
  Prostata ventral fixed
    Immature stage.
    Secretion, grade 1.
  Prostate, dorso-lateral, fixed
    Immature stage.
  Seminal vesicle
    Immature stage.
  All other organs examined without microscopic findings.

Animal 340

General information
  Sex : Male
  Group : 3 (20 RON/ 0.25 FLT/ 30 590F mg/kg)
  Sacrifice : Subset 1 (PND 21)
  Necropsy status : Planned sacrifice
  Date of death : 13.Apr.2013

Macroscopic findings
  Animal without particular findings.

Microscopic findings
  Coagulating glands
    Immature stage.
  Left epididymis
    Immature stage.
  Left testicle
    Immature stage.
  Prostata ventral fixed
    Immature stage.
    Secretion, grade 1.
  Prostate, dorso-lateral, fixed
    Immature stage.
  Seminal vesicle
    Immature stage.
  All other organs examined without microscopic findings.

SINGLE ANIMAL SHEET
(GROSS LESIONS AND MICROSCOPIC FINDINGS)

Sacrifice R1
Sex M
Group 4
Animal 341

General information
Sex : Male
Group : 4 (Flutamide 0.00025 mg/kg)
Sacrifice : Subset 1 (PND 21)
Necropsy status : Planned sacrifice
Date of death : 08.Apr.2013

Macroscopic findings
Animal without particular findings.

Microscopic findings
Coagulating glands
Immature stage.
Left epididymis
Immature stage.
Left testicle
Immature stage.
Prostata ventral fixed
Immature stage.
Secretion, grade 1.
Prostate, dorso-lateral, fixed
Immature stage.
Seminal vesicle
Immature stage.
All other organs examined without microscopic findings.

Animal 342

General information
Sex : Male
Group : 4 (Flutamide 0.00025 mg/kg)
Sacrifice : Subset 1 (PND 21)
Necropsy status : Planned sacrifice
Date of death : 09.Apr.2013

Macroscopic findings
Animal without particular findings.

Microscopic findings
Coagulating glands
Immature stage.
Left epididymis
Immature stage.
Left testicle
Immature stage.

SINGLE ANIMAL SHEET
(GROSS LESIONS AND MICROSCOPIC FINDINGS)

Sacrifice R1
Sex M
Group 4
cont. Animal 342

Prostata ventral fixed
Immature stage.
Secretion, grade 1.
Prostate, dorso-lateral, fixed
Immature stage.
Seminal vesicle
Immature stage.
All other organs examined without microscopic findings.

Animal 343

General information
Sex : Male
Group : 4 (Flutamide 0.00025 mg/kg)
Sacrifice : Subset 1 (PND 21)
Necropsy status : Planned sacrifice
Date of death : 09.Apr.2013

Macroscopic findings
Animal without particular findings.

Microscopic findings
Coagulating glands
Immature stage.
Left epididymis
Immature stage.
Left testicle
Immature stage.
Prostata ventral fixed
Immature stage.
Secretion, grade 1.
Prostate, dorso-lateral, fixed
No or insufficent tissue present on slide.
Seminal vesicle
Immature stage.
All other organs examined without microscopic findings.

Animal 344

General information
Sex : Male
Group : 4 (Flutamide 0.00025 mg/kg)
Sacrifice : Subset 1 (PND 21)
Necropsy status : Planned sacrifice
Date of death : 09.Apr.2013

SINGLE ANIMAL SHEET
(GROSS LESIONS AND MICROSCOPIC FINDINGS)

Sacrifice R1
Sex M
Group 4
cont. Animal 344

Macroscopic findings
  Animal without particular findings.

Microscopic findings
  Coagulating glands
    Immature stage.
  Left epididymis
    Immature stage.
  Left testicle
    Immature stage.
  Prostata ventral fixed
    Immature stage.
    Secretion, grade 1.
  Prostate, dorso-lateral, fixed
    Immature stage.
  Seminal vesicle
    Immature stage.
  All other organs examined without microscopic findings.

Animal 345

General information
  Sex : Male
  Group : 4 (Flutamide 0.00025 mg/kg)
  Sacrifice : Subset 1 (PND 21)
  Necropsy status : Planned sacrifice
  Date of death : 09.Apr.2013

Macroscopic findings
  Animal without particular findings.

Microscopic findings
  Coagulating glands
    Immature stage.
  Left epididymis
    Immature stage.
  Left testicle
    Immature stage.
  Prostata ventral fixed
    Immature stage.
    Secretion, grade 1.
  Prostate, dorso-lateral, fixed
    Immature stage.
  Seminal vesicle
    Immature stage.
  All other organs examined without microscopic findings.

SINGLE ANIMAL SHEET
(GROSS LESIONS AND MICROSCOPIC FINDINGS)

Sacrifice R1
Sex M
Group 4
Animal 346

General information
- Sex : Male
- Group : 4 (Flutamide 0.00025 mg/kg)
- Sacrifice : Subset 1 (PND 21)
- Necropsy status : Planned sacrifice
- Date of death : 10.Apr.2013

Macroscopic findings
Animal without particular findings.

Microscopic findings
- Coagulating glands
  - Immature stage.
- Left epididymis
  - Immature stage.
- Left testicle
  - Immature stage.
- Prostata ventral fixed
  - Immature stage.
  - Secretion, grade 1.
- Prostate, dorso-lateral, fixed
  - No or insufficent tissue present on slide.
- Seminal vesicle
  - Immature stage.
- All other organs examined without microscopic findings.

Animal 347

General information
- Sex : Male
- Group : 4 (Flutamide 0.00025 mg/kg)
- Sacrifice : Subset 1 (PND 21)
- Necropsy status : Planned sacrifice
- Date of death : 10.Apr.2013

Macroscopic findings
Animal without particular findings.

Microscopic findings
- Coagulating glands
  - Immature stage.
- Left epididymis
  - Immature stage.
- Left testicle
  - Immature stage.

SINGLE ANIMAL SHEET
(GROSS LESIONS AND MICROSCOPIC FINDINGS)

Sacrifice R1
Sex M
Group 4
cont. Animal 347

Prostata ventral fixed
Immature stage.
Secretion, grade 1.
Prostate, dorso-lateral, fixed
Immature stage.
Seminal vesicle
Immature stage.
All other organs examined without microscopic findings.

Animal 348

General information
Sex : Male
Group : 4 (Flutamide 0.00025 mg/kg)
Sacrifice : Subset 1 (PND 21)
Necropsy status : Planned sacrifice
Date of death : 10.Apr.2013

Macroscopic findings
Animal without particular findings.

Microscopic findings
Coagulating glands
Immature stage.
Left epididymis
Immature stage.
Left testicle
Immature stage.
Prostata ventral fixed
Immature stage.
Secretion, grade 1.
Prostate, dorso-lateral, fixed
Immature stage.
Seminal vesicle
Immature stage.
All other organs examined without microscopic findings.

Animal 349

General information
Sex : Male
Group : 4 (Flutamide 0.00025 mg/kg)
Sacrifice : Subset 1 (PND 21)
Necropsy status : Planned sacrifice
Date of death : 12.Apr.2013

SINGLE ANIMAL SHEET
(GROSS LESIONS AND MICROSCOPIC FINDINGS)

Sacrifice R1
Sex M
Group 4
cont. Animal 349

Macroscopic findings
  Animal without particular findings.

Microscopic findings
  Coagulating glands
    Immature stage.
  Left epididymis
    Immature stage.
  Left testicle
    Immature stage.
  Prostata ventral fixed
    Immature stage.
    Secretion, grade 1.
  Prostate, dorso-lateral, fixed
    Immature stage.
  Seminal vesicle
    Immature stage.
  All other organs examined without microscopic findings.

Animal 350

General information
  Sex : Male
  Group : 4 (Flutamide 0.00025 mg/kg)
  Sacrifice : Subset 1 (PND 21)
  Necropsy status : Planned sacrifice
  Date of death : 12.Apr.2013

Macroscopic findings
  Animal without particular findings.

Microscopic findings
  Coagulating glands
    Immature stage.
  Left epididymis
    Immature stage.
  Left testicle
    Immature stage.
  Prostata ventral fixed
    Immature stage.
    Secretion, grade 1.
  Prostate, dorso-lateral, fixed
    Immature stage.
  Seminal vesicle
    Immature stage.
  All other organs examined without microscopic findings.

SINGLE ANIMAL SHEET
(GROSS LESIONS AND MICROSCOPIC FINDINGS)

Sacrifice	R1
Sex	F
Group	0
Animal	401

General information
Sex : Female
Group : 0 (0 mg/kg)
Sacrifice : Subset 1 (PND 21)
Necropsy status : Planned sacrifice
Date of death : 09.Apr.2013

Macroscopic findings
Animal without particular findings.

Microscopic findings
No histologic examination performed.

Animal 402

General information
Sex : Female
Group : 0 (0 mg/kg)
Sacrifice : Subset 1 (PND 21)
Necropsy status : Planned sacrifice
Date of death : 09.Apr.2013

Macroscopic findings
Animal without particular findings.

Microscopic findings
No histologic examination performed.

Animal 403

General information
Sex : Female
Group : 0 (0 mg/kg)
Sacrifice : Subset 1 (PND 21)
Necropsy status : Planned sacrifice
Date of death : 09.Apr.2013

Macroscopic findings
Animal without particular findings.

Microscopic findings
No histologic examination performed.

SINGLE ANIMAL SHEET
(GROSS LESIONS AND MICROSCOPIC FINDINGS)

Sacrifice	R1
Sex	F
Group	0
Animal	404

General information
- Sex : Female
- Group : 0 (0 mg/kg)
- Sacrifice : Subset 1 (PND 21)
- Necropsy status : Planned sacrifice
- Date of death : 10.Apr.2013

Macroscopic findings
Animal without particular findings.

Microscopic findings
No histologic examination performed.

Animal 405

General information
- Sex : Female
- Group : 0 (0 mg/kg)
- Sacrifice : Subset 1 (PND 21)
- Necropsy status : Planned sacrifice
- Date of death : 10.Apr.2013

Macroscopic findings
Animal without particular findings.

Microscopic findings
No histologic examination performed.

Animal 406

General information
- Sex : Female
- Group : 0 (0 mg/kg)
- Sacrifice : Subset 1 (PND 21)
- Necropsy status : Planned sacrifice
- Date of death : 10.Apr.2013

Macroscopic findings
Animal without particular findings.

Microscopic findings
No histologic examination performed.

SINGLE ANIMAL SHEET
(GROSS LESIONS AND MICROSCOPIC FINDINGS)

Sacrifice	R1
Sex	F
Group	0
Animal	407

General information
Sex : Female
Group : 0 (0 mg/kg)
Sacrifice : Subset 1 (PND 21)
Necropsy status : Planned sacrifice
Date of death : 11.Apr.2013

Macroscopic findings
Animal without particular findings.

Microscopic findings
No histologic examination performed.

Animal 408

General information
Sex : Female
Group : 0 (0 mg/kg)
Sacrifice : Subset 1 (PND 21)
Necropsy status : Planned sacrifice
Date of death : 11.Apr.2013

Macroscopic findings
Animal without particular findings.

Microscopic findings
No histologic examination performed.

Animal 409

General information
Sex : Female
Group : 0 (0 mg/kg)
Sacrifice : Subset 1 (PND 21)
Necropsy status : Planned sacrifice
Date of death : 11.Apr.2013

Macroscopic findings
Animal without particular findings.

Microscopic findings
No histologic examination performed.

Reproductive Toxicity Study to detect potential effects to anti-androgenic substances in Wistar Rats (Gavage) 28.Mar.2014 SIGR

SINGLE ANIMAL SHEET
(GROSS LESIONS AND MICROSCOPIC FINDINGS)

Sacrifice	R1
Sex	F
Group	0
Animal	410

General information

Sex	: Female
Group	: 0 (0 mg/kg)
Sacrifice	: Subset 1 (PND 21)
Necropsy status	: Planned sacrifice
Date of death	: 12.Apr.2013

Macroscopic findings

Animal without particular findings.

Microscopic findings

No histologic examination performed.

SINGLE ANIMAL SHEET
(GROSS LESIONS AND MICROSCOPIC FINDINGS)

Sacrifice R1
Sex F
Group 1
Animal 411

General information
Sex : Female
Group : 1 (0.005RON/0.00025FLT/0.01 590F mg/kg)
Sacrifice : Subset 1 (PND 21)
Necropsy status : Planned sacrifice
Date of death : 08.Apr.2013

Macroscopic findings
Animal without particular findings.

Microscopic findings
No histologic examination performed.

Animal 412

General information
Sex : Female
Group : 1 (0.005RON/0.00025FLT/0.01 590F mg/kg)
Sacrifice : Subset 1 (PND 21)
Necropsy status : Planned sacrifice
Date of death : 09.Apr.2013

Macroscopic findings
Animal without particular findings.

Microscopic findings
No histologic examination performed.

Animal 413

General information
Sex : Female
Group : 1 (0.005RON/0.00025FLT/0.01 590F mg/kg)
Sacrifice : Subset 1 (PND 21)
Necropsy status : Planned sacrifice
Date of death : 09.Apr.2013

Macroscopic findings
Animal without particular findings.

Microscopic findings
No histologic examination performed.

SINGLE ANIMAL SHEET
(GROSS LESIONS AND MICROSCOPIC FINDINGS)

Sacrifice R1
Sex F
Group 1
Animal 414

General information
Sex : Female
Group : 1 (0.005RON/0.00025FLT/0.01 590F mg/kg)
Sacrifice : Subset 1 (PND 21)
Necropsy status : Planned sacrifice
Date of death : 09.Apr.2013

Macroscopic findings
Animal without particular findings.

Microscopic findings
No histologic examination performed.

Animal 415

General information
Sex : Female
Group : 1 (0.005RON/0.00025FLT/0.01 590F mg/kg)
Sacrifice : Subset 1 (PND 21)
Necropsy status : Planned sacrifice
Date of death : 10.Apr.2013

Macroscopic findings
Animal without particular findings.

Microscopic findings
No histologic examination performed.

Animal 416

General information
Sex : Female
Group : 1 (0.005RON/0.00025FLT/0.01 590F mg/kg)
Sacrifice : Subset 1 (PND 21)
Necropsy status : Planned sacrifice
Date of death : 10.Apr.2013

Macroscopic findings
Animal without particular findings.

Microscopic findings
No histologic examination performed.

SINGLE ANIMAL SHEET
(GROSS LESIONS AND MICROSCOPIC FINDINGS)

Sacrifice R1
Sex F
Group 1
Animal 417

General information
Sex : Female
Group : 1 (0.005RON/0.00025FLT/0.01 590F mg/kg)
Sacrifice : Subset 1 (PND 21)
Necropsy status : Planned sacrifice
Date of death : 11.Apr.2013

Macroscopic findings
Animal without particular findings.

Microscopic findings
No histologic examination performed.

Animal 418

General information
Sex : Female
Group : 1 (0.005RON/0.00025FLT/0.01 590F mg/kg)
Sacrifice : Subset 1 (PND 21)
Necropsy status : Planned sacrifice
Date of death : 11.Apr.2013

Macroscopic findings
Animal without particular findings.

Microscopic findings
No histologic examination performed.

Animal 419

General information
Sex : Female
Group : 1 (0.005RON/0.00025FLT/0.01 590F mg/kg)
Sacrifice : Subset 1 (PND 21)
Necropsy status : Planned sacrifice
Date of death : 11.Apr.2013

Macroscopic findings
Animal without particular findings.

Microscopic findings
No histologic examination performed.

SINGLE ANIMAL SHEET
(GROSS LESIONS AND MICROSCOPIC FINDINGS)

Sacrifice	R1
Sex	F
Group	1
Animal	420

General information
Sex : Female
Group : 1 (0.005RON/0.00025FLT/0.01 590F mg/kg)
Sacrifice : Subset 1 (PND 21)
Necropsy status : Planned sacrifice
Date of death : 12.Apr.2013

Macroscopic findings
Animal without particular findings.

Microscopic findings
No histologic examination performed.

SINGLE ANIMAL SHEET
(GROSS LESIONS AND MICROSCOPIC FINDINGS)

Sacrifice R1
Sex F
Group 2
Animal 421

General information
Sex : Female
Group : 2 (4 RON/ 0.025 FLT/ 5 590F mg/kg)
Sacrifice : Subset 1 (PND 21)
Necropsy status : Planned sacrifice
Date of death : 09.Apr.2013

Macroscopic findings
Animal without particular findings.

Microscopic findings
No histologic examination performed.

Animal 422

General information
Sex : Female
Group : 2 (4 RON/ 0.025 FLT/ 5 590F mg/kg)
Sacrifice : Subset 1 (PND 21)
Necropsy status : Planned sacrifice
Date of death : 09.Apr.2013

Macroscopic findings
Animal without particular findings.

Microscopic findings
No histologic examination performed.

Animal 423

General information
Sex : Female
Group : 2 (4 RON/ 0.025 FLT/ 5 590F mg/kg)
Sacrifice : Subset 1 (PND 21)
Necropsy status : Planned sacrifice
Date of death : 09.Apr.2013

Macroscopic findings
Animal without particular findings.

Microscopic findings
No histologic examination performed.

SINGLE ANIMAL SHEET
(GROSS LESIONS AND MICROSCOPIC FINDINGS)

Sacrifice R1
Sex F
Group 2
Animal 424

General information
Sex : Female
Group : 2 (4 RON/ 0.025 FLT/ 5 590F mg/kg)
Sacrifice : Subset 1 (PND 21)
Necropsy status : Planned sacrifice
Date of death : 10.Apr.2013

Macroscopic findings
Animal without particular findings.

Microscopic findings
No histologic examination performed.

Animal 425

General information
Sex : Female
Group : 2 (4 RON/ 0.025 FLT/ 5 590F mg/kg)
Sacrifice : Subset 1 (PND 21)
Necropsy status : Planned sacrifice
Date of death : 10.Apr.2013

Macroscopic findings
Animal without particular findings.

Microscopic findings
No histologic examination performed.

Animal 426

General information
Sex : Female
Group : 2 (4 RON/ 0.025 FLT/ 5 590F mg/kg)
Sacrifice : Subset 1 (PND 21)
Necropsy status : Planned sacrifice
Date of death : 11.Apr.2013

Macroscopic findings
Animal without particular findings.

Microscopic findings
No histologic examination performed.

SINGLE ANIMAL SHEET
(GROSS LESIONS AND MICROSCOPIC FINDINGS)

Sacrifice R1
Sex F
Group 2
Animal 427

General information
Sex : Female
Group : 2 (4 RON/ 0.025 FLT/ 5 590F mg/kg)
Sacrifice : Subset 1 (PND 21)
Necropsy status : Planned sacrifice
Date of death : 11.Apr.2013

Macroscopic findings
Animal without particular findings.

Microscopic findings
No histologic examination performed.

Animal 428

General information
Sex : Female
Group : 2 (4 RON/ 0.025 FLT/ 5 590F mg/kg)
Sacrifice : Subset 1 (PND 21)
Necropsy status : Planned sacrifice
Date of death : 12.Apr.2013

Macroscopic findings
Animal without particular findings.

Microscopic findings
No histologic examination performed.

Animal 429

General information
Sex : Female
Group : 2 (4 RON/ 0.025 FLT/ 5 590F mg/kg)
Sacrifice : Subset 1 (PND 21)
Necropsy status : Planned sacrifice
Date of death : 13.Apr.2013

Macroscopic findings
Animal without particular findings.

Microscopic findings
No histologic examination performed.

Reproductive Toxicity Study to detect potential effects to anti-androgenic substances in Wistar Rats (Gavage) 28.Mar.2014 SIGR

SINGLE ANIMAL SHEET
(GROSS LESIONS AND MICROSCOPIC FINDINGS)

Sacrifice	R1
Sex	F
Group	2
Animal	430

General information

Sex : Female
Group : 2 (4 RON/ 0.025 FLT/ 5 590F mg/kg)
Sacrifice : Subset 1 (PND 21)
Necropsy status : Planned sacrifice
Date of death : 13.Apr.2013

Macroscopic findings

Animal without particular findings.

Microscopic findings

No histologic examination performed.

SINGLE ANIMAL SHEET
(GROSS LESIONS AND MICROSCOPIC FINDINGS)

Sacrifice	R1
Sex	F
Group	3
Animal	431

General information
Sex : Female
Group : 3 (20 RON/ 0.25 FLT/ 30 590F mg/kg)
Sacrifice : Subset 1 (PND 21)
Necropsy status : Planned sacrifice
Date of death : 09.Apr.2013

Macroscopic findings
Animal without particular findings.

Microscopic findings
No histologic examination performed.

Animal 432

General information
Sex : Female
Group : 3 (20 RON/ 0.25 FLT/ 30 590F mg/kg)
Sacrifice : Subset 1 (PND 21)
Necropsy status : Planned sacrifice
Date of death : 11.Apr.2013

Macroscopic findings
Animal without particular findings.

Microscopic findings
No histologic examination performed.

Animal 433

General information
Sex : Female
Group : 3 (20 RON/ 0.25 FLT/ 30 590F mg/kg)
Sacrifice : Subset 1 (PND 21)
Necropsy status : Planned sacrifice
Date of death : 11.Apr.2013

Macroscopic findings
Animal without particular findings.

Microscopic findings
No histologic examination performed.

SINGLE ANIMAL SHEET
(GROSS LESIONS AND MICROSCOPIC FINDINGS)

Sacrifice R1
Sex F
Group 3
Animal 434

General information
- Sex : Female
- Group : 3 (20 RON/ 0.25 FLT/ 30 590F mg/kg)
- Sacrifice : Subset 1 (PND 21)
- Necropsy status : Planned sacrifice
- Date of death : 11.Apr.2013

Macroscopic findings
Animal without particular findings.

Microscopic findings
No histologic examination performed.

Animal 435

General information
- Sex : Female
- Group : 3 (20 RON/ 0.25 FLT/ 30 590F mg/kg)
- Sacrifice : Subset 1 (PND 21)
- Necropsy status : Planned sacrifice
- Date of death : 11.Apr.2013

Macroscopic findings
Animal without particular findings.

Microscopic findings
No histologic examination performed.

Animal 436

General information
- Sex : Female
- Group : 3 (20 RON/ 0.25 FLT/ 30 590F mg/kg)
- Sacrifice : Subset 1 (PND 21)
- Necropsy status : Planned sacrifice
- Date of death : 12.Apr.2013

Macroscopic findings
Animal without particular findings.

Microscopic findings
No histologic examination performed.

SINGLE ANIMAL SHEET
(GROSS LESIONS AND MICROSCOPIC FINDINGS)

Sacrifice	R1
Sex	F
Group	3
Animal	437

General information
Sex : Female
Group : 3 (20 RON/ 0.25 FLT/ 30 590F mg/kg)
Sacrifice : Subset 1 (PND 21)
Necropsy status : Planned sacrifice
Date of death : 12.Apr.2013

Macroscopic findings
Animal without particular findings.

Microscopic findings
No histologic examination performed.

Animal 438

General information
Sex : Female
Group : 3 (20 RON/ 0.25 FLT/ 30 590F mg/kg)
Sacrifice : Subset 1 (PND 21)
Necropsy status : Planned sacrifice
Date of death : 13.Apr.2013

Macroscopic findings
Animal without particular findings.

Microscopic findings
No histologic examination performed.

Animal 439

General information
Sex : Female
Group : 3 (20 RON/ 0.25 FLT/ 30 590F mg/kg)
Sacrifice : Subset 1 (PND 21)
Necropsy status : Planned sacrifice
Date of death : 13.Apr.2013

Macroscopic findings
Animal without particular findings.

Microscopic findings
No histologic examination performed.

SINGLE ANIMAL SHEET
(GROSS LESIONS AND MICROSCOPIC FINDINGS)

Sacrifice	R1
Sex	F
Group	3
Animal	440

General information

Sex : Female
Group : 3 (20 RON/ 0.25 FLT/ 30 590F mg/kg)
Sacrifice : Subset 1 (PND 21)
Necropsy status : Planned sacrifice
Date of death : 13.Apr.2013

Macroscopic findings

Animal without particular findings.

Microscopic findings

No histologic examination performed.

SINGLE ANIMAL SHEET
(GROSS LESIONS AND MICROSCOPIC FINDINGS)

Sacrifice R1
Sex F
Group 4
Animal 441

General information
Sex : Female
Group : 4 (Flutamide 0.00025 mg/kg)
Sacrifice : Subset 1 (PND 21)
Necropsy status : Planned sacrifice
Date of death : 08.Apr.2013

Macroscopic findings
Animal without particular findings.

Microscopic findings
No histologic examination performed.

Animal 442

General information
Sex : Female
Group : 4 (Flutamide 0.00025 mg/kg)
Sacrifice : Subset 1 (PND 21)
Necropsy status : Planned sacrifice
Date of death : 09.Apr.2013

Macroscopic findings
Animal without particular findings.

Microscopic findings
No histologic examination performed.

Animal 443

General information
Sex : Female
Group : 4 (Flutamide 0.00025 mg/kg)
Sacrifice : Subset 1 (PND 21)
Necropsy status : Planned sacrifice
Date of death : 10.Apr.2013

Macroscopic findings
Animal without particular findings.

Microscopic findings
No histologic examination performed.

SINGLE ANIMAL SHEET
(GROSS LESIONS AND MICROSCOPIC FINDINGS)

Sacrifice R1
Sex F
Group 4
Animal 444

General information
Sex : Female
Group : 4 (Flutamide 0.00025 mg/kg)
Sacrifice : Subset 1 (PND 21)
Necropsy status : Planned sacrifice
Date of death : 10.Apr.2013

Macroscopic findings
Animal without particular findings.

Microscopic findings
No histologic examination performed.

Animal 445

General information
Sex : Female
Group : 4 (Flutamide 0.00025 mg/kg)
Sacrifice : Subset 1 (PND 21)
Necropsy status : Planned sacrifice
Date of death : 11.Apr.2013

Macroscopic findings
Animal without particular findings.

Microscopic findings
No histologic examination performed.

Animal 446

General information
Sex : Female
Group : 4 (Flutamide 0.00025 mg/kg)
Sacrifice : Subset 1 (PND 21)
Necropsy status : Planned sacrifice
Date of death : 11.Apr.2013

Macroscopic findings
Animal without particular findings.

Microscopic findings
No histologic examination performed.

SINGLE ANIMAL SHEET
(GROSS LESIONS AND MICROSCOPIC FINDINGS)

Sacrifice R1
Sex F
Group 4
Animal 447

General information
Sex : Female
Group : 4 (Flutamide 0.00025 mg/kg)
Sacrifice : Subset 1 (PND 21)
Necropsy status : Planned sacrifice
Date of death : 12.Apr.2013

Macroscopic findings
Animal without particular findings.

Microscopic findings
No histologic examination performed.

Animal 448

General information
Sex : Female
Group : 4 (Flutamide 0.00025 mg/kg)
Sacrifice : Subset 1 (PND 21)
Necropsy status : Planned sacrifice
Date of death : 12.Apr.2013

Macroscopic findings
Animal without particular findings.

Microscopic findings
No histologic examination performed.

Animal 449

General information
Sex : Female
Group : 4 (Flutamide 0.00025 mg/kg)
Sacrifice : Subset 1 (PND 21)
Necropsy status : Planned sacrifice
Date of death : 12.Apr.2013

Macroscopic findings
Animal without particular findings.

Microscopic findings
No histologic examination performed.

SINGLE ANIMAL SHEET
(GROSS LESIONS AND MICROSCOPIC FINDINGS)

Sacrifice	R1
Sex	F
Group	4
Animal	450

General information
- Sex : Female
- Group : 4 (Flutamide 0.00025 mg/kg)
- Sacrifice : Subset 1 (PND 21)
- Necropsy status : Planned sacrifice
- Date of death : 12.Apr.2013

Macroscopic findings
Animal without particular findings.

Microscopic findings
No histologic examination performed.

SINGLE ANIMAL SHEET
(GROSS LESIONS AND MICROSCOPIC FINDINGS)

Sacrifice R2
Sex M
Group 0
Animal 501

General information
Sex : Male
Group : 0 (0 mg/kg)
Sacrifice : Subset 2 (sexual maturity)
Necropsy status : Planned sacrifice
Date of death : 08.May.2013
29 days after start of exposure
1 day after end of exposure

Macroscopic findings
Animal without particular findings.

Microscopic findings
Seminal vesicle
Juvenile stage with secretion.
All other organs examined without microscopic findings.

Animal 502

General information
Sex : Male
Group : 0 (0 mg/kg)
Sacrifice : Subset 2 (sexual maturity)
Necropsy status : Planned sacrifice
Date of death : 02.May.2013
23 days after start of exposure
1 day after end of exposure

Macroscopic findings
Animal without particular findings.

Microscopic findings
Coagulating glands
Juvenile stage with secretion.
Left epididymis
Juvenile stage without sperms.
Left testicle
Spermatogenic cycle, developed.
Prostata ventral fixed
Juvenile stage with secretion.
Prostate, dorso-lateral, fixed
Juvenile stage with secretion.
Seminal vesicle
Juvenile stage with secretion.
All other organs examined without microscopic findings.

SINGLE ANIMAL SHEET
(GROSS LESIONS AND MICROSCOPIC FINDINGS)

Sacrifice R2
Sex M
Group 0
Animal 503

General information
- Sex : Male
- Group : 0 (0 mg/kg)
- Sacrifice : Subset 2 (sexual maturity)
- Necropsy status : Planned sacrifice
- Date of death : 30.Apr.2013
  21 days after start of exposure
  1 day after end of exposure

Macroscopic findings
Animal without particular findings.

Microscopic findings
- Coagulating glands
  Juvenile stage with secretion.
- Left epididymis
  Juvenile stage without sperms.
- Left testicle
  Spermatogenic cycle, developed.
- Prostata ventral fixed
  Juvenile stage with secretion.
- Prostate, dorso-lateral, fixed
  Juvenile stage with secretion.
- Seminal vesicle
  Juvenile stage with secretion.
- All other organs examined without microscopic findings.

Animal 504

General information
- Sex : Male
- Group : 0 (0 mg/kg)
- Sacrifice : Subset 2 (sexual maturity)
- Necropsy status : Planned sacrifice
- Date of death : 08.May.2013
  28 days after start of exposure
  1 day after end of exposure

Macroscopic findings
Animal without particular findings.

SINGLE ANIMAL SHEET
(GROSS LESIONS AND MICROSCOPIC FINDINGS)

Sacrifice R2
Sex M
Group 0
cont. Animal 504

Microscopic findings
  Coagulating glands
    Juvenile stage with secretion.
  Left epididymis
    Juvenile stage, with sperms, grade 1.
  * Caput.
  Left testicle
    Spermatogenic cycle, developed.
  Prostata ventral fixed
    Juvenile stage with secretion.
  Prostate, dorso-lateral, fixed
    Juvenile stage with secretion.
  Seminal vesicle
    Juvenile stage with secretion.
  All other organs examined without microscopic findings.

Animal 505

General information
  Sex : Male
  Group : 0 (0 mg/kg)
  Sacrifice : Subset 2 (sexual maturity)
  Necropsy status : Planned sacrifice
  Date of death : 06.May.2013
    26 days after start of exposure
    1 day after end of exposure

Macroscopic findings
  Animal without particular findings.

Microscopic findings
  Coagulating glands
    Juvenile stage with secretion.
  Left epididymis
    Juvenile stage, with sperms, grade 1.
  * Caput.
  Left testicle
    Spermatogenic cycle, developed.
  Prostata ventral fixed
    Juvenile stage with secretion.
  Prostate, dorso-lateral, fixed
    Juvenile stage with secretion.
  Seminal vesicle
    Juvenile stage with secretion.
  All other organs examined without microscopic findings.

SINGLE ANIMAL SHEET
(GROSS LESIONS AND MICROSCOPIC FINDINGS)

Sacrifice R2
Sex M
Group 0
Animal 506

General information
Sex : Male
Group : 0 (0 mg/kg)
Sacrifice : Subset 2 (sexual maturity)
Necropsy status : Planned sacrifice
Date of death : 01.May.2013
21 days after start of exposure
1 day after end of exposure

Macroscopic findings
Animal without particular findings.

Microscopic findings
Coagulating glands
Juvenile stage with secretion.
Left epididymis
Juvenile stage without sperms.
Left testicle
Spermatogenic cycle, developed.
Pituitary gland
Cyst(s), craniopharyngeal, pars nervosa.
Prostata ventral fixed
Juvenile stage with secretion.
Prostate, dorso-lateral, fixed
Juvenile stage with secretion.
Seminal vesicle
Juvenile stage with secretion.
All other organs examined without microscopic findings.

Animal 507

General information
Sex : Male
Group : 0 (0 mg/kg)
Sacrifice : Subset 2 (sexual maturity)
Necropsy status : Planned sacrifice
Date of death : 08.May.2013
27 days after start of exposure
1 day after end of exposure

Macroscopic findings
Animal without particular findings.

SINGLE ANIMAL SHEET
(GROSS LESIONS AND MICROSCOPIC FINDINGS)

Sacrifice	R2
Sex	M
Group	0
cont. Animal	507

Microscopic findings
  Coagulating glands
    Juvenile stage with secretion.
  Left epididymis
    Juvenile stage, with sperms, grade 1.
  * Caput and proximal part of corpus.
  Left testicle
    Spermatogenic cycle, developed.
  Prostata ventral fixed
    Juvenile stage with secretion.
  Prostate, dorso-lateral, fixed
    Juvenile stage with secretion.
  Seminal vesicle
    Juvenile stage with secretion.
  All other organs examined without microscopic findings.

Animal 508

General information
  Sex : Male
  Group : 0 (0 mg/kg)
  Sacrifice : Subset 2 (sexual maturity)
  Necropsy status : Planned sacrifice
  Date of death : 09.May.2013
    28 days after start of exposure
    1 day after end of exposure

Macroscopic findings
  Animal without particular findings.

Microscopic findings
  Coagulating glands
    Juvenile stage with secretion.
  Left epididymis
    Juvenile stage, with sperms, grade 2.
  * Caput, corpus and cauda.
  Left testicle
    Spermatogenic cycle, developed.
  Prostata ventral fixed
    Juvenile stage with secretion.
  Prostate, dorso-lateral, fixed
    Juvenile stage with secretion.
  Seminal vesicle
    Juvenile stage with secretion.
  All other organs examined without microscopic findings.

SINGLE ANIMAL SHEET
(GROSS LESIONS AND MICROSCOPIC FINDINGS)

Sacrifice R2
Sex M
Group 0
Animal 509

General information
  Sex : Male
  Group : 0 (0 mg/kg)
  Sacrifice : Subset 2 (sexual maturity)
  Necropsy status : Planned sacrifice
  Date of death : 08.May.2013
  27 days after start of exposure
  1 day after end of exposure

Macroscopic findings
  Animal without particular findings.

Microscopic findings
  Coagulating glands
    Juvenile stage with secretion.
  Left epididymis
    Juvenile stage, with sperms, grade 2.
  * Caput, corpus, cauda.
  Left testicle
    Spermatogenic cycle, developed.
  Prostata ventral fixed
    Juvenile stage with secretion.
  Prostate, dorso-lateral, fixed
    Juvenile stage with secretion.
  Seminal vesicle
    Juvenile stage with secretion.
  All other organs examined without microscopic findings.

Animal 510

General information
  Sex : Male
  Group : 0 (0 mg/kg)
  Sacrifice : Subset 2 (sexual maturity)
  Necropsy status : Planned sacrifice
  Date of death : 08.May.2013
  26 days after start of exposure
  1 day after end of exposure

Macroscopic findings
  Animal without particular findings.

Reproductive Toxicity Study to detect potential effects to anti-androgenic substances in Wistar Rats (Gavage) 28.Mar.2014 SIGR

SINGLE ANIMAL SHEET
(GROSS LESIONS AND MICROSCOPIC FINDINGS)

Sacrifice	R2
Sex	M
Group	0
cont. Animal	510

Microscopic findings
  Coagulating glands
    Juvenile stage with secretion.
  Left epididymis
    Juvenile stage, with sperms, grade 1.
  * Caput and proximal part of corpus.
  Left testicle
    Spermatogenic cycle, developed.
  Prostata ventral fixed
    Juvenile stage with secretion.
  Prostate, dorso-lateral, fixed
    Juvenile stage with secretion.
  Seminal vesicle
    Juvenile stage with secretion.
  All other organs examined without microscopic findings.

SINGLE ANIMAL SHEET
(GROSS LESIONS AND MICROSCOPIC FINDINGS)

Sacrifice R2
Sex M
Group 1
Animal 511

General information
  Sex : Male
  Group : 1 (0.005RON/0.00025FLT/0.01 590F mg/kg)
  Sacrifice : Subset 2 (sexual maturity)
  Necropsy status : Planned sacrifice
  Date of death : 06.May.2013
    27 days after start of exposure
    1 day after end of exposure

Macroscopic findings
  Animal without particular findings.

Microscopic findings
  Coagulating glands
    Juvenile stage with secretion.
  Left epididymis
    Juvenile stage, with sperms, grade 1.
  * Caput .
  Left testicle
    Spermatogenic cycle, developed.
  Prostata ventral fixed
    Juvenile stage with secretion.
  Prostate, dorso-lateral, fixed
    Juvenile stage with secretion.
  Seminal vesicle
    Juvenile stage with secretion.
  All other organs examined without microscopic findings.

Animal 512

General information
  Sex : Male
  Group : 1 (0.005RON/0.00025FLT/0.01 590F mg/kg)
  Sacrifice : Subset 2 (sexual maturity)
  Necropsy status : Planned sacrifice
  Date of death : 30.Apr.2013
    21 days after start of exposure
    1 day after end of exposure

Macroscopic findings
  Animal without particular findings.

SINGLE ANIMAL SHEET
(GROSS LESIONS AND MICROSCOPIC FINDINGS)

Sacrifice	R2
Sex	M
Group	1
cont. Animal	512

Microscopic findings
  Coagulating glands
    Juvenile stage with secretion.
  Left epididymis
    Juvenile stage without sperms.
  Left testicle
    Spermatogenic cycle, developed.
  Prostata ventral fixed
    Juvenile stage with secretion.
  Prostate, dorso-lateral, fixed
    Juvenile stage with secretion.
  Seminal vesicle
    Juvenile stage with secretion.
  All other organs examined without microscopic findings.

Animal 513

General information
  Sex : Male
  Group : 1 (0.005RON/0.00025FLT/0.01 590F mg/kg)
  Sacrifice : Subset 2 (sexual maturity)
  Necropsy status : Planned sacrifice
  Date of death : 30.Apr.2013
    21 days after start of exposure
    1 day after end of exposure

Macroscopic findings
  Animal without particular findings.

Microscopic findings
  Coagulating glands
    Juvenile stage with secretion.
  Left epididymis
    Juvenile stage without sperms.
  Left testicle
    Spermatogenic cycle, developed.
  Prostata ventral fixed
    Juvenile stage with secretion.
  Prostate, dorso-lateral, fixed
    Juvenile stage with secretion.
  Seminal vesicle
    Juvenile stage with secretion.
  All other organs examined without microscopic findings.

SINGLE ANIMAL SHEET
(GROSS LESIONS AND MICROSCOPIC FINDINGS)

Sacrifice R2
Sex M
Group 1
Animal 514

General information
Sex : Male
Group : 1 (0.005RON/0.00025FLT/0.01 590F mg/kg)
Sacrifice : Subset 2 (sexual maturity)
Necropsy status : Planned sacrifice
Date of death : 07.May.2013
27 days after start of exposure
1 day after end of exposure

Macroscopic findings
Animal without particular findings.

Microscopic findings
Coagulating glands
Juvenile stage with secretion.
Left epididymis
Juvenile stage, with sperms, grade 1.
* Caput.
Left testicle
Spermatogenic cycle, developed.
Prostata ventral fixed
Juvenile stage with secretion.
Prostate, dorso-lateral, fixed
Juvenile stage with secretion.
Seminal vesicle
Juvenile stage with secretion.
All other organs examined without microscopic findings.

Animal 515

General information
Sex : Male
Group : 1 (0.005RON/0.00025FLT/0.01 590F mg/kg)
Sacrifice : Subset 2 (sexual maturity)
Necropsy status : Planned sacrifice
Date of death : 04.May.2013
24 days after start of exposure
1 day after end of exposure

Macroscopic findings
Animal without particular findings.

SINGLE ANIMAL SHEET
(GROSS LESIONS AND MICROSCOPIC FINDINGS)

Sacrifice R2
Sex M
Group 1
cont. Animal 515

Microscopic findings
  Coagulating glands
    Juvenile stage with secretion.
  Left epididymis
    Juvenile stage, with sperms, grade 1.
  * Caput.
  Left testicle
    Spermatogenic cycle, developed.
  Prostata ventral fixed
    Juvenile stage with secretion.
  Prostate, dorso-lateral, fixed
    Juvenile stage with secretion.
  Seminal vesicle
    Juvenile stage with secretion.
  All other organs examined without microscopic findings.

Animal 516

General information
  Sex : Male
  Group : 1 (0.005RON/0.00025FLT/0.01 590F mg/kg)
  Sacrifice : Subset 2 (sexual maturity)
  Necropsy status : Planned sacrifice
  Date of death : 02.May.2013
    22 days after start of exposure
    1 day after end of exposure

Macroscopic findings
  Animal without particular findings.

Microscopic findings
  Coagulating glands
    Juvenile stage with secretion.
  Left epididymis
    Juvenile stage without sperms.
  Left testicle
    Spermatogenic cycle, developed.
  Prostata ventral fixed
    Juvenile stage with secretion.
  Prostate, dorso-lateral, fixed
    Juvenile stage with secretion.
  Seminal vesicle
    Juvenile stage with secretion.
  All other organs examined without microscopic findings.

SINGLE ANIMAL SHEET
(GROSS LESIONS AND MICROSCOPIC FINDINGS)

Sacrifice R2
Sex M
Group 1
Animal 517

General information
Sex : Male
Group : 1 (0.005RON/0.00025FLT/0.01 590F mg/kg)
Sacrifice : Subset 2 (sexual maturity)
Necropsy status : Planned sacrifice
Date of death : 07.May.2013
27 days after start of exposure
1 day after end of exposure

Macroscopic findings
Animal without particular findings.

Microscopic findings
Coagulating glands
Juvenile stage with secretion.
Left epididymis
Juvenile stage, with sperms, grade 1.
* Caput.
Left testicle
Spermatogenic cycle, developed.
Prostata ventral fixed
Juvenile stage with secretion.
Prostate, dorso-lateral, fixed
Juvenile stage with secretion.
Seminal vesicle
Juvenile stage with secretion.
All other organs examined without microscopic findings.

Animal 518

General information
Sex : Male
Group : 1 (0.005RON/0.00025FLT/0.01 590F mg/kg)
Sacrifice : Subset 2 (sexual maturity)
Necropsy status : Planned sacrifice
Date of death : 09.May.2013
28 days after start of exposure
1 day after end of exposure

Macroscopic findings
Animal without particular findings.

Reproductive Toxicity Study to detect potential effects to anti-androgenic substances in Wistar Rats (Gavage) 28.Mar.2014 SIGR

SINGLE ANIMAL SHEET
(GROSS LESIONS AND MICROSCOPIC FINDINGS)

Sacrifice	R2
Sex	M
Group	1
cont. Animal	518

Microscopic findings
  Coagulating glands
    Juvenile stage with secretion.
  Left epididymis
    Juvenile stage, with sperms, grade 1.
  * Caput.
  Left testicle
    Spermatogenic cycle, developed.
  Prostata ventral fixed
    Juvenile stage with secretion.
  Prostate, dorso-lateral, fixed
    Juvenile stage with secretion.
  Seminal vesicle
    Juvenile stage with secretion.
  All other organs examined without microscopic findings.

Animal 519

General information
  Sex : Male
  Group : 1 (0.005RON/0.00025FLT/0.01 590F mg/kg)
  Sacrifice : Subset 2 (sexual maturity)
  Necropsy status : Planned sacrifice
  Date of death : 05.May.2013
    24 days after start of exposure
    1 day after end of exposure

Macroscopic findings
  Animal without particular findings.

Microscopic findings
  Coagulating glands
    Juvenile stage with secretion.
  Left epididymis
    Juvenile stage without sperms.
  Left testicle
    Spermatogenic cycle, developed.
  Prostata ventral fixed
    Juvenile stage with secretion.
  Prostate, dorso-lateral, fixed
    Juvenile stage with secretion.
  Seminal vesicle
    Juvenile stage with secretion.
  All other organs examined without microscopic findings.

Reproductive Toxicity Study to detect potential effects to anti-androgenic substances in Wistar Rats (Gavage) 28.Mar.2014 SIGR

SINGLE ANIMAL SHEET
(GROSS LESIONS AND MICROSCOPIC FINDINGS)

Sacrifice	R2
Sex	M
Group	1
Animal	520

General information
- Sex : Male
- Group : 1 (0.005RON/0.00025FLT/0.01 590F mg/kg)
- Sacrifice : Subset 2 (sexual maturity)
- Necropsy status : Planned sacrifice
- Date of death : 08.May.2013
  26 days after start of exposure
  1 day after end of exposure

Macroscopic findings
  Animal without particular findings.

Microscopic findings
  Coagulating glands
    Juvenile stage with secretion.
  Left epididymis
    Juvenile stage, with sperms, grade 1.
  * Caput.
  Left testicle
    Spermatogenic cycle, developed.
  Prostata ventral fixed
    Juvenile stage with secretion.
  Prostate, dorso-lateral, fixed
    Juvenile stage with secretion.
  Seminal vesicle
    Juvenile stage with secretion.
  All other organs examined without microscopic findings.

SINGLE ANIMAL SHEET
(GROSS LESIONS AND MICROSCOPIC FINDINGS)

Sacrifice R2
Sex M
Group 2
Animal 521

General information
- Sex : Male
- Group : 2 (4 RON/ 0.025 FLT/ 5 590F mg/kg)
- Sacrifice : Subset 2 (sexual maturity)
- Necropsy status : Planned sacrifice
- Date of death : 30.Apr.2013
  21 days after start of exposure
  1 day after end of exposure

Macroscopic findings
  Animal without particular findings.

Microscopic findings
  Coagulating glands
    Juvenile stage with secretion.
  Left epididymis
    Juvenile stage without sperms.
  Left testicle
    Spermatogenic cycle, developed.
  Prostata ventral fixed
    Juvenile stage with secretion.
  Prostate, dorso-lateral, fixed
    Juvenile stage with secretion.
  Seminal vesicle
    Juvenile stage with secretion.
  All other organs examined without microscopic findings.

Animal 522

General information
- Sex : Male
- Group : 2 (4 RON/ 0.025 FLT/ 5 590F mg/kg)
- Sacrifice : Subset 2 (sexual maturity)
- Necropsy status : Planned sacrifice
- Date of death : 30.Apr.2013
  21 days after start of exposure
  1 day after end of exposure

Macroscopic findings
  Animal without particular findings.

SINGLE ANIMAL SHEET
(GROSS LESIONS AND MICROSCOPIC FINDINGS)

Sacrifice R2
Sex M
Group 2
cont. Animal 522

Microscopic findings
  Coagulating glands
    Juvenile stage with secretion.
  Left epididymis
    Juvenile stage without sperms.
  Left testicle
    Spermatogenic cycle, developed.
  Prostata ventral fixed
    Juvenile stage with secretion.
  Prostate, dorso-lateral, fixed
    Juvenile stage with secretion.
  Seminal vesicle
    Juvenile stage with secretion.
  All other organs examined without microscopic findings.

Animal 523

General information
  Sex : Male
  Group : 2 (4 RON/ 0.025 FLT/ 5 590F mg/kg)
  Sacrifice : Subset 2 (sexual maturity)
  Necropsy status : Planned sacrifice
  Date of death : 04.May.2013
    25 days after start of exposure
    1 day after end of exposure

Macroscopic findings
  Animal without particular findings.

Microscopic findings
  Coagulating glands
    Juvenile stage with secretion.
  Left epididymis
    Juvenile stage, with sperms, grade 1.
  * Caput.
  Left testicle
    Spermatogenic cycle, developed.
  Prostata ventral fixed
    Juvenile stage with secretion.
  Prostate, dorso-lateral, fixed
    Juvenile stage with secretion.
  Seminal vesicle
    Juvenile stage with secretion.
  All other organs examined without microscopic findings.

SINGLE ANIMAL SHEET
(GROSS LESIONS AND MICROSCOPIC FINDINGS)

Sacrifice R2
Sex M
Group 2
Animal 524

General information
  Sex : Male
  Group : 2 (4 RON/ 0.025 FLT/ 5 590F mg/kg)
  Sacrifice : Subset 2 (sexual maturity)
  Necropsy status : Planned sacrifice
  Date of death : 02.May.2013
    22 days after start of exposure
    1 day after end of exposure

Macroscopic findings
  Animal without particular findings.

Microscopic findings
  Coagulating glands
    Juvenile stage with secretion.
  Left epididymis
    Juvenile stage without sperms.
  Left testicle
    Spermatogenic cycle, developed.
  Prostata ventral fixed
    Juvenile stage with secretion.
  Prostate, dorso-lateral, fixed
    Juvenile stage with secretion.
  Seminal vesicle
    Juvenile stage with secretion.
  All other organs examined without microscopic findings.

Animal 525

General information
  Sex : Male
  Group : 2 (4 RON/ 0.025 FLT/ 5 590F mg/kg)
  Sacrifice : Subset 2 (sexual maturity)
  Necropsy status : Planned sacrifice
  Date of death : 05.May.2013
    25 days after start of exposure
    1 day after end of exposure

Macroscopic findings
  Kidneys
    Cyst, left side, diameter 1.0 mm.
  All other organs without macroscopic findings.

SINGLE ANIMAL SHEET
(GROSS LESIONS AND MICROSCOPIC FINDINGS)

Sacrifice R2
Sex M
Group 2
cont. Animal 525

Microscopic findings
  Coagulating glands
    Juvenile stage with secretion.
  Kidneys
    Gross lesion(s) evaluated histopathologically.
    Cyst(s), unilateral, correlates to gross lesion Cyst.
  Left epididymis
    Juvenile stage, with sperms, grade 1.
  * Caput.
  Left testicle
    Spermatogenic cycle, developed.
  Prostata ventral fixed
    Juvenile stage with secretion.
  Prostate, dorso-lateral, fixed
    Juvenile stage with secretion.
  Seminal vesicle
    Juvenile stage with secretion.
  All other organs examined without microscopic findings.

Animal 526

General information
  Sex : Male
  Group : 2 (4 RON/ 0.025 FLT/ 5 590F mg/kg)
  Sacrifice : Subset 2 (sexual maturity)
  Necropsy status : Planned sacrifice
  Date of death : 09.May.2013
    28 days after start of exposure
    1 day after end of exposure

Macroscopic findings
  Animal without particular findings.

Microscopic findings
  Adrenal medulla
    Unilaterally investigated or present. No histopathologic findings noted.
  Coagulating glands
    Juvenile stage with secretion.
  Left epididymis
    Juvenile stage, with sperms, grade 3.
  * Caput, corpus, cauda.
  Left testicle
    Spermatogenic cycle, developed.
  Prostata ventral fixed
    Juvenile stage with secretion.

SINGLE ANIMAL SHEET
(GROSS LESIONS AND MICROSCOPIC FINDINGS)

Sacrifice R2
Sex M
Group 2
cont. Animal 526

Prostate, dorso-lateral, fixed
Juvenile stage with secretion.
Seminal vesicle
Juvenile stage with secretion.
All other organs examined without microscopic findings.

Animal 527

General information
Sex : Male
Group : 2 (4 RON/ 0.025 FLT/ 5 590F mg/kg)
Sacrifice : Subset 2 (sexual maturity)
Necropsy status : Planned sacrifice
Date of death : 05.May.2013
24 days after start of exposure
1 day after end of exposure

Macroscopic findings
Animal without particular findings.

Microscopic findings
Coagulating glands
Juvenile stage with secretion.
Left epididymis
Juvenile stage without sperms.
Left testicle
Spermatogenic cycle, developed.
Prostata ventral fixed
Juvenile stage with secretion.
Prostate, dorso-lateral, fixed
Juvenile stage with secretion.
Seminal vesicle
Juvenile stage with secretion.
All other organs examined without microscopic findings.

Animal 528

General information
Sex : Male
Group : 2 (4 RON/ 0.025 FLT/ 5 590F mg/kg)
Sacrifice : Subset 2 (sexual maturity)
Necropsy status : Planned sacrifice
Date of death : 08.May.2013
26 days after start of exposure
1 day after end of exposure

SINGLE ANIMAL SHEET
(GROSS LESIONS AND MICROSCOPIC FINDINGS)

Sacrifice R2
Sex M
Group 2
cont. Animal 528

Macroscopic findings
  Animal without particular findings.

Microscopic findings
  Coagulating glands
    Juvenile stage with secretion.
  Left epididymis
    Juvenile stage, with sperms, grade 2.
  * Caput and proximal part of corpus.
  Left testicle
    Spermatogenic cycle, developed.
  Prostata ventral fixed
    Juvenile stage with secretion.
  Prostate, dorso-lateral, fixed
    Juvenile stage with secretion.
  Seminal vesicle
    Juvenile stage with secretion.
  All other organs examined without microscopic findings.

Animal 529

General information
  Sex : Male
  Group : 2 (4 RON/ 0.025 FLT/ 5 590F mg/kg)
  Sacrifice : Subset 2 (sexual maturity)
  Necropsy status : Planned sacrifice
  Date of death : 12.May.2013
    30 days after start of exposure
    1 day after end of exposure

Macroscopic findings
  Animal without particular findings.

Microscopic findings
  Coagulating glands
    Juvenile stage with secretion.
  Left epididymis
    Juvenile stage, with sperms, grade 3.
  * Caput, corpus, cauda.
  Left testicle
    Spermatogenic cycle, developed.
  Prostata ventral fixed
    Juvenile stage with secretion.
  Prostate, dorso-lateral, fixed
    Juvenile stage with secretion.

SINGLE ANIMAL SHEET
(GROSS LESIONS AND MICROSCOPIC FINDINGS)

Sacrifice	R2
Sex	M
Group	2
cont. Animal	529

Seminal vesicle
Juvenile stage with secretion.
All other organs examined without microscopic findings.

Animal 530

General information
Sex : Male
Group : 2 (4 RON/ 0.025 FLT/ 5 590F mg/kg)
Sacrifice : Subset 2 (sexual maturity)
Necropsy status : Planned sacrifice
Date of death : 10.May.2013
27 days after start of exposure
1 day after end of exposure

Macroscopic findings
Animal without particular findings.

Microscopic findings
Coagulating glands
Juvenile stage with secretion.
Left epididymis
Juvenile stage, with sperms, grade 1.
* Caput and proximal part of corpus.
Left testicle
Spermatogenic cycle, developed.
Prostata ventral fixed
Juvenile stage with secretion.
Prostate, dorso-lateral, fixed
Juvenile stage with secretion.
Seminal vesicle
Juvenile stage with secretion.
All other organs examined without microscopic findings.

SINGLE ANIMAL SHEET
(GROSS LESIONS AND MICROSCOPIC FINDINGS)

Sacrifice R2
Sex M
Group 3
Animal 531

General information
Sex : Male
Group : 3 (20 RON/ 0.25 FLT/ 30 590F mg/kg)
Sacrifice : Subset 2 (sexual maturity)
Necropsy status : Planned sacrifice
Date of death : 18.May.2013
39 days after start of exposure
1 day after end of exposure

Macroscopic findings
Animal without particular findings.

Microscopic findings
Coagulating glands
Juvenile stage with secretion.
Left epididymis
Juveno-adult transition, with sperms, grade 5.
* Caput, corpus, cauda.
Oligospermia, grade 1.
Left testicle
Spermatogenic cycle, developed.
Prostata ventral fixed
Juvenile stage with secretion.
Prostate, dorso-lateral, fixed
Juvenile stage with secretion.
Seminal vesicle
Juvenile stage with secretion.
All other organs examined without microscopic findings.

Animal 532

General information
Sex : Male
Group : 3 (20 RON/ 0.25 FLT/ 30 590F mg/kg)
Sacrifice : Subset 2 (sexual maturity)
Necropsy status : Planned sacrifice
Date of death : 10.May.2013
30 days after start of exposure
1 day after end of exposure

Macroscopic findings
Animal without particular findings.

SINGLE ANIMAL SHEET
(GROSS LESIONS AND MICROSCOPIC FINDINGS)

Sacrifice R2
Sex M
Group 3
cont. Animal 532

Microscopic findings
  Coagulating glands
    Juvenile stage with secretion.
  Left epididymis
    Juveno-adult transition, with sperms, grade 3.
  * caput, corpus, cauda.
    Oligospermia, grade 3.
  Left testicle
    Spermatogenic cycle, developed.
    Leydig cells, increased, grade 1.
  Prostata ventral fixed
    Juvenile stage with secretion.
  Prostate, dorso-lateral, fixed
    Juvenile stage with secretion.
  Seminal vesicle
    Juvenile stage with secretion.
  All other organs examined without microscopic findings.

Animal 533

General information
  Sex : Male
  Group : 3 (20 RON/ 0.25 FLT/ 30 590F mg/kg)
  Sacrifice : Subset 2 (sexual maturity)
  Necropsy status : Planned sacrifice
  Date of death : 08.May.2013
    27 days after start of exposure
    1 day after end of exposure

Macroscopic findings
  Animal without particular findings.

Microscopic findings
  Coagulating glands
    Juvenile stage with secretion.
  Left epididymis
    Juveno-adult transition, with sperms, grade 3.
  * Caput, corpus and cauda.
    Oligospermia, grade 3.
  Left testicle
    Spermatogenic cycle, developed.
  Prostata ventral fixed
    Juvenile stage with secretion.
  Prostate, dorso-lateral, fixed
    Juvenile stage with secretion.

SINGLE ANIMAL SHEET
(GROSS LESIONS AND MICROSCOPIC FINDINGS)

Sacrifice R2
Sex M
Group 3
cont. Animal 533

Seminal vesicle
Juvenile stage with secretion.
All other organs examined without microscopic findings.

Animal 534

General information
Sex : Male
Group : 3 (20 RON/ 0.25 FLT/ 30 590F mg/kg)
Sacrifice : Subset 2 (sexual maturity)
Necropsy status : Planned sacrifice
Date of death : 13.May.2013
32 days after start of exposure
1 day after end of exposure

Macroscopic findings
Animal without particular findings.

Microscopic findings
Coagulating glands
Juvenile stage with secretion.
Left epididymis
Juveno-adult transition, with sperms, grade 3.
* Caput, corpus, cauda.
Oligospermia, grade 3.
Left testicle
Spermatogenic cycle, developed.
Prostata ventral fixed
Juvenile stage with secretion.
Prostate, dorso-lateral, fixed
Juvenile stage with secretion.
Seminal vesicle
Juvenile stage with secretion.
All other organs examined without microscopic findings.

Animal 535

General information
Sex : Male
Group : 3 (20 RON/ 0.25 FLT/ 30 590F mg/kg)
Sacrifice : Subset 2 (sexual maturity)
Necropsy status : Planned sacrifice
Date of death : 12.May.2013
31 days after start of exposure
1 day after end of exposure

SINGLE ANIMAL SHEET
(GROSS LESIONS AND MICROSCOPIC FINDINGS)

Sacrifice R2
Sex M
Group 3
cont. Animal 535

Macroscopic findings
  Right epididymis
    Enlarged, right side, moderate.
  All other organs without macroscopic findings.

Microscopic findings
  Coagulating glands
    Juvenile stage with secretion.
  Left epididymis
    Juveno-adult transition, with sperms, grade 4.
  * Caput, corpus, cauda.
    Oligospermia, grade 2.
  Left testicle
    Spermatogenic cycle, developed.
  Prostata ventral fixed
    Juvenile stage with secretion.
  Prostate, dorso-lateral, fixed
    Juvenile stage with secretion.
  Right epididymis
    Gross lesion(s) evaluated histopathologically.
    Edema, diffuse, in the caudal region, grade 2, correlates to gross lesion Enlarged.
  Seminal vesicle
    Juvenile stage with secretion.
  All other organs examined without microscopic findings.

Animal 536

General information
  Sex : Male
  Group : 3 (20 RON/ 0.25 FLT/ 30 590F mg/kg)
  Sacrifice : Subset 2 (sexual maturity)
  Necropsy status : Planned sacrifice
  Date of death : 16.May.2013
    35 days after start of exposure
    1 day after end of exposure

Macroscopic findings
  Animal without particular findings.

Microscopic findings
  Coagulating glands
    Juvenile stage with secretion.
  Left epididymis
    Juveno-adult transition, with sperms, grade 4.
  * Caput, corpus, cauda.
    Oligospermia, grade 2.

SINGLE ANIMAL SHEET
(GROSS LESIONS AND MICROSCOPIC FINDINGS)

Sacrifice R2
Sex M
Group 3
cont. Animal 536

Left testicle
Spermatogenic cycle, developed.
Prostata ventral fixed
Juvenile stage with secretion.
Prostate, dorso-lateral, fixed
Juvenile stage with secretion.
Seminal vesicle
Juvenile stage with secretion.
All other organs examined without microscopic findings.

Animal 537

General information
Sex : Male
Group : 3 (20 RON/ 0.25 FLT/ 30 590F mg/kg)
Sacrifice : Subset 2 (sexual maturity)
Necropsy status : Planned sacrifice
Date of death : 16.May.2013
34 days after start of exposure
1 day after end of exposure

Macroscopic findings
Animal without particular findings.

Microscopic findings
Coagulating glands
Juvenile stage with secretion.
Left epididymis
Juveno-adult transition, with sperms, grade 4.
* Caput, corpus, cauda.
Oligospermia, grade 2.
Left testicle
Spermatogenic cycle, developed.
Prostata ventral fixed
Juvenile stage with secretion.
Prostate, dorso-lateral, fixed
Juvenile stage with secretion.
Seminal vesicle
Juvenile stage with secretion.
All other organs examined without microscopic findings.

SINGLE ANIMAL SHEET
(GROSS LESIONS AND MICROSCOPIC FINDINGS)

Sacrifice R2
Sex M
Group 3
Animal 538

General information
Sex : Male
Group : 3 (20 RON/ 0.25 FLT/ 30 590F mg/kg)
Sacrifice : Subset 2 (sexual maturity)
Necropsy status : Planned sacrifice
Date of death : 18.May.2013
36 days after start of exposure
1 day after end of exposure

Macroscopic findings
Animal without particular findings.

Microscopic findings
Coagulating glands
Juvenile stage with secretion.
Left epididymis
Juveno-adult transition, with sperms, grade 4.
* Caput, corpus, cauda.
Oligospermia, grade 2.
Left testicle
Spermatogenic cycle, developed.
Prostata ventral fixed
Juvenile stage with secretion.
Prostate, dorso-lateral, fixed
Juvenile stage with secretion.
Seminal vesicle
Juvenile stage with secretion.
All other organs examined without microscopic findings.

Animal 539

General information
Sex : Male
Group : 3 (20 RON/ 0.25 FLT/ 30 590F mg/kg)
Sacrifice : Subset 2 (sexual maturity)
Necropsy status : Planned sacrifice
Date of death : 19.May.2013
36 days after start of exposure
1 day after end of exposure

Macroscopic findings
Animal without particular findings.

SINGLE ANIMAL SHEET
(GROSS LESIONS AND MICROSCOPIC FINDINGS)

Sacrifice R2
Sex M
Group 3
cont. Animal 539

Microscopic findings
  Coagulating glands
    Juvenile stage with secretion.
  Left epididymis
    Juveno-adult transition, with sperms, grade 5.
  * Caput, corpus, cauda.
    Oligospermia, grade 1.
  Left testicle
    Spermatogenic cycle, developed.
  Prostata ventral fixed
    Juvenile stage with secretion.
  Prostate, dorso-lateral, fixed
    Juvenile stage with secretion.
  Seminal vesicle
    Juvenile stage with secretion.
  All other organs examined without microscopic findings.

Animal 540

General information
  Sex : Male
  Group : 3 (20 RON/ 0.25 FLT/ 30 590F mg/kg)
  Sacrifice : Subset 2 (sexual maturity)
  Necropsy status : Planned sacrifice
  Date of death : 19.May.2013
    36 days after start of exposure
    1 day after end of exposure

Macroscopic findings
  Animal without particular findings.

Microscopic findings
  Coagulating glands
    Juvenile stage with secretion.
  Left epididymis
    Juveno-adult transition, with sperms, grade 5.
  * Caput, corpus, cauda.
    Oligospermia, grade 1.
  Left testicle
    Spermatogenic cycle, developed.
  Prostata ventral fixed
    Juvenile stage with secretion.
  Prostate, dorso-lateral, fixed
    Juvenile stage with secretion.
  Seminal vesicle
    Juvenile stage with secretion.
  All other organs examined without microscopic findings.

SINGLE ANIMAL SHEET
(GROSS LESIONS AND MICROSCOPIC FINDINGS)

Sacrifice R2
Sex M
Group 4
Animal 541

General information
Sex : Male
Group : 4 (Flutamide 0.00025 mg/kg)
Sacrifice : Subset 2 (sexual maturity)
Necropsy status : Planned sacrifice
Date of death : 04.May.2013
26 days after start of exposure
1 day after end of exposure

Macroscopic findings
Animal without particular findings.

Microscopic findings
Coagulating glands
Juvenile stage with secretion.
Left epididymis
Juvenile stage, with sperms, grade 1.
* Caput.
Left testicle
Spermatogenic cycle, developed.
Prostata ventral fixed
Juvenile stage with secretion.
Prostate, dorso-lateral, fixed
Juvenile stage with secretion.
Seminal vesicle
Juvenile stage with secretion.
All other organs examined without microscopic findings.

Animal 542

General information
Sex : Male
Group : 4 (Flutamide 0.00025 mg/kg)
Sacrifice : Subset 2 (sexual maturity)
Necropsy status : Planned sacrifice
Date of death : 05.May.2013
26 days after start of exposure
1 day after end of exposure

Macroscopic findings
Animal without particular findings.

SINGLE ANIMAL SHEET
(GROSS LESIONS AND MICROSCOPIC FINDINGS)

Sacrifice R2
Sex M
Group 4
cont. Animal 542

Microscopic findings
  Coagulating glands
    Juvenile stage with secretion.
  Left epididymis
    Juvenile stage, with sperms, grade 1.
  * Caput.
  Left testicle
    Spermatogenic cycle, developed.
  Prostata ventral fixed
    Juvenile stage with secretion.
  Prostate, dorso-lateral, fixed
    Juvenile stage with secretion.
  Seminal vesicle
    Juvenile stage with secretion.
  All other organs examined without microscopic findings.

Animal 543

General information
  Sex : Male
  Group : 4 (Flutamide 0.00025 mg/kg)
  Sacrifice : Subset 2 (sexual maturity)
  Necropsy status : Planned sacrifice
  Date of death : 05.May.2013
    26 days after start of exposure
    1 day after end of exposure

Macroscopic findings
  Animal without particular findings.

Microscopic findings
  Coagulating glands
    Juvenile stage with secretion.
  Left epididymis
    Juvenile stage, with sperms, grade 1.
  * Caput.
  Left testicle
    Spermatogenic cycle, developed.
  Prostata ventral fixed
    Juvenile stage with secretion.
  Prostate, dorso-lateral, fixed
    Juvenile stage with secretion.
  Seminal vesicle
    Juvenile stage with secretion.
  All other organs examined without microscopic findings.

SINGLE ANIMAL SHEET
(GROSS LESIONS AND MICROSCOPIC FINDINGS)

Sacrifice R2
Sex M
Group 4
Animal 544

General information
  Sex : Male
  Group : 4 (Flutamide 0.00025 mg/kg)
  Sacrifice : Subset 2 (sexual maturity)
  Necropsy status : Planned sacrifice
  Date of death : 30.Apr.2013
  21 days after start of exposure
  1 day after end of exposure

Macroscopic findings
  Animal without particular findings.

Microscopic findings
  Coagulating glands
    Juvenile stage with secretion.
  Left epididymis
    Juvenile stage without sperms.
  Left testicle
    Spermatogenic cycle, developed.
  Prostata ventral fixed
    Juvenile stage with secretion.
  Prostate, dorso-lateral, fixed
    Juvenile stage with secretion.
  Seminal vesicle
    Juvenile stage with secretion.
  All other organs examined without microscopic findings.

Animal 545

General information
  Sex : Male
  Group : 4 (Flutamide 0.00025 mg/kg)
  Sacrifice : Subset 2 (sexual maturity)
  Necropsy status : Planned sacrifice
  Date of death : 29.Apr.2013
  19 days after start of exposure
  1 day after end of exposure

Macroscopic findings
  Animal without particular findings.
  Thyroid glands
  * Organ damaged at necropsy.

SINGLE ANIMAL SHEET
(GROSS LESIONS AND MICROSCOPIC FINDINGS)

Sacrifice R2
Sex M
Group 4
cont. Animal 545

Microscopic findings
  Coagulating glands
    Juvenile stage with secretion.
  Left epididymis
    Juvenile stage without sperms.
  Left testicle
    Spermatogenic cycle, developed.
  Prostata ventral fixed
    Juvenile stage with secretion.
  Prostate, dorso-lateral, fixed
    Juvenile stage with secretion.
  Seminal vesicle
    Juvenile stage with secretion.
  All other organs examined without microscopic findings.

Animal 546

General information
  Sex : Male
  Group : 4 (Flutamide 0.00025 mg/kg)
  Sacrifice : Subset 2 (sexual maturity)
  Necropsy status : Planned sacrifice
  Date of death : 07.May.2013
    27 days after start of exposure
    1 day after end of exposure

Macroscopic findings
  Animal without particular findings.

Microscopic findings
  Coagulating glands
    Juvenile stage with secretion.
  Left epididymis
    Juvenile stage, with sperms, grade 1.
  * Caput.
  Left testicle
    Spermatogenic cycle, developed.
  Prostata ventral fixed
    Juvenile stage with secretion.
  Prostate, dorso-lateral, fixed
    Juvenile stage with secretion.
  Seminal vesicle
    Juvenile stage with secretion.
  All other organs examined without microscopic findings.

SINGLE ANIMAL SHEET
(GROSS LESIONS AND MICROSCOPIC FINDINGS)

Sacrifice R2
Sex M
Group 4
Animal 547

General information
Sex : Male
Group : 4 (Flutamide 0.00025 mg/kg)
Sacrifice : Subset 2 (sexual maturity)
Necropsy status : Planned sacrifice
Date of death : 01.May.2013
20 days after start of exposure
1 day after end of exposure

Macroscopic findings
Animal without particular findings.

Microscopic findings
Coagulating glands
Juvenile stage with secretion.
Left epididymis
Juvenile stage without sperms.
Left testicle
Spermatogenic cycle, developed.
Prostata ventral fixed
Juvenile stage with secretion.
Prostate, dorso-lateral, fixed
Juvenile stage with secretion.
Seminal vesicle
Juvenile stage with secretion.
All other organs examined without microscopic findings.

Animal 548

General information
Sex : Male
Group : 4 (Flutamide 0.00025 mg/kg)
Sacrifice : Subset 2 (sexual maturity)
Necropsy status : Planned sacrifice
Date of death : 09.May.2013
27 days after start of exposure
1 day after end of exposure

Macroscopic findings
Animal without particular findings.

SINGLE ANIMAL SHEET
(GROSS LESIONS AND MICROSCOPIC FINDINGS)

Sacrifice R2
Sex M
Group 4
cont. Animal 548

Microscopic findings
  Coagulating glands
    Juvenile stage with secretion.
  Left epididymis
    Juvenile stage, with sperms, grade 2.
  * Caput, corpus, cauda.
  Left testicle
    Spermatogenic cycle, developed.
  Prostata ventral fixed
    Juvenile stage with secretion.
  Prostate, dorso-lateral, fixed
    Juvenile stage with secretion.
  Seminal vesicle
    Juvenile stage with secretion.
  All other organs examined without microscopic findings.

Animal 549

General information
  Sex : Male
  Group : 4 (Flutamide 0.00025 mg/kg)
  Sacrifice : Subset 2 (sexual maturity)
  Necropsy status : Planned sacrifice
  Date of death : 05.May.2013
    23 days after start of exposure
    1 day after end of exposure

Macroscopic findings
  Kidneys
    Pelvic dilation, unilateral, right side, slight.
  All other organs without macroscopic findings.

Microscopic findings
  Coagulating glands
    Juvenile stage with secretion.
  Kidneys
    Gross lesion(s) evaluated histopathologically.
    Dilation, renal pelvis, unilateral, grade 3, correlates to gross lesion
    Pelvic dilation.
  Left epididymis
    Juvenile stage without sperms.
  Left testicle
    Spermatogenic cycle, developed.
  Prostata ventral fixed
    Juvenile stage with secretion.

SINGLE ANIMAL SHEET
(GROSS LESIONS AND MICROSCOPIC FINDINGS)

Sacrifice R2
Sex M
Group 4
cont. Animal 549

Prostate, dorso-lateral, fixed
Juvenile stage with secretion.
Seminal vesicle
Juvenile stage with secretion.
All other organs examined without microscopic findings.

Animal 550

General information
Sex : Male
Group : 4 (Flutamide 0.00025 mg/kg)
Sacrifice : Subset 2 (sexual maturity)
Necropsy status : Planned sacrifice
Date of death : 10.May.2013
28 days after start of exposure
1 day after end of exposure

Macroscopic findings
Animal without particular findings.

Microscopic findings
Coagulating glands
Juvenile stage with secretion.
Left epididymis
Juvenile stage, with sperms, grade 3.
* Caput, corpus, cauda.
Left testicle
Spermatogenic cycle, developed.
Prostata ventral fixed
Juvenile stage with secretion.
Prostate, dorso-lateral, fixed
Juvenile stage with secretion.
Seminal vesicle
Juvenile stage with secretion.
All other organs examined without microscopic findings.

SINGLE ANIMAL SHEET
(GROSS LESIONS AND MICROSCOPIC FINDINGS)

Sacrifice R2
Sex F
Group 0
Animal 601

General information
Sex : Female
Group : 0 (0 mg/kg)
Sacrifice : Subset 2 (sexual maturity)
Necropsy status : Planned sacrifice
Date of death : 20.Apr.2013
11 days after start of exposure
1 day after end of exposure

Macroscopic findings
Animal without particular findings.

Microscopic findings
No histologic examination performed.

Animal 602

General information
Sex : Female
Group : 0 (0 mg/kg)
Sacrifice : Subset 2 (sexual maturity)
Necropsy status : Planned sacrifice
Date of death : 20.Apr.2013
11 days after start of exposure
1 day after end of exposure

Macroscopic findings
Uterus
Dilation, moderate.
All other organs without macroscopic findings.

Microscopic findings
Uterus
Histopathologic evaluation of gross lesion(s) will not be performed.
No histologic examination performed.

Animal 603

General information
Sex : Female
Group : 0 (0 mg/kg)
Sacrifice : Subset 2 (sexual maturity)
Necropsy status : Planned sacrifice
Date of death : 16.Apr.2013
7 days after start of exposure
1 day after end of exposure

SINGLE ANIMAL SHEET
(GROSS LESIONS AND MICROSCOPIC FINDINGS)

Sacrifice R2
Sex F
Group 0
cont. Animal 603

Macroscopic findings
  Animal without particular findings.

Microscopic findings
  No histologic examination performed.

Animal 604

General information
  Sex : Female
  Group : 0 (0 mg/kg)
  Sacrifice : Subset 2 (sexual maturity)
  Necropsy status : Planned sacrifice
  Date of death : 18.Apr.2013
  9 days after start of exposure
  1 day after end of exposure

Macroscopic findings
  Animal without particular findings.

Microscopic findings
  No histologic examination performed.

Animal 605

General information
  Sex : Female
  Group : 0 (0 mg/kg)
  Sacrifice : Subset 2 (sexual maturity)
  Necropsy status : Planned sacrifice
  Date of death : 20.Apr.2013
  10 days after start of exposure
  1 day after end of exposure

Macroscopic findings
  Animal without particular findings.

Microscopic findings
  No histologic examination performed.

Animal 606

General information
  Sex : Female
  Group : 0 (0 mg/kg)
  Sacrifice : Subset 2 (sexual maturity)
  Necropsy status : Planned sacrifice
  Date of death : 21.Apr.2013
  11 days after start of exposure

SINGLE ANIMAL SHEET
(GROSS LESIONS AND MICROSCOPIC FINDINGS)

Sacrifice R2
Sex F
Group 0
cont. Animal 606

1 day after end of exposure

Macroscopic findings
Animal without particular findings.

Microscopic findings
No histologic examination performed.

Animal 607

General information
Sex : Female
Group : 0 (0 mg/kg)
Sacrifice : Subset 2 (sexual maturity)
Necropsy status : Planned sacrifice
Date of death : 19.Apr.2013
8 days after start of exposure
1 day after end of exposure

Macroscopic findings
Animal without particular findings.

Microscopic findings
No histologic examination performed.

Animal 608

General information
Sex : Female
Group : 0 (0 mg/kg)
Sacrifice : Subset 2 (sexual maturity)
Necropsy status : Planned sacrifice
Date of death : 21.Apr.2013
10 days after start of exposure
1 day after end of exposure

Macroscopic findings
Ovaries
Cyst, left side, diameter 1.0 mm.
All other organs without macroscopic findings.

SINGLE ANIMAL SHEET
(GROSS LESIONS AND MICROSCOPIC FINDINGS)

Sacrifice R2
Sex F
Group 0
cont. Animal 608

Microscopic findings
  Ovaries
    Histopathologic evaluation of gross lesion(s) will not be performed.
  No histologic examination performed.

Animal 609

General information
  Sex : Female
  Group : 0 (0 mg/kg)
  Sacrifice : Subset 2 (sexual maturity)
  Necropsy status : Planned sacrifice
  Date of death : 21.Apr.2013
  9 days after start of exposure
  1 day after end of exposure

Macroscopic findings
  Animal without particular findings.

Microscopic findings
  No histologic examination performed.

Animal 610

General information
  Sex : Female
  Group : 0 (0 mg/kg)
  Sacrifice : Subset 2 (sexual maturity)
  Necropsy status : Planned sacrifice
  Date of death : 21.Apr.2013
  9 days after start of exposure
  1 day after end of exposure

Macroscopic findings
  Animal without particular findings.

Microscopic findings
  No histologic examination performed.

SINGLE ANIMAL SHEET
(GROSS LESIONS AND MICROSCOPIC FINDINGS)

Sacrifice R2
Sex F
Group 1
Animal 611

General information
Sex : Female
Group : 1 (0.005RON/0.00025FLT/0.01 590F mg/kg)
Sacrifice : Subset 2 (sexual maturity)
Necropsy status : Planned sacrifice
Date of death : 18.Apr.2013
10 days after start of exposure
1 day after end of exposure

Macroscopic findings
Animal without particular findings.

Microscopic findings
No histologic examination performed.

Animal 612

General information
Sex : Female
Group : 1 (0.005RON/0.00025FLT/0.01 590F mg/kg)
Sacrifice : Subset 2 (sexual maturity)
Necropsy status : Planned sacrifice
Date of death : 19.Apr.2013
10 days after start of exposure
1 day after end of exposure

Macroscopic findings
Animal without particular findings.

Microscopic findings
No histologic examination performed.

Animal 613

General information
Sex : Female
Group : 1 (0.005RON/0.00025FLT/0.01 590F mg/kg)
Sacrifice : Subset 2 (sexual maturity)
Necropsy status : Planned sacrifice
Date of death : 21.Apr.2013
12 days after start of exposure
1 day after end of exposure

SINGLE ANIMAL SHEET
(GROSS LESIONS AND MICROSCOPIC FINDINGS)

Sacrifice R2
Sex F
Group 1
cont. Animal 613

Macroscopic findings
Animal without particular findings.

Microscopic findings
No histologic examination performed.

Animal 614

General information
Sex : Female
Group : 1 (0.005RON/0.00025FLT/0.01 590F mg/kg)
Sacrifice : Subset 2 (sexual maturity)
Necropsy status : Planned sacrifice
Date of death : 18.Apr.2013
9 days after start of exposure
1 day after end of exposure

Macroscopic findings
Animal without particular findings.

Microscopic findings
No histologic examination performed.

Animal 615

General information
Sex : Female
Group : 1 (0.005RON/0.00025FLT/0.01 590F mg/kg)
Sacrifice : Subset 2 (sexual maturity)
Necropsy status : Planned sacrifice
Date of death : 19.Apr.2013
10 days after start of exposure
1 day after end of exposure

Macroscopic findings
Animal without particular findings.

Microscopic findings
No histologic examination performed.

Animal 616

General information
Sex : Female
Group : 1 (0.005RON/0.00025FLT/0.01 590F mg/kg)
Sacrifice : Subset 2 (sexual maturity)
Necropsy status : Planned sacrifice
Date of death : 20.Apr.2013
10 days after start of exposure

SINGLE ANIMAL SHEET
(GROSS LESIONS AND MICROSCOPIC FINDINGS)

Sacrifice R2
Sex F
Group 1
cont. Animal 616

1 day after end of exposure

Macroscopic findings
Animal without particular findings.

Microscopic findings
No histologic examination performed.

Animal 617

General information
Sex : Female
Group : 1 (0.005RON/0.00025FLT/0.01 590F mg/kg)
Sacrifice : Subset 2 (sexual maturity)
Necropsy status : Planned sacrifice
Date of death : 19.Apr.2013
9 days after start of exposure
1 day after end of exposure

Macroscopic findings
Animal without particular findings.

Microscopic findings
No histologic examination performed.

Animal 618

General information
Sex : Female
Group : 1 (0.005RON/0.00025FLT/0.01 590F mg/kg)
Sacrifice : Subset 2 (sexual maturity)
Necropsy status : Planned sacrifice
Date of death : 18.Apr.2013
7 days after start of exposure
1 day after end of exposure

Macroscopic findings
Animal without particular findings.

Microscopic findings
No histologic examination performed.

SINGLE ANIMAL SHEET
(GROSS LESIONS AND MICROSCOPIC FINDINGS)

Sacrifice	R2
Sex	F
Group	1
Animal	619

General information
- Sex : Female
- Group : 1 (0.005RON/0.00025FLT/0.01 590F mg/kg)
- Sacrifice : Subset 2 (sexual maturity)
- Necropsy status : Planned sacrifice
- Date of death : 20.Apr.2013
  9 days after start of exposure
  1 day after end of exposure

Macroscopic findings
Animal without particular findings.

Microscopic findings
No histologic examination performed.

Animal 620

General information
- Sex : Female
- Group : 1 (0.005RON/0.00025FLT/0.01 590F mg/kg)
- Sacrifice : Subset 2 (sexual maturity)
- Necropsy status : Planned sacrifice
- Date of death : 20.Apr.2013
  8 days after start of exposure
  1 day after end of exposure

Macroscopic findings
Animal without particular findings.

Microscopic findings
No histologic examination performed.

SINGLE ANIMAL SHEET
(GROSS LESIONS AND MICROSCOPIC FINDINGS)

Sacrifice R2
Sex F
Group 2
Animal 621

General information
Sex : Female
Group : 2 (4 RON/ 0.025 FLT/ 5 590F mg/kg)
Sacrifice : Subset 2 (sexual maturity)
Necropsy status : Planned sacrifice
Date of death : 18.Apr.2013
9 days after start of exposure
1 day after end of exposure

Macroscopic findings
Animal without particular findings.

Microscopic findings
No histologic examination performed.

Animal 622

General information
Sex : Female
Group : 2 (4 RON/ 0.025 FLT/ 5 590F mg/kg)
Sacrifice : Subset 2 (sexual maturity)
Necropsy status : Planned sacrifice
Date of death : 18.Apr.2013
9 days after start of exposure
1 day after end of exposure

Macroscopic findings
Animal without particular findings.

Microscopic findings
No histologic examination performed.

Animal 623

General information
Sex : Female
Group : 2 (4 RON/ 0.025 FLT/ 5 590F mg/kg)
Sacrifice : Subset 2 (sexual maturity)
Necropsy status : Planned sacrifice
Date of death : 16.Apr.2013
7 days after start of exposure
1 day after end of exposure

SINGLE ANIMAL SHEET
(GROSS LESIONS AND MICROSCOPIC FINDINGS)

Sacrifice	R2
Sex	F
Group	2
cont. Animal	623

Macroscopic findings
Animal without particular findings.

Microscopic findings
No histologic examination performed.

Animal 624

General information
Sex : Female
Group : 2 (4 RON/ 0.025 FLT/ 5 590F mg/kg)
Sacrifice : Subset 2 (sexual maturity)
Necropsy status : Planned sacrifice
Date of death : 20.Apr.2013
10 days after start of exposure
1 day after end of exposure

Macroscopic findings
Uterus
Dilation, moderate.
All other organs without macroscopic findings.

Microscopic findings
Uterus
Histopathologic evaluation of gross lesion(s) will not be performed.
No histologic examination performed.

Animal 625

General information
Sex : Female
Group : 2 (4 RON/ 0.025 FLT/ 5 590F mg/kg)
Sacrifice : Subset 2 (sexual maturity)
Necropsy status : Planned sacrifice
Date of death : 19.Apr.2013
8 days after start of exposure
1 day after end of exposure

Macroscopic findings
Animal without particular findings.

SINGLE ANIMAL SHEET
(GROSS LESIONS AND MICROSCOPIC FINDINGS)

Sacrifice R2
Sex F
Group 2
cont. Animal 625

Microscopic findings
No histologic examination performed.

Animal 626

General information
Sex : Female
Group : 2 (4 RON/ 0.025 FLT/ 5 590F mg/kg)
Sacrifice : Subset 2 (sexual maturity)
Necropsy status : Planned sacrifice
Date of death : 21.Apr.2013
10 days after start of exposure
1 day after end of exposure

Macroscopic findings
Animal without particular findings.

Microscopic findings
No histologic examination performed.

Animal 627

General information
Sex : Female
Group : 2 (4 RON/ 0.025 FLT/ 5 590F mg/kg)
Sacrifice : Subset 2 (sexual maturity)
Necropsy status : Planned sacrifice
Date of death : 21.Apr.2013
10 days after start of exposure
1 day after end of exposure

Macroscopic findings
Animal without particular findings.

Microscopic findings
No histologic examination performed.

Animal 628

General information
Sex : Female
Group : 2 (4 RON/ 0.025 FLT/ 5 590F mg/kg)
Sacrifice : Subset 2 (sexual maturity)
Necropsy status : Planned sacrifice
Date of death : 22.Apr.2013
11 days after start of exposure
1 day after end of exposure

SINGLE ANIMAL SHEET
(GROSS LESIONS AND MICROSCOPIC FINDINGS)

Sacrifice R2
Sex F
Group 2
cont. Animal 628

Macroscopic findings
  Animal without particular findings.

Microscopic findings
  No histologic examination performed.

Animal 629

General information
  Sex : Female
  Group : 2 (4 RON/ 0.025 FLT/ 5 590F mg/kg)
  Sacrifice : Subset 2 (sexual maturity)
  Necropsy status : Planned sacrifice
  Date of death : 21.Apr.2013
  8 days after start of exposure
  1 day after end of exposure

Macroscopic findings
  Animal without particular findings.

Microscopic findings
  No histologic examination performed.

Animal 630

General information
  Sex : Female
  Group : 2 (4 RON/ 0.025 FLT/ 5 590F mg/kg)
  Sacrifice : Subset 2 (sexual maturity)
  Necropsy status : Planned sacrifice
  Date of death : 22.Apr.2013
  9 days after start of exposure
  1 day after end of exposure

Macroscopic findings
  Pericard
    Dilation.
  All other organs without macroscopic findings.

Microscopic findings
  Pericard
    Histopathologic evaluation of gross lesion(s) will not be performed.
  No histologic examination performed.

SINGLE ANIMAL SHEET
(GROSS LESIONS AND MICROSCOPIC FINDINGS)

Sacrifice R2
Sex F
Group 3
Animal 631

General information
Sex : Female
Group : 3 (20 RON/ 0.25 FLT/ 30 590F mg/kg)
Sacrifice : Subset 2 (sexual maturity)
Necropsy status : Planned sacrifice
Date of death : 18.Apr.2013
8 days after start of exposure
1 day after end of exposure

Macroscopic findings
Animal without particular findings.

Microscopic findings
No histologic examination performed.

Animal 632

General information
Sex : Female
Group : 3 (20 RON/ 0.25 FLT/ 30 590F mg/kg)
Sacrifice : Subset 2 (sexual maturity)
Necropsy status : Planned sacrifice
Date of death : 22.Apr.2013
12 days after start of exposure
1 day after end of exposure

Macroscopic findings
Animal without particular findings.

Microscopic findings
No histologic examination performed.

Animal 633

General information
Sex : Female
Group : 3 (20 RON/ 0.25 FLT/ 30 590F mg/kg)
Sacrifice : Subset 2 (sexual maturity)
Necropsy status : Planned sacrifice
Date of death : 19.Apr.2013
8 days after start of exposure
1 day after end of exposure

SINGLE ANIMAL SHEET
(GROSS LESIONS AND MICROSCOPIC FINDINGS)

Sacrifice R2
Sex F
Group 3
cont. Animal 633

Macroscopic findings
  Animal without particular findings.

Microscopic findings
  No histologic examination performed.

Animal 634

General information
  Sex : Female
  Group : 3 (20 RON/ 0.25 FLT/ 30 590F mg/kg)
  Sacrifice : Subset 2 (sexual maturity)
  Necropsy status : Planned sacrifice
  Date of death : 21.Apr.2013
  10 days after start of exposure
  1 day after end of exposure

Macroscopic findings
  Animal without particular findings.

Microscopic findings
  No histologic examination performed.

Animal 635

General information
  Sex : Female
  Group : 3 (20 RON/ 0.25 FLT/ 30 590F mg/kg)
  Sacrifice : Subset 2 (sexual maturity)
  Necropsy status : Planned sacrifice
  Date of death : 19.Apr.2013
  8 days after start of exposure
  1 day after end of exposure

Macroscopic findings
  Oviducts
    Cyst, right side, diameter 2.0 mm.
  All other organs without macroscopic findings.

Microscopic findings
  Oviducts
    Histopathologic evaluation of gross lesion(s) will not be performed.
  No histologic examination performed.

SINGLE ANIMAL SHEET
(GROSS LESIONS AND MICROSCOPIC FINDINGS)

Sacrifice R2
Sex F
Group 3
Animal 636

General information
Sex : Female
Group : 3 (20 RON/ 0.25 FLT/ 30 590F mg/kg)
Sacrifice : Subset 2 (sexual maturity)
Necropsy status : Planned sacrifice
Date of death : 21.Apr.2013
9 days after start of exposure
1 day after end of exposure

Macroscopic findings
Animal without particular findings.

Microscopic findings
No histologic examination performed.

Animal 637

General information
Sex : Female
Group : 3 (20 RON/ 0.25 FLT/ 30 590F mg/kg)
Sacrifice : Subset 2 (sexual maturity)
Necropsy status : Planned sacrifice
Date of death : 21.Apr.2013
9 days after start of exposure
1 day after end of exposure

Macroscopic findings
Animal without particular findings.

Microscopic findings
No histologic examination performed.

Animal 638

General information
Sex : Female
Group : 3 (20 RON/ 0.25 FLT/ 30 590F mg/kg)
Sacrifice : Subset 2 (sexual maturity)
Necropsy status : Planned sacrifice
Date of death : 22.Apr.2013
10 days after start of exposure
1 day after end of exposure

SINGLE ANIMAL SHEET
(GROSS LESIONS AND MICROSCOPIC FINDINGS)

Sacrifice R2
Sex F
Group 3
cont. Animal 638

Macroscopic findings
  Animal without particular findings.

Microscopic findings
  No histologic examination performed.

Animal 639

General information
  Sex : Female
  Group : 3 (20 RON/ 0.25 FLT/ 30 590F mg/kg)
  Sacrifice : Subset 2 (sexual maturity)
  Necropsy status : Planned sacrifice
  Date of death : 23.Apr.2013
  10 days after start of exposure
  1 day after end of exposure

Macroscopic findings
  Animal without particular findings.

Microscopic findings
  No histologic examination performed.

Animal 640

General information
  Sex : Female
  Group : 3 (20 RON/ 0.25 FLT/ 30 590F mg/kg)
  Sacrifice : Subset 2 (sexual maturity)
  Necropsy status : Planned sacrifice
  Date of death : 23.Apr.2013
  10 days after start of exposure
  1 day after end of exposure

Macroscopic findings
  Animal without particular findings.

Microscopic findings
  No histologic examination performed.

SINGLE ANIMAL SHEET
(GROSS LESIONS AND MICROSCOPIC FINDINGS)

Sacrifice R2
Sex F
Group 4
Animal 641

General information
Sex : Female
Group : 4 (Flutamide 0.00025 mg/kg)
Sacrifice : Subset 2 (sexual maturity)
Necropsy status : Planned sacrifice
Date of death : 17.Apr.2013
9 days after start of exposure
1 day after end of exposure

Macroscopic findings
Animal without particular findings.

Microscopic findings
No histologic examination performed.

Animal 642

General information
Sex : Female
Group : 4 (Flutamide 0.00025 mg/kg)
Sacrifice : Subset 2 (sexual maturity)
Necropsy status : Planned sacrifice
Date of death : 19.Apr.2013
10 days after start of exposure
1 day after end of exposure

Macroscopic findings
Animal without particular findings.

Microscopic findings
No histologic examination performed.

Animal 643

General information
Sex : Female
Group : 4 (Flutamide 0.00025 mg/kg)
Sacrifice : Subset 2 (sexual maturity)
Necropsy status : Planned sacrifice
Date of death : 17.Apr.2013
8 days after start of exposure
1 day after end of exposure

SINGLE ANIMAL SHEET
(GROSS LESIONS AND MICROSCOPIC FINDINGS)

Sacrifice R2
Sex F
Group 4
cont. Animal 643

Macroscopic findings
Animal without particular findings.

Microscopic findings
No histologic examination performed.

Animal 644

General information
Sex : Female
Group : 4 (Flutamide 0.00025 mg/kg)
Sacrifice : Subset 2 (sexual maturity)
Necropsy status : Planned sacrifice
Date of death : 22.Apr.2013
12 days after start of exposure
1 day after end of exposure

Macroscopic findings
Animal without particular findings.

Microscopic findings
No histologic examination performed.

Animal 645

General information
Sex : Female
Group : 4 (Flutamide 0.00025 mg/kg)
Sacrifice : Subset 2 (sexual maturity)
Necropsy status : Planned sacrifice
Date of death : 19.Apr.2013
9 days after start of exposure
1 day after end of exposure

Macroscopic findings
Animal without particular findings.

Microscopic findings
No histologic examination performed.

Animal 646

General information
Sex : Female
Group : 4 (Flutamide 0.00025 mg/kg)
Sacrifice : Subset 2 (sexual maturity)
Necropsy status : Planned sacrifice
Date of death : 20.Apr.2013
10 days after start of exposure

SINGLE ANIMAL SHEET
(GROSS LESIONS AND MICROSCOPIC FINDINGS)

Sacrifice R2
Sex F
Group 4
cont. Animal 646

1 day after end of exposure

Macroscopic findings
Uterus
Dilation, moderate.
All other organs without macroscopic findings.

Microscopic findings
Uterus
Histopathologic evaluation of gross lesion(s) will not be performed.
No histologic examination performed.

Animal 647

General information
Sex : Female
Group : 4 (Flutamide 0.00025 mg/kg)
Sacrifice : Subset 2 (sexual maturity)
Necropsy status : Planned sacrifice
Date of death : 18.Apr.2013
7 days after start of exposure
1 day after end of exposure

Macroscopic findings
Animal without particular findings.

Microscopic findings
No histologic examination performed.

Animal 648

General information
Sex : Female
Group : 4 (Flutamide 0.00025 mg/kg)
Sacrifice : Subset 2 (sexual maturity)
Necropsy status : Planned sacrifice
Date of death : 22.Apr.2013
10 days after start of exposure
1 day after end of exposure

Macroscopic findings
Animal without particular findings.

SINGLE ANIMAL SHEET
(GROSS LESIONS AND MICROSCOPIC FINDINGS)

Sacrifice R2
Sex F
Group 4
cont. Animal 648

Microscopic findings
No histologic examination performed.

Animal 649

General information
Sex : Female
Group : 4 (Flutamide 0.00025 mg/kg)
Sacrifice : Subset 2 (sexual maturity)
Necropsy status : Planned sacrifice
Date of death : 22.Apr.2013
10 days after start of exposure
1 day after end of exposure

Macroscopic findings
Animal without particular findings.

Microscopic findings
No histologic examination performed.

Animal 650

General information
Sex : Female
Group : 4 (Flutamide 0.00025 mg/kg)
Sacrifice : Subset 2 (sexual maturity)
Necropsy status : Planned sacrifice
Date of death : 23.Apr.2013
11 days after start of exposure
1 day after end of exposure

Macroscopic findings
Animal without particular findings.

Microscopic findings
No histologic examination performed.

SINGLE ANIMAL SHEET
(GROSS LESIONS AND MICROSCOPIC FINDINGS)

Sacrifice R3
Sex M
Group 0
Animal 701

General information
Sex : Male
Group : 0 (0 mg/kg)
Sacrifice : Subset 3 (PND 83+-2)
Necropsy status : Planned sacrifice
Date of death : 10.Jun.2013
62 days after start of exposure
1 day after end of exposure

Macroscopic findings
Animal without particular findings.

Microscopic findings
All organs examined without pathologic findings.

Animal 702

General information
Sex : Male
Group : 0 (0 mg/kg)
Sacrifice : Subset 3 (PND 83+-2)
Necropsy status : Planned sacrifice
Date of death : 10.Jun.2013
62 days after start of exposure
1 day after end of exposure

Macroscopic findings
Animal without particular findings.

Microscopic findings
All organs examined without pathologic findings.

Animal 703

General information
Sex : Male
Group : 0 (0 mg/kg)
Sacrifice : Subset 3 (PND 83+-2)
Necropsy status : Sacrificed moribund
Date of death : 22.Apr.2013
13 days after start of exposure
1 day after end of exposure

SINGLE ANIMAL SHEET
(GROSS LESIONS AND MICROSCOPIC FINDINGS)

Sacrifice	R3
Sex	M
Group	0
cont. Animal	703

Macroscopic findings
  Skeletal muscle
    Effusion, in the head region, content foamy, yellow glassy transparent.
  Thoracic cavity
    Effusion, content 1.0 ml, yellow glassy transparent.
  All other organs without macroscopic findings.

Microscopic findings
  Coagulating glands
    Juvenile stage with secretion.
  Left epididymis
    Immature stage.
  Left testicle
    Immature stage.
  Prostata ventral fixed
    Juvenile stage with secretion.
  Prostate, dorso-lateral, fixed
    Juvenile stage with secretion.
  Seminal vesicle
    Juvenile stage with secretion.
  Skeletal muscle
    Histopathologic evaluation of gross lesion(s) will not be performed.
  Thoracic cavity
    Histopathologic evaluation of gross lesion(s) will not be performed.
  All other organs examined without microscopic findings.

Animal 704

General information
  Sex : Male
  Group : 0 (0 mg/kg)
  Sacrifice : Subset 3 (PND 83+-2)
  Necropsy status : Planned sacrifice
  Date of death : 10.Jun.2013
    61 days after start of exposure
    1 day after end of exposure

Macroscopic findings
  Animal without particular findings.

Microscopic findings
  Left testicle
    Degeneration, tubular, (multi)focal, grade 1.
  All other organs examined without microscopic findings.

SINGLE ANIMAL SHEET
(GROSS LESIONS AND MICROSCOPIC FINDINGS)

Sacrifice R3
Sex M
Group 0
Animal 705

General information
Sex : Male
Group : 0 (0 mg/kg)
Sacrifice : Subset 3 (PND 83+-2)
Necropsy status : Planned sacrifice
Date of death : 10.Jun.2013
61 days after start of exposure
1 day after end of exposure

Macroscopic findings
Animal without particular findings.

Microscopic findings
All organs examined without pathologic findings.

Animal 706

General information
Sex : Male
Group : 0 (0 mg/kg)
Sacrifice : Subset 3 (PND 83+-2)
Necropsy status : Planned sacrifice
Date of death : 10.Jun.2013
60 days after start of exposure
1 day after end of exposure

Macroscopic findings
Animal without particular findings.

Microscopic findings
All organs examined without pathologic findings.

Animal 707

General information
Sex : Male
Group : 0 (0 mg/kg)
Sacrifice : Subset 3 (PND 83+-2)
Necropsy status : Planned sacrifice
Date of death : 13.Jun.2013
63 days after start of exposure
1 day after end of exposure

SINGLE ANIMAL SHEET
(GROSS LESIONS AND MICROSCOPIC FINDINGS)

Sacrifice	R3
Sex	M
Group	0
cont. Animal	707

Macroscopic findings
Animal without particular findings.

Microscopic findings
All organs examined without pathologic findings.

Animal 708

General information
Sex : Male
Group : 0 (0 mg/kg)
Sacrifice : Subset 3 (PND 83+-2)
Necropsy status : Planned sacrifice
Date of death : 13.Jun.2013
64 days after start of exposure
1 day after end of exposure

Macroscopic findings
Animal without particular findings.

Microscopic findings
Prostata ventral fixed
Infiltration, lymphoid cell, (multi)focal, grade 1.
All other organs examined without microscopic findings.

Animal 709

General information
Sex : Male
Group : 0 (0 mg/kg)
Sacrifice : Subset 3 (PND 83+-2)
Necropsy status : Planned sacrifice
Date of death : 13.Jun.2013
62 days after start of exposure
1 day after end of exposure

Macroscopic findings
Animal without particular findings.

Microscopic findings
All organs examined without pathologic findings.

SINGLE ANIMAL SHEET
(GROSS LESIONS AND MICROSCOPIC FINDINGS)

Sacrifice	R3
Sex	M
Group	0
Animal	710

General information

```
  Sex              : Male
  Group            : 0 (0 mg/kg)
  Sacrifice        : Subset 3 (PND 83+-2)
  Necropsy status  : Planned sacrifice
  Date of death    : 13.Jun.2013
                     62 days after start of exposure
                     1 day after end of exposure
```

Macroscopic findings
Animal without particular findings.

Microscopic findings
All organs examined without pathologic findings.

SINGLE ANIMAL SHEET
(GROSS LESIONS AND MICROSCOPIC FINDINGS)

Sacrifice R3
Sex M
Group 1
Animal 711

General information
Sex : Male
Group : 1 (0.005RON/0.00025FLT/0.01 590F mg/kg)
Sacrifice : Subset 3 (PND 83+-2)
Necropsy status : Planned sacrifice
Date of death : 10.Jun.2013
63 days after start of exposure
1 day after end of exposure

Macroscopic findings
Animal without particular findings.

Microscopic findings
All organs examined without pathologic findings.

Animal 712

General information
Sex : Male
Group : 1 (0.005RON/0.00025FLT/0.01 590F mg/kg)
Sacrifice : Subset 3 (PND 83+-2)
Necropsy status : Planned sacrifice
Date of death : 10.Jun.2013
62 days after start of exposure
1 day after end of exposure

Macroscopic findings
Animal without particular findings.

Microscopic findings
All organs examined without pathologic findings.

Animal 713

General information
Sex : Male
Group : 1 (0.005RON/0.00025FLT/0.01 590F mg/kg)
Sacrifice : Subset 3 (PND 83+-2)
Necropsy status : Planned sacrifice
Date of death : 10.Jun.2013
62 days after start of exposure
1 day after end of exposure

SINGLE ANIMAL SHEET
(GROSS LESIONS AND MICROSCOPIC FINDINGS)

Sacrifice R3
Sex M
Group 1
cont. Animal 713

Macroscopic findings
Animal without particular findings.

Microscopic findings
All organs examined without pathologic findings.

Animal 714

General information
Sex : Male
Group : 1 (0.005RON/0.00025FLT/0.01 590F mg/kg)
Sacrifice : Subset 3 (PND 83+-2)
Necropsy status : Planned sacrifice
Date of death : 10.Jun.2013
62 days after start of exposure
1 day after end of exposure

Macroscopic findings
Animal without particular findings.

Microscopic findings
All organs examined without pathologic findings.

Animal 715

General information
Sex : Male
Group : 1 (0.005RON/0.00025FLT/0.01 590F mg/kg)
Sacrifice : Subset 3 (PND 83+-2)
Necropsy status : Planned sacrifice
Date of death : 10.Jun.2013
61 days after start of exposure
1 day after end of exposure

Macroscopic findings
Animal without particular findings.

Microscopic findings
All organs examined without pathologic findings.

Animal 716

General information
Sex : Male
Group : 1 (0.005RON/0.00025FLT/0.01 590F mg/kg)
Sacrifice : Subset 3 (PND 83+-2)
Necropsy status : Planned sacrifice
Date of death : 10.Jun.2013
61 days after start of exposure

SINGLE ANIMAL SHEET
(GROSS LESIONS AND MICROSCOPIC FINDINGS)

Sacrifice R3
Sex M
Group 1
cont. Animal 716

....

1 day after end of exposure

Macroscopic findings
Animal without particular findings.

Microscopic findings
Left testicle
Degeneration, tubular, (multi)focal, grade 1.
All other organs examined without microscopic findings.

Animal 717

....

General information
Sex : Male
Group : 1 (0.005RON/0.00025FLT/0.01 590F mg/kg)
Sacrifice : Subset 3 (PND 83+-2)
Necropsy status : Planned sacrifice
Date of death : 10.Jun.2013
61 days after start of exposure
1 day after end of exposure

Macroscopic findings
Animal without particular findings.

Microscopic findings
All organs examined without pathologic findings.

Animal 718

....

General information
Sex : Male
Group : 1 (0.005RON/0.00025FLT/0.01 590F mg/kg)
Sacrifice : Subset 3 (PND 83+-2)
Necropsy status : Planned sacrifice
Date of death : 13.Jun.2013
63 days after start of exposure
1 day after end of exposure

Macroscopic findings
Animal without particular findings.

SINGLE ANIMAL SHEET
(GROSS LESIONS AND MICROSCOPIC FINDINGS)

Sacrifice	R3
Sex	M
Group	1
cont. Animal	718

Microscopic findings
All organs examined without pathologic findings.

Animal 719

General information
Sex : Male
Group : 1 (0.005RON/0.00025FLT/0.01 590F mg/kg)
Sacrifice : Subset 3 (PND 83+-2)
Necropsy status : Planned sacrifice
Date of death : 13.Jun.2013
62 days after start of exposure
1 day after end of exposure

Macroscopic findings
Animal without particular findings.

Microscopic findings
All organs examined without pathologic findings.

Animal 720

General information
Sex : Male
Group : 1 (0.005RON/0.00025FLT/0.01 590F mg/kg)
Sacrifice : Subset 3 (PND 83+-2)
Necropsy status : Planned sacrifice
Date of death : 13.Jun.2013
62 days after start of exposure
1 day after end of exposure

Macroscopic findings
Animal without particular findings.

Microscopic findings
All organs examined without pathologic findings.

SINGLE ANIMAL SHEET
(GROSS LESIONS AND MICROSCOPIC FINDINGS)

Sacrifice R3
Sex M
Group 2
Animal 721

General information
Sex : Male
Group : 2 (4 RON/ 0.025 FLT/ 5 590F mg/kg)
Sacrifice : Subset 3 (PND 83+-2)
Necropsy status : Planned sacrifice
Date of death : 10.Jun.2013
62 days after start of exposure
1 day after end of exposure

Macroscopic findings
Animal without particular findings.

Microscopic findings
All organs examined without pathologic findings.

Animal 722

General information
Sex : Male
Group : 2 (4 RON/ 0.025 FLT/ 5 590F mg/kg)
Sacrifice : Subset 3 (PND 83+-2)
Necropsy status : Planned sacrifice
Date of death : 10.Jun.2013
62 days after start of exposure
1 day after end of exposure

Macroscopic findings
Animal without particular findings.

Microscopic findings
All organs examined without pathologic findings.

Animal 723

General information
Sex : Male
Group : 2 (4 RON/ 0.025 FLT/ 5 590F mg/kg)
Sacrifice : Subset 3 (PND 83+-2)
Necropsy status : Planned sacrifice
Date of death : 10.Jun.2013
62 days after start of exposure
1 day after end of exposure

SINGLE ANIMAL SHEET
(GROSS LESIONS AND MICROSCOPIC FINDINGS)

Sacrifice R3
Sex M
Group 2
cont. Animal 723

Macroscopic findings
Animal without particular findings.

Microscopic findings
All organs examined without pathologic findings.

Animal 724

General information
Sex : Male
Group : 2 (4 RON/ 0.025 FLT/ 5 590F mg/kg)
Sacrifice : Subset 3 (PND 83+-2)
Necropsy status : Planned sacrifice
Date of death : 10.Jun.2013
61 days after start of exposure
1 day after end of exposure

Macroscopic findings
Animal without particular findings.

Microscopic findings
All organs examined without pathologic findings.

Animal 725

General information
Sex : Male
Group : 2 (4 RON/ 0.025 FLT/ 5 590F mg/kg)
Sacrifice : Subset 3 (PND 83+-2)
Necropsy status : Planned sacrifice
Date of death : 13.Jun.2013
63 days after start of exposure
1 day after end of exposure

Macroscopic findings
Animal without particular findings.

Microscopic findings
Left testicle
Degeneration, tubular, (multi)focal, grade 1.
All other organs examined without microscopic findings.

SINGLE ANIMAL SHEET
(GROSS LESIONS AND MICROSCOPIC FINDINGS)

Sacrifice R3
Sex M
Group 2
Animal 726

General information
Sex : Male
Group : 2 (4 RON/ 0.025 FLT/ 5 590F mg/kg)
Sacrifice : Subset 3 (PND 83+-2)
Necropsy status : Planned sacrifice
Date of death : 13.Jun.2013
63 days after start of exposure
1 day after end of exposure

Macroscopic findings
Animal without particular findings.

Microscopic findings
All organs examined without pathologic findings.

Animal 727

General information
Sex : Male
Group : 2 (4 RON/ 0.025 FLT/ 5 590F mg/kg)
Sacrifice : Subset 3 (PND 83+-2)
Necropsy status : Planned sacrifice
Date of death : 13.Jun.2013
63 days after start of exposure
1 day after end of exposure

Macroscopic findings
Animal without particular findings.

Microscopic findings
Left testicle
Degeneration, tubular, (multi)focal, grade 1.
All other organs examined without microscopic findings.

Animal 728

General information
Sex : Male
Group : 2 (4 RON/ 0.025 FLT/ 5 590F mg/kg)
Sacrifice : Subset 3 (PND 83+-2)
Necropsy status : Planned sacrifice
Date of death : 13.Jun.2013
63 days after start of exposure
1 day after end of exposure

SINGLE ANIMAL SHEET
(GROSS LESIONS AND MICROSCOPIC FINDINGS)

Sacrifice	R3
Sex	M
Group	2
cont. Animal	728

Macroscopic findings
Animal without particular findings.

Microscopic findings
All organs examined without pathologic findings.

Animal 729

General information
Sex : Male
Group : 2 (4 RON/ 0.025 FLT/ 5 590F mg/kg)
Sacrifice : Subset 3 (PND 83+-2)
Necropsy status : Planned sacrifice
Date of death : 13.Jun.2013
61 days after start of exposure
1 day after end of exposure

Macroscopic findings
Animal without particular findings.

Microscopic findings
All organs examined without pathologic findings.

Animal 730

General information
Sex : Male
Group : 2 (4 RON/ 0.025 FLT/ 5 590F mg/kg)
Sacrifice : Subset 3 (PND 83+-2)
Necropsy status : Planned sacrifice
Date of death : 13.Jun.2013
61 days after start of exposure
1 day after end of exposure

Macroscopic findings
Animal without particular findings.

Microscopic findings
All organs examined without pathologic findings.

SINGLE ANIMAL SHEET
(GROSS LESIONS AND MICROSCOPIC FINDINGS)

Sacrifice R3
Sex M
Group 3
Animal 731

General information
Sex : Male
Group : 3 (20 RON/ 0.25 FLT/ 30 590F mg/kg)
Sacrifice : Subset 3 (PND 83+-2)
Necropsy status : Planned sacrifice
Date of death : 10.Jun.2013
61 days after start of exposure
1 day after end of exposure

Macroscopic findings
Animal without particular findings.

Microscopic findings
All organs examined without pathologic findings.

Animal 732

General information
Sex : Male
Group : 3 (20 RON/ 0.25 FLT/ 30 590F mg/kg)
Sacrifice : Subset 3 (PND 83+-2)
Necropsy status : Planned sacrifice
Date of death : 10.Jun.2013
61 days after start of exposure
1 day after end of exposure

Macroscopic findings
Animal without particular findings.

Microscopic findings
Left testicle
Degeneration, tubular, (multi)focal, grade 1.
All other organs examined without microscopic findings.

Animal 733

General information
Sex : Male
Group : 3 (20 RON/ 0.25 FLT/ 30 590F mg/kg)
Sacrifice : Subset 3 (PND 83+-2)
Necropsy status : Planned sacrifice
Date of death : 13.Jun.2013
63 days after start of exposure
1 day after end of exposure

SINGLE ANIMAL SHEET
(GROSS LESIONS AND MICROSCOPIC FINDINGS)

Sacrifice R3
Sex M
Group 3
cont. Animal 733

Macroscopic findings
Animal without particular findings.

Microscopic findings
All organs examined without pathologic findings.

Animal 734

General information
Sex : Male
Group : 3 (20 RON/ 0.25 FLT/ 30 590F mg/kg)
Sacrifice : Subset 3 (PND 83+-2)
Necropsy status : Planned sacrifice
Date of death : 13.Jun.2013
63 days after start of exposure
1 day after end of exposure

Macroscopic findings
Animal without particular findings.

Microscopic findings
All organs examined without pathologic findings.

Animal 735

General information
Sex : Male
Group : 3 (20 RON/ 0.25 FLT/ 30 590F mg/kg)
Sacrifice : Subset 3 (PND 83+-2)
Necropsy status : Planned sacrifice
Date of death : 13.Jun.2013
63 days after start of exposure
1 day after end of exposure

Macroscopic findings
Animal without particular findings.

Microscopic findings
Prostata ventral fixed
Infiltration, lymphoid cell, (multi)focal, grade 1.
All other organs examined without microscopic findings.

SINGLE ANIMAL SHEET
(GROSS LESIONS AND MICROSCOPIC FINDINGS)

Sacrifice	R3
Sex	M
Group	3
Animal	736

General information
- Sex : Male
- Group : 3 (20 RON/ 0.25 FLT/ 30 590F mg/kg)
- Sacrifice : Subset 3 (PND 83+-2)
- Necropsy status : Planned sacrifice
- Date of death : 13.Jun.2013
  62 days after start of exposure
  1 day after end of exposure

Macroscopic findings
Animal without particular findings.

Microscopic findings
All organs examined without pathologic findings.

Animal 737

General information
- Sex : Male
- Group : 3 (20 RON/ 0.25 FLT/ 30 590F mg/kg)
- Sacrifice : Subset 3 (PND 83+-2)
- Necropsy status : Planned sacrifice
- Date of death : 13.Jun.2013
  62 days after start of exposure
  1 day after end of exposure

Macroscopic findings
Animal without particular findings.

Microscopic findings
All organs examined without pathologic findings.

Animal 738

General information
- Sex : Male
- Group : 3 (20 RON/ 0.25 FLT/ 30 590F mg/kg)
- Sacrifice : Subset 3 (PND 83+-2)
- Necropsy status : Planned sacrifice
- Date of death : 13.Jun.2013
  62 days after start of exposure
  1 day after end of exposure

SINGLE ANIMAL SHEET
(GROSS LESIONS AND MICROSCOPIC FINDINGS)

Sacrifice R3
Sex M
Group 3
cont. Animal 738

Macroscopic findings
Animal without particular findings.

Microscopic findings
All organs examined without pathologic findings.

Animal 739

General information
Sex : Male
Group : 3 (20 RON/ 0.25 FLT/ 30 590F mg/kg)
Sacrifice : Subset 3 (PND 83+-2)
Necropsy status : Planned sacrifice
Date of death : 13.Jun.2013
61 days after start of exposure
1 day after end of exposure

Macroscopic findings
Animal without particular findings.

Microscopic findings
All organs examined without pathologic findings.

Animal 740

General information
Sex : Male
Group : 3 (20 RON/ 0.25 FLT/ 30 590F mg/kg)
Sacrifice : Subset 3 (PND 83+-2)
Necropsy status : Planned sacrifice
Date of death : 13.Jun.2013
61 days after start of exposure
1 day after end of exposure

Macroscopic findings
Animal without particular findings.

Microscopic findings
All organs examined without pathologic findings.

SINGLE ANIMAL SHEET
(GROSS LESIONS AND MICROSCOPIC FINDINGS)

Sacrifice R3
Sex M
Group 4
Animal 741

General information
Sex : Male
Group : 4 (Flutamide 0.00025 mg/kg)
Sacrifice : Subset 3 (PND 83+-2)
Necropsy status : Planned sacrifice
Date of death : 10.Jun.2013
63 days after start of exposure
1 day after end of exposure

Macroscopic findings
Animal without particular findings.

Microscopic findings
All organs examined without pathologic findings.

Animal 742

General information
Sex : Male
Group : 4 (Flutamide 0.00025 mg/kg)
Sacrifice : Subset 3 (PND 83+-2)
Necropsy status : Planned sacrifice
Date of death : 10.Jun.2013
62 days after start of exposure
1 day after end of exposure

Macroscopic findings
Animal without particular findings.

Microscopic findings
All organs examined without pathologic findings.

Animal 743

General information
Sex : Male
Group : 4 (Flutamide 0.00025 mg/kg)
Sacrifice : Subset 3 (PND 83+-2)
Necropsy status : Planned sacrifice
Date of death : 10.Jun.2013
61 days after start of exposure
1 day after end of exposure

SINGLE ANIMAL SHEET
(GROSS LESIONS AND MICROSCOPIC FINDINGS)

Sacrifice R3
Sex M
Group 4
cont. Animal 743

Macroscopic findings
Animal without particular findings.

Microscopic findings
All organs examined without pathologic findings.

Animal 744

General information
Sex : Male
Group : 4 (Flutamide 0.00025 mg/kg)
Sacrifice : Subset 3 (PND 83+-2)
Necropsy status : Planned sacrifice
Date of death : 10.Jun.2013
61 days after start of exposure
1 day after end of exposure

Macroscopic findings
Animal without particular findings.

Microscopic findings
All organs examined without pathologic findings.

Animal 745

General information
Sex : Male
Group : 4 (Flutamide 0.00025 mg/kg)
Sacrifice : Subset 3 (PND 83+-2)
Necropsy status : Planned sacrifice
Date of death : 13.Jun.2013
63 days after start of exposure
1 day after end of exposure

Macroscopic findings
Animal without particular findings.

Microscopic findings
All organs examined without pathologic findings.

Animal 746

General information
Sex : Male
Group : 4 (Flutamide 0.00025 mg/kg)
Sacrifice : Subset 3 (PND 83+-2)
Necropsy status : Planned sacrifice
Date of death : 13.Jun.2013
63 days after start of exposure

SINGLE ANIMAL SHEET
(GROSS LESIONS AND MICROSCOPIC FINDINGS)

Sacrifice R3
Sex M
Group 4
cont. Animal 746

1 day after end of exposure

Macroscopic findings
  Animal without particular findings.

Microscopic findings
  All organs examined without pathologic findings.

Animal 747

General information
  Sex : Male
  Group : 4 (Flutamide 0.00025 mg/kg)
  Sacrifice : Subset 3 (PND 83+-2)
  Necropsy status : Planned sacrifice
  Date of death : 13.Jun.2013
  62 days after start of exposure
  1 day after end of exposure

Macroscopic findings
  Kidneys
    Cyst, right side, diameter 2.0 mm.
  All other organs without macroscopic findings.

Microscopic findings
  Kidneys
    Gross lesion(s) evaluated histopathologically.
    Cyst(s), unilateral, correlates to gross lesion Cyst.
  All other organs examined without microscopic findings.

Animal 748

General information
  Sex : Male
  Group : 4 (Flutamide 0.00025 mg/kg)
  Sacrifice : Subset 3 (PND 83+-2)
  Necropsy status : Planned sacrifice
  Date of death : 13.Jun.2013
  62 days after start of exposure
  1 day after end of exposure

SINGLE ANIMAL SHEET
(GROSS LESIONS AND MICROSCOPIC FINDINGS)

Sacrifice R3
Sex M
Group 4
cont. Animal 748

Macroscopic findings
Animal without particular findings.

Microscopic findings
All organs examined without pathologic findings.

Animal 749

General information
Sex : Male
Group : 4 (Flutamide 0.00025 mg/kg)
Sacrifice : Subset 3 (PND 83+-2)
Necropsy status : Planned sacrifice
Date of death : 13.Jun.2013
62 days after start of exposure
1 day after end of exposure

Macroscopic findings
Animal without particular findings.

Microscopic findings
All organs examined without pathologic findings.

Animal 750

General information
Sex : Male
Group : 4 (Flutamide 0.00025 mg/kg)
Sacrifice : Subset 3 (PND 83+-2)
Necropsy status : Planned sacrifice
Date of death : 13.Jun.2013
62 days after start of exposure
1 day after end of exposure

Macroscopic findings
Animal without particular findings.

Microscopic findings
All organs examined without pathologic findings.

SINGLE ANIMAL SHEET
(GROSS LESIONS AND MICROSCOPIC FINDINGS)

Sacrifice	R3
Sex	F
Group	0
Animal	801

General information
Sex : Female
Group : 0 (0 mg/kg)
Sacrifice : Subset 3 (PND 83+-2)
Necropsy status : Planned sacrifice
Date of death : 10.Jun.2013
62 days after start of exposure
1 day after end of exposure

Macroscopic findings
Animal without particular findings.

Microscopic findings
No histologic examination performed.

Animal 802

General information
Sex : Female
Group : 0 (0 mg/kg)
Sacrifice : Subset 3 (PND 83+-2)
Necropsy status : Planned sacrifice
Date of death : 10.Jun.2013
62 days after start of exposure
1 day after end of exposure

Macroscopic findings
Animal without particular findings.

Microscopic findings
No histologic examination performed.

Animal 803

General information
Sex : Female
Group : 0 (0 mg/kg)
Sacrifice : Subset 3 (PND 83+-2)
Necropsy status : Planned sacrifice
Date of death : 10.Jun.2013
62 days after start of exposure
1 day after end of exposure

SINGLE ANIMAL SHEET
(GROSS LESIONS AND MICROSCOPIC FINDINGS)

Sacrifice R3
Sex F
Group 0
cont. Animal 803

Macroscopic findings
Animal without particular findings.

Microscopic findings
No histologic examination performed.

Animal 804

General information
Sex : Female
Group : 0 (0 mg/kg)
Sacrifice : Subset 3 (PND 83+-2)
Necropsy status : Planned sacrifice
Date of death : 10.Jun.2013
62 days after start of exposure
1 day after end of exposure

Macroscopic findings
Animal without particular findings.

Microscopic findings
No histologic examination performed.

Animal 805

General information
Sex : Female
Group : 0 (0 mg/kg)
Sacrifice : Subset 3 (PND 83+-2)
Necropsy status : Sacrificed moribund
Date of death : 23.Apr.2013
13 days after start of exposure
1 day after end of exposure

Macroscopic findings
Animal without particular findings.

Microscopic findings
No histologic examination performed.

Animal 806

General information
Sex : Female
Group : 0 (0 mg/kg)
Sacrifice : Subset 3 (PND 83+-2)
Necropsy status : Planned sacrifice
Date of death : 10.Jun.2013
61 days after start of exposure

SINGLE ANIMAL SHEET
(GROSS LESIONS AND MICROSCOPIC FINDINGS)

Sacrifice R3
Sex F
Group 0
cont. Animal 806

1 day after end of exposure

Macroscopic findings
Animal without particular findings.

Microscopic findings
No histologic examination performed.

Animal 807

General information
Sex : Female
Group : 0 (0 mg/kg)
Sacrifice : Subset 3 (PND 83+-2)
Necropsy status : Planned sacrifice
Date of death : 13.Jun.2013
63 days after start of exposure
1 day after end of exposure

Macroscopic findings
Animal without particular findings.

Microscopic findings
No histologic examination performed.

Animal 808

General information
Sex : Female
Group : 0 (0 mg/kg)
Sacrifice : Subset 3 (PND 83+-2)
Necropsy status : Planned sacrifice
Date of death : 13.Jun.2013
63 days after start of exposure
1 day after end of exposure

Macroscopic findings
Animal without particular findings.

Microscopic findings
No histologic examination performed.

SINGLE ANIMAL SHEET
(GROSS LESIONS AND MICROSCOPIC FINDINGS)

Sacrifice R3
Sex F
Group 0
Animal 809

General information
Sex : Female
Group : 0 (0 mg/kg)
Sacrifice : Subset 3 (PND 83+-2)
Necropsy status : Planned sacrifice
Date of death : 13.Jun.2013
63 days after start of exposure
1 day after end of exposure

Macroscopic findings
Animal without particular findings.

Microscopic findings
No histologic examination performed.

Animal 810

General information
Sex : Female
Group : 0 (0 mg/kg)
Sacrifice : Subset 3 (PND 83+-2)
Necropsy status : Planned sacrifice
Date of death : 13.Jun.2013
62 days after start of exposure
1 day after end of exposure

Macroscopic findings
Animal without particular findings.

Microscopic findings
No histologic examination performed.

SINGLE ANIMAL SHEET
(GROSS LESIONS AND MICROSCOPIC FINDINGS)

Sacrifice R3
Sex F
Group 1
Animal 811

General information
Sex : Female
Group : 1 (0.005RON/0.00025FLT/0.01 590F mg/kg)
Sacrifice : Subset 3 (PND 83+-2)
Necropsy status : Planned sacrifice
Date of death : 10.Jun.2013
62 days after start of exposure
1 day after end of exposure

Macroscopic findings
Animal without particular findings.

Microscopic findings
No histologic examination performed.

Animal 812

General information
Sex : Female
Group : 1 (0.005RON/0.00025FLT/0.01 590F mg/kg)
Sacrifice : Subset 3 (PND 83+-2)
Necropsy status : Planned sacrifice
Date of death : 10.Jun.2013
62 days after start of exposure
1 day after end of exposure

Macroscopic findings
Animal without particular findings.

Microscopic findings
No histologic examination performed.

Animal 813

General information
Sex : Female
Group : 1 (0.005RON/0.00025FLT/0.01 590F mg/kg)
Sacrifice : Subset 3 (PND 83+-2)
Necropsy status : Planned sacrifice
Date of death : 10.Jun.2013
62 days after start of exposure
1 day after end of exposure

SINGLE ANIMAL SHEET
(GROSS LESIONS AND MICROSCOPIC FINDINGS)

Sacrifice R3
Sex F
Group 1
cont. Animal 813

Macroscopic findings
  Animal without particular findings.

Microscopic findings
  No histologic examination performed.

Animal 814

General information
  Sex : Female
  Group : 1 (0.005RON/0.00025FLT/0.01 590F mg/kg)
  Sacrifice : Subset 3 (PND 83+-2)
  Necropsy status : Planned sacrifice
  Date of death : 10.Jun.2013
  62 days after start of exposure
  1 day after end of exposure

Macroscopic findings
  Iliac lymph nodes
  * Tissue preserved in cassette, right side.
  Uterus
    Discoloration, right side, length 10.0 mm, red.
    Mass, right side, diameter 5.0 mm, red.
  All other organs without macroscopic findings.

Microscopic findings
  Uterus
    Histopathologic evaluation of gross lesion(s) will not be performed.
  No histologic examination performed.

Animal 815

General information
  Sex : Female
  Group : 1 (0.005RON/0.00025FLT/0.01 590F mg/kg)
  Sacrifice : Subset 3 (PND 83+-2)
  Necropsy status : Planned sacrifice
  Date of death : 13.Jun.2013
  64 days after start of exposure
  1 day after end of exposure

SINGLE ANIMAL SHEET
(GROSS LESIONS AND MICROSCOPIC FINDINGS)

Sacrifice R3
Sex F
Group 1
cont. Animal 815

Macroscopic findings
Animal without particular findings.

Microscopic findings
No histologic examination performed.

Animal 816

General information
Sex : Female
Group : 1 (0.005RON/0.00025FLT/0.01 590F mg/kg)
Sacrifice : Subset 3 (PND 83+-2)
Necropsy status : Planned sacrifice
Date of death : 10.Jun.2013
61 days after start of exposure
1 day after end of exposure

Macroscopic findings
Animal without particular findings.

Microscopic findings
No histologic examination performed.

Animal 817

General information
Sex : Female
Group : 1 (0.005RON/0.00025FLT/0.01 590F mg/kg)
Sacrifice : Subset 3 (PND 83+-2)
Necropsy status : Planned sacrifice
Date of death : 10.Jun.2013
61 days after start of exposure
1 day after end of exposure

Macroscopic findings
Animal without particular findings.

Microscopic findings
No histologic examination performed.

Animal 818

General information
Sex : Female
Group : 1 (0.005RON/0.00025FLT/0.01 590F mg/kg)
Sacrifice : Subset 3 (PND 83+-2)
Necropsy status : Planned sacrifice
Date of death : 10.Jun.2013
60 days after start of exposure

SINGLE ANIMAL SHEET
(GROSS LESIONS AND MICROSCOPIC FINDINGS)

Sacrifice R3
Sex F
Group 1
cont. Animal 818

1 day after end of exposure

Macroscopic findings
Animal without particular findings.

Microscopic findings
No histologic examination performed.

Animal 819

General information
Sex : Female
Group : 1 (0.005RON/0.00025FLT/0.01 590F mg/kg)
Sacrifice : Subset 3 (PND 83+-2)
Necropsy status : Planned sacrifice
Date of death : 13.Jun.2013
63 days after start of exposure
1 day after end of exposure

Macroscopic findings
Animal without particular findings.

Microscopic findings
No histologic examination performed.

Animal 820

General information
Sex : Female
Group : 1 (0.005RON/0.00025FLT/0.01 590F mg/kg)
Sacrifice : Subset 3 (PND 83+-2)
Necropsy status : Planned sacrifice
Date of death : 13.Jun.2013
63 days after start of exposure
1 day after end of exposure

Macroscopic findings
Animal without particular findings.

Microscopic findings
No histologic examination performed.

SINGLE ANIMAL SHEET
(GROSS LESIONS AND MICROSCOPIC FINDINGS)

Sacrifice R3
Sex F
Group 2
Animal 821

General information
Sex : Female
Group : 2 (4 RON/ 0.025 FLT/ 5 590F mg/kg)
Sacrifice : Subset 3 (PND 83+-2)
Necropsy status : Planned sacrifice
Date of death : 10.Jun.2013
62 days after start of exposure
1 day after end of exposure

Macroscopic findings
Animal without particular findings.

Microscopic findings
No histologic examination performed.

Animal 822

General information
Sex : Female
Group : 2 (4 RON/ 0.025 FLT/ 5 590F mg/kg)
Sacrifice : Subset 3 (PND 83+-2)
Necropsy status : Planned sacrifice
Date of death : 10.Jun.2013
62 days after start of exposure
1 day after end of exposure

Macroscopic findings
Kidneys
Cyst, right side, diameter 3.0 mm.
All other organs without macroscopic findings.

Microscopic findings
Kidneys
Histopathologic evaluation of gross lesion(s) will not be performed.
No histologic examination performed.

Animal 823

General information
Sex : Female
Group : 2 (4 RON/ 0.025 FLT/ 5 590F mg/kg)
Sacrifice : Subset 3 (PND 83+-2)
Necropsy status : Planned sacrifice
Date of death : 10.Jun.2013
62 days after start of exposure
1 day after end of exposure

SINGLE ANIMAL SHEET
(GROSS LESIONS AND MICROSCOPIC FINDINGS)

Sacrifice R3
Sex F
Group 2
cont. Animal 823

Macroscopic findings
Animal without particular findings.

Microscopic findings
No histologic examination performed.

Animal 824

General information
Sex : Female
Group : 2 (4 RON/ 0.025 FLT/ 5 590F mg/kg)
Sacrifice : Subset 3 (PND 83+-2)
Necropsy status : Planned sacrifice
Date of death : 10.Jun.2013
61 days after start of exposure
1 day after end of exposure

Macroscopic findings
Animal without particular findings.

Microscopic findings
No histologic examination performed.

Animal 825

General information
Sex : Female
Group : 2 (4 RON/ 0.025 FLT/ 5 590F mg/kg)
Sacrifice : Subset 3 (PND 83+-2)
Necropsy status : Planned sacrifice
Date of death : 10.Jun.2013
61 days after start of exposure
1 day after end of exposure

Macroscopic findings
Animal without particular findings.

Microscopic findings
No histologic examination performed.

Animal 826

General information
Sex : Female
Group : 2 (4 RON/ 0.025 FLT/ 5 590F mg/kg)
Sacrifice : Subset 3 (PND 83+-2)
Necropsy status : Planned sacrifice
Date of death : 13.Jun.2013
63 days after start of exposure

SINGLE ANIMAL SHEET
(GROSS LESIONS AND MICROSCOPIC FINDINGS)

Sacrifice R3
Sex F
Group 2
cont. Animal 826

1 day after end of exposure

Macroscopic findings
Animal without particular findings.

Microscopic findings
No histologic examination performed.

Animal 827

General information
Sex : Female
Group : 2 (4 RON/ 0.025 FLT/ 5 590F mg/kg)
Sacrifice : Subset 3 (PND 83+-2)
Necropsy status : Planned sacrifice
Date of death : 13.Jun.2013
63 days after start of exposure
1 day after end of exposure

Macroscopic findings
Animal without particular findings.

Microscopic findings
No histologic examination performed.

Animal 828

General information
Sex : Female
Group : 2 (4 RON/ 0.025 FLT/ 5 590F mg/kg)
Sacrifice : Subset 3 (PND 83+-2)
Necropsy status : Planned sacrifice
Date of death : 13.Jun.2013
62 days after start of exposure
1 day after end of exposure

Macroscopic findings
Animal without particular findings.

Microscopic findings
No histologic examination performed.

SINGLE ANIMAL SHEET
(GROSS LESIONS AND MICROSCOPIC FINDINGS)

Sacrifice R3
Sex F
Group 2
Animal 829

General information
Sex : Female
Group : 2 (4 RON/ 0.025 FLT/ 5 590F mg/kg)
Sacrifice : Subset 3 (PND 83+-2)
Necropsy status : Planned sacrifice
Date of death : 13.Jun.2013
62 days after start of exposure
1 day after end of exposure

Macroscopic findings
Animal without particular findings.
Uterus
* Organ damaged at necropsy.

Microscopic findings
No histologic examination performed.

Animal 830

General information
Sex : Female
Group : 2 (4 RON/ 0.025 FLT/ 5 590F mg/kg)
Sacrifice : Subset 3 (PND 83+-2)
Necropsy status : Planned sacrifice
Date of death : 13.Jun.2013
61 days after start of exposure
1 day after end of exposure

Macroscopic findings
Animal without particular findings.

Microscopic findings
No histologic examination performed.

SINGLE ANIMAL SHEET
(GROSS LESIONS AND MICROSCOPIC FINDINGS)

Sacrifice R3
Sex F
Group 3
Animal 831

General information
Sex : Female
Group : 3 (20 RON/ 0.25 FLT/ 30 590F mg/kg)
Sacrifice : Subset 3 (PND 83+-2)
Necropsy status : Planned sacrifice
Date of death : 10.Jun.2013
62 days after start of exposure
1 day after end of exposure

Macroscopic findings
Animal without particular findings.

Microscopic findings
No histologic examination performed.

Animal 832

General information
Sex : Female
Group : 3 (20 RON/ 0.25 FLT/ 30 590F mg/kg)
Sacrifice : Subset 3 (PND 83+-2)
Necropsy status : Planned sacrifice
Date of death : 10.Jun.2013
61 days after start of exposure
1 day after end of exposure

Macroscopic findings
Animal without particular findings.

Microscopic findings
No histologic examination performed.

Animal 833

General information
Sex : Female
Group : 3 (20 RON/ 0.25 FLT/ 30 590F mg/kg)
Sacrifice : Subset 3 (PND 83+-2)
Necropsy status : Planned sacrifice
Date of death : 13.Jun.2013
63 days after start of exposure
1 day after end of exposure

SINGLE ANIMAL SHEET
(GROSS LESIONS AND MICROSCOPIC FINDINGS)

Sacrifice R3
Sex F
Group 3
cont. Animal 833

Macroscopic findings
Animal without particular findings.

Microscopic findings
No histologic examination performed.

Animal 834

General information
Sex : Female
Group : 3 (20 RON/ 0.25 FLT/ 30 590F mg/kg)
Sacrifice : Subset 3 (PND 83+-2)
Necropsy status : Planned sacrifice
Date of death : 13.Jun.2013
63 days after start of exposure
1 day after end of exposure

Macroscopic findings
Animal without particular findings.

Microscopic findings
No histologic examination performed.

Animal 835

General information
Sex : Female
Group : 3 (20 RON/ 0.25 FLT/ 30 590F mg/kg)
Sacrifice : Subset 3 (PND 83+-2)
Necropsy status : Planned sacrifice
Date of death : 13.Jun.2013
63 days after start of exposure
1 day after end of exposure

Macroscopic findings
Animal without particular findings.

Microscopic findings
No histologic examination performed.

Animal 836

General information
Sex : Female
Group : 3 (20 RON/ 0.25 FLT/ 30 590F mg/kg)
Sacrifice : Subset 3 (PND 83+-2)
Necropsy status : Planned sacrifice
Date of death : 13.Jun.2013
62 days after start of exposure

SINGLE ANIMAL SHEET
(GROSS LESIONS AND MICROSCOPIC FINDINGS)

Sacrifice R3
Sex F
Group 3
cont. Animal 836

1 day after end of exposure

Macroscopic findings
Animal without particular findings.

Microscopic findings
No histologic examination performed.

Animal 837

General information
Sex : Female
Group : 3 (20 RON/ 0.25 FLT/ 30 590F mg/kg)
Sacrifice : Subset 3 (PND 83+-2)
Necropsy status : Planned sacrifice
Date of death : 13.Jun.2013
62 days after start of exposure
1 day after end of exposure

Macroscopic findings
Animal without particular findings.

Microscopic findings
No histologic examination performed.

Animal 838

General information
Sex : Female
Group : 3 (20 RON/ 0.25 FLT/ 30 590F mg/kg)
Sacrifice : Subset 3 (PND 83+-2)
Necropsy status : Planned sacrifice
Date of death : 13.Jun.2013
62 days after start of exposure
1 day after end of exposure

Macroscopic findings
Animal without particular findings.

Microscopic findings
No histologic examination performed.

SINGLE ANIMAL SHEET
(GROSS LESIONS AND MICROSCOPIC FINDINGS)

Sacrifice R3
Sex F
Group 3
Animal 839

General information
- Sex : Female
- Group : 3 (20 RON/ 0.25 FLT/ 30 590F mg/kg)
- Sacrifice : Subset 3 (PND 83+-2)
- Necropsy status : Planned sacrifice
- Date of death : 13.Jun.2013
  61 days after start of exposure
  1 day after end of exposure

Macroscopic findings
Animal without particular findings.

Microscopic findings
No histologic examination performed.

Animal 840

General information
- Sex : Female
- Group : 3 (20 RON/ 0.25 FLT/ 30 590F mg/kg)
- Sacrifice : Subset 3 (PND 83+-2)
- Necropsy status : Planned sacrifice
- Date of death : 13.Jun.2013
  61 days after start of exposure
  1 day after end of exposure

Macroscopic findings
Animal without particular findings.

Microscopic findings
No histologic examination performed.

SINGLE ANIMAL SHEET
(GROSS LESIONS AND MICROSCOPIC FINDINGS)

Sacrifice R3
Sex F
Group 4
Animal 841

General information
Sex : Female
Group : 4 (Flutamide 0.00025 mg/kg)
Sacrifice : Subset 3 (PND 83+-2)
Necropsy status : Planned sacrifice
Date of death : 10.Jun.2013
62 days after start of exposure
1 day after end of exposure

Macroscopic findings
Animal without particular findings.

Microscopic findings
No histologic examination performed.

Animal 842

General information
Sex : Female
Group : 4 (Flutamide 0.00025 mg/kg)
Sacrifice : Subset 3 (PND 83+-2)
Necropsy status : Planned sacrifice
Date of death : 10.Jun.2013
62 days after start of exposure
1 day after end of exposure

Macroscopic findings
Animal without particular findings.

Microscopic findings
No histologic examination performed.

Animal 843

General information
Sex : Female
Group : 4 (Flutamide 0.00025 mg/kg)
Sacrifice : Subset 3 (PND 83+-2)
Necropsy status : Planned sacrifice
Date of death : 10.Jun.2013
62 days after start of exposure
1 day after end of exposure

SINGLE ANIMAL SHEET
(GROSS LESIONS AND MICROSCOPIC FINDINGS)

Sacrifice R3
Sex F
Group 4
cont. Animal 843

Macroscopic findings
Animal without particular findings.

Microscopic findings
No histologic examination performed.

Animal 844

General information
Sex : Female
Group : 4 (Flutamide 0.00025 mg/kg)
Sacrifice : Subset 3 (PND 83+-2)
Necropsy status : Planned sacrifice
Date of death : 10.Jun.2013
62 days after start of exposure
1 day after end of exposure

Macroscopic findings
Animal without particular findings.

Microscopic findings
No histologic examination performed.

Animal 845

General information
Sex : Female
Group : 4 (Flutamide 0.00025 mg/kg)
Sacrifice : Subset 3 (PND 83+-2)
Necropsy status : Planned sacrifice
Date of death : 10.Jun.2013
61 days after start of exposure
1 day after end of exposure

Macroscopic findings
Animal without particular findings.

Microscopic findings
No histologic examination performed.

Animal 846

General information
Sex : Female
Group : 4 (Flutamide 0.00025 mg/kg)
Sacrifice : Subset 3 (PND 83+-2)
Necropsy status : Planned sacrifice
Date of death : 10.Jun.2013
61 days after start of exposure

SINGLE ANIMAL SHEET
(GROSS LESIONS AND MICROSCOPIC FINDINGS)

Sacrifice R3
Sex F
Group 4
cont. Animal 846

1 day after end of exposure

Macroscopic findings
  Animal without particular findings.

Microscopic findings
  No histologic examination performed.

Animal 847

General information
  Sex : Female
  Group : 4 (Flutamide 0.00025 mg/kg)
  Sacrifice : Subset 3 (PND 83+-2)
  Necropsy status : Planned sacrifice
  Date of death : 13.Jun.2013
  64 days after start of exposure
  1 day after end of exposure

Macroscopic findings
  Animal without particular findings.

Microscopic findings
  No histologic examination performed.

Animal 848

General information
  Sex : Female
  Group : 4 (Flutamide 0.00025 mg/kg)
  Sacrifice : Subset 3 (PND 83+-2)
  Necropsy status : Planned sacrifice
  Date of death : 13.Jun.2013
  62 days after start of exposure
  1 day after end of exposure

Macroscopic findings
  Animal without particular findings.

Microscopic findings
  No histologic examination performed.

SINGLE ANIMAL SHEET
(GROSS LESIONS AND MICROSCOPIC FINDINGS)

Sacrifice	R3
Sex	F
Group	4
Animal	849

General information

Sex : Female
Group : 4 (Flutamide 0.00025 mg/kg)
Sacrifice : Subset 3 (PND 83+-2)
Necropsy status : Planned sacrifice
Date of death : 13.Jun.2013
62 days after start of exposure
1 day after end of exposure

Macroscopic findings

Animal without particular findings.

Microscopic findings

No histologic examination performed.

Animal 850

General information

Sex : Female
Group : 4 (Flutamide 0.00025 mg/kg)
Sacrifice : Subset 3 (PND 83+-2)
Necropsy status : Planned sacrifice
Date of death : 13.Jun.2013
62 days after start of exposure
1 day after end of exposure

Macroscopic findings

Animal without particular findings.

Microscopic findings

No histologic examination performed.
