# Supplementary material for: Investigations on the dose–response relationship of combined exposure to low doses of three anti-androgens in Wistar rats
Source: Arch Toxicol. 2017 Sep 6;91(12):3961–89. doi: 10.1007/s00204-017-2053-3 (PMC5719133; doi:10.1007/s00204-017-2053-3)
Supplement: Supplementary file 6 — Supplementary material 6 (PDF 4710 kb) [file 204_2017_2053_MOESM6_ESM.pdf]

PR.NO.60R0375/88R002: REPRODUCTIVE TOX. STUDY TO DETECT EFFECTS
OF MIXED ANTI-ANDROGENIC SUBSTANCES IN RATS; ORAL ADM. (GAVAGE)
SUMMARY OF MATERNAL CLINICAL OBSERVATIONS DURING GESTATION

	GROUP#	DAY OF GESTATION 0	1	2	3	4	5	6	7	8	9	10	11	12	13	14	15	16	17	18	19	20	21	22	23	24	25	TOTAL
# OF FEMALES EXAMINED	0	25	25	25	25	25	25	25	25	25	25	25	25	25	25	25	25	25	25	25	25	25	19	0	0	0	0	
	1	25	25	25	25	25	25	25	25	25	25	25	25	25	25	25	25	25	25	25	25	25	15	0	0	0	0	
	2	25	25	25	25	25	25	25	25	25	25	25	25	25	25	25	25	25	25	25	25	25	20	6	0	0	0	
	3	25	25	25	25	25	25	25	25	25	25	25	25	25	25	25	25	25	25	25	25	25	20	18	2	0	0	
	4	25	25	25	25	25	25	25	25	25	25	25	25	25	25	25	25	25	25	25	25	25	18	4	1	1	1	
NORMAL																												
NOTHING ABNORMAL DETECTED	0	25	25	25	25	25	25	25	25	25	25	25	25	25	25	25	25	25	25	25	25	25	19	-	-	-	-	25
	1	25	25	25	25	25	25	25	25	25	25	25	25	25	25	25	25	25	25	25	25	25	15	-	-	-	-	25
	2	25	25	25	25	25	25	25	25	25	25	25	25	25	25	25	25	24	23	25	25	24	19	6	-	-	-	25
	3	25	25	25	25	25	25	25	25	25	25	25	21	20	12	11	10	8	6	12	11	12	5	4	0	-	-	25
	4	25	25	25	25	25	25	25	25	25	25	25	25	25	25	25	25	25	25	25	25	25	18	4	1	1	0	25
DEAD																												
SCHEDULED SACRIFICE	0	0	0	0	0	0	0	0	0	0	0	0	0	0	0	0	0	0	0	0	0	5	0	-	-	-	-	5
	1	0	0	0	0	0	0	0	0	0	0	0	0	0	0	0	0	0	0	0	0	5	0	-	-	-	-	5
	2	0	0	0	0	0	0	0	0	0	0	0	0	0	0	0	0	0	0	0	0	5	0	0	-	-	-	5
	3	0	0	0	0	0	0	0	0	0	0	0	0	0	0	0	0	0	0	0	0	5	0	0	0	-	-	5
	4	0	0	0	0	0	0	0	0	0	0	0	0	0	0	0	0	0	0	0	0	5	0	0	0	0	1	6
SACRIFICED MORIBUND	0	0	0	0	0	0	0	0	0	0	0	0	0	0	0	0	0	0	0	0	0	0	0	-	-	-	-	0
	1	0	0	0	0	0	0	0	0	0	0	0	0	0	0	0	0	0	0	0	0	0	0	-	-	-	-	0
	2	0	0	0	0	0	0	0	0	0	0	0	0	0	0	0	0	0	0	0	0	0	0	0	-	-	-	0
	3	0	0	0	0	0	0	0	0	0	0	0	0	0	0	0	0	0	0	0	0	0	0	0	1	-	-	1
	4	0	0	0	0	0	0	0	0	0	0	0	0	0	0	0	0	0	0	0	0	0	0	0	0	0	0	0
BODY POSITION																												
ABDOMINAL POSITION	0	0	0	0	0	0	0	0	0	0	0	0	0	0	0	0	0	0	0	0	0	0	0	-	-	-	-	0
	1	0	0	0	0	0	0	0	0	0	0	0	0	0	0	0	0	0	0	0	0	0	0	-	-	-	-	0
	2	0	0	0	0	0	0	0	0	0	0	0	0	0	0	0	0	0	0	0	0	0	0	0	-	-	-	0
	3	0	0	0	0	0	0	0	0	0	0	0	0	0	0	0	0	0	0	0	0	0	0	0	1	-	-	1
	4	0	0	0	0	0	0	0	0	0	0	0	0	0	0	0	0	0	0	0	0	0	0	0	0	0	0	0
GENERAL STATE																												
HYPOTHERMIA	0	0	0	0	0	0	0	0	0	0	0	0	0	0	0	0	0	0	0	0	0	0	0	-	-	-	-	0
	1	0	0	0	0	0	0	0	0	0	0	0	0	0	0	0	0	0	0	0	0	0	0	-	-	-	-	0
	2	0	0	0	0	0	0	0	0	0	0	0	0	0	0	0	0	0	0	0	0	0	0	0	-	-	-	0
	3	0	0	0	0	0	0	0	0	0	0	0	0	0	0	0	0	0	0	0	0	0	0	0	1	-	-	1
	4	0	0	0	0	0	0	0	0	0	0	0	0	0	0	0	0	0	0	0	0	0	0	0	0	0	0	0

PR.NO.60R0375/88R002: REPRODUCTIVE TOX. STUDY TO DETECT EFFECTS
OF MIXED ANTI-ANDROGENIC SUBSTANCES IN RATS; ORAL ADM. (GAVAGE)
SUMMARY OF MATERNAL CLINICAL OBSERVATIONS DURING GESTATION

		DAY OF GESTATION																										
	GROUP#	0	1	2	3	4	5	6	7	8	9	10	11	12	13	14	15	16	17	18	19	20	21	22	23	24	25	TOTAL
# OF FEMALES EXAMINED	0	25	25	25	25	25	25	25	25	25	25	25	25	25	25	25	25	25	25	25	25	25	19	0	0	0	0	
	1	25	25	25	25	25	25	25	25	25	25	25	25	25	25	25	25	25	25	25	25	25	15	0	0	0	0	
	2	25	25	25	25	25	25	25	25	25	25	25	25	25	25	25	25	25	25	25	25	25	20	6	0	0	0	
	3	25	25	25	25	25	25	25	25	25	25	25	25	25	25	25	25	25	25	25	25	25	20	18	2	0	0	
	4	25	25	25	25	25	25	25	25	25	25	25	25	25	25	25	25	25	25	25	25	25	18	4	1	1	1	
MISCELLANEOUS																												
NO PUPS DELIVERED (F1)	0	0	0	0	0	0	0	0	0	0	0	0	0	0	0	0	0	0	0	0	0	0	0	-	-	-	-	0
	1	0	0	0	0	0	0	0	0	0	0	0	0	0	0	0	0	0	0	0	0	0	0	-	-	-	-	0
	2	0	0	0	0	0	0	0	0	0	0	0	0	0	0	0	0	0	0	0	0	0	0	0	-	-	-	0
	3	0	0	0	0	0	0	0	0	0	0	0	0	0	0	0	0	0	0	0	0	0	0	0	0	-	-	0
	4	0	0	0	0	0	0	0	0	0	0	0	0	0	0	0	0	0	0	0	0	0	0	0	0	0	1	1
ORAL-BUCCAL																												
SALIVATION AFTER TREATMENT	0	0	0	0	0	0	0	0	0	0	0	0	0	0	0	0	0	0	0	0	0	0	0	-	-	-	-	0
	1	0	0	0	0	0	0	0	0	0	0	0	0	0	0	0	0	0	0	0	0	0	0	-	-	-	-	0
	2	0	0	0	0	0	0	0	0	0	0	0	0	0	0	0	0	1	2	0	0	1	1	0	-	-	-	2
	3	0	0	0	0	0	0	0	0	0	0	0	4	5	13	14	15	17	19	13	14	13	15	14	1	-	-	25
	4	0	0	0	0	0	0	0	0	0	0	0	0	0	0	0	0	0	0	0	0	0	0	0	0	0	0	0
SKIN/FUR																												
PILOERECTION	0	0	0	0	0	0	0	0	0	0	0	0	0	0	0	0	0	0	0	0	0	0	0	-	-	-	-	0
	1	0	0	0	0	0	0	0	0	0	0	0	0	0	0	0	0	0	0	0	0	0	0	-	-	-	-	0
	2	0	0	0	0	0	0	0	0	0	0	0	0	0	0	0	0	0	0	0	0	0	0	0	-	-	-	0
	3	0	0	0	0	0	0	0	0	0	0	0	0	0	0	0	0	0	0	0	0	0	0	0	1	-	-	1
	4	0	0	0	0	0	0	0	0	0	0	0	0	0	0	0	0	0	0	0	0	0	0	0	0	0	0	0
DAM / LITTER																												
UNABLE TO DELIVER	0	0	0	0	0	0	0	0	0	0	0	0	0	0	0	0	0	0	0	0	0	0	0	-	-	-	-	0
	1	0	0	0	0	0	0	0	0	0	0	0	0	0	0	0	0	0	0	0	0	0	0	-	-	-	-	0
	2	0	0	0	0	0	0	0	0	0	0	0	0	0	0	0	0	0	0	0	0	0	0	0	-	-	-	0
	3	0	0	0	0	0	0	0	0	0	0	0	0	0	0	0	0	0	0	0	0	0	0	0	1	-	-	1
	4	0	0	0	0	0	0	0	0	0	0	0	0	0	0	0	0	0	0	0	0	0	0	0	0	0	0	0

PR.NO.60R0375/88R002: REPRODUCTIVE TOX. STUDY TO DETECT EFFECTS
OF MIXED ANTI-ANDROGENIC SUBSTANCES IN RATS; ORAL ADM. (GAVAGE)
SUMMARY OF MATERNAL/PUP CLINICAL OBSERVATIONS DURING LACTATION

	GROUP#	DAY OF LACTATION 0	1	2	3	4	5	6	7	8	9	10	11	12	13	14	15	16	17	18	19	20	21	22	23	24	25	26	27	28	29	30	TOTAL
# OF FEMALES EXAMINED	0	20	20	20	20	20	20	20	20	20	20	20	20	20	20	20	20	20	20	20	20	20	20	20	20	20	20	20	20	20	20	20	
	1	20	20	20	20	20	20	20	20	20	20	20	20	20	20	20	20	20	20	20	20	20	20	20	20	20	20	20	20	20	20	20	
	2	20	20	20	20	20	20	20	20	20	20	20	20	20	20	20	20	20	20	20	20	20	20	20	20	20	20	20	20	20	20	20	
	3	19	19	19	19	19	19	19	19	19	19	19	19	19	19	19	19	19	19	19	19	19	19	19	19	19	19	19	19	19	19	19	
	4	19	19	19	19	19	19	19	19	19	19	19	19	19	19	19	19	19	19	19	19	19	19	19	19	19	19	19	19	19	19	19	
NORMAL																																	
NOTHING ABNORMAL DETECTED	0	20	20	20	20	20	20	20	20	20	20	20	20	20	20	20	20	20	20	20	20	20	20	20	20	20	20	20	20	20	20	20	20
	1	20	20	20	20	20	20	20	20	20	20	20	20	20	20	20	20	20	20	20	20	20	20	20	20	20	20	20	20	20	20	20	20
	2	20	19	19	19	19	20	20	20	20	20	20	19	19	20	20	20	20	19	19	20	19	19	20	19	19	18	19	18	19	20	18	20
	3	0	0	0	0	0	0	0	0	0	0	0	0	0	0	0	0	0	0	0	0	0	0	0	0	0	0	0	0	0	0	0	0
	4	19	18	19	19	19	19	19	19	19	19	19	19	19	19	19	19	19	19	19	19	19	19	19	19	19	19	19	19	19	19	19	19
ORAL-BUCCAL																																	
SALIVATION AFTER TREATMENT	0	0	0	0	0	0	0	0	0	0	0	0	0	0	0	0	0	0	0	0	0	0	0	0	0	0	0	0	0	0	0	0	0
	1	0	0	0	0	0	0	0	0	0	0	0	0	0	0	0	0	0	0	0	0	0	0	0	0	0	0	0	0	0	0	0	0
	2	0	0	0	0	0	0	0	0	0	0	0	1	1	0	0	0	0	1	1	0	1	1	0	1	1	2	1	2	1	0	2	3
	3	18	19	19	19	19	19	19	19	19	19	19	19	19	19	19	19	19	19	19	19	19	19	19	19	19	19	19	19	19	19	19	19
	4	0	0	0	0	0	0	0	0	0	0	0	0	0	0	0	0	0	0	0	0	0	0	0	0	0	0	0	0	0	0	0	0
DAM / LITTER																																	
ALL PUPS STILLBORN	0	0	0	0	0	0	0	0	0	0	0	0	0	0	0	0	0	0	0	0	0	0	0	0	0	0	0	0	0	0	0	0	0
	1	0	0	0	0	0	0	0	0	0	0	0	0	0	0	0	0	0	0	0	0	0	0	0	0	0	0	0	0	0	0	0	0
	2	0	0	0	0	0	0	0	0	0	0	0	0	0	0	0	0	0	0	0	0	0	0	0	0	0	0	0	0	0	0	0	0
	3	2	0	0	0	0	0	0	0	0	0	0	0	0	0	0	0	0	0	0	0	0	0	0	0	0	0	0	0	0	0	0	2
	4	0	0	0	0	0	0	0	0	0	0	0	0	0	0	0	0	0	0	0	0	0	0	0	0	0	0	0	0	0	0	0	0
PUP(S) NOT PROPERLY NURSED (NO/LESS MILK IN STOMACH)	0	0	0	0	0	0	0	0	0	0	0	0	0	0	0	0	0	0	0	0	0	0	0	0	0	0	0	0	0	0	0	0	0
	1	0	0	0	0	0	0	0	0	0	0	0	0	0	0	0	0	0	0	0	0	0	0	0	0	0	0	0	0	0	0	0	0
	2	0	1	1	1	1	0	0	0	0	0	0	0	0	0	0	0	0	0	0	0	0	0	0	0	0	0	0	0	0	0	0	1
	3	0	0	0	0	0	0	0	0	0	0	0	0	0	0	0	0	0	0	0	0	0	0	0	0	0	0	0	0	0	0	0	0
	4	0	0	0	0	0	0	0	0	0	0	0	0	0	0	0	0	0	0	0	0	0	0	0	0	0	0	0	0	0	0	0	0
NO MORE PUPS ALIVE	0	0	0	0	0	0	0	0	0	0	0	0	0	0	0	0	0	0	0	0	0	0	0	0	0	0	0	0	0	0	0	0	0
	1	0	0	0	0	0	0	0	0	0	0	0	0	0	0	0	0	0	0	0	0	0	0	0	0	0	0	0	0	0	0	0	0
	2	0	0	0	0	0	0	0	0	0	0	0	0	0	0	0	0	0	0	0	0	0	0	0	0	0	0	0	0	0	0	0	0
	3	0	0	0	0	0	0	0	0	0	0	0	0	0	0	0	0	0	0	0	0	0	0	0	0	0	0	0	0	0	0	0	0
	4	0	1	0	0	0	0	0	0	0	0	0	0	0	0	0	0	0	0	0	0	0	0	0	0	0	0	0	0	0	0	0	1

22-AUG-13

88R002 TABLE : IA- 004

PR.NO.60R0375/88R002: REPRODUCTIVE TOX. STUDY TO DETECT EFFECTS
OF MIXED ANTI-ANDROGENIC SUBSTANCES IN RATS; ORAL ADM. (GAVAGE)
SUMMARY OF MATERNAL/PUP CLINICAL OBSERVATIONS DURING LACTATION

	GROUP#	DAY OF LACTATION 31	32	33	34	35	36	37	38	39	40	41	42	43	44	45	46	47	48	49	50	51	52	TOTAL
# OF FEMALES EXAMINED	0	20	20	20	20	20	20	20	20	20	20	20	20	20	20	20	20	20	17	10	7	2	0	
	1	20	20	19	19	19	19	19	19	19	19	19	19	19	19	19	19	19	16	11	8	4	1	
	2	20	20	20	20	20	20	20	20	20	20	20	20	20	20	20	20	19	16	8	6	1	0	
	3	19	19	19	19	19	19	19	19	19	19	19	19	19	19	19	19	14	10	6	2	0	0	
	4	19	19	19	19	19	19	19	19	19	19	19	19	19	19	19	19	19	15	10	4	2	0	
NORMAL																								
NOTHING ABNORMAL DETECTED	0	20	20	20	20	20	20	20	20	20	20	20	20	20	20	20	20	20	17	10	7	2	-	20
	1	20	19	19	19	19	19	19	19	19	19	19	19	19	19	19	19	19	16	11	8	4	1	20
	2	18	18	20	20	19	20	19	20	19	20	20	20	20	20	20	19	19	16	8	6	1	-	20
	3	0	0	0	0	0	0	0	0	0	0	0	0	0	0	0	5	4	4	4	2	-	-	19
	4	19	19	19	19	19	19	19	19	19	19	19	19	19	19	19	19	19	15	10	4	2	-	19
DEAD																								
SCHEDULED SACRIFICE	0	0	0	0	0	0	0	0	0	0	0	0	0	0	0	0	0	3	7	3	5	2	-	20
	1	0	0	0	0	0	0	0	0	0	0	0	0	0	0	0	0	3	5	3	4	3	1	19
	2	0	0	0	0	0	0	0	0	0	0	0	0	0	0	0	1	3	8	2	5	1	-	20
	3	0	0	0	0	0	0	0	0	0	0	0	0	0	0	0	5	4	4	4	2	-	-	19
	4	0	0	0	0	0	0	0	0	0	0	0	0	0	0	0	0	4	5	6	2	2	-	19
FOUND DEAD	0	0	0	0	0	0	0	0	0	0	0	0	0	0	0	0	0	0	0	0	0	0	-	0
	1	0	1	0	0	0	0	0	0	0	0	0	0	0	0	0	0	0	0	0	0	0	0	1
	2	0	0	0	0	0	0	0	0	0	0	0	0	0	0	0	0	0	0	0	0	0	-	0
	3	0	0	0	0	0	0	0	0	0	0	0	0	0	0	0	0	0	0	0	0	-	-	0
	4	0	0	0	0	0	0	0	0	0	0	0	0	0	0	0	0	0	0	0	0	0	-	0
ORAL-BUCCAL																								
SALIVATION AFTER TREATMENT	0	0	0	0	0	0	0	0	0	0	0	0	0	0	0	0	0	0	0	0	0	0	-	0
	1	0	0	0	0	0	0	0	0	0	0	0	0	0	0	0	0	0	0	0	0	0	0	0
	2	2	2	0	0	1	0	1	0	1	0	0	0	0	0	0	1	0	0	0	0	0	-	2
	3	19	19	19	19	19	19	19	19	19	19	19	19	19	19	19	14	10	6	2	0	-	-	19
	4	0	0	0	0	0	0	0	0	0	0	0	0	0	0	0	0	0	0	0	0	0	-	0

PR.NO.60R0375/88R002: REPRODUCTIVE TOX. STUDY TO DETECT EFFECTS
OF MIXED ANTI-ANDROGENIC SUBSTANCES IN RATS; ORAL ADM. (GAVAGE)
MEAN MATERNAL FOOD CONSUMPTION DURING GESTATION -- GRAMS/ANIMAL/DAY

		TEST GROUP 0 0 MG/KG BW/D	TEST GROUP 1 ADI-MIX	TEST GROUP 2 NOAEL-MIX	TEST GROUP 3 LOAEL-MIX	TEST GROUP 4 0.00025 MG/KG BW/D
DAYS 0 TO 6	MEAN	16.1 D	16.0	15.9	16.7	16.7
	S.D.	1.57	1.49	1.67	1.27	1.46
	N	25	25	25	25	24
DAYS 6 TO 13	MEAN	17.1 D	17.1	17.1	17.1	17.9
	S.D.	2.06	1.58	2.00	1.25	1.66
	N	25	25	25	25	24
DAYS 13 TO 20	MEAN	19.2 D	19.4	18.6	18.5	20.0
	S.D.	2.32	1.60	2.00	1.44	2.14
	N	25	25	25	25	24
DAYS 6 TO 20	MEAN OF MEANS	18.1	18.3	17.8	17.8	19.0
	S.D.	1.47	1.63	1.05	1.05	1.43
	N	2	2	2	2	2
DAYS 0 TO 20	MEAN OF MEANS	17.4	17.5	17.2	17.4	18.2
	S.D.	1.56	1.74	1.31	0.97	1.63
	N	3	3	3	3	3

Statistics: D=Dunnett-test (two-sided)
* : $p<=0.05$ ** : $p<=0.01$

PR.NO.60R0375/88R002: REPRODUCTIVE TOX. STUDY TO DETECT EFFECTS
OF MIXED ANTI-ANDROGENIC SUBSTANCES IN RATS; ORAL ADM. (GAVAGE)
MEAN MATERNAL FOOD CONSUMPTION DURING LACTATION -- GRAMS/ANIMAL/DAY

		TEST GROUP 0<br>0 MG/KG BW/D	TEST GROUP 1<br>ADI-MIX	TEST GROUP 2<br>NOAEL-MIX	TEST GROUP 3<br>LOAEL-MIX	TEST GROUP 4<br>0.00025 MG/KG<br>BW/D
DAYS 0 TO 7	MEAN	30.6 D	31.2	29.1	28.4	30.3
	S.D.	3.73	3.74	4.80	7.35	5.94
	N	20	20	20	19	19
DAYS 7 TO 14	MEAN	48.9 D	50.5	47.4	45.5	48.6
	S.D.	5.44	5.05	7.89	13.30	10.27
	N	20	20	20	19	19
DAYS 14 TO 21	MEAN	59.9 D	61.4	59.0	57.5	60.0
	S.D.	5.35	6.38	9.39	17.26	12.32
	N	20	20	20	19	19
DAYS 0 TO 21	MEAN OF MEANS	46.5	47.7	45.2	43.8	46.3
	S.D.	14.80	15.34	15.05	14.60	14.95
	N	3	3	3	3	3

Statistics: D=Dunnett-test (two-sided)
* : p<=0.05 ** : p<=0.01

PR.NO.60R0375/88R002: REPRODUCTIVE TOX. STUDY TO DETECT EFFECTS
OF MIXED ANTI-ANDROGENIC SUBSTANCES IN RATS; ORAL ADM. (GAVAGE)
MEAN MATERNAL BODY WEIGHTS DURING GESTATION -- GRAMS

		TEST GROUP 0<br>0 MG/KG BW/D	TEST GROUP 1<br>ADI-MIX	TEST GROUP 2<br>NOAEL-MIX	TEST GROUP 3<br>LOAEL-MIX	TEST GROUP 4<br>0.00025 MG/KG BW/D
DAY 0	MEAN	168.8 D	170.6	169.4	168.8	170.1
	S.D.	10.55	13.49	12.40	12.41	10.79
	N	25	25	25	25	24
DAY 6	MEAN	197.7 D	199.5	197.6	199.3	200.5
	S.D.	13.70	15.47	14.85	12.38	12.68
	N	25	25	25	25	24
DAY 13	MEAN	228.8 D	231.9	227.7	229.3	233.1
	S.D.	18.04	17.37	16.80	12.98	15.94
	N	25	25	25	25	24
DAY 20	MEAN	288.9 D	295.6	288.0	293.5	295.0
	S.D.	24.60	21.79	22.71	20.19	23.02
	N	25	25	25	25	24

Statistics: D=Dunnett-test (two-sided)
* : $p<=0.05$ ** : $p<=0.01$

PR.NO.60R0375/88R002: REPRODUCTIVE TOX. STUDY TO DETECT EFFECTS OF MIXED ANTI-ANDROGENIC SUBSTANCES IN RATS; ORAL ADM. (GAVAGE)
MEAN MATERNAL BODY WEIGHT CHANGE DURING GESTATION -- GRAMS

		TEST GROUP 0 0 MG/KG BW/D	TEST GROUP 1 ADI-MIX	TEST GROUP 2 NOAEL-MIX	TEST GROUP 3 LOAEL-MIX	TEST GROUP 4 0.00025 MG/KG BW/D
DAYS 0 TO 6	MEAN	28.9 D	28.9	28.3	30.5	30.4
	S.D.	5.71	5.91	5.77	4.01	4.52
	N	25	25	25	25	24
DAYS 6 TO 13	MEAN	31.0 D	32.4	30.0	30.0	32.6
	S.D.	5.77	5.38	4.94	4.01	8.29
	N	25	25	25	25	24
DAYS 13 TO 20	MEAN	60.1 D	63.7	60.3	64.1	62.0
	S.D.	8.70	7.96	10.86	9.13	11.75
	N	25	25	25	25	24
DAYS 6 TO 20	MEAN	91.1 D	96.1	90.3	94.2	94.5
	S.D.	12.97	10.80	14.15	11.44	15.98
	N	25	25	25	25	24
DAYS 0 TO 20	MEAN	120.0 D	125.0	118.6	124.7	124.9
	S.D.	16.94	15.09	17.00	12.74	19.14
	N	25	25	25	25	24

Statistics: D=Dunnett-test (two-sided)
* : p<=0.05 ** : p<=0.01

PR.NO.60R0375/88R002: REPRODUCTIVE TOX. STUDY TO DETECT EFFECTS
OF MIXED ANTI-ANDROGENIC SUBSTANCES IN RATS; ORAL ADM. (GAVAGE)
MEAN MATERNAL BODY WEIGHTS DURING LACTATION -- GRAMS

		TEST GROUP 0 0 MG/KG BW/D	TEST GROUP 1 ADI-MIX	TEST GROUP 2 NOAEL-MIX	TEST GROUP 3 LOAEL-MIX	TEST GROUP 4 0.00025 MG/KG BW/D
DAY 0	MEAN	224.9 D	233.3	223.5	223.1	232.8
	S.D.	18.29	18.14	21.80	15.78	16.28
	N	20	20	20	19	19
DAY 7	MEAN	246.9 D	254.6	244.4	239.3	249.4
	S.D.	18.92	17.77	17.93	17.06	17.34
	N	20	20	20	19	19
DAY 14	MEAN	255.9 D	268.7	258.7	254.7	261.2
	S.D.	16.00	16.79	18.68	18.53	18.39
	N	20	20	20	19	19
DAY 21	MEAN	251.7 D	257.9	253.4	254.2	255.5
	S.D.	16.64	11.70	14.68	16.48	20.26
	N	20	20	20	19	19
DAY 28	MEAN	219.3 D	230.4	223.1	228.4	230.2
	S.D.	13.79	14.54	13.58	13.46	14.83
	N	20	20	20	19	19
DAY 35	MEAN	229.3 D	237.8	231.4	236.1	238.5
	S.D.	16.27	15.31	14.32	12.31	17.80
	N	20	19	20	19	19
DAY 42	MEAN	234.0 D	241.4	236.3	240.1	241.9
	S.D.	15.88	16.12	16.82	13.60	19.11
	N	20	19	20	19	19

Statistics: D=Dunnett-test (two-sided)
* : p<=0.05 ** : p<=0.01

PR.NO.60R0375/88R002: REPRODUCTIVE TOX. STUDY TO DETECT EFFECTS
OF MIXED ANTI-ANDROGENIC SUBSTANCES IN RATS; ORAL ADM. (GAVAGE)
MEAN MATERNAL BODY WEIGHT CHANGE DURING LACTATION -- GRAMS

		TEST GROUP 0 0 MG/KG BW/D	TEST GROUP 1 ADI-MIX	TEST GROUP 2 NOAEL-MIX	TEST GROUP 3 LOAEL-MIX	TEST GROUP 4 0.00025 MG/KG BW/D
DAYS 0 TO 7	MEAN	22.0 D	21.3	20.9	16.1	16.6
	S.D.	9.07	5.67	9.61	14.09	10.00
	N	20	20	20	19	19
DAYS 7 TO 14	MEAN	8.9 D	14.1	14.3	15.4	11.8
	S.D.	9.41	8.17	7.92	9.45	10.84
	N	20	20	20	19	19
DAYS 14 TO 21	MEAN	-4.2 D	-10.8	-5.3	-0.5	-5.7
	S.D.	8.42	10.77	9.64	11.15	8.98
	N	20	20	20	19	19
DAYS 21 TO 28	MEAN	-32.4 D	-27.5	-30.3	-25.8	-25.2
	S.D.	9.05	11.00	9.39	14.85	13.43
	N	20	20	20	19	19
DAYS 28 TO 35	MEAN	10.0 D	7.9	8.4	7.7	8.3
	S.D.	6.63	4.51	5.73	5.67	4.94
	N	20	19	20	19	19
DAYS 35 TO 42	MEAN	4.7 D	3.5	4.9	3.9	3.3
	S.D.	3.67	4.97	5.58	5.78	5.21
	N	20	19	20	19	19
DAYS 0 TO 42	MEAN	9.1 D	8.6	12.8	16.9	9.1
	S.D.	10.92	9.58	10.34	10.13	10.70
	N	20	19	20	19	19

Statistics: D=Dunnett-test (two-sided)
* : p<=0.05 ** : p<=0.01

PR.NO.60R0375/88R002: REPRODUCTIVE TOX. STUDY TO DETECT EFFECTS
OF MIXED ANTI-ANDROGENIC SUBSTANCES IN RATS; ORAL ADM. (GAVAGE)
SUMMARY OF MATERNAL NECROPSY OBSERVATIONS

		TEST GROUP 0<br>0 MG/KG BW/D	TEST GROUP 1<br>ADI-MIX	TEST GROUP 2<br>NOAEL-MIX	TEST GROUP 3<br>LOAEL-MIX	TEST GROUP 4<br>0.00025 MG/KG<br>BW/D
FEMALES EXAMINED	N	5	5	5	5	6
NOTHING ABNORMAL DETECTED	N	5	5	5	5	6
	%	100	100	100	100	100

PR.NO.60R0375/88R002: REPRODUCTIVE TOX. STUDY TO DETECT EFFECTS
OF MIXED ANTI-ANDROGENIC SUBSTANCES IN RATS; ORAL ADM. (GAVAGE)
SUMMARY OF REPRODUCTION DATA

		TEST GROUP 0<br>0 MG/KG BW/D	TEST GROUP 1<br>ADI-MIX	TEST GROUP 2<br>NOAEL-MIX	TEST GROUP 3<br>LOAEL-MIX	TEST GROUP 4<br>0.00025 MG/KG<br>BW/D
Females Mated	N	25	25	25	25	25
Pregnant	N	25	25	25	25	24
Conception Rate	%	100	100	100	100	96
Aborted	N	0	0	0	0	0
Premature Births	N	0	0	0	0	0
Dams with Viable Fetuses	N	5	5	5	5	5
Dams with all Resorptions	N	0	0	0	0	0
Female Mortality	N	0Fi	0	0	1	0
	%	0.0	0.0	0.0	4.0	0.0
Pregnant at Terminal Sacrifice	N	5Fi	5	5	5	5
	%	20	20	20	20	20
Corpora Lutea	MEAN	10.6 D	12.6	12.0	11.6	12.0
	S.D.	1.82	2.07	1.87	1.14	1.87
	TOTAL	53	63	60	58	60
Implantation Sites	MEAN	9.8 D	11.4	9.6	10.8	11.8
	S.D.	2.59	1.82	5.18	1.79	1.64
	TOTAL	49	57	48	54	59
Preimplantation Loss	MEAN%	8.8 D	9.1	20.0	7.0	1.4
	S.D.	10.23	9.63	40.23	10.95	3.19
Postimplantation Loss	MEAN%	7.1 D	3.5	7.5	1.7	4.6
	S.D.	7.22	4.91	10.69	3.73	6.88

Statistics: D=Dunnett-test (two-sided) Fi =Fisher's exact test (one-sided)
* : p<=0.05 ** : p<=0.01

PR.NO.60R0375/88R002: REPRODUCTIVE TOX. STUDY TO DETECT EFFECTS
OF MIXED ANTI-ANDROGENIC SUBSTANCES IN RATS; ORAL ADM. (GAVAGE)
SUMMARY OF REPRODUCTION DATA

		TEST GROUP 0 0 MG/KG BW/D	TEST GROUP 1 ADI-MIX	TEST GROUP 2 NOAEL-MIX	TEST GROUP 3 LOAEL-MIX	TEST GROUP 4 0.00025 MG/KG BW/D
Pregnant at Terminal Sacrifice	N	5	5	5	5	5
Resorptions: Total	MEAN	0.6 D	0.4	1.0	0.2	0.6
	S.D.	0.55	0.55	1.41	0.45	0.89
	TOTAL	3	2	5	1	3
	MEAN%	7.1 D	3.5	7.5	1.7	4.6
	S.D.	7.22	4.91	10.69	3.73	6.88
Early	MEAN	0.4 D	0.4	0.8	0.2	0.6
	S.D.	0.55	0.55	1.10	0.45	0.89
	TOTAL	2	2	4	1	3
	MEAN%	3.8 D	3.5	5.9	1.7	4.6
	S.D.	5.29	4.91	8.13	3.73	6.88
Late	MEAN	0.2 D	0.0	0.2	0.0	0.0
	S.D.	0.45	0.00	0.45	0.00	0.00
	TOTAL	1	0	1	0	0
	MEAN%	3.3 D	0.0	1.5	0.0	0.0
	S.D.	7.45	0.00	3.44	0.00	0.00
Dead Fetuses	N	0	0	0	0	0

Statistics: D=Dunnett-test (two-sided)
* : p<=0.05 ** : p<=0.01

PR.NO.60R0375/88R002: REPRODUCTIVE TOX. STUDY TO DETECT EFFECTS
OF MIXED ANTI-ANDROGENIC SUBSTANCES IN RATS; ORAL ADM. (GAVAGE)
SUMMARY OF REPRODUCTION DATA

		TEST GROUP 0 0 MG/KG BW/D	TEST GROUP 1 ADI-MIX	TEST GROUP 2 NOAEL-MIX	TEST GROUP 3 LOAEL-MIX	TEST GROUP 4 0.00025 MG/KG BW/D
Dams with Viable Fetuses	N	5	5	5	5	5
Live Fetuses	MEAN	9.2 D	11.0	8.6	10.6	11.2
	S.D.	2.77	1.87	4.39	1.67	1.30
	TOTAL	46	55	43	53	56
	MEAN%	92.9 D	96.5	92.5	98.3	95.4
	S.D.	7.22	4.91	10.69	3.73	6.88
Females	MEAN	4.4 D	4.4	5.4	5.4	6.2
	S.D.	1.52	1.52	2.70	1.14	1.92
	TOTAL	22	22	27	27	31
	MEAN%	43.9 D	38.7	64.5	50.3	52.3
	S.D.	6.26	13.40	20.81	9.10	13.14
Males	MEAN	4.8 D	6.6	3.2	5.2	5.0
	S.D.	1.30	1.52	1.79	1.30	1.00
	TOTAL	24	33	16	26	25
	MEAN%	49.0 D	57.8	28.0*	48.0	43.1
	S.D.	3.92	10.45	16.83	7.80	10.93
PER CENT LIVE FEMALES		47.8	40.0	62.8	50.9	55.4
PER CENT LIVE MALES		52.2	60.0	37.2	49.1	44.6

Statistics: D=Dunnett-test (two-sided)
* : p<=0.05 ** : p<=0.01

PR.NO.60R0375/88R002: REPRODUCTIVE TOX. STUDY TO DETECT EFFECTS
OF MIXED ANTI-ANDROGENIC SUBSTANCES IN RATS; ORAL ADM. (GAVAGE)
MEAN FETAL BODY WEIGHTS (ON A LITTER BASIS)

		TEST GROUP 0<br>0 MG/KG BW/D	TEST GROUP 1<br>ADI-MIX	TEST GROUP 2<br>NOAEL-MIX	TEST GROUP 3<br>LOAEL-MIX	TEST GROUP 4<br>0.00025 MG/KG BW/D
FETAL WEIGHTS UNITS: GRAMS						
of all Viable Fetuses	MEAN	3.6 D	3.4*	3.6	3.7	3.5
	S.D.	0.15	0.13	0.11	0.17	0.07
	N	5	5	5	5	5
of Male Fetuses	MEAN	3.7 D	3.4*	3.7	3.8	3.6
	S.D.	0.12	0.17	0.12	0.09	0.08
	N	5	5	4	5	5
of Female Fetuses	MEAN	3.5 D	3.3	3.5	3.6	3.4
	S.D.	0.18	0.15	0.12	0.22	0.03
	N	5	5	5	5	5

Statistics: D=Dunnett-test (two-sided)
* : p<=0.05 ** : p<=0.01

PR.NO.60R0375/88R002: REPRODUCTIVE TOX. STUDY TO DETECT EFFECTS
OF MIXED ANTI-ANDROGENIC SUBSTANCES IN RATS; ORAL ADM. (GAVAGE)
SUMMARY OF FETAL EXTERNAL OBSERVATIONS

		TEST GROUP 0 0 MG/KG BW/D	TEST GROUP 1 ADI-MIX	TEST GROUP 2 NOAEL-MIX	TEST GROUP 3 LOAEL-MIX	TEST GROUP 4 0.00025 MG/KG BW/D
Litters Evaluated	N	5	5	5	5	5
Fetuses Evaluated	N	46	55	43	53	56
Live	N	46	55	43	53	56
Dead	N	0	0	0	0	0
M HEAD MISSHAPEN						
Fetal Incidence	N	0	0	1	0	0
	%	0.0	0.0	2.3	0.0	0.0
Litter Incidence	N	0Fi	0	1	0	0
	%	0.0	0.0	20	0.0	0.0
Affected Fetuses/Litter	MEAN%	0.0Wi	0.0	20.0	0.0	0.0
	S.D.	0.00	0.00	44.72	0.00	0.00
V LIMB HYPEREXTENSION						
Fetal Incidence	N	0	0	1	0	0
	%	0.0	0.0	2.3	0.0	0.0
Litter Incidence	N	0Fi	0	1	0	0
	%	0.0	0.0	20	0.0	0.0
Affected Fetuses/Litter	MEAN%	0.0Wi	0.0	20.0	0.0	0.0
	S.D.	0.00	0.00	44.72	0.00	0.00
TOTAL FETAL EXTERNAL OBSERVATIONS						
Fetal Incidence	N	0	0	1	0	0
	%	0.0	0.0	2.3	0.0	0.0
Litter Incidence	N	0Fi	0	1	0	0
	%	0.0	0.0	20	0.0	0.0
Affected Fetuses/Litter	MEAN%	0.0Wi	0.0	20.0	0.0	0.0
	S.D.	0.00	0.00	44.72	0.00	0.00

Statistics: Fi =Fisher's exact test (one-sided) Wi =Wilcoxon-test (one-sided)
* : p<=0.05 ** : p<=0.01
OBSERVATION CODES: M=Malformation V=Variation

PR.NO.60R0375/88R002: REPRODUCTIVE TOX. STUDY TO DETECT EFFECTS
OF MIXED ANTI-ANDROGENIC SUBSTANCES IN RATS; ORAL ADM. (GAVAGE)
SUMMARY OF FEMALE REPRODUCTION AND DELIVERY DATA

		TEST GROUP 0 0 MG/KG BW/D	TEST GROUP 1 ADI-MIX	TEST GROUP 2 NOAEL-MIX	TEST GROUP 3 LOAEL-MIX	TEST GROUP 4 0.00025 MG/KG BW/D
Females on Study	N	20	20	20	20	20
Females Mated	N	20Fi	20	20	20	20
Female Mating Index	%	100	100	100	100	100
Females Pregnant	N	20Fi	20	20	20	19
Female Fertility Index	%	100	100	100	100	95
Duration of Gestation (Days)	MEAN	22.0 D	21.8	22.3*	22.9**	22.1
	S.D.	0.22	0.44	0.47	0.40	0.52
Implantation sites	TOTAL	199	211	211	193	184
	MEAN	9.9 D	10.6	10.6	10.2	9.7
	S.D.	1.47	1.19	1.54	1.61	2.52
	N	20	20	20	19	19
Postimplantation Loss	TOTAL	6	12	9	14	7
	MEAN	0.3 D	0.6	0.4	0.7	0.4
	S.D.	0.47	1.19	0.76	0.87	0.50
	N	20	20	20	19	19
% Postimplantation Loss	MEAN	3.0 D	5.7	4.5	7.2	3.4
	S.D.	4.74	10.95	7.76	8.59	4.61
	N	20	20	20	19	19
Females with Liveborn	N	20Fi	20	20	17	19
Gestation Index	%	100	100	100	85	100
with Stillborn Pups	N	1Fi	1	2	8**	2
	%	5.0	5.0	10	42	11
with all Stillborn	N	0Fi	0	0	2	0
	%	0.0	0.0	0.0	11	0.0
Pups Delivered	MEAN	9.6 D	9.9	10.1	9.4	9.3
	S.D.	1.42	1.67	1.80	1.71	2.38
	TOTAL	193	199	202	179	177
Liveborn	N	192Fi	198	200	153**	172
Live Birth Index	%	99	99	99	85	97
Stillborn	N	1Fi	1	2	26**	5
	%	0.5	0.5	1.0	15	2.8

Statistics: D=Dunnett-test (two-sided) Fi =Fisher's exact test (one-sided)
* : p<=0.05 ** : p<=0.01
THE INDICES ARE DEFINED IN THE TEXT

PR.NO.60R0375/88R002: REPRODUCTIVE TOX. STUDY TO DETECT EFFECTS
OF MIXED ANTI-ANDROGENIC SUBSTANCES IN RATS; ORAL ADM. (GAVAGE)
SUMMARY OF LITTER DATA

		TEST GROUP 0 0 MG/KG BW/D	TEST GROUP 1 ADI-MIX	TEST GROUP 2 NOAEL-MIX	TEST GROUP 3 LOAEL-MIX	TEST GROUP 4 0.00025 MG/KG BW/D
(Total Number of) Litters	N	20	20	20	19	19
Litters with Liveborn Pups	N	20Fi	20	20	17	19
	%	100	100	100	89	100
Litters with Stillborn Pups	N	1Fi	1	2	8**	2
	%	5.0	5.0	10	42	11
Litters with all Stillborn Pups	N	0Fi	0	0	2	0
	%	0.0	0.0	0.0	11	0.0
Pups Delivered	TOTAL	193	199	202	179	177
	MEAN	9.6 D	9.9	10.1	9.4	9.3
	S.D.	1.42	1.67	1.80	1.71	2.38
Pups Liveborn	N	192Fi	198	200	153**	172
	%	99	99	99	85	97
Pups Stillborn	N	1Fi	1	2	26**	5
	%	0.5	0.5	1.0	15	2.8
Pups Died	N	0Fi	0	7**	0	1
	%	0.0	0.0	3.5	0.0	0.6
Pups Sacrificed Moribund	N	0Fi	0	0	0	0
	%	0.0	0.0	0.0	0.0	0.0
Pups Cannibalized	N	0Fi	0	6*	3	1
	%	0.0	0.0	3.0	1.7	0.6
Pups Accidental Death	N	0	0	0	0	0
	%	0.0	0.0	0.0	0.0	0.0
Pups Sacrificed, Maternal Death	N	0	0	0	0	0
	%	0.0	0.0	0.0	0.0	0.0

Statistics: D=Dunnett-test (two-sided) Fi =Fisher's exact test (one-sided)
* : p<=0.05 ** : p<=0.01

PR.NO.60R0375/88R002: REPRODUCTIVE TOX. STUDY TO DETECT EFFECTS
OF MIXED ANTI-ANDROGENIC SUBSTANCES IN RATS; ORAL ADM. (GAVAGE)
SUMMARY OF LITTER DATA

		TEST GROUP 0 0 MG/KG BW/D	TEST GROUP 1 ADI-MIX	TEST GROUP 2 NOAEL-MIX	TEST GROUP 3 LOAEL-MIX	TEST GROUP 4 0.00025 MG/KG BW/D
Pups dead day 0	N	0	0	0	0	0
	%	0.0	0.0	0.0	0.0	0.0
days 1 to 4	N	0	0	13	2	2
	%	0.0	0.0	6.5	1.3	1.2
days 5 to 7	N	0	0	0	0	0
	%	0.0	0.0	0.0	0.0	0.0
days 8 to 14	N	0	0	0	1	0
	%	0.0	0.0	0.0	0.7	0.0
days 15 to 21	N	0	0	0	0	0
	%	0.0	0.0	0.0	0.0	0.0
Pups Surviving days 0 to 4	N	192Fi	198	187**	151	170
Viability Index	%	100	100	94	99	99
Pups Surviving days 4 to 21	N	192Fi	198	187	150	170
Lactation Index	%	100	100	100	99	100

Statistics: Fi =Fisher's exact test (one-sided)
* : p<=0.05 ** : p<=0.01
THE INDICES ARE DEFINED IN THE TEXT. Pups Dead = Pups Died + Sacrificed Moribund + Cannibalized

PR.NO.60R0375/88R002: REPRODUCTIVE TOX. STUDY TO DETECT EFFECTS
OF MIXED ANTI-ANDROGENIC SUBSTANCES IN RATS; ORAL ADM. (GAVAGE)
SUMMARY OF LITTER DATA

		TEST GROUP 0 0 MG/KG BW/D	TEST GROUP 1 ADI-MIX	TEST GROUP 2 NOAEL-MIX	TEST GROUP 3 LOAEL-MIX	TEST GROUP 4 0.00025 MG/KG BW/D
Live Pups/Litter						
day 1	MEAN	9.6	9.9	9.5	7.9	8.9
	S.D.	1.43	1.68	1.96	3.29	2.76
	TOTAL	192	198	190	151	170
day 4	MEAN	9.6	9.9	9.4	7.9	8.9
	S.D.	1.43	1.68	2.39	3.29	2.76
	TOTAL	192	198	187	151	170
day 7	MEAN	9.6	9.9	9.4	7.9	8.9
	S.D.	1.43	1.68	2.39	3.29	2.76
	TOTAL	192	198	187	151	170
day 14	MEAN	9.6	9.9	9.4	7.9	8.9
	S.D.	1.43	1.68	2.39	3.35	2.76
	TOTAL	192	198	187	150	170
day 21	MEAN	9.6	9.9	9.4	7.9	8.9
	S.D.	1.43	1.68	2.39	3.35	2.76
	TOTAL	192	198	187	150	170
Sex Ratio						
day 0						
- live Males	%	46.9	42.9	51.0	53.6	48.3
- live Females	%	53.1	57.1	49.0	46.4	51.7
day 21						
- live Males	%	46.9	42.9	51.9	54.0	48.8
- live Females	%	53.1	57.1	48.1	46.0	51.2
SELECTED AS PARENT/RAISED						
males	N	30	30	30	30	25
females	N	30	30	30	30	25

PR.NO.60R0375/88R002: REPRODUCTIVE TOX. STUDY TO DETECT EFFECTS
OF MIXED ANTI-ANDROGENIC SUBSTANCES IN RATS; ORAL ADM. (GAVAGE)
SUMMARY - PRESENCE OF AREOLAS/NIPPLES

		TEST GROUP 0 0 MG/KG BW/D	TEST GROUP 1 ADI-MIX	TEST GROUP 2 NOAEL-MIX	TEST GROUP 3 LOAEL-MIX	TEST GROUP 4 0.00025 MG/KG BW/D
AREOLAS/NIPPLES						
Litters tested	N	20	18	20	17	18
Pups tested	N	90	85	97	81	83
Pups reaching criteria	N	63	56	68	81	46
	%	70	66	70	100	55
Pups reaching criteria/litter	MEAN%	71.0Wi	66.3	66.9	100.0**	54.4
	S.D.	22.09	24.59	27.38	0.00	36.00

Statistics: Wi =Wilcoxon-test (one-sided) with Bonferroni-Holm-adjustment per substance
* : p<=0.05 ** : p<=0.01

PR.NO.60R0375/88R002: REPRODUCTIVE TOX. STUDY TO DETECT EFFECTS
OF MIXED ANTI-ANDROGENIC SUBSTANCES IN RATS; ORAL ADM. (GAVAGE)
SUMMARY - PRESENCE OF AREOLAS/NIPPLES

		TEST GROUP 0<br>0 MG/KG BW/D	TEST GROUP 1<br>ADI-MIX	TEST GROUP 2<br>NOAEL-MIX	TEST GROUP 3<br>LOAEL-MIX	TEST GROUP 4<br>0.00025 MG/KG<br>BW/D
AREOLAS/NIPPLES - SECOND OBS						
Litters tested	N	20	18	20	17	18
Pups tested	N	90	85	97	81	83
Pups reaching criteria	N	0	0	0	40	0
	%	0.0	0.0	0.0	49	0.0
Pups reaching criteria/litter	MEAN%	0.0Wi	0.0	0.0	51.5**	0.0
	S.D.	0.00	0.00	0.00	30.98	0.00

Statistics: Wi =Wilcoxon-test (one-sided) with Bonferroni-Holm-adjustment per substance
* : p<=0.05 ** : p<=0.01

PR.NO.60R0375/88R002: REPRODUCTIVE TOX. STUDY TO DETECT EFFECTS
OF MIXED ANTI-ANDROGENIC SUBSTANCES IN RATS; ORAL ADM. (GAVAGE)
SUMMARY OF PUP ANOGENITAL DISTANCE

		TEST GROUP 0 0 MG/KG BW/D	TEST GROUP 1 ADI-MIX	TEST GROUP 2 NOAEL-MIX	TEST GROUP 3 LOAEL-MIX	TEST GROUP 4 0.00025 MG/KG BW/D
AG DIST DAY 1 UNITS: MM						
of all Pups	MEAN	2.18 D	2.06	2.30	2.33	2.25
	S.D.	0.234	0.358	0.293	0.297	0.345
	N	20	20	20	17	18
of Male Pups	MEAN	3.04 D	2.97	3.06	2.94	3.06
	S.D.	0.108	0.164	0.181	0.232	0.214
	N	20	18	20	17	18
of Female Pups	MEAN	1.43 D	1.38	1.47	1.64**	1.46
	S.D.	0.087	0.118	0.121	0.140	0.165
	N	20	20	20	17	18

Statistics: D=Dunnett-test (two-sided)
* : p<=0.05 ** : p<=0.01

PR.NO.60R0375/88R002: REPRODUCTIVE TOX. STUDY TO DETECT EFFECTS
OF MIXED ANTI-ANDROGENIC SUBSTANCES IN RATS; ORAL ADM. (GAVAGE)
SUMMARY OF PUP ANOGENITAL INDEX

		TEST GROUP 0<br>0 MG/KG BW/D	TEST GROUP 1<br>ADI-MIX	TEST GROUP 2<br>NOAEL-MIX	TEST GROUP 3<br>LOAEL-MIX	TEST GROUP 4<br>0.00025 MG/KG<br>BW/D
AG DIST DAY 1 to BODY WEIGHT RATIO (AG Index)						
of all Pups	MEAN	0.31 D	0.30	0.33	0.31	0.31
	S.D.	0.032	0.056	0.043	0.035	0.044
	N	20	20	20	17	18
of Male Pups	MEAN	0.43 D	0.42	0.43	0.39**	0.42
	S.D.	0.020	0.041	0.055	0.027	0.040
	N	20	18	20	17	18
of Female Pups	MEAN	0.21 D	0.21	0.22	0.23	0.21
	S.D.	0.019	0.019	0.029	0.029	0.020
	N	20	20	20	17	18

Statistics: D=Dunnett-test (two-sided)
* : p<=0.05 ** : p<=0.01

PR.NO.60R0375/88R002: REPRODUCTIVE TOX. STUDY TO DETECT EFFECTS
OF MIXED ANTI-ANDROGENIC SUBSTANCES IN RATS; ORAL ADM. (GAVAGE)
SUMMARY OF PUP BODY WEIGHTS -- GRAMS

		TEST GROUP 0 0 MG/KG BW/D	TEST GROUP 1 ADI-MIX	TEST GROUP 2 NOAEL-MIX	TEST GROUP 3 LOAEL-MIX	TEST GROUP 4 0.00025 MG/KG BW/D
day 1 males	MEAN	7.1 D	7.1	7.2	7.7*	7.4
	S.D.	0.32	0.76	0.76	0.54	0.84
	N	20	18	20	17	18
day 1 females	MEAN	6.8 D	6.7	6.8	7.4*	7.1
	S.D.	0.37	0.69	0.68	0.65	0.80
	N	20	20	20	17	18
day 1 males+females	MEAN	6.9 D	6.8	7.0	7.5*	7.3
	S.D.	0.34	0.70	0.72	0.49	0.84
	N	20	20	20	17	18
day 4 males	MEAN	11.1 D	11.0	11.1	11.7	11.5
	S.D.	0.59	1.44	1.33	1.09	1.38
	N	20	18	20	17	18
day 4 females	MEAN	10.8 D	10.6	10.5	11.4	11.1
	S.D.	0.74	1.33	1.56	1.02	1.33
	N	20	20	20	17	18
day 4 males+females	MEAN	10.9 D	10.7	10.8	11.5	11.3
	S.D.	0.68	1.35	1.45	0.92	1.39
	N	20	20	20	17	18
day 7 males	MEAN	16.3 D	16.2	16.0	17.0	16.9
	S.D.	1.01	2.15	2.01	1.95	2.02
	N	20	18	20	17	18
day 7 females	MEAN	15.8 D	15.6	15.2	16.4	16.4
	S.D.	1.17	1.91	2.64	1.78	1.98
	N	20	20	20	17	18
day 7 males+females	MEAN	16.0 D	15.9	15.7	16.7	16.7
	S.D.	1.10	1.95	2.30	1.59	2.02
	N	20	20	20	17	18

Statistics: D=Dunnett-test (two-sided)
* : p<=0.05 ** : p<=0.01

PR.NO.60R0375/88R002: REPRODUCTIVE TOX. STUDY TO DETECT EFFECTS
OF MIXED ANTI-ANDROGENIC SUBSTANCES IN RATS; ORAL ADM. (GAVAGE)
SUMMARY OF PUP BODY WEIGHTS -- GRAMS

			TEST GROUP 0 0 MG/KG BW/D	TEST GROUP 1 ADI-MIX	TEST GROUP 2 NOAEL-MIX	TEST GROUP 3 LOAEL-MIX	TEST GROUP 4 0.00025 MG/KG BW/D
day 14	males	MEAN	29.8 D	29.7	29.3	31.1	31.0
		S.D.	2.39	3.81	3.48	3.21	4.04
		N	20	18	20	17	18
day 14	females	MEAN	29.0 D	29.0	28.0	30.7	30.3
		S.D.	2.45	3.49	4.44	3.05	4.03
		N	20	20	20	17	18
day 14	males+females	MEAN	29.4 D	29.3	28.7	30.9	30.7
		S.D.	2.42	3.49	3.91	3.06	4.03
		N	20	20	20	17	18
day 21	males	MEAN	46.0 D	46.0	45.6	48.9	48.5
		S.D.	3.89	6.21	4.93	4.48	5.76
		N	20	18	20	17	18
day 21	females	MEAN	44.9 D	45.1	43.8	48.4	47.3
		S.D.	3.22	5.55	6.56	4.17	5.29
		N	20	20	20	17	18
day 21	males+females	MEAN	45.5 D	45.5	44.8	48.6	47.8
		S.D.	3.56	5.60	5.64	4.10	5.54
		N	20	20	20	17	18

Statistics: D=Dunnett-test (two-sided)
* : p<=0.05 ** : p<=0.01

PR.NO.60R0375/88R002: REPRODUCTIVE TOX. STUDY TO DETECT EFFECTS
OF MIXED ANTI-ANDROGENIC SUBSTANCES IN RATS; ORAL ADM. (GAVAGE)
SUMMARY OF PUP BODY WEIGHT CHANGES -- GRAMS

			TEST GROUP 0<br>0 MG/KG BW/D	TEST GROUP 1<br>ADI-MIX	TEST GROUP 2<br>NOAEL-MIX	TEST GROUP 3<br>LOAEL-MIX	TEST GROUP 4<br>0.00025 MG/KG<br>BW/D
DAYS 1 TO 4							
	males	MEAN	4.0 D	3.9	3.9	4.0	4.1
		S.D.	0.43	0.75	0.67	0.72	0.77
		N	20	18	20	17	18
	females	MEAN	4.0 D	3.9	3.7	4.0	4.0
		S.D.	0.48	0.70	0.93	0.63	0.75
		N	20	20	20	17	18
	males+females	MEAN	4.0 D	3.9	3.8	4.0	4.1
		S.D.	0.45	0.72	0.78	0.64	0.76
		N	20	20	20	17	18
DAYS 4 TO 7							
	males	MEAN	5.2 D	5.2	4.9	5.3	5.3
		S.D.	0.49	0.79	0.78	1.03	0.79
		N	20	18	20	17	18
	females	MEAN	5.0 D	5.1	4.7	5.0	5.3
		S.D.	0.64	0.67	1.15	0.97	0.79
		N	20	20	20	17	18
	males+females	MEAN	5.1 D	5.1	4.8	5.1	5.3
		S.D.	0.54	0.69	0.93	0.83	0.77
		N	20	20	20	17	18
DAYS 7 TO 14							
	males	MEAN	13.5 D	13.6	13.2	14.1	14.1
		S.D.	1.58	1.79	1.70	1.66	2.21
		N	20	18	20	17	18
	females	MEAN	13.3 D	13.4	12.8	14.3	13.9
		S.D.	1.50	1.75	1.99	2.27	2.27
		N	20	20	20	17	18
	males+females	MEAN	13.4 D	13.5	13.0	14.2	14.0
		S.D.	1.52	1.69	1.81	1.95	2.21
		N	20	20	20	17	18

Statistics: D=Dunnett-test (two-sided)
* : p<=0.05 ** : p<=0.01

PR.NO.60R0375/88R002: REPRODUCTIVE TOX. STUDY TO DETECT EFFECTS
OF MIXED ANTI-ANDROGENIC SUBSTANCES IN RATS; ORAL ADM. (GAVAGE)
SUMMARY OF PUP BODY WEIGHT CHANGES -- GRAMS

			TEST GROUP 0<br>0 MG/KG BW/D	TEST GROUP 1<br>ADI-MIX	TEST GROUP 2<br>NOAEL-MIX	TEST GROUP 3<br>LOAEL-MIX	TEST GROUP 4<br>0.00025 MG/KG<br>BW/D
DAYS 14 TO 21							
	males	MEAN	16.2 D	16.3	16.3	17.8	17.5
		S.D.	1.79	2.57	1.95	1.67	1.98
		N	20	18	20	17	18
	females	MEAN	15.9 D	16.1	15.9	17.7*	16.9
		S.D.	1.25	2.27	2.41	1.66	1.44
		N	20	20	20	17	18
	males+females	MEAN	16.1 D	16.2	16.1	17.7*	17.1
		S.D.	1.48	2.28	2.08	1.45	1.71
		N	20	20	20	17	18
DAYS 1 TO 21							
	males	MEAN	38.9 D	38.9	38.4	41.2	41.0
		S.D.	3.70	5.55	4.31	4.41	5.31
		N	20	18	20	17	18
	females	MEAN	38.1 D	38.4	37.0	41.0	40.2
		S.D.	2.98	4.98	5.97	4.22	4.87
		N	20	20	20	17	18
	males+females	MEAN	38.6 D	38.7	37.8	41.1	40.5
		S.D.	3.34	5.02	5.01	4.17	5.08
		N	20	20	20	17	18

Statistics: D=Dunnett-test (two-sided)
* : p<=0.05 ** : p<=0.01

PR.NO.60R0375/88R002: REPRODUCTIVE TOX. STUDY TO DETECT EFFECTS
OF MIXED ANTI-ANDROGENIC SUBSTANCES IN RATS; ORAL ADM. (GAVAGE)
SUMMARY OF PUP NECROPSY OBSERVATIONS

		TEST GROUP 0 0 MG/KG BW/D	TEST GROUP 1 ADI-MIX	TEST GROUP 2 NOAEL-MIX	TEST GROUP 3 LOAEL-MIX	TEST GROUP 4 0.00025 MG/KG BW/D
Litters Evaluated	N	20	20	20	19	19
Pups Evaluated	N	133	139	136	116	116
Live	N	132	138	134	90	111
Stillborn	N	1	1	2	26	5
POST MORTEM AUTOLYSIS						
Pup Incidence	N	0	0	3	10	1
	%	0.0	0.0	2.2	8.6	0.9
Litter Incidence	N	0Fi	0	1	4*	1
	%	0.0	0.0	5.0	21	5.3
Affected Pups/Litter	MEAN%	0.0Wi	0.0	1.7	8.5*	0.9
	S.D.	0.00	0.00	7.45	19.40	3.82
PARTLY CANNIBALIZED						
Pup Incidence	N	0	0	0	0	1
	%	0.0	0.0	0.0	0.0	0.9
Litter Incidence	N	0Fi	0	0	0	1
	%	0.0	0.0	0.0	0.0	5.3
Affected Pups/Litter	MEAN%	0.0Wi	0.0	0.0	0.0	0.9
	S.D.	0.00	0.00	0.00	0.00	3.82
INCISORS SLOPED						
Pup Incidence	N	1	1	0	1	0
	%	0.8	0.7	0.0	0.9	0.0
Litter Incidence	N	1Fi	1	0	1	0
	%	5.0	5.0	0.0	5.3	0.0
Affected Pups/Litter	MEAN%	0.7Wi	0.6	0.0	0.8	0.0
	S.D.	3.19	2.48	0.00	3.28	0.00

Statistics: Fi =Fisher's exact test (one-sided) Wi =Wilcoxon-test (one-sided)
* : p<=0.05 ** : p<=0.01

PR.NO.60R0375/88R002: REPRODUCTIVE TOX. STUDY TO DETECT EFFECTS
OF MIXED ANTI-ANDROGENIC SUBSTANCES IN RATS; ORAL ADM. (GAVAGE)
SUMMARY OF PUP NECROPSY OBSERVATIONS

		TEST GROUP 0 0 MG/KG BW/D	TEST GROUP 1 ADI-MIX	TEST GROUP 2 NOAEL-MIX	TEST GROUP 3 LOAEL-MIX	TEST GROUP 4 0.00025 MG/KG BW/D
Litters Evaluated	N	20	20	20	19	19
Pups Evaluated	N	133	139	136	116	116
Live	N	132	138	134	90	111
Stillborn	N	1	1	2	26	5
DIAPHRAGMATIC HERNIA						
Pup Incidence	N	0	0	1	0	0
	%	0.0	0.0	0.7	0.0	0.0
Litter Incidence	N	0Fi	0	1	0	0
	%	0.0	0.0	5.0	0.0	0.0
Affected Pups/Litter	MEAN%	0.0Wi	0.0	0.8	0.0	0.0
	S.D.	0.00	0.00	3.73	0.00	0.00
INFARCT OF LIVER						
Pup Incidence	N	0	0	0	1	0
	%	0.0	0.0	0.0	0.9	0.0
Litter Incidence	N	0Fi	0	0	1	0
	%	0.0	0.0	0.0	5.3	0.0
Affected Pups/Litter	MEAN%	0.0Wi	0.0	0.0	0.8	0.0
	S.D.	0.00	0.00	0.00	3.28	0.00
EMPTY STOMACH						
Pup Incidence	N	0	0	1	0	0
	%	0.0	0.0	0.7	0.0	0.0
Litter Incidence	N	0Fi	0	1	0	0
	%	0.0	0.0	5.0	0.0	0.0
Affected Pups/Litter	MEAN%	0.0Wi	0.0	0.6	0.0	0.0
	S.D.	0.00	0.00	2.48	0.00	0.00

Statistics: Fi =Fisher's exact test (one-sided) Wi =Wilcoxon-test (one-sided)
* : p<=0.05 ** : p<=0.01

PR.NO.60R0375/88R002: REPRODUCTIVE TOX. STUDY TO DETECT EFFECTS OF MIXED ANTI-ANDROGENIC SUBSTANCES IN RATS; ORAL ADM. (GAVAGE)

SUMMARY OF PUP NECROPSY OBSERVATIONS

		TEST GROUP 0<br>0 MG/KG BW/D	TEST GROUP 1<br>ADI-MIX	TEST GROUP 2<br>NOAEL-MIX	TEST GROUP 3<br>LOAEL-MIX	TEST GROUP 4<br>0.00025 MG/KG BW/D
Litters Evaluated	N	20	20	20	19	19
Pups Evaluated	N	133	139	136	116	116
Live	N	132	138	134	90	111
Stillborn	N	1	1	2	26	5
DILATED RENAL PELVIS						
Pup Incidence	N	2	1	0	1	1
	%	1.5	0.7	0.0	0.9	0.9
Litter Incidence	N	2Fi	1	0	1	1
	%	10	5.0	0.0	5.3	5.3
Affected Pups/Litter	MEAN%	1.8Wi	0.7	0.0	1.3	0.9
	S.D.	5.67	3.19	0.00	5.74	3.82
HYDRONEPHROSIS						
Pup Incidence	N	1	0	0	0	0
	%	0.8	0.0	0.0	0.0	0.0
Litter Incidence	N	1Fi	0	0	0	0
	%	5.0	0.0	0.0	0.0	0.0
Affected Pups/Litter	MEAN%	1.0Wi	0.0	0.0	0.0	0.0
	S.D.	4.47	0.00	0.00	0.00	0.00
HYDROURETER						
Pup Incidence	N	1	0	0	0	0
	%	0.8	0.0	0.0	0.0	0.0
Litter Incidence	N	1Fi	0	0	0	0
	%	5.0	0.0	0.0	0.0	0.0
Affected Pups/Litter	MEAN%	1.0Wi	0.0	0.0	0.0	0.0
	S.D.	4.47	0.00	0.00	0.00	0.00

Statistics: Fi =Fisher's exact test (one-sided) Wi =Wilcoxon-test (one-sided)
* : p<=0.05 ** : p<=0.01

PR.NO.60R0375/88R002: REPRODUCTIVE TOX. STUDY TO DETECT EFFECTS OF MIXED ANTI-ANDROGENIC SUBSTANCES IN RATS; ORAL ADM. (GAVAGE)

SUMMARY OF PUP NECROPSY OBSERVATIONS

		TEST GROUP 0 0 MG/KG BW/D	TEST GROUP 1 ADI-MIX	TEST GROUP 2 NOAEL-MIX	TEST GROUP 3 LOAEL-MIX	TEST GROUP 4 0.00025 MG/KG BW/D
TOTAL PUP NECROPSY OBSERVATIONS						
Pup Incidence	N	3	2	5	13	3
	%	2.3	1.4	3.7	11	2.6
Litter Incidence	N	3Fi	2	2	7	2
	%	15	10	10	37	11
Affected Pups/Litter	MEAN%	2.5Wi	1.3	3.1	11.3	2.6
	S.D.	6.29	3.94	10.43	19.36	8.36

Statistics: Fi =Fisher's exact test (one-sided) Wi =Wilcoxon-test (one-sided)
* : p<=0.05 ** : p<=0.01

PR.NO.60R0375/88R002: REPRODUCTIVE TOX. STUDY TO DETECT EFFECTS
OF MIXED ANTI-ANDROGENIC SUBSTANCES IN RATS; ORAL ADM. (GAVAGE)

MALES

SUMMARY OF MALE CLINICAL OBSERVATIONS

	GROUP#	WEEK OF STUDY 0	1	2	3	4	5	TOTAL
# OF ANIMALS EXAMINED	0	10	10	10	10	3	0	
	1	10	10	10	10	1	0	
	2	10	10	10	10	2	0	
	3	10	10	10	10	9	5	
	4	10	10	10	8	1	0	
NORMAL								
NOTHING ABNORMAL DETECTED	0	10	10	10	10	3	-	10
	1	10	10	10	10	1	-	10
	2	10	10	10	10	2	-	10
	3	10	10	3	1	4	5	10
	4	10	10	10	8	1	-	10
DEAD								
SCHEDULED SACRIFICE	0	0	0	0	7	3	-	10
	1	0	0	0	9	1	-	10
	2	0	0	0	8	2	-	10
	3	0	0	0	1	4	5	10
	4	0	0	2	7	1	-	10
ORAL-BUCCAL								
SALIVATION AFTER TREATMENT	0	0	0	0	0	0	-	0
	1	0	0	0	0	0	-	0
	2	0	0	0	0	0	-	0
	3	0	8	10	10	9	4	10
	4	0	0	0	0	0	-	0

PR.NO.60R0375/88R002: REPRODUCTIVE TOX. STUDY TO DETECT EFFECTS
OF MIXED ANTI-ANDROGENIC SUBSTANCES IN RATS; ORAL ADM. (GAVAGE)

FEMALES

SUMMARY OF FEMALE CLINICAL OBSERVATIONS

	GROUP#	WEEK OF STUDY 0	1	TOTAL
# OF ANIMALS EXAMINED	0	10	10	
	1	10	10	
	2	10	10	
	3	10	10	
	4	10	10	
NORMAL				
NOTHING ABNORMAL DETECTED	0	10	10	10
	1	10	10	10
	2	10	10	10
	3	10	10	10
	4	10	10	10
DEAD				
SCHEDULED SACRIFICE	0	0	10	10
	1	0	10	10
	2	0	10	10
	3	0	10	10
	4	0	10	10

PR.NO.60R0375/88R002: REPRODUCTIVE TOX. STUDY TO DETECT EFFECTS
OF MIXED ANTI-ANDROGENIC SUBSTANCES IN RATS; ORAL ADM. (GAVAGE)

MALES

SUMMARY OF MALE CLINICAL OBSERVATIONS

	GROUP#	WEEK OF STUDY 0	1	2	3	4	5	6	7	8	9	TOTAL
# OF ANIMALS EXAMINED	0	10	10	9	9	9	9	9	9	9	2	
	1	10	10	10	10	10	10	10	10	10	2	
	2	10	10	10	10	10	10	10	10	10	4	
	3	10	10	10	10	10	10	10	10	10	3	
	4	10	10	10	10	10	10	10	10	10	3	
NORMAL												
NOTHING ABNORMAL DETECTED	0	10	10	9	9	9	9	9	9	9	2	10
	1	10	10	10	10	10	10	10	10	10	2	10
	2	10	10	10	10	10	10	10	10	10	4	10
	3	10	10	0	0	0	0	1	8	8	3	10
	4	10	10	10	10	10	10	10	10	10	3	10
DEAD												
SACRIFICED MORIBUND	0	0	1	0	0	0	0	0	0	0	0	1
	1	0	0	0	0	0	0	0	0	0	0	0
	2	0	0	0	0	0	0	0	0	0	0	0
	3	0	0	0	0	0	0	0	0	0	0	0
	4	0	0	0	0	0	0	0	0	0	0	0
SCHEDULED SACRIFICE	0	0	0	0	0	0	0	0	0	7	2	9
	1	0	0	0	0	0	0	0	0	8	2	10
	2	0	0	0	0	0	0	0	0	6	4	10
	3	0	0	0	0	0	0	0	0	7	3	10
	4	0	0	0	0	0	0	0	0	7	3	10
BODY POSITION												
OBLIQUE HEAD POSITION	0	0	1	0	0	0	0	0	0	0	0	1
	1	0	0	0	0	0	0	0	0	0	0	0
	2	0	0	0	0	0	0	0	0	0	0	0
	3	0	0	0	0	0	0	0	0	0	0	0
	4	0	0	0	0	0	0	0	0	0	0	0
ANIMAL BODY												
HEAD: SWELLING	0	0	1	0	0	0	0	0	0	0	0	1
	1	0	0	0	0	0	0	0	0	0	0	0
	2	0	0	0	0	0	0	0	0	0	0	0
	3	0	0	0	0	0	0	0	0	0	0	0
	4	0	0	0	0	0	0	0	0	0	0	0

PR.NO.60R0375/88R002: REPRODUCTIVE TOX. STUDY TO DETECT EFFECTS
OF MIXED ANTI-ANDROGENIC SUBSTANCES IN RATS; ORAL ADM. (GAVAGE)
SUMMARY OF MALE CLINICAL OBSERVATIONS

MALES

		WEEK OF STUDY										
	GROUP#	0	1	2	3	4	5	6	7	8	9	TOTAL
# OF ANIMALS EXAMINED	0	10	10	9	9	9	9	9	9	9	2	
	1	10	10	10	10	10	10	10	10	10	2	
	2	10	10	10	10	10	10	10	10	10	4	
	3	10	10	10	10	10	10	10	10	10	3	
	4	10	10	10	10	10	10	10	10	10	3	
EYES												
LACRIMATION	0	0	1	0	0	0	0	0	0	0	0	1
	1	0	0	0	0	0	0	0	0	0	0	0
	2	0	0	0	0	0	0	0	0	0	0	0
	3	0	0	0	0	0	0	0	0	0	0	0
	4	0	0	0	0	0	0	0	0	0	0	0
ORAL-BUCCAL												
SALIVATION AFTER TREATMENT	0	0	0	0	0	0	0	0	0	0	0	0
	1	0	0	0	0	0	0	0	0	0	0	0
	2	0	0	0	0	0	0	0	0	0	0	0
	3	0	10	10	10	10	10	10	10	10	0	10
	4	0	0	0	0	0	0	0	0	0	0	0

PR.NO.60R0375/88R002: REPRODUCTIVE TOX. STUDY TO DETECT EFFECTS
OF MIXED ANTI-ANDROGENIC SUBSTANCES IN RATS; ORAL ADM. (GAVAGE)

FEMALES

SUMMARY OF FEMALE CLINICAL OBSERVATIONS

	GROUP#	WEEK OF STUDY 0	1	2	3	4	5	6	7	8	9	TOTAL
# OF ANIMALS EXAMINED	0	10	10	9	9	9	9	9	9	9	3	
	1	10	10	10	10	10	10	10	10	10	3	
	2	10	10	10	10	10	10	10	10	10	2	
	3	10	10	10	10	10	10	10	10	10	3	
	4	10	10	10	10	10	10	10	10	10	1	
NORMAL												
NOTHING ABNORMAL DETECTED	0	10	10	9	9	9	9	9	9	9	3	10
	1	10	10	10	10	10	10	10	10	10	3	10
	2	10	10	10	10	10	10	10	10	10	2	10
	3	10	10	0	2	0	0	2	6	7	3	10
	4	10	10	10	10	10	10	10	10	10	1	10
DEAD												
SACRIFICED MORIBUND	0	0	1	0	0	0	0	0	0	0	0	1
	1	0	0	0	0	0	0	0	0	0	0	0
	2	0	0	0	0	0	0	0	0	0	0	0
	3	0	0	0	0	0	0	0	0	0	0	0
	4	0	0	0	0	0	0	0	0	0	0	0
SCHEDULED SACRIFICE	0	0	0	0	0	0	0	0	0	6	3	9
	1	0	0	0	0	0	0	0	0	7	3	10
	2	0	0	0	0	0	0	0	0	8	2	10
	3	0	0	0	0	0	0	0	0	7	3	10
	4	0	0	0	0	0	0	0	0	9	1	10
ANIMAL BODY												
INDRAWN FLANKS	0	0	1	0	0	0	0	0	0	0	0	1
	1	0	0	0	0	0	0	0	0	0	0	0
	2	0	0	0	0	0	0	0	0	0	0	0
	3	0	0	0	0	0	0	0	0	0	0	0
	4	0	0	0	0	0	0	0	0	0	0	0
MISCELLANEOUS												
VAGINAL HEMORRHAGE	0	0	0	0	0	0	0	0	0	0	0	0
	1	0	0	0	0	0	0	0	0	1	0	1
	2	0	0	0	0	0	0	0	0	0	0	0
	3	0	0	0	0	0	0	0	0	0	0	0
	4	0	0	0	0	0	0	0	0	0	0	0

PR.NO.60R0375/88R002: REPRODUCTIVE TOX. STUDY TO DETECT EFFECTS
OF MIXED ANTI-ANDROGENIC SUBSTANCES IN RATS; ORAL ADM. (GAVAGE)

FEMALES

SUMMARY OF FEMALE CLINICAL OBSERVATIONS

	GROUP#	WEEK OF STUDY 0	1	2	3	4	5	6	7	8	9	TOTAL
# OF ANIMALS EXAMINED	0	10	10	9	9	9	9	9	9	9	3	
	1	10	10	10	10	10	10	10	10	10	3	
	2	10	10	10	10	10	10	10	10	10	2	
	3	10	10	10	10	10	10	10	10	10	3	
	4	10	10	10	10	10	10	10	10	10	1	
ORAL-BUCCAL												
SALIVATION AFTER TREATMENT	0	0	0	0	0	0	0	0	0	0	0	0
	1	0	0	0	0	0	0	0	0	0	0	0
	2	0	0	0	0	0	0	0	0	0	0	0
	3	0	10	10	10	10	10	10	10	10	0	10
	4	0	0	0	0	0	0	0	0	0	0	0
RESPIRATION												
LABORED RESPIRATION	0	0	1	0	0	0	0	0	0	0	0	1
	1	0	0	0	0	0	0	0	0	0	0	0
	2	0	0	0	0	0	0	0	0	0	0	0
	3	0	0	0	0	0	0	0	0	0	0	0
	4	0	0	0	0	0	0	0	0	0	0	0
SKIN/FUR												
PILOERECTION	0	0	1	0	0	0	0	0	0	0	0	1
	1	0	0	0	0	0	0	0	0	0	0	0
	2	0	0	0	0	0	0	0	0	0	0	0
	3	0	0	0	0	0	0	0	0	0	0	0
	4	0	0	0	0	0	0	0	0	0	0	0

PR.NO.60R0375/88R002: REPRODUCTIVE TOX. STUDY TO DETECT EFFECTS
OF MIXED ANTI-ANDROGENIC SUBSTANCES IN RATS; ORAL ADM. (GAVAGE)
MEAN FOOD CONSUMPTION -- GRAMS/ANIMAL/DAY

MALES

		TEST GROUP 0<br>0 MG/KG BW/D	TEST GROUP 1<br>ADI-MIX	TEST GROUP 2<br>NOAEL-MIX	TEST GROUP 3<br>LOAEL-MIX	TEST GROUP 4<br>0.00025 MG/KG BW/D
WEEK 0 TO 1	MEAN	8.6 D	8.8	9.0	8.6	9.4
	S.D.	0.73	0.63	0.36	0.72	1.02
	N	10	10	10	10	10
WEEK 1 TO 2	MEAN	13.7 D	14.3	14.1	13.3	15.1*
	S.D.	1.03	1.13	0.84	0.75	1.23
	N	10	10	10	10	10
WEEK 0 TO 2	MEAN OF MEANS	11.2	11.5	11.5	10.9	12.2
	S.D.	3.60	3.82	3.61	3.30	4.04
	N	2	2	2	2	2

Statistics: D=Dunnett-test (two-sided)
* : p<=0.05 ** : p<=0.01

PR.NO.60R0375/88R002: REPRODUCTIVE TOX. STUDY TO DETECT EFFECTS
OF MIXED ANTI-ANDROGENIC SUBSTANCES IN RATS; ORAL ADM. (GAVAGE)
MEAN FOOD CONSUMPTION -- GRAMS/ANIMAL/DAY

FEMALES

		TEST GROUP 0 0 MG/KG BW/D	TEST GROUP 1 ADI-MIX	TEST GROUP 2 NOAEL-MIX	TEST GROUP 3 LOAEL-MIX	TEST GROUP 4 0.00025 MG/KG BW/D
WEEK 0 TO 1	MEAN	8.5 D	8.5	8.6	8.3	8.5
	S.D.	0.84	0.65	0.50	0.66	0.87
	N	10	10	10	10	10

Statistics: D=Dunnett-test (two-sided)
* : p<=0.05 ** : p<=0.01

PR.NO.60R0375/88R002: REPRODUCTIVE TOX. STUDY TO DETECT EFFECTS
OF MIXED ANTI-ANDROGENIC SUBSTANCES IN RATS; ORAL ADM. (GAVAGE)
MEAN FOOD CONSUMPTION -- GRAMS/ANIMAL/DAY

MALES

		TEST GROUP 0 0 MG/KG BW/D	TEST GROUP 1 ADI-MIX	TEST GROUP 2 NOAEL-MIX	TEST GROUP 3 LOAEL-MIX	TEST GROUP 4 0.00025 MG/KG BW/D
WEEK 0 TO 1	MEAN	8.8 D	8.9	9.0	8.9	9.5
	S.D.	0.74	0.72	0.40	0.35	0.77
	N	10	10	10	10	10
WEEK 1 TO 2	MEAN	14.0 D	14.0	13.9	13.0	14.8
	S.D.	0.92	0.77	0.74	2.10	1.05
	N	9	10	10	10	10
WEEK 2 TO 3	MEAN	18.3 D	18.4	17.5	17.1	19.3
	S.D.	1.51	1.17	1.24	1.29	1.33
	N	9	10	10	10	10
WEEK 3 TO 4	MEAN	19.3 D	19.1	18.4	18.5	20.2
	S.D.	1.28	1.44	1.25	1.49	1.34
	N	9	10	10	10	10
WEEK 4 TO 5	MEAN	20.5 D	19.6	19.7	19.5	20.8
	S.D.	1.66	1.26	1.62	1.74	1.83
	N	9	10	10	10	10
WEEK 5 TO 6	MEAN	20.5 D	19.9	20.1	20.3	21.1
	S.D.	1.26	1.51	1.91	1.75	1.51
	N	9	10	10	10	10
WEEK 6 TO 7	MEAN	20.0 D	19.3	19.3	19.9	20.3
	S.D.	1.61	2.02	1.86	1.95	1.74
	N	9	10	10	10	10
WEEK 7 TO 8	MEAN	19.9 D	18.8	19.3	19.6	20.2
	S.D.	1.75	1.92	2.07	2.32	1.57
	N	9	10	10	10	10
WEEK 0 TO 8	MEAN OF MEANS	17.7	17.2	17.1	17.1	18.3
	S.D.	4.17	3.86	3.86	4.08	4.06
	N	8	8	8	8	8

Statistics: D=Dunnett-test (two-sided)
* : p<=0.05 ** : p<=0.01

PR.NO.60R0375/88R002: REPRODUCTIVE TOX. STUDY TO DETECT EFFECTS
OF MIXED ANTI-ANDROGENIC SUBSTANCES IN RATS; ORAL ADM. (GAVAGE)
MEAN FOOD CONSUMPTION -- GRAMS/ANIMAL/DAY

FEMALES

		TEST GROUP 0<br>0 MG/KG BW/D	TEST GROUP 1<br>ADI-MIX	TEST GROUP 2<br>NOAEL-MIX	TEST GROUP 3<br>LOAEL-MIX	TEST GROUP 4<br>0.00025 MG/KG<br>BW/D
WEEK 0 TO 1	MEAN	8.2 D	8.2	8.5	8.7	8.8
	S.D.	0.71	0.70	0.53	0.60	0.96
	N	10	10	10	10	10
WEEK 1 TO 2	MEAN	12.1 D	12.1	11.9	12.4	12.8
	S.D.	1.57	1.44	1.49	0.67	1.26
	N	9	10	10	10	10
WEEK 2 TO 3	MEAN	13.7 D	14.2	14.0	13.9	14.5
	S.D.	1.10	1.19	1.15	0.85	1.27
	N	9	10	10	10	10
WEEK 3 TO 4	MEAN	13.7 D	14.0	14.3	14.2	14.0
	S.D.	0.65	0.68	1.42	0.92	1.34
	N	9	10	10	10	10
WEEK 4 TO 5	MEAN	13.6 D	14.0	14.3	14.4	14.1
	S.D.	0.90	0.91	1.19	1.35	1.19
	N	9	10	10	10	10
WEEK 5 TO 6	MEAN	13.8 D	14.1	14.6	14.5	14.1
	S.D.	1.15	0.74	0.99	1.07	1.51
	N	9	10	10	10	10
WEEK 6 TO 7	MEAN	14.1 D	14.7	15.5	16.1*	14.6
	S.D.	1.29	1.85	1.32	1.60	1.22
	N	9	10	10	10	10
WEEK 7 TO 8	MEAN	14.6 D	14.2	15.3	15.1	14.9
	S.D.	1.56	1.01	1.93	1.67	1.98
	N	9	10	10	10	10
WEEK 0 TO 8	MEAN OF MEANS	13.0	13.2	13.5	13.7	13.5
	S.D.	2.05	2.17	2.31	2.27	1.98
	N	8	8	8	8	8

Statistics: D=Dunnett-test (two-sided)
* : p<=0.05 ** : p<=0.01

PR.NO.60R0375/88R002: REPRODUCTIVE TOX. STUDY TO DETECT EFFECTS
OF MIXED ANTI-ANDROGENIC SUBSTANCES IN RATS; ORAL ADM. (GAVAGE)

MALES

MEAN BODY WEIGHTS -- GRAMS

		TEST GROUP 0<br>0 MG/KG BW/D	TEST GROUP 1<br>ADI-MIX	TEST GROUP 2<br>NOAEL-MIX	TEST GROUP 3<br>LOAEL-MIX	TEST GROUP 4<br>0.00025 MG/KG<br>BW/D
WEEK 0	MEAN	43.5 D	45.4	46.3	47.9	47.9
	S.D.	3.13	5.37	4.51	2.16	6.37
	N	10	10	10	10	10
WEEK 1	MEAN	75.8 D	77.8	78.2	76.2	82.8
	S.D.	5.58	6.52	4.73	4.58	9.20
	N	10	10	10	10	10
WEEK 2	MEAN	120.1 D	123.4	123.2	117.8	130.6*
	S.D.	8.29	9.41	7.17	5.24	12.26
	N	10	10	10	10	10

Statistics: D=Dunnett-test (two-sided)
* : p<=0.05 ** : p<=0.01

PR.NO.60R0375/88R002: REPRODUCTIVE TOX. STUDY TO DETECT EFFECTS OF MIXED ANTI-ANDROGENIC SUBSTANCES IN RATS; ORAL ADM. (GAVAGE)

FEMALES

MEAN BODY WEIGHTS -- GRAMS

		TEST GROUP 0<br>0 MG/KG BW/D	TEST GROUP 1<br>ADI-MIX	TEST GROUP 2<br>NOAEL-MIX	TEST GROUP 3<br>LOAEL-MIX	TEST GROUP 4<br>0.00025 MG/KG BW/D
WEEK 0	MEAN	44.4 D	44.0	44.2	46.7	45.3
	S.D.	4.48	5.45	3.89	4.07	5.81
	N	10	10	10	10	10
WEEK 1	MEAN	73.5 D	73.5	74.0	73.4	74.2
	S.D.	5.21	7.64	4.37	5.07	6.81
	N	10	10	10	10	10

Statistics: D=Dunnett-test (two-sided)
* : p<=0.05 ** : p<=0.01

PR.NO.60R0375/88R002: REPRODUCTIVE TOX. STUDY TO DETECT EFFECTS OF MIXED ANTI-ANDROGENIC SUBSTANCES IN RATS; ORAL ADM. (GAVAGE)

MALES

MEAN BODY WEIGHTS -- GRAMS

		TEST GROUP 0<br>0 MG/KG BW/D	TEST GROUP 1<br>ADI-MIX	TEST GROUP 2<br>NOAEL-MIX	TEST GROUP 3<br>LOAEL-MIX	TEST GROUP 4<br>0.00025 MG/KG BW/D
WEEK 0	MEAN	44.7 D	44.9	46.4	48.8	47.5
	S.D.	3.21	5.30	3.54	4.99	5.67
	N	10	10	10	10	10
WEEK 1	MEAN	76.8 D	77.2	78.3	78.5	80.7
	S.D.	5.40	7.41	4.00	5.64	7.52
	N	10	10	10	10	10
WEEK 2	MEAN	120.9 D	121.0	121.6	118.4	126.4
	S.D.	7.34	9.00	5.66	11.40	10.41
	N	9	10	10	10	10
WEEK 3	MEAN	162.6 D	164.6	164.1	157.9	171.1
	S.D.	10.04	13.15	8.72	11.68	12.39
	N	9	10	10	10	10
WEEK 4	MEAN	202.3 D	201.6	198.6	195.8	210.0
	S.D.	12.50	16.71	14.39	14.78	17.36
	N	9	10	10	10	10
WEEK 5	MEAN	239.3 D	235.7	235.0	230.3	248.9
	S.D.	15.99	19.73	17.77	16.14	20.08
	N	9	10	10	10	10
WEEK 6	MEAN	265.4 D	262.3	261.8	258.4	274.0
	S.D.	18.94	24.04	23.20	19.48	21.47
	N	9	10	10	10	10
WEEK 7	MEAN	289.0 D	282.9	287.0	281.4	295.7
	S.D.	23.53	28.93	24.41	22.54	23.81
	N	9	10	10	10	10
WEEK 8	MEAN	309.1 D	301.5	306.4	299.5	315.4
	S.D.	25.44	31.31	27.97	26.65	27.25
	N	9	10	10	10	10

Statistics: D=Dunnett-test (two-sided)
* : p<=0.05 ** : p<=0.01

PR.NO.60R0375/88R002: REPRODUCTIVE TOX. STUDY TO DETECT EFFECTS
OF MIXED ANTI-ANDROGENIC SUBSTANCES IN RATS; ORAL ADM. (GAVAGE)

FEMALES

MEAN BODY WEIGHTS -- GRAMS

		TEST GROUP 0 0 MG/KG BW/D	TEST GROUP 1 ADI-MIX	TEST GROUP 2 NOAEL-MIX	TEST GROUP 3 LOAEL-MIX	TEST GROUP 4 0.00025 MG/KG BW/D
WEEK 0	MEAN	44.8 D	44.5	44.0	46.3	49.1
	S.D.	3.70	6.41	4.22	3.12	5.60
	N	10	10	10	10	10
WEEK 1	MEAN	73.2 D	72.5	73.3	74.6	79.2
	S.D.	4.60	8.22	6.01	4.41	8.30
	N	10	10	10	10	10
WEEK 2	MEAN	104.7 D	106.9	105.9	106.8	114.2
	S.D.	10.76	13.54	12.23	5.29	11.35
	N	9	10	10	10	10
WEEK 3	MEAN	128.1 D	130.7	131.4	130.1	135.1
	S.D.	10.96	13.10	9.90	6.96	10.10
	N	9	10	10	10	10
WEEK 4	MEAN	144.5 D	148.5	150.1	147.1	152.0
	S.D.	9.85	12.68	10.91	6.49	14.19
	N	9	10	10	10	10
WEEK 5	MEAN	158.0 D	161.8	164.8	161.4	167.1
	S.D.	11.95	10.50	12.89	7.95	13.05
	N	9	10	10	10	10
WEEK 6	MEAN	172.8 D	175.7	178.5	178.8	179.7
	S.D.	10.46	12.37	12.60	9.74	13.30
	N	9	10	10	10	10
WEEK 7	MEAN	186.0 D	190.9	197.2	198.0	192.0
	S.D.	12.42	15.89	13.57	12.38	14.26
	N	9	10	10	10	10
WEEK 8	MEAN	195.1 D	197.2	205.5	201.2	204.9
	S.D.	14.82	11.55	16.07	10.74	17.36
	N	9	10	10	10	10

Statistics: D=Dunnett-test (two-sided)
* : p<=0.05 ** : p<=0.01

PR.NO.60R0375/88R002: REPRODUCTIVE TOX. STUDY TO DETECT EFFECTS OF MIXED ANTI-ANDROGENIC SUBSTANCES IN RATS; ORAL ADM. (GAVAGE)

MALES

MEAN BODY WEIGHT CHANGE -- GRAMS

		TEST GROUP 0 0 MG/KG BW/D	TEST GROUP 1 ADI-MIX	TEST GROUP 2 NOAEL-MIX	TEST GROUP 3 LOAEL-MIX	TEST GROUP 4 0.00025 MG/KG BW/D
WEEK 0 TO 1	MEAN	32.3 D	32.3	31.9	28.4*	34.9
	S.D.	3.48	2.60	1.91	3.29	3.90
	N	10	10	10	10	10
WEEK 1 TO 2	MEAN	44.3 D	45.6	45.0	41.6	47.8
	S.D.	3.97	4.22	3.27	1.71	4.41
	N	10	10	10	10	10
WEEK 0 TO 2	MEAN	76.6 D	78.0	76.9	69.9*	82.7
	S.D.	6.74	5.28	3.79	4.24	7.71
	N	10	10	10	10	10

Statistics: D=Dunnett-test (two-sided)
* : p<=0.05 ** : p<=0.01

PR.NO.60R0375/88R002: REPRODUCTIVE TOX. STUDY TO DETECT EFFECTS
OF MIXED ANTI-ANDROGENIC SUBSTANCES IN RATS; ORAL ADM. (GAVAGE)

FEMALES

MEAN BODY WEIGHT CHANGE -- GRAMS

		TEST GROUP 0 0 MG/KG BW/D	TEST GROUP 1 ADI-MIX	TEST GROUP 2 NOAEL-MIX	TEST GROUP 3 LOAEL-MIX	TEST GROUP 4 0.00025 MG/KG BW/D
WEEK 0 TO 1	MEAN	29.1 D	29.5	29.8	26.7	28.9
	S.D.	2.95	2.94	1.50	2.73	3.69
	N	10	10	10	10	10

Statistics: D=Dunnett-test (two-sided)
* : p<=0.05 ** : p<=0.01

PR.NO.60R0375/88R002: REPRODUCTIVE TOX. STUDY TO DETECT EFFECTS
OF MIXED ANTI-ANDROGENIC SUBSTANCES IN RATS; ORAL ADM. (GAVAGE)

MALES

MEAN BODY WEIGHT CHANGE -- GRAMS

		TEST GROUP 0<br>0 MG/KG BW/D	TEST GROUP 1<br>ADI-MIX	TEST GROUP 2<br>NOAEL-MIX	TEST GROUP 3<br>LOAEL-MIX	TEST GROUP 4<br>0.00025 MG/KG<br>BW/D
WEEK 0 TO 1	MEAN	32.1 D	32.2	32.0	29.6	33.3
	S.D.	3.52	2.68	2.30	2.34	3.53
	N	10	10	10	10	10
WEEK 1 TO 2	MEAN	43.8 D	43.8	43.2	40.0	45.7
	S.D.	2.35	3.51	3.04	9.59	4.78
	N	9	10	10	10	10
WEEK 2 TO 3	MEAN	41.6 D	43.6	42.5	39.4	44.7
	S.D.	4.79	4.60	3.95	3.39	4.67
	N	9	10	10	10	10
WEEK 3 TO 4	MEAN	39.7 D	37.0	34.5	38.0	38.9
	S.D.	4.16	4.75	7.33	4.07	6.42
	N	9	10	10	10	10
WEEK 4 TO 5	MEAN	37.0 D	34.0	36.4	34.5	38.9
	S.D.	4.05	3.94	5.52	4.07	5.41
	N	9	10	10	10	10
WEEK 5 TO 6	MEAN	26.1 D	26.6	26.8	28.1	25.1
	S.D.	4.38	5.90	7.27	5.96	6.85
	N	9	10	10	10	10
WEEK 6 TO 7	MEAN	23.6 D	20.6	25.2	23.0	21.7
	S.D.	6.12	5.73	7.44	5.45	6.83
	N	9	10	10	10	10
WEEK 7 TO 8	MEAN	20.2 D	18.6	19.4	18.1	19.7
	S.D.	5.63	4.11	4.71	5.51	6.85
	N	9	10	10	10	10
WEEK 0 TO 8	MEAN	264.3 D	256.5	260.0	250.6	267.9
	S.D.	24.84	27.71	26.53	28.19	29.65
	N	9	10	10	10	10

Statistics: D=Dunnett-test (two-sided)
* : p<=0.05 ** : p<=0.01

PR.NO.60R0375/88R002: REPRODUCTIVE TOX. STUDY TO DETECT EFFECTS
OF MIXED ANTI-ANDROGENIC SUBSTANCES IN RATS; ORAL ADM. (GAVAGE)
MEAN BODY WEIGHT CHANGE -- GRAMS

FEMALES

		TEST GROUP 0<br>0 MG/KG BW/D	TEST GROUP 1<br>ADI-MIX	TEST GROUP 2<br>NOAEL-MIX	TEST GROUP 3<br>LOAEL-MIX	TEST GROUP 4<br>0.00025 MG/KG BW/D
WEEK 0 TO 1	MEAN	28.4 D	28.0	29.4	28.3	30.1
	S.D.	3.06	2.86	3.58	2.81	4.61
	N	10	10	10	10	10
WEEK 1 TO 2	MEAN	30.8 D	34.4	32.6	32.3	34.9
	S.D.	7.37	6.78	7.18	2.69	4.03
	N	9	10	10	10	10
WEEK 2 TO 3	MEAN	23.5 D	23.8	25.4	23.2	21.0
	S.D.	4.38	6.72	6.33	2.90	4.00
	N	9	10	10	10	10
WEEK 3 TO 4	MEAN	16.4 D	17.8	18.8	17.1	16.9
	S.D.	3.59	2.57	3.88	2.44	5.84
	N	9	10	10	10	10
WEEK 4 TO 5	MEAN	13.5 D	13.4	14.7	14.3	15.1
	S.D.	4.40	3.66	3.62	6.96	4.26
	N	9	10	10	10	10
WEEK 5 TO 6	MEAN	14.7 D	13.9	13.7	17.4	12.6
	S.D.	4.19	5.27	6.66	4.47	5.29
	N	9	10	10	10	10
WEEK 6 TO 7	MEAN	13.2 D	15.2	18.6	19.2	12.2
	S.D.	6.04	7.47	8.54	6.19	6.87
	N	9	10	10	10	10
WEEK 7 TO 8	MEAN	9.1 D	6.2	8.4	3.2	13.0
	S.D.	5.45	10.21	6.85	4.91	8.20
	N	9	10	10	10	10
WEEK 0 TO 8	MEAN	150.3 D	152.7	161.6	155.0	155.8
	S.D.	11.90	6.44	17.15	11.61	16.06
	N	9	10	10	10	10

Statistics: D=Dunnett-test (two-sided)
* : p<=0.05 ** : p<=0.01

PR.NO.60R0375/88R002: REPRODUCTIVE TOX. STUDY TO DETECT EFFECTS
OF MIXED ANTI-ANDROGENIC SUBSTANCES IN RATS; ORAL ADM. (GAVAGE)
SUMMARY OF SEXUAL MATURATION DATA (CUMULATED VALUE)

		TEST GROUP 0 0 MG/KG BW/D	TEST GROUP 1 ADI-MIX	TEST GROUP 2 NOAEL-MIX	TEST GROUP 3 LOAEL-MIX	TEST GROUP 4 0.00025 MG/KG BW/D
VAGINAL OPENING Pups reaching criteria						
Pups tested	N	20	20	20	20	20
day 27	N	0 F	0	0	1	0
	%	0.0	0.0	0.0	5.0	0.0
day 28	N	2 F	2	2	2	1
	%	10	10	10	10	5.0
day 29	N	4 F	5	8	6	4
	%	20	25	40	30	20
day 30	N	10 F	9	13	13	11
	%	50	45	65	65	55
day 31	N	12 F	16	18	19*	16
	%	60	80	90	95	80
day 32	N	15 F	17	19	19	18
	%	75	85	95	95	90
day 33	N	19 F	20	19	20	19
	%	95	100	95	100	95
day 34	N	20 F	20	20	20	19
	%	100	100	100	100	95
day 35	N	20 F	20	20	20	20
	%	100	100	100	100	100
days to criterion	MEAN	30.9 D	30.5	30.0	30.0	30.6
	S.D.	1.77	1.50	1.43	1.30	1.57
	N	20	20	20	20	20
body weight at criterion	MEAN	84.2 D	85.6	82.6	83.1	90.2
	S.D.	9.07	7.04	7.39	6.59	9.66
	N	16	15	17	17	17

Statistics: D=Dunnett-test (two-sided) F=Fisher's exact test (two-sided)
* : p<=0.05 ** : p<=0.01

PR.NO.60R0375/88R002: REPRODUCTIVE TOX. STUDY TO DETECT EFFECTS
OF MIXED ANTI-ANDROGENIC SUBSTANCES IN RATS; ORAL ADM. (GAVAGE)
SUMMARY OF SEXUAL MATURATION DATA (CUMULATED VALUE)

		TEST GROUP 0 0 MG/KG BW/D	TEST GROUP 1 ADI-MIX	TEST GROUP 2 NOAEL-MIX	TEST GROUP 3 LOAEL-MIX	TEST GROUP 4 0.00025 MG/KG BW/D
PREPUTIAL SEPARATION Pups reaching criteria						
Pups tested	N	19	20	20	20	20
day 40	N	0 F	0	0	0	2
	%	0.0	0.0	0.0	0.0	10
day 41	N	1 F	0	0	0	3
	%	5.3	0.0	0.0	0.0	15
day 42	N	3 F	3	2	0	4
	%	16	15	10	0.0	20
day 43	N	4 F	6	4	0*	5
	%	21	30	20	0.0	25
day 44	N	7 F	8	4	0**	6
	%	37	40	20	0.0	30
day 45	N	8 F	12	5	0**	8
	%	42	60	25	0.0	40
day 46	N	9 F	13	10	0**	8
	%	47	65	50	0.0	40
day 47	N	12 F	15	12	0**	14
	%	63	75	60	0.0	70
day 48	N	15 F	18	14	1**	17
	%	79	90	70	5.0	85
day 49	N	18 F	19	17	2**	20
	%	95	95	85	10	100
day 50	N	19 F	20	18	4**	20
	%	100	100	90	20	100
day 51	N	19 F	20	19	5**	20
	%	100	100	95	25	100

Statistics: F=Fisher's exact test (two-sided)
* : p<=0.05 ** : p<=0.01

PR.NO.60R0375/88R002: REPRODUCTIVE TOX. STUDY TO DETECT EFFECTS OF MIXED ANTI-ANDROGENIC SUBSTANCES IN RATS; ORAL ADM. (GAVAGE)
SUMMARY OF SEXUAL MATURATION DATA (CUMULATED VALUE)

		TEST GROUP 0 0 MG/KG BW/D	TEST GROUP 1 ADI-MIX	TEST GROUP 2 NOAEL-MIX	TEST GROUP 3 LOAEL-MIX	TEST GROUP 4 0.00025 MG/KG BW/D
PREPUTIAL SEPARATION Pups reaching criteria						
day 52	N	19 F	20	19	6**	20
	%	100	100	95	30	100
day 53	N	19 F	20	20	8**	20
	%	100	100	100	40	100
day 54	N	19 F	20	20	9**	20
	%	100	100	100	45	100
day 55	N	19 F	20	20	11**	20
	%	100	100	100	55	100
day 56	N	19 F	20	20	13**	20
	%	100	100	100	65	100
day 57	N	19 F	20	20	16	20
	%	100	100	100	80	100
day 58	N	19 F	20	20	19	20
	%	100	100	100	95	100
day 60	N	19 F	20	20	20	20
	%	100	100	100	100	100
days to criterion	MEAN	45.9 D	45.3	46.8	54.3**	45.7
	S.D.	2.76	2.47	2.95	3.45	3.00
	N	19	20	20	20	20
body weight at criterion	MEAN	183.9 D	185.2	189.6	220.3**	189.8
	S.D.	15.45	13.04	17.22	19.74	14.23
	N	16	15	17	17	16

Statistics: D=Dunnett-test (two-sided) F=Fisher's exact test (two-sided)
* : p<=0.05 ** : p<=0.01

PR.NO.60R0375/88R002: REPRODUCTIVE TOX. STUDY TO DETECT EFFECTS
OF MIXED ANTI-ANDROGENIC SUBSTANCES IN RATS; ORAL ADM. (GAVAGE)
SUMMARY OF MEAN ESTROUS CYCLE DURATION

	TEST GROUP 0 0 MG/KG BW/D	TEST GROUP 1 ADI-MIX	TEST GROUP 2 NOAEL-MIX	TEST GROUP 3 LOAEL-MIX	TEST GROUP 4 0.00025 MG/KG BW/D
MEAN# OF DAYS FROM ESTRUS TO ESTRUS	5.2 D	6.3	6.0	6.8	5.3
S.D.	1.99	4.24	1.98	1.17	2.06
N	9	10	10	10	10

Statistics: D=Dunnett-test (two-sided)
* : p<=0.05 ** : p<=0.01

Study	**60R0375/88R002**	Study Type	**R Reproduction**
Species	**Rat**	Article	

Sex: **Female** - Phase: **In-life**

		G 0 / F	G 1 / F	G 2 / F	G 3 / F
day 31	Mean	26.76 x	23.32	19.87 **	16.66 **
	S.d.	7.22	7.06	6.09	8.45
	N	19	20	19	18
	Median	27.88	23.49	20.48	17.18

Statistic Profile = Wilcoxon test (two-sided), * p<=0.05, ** p <=0.01, X = Group excluded from statistics
x=WILCOX

Study	**60R0375/88R002**	Study Type	**R Reproduction**
Species	**Rat**	Article	

Sex: **Female** - Phase: **In-life**

		G 0 / F	G 4 / F
day 31	Mean	26.76 x	26.79
	S.d.	7.22	5.89
	N	19	19
	Median	27.88	26.45

Statistic Profile = Wilcoxon test (two-sided), * p<=0.05, ** p <=0.01, X = Group excluded from statistics
x=WILCOX

Study	**60R0375/88R002**		Study Type	**R Reproduction**
Species	**Rat**		Article	

Sex: **Female** - Phase: **In-life**

		G 0 / F	G 1 / F	G 2 / F	G 3 / F
day 29	Mean	8.80 x	9.14	8.30	8.90
	S.d.	2.72	3.81	3.00	4.06
	N	10	10	10	10
	Median	8.54	9.22	7.45	8.38

Statistic Profile = Wilcoxon test (two-sided), * p<=0.05, ** p <=0.01, X = Group excluded from statistics
x=WILCOX

Study	**60R0375/88R002**	Study Type	**R Reproduction**
Species	**Rat**	Article	

Sex: **Female** - Phase: **In-life**

		G 0 / F	G 4 / F
day 29	Mean	8.80 x	9.02
	S.d.	2.72	2.88
	N	10	10
	Median	8.54	9.19

Statistic Profile = Wilcoxon test (two-sided), * p<=0.05, ** p <=0.01, X = Group excluded from statistics
x=WILCOX

Study	**60R0375/88R002**	Study Type	**R Reproduction**
Species	**Rat**	Article	

Sex: **Female** - Phase: **In-life**

		G 0 / F	G 1 / F	G 2 / F	G 3 / F
day 70	Mean	23.55 x	21.90	17.00	18.12
	S.d.	9.78	6.05	8.38	6.72
	N	8	10	10	10
	Median	24.61	21.38	15.88	19.05

Statistic Profile = Wilcoxon test (two-sided), * p<=0.05, ** p <=0.01, X = Group excluded from statistics
x=WILCOX

Study	**60R0375/88R002**	Study Type	**R Reproduction**
Species	**Rat**	Article	

Sex: **Female** - Phase: **In-life**

		G 0 / F	G 4 / F
day 70	Mean	23.55 x	23.28
	S.d.	9.78	4.70
	N	8	10
	Median	24.61	22.70

Statistic Profile = Wilcoxon test (two-sided), * p<=0.05, ** p <=0.01, X = Group excluded from statistics
x=WILCOX

Study	**60R0375/88R002**	Study Type	**R Reproduction**
Species	**Rat**	Article	

Sex: **Male** - Phase: **In-life**

		G 0 / M		G 1 / M	G 2 / M	G 3 / M
MOTILE_C	Mean	91	x-	88	92	92
[%]	S.d.	6		8	4	6
day 84	N	9		10	10	10
	Median	91		90	90	94
TS/gT	Mean	199	x-	209	213	198
[---]	S.d.	23		32	27	22
day 84	N	9		10	10	10
	Median	201		205	216	200
TS/gC	Mean	643	x-	614	725	731
[---]	S.d.	100		110	188	262
day 84	N	9		10	10	10
	Median	608		615	763	679
ABNORMAL6_C	Mean	2	x+	2	2	2
[%]	S.d.	1		2	1	1
day 84	N	9		10	10	10
	Median	2		2	2	2

Statistic Profile = Wilcoxon test (one-sided+), Wilcoxon with Bonferroni-Holm (one-sided-), * p<=0.05, ** p <=0.01, X = Group excluded from statistics
x=WILCOX

Study	**60R0375/88R002**	Study Type	**R Reproduction**
Species	**Rat**	Article	

Sex: **Male** - Phase: **In-life**

		G 0 / M	G 4 / M
MOTILE_C	Mean	91 x-	92
[%]	S.d.	6	6
day 84	N	9	10
	Median	91	94
TS/gT	Mean	199 x-	233
[---]	S.d.	23	49
day 84	N	9	10
	Median	201	227
TS/gC	Mean	643 x-	677
[---]	S.d.	100	125
day 84	N	9	10
	Median	608	668
ABNORMAL6_C	Mean	2 x+	2
[%]	S.d.	1	1
day 84	N	9	10
	Median	2	1

Statistic Profile = Wilcoxon test (one-sided+), Wilcoxon with Bonferroni-Holm (one-sided-), * p<=0.05, ** p <=0.01, X = Group excluded from statistics
x=WILCOX

Dams after weaning in the proestrus

Compound	Dose (mg/kg bw/d)	Group	Androstenedione (nmol/L)				Testosterone (nmol/L)				Progesterone (nmol/L)				11-Deoxycorticosterone (nmol/L)				Corticosterone (nmol/L)			
			N	Mean	SD	Median	N	Mean	SD	Median	N	Mean	SD	Median	N	Mean	SD	Median	N	Mean	SD	Median
Controls (ctrl)		0	17	1.60	0.99	1.28	17	0.42	0.19	0.35	17	24.65	41.11	13.04	17	6.34	4.55	4.67	17	731.6	373.8	650.3
Vinclo+Flut+Prochl.	0.005+0.00025+0.01	1	20	1.26	0.78	1.00	20	0.39	0.11	0.35	20	32.27	48.28	15.12	20	10.96	10.37	6.42	20	863.3	438.6	764.4
	0.005+0.00025+0.01/ctrl			0.79		0.78				1.00				1.16				1.37				1.2
	4+0.025+5	2	19	1.55	0.88	1.48	19	0.44	0.17	0.35	19	27.66	39.67	15.84	19	7.76	7.65	5.69	19	843	448.6	849.7
	4+0.025+5/ctrl			0.97		1.16				1.00				1.21				1.22				1.3
	20+0.25+30	3	17	2.93	2.51	2.45	17	0.55	0.25	0.48	17	55.03	79.39	20.07	17	10.75	8.86	8.78	17	965.2	396.2	997.1
	20+0.25+30/ctrl			1.83		1.91				1.37				1.54				1.88				1.5

Kruskal-Wallis test + Mann-Whitney-U-test (two-sided), + p ≤ 0.05, ++ p ≤ 0.01
Vinclo = vinclozolin; Flut = flutamide; Prochl = prochloraz

Dams after weaning in the proestrus

Compound	Dose (mg/kg bw/d)	Group	Androstenedione (nmol/L)				Testosterone (nmol/L)				Progesterone (nmol/L)				11-Deoxycorticosterone (nmol/L)				Corticosterone (nmol/L)			
			N	Mean	SD	Median	N	Mean	SD	Median	N	Mean	SD	Median	N	Mean	SD	Median	N	Mean	SD	Median
Controls		0	17	1.60	0.99	1.28	17	0.42	0.19	0.35	17	24.65	41.11	13.04	17	6.34	4.55	4.67	17	731.6	373.8	650.3
Flutamide	0.00025	4	18	1.48	0.75	1.27	18	0.39	0.08	0.35	18	22.00	22.48	14.76	18	10.84	11.02	8.03	18	969.9	565.4	984.0
	0.00025/ctrl			0.93		0.99				1.00				1.13				1.72				1.51

Mann-Whitney-U-test (two-sided), + $p \leq 0.05$, ++ $p \leq 0.01$

Males PND 21

Compound	Dose (mg/kg bw/d)	Group	Androstenedione (nmol/L)								Testosterone (nmol/L)							
			N	Mean	SD	Median	25th Percentile	75th Percentile	Min	Max	N	Mean	SD	Median	25th Percentile	75th Percentile	Min	Max
Controls (ctrl)		0	10	0.62	0.48	0.38	0.35	0.55	0.35	1.51	10	0.72	0.64	0.35	0.35	0.69	0.35	1.96
Vinclo+Flut+Prochl.	0.005+0.00025+0.01	1	9	0.48	0.29	0.35	0.35	0.45	0.35	1.23	9	0.59	0.47	0.35	0.35	0.66	0.35	1.76
	0.005+0.00025+0.01/ctrl			0.77		0.92								1.00				
	4+0.025+5	2	9	0.53	0.26	0.35	0.35	0.69	0.35	1.07	9	0.64	0.42	0.35	0.35	0.82	0.35	1.58
	4+0.025+5/ctrl			0.85		0.92								1.00				
	20+0.25+30	3	9	0.54	0.54	0.35	0.35	0.37	0.35	1.97	9	0.67	0.89	0.35	0.35	0.36	0.35	3.03
	20+0.25+30/ctrl			0.87		0.92								1.00				

Kruskal-Wallis test + Mann-Whitney-U-test (two-sided), + $p \leq 0.05$, ++ $p \leq 0.01$
Vinclo = vinclozolin; Flut = flutamide; Prochl = prochloraz

Males PND 21

Compound	Dose (mg/kg bw/d)	Group	Progesterone (nmol/L)								Corticosterone (nmol/L)							
			N	Mean	SD	Median	25th Percentile	75th Percentile	Min	Max	N	Mean	SD	Median	25th Percentile	75th Percentile	Min	Max
Controls (ctrl)		0	10	6.96	3.18	6.81	4.79	7.49	2.97	13.86	10	811.2	106.8	851.1	730.4	883.6	638.7	936.4
Vinclo+Flut+Prochl.	0.005+0.00025+0.01	1	9	5.57	3.96	3.78	2.61	9.54	1.41	11.03	9	673.7	146.1	647.4	621.4	794.8	436.4	864.1 +
	0.005+0.00025+0.01/ctrl					0.56								0.8				
	4+0.025+5	2	9	3.4	1.55	3.37	1.78	4.42	1.35	5.57 ++	9	739.5	153.5	710.9	641.6	823.7	500.0	971.0
	4+0.025+5/ctrl					0.49								0.8				
	20+0.25+30	3	9	2.44	1.03	2.04	1.59	3.40	1.40	3.91 ++	9	689.1	93.0	702.3	601.1	731.2	572.2	823.7 ++
	20+0.25+30/ctrl					0.30								0.8				

Kruskal-Wallis test + Mann-Whitney-U-test (two-sided), + $p \leq 0.05$, ++ $p \leq 0.01$
Vinclo = vinclozolin; Flut = flutamide; Prochl = prochloraz

Males PND 21

Compound	Dose (mg/kg bw/d)	Group	Cortisol (nmol/L)							
			N	Mean	SD	Median	25th Percentile	75th Percentile	Min	Max
Controls (ctrl)		0	10	0.79	0.3	0.83	0.61	1.01	0.28	1.18
Vinclo+Flut+Prochl.	0.005+0.00025+0.01	1	8	0.56	0.39	0.43	0.37	0.54	0.30	1.50
	0.005+0.00025+0.01/ctrl					0.52				
	4+0.025+5	2	9	0.8	0.42	0.62	0.52	0.88	0.31	1.62
	4+0.025+5/ctrl					0.75				
	20+0.25+30	3	9	0.7	0.32	0.61	0.59	0.78	0.28	1.41
	20+0.25+30/ctrl					0.73				

Kruskal-Wallis test + Mann-Whitney-U-test (two-sided), + $p \leq 0.05$, ++ $p \leq 0.01$
Vinclo = vinclozolin; Flut = flutamide; Prochl = prochloraz

Males PND 21

Compound	Dose (mg/kg bw/d)	Group	Androstenedione (nmol/L)								Testosterone (nmol/L)							
			N	Mean	SD	Median	25th Percentile	75th Percentile	Min	Max	N	Mean	SD	Median	25th Percentile	75th Percentile	Min	Max
Controls		0	10	0.62	0.48	0.38	0.35	0.55	0.35	1.51	10	0.72	0.64	0.35	0.35	0.69	0.35	1.96
Flutamide	0.00025	4	10	0.94	1.21	0.40	0.35	0.77	0.35	4.11	10	0.90	0.98	0.35	0.35	0.92	0.35	3.07
	0.00025/ctrl			1.52		1.05								1.00				

Mann-Whitney-U-test (two-sided), + p ≤ 0.05, ++ p ≤ 0.01

Males PND 21

Compound	Dose (mg/kg bw/d)	Group	Progesterone (nmol/L)								Corticosterone (nmol/L)							
			N	Mean	SD	Median	25th Percentile	75th Percentile	Min	Max	N	Mean	SD	Median	25th Percentile	75th Percentile	Min	Max
Controls		0	10	6.96	3.18	6.81	4.79	7.49	2.97	13.86	10	811.2	106.8	851.1	730.4	883.6	638.7	936.4
Flutamide	0.00025	4	10	5.04	3.36	4.40	3.23	5.83	0.84	13.45	10	743.6	102.3	784.6	638.0	815.0	615.6	881.5
	0.00025/ctrl					0.65								0.9				

Mann-Whitney-U-test (two-sided), + p ≤ 0.05, ++ p ≤ 0.01

Males PND 21

Compound	Dose (mg/kg bw/d)	Group	Cortisol (nmol/L)							
			N	Mean	SD	Median	25th Percentile	75th Percentile	Min	Max
Controls		0	10	0.79	0.3	0.83	0.61	1.01	0.28	1.18
Flutamide	0.00025	4	10	0.98	0.61	0.81	0.53	1.27	0.28	2.00
	0.00025/ctrl					0.98				

Mann-Whitney-U-test (two-sided), + p ≤ 0.05, ++ p ≤ 0.01

Females PND21

Compound	Dose (mg/kg bw/d)	Group	Androstenedione (nmol/L) N	Mean	SD	Median	25th Percentile	75th Percentile	Min	Max	Progesterone (nmol/L) N	Mean	SD	Median	25th Percentile	75th Percentile	Min	Max
Controls (ctrl)		0	10	0.35	0.02	0.35	0.35	0.35	0.35	0.42	9	5.52	2.5	5.37	4.01	7.22	2.10	9.03
Vinclo+Flut+Prochl.	0.005+0.00025+0.01	1	10	0.82	1.42	0.35	0.35	0.38	0.35	4.86	9	5.20	2.15	4.83	4.55	7.22	1.37	8.17
	0.005+0.00025+0.01/ctrl			2.34		1.43								0.90				
	4+0.025+5	2	10	0.57	0.44	0.35	0.35	0.54	0.35	1.66	10	4.00	1.79	4.02	2.73	4.59	1.41	7.79
	4+0.025+5/ctrl			1.63		1.00								0.75				
	20+0.25+30	3	10	0.36	0.04	0.35	0.35	0.35	0.35	0.48	10	4.02	1.99	4.09	2.90	5.03	0.57	7.85
	20+0.25+30/ctrl			1.03		1.00								0.76				

Kruskal-Wallis test + Mann-Whitney-U-test (two-sided), + p ≤ 0.05, ++ p ≤ 0.01
Vinclo = vinclozolin; Flut = flutamide; Prochl = prochloraz

Females PND21

Compound	Dose (mg/kg bw/d)	Group	Corticosterone (nmol/L) N	Mean	SD	Median	25th Percentile	75th Percentile	Min	Max	Cortisol (nmol/L) N	Mean	SD	Median	25th Percentile	75th Percentile	Min	Max
Controls (ctrl)		0	10	802.6	211.4	751.4	616.3	980.4	572.2	1161.8	10	1.02	0.51	1.07	0.70	1.27	0.28	1.75
Vinclo+Flut+Prochl.	0.005+0.00025+0.01	1	10	756.6	106.3	758.6	700.1	844.6	589.6	893.0	9	0.59	0.33	0.48	0.43	0.59	0.28	1.24
	0.005+0.00025+0.01/ctrl					1.0								0.45				
	4+0.025+5	2	10	762.1	114.08	765.9	729.0	846.0	485.5	869.9	10	0.77	0.56	0.50	0.40	0.92	0.30	1.96
	4+0.025+5/ctrl					1.0								0.47				
	20+0.25+30	3	10	689.6	189.2	716.7	552.0	842.4	387.3	904.6	10	0.58	0.31	0.55	0.35	0.63	0.28	1.18 +
	20+0.25+30/ctrl					1.0								0.51				

Kruskal-Wallis test + Mann-Whitney-U-test (two-sided), + p ≤ 0.05, ++ p ≤ 0.01
Vinclo = vinclozolin; Flut = flutamide; Prochl = prochloraz

Females PND 21

Compound	Dose (mg/kg bw/d)	Group	Androstenedione (nmol/L) N	Mean	SD	Median	25th Percentile	75th Percentile	Min	Max	Progesterone (nmol/L) N	Mean	SD	Median	25th Percentile	75th Percentile	Min	Max
Controls		0	10	0.35	0.02	0.35	0.35	0.35	0.35	0.42	9	5.52	2.50	5.37	4.01	7.22	2.10	9.03
Flutamide	0.00025	4	10	0.54	0.43	0.38	0.35	0.51	0.35	1.75 +	10	5.58	2.74	5.28	3.89	6.47	1.34	10.40
	0.00025/ctrl			1.54		1.09								0.98				

Mann-Whitney-U-test (two-sided), + p ≤ 0.05, ++ p ≤ 0.01

Females PND 21

Compound	Dose (mg/kg bw/d)	Group	Corticosterone (nmol/L) N	Mean	SD	Median	25th Percentile	75th Percentile	Min	Max	Cortisol (nmol/L) N	Mean	SD	Median	25th Percentile	75th Percentile	Min	Max
Controls		0	10	802.6	211.4	751.4	616.3	980.4	572.2	1161.8	10	1.02	0.51	1.07	0.70	1.27	0.28	1.75
Flutamide	0.00025	4	10	781.7	134.7	761.5	685.7	856.9	589.6	988.4	9	0.65	0.31	0.64	0.54	0.71	0.28	1.39
	0.00025/ctrl					1.0								0.60				

Mann-Whitney-U-test (two-sided), + p ≤ 0.05, ++ p ≤ 0.01

Males in sexual maturity

Compound	Dose (mg/kg bw/d)	Group	Androstenedione (nmol/L) N	Mean	SD	Median	25th Percentile	75th Percentile	Min	Max
Controls (ctrl)		0	10	0.65	0.54	0.40	0.35	0.64	0.35	2.06
Vinclo+Flut+Prochl.	0.005+0.00025+0.01	1	10	0.60	0.29	0.54	0.35	0.74	0.35	1.16
	0.005+0.00025+0.01/ctrl			0.92		1.35				
	4+0.025+5	2	10	0.57	0.27	0.54	0.35	0.64	0.35	1.21
	4+0.025+5/ctrl			0.88		1.35				
	20+0.25+30	3	10	0.62	0.31	0.52	0.35	0.89	0.35	1.19
	20+0.25+30/ctrl			0.95		1.30				

Compound	Dose (mg/kg bw/d)	Group	Testosterone (nmol/L) N	Mean	SD	Median	25th Percentile	75th Percentile	Min	Max
Controls (ctrl)		0	10	3.06	3.06	1.74	1.05	4.03	0.54	10.13
Vinclo+Flut+Prochl.	0.005+0.00025+0.01	1	10	2.96	1.81	3.52	1.18	4.29	0.64	5.31
	0.005+0.00025+0.01/ctrl					2.02				
	4+0.025+5	2	9	2.77	1.69	2.70	1.25	3.85	0.60	5.79
	4+0.025+5/ctrl					1.55				
	20+0.25+30	3	10	3.94	2.03	3.41	2.28	6.06	1.39	6.52
	20+0.25+30/ctrl					1.96				

Kruskal-Wallis test + Mann-Whitney-U-test (two-sided), + p ≤ 0.05, ++ p ≤ 0.01
Vinclo = vinclozolin; Flut = flutamide; Prochl = prochloraz

Males in sexual maturity

Compound	Dose (mg/kg bw/d)	Group	Progesterone (nmol/L) N	Mean	SD	Median	25th Percentile	75th Percentile	Min	Max
Controls (ctrl)		0	10	1.95	1.60	1.56	0.56	3.15	0.32	4.80
Vinclo+Flut+Prochl.	0.005+0.00025+0.01	1	10	3.89	4.22	2.52	0.91	4.52	0.75	13.64
	0.005+0.00025+0.01/ctrl					1.62				
	4+0.025+5	2	10	3.03	3.41	2.23	0.79	3.30	0.32	11.67
	4+0.025+5/ctrl					1.43				
	20+0.25+30	3	10	2.34	2.58	1.62	0.40	3.48	0.32	8.55
	20+0.25+30/ctrl					1.04				

Compound	Dose (mg/kg bw/d)	Group	11-Deoxycorticosterone (nmol/L) N	Mean	SD	Median	25th Percentile	75th Percentile	Min	Max
Controls (ctrl)		0	10	2.43	1.01	2.30	1.59	2.51	1.59	4.58
Vinclo+Flut+Prochl.	0.005+0.00025+0.01	1	10	3.81	2.95	3.02	1.64	4.77	1.59	11.10
	0.005+0.00025+0.01/ctrl					1.31				
	4+0.025+5	2	10	3.21	1.51	3.06	1.88	3.90	1.59	5.69
	4+0.025+5/ctrl					1.33				
	20+0.25+30	3	10	2.92	1.97	1.75	1.59	3.51	1.59	7.09
	20+0.25+30/ctrl					0.76				

Kruskal-Wallis test + Mann-Whitney-U-test (two-sided), + p ≤ 0.05, ++ p ≤ 0.01
Vinclo = vinclozolin; Flut = flutamide; Prochl = prochloraz

Males in sexual maturity

Compound	Dose (mg/kg bw/d)	Group	Corticosterone (nmol/L) N	Mean	SD	Median	25th Percentile	75th Percentile	Min	Max
Controls (ctrl)		0	10	466.8	335.5	504.3	125.0	789.7	82.1	887.2
Vinclo+Flut+Prochl.	0.005+0.00025+0.01	1	10	635.9	274.2	667.6	465.3	847.5	137.0	1028.8
	0.005+0.00025+0.01/ctrl					1.3				
	4+0.025+5	2	10	476.0	273.3	554.9	340.3	668.3	26.7	838.1
	4+0.025+5/ctrl					1.1				
	20+0.25+30	3	10	428.8	358.1	343.9	120.6	713.1	43.6	1075.1
	20+0.25+30/ctrl					0.7				

Kruskal-Wallis test + Mann-Whitney-U-test (two-sided), + p ≤ 0.05, ++ p ≤ 0.01
Vinclo = vinclozolin; Flut = flutamide; Prochl = prochloraz

Males in sexual maturity

Compound	Dose (mg/kg bw/d)	Group	Androstenedione (nmol/L) N	Mean	SD	Median	25th Percentile	75th Percentile	Min	Max	Testosterone (nmol/L) N	Mean	SD	Median	25th Percentile	75th Percentile	Min	Max
Controls		0	10	0.65	0.54	0.40	0.35	0.64	0.35	2.06	10	3.06	3.06	1.74	1.05	4.03	0.54	10.13
Flutamide	0.00025	4	10	0.46	0.16	0.35	0.35	0.57	0.35	0.77	10	1.94	1.32	1.78	0.84	2.47	0.35	4.27
	0.00025/ctrl			0.71		0.88								1.02				

Mann-Whitney-U-test (two-sided), + p ≤ 0.05, ++ p ≤ 0.01

Males in sexual maturity

Compound	Dose (mg/kg bw/d)	Group	Progesterone (nmol/L) N	Mean	SD	Median	25th Percentile	75th Percentile	Min	Max	11-Deoxycorticosterone (nmol/L) N	Mean	SD	Median	25th Percentile	75th Percentile	Min	Max
Controls		0	10	1.95	1.60	1.56	0.56	3.15	0.32	4.80	10	2.43	1.01	2.30	1.59	2.51	1.59	4.58
Flutamide	0.00025	4	10	5.51	12.77	1.02	0.47	3.21	0.32	41.66	10	3.73	4.86	1.72	1.59	2.85	1.59	17.20
	0.00025/ctrl					0.65								0.75				

Mann-Whitney-U-test (two-sided), + p ≤ 0.05, ++ p ≤ 0.01

Males in sexual maturity

Compound	Dose (mg/kg bw/d)	Group	Corticosterone (nmol/L) N	Mean	SD	Median	25th Percentile	75th Percentile	Min	Max
Controls		0	10	466.8	335.5	504.3	125.0	789.7	82.1	887.2
Flutamide	0.00025	4	10	503.4	353.5	391.6	289.7	713.8	18.8	1265.8
	0.00025/ctrl					0.8				

Mann-Whitney-U-test (two-sided), + p ≤ 0.05, ++ p ≤ 0.01

Females in sexual maturity

Compound	Dose (mg/kg bw/d)	Group	Androstenedione (nmol/L) N	Mean	SD	Median	25th Percentile	75th Percentile	Min	Max
Controls (ctrl)		0	10	0.36	0.04	0.35	0.35	0.35	0.35	0.49
Vinclo+Flut+Prochl.	0.005+0.00025+0.01	1	10	0.35	0.01	0.35	0.35	0.35	0.35	0.36
	0.005+0.00025+0.01/ctrl			0.97		1.00				
	4+0.025+5	2	10	0.36	0.03	0.35	0.35	0.35	0.35	0.42
	4+0.025+5/ctrl			1.00		1.00				
	20+0.25+30	3	10	0.44	0.13	0.39	0.35	0.52	0.35	0.72
	20+0.25+30/ctrl			1.22		1.11				

Compound	Dose (mg/kg bw/d)	Group	Testosterone (nmol/L) N	Mean	SD	Median	25th Percentile	75th Percentile	Min	Max
Controls (ctrl)		0	9	0.36	0.05	0.35	0.35	0.35	0.35	0.50
Vinclo+Flut+Prochl.	0.005+0.00025+0.01	1	10	0.35	0.01	0.35	0.35	0.35	0.35	0.37
	0.005+0.00025+0.01/ctrl					1.00				
	4+0.025+5	2	10	0.35	0	0.35	0.35	0.35	0.35	0.35
	4+0.025+5/ctrl					1.00				
	20+0.25+30	3	10	0.35	0	0.35	0.35	0.35	0.35	0.35
	20+0.25+30/ctrl					1.00				

Kruskal-Wallis test + Mann-Whitney-U-test (two-sided), + p ≤ 0.05, ++ p ≤ 0.01
Vinclo = vinclozolin; Flut = flutamide; Prochl = prochloraz

Females in sexual maturity

Compound	Dose (mg/kg bw/d)	Group	Progesterone (nmol/L) N	Mean	SD	Median	25th Percentile	75th Percentile	Min	Max
Controls (ctrl)		0	10	9.93	3.12	10.08	8.35	11.53	3.69	15.49
Vinclo+Flut+Prochl.	0.005+0.00025+0.01	1	10	9.85	6.57	9.08	6.27	11.15	1.20	21.15
	0.005+0.00025+0.01/ctrl					0.90				
	4+0.025+5	2	10	11.74	10.48	10.08	3.02	17.04	0.45	30.40
	4+0.025+5/ctrl					1.00				
	20+0.25+30	3	9	8.22	7.91	4.77	2.85	12.21	0.43	22.61
	20+0.25+30/ctrl					0.47				

Compound	Dose (mg/kg bw/d)	Group	11-Deoxycorticosterone (nmol/L) N	Mean	SD	Median	25th Percentile	75th Percentile	Min	Max
Controls (ctrl)		0	10	2.06	0.98	1.59	1.59	1.66	1.59	4.20
Vinclo+Flut+Prochl.	0.005+0.00025+0.01	1	10	1.91	0.83	1.59	1.59	1.59	1.59	4.20
	0.005+0.00025+0.01/ctrl					1.00				
	4+0.025+5	2	10	2.3	1.28	1.59	1.59	2.31	1.59	4.80
	4+0.025+5/ctrl					1.00				
	20+0.25+30	3	10	3.02	2.4	1.79	1.59	3.37	1.59	8.55
	20+0.25+30/ctrl					1.13				

Kruskal-Wallis test + Mann-Whitney-U-test (two-sided), + p ≤ 0.05, ++ p ≤ 0.01
Vinclo = vinclozolin; Flut = flutamide; Prochl = prochloraz

Females in sexual maturity

Compound	Dose (mg/kg bw/d)	Group	Corticosterone (nmol/L) N	Mean	SD	Median	25th Percentile	75th Percentile	Min	Max
Controls (ctrl)		0	10	343.7	417.6	142.9	57.5	474.7	13.9	1132.9
Vinclo+Flut+Prochl.	0.005+0.00025+0.01	1	10	324.0	324.6	191.8	48.0	606.9	18.5	858.3
	0.005+0.00025+0.01/ctrl					1.3				
	4+0.025+5	2	10	463.2	572.1	160.0	74.1	900.4	46.2	1557.7
	4+0.025+5/ctrl					1.1				
	20+0.25+30	3	10	636.8	568.2	450.8	130.8	1179.1	28.7	1456.6
	20+0.25+30/ctrl					3.2				

Kruskal-Wallis test + Mann-Whitney-U-test (two-sided), + p ≤ 0.05, ++ p ≤ 0.01
Vinclo = vinclozolin; Flut = flutamide; Prochl = prochloraz

Females in sexual maturity

Compound	Dose (mg/kg bw/d)	Group	Androstenedione (nmol/L)							
			N	Mean	SD	Median	25th Percentile	75th Percentile	Min	Max
Controls		0	10	0.36	0.04	0.35	0.35	0.35	0.35	0.49
Flutamide	0.00025	4	10	0.38	0.11	0.35	0.35	0.35	0.35	0.70
	0.00025/ctrl			1.06		1.00				

Compound	Dose (mg/kg bw/d)	Group	Testosterone (nmol/L)							
			N	Mean	SD	Median	25th Percentile	75th Percentile	Min	Max
Controls		0	9	0.36	0.05	0.35	0.35	0.35	0.35	0.50
Flutamide	0.00025	4	8	0.35	0.00	0.35	0.35	0.35	0.35	0.35
	0.00025/ctrl					1.00				

Mann-Whitney-U-test (two-sided), + p ≤ 0.05, ++ p ≤ 0.01

Females in sexual maturity

Compound	Dose (mg/kg bw/d)	Group	Progesterone (nmol/L)							
			N	Mean	SD	Median	25th Percentile	75th Percentile	Min	Max
Controls		0	10	9.93	3.12	10.08	8.35	11.53	3.69	15.49
Flutamide	0.00025	4	10	9	8.02	8.75	3.19	10.72	0.91	29.10
	0.00025/ctrl					0.87				

Compound	Dose (mg/kg bw/d)	Group	11-Deoxycorticosterone (nmol/L)							
			N	Mean	SD	Median	25th Percentile	75th Percentile	Min	Max
Controls		0	10	2.06	0.98	1.59	1.59	1.66	1.59	4.20
Flutamide	0.00025	4	10	2.24	1.87	1.59	1.59	1.59	1.59	7.54
	0.00025/ctrl					1.00				

Mann-Whitney-U-test (two-sided), + p ≤ 0.05, ++ p ≤ 0.01

Females in sexual maturity

Compound	Dose (mg/kg bw/d)	Group	Corticosterone (nmol/L)							
			N	Mean	SD	Median	25th Percentile	75th Percentile	Min	Max
Controls		0	10	343.7	417.6	142.9	57.5	474.7	13.9	1132.9
Flutamide	0.00025	4	10	381	377.8	285.2	108.2	461.0	32.1	1144.4
	0.00025/ctrl					2.0				

Mann-Whitney-U-test (two-sided), + p ≤ 0.05, ++ p ≤ 0.01

Males PND 83

Compound	Dose (mg/kg bw/d)	Group	Androstenedione (nmol/L)								Testosterone (nmol/L)							
			N	Mean	SD	Median	25th Percentile	75th Percentile	Min	Max	N	Mean	SD	Median	25th Percentile	75th Percentile	Min	Max
Controls (ctrl)		0	8	1.15	0.84	0.88	0.51	1.72	0.37	2.70	8	6.48	4.76	5.00	3.01	9.21	2.01	15.68
Vinclo+Flut+Prochl.	0.005+0.00025+0.01	1	10	1.29	1.38	0.78	0.48	1.22	0.35	4.86	10	5.69	5.16	4.08	2.38	6.80	1.49	18.70
	0.005+0.00025+0.01/ctrl			1.12		0.89								0.82				
	4+0.025+5	2	10	1.06	0.95	0.77	0.51	1.00	0.36	3.45	10	3.92	2.07	3.44	2.08	4.75	1.87	8.19
	4+0.025+5/ctrl			0.92		0.88								0.69				
	20+0.25+30	3	10	2.05	1.52	1.63	1.36	2.24	0.59	5.87	10	6.37	4.24	4.98	3.11	8.91	1.85	13.74
	20+0.25+30/ctrl			1.78		1.85								1.00				

Kruskal-Wallis test + Mann-Whitney-U-test (two-sided), + $p \le 0.05$, ++ $p \le 0.01$
Vinclo = vinclozolin; Flut = flutamide; Prochl = prochloraz

Males PND 83

Compound	Dose (mg/kg bw/d)	Group	Progesterone (nmol/L)								11-Deoxycorticosterone (nmol/L)							
			N	Mean	SD	Median	25th Percentile	75th Percentile	Min	Max	N	Mean	SD	Median	25th Percentile	75th Percentile	Min	Max
Controls (ctrl)		0	8	2.06	1.7	1.63	0.61	3.37	0.43	4.74	8	7.39	6.13	5.04	2.89	11.52	1.59	18.03
Vinclo+Flut+Prochl.	0.005+0.00025+0.01	1	10	4.93	4.37	3.79	1.24	7.46	0.68	13.71	10	9.63	8.09	7.55	3.45	12.81	2.55	28.37
	0.005+0.00025+0.01/ctrl					2.33								1.50				
	4+0.025+5	2	10	2.28	1.95	1.79	0.97	3.00	0.32	6.23	10	8.57	7.05	5.44	3.09	13.32	1.59	21.34
	4+0.025+5/ctrl					1.10								1.08				
	20+0.25+30	3	10	2.76	2.39	2.07	1.24	2.72	0.39	7.47	10	8.06	6.29	5.88	3.25	11.25	1.59	19.59
	20+0.25+30/ctrl					1.27								1.17				

Kruskal-Wallis test + Mann-Whitney-U-test (two-sided), + $p \le 0.05$, ++ $p \le 0.01$
Vinclo = vinclozolin; Flut = flutamide; Prochl = prochloraz

Males PND 83

Compound	Dose (mg/kg bw/d)	Group	Corticosterone (nmol/L)							
			N	Mean	SD	Median	25th Percentile	75th Percentile	Min	Max
Controls (ctrl)		0	8	434.5	250.3	395.9	252.1	621.4	141.0	812.1
Vinclo+Flut+Prochl.	0.005+0.00025+0.01	1	10	674.1	457.7	593.9	250.2	1088.1	176.3	1378.5
	0.005+0.00025+0.01/ctrl					1.5				
	4+0.025+5	2	10	564.1	339.7	465.3	324.4	812.8	145.7	1144.4
	4+0.025+5/ctrl					1.2				
	20+0.25+30	3	10	568.4	414.6	426.3	221.5	792.6	174.0	1358.3
	20+0.25+30/ctrl					1.1				

Kruskal-Wallis test + Mann-Whitney-U-test (two-sided), + $p \le 0.05$, ++ $p \le 0.01$
Vinclo = vinclozolin; Flut = flutamide; Prochl = prochloraz

Males PND 83

Compound	Dose (mg/kg bw/d)	Group	Androstenedione (nmol/L)							
			N	Mean	SD	Median	25th Percentile	75th Percentile	Min	Max
Controls		0	8	1.15	0.84	0.88	0.51	1.72	0.37	2.70
Flutamide	0.00025	4	10	1.09	1.07	0.81	0.38	1.14	0.35	3.83
	0.00025/ctrl			0.95		0.92				

Compound	Dose (mg/kg bw/d)	Group	Testosterone (nmol/L)							
			N	Mean	SD	Median	25th Percentile	75th Percentile	Min	Max
Controls		0	8	6.48	4.76	5.00	3.01	9.21	2.01	15.68
Flutamide	0.00025	4	10	6.40	6.84	4.35	1.93	7.22	1.03	23.91
	0.00025/ctrl					0.87				

Mann-Whitney-U-test (two-sided), + p ≤ 0.05, ++ p ≤ 0.01

Males PND 83

Compound	Dose (mg/kg bw/d)	Group	Progesterone (nmol/L)							
			N	Mean	SD	Median	25th Percentile	75th Percentile	Min	Max
Controls		0	8	2.06	1.70	1.63	0.61	3.37	0.43	4.74
Flutamide	0.00025	4	10	5.19	4.7	3.75	3.09	4.49	0.32	16.25
	0.00025/ctrl					2.30				

Compound	Dose (mg/kg bw/d)	Group	11-Deoxycorticosterone (nmol/L)							
			N	Mean	SD	Median	25th Percentile	75th Percentile	Min	Max
Controls		0	8	7.39	6.13	5.04	2.89	11.52	1.59	18.03
Flutamide	0.00025	4	10	13.93	9.36	11.75	7.03	18.27	1.59	30.56
	0.00025/ctrl					2.33				

Mann-Whitney-U-test (two-sided), + p ≤ 0.05, ++ p ≤ 0.01

Males PND 83

Compound	Dose (mg/kg bw/d)	Group	Corticosterone (nmol/L)							
			N	Mean	SD	Median	25th Percentile	75th Percentile	Min	Max
Controls		0	8	434.5	250.3	395.9	252.1	621.4	141.0	812.1
Flutamide	0.00025	4	10	716	375.8	752.8	466.0	1001.4	73.4	1239.8
	0.00025/ctrl					1.9				

Mann-Whitney-U-test (two-sided), + p ≤ 0.05, ++ p ≤ 0.01

Females PND 83

Compound	Dose (mg/kg bw/d)	Group	Androstenedione (nmol/L)								Testosterone (nmol/L)							
			N	Mean	SD	Median	25th Percentile	75th Percentile	Min	Max	N	Mean	SD	Median	25th Percentile	75th Percentile	Min	Max
Controls (ctrl)		0	9	1.87	1.28	1.43	0.83	3.17	0.56	4.04	9	0.47	0.16	0.39	0.35	0.54	0.35	0.76
Vinclo+Flut+Prochl.	0.005+0.00025+0.01	1	10	1.61	0.89	1.39	1.02	1.93	0.55	3.17	10	0.39	0.07	0.35	0.35	0.40	0.35	0.52
	0.005+0.00025+0.01/ctrl			0.86		0.97								0.90				
	4+0.025+5	2	10	1.86	2.54	0.81	0.47	2.02	0.35	8.56	10	0.46	0.34	0.35	0.35	0.35	0.35	1.42
	4+0.025+5/ctrl			0.99		0.57								0.90				
	20+0.25+30	3	10	6.29	7.58	2.52	1.52	7.33	1.02	21.42	10	0.88	0.79	0.46	0.37	1.12	0.35	2.58
	20+0.25+30/ctrl			3.36		1.76								1.18				

Kruskal-Wallis test + Mann-Whitney-U-test (two-sided), + p ≤ 0.05, ++ p ≤ 0.01
Vinclo = vinclozolin; Flut = flutamide; Prochl = prochloraz

Females PND 83

Compound	Dose (mg/kg bw/d)	Group	Progesterone (nmol/L)								11-Deoxycorticosterone (nmol/L)							
			N	Mean	SD	Median	25th Percentile	75th Percentile	Min	Max	N	Mean	SD	Median	25th Percentile	75th Percentile	Min	Max
Controls (ctrl)		0	9	40.27	60.15	10.53	7.60	54.38	4.80	190.80	9	4.90	4.62	1.73	1.59	10.56	1.59	11.26
Vinclo+Flut+Prochl.	0.005+0.00025+0.01	1	10	20.25	28.40	9.83	7.32	19.13	3.34	98.58	10	10.22	13.79	3.66	2.13	10.75	1.59	42.61
	0.005+0.00025+0.01/ctrl					0.93								2.12				
	4+0.025+5	2	10	33.66	51.53	13.28	9.06	20.26	6.42	171.72	10	9.32	7.43	5.57	3.70	17.50	1.59	19.21
	4+0.025+5/ctrl					1.26								3.22				
	20+0.25+30	3	10	58.93	70.64	18.29	11.15	110.35	9.51	192.71	10	14.83	14.16	11.69	9.68	13.96	2.97	53.42 ++
	20+0.25+30/ctrl					1.74								6.76				

Kruskal-Wallis test + Mann-Whitney-U-test (two-sided), + p ≤ 0.05, ++ p ≤ 0.01
Vinclo = vinclozolin; Flut = flutamide; Prochl = prochloraz

Females PND 83

Compound	Dose (mg/kg bw/d)	Group	Corticosterone (nmol/L)							
			N	Mean	SD	Median	25th Percentile	75th Percentile	Min	Max
Controls (ctrl)		0	9	460.1	491.3	225.7	72.3	930.6	35.3	1286.1
Vinclo+Flut+Prochl.	0.005+0.00025+0.01	1	10	701.2	609.7	354.0	261.2	1083.8	178.6	1875.6
	0.005+0.00025+0.01/ctrl					1.6				
	4+0.025+5	2	10	750.6	540.8	682.0	311.5	1210.2	64.2	1525.9
	4+0.025+5/ctrl					3.0				
	20+0.25+30	3	10	1083.2	488.0	1170.5	838.8	1513.6	326.6	1653.1 ++
	20+0.25+30/ctrl					5.2				

Kruskal-Wallis test + Mann-Whitney-U-test (two-sided), + p ≤ 0.05, ++ p ≤ 0.01
Vinclo = vinclozolin; Flut = flutamide; Prochl = prochloraz

Project 60R0375/88R002

Females PND 83

Compound	Dose (mg/kg bw/d)	Group	Androstenedione (nmol/L)									Testosterone (nmol/L)								
			N	Mean	SD	Median	25th Percentile	75th Percentile	Min	Max		N	Mean	SD	Median	25th Percentile	75th Percentile	Min	Max	
Controls		0	9	1.87	1.28	1.43	0.83	3.17	0.56	4.04		9	0.47	0.16	0.39	0.35	0.54	0.35	0.76	
Flutamide	0.00025	4	10	2.42	1.36	2.00	1.28	3.31	0.90	4.76		10	0.45	0.12	0.41	0.35	0.56	0.35	0.61	
	0.00025/ctrl			1.29		1.40									1.05					

Mann-Whitney-U-test (two-sided), + p ≤ 0.05, ++ p ≤ 0.01

Females PND 83

Compound	Dose (mg/kg bw/d)	Group	Progesterone (nmol/L)									11-Deoxycorticosterone (nmol/L)								
			N	Mean	SD	Median	25th Percentile	75th Percentile	Min	Max		N	Mean	SD	Median	25th Percentile	75th Percentile	Min	Max	
Controls		0	9	40.27	60.15	10.53	7.60	54.38	4.80	190.80		9	4.90	4.62	1.73	1.59	10.56	1.59	11.26	
Flutamide	0.00025	4	10	28.05	26.12	16.89	14.12	26.03	5.15	77.27		10	17.53	10.18	14.74	11.47	26.57	2.53	31.20	++
	0.00025/ctrl					1.60									8.52					

Mann-Whitney-U-test (two-sided), + p ≤ 0.05, ++ p ≤ 0.01

Females PND 83

Compound	Dose (mg/kg bw/d)	Group	Corticosterone (nmol/L)								
			N	Mean	SD	Median	25th Percentile	75th Percentile	Min	Max	
Controls		0	9	460.1	491.3	225.7	72.3	930.6	35.3	1286.1	
Flutamide	0.00025	4	10	1109.5	343.4	1158.9	1021.6	1281.7	274.8	1497.0	++
	0.00025/ctrl					5.1					

Mann-Whitney-U-test (two-sided), + p ≤ 0.05, ++ p ≤ 0.01

Project No.: 60R0375/88R002

Testosterone in testis culture, fetus GD 20

Mean testosterone of both testes

		Testosterone (nmol/g testis)
Group 0	N	24
	mean	19.1
	SD	3.6
	median	19.4
Group 1	N	32
	mean	19.7
	SD	6.5
	median	20.6
Group 2	N	16
	mean	18.8
	SD	3.6
	median	19.3
Group 3	N	26
	mean	****15.8**
	SD	4.6
	median	16.0
Group 4	N	24
	mean	18.7
	SD	5.8
	median	18.6

Kruskal-Wallis Test (Group 0 - 3, only) + Mann-Whitney U Test, two-sided: * $p < 0.05$; ** $p < 0.01$

Reproductive Toxicity Study to detect potential effects to anti-androgenic substances in Wistar Rats (Gavage) 28.Mar.2014 SIGR

ABSOLUTE WEIGHTS - MEAN VALUES
COMPARISON OF GROUP 0 WITH GROUPS 1,2 AND 3 (MIX)
PARENTAL FEMALES

Sacrifice			F1			
Sex			F			
Group			0	1	2	3
Terminal body weight	g	M	231.23	236.437	231.87	234.953
		% dev	100	102	100	102
		SD	15.499	16.024	15.524	13.556
		n	20	19	20	19
Adrenal glands	mg	M	70.75	72.158	74.9	82.789**
		% dev	100	102	106	117
		SD	8.583	11.102	9.894	13.138
		n	20	19	20	19
Brain	g	M	1.862	1.908	1.88	1.884
		% dev	100	102	101	101
		SD	0.07	0.065	0.063	0.054
		n	20	19	20	19
Kidneys	g	M	1.616	1.641	1.633	1.74 **
		% dev	100	102	101	108
		SD	0.125	0.142	0.088	0.117
		n	20	19	20	19
Liver	g	M	7.46	7.352	7.358	9.066**
		% dev	100	99	99	122
		SD	0.547	0.892	0.472	0.884
		n	20	19	20	19
Ovaries	mg	M	115.35	122.211	124.05	131.368
		% dev	100	106	108	114
		SD	18.933	15.292	21.951	23.726
		n	20	19	20	19
Pituitary gland	mg	M	11.85	13.474	12.8	12.0
		% dev	100	114	108	101
		SD	1.348	2.458	1.881	1.528
		n	20	19	20	19
Spleen	g	M	0.448	0.481	0.463	0.481
		% dev	100	107	103	107
		SD	0.074	0.068	0.083	0.052
		n	20	19	20	19
Thyroid glands	mg	M	18.85	19.632	19.65	20.947
		% dev	100	104	104	111
		SD	3.066	4.072	3.856	4.196
		n	20	19	20	19

*: P <= 0.05, **: P <= 0.01
Kruskal-Wallis H and Wilcoxon test, two sided

ABSOLUTE WEIGHTS - MEAN VALUES
COMPARISON OF GROUP 0 WITH GROUPS 1,2 AND 3 (MIX)
PARENTAL FEMALES

Sacrifice			F1			
Sex			F			
Group			0	1	2	3
Uterus	g	M	0.709	0.693	0.633	0.682
		% dev	100	98	89	96
		SD	0.276	0.242	0.193	0.173
		n	20	19	20	19

*: P <= 0.05, **: P <= 0.01
Kruskal-Wallis H and Wilcoxon test, two sided

Reproductive Toxicity Study to detect potential effects to anti-androgenic substances in Wistar Rats (Gavage) 28.Mar.2014 SIGR

ABSOLUTE WEIGHTS - MEAN VALUES
COMPARISON OF GROUP 0 WITH GROUP 4 (FLUTAMIDE)
PARENTAL FEMALES

Sacrifice			F1	
Sex			F	
Group			0	4
Terminal body weight	g	M	231.23	237.437
		% dev	100	103
		SD	15.499	18.521
		n	20	19
Adrenal glands	mg	M	70.75	76.053
		% dev	100	107
		SD	8.583	10.363
		n	20	19
Brain	g	M	1.862	1.891
		% dev	100	102
		SD	0.07	0.086
		n	20	19
Kidneys	g	M	1.616	1.685*
		% dev	100	104
		SD	0.125	0.147
		n	20	19
Liver	g	M	7.46	7.389
		% dev	100	99
		SD	0.547	0.727
		n	20	19
Ovaries	mg	M	115.35	112.842
		% dev	100	98
		SD	18.933	14.237
		n	20	19
Pituitary gland	mg	M	11.85	11.474
		% dev	100	97
		SD	1.348	1.867
		n	20	19
Spleen	g	M	0.448	0.472
		% dev	100	105
		SD	0.074	0.06
		n	20	19
Thyroid glands	mg	M	18.85	19.526
		% dev	100	104
		SD	3.066	3.627
		n	20	19

*: P <= 0.05, **: P <= 0.01
Wilcoxon test, two sided

ABSOLUTE WEIGHTS - MEAN VALUES
COMPARISON OF GROUP 0 WITH GROUP 4 (FLUTAMIDE)
PARENTAL FEMALES

Sacrifice			F1	
Sex			F	
Group			0	4
Uterus	g	M	0.709	0.677
		% dev	100	96
		SD	0.276	0.289
		n	20	19

*: P <= 0.05, **: P <= 0.01
Wilcoxon test, two sided

RELATIVE WEIGHTS - MEAN VALUES
COMPARISON OF GROUP 0 WITH GROUPS 1,2 AND 3 (MIX)
PARENTAL FEMALES

Sacrifice			F1			
Sex			F			
Group			0	1	2	3
Terminal body weight	%	M	100.0	100.0	100.0	100.0
		% dev	100	100	100	100
		n	20	19	20	19
Adrenal glands	%	M	0.031	0.03	0.032	0.035**
		% dev	100	99	106	115
		SD	0.004	0.004	0.004	0.005
		n	20	19	20	19
Brain	%	M	0.808	0.81	0.814	0.804
		% dev	100	100	101	100
		SD	0.048	0.055	0.053	0.047
		n	20	19	20	19
Kidneys	%	M	0.699	0.694	0.706	0.742**
		% dev	100	99	101	106
		SD	0.031	0.035	0.037	0.047
		n	20	19	20	19
Liver	%	M	3.227	3.106	3.177	3.856**
		% dev	100	96	98	119
		SD	0.128	0.264	0.157	0.27
		n	20	19	20	19
Ovaries	%	M	0.05	0.052	0.054	0.056
		% dev	100	104	107	113
		SD	0.008	0.007	0.009	0.012
		n	20	19	20	19
Pituitary gland	%	M	0.005	0.006*	0.006*	0.005
		% dev	100	111	108	100
		SD	0.001	0.001	0.001	0.001
		n	20	19	20	19
Spleen	%	M	0.193	0.204	0.2	0.205
		% dev	100	105	103	106
		SD	0.027	0.025	0.034	0.022
		n	20	19	20	19
Thyroid glands	%	M	0.008	0.008	0.009	0.009
		% dev	100	102	104	109
		SD	0.001	0.002	0.002	0.002
		n	20	19	20	19

*: P <= 0.05, **: P <= 0.01
Kruskal-Wallis H and Wilcoxon test, two sided

Reproductive Toxicity Study to detect potential effects to anti-androgenic substances in Wistar Rats (Gavage) 28.Mar.2014 SIGR

RELATIVE WEIGHTS - MEAN VALUES
COMPARISON OF GROUP 0 WITH GROUPS 1,2 AND 3 (MIX)
PARENTAL FEMALES

Sacrifice			F1			
Sex			F			
Group			0	1	2	3
Uterus	%	M	0.309	0.295	0.275	0.29
		% dev	100	95	89	94
		SD	0.121	0.11	0.092	0.072
		n	20	19	20	19

*: P <= 0.05, **: P <= 0.01
Kruskal-Wallis H and Wilcoxon test, two sided

RELATIVE WEIGHTS - MEAN VALUES
COMPARISON OF GROUP 0 AND GROUP 4 (FLUTAMIDE)
PARENTAL FEMALES

Sacrifice			F1	
Sex			F	
Group			0	4
Terminal body weight	%	M	100.0	100.0
		% dev	100	100
		n	20	19
Adrenal glands	%	M	0.031	0.032
		% dev	100	105
		SD	0.004	0.004
		n	20	19
Brain	%	M	0.808	0.799
		% dev	100	99
		SD	0.048	0.046
		n	20	19
Kidneys	%	M	0.699	0.711
		% dev	100	102
		SD	0.031	0.051
		n	20	19
Liver	%	M	3.227	3.11 *
		% dev	100	96
		SD	0.128	0.156
		n	20	19
Ovaries	%	M	0.05	0.048
		% dev	100	96
		SD	0.008	0.007
		n	20	19
Pituitary gland	%	M	0.005	0.005
		% dev	100	94
		SD	0.001	0.001
		n	20	19
Spleen	%	M	0.193	0.199
		% dev	100	103
		SD	0.027	0.025
		n	20	19
Thyroid glands	%	M	0.008	0.008
		% dev	100	101
		SD	0.001	0.002
		n	20	19

*: P <= 0.05, **: P <= 0.01
Wilcoxon test, two sided

RELATIVE WEIGHTS - MEAN VALUES
COMPARISON OF GROUP 0 AND GROUP 4 (FLUTAMIDE)
PARENTAL FEMALES

Sacrifice			F1	
Sex			F	
Group			0	4
Uterus	%	M	0.309	0.283
		% dev	100	92
		SD	0.121	0.11
		n	20	19

*: P <= 0.05, **: P <= 0.01
Wilcoxon test, two sided

ABSOLUTE WEIGHTS - MEAN VALUES (MALE)
COMPARISON OF GROUP 0 WITH GROUPS 1,2 AND 3 (MIX)
SUBSET 1 (POSTNATAL DAY 21)

Sacrifice			R1			
Sex			M			
Group			0	1	2	3
Terminal body weight	g	M	44.77	44.64	44.33	47.37
		% dev	100	100	99	106
		SD	5.531	5.495	5.091	3.609
		n	10	10	10	10
Adrenal glands	mg	M	12.5	12.0	13.6	13.1
		% dev	100	96	109	105
		SD	4.17	4.967	4.402	3.213
		n	10	10	10	10
Brain	g	M	1.449	1.467	1.484	1.497
		% dev	100	101	102	103
		SD	0.091	0.05	0.098	0.059
		n	10	10	10	10
Kidneys	g	M	0.462	0.423	0.461	0.49
		% dev	100	92	100	106
		SD	0.078	0.124	0.059	0.05
		n	10	10	10	10
Liver	g	M	1.584	1.608	1.555	1.705
		% dev	100	102	98	108
		SD	0.251	0.269	0.244	0.136
		n	10	10	10	10
Pituitary gland	mg	M	1.2	1.14	0.82 *	1.79 *
		% dev	100	95	68	149
		SD	0.368	0.479	0.478	0.939
		n	10	10	10	10
Spleen	g	M	0.173	0.174	0.162	0.178
		% dev	100	101	94	103
		SD	0.048	0.047	0.039	0.038
		n	10	10	10	10
Thyroid glands	mg	M	4.85	5.32	6.15	7.46
		% dev	100	110	127	154
		SD	2.058	1.831	2.507	2.191
		n	10	10	10	10

*: P <= 0.05, **: P <= 0.01
Kruskal-Wallis H and Wilcoxon test, two sided

ABSOLUTE WEIGHTS - MEAN VALUES (MALE) SEX ORGANS
COMPARISON OF GROUP 0 WITH GROUPS 1,2 AND 3 (MIX)
SUBSET 1 (POSTNATAL DAY 21)

Sacrifice			R1			
Sex			M			
Group			0	1	2	3
Cauda epididymis	mg	M	10.0	12.7	9.4	9.2
		% dev	100	127	94	92
		SD	3.916	5.293	3.026	2.53
		n	10	10	10	10
Epididymides	mg	M	29.0	31.6	29.4	26.6
		% dev	100	109	101	92
		SD	5.981	8.235	5.621	2.633
		n	10	10	10	10
Muscles bulb + l.ani	mg	M	35.45	32.61	33.76	35.5
		% dev	100	92	95	100
		SD	8.759	7.758	7.186	9.647
		n	10	10	10	10
Prostate	mg	M	48.51	47.09	45.24	42.35
		% dev	100	97	93	87
		SD	5.773	8.504	6.754	8.452
		n	10	10	10	10
Prostate ventr.fresh	mg	M	24.4	24.36	24.03	19.21
		% dev	100	100	98	79
		SD	3.25	3.59	6.009	5.633
		n	10	10	10	10
Seminal vesicle	mg	M	9.27	9.85	9.17	8.15
		% dev	100	106	99	88
		SD	1.989	1.531	1.532	1.756
		n	10	10	10	10
Testes	mg	M	237.8	245.0	234.1	257.9
		% dev	100	103	98	108
		SD	27.868	38.404	51.682	34.594
		n	10	10	10	10

*: P <= 0.05, **: P <= 0.01
Kruskal-Wallis H and Wilcoxon test, one sided

ABSOLUTE WEIGHTS - MEAN VALUES (MALE)
COMPARISON OF GROUP 0 WITH GROUP 4 (FLUTAMIDE)
SUBSET 1 (POSTNATAL DAY 21)

Sacrifice			R1	
Sex			M	
Group			0	4
Terminal body weight	g	M	44.77	46.83
		% dev	100	105
		SD	5.531	5.576
		n	10	10
Adrenal glands	mg	M	12.5	12.6
		% dev	100	101
		SD	4.17	2.413
		n	10	10
Brain	g	M	1.449	1.492
		% dev	100	103
		SD	0.091	0.048
		n	10	10
Kidneys	g	M	0.462	0.489
		% dev	100	106
		SD	0.078	0.064
		n	10	10
Liver	g	M	1.584	1.698
		% dev	100	107
		SD	0.251	0.255
		n	10	10
Pituitary gland	mg	M	1.2	1.35
		% dev	100	113
		SD	0.368	0.46
		n	10	10
Spleen	g	M	0.173	0.18
		% dev	100	104
		SD	0.048	0.037
		n	10	10
Thyroid glands	mg	M	4.85	6.92
		% dev	100	143
		SD	2.058	3.293
		n	10	10

*: P <= 0.05, **: P <= 0.01
Wilcoxon test, two sided

Reproductive Toxicity Study to detect potential effects to anti-androgenic substances in Wistar Rats (Gavage) 28.Mar.2014 SIGR

ABSOLUTE WEIGHTS - MEAN VALUES (MALE) SEX ORGANS
COMPARISON OF GROUP 0 WITH GROUP 4 (FLUTAMIDE)
SUBSET 1 (POSTNATAL DAY 21)

Sacrifice			R1	
Sex			M	
Group			0	4
Cauda epididymis	mg	M	10.0	10.4
		% dev	100	104
		SD	3.916	2.011
		n	10	10
Epididymides	mg	M	29.0	30.3
		% dev	100	104
		SD	5.981	4.029
		n	10	10
Muscles bulb + l.ani	mg	M	35.45	36.95
		% dev	100	104
		SD	8.759	6.688
		n	10	10
Prostate	mg	M	48.51	51.64
		% dev	100	106
		SD	5.773	4.062
		n	10	10
Prostate ventr.fresh	mg	M	24.4	26.21
		% dev	100	107
		SD	3.25	3.529
		n	10	10
Seminal vesicle	mg	M	9.27	9.54
		% dev	100	103
		SD	1.989	1.936
		n	10	10
Testes	mg	M	237.8	258.0
		% dev	100	108
		SD	27.868	36.518
		n	10	10

*: P <= 0.05, **: P <= 0.01
Wilcoxon test, one sided

ABSOLUTE WEIGHTS - MEAN VALUES (FEMALE)
COMPARISON OF GROUP 0 WITH GROUPS 1,2 AND 3 (MIX)
SUBSET 1 (POSTNATAL DAY 21)

Sacrifice			R1			
Sex			F			
Group			0	1	2	3
Terminal body weight	g	M	43.37	44.46	43.53	46.66
		% dev	100	103	100	108
		SD	2.4	5.213	3.891	3.84
		n	10	10	10	10
Adrenal glands	mg	M	15.7	13.2	13.0	16.9
		% dev	100	84	83	108
		SD	3.093	2.7	3.055	3.665
		n	10	10	10	10
Brain	g	M	1.438	1.429	1.427	1.465
		% dev	100	99	99	102
		SD	0.035	0.069	0.041	0.054
		n	10	10	10	10
Kidneys	g	M	0.472	0.495	0.484	0.514
		% dev	100	105	103	109
		SD	0.038	0.076	0.048	0.063
		n	10	10	10	10
Liver	g	M	1.538	1.593	1.591	1.717
		% dev	100	104	103	112
		SD	0.124	0.222	0.19	0.154
		n	10	10	10	10
Ovaries	mg	M	14.0	13.6	13.5	15.9
		% dev	100	97	96	114
		SD	1.491	1.955	4.673	4.04
		n	10	10	10	10
Pituitary gland	mg	M	1.44	1.67	1.41	1.68
		% dev	100	116	98	117
		SD	0.677	0.729	0.367	0.485
		n	10	10	10	10
Spleen	g	M	0.175	0.171	0.171	0.204
		% dev	100	98	97	117
		SD	0.032	0.038	0.029	0.052
		n	10	10	10	10
Thyroid glands	mg	M	6.1	5.32	6.37	8.8 *
		% dev	100	87	104	144
		SD	1.493	2.926	3.355	2.878
		n	10	10	10	10

*: P <= 0.05, **: P <= 0.01
Kruskal-Wallis H and Wilcoxon test, two sided

Reproductive Toxicity Study to detect potential effects to anti-androgenic substances in Wistar Rats (Gavage) 28.Mar.2014 SIGR

ABSOLUTE WEIGHTS - MEAN VALUES (FEMALE)
COMPARISON OF GROUP 0 WITH GROUPS 1,2 AND 3 (MIX)
SUBSET 1 (POSTNATAL DAY 21)

Sacrifice			R1			
Sex			F			
Group			0	1	2	3
Uterus	mg	M	36.5	40.0	40.4	44.0 **
		% dev	100	110	111	121
		SD	4.72	5.715	3.471	4.163
		n	10	10	10	10

*: P <= 0.05, **: P <= 0.01
Kruskal-Wallis H and Wilcoxon test, two sided

ABSOLUTE WEIGHTS - MEAN VALUES (FEMALE)
COMPARISON OF GROUP 0 WITH GROUP 4 (FLUTAMIDE)
SUBSET 1 (POSTNATAL DAY 21)

Sacrifice			R1	
Sex			F	
Group			0	4
Terminal body weight	g	M	43.37	45.27
		% dev	100	104
		SD	2.4	6.063
		n	10	10
Adrenal glands	mg	M	15.7	15.4
		% dev	100	98
		SD	3.093	3.836
		n	10	10
Brain	g	M	1.438	1.479*
		% dev	100	103
		SD	0.035	0.039
		n	10	10
Kidneys	g	M	0.472	0.51
		% dev	100	108
		SD	0.038	0.065
		n	10	10
Liver	g	M	1.538	1.651
		% dev	100	107
		SD	0.124	0.224
		n	10	10
Ovaries	mg	M	14.0	15.7
		% dev	100	112
		SD	1.491	3.889
		n	10	10
Pituitary gland	mg	M	1.44	1.85
		% dev	100	128
		SD	0.677	0.443
		n	10	10
Spleen	g	M	0.175	0.192
		% dev	100	110
		SD	0.032	0.067
		n	10	10
Thyroid glands	mg	M	6.1	7.3
		% dev	100	120
		SD	1.493	2.785
		n	10	10

*: P <= 0.05, **: P <= 0.01
Wilcoxon test, two sided

ABSOLUTE WEIGHTS - MEAN VALUES (FEMALE)
COMPARISON OF GROUP 0 WITH GROUP 4 (FLUTAMIDE)
SUBSET 1 (POSTNATAL DAY 21)

Sacrifice			R1	
Sex			F	
Group			0	4
Uterus	mg	M	36.5	39.5
		% dev	100	108
		SD	4.72	8.436
		n	10	10

*: P <= 0.05, **: P <= 0.01
Wilcoxon test, two sided

RELATIVE WEIGHTS - MEAN VALUES (MALE)
COMPARISON OF GROUP 0 WITH GROUPS 1,2 AND 3 (MIX)
SUBSET 1 (POSTNATAL DAY 21)

Sacrifice			R1			
Sex			M			
Group			0	1	2	3
Terminal body weight	%	M	100.0	100.0	100.0	100.0
		% dev	100	100	100	100
		n	10	10	10	10
Adrenal glands	%	M	0.028	0.027	0.031	0.028
		% dev	100	96	110	100
		SD	0.008	0.01	0.009	0.008
		n	10	10	10	10
Brain	%	M	3.266	3.326	3.37	3.174
		% dev	100	102	103	97
		SD	0.328	0.368	0.244	0.251
		n	10	10	10	10
Kidneys	%	M	1.029	0.954	1.039	1.033
		% dev	100	93	101	100
		SD	0.06	0.259	0.033	0.058
		n	10	10	10	10
Liver	%	M	3.525	3.584	3.495	3.599
		% dev	100	102	99	102
		SD	0.204	0.226	0.188	0.104
		n	10	10	10	10
Pituitary gland	%	M	0.003	0.003	0.002	0.004
		% dev	100	94	68	138
		SD	0.001	0.001	0.001	0.002
		n	10	10	10	10
Spleen	%	M	0.383	0.383	0.361	0.373
		% dev	100	100	94	97
		SD	0.075	0.067	0.054	0.061
		n	10	10	10	10
Thyroid glands	%	M	0.011	0.012	0.014	0.016
		% dev	100	109	126	146
		SD	0.005	0.004	0.005	0.005
		n	10	10	10	10

*: P <= 0.05, **: P <= 0.01
Kruskal-Wallis H and Wilcoxon test, two sided

RELATIVE WEIGHTS - MEAN VALUES (MALE) SEX ORGANS
COMPARISON OF GROUP 0 WITH GROUPS 1,2 AND 3 (MIX)
SUBSET 1 (POSTNATAL DAY 21)

Sacrifice			R1			
Sex			M			
Group			0	1	2	3
Terminal body weight	%	M	100.0	100.0	100.0	100.0
		% dev	100	100	100	100
		n	10	10	10	10
Cauda epididymis	%	M	0.023	0.029	0.021	0.02
		% dev	100	126	92	85
		SD	0.01	0.013	0.006	0.006
		n	10	10	10	10
Epididymides	%	M	0.065	0.071	0.066	0.056
		% dev	100	108	101	86
		SD	0.014	0.018	0.009	0.006
		n	10	10	10	10
Muscles bulb + l.ani	%	M	0.081	0.074	0.077	0.075
		% dev	100	91	95	93
		SD	0.023	0.019	0.016	0.02
		n	10	10	10	10
Prostate	%	M	0.11	0.106	0.102	0.09
		% dev	100	96	93	82
		SD	0.02	0.015	0.009	0.018
		n	10	10	10	10
Prostate ventr.fresh	%	M	0.055	0.055	0.054	0.041**
		% dev	100	100	98	74
		SD	0.008	0.008	0.009	0.012
		n	10	10	10	10
Seminal vesicle	%	M	0.021	0.022	0.021	0.017
		% dev	100	106	101	83
		SD	0.005	0.003	0.005	0.004
		n	10	10	10	10
Testes	%	M	0.532	0.548	0.523	0.544
		% dev	100	103	98	102
		SD	0.026	0.043	0.066	0.058
		n	10	10	10	10

*: P <= 0.05, **: P <= 0.01
Kruskal-Wallis H and Wilcoxon test, one sided

RELATIVE WEIGHTS - MEAN VALUES (MALE)
COMPARISON OF GROUP 0 WITH GROUPS 4 (FLUTAMIDE)
SUBSET 1 (POSTNATAL DAY 21)

Sacrifice			R1	
Sex			M	
Group			0	4
Terminal body weight	%	M	100.0	100.0
		% dev	100	100
		n	10	10
Adrenal glands	%	M	0.028	0.027
		% dev	100	97
		SD	0.008	0.004
		n	10	10
Brain	%	M	3.266	3.222
		% dev	100	99
		SD	0.328	0.345
		n	10	10
Kidneys	%	M	1.029	1.043
		% dev	100	101
		SD	0.06	0.033
		n	10	10
Liver	%	M	3.525	3.615
		% dev	100	103
		SD	0.204	0.127
		n	10	10
Pituitary gland	%	M	0.003	0.003
		% dev	100	107
		SD	0.001	0.001
		n	10	10
Spleen	%	M	0.383	0.382
		% dev	100	100
		SD	0.075	0.047
		n	10	10
Thyroid glands	%	M	0.011	0.015
		% dev	100	137
		SD	0.005	0.008
		n	10	10

*: P <= 0.05, **: P <= 0.01
Wilcoxon test, two sided

RELATIVE WEIGHTS - MEAN VALUES (MALE) SEX ORGANS
COMPARISON OF GROUP 0 WITH GROUP 4 (FLUTAMIDE)
SUBSET 1 (POSTNATAL DAY 21)

Sacrifice			R1	
Sex			M	
Group			0	4
Terminal body weight	%	M	100.0	100.0
		% dev	100	100
		n	10	10
Cauda epididymis	%	M	0.023	0.022
		% dev	100	98
		SD	0.01	0.005
		n	10	10
Epididymides	%	M	0.065	0.065
		% dev	100	99
		SD	0.014	0.007
		n	10	10
Muscles bulb + l.ani	%	M	0.081	0.079
		% dev	100	99
		SD	0.023	0.016
		n	10	10
Prostate	%	M	0.11	0.111
		% dev	100	102
		SD	0.02	0.014
		n	10	10
Prostate ventr.fresh	%	M	0.055	0.056
		% dev	100	103
		SD	0.008	0.009
		n	10	10
Seminal vesicle	%	M	0.021	0.02
		% dev	100	97
		SD	0.005	0.003
		n	10	10
Testes	%	M	0.532	0.551
		% dev	100	104
		SD	0.026	0.041
		n	10	10

*: P <= 0.05, **: P <= 0.01
Wilcoxon test, one sided

RELATIVE WEIGHTS - MEAN VALUES (FEMALE)
COMPARISON OF GROUP 0 WITH GROUPS 1,2 AND 3 (MIX)
SUBSET 1 (POSTNATAL DAY 21)

Sacrifice			R1			
Sex			F			
Group			0	1	2	3
Terminal body weight	%	M	100.0	100.0	100.0	100.0
		% dev	100	100	100	100
		n	10	10	10	10
Adrenal glands	%	M	0.036	0.03 *	0.03	0.036
		% dev	100	83	82	100
		SD	0.006	0.005	0.006	0.007
		n	10	10	10	10
Brain	%	M	3.322	3.24	3.296	3.155
		% dev	100	98	99	95
		SD	0.133	0.265	0.235	0.231
		n	10	10	10	10
Kidneys	%	M	1.087	1.111	1.114	1.099
		% dev	100	102	102	101
		SD	0.045	0.064	0.073	0.061
		n	10	10	10	10
Liver	%	M	3.544	3.581	3.655	3.682
		% dev	100	101	103	104
		SD	0.14	0.246	0.311	0.192
		n	10	10	10	10
Ovaries	%	M	0.032	0.031	0.031	0.034
		% dev	100	96	96	106
		SD	0.004	0.006	0.011	0.009
		n	10	10	10	10
Pituitary gland	%	M	0.003	0.004	0.003	0.004
		% dev	100	110	97	107
		SD	0.002	0.001	0.001	0.001
		n	10	10	10	10
Spleen	%	M	0.402	0.382	0.39	0.434
		% dev	100	95	97	108
		SD	0.057	0.057	0.04	0.082
		n	10	10	10	10
Thyroid glands	%	M	0.014	0.012	0.014	0.019
		% dev	100	85	102	133
		SD	0.004	0.006	0.007	0.006
		n	10	10	10	10

*: P <= 0.05, **: P <= 0.01
Kruskal-Wallis H and Wilcoxon test, two sided

Reproductive Toxicity Study to detect potential effects to anti-androgenic substances in Wistar Rats (Gavage) 28.Mar.2014 SIGR

RELATIVE WEIGHTS - MEAN VALUES (FEMALE)
COMPARISON OF GROUP 0 WITH GROUPS 1,2 AND 3 (MIX)
SUBSET 1 (POSTNATAL DAY 21)

Sacrifice			R1			
Sex			F			
Group			0	1	2	3
Uterus	%	M	0.084	0.091	0.094	0.095
		% dev	100	108	111	113
		SD	0.008	0.014	0.013	0.013
		n	10	10	10	10

*: P <= 0.05, **: P <= 0.01
Kruskal-Wallis H and Wilcoxon test, two sided

RELATIVE WEIGHTS - MEAN VALUES (FEMALE)
COMPARISON OF GROUP 0 WITH GROUP 4 (FLUTAMIDE)
SUBSET 1 (POSTNATAL DAY 21)

Sacrifice			R1	
Sex			F	
Group			0	4
Terminal body weight	%	M	100.0	100.0
		% dev	100	100
		n	10	10
Adrenal glands	%	M	0.036	0.034
		% dev	100	94
		SD	0.006	0.005
		n	10	10
Brain	%	M	3.322	3.31
		% dev	100	100
		SD	0.133	0.37
		n	10	10
Kidneys	%	M	1.087	1.129
		% dev	100	104
		SD	0.045	0.069
		n	10	10
Liver	%	M	3.544	3.649
		% dev	100	103
		SD	0.14	0.184
		n	10	10
Ovaries	%	M	0.032	0.035
		% dev	100	107
		SD	0.004	0.007
		n	10	10
Pituitary gland	%	M	0.003	0.004
		% dev	100	122
		SD	0.002	0.001
		n	10	10
Spleen	%	M	0.402	0.418
		% dev	100	104
		SD	0.057	0.099
		n	10	10
Thyroid glands	%	M	0.014	0.017
		% dev	100	116
		SD	0.004	0.007
		n	10	10

*: P <= 0.05, **: P <= 0.01
Wilcoxon test, two sided

RELATIVE WEIGHTS - MEAN VALUES (FEMALE)
COMPARISON OF GROUP 0 WITH GROUP 4 (FLUTAMIDE)
SUBSET 1 (POSTNATAL DAY 21)

Sacrifice			R1	
Sex			F	
Group			0	4
Uterus	%	M	0.084	0.087
		% dev	100	104
		SD	0.008	0.014
		n	10	10

*: P <= 0.05, **: P <= 0.01
Wilcoxon test, two sided

ABSOLUTE WEIGHTS - MEAN VALUES (MALE)
COMPARISON OF GROUP 0 WITH GROUPS 1,2 AND 3 (MIX)
SUBSET 2 (SEXUAL MATURITY)

Sacrifice			R2			
Sex			M			
Group			0	1	2	3
Terminal body weight	g	M	184.71	186.5	186.08	218.17 **
		% dev	100	101	101	118
		SD	18.572	9.893	18.631	18.994
		n	10	10	10	10
Adrenal glands	mg	M	38.6	38.0	38.7	46.5 *
		% dev	100	98	100	120
		SD	7.168	5.142	6.684	7.106
		n	10	10	10	10
Brain	g	M	1.813	1.829	1.796	1.835
		% dev	100	101	99	101
		SD	0.067	0.084	0.102	0.074
		n	10	10	10	10
Kidneys	g	M	1.582	1.577	1.57	1.788*
		% dev	100	100	99	113
		SD	0.155	0.114	0.161	0.175
		n	10	10	10	10
Liver	g	M	8.294	8.485	8.437	9.775*
		% dev	100	102	102	118
		SD	1.294	0.582	0.967	0.665
		n	10	10	10	10
Pituitary gland	mg	M	6.64	6.75	6.56	6.77
		% dev	100	102	99	102
		SD	0.916	0.45	0.809	1.029
		n	10	10	10	10
Spleen	g	M	0.503	0.503	0.498	0.543
		% dev	100	100	99	108
		SD	0.084	0.048	0.111	0.075
		n	10	10	10	10
Thyroid glands	mg	M	13.87	15.16	14.25	17.88 **
		% dev	100	109	103	129
		SD	2.62	2.447	2.718	2.304
		n	10	10	10	10

*: P <= 0.05, **: P <= 0.01
Kruskal-Wallis H and Wilcoxon test, two sided

Reproductive Toxicity Study to detect potential effects to anti-androgenic substances in Wistar Rats (Gavage) 28.Mar.2014 SIGR

ABSOLUTE WEIGHTS - MEAN VALUES (MALE) SEX ORGANS
COMPARISON OF GROUP 0 WITH GROUPS 1,2 AND 3 (MIX)
SUBSET 2 (SEXUAL MATURITY)

Sacrifice			R2			
Sex			M			
Group			0	1	2	3
Bulbo-urethral gland	mg	M	13.86	15.43	13.94	11.36
		% dev	100	111	101	82
		SD	3.294	4.451	5.03	6.218
		n	10	10	10	10
Cauda epididymis	mg	M	57.7	59.7	57.6	80.0
		% dev	100	103	100	139
		SD	11.461	10.884	9.594	22.341
		n	10	10	10	10
Epididymides	mg	M	220.2	225.9	227.8	351.2
		% dev	100	103	103	159
		SD	44.296	32.378	35.273	68.14
		n	10	10	10	10
Glans penis	mg	M	49.53	52.69	52.82	42.16 *
		% dev	100	106	107	85
		SD	7.358	10.139	10.388	6.29
		n	10	10	10	10
Muscles bulb + l.ani	mg	M	194.66	213.13	191.78	199.0
		% dev	100	109	99	102
		SD	33.864	41.405	30.67	42.468
		n	10	10	10	10
Prostate	mg	M	187.1	204.36	185.06	192.23
		% dev	100	109	99	103
		SD	38.297	32.898	36.113	38.35
		n	10	10	10	10
Prostate ventr.fresh	mg	M	106.59	114.06	97.2	101.38
		% dev	100	107	91	95
		SD	29.408	20.204	24.405	22.219
		n	10	10	10	10
Seminal vesicle	mg	M	109.97	117.75	117.08	137.3
		% dev	100	107	106	125
		SD	36.366	37.173	38.851	54.67
		n	10	10	10	10
Testes	mg	M	2227.1	2093.9	2190.6	2708.6
		% dev	100	94	98	122
		SD	321.053	154.209	283.883	217.225
		n	10	10	10	10

*: P <= 0.05, **: P <= 0.01
Kruskal-Wallis H and Wilcoxon test, one sided

ABSOLUTE WEIGHTS - MEAN VALUES (MALE)
COMPARISON OF GROUP 0 WITH GROUP 4 (FLUTAMIDE)
SUBSET 2 (SEXUAL MATURITY)

Sacrifice			R2	
Sex			M	
Group			0	4
Terminal body weight	g	M	184.71	189.1
		% dev	100	102
		SD	18.572	16.616
		n	10	10
Adrenal glands	mg	M	38.6	40.3
		% dev	100	104
		SD	7.168	3.129
		n	10	10
Brain	g	M	1.813	1.842
		% dev	100	102
		SD	0.067	0.063
		n	10	10
Kidneys	g	M	1.582	1.674
		% dev	100	106
		SD	0.155	0.191
		n	10	10
Liver	g	M	8.294	8.686
		% dev	100	105
		SD	1.294	0.819
		n	10	10
Pituitary gland	mg	M	6.64	6.71
		% dev	100	101
		SD	0.916	0.551
		n	10	10
Spleen	g	M	0.503	0.512
		% dev	100	102
		SD	0.084	0.077
		n	10	10
Thyroid glands	mg	M	13.87	16.111*
		% dev	100	116
		SD	2.62	2.177
		n	10	9

*: P <= 0.05, **: P <= 0.01
Wilcoxon test, two sided

ABSOLUTE WEIGHTS - MEAN VALUES (MALE) SEX ORGANS
COMPARISON OF GROUP 0 WITH GROUP 4 (FLUTAMIDE)
SUBSET 2 (SEXUAL MATURITY)

Sacrifice			R2	
Sex			M	
Group			0	4
Bulbo-urethral gland	mg	M	13.86	13.97
		% dev	100	101
		SD	3.294	4.559
		n	10	10
Cauda epididymis	mg	M	57.7	58.9
		% dev	100	102
		SD	11.461	11.808
		n	10	10
Epididymides	mg	M	220.2	226.1
		% dev	100	103
		SD	44.296	42.275
		n	10	10
Glans penis	mg	M	49.53	52.43
		% dev	100	106
		SD	7.358	8.923
		n	10	10
Muscles bulb + l.ani	mg	M	194.66	215.3
		% dev	100	111
		SD	33.864	51.306
		n	10	10
Prostate	mg	M	187.1	222.12
		% dev	100	119
		SD	38.297	39.177
		n	10	10
Prostate ventr.fresh	mg	M	106.59	120.63
		% dev	100	113
		SD	29.408	27.322
		n	10	10
Seminal vesicle	mg	M	109.97	110.54
		% dev	100	101
		SD	36.366	36.083
		n	10	10
Testes	mg	M	2227.1	2144.5
		% dev	100	96
		SD	321.053	282.858
		n	10	10

*: P <= 0.05, **: P <= 0.01
Wilcoxon test, one sided

ABSOLUTE WEIGHTS - MEAN VALUES (FEMALE)
COMPARISON OF GROUP 0 WITH GROUPS 1,2 AND 3 (MIX)
SUBSET 2 (SEXUAL MATURITY)

Sacrifice			R2			
Sex			F			
Group			0	1	2	3
Terminal body weight	g	M	84.5	82.15	82.12	83.64
		% dev	100	97	97	99
		SD	8.125	8.242	8.496	7.062
		n	10	10	10	10
Adrenal glands	mg	M	28.4	25.7	26.9	27.8
		% dev	100	90	95	98
		SD	8.796	7.454	7.37	3.967
		n	10	10	10	10
Brain	g	M	1.532	1.564	1.578	1.575
		% dev	100	102	103	103
		SD	0.044	0.06	0.039	0.049
		n	10	10	10	10
Kidneys	g	M	0.913	0.94	0.902	0.905
		% dev	100	103	99	99
		SD	0.072	0.084	0.092	0.091
		n	10	10	10	10
Liver	g	M	3.93	3.907	3.778	4.091
		% dev	100	99	96	104
		SD	0.519	0.545	0.459	0.387
		n	10	10	10	10
Ovaries	mg	M	35.9	36.1	30.7	28.9
		% dev	100	101	86	81
		SD	9.469	7.965	7.602	5.567
		n	10	10	10	10
Pituitary gland	mg	M	5.49	5.76	4.71	3.97 **
		% dev	100	105	86	72
		SD	0.824	1.071	0.829	0.556
		n	10	10	10	10
Spleen	g	M	0.252	0.26	0.254	0.266
		% dev	100	103	101	106
		SD	0.031	0.053	0.057	0.032
		n	10	10	10	10
Thyroid glands	mg	M	11.28	10.19	12.89	13.76
		% dev	100	90	114	122
		SD	1.827	1.233	3.552	3.247
		n	10	10	10	10

*: P <= 0.05, **: P <= 0.01
Kruskal-Wallis H and Wilcoxon test, two sided

ABSOLUTE WEIGHTS - MEAN VALUES (FEMALE)
COMPARISON OF GROUP 0 WITH GROUPS 1,2 AND 3 (MIX)
SUBSET 2 (SEXUAL MATURITY)

Sacrifice			R2			
Sex			F			
Group			0	1	2	3
Uterus	mg	M	299.5	322.7	318.7	325.5
		% dev	100	108	106	109
		SD	53.303	99.992	96.701	121.802
		n	10	10	10	10

*: P <= 0.05, **: P <= 0.01
Kruskal-Wallis H and Wilcoxon test, two sided

ABSOLUTE WEIGHTS - MEAN VALUES (FEMALE)
COMPARISON OF GROUP 0 WITH GROUP 4 (FLUTAMIDE)
SUBSET 2 (SEXUAL MATURITY)

Sacrifice			R2	
Sex			F	
Group			0	4
Terminal body weight	g	M	84.5	86.92
		% dev	100	103
		SD	8.125	8.916
		n	10	10
Adrenal glands	mg	M	28.4	25.8
		% dev	100	91
		SD	8.796	4.872
		n	10	10
Brain	g	M	1.532	1.572
		% dev	100	103
		SD	0.044	0.056
		n	10	10
Kidneys	g	M	0.913	0.909
		% dev	100	99
		SD	0.072	0.092
		n	10	10
Liver	g	M	3.93	3.845
		% dev	100	98
		SD	0.519	0.492
		n	10	10
Ovaries	mg	M	35.9	31.0
		% dev	100	86
		SD	9.469	7.439
		n	10	10
Pituitary gland	mg	M	5.49	5.29
		% dev	100	96
		SD	0.824	0.684
		n	10	10
Spleen	g	M	0.252	0.257
		% dev	100	102
		SD	0.031	0.035
		n	10	10
Thyroid glands	mg	M	11.28	11.93
		% dev	100	106
		SD	1.827	2.016
		n	10	10

*: P <= 0.05, **: P <= 0.01
Wilcoxon test, two sided

ABSOLUTE WEIGHTS - MEAN VALUES (FEMALE)
COMPARISON OF GROUP 0 WITH GROUP 4 (FLUTAMIDE)
SUBSET 2 (SEXUAL MATURITY)

Sacrifice			R2	
Sex			F	
Group			0	4
Uterus	mg	M	299.5	294.4
		% dev	100	98
		SD	53.303	101.44
		n	10	10

*: P <= 0.05, **: P <= 0.01
Wilcoxon test, two sided

RELATIVE WEIGHTS - MEAN VALUES (MALE)
COMPARISON OF GROUP 0 WITH GROUPS 1,2 AND 3 (MIX)
SUBSET 2 (SEXUAL MATURITY)

Sacrifice			R2			
Sex			M			
Group			0	1	2	3
Terminal body weight	%	M	100.0	100.0	100.0	100.0
		% dev	100	100	100	100
		n	10	10	10	10
Adrenal glands	%	M	0.021	0.02	0.021	0.021
		% dev	100	98	100	102
		SD	0.002	0.002	0.003	0.003
		n	10	10	10	10
Brain	%	M	0.988	0.982	0.97	0.845**
		% dev	100	99	98	86
		SD	0.078	0.045	0.069	0.06
		n	10	10	10	10
Kidneys	%	M	0.858	0.845	0.844	0.819
		% dev	100	99	98	96
		SD	0.036	0.039	0.041	0.025
		n	10	10	10	10
Liver	%	M	4.472	4.548	4.531	4.492
		% dev	100	102	101	100
		SD	0.294	0.141	0.161	0.233
		n	10	10	10	10
Pituitary gland	%	M	0.004	0.004	0.004	0.003*
		% dev	100	101	98	86
		SD	0.0	0.0	0.0	0.0
		n	10	10	10	10
Spleen	%	M	0.272	0.271	0.267	0.251
		% dev	100	99	98	92
		SD	0.039	0.032	0.05	0.044
		n	10	10	10	10
Thyroid glands	%	M	0.008	0.008	0.008	0.008
		% dev	100	108	101	109
		SD	0.001	0.001	0.001	0.001
		n	10	10	10	10

*: P <= 0.05, **: P <= 0.01
Kruskal-Wallis H and Wilcoxon test, two sided

Reproductive Toxicity Study to detect potential effects to anti-androgenic substances in Wistar Rats (Gavage) 28.Mar.2014 SIGR

RELATIVE WEIGHTS - MEAN VALUES (MALE) SEX ORGANS
COMPARISON OF GROUP 0 WITH GROUPS 1,2 AND 3 (MIX)
SUBSET 2 (SEXUAL MATURITY)

Sacrifice			R2			
Sex			M			
Group			0	1	2	3
Terminal body weight	%	M	100.0	100.0	100.0	100.0
		% dev	100	100	100	100
		n	10	10	10	10
Bulbo-urethral gland	%	M	0.007	0.008	0.007	0.005**
		% dev	100	110	100	68
		SD	0.002	0.002	0.002	0.003
		n	10	10	10	10
Cauda epididymis	%	M	0.031	0.032	0.031	0.037
		% dev	100	103	100	118
		SD	0.004	0.006	0.005	0.011
		n	10	10	10	10
Epididymides	%	M	0.119	0.121	0.122	0.16
		% dev	100	102	103	135
		SD	0.017	0.014	0.014	0.022
		n	10	10	10	10
Glans penis	%	M	0.027	0.028	0.028	0.019**
		% dev	100	105	105	73
		SD	0.003	0.005	0.004	0.003
		n	10	10	10	10
Muscles bulb + l.ani	%	M	0.105	0.114	0.103	0.091**
		% dev	100	108	98	86
		SD	0.015	0.018	0.011	0.015
		n	10	10	10	10
Prostate	%	M	0.101	0.109	0.1	0.088
		% dev	100	109	99	87
		SD	0.015	0.016	0.018	0.014
		n	10	10	10	10
Prostate ventr.fresh	%	M	0.057	0.061	0.052	0.046*
		% dev	100	107	92	81
		SD	0.013	0.009	0.012	0.008
		n	10	10	10	10
Seminal vesicle	%	M	0.059	0.063	0.062	0.062
		% dev	100	107	106	105
		SD	0.015	0.018	0.017	0.02
		n	10	10	10	10

*: P <= 0.05, **: P <= 0.01
Kruskal-Wallis H and Wilcoxon test, one sided

Reproductive Toxicity Study to detect potential effects to anti-androgenic substances in Wistar Rats (Gavage) 28.Mar.2014 SIGR

RELATIVE WEIGHTS - MEAN VALUES (MALE) SEX ORGANS
COMPARISON OF GROUP 0 WITH GROUPS 1,2 AND 3 (MIX)
SUBSET 2 (SEXUAL MATURITY)

Sacrifice			R2			
Sex			M			
Group			0	1	2	3
Testes	%	M	1.205	1.123	1.175	1.243
		% dev	100	93	98	103
		SD	0.116	0.066	0.073	0.046
		n	10	10	10	10

*: P <= 0.05, **: P <= 0.01
Kruskal-Wallis H and Wilcoxon test, one sided

Reproductive Toxicity Study to detect potential effects to anti-androgenic substances in Wistar Rats (Gavage) 28.Mar.2014 SIGR

RELATIVE WEIGHTS - MEAN VALUES (MALE)
COMPARISON OF GROUP 0 WITH GROUP 4 (FLUTAMIDE)
SUBSET 2 (SEXUAL MATURITY)

Sacrifice			R2	
Sex			M	
Group			0	4
Terminal body weight	%	M	100.0	100.0
		% dev	100	100
		n	10	10
Adrenal glands	%	M	0.021	0.021
		% dev	100	103
		SD	0.002	0.002
		n	10	10
Brain	%	M	0.988	0.979
		% dev	100	99
		SD	0.078	0.072
		n	10	10
Kidneys	%	M	0.858	0.886
		% dev	100	103
		SD	0.036	0.075
		n	10	10
Liver	%	M	4.472	4.595
		% dev	100	103
		SD	0.294	0.233
		n	10	10
Pituitary gland	%	M	0.004	0.004
		% dev	100	99
		SD	0.0	0.0
		n	10	10
Spleen	%	M	0.272	0.272
		% dev	100	100
		SD	0.039	0.041
		n	10	10
Thyroid glands	%	M	0.008	0.008
		% dev	100	112
		SD	0.001	0.001
		n	10	9

*: P <= 0.05, **: P <= 0.01
Wilcoxon test, two sided

RELATIVE WEIGHTS - MEAN VALUES (MALE) SEX ORGANS
COMPARISON OF GROUP 0 WITH GROUP 4 (FLUTAMIDE)
SUBSET 2 (SEXUAL MATURITY)

Sacrifice			R2	
Sex			M	
Group			0	4
Terminal body weight	%	M	100.0	100.0
		% dev	100	100
		n	10	10
Bulbo-urethral gland	%	M	0.007	0.007
		% dev	100	98
		SD	0.002	0.002
		n	10	10
Cauda epididymis	%	M	0.031	0.031
		% dev	100	100
		SD	0.004	0.004
		n	10	10
Epididymides	%	M	0.119	0.119
		% dev	100	100
		SD	0.017	0.013
		n	10	10
Glans penis	%	M	0.027	0.028
		% dev	100	103
		SD	0.003	0.004
		n	10	10
Muscles bulb + l.ani	%	M	0.105	0.113
		% dev	100	108
		SD	0.015	0.022
		n	10	10
Prostate	%	M	0.101	0.117
		% dev	100	116
		SD	0.015	0.013
		n	10	10
Prostate ventr.fresh	%	M	0.057	0.063
		% dev	100	111
		SD	0.013	0.01
		n	10	10
Seminal vesicle	%	M	0.059	0.058
		% dev	100	98
		SD	0.015	0.016
		n	10	10

*: P <= 0.05, **: P <= 0.01
Wilcoxon test, one sided

RELATIVE WEIGHTS - MEAN VALUES (MALE) SEX ORGANS
COMPARISON OF GROUP 0 WITH GROUP 4 (FLUTAMIDE)
SUBSET 2 (SEXUAL MATURITY)

Sacrifice			R2	
Sex			M	
Group			0	4
Testes	%	M	1.205	1.131
		% dev	100	94
		SD	0.116	0.071
		n	10	10

*: P <= 0.05, **: P <= 0.01
Wilcoxon test, one sided

RELATIVE WEIGHTS - MEAN VALUES (FEMALE)
COMPARISON OF GROUP 0 WITH GROUPS 1,2 AND 3 (MIX)
SUBSET 2 (SEXUAL MATURITY)

Sacrifice			R2			
Sex			F			
Group			0	1	2	3
Terminal body weight	%	M	100.0	100.0	100.0	100.0
		% dev	100	100	100	100
		n	10	10	10	10
Adrenal glands	%	M	0.033	0.031	0.033	0.034
		% dev	100	93	99	100
		SD	0.009	0.008	0.01	0.006
		n	10	10	10	10
Brain	%	M	1.828	1.921	1.941	1.893
		% dev	100	105	106	104
		SD	0.18	0.199	0.209	0.14
		n	10	10	10	10
Kidneys	%	M	1.083	1.149	1.1	1.082
		% dev	100	106	102	100
		SD	0.034	0.112	0.06	0.056
		n	10	10	10	10
Liver	%	M	4.646	4.772	4.597	4.891
		% dev	100	103	99	105
		SD	0.367	0.669	0.217	0.229
		n	10	10	10	10
Ovaries	%	M	0.042	0.045	0.037	0.035
		% dev	100	106	88	82
		SD	0.008	0.011	0.007	0.007
		n	10	10	10	10
Pituitary gland	%	M	0.007	0.007	0.006	0.005**
		% dev	100	108	88	73
		SD	0.001	0.001	0.001	0.001
		n	10	10	10	10
Spleen	%	M	0.3	0.316	0.307	0.319
		% dev	100	106	103	106
		SD	0.037	0.06	0.055	0.034
		n	10	10	10	10
Thyroid glands	%	M	0.013	0.013	0.016	0.016*
		% dev	100	94	118	122
		SD	0.002	0.002	0.005	0.003
		n	10	10	10	10

*: P <= 0.05, **: P <= 0.01
Kruskal-Wallis H and Wilcoxon test, two sided

RELATIVE WEIGHTS - MEAN VALUES (FEMALE)
COMPARISON OF GROUP 0 WITH GROUPS 1,2 AND 3 (MIX)
SUBSET 2 (SEXUAL MATURITY)

Sacrifice			R2			
Sex			F			
Group			0	1	2	3
Uterus	%	M	0.36	0.39	0.392	0.389
		% dev	100	108	109	108
		SD	0.091	0.099	0.128	0.147
		n	10	10	10	10

*: P <= 0.05, **: P <= 0.01
Kruskal-Wallis H and Wilcoxon test, two sided

Reproductive Toxicity Study to detect potential effects to anti-androgenic substances in Wistar Rats (Gavage) 28.Mar.2014 SIGR

RELATIVE WEIGHTS - MEAN VALUES (FEMALE)
COMPARISON OF GROUP 0 WITH GROUP 4 (FLUTAMIDE)
SUBSET 2 (SEXUAL MATURITY)

Sacrifice			R2	
Sex			F	
Group			0	4
Terminal body weight	%	M	100.0	100.0
		% dev	100	100
		n	10	10
Adrenal glands	%	M	0.033	0.03
		% dev	100	89
		SD	0.009	0.005
		n	10	10
Brain	%	M	1.828	1.827
		% dev	100	100
		SD	0.18	0.221
		n	10	10
Kidneys	%	M	1.083	1.049
		% dev	100	97
		SD	0.034	0.087
		n	10	10
Liver	%	M	4.646	4.418
		% dev	100	95
		SD	0.367	0.256
		n	10	10
Ovaries	%	M	0.042	0.036
		% dev	100	85
		SD	0.008	0.008
		n	10	10
Pituitary gland	%	M	0.007	0.006
		% dev	100	94
		SD	0.001	0.001
		n	10	10
Spleen	%	M	0.3	0.297
		% dev	100	99
		SD	0.037	0.033
		n	10	10
Thyroid glands	%	M	0.013	0.014
		% dev	100	102
		SD	0.002	0.002
		n	10	10

*: P <= 0.05, **: P <= 0.01
Wilcoxon test, two sided

RELATIVE WEIGHTS - MEAN VALUES (FEMALE)
COMPARISON OF GROUP 0 WITH GROUP 4 (FLUTAMIDE)
SUBSET 2 (SEXUAL MATURITY)

Sacrifice			R2	
Sex			F	
Group			0	4
Uterus	%	M	0.36	0.341
		% dev	100	95
		SD	0.091	0.125
		n	10	10

*: P <= 0.05, **: P <= 0.01
Wilcoxon test, two sided

ABSOLUTE WEIGHTS - MEAN VALUES (MALE)
COMPARISON OF GROUP 0 WITH GROUPS 1,2 AND 3 (MIX)
SUBSET 3 (POSTNATAL DAY 83 +/- 2 DAYS)

Sacrifice			R3			
Sex			M			
Group			0	1	2	3
Terminal body weight	g	M	315.945	306.83	313.16	301.65
		% dev	100	97	99	95
		SD	27.423	33.902	31.418	29.511
		n	9	10	10	10
Adrenal glands	mg	M	53.111	56.5	50.5	56.4
		% dev	100	106	95	106
		SD	6.772	10.08	7.59	8.514
		n	9	10	10	10
Brain	g	M	1.945	1.989	1.942	1.939
		% dev	100	102	100	100
		SD	0.082	0.072	0.066	0.05
		n	9	10	10	10
Kidneys	g	M	2.186	2.197	2.305	2.228
		% dev	100	101	105	102
		SD	0.208	0.233	0.276	0.151
		n	9	10	10	10
Liver	g	M	11.201	10.551	10.967	11.516
		% dev	100	94	98	103
		SD	1.671	1.53	1.295	1.522
		n	9	10	10	10
Pituitary gland	mg	M	9.156	8.86	8.62	8.22
		% dev	100	97	94	90
		SD	0.794	0.914	0.973	0.872
		n	9	10	10	10
Spleen	g	M	0.532	0.523	0.538	0.546
		% dev	100	98	101	103
		SD	0.062	0.068	0.093	0.075
		n	9	10	10	10
Thyroid glands	mg	M	21.122	22.05	20.85	23.22
		% dev	100	104	99	110
		SD	3.597	4.331	3.553	4.487
		n	9	10	10	10

*: P <= 0.05, **: P <= 0.01
Kruskal-Wallis H and Wilcoxon test, two sided

ABSOLUTE WEIGHTS - MEAN VALUES (MALE) SEX ORGANS
COMPARISON OF GROUP 0 WITH GROUPS 1,2 AND 3 (MIX)
SUBSET 3 (POSTNATAL DAY 83 +/-2 DAYS)

Sacrifice			R3			
Sex			M			
Group			0	1	2	3
Bulbo-urethral gland	mg	M	74.867	69.79	62.45	42.09 **
		% dev	100	93	83	56
		SD	20.09	9.276	7.508	12.354
		n	9	10	10	10
Cauda epididymis	mg	M	324.667	311.0	288.6 *	277.2 **
		% dev	100	96	89	85
		SD	31.914	36.283	29.871	29.439
		n	9	10	10	10
Epididymides	mg	M	897.111	899.3	917.3	833.5
		% dev	100	100	102	93
		SD	80.093	81.4	203.492	72.995
		n	9	10	10	10
Glans penis	mg	M	89.878	89.48	87.52	73.81 **
		% dev	100	100	97	82
		SD	10.305	12.615	10.282	12.521
		n	9	10	10	10
Muscles bulb + l.ani	mg	M	711.167	680.97	640.78	449.97 **
		% dev	100	96	90	63
		SD	83.305	100.956	136.849	72.34
		n	9	10	10	10
Prostate	mg	M	642.556	607.7	628.74	443.89 **
		% dev	100	95	98	69
		SD	61.158	88.399	99.961	86.817
		n	9	10	10	10
Prostate ventr.fresh	mg	M	347.578	323.82	345.33	239.12 **
		% dev	100	93	99	69
		SD	52.268	65.265	65.235	42.61
		n	9	10	10	10
Seminal vesicle	mg	M	989.233	931.65	858.62	611.7 **
		% dev	100	94	87	62
		SD	176.964	122.39	65.788	104.974
		n	9	10	10	10
Testes	mg	M	3530.222	3516.9	3412.6	3616.6
		% dev	100	100	97	102
		SD	253.004	328.563	319.826	280.736
		n	9	10	10	10

*: P <= 0.05, **: P <= 0.01
Kruskal-Wallis H and Wilcoxon test, one sided

ABSOLUTE WEIGHTS - MEAN VALUES (MALE)
COMPARISON OF GROUP 0 WITH GROUP 4 (FLUTAMIDE)
SUBSET 3 (POSTNATAL DAY 83 +/- 2 DAYS)

Sacrifice			R3	
Sex			M	
Group			0	4
Terminal body weight	g	M	315.945	322.73
		% dev	100	102
		SD	27.423	29.78
		n	9	10
Adrenal glands	mg	M	53.111	55.4
		% dev	100	104
		SD	6.772	9.582
		n	9	10
Brain	g	M	1.945	1.992
		% dev	100	102
		SD	0.082	0.065
		n	9	10
Kidneys	g	M	2.186	2.223
		% dev	100	102
		SD	0.208	0.176
		n	9	10
Liver	g	M	11.201	10.851
		% dev	100	97
		SD	1.671	1.154
		n	9	10
Pituitary gland	mg	M	9.156	8.77
		% dev	100	96
		SD	0.794	1.046
		n	9	10
Spleen	g	M	0.532	0.545
		% dev	100	102
		SD	0.062	0.077
		n	9	10
Thyroid glands	mg	M	21.122	21.9
		% dev	100	104
		SD	3.597	3.637
		n	9	10

*: P <= 0.05, **: P <= 0.01
Wilcoxon test, two sided

ABSOLUTE WEIGHTS - MEAN VALUES (MALE) SEX ORGANS
COMPARISON OF GROUP 0 WITH GROUP 4 (FLUTAMIDE)
SUBSET 3 (POSTNATAL DAY 83 +/- 2 DAYS)

Sacrifice			R3	
Sex			M	
Group			0	4
Bulbo-urethral gland	mg	M	74.867	71.91
		% dev	100	96
		SD	20.09	13.308
		n	9	10
Cauda epididymis	mg	M	324.667	330.0
		% dev	100	102
		SD	31.914	36.056
		n	9	10
Epididymides	mg	M	897.111	914.5
		% dev	100	102
		SD	80.093	52.505
		n	9	10
Glans penis	mg	M	89.878	85.09
		% dev	100	95
		SD	10.305	6.802
		n	9	10
Muscles bulb + l.ani	mg	M	711.167	674.29
		% dev	100	95
		SD	83.305	111.897
		n	9	10
Prostate	mg	M	642.556	600.93
		% dev	100	94
		SD	61.158	86.794
		n	9	10
Prostate ventr.fresh	mg	M	347.578	325.95
		% dev	100	94
		SD	52.268	55.203
		n	9	10
Seminal vesicle	mg	M	989.233	862.33
		% dev	100	87
		SD	176.964	187.161
		n	9	10
Testes	mg	M	3530.222	3484.2
		% dev	100	99
		SD	253.004	214.774
		n	9	10

*: P <= 0.05, **: P <= 0.01
Wilcoxon test, one sided

ABSOLUTE WEIGHTS - MEAN VALUES (FEMALE)
COMPARISON OF GROUP 0 WITH GROUPS 1,2 AND 3 (MIX)
SUBSET 3 (POSTNATAL DAY 83 +/- 2 DAYS)

Sacrifice			R3			
Sex			F			
Group			0	1	2	3
Terminal body weight	g	M	196.978	200.45	208.14	202.35
		% dev	100	102	106	103
		SD	14.122	14.077	13.504	11.604
		n	9	10	10	10
Adrenal glands	mg	M	63.556	59.6	66.2	67.6
		% dev	100	94	104	106
		SD	12.126	6.753	9.964	7.677
		n	9	10	10	10
Brain	g	M	1.841	1.891	1.862	1.784
		% dev	100	103	101	97
		SD	0.056	0.089	0.074	0.058
		n	9	10	10	10
Kidneys	g	M	1.523	1.513	1.559	1.57
		% dev	100	99	102	103
		SD	0.146	0.211	0.148	0.083
		n	9	10	10	10
Liver	g	M	6.577	6.992	7.507	7.478*
		% dev	100	106	114	114
		SD	0.829	0.732	0.826	0.619
		n	9	10	10	10
Ovaries	mg	M	94.111	96.6	106.0	102.2
		% dev	100	103	113	109
		SD	8.667	12.747	16.892	17.035
		n	9	10	10	10
Pituitary gland	mg	M	10.333	10.64	10.91	7.83 **
		% dev	100	103	106	76
		SD	2.009	0.765	1.623	1.26
		n	9	10	10	10
Spleen	g	M	0.444	0.445	0.507	0.465
		% dev	100	100	114	105
		SD	0.071	0.048	0.12	0.059
		n	9	10	10	10
Thyroid glands	mg	M	18.189	17.74	20.11	20.91
		% dev	100	98	111	115
		SD	3.747	2.95	1.557	2.433
		n	9	10	10	10

*: P <= 0.05, **: P <= 0.01
Kruskal-Wallis H and Wilcoxon test, two sided

ABSOLUTE WEIGHTS - MEAN VALUES (FEMALE)
COMPARISON OF GROUP 0 WITH GROUPS 1,2 AND 3 (MIX)
SUBSET 3 (POSTNATAL DAY 83 +/- 2 DAYS)

Sacrifice			R3			
Sex			F			
Group			0	1	2	3
Uterus	mg	M	454.778	546.0	662.445	565.6
		% dev	100	120	146	124
		SD	98.673	216.411	266.359	236.459
		n	9	10	9	10

*: P <= 0.05, **: P <= 0.01
Kruskal-Wallis H and Wilcoxon test, two sided

ABSOLUTE WEIGHTS - MEAN VALUES (FEMALE)
COMPARISON OF GROUP 0 WITH GROUP 4 (FLUTAMIDE)
SUBSET 3 (POSTNATAL DAY 83 +/- 2 DAYS)

Sacrifice			R3	
Sex			F	
Group			0	4
Terminal body weight	g	M	196.978	204.17
		% dev	100	104
		SD	14.122	16.63
		n	9	10
Adrenal glands	mg	M	63.556	62.8
		% dev	100	99
		SD	12.126	5.865
		n	9	10
Brain	g	M	1.841	1.889
		% dev	100	103
		SD	0.056	0.07
		n	9	10
Kidneys	g	M	1.523	1.58
		% dev	100	104
		SD	0.146	0.133
		n	9	10
Liver	g	M	6.577	6.772
		% dev	100	103
		SD	0.829	0.67
		n	9	10
Ovaries	mg	M	94.111	97.1
		% dev	100	103
		SD	8.667	13.932
		n	9	10
Pituitary gland	mg	M	10.333	11.16
		% dev	100	108
		SD	2.009	1.747
		n	9	10
Spleen	g	M	0.444	0.454
		% dev	100	102
		SD	0.071	0.083
		n	9	10
Thyroid glands	mg	M	18.189	19.13
		% dev	100	105
		SD	3.747	2.079
		n	9	10

*: P <= 0.05, **: P <= 0.01
Wilcoxon test, two sided

ABSOLUTE WEIGHTS - MEAN VALUES (FEMALE)
COMPARISON OF GROUP 0 WITH GROUP 4 (FLUTAMIDE)
SUBSET 3 (POSTNATAL DAY 83 +/- 2 DAYS)

Sacrifice			R3	
Sex			F	
Group			0	4
Uterus	mg	M	454.778	566.2
		% dev	100	125
		SD	98.673	201.377
		n	9	10

*: P <= 0.05, **: P <= 0.01
Wilcoxon test, two sided

RELATIVE WEIGHTS - MEAN VALUES (MALE)
COMPARISON OF GROUP 0 WITH GROUPS 1,2 AND 3 (MIX)
SUBSET 3 (POSTNATAL DAY 83 +/- 2 DAYS)

Sacrifice			R3			
Sex			M			
Group			0	1	2	3
Terminal body weight	%	M	100.0	100.0	100.0	100.0
		% dev	100	100	100	100
		n	9	10	10	10
Adrenal glands	%	M	0.017	0.019	0.016	0.019
		% dev	100	110	95	110
		SD	0.003	0.004	0.002	0.002
		n	9	10	10	10
Brain	%	M	0.618	0.653	0.625	0.649
		% dev	100	106	101	105
		SD	0.042	0.057	0.058	0.065
		n	9	10	10	10
Kidneys	%	M	0.692	0.717	0.738	0.741
		% dev	100	104	107	107
		SD	0.033	0.043	0.08	0.039
		n	9	10	10	10
Liver	%	M	3.539	3.43	3.5	3.812
		% dev	100	97	99	108
		SD	0.368	0.159	0.192	0.245
		n	9	10	10	10
Pituitary gland	%	M	0.003	0.003	0.003	0.003
		% dev	100	100	95	94
		SD	0.0	0.0	0.0	0.0
		n	9	10	10	10
Spleen	%	M	0.17	0.171	0.171	0.182
		% dev	100	101	101	107
		SD	0.025	0.02	0.021	0.027
		n	9	10	10	10
Thyroid glands	%	M	0.007	0.007	0.007	0.008
		% dev	100	107	99	115
		SD	0.001	0.001	0.001	0.002
		n	9	10	10	10

*: P <= 0.05, **: P <= 0.01
Kruskal-Wallis H and Wilcoxon test, two sided

RELATIVE WEIGHTS - MEAN VALUES (MALE) SEX ORGANS
COMPARISON OF GROUP 0 WITH GROUPS 1,2 AND 3 (MIX)
SUBSET 3 (POSTNATAL DAY 83 +/-2 DAYS)

Sacrifice			R3			
Sex			M			
Group			0	1	2	3
Terminal body weight	%	M	100.0	100.0	100.0	100.0
		% dev	100	100	100	100
		n	9	10	10	10
Bulbo-urethral gland	%	M	0.024	0.023	0.02 *	0.014**
		% dev	100	97	84	59
		SD	0.006	0.003	0.002	0.004
		n	9	10	10	10
Cauda epididymis	%	M	0.103	0.102	0.093	0.092
		% dev	100	99	90	90
		SD	0.011	0.014	0.011	0.01
		n	9	10	10	10
Epididymides	%	M	0.285	0.294	0.296	0.278
		% dev	100	103	104	98
		SD	0.025	0.025	0.075	0.028
		n	9	10	10	10
Glans penis	%	M	0.029	0.029	0.028	0.025
		% dev	100	102	98	86
		SD	0.004	0.004	0.002	0.005
		n	9	10	10	10
Muscles bulb + l.ani	%	M	0.226	0.222	0.203	0.149**
		% dev	100	98	90	66
		SD	0.033	0.025	0.029	0.02
		n	9	10	10	10
Prostate	%	M	0.204	0.198	0.2	0.147**
		% dev	100	97	98	72
		SD	0.018	0.017	0.014	0.026
		n	9	10	10	10
Prostate ventr.fresh	%	M	0.11	0.105	0.109	0.08 **
		% dev	100	95	99	72
		SD	0.016	0.015	0.01	0.014
		n	9	10	10	10
Seminal vesicle	%	M	0.313	0.305	0.277	0.204**
		% dev	100	97	88	65
		SD	0.05	0.037	0.036	0.033
		n	9	10	10	10

*: P <= 0.05, **: P <= 0.01
Kruskal-Wallis H and Wilcoxon test, one sided

Reproductive Toxicity Study to detect potential effects to anti-androgenic substances in Wistar Rats (Gavage) 28.Mar.2014 SIGR

RELATIVE WEIGHTS - MEAN VALUES (MALE) SEX ORGANS
COMPARISON OF GROUP 0 WITH GROUPS 1,2 AND 3 (MIX)
SUBSET 3 (POSTNATAL DAY 83 +/-2 DAYS)

Sacrifice			R3			
Sex			M			
Group			0	1	2	3
Testes	%	M	1.123	1.152	1.099	1.207
		% dev	100	103	98	107
		SD	0.114	0.105	0.146	0.121
		n	9	10	10	10

*: P <= 0.05, **: P <= 0.01
Kruskal-Wallis H and Wilcoxon test, one sided

Reproductive Toxicity Study to detect potential effects to anti-androgenic substances in Wistar Rats (Gavage)

28.Mar.2014 SIGR

RELATIVE WEIGHTS - MEAN VALUES (MALE)
COMPARISON OF GROUP 0 WITH GROUP 4 (FLUTAMIDE)
SUBSET 3 (POSTNATAL DAY 83 +/- 2 DAYS)

Sacrifice			R3	
Sex			M	
Group			0	4
Terminal body weight	%	M	100.0	100.0
		% dev	100	100
		n	9	10
Adrenal glands	%	M	0.017	0.017
		% dev	100	102
		SD	0.003	0.003
		n	9	10
Brain	%	M	0.618	0.621
		% dev	100	100
		SD	0.042	0.053
		n	9	10
Kidneys	%	M	0.692	0.69
		% dev	100	100
		SD	0.033	0.036
		n	9	10
Liver	%	M	3.539	3.362
		% dev	100	95
		SD	0.368	0.169
		n	9	10
Pituitary gland	%	M	0.003	0.003
		% dev	100	94
		SD	0.0	0.0
		n	9	10
Spleen	%	M	0.17	0.169
		% dev	100	99
		SD	0.025	0.016
		n	9	10
Thyroid glands	%	M	0.007	0.007
		% dev	100	101
		SD	0.001	0.001
		n	9	10

*: P <= 0.05, **: P <= 0.01
Wilcoxon test, two sided

RELATIVE WEIGHTS - MEAN VALUES (MALE) SEX ORGANS
COMPARISON OF GROUP 0 WITH GROUP 4 (FLUTAMIDE)
SUBSET 3 (POSTNATAL DAY 83 +/- 2 DAYS)

Sacrifice			R3	
Sex			M	
Group			0	4
Terminal body weight	%	M	100.0	100.0
		% dev	100	100
		n	9	10
Bulbo-urethral gland	%	M	0.024	0.022
		% dev	100	94
		SD	0.006	0.004
		n	9	10
Cauda epididymis	%	M	0.103	0.103
		% dev	100	99
		SD	0.011	0.011
		n	9	10
Epididymides	%	M	0.285	0.285
		% dev	100	100
		SD	0.025	0.021
		n	9	10
Glans penis	%	M	0.029	0.027
		% dev	100	93
		SD	0.004	0.003
		n	9	10
Muscles bulb + l.ani	%	M	0.226	0.209
		% dev	100	92
		SD	0.033	0.028
		n	9	10
Prostate	%	M	0.204	0.187 *
		% dev	100	92
		SD	0.018	0.026
		n	9	10
Prostate ventr.fresh	%	M	0.11	0.101
		% dev	100	92
		SD	0.016	0.016
		n	9	10
Seminal vesicle	%	M	0.313	0.267 *
		% dev	100	85
		SD	0.05	0.052
		n	9	10

*: P <= 0.05, **: P <= 0.01
Wilcoxon test, one sided

Reproductive Toxicity Study to detect potential effects to anti-androgenic substances in Wistar Rats (Gavage)
28.Mar.2014 SIGR

RELATIVE WEIGHTS - MEAN VALUES (MALE) SEX ORGANS
COMPARISON OF GROUP 0 WITH GROUP 4 (FLUTAMIDE)
SUBSET 3 (POSTNATAL DAY 83 +/- 2 DAYS)

Sacrifice			R3	
Sex			M	
Group			0	4
Testes	%	M	1.123	1.088
		% dev	100	97
		SD	0.114	0.119
		n	9	10

*: P <= 0.05, **: P <= 0.01
Wilcoxon test, one sided

RELATIVE WEIGHTS - MEAN VALUES (FEMALE)
COMPARISON OF GROUP 0 WITH GROUPS 1,2 AND 3 (MIX)
SUBSET 3 (POSTNATAL DAY 83 +/- 2 DAYS)

Sacrifice			R3			
Sex			F			
Group			0	1	2	3
Terminal body weight	%	M	100.0	100.0	100.0	100.0
		% dev	100	100	100	100
		n	9	10	10	10
Adrenal glands	%	M	0.032	0.03	0.032	0.033
		% dev	100	92	99	104
		SD	0.005	0.002	0.004	0.003
		n	9	10	10	10
Brain	%	M	0.938	0.947	0.896	0.884
		% dev	100	101	96	94
		SD	0.058	0.072	0.046	0.056
		n	9	10	10	10
Kidneys	%	M	0.773	0.753	0.748	0.777
		% dev	100	97	97	100
		SD	0.045	0.065	0.037	0.04
		n	9	10	10	10
Liver	%	M	3.343	3.487	3.602	3.7
		% dev	100	104	108	111
		SD	0.379	0.247	0.271	0.306
		n	9	10	10	10
Ovaries	%	M	0.048	0.049	0.051	0.051
		% dev	100	102	107	106
		SD	0.003	0.009	0.008	0.008
		n	9	10	10	10
Pituitary gland	%	M	0.005	0.005	0.005	0.004**
		% dev	100	102	100	74
		SD	0.001	0.0	0.001	0.001
		n	9	10	10	10
Spleen	%	M	0.225	0.222	0.242	0.23
		% dev	100	99	108	102
		SD	0.03	0.02	0.047	0.027
		n	9	10	10	10
Thyroid glands	%	M	0.009	0.009	0.01	0.01
		% dev	100	97	105	113
		SD	0.002	0.002	0.001	0.001
		n	9	10	10	10

*: P <= 0.05, **: P <= 0.01
Kruskal-Wallis H and Wilcoxon test, two sided

RELATIVE WEIGHTS - MEAN VALUES (FEMALE)
COMPARISON OF GROUP 0 WITH GROUPS 1,2 AND 3 (MIX)
SUBSET 3 (POSTNATAL DAY 83 +/- 2 DAYS)

Sacrifice			R3			
Sex			F			
Group			0	1	2	3
Uterus	%	M	0.229	0.272	0.316	0.28
		% dev	100	119	137	122
		SD	0.038	0.106	0.124	0.118
		n	9	10	9	10

*: P <= 0.05, **: P <= 0.01
Kruskal-Wallis H and Wilcoxon test, two sided

RELATIVE WEIGHTS - MEAN VALUES (FEMALE)
COMPARISON OF GROUP 0 WITH GROUP 4 (FLUTAMIDE)
SUBSET 3 (POSTNATAL DAY 83 +/- 2 DAYS)

Sacrifice			R3	
Sex			F	
Group			0	4
Terminal body weight	%	M	100.0	100.0
		% dev	100	100
		n	9	10
Adrenal glands	%	M	0.032	0.031
		% dev	100	96
		SD	0.005	0.003
		n	9	10
Brain	%	M	0.938	0.93
		% dev	100	99
		SD	0.058	0.078
		n	9	10
Kidneys	%	M	0.773	0.775
		% dev	100	100
		SD	0.045	0.053
		n	9	10
Liver	%	M	3.343	3.327
		% dev	100	100
		SD	0.379	0.327
		n	9	10
Ovaries	%	M	0.048	0.048
		% dev	100	100
		SD	0.003	0.007
		n	9	10
Pituitary gland	%	M	0.005	0.005
		% dev	100	104
		SD	0.001	0.001
		n	9	10
Spleen	%	M	0.225	0.224
		% dev	100	100
		SD	0.03	0.048
		n	9	10
Thyroid glands	%	M	0.009	0.009
		% dev	100	102
		SD	0.002	0.001
		n	9	10

*: P <= 0.05, **: P <= 0.01
Wilcoxon test, two sided

Reproductive Toxicity Study to detect potential effects to anti-androgenic substances in Wistar Rats (Gavage) 28.Mar.2014 SIGR

RELATIVE WEIGHTS - MEAN VALUES (FEMALE)
COMPARISON OF GROUP 0 WITH GROUP 4 (FLUTAMIDE)
SUBSET 3 (POSTNATAL DAY 83 +/- 2 DAYS)

Sacrifice			R3	
Sex			F	
Group			0	4
Uterus	%	M	0.229	0.28
		% dev	100	122
		SD	0.038	0.106
		n	9	10

*: P <= 0.05, **: P <= 0.01
Wilcoxon test, two sided

INCIDENCE OF GROSS LESIONS
COMPARISON OF GROUP 0 WITH GROUPS 1,2 AND 3 (MIX)
PARENTAL FEMALES

Sacrifice	F1			
Sex	F			
Group	0	1	2	3
Animals in selected group	20	20	20	20
No abnormalities	20	18	20	19
Abdominal cavity	.	.	.	.
Effusion	.	1	.	.
Forestomach	.	.	.	.
Erosion/ulcer	.	.	.	1
Glandular stomach	.	.	.	.
Erosion/ulcer	.	.	.	1
Liver	.	.	.	.
Torsion	.	1	.	.
Ovaries	.	.	.	.
Cyst	.	1	.	.

INCIDENCE OF GROSS LESIONS
COMPARISON OF GROUP 0 WITH GROUP 4 (FLUTAMIDE)
PARENTAL FEMALES

Sacrifice	F1	
Sex	F	
Group	0	4
Animals in selected group	20	19
No abnormalities	20	19

INCIDENCE OF GROSS LESIONS
COMPARISON OF GROUP 0 WITH GROUPS 1,2 AND 3 (MIX)
SUBSET 1 (POSTNATAL DAY 21)

Sacrifice	R1							
Sex	M				F			
Group	0	1	2	3	0	1	2	3
Animals in selected group	10	10	10	10	10	10	10	10
No abnormalities	10	10	10	10	10	10	10	10

INCIDENCE OF GROSS LESIONS
COMPARISON OF GROUP 0 WITH GROUP 4 (FLUTAMIDE)
SUBSET 1 (POSTNATAL DAY 21)

Sacrifice	R1			
Sex	M		F	
Group	0	4	0	4
Animals in selected group	10	10	10	10
No abnormalities	10	10	10	10

INCIDENCE OF GROSS LESIONS
COMPARISON OF GROUP 0 WITH GROUPS 1,2 AND 3 (MIX)
SUBSET 2 (SEXUAL MATURITY)

Sacrifice	R2							
Sex	M				F			
Group	0	1	2	3	0	1	2	3
Animals in selected group	10	10	10	10	10	10	10	10
No abnormalities	10	10	9	9	8	10	8	9
Kidneys	.	.	.	.	.	.	.	.
Cyst	.	.	1	.	.	.	.	.
Ovaries	.	.	.	.	.	.	.	.
Cyst	.	.	.	.	1	.	.	.
Oviducts	.	.	.	.	.	.	.	.
Cyst	.	.	.	.	.	.	.	1
Pericard	.	.	.	.	.	.	.	.
Dilation	.	.	.	.	.	.	1	.
Right epididymis	.	.	.	.	.	.	.	.
Enlarged	.	.	.	1	.	.	.	.
Uterus	.	.	.	.	.	.	.	.
Dilation	.	.	.	.	1	.	1	.

INCIDENCE OF GROSS LESIONS
COMPARISON OF GROUP 0 WITH GROUP 4 (FLUTAMIDE)
SUBSET 2 (SEXUAL MATURITY)

Sacrifice	R2			
Sex	M		F	
Group	0	4	0	4
Animals in selected group	10	10	10	10
No abnormalities	10	9	8	9
Kidneys	.	.	.	.
Pelvic dilation	.	1	.	.
Ovaries	.	.	.	.
Cyst	.	.	1	.
Uterus	.	.	.	.
Dilation	.	.	1	1

INCIDENCE OF GROSS LESIONS
COMPARISON OF GROUP 0 WITH GROUPS 1,2 AND 3 (MIX)
SUBSET 3 (POSTNATAL DAY 83 +/- 2 DAYS)

Sacrifice	R3							
Sex	M				F			
Group	0	1	2	3	0	1	2	3
Animals in selected group	10	10	10	10	10	10	10	10
No abnormalities	9	10	10	10	10	9	9	10
Kidneys	.	.	.	.	.	.	.	.
Cyst	.	.	.	.	.	.	1	.
Skeletal muscle	.	.	.	.	.	.	.	.
Effusion	1	.	.	.	.	.	.	.
Thoracic cavity	.	.	.	.	.	.	.	.
Effusion	1	.	.	.	.	.	.	.
Uterus	.	.	.	.	.	.	.	.
Discoloration	.	.	.	.	.	1	.	.
Mass	.	.	.	.	.	1	.	.

INCIDENCE OF GROSS LESIONS
COMPARISON OF GROUP 0 WITH GROUP 4 (FLUTAMIDE)
SUBSET 3 (POSTNATAL DAY 83 +/- 2 DAYS)

Sacrifice	R3			
Sex	M		F	
Group	0	4	0	4
Animals in selected group	10	10	10	10
No abnormalities	9	9	10	10
Kidneys	.	.	.	.
Cyst	.	1	.	.
Skeletal muscle	.	.	.	.
Effusion	1	.	.	.
Thoracic cavity	.	.	.	.
Effusion	1	.	.	.

Reproductive Toxicity Study to detect potential effects to anti-androgenic substances in Wistar Rats (Gavage) 28.Mar.2014 SIGR

INCIDENCE OF ALL MICROSCOPIC FINDINGS
SUBSET 1 (POSTNATAL DAY 21)

Sacrifice		R1				
Sex		M				
Group		0	1	2	3	4
Animals in selected group		10	10	10	10	10
Adrenal cortex	exam.	9	10	10	10	10
Adrenal medulla	exam.	9	10	10	10	10
Coagulating glands	exam.	10	10	10	10	10
Immature stage		10	10	10	10	10
Left epididymis	exam.	10	10	10	10	10
Immature stage		10	10	10	10	10
Left testicle	exam.	10	10	10	10	10
Immature stage		10	10	10	10	10
Pituitary gland	exam.	7	9	8	10	10
Prostata ventr fixed	exam.	10	10	10	10	10
Immature stage		10	10	10	10	10
Secretion		10	10	10	10	10
Prostate, d.l. fixed	exam.	9	10	8	9	8
Immature stage		9	10	8	9	8
Seminal vesicle	exam.	10	10	10	10	10
Immature stage		10	10	10	10	10

INCIDENCE OF ALL MICROSCOPIC FINDINGS
SUBSET 2 (SEXUAL MATURITY)

Sacrifice		R2				
Sex		M				
Group		0	1	2	3	4
Animals in selected group		10	10	10	10	10
Adrenal cortex	exam.	10	10	10	10	10
Adrenal medulla	exam.	10	10	10	10	10
Coagulating glands	exam.	9	10	10	10	10
Juvenile stage with secretio		9	10	10	10	10
Kidneys	exam.	.	.	1	.	1
Dilation, renal pelvis		.	.	.	.	1
Cyst(s)		.	.	1	.	.
Left epididymis	exam.	9	10	10	10	10
Juvenile stage		3	4	4	.	4
Juvenile stage, with sperms		6	6	6	.	6
Juveno-adult transition, wit		.	.	.	10	.
Oligospermia		.	.	.	10	.
Left testicle	exam.	9	10	10	10	10
Spermatogenic cycle, devel.		9	10	10	10	10
Leydig cells, increased		.	.	.	1	.
Pituitary gland	exam.	10	10	10	10	10
Cyst(s), craniopharyngeal		1	.	.	.	.
Prostata ventr fixed	exam.	9	10	10	10	10
Juvenile stage with secretio		9	10	10	10	10
Prostate, d.l. fixed	exam.	9	10	10	10	10
Juvenile stage with secretio		9	10	10	10	10
Right epididymis	exam.	.	.	.	1	.
Edema		.	.	.	1	.
Seminal vesicle	exam.	10	10	10	10	10
Juvenile stage with secretio		10	10	10	10	10

Reproductive Toxicity Study to detect potential effects to anti-androgenic substances in Wistar Rats (Gavage) 28.Mar.2014 SIGR

INCIDENCE OF ALL MICROSCOPIC FINDINGS
SUBSET 3 (POSTNATAL DAY 83+/-2)

Sacrifice		R3				
Sex		M				
Group		0	1	2	3	4
Animals in selected group		10	10	10	10	10
Adrenal cortex	exam.	10	10	10	10	10
Adrenal medulla	exam.	10	10	10	10	10
Coagulating glands	exam.	10	10	10	10	10
Juvenile stage with secretio		1	.	.	.	.
Kidneys	exam.	.	.	.	.	1
Cyst(s)		.	.	.	.	1
Left epididymis	exam.	10	10	10	10	10
Immature stage		1	.	.	.	.
Left testicle	exam.	10	10	10	10	10
Immature stage		1	.	.	.	.
Degeneration, tubular, (m)f		1	1	2	1	.
Pituitary gland	exam.	10	10	10	10	10
Prostata ventr fixed	exam.	10	10	10	10	10
Juvenile stage with secretio		1	.	.	.	.
Infiltration, lymphoid, (m)f		1	.	.	1	.
Prostate, d.l. fixed	exam.	10	10	10	10	10
Juvenile stage with secretio		1	.	.	.	.
Seminal vesicle	exam.	10	10	10	10	10
Juvenile stage with secretio		1	.	.	.	.

INCIDENCE AND GRADING OF SELECTED MICROSCOPIC FINDINGS
SUBSET 1 (POSTNATAL DAY 21)

Sacrifice		R1				
Sex		M				
Group		0	1	2	3	4
Animals in selected group		10	10	10	10	10
Coagulating glands	exam.	10	10	10	10	10
Immature stage		10	10	10	10	10
	. P.	10	10	10	10	10
Left epididymis	exam.	10	10	10	10	10
Immature stage		10	10	10	10	10
	. P.	10	10	10	10	10
Left testicle	exam.	10	10	10	10	10
Immature stage		10	10	10	10	10
	. P.	10	10	10	10	10
Prostata ventr fixed	exam.	10	10	10	10	10
Immature stage		10	10	10	10	10
	. P.	10	10	10	10	10
Secretion		10	10	10	10	10
	. 1.	10	10	10	10	10
Prostate, d.l. fixed	exam.	9	10	8	9	8
Immature stage		9	10	8	9	8
	. P.	9	10	8	9	8
Seminal vesicle	exam.	10	10	10	10	10
Immature stage		10	10	10	10	10
	. P.	10	10	10	10	10

Reproductive Toxicity Study to detect potential effects to anti-androgenic substances in Wistar Rats (Gavage) 28.Mar.2014 SIGR

INCIDENCE AND GRADING OF SELECTED MICROSCOPIC FINDINGS
SUBSET 2 (SEXUAL MATURITY)

Sacrifice	R2				
Sex	M				
Group	0	1	2	3	4
Animals in selected group	10	10	10	10	10
Coagulating glands exam.	9	10	10	10	10
Juvenile stage with secretio	9	10	10	10	10
. P.	9	10	10	10	10
Left epididymis exam.	9	10	10	10	10
Juvenile stage	3	4	4	.	4
. P.	3	4	4	.	4
Juvenile stage, with sperms	6	6	6	.	6
. 1.	4	6	3	.	4
. 2.	2	.	1	.	1
. 3.	.	.	2	.	1
Juveno-adult transition, wit	.	.	.	10	.
. 3.	.	.	.	3	.
. 4.	.	.	.	4	.
. 5.	.	.	.	3	.
Oligospermia	.	.	.	10	.
. 1.	.	.	.	3	.
. 2.	.	.	.	4	.
. 3.	.	.	.	3	.
Left testicle exam.	9	10	10	10	10
Spermatogenic cycle, devel.	9	10	10	10	10
. P.	9	10	10	10	10
Leydig cells, increased	.	.	.	1	.
. 1.	.	.	.	1	.
Prostata ventr fixed exam.	9	10	10	10	10
Juvenile stage with secretio	9	10	10	10	10
. P.	9	10	10	10	10
Prostate, d.l. fixed exam.	9	10	10	10	10
Juvenile stage with secretio	9	10	10	10	10
. P.	9	10	10	10	10
Seminal vesicle exam.	10	10	10	10	10
Juvenile stage with secretio	10	10	10	10	10
. P.	10	10	10	10	10

INCIDENCE AND GRADING OF SELECTED MICROSCOPIC FINDINGS
SUBSET 3 (POSTNATAL DAY 83+/-2)

Sacrifice		R3				
Sex		M				
Group		0	1	2	3	4
Animals in selected group		10	10	10	10	10
Coagulating glands	exam.	10	10	10	10	10
Juvenile stage with secretio		1	.	.	.	.
	. P.	1	.	.	.	.
Left epididymis	exam.	10	10	10	10	10
Immature stage		1	.	.	.	.
	. P.	1	.	.	.	.
Left testicle	exam.	10	10	10	10	10
Immature stage		1	.	.	.	.
	. P.	1	.	.	.	.
Degeneration, tubular, (m)f		1	1	2	1	.
	. 1.	1	1	2	1	.
Prostata ventr fixed	exam.	10	10	10	10	10
Juvenile stage with secretio		1	.	.	.	.
	. P.	1	.	.	.	.
Infiltration, lymphoid, (m)f		1	.	.	1	.
	. 1.	1	.	.	1	.
Prostate, d.l. fixed	exam.	10	10	10	10	10
Juvenile stage with secretio		1	.	.	.	.
	. P.	1	.	.	.	.
Seminal vesicle	exam.	10	10	10	10	10
Juvenile stage with secretio		1	.	.	.	.
	. P.	1	.	.	.	.
