# Supplementary material for: Investigations on the dose–response relationship of combined exposure to low doses of three anti-androgens in Wistar rats
Source: Arch Toxicol. 2017 Sep 6;91(12):3961–89. doi: 10.1007/s00204-017-2053-3 (PMC5719133; doi:10.1007/s00204-017-2053-3)
Supplement: Supplementary file 7 — Supplementary material 7 (PDF 9532 kb) [file 204_2017_2053_MOESM7_ESM.pdf]

BATTELLE
UK LTD

Battelle UK Ltd.

Langstone Technology Park, Langstone Road, Havant, Hants, PO9 1SA, UK

Certificate of Analysis

**BAS-No. :**	**BAS 352 F**	**Batch No. :**	**N183TOX**
**Formulation Type :**	TGAI	**Date of Production :**	March 1988
**Date of Initial Analysis :**	August 2011	**Study Code :**	MX/11/007/1

Ingredient<br>Name / BAS Number	Analyzed Content	Determination by
Vinclozolin / BAS 352 F	99.1% w/w	GC

Homogeneity: Given

Identity: Confirmed

Additional Information

Storage Advice : Keep at ambient temperature (+5 to +30 °C)

Expiration Date : 01 August 2016

Recipients should ensure that the label information on the corresponding substance container(s) corresponds(s) with that on this certificate of analysis

**Study Director :**	L.J.Stickland	**Study Completion Date :**	13 September 2011
**Issued on :**	13 September 2011	**Issued by :**	[signature]

This document has been computer generated and is based on the GLP-studies cited above
Released by L.J.Stickland

03/0525-6 erg. 25.09.09 La

Certificate of Analysis

SIGMA-ALDRICH

**Product Name**	Flutamide
**Product Number**	F9397
**Product Brand**	SIGMA
**CAS Number**	13311-84-7
**Molecular Formula**	$C_{11}H_{11}F_3N_2O_3$
**Molecular Weight**	276.21

**TEST**	**SPECIFICATION**	**LOT 069K1581 RESULTS**
**Appearance (Color)**	Yellow	Yellow
**Appearance (Form)**	Powder	Powder
**Solubility (Color)**	Yellow to Yellow-Green	Yellow - Green
**Solubility (Turbidity)]**	Clear to Hazy	Clear
	At 50 mg/ml in EtOH	
**Carbon**	46.8 - 49.8 %	47.8 %
**Nitrogen**	9.8 - 10.4 %	10.0 %
**Purity (TLC)**	≥99 %	100 %
**Specification Date:**		MAR 2009

Rodney Burbach

Rodney Burbach, Manager
Quality Control
St. Louis, Missouri USA

04/0611 - 4
[illegible]

BASF
The Chemical Company

BASF SE
APR/DA - Global Analytics

BASF Agricultural Center Limburgerhof - P.O. Box 120, D-67114 Limburgerhof, Germany

Certificate of Analysis

**BAS Code :**	**BAS 590 F**	**Common Name :**	**Prochloraz**
**Reg.No. :**	**193420**	**Batch No. :**	**COD-000718**
**Substance Type :**	TGAI (=TC)	**Date of Production :**	December 07, 2005
**Date of Initial Analysis :**	January 12, 2006	**Study Code :**	242959_2

Purity : 98.0 % (tolerance ± 1.0%)

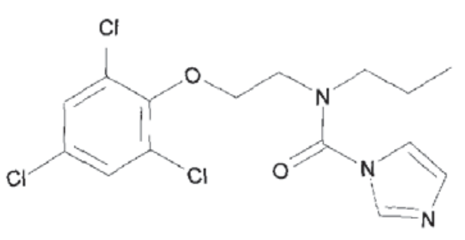

**CL-No.**	182364
**CAS No.**	67747-09-5
**Core Project**	590F
**Internal (Metabolite) Code**	
**Molecular Formula**	$C_{15}H_{16}Cl_3N_3O_2$
**Molecular Weight**	376.7

**IUPAC-Name :**	N-propyl-N-[2-(2,4,6-trichlorophenoxy)ethyl]-1H-imidazole-1-carboxamide
**Determination by :**	HPLC
**Homogeneity :**	given

Additional Information

**Storage Advice :**	keep at ambient temperature (+5 to +30 °C)
**Expiration Date :**	January 31, 2011

Recipients should ensure that the label information on the corresponding substance container(s) correspond(s) with that on this Certificate of Analysis

Study Director : Daum, Ansgar

Study Completion Date : February 17, 2006

Issued on : January 21, 2009

Issued by : [illegible]

This document has been computer generated and is based on the GLP-studies cited above
Released November 04, 2008 by Daum, Ansgar

BASF
The Chemical Company

STUDY TITLE

ANALYTICAL REPORT

BAS 352 F (Vinclozolin)

Stability Analysis in

corn oil

AUTHOR(S)

Dr. Matthias Becker
Dr. Hennicke Kamp

STUDY COMPLETION DATE

13 June 2012

TEST FACILITY

BASF SE
Experimental Toxicology and Ecology
67056 Ludwigshafen, Germany

TEST FACILITY PROJECT IDENTIFICATION

Project No.: 01Y0375/88Y001

SPONSOR

BASF SE
67056 Ludwigshafen, Germany

This document contains manufacturing and trade secrets of the sponsor(s). It is the property of the sponsor(s) and may be used only for that purpose for which it was intended by the sponsor(s). Every other or additional use, exploitation, reproduction, publication or submission to other parties require the written permission of the sponsor(s), with the exception of regulatory agencies acting within the limits of their administrative authority.

This document is the property of the sponsors (specified on page 1), use by other parties requires written permission by the sponsors

THIS PAGE IS INTENTIONALLY LEFT BLANK

BASF
The Chemical Company

GLP COMPLIANCE STATEMENT

This study was conducted in accordance with the OECD Principles of Good Laboratory Practice and the GLP Principles of the German "Chemikaliengesetz" (Chemicals Act) which meet the United States Environmental Protection Agency Good Laboratory Practice Standards [40 CFR Part 160 (FIFRA) and Part 792 (TSCA)], with the exception that recognized differences exist between the GLP Principles/Standards of OECD and the Principles/Standards of FIFRA and TSCA.

Study Director ....[signature].......... Date: 13 June 2012

Typed name of Study Director: Dr. Mathias. Becker

Typed name of Laboratory: BASF SE
Experimental Toxicology and Ecology
67056 Ludwigshafen
Germany

This document is the property of the sponsors (specified on page 1), use by other parties requires written permission by the sponsors

THIS PAGE IS INTENTIONALLY LEFT BLANK

SIGNATURES

Study Director: ...., 13 June 2012
Dr. Becker

Management: ...., 11. June 2012
Dr. Hennicke/Kamp

This document is the property of the sponsors (specified on page 1), use by other parties requires written permission by the sponsors

STATEMENT OF THE QUALITY ASSURANCE UNIT

The Quality Assurance Unit (QAU) inspected the study and reported any inspection results to the Study Director and to Management.

The final report reflects the raw data.

Phase of study	Date of inspection<br><br>(mm-dd-yyyy)	Reported to Study Director and to Management (mm-dd-yyyy)
Study Plan:	04-19-2011	04-19-2011
Conduct of study:	04-28-2011	04-28-2011
Report:	03-13-2012	03-13-2012

Ludwigshafen, 12 Jun 2012

....

M.ABT

This document is the property of the sponsors (specified on page 1), use by other parties requires written permission by the sponsors

STATEMENT OF GLP COMPLIANCE (FROM THE COMPETENT AUTHORITY)

Rheinland Pfalz

Gute Laborpraxis / Good Laboratory Practice

GLP-Bescheinigung / Statement of GLP Compliance

(gem. /according to § 19 Abs. 1 Chemikaliengesetz)

Eine GLP-Inspektion zur Überwachung und der Einhaltung der GLP-Grundsätze gemäß Chemikaliengesetz bzw. Richtlinie 2004/9/EG wurde durchgeführt in:

Assessment of conformity with GLP according to Chemikaliengesetz and Directive 2004/9/EC at::

Prüfeinrichtung / Test facility

BASF SE
Experimentelle Toxikologie und Ökologie
67056 Ludwigshafen

BASF SE
Experimental Toxicology and Ecology
67056 Ludwigshafen, Germany

Prüfung nach Kategorien / Areas of Expertise
(gem. / according ChemVwV-GLP Nr. 5.3/OECD guidance)
1,2,3,4,5,8,9
Kat. 9 – Biochemische und pathologische Untersuchungen zu Wirkmechanismen / Biochemical and pathological examinations concerning mode of action

Datum der Inspektion / Date of Inspection
(Tag.Monat Jahr / day.month year)
19.05.2009 & 06. bis 08.07.2009

Die genannte Prüfeinrichtung befindet sich im nationalen GLP-Überwachungsverfahren und wird regelmäßig auf Einhaltung der GLP-Grundsätze überwacht.

The above mentioned test facility is included in the national GLP Compliance Programme and is inspected on a regular basis.

Auf der Grundlage des Inspektionsberichtes wird hiermit bestätigt, dass in dieser Prüfeinrichtung die oben genannten Prüfungen unter Einhaltung der GLP-Grundsätze durchgeführt werden können.
Eine erneute behördliche Überprüfung der Einhaltung der GLP-Grundsätze durch die Prüfeinrichtung ist so rechtzeitig zu beantragen, dass die Folgeinspektion spätestens vier Jahre nach dem Beginn der o.g. Inspektion stattfinden kann. Ohne diesen Antrag wird die Prüfeinrichtung nach Ablauf der Frist aus dem deutschen GLP-Überwachungsprogramm genommen und diese GLP-Bescheinigung verliert ihre Gültigkeit.

Based on the inspection report it can be confirmed, that the test facility is able to conduct the aforementioned studies in compliance with the Principles of GLP.

Verification of the compliance of the test facility with the Priciples of the GLP has to be applied for in time to allow for a follow-up inspection to take place within four years after commencing the above mentioned inspection. Elapsing this term, the test facility will be taken out of the German GLP-Monitoring Programme and this GLP Certificate becomes invalid.

Unterschrift, Datum / Signature, Date

Dr. Pia Hirsch - stellv. Präsidentin -
(Name und Funktion der verantwortlichen Person / name and function of responsible person)

Siegel

Landesamt für Umwelt, Wasserwirtschaft und Gewerbeaufsicht
Kaiser-Friedrich-Straße 7
55116 Mainz

(Name und Adresse der GLP-Überwachungsbehörde / Name and adress of the GLP Monitoring Authority)

Landesamt für Umwelt, Wasserwirtschaft und Gewerbeaufsicht

This document is the property of the sponsors (specified on page 1), use by other parties requires written permission by the sponsors

CONTENTS

TITLE PAGE
BLANK PAGE
GLP COMPLIANCE STATEMENT
BLANK PAGE
SIGNATURES
STATEMENT OF THE QUALITY ASSURANCE UNIT
STATEMENT OF GLP COMPLIANCE (FROM THE COMPETENT AUTHORITY)
CONTENTS
1. INTRODUCTION
2. RETENTION OF RECORDS
3. TIME SCHEDULE
4. MATERIAL AND METHODS
4.1. TEST ITEM
4.2. SAMPLE DATA
4.3. SAMPLE PREPARATION FOR ANALYSIS
4.4. ANALYTICAL METHOD
5. RESULTS AND DISCUSSION
5.1. ANALYSIS OF STABILITY
FIGURES

This document is the property of the sponsors (specified on page 1), use by other parties requires written permission by the sponsors

1. INTRODUCTION

In the context of toxicological studies the stability of the test item (= test substance) BAS 352 F (Vinclozolin) in the vehicle corn oil has to be verified. The results of these analyses are reported and discussed.

2. RETENTION OF RECORDS

GLP-relevant records and materials are stored at BASF SE for at least the period of time specified in the GLP principles. Details concerning responsibilities or locations of archiving can be seen from the respective SOPs and from the raw data.

3. TIME SCHEDULE

Study initiation date:	18 April 2011
Experimental starting date:	19 April 2011
Experimental completion date:	27 April 2011

4. MATERIAL AND METHODS

4.1. TEST ITEM

The analyses of the test item (= test substance) have been carried out at Battelle UK Ltd. (Langstone Technology Park, Langstone Road, Havant, Hants, PO9 1SA, UK).

The sponsor is responsible for compliance for all test substance information and their storage, except for those test substance investigations commissioned by the test facility.

This document is the property of the sponsors (specified on page 1), use by other parties requires written permission by the sponsors

Name of the test substance:	BAS 352 F (Vinclozolin)
Test substance No.:	88/0375-1
Batch No.:	N183
CAS No.:	50471-44-8
Purity:	99.1 % w/w
	(according to project no: MX/11/007/1)
Homogeneity:	Homogeneous
Stability:	Expiry date: 01 Aug 2016
	The stability of the test substance under storage conditions over the test period was guaranteed by the sponsor, and the sponsor holds this responsibility.

Additional Test Substance Information

Date of production:	16 Mar 1988
Physical state / appearance:	Solid / white
Storage conditions:	Ambient

4.2. SAMPLE DATA

Sponsor:	Dr. Schneider
Vehicle:	Corn oil
Nominal concentration:	0.125 mg/100 mL
Duration of the stability test period:	7 days
Storage conditions of the samples during the stability period:	Ambient

This document is the property of the sponsors (specified on page 1), use by other parties requires written permission by the sponsors

4.3. SAMPLE PREPARATION FOR ANALYSIS

Preparation of the stability sample:
A stock solution was prepared by weighting approximately 50 mg of the test substance into a 100 mL measuring flask which was filled up to the calibration marc with corn. The dilution was sonificated for 10 minutes. 0.25 mL of this solution was diluted with corn oil to a total volume of 100 mL.

1 mL of the stability sample was diluted with acetone to a total volume of 10 mL. Aliquots were used for GC/MS- analysis.

4.4. ANALYTICAL METHOD

GC/MS with external calibration

Column:	HP- 5MS 5% Phenyl Methyl Siloxane (30m x 0.25mm x 0.25µm)
Oven temperature:	150°C, gradient with 10°C/min to 280°C, 12 min at 280°C
Carrier:	Helium
Flow:	0.9 mL/min
Injector temp.:	280°C
Transfer line:	280°C
Split:	100:1
MS- Parameter	
Solvent delay:	3.00 min
MS Quad:	150°C
MS Source:	230°C
SIM- Parameter	
Resolution:	Low
Ions/Dwell (Mass/Dwell):	186.90 / 100; 198.00 / 100; 212.00 / 100; 285.00 / 100
Nominal concentration of the stock solution for the stability preparation:	0h and 7d: 50.4 mg/100 mL corn oil<br>24h: 50.2 mg/100 mL corn oil
Stock solution concentration:	51.0 mg/100 mL (prepared on 20 April 2011)<br>50.9 mg/100 mL (prepared on 27 April 2011)
Storage of the stock solution:	Prepared freshly
Stock standard diluent:	Acetone
Matrix:	10 mL corn oil was diluted with acetone to a total volume of 100 mL.

This document is the property of the sponsors (specified on page 1), use by other parties requires written permission by the sponsors

Chromatographic Standards: For quantification, one standard solution (Kalib 5) was prepared by diluting the stock solutions with matrix. Following four standard solutions (Kalib 1- 4) were prepared by diluting the fifth standard solution with matrix.

External calibration: Under the described chromatographic conditions, aliquots (1 µL) of the standards were analyzed by GC. Record peak response was recorded as area under the curve (abundance). Each sample was analyzed twice. Means were used for quantification. Linearity between concentration and area under the curve was given in the range of the analysis. One calibration curve is shown Figure 1.

Limit of quantification (loq): 0.0636 mg/L (lowest concentration in calibration)

The calibration curve and examples of chromatograms will follow within this report.

5. RESULTS AND DISCUSSION

5.1. ANALYSIS OF STABILITY

The results obtained for the stability of BAS 352 F (Vinclozolin) in corn oil are summarized in the following table:

Nominal concentration [mg/100 mL]	Time after starting	Analytical value [mg/100 mL]			% of nominal value
		Sample I	Sample II	Mean	
0.1260	0h	0.1255	0.1267	0.1261	100.1
0.1255	24h	0.1252	0.1334	0.1293	103.0
0.1260	7d	0.1213	0.1203	0.1208	95.9

Based on the results obtained for the analysis of stability (storage ambient) it is concluded, that BAS 352 F (Vinclozolin) is stable in corn oil over a period of 7 days.
All determined concentrations were in the range between 90 % and 110 % of the nominal concentration.

This document is the property of the sponsors (specified on page 1), use by other parties requires written permission by the sponsors

FIGURES

Figure 1 Standard calibration curve (measured on 27 April 2011)

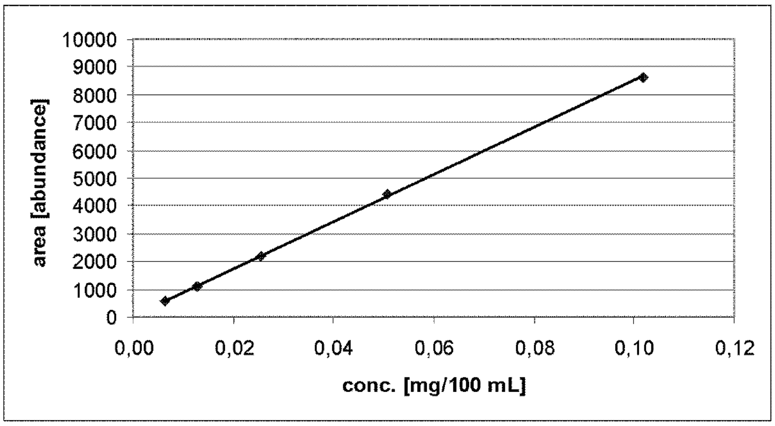

Figure 2 Chromatogram of a blank sample

```
Data Path : C:\msdchem\1\DATA\88-0375\88Y001\27_04_11\
Data File : Matrix 1.D
Acq On    : 27 Apr 2011  13:55
Operator  : 01Y0375_88Y001
Sample    : Matrix
Misc      :
ALS Vial  : 8   Sample Multiplier: 1

Integration Parameters: autoint1.e
Integrator: ChemStation

Method    : C:\msdchem\1\METHODS\88-0375.M
Title     :

Signal     : TIC: Matrix 1.D\data.ms

peak  R.T. first  max last  PK   peak      corr.   corr.    % of
  #   min   scan scan scan  TY  height     area    % max.   total
---  ----- ----- ---- ---- --- -------   -------  ------  -------

    No peaks were detected using the method integration parameters!

88-0375.M Thu Apr 28 06:48:51 2011 3000HANOVER
```

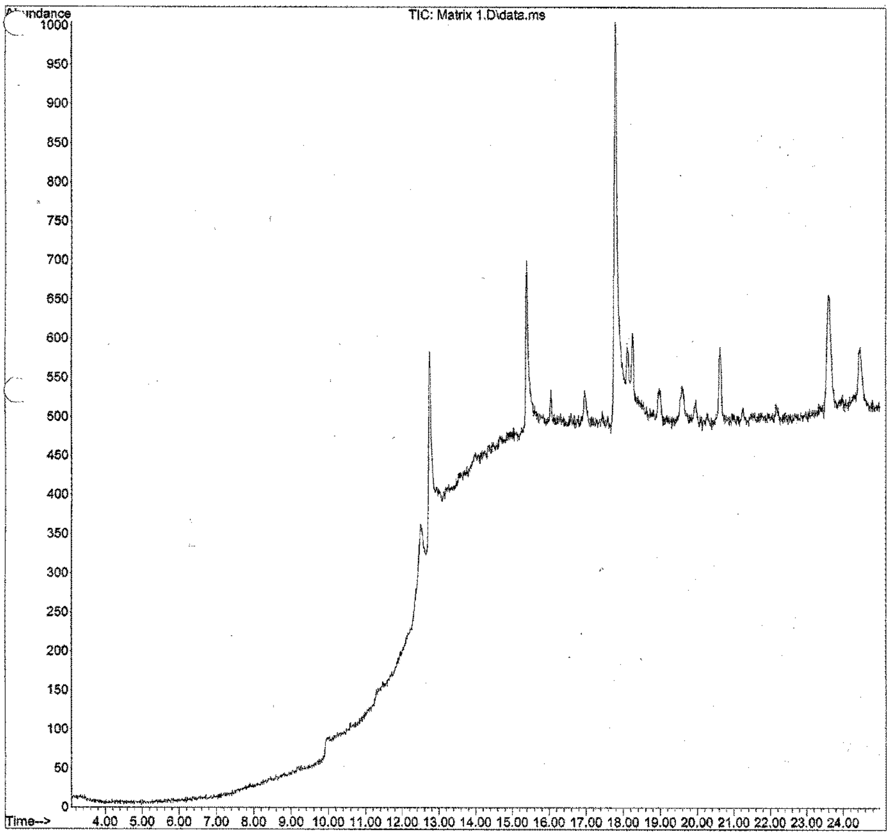

Figure 3 Chromatogram of the standard solution with the lowest substance concentration (limit of quantification 0.0636 mg/L)

```
Data Path : C:\msdchem\1\DATA\88-0375\88Y001\27_04_11\
Data File : Kalib 1 1.D
Acq On    : 27 Apr 2011   7:46
Operator  : 01Y0375_88Y001
Sample    : Kalib 1
Misc      :
ALS Vial  : 1   Sample Multiplier: 1

Integration Parameters: autoint1.e
Integrator: ChemStation

Method    : C:\msdchem\1\METHODS\88-0375.M
Title     :

Signal     : TIC: Kalib 1 1.D\data.ms
```

peak #	R.T. min	first scan	max scan	last scan	PK TY	peak height	corr. area	corr. % max.	% of total
1	7.897	678	683	687	M	33	533	100.00%	100.000%

Sum of corrected areas: 533

88-0375.M Thu Apr 28 06:47:02 2011 3000HANOVER

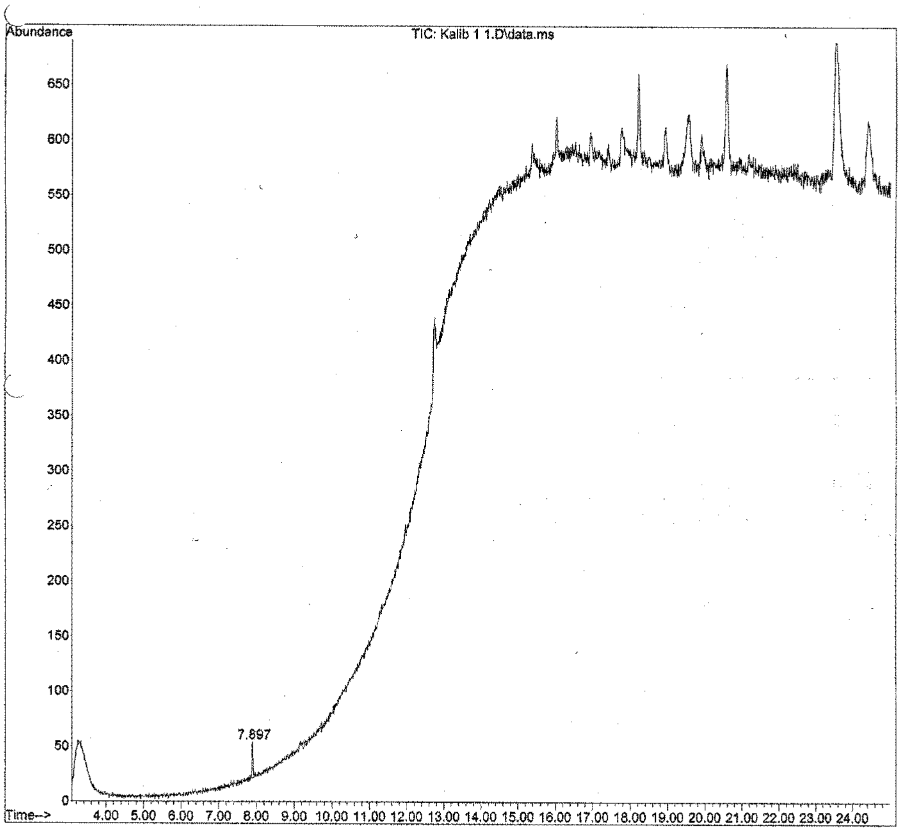

Figure 4 Chromatogram of the sample containing (a nominal value of nominally) 0.1260 mg/100 mL test substance, analyzed immediately after sample preparation

```
Data Path : C:\msdchem\1\DATA\88-0375\88Y001\20_04_11\
Data File : 0h 1.D
Acq On    : 20 Apr 2011  12:12
Operator  : 01Y0375_88Y001
Sample    : 0h
Misc      :
ALS Vial  : 6   Sample Multiplier: 1

Integration Parameters: autoint1.e
Integrator: ChemStation

Method    : C:\msdchem\1\METHODS\88-0375.M
Title     :

Signal     : TIC: 0h 1.D\data.ms
```

peak #	R.T. min	first scan	max scan	last scan	PK TY	peak height	corr. area	corr. % max.	% of total
1	7.872	675	679	684	M2	67	1098	100.00%	100.000%

Sum of corrected areas: 1098

88-0375.M Wed Apr 20 14:22:01 2011 3000HANOVER

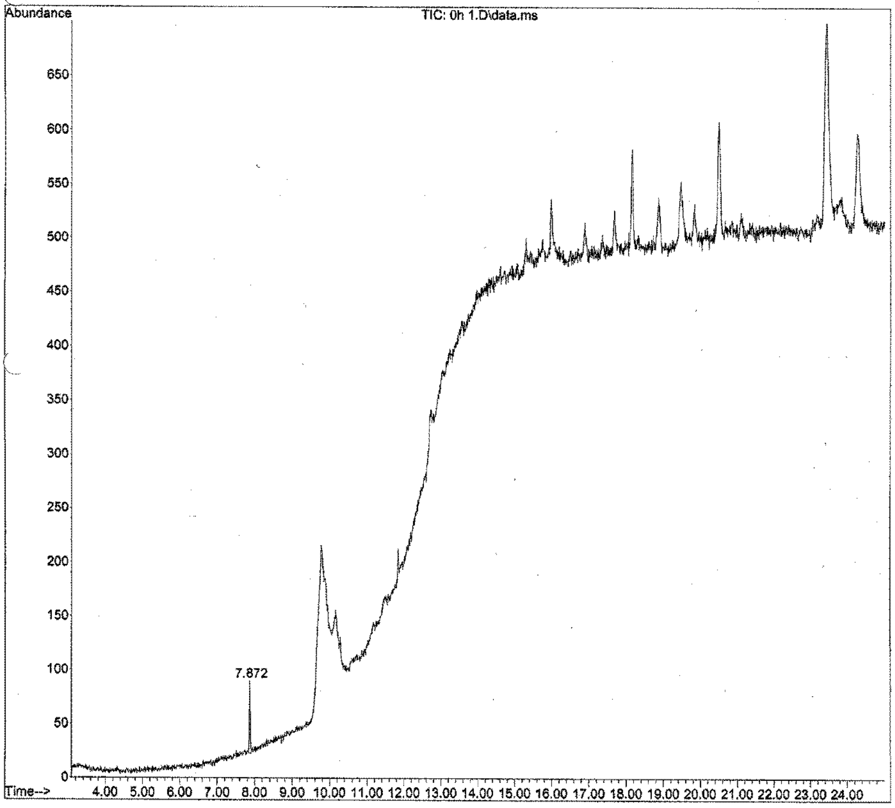

This document is the property of the sponsors (specified on page 1), use by other parties requires written permission by the sponsors

Figure 5 Chromatogram of the sample containing (a nominal value of nominally) 0.1260 mg/100 mL test substance, analyzed approximately 7 days after sample preparation

```
Data Path : C:\msdchem\1\DATA\88-0375\88Y001\27_04_11\
Data File : 7d 1.D
Acq On    : 27 Apr 2011  12:30
Operator  : 01Y0375_88Y001
Sample    : 7d
Misc      :
ALS Vial  : 6   Sample Multiplier: 1

Integration Parameters: autoint1.e
Integrator: ChemStation

Method    : C:\msdchem\1\METHODS\88-0375.M
Title     :

Signal     : TIC: 7d 1.D\data.ms
```

peak #	R.T. min	first scan	max scan	last scan	PK TY	peak height	corr. area	corr. % max.	% of total
1	7.910	680	685	691	M	64	1074	100.00%	100.000%

Sum of corrected areas: 1074

88-0375.M Thu Apr 28 06:48:31 2011 3000HANOVER

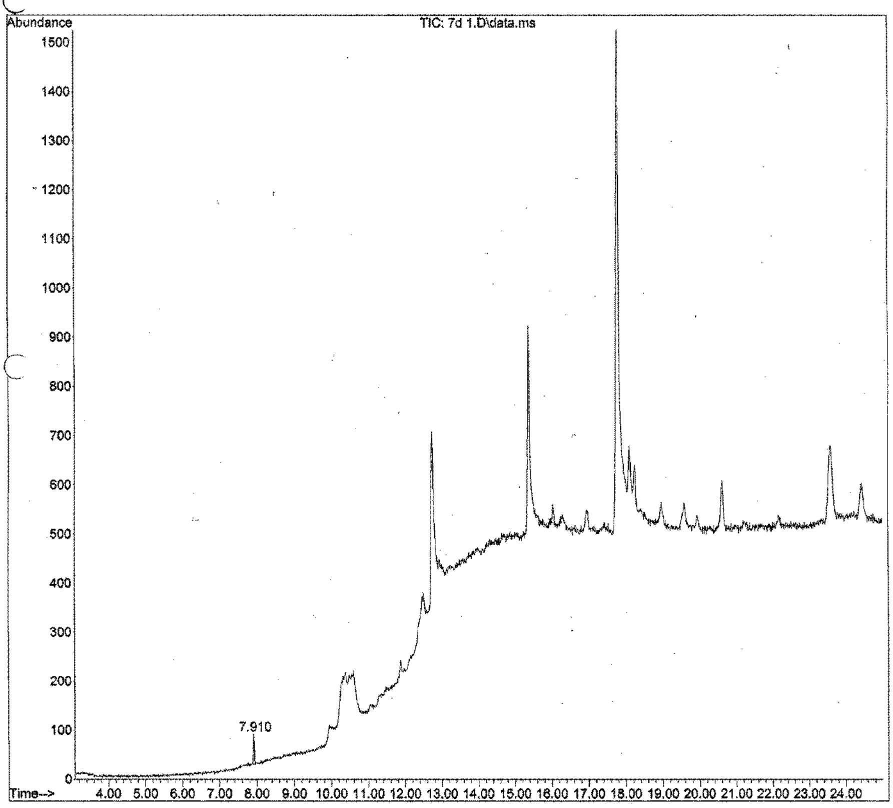

The Chemical Company

STUDY TITLE

ANALYTICAL REPORT

Flutamide

Stability Analysis in

Corn oil

AUTHOR

Dr. Matthias Becker
Dr. Hennicke Kamp

STUDY COMPLETED ON

08 August 2011

Test Facility

Experimental Toxicology and Ecology
BASF SE
67056 Ludwigshafen, Germany

TEST FACILITY PROJECT IDENTIFICATION

Project No.: 01Y0525/03Y003

SPONSOR

BASF SE
67056 Ludwigshafen, Germany

This document contains manufacturing and trade secrets of the sponsor(s). It is the property of the sponsor(s) and may be used only for that purpose for which it was intended by the sponsor(s). Every other or additional use, exploitation, reproduction, publication or submission to other parties require the written permission of the sponsor(s), with the exception of regulatory agencies acting within the limits of their administrative authority.

.

This document is the property of the sponsors (specified on page 1), use by other parties requires written permission by the sponsors

THIS PAGE IS INTENTIONALLY LEFT BLANK

GLP COMPLIANCE STATEMENT

This study was conducted in accordance with the OECD Principles of Good Laboratory Practice and the GLP Principles of the German "Chemikaliengesetz" (Chemicals Act) which meet the United States Environmental Protection Agency Good Laboratory Practice Standards [40 CFR Part 160 (FIFRA) and Part 792 (TSCA)], with the exception that recognized differences exist between the GLP Principles/Standards of OECD and the Principles/Standards of FIFRA and TSCA.

Study Director .... 08 Aug 2011
Dr. M. Becker Date

THIS PAGE IS INTENTIONALLY LEFT BLANK

SIGNATURE PAGE

Study Director:

08 Aug 2011
Dr. Matthias Becker

Management:

08 Aug 2011
Dr. Hennicke Kamp

This document is the property of the sponsors (specified on page 1), use by other parties requires written permission by the sponsors

STATEMENT OF THE QUALITY ASSURANCE UNIT

The Quality Assurance Unit (QAU) performed the inspections given below, and reported any inspection results to the Study Director and to Management. The conduct of this short-term study was not inspected; the processes of the laboratory and of the study involved are inspected in regular intervals.

The final report reflects the raw data.

Phase of study	Date of inspection (mm-dd-yyyy)	Reported to Study Director and to Management (mm-dd-yyyy)
Study Plan:	04-19-2011	04-19-2011
Report:	07-25-2011	07-25-2011

Ludwigshafen, 08 August 2011

Zachmann

GLP CERTIFICATE (FROM THE COMPETENT AUTHORITY)

RheinlandPfalz

Gute Laborpraxis / Good Laboratory Practice

GLP-Bescheinigung / Statement of GLP Compliance
(gem. /according to § 19 Abs. 1 Chemikaliengesetz)

Eine GLP-Inspektion zur Überwachung und der Einhaltung der GLP-Grundsätze gemäß Chemikaliengesetz bzw. Richtlinie 2004/9/EG wurde durchgeführt in:

Assessment of conformity with GLP according to Chemikaliengesetz and Directive 2004/9/EC at::

Prüfeinrichtung / Test facility

BASF SE
Experimentelle Toxikologie und Ökologie
67056 Ludwigshafen

BASF SE
Experimental Toxicology and Ecology
67056 Ludwigshafen, Germany

Prüfung nach Kategorien / Areas of Expertise
(gem. / according ChemVwV-GLP Nr. 5.3/OECD guidance)
1,2,3,4,5,8,9
Kat. 9 – Biochemische und pathologische Untersuchungen zu Wirkmechanismen / Biochemical and pathological examinations concerning mode of action

Datum der Inspektion / Date of Inspection
(Tag.Monat Jahr / day.month year)
19.05.2009 & 06. bis 08.07.2009

Die genannte Prüfeinrichtung befindet sich im nationalen GLP-Überwachungsverfahren und wird regelmäßig auf Einhaltung der GLP-Grundsätze überwacht.

The above mentioned test facility is included in the national GLP Compliance Programme and is inspected on a regular basis.

Auf der Grundlage des Inspektionsberichtes wird hiermit bestätigt, dass in dieser Prüfeinrichtung die oben genannten Prüfungen unter Einhaltung der GLP-Grundsätze durchgeführt werden können.
Eine erneute behördliche Überprüfung der Einhaltung der GLP-Grundsätze durch die Prüfeinrichtung ist so rechtzeitig zu beantragen, dass die Folgeinspektion spätestens vier Jahre nach dem Beginn der o.g. Inspektion stattfinden kann. Ohne diesen Antrag wird die Prüfeinrichtung nach Ablauf der Frist aus dem deutschen GLP-Überwachungsprogrann genommen und diese GLP-Bescheinigung verliert ihre Gültigkeit:

Based on the inspection report it can be confirmed, that the test facility is able to conduct the aforementioned studies in compliance with the Principles of GLP.

Verification of the compliance of the test facility with the Pricipies of the GLP has to be applied for in time to allow for a follow-up inspection to take place within four years after commencing the above mentioned inspection. Elapsing this term, the test facility will be taken out of the German GLP-Monitoring Programme and this GLP Certificate becomes invalid.

Unterschrift, Datum / Signature, Date

[signature] 22.10.2009
Dr. Pia Hirsch - stellv. Präsidentin -
(Name und Funktion der verantwortlichen Person / name and function of responsible person)

Landesamt für Umwelt, Wasserwirtschaft und Gewerbeaufsicht 1 Rheinland-Pfalz
Siegel

Landesamt für Umwelt, Wasserwirtschaft und Gewerbeaufsicht
Kaiser-Friedrich-Straße 7
55116 Mainz
(Name und Adresse der GLP-Überwachungsbehörde / Name and adress of the GLP Monitoring Authority)

Landesamt für Umwelt, Wasserwirtschaft und Gewerbeaufsicht

CONTENTS

TITLE PAGE
BLANK PAGE
GLP COMPLIANCE STATEMENT
BLANK PAGE
SIGNATURE PAGE
STATEMENT OF THE QUALITY ASSURANCE UNIT
GLP CERTIFICATE (FROM THE COMPETENT AUTHORITY)
CONTENTS
1. INTRODUCTION
2. RETENTION OF RECORDS
3. TIME SCHEDULE
4. MATERIAL AND METHODS
4.1. TEST ITEM
4.2. SAMPLE DATA
4.3. TEST SUBSTANCE PREPARATION
4.4. SAMPLE PREPARATION AND ANALYSIS
4.5. LIST OF DEVIATIONS
4.5.1. List of deviations from the control procedure
5. RESULTS AND DISCUSSION
5.1. ANALYSIS OF STABILITY
5.2. DISCUSSION
6. APPENDIX
6.1. CONTROL PROCEDURE 03/0525_01-01

1. INTRODUCTION

In the context of toxicological studies the stability of the test substance Flutamide in the vehicle corn oil has to be verified. The results of these analyses are reported and discussed.

2. RETENTION OF RECORDS

GLP-relevant records and materials are stored at BASF SE for at least the period of time specified in the GLP principles. Details concerning responsibilities or locations of archiving can be seen from the respective SOPs and from the raw data.

3. TIME SCHEDULE

Study initiation date:	18 April 2011
Experimental starting date:	01 June 2011
Experimental completion date:	08 June 2011

4. MATERIAL AND METHODS

4.1. TEST ITEM

The test item (= test substance) was used with the given specifications of the producer (Sigma-Aldrich, Taufkirchen, Germany). No further analyses were conducted.

This document is the property of the sponsors (specified on page 1), use by other parties requires written permission by the sponsors

Name of test substance:	Flutamide
Test substance No.:	03/0525-8
Batch identification:	021M1406V
CAS No.:	13311-84-7
Purity:	100 %
Homogeneity:	Given
Storage stability:	stable until: 13 Apr 2014<br>The stability of the test substance under storage conditions over the test period was guaranteed by the manufacturer, and the manufacturer holds this responsibility.

Additional Test Substance Information

Date of production:	Unknown
Physical state/ Appearance:	Solid / yellow
Storage conditions:	ambient

4.2. SAMPLE DATA

Sponsor:	Dr. Schneider
Vehicle:	Corn oil
Target concentration:	0.0625 mg / 100 mL
Duration of the stability test period:	7 days
Storage conditions of the samples during the stability period:	ambient

4.3. TEST SUBSTANCE PREPARATION

61.7 mg of the test substance were dissolved in 100 ml corn oil. This solution was further diluted 1:1000 with corn oil. The final nominal concentration was 0.0617 mg / 100 mL.

This document is the property of the sponsors (specified on page 1), use by other parties requires written permission by the sponsors

4.4. SAMPLE PREPARATION AND ANALYSIS

The sample preparation and analysis of the test substance was carried out according to the valid control procedure 03/0525_01-01.

A detailed description of the control procedure is given in the appendix of this report.

4.5. LIST OF DEVIATIONS

4.5.1. List of deviations from the control procedure

There was one deviation from the described control procedure 03/0525_01-01. The system-suitability solution (stability analysis on day 7) exceeded slightly the specification of 95 % to 105 %. The determined values were 108.5 % and 108.9 %. Considering the very low analytical concentration and the variability of the analytical method, this result can be regarded as acceptable with no significant impact on the final analytical results.

5. RESULTS AND DISCUSSION

5.1. ANALYSIS OF STABILITY

The results obtained for the stability of the test substance in Corn oil are summarized in the following table.

**Nominal concentration [mg / 100 mL]**	**Time after starting**	**Concentration found [mg / 100 mL]**	**Nominal concentration (%)**
0.0617	0	0.06025	97.6
0.0617	5 days	0.06750	109.4
0.0617	7 days	0.06200	100.5

5.2. DISCUSSION

Based on the analytical results it is concluded, that Flutamide is stable in corn oil over a period of 7 days at ambient temperature. All determined concentrations were in range between 90 % and 110 % of the nominal concentration.

This document is the property of the sponsors (specified on page 1), use by other parties requires written permission by the sponsors

6. APPENDIX

6.1. CONTROL PROCEDURE 03/0525_01-01

BASF SE
Test Facility
Experimental Toxicology and Ecology / Analytical Chemistry

BASF The Chemical Company

CONTROL TEST

Test substance number: 03/0525	No.: 03/0525_01-01
Name test substance: Flutamide	Effective from: 01.06.2011
Control procedure: Content (LC-MS) / Corn oil	Page 1 of 6

Technique LC-MS

System: Waters 2695 HPLC with autosampler, Waters 2487 UV, Micromass Quattro Micro LC-MS, MassLynx – Software (Waters), or equivalent system

Column: Length: 100 mm
Inner diameter: 2 mm

Stationary Phase: Luna C-18, 3 µm, Phenomenex or equivalent

Mobile Phase A: 1000 mL acetonitrile are mixed with 1 mL formic acid

Mobile Phase B: 1000 mL water are mixed with 1 mL formic acid

Gradient:

Time (min)	Mobile Phase A	Mobile Phase B
0	30	70
7	90	10
10	90	10
10.5	30	70
15	30	70

Injection volume: 10 µL

Flow rate: 0.4 mL/min

Detection: MS, ESI (negative)
SIM (single ion monitoring)
Ions (m/z): 275.0

Column temperature: ambient

Run time: approx. 15 min

BASF SE
Test Facility
Experimental Toxicology and Ecology / Analytical Chemistry

BASF
The Chemical Company

CONTROL TEST

Test substance number: 03/0525	No.: 03/0525_01-01
Name test substance: Flutamide	Effective from: 01.06.2011
Control procedure: Content (LC-MS) / Corn oil	Page 2 of 6

Ms parameter:

**Source (ES-)**	**Settings**
Capillary (kV)	3.50
Cone (V)	40.00
Extractor (V)	2.00
RF Lens (V)	0.0
Source Temperature (°C)	120
Desolvation Temperature (°C)	250
Cone Gas Flow (L/Hr)	50
Desolvation Gas Flow (L/Hr)	650

**Analyser**	**Settings**
LM 1 Resolution	14.0
HM 1 Resolution	14.0
Ion Energy 1	0.5
Entrance	50
Collision	2
Exit	50
LM 2 Resolution	15.0
HM 2 Resolution	15.0
Ion Energy 2	3.0
Multiplier (V)	650
Syringe Pump Flow (uL/min)	10.0

This document is the property of the sponsors (specified on page 1), use by other parties requires written permission by the sponsors

BASF SE
Test Facility
Experimental Toxicology and Ecology / Analytical Chemistry

BASF
The Chemical Company

CONTROL TEST

Test substance number: 03/0525	No.: 03/0525_01-01
Name test substance: Flutamide	Effective from: 01.06.2011
Control procedure: Content (LC-MS) / Corn oil	Page 3 of 6

Sample solution: Samples are diluted completely with acetone using appropriate volumetric flasks to obtain sample solutions with test substance concentrations that match the calibration range. The samples have to be diluted at least 1:25, v/v. If required, all dilutions are sonicated for 5 minutes to ensure a complete dissolution of the test substance.

Annotation: If the amount of test substance in the sample solution is outside the calibration range (calibration solutions 1 – 5), an adequate dilution step with matrix solution has to be performed to match the described concentration range.

Matrix solution: The preparation of the matrix solution has to be performed according to the procedure described for sample solution preparation

Stock solution: Approx. 50 mg test substance are dissolved to a final volume of 100 mL with acetone (50 mg/100 mL). This solution is further diluted 1:100 with acetone (0.5 mg/100 mL).

Calibration solution 1: 0.2 mL stock solution are diluted with matrix solution to 100 mL (0.001 mg/100 mL)
Calibration solution 2: 0.5 mL stock solution are diluted with matrix solution to 100 mL (0.0025 mg/100 mL)
Calibration solution 3: 1.0 mL stock solution are diluted with matrix solution to 100 mL (0.005 mg/100 mL)
Calibration solution 4: 1.0 mL stock solution are diluted with matrix solution to 50 mL (0.010 mg/100 mL)
Calibration solution 5: 1.0 mL stock solution are diluted with matrix solution to 25 mL (0.020 mg/100 mL)

System-suitability solution:
System-suitability solution is prepared with a second independent weighting according to calibration solution 3 (0.005 mg/100mL)

Procedure: After conditioning the LC-MS system, sample solutions, matrix solution, calibration solutions and system-suitability solution are injected according to the sequence described in the raw data. All solutions are injected at least once.

Retention time:

Test substance: Flutamide:
approx. 6.1 min

System suitability:

The calculated content of the system-suitability solution, has to be in the range from 95 % to 105 %.

The coefficient of determination (R^2) has to be ≥ 0.990. If the correlation coefficient (R) is used, this value has to be ≥ 0.995.

Calculation:

The concentration control measurements are based on external calibration (calibration solutions 1 – 5).

The calculation of the content as well as the recovery in percent of test substance in corn oil is performed electronically (e.g. Dionex Chromeleon – Software, Microsoft Excel). Basic formulas for calculations are described below.

Formulas:

Calibration curve

$Y = a \cdot x + b$

a = slope of calibration curve
b = intercept

Analysed concentration (C_A)

$$C_A = \frac{(Y-b)}{a} \bullet \frac{V \bullet d}{w}$$

w = weight sample
V = final sample volume
d = dilution factor

BASF SE
Test Facility
Experimental Toxicology and Ecology / Analytical Chemistry

The Chemical Company

CONTROL TEST

Test substance number: 03/0525	No.: 03/0525_01-01
Name test substance: Flutamide	Effective from: 01.06.2011
Control procedure: Content (LC-MS) / Corn oil	Page 5 of 6

Figure 1.1: Chromatogram matrix solution (01 June 2011, Project no.: 01Y0525/03Y003)

Name: 03Y003_2011-06-01_003, Vial: 75, Date: 01-Jun-2011, Time: 10:58:15, Description: Matrix

Flutamide

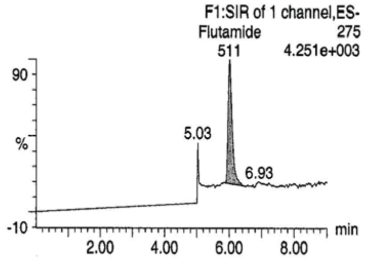

Figure 1.2: Chromatogram calibration solution 3 (01 June 2011, Project no.: 01Y0525/03Y003)

Name: 03Y003_2011-06-01_007, Vial: 78, Date: 01-Jun-2011, Time: 12:16:21, Description: Kalib 3

ɹtamide

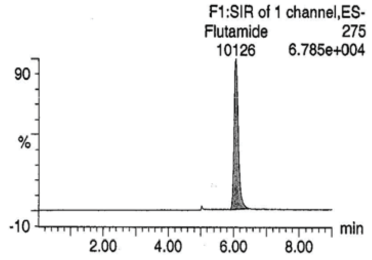

BASF SE
Test Facility
Experimental Toxicology and Ecology / Analytical Chemistry

The Chemical Company

CONTROL TEST

Test substance number: 03/0525	No.: 03/0525_01-01
Name test substance: Flutamide	Effective from: 01.06.2011
Control procedure: Content (LC-MS) / Corn oil	Page 6 of 6

Figure 1.3 Chromatogram of a representative sample solution (sample Stab 0h, 01 June 2011, Project no.: 01Y0525/03Y003))

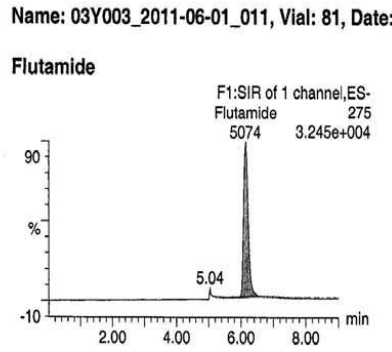

Figure 1.4 calibration curve (01 June 2011, concentration range: 0.001 - 0.020 mg/100 mL, Project no.: 01Y0525/03Y003)

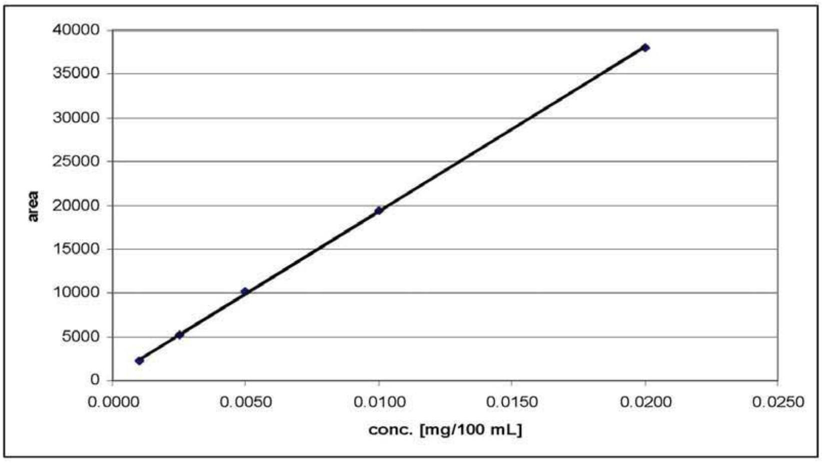

The Chemical Company

STUDY TITLE

ANALYTICAL REPORT

BAS 590 F (Prochloraz)

Stability Analysis in

corn oil

AUTHOR

Dr. Becker
Dr. Hennicke Kamp

STUDY COMPLETED ON

15 December 2011

Test Facility

Experimental Toxicology and Ecology
BASF SE
67056 Ludwigshafen, Germany

TEST FACILITY PROJECT IDENTIFICATION

Project No.: 01Y0611/04Y011

SPONSOR

BASF SE
67056 Ludwigshafen, Germany

This document contains manufacturing and trade secrets of the sponsor(s). It is the property of the sponsor(s) and may be used only for that purpose for which it was intended by the sponsor(s). Every other or additional use, exploitation, reproduction, publication or submission to other parties require the written permission of the sponsor(s), with the exception of regulatory agencies acting within the limits of their administrative authority.

.

THIS PAGE IS INTENTIONALLY LEFT BLANK

GLP COMPLIANCE STATEMENT

This study was conducted in accordance with the OECD Principles of Good Laboratory Practice and the GLP Principles of the German "Chemikaliengesetz" (Chemicals Act) which meet the United States Environmental Protection Agency Good Laboratory Practice Standards [40 CFR Part 160 (FIFRA) and Part 792 (TSCA)], with the exception that recognized differences exist between the GLP Principles/Standards of OECD and the Principles/Standards of FIFRA and TSCA.

M. Becker 15 Dec 2011
Dr. Matthias Becker
(Study Director)

THIS PAGE IS INTENTIONALLY LEFT BLANK

SIGNATURES

Study Director:

15. Dec 2011
Dr. Matthias Becker

Management:

15. Dec 2011
Dr. Hennicke Kamp

STATEMENT OF THE QUALITY ASSURANCE UNIT

The Quality Assurance Unit (QAU) performed the inspections given below, and reported any inspection results to the Study Director and to Management. The conduct of this short-term study was not inspected; the processes of the laboratory and of the study involved are inspected in regular intervals.

The final report reflects the raw data.

Phase of study	Date of inspection (mm-dd-yyyy)	Reported to Study Director and to Management (mm-dd-yyyy)
Study Plan:	04-19-2011	04-19-2011
Report:	08-17-2011<br>12-14-2011	08-17-2011<br>12-14-2011

Ludwigshafen, 15 December 2011 N. Keller

N. Keller

STATEMENT OF GLP COMPLIANCE (FROM THE COMPETENT AUTHORITY)

RheinlandPfalz

Gute Laborpraxis / Good Laboratory Practice

GLP-Bescheinigung / Statement of GLP Compliance

(gem. /according to § 19 Abs. 1 Chemikaliengesetz)

Eine GLP-Inspektion zur Überwachung und der Einhaltung der GLP-Grundsätze gemäß Chemikaliengesetz bzw. Richtlinie 2004/9/EG wurde durchgeführt in:

Assessment of conformity with GLP according to Chemikaliengesetz and Directive 2004/9/EC at::

Prüfeinrichtung / Test facility

BASF SE
Experimentelle Toxikologie und Ökologie
67056 Ludwigshafen

BASF SE
Experimental Toxicology and Ecology
67056 Ludwigshafen, Germany

Prüfung nach Kategorien / Areas of Expertise
(gem. / according ChemVwV-GLP Nr. 5.3/OECD guidance)
1,2,3,4,5,8,9
Kat. 9 – Biochemische und pathologische Untersuchungen zu Wirkmechanismen / Biochemical and pathological examinations concerning mode of action

Datum der Inspektion / Date of Inspection
(Tag.Monat Jahr / day.month year)
19.05.2009 & 06. bis 08.07.2009

Die genannte Prüfeinrichtung befindet sich im nationalen GLP-Überwachungsverfahren und wird regelmäßig auf Einhaltung der GLP-Grundsätze überwacht.

The above mentioned test facility is included in the national GLP Compliance Programme and is inspected on a regular basis.

Auf der Grundlage des Inspektionsberichtes wird hiermit bestätigt, dass in dieser Prüfeinrichtung die oben genannten Prüfungen unter Einhaltung der GLP-Grundsätze durchgeführt werden können.
Eine erneute behördliche Überprüfung der Einhaltung der GLP-Grundsätze durch die Prüfeinrichtung ist so rechtzeitig zu beantragen, dass die Folgeinspektion spätestens vier Jahre nach dem Beginn der o.g. Inspektion stattfinden kann. Ohne diesen Antrag wird die Prüfeinrichtung nach Ablauf der Frist aus dem deutschen GLP-Überwachungsprogrann genommen und diese GLP-Bescheinigung verliert ihre Gültigkeit:

Based on the inspection report it can be confirmed, that the test facility is able to conduct the aforementioned studies in compliance with the Principles of GLP.

Verification of the compliance of the test facility with the Priciples of the GLP has to be applied for in time to allow for a follow-up inspection to take place within four years after commencing the above mentioned inspection. Elapsing this term, the test facility will be taken out of the German GLP-Monitoring Programme and this GLP Certificate becomes invalid.

Unterschrift, Datum / Signature, Date

[signature] 22.10.2009
Dr. Pia Hirsch - stellv. Präsidentin -
(Name und Funktion der verantwortlichen Person / name and function of responsible person)

Landesamt für Umwelt, Wasserwirtschaft und Gewerbeaufsicht 1 Rheinland-Pfalz
Siegel

Landesamt für Umwelt, Wasserwirtschaft und Gewerbeaufsicht
Kaiser-Friedrich-Straße 7
55116 Mainz

(Name und Adresse der GLP-Überwachungsbehörde / Name and adress of the GLP Monitoring Authority)

Landesamt für Umwelt, Wasserwirtschaft und Gewerbeaufsicht

CONTENTS

TITLE PAGE
BLANK PAGE
GLP COMPLIANCE STATEMENT
BLANK PAGE
SIGNATURES
STATEMENT OF THE QUALITY ASSURANCE UNIT
STATEMENT OF GLP COMPLIANCE (FROM THE COMPETENT AUTHORITY)
CONTENTS
1. INTRODUCTION
2. RETENTION OF RECORDS
3. TIME SCHEDULE
4. MATERIAL AND METHODS
4.1. TEST ITEM
4.2. SAMPLE DATA
4.3. SAMPLE PREPARATION FOR ANALYSIS
4.4. ANALYTICAL METHOD
5. RESULTS AND DISCUSSION
5.1. ANALYSIS OF STABILITY
FIGURES

1. INTRODUCTION

In the context of toxicological studies the stability of the test item (= test substance) BAS 590 F (Prochloraz) in the vehicle corn oil has to be verified. The results of these analyses are reported and discussed.

2. RETENTION OF RECORDS

GLP-relevant records and materials are stored at BASF SE for at least the period of time specified in the GLP principles. Details concerning responsibilities or locations of archiving can be seen from the respective SOPs and from the raw data.

3. TIME SCHEDULE

Study initiation date:	18 Apr 2011
Experimental starting date:	19 Apr 2011
Experimental completion date:	26 Apr 2011

4. MATERIAL AND METHODS

4.1. TEST ITEM

The analyses of the test item (= test substance) were carried out at the test facility Crop Protection - Ecology and Environmental Analytics of BASF SE, Speyerer Straße 2, 67117 Limburgerhof, Germany.

Name of the test substance:	BAS 590 F (Prochloraz)
Test substance No.:	04/0611-4
Batch No.:	COD-000718
CAS No.:	67747-09-5
Purity/Composition:	98.0 +- 1 %<br>(according to project no:242959_2)
Homogeneity:	Given

This document is the property of the sponsors (specified on page 1), use by other parties requires written permission by the sponsors

Stability:	Expiry date: 14 Apr 2016<br>The stability of the test substance under storage conditions over the test period was guaranteed by the sponsor, and the sponsor holds this responsibility.

Additional Test Substance Information

Date of production:	07 Dec 2005
Physical state / appearance:	Solid / melt, brownish
Storage conditions:	Ambient / light exclusion

4.2. SAMPLE DATA

Sender:	Dr. Schneider
Vehicle:	Corn oil
Nominal concentration:	0.25 mg/100 mL
Duration of the stability test period:	7 days
Storage conditions of the samples during the stability period:	Ambient

4.3. SAMPLE PREPARATION FOR ANALYSIS

Approximately 50 mg of the test substance were weighed into a 100 mL measuring flask and filled up to the calibration mark with corn oil. Under heat supply, using a magnetic stirrer, the test substance was dissolved completely, further diluted with corn oil up to the required concentration.

At given instants 1 mL of the sample was diluted in a ratio of 1+9 with acetone.
Aliquots of the dilutions were used for HPLC/MS-analysis.

4.4. ANALYTICAL METHOD

HPLC/MS with external calibration

Column:	Luna 3µ C18 (2), 100 x 2 mm
Eluent:	A: Acetonitrile + formic acid (1,000 mL + 1 mL)<br>B: Highly deionized water + formic acid (1,000 mL + 1 mL)
Flow rate:	0.4 mL/min
Column temperature:	Ambient
Injection volume:	10 µL
MS Conditions:	MS, ES+<br>ETU: Mass 376, Dwell 0.5 s, Cone 30 V

Tune Parameter:

Source (ES+)	Settings
Capillary (kV)	3.20
Cone (V)	30.00
Extractor (V)	2.00
RF Lens (V)	0.0
Source Temperature (°C)	120
Desolvation Temperature (°C)	250
Cone Gas Flow (L/Hr)	50
Desolvation Gas Flow (L/Hr)	650

Analyser	Settings
LM 1 Resolution	15.0
HM 1 Resolution	15.0
Ion Energy 1	0.2
Entrance	50
Collision	2
Exit	50
LM 2 Resolution	15.0
HM 2 Resolution	15.0
Ion Energy 2	3.0
Multiplier (V)	650
Syringe Pump Flow (uL/min)	10.0

Pressure Gauges	
Gas Cell Pirani Pressure(mbar)	< 1e-4 mBars

Nominal concentration for the stability preparation	0h: 0.249 mg/100 mL corn oil
Stock solution concentration:	50.2 mg/100 mL (prepared on 19 Apr 2011)<br>49.6 mg/100 mL (prepared on 20 Apr 2011)<br>49.8 mg/100 mL (prepared on 26 Apr 2011)
Stock standard diluent	Acetone
Chromatographic Standards:	For quantification, three standard solutions (Kalib 1-3) were prepared by diluting the stock solution with stock standard diluent.

This document is the property of the sponsors (specified on page 1), use by other parties requires written permission by the sponsors

External calibration: Under the described chromatographic conditions, aliquots (10 µL) of the standards were analyzed by HPLC/MS. Record peak response was recorded as area under the curve (mAU). Each sample was analyzed twice. Means were used for quantification. Linearity between concentration and area under the curve was given in the range of the analysis. One calibration curve is shown Figure 1.

Figures of the calibration curve and examples of chromatograms will follow within this report.

5. RESULTS AND DISCUSSION

5.1. ANALYSIS OF STABILITY

The results obtained for the stability of BAS 590 F (Prochloraz) in corn oil are summarized in the following table:

Nominal concentration [mg/100 mL]	Time after starting	Analytical value [mg/100 mL]			% of nominal value
		Sample I	Sample II	Mean	
0.249	0h	0.2491	0.2427	0.2459	98.8
	4h	0.2512	0.2509	0.2511	100.8
	24h	0.2584	0.2615	0.2600	104.4
	7d	0.2408	0.2592	0.2500	100.4

Based on the results obtained for the analysis of stability (storage ambient) it is concluded, that BAS 590 F (Prochloraz) is stable in corn oil over a period of 7 days.

This document is the property of the sponsors (specified on page 1), use by other parties requires written permission by the sponsors

FIGURES

Figure 1 Standard calibration curve (measured on 20 Apr 2011)

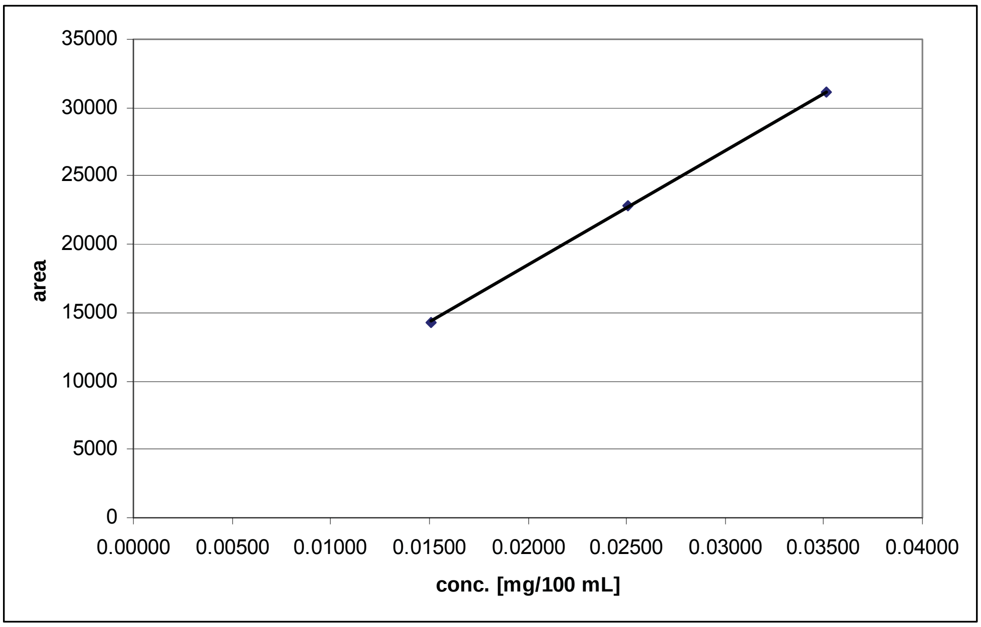

Figure 2 Chromatogram of a blank sample

Name: 04Y011_2011-04-19_004, Vial: 3, Date: 19-Apr-2011, Time: 14:17:06, Description: Traegerstoff

BAS 590 F

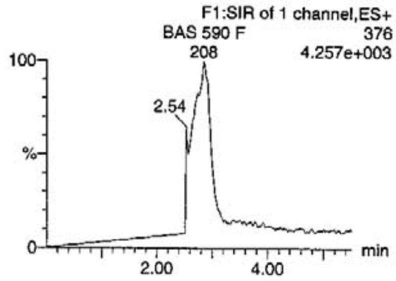

Figure 3 Chromatogram of the standard solution with the lowest substance concentration (0.1488 mg/L)

Name: 04Y011_2011-04-20_007, Vial: 4, Date: 20-Apr-2011, Time: 13:38:33, Description: Kalib 1

BAS 590 F

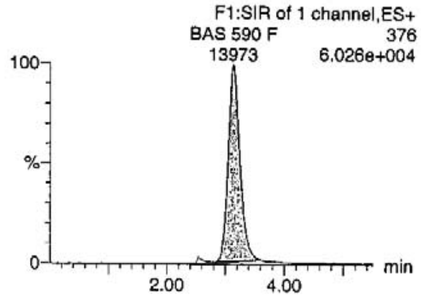

Figure 4 Chromatogram of the sample containing a nominal value of nominally 0.249 mg/100 mL test substance, analyzed immediately after sample preparation

Name: 04Y011_2011-04-19_001, Vial: 1, Date: 19-Apr-2011, Time: 13:24:58, Description: Stab 0h

BAS 590 F

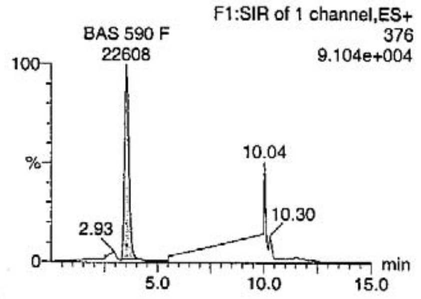

Figure 5 Chromatogram of the sample containing a nominal value of nominally 0.249 mg/100 mL test substance, analyzed approximately 7 days after sample preparation

Name: 04Y011_2011-04-26_001, Vial: 1, Date: 26-Apr-2011, Time: 10:58:03, Description: Stab 7d

BAS 590 F

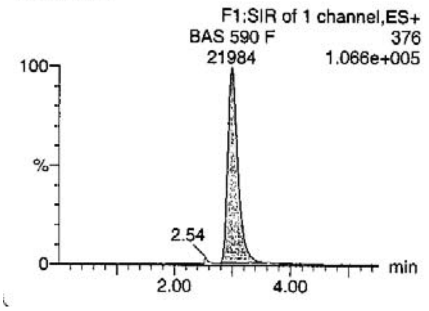

REPORT TITLE

Analysis of Vinclozolin, Prochloraz and Flutamide in Vehicle (Corn Oil)

STUDY IDENTIFICATION

PTRL Europe ID P 2854 G
BASF ID 02Y0375/88X002

AUTHOR

Reiner Bacher

STUDY COMPLETION ON

04-Jul-13

TEST FACILITY

PTRL Europe
Helmholtzstr. 22, Science Park
D-89081 Ulm, Germany

SPONSOR

BASF SE, Experimental Toxicology and Ecology
67056 Ludwigshafen, Germany

Original 1 (of 1)

Total number of pages: 25

GLP-CERTIFICATE OF TEST FACILITY

Baden-Württemberg

LANDESANSTALT FÜR UMWELT, MESSUNGEN UND NATURSCHUTZ BADEN-WÜRTTEMBERG

Gute Laborpraxis /Good Laboratory Practice

GLP-Bescheinigung / Statement of GLP Compliance

(gemäß / according to § 19 b Chemikaliengesetz)

Eine GLP-Inspektion zur Überwachung der Einhaltung der GLP-Grundsätze gemäß Chemikaliengesetz bzw. Richtlinie 2004/9/EG wurde durchgeführt in:

Assessment of conformity with GLP according to Chemikaliengesetz and Directive 2004/9/EC at:

☒ Prüfeinrichtung/Test facility ☐ Prüfstandort/Test site

PTRL Europe GmbH

Helmholtzstr. 22 Science Park I

89081 Ulm

(Unverwechselbare Bezeichnung und Adresse/Unequivocal name and adress)

Prüfungen nach Kategorien/Areas of Expertise

(gemäß/according ChemVwV-GLP Nr. 5.3/OECD guidance)

1	Prüfungen zur Bestimmung der physikalisch-chemischen Eigenschaften und Gehaltsbestimmungen	physical-chemical testing
5	Prüfungen zum Verhalten im Boden, im Wasser und in der Luft; Prüfungen zur Bioakkumulation und zur Metabolisierung	studies on behaviour in water, soil and air; bioaccumulation
6	Prüfungen zur Bestimmung von Rückständen	residue studies
8	Analytische Prüfungen an biologischen Materialien	analytical and clinical chemistry testing

Datum der Inspektion/Date of Inspection

(Tag, Monat, Jahr/day. month.year)

24.03.2011

Die/Der genannte Prüfeinrichtung/Prüfstandort befindet sich im nationalen GLP-Überwachungsverfahren und wird regelmäßig auf Einhaltung der GLP-Grundsätze überwacht.
Auf der Grundlage des Inspektionsberichtes wird hiermit bestätigt, dass in dieser Prüfeinrichtung/diesem Prüfstandort die oben genannten Prüfungen unter Einhaltung der GLP-Grundsätze durchgeführt werden können.

The above mentioned test facility/test site is included in the national GLP Compliance Programme and is inspected on a regular basis.
Based on the inspection report it can be confirmed, that this test facility/test site is able to conduct the aforementioned studies in compliance with the Principles of GLP.

Unterschrift, Datum/Signature, Date

Dr. Volker Giraud Volker Giraud **Karlsruhe, den** 20.6.2011

Leiter der Abteilung Technischer Arbeits- und Umweltschutz

(Name und Funktion der verantwortlichen Person/Name and function of responsible person)

LUBW Landesanstalt für Umwelt, Messungen und Naturschutz Baden-Württemberg,
Postfach 10 01 63, 76231 Karlsruhe

(Name und Adresse der GLP-Überwachungsbehörde/Name and address of GLP Monitoring Authority)

GOOD LABORATORY PRACTICE COMPLIANCE STATEMENT

The study forming the subject of this report was performed under the supervision of the Study Director in accordance with the procedures described therein. The report provides an accurate record of the procedures and the results obtained.

The study was conducted in compliance with the current German Principles of Good Laboratory Practice (GLP):

Anhang 1 zu § 19a des Chemikaliengesetzes (ChemG) i.d.F. vom 09.11.2011.

The German requirements are based on the OECD Principles of GOOD LABORATORY PRACTICE as revised in 1997 (ENV/MC/CHEM (98) 17, Paris, France, 1998) which are accepted by Regulatory Authorities throughout the European Community, the United States of America (FDA and EPA) and Japan (MHW, MAFF and METI).

04-Jul-13

Thomas Class, Study Director Date

COMPLIANCE TO GUIDELINES AND CERTIFICATION OF AUTHENTICITY

I, the undersigned, hereby declare that this study was performed under my supervision according to the procedures described herein, and that this report presents a true and accurate record of the results obtained.

Date: 04-Jul-13

Thomas Class, PTRL Europe
Helmholtzstr. 22, Science Park, D-89081 Ulm, Germany
Tel. (49)-(0)731-400 693-14, Thomas.Class@ptrl-europe.de

STUDY PERSONNEL

Position	Name
Study Director:	Thomas Class
Chemists:	Reiner Bacher, Tanja Kuhn
Technician(s):	Thomas Stolz, Petra Ilg, Martin Schweizer

QUALITY ASSURANCE STATEMENT

The Quality Assurance Unit has reviewed this report in accordance with Good Laboratory Practice Standards. Based upon the documentation provided, the reported data reviewed are determined to be an accurate reflection of the raw data in this study.

The following inspections were performed by the Quality Assurance Unit during the conduct of this study:

Part of Study	Inspection Date	Date of Submission of Inspection Report to	
		Study Director<br>T. Class<br>PTRL Europe	Management<br>L. Ruzo<br>PTRL Europe
Study Plan Review	04-Mar-2013	04-Mar-2013	04-Mar-2013
Critical Phase Audit *	08-Mar-2013	08-Mar-2013	19-Mar-2013
Raw Data Review and Draft Report	20/24-Jun-2013	24-Jun-2013	24-Jun-2013
Revised Draft and Final Report	04-Jul-2013	04-Jul-2013	04-Jul-2013

* Preparation of stock solutions.

Prepared by:

S. Haus[illegible] Date: 04-Jul-2013

Sandra Hausmann, PTRL Europe Quality Assurance

TABLE OF CONTENTS

Report Title ... 1
GLP-Certificate of Test Facility ... 2
Good Laboratory Practice Compliance Statement ... 3
Compliance to Guidelines and Certification of Authenticity ... 3
Study Personnel ... 3
Quality Assurance Statement ... 4
Table of Contents ... 5
Study Identification, Chronology of the Study and Archiving ... 6
1. Introduction ... 7
2. Experimental ... 7
- 2.1 Test System ... 7
- 2.2 Test/Reference Item(s) ... 7
- 2.3 Materials and Methods ... 8
- 2.4 Preparation of Stock Solutions ... 8
- 2.5 Preparation of Calibration Solutions ... 8
- 2.6 Dilution of Corn Oil Preparations for Analyte Determination ... 8
- 2.7 GC-MS/MS Determination of Vinclozolin ... 10
- 2.8 LC-MS/MS Determination of Prochloraz and Flutamide ... 10
- 2.9 Calculations with Example ... 12

3. Results and Discussion ... 12

Table 1: Vinclozolin in Corn Oil at Start of Administration ... 14
Table 2: Prochloraz in Corn Oil at Start of Administration ... 15
Table 3: Flutamide in Corn Oil at Start of Administration ... 16
Table 4: Vinclozolin in Corn Oil at End of Administration ... 17
Table 5: Prochloraz in Corn Oil at End of Administration ... 18
Table 6: Flutamide in Corn Oil at End of Administration ... 19

Figure 1 Representative GC/MS Calibration Curve and Function for Vinclozolin ... 20
Figure 2 Representative GC/MS Chromatograms: Calibration (10 ng/mL) and Vinclozolin in Corn Oil (1.25 µg/mL) (Sample 5) ... 21
Figure 3 Representative LC-MS/MS Calibration Curve and Function for Prochloraz ... 22
Figure 4 Representative LC-MS/MS Chromatograms: Calibration (5.0 ng/mL) and Prochloraz in Corn Oil (2.5 µg/mL) (Sample 5) ... 23
Figure 5 Representative LC-MS/MS Calibration Curve and Function for Flutamide ... 24
Figure 6 Representative LC/MS Chromatograms: Calibration (0.10 ng/mL) and Flutamide in Corn Oil (0.0625 µg/mL) (Sample 5) ... 25

STUDY IDENTIFICATION, CHRONOLOGY OF THE STUDY AND ARCHIVING

STUDY AND REPORT TITLE:

Analysis of Vinclozolin, Prochloraz and Flutamide in Vehicle (Corn Oil)

PTRL Europe ID:	P 2854 G
BASF ID:	02Y0375/88X002
Test Facility:	PTRL Europe GmbH<br>Helmholtzstr. 22, Science Park,<br>D-89081 Ulm, Germany
Study Director:	Thomas Class, PTRL Europe<br>Tel 0731-400 693-14, Thomas.Class@ptrl-europe.de
Sponsor:	BASF SE, Experimental Toxicology and Ecology<br>D-67056 Ludwigshafen, Germany
Study Monitor:	Matthias Becker, BASF,<br>GV/TE – Z470, D-67056 Ludwigshafen<br>Tel. 0621 60-56756, Matthias.d.becker@basf.com

CHRONOLOGY OF THE STUDY:

Approval of study plan:	04-Mar-13
Start of practical work:	08-Mar-13
End of practical work:	17-Jun-13
Study completion date:	04-Jul-13

ARCHIVING:

Upon issue of the final report all raw data, the study plan and the original of the final report are maintained in the archives of the Sponsor (Frankenthal) according to GLP regulations.

Samples of the test/reference items are archived at PTRL Europe for the period required by GLP regulations. No specimens are archived.

PTRL Europe archives the quality assurance reports.

1. Introduction

The objective of this study was to examine the concentration and homogeneity of vinclozolin, prochloraz and flutamide in corn oil used as vehicle in the toxicity study 60R0375/88R002.

2. Experimental

2.1 Test System

Preparation(s) of the test items in corn oil (vehicle) prepared in the related toxicity study 60R0375/88R002 at the following communicated nominal concentrations:

Vinclozolin:	0.125 mg/100 mL = 1.25 µg/mL
Flutamide:	0.00625 mg/100 mL = 62.5 ng/mL
Prochloraz:	0.25 mg/100 mL = 2.5 µg/mL
Vinclozolin:	100 mg/100 mL = 1.0 mg/mL
Flutamide:	0.625 mg/100 mL = 6.25 µg/mL
Prochloraz:	125 mg/100 mL = 1.25 mg/mL
Vinclozolin:	500 mg/100 mL = 5.0 mg/mL
Flutamide:	6.25 mg/100 mL = 62.5 µg/mL
Prochloraz:	750 mg/100 mL = 7.5 mg/mL
Flutamide:	0.00625 mg/100 mL = 62.5 ng/mL.

Two samplings from the test items were performed at the beginning and at the end of the administration (02-Mar-2013 and 09-Jun-2013).

2.2 Test/Reference Item(s)

Vinclozolin (BAS 352 F, PSN 88/0375-1)

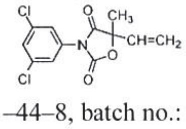

CAS RN: 50471–44–8, batch no.: N183, expiration date: 01-Aug-16

Purity: 99.1 % w/w. Appearance: Solid (powder), white. Storage: Ambient

Prochloraz (BAS 590 F, PSN 04/0611-4)

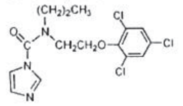

CAS RN: 67747–09–5, batch no.: COD-000718, expiration date: 14-Apr-16

Purity: 98.0 % w/w. Appearance: Solid, melt, brownish. Storage: Ambient

Flutamide (PSN 03/0525-8)

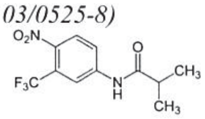

CAS RN: 13311-84-7, lot no.: 021M1406V

Expiration date: 13-Apr-14

Purity: 100 % w/w. Appearance: Powder, solid, light yellow. Storage: Ambient

2.3 Materials and Methods

Solvent and Chemicals:

Acetone, ethyl acetate, Pesticide grade, Promochem.

Acetonitrile, Promochem HPLC grade. Millipore water, PTRL Europe supply.

Formic acid, 98-100 %, Sigma-Aldrich.

Equipment:

Analytical balance Mettler-Toledo XR205DR, or equivalent.

Assorted lab ware, volumetric pipettes, syringes, flasks, vials, as needed.

2.4 Preparation of Stock Solutions

Stock solutions were prepared by weighing accurately 10 mg of the analytical standards into 10-mL volumetric flasks to obtain 1.0 mg/mL concentrations (purities considered) of the chemicals in acetone.

2.5 Preparation of Calibration Solutions

For GC-MS/MS determination of vinclozolin the 1-mg/mL stock solution was diluted volumetrically into ethyl acetate to obtain a 1.0 µg/mL intermediate solution. This intermediate solution was subsequently diluted volumetrically to obtain calibration solutions with concentrations in the range from 1.0 to 100 ng/mL in ethyl acetate.

For LC-MS/MS determination of prochloraz and flutamide the 1-mg/mL stock solutions were diluted volumetrically into acetone to obtain a intermediate solution at 1.0 µg/mL for flutamide and 10 µg/mL for prochloraz. This intermediate solution was subsequently diluted volumetrically into acetone (respectively acetone + 0.2 % corn oil) to obtain calibration solutions with concentrations in the range from 0.050 to 1.0 ng/mL for flutamide and 0.50 ng/mL to 10 ng/mL for prochloraz.

2.6 Dilution of Corn Oil Preparations for Analyte Determination

The density of the corn oil was determined gravimetrically to be 0.899 g/mL (in a related study PTRL Europe ID P 2800 G).

Diluted extracts of the different corn oil preparations were prepared in ethyl acetate for GC-MS/MS analysis of vinclozolin and in acetone for LC-MS/MS analysis of prochloraz and

flutamide. The final concentrations of the different extracts are designated to fit in the linear range of the different calibration curves:

- 1 - 100 ng/mL for vinclozolin
- 0.50 ng/mL to 10 ng/mL for prochloraz
- 0.05 ng/mL to 1.0 ng/mL for flutamide.

Dosing was performed using a positive-displacement pipette and monitoring of the exact weight of the individual sample aliquot. A summary of the dilution procedures for the different samples analyzed is given in the following table:

Sample ID BASF	Analyte		
	Vinclozolin	Prochloraz	Flutamide
5-7 + 19	Nominal concentration: 1.25 µg/mL<br>Sample aliquot: ≈ 90 mg (100 µL)<br>Dilution 1: 10 mL<br>Approx. final concentration:<br>12.5 ng/mL. Approx. total DF: 100<br>Theoretical V_{End}: 10 mL	Nominal concentration: 2.5 µg/mL<br>Sample aliquot: ≈ 45 mg (50 µL)<br>Dilution 1: 20 mL<br>Approx. final concentration:<br>6.25 ng/mL. Approx. total DF: 400<br>Theoretical V_{End}: 20 mL	Nominal concentration: 0.0625 µg/mL<br>Sample aliquot: ≈ 45 mg (50 µL)<br>Dilution 1: 20 mL<br>Approx. final concentration:<br>0.156 ng/m.L Approx. total DF: 400<br>Theoretical V_{End}: 20 mL
8 + 20	Nominal concentration: 1000 µg/mL<br>Sample aliquot: ≈ 90 mg (100 µL)<br>Dilution 1: 50 mL<br>Dilution 2: 0.100 mL into 10 mL<br>Approx. final concentration:<br>20 ng/mL. Approx. total DF: 50000<br>Theoretical V_{End}: 5000 mL	Nominal concentration: 1250 µg/mL<br>Sample aliquot: ≈ 45 mg (50 µL)<br>Dilution 1: 20 mL<br>Dilution 2: 0.050 mL into 50 mL<br>Approx. final concentration:<br>3.125 ng/mL. Approx. total DF: 400000<br>Theoretical V_{End}: 20000 mL	Nominal concentration: 6.25 µg/mL<br>Sample aliquot: ≈ 45 mg (50 µL)<br>Dilution 1: 20 mL<br>Dilution 2: 0.100 mL into 10 mL<br>Approx. final concentration:<br>0.156 ng/mL. Approx. total DF: 40000<br>Theoretical V_{End}: 2000 mL
9-11 + 21	Nominal concentration: 5000 µg/mL<br>Sample aliquot: ≈ 90 mg (100 µL)<br>Dilution 1: 50 mL<br>Dilution 2: 0.050 mL into 25 mL<br>Approx. final concentration:<br>20 ng/mL. Approx. total DF: 250000<br>Theoretical V_{End}: 25000 mL	Nominal concentration: 7500 µg/mL<br>Sample aliquot: ≈ 45 mg (50 µL)<br>Dilution 1: 20 mL<br>Dilution 2: 0.020 mL into 100 mL<br>Approx. final concentration:<br>3.75 ng/mL. Approx. total DF 2000000<br>Theoretical V_{End}: 100000 mL	Nominal concentration: 62.5 µg/mL<br>Sample aliquot: ≈ 45 mg (50 µL)<br>Dilution 1: 20 mL<br>Dilution 2: 0.050 mL into 50 mL<br>Approx. final concentration:<br>0.156 ng/mL. Approx. total DF: 400000<br>Theoretical V_{End}: 20000 mL
12-14 + 22	Not applicable	Not applicable	Nominal concentration: 0.0625 µg/mL<br>Sample aliquot: ≈ 45 mg (50 µL)<br>Dilution 1: 20 mL<br>Approx. final concentration:<br>0.156 ng/mL. Approx. total DF: 400<br>Theoretical V_{End}: 20 mL

DF: Dilution factor

2.7 GC-MS/MS Determination of Vinclozolin

GC-MS/MS determination of vinclozolin used a Thermo TSQ Quantum GC/MS system with:
TriPlus autosampler, Trace Ultra GC with split/splitless injector (220 °C, 1 µL injection,
2 min splitless), Agilent VF-5ms capillary (30 m length, 0.25 mm ID, 0.25 µm film),
Helium carrier gas flow 1.7 mL/min,
Oven program: 70 °C, 2 min, 30 °C/min to 310 °C, 3 min hold.

The triple-quadrupole TSQ Quantum mass spectrometer was operated in the positive EI SRM mode isolating the intensive 212 m/z fragment ion of vinclozolin for collision induced fragmentation (collision energy 15 eV) to monitor the intensive 172 m/z daughter ion for quantitation.

Additional SRM ion pairs (285 m/z -> 212 m/z, 285 m/z -> 178 m/z) were monitored for confirmation, but not evaluated quantitatively.

2.8 LC-MS/MS Determination of Prochloraz and Flutamide

Prochloraz (positive detection mode, ESI+) and flutamide (negative detection mode, ESI-) were determined simultaneously in one LC-MS/MS applying the two experiment feature of the AB Sciex Analyst software.

Details of the parameters of the LC-MS/MS method are presented in the following table:

LC System	Agilent 1200 HPLC system (vacuum solvent degasser, binary HPLC pump, column oven), and CTC Analytics HTC-Pal Autosampler.
LC Column	YMC Triart HPLC column: Length: 150 mm, i.d.: 3.0 mm,<br>particle size: 3 µm, column temperature 60 °C.
LC Injection Volume	10 µL.
LC Method	Solvent A: 0.1 % formic acid in water.<br>Solvent B: 0.1 % formic acid in acetonitrile.<br><br>Mobile Phase Composition:<br>Time (min) Flow rate (mL/min) % A % B<br>0.0 0.40 60 40<br>1.0 0.40 60 40<br>6.0 0.40 5 95<br>10.0 0.40 5 95<br>10.1 0.40 60 40<br>15.0 0.40 60 40
Retention times	Prochloraz: approx. 8.3 min<br>Flutamide: approx. 8.7 min
MS/MS System	AB Sciex API 5500 triple quadrupole LC-MS/MS system with Turbo IonSpray (ESI) source, detection of positive ions (prochloraz) or negative ions (flutamide).
Ion Source Conditions ESI Positive Polarity (Detection of Prochloraz)	Source temperature: 500 °C<br>Gas supply GS 1: 40 (arbitrary units)<br>Gas supply GS 2: 60 (arbitrary units)<br>Curtain gas: 30 (arbitrary units)<br>IonSpray voltage: 4500 V<br>Entrance potential: 10 V<br>*Prochloraz:*<br>Declustering potential (DP): 76 V
MS/MS Conditions for Prochloraz	*Prochloraz (376 m/z → 308 m/z) for quantification*<br>CE: 17 V<br>CXP: 10 V<br>*Prochloraz (378 m/z → 310 m/z) for confirmation*<br>CAD: Medium<br>Dwell time per transition: 30 ms<br>Resolution Q1: unit<br>Resolution Q3: unit
Ion Source Conditions ESI Negative Polarity (Detection of Flutamide)	Source temperature: 500 °C<br>Gas supply GS 1: 40 (arbitrary units)<br>Gas supply GS 2: 60 (arbitrary units)<br>Curtain gas: 30 (arbitrary units)<br>IonSpray voltage: -4500 V<br>Entrance potential: -10 V<br>*Flutamide:*<br>Declustering potential (DP): -91 V
MS/MS Conditions for Flutamide	*Flutamide (275 m/z → 202 m/z) for quantification*<br>CE: -32 V<br>CXP: -9.0 V<br>*Flutamide: (275 m/z →205 m/z) for confirmation*<br>CAD: Medium<br>Dwell time per transition: 200 ms<br>Resolution Q1: unit<br>Resolution Q3: unit

2.9 Calculations with Example

Detailed results are given in Table 1 to Table 6.

Calculations are exemplified for GC-MS/MS determination of vinclozolin.

GC-MS/MS injections of diluted corn oil preparations were evaluated for vinclozolin based on external standard calibration.

The actual concentration C_{Found} (in µg/mL) of the chemical in the corn oil preparations was calculated as follows:

C_{Found} (in µg/mL) = $c_{Found} \times D \times V_{End} \times 1000 / (W \times 1000)$

c_{Found}:	Concentration of vinclozolin detected for diluted extract (ng/mL)
D:	Density of corn oil: 0.899 g/mL
V_{End}:	Theoretical final volume of extract (mL)
W:	Weight of sample (mg)
1000:	Conversion factor to adjust dimensions (mg/g)
1000:	Conversion factor to adjust dimensions (ng/µg)

Example:

Specimen extract P2854-40/1 (see Table 1) was prepared by weighing 95.41 mg of the corn oil formulation (sample 5) into a 10 mL volumetric flask and adjusting the volume to 10 mL with ethyl acetate.

GC/MS determination (GC/MS file P2854-009) of the vinclozolin, resulting in a detected concentration c_{Found} of 13.01 ng/mL. The actual concentration C_{Found} of vinclozolin in the preparation was calculated as follows:

C_{Found} (in µg/mL) = 13.01 ng/mL x 0.899 g/mL x 10 mL x 1000 mg/g / (95.41 mg x 1000 ng/µg)

C_{Found} (in µg/mL) = 1.23 µg/mL.

3. Results and Discussion

The objective of this study was to examine the concentration and homogeneity of vinclozolin, prochloraz and flutamide in corn oil used as vehicle in the toxicity study 60R0375/88R002.

Preparation(s) of the test items in corn oil (vehicle) were received at the following nominal concentrations:

Vinclozolin:	0.125 mg/100 mL = 1.25 µg/mL
Flutamide:	0.00625 mg/100 mL = 62.5 ng/mL
Prochloraz:	0.25 mg/100 mL = 2.5 µg/mL

Vinclozolin: 100 mg/100 mL = 1.0 mg/mL
Flutamide: 0.625 mg/100 mL = 6.25 µg/mL
Prochloraz: 125 mg/100 mL = 1.25 mg/mL

Vinclozolin: 500 mg/100 mL = 5.0 mg/mL
Flutamide: 6.25 mg/100 mL = 62.5 µg/mL
Prochloraz: 750 mg/100 mL = 7.5 mg/mL

Flutamide: 0.00625 mg/100 mL = 62.5 ng/mL.

A GC-MS/MS method was applied for the determination of vinclozolin in diluted corn oil preparations. A LC-MS/MS method was used for the determination of prochloraz and flutamide in diluted corn oil preparations.

Individual results for the three analytes at the start and at the end of the administration are listed in Table 1 to Table 6.

At the start of the administration, all concentrations detected for the three analytes in the corn oil preparations were within a range of 85 % to 112 % of the nominal concentrations. The concentrations of the three analytes in the corn oil preparations at the end of the administration ranged between 90 % and 110 % of the nominal concentrations.

These results indicate a correct application of the analytes into the corn oil preparations and a sufficient stability and homogeneity of the three analytes in this matrix.

TABLE 1: VINCLOZOLIN IN CORN OIL AT START OF ADMINISTRATION

Vinclozolin in Corn Oil (GC-MS/MS) - First Sampling

Sample ID	GC-MS/MS Run File	c_{Found}	Weight of Sample for Analysis	Vinclozolin Found (212 -> 172 m/z)			
				C_{Found}		of nominal	
P2854-	P2854-	ng/mL	mg	µg/mL			
Sample 5 (Dilution Factor approx. 100)							
		$C_{Nominal}$	1.25	µg/mL			
40/1	009	13.01	95.41	1.23	Mean	98%	Mean
40/2	011	14.12	90.34	1.41	1.37	112%	109%
40/3	012	14.98	91.71	1.47		117%	
Sample 6 (Dilution Factor approx. 100)							
		$C_{Nominal}$	1.25	µg/mL			
41/1	013	13.54	93.24	1.31	Mean	104%	Mean
41/2	014	14.01	90.83	1.39	1.36	111%	109%
41/3	015	14.71	95.21	1.39		111%	
Sample 7 (Dilution Factor approx. 100)							
		$C_{Nominal}$	1.25	µg/mL			
42/1	018	13.36	90.33	1.33	Mean	106%	Mean
42/2	019	14.46	94.60	1.37	1.36	110%	109%
42/3	020	14.48	94.16	1.38		111%	
Sample 8 (Dilution Factor approx. 50000)							
		$C_{Nominal}$	1000	µg/mL			
43/1A	021	20.17	95.88	946	Mean	95%	Mean
43/2A	022	21.46	88.12	1095	1019	109%	102%
43/3A	023	21.01	92.88	1017		102%	
Sample 9 (Dilution Factor approx. 250000)							
		$C_{Nominal}$	5000	µg/mL			
44/1A	026	22.56	90.32	5615	Mean	112%	Mean
44/2A	027	20.95	90.89	5181	5350	104%	107%
44/3A	028	22.31	95.41	5256		105%	
Sample 10 (Dilution Factor approx. 250000)							
		$C_{Nominal}$	5000	µg/mL			
45/1A	029	18.99	88.75	4810	Mean	96%	Mean
45/2A	030	20.57	91.55	5050	4899	101%	98%
45/3A	031	19.56	90.91	4836		97%	
Sample 11 (Dilution Factor approx. 250000)							
		$C_{Nominal}$	5000	µg/mL			
46/1A	034	20.05	88.25	5107	Mean	102%	Mean
46/2A	035	19.85	91.10	4897	4922	98%	98%
46/3A	036	18.89	89.13	4763		95%	

TABLE 2: PROCHLORAZ IN CORN OIL AT START OF ADMINISTRATION

Prochloraz in Corn Oil (LC-MS/MS) - First Sampling

Sample ID	LC-MS/MS Run File	c_{Found}	Weight of Sample for Analysis	Prochloraz Found (376 -> 308 m/z)			
				C_{Found}		of nominal	
P2854-	P2854-	ng/mL	mg	µg/mL			
Sample 5 (Dilution Factor approx. 400)							
		$C_{Nominal}$	2.5	µg/mL			
82/1	124	6.74	47.82	2.53	Mean	101%	Mean
82/2	125	7.03	47.07	2.69	2.74	107%	110%
82/3	126	7.86	46.99	3.01		120%	
Sample 6 (Dilution Factor approx. 400)							
		$C_{Nominal}$	2.5	µg/mL			
83/1	128	8.15	47.83	3.06	Mean	123%	Mean
83/2	129	7.68	47.30	2.92	2.80	117%	112%
83/3	130	6.22	46.10	2.43		97%	
Sample 7 (Dilution Factor approx. 400)							
		$C_{Nominal}$	2.5	µg/mL			
84/1	132	6.42	47.98	2.41	Mean	96%	Mean
84/2	133	6.62	46.75	2.55	2.46	102%	98%
84/3	134	6.37	47.06	2.43		97%	
Sample 8 (Dilution Factor approx. 400000)							
		$C_{Nominal}$	1250	µg/mL			
50/1B	108	2.89	45.11	1152	Mean	92%	Mean
50/2B	109	2.73	45.27	1084	1124	87%	90%
50/3B	110	2.84	44.92	1137		91%	
Sample 9 (Dilution Factor approx. 2000000)							
		$C_{Nominal}$	7500	µg/mL			
51/1B	112	3.30	44.27	6701	Mean	89%	Mean
51/2B	113	3.37	44.88	6751	6700	90%	89%
51/3B	114	3.32	44.89	6649		89%	
Sample 10 (Dilution Factor approx. 2000000)							
		$C_{Nominal}$	7500	µg/mL			
52/1B	116	3.16	45.44	6252	Mean	83%	Mean
52/2B	117	3.39	46.90	6498	6496	87%	87%
52/3B	118	3.33	44.43	6738		90%	
Sample 11 (Dilution Factor approx. 2000000)							
		$C_{Nominal}$	7500	µg/mL			
53/1B	119	3.43	45.54	6771	Mean	90%	Mean
53/2B	120	3.38	45.47	6683	6745	89%	90%
53/3B	121	3.35	44.41	6781		90%	

TABLE 3: FLUTAMIDE IN CORN OIL AT START OF ADMINISTRATION

Flutamide in Corn Oil (LC-MS/MS) - First Sampling

Sample ID	LC-MS/MS Run File	c_{Found}	Weight of Sample for Analysis	Flutamide Found (275 -> 202 m/z)			
				C_{Found}		of nominal	
P2854-	P2854-	ng/mL	mg	µg/mL			
Sample 5 (Dilution Factor approx. 400)							
		$C_{Nominal}$	0.0625	µg/mL			
82/1	124	0.177	47.82	0.0666	Mean	106%	Mean
82/2	125	0.153	47.07	0.0584	0.0655	94%	105%
82/3	126	0.187	46.99	0.0716		114%	
Sample 6 (Dilution Factor approx. 400)							
		$C_{Nominal}$	0.0625	µg/mL			
83/1	128	0.167	47.83	0.0628	Mean	100%	Mean
83/2	129	0.162	47.30	0.0616	0.0612	99%	98%
83/3	130	0.152	46.10	0.0593		95%	
Sample 7 (Dilution Factor approx. 400)							
		$C_{Nominal}$	0.0625	µg/mL			
84/1	132	0.157	47.98	0.0588	Mean	94%	Mean
84/2	133	0.168	46.75	0.0646	0.0627	103%	100%
84/3	134	0.169	47.06	0.0646		103%	
Sample 8 (Dilution Factor approx. 40000)							
		$C_{Nominal}$	6.25	µg/mL			
50/1A	093	0.157	45.11	6.26	Mean	100%	Mean
50/2A	094	0.140	45.27	5.56	5.95	89%	95%
50/3A	095	0.151	44.92	6.04		97%	
Sample 9 (Dilution Factor approx. 400000)							
		$C_{Nominal}$	62.5	µg/mL			
51/1A	096	0.128	44.27	52.0	Mean	83%	Mean
51/2A	097	0.138	44.88	55.3	54.8	88%	88%
51/3A	098	0.143	44.89	57.3		92%	
Sample 10 (Dilution Factor approx. 400000)							
		$C_{Nominal}$	62.5	µg/mL			
52/1A	100	0.137	45.44	54.2	Mean	87%	Mean
52/2A	101	0.134	46.90	51.4	53.1	82%	85%
52/3A	102	0.133	44.43	53.8		86%	
Sample 11 (Dilution Factor approx. 400000)							
		$C_{Nominal}$	62.5	µg/mL			
53/1A	104	0.137	45.54	54.1	Mean	87%	Mean
53/2A	105	0.128	45.47	50.6	53.4	81%	85%
53/3A	106	0.137	44.41	55.5		89%	
Sample 12 (Dilution Factor approx. 400)							
		$C_{Nominal}$	0.0625	µg/mL			
85/1	137	0.159	48.07	0.0595	Mean	95%	Mean
85/2	138	0.177	48.81	0.0652	0.0611	104%	98%
85/3	139	0.156	47.82	0.0587		94%	
Sample 13 (Dilution Factor approx. 400)							
		$C_{Nominal}$	0.0625	µg/mL			
86/1	141	0.183	48.75	0.0675	Mean	108%	Mean
86/2	142	0.169	48.83	0.0622	0.0632	100%	101%
86/3	143	0.159	47.85	0.0597		96%	
Sample 14 (Dilution Factor approx. 400)							
		$C_{Nominal}$	0.0625	µg/mL			
87/1	145	0.157	46.89	0.0602	Mean	96%	Mean
87/2	146	0.163	49.47	0.0592	0.0582	95%	93%
87/3	147	0.152	49.65	0.0550		88%	

TABLE 4: VINCLOZOLIN IN CORN OIL AT END OF ADMINISTRATION

Vinclozolin in Corn Oil (GC-MS/MS) - Second Sampling

Sample ID	GC-MS/MS Run File	c_{Found}	Weight of Sample for Analysis	Vinclozolin Found (212 -> 172 m/z)			
				C_{Found}		of nominal	
P2854-	P2854-	ng/mL	mg	µg/mL			
Sample 19 (Dilution Factor approx. 100)							
		$C_{Nominal}$	1.25	µg/mL			
114/1	048	14.08	91.92	1.38	Mean	110%	Mean
114/2	049	14.76	93.36	1.42	1.38	114%	110%
114/3	050	14.06	94.61	1.34		107%	
Sample 20 (Dilution Factor approx. 50000)							
		$C_{Nominal}$	1000	µg/mL			
115/1	052	21.48	95.91	1007	Mean	101%	Mean
115/2	053	20.75	91.76	1016	1035	102%	103%
115/3	054	23.08	95.96	1081		108%	
Sample 21 (Dilution Factor approx. 250000)							
		$C_{Nominal}$	5000	µg/mL			
116/1	056	21.09	96.17	4929	Mean	99%	Mean
116/2	057	20.07	94.63	4766	4872	95%	97%
116/3	058	19.75	90.20	4922		98%	

TABLE 5: PROCHLORAZ IN CORN OIL AT END OF ADMINISTRATION

Prochloraz in Corn Oil (LC-MS/MS) - Second Sampling

Sample ID	LC-MS/MS Run File	c_{Found}	Weight of Sample for Analysis	Prochloraz Found (376 -> 308 m/z)			
				C_{Found}		of nominal	
P2854-	P2854-	ng/mL	mg	µg/mL			
Sample 19 (Dilution Factor approx. 400)							
		$C_{Nominal}$	2.5	µg/mL			
117/1	173	6.45	47.86	2.42	Mean	97%	Mean
117/2	174	6.89	47.74	2.59	2.50	104%	100%
117/3	175	6.79	49.20	2.48		99%	
Sample 20 (Dilution Factor approx. 400000)							
		$C_{Nominal}$	1250	µg/mL			
118/1	177	3.04	48.62	1124	Mean	90%	Mean
118/2	178	3.05	47.84	1146	1148	92%	92%
118/3	179	3.10	47.50	1173		94%	
Sample 21 (Dilution Factor approx. 2000000)							
		$C_{Nominal}$	7500	µg/mL			
119/1	181	3.56	45.17	7085	Mean	94%	Mean
119/2	182	3.55	45.59	7000	6954	93%	93%
119/3	183	3.71	49.21	6778		90%	

TABLE 6: FLUTAMIDE IN CORN OIL AT END OF ADMINISTRATION

Flutamide in Corn Oil (LC-MS/MS) - Second Sampling

Sample ID	LC-MS/MS Run File	c_{Found}	Weight of Sample for Analysis	Flutamide Found (275 -> 202 m/z)			
				C_{Found}		of nominal	
P2854-	P2854-	ng/mL	mg	µg/mL			
Sample 19 (Dilution Factor approx. 400)							
		$C_{Nominal}$	0.0625	µg/mL			
120/1	153	0.170	48.30	0.0633	Mean	101%	Mean
120/2	154	0.158	46.23	0.0615	0.0629	98%	101%
120/3	155	0.169	47.40	0.0641		103%	
Sample 20 (Dilution Factor approx. 40000)							
		$C_{Nominal}$	6.25	µg/mL			
121/1	157	0.163	48.01	6.10	Mean	98%	Mean
121/2	158	0.169	47.39	6.41	6.26	103%	100%
Sample 21 (Dilution Factor approx. 400000)							
		$C_{Nominal}$	62.5	µg/mL			
122/1	161	0.154	47.53	58.3	Mean	93%	Mean
122/2	162	0.144	47.44	54.6	55.9	87%	90%
122/3	163	0.142	46.43	55.0		88%	
Sample 22 (Dilution Factor approx. 400)							
		$C_{Nominal}$	0.0625	µg/mL			
123/1	165	0.178	49.00	0.0653	Mean	105%	Mean
123/2	166	0.160	47.29	0.0608	0.0627	97%	100%
123/3	167	0.161	46.70	0.0620		99%	

FIGURE 1 REPRESENTATIVE GC/MS CALIBRATION CURVE AND FUNCTION FOR VINCLOZOLIN

Top: Calibration diagram and function obtained for vinclozolin (212 m/z -> 172 m/z)
Linear Regression with 1 / x weighting gave the following equation:
Y = 8964.91 x X - 4770.75 (r = 0.9920)

Bottom: Table with peak areas of calibration injections.

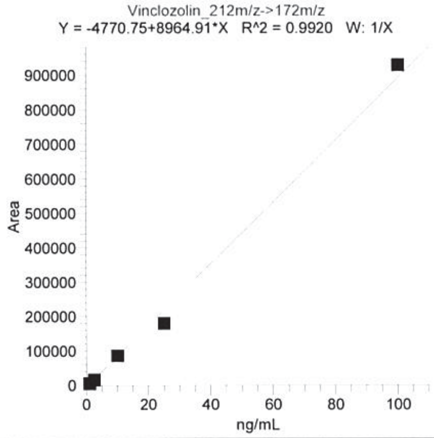

GC/MS/MS Run			Vinclozolin 212 m/z -> 172 m/z
P2854	K2854	ng/mL	Peak Area
38	16	100	928987
40	57	25	180422
25	17	10	86558
42	58	2.5	15921
8	18	1.0	5899

FIGURE 2 REPRESENTATIVE GC/MS CHROMATOGRAMS: CALIBRATION (10 NG/ML) AND VINCLOZOLIN IN CORN OIL (1.25 µG/ML) (SAMPLE 5)

Top: 10 ng/mL calibration solution.
Bottom: Preparation in corn oil, 100-fold diluted.

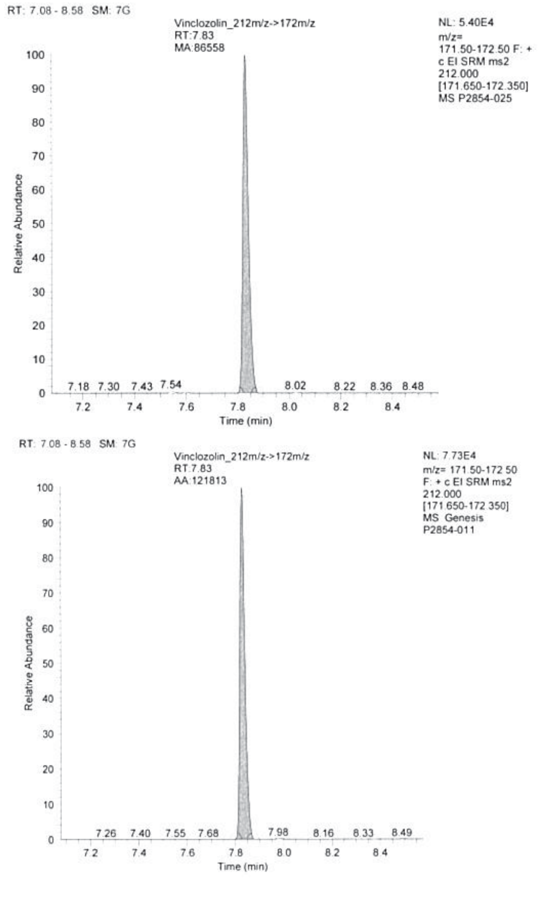

FIGURE 3 REPRESENTATIVE LC-MS/MS CALIBRATION CURVE AND FUNCTION FOR PROCHLORAZ

Top: Calibration diagram and function obtained for prochloraz (376 m/z -> 308 m/z)
Linear Regression with 1 / x weighting gave the following equation:
$Y = 4.63 \times 10^5 \times X + 5.56 \times 10^4$ (r = 0.9974)

Bottom: Table with peak areas of calibration injections.

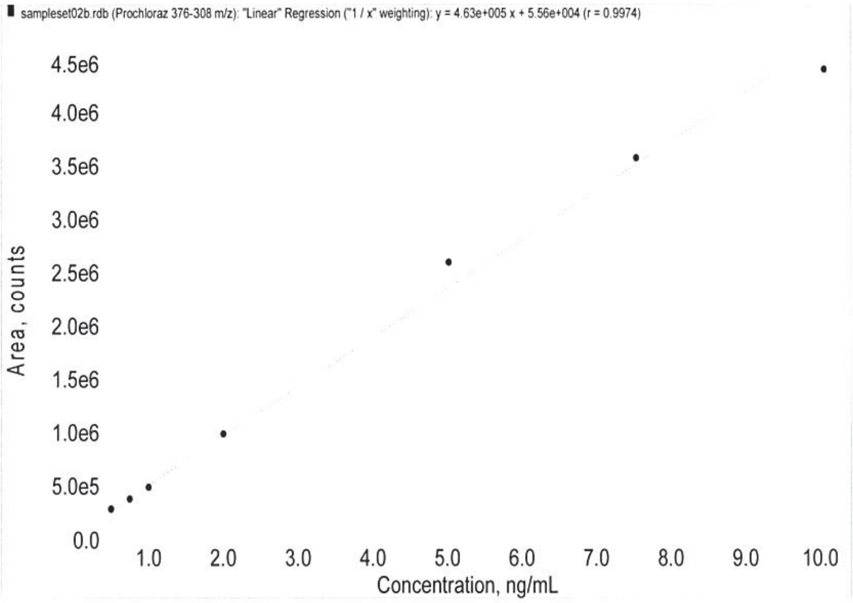

File Name	Solution ID	Conc. ng/mL	Prochloraz 376 m/z -> 308 m/z
P2854API5500#148.wiff	K2854-70	10.0	4.41E+06
P2854API5500#111.wiff	K2854-71	7.5	3.58E+06
P2854API5500#127.wiff	K2854-72	5.0	2.60E+06
P2854API5500#131.wiff	K2854-73	2.0	9.92E+05
P2854API5500#135.wiff	K2854-74	1.0	4.95E+05
P2854API5500#136.wiff	K2854-75	0.75	3.86E+05
P2854API5500#140.wiff	K2854-76	0.50	2.93E+05

FIGURE 4 REPRESENTATIVE LC-MS/MS CHROMATOGRAMS: CALIBRATION (5.0 NG/ML) AND PROCHLORAZ IN CORN OIL (2.5 µG/ML) (SAMPLE 5)

Top: 5.0 ng/mL calibration solution.
Bottom: Preparation in corn oil, approx. 400-fold diluted.

Sample Name: "K2854-72" Sample ID: "" File: "P2854API5500#127.wiff"
Peak Name: "Prochloraz 376-308 m/z" Mass(es): "376.000/308.000 Da"
Comment: "Cal 0.5/5.0 ng/mL" Annotation: ""

```
Sample Index:        1
Sample Type:      Standard
Concentration:       5.00     ng/mL
Calculated Conc:     5.51     ng/mL
Acq. Date:        19.03.2013
Acq. Time:        12:06:32

Modified:            No
Proc. Algorithm: Specify Parameters - MQ III
Noise Percentage:      50
Base. Sub. Window:     1.00   min
Peak-Split. Factor:    2
Report Largest Peak:    Yes
Min. Peak Height:       0.00   cps
Min. Peak Width:        0.00   sec
Smoothing Width:        7       points
RT Window:          30.0    sec
Expected RT:           7.84   min
Use Relative RT:       No

Int. Type:          Valley
Retention Time:        7.86   min
Area:            2.60e+006   counts
Height:            3.96e+005  cps
Start Time:            7.75   min
End Time:              8.15   min
```

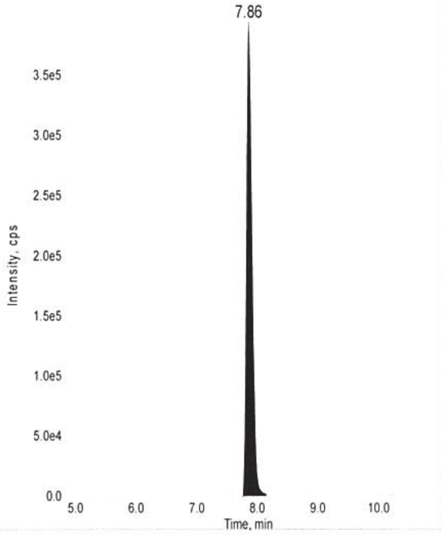

Sample Name: "P2854-82/2" Sample ID: "" File: "P2854API5500#125.wiff"
Peak Name: "Prochloraz 376-308 m/z" Mass(es): "376.000/308.000 Da"
Comment: "Sample 5 1:400" Annotation: ""

```
Sample Index:        1
Sample Type:      Unknown
Concentration:       N/A
Calculated Conc:     7.03     ng/mL
Acq. Date:        19.03.2013
Acq. Time:        11:34:26

Modified:            Yes
Proc. Algorithm: Specify Parameters - MQ III
Noise Percentage:      50
Base. Sub. Window:     1.00   min
Peak-Split. Factor:    2
Report Largest Peak:    Yes
Min. Peak Height:       0.00   cps
Min. Peak Width:        0.00   sec
Smoothing Width:        7       points
RT Window:          30.0    sec
Expected RT:           7.84   min
Use Relative RT:       No

Int. Type:          Manual
Retention Time:        7.86   min
Area:            3.31e+006   counts
Height:            5.40e+005  cps
Start Time:            7.76   min
End Time:              8.09   min
```

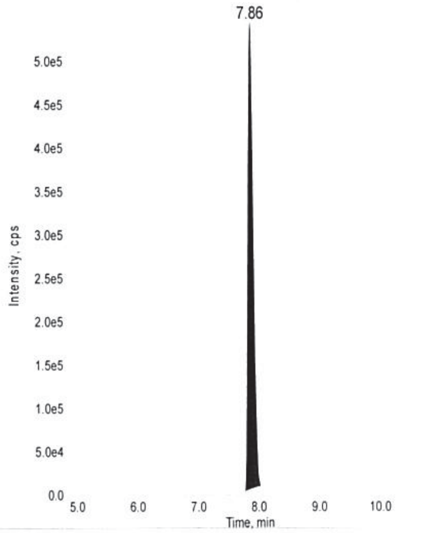

FIGURE 5 REPRESENTATIVE LC-MS/MS CALIBRATION CURVE AND FUNCTION FOR FLUTAMIDE

Top: Calibration diagram and function obtained for flutamide (275 m/z -> 202 m/z)
Linear Regression with 1 / x weighting gave the following equation:
$Y = 2.23 \times 10^6 \times X + 5.53 \times 10^4$ (r = 0.9954)

Bottom: Table with peak areas of calibration injections.

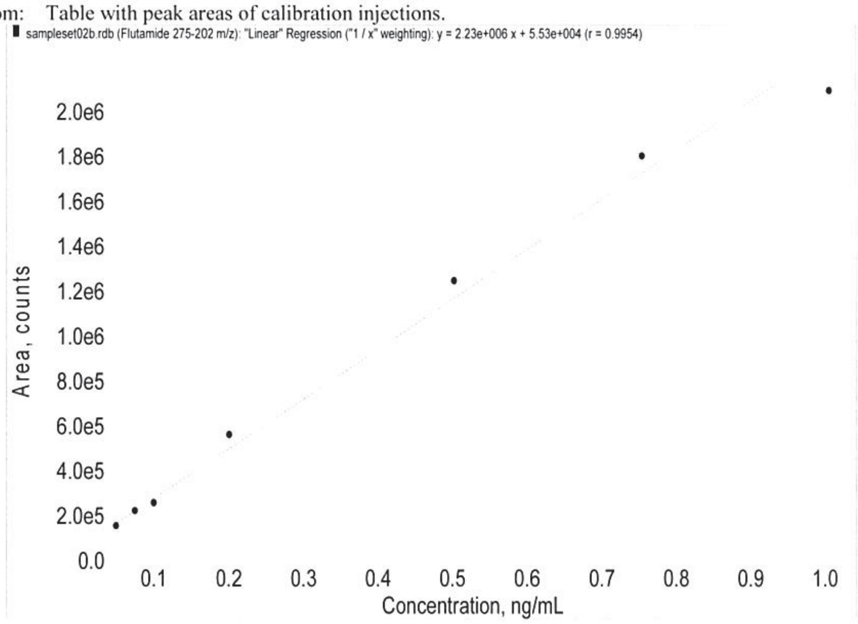

File Name	Solution ID	Conc. ng/mL	Flutamide 275 m/z -> 202 m/z
P2854API5500#148.wiff	K2854-70	1.0	2.10E+06
P2854API5500#111.wiff	K2854-71	0.75	1.81E+06
P2854API5500#127.wiff	K2854-72	0.50	1.25E+06
P2854API5500#131.wiff	K2854-73	0.20	5.65E+05
P2854API5500#135.wiff	K2854-74	0.10	2.59E+05
P2854API5500#136.wiff	K2854-75	0.075	2.24E+05
P2854API5500#140.wiff	K2854-76	0.050	1.57E+05

FIGURE 6 REPRESENTATIVE LC/MS CHROMATOGRAMS: CALIBRATION (0.10 NG/ML) AND FLUTAMIDE IN CORN OIL (0.0625 µG/ML) (SAMPLE 5)

Top: 0.10 ng/mL calibration solution.
Bottom: Preparation in corn oil, approx. 400-fold diluted.

Sample Name: "K2854-74" Sample ID: "" File: "P2854API5500#135.wiff"
Peak Name: "Flutamide 275-202 m/z" Mass(es): "275.000/202.000 Da"
Comment: "Cal 0.1/1.0 ng/mL" Annotation: ""

```
Sample Index:        1
Sample Type:      Standard
Concentration:     0.100    ng/mL
Calculated Conc:  0.0913    ng/mL
Acq. Date:        19.03.2013
Acq. Time:        14:15:02

Modified:            No
Proc. Algorithm: Specify Parameters - MQ III
Noise Percentage:     50
Base. Sub. Window:    1.00   min
Peak-Split. Factor:   2
Report Largest Peak:   Yes
Min. Peak Height:      0.00   cps
Min. Peak Width:       0.00   sec
Smoothing Width:       7        points
RT Window:          30.0    sec
Expected RT:         8.18   min
Use Relative RT:     No

Int. Type:          Valley
Retention Time:      8.35   min
Area:          2.59e+005   counts
Height:          4.36e+004  cps
Start Time:          8.24   min
End Time:            8.61   min
```

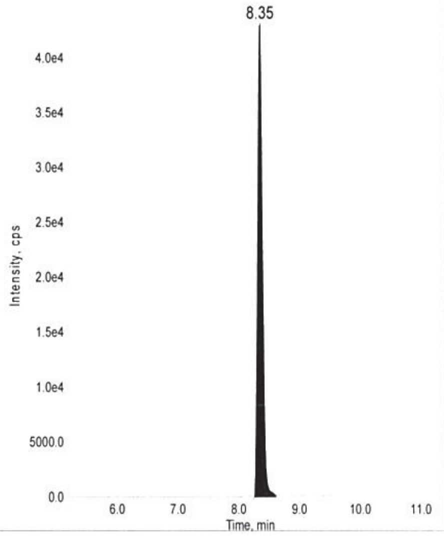

Sample Name: "P2854-82/2" Sample ID: "" File: "P2854API5500#125.wiff"
Peak Name: "Flutamide 275-202 m/z" Mass(es): "275.000/202.000 Da"
Comment: "Sample 5 1:400" Annotation: ""

```
Sample Index:        1
Sample Type:      Unknown
Concentration:       N/A
Calculated Conc:   0.153    ng/mL
Acq. Date:        19.03.2013
Acq. Time:        11:34:26

Modified:            No
Proc. Algorithm: Specify Parameters - MQ III
Noise Percentage:     50
Base. Sub. Window:    1.00   min
Peak-Split. Factor:   2
Report Largest Peak:   Yes
Min. Peak Height:      0.00   cps
Min. Peak Width:       0.00   sec
Smoothing Width:       7        points
RT Window:          30.0    sec
Expected RT:         8.18   min
Use Relative RT:     No

Int. Type:          Valley
Retention Time:      8.35   min
Area:          3.97e+005   counts
Height:          6.55e+004  cps
Start Time:          8.26   min
End Time:            8.71   min
```

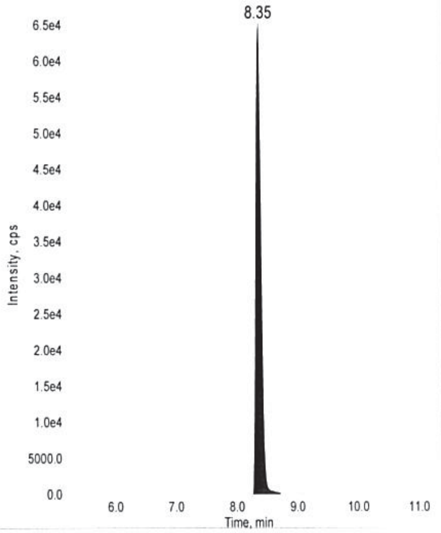

Proj.No.: 60R0375/88R002

Number of areolas/nipples ♂ pups PND 12

Test-group:	♂ Pup Nr.: / Dam No.:	1	2	3	4	5	6	7	8	9	10
1	30	2	0	3	2	2	1	0			
4	103	5	4	2	2						

Proj.No.: 60R0375/88R002

Number of areolas/nipples ♂ pups PND 12

Test-group:	♂ Pup Nr.: / Dam No.:	1	2	3	4	5	6	7	8	9	10
0	1	3	3	1	0	0	2				
0	2	0	2	1	4	2					
0	3	0	0	3	2	2					
0	4	0	0	0	1						
0	5	3	1	2	0	2	3	2			
0	6	0	1	0	3						
0	7	0	4	0	0	3					
1	26	0	1	1	0	0					
1	27	2	2	1	3						
1	28	2	4	3	1						
1	29	2	0	3	0	2	3	0	2	0	
1	31	2	0								
1	32	0	1								
1	37	0	0	1	1	3					
2	51	3	3	2							
2	52	3	4	0	3	0					
2	53	2	2	0	2	2					
2	54	2	2	2	0	0	3				
2	55	0	4	4	1	2	3	0			
2	57	2	1	1	0	2	0				
3	77	5	6	7	2						
4	102	2	0	0	0	2	0	2			
4	104	0	0	0	2	2	0	1	0		
4	105	3	0	4	2	1					
4	106	0	0	0	0	2	0				
4	107	2	4	1	2						

Proj.No.: 60R0375/88R002

Number of areolas/nipples ♂ pups PND 12

Test-group:	♂ Pup Nr.: / Dam No.:	1	2	3	4	5	6	7	8	9	10
0	8	2	2	2	0						
0	9	2	3	2	2						
0	10	2	3	0	2	1					
0	11	0	0	1	2	0					
0	22	2	4	2	0						
1	33	0	3	1	1						
1	34	0	1	1	0	1					
1	35	0	0	1							
1	36	0	4	0	4						
1	45	1	1	0	2	4					
1	46	1	2	1	2	0	2	0			
2	56	0									
2	59	5	1	2	3						
2	60	0	2	0	0						
2	61	0	0	3	0	2					
3	78	8	2	8	2	7	7	8			
3	80	6	2	4							
3	82	7	3	6	8	6	8				
4	101	0	0	0	0	0	0				
4	108	0	3	4							
4	109	0	0	2	1						
4	110										
4	111	0	0	1	2	1	2				
4	121	0									

Proj.No.: 60R0375/88R002

Number of areolas/nipples ♂ pups PND 12

Test-group:	♂ Pup Nr.: / Dam No.:	1	2	3	4	5	6	7	8	9	10
0	17	1	1								
0	18	1	2	1	0	1					
0	19	2	0								
0	20	3	4								
0	21	3	3	4	2	2	4	0			
1	43	0	0	0	0	2	0				
1	47	4	2	1	4						
2	58	0	0	0	0	1					
2	62	0	3	1	0						
2	68	4	2	2	3	4	1				
2	69	1	1	1	2	1	2	0	0		
2	71	3	0	2							
2	72	2	3	2	2	2	1	2	3		
3	83	8	8	3	8						
3	84	6	8	9	8	2	8				
3	85	6									
3	86	1	9	8	8	6					
3	87	5	8	4	8	8					
3	95	8	8	8	8						
4	117	0	0	2							
4	118	5	2	4	3	0	0				

Proj.No.: 60R0375/88R002

Number of areolas/nipples ♂ pups PND 12

Test-group:	♂ Pup Nr.: / Dam No.:	1	2	3	4	5	6	7	8	9	10
0	23	3	2	4							
0	24	1	0	2	0	1	2				
0	25	2	1	0	2	0					
1	49	3	3	3	2	4					
1	50	1	2	0	2						
2	70	0	4	2							
2	75	0	2	4	2	1	3				
3	93	6	8	2	8	8					
3	94	6	4	6	8	6	8				
3	96	5	6	8	8	8					
3	97	6	6	8	8	8	8	8			
4	119	0	0	0							
4	120	0	2	0							
4	123	3	4	2	2						
4	124	1	2	4							
4	125	2	4	2	2	0	4	2			

Proj.No.: 60R0375/88R002

Number of areolas/nipples ♂ pups PND 12

Test-group:	♂ Pup Nr.: / Dam No.:	1	2	3	4	5	6	7	8	9	10
2	73	3	1	2	0						
2	74	4	0	3	2						
3	98	2	3	8	6						
3	99	6	8	5	8	4					
3	100	8	8	8	8						

Proj.No.: 60R0375/88R002

Number of areolas/nipples ♂ pups PND 20

Test-group:	♂ Pup Nr.: / Dam No.:	1	2	3	4	5	6	7	8	9	10
1	30	0	0	0	0	0	0	0			
4	103	0	0	0	0						

Proj.No.: 60R0375/88R002

Number of areolas/nipples ♂ pups PND 20

Test-group:	♂ Pup Nr.: / Dam No.:	1	2	3	4	5	6	7	8	9	10
0	1	0	0	0	0	0	0				
0	2	0	0	0	0	0					
0	3	0	0	0	0	0					
0	4	0	0	0	0						
0	5	0	0	0	0	0	0	0			
0	6	0	0	0	0						
0	7	0	0	0	0	0					
1	26	0	0	0	0	0					
1	27	0	0	0	0						
1	28	0	0	0	0						
1	29	0	0	0	0	0	0	0	0	0	
1	31	0	0								
1	32	0	0								
1	37	0	0	0	0	0					
2	51	0	0	0							
2	52	0	0	0	0	0					
2	53	0	0	0	0	0					
2	54	0	0	0	0	0	0				
2	55	0	0	0	0	0	0	0			
2	57	0	0	0	0	0	0				
3	77	0	0	0	0						
4	102	0	0	0	0	0	0	0			
4	104	0	0	0	0	0	0	0	0		
4	105	0	0	0	0	0					
4	106	0	0	0	0	0	0				
4	107	0	0	0	0						

Proj.No.: 60R0375/88R002

Number of areolas/nipples ♂ pups PND 20

Test-group:	♂ Pup Nr.: / Dam No.:	1	2	3	4	5	6	7	8	9	10
0	8	0	0	0	0						
0	9	0	0	0	0						
0	10	0	0	0	0	0					
0	11	0	0	0	0	0					
0	22	0	0	0	0						
1	33	0	0	0	0						
1	34	0	0	0	0	0					
1	35	0	0	0							
1	36	0	0	0	0						
1	45	0	0	0	0	0					
1	46	0	0	0	0	0	0	0			
2	56	0									
2	59	0	0	0	0						
2	60	0	0	0	0						
2	61	0	0	0	0	0					
3	78	2	0	1	0	4	3	2			
3	80	0	0	2							
3	82	0	0	3	3	1	4				
4	101	0	0	0	0	0	0				
4	108	0	0	0							
4	109	0	0	0	0						
4	111	0	0	0	0	0	0				
4	121	0									

Proj.No.: 60R0375/88R002

Number of areolas/nipples ♂ pups PND 20

Test-group:	♂ Pup Nr.: / Dam No.:	1	2	3	4	5	6	7	8	9	10
0	17	0	0								
0	18	0	0	0	0	0					
0	19	0	0								
0	20	0	0								
0	21	0	0	0	0	0	0	0			
1	43	0	0	0	0	0	0				
1	47	0	0	0	0						
2	58	0	0	0	0	0					
2	62	0	0	0	0	0	0				
2	68	0	0	0	0						
2	69	0	0	0	0	0	0	0	0		
2	71	0	0	0							
2	72	0	0	0	0	0	0	0	0		
3	83	1	1	2	0						
3	84	2	1	0	2	0					
3	85	2									
3	86	0	4	1	0	0					
3	87	0	2	0	2	1					
3	95	3	2	2	0						
4	117	0	0	0							
4	118	0	0	0	0	0	0				

Proj.No.: 60R0375/88R002

Number of areolas/nipples ♂ pups PND 20

Test-group:	♂ Pup Nr.: / Dam No.:	1	2	3	4	5	6	7	8	9	10
0	23	0	0	0							
0	24	0	0	0	0	0	0				
0	25	0	0	0	0	0					
1	49	0	0	0	0						
1	50	0	0	0	0	0					
2	70	0	0	0							
2	75	0	0	0	0	0	0				
3	93	1	1	0	0	1					
3	94	1	0	0	2	0	0				
3	96	0	0	0	0	2					
3	97	0	0	0	0	1	0	0			
4	119	0	0	0							
4	120	0	0	0							
4	123	0	0	0	0						
4	124	0	0	0							
4	125	0	0	0	0	0	0	0			

Proj.No.: 60R0375/88R002

Number of areolas/nipples ♂ pups PND 20

Test-group:	♂ Pup Nr.: / Dam No.:	1	2	3	4	5	6	7	8	9	10
2	73	0	0	0	0						
2	74	0	0	0	0						
3	98	0	0	0	0						
3	99	4	1	0	4	0					
3	100	3	2	2	1						

Proj.No.: 60R0375/88R002

Subset 2

Number of areolas/nipples ♂ pups PND 38

Test group	Subset 2 No.	Dam / Pup	Number
0	501	3 / 1	0
0	502	4 / 4	0
0	503	6 / 2	0
0	504	8 / 4	0
0	505	10 / 2	0
0	506	22 / 4	0
0	507	17 / 1	0
0	508	19 / 1	0
0	509	21 / 5	0
0	510	23 / 2	0
3	531	77 / 3	3
3	532	80 / 1	1
3	533	83 / 4	1
3	534	86 / 2	2
3	535	87 / 1	0
3	536	95 / 1	4
3	537	93 / 3	1
3	538	96 / 5	2
3	539	98 / 3	4
3	540	99 / 1	4

Proj.No.: 60R0375/88R002

Subset 3

Number of areolas/nipples ♂ pups PND 38

Test group	Subset 3 No.	Dam / Pup	Number
0	701	1 / 4	0
0	702	4 / 2	0
0	704	9 / 1	0
0	705	10 / 5	0
0	706	22 / 1	0
0	707	18 / 3	0
0	708	20 / 2	0
0	709	24 / 3	0
0	710	25 / 5	0
3	731	78 / 7	5
3	732	82 / 2	4
3	733	84 / 3	5
3	734	85 / 1	2
3	735	87 / 5	2
3	736	94 / 4	2
3	737	96 / 3	2
3	738	97 / 7	1
3	739	99 / 2	4
3	740	100 / 4	4

Proj.No.: 60R0375/88R002

Subset 2

Number of areolas/nipples ♂ pups (preputial seperation: positive)

Test group	Subset 2 No.	Dam / Pup	Number
0	501	3 / 1	0
0	502	4 / 4	0
0	503	6 / 2	0
0	504	8 / 4	0
0	505	10 / 2	0
0	506	22 / 4	0
0	507	17 / 1	0
0	508	19 / 1	0
0	509	21 / 5	0
0	510	23 / 2	0
3	531	77 / 3	4
3	532	80 / 1	2
3	533	83 / 4	2
3	534	86 / 2	4
3	535	87 / 1	2
3	536	95 / 1	4
3	537	93 / 3	1
3	538	96 / 5	5
3	539	98 / 3	4
3	540	99 / 1	4

Proj.No.: 60R0375/88R002

Subset 3

Number of areolas/nipples ♂ pups (preputial seperation: positive)

Test group	Subset 3 No.	Dam / Pup	Number
0	701	1 / 4	0
0	702	4 / 2	0
0	704	9 / 1	0
0	705	10 / 5	0
0	706	22 / 1	0
0	707	18 / 3	0
0	708	20 / 2	0
0	709	24 / 3	0
0	710	25 / 5	0
3	731	78 / 7	2
3	732	82 / 2	3
3	733	84 / 3	4
3	734	85 / 1	2
3	735	87 / 5	4
3	736	94 / 4	4
3	737	96 / 3	4
3	738	97 / 7	3
3	739	99 / 2	2
3	740	100 / 4	4

Selection for Subset I

Project-No.: 60R0375/88R002

Dam No.	Pup No. ♂	Subset No.	Dam No.	Pup No. ♀	Subset No.	Test group
1	2	**301**	3	6	**401**	0 (0 mg/kg bw/d)
2	5	**302**	4	9	**402**	
5	3	**303**	6	5	**403**	
7	1	**304**	8	7	**404**	
9	4	**305**	10	10	**405**	
11	3	**306**	22	6	**406**	
18	2	**307**	17	4	**407**	
21	7	**308**	19	7	**408**	
24	6	**309**	20	10	**409**	
25	1	**310**	23	8	**410**	
27	1	**311**	30	12	**411**	1 (ADI-MIX)
29	8	**312**	26	7	**412**	
31	1	**313**	28	5	**413**	
37	5	**314**	32	8	**414**	
33	2	**315**	35	4	**415**	
34	3	**316**	46	13	**416**	
36	1	**317**	43	10	**417**	
45	4	**318**	44	2	**418**	
47	3	**319**	48	6	**419**	
50	2	**320**	49	10	**420**	
52	3	**321**	51	7	**421**	2 (NOAEL-MIX)
53	1	**322**	55	8	**422**	
54	5	**323**	57	12	**423**	
56	2	**324**	59	5	**424**	
60	1	**325**	61	9	**425**	
58	4	**326**	68	6	**426**	
62	4	**327**	71	9	**427**	
69	8	**328**	70	4	**428**	
72	1	**329**	73	7	**429**	
75	6	**330**	74	6	**430**	
78	2	**331**	77	8	**431**	3 (LOAEL-MIX)
80	3	**332**	83	10	**432**	
82	6	**333**	85	5	**433**	
84	1	**334**	87	7	**434**	
86	4	**335**	95	7	**435**	
93	5	**336**	94	9	**436**	
97	4	**337**	96	8	**437**	
98	2	**338**	98	5	**438**	
99	3	**339**	99	7	**439**	
100	1	**340**	100	9	**440**	
103	2	**341**	103	10	**441**	4 (0.00025 mg/kdbw/)
102	3	**342**	107	5	**442**	
104	1	**343**	108	8	**443**	
105	4	**344**	121	9	**444**	
106	2	**345**	117	8	**445**	
101	6	**346**	118	11	**446**	
109	3	**347**	119	7	**447**	
111	6	**348**	120	5	**448**	
123	4	**349**	124	11	**449**	
125	1	**350**	125	8	**450**	

Selection for Subset II

Project-No.: 60R0375/88R002

Dam No.	Pup No. ♂	Subset No.	Dam No.	Pup No. ♀	Subset No.	Test group
3	1	**501**	1	7	**601**	0 (0 mg/kg bw/d)
4	4	**502**	2	10	**602**	
6	2	**503**	5	8	**603**	
8	4	**504**	7	6	**604**	
10	2	**505**	9	5	**605**	
22	4	**506**	11	12	**606**	
17	1	**507**	18	8	**607**	
19	1	**508**	20	3	**608**	
21	5	**509**	24	7	**609**	
23	2	**510**	25	9	**610**	
26	3	**511**	30	9	**611**	1 (ADI-MIX)
28	2	**512**	27	9	**612**	
29	6	**513**	31	10	**613**	
33	1	**514**	32	4	**614**	
34	5	**515**	37	9	**615**	
35	3	**516**	36	6	**616**	
46	7	**517**	45	6	**617**	
43	4	**518**	44	8	**618**	
47	2	**519**	48	1	**619**	
49	4	**520**	50	8	**620**	
51	2	**521**	52	10	**621**	2 (NOAEL-MIX)
55	4	**522**	53	8	**622**	
57	1	**523**	54	7	**623**	
59	3	**524**	60	8	**624**	
61	2	**525**	58	6	**625**	
62	3	**526**	68	10	**626**	
72	5	**527**	69	9	**627**	
70	1	**528**	71	6	**628**	
75	3	**529**	73	9	**629**	
74	1	**530**	74	8	**630**	
77	3	**531**	78	9	**631**	3 (LOAEL-MIX)
80	1	**532**	82	8	**632**	
83	4	**533**	84	7	**633**	
86	2	**534**	85	9	**634**	
87	1	**535**	87	10	**635**	
95	1	**536**	94	7	**636**	
93	3	**537**	96	9	**637**	
96	5	**538**	97	9	**638**	
98	3	**539**	98	10	**639**	
99	1	**540**	100	5	**640**	
103	1	**541**	103	7	**641**	4 (0.00025 mg/kdbw/)
102	5	**542**	106	8	**642**	
104	8	**543**	107	6	**643**	
105	3	**544**	108	7	**644**	
101	2	**545**	109	9	**645**	
111	3	**546**	121	2	**646**	
118	5	**547**	117	7	**647**	
119	1	**548**	120	6	**648**	
123	1	**549**	124	5	**649**	
124	2	**550**	125	10	**650**	

Selection for Subset III

Project-No.: 60R0375/88R002

Dam No.	Pup No. ♂	Subset No.	Dam No.	Pup No. ♀	Subset No.	Test group
1	4	**701**	2	8	**801**	0 (0 mg/kg bw/d)
4	2	**702**	3	7	**802**	
6	4	**703**	5	10	**803**	
9	1	**704**	7	7	**804**	
10	5	**705**	8	9	**805**	
22	1	**706**	11	6	**806**	
18	3	**707**	17	8	**807**	
20	2	**708**	19	4	**808**	
24	3	**709**	21	11	**809**	
25	5	**710**	23	5	**810**	
30	4	**711**	27	6	**811**	1 (ADI-MIX)
26	2	**712**	29	10	**812**	
28	1	**713**	31	3	**813**	
32	2	**714**	37	7	**814**	
36	4	**715**	33	6	**815**	
45	3	**716**	34	9	**816**	
46	1	**717**	35	8	**817**	
43	6	**718**	44	5	**818**	
49	2	**719**	47	5	**819**	
50	1	**720**	48	8	**820**	
53	2	**721**	51	4	**821**	2 (NOAEL-MIX)
54	3	**722**	52	6	**822**	
57	5	**723**	55	10	**823**	
61	5	**724**	59	7	**824**	
58	3	**725**	60	11	**825**	
68	1	**726**	62	7	**826**	
69	5	**727**	72	11	**827**	
71	3	**728**	70	6	**828**	
73	2	**729**	75	7	**829**	
74	4	**730**	73	6	**830**	
78	7	**731**	77	5	**831**	3 (LOAEL-MIX)
82	2	**732**	80	7	**832**	
84	3	**733**	83	9	**833**	
85	1	**734**	86	6	**834**	
87	5	**735**	95	10	**835**	
94	4	**736**	93	6	**836**	
96	3	**737**	94	8	**837**	
97	7	**738**	96	6	**838**	
99	2	**739**	98	6	**839**	
100	4	**740**	99	8	**840**	
103	4	**741**	102	9	**841**	4 (0.00025 mg/kdbw/)
107	1	**742**	104	11	**842**	
108	3	**743**	105	6	**843**	
121	1	**744**	106	7	**844**	
117	2	**745**	101	8	**845**	
118	1	**746**	109	5	**846**	
119	2	**747**	111	9	**847**	
120	3	**748**	119	5	**848**	
124	3	**749**	123	6	**849**	
125	7	**750**	124	9	**850**	

16-APR-14

TABLE :

HISTORICAL CONTROL DATA

01-JAN-07 to 01-MAR-14

SPECIES: RAT
STRAIN: WISTAR
SUPPLIER: CHARLES RIVER
FILENAME: 88R002

Study No./Control Group		Start Date	End Date	Route of Administration
00165F1	1	23-JUN-08		DIET
00165F2	1	27-OCT-08		DIET
01232F1	1	2-FEB-09		DRINKING WATER
01232F2	1	8-JUN-09		DRINKING WATER
01R001L1	1	14-JUN-10		DIET
01R001L2	1	18-OCT-10		DIET
02087F1	1	11-JUN-07		DIET
02087F2	1	15-OCT-07		DIET
03091F1	1	26-MAR-07		DIET
03091F2	1	6-AUG-07		DIET
04084F2	1	29-JAN-07		DIET
04112F1	1	15-SEP-08		DIET
05055F2	1	26-FEB-07		DIET
05092F1	1	26-MAY-08		DIET
05092F2	1	29-SEP-08		DIET
06R007L1	1	28-FEB-11		DIET
07101F1	1	11-AUG-08		GAVAGE
07101F2	1	15-DEC-08		GAVAGE
08062F1	1	4-MAY-09		DIET
08070F1	1	29-JUN-09		GAVAGE
09R003L1	1	19-APR-10		DIET
09014F1	1	12-OCT-09		GAVAGE

TABLE :
01-JAN-07 to 01-MAR-14

HISTORICAL CONTROL DATA
MEAN MATERNAL BODY WEIGHTS DURING GESTATION GRAMS

SPECIES: RAT
STRAIN: WISTAR
SUPPLIER: CHARLES RIVER

	NO. OF ANIMALS	MEAN	RANGE OF ACTUAL VALUES		95 % SPREAD 2.5%	97.5%
DAY 0	522	227.3	172.7	298.9	191.2	263.5
DAY 7	522	249.7	188.7	331.3	211.6	287.9
DAY 14	522	272.7	207.7	350.3	231.9	313.5
DAY 20	522	326.7	225.6	418.3	274.2	379.2

16-APR-14

TABLE :

SPECIES RAT

HISTORICAL CONTROL DATA

DATES: 01-JAN-07 - 01-MAR-14

STRAIN WISTAR
SUPPLIER CHARLES RIVER

MEAN MATERNAL BODY WEIGHTS DURING LACTATION -- GRAMS

	NO. OF ANIMALS	MEAN	RANGE OF ACTUAL VALUES		95 % SPREAD 2.5%	97.5%
DAY 0	87	239.9	187.6	288.3	201.2	278.6
DAY 1	518	249.8	180.8	331.3	208.8	290.9
DAY 4	518	264.4	192.7	348.6	223.8	305.0
DAY 7	518	270.4	199.5	338.1	231.4	309.3
DAY 14	518	282.4	203.5	358.3	241.3	323.5
DAY 21	517	273.7	198.3	329.1	236.5	310.9

16-APR-14

TABLE :

SPECIES RAT

HISTORICAL CONTROL DATA

DATES: 01-JAN-07 - 01-MAR-14

STRAIN WISTAR

MEAN ESTROUS CYCLE DURATION (days)

SUPPLIER CHARLES RIVER

NO. OF ANIMALS	MEAN	RANGE OF ACTUAL VALU		95 % SPREAD 2.5%	97.5%
320	4.7	3.3	20.0	3.3	8.5

25-SEP-13

TABLE:

HISTORICAL CONTROL DATA

REPRODUCTION AND LITTER DATA

SPECIES RAT
STRAIN WISTAR
SUPPLIER CHARLES RIVER

STUDY NO.		RUNTIME	FEMALES MATED [N]	MALE MATING INDEX [%]	MALE FERTILITY INDEX [%]	FEMALE MATING INDEX [%]	MATING DAYS – 0 P.C. [DAYS]	FEMALE FERTILITY INDEX [%]	DURATION OF GESTATION [DAYS]	IMPLANTATION SITES [MEAN]
00037	F1	2000	25	100	100	100	2.7	100	21.8	11.0
	F2	2001	24	100	96	100	2.5	96	22.1	10.2
00120	F1	2001	25	100	96	100	2.5	96	21.9	--[1)]
00165	F1	2008	24	96	96	96	2.9	100	22.2	11.9
	F2	2008	25	100	96	100	3.0	96	21.9	12.6
01021	F1	2001	25	100	100	100	2.8	100	22.0	--[1)]
	F1B	2002	25	100	96	100	2.2	96	22.0	--[1)]
	F2	2002	25	100	96	100	2.5	96	22.1	11.0
01057	F1	2002	25	100	96	100	2.6	96	22.1	12.0
	F2	2002	25	100	96	100	3.0	96	22.0	12.4
01058	F1	2002	25	100	96	100	2.3	96	22.1	--[1)]
	F1B	2002	23	100	100	100	2.4	100	22.3	--[1)]
	F2	2003	25	96	96	96	2.5	100	22.1	11.7
01086	F1	2003	25	100	96	100	2.8	96	21.9	--[1)]
	F1B	2003	25	100	92	100	2.8	92	22.2	--[1)]
	F2	2003	24	100	92	100	2.1	92	21.5	10.6
01140	F1	2004	25	100	100	100	2.4	100	22.1	10.3
01200	F1	2006	25	100	100	100	2.8	100	21.8	12.2
	F2	2006	25	100	96	100	2.7	96	21.7	10.8
01232	F1	2009	25	100	96	100	2.5	96	21.9	11.7
	F2	2009	25	100	100	100	2.2	100	22.1	12.2

1) No Data available

TABLE:

HISTORICAL CONTROL DATA

REPRODUCTION AND LITTER DATA

SPECIES RAT
STRAIN WISTAR
SUPPLIER CHARLES RIVER

STUDY NO.		RUNTIME	FEMALES MATED [N]	MALE MATING INDEX [%]	MALE FERTILITY INDEX [%]	FEMALE MATING INDEX [%]	MATING DAYS – 0 P.C. [DAYS]	FEMALE FERTILITY INDEX [%]	DURATION OF GESTATION [DAYS]	IMPLANTATION SITES [MEAN]
01R001	L1	2010	25	100	100	100	2.4	100	22.0	12.1
	L2	2010	25	100	92	100	3.5	92	22.1	12.4
02017	F1	2002	24	96	96	96	2.5	100	22.0	10.8
02075	F1	2004	25	100	92	100	2.2	92	21.9	11.6
	F2	2004	24	100	88	100	2.6	88	21.9	10.8
02080	F1	2005	25	100	100	100	3.1	100	22.0	12.1
	F2	2006	25	100	100	100	2.5	100	22.2	11.9
02087	F1	2007	24	100	100	100	2.8	100	22.3	13.3
	F2	2007	25	100	96	100	2.7	96	22.1	13.7
03041	F1	2004	25	100	100	100	2.8	100	22.1	10.2
	F2	2004	25	100	96	100	2.4	96	22.1	11.1
03091	F1	2007	24	96	96	96	5.1	100	22.1	11.9
	F2	2007	25	100	92	100	5.3	92	22.0	12.1
04084	F1	2006	25	100	100	100	2.8	100	22.0	11.3
	F2	2007	25	100	96	100	3.0	96	22.0	12.3
04112	F1	2008	25	100	92	100	2.6	92	22.4	13.1
05040	F1	2006	25	100	96	100	3.1	96	22.1	12.8
05055	F1	2006	25	100	100	100	2.4	100	21.8	11.8
	F2	2007	25	100	96	100	2.5	96	21.8	11.3
05092	F1	2008	25	100	96	100	2.2	96	22.5	12.3
	F2	2008	25	100	96	100	2.8	96	22.0	12.0
06R007	L1	2011	25	100	100	100	2.9	100	22.0	12.9

25-SEP-13

TABLE:

HISTORICAL CONTROL DATA

REPRODUCTION AND LITTER DATA

SPECIES RAT
STRAIN WISTAR
SUPPLIER CHARLES RIVER

STUDY NO.		RUNTIME	FEMALES MATED [N]	MALE MATING INDEX [%]	MALE FERTILITY INDEX [%]	FEMALE MATING INDEX [%]	MATING DAYS – 0 P.C. [DAYS]	FEMALE FERTILITY INDEX [%]	DURATION OF GESTATION [DAYS]	IMPLANTATION SITES [MEAN]
07101	F1	2008	25	100	88	100	2.6	88	22.2	13.4
	F2	2008	25	100	96	100	2.8	96	22.2	12.2
08062	F1	2009	25	100	100	100	2.8	100	22.0	13.0
08070	F1	2009	25	100	96	100	2.7	96	22.0	12.0
09014	F1	2009	20	100	95	100	2.7	95	22.1	11.6
09R003	L1	2010	25	100	100	100	2.6	100	21.9	13.3
97170	F1	2000	25	96	92	96	3.0	96	22.0	10.8
	F2	2000	27	100	100	100	2.2	100	22.1	10.6
98132	F1	2000	25	100	92	100	2.6	92	21.8	10.9
	F2	2001	25	100	96	100	2.7	96	22.1	11.0
98136	F1	2000	25	100	100	100	2.3	100	21.9	11.1
	F2	2001	24	100	96	100	2.3	96	22.0	11.2
99080	F1	2000	25	100	96	100	3.1	96	22.1	--[1)]
99099	F1	2000	25	100	96	100	3.6	96	22.0	11.5
99118	F1A	2001	25	100	84	100	2.6	84	21.9	--[1)]
	F1B	2001	25	100	96	100	2.5	96	21.8	--[1)]
	F2A	2001	24	96	88	96	2.1	92	21.9	--[1)]
	F2B	2001	23	92	88	92	2.3	96	21.7	--[1)]
99140	F1	2002	25	100	92	100	2.7	92	22.0	10.8
	F2	2002	24	96	96	96	2.8	100	22.2	11.2
RANGE										
	Min.	2000	20	92	84	92	2.1	84	21.5	10.2
	Max.	2011	27	100	100	100	5.3	100	22.5	13.7

1) No Data available

TABLE:

HISTORICAL CONTROL DATA

REPRODUCTION AND LITTER DATA

SPECIES RAT
STRAIN WISTAR
SUPPLIER CHARLES RIVER

STUDY NO.		DAMS WITH TOTAL LITTER LOSS [N]	STILLBORN PUPS [N]	%POSTIMPLANTATION LOSS [MEAN]	GESTATION INDEX [%]	PUPS DEL/DAM [MEAN]	PUPS STILLBORN [%]	LIVE BIRTH INDEX [%]	VIABILITY INDEX [%]	LACTATION INDEX [%]
00037	F1	0	0	3.5	100	10.6	3.0	97	97	99
	F2	0	0	8.4	96	10.1	0.9	99	99	99
00120	F1	0	0	--[1]	100	11.5	1.1	99	97	99
00165	F1	0	0	8.3	100	11.0	4.2	96	98	100
	F2	0	0	5.1	100	12.0	0.7	99	98	100
01021	F1	0	0	--[1]	100	11.0	2.9	97	98	100
	F1B	0	0	--[1]	100	10.7	1.6	98	100	99
	F2	0	0	5.9	100	10.3	0.8	99	100	98
01057	F1	0	0	8.1	100	11.0	1.1	99	98	100
	F2	0	0	8.8	100	11.4	0.7	99	97	99
01058	F1	0	0	--[1]	100	10.7	0.0	100	99	100
	F1B	1	1	--[1]	96	9.3	2.2	98	100	100
	F2	0	0	5.6	100	11.0	1.6	98	94	99
01086	F1	0	0	--[1]	100	11.0	4.5	95	95	100
	F1B	1	1	--[1]	91	10.5	1.3	99	100	100
	F2	0	0	7.8	95	10.6	1.3	99	99	100
01140	F1	1	1	5.9	92	9.9	4.2	99	99	99
01200	F1	0	0	6.8	100	11.4	0.7	99	100	100
	F2	0	0	5.0	100	10.3	1.6	98	100	100
01232	F1	0	0	11.4	100	10.3	0.8	99	99	98
	F2	0	0	11.5	100	10.9	0.7	99	99	99

1) No Data available

25-SEP-13

TABLE:

HISTORICAL CONTROL DATA

REPRODUCTION AND LITTER DATA

SPECIES RAT
STRAIN WISTAR
SUPPLIER CHARLES RIVER

STUDY NO.		DAMS WITH TOTAL LITTER LOSS [N]	STILLBORN PUPS [N]	%POSTIMPLANTATION LOSS [MEAN]	GESTATION INDEX [%]	PUPS DEL/DAM [MEAN]	PUPS STILLBORN [%]	LIVE BIRTH INDEX [%]	VIABILITY INDEX [%]	LACTATION INDEX [%]
01R001	L1	0	0	5.9	100	11.4	0.7	99	99	100
	L2	0	0	9.7	100	11.4	0.8	99	99	100
02017	F1	0	0	7.4	100	10.0	2.1	98	98	99
02075	F1	0	0	4.4	100	11.0	0.0	100	99	99
	F2	0	0	7.2	100	10.1	1.9	98	99	100
02080	F1	0	0	9.7	100	11.0	2.2	98	99	95
	F2	0	0	4.0	100	11.4	1.4	99	99	100
02087	F1	0	0	6.0	100	12.4	2.7	97	99	100
	F2	0	0	6.7	100	12.8	0.0	100	99	100
03041	F1	0	0	5.9	100	9.6	1.3	99	100	100
	F2	0	0	9.0	100	10.1	0.4	100	100	99
03091	F1	0	0	3.9	100	11.4	0.7	99	99	100
	F2	0	0	7.9	100	11.2	1.2	99	100	98
04084	F1	0	0	9.0	100	10.3	0.4	100	99	99
	F2	1	2	12.9	96	10.7	1.2	99	100	99
04112	F1	0	0	8.3	100	12.1	1.1	99	99	98
05040	F1	0	0	8.1	100	11.8	0.0	100	100	100
05055	F1	0	0	5.7	100	11.1	0.7	99	99	100
	F2	0	0	4.9	100	10.8	0.0	100	100	100
05092	F1	0	0	10.7	96	11.8	2.2	98	100	100
	F2	0	0	9.3	96	11.9	1.5	99	99	100
06R007	L1	0	0	5.0	100	12.2	0.3	100	97	--[1)]

1) No Data available

TABLE:

HISTORICAL CONTROL DATA

REPRODUCTION AND LITTER DATA

SPECIES RAT
STRAIN WISTAR
SUPPLIER CHARLES RIVER

STUDY NO.		DAMS WITH TOTAL LITTER LOSS [N]	STILLBORN PUPS [N]	%POSTIMPLANTATION LOSS [MEAN]	GESTATION INDEX [%]	PUPS DEL/DAM [MEAN]	PUPS STILLBORN [%]	LIVE BIRTH INDEX [%]	VIABILITY INDEX [%]	LACTATION INDEX [%]
07101	F1	0	0	7.3	100	12.4	1.1	99	99	99
	F2	0	0	9.7	100	11.1	0.8	99	99	100
08062	F1	0	0	5.0	100	12.3	2.3	98	94	100
08070	F1	0	0	11.1	96	11.4	0.0	100	100	99
09014	F1	0	0	17.7	95	10.8	0.0	100	100	100
09R003	L1	0	1	4.6	100	12.7	0.3	100	99	100
97170	F1	0	0	3.5	100	10.4	1.2	99	98	97
	F2	0	0	3.7	100	10.2	2.5	97	97	99
98132	F1	0	0	4.1	100	10.4	0.8	99	98	98
	F2	0	0	7.4	100	10.1	0.4	100	98	100
98136	F1	0	0	2.5	100	10.8	1.5	99	100	100
	F2	0	0	6.8	100	10.4	0.8	99	98	95
99080	F1	1	7	--[1)]	96	9.8	4.3	96	98	99
99099	F1	0	0	7.4	100	10.5	2.0	98	97	99
99118	F1A	0	0	--[1)]	100	9.9	1.0	99	100	99
	F1B	0	0	--[1)]	100	11.0	0.4	100	99	99
	F2A	0	0	--[1)]	100	11.2	0.0	100	97	99
	F2B	0	0	--[1)]	100	11.7	0.4	100	96	100
99140	F1	0	0	5.8	96	11.0	2.1	98	98	99
	F2	0	0	6.0	100	10.6	1.6	98	96	99
RANGE	Min.	0	0	2.5	91	9.3	0.0	95	94	95
	Max.	1	7	17.7	100	12.8	4.5	100	100	100

1) No Data available

16-APR-14

TABLE :

SPECIES RAT

HISTORICAL CONTROL DATA

DATES: 01-JAN-07 01-MAR-14

STRAIN WISTAR

PUP WEIGHTS - grams

SUPPLIER CHARLES RIVER

DAY		NO. OF LITTERS	MEAN	RANGE OF LITTER MEANS		RANGE OF STUDY MEANS	
1	MALES	514	6.5	4.5	8.9	5.9	7.0
	FEMALES	515	6.2	4.0	8.4	5.7	6.7
	BOTH	517	6.3	4.2	8.4	5.8	6.9
4	MALES	514	9.9	6.7	14.8	9.3	10.8
	FEMALES	515	9.6	6.3	14.0	8.9	10.4
	BOTH	517	9.7	6.5	14.4	9.2	10.6
7	MALES	513	16.1	8.0	35.2	14.7	17.6
	FEMALES	515	15.6	9.8	21.1	14.2	16.9
	BOTH	517	15.8	11.1	24.9	14.7	17.3
14	MALES	513	32.3	22.6	43.1	29.3	35.1
	FEMALES	515	31.6	20.6	41.7	28.7	34.2
	BOTH	517	31.9	20.9	42.8	29.2	34.7
21	MALES	512	51.4	35.7	66.8	46.5	58.3
	FEMALES	514	49.8	33.0	66.7	45.5	55.7
	BOTH	517	50.6	37.1	66.8	46.2	56.8

16-APR-14

TABLE :

SPECIES RAT

HISTORICAL CONTROL DATA

PUP NECROPSY OBSERVATIONS

DATES: 01-JAN-07 - 01-MAR-14

STRAIN WISTAR
SUPPLIER CHARLES RIVER

	PUPS N	PUPS %	%RANGE (per study) %	%RANGE (per study) %	LITTERS N	LITTERS %	%RANGE (per study) %	%RANGE (per study) %MEAN	AFFECTED PUPS /LITTER %MEAN %	RANGE (per study) %	RANGE (per study) %
NUMBER EVALUATED	5595				518						
Live	5539										
Dead	56										
POST MORTEM AUTOLYSIS	11	0.2	0.0	2.7	5	1.0	0.0	8.3	0.2	0.0	2.7
PARTLY CANNIBALIZED	1	0.02	0.0	0.4	1	0.2	0.0	4.0	0.0	0.0	0.4
INCISORS SLOPED	4	0.07	0.0	0.4	4	0.8	0.0	4.3	0.1	0.0	0.4
SITUS INVERSUS	1	0.02	0.0	0.4	1	0.2	0.0	4.3	0.0	0.0	0.6
AGLOSSIA	1	0.02	0.0	0.5	1	0.2	0.0	4.2	0.0	0.0	0.4
MICROPHTHALMIA	1	0.02	0.0	0.5	1	0.2	0.0	4.2	0.0	0.0	0.4
HEMORRHAGIC THYMUS	3	0.05	0.0	0.6	3	0.6	0.0	8.0	0.1	0.0	0.6
DIAPHRAGMATIC HERNIA	3	0.05	0.0	0.5	3	0.6	0.0	4.2	0.1	0.0	2.1
SUPERNUMERARY LIVER LOBE	1	0.02	0.0	0.4	1	0.2	0.0	4.0	0.0	0.0	0.4
INFARCT OF LIVER	1	0.02	0.0	0.4	1	0.2	0.0	4.3	0.0	0.0	0.4
ABNORMAL LIVER LOBATION	1	0.02	0.0	0.4	1	0.2	0.0	4.3	0.0	0.0	0.4
EMPTY STOMACH	6	0.1	0.0	0.7	5	1.0	0.0	4.3	0.1	0.0	0.8
EMPTY DUODENUM	1	0.02	0.0	0.4	1	0.2	0.0	4.3	0.0	0.0	0.4
DILATED RENAL PELVIS	19	0.3	0.0	2.3	15	2.9	0.0	20.0	0.3	0.0	2.1
HYDROURETER	1	0.02	0.0	0.3	1	0.2	0.0	4.0	0.0	0.0	0.4
HEMORRHAGIC TESTIS	3	0.05	0.0	0.4	3	0.6	0.0	4.3	0.0	0.0	0.4
SMALL TESTIS	9	0.2	0.0	0.8	8	1.5	0.0	8.7	0.1	0.0	0.8

16-APR-14

TABLE :

SPECIES RAT

STRAIN WISTAR
SUPPLIER CHARLES RIVER

HISTORICAL CONTROL DATA

PUP NECROPSY OBSERVATIONS

DATES: 01-JAN-07 - 01-MAR-14

	PUPS		%RANGE (per study)		LITTERS		%RANGE (per study)		AFFECTED PUPS /LITTER	RANGE (per study)	
	N	%	%	%	N	%	%	%	%MEAN %	%	%
NUMBER EVALUATED	5595				518						
Live	5539										
Dead	56										
ABSENT TESTIS	1	0.02	0.0	0.4	1	0.2	0.0	4.3	0.0	0.0	0.4
ABSENT EPIDIDYMIS	1	0.02	0.0	0.4	1	0.2	0.0	4.3	0.0	0.0	0.4
OVARY: CYSTIC DILATATION	1	0.02	0.0	0.4	1	0.2	0.0	4.2	0.0	0.0	0.5
MANDIBULAR MICROGNATHIA	1	0.02	0.0	0.5	1	0.2	0.0	4.2	0.0	0.0	0.4
MANDIBULAR AGNATHIA	1	0.02	0.0	0.4	1	0.2	0.0	4.3	0.0	0.0	0.4
KINKED TAIL	1	0.02	0.0	0.5	1	0.2	0.0	4.2	0.0	0.0	0.5
MULTIPLE MALFORMED HINDLIMB	1	0.02	0.0	0.4	1	0.2	0.0	4.2	0.0	0.0	0.3
TOTAL PUP NECROPSY OBSERVATIONS	68	1.2	0.0	3.7	56	10.8	0.0	28.0	1.2	0.0	3.8

TABLE :

SPECIES RAT

HISTORICAL CONTROL DATA

DATES: 01-JAN-00 01-MAR-14

STRAIN WISTAR
SUPPLIER CHARLES RIVER
FileName: 88R002

SEXUAL MATURATION DATA
AGE AND WEIGHT OF PUPS REACHING CRITERIA

Study No./Control Group		Start Date	End Date	Route of Administration
00037F1	1	16-OCT-00		DIET
00165F1	1	23-JUN-08		DIET
01021F1	1	5-NOV-01		DIET
01021F2	1	11-MAR-02		DIET
01057F1	1	13-MAY-02		DIET
01057F2	1	16-SEP-02		DIET
01058F1	1	27-MAY-02		GAVAGE
01058F1B	1	30-SEP-02		GAVAGE
01086F1	1	27-JAN-03		DIET
01086F1B	1	9-JUN-03		DIET
01140F1	1	12-APR-04		DIET
01200F1	1	19-JUN-06		DIET
01232F1	1	2-FEB-09		DRINKING WATER
01R001L1	1	14-JUN-10		DIET
01R001L2	1	18-OCT-10		DIET
02017F1	1	4-NOV-02		DIET
02075F1	1	9-FEB-04		DIET
02080F1	1	17-OCT-05		DIET
02087F1	1	11-JUN-07		DIET
03041F1	1	3-MAY-04		GAVAGE
03091F1	1	26-MAR-07		DIET
04084F1	1	25-SEP-06		DIET
04112F1	1	15-SEP-08		DIET
05040F1	1	6-MAR-06		DIET
05055F1	1	23-OCT-06		DIET
05092F1	1	26-MAY-08		DIET
07101F1	1	11-AUG-08		GAVAGE
08062F1	1	4-MAY-09		DIET
08070F1	1	29-JUN-09		GAVAGE
09R003L1	1	19-APR-10		DIET
97170F1	1	29-MAY-00		DRINKING WATER
98132F1	1	16-OCT-00		DIET
98136F1	1	18-DEC-00		DIET
99099F1	1	17-JUL-00		GAVAGE
99118F1A	1	29-JAN-01		DIET
99140F1	1	28-JAN-02		DIET

25-AUG-14

TABLE :

SPECIES RAT

HISTORICAL CONTROL DATA

DATES: 01-JAN-00 01-MAR-14

STRAIN WISTAR
SUPPLIER CHARLES RIVER

SEXUAL MATURATION DATA
AGE AND WEIGHT OF PUPS REACHING CRITERIA

	NO. OF LITTERS	MEAN	RANGE OF STUDY MEANS	
PREPUTIAL SEPARATION				
age in days at criterion	747	42.4	39.7	45.1
body weight at criterion	745	172.0	156.5	181.0
VAGINAL OPENING				
age in days at criterion	746	31.6	30.0	33.8
body weight at criterion	746	92.7	85.5	101.5

CONTROL DATA OF STEROID HORMONES [1)]

Species:	Rat
Sex/Age	Dams after weaning in proestrus
Strain:	Wistar
Anaesthesia	Isoflurane
Data print out at	2-Jul-14
Estradiol	ELISA, DRG, Marburg, cat no EIA4399
Other steroid hormones	SPE-LC-MS/MS, metanomics, Berlin

Study No.	Androstenedione (nmol/L)				Testosterone (nmol/L)				Progesterone (nmol/L)				11-Deoxycorticosterone (nmol/L)				Corticosterone (nmol/L)				Estradiol (pmol/L)			
	N	Mean	SD	Median	N	Mean	SD	Median	N	Mean	SD	Median	N	Mean	SD	Median	N	Mean	SD	Median	N	Mean	SD	Median
88R001	20	2.15	1.44	1.96	20	0.52	0.14	0.5	19	40.6	50.16	11.83	20	3.37	3.88	1.52	20	435.8	485.6	268.8	20	10.45	11.17	4.83
03R003	19	1.5	0.74	1.42					19	18.3	6.53	16.76	19	11.75	10.74	8.27	19	852	516.5	1011.5	19	25.93	6.85	29.2
88R002	17	1.6	0.99	1.28	17	0.42	0.19	0.35	17	24.65	41.11	13.04	17	6.34	4.55	4.67	17	731.6	0.51	650.3	19	26.76	7.22	27.88

1) Source: All data were collected and archived at the test facility Experimental Toxicology and Ecology, BASF SE, 67056 Ludwigshafen, Germany, in accordance with the OECD principles of Good Laboratory Practice (GLP) and the GLP principles of the German "Chemikaliengesetz" (Chemicals Act)

CONTROL DATA OF STEROID HORMONES [1)]

Species:	Rat
Sex/Age	Males PND21
Strain:	Wistar
Anaesthesia	Isoflurane
Data print out at	2-Jul-14
Estradiol	ELISA, DRG, Marburg, cat no.EIA4399
Other steroid hormones	SPE-LC-MS/MS, metanomics, Berlin

Study No.	Androstenedione (nmol/L)				Testosterone (nmol/L)				Progesterone (nmol/L)				Corticosterone (nmol/L)				Cortisol (nmol/L)			
	N	Mean	SD	Median	N	Mean	SD	Median	N	Mean	SD	Median	N	Mean	SD	Median	N	Mean	SD	Median
88R001	9	1.03	1.57	0.37	10	0.86	0.94	0.5	9	6.49	4.04	5.25	9	756.2	197	713.8	10	0.82	0.35	0.66
03R003	4	0.62	0.35	0.52	4	0.7	0.53	0.48	5	6.21	4.12	7.22	5	604.8	273.5	670.5				
88R002	10	0.62	0.48	0.38	10	0.72	0.64	0.35	10	6.96	3.18	6.81	10	811.2	106.8	851.1	10	0.79	0.3	0.83

1) Source: All data were collected and archived at the test facility Experimental Toxicology and Ecology, BASF SE, 67056 Ludwigshafen, Germany, in accordance with the OECD principles of Good Laboratory Practice (GLP) and the GLP principles of the German "Chemikaliengesetz" (Chemicals Act)

CONTROL DATA OF STEROID HORMONES [1)]

Species:	Rat
Sex/Age	Females PND21
Strain:	Wistar
Anaesthesia	Isoflurane
Data print out at	2-Jul-14
Estradiol	ELISA, DRG, Marburg, cat no.EIA4399
Other steroid hormones	SPE-LC-MS/MS, metanomics, Berlin

Study No.	Androstenedione (nmol/L)				Progesterone (nmol/L)				Corticosterone (nmol/L)				Cortisol (nmol/L)			
	N	Mean	SD	Median	N	Mean	SD	Median	N	Mean	SD	Median	N	Mean	SD	Median
88R001	10	0.42	0.17	0.35	10	3.9	2.61	4.18	9	751	289.3	797.6	9	1.05	0.52	0.94
03R003	10	0.72	1.13	0.35	10	7.24	3.71	6.92	10	770.6	241.4	842.4				
88R002	10	0.35	0.02	0.35	9	5.52	2.5	5.37	10	802.6	211.4	751.4	10	1.02	0.51	1.07

1) Source: All data were collected and archived at the test facility Experimental Toxicology and Ecology, BASF SE, 67056 Ludwigshafen, Germany, in accordance with the OECD principles of Good Laboratory Practice (GLP) and the GLP principles of the German "Chemikaliengesetz" (Chemicals Act)

CONTROL DATA OF STEROID HORMONES [1)]

Species:	Rat
Sex/Age	Males at sexual maturity
Strain:	Wistar
Anaesthesia	Isoflurane
Data print out at	2-Jul-14
Estradiol	ELISA, DRG, Marburg, cat no.EIA4399
Other steroid hormones	SPE-LC-MS/MS, metanomics, Berlin

Study No.	Androstenedione (nmol/L)				Testosterone (nmol/L)				Progesterone (nmol/L)				11-Deoxycorticosterone (nmol/L)				Corticosterone (nmol/L)			
	N	Mean	SD	Median	N	Mean	SD	Median	N	Mean	SD	Median	N	Mean	SD	Median	N	Mean	SD	Median
88R001	10	1.6	1.71	0.72	10	4.72	4.13	3.19	10	7.24	8	3.98	10	4.91	4.25	2.76	10	765.9	660.2	377.2
03R003	10	0.54	0.28	0.39	10	2.03	1.71	1.34	10	7.96	5.62	6.93	10	8.08	6.78	6.04	10	814.1	363.8	893
88R002	10	0.65	0.54	0.4	10	3.06	3.06	1.74	10	1.95	1.6	1.56	10	2.43	1.01	2.3	10	466.8	335.5	504.3

1) Source: All data were collected and archived at the test facility Experimental Toxicology and Ecology, BASF SE, 67056 Ludwigshafen, Germany, in accordance with the OECD principles of Good Laboratory Practice (GLP) and the GLP principles of the German "Chemikaliengesetz" (Chemicals Act)

CONTROL DATA OF STEROID HORMONES [1)]

Species:	Rat
Sex/Age	Females at sexual maturity
Strain:	Wistar
Anaesthesia	Isoflurane
Data print out at	2-Jul-14
Estradiol	ELISA, DRG, Marburg, cat no.EIA4399
Other steroid hormones	SPE-LC-MS/MS, metanomics, Berlin

Study No.	Androstenedione (nmol/L)				Testosterone (nmol/L)				Progesterone (nmol/L)				11-Deoxycorticosterone (nmol/L)				Corticosterone (nmol/L)				Estradiol (pmol/L)			
	N	Mean	SD	Median	N	Mean	SD	Median	N	Mean	SD	Median	N	Mean	SD	Median	N	Mean	SD	Median	N	Mean	SD	Median
88R001	10	0.56	0.52	0.35	10	0.55	0.15	0.52	10	14.89	11.29	8.48	10	2.64	1.2	2.77	10	844.4	527.8	880	10	4.08	7.9	0
03R003	10	0.38	0.08	0.35					10	23.46	17.35	19.73	9	2.75	2.47	1.78	10	494.1	605.7	356	10	6.34	1.66	5.74
88R002	10	0.36	0.04	0.35	9	0.36	0.05	0.35	10	9.93	3.12	10.08	10	2.06	0.98	1.59	10	343.7	417.6	142.9	10	8.8	2.72	8.54

1) Source: All data were collected and archived at the test facility Experimental Toxicology and Ecology, BASF SE, 67056 Ludwigshafen, Germany, in accordance with the OECD principles of Good Laboratory Practice (GLP) and the GLP principles of the German "Chemikaliengesetz" (Chemicals Act)

CONTROL DATA OF STEROID HORMONES [1)]

Species:	Rat
Sex/Age	Males PND83
Strain:	Wistar
Anaesthesia	Isoflurane
Data print out at	2-Jul-14
Estradiol	ELISA, DRG, Marburg, cat no.EIA4399
Other steroid hormones	SPE-LC-MS/MS, metanomics, Berlin

Study No.	Androstenedione (nmol/L)				Testosterone (nmol/L)				Progesterone (nmol/L)				11-Deoxycorticosterone (nmol/L)				Corticosterone (nmol/L)			
	N	Mean	SD	Median	N	Mean	SD	Median	N	Mean	SD	Median	N	Mean	SD	Median	N	Mean	SD	Median
88R001	10	2.27	1.81	1.72	10	5.79	5.27	4.45	10	11.26	7.93	9.99	10	19.23	11.88	17.09	10	959.2	446.6	1020.2
03R003	10	1.29	0.73	1.18	10	6.15	5.94	4.94	9	9.27	8.84	5.79	9	21.14	15.75	17.24	10	838.9	390.2	965.3
88R002	8	1.15	0.84	0.88	8	6.48	4.76	5	8	2.06	1.7	1.63	8	7.39	6.13	5.04	8	434.5	250.3	395.9

1) Source: All data were collected and archived at the test facility Experimental Toxicology and Ecology, BASF SE, 67056 Ludwigshafen, Germany, in accordance with the OECD principles of Good Laboratory Practice (GLP) and the GLP principles of the German "Chemikaliengesetz" (Chemicals Act)

CONTROL DATA OF STEROID HORMONES [1)]

Species:	Rat
Sex/Age	Females in proestrus around PND83
Strain:	Wistar
Anaesthesia	Isoflurane
Data print out at	2-Jul-14
Estradiol	ELISA, DRG, Marburg, cat no.EIA4399
Other steroid hormones	SPE-LC-MS/MS, metanomics, Berlin

Study No.	Androstenedione (nmol/L)				Testosterone (nmol/L)				Progesterone (nmol/L)				11-Deoxycorticosterone (nmol/L)				Corticosterone (nmol/L)				Estradiol (pmol/L)			
	N	Mean	SD	Median	N	Mean	SD	Median	N	Mean	SD	Median	N	Mean	SD	Median	N	Mean	SD	Median	N	Mean	SD	Median
88R001	10	1.54	0.89	1.36	10	0.43	0.08	0.43	10	10.82	4.11	10.29	10	5.98	5.89	3.81	10	697.1	356.6	645.9	10	16.62	11.6	17.17
03R003	10	1.16	0.24	1.28					10	12	3.31	11.11	10	9.36	8.71	7.68	10	662	451.2	543.3	10	23.06	6.1	20.91
88R002	9	1.87	1.28	1.43	9	0.47	0.16	0.39	9	40.27	60.15	10.53	9	4.9	4.62	1.73	9	460.1	491.3	225.7	8	23.55	9.78	24.61

1) Source: All data were collected and archived at the test facility Experimental Toxicology and Ecology, BASF SE, 67056 Ludwigshafen, Germany, in accordance with the OECD principles of Good Laboratory Practice (GLP) and the GLP principles of the German "Chemikaliengesetz" (Chemicals Act)

CONTROL DATA OF MALE SEX ORGAN WEIGHTS [1)]

Species: Rat
Sex/Age: Males PND 21
Strain: Wistar
Breeder: Charles River Labs, Sulzfeld Germany
Data print out at 8-May-14

Absolute Male Sex Organ Weights on PND 21 (Subset 1)

Study No.	Cauda epididymis			Epididymides			Muscles bulb + l.ani		
	N	Mean	SD	N	Mean	SD	N	Mean	SD
88R001	10	12.56	4.582	10	30.79	7.063	10	35.49	10.009
03R003	10	11.6	3.806	10	31.4	3.373	10	50.22	9.632
88R002	10	10.0	3.916	10	29.0	5.981	10	35.45	8.759

Study No.	Prostate			Prostate ventr.fresh			Seminal vesicle			Testes		
	N	Mean	SD	N	Mean	SD	N	Mean	SD	N	Mean	SD
88R001	10	50.59	9.172	10	28.06	5.232	10	10.44	2.511	10	240.41	27.32
03R003	10	51.54	5.826	10	28.86	3.093	10	9.96	1.722	10	246	20
88R002	10	48.51	5.773	10	24.4	3.25	10	9.27	1.989	10	237.8	27.868

1) Source: All data were collected and archived at the test facility Experimental Toxicology and Ecology, BASF SE, 67056 Ludwigshafen, Germany, in accordance with the OECD principles of Good Laboratory Practice (GLP) and the GLP principles of the German "Chemikaliengesetz" (Chemicals Act)

CONTROL DATA OF MALE SEX ORGAN WEIGHTS [1)]

Species: Rat
Sex/Age: Males PND 21
Strain: Wistar
Breeder: Charles River Labs, Sulzfeld Germany
Data print out at 8-May-14

Relative Male Sex Organ Weights on PND 21 (Subset 1)

Study No.	Cauda epididymis			Epididymides			Muscles bulb + l.ani		
	N	Mean	SD	N	Mean	SD	N	Mean	SD
88R001	10	0.027	0.009	10	0.066	0.013	10	0.076	0.019
03R003	10	0.026	0.009	10	0.069	0.009	10	0.111	0.024
88R002	10	0.023	0.01	10	0.065	0.014	10	0.081	0.023

Study No.	Prostate			Prostate ventr.fresh			Seminal vesicle			Testes		
	N	Mean	SD	N	Mean	SD	N	Mean	SD	N	Mean	SD
88R001	10	0.109	0.017	10	0.06	0.01	10	0.022	0.005	10	0.518	0.037
03R003	10	0.114	0.017	10	0.064	0.009	10	0.022	0.005	10	0.541	0.033
88R002	10	0.11	0.02	10	0.055	0.008	10	0.021	0.005	10	0.532	0.026

1) Source: All data were collected and archived at the test facility Experimental Toxicology and Ecology, BASF SE, 67056 Ludwigshafen, Germany, in accordance with the OECD principles of Good Laboratory Practice (GLP) and the GLP principles of the German "Chemikaliengesetz" (Chemicals Act)

CONTROL DATA OF MALE SEX ORGAN WEIGHTS [1)]

Species:	Rat
Sex/Age:	Males Puberty
Strain:	Wistar
Breeder:	Charles River Labs, Sulzfeld Germany
Data print out at	8-May-14

Absolute Male Sex Organ Weights at Puberty (Subset 2)

Study No.	Bulbo-urethral gland			Cauda epididymis			Epididymides			Glans penis			Muscles bulb + l.ani		
	N	Mean	SD	N	Mean	SD	N	Mean	SD	N	Mean	SD	N	Mean	SD
88R001	10	12.74	2.76	10	61.7	8.138	10	211.9	19.255	10	42.24	8.49	10	186.04	27.447
03R003	10	14.83	5.418	10	65.0	17.185	10	204.6	49.119	10	46.19	9.484	10	180.2	43.254
88R002	10	13.86	3.294	10	57.7	11.461	10	220.2	44.296	10	49.53	7.358	10	194.66	33.864

Study No.	Prostate			Prostate ventr.fresh			Seminal vesicle			Testes		
	N	Mean	SD	N	Mean	SD	N	Mean	SD	N	Mean	SD
88R001	10	194.44	23.942	10	107.74	16.199	10	91.11	15.158	10	1967.9	140.091
03R003	10	204.77	46.436	10	114.05	27.431	10	101.73	49.42	10	1919	445
88R002	10	187.1	38.297	10	106.59	29.408	10	109.97	36.366	10	2227.1	321.053

1) Source: All data were collected and archived at the test facility Experimental Toxicology and Ecology, BASF SE, 67056 Ludwigshafen, Germany, in accordance with the OECD principles of Good Laboratory Practice (GLP) and the GLP principles of the German "Chemikaliengesetz" (Chemicals Act)

CONTROL DATA OF MALE SEX ORGAN WEIGHTS [1)]

Species:	Rat
Sex/Age:	Males Puberty
Strain:	Wistar
Breeder:	Charles River Labs, Sulzfeld Germany
Data print out at	8-May-14

Relative Male Sex Organ Weights at Puberty (Subset 2)

Study No.	Bulbo-urethral gland			Cauda epididymis			Epididymides			Glans penis			Muscles bulb + l.ani		
	N	Mean	SD	N	Mean	SD	N	Mean	SD	N	Mean	SD	N	Mean	SD
88R001	10	0.007	0.001	10	0.034	0.004	10	0.118	0.008	10	0.023	0.004	10	0.104	0.017
03R003	10	0.008	0.002	10	0.037	0.008	10	0.116	0.021	10	0.026	0.005	10	0.103	0.018
88R002	10	0.007	0.002	10	0.031	0.004	10	0.119	0.017	10	0.027	0.003	10	0.105	0.015

Study No.	Prostate			Prostate ventr.fresh			Seminal vesicle			Testes		
	N	Mean	SD	N	Mean	SD	N	Mean	SD	N	Mean	SD
88R001	10	0.108	0.01	10	0.06	0.008	10	0.05	0.007	10	1.094	0.081
03R003	10	0.117	0.02	10	0.065	0.013	10	0.057	0.024	10	1.099	0.243
88R002	10	0.101	0.015	10	0.057	0.013	10	0.059	0.015	10	1.205	0.116

1) Source: All data were collected and archived at the test facility Experimental Toxicology and Ecology, BASF SE, 67056 Ludwigshafen, Germany, in accordance with the OECD principles of Good Laboratory Practice (GLP) and the GLP principles of the German "Chemikaliengesetz" (Chemicals Act)

CONTROL DATA OF MALE SEX ORGAN WEIGHTS [1)]

Species: Rat
Sex/Age: Males PND 83±2
Strain: Wistar
Breeder: Charles River Labs, Sulzfeld Germany
Data print out at 8-May-14

Absolute Male Sex Organ Weights on PND 83±2 (Subset 3)

Study No.	Bulbo-urethral gland			Cauda epididymis			Epididymides			Glans penis			Muscles bulb + l.ani		
	N	Mean	SD	N	Mean	SD	N	Mean	SD	N	Mean	SD	N	Mean	SD
88R001	10	69.74	16.977	10	312.3	33.34	10	875.3	79.068	10	88.98	7.785	10	696.32	83.875
03R003	10	70.2	13.143	10	324.3	50.33	10	910.1	100.285	10	95.3	13.954	10	672.6	129.178
88R002	9	74.867	20.09	9	324.667	31.914	9	897.111	80.093	9	89.878	10.305	9	711.167	83.305

Study No.	Prostate			Prostate ventr.fresh			Seminal vesicle			Testes		
	N	Mean	SD	N	Mean	SD	N	Mean	SD	N	Mean	SD
88R001	10	606.48	84.302	10	339.38	45.478	10	842.38	126.195	10	3387.4	260.32
03R003	10	730.24	134.271	10	382.32	73.97	10	906.38	120.558	10	3428	360
88R002	9	642.556	61.158	9	347.578	52.268	9	989.233	176.964	9	3530.222	253.004

1) Source: All data were collected and archived at the test facility Experimental Toxicology and Ecology, BASF SE, 67056 Ludwigshafen, Germany, in accordance with the OECD principles of Good Laboratory Practice (GLP) and the GLP principles of the German "Chemikaliengesetz" (Chemicals Act)

CONTROL DATA OF MALE SEX ORGAN WEIGHTS [1)]

Species:	Rat
Sex/Age:	Males PND 83±2
Strain:	Wistar
Breeder:	Charles River Labs, Sulzfeld Germany
Data print out at	8-May-14

Relative Male Sex Organ Weights on PND 83±2 (Subset 3)

Study No.	Bulbo-urethral gland			Cauda epididymis			Epididymides			Glans penis			Muscles bulb + l.ani		
	N	Mean	SD	N	Mean	SD	N	Mean	SD	N	Mean	SD	N	Mean	SD
88R001	10	0.02	0.004	10	0.09	0.007	10	0.252	0.018	10	0.026	0.002	10	0.2	0.021
03R003	10	0.022	0.004	10	0.1	0.019	10	0.281	0.032	10	0.03	0.005	10	0.208	0.044
88R002	9	0.024	0.006	9	0.103	0.011	9	0.285	0.025	9	0.029	0.004	9	0.226	0.033

Study No.	Prostate			Prostate ventr.fresh			Seminal vesicle			Testes		
	N	Mean	SD	N	Mean	SD	N	Mean	SD	N	Mean	SD
88R001	10	0.174	0.017	10	0.097	0.008	10	0.241	0.025	10	0.976	0.079
03R003	10	0.226	0.043	10	0.119	0.027	10	0.281	0.046	10	1.057	0.107
88R002	9	0.204	0.018	9	0.11	0.016	9	0.313	0.05	9	1.123	0.114

1) Source: All data were collected and archived at the test facility Experimental Toxicology and Ecology, BASF SE, 67056 Ludwigshafen, Germany, in accordance with the OECD principles of Good Laboratory Practice (GLP) and the GLP principles of the German "Chemikaliengesetz" (Chemicals Act)
